# Insulin, Semaglutide and Dapagliflozin in Adults With Type 1 Diabetes: Design and Methods of Triple Therapy for Type 1 Diabetes (TTT1)—An International Phase 3 Clinical Trial

**DOI:** 10.1111/dom.71076

**Published:** 2026-07-19

**Authors:** Joseph G. Timmons, Husam Ghanim, James G. Boyle, Nicola Greenlaw, Ajay Chaudhuri, Manav A. Batra, Robert S. Lindsay, Lisa Jolly, Caroline Bonner, Alex McConnachie, John R. Petrie, Paresh Dandona

**Affiliations:** ^1^ School of Cardiovascular and Medical Sciences, College of Medical, Veterinary and Life Sciences University of Glasgow Glasgow UK; ^2^ State University of New York at Buffalo Buffalo New York USA; ^3^ School of Medicine, Dentistry and Nursing, College of Medical, Veterinary and Life Sciences University of Glasgow Glasgow UK; ^4^ Robertson Centre for Biostatistics/Glasgow Clinical Trials Unit, School of Health and Wellbeing, College of Medical, Veterinary and Life Sciences University of Glasgow Glasgow Scotland UK; ^5^ Department of Research and Innovation, NHS Greater Glasgow and Clyde Gartnavel Royal Hospital Glasgow UK; ^6^ University of Lille CHU Lille, Inserm U1190, European Genomic Institute for Diabetes (EGID), Institut Pasteur de Lille Lille France

**Keywords:** adjunct therapy, adjunctive therapy, combination therapy dapagliflozin, ketosis, semaglutide, type 1 diabetes

## Abstract

**Aims:**

Attaining target glycaemia can be a challenge in Type 1 Diabetes (T1D) due to insulin‐induced weight gain. Adjunct therapy with modern glucose‐lowering agents developed for type 2 diabetes (T2D) has great potential but may be insufficiently efficacious and carries risks of hypoglycaemia and ketosis. We designed the first Phase 3 clinical trial to assess the efficacy and safety of adding a Glucagon‐Like Peptide 1 receptor agonist (GLP‐1RA) and a Sodium‐Glucose Co‐transporter (SGLT2) Inhibitor to insulin therapy in overweight and obese adults with T1D and glycaemia above target (HbA1c 7.5%–11.0% inclusive) (NCT03899402).

**Materials and Methods:**

In Period 1, participants are randomized 2:1 (open label) for 26 weeks to semaglutide and insulin (uptitrated to 1.0 mg weekly) or standard insulin therapy. In Period 2, those randomized to semaglutide and insulin in Period 1 are further randomized (double‐blind) for 26 weeks to dapagliflozin (10 mg daily) or placebo, in addition to semaglutide. The primary objective is to compare change in HbA1c on ‘triple therapy’ (dapagliflozin, semaglutide and insulin) with ‘dual therapy’ (placebo, semaglutide and insulin). Secondary objectives include comparisons of triple therapy with standard insulin therapy and dual therapy (semaglutide and insulin) with standard insulin therapy. Safety outcomes include hypoglycaemia and ketosis. A sample size recalculation during the trial based on analysis of masked data revised the original recruitment target from 114 to 82 participants.

**Conclusion:**

The TTT1 trial will provide clinically useful information on combination adjunct therapy in the treatment of T1D.

**Abbreviations**
AUC: area under the curve
BMI: body mass index
CGM: continuous glucose monitoring
CSII: continuous subcutaneous insulin infusion
DSQOLS: Diabetes Specific Quality of Life Scale
FGM: flash glucose monitoring
GLP‐1RA: glucagon‐like peptide 1 receptor agonist
HFHC: high fat high carbohydrate
IDMC: Independent Data Monitoring Committee
IMP: Investigational Medicinal Product
ITT: intention‐to‐treat
MDI: multiple daily injections
mITT: modified intention‐to‐treat
PAID: Problem Areas in Diabetes Scale
SGLT2: sodium‐glucose co‐transporter 2
TSC: Trial Steering Committee
TTT1: Triple Therapy in Type 1 Diabetes

## 1 Introduction

Even with the most advanced insulin preparations and delivery systems currently available, it remains a challenge for many people with T1D to achieve target levels of glycaemia [1]. Many experience insulin‐induced weight gain and associated insulin resistance leading to ongoing escalation of insulin doses. The increasing societal prevalence of obesity is also reflected in people with T1D, around 60% of whom are now overweight or obese [2, 3, 4, 5]. In the quartile of individuals with the largest increase in body mass index (BMI) on intensive treatment during the diabetes control and complications trial (DCCT), the cardiovascular benefits of intensive vs. conventional glucose control were lost in long‐term follow‐up in the Epidemiology of Diabetes Interventions and Complications (EDIC) study. Such individuals can be considered as having ‘double diabetes’ [6, 7]. Recent data indicate that increasing use of hybrid closed‐loop systems may exacerbate rather than solve the issue [8].

Adjunct therapy in T1D consists of adding in glucose‐lowering agents other than insulin with the aim of lowering HbA1c and improving other health outcomes, including weight. It is an attractive potential strategy for achieving glycaemic targets, particularly in those who are obese or prone to weight gain [9]. However, when metformin was tested over 3 years in the REMOVAL trial, there were quite modest effects on weight and LDL cholesterol, and there was no sustained effect on HbA1c [10]. More recently, members of the SGLT‐2 inhibitor and GLP‐1RA drug classes have been investigated as adjunctive agents in view of more profound effects on bodyweight combined with protective effects against cardiovascular and renal complications [11, 12, 13, 14, 15]. With such beneficial effects demonstrated even in individuals without diabetes, and increasing demand for off‐label prescribing from people with T1D, it seems particularly important to establish whether these agents are safe and efficacious in this population. However, trials to date with SGLT‐2 inhibitors in T1D have not supported widespread use for this indication due to appreciable elevations in the risks of ketosis and ketoacidosis [16, 17, 18, 19]. Similarly, GLP‐1RAs (and dual agonists) have not so far been licensed for use in T1D, as effect sizes for outcomes such as HbA1c, body weight and total daily insulin dose in trials with the daily GLP‐1 agonists were smaller than desired and offset by increased rates of adverse effects including hypoglycaemia and ketosis (depending on the insulin dosing schedule) [20, 21].

Nevertheless, a recent trial of the weekly GLP‐1RA semaglutide in adults with T1D using hybrid closed‐loop systems has shown promise [22] and several other trials with weekly agents are currently in progress. The combination of SGLT‐2 inhibitor and GLP‐1RA therapy is potentially attractive as: (i) agents from the two drug classes may work synergistically to improve glycaemia, reduce weight and total daily insulin dose [9]; and (ii) there may be a safety advantage when the glucagon secretion driving SGLT‐2 inhibitor‐associated ketosis is suppressed by GLP‐1RAs [23]. This provides a rationale for stabilizing management on a GLP‐1 agonist before adding in an SGLT‐2 inhibitor.

The Triple Therapy for T1D with insulin, semaglutide and dapagliflozin (TTT1) trial was therefore designed to examine the efficacy and safety of combination GLP‐1 and SGLT‐2 inhibitor adjunct therapy in overweight and obese people with T1D using two 26‐week treatment periods. In Period 1, the weekly subcutaneous GLP‐1RA agonist semaglutide is uptitrated to the maximal tolerated dose (‘dual therapy’); in Period 2, the daily oral SGLT‐2 inhibitor dapagliflozin is added in (triple therapy). All participants are maintained throughout on standard of care insulin therapy titrated to target.

We selected comparison of the effect of triple vs. dual therapy on HbA1c (in Period 2) as the primary outcome in order to focus on whether adding in an SGLT2 inhibitor to ongoing GLP‐1 therapy improvesoverall efficacy without compromising safety. Pre‐specified key secondary outcomes (Table 1) include comparison of the effects on HbA1c of: (i) dual therapy vs. standard of care insulin therapy (in Period 1); and (ii) triple therapy vs. standard of care insulin therapy (over Periods 1 and 2 combined). In addition, as triple therapy is compared with dual therapy in Period 2, and dual therapy is compared with standard of care in Period 1, an indirect comparison between triple therapy and standard of care is incorporated.

**TABLE 1:** Primary, secondary and tertiary outcomes.

	Objectives	Outcome measures
Primary	To assess in people with type 1 diabetes whether triple therapy (dapagliflozin, semaglutide and insulin) reduces HbA1c in comparison with dual therapy (placebo, semaglutide and insulin—period 2).	HbA1c
Secondary	To assess in people with type 1 diabetes whether triple therapy (dapagliflozin, semaglutide and insulin) reduces HbA1c in comparison with standard therapy (insulin alone)	(i) HbA1c
	To assess in people with type 1 diabetes whether dual therapy (semaglutide and insulin‐period 1) reduces HbA1c in comparison with standard therapy (insulin alone)	(ii) HbA1c
	To assess in people with type 1 diabetes whether triple therapy (dapagliflozin, semaglutide and insulin) improves glucose control as assessed by CGM, insulin requirement, body weight, blood pressure and quality of life in comparison with (a) dual therapy (placebo, semaglutide and insulin—period 2) and (b) standard therapy (insulin alone)	(iii) Mean fasting blood glucose (iv) Mean blood glucose^*^ (v) Percentage time in range (vi) Glycaemic variability^*^ (vii) Percentage time in hyperglycaemia Level 1 (10.1–13.9 mmol/)^*^ (viii) Percentage time in hyperglycaemia Level 2 (> 13.9 mmol/L)^*^ (ix) Percentage time in hypoglycaemia Level 1 (3.0–3.8 mmol/L)^*^ (x) Insulin requirement (xi) Body weight (xii) Systolic/diastolic blood pressure (xiii) Number of antihypertensive agents (xiv) Diabetes Specific Quality of Life Scale (DSQOLS) (xv) Problem Areas In Diabetes (PAID) questionnaire
Tertiary outcomes	As per above comparisons	Postprandial glucose (AUC) – meal tolerance test Postprandial glucagon (AUC)—meal tolerance test Postprandial FFA (AUC)—meal tolerance test Postprandial GLP‐1 (AUC)—meal tolerance test
Safety Endpoint 1	(a) Triple therapy (dapagliflozin, semaglutide and insulin) in comparison with dual therapy (placebo, semaglutide, insulin—period 2) (b) Dual therapy (semaglutide and insulin—period 1) in comparison with standard therapy (insulin only)	Difference between treatment arms in: (i) Rate of Level 2 (< 3.0 mmol/L) hypoglycaemic events (by Dexcom G6 CGM) (ii) Rate of Level 3 hypoglycaemic events (severe event with altered mental and/or physical status requiring assistance)
Safety Endpoint 2	(a) Triple therapy (dapagliflozin, semaglutide and insulin) in comparison with dual therapy (placebo, semaglutide and insulin – period 2) (b) Dual therapy (semaglutide and insulin –period 1) in comparison with standard therapy (insulin only)	Difference between treatment arms in: Rate of diabetic ketoacidosis (serum ketones or urinary ketones above normal limits AND serum bicarbonate < 15 mmol/L OR blood pH < 7.3)

^*^ CGM (by masked Dexcom G6).

## 2 Materials and Methods

### 2.1 Objectives and Endpoints

**FIGURE 1:**
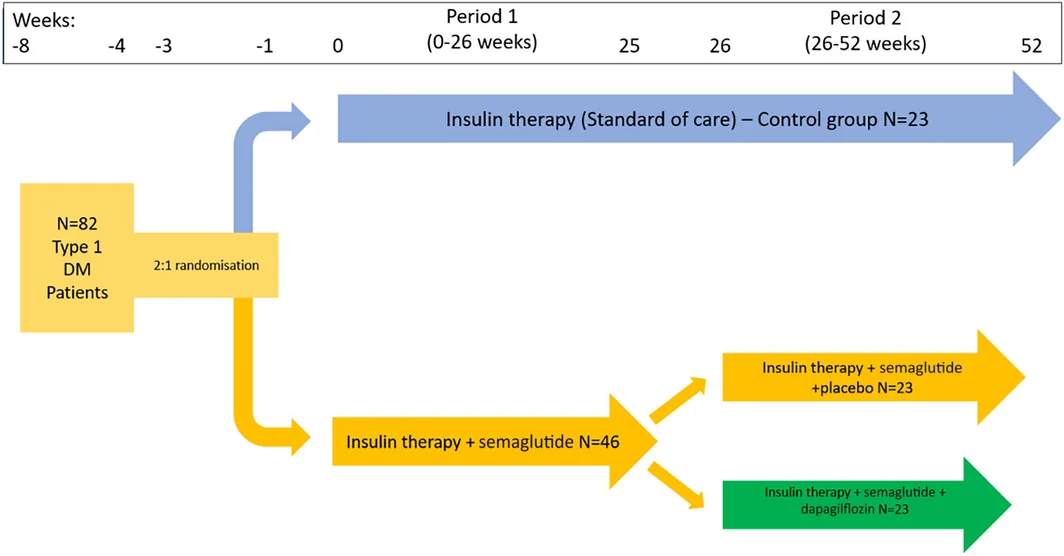
Triple therapy in Type 1 Diabetes (TTT1) trial design.

The primary objective of TTT1 is to assess in overweight and obese (BMI ≥ 25 kg/m^2^) people with T1D and glycaemia above target (HbA1c 7.5%–11.0% inclusive) whether triple therapy (dapagliflozin, semaglutide and insulin) for 26 weeks improves glycaemic control (as measured by HbA1c) compared with dual therapy (placebo, semaglutide and insulin) (Figure 1).

Secondary objectives (Table 1) include assessing: whether triple therapy (dapagliflozin, semaglutide and insulin) improves glycaemic control compared with standard therapy (insulin) alone; whether dual therapy (semaglutide and insulin) improves glycaemic control in comparison with standard of care therapy (insulin only); and the comparative effects of these treatment strategies on continuous glucose monitoring (CGM) parameters (including Time in Range and Time Below Range) [24], body weight, daily insulin requirement, blood pressure and Quality of Life [as assessed by validated questionnaires Problem Areas in Diabetes Scale (PAID) and Diabetes Specific Quality of Life Scale (DSQOLS)] [25, 26]. CGM data were collected using three 20‐day periods of masked Dexcom G6 to avoid any bias arising from open‐label treatment allocation to semaglutide or standard of care. Participants were free to use any established ‘flash’ or CGM monitoring as per their standard of care prior to trial enrolment.

Safety outcomes examine whether the addition of semaglutide with and without dapagliflozin to standard insulin therapy increases the rate of Level 2 (< 3.0 mmol/L/< 54 mg/dL) or Level 3 (severe event with altered mental and/or physical status requiring assistance) hypoglycaemic events as well as the number of diabetic ketoacidosis events defined as elevated serum or urine ketones (greater than the upper limit of the normal range) and serum bicarbonate < 15 mmol/L or blood pH < 7.3 [17, 27, 28, 29].

Tertiary outcomes explore the effect of semaglutide with or without dapagliflozin added to standard insulin therapy on postprandial glucose, glucagon, free fatty acids and native GLP‐1 measured across a standard high fat, high carbohydrate (HFHC) mixed meal challenge test. Urine and plasma samples (frozen) are retained for analysis of exploratory outcomes, including measures of inflammatory stress.

### 2.2 Trial Management and Oversight

The trial is conducted in two locations: State University of New York at Buffalo, USA and the University of Glasgow/NHS Greater Glasgow and Clyde, Scotland, UK. Separate trial protocols are implemented for each site to meet local governance requirements (see Tables S2 and S3 and uploaded Supporting Information). A single electronic Case Report Form (eCRF) is used by both sites to collect a single set of study data. Approvals were from the Research Ethics Committee, State University of New York, Buffalo, NY, USA and the West of Scotland Research Ethics Committee (REC1) respectively. Trial management, oversight and monitoring are the responsibility of the University at Buffalo Institutional Review Board (UBIRB), The State University of New York, NY (US site) and the University of Glasgow and NHS Greater Glasgow and Clyde as co‐sponsors (UK site). Data management and statistical analysis on the final dataset will be undertaken by The Robertson Centre for Biostatistics/Glasgow Clinical Trials Unit, University of Glasgow, UK. Participant data are uploaded by trained staff at both sites and stored on a secure, purpose‐designed eCRF. An Independent Trial Steering Committee (TSC) meets six monthly to monitor the design and conduct of the trial and to make recommendations to the Chief Investigator and Sponsor on modifications to the protocol and/or trial procedures. In addition, an Independent Data Monitoring Committee (IDMC) meets six monthly to monitor patient safety, the quality of efficacy data collection and study conduct. The IDMC has the potential to recommend early discontinuation of the trial to the TSC for safety or other reasons.

### 2.3 Investigational Medicinal Products (IMPs)

Doses of semaglutide (Ozempic, Novo Nordisk) (0.25, 0.5 and 1.0 mg as prefilled pens for subcutaneous injection) were purchased directly on the market for open‐label use during the trial in both countries. Following supply issues in the UK (commencing September 2022), two batches were donated by Novo Nordisk UK for use on the trial at the request of the UK Chief Investigator.

Dapagliflozin (Farxiga US) (5, 10 mg) and matching placebo for oral administration was donated by AstraZeneca (USA) for use at the US site. However, as this could not be exported to the UK for regulatory reasons, dapagliflozin (Forxiga) (5, 10 mg) was purchased on the market for the UK site and over‐encapsulated with matching placebo by Pharmaceutical Services Scotland (Dundee, UK).

### 2.4 Screening, Eligibility, Enrolment and Run‐In Period

Potential participants identified at each site are invited to an initial visit for screening against the inclusion and exclusion criteria (see Table S1).

The study includes adults aged 18 to 70 years with type 1 diabetes for at least 1 year who have been using a stable insulin regimen for the preceding 3 months. Eligible participants must have a fasting C‐peptide level below 0.23 nmol/L at the screening visit, a minimum daily insulin dose of 0.5 U/kg for Multiple Daily Injections (MDI) [or 0.4 U/kg for continuous subcutaneous insulin infusion (CSII)] and regularly monitor blood glucose at least four times daily, either by fingerstick testing or CGM/flash glucose monitoring (FGM). Additional inclusion criteria include an HbA1c between 7.5% and 11.0% at screening, a body mass index (BMI) of at least 25 kg/m^2^ and proficiency in carbohydrate counting as assessed by the site Principal Investigator.

Participants are excluded if they have had type 1 diabetes for less than 12 months, type 2 diabetes, Maturity Onset Diabetes of the Young, or chronic pancreatitis, or if they have used any non‐insulin glucose‐lowering agent within the last 3 months. Other exclusion criteria include recent diabetic ketoacidosis requiring medical care, adherence to a ketogenic or very low‐carbohydrate diet, hypoglycaemia unawareness/frequent severe hypoglycaemia, or poorly controlled diabetes symptoms. Individuals engaged in active weight loss or intensive exercise programs, or those who have had bariatric or weight‐loss surgery within the past year, are also excluded. Further exclusion criteria include renal impairment (eGFR < 45 mL/min/1.73 m^2^), a history of gastroparesis, medullary thyroid carcinoma, Multiple Endocrine Neoplasia‐2 syndrome, gallstones and proliferative retinopathy. Participants considered unlikely to comply with study procedures or dietary requirements (e.g., vegetarian or vegan diets preventing participation in a HFHC mixed meal challenge test) are also ineligible.

### 2.5 Screening Visit

At the screening assessment, demographic data (date of birth, sex, ethnicity, date of diagnosis and height), medical history and current medications including usual insulin dose and method of insulin delivery are recorded (MDI, CSII, low glucose suspend, or hybrid closed‐loop). Smoking and alcohol use, history of hypertension, microalbuminuria, proteinuria, neuropathy, cardiovascular disease, amputation and recent hospitalizations for diabetic ketoacidosis are recorded, along with their highest level of education. A physical examination includes measurements of blood pressure (after 5 min of rest), heart rate, body weight and capillary blood glucose and ketones. Blood samples are taken to assess HbA1c, full blood count, renal function (urea, electrolytes, creatinine), liver function (AST, ALT, ALP), bone profile (calcium, phosphate) and C‐peptide. Urine samples are collected to test for ketones and for pregnancy in women of childbearing potential.

### 2.6 Run‐In Period

The protocol includes 17 study visits in total over 56–60 weeks (Table 2). During the 3–8 weeks prior to Period 1 randomization, participants meeting entry criteria undergo two preparatory visits to optimize diabetes management and ensure study readiness. At Visit 1 (−3 weeks), glucose monitoring data (from diaries, CGM, or insulin pump) are reviewed; insulin doses are adjusted to maintain fasting glucose between 5 and 10 mmol/L, and participants are trained on the use of masked Dexcom G6 CGM. At Visit 2 (−1 week), participants attend fasting and (after confirming appropriate glucose and ketone levels) undergo a HFHC mixed meal challenge test with serial blood sampling. Instructions and a container are given so that a 24‐h urine collection can be provided at the next visit.

**TABLE 2:** Schedule of assessments.

Visit #	—	1	2	3	4^c^	5^c^	6	7	8	9	10	11^c^	12	13	14	15	16
Week# (±7 days; see below)	−8 to −4	−3	−1	0	1	2	4	13	23	25	26	27	31	39	49	51	52
Study Procedures	Screening	Run‐in period		Randomize							Randomize						End
Informed Consent	X																
Fasting visit			X	X				X		X	X			X		X	X
History and demographics	X																
Physical	X																X
Education on hypoglycaemia and ketosis avoidance				X							X						
BP, heart rate, weight	X	X	X	X	X	X	X	X	X	X	X	X	X	X	X	X	X
Bloods (FBC, kidney and liver profiles)	X			X				X			X		X	X			X
C‐peptide	X										X						X
Pregnancy test	X			X			X^a^	X^a^	X^a^		X^a^		X^a^	X^a^	X^a^	X^a^	X^a^
Dispense open‐label semaglutide^b^				X		X^b^	X^b^	X^b^			X^b^			X^b^			
Titrate open‐label semaglutide^b^						X^b^	X^b^	X^b^									
Dose Semaglutide (on‐site if dose is due on visit day)				X	X	X											
Dispense dapagliflozin/placebo											X	X		X			
Dapagliflozin/placebo return capsule counts												X		X			X
HbA1c	X			X				X		X				X			X
Blood endpoints				X				X			X			X			X
Masked CGM fit		X							X						X		
Masked CGM read				X							X						X
Daily glucose monitoring review		X	X	X	X	X	X	X	X	X	X	X	X	X	X	X	X
Daily urinary ketones review												X	X	X	X	X	X
Insulin dose review		X	X	X	X	X	X	X	X	X	X	X	X	X	X	X	X
Ketone bodies (blood/urine)	X	X	X	X	X	X	X	X	X	X	X	X	X	X	X	X	X
Adverse Event reporting		X	X	X	X	X	X	X	X	X	X	X	X	X	X	X	X
Meal Challenge Test			X							X						X	
24 h Urine dispensed			X				X			X			X			X	
24 h Urine collected				X				X			X			X			X
Quality of life questionnaires				X							X						X

*Note:* N.B. Safety phone calls will be conducted at weeks 3, 5, 8, 16, 20, 29, 35, 44 and at 2 weeks after the end of the study to document any side effects or other changes. Additional visits or phone calls may be scheduled with the verbal consent of the participant if study team believe necessary, for example, closer monitoring is appropriate. Visit dates should be within 7 days of the visit time; however, a longer or shorter interval does not constitute a protocol deviation.

^a^ Not required in women randomized to ‘Insulin therapy (standard of care)’.

^b^ According to randomization schedule: resupply of open‐label semaglutide after Visit 3 will be according to individual requirements as tolerated.

^c^ Visits 4, 5 and 11 have the option of being carried out by telephone for those participants allocated to the standard care arm only.

### 2.7 Main Study Period and Randomization

At visit 3, following further baseline assessments outlined below, participants proceed to randomization in Period 1 of the trial using an interactive web‐based randomization system (IWRS), allocating in a 2:1 ratio to either open‐label semaglutide or standard care using permuted blocks of variable size to minimize predictability, stratified by site. In addition to the in‐person study visits outlined below, safety phone calls are conducted at weeks 3, 5, 8, 16, 20, 29, 35, 44 and at 2 weeks after the end of the study to support participants and document any side effects or other changes.

#### 2.7.1 First Randomization Visit: Period 1

Participants attend in the fasting state (no food or calorie intake for 10 h). Capillary glucose and ketone levels are measured using a combined glucose and ketone meter (see below) and daily home blood glucose monitoring levels (recorded in study diaries) are collected and reviewed. Each participant's own CGM or FGM data or insulin pump download data are also collected and reviewed and masked study CGM (Dexcom G6) discontinued. Daily insulin dose is adjusted following review of study diaries and other data (see below).

A fasting blood sample is taken for routine blood tests and study endpoints. A urine sample is collected for evaluation of ketones (and pregnancy status if appropriate). The 24‐h urine container is collected. Participants are asked to complete Quality of Life questionnaires. At the time of writing, the trial had closed to recruitment: the baseline characteristics of all participants who had undergone the first randomization are shown in Table 3.

**TABLE 3:** Baseline demographics of participants at first randomization (ITT population).

	Standard of care (insulin) and semaglutide (*n* = 51)	Standard of care (insulin) (*n* = 27)
Age	46 ± 13.6	44 ± 14.2
Sex (*n*, % female)	26 (51)	14 (52)
Ethnicity: Non‐Hispanic White/Black/Hispanic (*n*)	49/1/1	24/3/0
Ethnicity: Non‐Hispanic White/Black/Hispanic (%)	(96/2/2)	(89/11/0)
Education		
College educated (US) (*n*, %)	28 (78)	15 (83)
University educated (UK) (*n*, %)	7 (47)	4 (44)
Duration of Diabetes (years)	24 ± 11.7	27 ± 12.7
C‐peptide detectable (*n*, %)		
US site^a^	4 (11)	2 (11)
UK site	1 (7)	0 (0)
HbA1c %	8.2 ± 0.8	8.3 ± 0.7
HbA1c (mmol/mol)	67 ± 8.3	67 ± 7.2
Time in range (%)	48 ± 11.0	44 ± 14.2
Time in level 1 hypoglycaemia (%)	1.0 (1.0, 2.0)^b^	1.0 (1.0, 2.0)^b^
Insulin regimen (*n*, %)		
Multiple daily doses	23 (45)	8 (30)
Standard pump	15 (29)	8 (30)
Low glucose suspend	3 (6)	3 (11)
Hybrid closed loop	10 (20)	8 (30)
Total daily insulin dose	67 ± 28.9	71 ± 34.6
Total daily insulin dose (per kg)	0.70 ± 0.24	0.74 ± 0.27
Blood ketones (mmol/L)	0.1 (0.10–0.20)^b^	0.1 (0.10–0.20)^b^
Hospitalization for DKA in previous year (*n*, %)	2 (4)	0 (0)
BMI (kg/m^2^)	34 ± 6.3	32 ± 5.7
Body weight (kg)	94.3 ± 19.2	92.0 ± 17.9
Systolic BP (mmHg)	129 ± 14.7	128 ± 16.4
Diastolic BP (mmHg)	80 ± 7.7	80 ± 10.1
History of hypertension (%)	29	33
Prescribed Angiotensin Converting Enzyme inhibitor (%)	13 (25)	10 (37)
Prescribed Angiotensin Receptor Blocker (%)	5 (10)	4 (15)
Prescribed statin (%)	23 (45)	14 (52)
Prescribed aspirin (%)	9 (18)	6 (22)
Smoking status (*n*, %)		
Never	37 (73)	19 (70)
Former	13 (25)	6 (22)
Current	1 (2)	2 (8)
Triglycerides (mmol/L)	1.0 ± 0.40	1.1 ± 0.43
Weekly alcohol intake (units)	1 (0.0, 4.0)^b^	2 (2.0, 6.0)^b^
Cardiovascular disease (*n*, %)	0 (0)	1 (4)
eGFR (ml/min/1.73 m^2^)	86 ± 22.7	85 ± 24.9
Microalbuminuria (%)	0	0
Retinopathy (*n*, %)	12 (24)	5 (19)
Neuropathy (*n*, %)	6 (12)	4 (15)

*Note:* All data are shown as mean ± SD unless otherwise stated. Rounded percentages may not total 100%.

^a^ Ultrasensitive assay.

^b^ Median, interquartile range (IQR).

For those randomized to once weekly open‐label semaglutide, the first dose (0.25 mg) is given on site. Participants are instructed to call site staff directly in case of any problem or side effects, and specifically if they have hypoglycaemia (blood glucose < 3.9 mmol/L) or Level 2 hyperglycaemia (blood glucose > 13.9 mmol/L) on more than one occasion at any time during participation in the trial.

Semaglutide is uptitrated at two weekly intervals, as tolerated, to a maximum dose of 1.0 mg once weekly. Where participants are unable to tolerate higher doses, the maximum tolerable dose is continued for the remainder of the trial. Where semaglutide is stopped due to intolerable side effects, participants are encouraged to continue to attend study visits and undertake study assessments as per the intention‐to‐treat principle.

#### 2.7.2 Second Randomization: Period 2

Participants previously randomized to open‐label semaglutide are subsequently randomized (1:1) at visit 10 (week 26) to dapagliflozin or matching placebo using permuted blocks of variable size to minimize predictability with stratification for site and HbA1c (HbA1c ≤ 7.5% vs. HbA1c > 7.5%). Participants are asked to monitor ketone levels in the 3 days prior to and including the day of the second randomization. For those with ketone values of 0.6 mmol/L or higher on one of those days, blood glucose levels, insulin dose and carbohydrate intake are reviewed and advice given on hydration, adequate CHO intake and insulin dosing. The visit is rescheduled 1 week later with repeat ketone testing for 3 days prior.

Dapagliflozin (or placebo) is started at a dose of 5 mg once daily, titrated to 10 mg daily the following week if tolerated and continued at that dose for the remainder of the trial.

Those previously randomized to standard of care continue as before.

#### 2.7.3 HFHC Mixed Meal Challenge Test

To examine the effects of semaglutide alone or in combination with dapagliflozin on glucose, glucagon, free fatty acids and GLP‐1 (tertiary trial outcomes), a HFHC mixed meal challenge test is undertaken prior to the first randomization, the second randomization and the end of trial (visits 2, 9 and 15). This is performed using an approximately 900 kcal HFHC mixed meal including an egg muffin sandwich, a sausage muffin sandwich and two hash browns [containing approximately 88 g carbohydrate, 51 g fat (33% saturated) and 34 g protein]. Participants are asked to attend fasting prior to breakfast insulin bolus (while continuing basal insulin); they are asked not to take bolus insulin until the 3 h time point unless blood glucose values reach > 400 mg/dL/22.2 mmol/L (in which case the test is stopped early for safety). Blood samples are collected from an indwelling intravenous canula in a superficial forearm vein for glucose, glucagon, FFA, GLP‐1 [area under the curve (AUC)]. Sequential blood samples are obtained at 0, 15, 30, 45, 60, 90, 120, 150 and 180 min after consuming the meal.

### 2.8 End of Trial and Post‐Trial Procedures

At the end of the trial period, all participants will discontinue study medications and revert to standard of care (insulin therapy only). The final safety phone call is made 2 weeks after the last visit to provide a further opportunity to advise on insulin dosing and ensure no ongoing problems or concerns related to the study. The trial team will liaise with the usual care team to ensure a safe transition back to standard therapy.

If study medication requires to be stopped before the end of the trial, the reason for discontinuation is recorded where given and the trial team facilitates safe transition back to the usual standard of care. For participants who voluntarily choose to leave the trial before the specified end date, again all efforts are made by the trial team to facilitate safe transition back to usual standard of care. A follow‐up safety visit is offered to all such participants and ongoing contact details are provided in case of any further issues relating to the clinical trial.

If the trial ends early for safety reasons, all participants will be informed and safe discontinuation of study medications will be undertaken under the supervision of the site Principal Investigator.

### 2.9 Safety Procedures and Risk Management

All participants are provided with a Patient Alert Card and asked to carry it at all times. This contains a listing of the investigational study drugs, the study number, the site investigator's name, a contact number for the trial team, and emergency unblinding procedures.

#### 2.9.1 Insulin Dose Adjustment

If insulin dose is reduced to mitigate risk of hypoglycaemia at the first randomization visit, this is not by more than 20% from the baseline level. This is also the case with respect to the new baseline level at the second randomization visit. Reductions in insulin dose at any other time will not usually be greater than 10% from current levels. In Period 1, participants requiring an insulin dose reduction by > 20% from baseline and with total resulting insulin dose < 0.4 U/kg (current weight) are monitored closely for insulin dose, carbohydrate intake, ketones and blood glucose. In Period 2, dapagliflozin/placebo is temporarily discontinued if insulin dose falls below this threshold and the participant is withdrawn from the study if this remains the case at review in 1 week.

#### 2.9.2 Ketone Monitoring

In Period 2, participants are supported and encouraged to monitor urine or blood ketones at home at least once daily: 3 days before visit 10 (week 26), they are asked to start daily measurements of urine ketones or blood ketones (using specified strips and devices) and record these in the study diary provided. In addition, ketones (blood and urine) are measured at every visit. If ketones in urine are +1 or greater (≥ 0.6 mM in blood), participants are advised to keep well hydrated and notify the study team as soon as possible due to the risk of ketoacidosis. They are asked to follow written safety advice (provided in study diaries) and interrupt dapagliflozin temporarily if ketones are found to be raised (fasting urine > + or blood > 0.6 mmol), informing study staff.

If ketones are +2 or more in urine (> 1.5 mmol/L) participants are advised to initiate the STop SGLT‐2, Insulin administration, Carbohydrate consumption Hydration (STICH) Protocol immediately and contact the study team [30]. This advice is reinforced verbally at study visits and also via the Patient Alert Card. If blood ketones remain at these levels, study medication is permanently discontinued. If blood ketone readings are confirmed as > 3.0 mmol/L (or urine ketones reading +++) participants are advised to stop dapagliflozin/placebo and seek emergency medical care without delay.

#### 2.9.3 Weight Management

As excessive weight loss may lead to increased risk of hypoglycaemia or decreased insulin requirement and increase the risk of ketoacidosis, weight loss during any phase of the study will be limited to ≤ 15% reduction in body weight from randomization or reaching a BMI < 22 kg/m^2^. In participants approaching this threshold, IMP can be sequentially reduced at the discretion of the site Principal Investigator with semaglutide reduced to 0.25 or 0.5 mg weekly and dapagliflozin to 5 mg daily.

### 2.10 Statistical Considerations

For the preparation of all analyses, including in Period 1 with open‐label semaglutide, the study statistician will remain masked to treatment allocation until after the statistical analysis plan has been agreed and the database locked. The original sample size calculation assumed HbA1c reduction in the semaglutide and dapagliflozin arm in Period 2 by a mean of 0.5% (SD ≤ 0.7) compared with semaglutide alone. This required 38 patients (32 evaluable) in each group to provide 80% power at a 5% significance level, taking into consideration a 15% drop‐out rate. Given the overall study design, this equated to randomizing 114 participants in Period 1. 50% of participants were to be recruited in Glasgow, UK and 50% at State University of New York at Buffalo, USA. A sample size reduction was recommended by the trial oversight committees during the trial (see Section 2.13 below).

Four efficacy and four corresponding safety populations will be defined to address the pre‐specified primary, secondary, tertiary and safety outcomes analysed during the two periods of the trial. All efficacy outcomes will be analysed using the relevant modified intention‐to‐treat (mITT) population, including all patients from the respective ITT populations with data available (without imputation) for analysis of each individual outcome. The primary outcome and key secondary outcomes [(i), (ii), (v), (ix) and (xi) in Table 1] will be additionally analysed using the relevant per protocol populations (excluding any participants affected by major protocol deviations relevant to the outcome being analysed) if different from the mITT population. Linear regression models will be used for analysis with change from baseline as the outcome and predictor variables of treatment group, stratification factors and the baseline value. Transformations of non‐normally distributed variables will be considered prior to analysis.

Given the inclusion of an interim analysis which used a 1‐sided *p*‐value < 0.0034 for efficacy (see below), the estimate for the main effect of treatment (dapagliflozin—placebo) in the final analysis of the primary outcome will be deemed statistically significant with a 1‐sided *p*‐value < 0.0239 to maintain the overall (two‐sided) alpha of 5%. The treatment estimate will be reported with the corresponding 95% confidence interval. For all other outcomes, the treatment effect estimate and corresponding 95% confidence interval and *p*‐value will be reported using two‐sided tests and significance level of 5%. Tertiary analyses will be carried out using imputation for missing data for individuals having to terminate the HFHC mixed meal challenge test early due to pre‐specified hyperglycaemia criteria (see above Section 2.7.3). In this instance, missing observations will use last observation carried forward when calculating the AUC.

In the evaluation of safety outcomes, over‐dispersion will be accounted for using a Negative‐Binomial regression model (where possible), with the logarithm of observation time as an offset variable. The Incidence Rate Ratio, corresponding 95% confidence interval, and *p*‐value will be reported.

Subgroup analyses will be conducted on the primary outcome, key secondary glycaemic outcomes and safety outcome (i) ‘Rate of Level 2 (< 3.0 mmol/L/< 54mg/dL) hypoglycaemic events’; (where possible) adjusting for each participant's usual method of: (a) insulin delivery; and (b) glucose monitoring.

An exploratory sensitivity analysis for the primary outcome will additionally be carried out after using multiple imputation to impute missing week 52 HbA1c values in the event the participant had not died for participants that had week 25 HbA1c available. The multiple imputation will generate 100 complete data sets using SAS PROC MI and results combined based on Rubin's rules using SAS PROC MIANALYZE. The multiple imputation approach will use the baseline covariates included in the primary outcome model (HbA1c as recorded at week 25 and study site), imputing missing outcomes for each relevant randomization group separately.

A similar exploratory approach will also be used to impute missing outcome HbA1c for the secondary outcome analyses, as well as missing outcome percentage time in range, body weight and total daily insulin dose, using the relevant baseline values for each missing outcome imputation.

### 2.11 Trial Progress and Challenges

The initial participants were recruited at the US site prior to the worldwide COVID‐19 pandemic in February 2020. In the UK site, recruitment did not commence until April 2021 as all non‐vaccine or COVID‐related clinical trials were halted during and for several months after the pandemic. At this time, in response to financial difficulties, the funder had to implement a 10% budget cut—this was fortunately offset by a donation from Novo Nordisk UK. The first UK participant was randomized in June 2021 but many potential participants in both sites remained reluctant to visit hospitals or to give their time to clinical research, often in the context of personal difficulties relating to the pandemic. Recruitment, already behind anticipated targets, was then impacted by global supply issues of semaglutide from September 2022. As at this time, semaglutide could not be purchased on the UK market, and as a secure supply of semaglutide could not be guaranteed for any new participant randomized, a cap on enrolment per month was enforced by the UK Sponsor for several months until the above‐mentioned donation of semaglutide was received. At the US site, increasing ‘off‐label’ use of semaglutide in routine care was a further key factor impacting recruitment.

Changes to the standard of care during a prolonged recruitment phase were also a challenge at both sites with increasing adoption of hybrid closed‐loop systems into routine use. On the recommendation of the IDMC, participants who changed insulin delivery system during the trial were withdrawn to mitigate the potential confounding effect on glycaemic endpoints. The IDMC also recommended the sensitivity analyses outlined above according to subgroups of main method of insulin delivery and main method of glucose monitoring.

### 2.12 Protocol Amendments

As a result of the above issues, seven amendments to the original protocol were required at both the UK and US sites (see Tables S1 and S2 respectively): final versions of the Protocol at each site were numbered 8.0.

### 2.13 Sample Size Reduction

When 59 participants had been randomized in Period 1, the TSC asked the trial statisticians (May 2023) in response to slow recruitment to undertake a review of the required sample size based on masked review of primary outcome data (SD of change of HbA1c; correlation between HbA1c data at baseline and end of Period 2). No unblinded data were used for this review. This analysis supported a reduction in the sample size by a factor of 0.59, that is, from 32 to 19 evaluable patients per group. This recommendation was reviewed by the IDMC in August 2023, by the TSC in September 2023 and at an additional IDMC meeting in October 2023. Maintaining the same assumptions on drop‐out, it was calculated that 23 rather than 32 evaluable participants were required per group in Period 2, reducing the overall recruitment target from 114 to 82. Following endorsement of this recommendation by both committees, this change was implemented from Protocol Version 6.0 (UK) and Protocol Version 7.0 (US).

### 2.14 Interim Analysis

As recruitment remained slow, the IDMC further required a formal interim analysis on all those with mature data on the primary outcome at the end of April 2024 with a plan for recruitment to continue (unless formally stopped by the funder) following a subsequent recommendation by the TSC on the basis of either: (a) overwhelming evidence of benefit; or (b) futility; and provided the study had recruited sufficient numbers between November 2023 and April 2024 to make reaching the new target in a reasonable time feasible within budget. The specified single interim analysis on the primary outcome (Period 2) used linear regression analysis of the change in HbA1c at week 52 from week 25, adjusting for the stratification variables, HbA1c at week 25 and study site. It was performed by unblinded statisticians using a group sequential design and included both efficacy and futility boundaries for early stopping. The Lan‐DeMets spending function approximation to O'Brien‐Fleming boundaries was used to obtain the required boundary values given the available sample size at the time of the interim analysis while retaining the overall power of 80%.

Based on the treatment effect estimate, corresponding 95% confidence interval, *p*‐value and *z*‐score provided, along with the obtained boundary values and their corresponding *p*‐values, the IDMC recommended following its seventh meeting (9th May 2024) to the TSC that the trial should continue as described above (Section 2.10).

## 3 Results and Discussion

TTT1 is the first clinical trial to assess the efficacy and safety of combination therapy with a GLP‐1RA and an SGLT‐2 inhibitor (semaglutide and dapagliflozin respectively) in overweight and obese people with T1D and glycaemia above target levels as measured by HbA1c.

Dapagliflozin inhibits the SGLT‐2 channel in the proximal convoluted tubule, inducing glycosuria by lowering the renal threshold for glucose re‐absorption. At steady state, this results in urinary loss of glucose equivalent to ≈200 kcal per day, lowering blood glucose and promoting weight loss [31]. There is robust evidence that dapagliflozin reduces rates of cardiovascular events [32] in people with and without T2D and with and without heart failure (whether with reduced or preserved left ventricular function). T1D is associated with reduced life expectancy in large part due to cardiovascular disease [33]. There have been no cardiovascular outcome trials in T1D per se, but it is highly plausible that effects demonstrated in other populations could be extrapolated to T1D if dapagliflozin could be used safely in this condition [34, 35, 36]. A further advantage of this strategy would be the beneficial effects of dapagliflozin on renal outcomes, which have again been observed in people with and without T2D [14, 37].

SGLT‐2 inhibitors are, however, associated with ketosis and ketoacidosis in people with T2D. This can occur in the absence of hyperglycaemia and is thought to be driven by a decrease in the insulin: glucagon ratio due to counterregulatory glucagon secretion (and enhanced fatty acid oxidation) in response to urinary glucose loss [38]. In the large Phase 3 trials with dapagliflozin and other agents, absolute numbers of cases of ketoacidosis were low, but relative risk was increased several‐fold [38]. Dapagliflozin briefly gained a licensed indication as an adjunct agent in T1D in Europe, but this was withdrawn by the manufacturer in 2019 [15] during the COVID‐19 pandemic; of note, it has never had a licence for T1D in the USA. In TTT1, all participants randomized to dapagliflozin or placebo have already been treated with semaglutide for 26 weeks as it is hypothesized that GLP‐1RA inhibition of glucagon secretion may mitigate ketosis [23]. In addition, participants are selected as: being proficient in self‐monitoring, carbohydrate counting and insulin dose adjustment; educated on the use of the STICH Protocol*; and supported to monitor urine and blood ketone levels closely [30]. Ketosis and ketoacidosis are important pre‐specified safety outcomes.

GLP‐1 RAs are increasingly prescribed for people with T2D and/or obesity. Whether released endogenously from the L‐cells of the small intestine as the native hormone or supplemented pharmacologically, circulating GLP‐1 acts on its receptor to promote satiety, decrease appetite and thereby decrease caloric intake, reducing glycaemic exposure. Gastrointestinal effects include reduction in gastric and intestinal motility, further reducing oral intake, encouraging weight loss and reducing blood glucose. In the pancreatic islets, activation of GLP‐1 receptors on α‐cells is thought to reduce dysfunctional glucagon secretion/hyperglucagonemia [39, 40]. As discussed above, this may be a beneficial effect in the context of SGLT‐2 inhibition in T1D, potentially mitigating the risk of ketosis and ketoacidosis [23].

Most previous trials of GLP‐1 RAs in T1D have investigated daily agents, for example liraglutide, rather than weekly semaglutide. In the large Phase 3 trials, ADJUNCT‐ONE and ADJUNCT‐TWO, there were unacceptable rates of hypoglycaemia and severe hypoglycaemia with liraglutide, potentially attributable to the use of relatively simple insulin dose adjustment rules [41]. In TTT1, hypoglycaemia is mitigated by a strategy of individual insulin dose adjustments made frequently in response to individual blood glucose data at in‐person and telephone study visits. It is further mitigated by advances in blood glucose monitoring as standard of care since the above‐mentioned trials were conducted: many TTT1 participants use CGM and FGM technology with low glucose alarms [42, 43] rather than fingerstick monitoring, reducing the risk of hypoglycaemia [44].

## 4 Conclusion

The TTT1 trial will provide clinically useful information on the efficacy and safety of combination adjunct therapy, adding in the weekly subcutaneous GLP‐1 RA semaglutide and the daily oral SGLT‐2 inhibitor dapagliflozin with standard of care insulin in people with T1D. Comparison of the efficacy of triple therapy (semaglutide and dapaglifozin added in with standard of care insulin therapy) vs. dual therapy (semaglutide and standard of care insulin therapy) on HbA1c is the primary outcome, but the sequential trial design also provides direct and indirect evidence on the effects of triple therapy and dual therapy individually vs. standard of care insulin therapy. If these highly effective glucose‐ and weight‐lowering agents can be used safely and in combination in T1D, it is likely that substantial cardiorenal benefits can be realized in a population at high risk.

## Author Contributions

**Manav A. Batra:** funding acquisition, investigation, review and editing. **Caroline Bonner:** funding acquisition, review and editing. **James G. Boyle:** funding acquisition, supervision, investigation, review and editing (UK co‐investigator). **Ajay Chaudhuri:** investigation, review and editing. **Paresh Dandona:** conceptualization, funding acquisition, methodology, supervision, review and editing (US Chief Investigator). **Husam Ghanim:** conceptualization, funding acquisition, methodology, project administration, supervision, investigation, review and editing (US co‐investigator). **Nicola Greenlaw:** methodology, review and editing. **Lisa Jolly:** project administration, review and editing. **Robert S. Lindsay:** funding acquisition, methodology, review and editing. **Alex McConnachie:** methodology (Lead Statistician), supervision, review and editing. **John R. Petrie:** conceptualization, funding acquisition, methodology, project administration, supervision, investigation, writing original draft (supporting) (UK Chief Investigator). **Joseph G. Timmons:** writing – original draft (lead), investigation, methodology, visualization, review and editing.

## Funding

This work was supported by JDRF, 3‐SRA‐2019‐669‐M‐B.

## Conflicts of Interest

The authors declare no conflicts of interest. Caroline Bonner: Dr. Bonner declares no conflict of interest. James Boyle: Prof. Boyle reports personal fees from Elsevier (textbook royalties) and Korea University (education expert advisory) both outside the submitted work. Ajay Chaudhuri: Dr. Chaudhuri reports: research support from Lilly and Novo Nordisk; and personal fees from Lilly and Novo Nordisk (consultancy). Paresh Dandona: Prof. Dandona declares no conflict of interest. Husam Ghanim: Dr. Ghanim reports donation of study medication (dapagliflozin and matching placebo) for the US site from Astra Zeneca (relevant to the work); and receipt of discounted CGM sensors from Dexcom (relevant to the work). Nicola Greenlaw: Ms. Greenlaw declares no conflict of interest. Lisa Jolly: Ms. Jolly declares no conflict of interest. Alex McConnachie: Prof McConnachie declares no conflict of interest. Robert Lindsay: Dr. Lindsay declares no conflict of interest. John R. Petrie: Prof. Petrie reports: donation of active semaglutide study medication for the trial during a period of market supply problems (relevant to the work); personal fees from Merck KGaA (preparation of a review article); personal fees (via his employing institution) from IQVIA (Boehringer Ingelheim Adjudication Committees); personal fees from the Academy of Finland (funding panel membership); personal fees from the Danish Diabetes and Endocrine Academy (grant reviewing); personal fees from Elsevier (textbook royalties); and travel support from Sanofi—all outside the submitted work. Joseph Timmons: Dr. Timmons reports no conflict of interest.

## Supporting information


**Table S1:** Entry criteria.

**Table S2:** Summary of amendments to UK protocol.

**Table S3:** Summary of amendments to US protocol.

**Supporting information 1:** TTT1 all UK protocol versions March 2026.

**Supporting information 2:** TTT1 all US protocol versions March 2026.

## Data Availability

This protocol paper includes only baseline characteristics of participants. Main trial results data will be published in a forthcoming separate paper which will include a Data Availability Statement.
