# Supplementary material for: Insulin, Semaglutide and Dapagliflozin in Adults With Type 1 Diabetes: Design and Methods of Triple Therapy for Type 1 Diabetes (TTT1)—An International Phase 3 Clinical Trial
Source: Diabetes Obes Metab. 2026 Jul 19;28(10):8812–24. doi: 10.1111/dom.71076 (PMC13538731; doi:10.1111/dom.71076)
Supplement: Supplementary file 1 [file DOM-28-8812-s001.pdf]

University at Buffalo
The State University of New York

University at Buffalo Institutional Review Board (UBIRB)
Office of Research Compliance | Clinical and Translational Research Center Room 5018
875 Ellicott St. | Buffalo, NY 14203
UB Federalwide Assurance ID#: FWA00008824

Complete Research Protocol (HRP-503)

Table of Contents

Template Instructions 2
1.0 Objectives 4
2.0 Scientific Endpoints 5
3.0 Background 6
4.0 Study Design 12
5.0 Local Number of Subjects 13
6.0 Inclusion and Exclusion Criteria 13
7.0 Vulnerable Populations 16
8.0 Eligibility Screening 17
9.0 Recruitment Methods 18
10.0 Procedures Involved 19
11.0 Study Timelines 31
12.0 Setting 31
13.0 Community-Based Participatory Research 32
14.0 Resources and Qualifications 33
15.0 Other Approvals 34
16.0 Provisions to Protect the Privacy Interests of Subjects 34
17.0 Data Management and Analysis 35
18.0 Confidentiality 38
A. Confidentiality of Study Data 38
B. Confidentiality of Study Specimens 39
19.0 Provisions to Monitor the Data to Ensure the Safety of Subjects 40
20.0 Withdrawal of Subjects 44
21.0 Risks to Subjects 45
22.0 Potential Benefits to Subjects 50
23.0 Compensation for Research-Related Injury 51
24.0 Economic Burden to Subjects 51
25.0 Compensation for Participation 52
26.0 Consent Process 53
27.0 Waiver or Alteration of Consent Process 57
28.0 Process to Document Consent 58
29.0 Multi-Site Research (Multisite/Multicenter Only) 58
30.0 Banking Data or Specimens for Future Use 59
31.0 Drugs or Devices 60
32.0 Humanitarian Use Devices 62

Template Instructions

Sections that do not apply:

- *In several sections, the addition of checkboxes for* ***Not Applicable*** *have been added to the template as responses.*
    - *If an N/A checkbox is present, select the appropriate justification from the list.*
    - *If an N/A checkbox is not present, or if none of the existing checkboxes apply to your study, you must write in your own justification.*
- *In addition:*
    - *For research where the only study procedures are records/chart review: Sections 19, 20, 22, 23, 24, 25, 31, and 32 do not apply.*
    - *For exempt research: Sections 31 and 32 do not apply.*

Studies with multiple participant groups:

- *If this study involves multiple participant groups (e.g. parents and children), provide information in applicable sections for each participant group. Clearly label responses when they differ. For example:*

Response:

<u>Intervention Group:</u>

<u>Control Group:</u>

Formatting:

- *Do not remove template instructions or section headings when they do not apply to your study.*

If you are pasting information from other documents using the "Merge Formatting" Paste option will maintain the formatting of the response boxes.

Amendments:

- *When making modifications or revisions to this and other documents, use the* ***Track Changes*** *function in Microsoft Word.*
- *Update the version date or number* ***on Page 3.***

PROTOCOL TITLE:

Include the full protocol title.

Response: **Triple Therapy for Type 1 Diabetes with Insulin, Semaglutide, and Dapagliflozin**

PRINCIPAL INVESTIGATOR:

Response:

**Principal Investigator:**	**Paresh Dandona, MBBS, PhD**
**Co- Investigators:**	**Husam Ghanim, PhD**
	**Antoine Makdissi, MD**
	**Manav Batra, MD**
**Co- Principal Investigator:**	**John R Petrie, MBChB, PhD**

Paresh Dandona, M.B.B.S., Ph.D., F.R.C.P, F.A.C.P, F.A.C.C, FACE,
SUNY Distinguished Professor,
Director, Diabetes-Endocrinology Center of Western NY
Chief of Endocrinology, Diabetes, Endocrinology and Metabolism, SUNY at Buffalo
1000 Youngs Road, Suite 105
Williamsville NY 14221
Telephone: (716) 535-1850
Fax: 716-639-1200
E-mail: pdandona@kaleidahealth.org

CO-PI:
Professor John R Petrie
BSc MBChB PhD FRCP(Ed) FRCPSG
Professor of Diabetic Medicine
Institute of Cardiovascular and Medical Sciences
BHF Glasgow Cardiovascular Research Centre
University of Glasgow
126 University Place
Glasgow G12 8TA
Tel: +44(0)141 330 3325
Email: john.petrie@glasgow.ac.uk
Web: http://www.gla.ac.uk/researchinstitutes/icams/staff/johnpetrie/
The University of Glasgow

VERSION:

Include the version date or number.

Response: 1/15/2019, V1

GRANT APPLICABILITY:

Indicate whether this protocol is funded by a grant (e.g. NIH, foundation grant). For a grant with multiple aims, indicate which aims are covered by this research proposal.

NOTE: This question does not apply to studies funded by a sponsor contract.

Include a copy of the grant proposal with your submission.

Response: Research support provided by JDRF and AZ

RESEARCH REPOSITORY:

Indicate where the research files will be kept, including when the study has been closed. The repository should include, at minimum, copies of IRB correspondence (approval, determination letters) as well as signed consent documents. This documentation should be maintained for 3 years after the study has been closed.

Response:
Site 1: Diabetes Endocrinology Research Center of WNY
Address: 1000 Youngs Road, Suite 105, Williamsville NY 14221
Department: Diabetes Endocrinology

Site 2: University of Glasgow. Institute of Cardiovascular and Medical Sciences University of Glasgow 126 University Place Glasgow, G12 8TA

1.0 Objectives

1.1 Describe the purpose, specific aims, or objectives of this research.

Response:

Primary Objective: To assess whether the addition of dapagliflozin to semaglutide and insulin (triple therapy) improves glycemic control in patients with type 1 diabetes compared with semaglutide and insulin (dual therapy) and insulin only (standard) treatment.

General hypothesis: The addition of dapagliflozin to semaglutide and insulin treatment improves glycemic control, weight loss and blood pressure in adults with type 1 diabetes.

1.2 State the hypotheses to be tested, if applicable.

NOTE: A hypothesis is a specific, testable prediction about what you expect to happen in your study that corresponds with your above listed objectives.

Response:

Specific hypotheses:

Primary: combined dapagliflozin and semaglutide treatment in addition to insulin will improve HbA1c levels compared with semaglutide in addition to insulin (by ≥0.5%) or insulin therapy alone (by ≥1%).

Secondary 1: semaglutide in addition to insulin treatment over 26 and 52 weeks will improve HbA1c levels compared with insulin therapy alone.

Secondary 2: combined dapagliflozin and semaglutide treatment in addition to insulin will improve CGM based weekly mean and fasting blood glucose, glycemic oscillations and percent of time in range (80-160mg/dl) with lower insulin requirements compared with semaglutide and insulin (dual therapy) or insulin only (standard) treatment.

Secondary 3: combined dapagliflozin and semaglutide treatment in addition to insulin therapy will improve postprandial glucose excursions and suppress postprandial glucagon compared with semaglutide and insulin or insulin therapy alone.

Secondary 4: combined dapagliflozin and semaglutide in addition to insulin therapy will reduce systolic and diastolic blood pressure compared with semaglutide and insulin (dual therapy) or insulin only (standard) treatment.

Secondary 5: combined dapagliflozin and semaglutide in addition to insulin therapy (triple therapy) will result in weight loss compared to dual and standard therapy.

Secondary 6: combined dapagliflozin and semaglutide in addition to insulin therapy will result in improved scores on the Diabetes Specific Quality of Life Scale (DSQOLS) and the Problem Areas In Diabetes (PAID) survey compared with dual and standard therapy.

Exploratory Objectives (EO) for future ancillary studies (PLEASE NOTE: samples will be collected to facilitate the measurements of these end points in future ancillary studies once submitted and approved by JDRF so cost of assays is not included in the current budget) : The addition of dapagliflozin to semaglutide and insulin in type 1 diabetes will:

EO 1: increase plasma concentrations of vasodilatation mediators and reduce plasma concentrations of vasoconstriction mediators compared to dual and standard therapy groups.

EO 2: exert a systemic anti-inflammatory effect including reducing mRNA expression of inflammatory mediators and NFκB binding in peripheral mononuclear cells (MNC) and adipose tissue (optional) and their circulating levels in plasma while increasing levels of IL-RA and adiponectin.

2.0 Scientific Endpoints

2.1 Describe the scientific endpoint(s), the main result or occurrence under study.

NOTE: Scientific endpoints are outcomes defined before the study begins to determine whether the objectives of the study have been met and to draw conclusions from the data. Include primary and secondary endpoints. Some example endpoints are: reduction of symptoms, improvement in quality of life, or survival. Your response should ***not*** *be a date.*

Response:
Primary endpoint: Change in HbA1c at 6 months following dapagliflozin or placebo therapy in addition to combined semaglutide and insulin treatment.
Efficacy Secondary endpoints:

1. Change in HbA1c from baseline at 12 months in triple therapy group compared to insulin only group.
2. Change in HbA1c from baseline at six and 12 months in dual therapy group compared to insulin only group.
3. Change in percent Time in Range (70– 180 mg/dl), percent time in hyperglycemia Level 1(180-250mg/dl) and Level 2 (>250mg/dl) and percent time in hypoglycemia Level 1 (54-70mg/dl) as assessed by CGM
4. Other indices of glycemic control: fructosamine, weekly mean and fasting glucose, standard deviation, insulin requirements, postprandial glucose excursions and postprandial (Area Under curve) FFA, GLP-1 and glucagon.
5. Change in body weight and quality of life as assessed by DSQOLS and PAID at six and 12 months.
6. Anti-hypertensive effects including change in systolic and diastolic BP, numbers of BP medications required.

Safety Secondary endpoints:

1- Differences in rates of hypoglycemic events Level 2 (<54mg/dl) and Level 3 (A severe event characterized by altered mental and/or physical status requiring assistance) between investigative arms and standard therapy arm.

2- Differences in rates of diabetic ketoacidosis defined as elevated serum or urine ketones (greater than the upper limit of the normal range), and serum bicarbonate < 15 mmol/L or Blood pH < 7.3 between investigative arms and standard therapy arm

Tertiary/ exploratory endpoints: (to be performed in future on collected samples from this study if additional funding is approved by JDRF):

1. Indices of inflammatory stress. These will be CRP, FFA, SAA, IL-1β, IL-18, IL-1RA, adiponectin and TNFα concentrations in plasma and isoprostane in urine.
2. Expression of IL-1β, TNFα, IL-12, TLR2, TLR4, adiponectin, IκBα as well as NFκB binding in peripheral MNC and adipose tissue (optional).
3. Indices of vasodilatation and vasoconstriction: ANP, b-NP, cGMP, cAMP, angiotensinogen, renin and angiotensin II concentrations.

3.0 Background

3.1 Provide the scientific or scholarly background, rationale, and significance of the research based on the existing literature and how it will contribute to existing knowledge. Describe any gaps in current knowledge. Include relevant preliminary findings or prior research by the investigator.

Response:

The control of glucose homeostasis in type 1 diabetes is fragile since β-cell reserve is negligible. Traditionally, insulin has been the only treatment approach available. This approach, often leads to erratic blood sugar control given the challenge of matching exogenous insulin to mealtime glucose excursions in the face of a lack of any endogenous insulin to compensate for variation in requirements in response to meals and activity levels. Injected insulin boluses rarely match precise insulin requirements

either in terms of dose administered or bio-availability. The result often is wide fluctuations in blood glucose readings. Based on our work to date and other published reports, previous attempts to add other glucose-lowering medications to insulin therapy yielded minimal benefits on glycemia (although metformin may have other benefits (1-3)

We have recently shown in two preliminary retrospective studies that the addition of liraglutide, a Glucagon Like Peptide -1 receptor agonist (GLP-1RA), to insulin results in consistent improvements in glycemic control in type 1 diabetes as demonstrated by reductions in HbA1c, glycemic excursions, and insulin requirements accompanied by improvements in body weight and systolic blood pressure (4, 5). We have now also completed the first prospective randomized controlled trial of this drug in 72 people with type 1 diabetes. Our data not only confirmed improvements in glycemic control, HbA1c, body weight and systolic BP, but also demonstrated a reduction in appetite, carbohydrate intake in low grade inflammation as measured by plasma CRP concentrations (6). Importantly, quality of life was also improved. Our recent studies in type 2 diabetes have revealed that systolic blood pressure reduction with GLP-1RA (exenatide) treatment in type 2 diabetes is associated with increased plasma concentrations of ANP, cGMP and cAMP along with reduced concentrations of angiotensinogen, renin and angiotensin II (7). It is possible that these mechanisms play a role in the prevention of cardiovascular complications in type 1 diabetes.

While GLP-1RAs, therefore, have great potential as adjunct therapy for type 1 diabetes, mean HbA1c was still ≥7% after liraglutide treatment (6) and larger pharma-sponsored trials reported increased rates of hypoglycemia (8, 9). In seeking further improvement, the advent of sodium-glucose transport protein-2 (SGLT2) inhibitors is of importance since their mode of action is independent of insulinogenesis. Introduced for use in type 2 diabetes, SGLT2 inhibitors are selective antagonists of the sodium-glucose transporter subtype expressed in the proximal convoluted tubules of the kidneys. They act by inhibiting renal glucose re-absorption and therefore stimulating excretion of 60-100 grams per day of glucose in urine. SGLT2 inhibitors have been studied extensively in type 2 diabetes, including the landmark cardiovascular outcome trial EMPA-REG (10), but data have only quite recently begun to accumulate in type 1(11). Short term effects on urine glucose excretion of remogliflozin and empagliflozin are similar to those seen in type 2 diabetes and these translate into improved glycemic and other indices including HbA1c; fasting blood glucose, rates of hypoglycemia, daily insulin dose and weight (12). Weight loss accompanying increased urinary glucose excretion could translate into reduced cardiovascular complications as in type 2 diabetes, but there are no data so far available to support this hypothesis. More recently, in the Phase 3 trial DEPICT-1 we showed that dapagliflozin improves glycemic control and HbA1c over six months by about 0.45% with no severe hypoglycemia or diabetic ketoacidosis (13). Similarly, sotagliflozin has been shown in a Phase 3 trial to allow a larger proportion of patients to achieve HbA1c lower than 7.0% with no severe hypoglycemia although the rate of diabetic ketoacidosis was higher in the sotagliflozin group (14). Both products are now being reviewed by regulatory agencies for the type 1 diabetes indication.

On the basis of the above, we hypothesize that adding an SGLT2 inhibitor to a combination of insulin and semaglutide (a weekly and potent GLP-1RA) will improve glycemic control in type 1 diabetes such that HbA1c levels of ≤7% are achieved more consistently. Our protocol is designed to allow an objective assessment of whether this triple therapy combination will work effectively and safely so that a majority of people with type 1 diabetes can reach a glycemic target HbA1c of ≤7%. In addition, since both semaglutide and dapagliflozin inhibitors reduce blood pressure and weight, it will allow an assessment of the combined effect of GLP-1RAs and SGLT2 inhibitors on these outcomes. Since there are no previous data on the effects of once weekly semaglutide or the combination of semaglutide and dapagliflozin compared to standard insulin treatment in type 1 diabetes, the design requires allocation of some participants to insulin therapy only.

Preliminary data

1) Liraglutide treatment in patients with type 1 diabetes (4): We treated 8 type 1 diabetes patients; mean age (38.5 years) and duration of diabetes (20±4 yrs), with liraglutide in addition to insulin for 24 weeks. As shown in table 1, there was a significant reduction in fasting and mean blood glucose concentrations, insulin doses, HbA1c and a reduction in appetite and caloric intake with weight loss. In addition, there was a reduction in the excursions of blood glucose concentrations, i.e., the standard deviations of blood glucose concentrations were markedly reduced (**figure 1**). The changes were observed within 48h and improved marginally thereafter with fine tuning of insulin doses and a further reduction in these doses. These observations are noteworthy in that study participants were well controlled and meticulous users of CSII and CGM. They had all tried hard to improve their diabetic control by adjusting insulin doses but had not succeeded because of hypoglycemia. We then demonstrated that poorly controlled and obese individuals with type 1 diabetes also experienced similar benefits of liraglutide treatment (2).

2) Liraglutide As Additional Treatment to Insulin in Patients with Type 1 Diabetes Mellitus: A Randomized Clinical Trial (6): Following the two above non-randomized studies we recently conducted a randomized clinical trial in which 72 individuals (Placebo=18; liraglutide = 54) with TYPE 1 DIABETES for at least one year, on insulin therapy and with no detectable C-peptide in plasma (mean BMI: 30±1; mean body weight: 84±2 kg ; mean HbA1c: 7.57±0.09%; mean age: 44±2 years; mean age of TYPE 1 DIABETES diagnosis: 20±1 years) were randomized to receive placebo, 0.6, 1.2 or 1.8mg of liraglutide daily for 12 weeks. In the 1.2 mg and 1.8 mg groups, mean change in average blood glucose was -10±2 and -10.0±1mg/dl, respectively, ($p<0.0001$ vs. placebo). In the 1.2mg group, HbA1c fell by 0.78% from 7.84±0.17% to 7.06±0.15% ($p<0.0001$, $p<0.01$ vs. placebo) and in the 1.8mg group fell by 0.42% from 7.41±0.15 to 6.99±0.15 ($p=0.001$, $p=0.39$ vs placebo). Time spent between 70 to 160 mg/dl increased by 5±1% ($p<0.05$ vs placebo) and time between 160-400 mg/dl decreased by 7±1% ($p<0.01$ vs placebo) in the 1.8 mg group with no additional hypoglycemia. During the 12 week period, the average total daily dose of insulin in the two groups fell by 12.4±3.9 and 10.0±2.3 units respectively ($p<0.05$ vs baseline and placebo). There was a reduction in body weight (from 83±4 to 78±5 kg, $p<0.0001$, in the 1.8mg group, from 96±4 to 91±4 kg, $p<0.001$, in the 1.2 mg group and from 80±4

to 77±4 kg, p<0.01 in the 0.6 mg group). A reduction in daily carbohydrate intake (171±17g vs. 127±18g; p<0.01 and 153±18g vs 115±16g, p<0.01 in the 1.2 mg and 1.8 mg groups, respectively) was also observed over 12 weeks. Systolic BP (SBP) in the 1.8 mg group fell by 3 mmHg (120±2 to 117±3 mmHg, p=0.01). CRP fell by 15±6 and 19±8% in the 1.2 and 1.8mg groups, respectively (p<0.05). Quality of life improved significantly compared to baseline and placebo (unpublished data).

3) Dapagliflozin as an Additional Treatment to Insulin and Liraglutide in Patients with Type 1 Diabetes Mellitus (15): The objective of this randomized clinical trial was to investigate whether addition of dapagliflozin to insulin and liraglutide results in a significant reduction in glycemia and body weight. 30 participants with TYPE 1 DIABETES on liraglutide therapy for at least last 6 months were randomized (in 2:1 ratio) to receive either dapagliflozin 10 mg or placebo daily for 12 weeks. With 12 weeks of dapagliflozin HbA1c fell by 0.66±0.08% from 7.8±0.21% (p < .01 vs placebo) but did not change significantly with placebo (7.40±0.20% to 7.30±0.20%). In one patient within one week of addition of dapagliflozin average glucose fell from 170 to 122 mg/dl while standard deviation was reduced from 71 to 58 (**Figure 2-diminished glycemic excursions)**. Body weight fell by1.9 +/- 0.54kg (p < 0.05 vs placebo). There was no additional hypoglycemia (blood glucose < 3.88 mmol/L; p = 0.52 vs placebo). In the dapagliflozin group, there were significant increases in the plasma concentrations of glucagon by 35% +/- 13% (p < 0.05), hormone-sensitive lipase by 29% +/- 11% (p < 0.05), free fatty acids by 74% +/- 32% (p < 0.05), acetoacetate by 67% +/- 34% (p < 0.05), and beta-hydroxybutyrate by 254% +/- 81% (p < 0.05). Urinary ketone levels also increased significantly (P < .05). None of these changes was observed in the placebo group. Two individuals in the dapagliflozin group developed ketoacidosis. Overall, addition of dapagliflozin to insulin and liraglutide in patients with TYPE 1 DIABETES resulted in a significant improvement in glycemia and in weight loss but tended to increase ketosis.

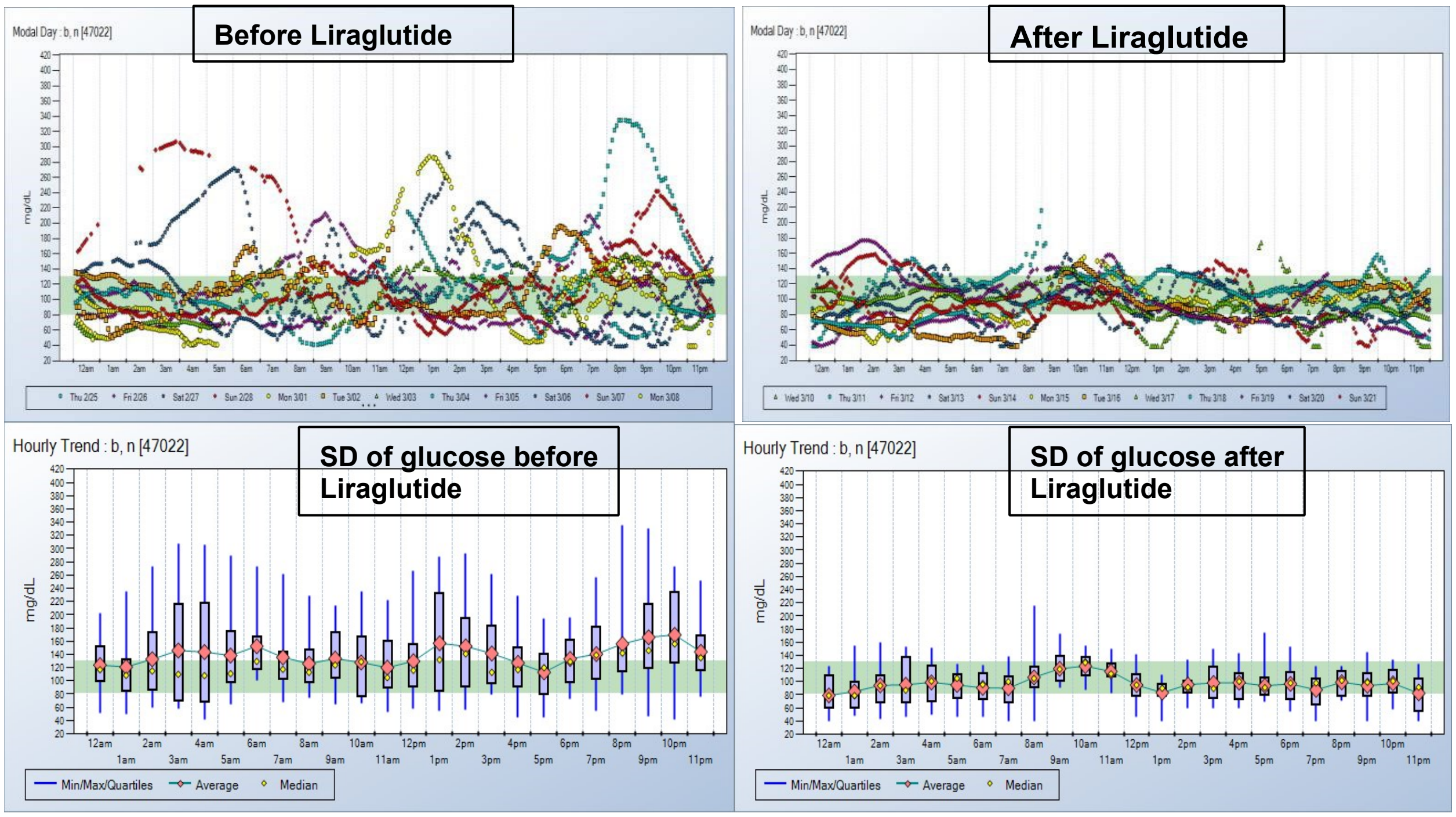

Figure 1: Weekly continuous glucose monitoring record of one patient prior to and following 1 week of liraglutide. Note the glycemic variability (SD) and the frequency of glucose concentrations within the target range prior to and following liraglutide

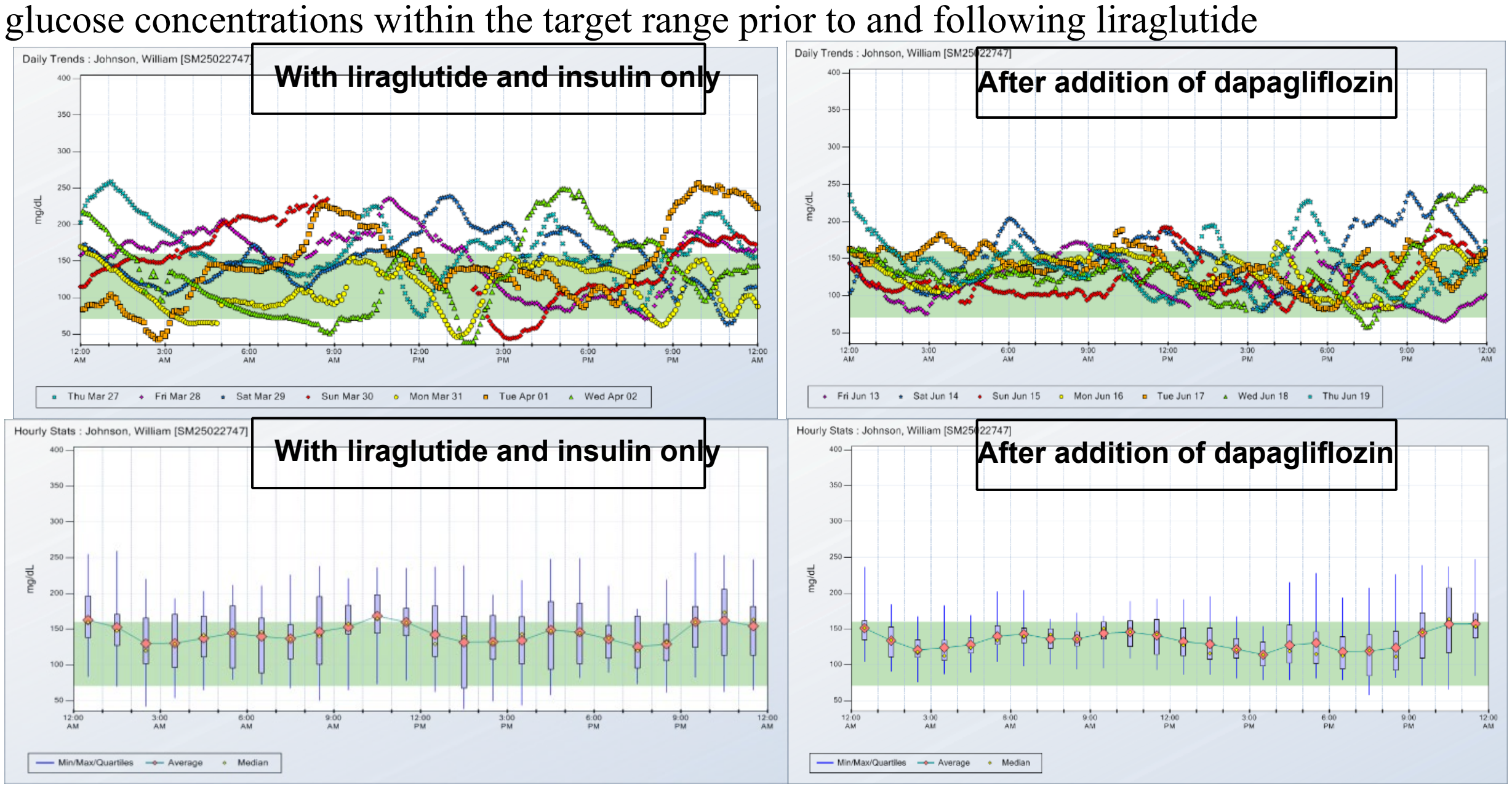

Figure 2: Weekly continuous glucose monitoring record of one patient prior to and following 8 weeks of the triple therapy combination with dapagliflozin and liraglutide. Note the glycemic variability (SD) (bottom) and the frequency of glucose concentrations within the target range prior to and following dapagliflozin addition.

3.2 Include complete citations or references.

Response:

1. Konrad K, Datz N, Engelsberger I, Grulich-Henn J, Hoertenhuber T, Knauth B, et al. Current use of metformin in addition to insulin in pediatric patients with type 1 diabetes mellitus: an analysis based on a large diabetes registry in Germany and Austria. *Pediatr Diabetes.* 2014.
2. Vella S, Buetow L, Royle P, Livingstone S, Colhoun HM, and Petrie JR. The use of metformin in type 1 diabetes: a systematic review of efficacy. *Diabetologia.* 2010;53(5):809-20.
3. Vella S, Buetow L, Royle P, Livingstone S, and Petrie JR. Metformin in type 1 diabetes reduces insulin requirements without significantly improving glycaemic control. Reply to Schatz H [letter]. *Diabetologia.* 2011;54(1):203-4.
4. Varanasi A, Bellini N, Rawal D, Vora M, Makdissi A, Dhindsa S, et al. Liraglutide as additional treatment for type 1 diabetes. *Eur J Endocrinol.* 2011;165(1):77-84.
5. Kuhadiya ND, Malik R, Bellini NJ, Patterson JL, Traina A, Makdissi A, et al. Liraglutide as additional treatment to insulin in obese patients with type 1 diabetes mellitus. *Endocrine practice : official journal of the American College of Endocrinology and the American Association of Clinical Endocrinologists.* 2013;19(6):963-7.
6. Kuhadiya ND, Dhindsa S, Ghanim H, Mehta A, Makdissi A, Batra M, et al. Addition of Liraglutide to Insulin in Patients With Type 1 Diabetes: A Randomized Placebo-Controlled Clinical Trial of 12 Weeks. *Diabetes Care.* 2016;39(6):1027-35.
7. Chaudhuri A, Ghanim H, Makdissi A, Green K, Abuaysheh S, Batra M, et al. Exenatide induces an increase in vasodilatory and a decrease in vasoconstrictive mediators. *Diabetes Obes Metab.* 2017;19(5):729-33.
8. Mathieu C, Zinman B, Hemmingsson JU, Woo V, Colman P, Christiansen E, et al. Efficacy and Safety of Liraglutide Added to Insulin Treatment in Type 1 Diabetes: The ADJUNCT ONE Treat-To-Target Randomized Trial. *Diabetes Care.* 2016;39(10):1702-10.
9. Ahren B, Hirsch IB, Pieber TR, Mathieu C, Gomez-Peralta F, Hansen TK, et al. Efficacy and Safety of Liraglutide Added to Capped Insulin Treatment in Subjects With Type 1 Diabetes: The ADJUNCT TWO Randomized Trial. *Diabetes Care.* 2016;39(10):1693-701.
10. Zinman B, Wanner C, Lachin JM, Fitchett D, Bluhmki E, Hantel S, et al. Empagliflozin, Cardiovascular Outcomes, and Mortality in Type 2 Diabetes. *N Engl J Med.* 2015;373(22):2117-28.
11. Petrie JR. SGLT2 inhibitors in type 1 diabetes: knocked down, but up again? *Lancet Diabetes Endocrinol.* 2017;5(11):841-3.
12. Mudaliar S, Armstrong DA, Mavian AA, O'Connor-Semmes R, Mydlow PK, Ye J, et al. Remogliflozin etabonate, a selective inhibitor of the sodium-glucose transporter 2, improves serum glucose profiles in type 1 diabetes. *Diabetes Care.* 2012;35(11):2198-200.
13. Dandona P, Mathieu C, Phillip M, Hansen L, Griffen SC, Tschope D, et al. Efficacy and safety of dapagliflozin in patients with inadequately controlled type 1 diabetes

(DEPICT-1): 24 week results from a multicentre, double-blind, phase 3, randomised controlled trial. *Lancet Diabetes Endocrinol.* 2017;5(11):864-76.
14. Garg SK, Henry RR, Banks P, Buse JB, Davies MJ, Fulcher GR, et al. Effects of Sotagliflozin Added to Insulin in Patients with Type 1 Diabetes. *N Engl J Med.* 2017;377(24):2337-48.
15. Kuhadiya ND, Ghanim H, Mehta A, Garg M, Khan S, Hejna J, et al. Dapagliflozin as Additional Treatment to Liraglutide and Insulin in Patients With Type 1 Diabetes. *J Clin Endocrinol Metab.* 2016;101(9):3506-15.
16. Henry RR, Thakkar P, Tong C, Polidori D, and Alba M. Efficacy and Safety of Canagliflozin, a Sodium-Glucose Cotransporter 2 Inhibitor, as Add-on to Insulin in Patients With Type 1 Diabetes. *Diabetes Care.* 2015;38(12):2258-65.
17. Peters AL, Buschur EO, Buse JB, Cohan P, Diner JC, and Hirsch IB. Euglycemic Diabetic Ketoacidosis: A Potential Complication of Treatment With Sodium-Glucose Cotransporter 2 Inhibition. *Diabetes Care.* 2015;38(9):1687-93.
18. Ahmann AJ, Capehorn M, Charpentier G, Dotta F, Henkel E, Lingvay I, et al. Efficacy and Safety of Once-Weekly Semaglutide Versus Exenatide ER in Subjects With Type 2 Diabetes (SUSTAIN 3): A 56-Week, Open-Label, Randomized Clinical Trial. *Diabetes Care.* 2017.
19. Andrews NC, and Faller DV. A rapid micropreparation technique for extraction of DNA-binding proteins from limiting numbers of mammalian cells. *Nucleic Acids Res.* 1991;19(9):2499.
20. Perkins BA, Cherney DZ, Partridge H, Soleymanlou N, Tschirhart H, Zinman B, et al. Sodium-glucose cotransporter 2 inhibition and glycemic control in type 1 diabetes: results of an 8-week open-label proofof-concept trial. *Diabetes Care.* 2014;37(5):1480-3.
21. Cherney DZ, Perkins BA, Soleymanlou N, Maione M, Lai V, Lee A, et al. Renal hemodynamic effect of sodium-glucose cotransporter 2 inhibition in patients with type 1 diabetes mellitus. *Circulation.* 2014;129(5):587-97.

4.0 Study Design

4.1 Describe and explain the study design (e.g. case-control, cross-sectional, ethnographic, experimental, interventional, longitudinal, observational).

Response: This will be a two-center, randomized, open-label then double-blind, parallel group, placebo and standard of care controlled prospective study in type 1 diabetes. One hundred and (114) patients will be randomized (open label, see below) into three groups (38 each) and will be either maintained on their regular insulin therapy for 12 months (standard therapy group) or started on semaglutide in addition to regular insulin treatment for six month (dual therapy and triple therapy groups). For those on insulin and semaglutide, after six months of stable treatment, dapagliflozin (triple therapy group) or placebo (dual therapy group) treatment will be initiated for an additional six months according to a second randomization (double-blind).

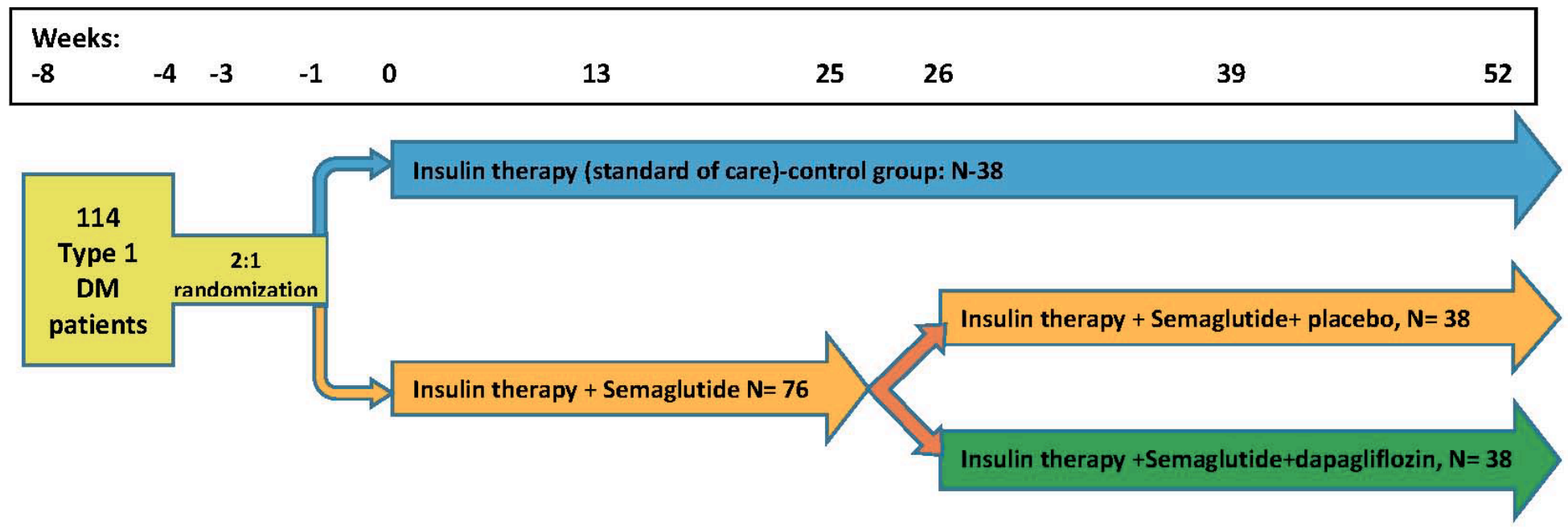

5.0 Local Number of Subjects

5.1 Indicate the total number of subjects that will be enrolled or records that will be reviewed locally.

Response: 57 patients will be enrolled locally with another 57 patients recruited at the University of Glasgow site: a total of 114.

5.2 If applicable, indicate how many subjects you expect to screen to reach your target sample (i.e. your screen failure rate).

Response: All screened and qualified patients will be enrolled and randomized

5.3 Justify the feasibility of recruiting the proposed number of eligible subjects within the anticipated recruitment period. For example, how many potential subjects do you have access to? What percentage of those potential subjects do you need to recruit?

Response: The Diabetes and Endocrinology Center of WNY is the largest Diabetes center in the WNY area, seeing between 60 to 100 type 1 diabetic patient every month. Therefore, the majority of recruited patients are our clinic patients. We also recruit a study participants through advertisement and other physician's references. These sources will suffice to recruit the needed number to subjects. The Glasgow center (Stobhill Ambulatory Care Hospital) reviews more than 300 patients per month.

6.0 Inclusion and Exclusion Criteria

6.1 Describe the criteria that define who will be ***included*** *in your final study sample.*

NOTE: This may be done in bullet point fashion.

Response:

Inclusion Criteria

1) Type 1 Diabetes for at least 1 year on staple use of continuous subcutaneous insulin infusion (CSII) or multiple daily (four or more) injections (MDI) of insulin for last 3 months.
2) C-peptide >0.23 nM
3) Minimum dose of insulin in Units/kg at entry: 0.5 U/kg for MDI and 0.4 U/kg for CSII
4) Regularly measuring blood sugars four or more times daily.
5) HbA1c of >7.5%.
6) Well versed in CHO counting*
7) Age 18-70 years.
8) BMI ≥25 kg/m^2.

*In the opinion of the site Principal Investigator

6.2 Describe the criteria that define who will be ***excluded*** *from your final study sample.*

NOTE: This may be done in bullet point fashion.

Response:

Exclusion Criteria

1) Type 1 diabetes for less than 12 months, type 2 diabetes, chronic pancreatitis, MODY
2) Previous use of any agent other than insulin for treatment of diabetes in the last 3 months.
3) History of diabetic ketoacidosis (DKA) requiring medical intervention (eg, emergency room visit and/or hospitalization) within 1 month prior to the screening visit
4) Frequent episodes of severe hypoglycemia as defined by more than one episode requiring medical assistance, emergency care (paramedics or emergency room care), and/or glucagon therapy administered by a third-party individual within 1 month prior to the screening visit
5) Symptoms of poorly controlled diabetes that would preclude participation in this trial
6) Subjects on a commercial weight loss program with ongoing weight loss, or on an intensive exercise program
7) History of bariatric surgery or lap-band procedure within 12 months prior to screening
8) History of Addison's disease or chronic adrenal insufficiency
9) History of diabetes insipidus
10) Aspartate Aminotransferase (AST) > 3X Upper limit of normal (ULN)
11) Alanine aminotransferase (ALT) > 3X ULN
12) Serum Total Bilirubin > 2X ULN unless exclusively caused by Gilbert's Syndrome
13) Hemoglobin < 11.0 g/dL (110 g/L) for men; hemoglobin < 10.0 g/dL (100 g/L) for women.

14) Coronary event or procedure (myocardial infarction, unstable angina, coronary artery bypass, surgery or coronary angioplasty) in the previous 3 months or patients with congestive heart failure.
15) Hepatic disease (transaminase > 3 times normal) or cirrhosis
16) ESRD on hemodialysis; and or e-GFR < 60 ml/min/1.73m^2
17) HIV or Hepatitis B/C positive status
18) Any other life-threatening, noncardiac disease
19) History of pancreatitis
20) Women who are pregnant or women of childbearing potential who are not using adequate contraception or who are breast feeding
21) Inability to give informed consent
22) History of gastroparesis
23) History of medullary thyroid carcinoma or MEN 2 syndrome
24) History of serious hypersensitivity reaction to these agents
25) Painful gallstones
26) Alcoholism
27) Hypertriglyceridemia (>500 mg/dl)
28) Recurrent genital mycotic infection.
29) Hypovolemic patients or with chronic renal insufficiency.
30) Patients with an active bladder cancer
31) Patients with a history of diabetic retinopathy
32) Use of an investigational agent or therapeutic regimen within 30 days of study
33) Participation in any other concurrent interventional clinical trial

6.3 *Indicate specifically whether you will include any of the following special populations in your study using the checkboxes below.*

NOTE: Members of special populations may not be targeted for enrollment in your study unless you indicate this in your inclusion criteria.

Response: NA

☐ Adults unable to consent

☐ Individuals who are not yet adults (infants, children, teenagers)

☐ Pregnant women

☐ Prisoners

6.4 *Indicate whether you will include non-English speaking individuals in your study.* ***Provide justification if you will exclude non-English speaking individuals.***

In order to meet one of the primary ethical principles of equitable selection of subjects, non-English speaking individuals may ***not*** *be routinely excluded from research as a matter of convenience.*

In cases where the research is of therapeutic intent or is designed to investigate areas that would necessarily require certain populations who may not speak English, the researcher is required to make efforts to recruit and include non-English speaking individuals. However, there are studies in which it would be reasonable to limit subjects to those who speak English. Some examples include pilot studies, small unfunded studies with validated instruments not available in other languages, studies with numerous questionnaires, and some non-therapeutic studies which offer no direct benefit.

Response:

We have no non-English speaking patients in this population. We have patients that English is a second language, but they are able to read, write and understand it. This population is less than 10% of the total population. No benefit is being withheld from not participating in the study.

7.0 Vulnerable Populations

*If the research involves special populations that are considered vulnerable, **describe the safeguards included to protect their rights and welfare.***

NOTE: You should refer to the appropriate checklists, referenced below, to ensure you have provided adequate detail regarding safeguards and protections. You do not, however, need to provide these checklists to the IRB.

*7.1 For research that involves **pregnant women,** safeguards include:*
NOTE CHECKLIST: Pregnant Women (HRP-412)

Response: We will not be recruiting subjects from vulnerable populations

☒ **N/A**: This research does not involve pregnant women.

*7.2 For research that involves **neonates of uncertain viability or non-viable neonates,** safeguards include:*
NOTE CHECKLISTS: Non-Viable Neonates (HRP-413), or Neonates of Uncertain Viability (HRP-414)

Response:

☒ **N/A:** This research does not involve non-viable neonates or neonates of uncertain viability.

*7.3 For research that involves **prisoners**, safeguards include:*
NOTE CHECKLIST: Prisoners (HRP-415)

Response:

☒ **N/A:** This research does not involve prisoners.

*7.4 For research that involves **persons who have not attained the legal age for consent to treatments or procedures involved in the research ("children"),***

safeguards include:
NOTE CHECKLIST: Children (HRP-416)

Response:

☒ **N/A:** This research does not involve persons who have not attained the legal age for consent to treatments or procedures ("children").

7.5 For research that involves ***cognitively impaired adults****, safeguards include:*
NOTE CHECKLIST: Cognitively Impaired Adults (HRP-417)

Response:

☒ **N/A:** This research does not involve cognitively impaired adults.

7.6 Consider if other specifically targeted populations such as students, employees of a specific firm, or educationally or economically disadvantaged persons are vulnerable. ***Provide information regarding their safeguards and protections, including safeguards to eliminate coercion or undue influence.***

Response: No specific populations or vulnerable groups will be targeted. All subjects enrolled in this study will be of legal adult consenting age with the ability to speak, read and interpret the English language. Patients will have the ability to speak with the research team regarding any questions or concern they have before signing the consent. Research team have the ability to evaluate patient's cognitive abilities through these interactions with patients. Patients are made aware that this study is voluntary and they are able to stop participating at any time they feel uncomfortable. Patients are not be pressured into participating and their clinic standard of care will remain the same if they participate or choose not to participate.

8.0 Eligibility Screening

8.1 Describe ***screening procedures*** *for determining subjects' eligibility. Screening refers to determining if prospective participants meet inclusion and exclusion criteria.*

Include all relevant screening documents with your submission (e.g. screening protocol, script, questionnaire).

Response:

After obtaining partial HIPAA waiver, patient charts will be screened according to the study inclusion and exclusion criteria by our trained clinical staff and physicians. In additions, the study clinical team will evaluate their clinic patients for possible participation in this study according to the inclusion and exclusion criteria at the Diabetes and Endocrinology Center of WNY clinics. Physicians will speak to them about their interests in participating in research. If the patient agrees, their information will be given to the research coordinator to be contacted for further evaluation. The research coordinator will contact interested patients

and those meeting the inclusion and exclusion criteria based on preliminary phone evaluation will be invited to participate in the study. All personal information will be kept confidential and locked in the coordinator office.

Prospective participants will be asked to read the consent and any questions they may have regarding the protocol will be answered. If the subject wants to participate in the study, they will be asked to sign the informed consent form. The subject's medical history and current medications will be obtained as well as their blood pressure and vitals. A physical examination will also be done. Blood and urine samples will be taken in order to evaluate HbA1c, CBC, CMP and liver and kidney functions and pregnancy status. Females of childbearing age, will have to be on two forms of birth control to prevent pregnancy. Patients meeting all the inclusion and exclusion criteria based on all screening tests will be enrolled in the study.

☐ **N/A:** There is no screening as part of this protocol.

9.0 Recruitment Methods

☐ **N/A:** This is a records review only, and subjects will not be recruited. NOTE: If you select this option, please make sure that all records review procedures and inclusion/exclusion screening are adequately described in other sections.

9.1 Describe when, where, and how potential subjects will be recruited.

NOTE: Recruitment refers to how you are identifying potential participants and introducing them to the study. Include specific methods you will use (e.g. searching charts for specific ICD code numbers, Research Participant Groups, posted advertisements, etc.).

Response:

Participants will be identified by prescreening clinical charts, patient doctor interaction at the time of their visits to the Diabetes Endocrinology Center of WNY Locations include:

1. 1020 Youngs Road, Williamsville NY 14221
2. 705 Maple Road, Williamsville NY 14221
3. 462 Grider Street, Buffalo NY 14215
4. 1000 Youngs Road, Suite 105, Williamsville NY 14221

Patients can be also referred by other physicians and providers

The remainder of the participants (57 patients) will be recruited from NHS Greater Glasgow and Clyde Diabetes clinics (Scotland, UK). Separate local IRB/ Ethical approval will be sought to cover this site (based on discussions with UBIRB director).

The study clinical team will evaluate their clinic patients for possible participation in this study according to the inclusion and exclusion criteria at the Diabetes and Endocrinology Center of WNY clinics. Patients that may qualify for the study are referred to the research team for further eligibility evolution. Patients meeting the

inclusion and exclusion criteria based on preliminary phone evaluation will be invited to participate in the study.

Participants will also be identified by flyers, advertisements on Facebook and Craigslist and researchmatch.org. The study will also be put on UB's Study Information Portal (SIP) website.

9.2 Describe how you will protect the privacy interests of prospective subjects during the recruitment process.

NOTE: Privacy refers to an individual's right to control access to him or herself.

Response: Patient charts will be screened according to the study inclusion and exclusion criteria by our trained clinical staff and physicians. If the patient qualifies and is of consenting age, the physicians will speak to them about their interests in participating in research in a private room where no one else can hear the conversation. If the patient agrees, their information will be given to the research coordinator to be contacted for further evaluation. No personal information will be shared without patient's approval and will not be used for any purpose except for this research. All personal information will be kept confidential and locked in the coordinator office.

9.3 Identify any materials that will be used to recruit subjects.

NOTE: Examples include scripts for telephone calls, in person announcements / presentations, email invitations.

For advertisements, include the final copy of printed advertisements with your submission. When advertisements are taped for broadcast, attach the final audio/video tape. NOTE: You may submit the wording of the advertisement prior to taping to ensure there will be no IRB-required revisions, provided the IRB also reviews and approves the final version.

Response: In addition to screening clinical charts, participations will be identified through; flyer advertisement, researchmatch.org, craigslist.com, and Facebook. The study will also be put on UB's Study Information Portal (SIP) website.

10.0 Procedures Involved

*10.1 Provide a description of **all research procedures or activities** being performed and when they are performed once a subject is screened and determined to be eligible. Provide as much detail as possible.*

NOTE: This should serve as a blueprint for your study and include enough detail so that another investigator could pick up your protocol and replicate the research. For studies that have multiple or complex visits or procedures, consider the addition of a schedule of events table in in your response.

Response:

Each patient will spend about 55 weeks in the trial comprising three weeks of monitoring to stabilize baselines and ensure adherence and commitment to participation before randomization into an intervention arm for an additional period of 52 weeks. Following a completed screening visit and meeting the inclusion and exclusion criteria, each patient will have a total of 16 study visits. At weeks 3, 5, 8, 16, 20, 29, 35, 44 and at 2 weeks after the end of the study, each patient will receive a phone call to document any adverse effects or other changes. There will be no long term follow-up following the end of the study.

Participants will be treated with either insulin only (control arm) for the whole duration of the study or with insulin and semaglutide weekly s.c. injection (titrated up to 1 mg, see below) for the first six months then either dapagliflozin (Triple therapy arm) or placebo (dual therapy) daily will be added for an additional six months. Semaglutide will be started at a dose 0.25 mg once weekly for two weeks then increased to 0.5 mg dose. If well tolerated it will be increased to 1 mg once weekly at week 4. Dapagliflozin (or placebo) will be started at a dose of 5 mg daily at week 26 and increased after 1 week to 10 mg (2x5mg tablets) daily to ensure drug tolerability and to avoid any hypoglycemia or hypotension.

Treatments used

1- Insulin: All patients (114 patients) will continue to self-administer their prescribed insulin as part of their standard of care. Insulin dose will be titrated during the study to control blood glucose average between 90-180 mg/dl and to avoid hypoglycemia (blood sugar <70 mg/dl) or hyperglycemia (blood sugar >250 mg/dl). Among all patients, 38 patients will be randomized (open-label) to remain on insulin therapy only (standard therapy group) while the other 76 will start the investigational therapies.
2- Semaglutide: a once weekly a GLP-1 mimetic injection (pen) indicated for type 2 diabetes. Doses to be used: start at 0.25mg then increase to 0.5mg following 2 weeks and to 1mg weekly s.c. injection 2 weeks later. Semaglutide will be added to a background of insulin treatment for 12 months in 76 patients who will be randomized (double-blind) into 2 groups (dual and triple therapy groups) at the end of 26 weeks.
3- Farxiga (dapagliflozin): a once daily SGLT-2 inhibitor tablet indicated for type 2 diabetes. Doses used: starting with 5mg and increase after 1 week to 10mg oral administration daily. Dapagliflozin will be started according to the randomization (Interactive Web System, hosted by University of Glasgow) in 38 patients at 26 weeks as an additional treatment on a background of insulin and semaglutide therapy and end at 52 weeks (triple therapy group).
4- Placebo: dapagliflozin matching placebo (supplied by Astra Zeneca) will be started in 38 patients at 26 weeks as an additional treatment on a background of insulin and semaglutide therapy and end at 52 weeks (dual therapy group).

As shown in **Table 1,** fasting blood and urine samples will be obtained prior to drug administration (weeks 0) and at 13, 26, 39 and 52 weeks after the commencement of the study. Optional fat biopsies from the abdominal subcutaneous tissue will be collected at weeks 0, 26 and 52. Continuous Glucose Monitoring (Dexcom G6 with blinded receiver) will be performed for three 21-days periods on weeks -3 to 0, 23 to 26 and from 49 to 52 weeks. An optional 24hr blood pressure monitoring will be performed at baseline (week -

1) and at 13, 25, 39 and 51 weeks in order to characterize the time course of antihypertensive effects, which may be early (i.e. independent of weight loss) as indicated by our preliminary data. High fat high caloric (HFHC) Meal challenges will be performed at baseline (week -1) and at 25 and 51 weeks with assessment of glucose excursions and postprandial glucagon (AUC) as well as postprandial inflammation and oxidative stress. Telephone calls will be conducted at weeks 3, 5, 8, 16, 20, 29, 35, 44 and at two weeks after the end of the study to document any side effects or any other patient's reported issues or comments.

Study Visits:

Screening Visit (Screening Visit, Week -8 to -4): Patients will be asked to read and understand the consent and any questions regarding the protocol will be answered. If they want to participate in the study, they will be asked to sign the informed consent form. Their medical history and current medications will be obtained as well as blood pressure and vitals. A physical examination will also be done. Blood samples will be taken in order to evaluate HbA1c, CBC, CMP, kidney and liver functions, ketone bodies, and urine for pregnancy status in females.

Study Visit 1 (Week -3): Participants who meet entry criteria will be scheduled to for their next visit at -3 week. Vitals will be taken including resting (min. 10 minutes) blood pressure measurement (will be repeated from same arm every visit). Insulin pump will be downloaded and/or fingerstick blood sugar will be reviewed and the appropriate insulin adjustments will be made by the investigator to target fasting blood glucose concentrations between 90-180 mg/dl if possible at all times without having additional hypoglycemia (less than 70 mg/dl). Blood sugars and ketone bodies will be reviewed via a fingerstick blood sample using glucose and ketones meter (Abbott Laboratories) and daily blood sugars collected and reviewed. Urine for pregnancy status and ketone bodies assessment. We will instruct patients on how to use and insert the Continuous Glucose Monitoring System (Dexcom G6 with blinded receiver). All subjects will be advised to monitor their capillary blood glucose by fingerstick at least 4 times a day (before meals and at bedtime) throughout the study and to wear their CGM constantly for next 21 day. Carb counting technique will be reviewed and any questions answered on how to use the pump or calculate insulin doses.

Study Visit 2 (Week -1±2days): Patients will be asked to come in the fasting state (no food or calorie intake for 10-12 hours). Their vitals will be taken. Blood sugars and ketone bodies will be reviewed via a fingerstick blood sample using glucose and ketones meter (Abbott Laboratories) to assess their glycemic control prior to meal challenge. If their blood sugar is below 70mg/dl or more than 250mg/dl the visit will be rescheduled for an alternative date. Insulin pump will be downloaded and/or fingerstick blood sugar will be reviewed and insulin adjustment will be made if necessary. An IV port will be placed in order to obtain blood samples throughout the duration of the visit. Patients will undergo a meal challenge test (described below). At the end of the meal test, they will be asked to wear a 24 hour blood pressure monitor (optional). A 24 hr urine container will be dispensed

to collect a 24 hour urine sample to bring in at the Week 0 visit. Daily Glucose logs will be reviewed.

Study visit 3 (Week 0±2days): Patients will be asked to come in the fasting state (no food or calorie intake for 10-12 hours). Vitals will be taken. Blood sugars and ketone bodies will be reviewed via a fingerstick blood sample using glucose and ketones meter (Abbott Laboratories) and daily blood sugars collected and reviewed. The 24 hour urine container will be collected. A fasting blood sample will be taken (45 mL) to evaluate study endpoints, CBC, CMP, HbA1c, and fructosamine. A urine sample will be collected to evaluate ketone bodies and, in females, pregnancy status. Insulin pump will be downloaded and/or fingerstick blood sugar will be reviewed and the appropriate insulin adjustments will be made by the investigator. In general, insulin dose adjustments should not be >10% from current total insulin dose or >20% from baseline (week 0) total insulin dose. They will discontinue use of the study CGM. An optional fat biopsy will be performed. We will ask them to fill out quality of life questionnaires. Study drug will be dispensed depending on what group they are randomized to. This includes either (1) staying on the insulin regimen given by their doctor, or (2) a combination of their current insulin regimen and a once daily injection of semaglutide. Semaglutide will be started on site at the 0.25 mg dose. The 24 hr blood pressure monitor will be collected.

Study Visit 4 (Week 1±2days): Vitals will be taken. Blood sugars and ketone bodies will be reviewed via a fingerstick blood sample using glucose and ketones meter (Abbott Laboratories) and daily blood sugars collected and reviewed. Insulin dose will be adjusted if needed as in visit 3. At any time, if HbA1c is <7%, insulin dose adjustment should not be >5%. Semaglutide 0.25mg will be injected on site. Participants will be instructed to call their research center or their doctor directly in case of any problem or untoward side effects. They will be specifically asked to call if they have unmanaged hypoglycemia (blood sugar <70 mg/dl) or hyperglycemia (blood sugar >250 mg/dl) or if occurring in more than one occasion.

Study Visit 5 (Week 2±2days): Vitals will be taken. Blood sugars and ketone bodies will be reviewed via a fingerstick blood sample using glucose and ketones meter (Abbott Laboratories) and daily blood sugars collected and reviewed. Insulin dose will be adjusted if needed as in visit 3. Semaglutide 0.5mg dose will be injected on site. If they are experiencing mild nausea, they will be continued on the 0.25 mg dose for another week. Supply of the drug will be given to last until the next visit. Patients will be advised to increase the study dose to 0.5 mg a day.

Study Visit 6 (Week 4±1 week): Patients will be asked to bring in used study pens for drug accountability (to be recorded in eCRF). Vitals will be taken. Blood sugars and ketone bodies will be reviewed via a fingerstick blood sample using glucose and ketones meter (Abbott Laboratories) and daily blood sugars collected and reviewed. Insulin dose will be adjusted if needed. Supply of the drug will be given to last until the next visit. A urine sample will be collected to evaluate ketone bodies and, in females, pregnancy status. Patients will be advised to increase the study dose to 1.0 mg a day. If they experience mild

nausea, they will be dropped from the study. A 24 hr urine container will be dispensed to collect a 24 hour urine sample to bring in at the Week 13 visit.

Study Visit 7 (Week 13±1 week): Patients will be asked to come in the fasting state (10-12 hours fasting), and to bring in your used study pens for drug accountability. Blood sugars and ketone bodies will be reviewed via a fingerstick blood sample using glucose and ketones meter (Abbott Laboratories) and daily blood sugars collected and reviewed. Insulin dose will be adjusted if needed as in. Supply of the drug will be given to last until the next visit. Vitals will be taken. A urine sample will be collected to evaluate ketone bodies and, in females, pregnancy status. The 24 hour urine container will be collected. A fasting blood sample will be taken (45 mL) to evaluate study endpoints, CBC, CMP, HbA1c, and fructosamine. Patients who agreed to participate in the24 hour blood pressure monitoring will be asked to wear it for the next 24 hr.

Study Visit 8 (Week 23±1 week): Patients will be asked to bring in used study pens for drug accountability. Vitals will be taken. Blood sugars and ketone bodies will be reviewed via a fingerstick blood sample using glucose and ketones meter (Abbott Laboratories) and daily blood sugars collected and reviewed. A urine sample will be collected to evaluate ketone bodies and, in females, pregnancy status. Insulin dose will be adjusted if needed. Supply of the drug will be given to last until the next visit. All subjects will be reminded to monitor their capillary blood glucose by fingerstick at least 4 times a day (before meals and at bedtime) through-out the study. CGM will be inserted and patients asked to wear their CGM constantly for next 21 day.

Study visit 9 (Week 25±1 week): Patients will be asked to come in the fasting state, and to bring in used study pens for drug accountability. Vitals will be taken. A urine sample will be collected to evaluate ketone bodies. Blood sugars and ketone bodies will be reviewed via a fingerstick blood sample using glucose and ketones meter (Abbott Laboratories) and daily blood sugars collected and reviewed. Insulin dose will be adjusted if needed. Supply of the drug will be given to last until the next visit. A 24 hr urine container will be dispensed to collect a 24 hour urine sample to bring in at the Week 26 visit. A HFHC Meal challenges will be performed as in visit 2. Patients who agreed to participate will be asked to wear a 24 hour blood pressure monitoring for the next 24 hr.

Study visit 10 (Week 26±1 week): Patients will be asked to come in the fasting state, and to bring in used study pens for drug accountability. Vitals will be taken. A urine sample will be collected to evaluate ketone bodies. Blood sugars and ketone bodies will be reviewed via a fingerstick blood sample using glucose and ketones meter (Abbott Laboratories) and daily blood sugars collected and reviewed. Insulin dose will be adjusted if needed. In general, insulin dose adjustments should not be >10% from current total insulin dose (or 5% if HbA1c is <7%) or >20% from baseline total insulin dose. The adjusted total insulin dose in this visit will serve as the baseline total insulin dose for the remaining 6 months of the study and future limits of dose adjustments will be based on this new baseline. The CGM will be collected. A urine sample will be collected to evaluate ketone bodies and, in females, pregnancy status. The 24 hour urine container will be collected. A fasting blood sample will be taken (45 mL) to evaluate study endpoints, CBC,

CMP, HbA1c, and Fructosamine. An optional fat biopsy will be performed. We will ask patients to fill out quality of life questionnaires. At this point, those patients randomized to receive semaglutide, they will now be randomized to receive an actual dapagliflozin pill or a placebo pill that looks like dapagliflozin. If they are randomized to receive actual dapagliflozin, they will start at the 5 mg dose for one week.

Participants will be provided supplies for urine ketone bodies measurement (KetoStix). Research coordinator will instruct the patients on the use and reading of the measuring strips. Patients will be asked to perform a daily (preferably early morning or before bed daily) urinary test of ketone bodies using KetoStix and record all outcomes. If results are moderate or higher (+2 or more), they will be instructed to repeat the measurement within 60 min. If consecutive measurements show moderate or higher ketones in urine, the patients will be instructed to call the research center or their doctor directly. Patients with dual glucose and ketones meters will be instructed to record and report their blood ketones in dedicated study diaries. If blood ketones are 0.6 mM or above, they should repeat the measurement within 60 min. If consecutive measurements show 0.6mm or higher ketones in blood, the patients will be instructed to call the research center or their doctor directly.

Participants will be instructed to call their research center or their doctor directly in case of any problem or untoward side effects. They will be specifically asked to call if they have unmanaged hypoglycemia (blood sugar <70 mg/dl) or hyperglycemia (blood sugar >250 mg/dl) or if occurring in more than one occasion. Participants will be instructed to remain hydrated and report any genital mycotic or urinary tract infections.

Study visit 11 (Week 27±2 days): Patients will be asked to bring in used study pens for drug accountability (recorded in eCRF). Vitals will be taken. Daily blood sugars and urinary ketones measurements using KetoStix will be collected and reviewed. Blood sugars and ketone bodies will be measured via a fingerstick blood sample using glucose and ketones meter (Abbott Laboratories). A urine sample will be collected to evaluate urine ketone level. Insulin dose will be adjusted if needed. In general, insulin dose adjustments should not be >10% from current total insulin dose (or 5% if HbA1c is <7%) or >20% from NEW baseline total insulin dose established at visit 10. If at any time during the study total insulin dose is less than 0.30 U/Kg, additional monitoring protocol (see later) will be implemented. Supply of the drug will be given to last until the next visit. If patients are receiving actual Dapagliflozin, they will now start at the 10 mg dose for the remainder of the study.

Study Visit 12 (Week 31±1 week): Patients will be asked to bring in used study pens and tablets for drug accountability. Vitals will be taken. Daily blood sugars and urinary ketones measurements using KetoStix will be collected and reviewed. Blood sugars and ketone bodies will be measured via a fingerstick blood sample using glucose and ketones meter (Abbott Laboratories). A urine sample will be collected to evaluate ketone bodies and, in females, pregnancy status. Insulin dose will be adjusted if needed as in visit 11. Supply of the drugs will be given to last until the next visit. A blood sample will be taken (15 mL) to evaluate CBC and CMP. A 24 hr urine container will be dispensed to collect a 24 hour urine sample to bring in at the Week 39 visit.

<u>Study visit 13 (Week 39±1 week):</u> Patients will be asked to come in the fasting state, and to bring in used study pens and tablets for drug accountability. Vitals will be taken. Daily blood sugars and urinary ketones measurements using KetoStix will be collected and reviewed. Blood sugars and ketone bodies will be measured via a fingerstick blood sample using glucose and ketones meter (Abbott Laboratories). Insulin dose will be adjusted if needed as in visit 11. Supply of the drug will be given to last until the next visit. A urine sample will be collected to evaluate ketone bodies and, in females, pregnancy status. The 24 hour urine container will be collected. A fasting blood sample will be taken (45 mL) to evaluate study endpoints, CBC, CMP, HbA1c, and Fructosamine. Patients who have provided consent will be asked to wear a 24 hour blood pressure monitor.

<u>Study visit 14 (Week 49±1 week):</u> Patients will be asked to bring in used study pens and tablets for drug accountability. Vitals will be taken. Daily blood sugars and urinary ketones measurements using KetoStix will be collected and reviewed. Blood sugars and ketone bodies will be measured via a fingerstick blood sample using glucose and ketones meter (Abbott Laboratories). A urine sample will be collected to evaluate ketone bodies and, in females, pregnancy status. Insulin dose will be adjusted if needed as in visit 11. Supply of the drug will be given to last until the next visit. All subjects will be reminded to monitor their capillary blood glucose by fingerstick at least 4 times a day (before meals and at bedtime) through-out the study. CGM will be inserted and patients asked to wear their CGM constantly for the next 21 days.

<u>Study visit 15 (Week 51±1 week):</u> Patients will be asked to come in the fasting state, and to bring in used study pens and tablets for drug accountability. Vitals will be taken. Daily blood sugars and urinary ketones measurements using KetoStix will be collected and reviewed. Blood sugars and ketone bodies will be measured via a fingerstick blood sample using glucose and ketones meter (Abbott Laboratories). A urine sample will be collected to evaluate urine ketone levels. Insulin dose will be adjusted if needed as in visit 11. Supply of the drug will be given to last until the next visit. A 24 hr urine container will be dispensed to collect a 24 hour urine sample to bring in at the Week 52 visit. A HFHC Meal challenges will be performed as in visit 2. Patients will be asked to wear the optional 24 hour blood pressure monitor.

<u>Study visit 16 (Week 52±2 days):</u> Patients will be asked to come in the fasting state, and to bring in used study pens and tablets for drug accountability. Vitals will be taken. Daily blood sugars and urinary ketones measurements using KetoStix will be collected and reviewed. Blood sugars and ketone bodies will be measured via a fingerstick blood sample using glucose and ketones meter (Abbott Laboratories). Insulin dose will be adjusted if needed. The CGM will be collected. An optional fat biopsy will be performed. A urine sample will be collected to evaluate ketone bodies and, in females, pregnancy status. The 24 hour urine container will be collected. A fasting blood sample will be taken (45 mL) to evaluate study endpoints, CBC, CMP, HbA1c, and Fructosamine. We will ask the patients to fill out quality of life questionnaires. The patient will receive a physical. After this visit, they will be discharged from the study.
Safety phone calls will be conducted at weeks 3, 5, 8, 16, 20, 29, 35, 44 and at two weeks after the end of the study to document any side effects or other changes.

Visit #	-	1	2	3	4	5	6	7	8	9	10	11	12	13	14	15	16
Week#(±2-7 days as per text)	-8 to -4	-3	-1	0	1	2	4	13	23	25	26	27	31	39	49	51	52
Study Procedures	Screening	Run-in period		Randomization, start semaglutide							Randomize/Start dapagliflozin/ placebo						End of study
History	X																
Physical	X																X
Vitals	X	X	X	X	X	X	X	X	X	X	X	X	X	X	X	X	X
CBC	X			X				X			X		X	X			X
CMP	X			X				X			X		X	X			X
Kidney and liver profiles	X			X				X			X		X	X			X
Pregnancy test	X	X		X			X	X	X		X		X	X			X
Study drug titration					X	X	X					X					
Dispense medications				X	X	X	X	X	X	X	X	X	X	X	X	X	
HbA1c	X			X				X			X			X			X
Fructosamine				X				X			X			X			X
24 hour BP (optional)			X					X		X				X		X	
CGM		X	X						X	X					X	X	
Daily Fingerstick glucose review		X	X	X	X	X	X	X	X	X	X	X	X	X	X	X	X
Daily urinary ketones review												X	X	X	X	X	X
Insulin dose review		X	X	X	X	X	X	X	X	X	X	X	X	X	X	X	X
Ketone bodies (blood/urine)	X	X	X	X	X	X	X	X	X	X	X	X	X	X	X	X	X
Adverse Event reporting		X	X	X	X	X	X	X	X	X	X	X	X	X	X	X	X
Meal Challenge			X							X						X	X
Blood endpoints				X				X			X			X			X
Fat biopsy (optional)				X							X						X
24 hr Urine				X				X			X			X			X
Quality of life questionnaires				X							X						X

Drug Blinding:
The randomizations will be performed using an Interactive Web System, hosted by University of Glasgow. One hundred and fourteen (114) patients will be randomized as follows: participants will first be randomized to semaglutide plus usual insulin therapy or insulin therapy only (no placebo for semaglutide or other placebo injection will be provided), in a 2:1 ratio. Those who remain on semaglutide at six months will then be randomized in a 1:1 ratio to receive dapagliflozin or matched placebo. Each randomization will be stratified by study site. The randomization schedules will be generated by a statistician with no other involvement in the trial, using the method of randomized permuted blocks. Only those staff responsible for the maintenance of the randomization system will have access to the randomization sequences during the trial. The first randomization (which will be revealed to the researcher at the point of randomization) will use random block sizes to reduce predictability. Researchers at study sites will have no knowledge of the next allocation to be issued. The second randomization (which will be double-blind) will use a different. At the point of randomization, the researcher will be given a medication pack ID number, but will not know whether that medication pack contains Dapagliflozin or placebo. For each randomization, a web-based randomization system (developed and maintained by the Robertson Centre for Biostatistics) will be used. The system will include an emergency unblinding facility for the dapagliflozin/placebo randomization
Astra Zeneca Inc. will supply dapagliflozin and matching placebo tablets in identical packaging and labeling; semaglutide pens will be obtained commercially and will be used in an open label fashion.

Breaking of the study blind will be performed only: (i) for SUSARs; and, (ii) where knowledge of the treatment is considered by local health personnel absolutely necessary for further management of the patient. A central unblinding facility based at the Robertson Centre for Biostatistics, University of Glasgow, will be available by telephone. Notification of all unblinding will be sent to the Chief Investigator

During the course of the trial there will be no attempt to identify group assignment for any participant (except for unblinding in the case of safety concerns) and the investigators will execute the protocol in the manner it was approved independent of the group assignment.

Drugs will be stored in a locked cabinet or locked temperature controlled refrigerator at 4C at the research facility of the Diabetes and Endocrinology Center of WNY. IND application for use of all drugs (dapagliflozin and semaglutide) in type 1 diabetes will be submitted to FDA and no study procedure will start before all local and federal approvals are obtained.

Clinical and Laboratory Procedures

1) **CMP, CBC, HbA1c, fructosamine, glucose, glucagon, GLP-1 and FFA measurements:** Blood CMP, CBC, HbA1c, fructosamine and glucose will be measured through Quest diagnostic (US site) and NHS labs (UK site). Free fatty acid levels will be measured by a colorimetric assay (Wako, Richmond, VA). GLP-1 and

glucagon will be measured from samples collected in protease and DPP-IV inhibitors by ELISA kits from Millipore.

2) **Meal Challenge test:** In order to assess the postprandial changes induced by dapagliflozin, semaglutide or the combination of both compared to insulin only, a meal challenge will be performed before starting treatments, at six months and at end of study using a 910 Calorie High fat High carbohydrate meal (HFHC) as in several of our previous studies (HFHC meal includes an egg muffin sandwich, a sausage muffin sandwich and two hash browns which contain 88g carbohydrate, 51 g fat (33% saturated) and 34 g protein). Participants will be asked to attend fasting prior to breakfast insulin bolus; basal insulin will continue during the duration of the meal, however patients will not bolus until the 3 hour mark unless blood glucose values reach above 400 mg/dL. Sequential blood samples will be obtained at 0, 15, 30, 45, 60, 90, 120, 150 and 180 min after consuming the meal. Samples at 15, 30, 45, 90 and 150 min will be 5ml while those at 0, 60, 120 and 180min will be 30 ml (total volume=145 ml). Blood will be collected from an indwelling intravenous canula in a superficial forearm vein.
3) **Optional 24 hour Ambulatory BP monitoring:** Participants will be instructed on the use of 24 hour ambulatory BP monitoring device. This device will measure BP and pulse every 30 minutes for 24 hours. In addition to the scheduled measurements there will be an option for additional manual measurements if systolic blood pressure is >160 or <90 mmHg.
4) **Optional Fat aspiration procedure:** Abdominal subcutaneous fat tissue aspiration will be performed at a 10 cm distance from umbilicus. Participants will be asked not to take aspirin or NSAIDS in the previous 72 hours - if they have, the procedure will not be done. Skin will be prepared with povidone-iodine (Betadine) and alcohol. A sterile drape will be placed around the appropriate area. 3cc of 1% lidocaine will then be administered subcutaneously (the dose will not exceed 4.5mg/kg body weight). After adequate anesthesia has been achieved, aspiration of fat tissue will then be with a 3-holed canula (Tulip Instrumentation, length: 15cm, diameter: 2.1mm) fixed to a 10mL syringe. After getting adequate fat tissue (500mg-3g), the puncture site will be pressed for at least ten minutes before the patient rises up from supine position (to minimize bruising). Participants will then be discharged home. Adipose tissue will be centrifuged to remove blood and fluid contaminants. Upper adipose tissue will be collected into a separate sterile tube, washed twice with cold sterile Phosphate Buffered Saline (PBS) and centrifuged to remove the PBS. The adipose tissue sample will be weighed and approximately 500 mg transferred to a separate tube for analysis. Total RNA, nuclear extracts and total cell lysates will be prepared.
5) **Questionnaires:** Participants will be asked to complete the standard Diabetes Specific Quality of Life Scale (DSQOLS) and Problem Areas In Diabetes survey (PAID). Questionnaires attached at the end of this plan.
6) **MNC isolation (for future ancillary studies):** Blood samples will be collected in Na-EDTA as an anticoagulant. Three and a half mL of anticoagulated blood sample are carefully layered over 3.5 mL of PMN medium (Cedarlane Laboratories, Hornby, ON) and then centrifuged. At the end of this process, two bands separate out at the top of the RBC pellet. The top band consists of MNC, while the bottom consists of PMN. The

MNC band is harvested and washed twice with Hank's balanced salt solution (HBSS). This method provides yields greater than 95% pure PMN and MNC suspensions.

7) **NFκB DNA binding activity (for future ancillary studies):** Nuclear NFκB DNA binding activity is measured by EMSA. Nuclear extract is prepared from MNC and adipose tissue by high salt extraction as described by Andrews et al (19). The specificity of the bands is confirmed by supershifting these bands with specific antibodies against Rel-A (p65) and p50 (Santa Cruz Biotechnology, CA) and by competition with cold oligonucleotides. DNA binding activity of NFκB will be adjusted to Oct1 DNA binding activity to correct for any variable.
8) **Plasma concentrations of inflammatory mediators (for future ancillary studies):** Concentrations of TNFα, IL-1β, IL-18, adiponectin, IL-1RA will be measured in plasma using ELISA kits (R&D Systems, MN). CRP and SAA concentrations will be measured using an ELISA kit from Alpha Diagnostics International Inc. (San Antonio, TX).
9) **Plasma vasodilators and vasoconstrictors (for future ancillary studies):** Plasma ANP, NT-proBNP, b-NP, cGMP, cAMP, angiotensinogen, renin and angiotensin II concentrations will be measured by commercially available ELISA kits.
10) **15-isoprostane F_{2t}** (also known as 8-epi-PGF2α or 8-iso-PGF2α) **(for future ancillary studies):** will be measured in urine samples using an ELISA kit from Oxford Biomedical Research (Oxford, MI).
11) **Quantification of mRNA in MNC and adipose tissue (for future ancillary studies).** Total RNA is isolated from MNC and adipose tissue using commercially available RNAqueous®-4PCR Kit (Ambion, Austin, TX). The quality and quantity of the isolated RNA is determined before using the RNA. Real Time RT-PCR is performed using Cepheid Smart Cycler (Sunnyvale, CA) in which 2 µL cDNA, 10 µL Sybergreen Master mix (Qiagen, CA) along with 0.5 µL of 20 µM gene specific primers for IL-1β, IL-RA, TLR-4, TNFα, IL-12, SOCS-3 and JNK-1 (Life Technologies, MD) are used. The specificity and the size of the PCR products are tested by adding a melt curve at the end of the amplifications and by running it on a 2% agarose gel. All values are normalized to 18S expression and β-actin.

10.2 Describe what data will be collected.

NOTE: For studies with multiple data collection points or long-term follow up, consider the addition of a schedule or table in your response.

Response:

Data will be collected as described in table 1 above. The following aspects of the study will be performed and data collected and reported by patients:

1- A CGM device will be provided for use for 3 20-days periods. Detailed instructions on changing the sensor at 10 days will be provided. Patients will be instructed to wear their CGM constantly during the assigned study periods. They will be asked to keep a diary of their food intake to measure their carbohydrate intake during the CGM period. Insulin doses will be titrated aiming for blood glucose between 90-180 mg/dl while avoiding hypoglycemia (<70 mg/dl) or hyperglycemia (>250 mg/dl).

2- Participants who agree to participate in the 24-hr ambulatory blood pressure monitoring will be provided with a 24-hour ambulatory BP monitor to be worn for 24 hours during week -1, 13, 25, 39 and 51.
3- All participants will be asked to monitor capillary blood glucose by finger stick before each meal and at bedtime.
4- For the entire duration of the study, the patients will maintain a diary to record any hypoglycemia and other untoward side effects e.g. nausea, change in appetite.
5- Starting at the 6 months mark, participants will be asked to perform a daily urinary test of ketone bodies using KetoStix and record all outcomes. If results are moderate to large, they will be instructed to repeat the test and to call the research center or their doctor directly if results are confirmed. Patients with dual glucose and ketones meters will be instructed to record and report their blood ketones as well. If blood ketones are above 0.6mM, they should repeat the test and contact the research center of their doctor if levels are confirmed.

10.3 List any instruments or measurement tools used to collect data (e.g. questionnaire, interview guide, validated instrument, data collection form).

Include copies of these documents with your submission.

Response: Source documents will be used to collect patient information. The CGM or insulin pump will be used to collect glucose and insulin data. Glucose and ketone meter will be used to measure glucose and ketone in blood. KetoStix for measuring ketones in urine. Blood pressure monitor will be used for 24 hour blood pressure reading. Glucose, food and urinary ketones diaries will be provided for patients to record their results.

10.4 Describe any source records that will be used to collect data about subjects (e.g. school records, electronic medical records).

Response: electronic medical records and research files.

*10.5 Indicate whether or not **individual** subject results, such as results of investigational diagnostic tests, genetic tests, or incidental findings will be shared with subjects or others (e.g., the subject's primary care physician) and if so, describe how these will be shared.*

Response: Individual participant lab results will be disclosed to the participant upon their request. If the participant requests documentation be shared with another physician, physician office or hospital the participant must come to the research center to collect said documentation or the documentation can be mailed to their given home address.

Patients will be assigned a unique study number. All identifiable personal information will be stored locally in locked filing cabinets. Data entered on the

electronic Case Report Form will only be identified by the unique study number. Thus, no personal information will be shared with the data center (Robertson Centre for Biostatistics, University of Glasgow, UK).

*10.6 Indicate whether or not **study** results will be shared with subjects or others, and if so, describe how these will be shared.*

Response: Not Applicable. Study results will not be shared with the subjects. However, unidentifiable study results could be published in the form of a manuscript or abstract and will be reported to JDRF, Astra Zeneca, and to clinicaltrials.gov and other regulatory agencies including FDA.

11.0 Study Timelines

11.1 Describe the anticipated duration needed to enroll all study subjects.

Response: Accrual is expected to be at the rate of 4 subjects/month/site, thus we should complete enrollment of 57 patients/site in approximately 13-15 months. We expect to complete all study clinical activities in 30 months.

11.2 Describe the duration of an individual subject's participation in the study. Include length of study visits, and overall study follow-up time.

Response: 55 Weeks. Study visits will be 60-90 minutes long except for visits 2, 9 and 15 which will be about 6hr long each

11.3 Describe the estimated duration for the investigators to complete this study (i.e. all data is collected and all analyses have been completed).

Response: 36 Months

12.0 Setting

12.1 Describe all facilities/sites where you will be conducting research procedures. Include a description of the security and privacy of the facilities (e.g. locked facility, limited access, privacy barriers). Facility, department, and type of room are relevant. Do not abbreviate facility names.

NOTE: Examples of acceptable response may be: "A classroom setting in the Department of Psychology equipped with a computer with relevant survey administration software," "The angiogram suite at Buffalo General Medical Center, a fully accredited tertiary care institution within New York State with badge access," or, "Community Center meeting hall."

Response:

1) Research will be conducted at the Diabetes Endocrinology Research Center of WNY, located at *1000 Youngs Road, Suite 105, Williamsville NY 14221* and at the CTRC located in 875 Ellicott St. Buffalo NY14203.The Diabetes Research Center has facilities and exam rooms available for insulin pump download, CGM device download, meal and infusion studies and presence of study coordinator and registered nurse for data collection and blood work at all times. One of the investigators will be available at all times to address patients' related issues. CTRC location is a fully equipped laboratory with Equipment include ultra-low freezers for sample storage, centrifuges, microscopes for sample preparation, infusion pumps, ELISA, PCR and immunoblotting instrumentation.

2) Research will also be conducted at the NHS Greater Glasgow and Clyde Clinical Research Facility (CRF, Glasgow Royal Infirmary, UK and at the Diabetes Centre (Clinic D), Stobhill Ambulatory Care Hospital, Glasgow, UK. There are facilities and exam rooms available for insulin pump download, CGM device download, meal and infusion studies and presence of research nursing staff for data collection and blood work at all times. One of the investigators will be available at all times to address patients' related issues. The CRF has equipment for sample preparation, infusion pumps, ultra-low freezers for sample storage and centrifuges. ELISA, PCR and immunoblotting facilities are available in adjacent NHS and University facilities.

12.2 For research conducted outside of UB and its affiliates, describe:

- *Site-specific regulations or customs affecting the research*
- *Local scientific and ethical review structure*

NOTE: This question is referring to UB affiliated research taking place outside UB, i.e. research conducted in the community, school-based research, international research, etc. It is not referring to multi-site research. UB affiliated institutions include Kaleida Health, ECMC, and Roswell Park Cancer Institute.

Response:

☒ **N/A:** This study is not conducted outside of UB or its affiliates.

13.0 Community-Based Participatory Research

13.1 Describe involvement of the community in the design and conduct of the research.

NOTE: Community-Based Participatory Research (CBPR) is a collaborative approach to research that equitably involves all partners in the research process and recognizes the unique strengths that each brings. CBPR begins with a research topic of importance to the community, has the aim of combining knowledge with action and achieving social change to improve health outcomes and eliminate health disparities.

Response:

☒ **N/A:** This study does not utilize CBPR.

13.2 Describe the composition and involvement of a community advisory board.

Response:

☒ **N/A:** This study does not have a community advisory board.

14.0 Resources and Qualifications

14.1 Describe the qualifications (e.g., education, training, experience, expertise, or certifications) of the Principal Investigator ***and*** *staff to perform the research. When applicable describe their knowledge of the local study sites, culture, and society. Provide enough information to convince the IRB that you have qualified staff for the proposed research.*

NOTE: If you specify a person by name, a change to that person will require prior approval by the IRB. If you specify a person by role (e.g., coordinator, research assistant, co-investigator, or pharmacist), a change to that person will not usually require prior approval by the IRB, provided that the person meets the qualifications described to fulfill their roles.

Response: All study personnel are educated, trained, and licensed as required for their delegated role in this study. All study personnel have also received the required university training and will be trained by the PI before the study starts. The PIs, CO-investigators and research team have conducted similar studies in this patient population using similar agents and have the expertise to execute the study protocol and manage any adverse events.

Describe other resources available to conduct the research.

14.2 Describe the time and effort that the Principal Investigator and research staff will devote to conducting and completing the research.

NOTE: Examples include the percentage of Full Time Equivalents (FTE), hours per week. The question will elicit whether there are appropriate resources to conduct the research.

Response: The principal investigator supervises the research project and weekly research meetings are conducted to discuss the recruitment rate, resolve and discuss issues related to the conduct, safety, analysis of the study and related publications. PI is expected to spend 15% of his academic time on this research. The co-investigators, study coordinator and research staff provide coverage to the research related activity for 365 days a year.

14.3 Describe the availability of medical or psychological resources that subjects might need as a result of anticipated consequences of the human research, if applicable.

NOTE: One example includes: on-call availability of a counselor or psychologist for a study that screens subjects for depression.

Response: Available medical literature will be provided as deemed appropriate or requested by patient through UB libraries, PubMed, Google scholar as all the investigators have access to medical literature through listed resources above

The patient will also have access to physician (Investigators and Co-Investigators) who will be available to address any adverse effects or other questions during the course of the study who will be available to address any adverse effects or other questions during the course of the study

14.4 Describe your process to ensure that all persons assisting with the research are adequately informed about the protocol, the research procedures, and their duties and functions.

Response: Before beginning of enrollment all research staff will be asked to review the protocol and a training session will be conducted to provide protocol details, explain technical points and answer staff questions. All training session will have a sign in sheet to document such training. Continuous education through training meetings, conferences and discussions will continue through the study period and when revision are made to protocol.

15.0 Other Approvals

15.1 Describe any approvals that will be obtained prior to commencing the research (e.g., school, external site, funding agency, laboratory, radiation safety, or biosafety).

Response: FDA IND filling is required.

☐ **N/A:** This study does not require any other approvals.

16.0 Provisions to Protect the Privacy Interests of Subjects

16.1 Describe how you will protect subjects' privacy interests during the course of this research.

NOTE: Privacy refers to an individual's right to control access to him or herself. Privacy applies to the person. Confidentiality refers to how data collected about individuals for the research will be protected by the researcher from release. Confidentiality applies to the data.

Examples of appropriate responses include: "participant only meets with a study coordinator in a classroom setting where no one can overhear", or "the participant is reminded that they are free to refuse to answer any questions that they do not feel comfortable answering."

Response: Our clinical providers involved in the study will identify potential patients for recruitment from the Diabetes-Endocrinology Center of WNY Clinics according to the inclusions and exclusion criteria and through advertisements. Patient who qualify will be asked in private during their one on one consultation time with the physician if they wish to participate in the research study. If the patient agrees, the research coordinator will contact them privately for a possible telephone screening and ask if time is appropriate for this discussion. The patients who call for potential participation in the study due to advertisement flyers will be screened over the phone with the research coordinator, using our telephone screening form.

When the patient is being seen at our clinics for the first time they sign the "Consent to use and disclosure of protected health information" form which clearly states that their protected health information (PHI) can be used for review in preparation for possible research. Additionally, a partial HIPAA waiver will be obtained to access medical records when screening for patients.

If the patient passes the telephone screening, they will be asked to make an appointment to review and sign the consent. Patient will do this in a private room in the research unit and will be allowed to discuss the consent in detail with the research coordinator and or study doctor. Patient will be notified that it is completely voluntary to participate in the research study and can withdraw at any time.

We will not be accessing any medical information of the patients for whom the services are not provided by our clinic providers before partial HIPAA is obtained or IC is signed.

16.2 Indicate how the research team is permitted to access any sources of information about the subjects.

*NOTE: Examples of appropriate responses include: school permission for review of records, consent of the subject, HIPAA waiver. This question **does apply** to records reviews.*

Response: Consent of the subject and partial HIPAA waiver.

17.0 Data Management and Analysis

17.1 Describe the data analysis plan, including any statistical procedures. This section applies to both quantitative and qualitative analysis.

Response:

Data (e.g. date of diagnosis, past medical history, drug treatment) extracted from local clinical records will be entered on to the eCRF under the study-specific coded identifier according to structured questions as stipulated in the Study Protocol. Biological samples (blood, urine and adipose tissue aspirates) will be collected from the study participants at the study visits according to the Schedule of Assessments in the Study Protocol.

Measures relating to safety and routine care of the participants (e.g., biochemical measures of glycemic and lipidemic control, kidney function/ fluid balance, hepatic function, hematological variables) will be analysed at local clinical laboratories in order that they are available to study nurses and physicians. The confidentiality of these data will be secure, meeting or exceeding the regulations of the relevant Ethical Review Committees/ Institutional Review Boards. Samples for measurement of key study outcomes will also be measured at the central laboratory at the University of Buffalo. Blood, urine and other samples will be aliquoted into cryovials at site for freezing and storage. Electronic stored material (e.g., patient records, data from blood and urine samples, and data from Continuous Glucose Monitoring) will only be stored using the study specific coded identifier. Data will not be identifiable at the participant level without additional access to the code. The code will be available only to the investigators and the personnel performing the study. Access to any data held electronically will require study specific log-in codes. These are managed and logged by the Robertson Centre for Biostatistics, University of Glasgow, UK

Statistical plan. The primary objective of the proposed research is to evaluate the effect of addition of dapagliflozin to semaglutide on a background of insulin treatment in type 1 diabetes patients on HbA1c at six months following start of dapagliflozin. The similarities between the study groups, baseline values for subject's demographics and study endpoints will be compared using appropriate parametric tests. Transformations of the data in order to meet statistical assumptions may be considered. The primary endpoint of the study is to detect a difference in mean HbA1c change after six months of dapagliflozin or placebo addition to semaglutide and insulin treatment. Statistical analysis will be carried out using t-tests to detect difference between groups or the Wilcoxon test for paired data. Tests will be two-sided and tested at the 0.05 level. The results will be summarized as mean±SE. All randomized patients will be included in the analysis of the primary end point based on an Intent-to-treat model.

The secondary endpoints will be to compare changes in HbA1c between each of the triple therapy and dual therapy groups compared to standard of care group, mean weekly glucose concentrations, percent time spent in different glycemic thresholds, variability as measured by standard deviation, insulin dose, body weight, carbohydrate intake, blood pressure, and 24-hour blood pressure and QOL changes among the groups. A reduction in the glucose and glucagon AUC following the meal will constitute another secondary end point in this setting, ANCOVA analysis will be used.

17.2 If applicable, provide a power analysis.

NOTE: This may not apply to certain types of studies, including chart/records reviews, survey studies, or observational studies. This question is asked to elicit whether the investigator has an adequate sample size to achieve the study objectives and justify a conclusion.

Response:

Sample size determination is based in part on our current data showing that in type 1 diabetes, treatment with a GLP-1 agonist, liraglutide (1.2 and 1.8mg doses) for 3 months leads to HbA1c suppression by an average of 0.6% (SD= 0.5%). Dapagliflozin was recently shown to reduce HbA1c by 0.45% in type 1 diabetes patients (13). Additionally, empagliflozin treatment for 8 weeks was shown to reduce HbA1c by 0.4% in type 1 diabetics (20, 21) and our current data showing additional improvement in HbA1c by an additional 0.66% at 12 weeks when dapagliflozin was added to liraglutide and insulin therapy. Conservatively estimating that the combination therapy will suppress HbA1c by 0.5% (SD =<0.7%) compared to semaglutide alone (t-test), a sample size of 38 (32 evaluable patients) in each group will be sufficient to provide adequate power ($\beta = 0.2$) to detect a statistically significant difference ($\alpha = 0.05$) taking in consideration a 15% dropout rate.

17.3 Describe any procedures that will be used for quality control of collected data.

Response:

An electronic case report form (e-CRF) will be used to collect study data at each site. The e-CRF will be developed by the study Data Centre at the Robertson Centre for Biostatistics, University of Glasgow. Access to the e-CRF will be restricted, with only authorised site-specific personnel able to make entries or amendments to their patients' data. It is the investigator's responsibility to ensure completion and to review and approve all data captured in the e-CRF.

Data will be validated at the point of entry into the e-CRF and at regular intervals during the study. Data discrepancies will be flagged to the study site coordinator and any data changes will be recorded in order to maintain a complete audit trail (reason for change, date change made, who made change).

The Robertson Centre for Biostatistics at the University of Glasgow will be responsible for collating and cleaning the data for the study. The Robertson Centre will also be responsible for data back-up and security. This center will also manage the electronic reporting of SUSARS on behalf of the sponsor.

To enable evaluations and/or audits from regulatory authorities, the investigators agree to keep records, including the identity of all participating subjects (sufficient information to link records, all original signed informed consent forms serious adverse event forms, source documents, and detailed records of treatment disposition. The records should be retained by the study country coordinators and

investigator according to ICH GCP, local regulations, or as specified in the Clinical Study Agreement, whichever is longer.

Data from the study will be stored by the Chief Investigator for a minimum of 10 years. A final report of the study will be provided to the FDA and the MHRA as per requirements.

- No interim analysis is planned for efficacy so termination of study will be related to safety. Significant increases in hypoglycemic or DKA events as reviewed by DSMB might trigger termination or modification of study.
- Last observation carry forward will be used to fill missing data for the primary end point and some secondary end points. Missing data will not be filled for other endpoints.
- Any deviation(s) from the original statistical plan will be described and justified in the protocol and/or in the final report.
- All subjects entered into treatment will analyzed for primary end point on ITT bases. For secondary endpoints, analysis of completed and evaluable patients will performed per-protocol.

18.0 Confidentiality

A. Confidentiality of Study Data

Describe the local procedures for maintenance of confidentiality of ***study data and any records that will be reviewed for data collection****.*

18.1 A. Where and how will all data and records be stored? Include information about: password protection, encryption, physical controls, authorization of access, and separation of identifiers and data, as applicable. Include physical (e.g. paper) ***and*** *electronic files.*

Response: All data records will be stored on protected computers with unique passwords issued for each of the study team members and or in locked cabinets within the research center. These files will only be accessible by authorized study personnel.

18.2 A. How long will the data be stored?

Response: De-identified data storage has no expiration date and will be stored for a minimum of 7 years. The researchers may continue to rely on this for future use in research study.

18.3 A. Who will have access to the data?

Response: Those physicians, nurses, and laboratory staff that are on all documentation for the study will have access to the data.

18.4 A. Who is responsible for receipt or transmission of the data?

Response: Those physicians, nurses, and laboratory staff that are on all documentation for the study will have access to the data and can handle transfer of data. Study data will be transferred electronically (through eCRFs) to Robertson Centre for Biostatistics, University of Glasgow, UK

18.5 A. How will the data be transported?

Response: All data are stored at locally and will be transferred to Robertson Centre for Biostatistics, University of Glasgow, UK for data management and analysis. The transfer will be done through electronic CRFs processed on secure network.

Data will also be transported unless when is being archived. At that point files will be transferred to Iron Mountain for storage and archiving.

B. Confidentiality of Study Specimens

Describe the local procedures for maintenance of confidentiality of ***study specimens****.*

☐ **N/A:** No specimens will be collected or analyzed in this research. *(Skip to Section 19.0)*

18.6 B. Where and how will all specimens be stored? Include information about: physical controls, authorization of access, and labeling of specimens, as applicable.

Response: The specimens will be stored in the laboratory located at *1000 Youngs Road, Suite 105, Williamsville NY 14221*and at the CTRC located in 875 Ellicott St. Buffalo NY14203. Samples will be stored in a locked -80º C freezer. All specimens collected at the Glasgow site will be stored in NHS -80º C freezers within the eCRF until shipping to Buffalo (World Courier).

18.7 B. How long will the specimens be stored?

Response: Specimens storage has no expiration date and will be stored for a minimum of 7 years. The researchers may continue to rely on this for future use in research study

18.8 B. Who will have access to the specimens?

Response: Those physicians, nurses, and laboratory staff that are on all documentation for the study will have access to the specimens

18.9 B. Who is responsible for receipt or transmission of the specimens?

Response: Those physicians, nurses, and laboratory staff that are on all documentation for the study will have access to the specimens and can handle transfer of samples. Sample/specimens will transported from University of Glasgow site to the university of Buffalo CTRC site (875 Ellicott St, Buffalo, NY 14203) for storage in -80C until analyzed.

18.10 B. How will the specimens be transported?

Response: All study samples will be centrally stored in UB CTRC site. Therefore, samples collected at the UK site will be transported for analysis and final long-term storage. Transport will be done so using dry ice in a properly labeled Styrofoam container by the laboratory technician. Agreement regarding shipping and receiving the specimens form UK site will be part of the subcontract agreement.

19.0 Provisions to Monitor the Data to Ensure the Safety of Subjects

☐ **N/A:** This study is not enrolling subjects, or is limited to records review procedures only. This section does not apply.

NOTE: Minimal risk studies may be required to monitor subject safety if the research procedures include procedures that present unique risks to subjects that require monitoring. Some examples include: exercising to exertion, or instruments that elicit suicidality or substance abuse behavior. In such cases, N/A is not an acceptable response.

19.1 Describe the plan to periodically evaluate the data collected regarding both harms and benefits to determine whether subjects remain safe.

Response:

An independent Data safety and monitoring board (DSMB) will be established for this trial. The board will consist of independent faculty members from UB and other institutions with sufficient knowledge of the field of type 1 diabetes but not involved in the study. Data Management team at Robertson Centre will periodically provide the data on which to base regular reports to the Data and Safety Monitoring Board. The actual reports will then be generated by the unblinded statistician. The DSMB looks only at safety endpoints and not at futility. The DSMB will convene every six months to review safety end points, side-effects experienced by study subjects and any adverse events reported. The site PIs, CO-Is and study

coordinator(s) will also continuously monitor the data and safety of subjects enrolled in the study. The site PIs and/or the DSMB may recommend to terminate the study or specific aspects of the study if clear harm is observed with one treatment option compared to standard of care or placebo. Specific safety endpoints that will be reviewed include increases in hypoglycemic and ketosis/DKA events.

19.2 Describe what data are reviewed, including safety data, untoward events, and efficacy data.

Response:

Assessment of Efficacy

1- glycemic indices including HbA1c, fructosamine, post-prandial glucose excursions and glucagon. CGM data will be collected as efficacy parameter of glucose control. Total insulin dose will be also documented and analyzed.
2- body weight
3- blood pressure and use of blood pressure medications
4- QOL questionnaires.

The timing of recording of the above efficacy parameters is described in table 1.

Assessment of Safety

The following safety assessments assays and measurement will be reviewed:

1- Vitals, CBC, CMP and markers kidney and liver function will be collected at screening, during and at end of the study as in table 1.
2- Assessment of hypoglycemia: Glucose data will be reviewed from CGM and daily finger stick and during visits.
3- Assessment of ketosis: Ketones levels will be reviewed from daily urinary ketone diaries or blood ketone meters and from urine and blood at the time of each visit
4- Reports of side effects by patients.
5- Insulin dose titrations and pump downloads at each visit

The methods and timing for assessing, recording, and analyzing safety parameters.

The DSMB will convene every six months to assess the side-effects experienced by study subjects and any adverse events reported. The PIs, CO-Is and study coordinator(s) will also continuously monitor the safety of subjects enrolled in the study during their visits and during the safety phone calls.

Procedures for eliciting reports of and for recording and reporting adverse event and inter-current illnesses.

All adverse events will be reported at the time of the visit or by telephone when it occurs. Medical events that occur between the signing of the Informed Consent and the first intake of study drug will be documented in the medical history. Participants will be asked to report any adverse events in general, non-directed questioning. For each AE volunteered by the subject, the investigator will obtain all the information required to complete the adverse event page of the eCRF, in

accordance with the guidelines that accompany it. All adverse events, regardless of seriousness, severity, or presumed relationship to study therapy, will be recorded using medical terminology in the source document. Whenever possible, diagnoses will be given when signs and symptoms are due to a common etiology (e.g., cough, runny nose, sneezing, sore throat, and head congestion should be reported as "upper respiratory infection). Investigators will record on the CRF their opinion concerning the relationship of the adverse event to study therapy. The principal investigators assumes responsibility for appropriate reporting of AEs to the regulatory authorities. The investigator will report these events to the appropriate Independent Ethics Committee/Institutional Review Board (IEC/IRB) in accordance with local regulations. All measures required for AE management will be recorded in the source document and reported according to the State University of New York at Buffalo instructions. Non-serious events/problems that impacted on the rights and welfare of the subjects will be reported as they occur or at the time of knowledge or notification.

19.3 Describe any safety endpoints.

Response: These specific Safety endpoints will be collected:

1- Frequency and severity of hypoglycemia: Hypoglycemia will be assessed throughout the study via multiple daily finger stick glucose measurements, CGM data collected 3 times (about 3 weeks each) during the study and from patient reports during visits and phone calls. Hypoglycemic events will be characterized as in the experts consensus published in *Diabetes Care (2017 Dec; 40(12): 1622-1630):*

- Level 1: Glucose < 70 mg/dL (3.9 mmol/L) and Glucose ≥ 54 mg/dL (3.0 mmol/L)
- Level 2: Glucose < 54 mg/dL (3.0 mmol/L)
- Level 3: A severe event characterized by altered mental and/or physical status requiring assistance.

2- Ketones levels: Urinary ketones measured daily at home and logged in the ketones logbook. Blood and urinary ketones measured during each visit. Ketone levels will be characterized as normal, ketosis and severe ketosis:
-Normal blood ketones <0.6mM or urinary ketones of trace to small (≥+1)
-Ketosis: Blood ketones >0.6mM <2.5mM or urinary ketones of moderate to high (+2 and +3)
-Severe ketosis Blood ketones >2.5mM or urinary ketones of too high (+4)
3- Diabetes ketoacidosis episodes: any confirmed DKA episode (regardless of hospitalization) defined as:
• Elevated serum or urine ketones (greater than the upper limit of the normal range), and
• Serum bicarbonate < 15 mmol/L or Blood pH < 7.3

4- Low blood pressure: <90mmHg SBP and <50 mmHg DBP): Patients will be instructed to measure blood pressure daily and it will be will be assessed in each visit.

5- Nausea, vomiting and other GI side effects. Data will be collected by patients' safety reports, during visits and phone calls.

6- Genital mycotic infections or UTIs

7- Changes in liver and kidney function: CMP and kidney and liver profiles will be assessed as indicated in table 1.

19.4 Describe how the safety information will be collected (e.g., with case report forms, at study visits, by telephone calls with participants).

Response: The safety information will be collected at the time of the visit, and or during preplanned telephone calls with the participant or patient self-reporting (including glucose, food intake and ketones diaries). Data will be entered into eCRFs

19.5 Describe the frequency of safety data collection.

Response: Safety data collection will be done at all study visits and during preplanned phone calls as in table 1. The patient, however, will be asked to report any adverse event or safety related information via phone as soon as it occurs and it will be reviewed the same day.

19.6 Describe who will review the safety data.

Response: DSMB established for this study will review unblended safety data provided by the data management system every 6 months. Additionally, the PIs and coinvestigators will review safety data (listed above) at the completion of each visits by each subject and review any report of side effects or adverse events as they are presented by the patients. The investigators will also review collective blinded data every 3 months to assess the safety and any potential risks to the participants. Furthermore the investigators will assess other risks including the physical, psychological, social, legal and economic harm to these patients. The investigators will carefully watch for any invasion of privacy and breach of confidentiality. The principal investigator will be sharing the results of safety analysis of the study and serious adverse events with the local IRB, DSMB, JDRF, AZ and the FDA. The study groups will remain blinded. If there are a serious safety concerns or an emergency, then, and based on principal investigator discretion, study randomization center in Glasgow University or the unblended statistician will provide information about group assignment of the patient(s) involved. Un-blinded data will be reviewed by the principal investigator and co-investigators and decision to complete, withdraw or adjust dose of drug(s) will be made, documented and shared with IRB and DSMB.

19.7 Describe the frequency or periodicity of review of cumulative safety data.

Response: Safety data will be reviewed every 3 months. Study endpoint data will be reviewed once after half of the recruited patients have completed the study and then at the end of the study.

Response: Safety data will be reviewed every 6 months by DSMB (un-blinded), every 3 months by PIs and CO-Is (blinded) and at every visit by study team.

19.8 Describe the statistical tests for analyzing the safety data to determine whether harm is occurring.

Response: N/A

19.9 Describe any conditions that trigger an immediate suspension of the research.

Response: No interim analysis is planned for efficacy so termination of study will be related to safety. Significant increases in severe hypoglycemic or DKA events as reviewed by DSMB are possible causes that might trigger termination of study.

20.0 Withdrawal of Subjects

☐ **N/A:** This study is not enrolling subjects. This section does not apply.

*20.1 Describe **anticipated** circumstances under which subjects may be withdrawn from the research without their consent.*

1. The site PI can stop investigational study treatment or remove participants from the trial without participants approval if for any reason he/she feels is appropriate, including: severe side effect, injury or medical condition which may place the patient at risk of further complications if he/she continue to participate, failure to take the medication as instructed, failure to keep scheduled appointments, cancellation of the study by the sponsor, IRB, FDA or other administrative reasons.
2. If any of the participants become pregnant during the period of study, they will discontinue study treatment.
3. Participants who experience persistent severe nausea or vomiting after starting semaglutide which is not managed by dose reduction will discontinue the investigational study treatment. Those with mild or moderate nausea will continue for at least one more week. If they continue to experience significant nausea (defined as interfering with normal activities), dose will be reduced. If they continue to have these side effects, they will discontinue study medication(s).
4. Participants who develop a new thyroid tumor during the study will discontinue the study medication(s). Patients will be informed that they should let study investigators know if they develop any swelling in the neck.
5. Developing other conditions included in the exclusion criteria
6. Change in method of insulin delivery between insulin pumps to MDI or vice versa during the study. Also, patients who upgrade to the new "artificial pancreas" pump (Medtronic's MiniMed 670G hybrid) during the study will be withdrawn.

7. Hypoglycemia: Two episodes of severe hypoglycemia (requiring others help).
8. Ketosis (with no other identifiable precipitating factors): Persistent (1week) asymptomatic and unmanaged ketone levels >2.5 mM (+4 in urine) or persistent (1 week) symptomatic and unmanaged ketone levels >1.5 mM (+3 in urine).
9. Total insulin dose of <0.3U/Kg with persistent ketosis (>1.5mM or +3 in urine)
10. DKA: Two episode of DKA (with no identifiable precipitating factors) as defined by the expert consensus statement (*Diabetes Care (2017 Dec; 40(12): 1622-1630)*)
11. Two episodes of UTI.
12. Two episodes of mycotic genital infection.
13. Significant persistent change in eGFR > 15 ml/min
14. Pancreatitis: One episode of pancreatitis.
15. Other persistent side effects specific to study drugs

20.2 Describe any procedures for orderly termination.

NOTE: Examples may include return of study drug, exit interview with clinician. Include whether additional follow up is recommended for safety reasons for physical or emotional health.

Response: Participation in this study is voluntary. Participants have the right to refuse to participate or to withdraw from participation at any time for any reason. Refusal to participate or to withdraw from the study will involve no penalty or loss of entitled benefits, nor affect ongoing medical care. The site PI can stop investigational study treatment or remove participants from the trial without participants approval. If a patient withdraws consent and decide not to participate in the study, she/he will be asked to undergo a final study visit for his/her safety. If a subject withdraws from the research, the data collected to that point will be used toward the research finding. If applicable the subject will have to bring back any unused research drug and or devices.

20.3 Describe procedures that will be followed when subjects withdraw from the research, including retention of already collected data, and partial withdrawal from procedures with continued data collection, as applicable.

Response: We will use the data collected up to the point of stopping the study. No further information will be collected. Patients who stop study treatment and are interested in continuing in the study will be followed up until the end of the study for the primary end point and select secondary endpoints based on the Intent-To-Treat (ITT) analysis model. Withdrawn participants will not be replaced. The study sample size calculation includes an additional 15% subjects to compensate for drop out.

21.0 Risks to Subjects

21.1 List the reasonably foreseeable risks, discomforts, hazards, or inconveniences to the subjects related to their participation in the research. Consider physical, psychological, social, legal, and economic risks. Include a description of the probability, magnitude, duration, and reversibility of the risks.

NOTE: Breach of confidentiality is always a risk for identifiable subject data.

Response:

- Risks associated with intravenous (IV) line: All participants will be informed of the potential complications of an intravenous (IV) line, which include mild bruising at the site of IV line, usually resolving in a few days. They will also be informed about the possibility of infiltration of the IV line at the time of performing blood draws or during the meal challenge test in which case another IV line at a different site will be required (with risk of bruising at more than one site).
- Risks associated with 24 hour Ambulatory BP monitoring device: The BP cuff inflates every 15 minutes to record blood pressure over 24-hours. Participants will experience a pressure-like sensation over the arm where 24 hour BP device is worn at the time of the BP being measured due to the inflation of the cuff and release of this pressure at the time of deflation of the BP cuff. They will be informed to remove the cuff after 24 hours. If worn for longer periods they may experience mild bruising over the arm and some swelling, usually resolving 1-2 days after discontinuation.
- Risks associated with fat tissue aspiration: will probably lead to a bruise at the site of aspiration. The site may be mildly painful for 1-2 weeks. Participants are advised to call their study site staff if they have more severe pain or swelling at the site after the procedure. Rarely there are side effects such as low BP or heart rate and/or allergic reaction to local anesthetic including swelling of the throat.
- All subjects will be referred to semaglutide and dapagliflozin labeling safety information, and will be given a copy of the package insert of the medication. Discussion of the potential side effects and the different warning and precautions will take place at the time of informed consent. Patients will be informed they must not discontinue their insulin during the study.
- Potential risk of insulin, GLP-1 RA and SGLT-2 inhibitors use in type 1 diabetes patients is hypoglycemia. The combination therapy can possibly increase the magnitude and frequency of this risk.
- Potential side effects of GLP-1 RA and SGLT-2 inhibitors also include hypotension, nausea and other GI side effects, polydipsia, polyuria, dehydration, ketosis, and reduced glomerular filtration rate (GFR).
- In rodents, semaglutide causes dose-dependent and treatment-duration-dependent thyroid C-cell tumors at clinically relevant exposures. However, it received FDA-approval for the treatment of type 2 diabetes in 2017 following large Phase 3 trials including the cardiovascular safety trial SUSTAIN-6.
- On the basis of the above rodent studies, semaglutide is contraindicated in those with a personal or family history of medullary thyroid carcinoma and in patients with Multiple Endocrine Neoplasia syndrome type 2 (MEN 2). However, following review of human data with GLP-1RAs, FDA does not require or suggest routine monitoring of serum calcitonin/ thyroid ultrasound in people treated with semaglutide.
- Injection-site reactions: Serious injection-site reactions, with or without bumps (nodules), have happened in some people who use GLP-1RAs but are less frequent with semaglutide. Injection-site reactions have rarely required surgical

intervention. Participants will be instructed to report any injection site reactions including nodules to study investigators.

- Kidney injury: GLP-1 RA might cause acute kidney injury in patients without underlying renal disease especially in patient who had experienced nausea, vomiting, diarrhea or dehydration. Dapagliflozin also has warnings and precautions against acute kidney injury and impairment in renal function.
- As mentioned above, there is a risk of ketoacidosis, as shown by some studies and by our data, when SGLT2 inhibitors are used in type 1 diabetes. In our own preliminary studies on triple therapy, we observed euglycemic ketoacidosis in participants whose insulin doses had been reduced by > 20% of baseline levels. Thus, this adverse effect is largely attributable to insulinopenia as well as anti-insulin effects of SGLT2 inhibitors at the hepatic level (e.g. increased gluconeogenesis).
- The following other known adverse effects of SGLT-2 inhibitors will be discussed with participants when obtaining informed consent:

  1. Risk of dizziness/ symptomatic hypotension: can occur soon after initiation due to intravascular volume contraction.
  2. Impaired kidney function: As SGLT-2 inhibitors increase serum creatinine and decrease eGFR, this will be monitored closely.
  3. Genital mycotic infections: SGLT-2 inhibitors increase the risk of genital mycotic infections, particularly in women. Those with a prior history are more susceptible. Following topical treatment with anti-fungal medications they rarely re-occur.
  4. Bladder Cancer - Across 22 clinical studies, newly diagnosed cases of bladder cancer were reported in 10/6045 patients (0.17%) treated with Dapagliflozin and 1/3512 patient (0.03%) treated with placebo. After excluding patients in whom exposure to study drug was less than one year at the time of diagnosis, there were 4 cases of bladder cancer with dapagliflozin and no cases with placebo i.e. too few cases to determine causality.
  5. The dapagliflozin tablets and matching placebo contain lactose, which may cause discomfort in lactose-intolerant individuals.

- Severe allergic reaction (anaphylaxis) to study drugs or placebo ingredients or other interventions may accrue in rare cases.
- There might be other unforeseen risks associated with the use of these agents in type 1 diabetes and additional unforeseen risks associated with the combination therapy.
- There is always the risk of breach of confidentiality where patients' identifiable data is compromised.

21.2 Describe procedures performed to lessen the probability or magnitude of risks, including procedures being performed to monitor subjects for safety.

Response:

The following are procedures to lessen the probability and magnitude of risks listed above:

1- Hypoglycemia:

- Patients will be instructed to measure glucose at least 4 times a day (before meals and at bedtime) through-out the study and record all reading.
- Insulin dose adjustments will be done at every visit to avoid hypoglycemia. However, there will be no preemptive insulin dose reduction at start of treatment since all patients will have HbA1c >7.5% and the risk of hypoglycemia will be minimal when semaglutide is added.
- Daily fingerstick glucose, CGM and insulin pump data will be reviewed by the investigators for optimal titration of insulin dose to target glucose levels between 90-160mg/dl.
- In general, insulin dose adjustments should not be >10% from current total insulin dose (or 5% if HbA1c is <7%) or >20% from baseline total insulin dose at week 0 and then at week 26.
- Patients will be trained in CARB counting to avoid inaccurate insulin dose administration.
- Patients will be trained to recognize symptoms of hypoglycemia and how to manage it at home. These symptoms include feeling shaky, being nervous or anxious, sweating, chills and clamminess, confusion, fast heartbeat, feeling lightheaded or dizzy, hunger, nausea and color draining from the skin (pallor) among others. They will be asked to call the research office or the on call clinical services.
- When patients feel symptoms of hypoglycemia they will be asked to measure blood glucose and if <70mg/dl they will be instructed to consume carbohydrate or 4 tablets of glucose (4gm each). If after 15 min hypoglycemia continues, repeat carb intake until sugar levels are above 70 mg/dl. If sugar levels are below 55mg/dl, glucagon injection is permitted as rescue medication for severe hypoglycemia.
- Patients will be instructed to inform people who they are in frequent contact with (for example, friends, family members, and coworkers) on how to give glucagon to treat severe hypoglycemia. If patient have needed glucagon previously, it should be documented during screening visit so more emphasis on ways to prevent severe hypoglycemia in the future can be discussed.
- Patients/family will be instructed not to hesitate to call 911 if someone is unconscious and glucagon is not available or someone does not know how to use it and not to inject insulin (it will lower their blood glucose even more) or provide food or fluids (they can choke). They will be asked to document all details of the incident and report to research staff as soon as possible.
- Study medications dose will be reduced in patients who continue to have more than 2 measurements of glucose levels in the hypoglycemic range (<70mg/dl, regardless of symptoms) after insulin dose is reduced by the maximum allowed in this study.

2- Ketosis and Ketoacidosis:

- Patients will be instructed to measure ketone bodies in urine samples daily starting at week 26 with Ketostix (Bayer) and record it in diary provided. Ketones (blood urine) will be measured at every visit. If ketone bodies in urine measure +2 or greater (>4mM in urine), patients are required to notify the research center.

- Patients will be provided training to recognize the signs and symptoms of DKA and to self-test urine ketones on a PRN basis (in addition to once daily) if they develop any symptoms consistent with DKA, including nausea, vomiting, fatigue, confusion, or shortness of breath. This should be reported regardless of blood sugar levels since in type 1 diabetes on SGLT-2 inhibitors, DKA can occur even in normal glucose levels.
- Insulin dose changes should not be greater than 10% (5% if HbA1c is<7%) from current levels and not changed by more than 20% from baseline levels at 0 weeks and then at 26 weeks. Also, on the basis of our data and clinical experience and based of DEPICT-1 study total daily insulin dose should not be lowered below 0.3 U/Kg body weight.
- Patients requiring an insulin dose below 0.3U/Kg with blood ketones levels <1.5mM and no symptoms will be monitored closely for ketones and blood sugar. If levels are stable, patients will be allowed to continue in the study. If total insulin dose is <0.3 U/Kg and ketones levels are >1.5mM, regardless of symptoms, they will be withdrawn from study.
- We shall not increase dose of dapagliflozin in patients who exhibit significant ketonuria (+3) with the lower dose. Urinary ketones (and blood ketones if patients have a dual glucose and ketone meter) will be monitored daily and reported. Dapagliflozin will be temporarily (1 week) discontinued and STICH protocol (STop SGLT-2i, give Insulin bolus, Carbohydrate, and Hydration) activated in events of DKA or if based on investigator discretion if blood ketone levels are persistently (1 week) >1.5 mM (+3 in urine) with symptoms or >2.5 mM (+4 in urine) without symptom. These cut-off levels are below the cut-off levels of DKA >7-10mM. Dapagliflozin may also be temporarily stopped in clinical situations known to predispose to ketoacidosis (e.g. prolonged fasting due to acute illness or surgery). Patients will be instructed to stay hydrated and not reduce their caloric intake (especially carbohydrates) or start new dietary systems (especially ketogenic diets) during the study.
- If a patient develops symptoms of DKA or ketone levels exceed the allowed limit in this study, STICH protocol (STop SGLT-2i, give Insulin bolus, Carbohydrate, and Hydration) will be activated. They could be managed for up to a week. If symptoms and ketone levels persist over 1 week, patients will be withdrawn form study. Insulin infusions may be administered by ER or hospital if patients develop DKA. They will be asked to document all details of the incident and report to research staff as soon as possible.

3- Risk of acute kidney injury or renal function impairment: CBC, CMP, including kidney function test and urinary markers will be performed at screening, during and at end of the study (table 1). Additional testing will be performed in patients who suffer from sever nausea or GI symptoms or hypovolemic and during the up titration of investigation drugs. If acute kidney injury occurs dapagliflozin will be discontinued immediately. To lessen the chance of this risk, patients will eGFR<60 ml/min/1.73m^2 will be excluded. Patients will also discontinue study medications if eGFR falls by >15 ml/min in 2 repeated measurements. Also other patients with factors that may predispose patients to acute kidney injury e.g. hypovolemia, chronic renal insufficiency, congestive heart failure, if patients are

on AEI, ARB, NSAID will be excluded. Dapagliflozin will be temporarily discontinued in any setting of reduced oral intake such as acute illness or fasting or fluid losses such as gastrointestinal illnesses, or excessive heat exposure.

4- Injection site reactions, mycotic infections and complications of IV and biopsy collection will also be monitored, Physical examination will be performed at screening 26, and 52 weeks visits. Safety vital assessments will be performed at every visit. Pregnancy will be tested at screening, 0, 4 13, 26, 39, and 52 weeks. Phone calls will be made for safety and compliance at least 7 time during the study. The patients, however, will be instructed to report any adverse event or safety related information via phone as soon as it occurs. All safety data will be reviewed by the PI or sub-investigators within 1-2 days of collection.

5- Epinephrine is the primary treatment for anaphylaxis with no absolute contraindication to its use. Our site and staff are trained and equipped to assist patients with such reactions. Severe conditions that are not satisfactory managed in site will be transferred to the hospital emergency department for further assessment and help.

21.3 If applicable, indicate ***which procedures*** *may have risks to the subjects that are currently unforeseeable.*

Response:

There might be additional adverse effects of dapagliflozin not currently known or those unique to their use in Type 1 diabetes. There is no previous studies on the use of semaglutide in type 1 diabetes; therefore, additional unforeseen risk may be encountered in this study. Additionally, unforeseen risks might be associated with the combination therapy that might involve unknown drug-drug interactions.

21.4 If applicable, indicate which research procedures may have risks to an embryo or fetus should the subject be or become pregnant.

Response: Not applicable, we will not be enrolling pregnant participants or who may become pregnant or with in child baring years without signing the consent stating they will use at least two forms of birth control. If a participant becomes pregnant they will be withdrawn from the study immediately. They will be followed until term and any effects on the newborn will be documented.

21.5 If applicable, describe risks to others who are not subjects.

Response: We do not anticipate any additional risk to others not participating in this study.

22.0 Potential Benefits to Subjects

22.1 Describe the potential benefits that individual subjects may experience by taking part in the research. Include the probability, magnitude, and duration of the potential benefits. Indicate if there is no direct benefit.

*NOTE: Compensation **cannot** be stated as a benefit.*

Response: The investigational drugs used in this study are already approved as antidiabetic agents for treating type 2 diabetes. Both agents have demonstrated significant improvement in HbA1c, weight and blood pressure. It is our expectation that patients with type 1 diabetes will experience similar benefits compared to those who are treated with insulin alone. The magnitude and duration of the benefits and the safety profile may however differ and are the focus of this investigation.

23.0 Compensation for Research-Related Injury

☐ **N/A:** The research procedures for this study do not present risk of research related injury (e.g. survey studies, records review studies). This section does not apply.

*23.1 **If the research procedures carry a risk of research related injury,** describe the available compensation to subjects in the event that such injury should occur.*

Response: Routinely, Buffalo General Hospital, Erie County Medical Center, and/or the University at Buffalo, State University of New York, its agents, or its employees do not compensate for or provide free medical care for human subjects/participants in the event that any injury results from participation in a human research project. In the unlikely event that they become ill or injured as a direct result of participating in this study, they may receive medical care. Your study doctor will explain the treatment options to you and tell you where you can get treatment. Generally, this care will be billed to you, your insurance or other third party.

23.2 Provide a copy of contract language, if any, relevant to compensation for research related injury.

*NOTE: If the contract is not yet approved at the time of this submission, submit the current version here. If the contract is later approved with **different language regarding research related injury**, you must modify your response here and submit an amendment to the IRB for review and approval.*

Response: **N/A**

24.0 Economic Burden to Subjects

24.1 Describe any costs that subjects may be responsible for because of participation in the research.

NOTE: Some examples include transportation or parking.

Response: All research expenses will be covered by the study. The background use of insulin is considered as part of standard of care is the responsibility of the patients and his/her insurance. Participants will not be subjected to any other out of pocket cost.

☐ **N/A:** This study is not enrolling subjects, or is limited to records review procedures only. This section does not apply.

25.0 Compensation for Participation

25.1 Describe the amount and timing of any compensation to subjects, including monetary, course credit, or gift card compensation.

Response:
Visit schedule:

- Screen: $0
- Visit 1 (Week -3): Insulin adjustment: $10
- Visit 2 (Week -1): Meal ($75), insulin adjustment, and 24hr blood pressure monitor ($50): $125*
- Visit 3 (Week 0): 24 hr urine ($15), blood draw ($25), fat biopsy ($75), insulin adjustment, questionnaire ($10): $125
- Visit 4 (Week 1): Insulin adjustment: $10
- Visit 5 (Week 2): Insulin adjustment: $10
- Visit 6 (Week 4): Insulin adjustment: $10
- Visit 7 (Week 13): 24hr urine ($15), blood draw ($25), insulin adjustment, 24hr bp monitor ($50): $90*
- Visit 8 (Week 23): Insulin adjustment: $10
- Visit 9 (Week 25): Insulin adjustment, 24bp monitor ($50), Meal ($75): $125*
- Visit 10 (Week 26): Insulin adjustment, 24hr urine ($15), blood draw ($25), fat biopsy ($75), questionnaire ($10): $125*
- Visit 11 (Week 27): Insulin adjustment: $10
- Visit 12 (Weak 31): Insulin adjustment, blood draw: $25
- Visit 13 (Week 39): Insulin adjustment, 24hr urine ($15), blood draw ($25), 24hr bp monitor ($50): $90*
- Visit 14 (Week 49): Insulin adjustment: $10
- Visit 15 (Week 51): Insulin adjustment, 24bp monitor ($50), meal ($75): $125*
- Visit 16 (Week 52): Insulin adjustment, 24hr urine ($15), blood draw ($25), questionnaire ($10), fat biopsy ($75):$125*

  *Total compensation for all completed visits: up to $1,025.00. For those not wishing to participate in the optional blood pressure monitoring and/or fat biopsy procedure, the compensation will decrease by $250 for not completing the bp monitoring, and by yet another $225.00 for not completing the fat biopsies.

☐ **N/A:** This study is not enrolling subjects, or is limited to records review procedures only. This section does not apply.

☐ **N/A:** There is no compensation for participation. This section does not apply.

26.0 Consent Process

26.1 Indicate whether you will be obtaining consent.

NOTE: This does not refer to consent documentation, but rather whether you will be obtaining permission from subjects to participate in a research study. Consent documentation is addressed in Section 27.0.

☒ ***Yes*** *(If yes, Provide responses to each question in this Section)*
☐ ***No*** *(If no, Skip to Section 27.0)*

26.2 Describe where the consent process will take place. Include steps to maximize subjects' privacy.

Response: All participants will come to the research department to be consented. Participants will be placed in a private, screened off area and or room where they can review the consent. Participant questions and or concerns will be address with a member of the study team or research doctor if applicable. The research coordinator will discuss in length the participants requests for privacy of their PHI.

26.3 Describe how you will ensure that subjects are provided with a sufficient period of time to consider taking part in the research study.

NOTE: It is always a requirement that a prospective subject is given sufficient time to have their questions answered and consider their participation. See "SOP: Informed Consent Process for Research (HRP-090)" Sections 5.5 and 5.6.

Response: participants will be made aware that participating in research is completely voluntary, and they may withdraw at any time with no consequence to their routine clinic care. If the patients requires time to decide and or discuss partaking in a research study, the subject will be given said time.

26.4 Describe any process to ensure ongoing consent, defined as a subject's willingness to continue participation for the duration of the research study.

Response: The research coordinator and study team are available to answer any question or concerns with the patient during the duration of the research trial. At each study visit, the patient is asked a series of questions to ensure they are on task with the study visits and feel comfortable. Upon departing from their study visit, the patients are told of their next visit and given detail instruction for their next visit. If study is revised or amendment or new information becomes available

about drug safety that may affect patients participation, the patient may be re-consented to ensure patient ongoing consent

26.5 *Indicate whether you will be following "SOP: Informed Consent Process for Research (HRP-090)." If not, or if there are any exceptions or additional details to what is covered in the SOP, describe:*

- *The role of the individuals listed in the application who are involved in the consent process*
- *The time that will be devoted to the consent discussion*
- *Steps that will be taken to minimize the possibility of coercion or undue influence*
- *Steps that will be taken to ensure the subjects' understanding*

Response:

☒ We have reviewed and will be following "SOP: Informed Consent Process for Research (HRP-090)."

Non-English Speaking Subjects

☒ **N/A:** This study will not enroll Non-English speaking subjects.
(Skip to Section 26.8)

26.6 *Indicate which language(s) other than English are likely to be spoken/understood by your prospective study population or their legally authorized representatives.*

NOTE: The response to this Section should correspond with your response to Section 6.4 of this protocol.

Response:

26.7 *If subjects who do not speak English will be enrolled, describe the process to ensure that the oral and written information provided to those subjects will be in that language. Indicate the language that will be used by those obtaining consent.*

NOTE: Guidance is provided on "SOP: Informed Consent Process for Research (HRP-090)."

Response:

Cognitively Impaired Adults

☒ **N/A**: This study will not enroll cognitively impaired adults.
(Skip to Section 26.9)

26.8 Describe the process to determine whether an individual is capable of consent.

Response:

Adults Unable to Consent

☒ **N/A**: This study will not enroll adults unable to consent.
(*Skip to Section 26.13)*

When a person is not capable of consent due to cognitive impairment, a legally authorized representative should be used to provide consent (Sections 26.9 and 26.10) ***and, where possible, assent of the individual should also be solicited*** *(Sections 26.11 and 26.12).*

26.9 Describe how you will identify a Legally Authorized Representative (LAR). Indicate that you have reviewed the "SOP: Legally Authorized Representatives, Children, and Guardians (HRP-013)" for research in New York State.

NOTE: Examples of acceptable response includes: verifying the electronic medical record to determine if an LAR is recorded.

Response:

☐

26.10 ***For research conducted outside of New York State****, provide information that describes which individuals are authorized under applicable law to consent on behalf of a prospective subject to their participation in the research. One method of obtaining this information is to have a legal counsel or authority review your protocol along with the definition of "legally authorized representative" in "SOP: Legally Authorized Representatives, Children, and Guardians (HRP-013)."*

Response:

26.11 Describe the process for ***assent of the adults****:*

- *Indicate whether assent will be obtained from all, some, or none of the subjects. If some, indicate which adults will be required to assent and which will not.*

  Response:

- *If assent will not be obtained from some or all subjects, provide an explanation of why not.*

Response:

*26.12 Describe whether **assent of the adult** subjects will be documented and the process to document assent.*

NOTE: The IRB allows the person obtaining assent to document assent on the consent document using the "Template Consent Document (HRP-502)" Signature Block for Assent of Adults who are Legally Unable to Consent.

Response:

Subjects who are not yet Adults (Infants, Children, and Teenagers)

☒ **N/A**: This study will not enroll subjects who are not yet adults. *(Skip to Section 27.0)*

*26.13 Describe the criteria that will be used to determine **whether a prospective subject has not attained the legal age for consent to treatments or procedures involved in the research** under the applicable law of the jurisdiction in which the research will be conducted **(e.g., individuals under the age of 18 years)**. For research conducted in NYS, review "SOP: Legally Authorized Representatives, Children, and Guardians (HRP-013)" to be aware of which individuals in the state meet the definition of "children."*

NOTE: Examples of acceptable responses include: verification via electronic medical record, driver's license or state-issued ID, screening questionnaire.

Response:

*26.14**For research conducted outside of New York State**, provide information that describes which persons have not attained the legal age for consent to treatments or procedures involved the research, under the applicable law of the jurisdiction in which research will be conducted. One method of obtaining this information is to have a legal counsel or authority review your protocol along the definition of "children" in "SOP: Legally Authorized Representatives, Children, and Guardians (HRP-013)."*

Response:

26.15 Describe whether parental permission will be obtained from:

Response: N/A

☐ One parent even if the other parent is alive, known, competent, reasonably available, and shares legal responsibility for the care and custody of the child.

☐ Both parents unless one parent is deceased, unknown, incompetent, or not reasonably available, or when only one parent has legal responsibility for the care and custody of the child.

☐ Parent permission will not be obtained. A waiver of parent permission is being requested.

NOTE: The requirement for parent permission is a protocol-specific determination made by the IRB based on the risk level of the research. For guidance, review the "CHECKLIST: Children (HRP-416)."

*26.16 Describe whether permission will be obtained from individuals **other than parents**, and if so, who will be allowed to provide permission. Describe your procedure for determining an individual's authority to consent to the child's general medical care.*

Response:

*26.17 Indicate whether assent will be obtained from all, some, or none of the **children**. If assent will be obtained from some children, indicate which children will be required to assent.*

Response:

26.18 When assent of children is obtained, describe how it will be documented.

Response:

27.0 Waiver or Alteration of Consent Process

Consent will not be obtained, required information will not be disclosed, or the research involves deception.

☒ **N/A:** A waiver or alteration of consent is not being requested.

27.1 If the research involves a waiver or alteration of the consent process, please review the "CHECKLIST: Waiver or Alteration of Consent Process (HRP-410)" to ensure that you have provided sufficient information for the IRB to make the determination that a waiver or alteration can be granted.

NOTE: For records review studies, the first set of criteria on the "CHECKLIST: Waiver or Alteration of Consent Process (HRP-410)" applies.

Response:

27.2 If the research involves a waiver of the consent process for planned emergency research, please review the "CHECKLIST: Waiver of Consent for Emergency Research (HRP-419)" to ensure you have provided sufficient information for the IRB to make these determinations. Provide any additional information necessary here:

Response:

28.0 Process to Document Consent

☐ **N/A:** A Waiver of Consent is being requested.
(Skip to Section 29.0)

28.1 Indicate whether you will be following "SOP: Written Documentation of Consent (HRP-091)." If not or if there are any exceptions, describe whether and how consent of the subject will be obtained including whether or not it will be documented in writing.

NOTE: If your research presents no more than minimal risk of harm to subjects and involves no procedures for which written documentation of consent is normally required outside of the research context, the IRB will generally waive the requirement to obtain written documentation of consent. This is sometimes referred to as 'verbal consent.' Review "CHECKLIST: Waiver of Written Documentation of Consent (HRP-411)" to ensure that you have provided sufficient information.

If you will document consent in writing, attach a consent document with your submission. You may use "TEMPLATE CONSENT DOCUMENT (HRP-502)". If you will obtain consent, but not document consent in writing, attach the script of the information to be provided orally or in writing (i.e. consent script or Information Sheet).

Response:

☒ We will be following "SOP: Written Documentation of Consent" (HRP-091).

29.0 Multi-Site Research (Multisite/Multicenter Only)

☐ **N/A:** This study is not an investigator-initiated multi-site study. This section does not apply.

29.1 If this is a multi-site study ***where you are the lead investigator****, describe the processes to ensure communication among sites, such as:*

- *All sites have the most current version of the IRB documents, including the protocol, consent document, and HIPAA authorization.*
- *All required approvals have been obtained at each site (including approval by the site's IRB of record).*

- *All modifications have been communicated to sites, and approved (including approval by the site's IRB of record) before the modification is implemented.*
- *All engaged participating sites will safeguard data as required by local information security policies.*
- *All local site investigators conduct the study appropriately.*
- *All non-compliance with the study protocol or applicable requirements will be reported in accordance with local policy.*

Response:

29.2 Describe the method for communicating to engaged participating sites:

- *Problems*
- *Interim results*
- *Study closure*

Response:

29.3 Indicate the total number of subjects that will be enrolled or records that will be reviewed across all sites.

Response: 114

29.4 If this is a multicenter study for which UB will serve as the IRB of record, and subjects will be recruited by methods not under the control of the local site (e.g., call centers, national advertisements) describe those methods.

Response: N/A

30.0 Banking Data or Specimens for Future Use

☐ **N/A:** This study is not banking data or specimens for future use or research outside the scope of the present protocol. This section does not apply.

30.1 If data or specimens will be banked (stored) for ***future use, that is, use or research outside of the scope of the present protocol****, describe where the data/specimens will be stored, how long they will be stored, how the data/specimens will be accessed, and who will have access to the data/specimens.*

NOTE: Your response here must be consistent with your response at the "What happens if I say yes, I want to be in this research?" Section of the Template Consent Document (HRP-502).

Response: The study data/specimens will be stored in a locked closet or -80 freezer at the research facility of the Diabetes and Endocrinology Center of WNY for at least 7 years.

The research staff (study personnel including coordinator) only will be authorized to access data and or specimens

30.2 List the data to be stored or associated with each specimen.

Response: Patient ID number, study visit information and date of collection will be stored with specimen. Other data stored will include record files of all patients participating in the study, including data collection sheets, lab results, CGM and insulin pump data.

30.3 Describe the procedures to release banked data or specimens for future uses, including: the process to request a release, approvals required for release, who can obtain data or specimens, and the data to be provided with specimens.

Response: The copy of the individual patient data collected during the study period will be provided to these individual patients who can choose to hand carry it to their respective physicians and a copy will be faxed to their respective clinical providers upon verbal request from the patient. The data provided will include the insulin pump or CGM data or any of the lab results obtained during the study period. The results of the completed study will be made available to the patients if requested through published manuscript.

31.0 Drugs or Devices

☐ **N/A:** This study does not involve drugs or devices. This section does not apply.

31.1 If the research involves drugs or devices, list and describe all drugs and devices used in the research, the purpose of their use, and their regulatory approval status.

Response: **Drugs used:**

Description or Identity of investigational product(s)

Investigational product	***Dosage form and strength***	***Manufacturer***	***Description***
Dapagliflozin (Farxiga) 5 mg	*Green, plain, diamond shaped, film coated 5 mg tablet*	*AstraZeneca*	*sodium-glucose cotransporter 2 (SGLT2) inhibitor indicated as an adjunct to diet and exercise to improve glycemic control*
Matching placebo for dapagliflozin 5 mg	*Green, plain, diamond shaped, film coated tablet*	*AstraZeneca*	

Semaglutide (Ozempic) 0.25 mg, 0.5 mg, 1.0 mg	*clear, colorless solution that contains 2 mg of semaglutide in a 1.5 mL (1.34 mg/mL) pre-filled, disposable, single-patient-use pen injector*	*Novo Nordisk*	*glucagon-like peptide 1 (GLP-1) receptor agonist indicated as an adjunct to diet and exercise to improve glycemic control*

- *Dapagliflozin and its matching placebo tablets will be supplied in bottles and are provided by Astra Zeneca.*
- *Semaglutide will be supplied as prefilled injectable pens and will be purchased commercially through the University at Buffalo Research Pharmacy.*

Subjects will receive drugs in their unmodified form. Dapagliflozin is a once daily tablet, while Semaglutide is a weekly subcutaneous injection.

Insulin pumps (CSII) and long and short acting insulin is the only diabetes medication accepted before and during this trial. No other diabetes drug is permitted.
Blood pressure or lipid lowering drugs are permitted if on stable dose for at least three months prior to start of trial. Glucagon injection is permitted as rescue medication for severe hypoglycemia. Insulin infusions may be administered by ER or hospital if patients develop DKA.

31.2 Describe your plans to store, handle, and administer those drugs or devices so that they will be used only on subjects and be used only by authorized investigators.

Response: Dapagliflozin and matching placebo provided by Astra Zeneca will be stored in a locked cabinet in research pharmacy. Semaglutide provided by University at Buffalo research pharmacy will be stored in temperature controlled refrigerator at 4C at the research facility of the Diabetes and Endocrinology Center of WNY at 1000 Youngs Rd until dispensed to patients. Investigational drugs will be dispensed by PI or CO-Is only to study patients according to randomization plan.

If the drug is investigational (has an IND) or the device has an IDE or a claim of abbreviated IDE (non-significant risk device), include the following information:

31.3 Identify the holder of the IND/IDE/Abbreviated IDE.

Response: IND application for use of all drugs (dapagliflozin and semaglutide) in type 1 diabetes will be submitted to FDA and no study procedure will start before all local and federal approvals are obtained. FDA has granted IND for usage of dapagliflozin in type 1 diabetes. We will seek FDA approval for the use of semaglutide in this study. These IND letters will be submitted to IRB and JDRF along with final version of protocol before initiating any study activities.

31.4 Explain procedures followed to comply with FDA sponsor requirements for the following:

	Applicable to:		
FDA Regulation	***IND Studies***	***IDE studies***	***Abbreviated IDE studies***
21 CFR 11	***X***	***X***	
21 CFR 54	***X***	***X***	
21 CFR 210	***X***		
21 CFR 211	***X***		
21 CFR 312	***X***		
21 CFR 812		***X***	***X***
21 CFR 820		***X***	

Response: All FDA sponsor requirements have been reviewed and will be followed during the study procedures

32.0 Humanitarian Use Devices

☒ **N/A:** This study does not involve humanitarian use devices. This does not apply.

32.1 For Humanitarian Use Device (HUD) uses provide a description of the device, a summary of how you propose to use the device, including a description of any screening procedures, the HUD procedure, and any patient follow-up visits, tests or procedures.

Response:

32.2 For HUD uses provide a description of how the patient will be informed of the potential risks and benefits of the HUD and any procedures associated with its use.

Response:

Section	Original	New	Justification
1.2	Primary: combined dapagliflozin and semaglutide treatment in addition to insulin will improve HbA1c levels compared with semaglutide in addition to insulin (by ≥0.5%) or insulin therapy alone (by ≥1%).	Primary: combined dapagliflozin and semaglutide treatment in addition to insulin will improve HbA1c levels compared with semaglutide in addition to insulin.	the thresholds for HbA1c improvement are removed due to risk thresholds set in the primary objective are unmet in the study, and the study could be considered to not have fully met the primary objective.
1.2	Secondary 1: semaglutide in addition to insulin treatment over 26 and 52 weeks will improve HbA1c levels compared with insulin therapy alone.	Secondary 1: semaglutide in addition to insulin treatment over 26 weeks will improve HbA1c levels compared with insulin therapy alone.	
1.2	Secondary 2: combined dapagliflozin and semaglutide treatment in addition to insulin will improve CGM based weekly mean and fasting blood glucose, glycemic oscillations and percent of time in range (80-160mg/dl) with lower insulin requirements compared with semaglutide and insulin (dual therapy) or insulin only (standard) treatment.	Secondary 2: combined dapagliflozin and semaglutide treatment in addition to insulin will improve CGM based weekly mean and fasting blood glucose, glycemic oscillations and percent of time in range (70-180mg/dl) with lower insulin requirements compared with semaglutide and insulin (dual therapy) or insulin only (standard) treatment.	
1.2	Secondary 2: combined dapagliflozin and semaglutide treatment in addition to insulin will improve CGM based weekly mean and fasting blood glucose, glycemic oscillations and percent of time in range (80-160mg/dl) with lower insulin requirements compared with semaglutide and insulin (dual therapy) or insulin only (standard) treatment.	Tertiary 1: combined dapagliflozin and semaglutide treatment in addition to insulin therapy will improve fasting and postprandial glucose excursions and suppress postprandial glucagon, FFA, GLP-1 compared with semaglutide and insulin or insulin therapy alone.	Changed from secondary 2 into Tertiary 1 and 2

1.2	no statement	Tertiary 2: semaglutide in addition to insulin treatment over 52 weeks will improve HbA1c levels compared with insulin therapy alone.	additional data analysis
2.1	Change in HbA1c from baseline at six and 12 months in dual therapy group compared to insulin only group.	Change in HbA1c from baseline at 6 months in dual therapy group compared to insulin only group.	
2.1	Other indices of glycemic control: fructosamine, weekly mean and fasting glucose, standard deviation, insulin requirements, postprandial glucose excursions and postprandial (Area Under curve) FFA, GLP-1 and glucagon.	Other indices of glycemic control: fructosamine, weekly mean and fasting glucose, standard deviation, insulin requirements.	Items in red- changed into #1 in tertiary
3.1		Due to the potential ketogenic effect of SGLT-2 inhibitors, data from SGLT-2 inhibitors studies in type 1 diabetes were analyzed for it ketogenic effects and a consensus statement and guidelines on management of patients was recently published (Danne T et al., Diabetes Care. 2019 Feb 6).	added this statement to new protocol before preliminary data
6.1	HbA1c > 7.5%	HbA1c between 7.5-11% inclusive	per FDA letter from 3/26/2019
6.1	C-peptide >0.23 nM	C-peptide ≤ 0.23 nM	correct typo
6.1	Regularly measuring blood sugars four or more times daily	Regularly measuring blood sugars four or more times daily or use of CGM	Add CGM as method of monitoring glucose for patients who have their own CGM instead of finger stick

6.2	Combined exclusion 11 and 15 with 10		
6.2	No statement	Patients with any malignancy except treated in situ malignancy and basal cell carcinoma of the skin	added this exclusion as #28
6.2	No statement	Patients on ketogenic or low (<50g/day) carbohydrate diet or who practice intermittent fasting.	added based on FDA letter from 3/26/2019
10.1	Study Visit 7: deleted "the next"	Added "10"	Changed number of times we are distributing study drug
10.1	Study Visit 8: deleted "supply of study drug will be given to last until the next visit"		Changed number of times we are distributing study drug
10.1	Study visit 10: Refer to protocol, many changes		
10.1	Study visit 11: refer to protocol, many changes		
10.1	Study Visit 12: deleted "supply of study drug will be given to last until the next visit"		Changed number of times we are distributing study drug

10.1	Study visit 12: changed week 39 to visit 13		
10.1	Study visit 13: changed "next visit" to "end of study		
10.1	Study Visit 14: deleted "supply of study drug will be given to last until the next visit"		
10.1	Study Visit 15: deleted "supply of study drug will be given to last until the next visit"		
10.1	Chart- many changes made		
10.1	no statement	Screening visit: added C-peptide to list of blood drawn. Also added to chart	additional testing
10.1	no statement	Study visit 9: Add HbA1c testing and measurement of ketones	Phase 2 randomization will be balanced in terms of HbA1c values. Ketones will be measured to insure patients are not ketogenic at start of dapagliflozin treatment
10.1	Dapagliflozin (or placebo) will be started at a dose of 5 mg daily at week 26 and increased after 1 week to 10 mg (2x5mg tablets) daily to ensure drug tolerability and to avoid any hypoglycemia or hypotension.	. Dapagliflozin (or placebo) will be started at a dose of 5 mg daily at week 26 and if well tolerated increased after 1 week to 10 mg (2x5mg tablets) daily to ensure drug tolerability and to avoid any hypoglycemia or hypotension.	additional safety

10.1	no statement	Patients who do not tolerate the increase in dosing of either medications will be maintained at the highest tolerated dose.	as per FDA letter 3/26/2019
10.1	Astra Zeneca Inc. will supply dapagliflozin and matching placebo tablets in identical packaging; semaglutide pens will be obtained commercially and will be used in an open label fashion.	Astra Zeneca Inc. will supply dapagliflozin and matching placebo tablets in unlabeled and identical packaging; semaglutide pens will be obtained commercially and will be used in an open label fashion.	clarity
10.1	Meal challenge: no statement	Spot urine will be collected at baseline, 1 and 3hr.	additional sample collection
10.1 drug blinding	Each randomization will be stratified by study site	Each randomization will be stratified by study site and the second randomization will also include HbA1c stratification.	to avoid HbA1c imbalance between groups in 2nd phase
17.1	no statement	Since the secondary end-points are pre-specified and may as well be correlated, multiplicity control for multiple tests across secondary endpoints may not be necessary. However, Holm-Bonferroni correction for multiplicity testing will be implemented if additional endpoints are tested or if the pre-specified secondary end-points are found to be not correlated.	statistical justification

19.2	no statement	Included definition of adverse event, serious adverse event, who to report to, etc. Look at section for changes made	As per FDA letter 3/26/2019 and Astra Zeneca
20.1	Many changes made, look at this section		
21.1	• As mentioned above, there is a risk of ketoacidosis, as shown by some studies and by our data, when SGLT2 inhibitors are used in type 1 diabetes. In our own preliminary studies on triple therapy, we observed euglycemic ketoacidosis in participants whose insulin doses had been reduced by > 20% of baseline levels. Thus, this adverse effect is largely attributable to insulinopenia as well as anti-insulin effects of SGLT2 inhibitors at the hepatic level (e.g. increased gluconeogenesis).	• As mentioned above, there is a risk of ketoacidosis, as shown by some studies and by our data, when SGLT2 inhibitors are used in type 1 diabetes. In our own preliminary studies on triple therapy, we observed euglycemic ketoacidosis in participants whose insulin doses had been reduced by > 20% of baseline levels or in patients who are not adequately insulinzed. Thus, this adverse effect is largely attributable to insulinopenia as well as anti-insulin effects of SGLT2 inhibitors at the hepatic level (e.g. increased gluconeogenesis).	
21.2	Daily fingerstick glucose, CGM and insulin pump data will be reviewed by the investigators for optimal titration of insulin dose to target glucose levels between 90-160mg/dl.	Daily fingerstick glucose, CGM and insulin pump data will be reviewed by the investigators for optimal titration of insulin dose to target glucose levels between 90-180mg/dl.	

University at Buffalo
The State University of New York
University at Buffalo Institutional Review Board (UBIRB)
Office of Research Compliance | Clinical and Translational Research Center Room 5018
875 Ellicott St. | Buffalo, NY 14203
UB Federalwide Assurance ID#: FWA00008824

Complete Research Protocol (HRP-503)

Table of Contents

Template Instructions 2
1.0 Objectives 4
2.0 Scientific Endpoints 5
3.0 Background 6
4.0 Study Design 12
5.0 Local Number of Subjects 13
6.0 Inclusion and Exclusion Criteria 13
7.0 Vulnerable Populations 16
8.0 Eligibility Screening 17
9.0 Recruitment Methods 18
10.0 Procedures Involved 19
11.0 Study Timelines 32
12.0 Setting 32
13.0 Community-Based Participatory Research 33
14.0 Resources and Qualifications 34
15.0 Other Approvals 35
16.0 Provisions to Protect the Privacy Interests of Subjects 35
17.0 Data Management and Analysis 36
18.0 Confidentiality 39
A. Confidentiality of Study Data 39
B. Confidentiality of Study Specimens 40
19.0 Provisions to Monitor the Data to Ensure the Safety of Subjects 41
20.0 Withdrawal of Subjects 48
21.0 Risks to Subjects 49
22.0 Potential Benefits to Subjects 54
23.0 Compensation for Research-Related Injury 55
24.0 Economic Burden to Subjects 56
25.0 Compensation for Participation 56
26.0 Consent Process 57
27.0 Waiver or Alteration of Consent Process 62
28.0 Process to Document Consent 62
29.0 Multi-Site Research (Multisite/Multicenter Only) 63
30.0 Banking Data or Specimens for Future Use 64
31.0 Drugs or Devices 64
32.0 Humanitarian Use Devices 66

Template Instructions

Sections that do not apply:

- *In several sections, the addition of checkboxes for* ***Not Applicable*** *have been added to the template as responses.*
    - *If an N/A checkbox is present, select the appropriate justification from the list.*
    - *If an N/A checkbox is not present, or if none of the existing checkboxes apply to your study, you must write in your own justification.*
- *In addition:*
    - *For research where the only study procedures are records/chart review: Sections 19, 20, 22, 23, 24, 25, 31, and 32 do not apply.*
    - *For exempt research: Sections 31 and 32 do not apply.*

Studies with multiple participant groups:

- *If this study involves multiple participant groups (e.g. parents and children), provide information in applicable sections for each participant group. Clearly label responses when they differ. For example:*

Response:

Intervention Group:

Control Group:

Formatting:

- *Do not remove template instructions or section headings when they do not apply to your study.*

If you are pasting information from other documents using the "Merge Formatting" Paste option will maintain the formatting of the response boxes.

Amendments:

- *When making modifications or revisions to this and other documents, use the* ***Track Changes*** *function in Microsoft Word.*
- *Update the version date or number* ***on Page 3.***

PROTOCOL TITLE:

Include the full protocol title.

Response: **Triple Therapy for Type 1 Diabetes with Insulin, Semaglutide, and Dapagliflozin**

PRINCIPAL INVESTIGATOR:

Response:

**Principal Investigator:**	**Paresh Dandona, MBBS, PhD**
**Co- Investigators:**	**Husam Ghanim, PhD**
	**Antoine Makdissi, MD**
	**Manav Batra, MD**
**Co- Principal Investigator:**	**John R Petrie, MBChB, PhD**

Paresh Dandona, M.B.B.S., Ph.D., F.R.C.P, F.A.C.P, F.A.C.C, FACE,
SUNY Distinguished Professor,
Director, Diabetes-Endocrinology Center of Western NY
Chief of Endocrinology, Diabetes, Endocrinology and Metabolism, SUNY at Buffalo
1000 Youngs Road, Suite 105
Williamsville NY 14221
Telephone: (716) 535-1850
Fax: 716-639-1200
E-mail: pdandona@kaleidahealth.org

CO-PI:

Professor John R Petrie
BSc MBChB PhD FRCP(Ed) FRCPSG
Professor of Diabetic Medicine
Institute of Cardiovascular and Medical Sciences
BHF Glasgow Cardiovascular Research Centre
University of Glasgow
126 University Place
Glasgow G12 8TA
Tel: +44(0)141 330 3325
Email: john.petrie@glasgow.ac.uk
Web: http://www.gla.ac.uk/researchinstitutes/icams/staff/johnpetrie/
The University of Glasgow

VERSION:

Include the version date or number.

Response: 9/25/2019, V2

GRANT APPLICABILITY:

Indicate whether this protocol is funded by a grant (e.g. NIH, foundation grant). For a grant with multiple aims, indicate which aims are covered by this research proposal.

NOTE: This question does not apply to studies funded by a sponsor contract.

Include a copy of the grant proposal with your submission.

Response: Research support provided by JDRF and AZ

RESEARCH REPOSITORY:

Indicate where the research files will be kept, including when the study has been closed. The repository should include, at minimum, copies of IRB correspondence (approval, determination letters) as well as signed consent documents. This documentation should be maintained for 3 years after the study has been closed.

Response:
Site 1: Diabetes Endocrinology Research Center of WNY
Address: 1000 Youngs Road, Suite 105, Williamsville NY 14221
Department: Diabetes Endocrinology

Site 2: University of Glasgow. Institute of Cardiovascular and Medical Sciences University of Glasgow 126 University Place Glasgow, G12 8TA

1.0 Objectives

1.1 Describe the purpose, specific aims, or objectives of this research.

Response:

Primary Objective: To assess whether the addition of dapagliflozin to semaglutide and insulin (triple therapy) improves glycemic control in patients with type 1 diabetes compared with semaglutide and insulin (dual therapy) and insulin only (standard) treatment.

General hypothesis: The addition of dapagliflozin to semaglutide and insulin treatment improves glycemic control, weight loss and blood pressure in adults with type 1 diabetes.

1.2 State the hypotheses to be tested, if applicable.

NOTE: A hypothesis is a specific, testable prediction about what you expect to happen in your study that corresponds with your above listed objectives.

Response:

Specific hypotheses:

Primary: combined dapagliflozin and semaglutide treatment in addition to insulin will improve HbA1c levels compared with semaglutide in addition to insulin .

Secondary 1: semaglutide in addition to insulin treatment over 26 weeks will improve HbA1c levels compared with insulin therapy alone.

Secondary 2: combined dapagliflozin and semaglutide treatment in addition to insulin will improve HbA1c levels compared to insulin therapy alone at 52 weeks.

Secondary 3: combined dapagliflozin and semaglutide treatment in addition to insulin will improve CGM-based weekly mean and fasting blood glucose, glycemic oscillations and percent of time in range (70-180mg/dl) with lower insulin requirements compared with semaglutide and insulin (dual therapy) or insulin only (standard) treatment.

Secondary : combined dapagliflozin and semaglutide in addition to insulin therapy will reduce systolic and diastolic blood pressure compared with semaglutide and insulin (dual therapy) or insulin only (standard) treatment.

Secondary 5: combined dapagliflozin and semaglutide in addition to insulin therapy (triple therapy) will result in weight loss compared to dual and standard therapy.

Secondary 6: combined dapagliflozin and semaglutide in addition to insulin therapy will result in improved scores on the Diabetes Specific Quality of Life Scale (DSQOLS) and the Problem Areas In Diabetes (PAID) survey compared with dual and standard therapy.

Tertiary and Exploratory Objectives (EO) for future ancillary studies (PLEASE NOTE: For EO, samples will be collected to facilitate the measurements of these end points in future ancillary studies once submitted and approved by JDRF so cost of assays is not included in the current budget) : The addition of dapagliflozin to semaglutide and insulin in type 1 diabetes will:

Tertiary 1: combined dapagliflozin and semaglutide treatment in addition to insulin therapy will improve fasting and postprandial glucose excursions and suppress postprandial glucagon, FFA, GLP-1 compared with semaglutide and insulin or insulin therapy alone.

Tertiary 2: semaglutide in addition to insulin treatment over 52 weeks will improve HbA1c levels compared with insulin therapy alone.

EO 1: increase plasma concentrations of vasodilatation mediators and reduce plasma concentrations of vasoconstriction mediators compared to dual and standard therapy groups.

EO 2: exert a systemic anti-inflammatory effect including reducing mRNA expression of inflammatory mediators and NFκB binding in peripheral mononuclear cells (MNC) and adipose tissue (optional) and their circulating levels in plasma while increasing levels of IL-RA and adiponectin.

2.0 Scientific Endpoints

2.1 Describe the scientific endpoint(s), the main result or occurrence under study.

NOTE: Scientific endpoints are outcomes defined before the study begins to determine whether the objectives of the study have been met and to draw conclusions from the data. Include primary and secondary endpoints. Some example endpoints are: reduction of

*symptoms, improvement in quality of life, or survival. Your response should **not** be a date.*

Response:

Primary endpoint: Change in HbA1c at 6 months following dapagliflozin or placebo therapy in addition to combined semaglutide and insulin treatment.

Efficacy Secondary endpoints:

1. Change in HbA1c from baseline at 12 months in triple therapy group compared to insulin only group.
2. Change in HbA1c from baseline at 6 months in dual therapy group compared to insulin only group.
3. Change in percent Time in Range (70– 180 mg/dl), percent time in hyperglycemia Level 1(180-250mg/dl) and Level 2 (>250mg/dl) and percent time in hypoglycemia Level 1 (54-70mg/dl) as assessed by CGM
4. Other indices of glycemic control: fructosamine, weekly mean and fasting glucose, standard deviation, insulin requirements.
5. Change in body weight and quality of life as assessed by DSQOLS and PAID at 6 and 12 months.
6. Anti-hypertensive effects including change in systolic and diastolic BP, numbers of BP medications required.

Safety Secondary endpoints:

1- Differences in rates of hypoglycemic events Level 2 (<54mg/dl) and Level 3 (A severe event characterized by altered mental and/or physical status requiring assistance) between investigative arms and standard therapy arm.

2- *Differences in rates of diabetic ketoacidosis defined as elevated serum or urine ketones (greater than the upper limit of the normal range), and serum bicarbonate <15 mmol/L or Blood pH <7.3 between investigative arms and standard therapy arm.*

Tertiary/ exploratory endpoints: (items 2-4 to be performed in future on collected samples from this study if additional funding is approved by JDRF):

1- Fasting and postprandial (Area Under curve) glucose excursions, FFA, GLP-1 and glucagon and fasting fructosamine.

2- Indices of inflammatory stress. These will be CRP, FFA, SAA, IL-1β, IL-18, IL-1RA, adiponectin and TNFα concentrations in plasma and isoprostane in urine.

3- Expression of IL-1β, TNFα, IL-12, TLR2, TLR4, adiponectin, IκBα as well as NFκB binding in peripheral MNC and adipose tissue (optional).

4- Indices of vasodilatation and vasoconstriction: ANP, b-NP, cGMP, cAMP, angiotensinogen, renin and angiotensin II concentrations.

3.0 Background

3.1 Provide the scientific or scholarly background, rationale, and significance of the research based on the existing literature and how it will contribute to existing knowledge. Describe any gaps in current knowledge. Include relevant preliminary findings or prior research by the investigator.

Response:

The control of glucose homeostasis in type 1 diabetes is fragile since β-cell reserve is negligible. Traditionally, insulin has been the only treatment approach available. This approach, often leads to erratic blood sugar control given the challenge of matching exogenous insulin to mealtime glucose excursions in the face of a lack of any endogenous insulin to compensate for variation in requirements in response to meals and activity levels. Injected insulin boluses rarely match precise insulin requirements either in terms of dose administered or bio-availability. The result often is wide fluctuations in blood glucose readings. Based on our work to date and other published reports, previous attempts to add other glucose-lowering medications to insulin therapy yielded minimal benefits on glycemia (although metformin may have other benefits (1-3)

We have recently shown in two preliminary retrospective studies that the addition of liraglutide, a Glucagon Like Peptide -1 receptor agonist (GLP-1RA), to insulin results in consistent improvements in glycemic control in type 1 diabetes as demonstrated by reductions in HbA1c, glycemic excursions, and insulin requirements accompanied by improvements in body weight and systolic blood pressure (4, 5). We have now also completed the first prospective randomized controlled trial of this drug in 72 people with type 1 diabetes. Our data not only confirmed improvements in glycemic control, HbA1c, body weight and systolic BP, but also demonstrated a reduction in appetite, carbohydrate intake in low grade inflammation as measured by plasma CRP concentrations (6). Importantly, quality of life was also improved. Our recent studies in type 2 diabetes have revealed that systolic blood pressure reduction with GLP-1RA (exenatide) treatment in type 2 diabetes is associated with increased plasma concentrations of ANP, cGMP and cAMP along with reduced concentrations of angiotensinogen, renin and angiotensin II (7). It is possible that these mechanisms play a role in the prevention of cardiovascular complications in type 1 diabetes.

While GLP-1RAs, therefore, have great potential as adjunct therapy for type 1 diabetes, mean HbA1c was still ≥7% after liraglutide treatment (6) and larger pharma-sponsored trials reported increased rates of hypoglycemia (8, 9). In seeking further improvement, the advent of sodium-glucose transport protein-2 (SGLT2) inhibitors is of importance since their mode of action is independent of insulinogenesis. Introduced for use in type 2 diabetes, SGLT2 inhibitors are selective antagonists of the sodium-glucose transporter subtype expressed in the proximal convoluted tubules of the kidneys. They act by inhibiting renal glucose re-absorption and therefore stimulating excretion of 60-100 grams per day of glucose in urine. SGLT2 inhibitors have been studied extensively in type 2 diabetes, including the landmark cardiovascular outcome trial EMPA-REG (10), but data have only quite recently begun to accumulate in type 1(11). Short term effects on urine glucose excretion of remogliflozin and empagliflozin are similar to those seen in type 2 diabetes and these translate into improved glycemic and other indices including HbA1c; fasting blood glucose, rates of hypoglycemia, daily insulin dose and weight (12). Weight loss accompanying increased urinary glucose excretion could translate into reduced cardiovascular complications as in type 2 diabetes, but there are no data so far available to support this hypothesis. More recently, in the Phase 3 trial DEPICT-1 we showed that dapagliflozin improves glycemic control and HbA1c over six months by about 0.45% with no severe hypoglycemia or diabetic ketoacidosis (13). Similarly, sotagliflozin has been shown in a Phase 3 trial to allow a larger proportion of patients to achieve HbA1c lower than 7.0% with no severe hypoglycemia although the rate of diabetic ketoacidosis was

higher in the sotagliflozin group (14). Both products are now being reviewed by regulatory agencies for the type 1 diabetes indication.

On the basis of the above, we hypothesize that adding an SGLT2 inhibitor to a combination of insulin and semaglutide (a weekly and potent GLP-1RA) will improve glycemic control in type 1 diabetes such that HbA1c levels of ≤7% are achieved more consistently. Our protocol is designed to allow an objective assessment of whether this triple therapy combination will work effectively and safely so that a majority of people with type 1 diabetes can reach a glycemic target HbA1c of ≤7%. In addition, since both semaglutide and dapagliflozin inhibitors reduce blood pressure and weight, it will allow an assessment of the combined effect of GLP-1RAs and SGLT2 inhibitors on these outcomes. Since there are no previous data on the effects of once weekly semaglutide or the combination of semaglutide and dapagliflozin compared to standard insulin treatment in type 1 diabetes, the design requires allocation of some participants to insulin therapy only.

Due to the potential ketogenic effect of SGLT-2 inhibitors, data from SGLT-2 inhibitors studies in type 1 diabetes were analyzed for it ketogenic effects and a consensus statement and guidelines on management of patients was recently published (Danne T et al., Diabetes Care. 2019 Feb 6).

Preliminary data

1) Liraglutide treatment in patients with type 1 diabetes (4): We treated 8 type 1 diabetes patients; mean age (38.5 years) and duration of diabetes (20±4 yrs), with liraglutide in addition to insulin for 24 weeks. As shown in table 1, there was a significant reduction in fasting and mean blood glucose concentrations, insulin doses, HbA1c and a reduction in appetite and caloric intake with weight loss. In addition, there was a reduction in the excursions of blood glucose concentrations, i.e., the standard deviations of blood glucose concentrations were markedly reduced (**figure 1**). The changes were observed within 48h and improved marginally thereafter with fine tuning of insulin doses and a further reduction in these doses. These observations are noteworthy in that study participants were well controlled and meticulous users of CSII and CGM. They had all tried hard to improve their diabetic control by adjusting insulin doses but had not succeeded because of hypoglycemia. We then demonstrated that poorly controlled and obese individuals with type 1 diabetes also experienced similar benefits of liraglutide treatment (2).

2) Liraglutide As Additional Treatment to Insulin in Patients with Type 1 Diabetes Mellitus: A Randomized Clinical Trial (6): Following the two above non-randomized studies we recently conducted a randomized clinical trial in which 72 individuals (Placebo=18; liraglutide = 54) with TYPE 1 DIABETES for at least one year, on insulin therapy and with no detectable C-peptide in plasma (mean BMI: 30±1; mean body weight: 84±2 kg ; mean HbA1c: 7.57±0.09%; mean age: 44±2 years; mean age of TYPE 1 DIABETES diagnosis: 20±1 years) were randomized to receive placebo, 0.6, 1.2 or 1.8mg of liraglutide daily for 12 weeks. In the 1.2 mg and 1.8 mg groups, mean change in average blood glucose was -10±2 and -10.0±1mg/dl, respectively, ($p<0.0001$ vs. placebo). In the 1.2mg group, HbA1c fell by 0.78% from 7.84±0.17% to 7.06±0.15% ($p<0.0001$, $p<0.01$ vs. placebo) and in the 1.8mg group fell by 0.42% from 7.41±0.15 to 6.99±0.15 ($p=0.001$, $p=0.39$ vs placebo). Time spent between 70 to 160 mg/dl increased by 5±1% ($p<0.05$ vs

placebo) and time between 160-400 mg/dl decreased by 7±1% ($p<0.01$ vs placebo) in the 1.8 mg group with no additional hypoglycemia. During the 12 week period, the average total daily dose of insulin in the two groups fell by 12.4±3.9 and 10.0±2.3 units respectively ($p<0.05$ vs baseline and placebo). There was a reduction in body weight (from 83±4 to 78±5 kg, $p<0.0001$, in the 1.8mg group, from 96±4 to 91±4 kg, $p<0.001$, in the 1.2 mg group and from 80±4 to 77±4 kg, $p<0.01$ in the 0.6 mg group). A reduction in daily carbohydrate intake (171±17g vs. 127±18g; $p<0.01$ and 153±18g vs 115±16g, $p<0.01$ in the 1.2 mg and 1.8 mg groups, respectively) was also observed over 12 weeks. Systolic BP (SBP) in the 1.8 mg group fell by 3 mmHg (120±2 to 117±3 mmHg, $p=0.01$). CRP fell by 15±6 and 19±8% in the 1.2 and 1.8mg groups, respectively ($p<0.05$). Quality of life improved significantly compared to baseline and placebo (unpublished data).

3) Dapagliflozin as an Additional Treatment to Insulin and Liraglutide in Patients with Type 1 Diabetes Mellitus (15): The objective of this randomized clinical trial was to investigate whether addition of dapagliflozin to insulin and liraglutide results in a significant reduction in glycemia and body weight. 30 participants with TYPE 1 DIABETES on liraglutide therapy for at least last 6 months were randomized (in 2:1 ratio) to receive either dapagliflozin 10 mg or placebo daily for 12 weeks. With 12 weeks of dapagliflozin HbA1c fell by 0.66±0.08% from 7.8±0.21% ($p < .01$ vs placebo) but did not change significantly with placebo (7.40±0.20% to 7.30±0.20%). In one patient within one week of addition of dapagliflozin average glucose fell from 170 to 122 mg/dl while standard deviation was reduced from 71 to 58 (**Figure 2-diminished glycemic excursions)**. Body weight fell by1.9 +/- 0.54kg ($p < 0.05$ vs placebo). There was no additional hypoglycemia (blood glucose < 3.88 mmol/L; $p = 0.52$ vs placebo). In the dapagliflozin group, there were significant increases in the plasma concentrations of glucagon by 35% +/- 13% ($p < 0.05$), hormone-sensitive lipase by 29% +/- 11% ($p < 0.05$), free fatty acids by 74% +/- 32% ($p < 0.05$), acetoacetate by 67% +/- 34% ($p < 0.05$), and beta-hydroxybutyrate by 254% +/- 81% ($p < 0.05$). Urinary ketone levels also increased significantly ($P < .05$). None of these changes was observed in the placebo group. Two individuals in the dapagliflozin group developed ketoacidosis. Overall, addition of dapagliflozin to insulin and liraglutide in patients with TYPE 1 DIABETES resulted in a significant improvement in glycemia and in weight loss but tended to increase ketosis.

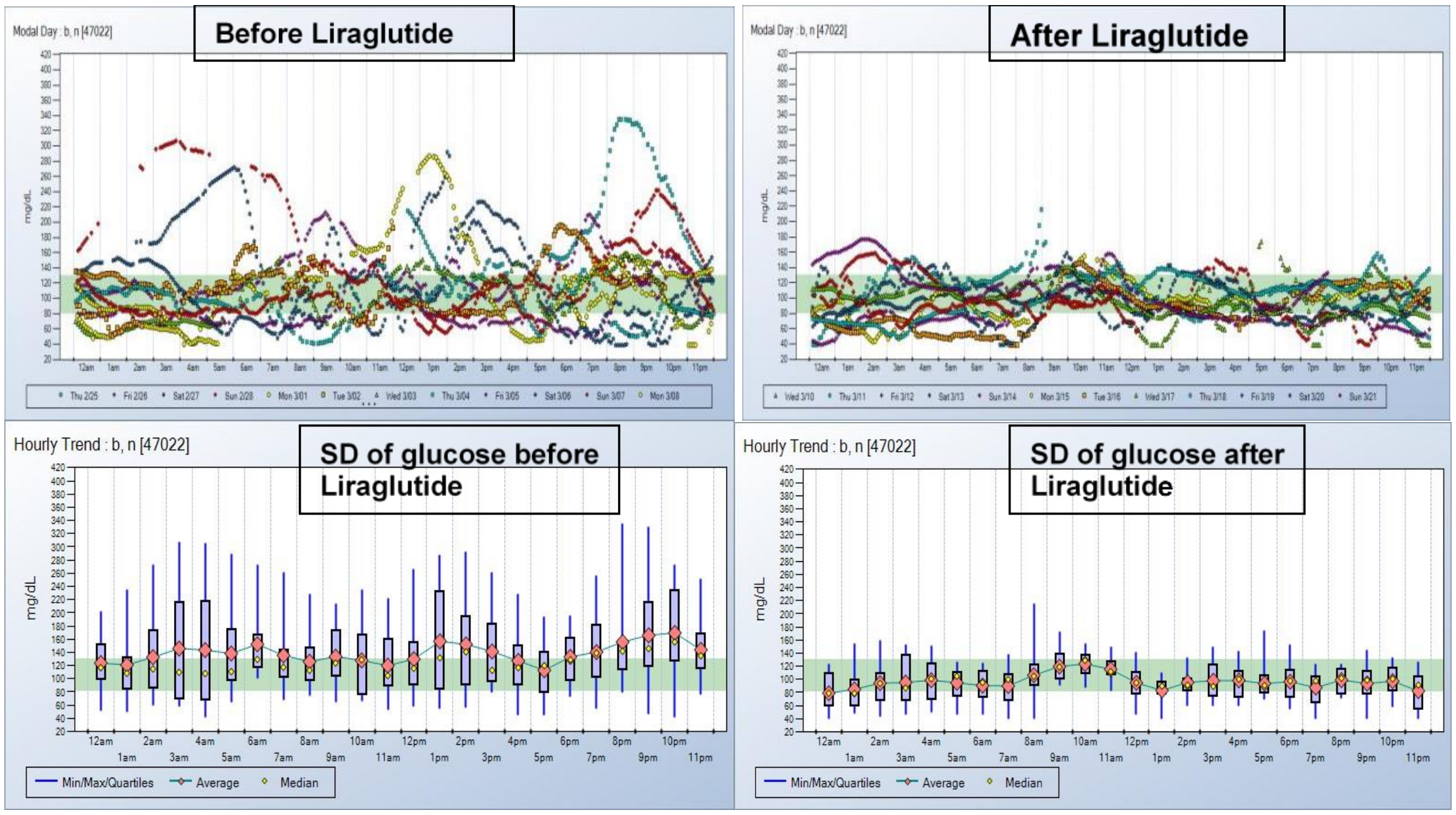

Figure 1: Weekly continuous glucose monitoring record of one patient prior to and following 1 week of liraglutide. Note the glycemic variability (SD) and the frequency of glucose concentrations within the target range prior to and following liraglutide

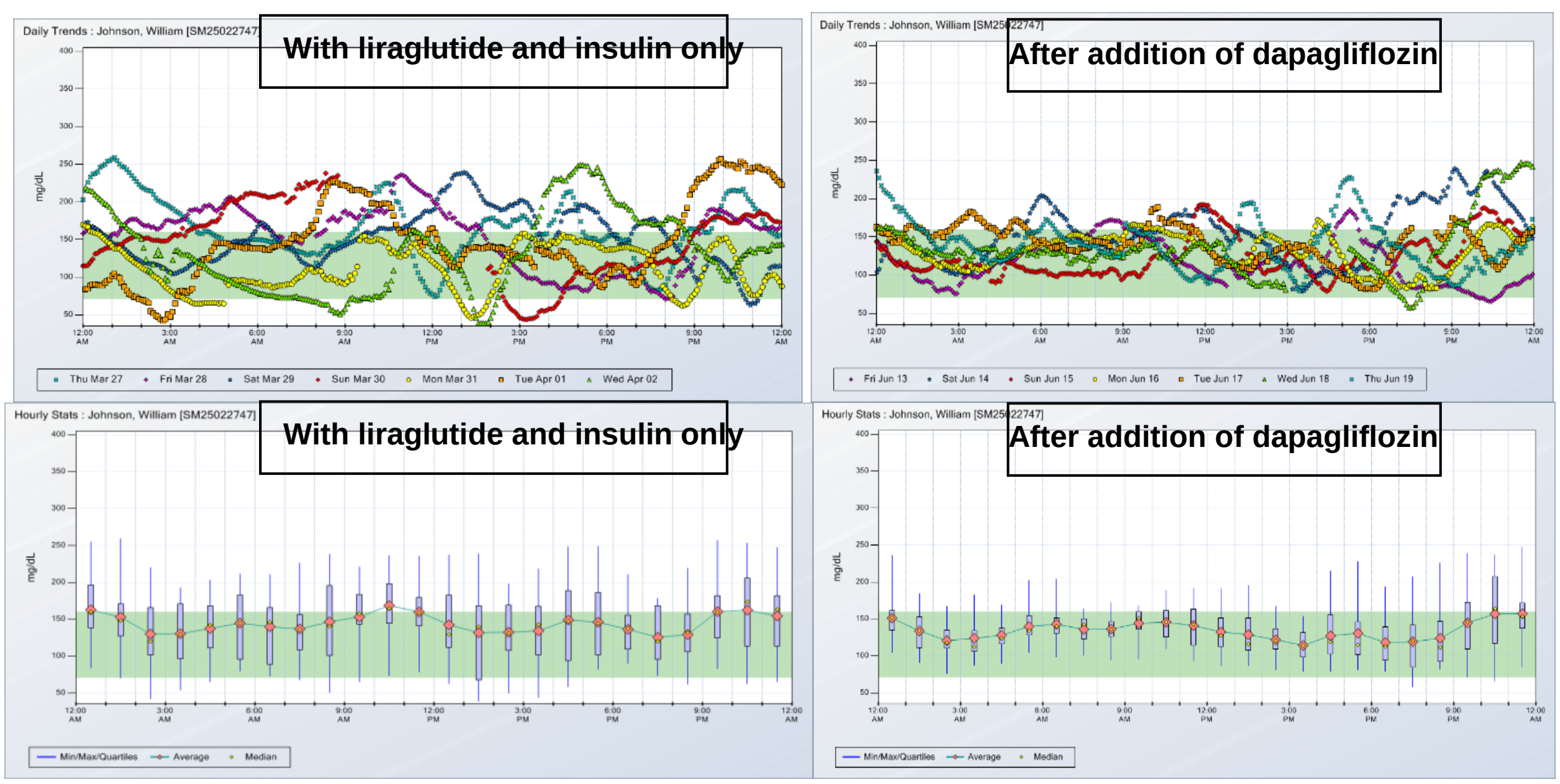

Figure 2: Weekly continuous glucose monitoring record of one patient prior to and following 8 weeks of the triple therapy combination with dapagliflozin and liraglutide. Note the glycemic variability (SD) (bottom) and the frequency of glucose concentrations within the target range prior to and following dapagliflozin addition.

3.2 Include complete citations or references.

Response:

1. Konrad K, Datz N, Engelsberger I, Grulich-Henn J, Hoertenhuber T, Knauth B, et al. Current use of metformin in addition to insulin in pediatric patients with type 1 diabetes mellitus: an analysis based on a large diabetes registry in Germany and Austria. *Pediatr Diabetes.* 2014.
2. Vella S, Buetow L, Royle P, Livingstone S, Colhoun HM, and Petrie JR. The use of metformin in type 1 diabetes: a systematic review of efficacy. *Diabetologia.* 2010;53(5):809-20.
3. Vella S, Buetow L, Royle P, Livingstone S, and Petrie JR. Metformin in type 1 diabetes reduces insulin requirements without significantly improving glycaemic control. Reply to Schatz H [letter]. *Diabetologia.* 2011;54(1):203-4.
4. Varanasi A, Bellini N, Rawal D, Vora M, Makdissi A, Dhindsa S, et al. Liraglutide as additional treatment for type 1 diabetes. *Eur J Endocrinol.* 2011;165(1):77-84.
5. Kuhadiya ND, Malik R, Bellini NJ, Patterson JL, Traina A, Makdissi A, et al. Liraglutide as additional treatment to insulin in obese patients with type 1 diabetes mellitus. *Endocrine practice : official journal of the American College of Endocrinology and the American Association of Clinical Endocrinologists.* 2013;19(6):963-7.
6. Kuhadiya ND, Dhindsa S, Ghanim H, Mehta A, Makdissi A, Batra M, et al. Addition of Liraglutide to Insulin in Patients With Type 1 Diabetes: A Randomized Placebo-Controlled Clinical Trial of 12 Weeks. *Diabetes Care.* 2016;39(6):1027-35.
7. Chaudhuri A, Ghanim H, Makdissi A, Green K, Abuaysheh S, Batra M, et al. Exenatide induces an increase in vasodilatory and a decrease in vasoconstrictive mediators. *Diabetes Obes Metab.* 2017;19(5):729-33.
8. Mathieu C, Zinman B, Hemmingsson JU, Woo V, Colman P, Christiansen E, et al. Efficacy and Safety of Liraglutide Added to Insulin Treatment in Type 1 Diabetes: The ADJUNCT ONE Treat-To-Target Randomized Trial. *Diabetes Care.* 2016;39(10):1702-10.
9. Ahren B, Hirsch IB, Pieber TR, Mathieu C, Gomez-Peralta F, Hansen TK, et al. Efficacy and Safety of Liraglutide Added to Capped Insulin Treatment in Subjects With Type 1 Diabetes: The ADJUNCT TWO Randomized Trial. *Diabetes Care.* 2016;39(10):1693-701.
10. Zinman B, Wanner C, Lachin JM, Fitchett D, Bluhmki E, Hantel S, et al. Empagliflozin, Cardiovascular Outcomes, and Mortality in Type 2 Diabetes. *N Engl J Med.* 2015;373(22):2117-28.
11. Petrie JR. SGLT2 inhibitors in type 1 diabetes: knocked down, but up again? *Lancet Diabetes Endocrinol.* 2017;5(11):841-3.
12. Mudaliar S, Armstrong DA, Mavian AA, O'Connor-Semmes R, Mydlow PK, Ye J, et al. Remogliflozin etabonate, a selective inhibitor of the sodium-glucose transporter 2, improves serum glucose profiles in type 1 diabetes. *Diabetes Care.* 2012;35(11):2198-200.
13. Dandona P, Mathieu C, Phillip M, Hansen L, Griffen SC, Tschope D, et al. Efficacy and safety of dapagliflozin in patients with inadequately controlled type 1 diabetes (DEPICT-1): 24 week results from a multicentre, double-blind, phase 3, randomised controlled trial. *Lancet Diabetes Endocrinol.* 2017;5(11):864-76.

14. Garg SK, Henry RR, Banks P, Buse JB, Davies MJ, Fulcher GR, et al. Effects of Sotagliflozin Added to Insulin in Patients with Type 1 Diabetes. *N Engl J Med.* 2017;377(24):2337-48.
15. Kuhadiya ND, Ghanim H, Mehta A, Garg M, Khan S, Hejna J, et al. Dapagliflozin as Additional Treatment to Liraglutide and Insulin in Patients With Type 1 Diabetes. *J Clin Endocrinol Metab.* 2016;101(9):3506-15.
16. Henry RR, Thakkar P, Tong C, Polidori D, and Alba M. Efficacy and Safety of Canagliflozin, a Sodium-Glucose Cotransporter 2 Inhibitor, as Add-on to Insulin in Patients With Type 1 Diabetes. *Diabetes Care.* 2015;38(12):2258-65.
17. Peters AL, Buschur EO, Buse JB, Cohan P, Diner JC, and Hirsch IB. Euglycemic Diabetic Ketoacidosis: A Potential Complication of Treatment With Sodium-Glucose Cotransporter 2 Inhibition. *Diabetes Care.* 2015;38(9):1687-93.
18. Ahmann AJ, Capehorn M, Charpentier G, Dotta F, Henkel E, Lingvay I, et al. Efficacy and Safety of Once-Weekly Semaglutide Versus Exenatide ER in Subjects With Type 2 Diabetes (SUSTAIN 3): A 56-Week, Open-Label, Randomized Clinical Trial. *Diabetes Care.* 2017.
19. Andrews NC, and Faller DV. A rapid micropreparation technique for extraction of DNA-binding proteins from limiting numbers of mammalian cells. *Nucleic Acids Res.* 1991;19(9):2499.
20. Perkins BA, Cherney DZ, Partridge H, Soleymanlou N, Tschirhart H, Zinman B, et al. Sodium-glucose cotransporter 2 inhibition and glycemic control in type 1 diabetes: results of an 8-week open-label proofof-concept trial. *Diabetes Care.* 2014;37(5):1480-3.
21. Cherney DZ, Perkins BA, Soleymanlou N, Maione M, Lai V, Lee A, et al. Renal hemodynamic effect of sodium-glucose cotransporter 2 inhibition in patients with type 1 diabetes mellitus. *Circulation.* 2014;129(5):587-97.

4.0 Study Design

4.1 Describe and explain the study design (e.g. case-control, cross-sectional, ethnographic, experimental, interventional, longitudinal, observational).

Response: This will be a two-center, randomized, open-label then double-blind, parallel group, placebo and standard of care controlled prospective study in type 1 diabetes. One hundred and (114) patients will be randomized (open label, see below) into three groups (38 each) and will be either maintained on their regular insulin therapy for 12 months (standard therapy group) or started on semaglutide in addition to regular insulin treatment for six month (dual therapy and triple therapy groups). For those on insulin and semaglutide, after six months of stable treatment, dapagliflozin (triple therapy group) or placebo (dual therapy group) treatment will be initiated for an additional six months according to a second randomization (double-blind).

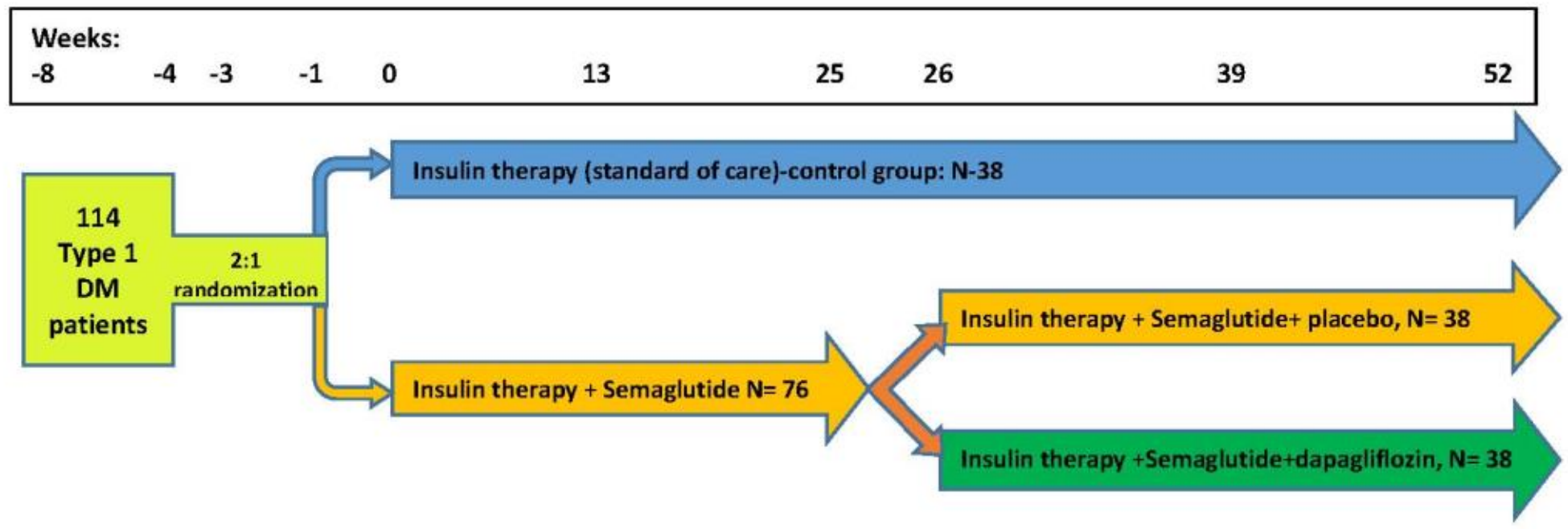

5.0 Local Number of Subjects

5.1 Indicate the total number of subjects that will be enrolled or records that will be reviewed locally.

Response: 57 patients will be enrolled locally with another 57 patients recruited at the University of Glasgow site: a total of 114.

5.2 If applicable, indicate how many subjects you expect to screen to reach your target sample (i.e. your screen failure rate).

Response: All screened and qualified patients will be enrolled and randomized

5.3 Justify the feasibility of recruiting the proposed number of eligible subjects within the anticipated recruitment period. For example, how many potential subjects do you have access to? What percentage of those potential subjects do you need to recruit?

Response: The Diabetes and Endocrinology Center of WNY is the largest Diabetes center in the WNY area, seeing between 60 to 100 type 1 diabetic patient every month. Therefore, the majority of recruited patients are our clinic patients. We also recruit a study participants through advertisement and other physician's references. These sources will suffice to recruit the needed number to subjects. The Glasgow center (Stobhill Ambulatory Care Hospital) reviews more than 300 patients per month.

6.0 Inclusion and Exclusion Criteria

6.1 Describe the criteria that define who will be ***included*** *in your final study sample.*

NOTE: This may be done in bullet point fashion.

Response:
Inclusion Criteria

1) Type 1 Diabetes for at least 1 year on staple use of continuous subcutaneous insulin infusion (CSII) or multiple daily (four or more) injections (MDI) of insulin for last 3 months.
2) C-peptide <0.23 nM
3) Minimum dose of insulin in Units/kg at entry: 0.5 U/kg for MDI and 0.4 U/kg for CSII
4) Regularly measuring blood sugars four or more times daily or use of CGM.
5) HbA1c between7.5-11% inclusive.
6) Well versed in CHO counting*
7) Age 18-70 years.
8) BMI ≥25 kg/m^2.

*In the opinion of the site Principal Investigator

6.2 Describe the criteria that define who will be ***excluded*** *from your final study sample.*

NOTE: This may be done in bullet point fashion.

Response:

Exclusion Criteria

1) Type 1 diabetes for less than 12 months, type 2 diabetes, chronic pancreatitis, MODY
2) Previous use of any agent other than insulin for treatment of diabetes in the last 3 months.
3) History of diabetic ketoacidosis (DKA) requiring medical intervention (e.g., emergency room visit and/or hospitalization) within 3 months prior to the screening visit
4) Patients on ketogenic or low (<50g/day) carbohydrate diet or who practice intermittent fasting.
5) Frequent episodes of severe hypoglycemia as defined by more than one episode requiring medical assistance, emergency care (paramedics or emergency room care), and/or glucagon therapy administered by a third-party individual within 1 month prior to the screening visit
6) Symptoms of poorly controlled diabetes that would preclude participation in this trial
7) Subjects on a commercial weight loss program with ongoing weight loss, or on an intensive exercise program
8) History of bariatric surgery or lap-band procedure within 12 months prior to screening
9) History of Addison's disease or chronic adrenal insufficiency
10) History of diabetes insipidus
11) Hepatic disease or cirrhosis
12) Aspartate Aminotransferase (AST) > 3X Upper limit of normal (ULN) *and/or* Alanine aminotransferase (ALT) > 3X ULN
13) Serum Total Bilirubin > 2X ULN unless exclusively caused by Gilbert's Syndrome

14) Hemoglobin < 11.0 g/dL (110 g/L) for men; hemoglobin < 10.0 g/dL (100 g/L) for women.
15) Coronary event or procedure (myocardial infarction, unstable angina, coronary artery bypass, surgery or coronary angioplasty) in the previous 3 months or patients with congestive heart failure.
16) ESRD on hemodialysis; and or e-GFR < 60 ml/min/1.73m^2
17) HIV or Hepatitis B/C positive status
18) Any other life-threatening, noncardiac disease
19) History of pancreatitis
20) Women who are pregnant or women of childbearing potential who are not using adequate contraception or who are breast feeding
21) Inability to give informed consent
22) History of gastroparesis
23) History of medullary thyroid carcinoma or MEN 2 syndrome
24) History of serious hypersensitivity reaction to these agents
25) Painful gallstones
26) Alcoholism
27) Hypertriglyceridemia (>500 mg/dl)
28) Recurrent genital mycotic infection.
29) Hypovolemic patients or with chronic renal insufficiency.
30) Patients with any malignancy except treated in situ malignancy and basal cell carcinoma of the skin
31) Unexplained hematuria
32) Patients with a history of diabetic retinopathy
33) Use of an investigational agent or therapeutic regimen within 30 days of study
34) Participation in any other concurrent interventional clinical trial

6.3 Indicate specifically whether you will include any of the following special populations in your study using the checkboxes below.

NOTE: Members of special populations may not be targeted for enrollment in your study unless you indicate this in your inclusion criteria.

Response: NA

☐ Adults unable to consent

☐ Individuals who are not yet adults (infants, children, teenagers)

☐ Pregnant women

☐ Prisoners

6.4 Indicate whether you will include non-English speaking individuals in your study. ***Provide justification if you will exclude non-English speaking individuals.***

In order to meet one of the primary ethical principles of equitable selection of subjects, non-English speaking individuals may ***not*** *be routinely excluded from research as a matter of convenience.*

In cases where the research is of therapeutic intent or is designed to investigate areas that would necessarily require certain populations who may not speak English, the researcher is required to make efforts to recruit and include non-English speaking individuals. However, there are studies in which it would be reasonable to limit subjects to those who speak English. Some examples include pilot studies, small unfunded studies with validated instruments not available in other languages, studies with numerous questionnaires, and some non-therapeutic studies which offer no direct benefit.

Response:

We have no non-English speaking patients in this population. We have patients that English is a second language, but they are able to read, write and understand it. This population is less than 10% of the total population. No benefit is being withheld from not participating in the study.

7.0 Vulnerable Populations

If the research involves special populations that are considered vulnerable, ***describe the safeguards included to protect their rights and welfare.***

NOTE: You should refer to the appropriate checklists, referenced below, to ensure you have provided adequate detail regarding safeguards and protections. You do not, however, need to provide these checklists to the IRB.

7.1 For research that involves ***pregnant women,*** *safeguards include:*
NOTE CHECKLIST: Pregnant Women (HRP-412)

Response: We will not be recruiting subjects from vulnerable populations

☒ **N/A**: This research does not involve pregnant women.

7.2 For research that involves ***neonates of uncertain viability or non-viable neonates,*** *safeguards include:*
NOTE CHECKLISTS: Non-Viable Neonates (HRP-413), or Neonates of Uncertain Viability (HRP-414)

Response:

☒ **N/A:** This research does not involve non-viable neonates or neonates of uncertain viability.

7.3 For research that involves ***prisoners****, safeguards include:*
NOTE CHECKLIST: Prisoners (HRP-415)

Response:

☒ **N/A:** This research does not involve prisoners.

*7.4 For research that involves **persons who have not attained the legal age for consent to treatments or procedures involved in the research ("children")**, safeguards include:*
NOTE CHECKLIST: Children (HRP-416)

Response:

☒ **N/A:** This research does not involve persons who have not attained the legal age for consent to treatments or procedures ("children").

*7.5 For research that involves **cognitively impaired adults**, safeguards include:*
NOTE CHECKLIST: Cognitively Impaired Adults (HRP-417)

Response:

☒ **N/A:** This research does not involve cognitively impaired adults.

*7.6 Consider if other specifically targeted populations such as students, employees of a specific firm, or educationally or economically disadvantaged persons are vulnerable. **Provide information regarding their safeguards and protections, including safeguards to eliminate coercion or undue influence.***

Response: No specific populations or vulnerable groups will be targeted. All subjects enrolled in this study will be of legal adult consenting age with the ability to speak, read and interpret the English language. Patients will have the ability to speak with the research team regarding any questions or concern they have before signing the consent. Research team have the ability to evaluate patient's cognitive abilities through these interactions with patients. Patients are made aware that this study is voluntary and they are able to stop participating at any time they feel uncomfortable. Patients are not be pressured into participating and their clinic standard of care will remain the same if they participate or choose not to participate.

8.0 Eligibility Screening

*8.1 Describe **screening procedures** for determining subjects' eligibility. Screening refers to determining if prospective participants meet inclusion and exclusion criteria.*

Include all relevant screening documents with your submission (e.g. screening protocol, script, questionnaire).

Response:

After obtaining partial HIPAA waiver, patient charts will be screened according to the study inclusion and exclusion criteria by our trained clinical staff and physicians. In additions, the study clinical team will evaluate their clinic patients for possible participation in this study according to the inclusion and exclusion criteria at the Diabetes and Endocrinology Center of WNY clinics. Physicians will

speak to them about their interests in participating in research. If the patient agrees, their information will be given to the research coordinator to be contacted for further evaluation. The research coordinator will contact interested patients and those meeting the inclusion and exclusion criteria based on preliminary phone evaluation will be invited to participate in the study. All personal information will be kept confidential and locked in the coordinator office.

Prospective participants will be asked to read the consent and any questions they may have regarding the protocol will be answered. If the subject wants to participate in the study, they will be asked to sign the informed consent form. The subject's medical history and current medications will be obtained as well as their blood pressure and vitals. A physical examination will also be done. Blood and urine samples will be taken in order to evaluate HbA1c, CBC, CMP and liver and kidney functions and pregnancy status. Females of childbearing age, will have to be on two forms of birth control to prevent pregnancy. Patients meeting all the inclusion and exclusion criteria based on all screening tests will be enrolled in the study.

☐ **N/A:** There is no screening as part of this protocol.

9.0 Recruitment Methods

☐ **N/A:** This is a records review only, and subjects will not be recruited. NOTE: If you select this option, please make sure that all records review procedures and inclusion/exclusion screening are adequately described in other sections.

9.1 Describe when, where, and how potential subjects will be recruited.

NOTE: Recruitment refers to how you are identifying potential participants and introducing them to the study. Include specific methods you will use (e.g. searching charts for specific ICD code numbers, Research Participant Groups, posted advertisements, etc.).

Response:

Participants will be identified by prescreening clinical charts, patient doctor interaction at the time of their visits to the Diabetes Endocrinology Center of WNY Locations include:

1. 1020 Youngs Road, Williamsville NY 14221
2. 705 Maple Road, Williamsville NY 14221
3. 462 Grider Street, Buffalo NY 14215
4. 1000 Youngs Road, Suite 105, Williamsville NY 14221

Patients can be also referred by other physicians and providers

The remainder of the participants (57 patients) will be recruited from NHS Greater Glasgow and Clyde Diabetes clinics (Scotland, UK). Separate local IRB/ Ethical approval will be sought to cover this site (based on discussions with UBIRB director).

The study clinical team will evaluate their clinic patients for possible participation in this study according to the inclusion and exclusion criteria at the Diabetes and Endocrinology Center of WNY clinics. Patients that may qualify for the study are referred to the research team for further eligibility evolution. Patients meeting the inclusion and exclusion criteria based on preliminary phone evaluation will be invited to participate in the study.

Participants will also be identified by flyers, advertisements on Facebook and Craigslist and researchmatch.org. The study will also be put on UB's Study Information Portal (SIP) website.

9.2 Describe how you will protect the privacy interests of prospective subjects during the recruitment process.

NOTE: Privacy refers to an individual's right to control access to him or herself.

Response: Patient charts will be screened according to the study inclusion and exclusion criteria by our trained clinical staff and physicians. If the patient qualifies and is of consenting age, the physicians will speak to them about their interests in participating in research in a private room where no one else can hear the conversation. If the patient agrees, their information will be given to the research coordinator to be contacted for further evaluation. No personal information will be shared without patient's approval and will not be used for any purpose except for this research. All personal information will be kept confidential and locked in the coordinator office.

9.3 Identify any materials that will be used to recruit subjects.

NOTE: Examples include scripts for telephone calls, in person announcements / presentations, email invitations.

For advertisements, include the final copy of printed advertisements with your submission. When advertisements are taped for broadcast, attach the final audio/video tape. NOTE: You may submit the wording of the advertisement prior to taping to ensure there will be no IRB-required revisions, provided the IRB also reviews and approves the final version.

Response: In addition to screening clinical charts, participations will be identified through; flyer advertisement, researchmatch.org, craigslist.com, and Facebook. The study will also be put on UB's Study Information Portal (SIP) website.

10.0 Procedures Involved

*10.1 Provide a description of **all research procedures or activities** being performed and when they are performed once a subject is screened and determined to be eligible. Provide as much detail as possible.*

NOTE: This should serve as a blueprint for your study and include enough detail so that another investigator could pick up your protocol and replicate the research.

For studies that have multiple or complex visits or procedures, consider the addition of a schedule of events table in in your response.

Response:

Each patient will spend about 55 weeks in the trial comprising three weeks of monitoring to stabilize baselines and ensure adherence and commitment to participation before randomization into an intervention arm for an additional period of 52 weeks. Following a completed screening visit and meeting the inclusion and exclusion criteria, each patient will have a total of 16 study visits. At weeks 3, 5, 8, 16, 20, 29, 35, 44 and at 2 weeks after the end of the study, each patient will receive a phone call to document any adverse effects or other changes. There will be no long term follow-up following the end of the study.

Participants will be treated with either insulin only (control arm) for the whole duration of the study or with insulin and semaglutide weekly s.c. injection (titrated up to 1 mg or the maximum tolerated dose, see below) for the first six months then either dapagliflozin (Triple therapy arm) or placebo (dual therapy) daily will be added for an additional six months. Semaglutide will be started at a dose 0.25 mg once weekly for two weeks then increased to 0.5 mg dose. If well tolerated it will be increased to 1 mg once weekly at week 4. Dapagliflozin (or placebo) will be started at a dose of 5 mg daily at week 26 and if well tolerated increased after 1 week to 10 mg (2x5mg tablets) daily to ensure drug tolerability and to avoid any hypoglycemia or hypotension. Patients who do not tolerate the increase in dosing of either medications will be maintained at the highest tolerated dose.

Treatments used

1- Insulin: All patients (114 patients) will continue to self-administer their prescribed insulin as part of their standard of care. Insulin dose will be titrated during the study to control blood glucose average between 90-180 mg/dl and to avoid hypoglycemia (blood sugar <70 mg/dl) or hyperglycemia (blood sugar >250 mg/dl). Among all patients, 38 patients will be randomized (open-label) to remain on insulin therapy only (standard therapy group) while the other 76 will start the investigational therapies.
2- Semaglutide: a once weekly a GLP-1 mimetic injection (pen) indicated for type 2 diabetes. Doses to be used: start at 0.25mg then increase to 0.5mg following 2 weeks and to 1mg weekly s.c. injection 2 weeks later. Semaglutide will be added to a background of insulin treatment for 12 months in 76 patients who will be randomized (double-blind) into 2 groups (dual and triple therapy groups) at the end of 26 weeks.
3- Farxiga (dapagliflozin): a once daily SGLT-2 inhibitor tablet indicated for type 2 diabetes. Doses used: starting with 5mg and increase after 1 week to 10mg oral administration daily. Dapagliflozin will be started according to the randomization (Interactive Web System, hosted by University of Glasgow) in 38 patients at 26 weeks as an additional treatment on a background of insulin and semaglutide therapy and end at 52 weeks (triple therapy group).
4- Placebo: dapagliflozin matching placebo (supplied by Astra Zeneca) will be started in 38 patients at 26 weeks as an additional treatment on a background of insulin and semaglutide therapy and end at 52 weeks (dual therapy group).

As shown in **Table 1,** fasting blood and urine samples will be obtained prior to drug administration (weeks 0) and at 13, 26, 39 and 52 weeks after the commencement of the study. Optional fat biopsies from the abdominal subcutaneous tissue will be collected at weeks 0, 26 and 52. Continuous Glucose Monitoring (Dexcom G6 with blinded receiver) will be performed for three 21-days periods on weeks -3 to 0, 23 to 26 and from 49 to 52 weeks. An optional 24hr blood pressure monitoring will be performed at baseline (week -1) and at 13, 25, 39 and 51 weeks in order to characterize the time course of antihypertensive effects, which may be early (i.e. independent of weight loss) as indicated by our preliminary data. High fat high caloric (HFHC) Meal challenges will be performed at baseline (week -1) and at 25 and 51 weeks with assessment of glucose excursions and postprandial glucagon (AUC) as well as postprandial inflammation and oxidative stress. Telephone calls will be conducted at weeks 3, 5, 8, 16, 20, 29, 35, 44 and at two weeks after the end of the study to document any side effects or any other patient's reported issues or comments.

Study Visits:

Screening Visit (Screening Visit, Week -8 to -4): Patients will be asked to read and understand the consent and any questions regarding the protocol will be answered. If they want to participate in the study, they will be asked to sign the informed consent form. Their medical history and current medications will be obtained as well as blood pressure and vitals. A physical examination will also be done. Blood samples will be taken in order to evaluate HbA1c, CBC, CMP, C-peptide, kidney and liver functions, ketone bodies, and urine for pregnancy status in females.

Study Visit 1 (Week -3): Participants who meet entry criteria will be scheduled to for their next visit at -3 week. Vitals will be taken including resting (min. 10 minutes) blood pressure measurement (will be repeated from same arm every visit). Insulin pump will be downloaded and/or CGM/fingerstick blood sugar will be reviewed and the appropriate insulin adjustments will be made by the investigator to target fasting blood glucose concentrations between 90-180 mg/dl if possible at all times without having additional hypoglycemia (less than 70 mg/dl). Blood sugars and ketone bodies will be measured via a fingerstick blood sample using glucose and ketones meter (Abbott Laboratories) and daily blood sugars collected and reviewed. Urine for pregnancy status and ketone bodies assessment. We will instruct patients on how to use and insert the Continuous Glucose Monitoring System (Dexcom G6 with blinded receiver). All subjects will be advised to monitor their capillary blood glucose by CGM or fingerstick at least 4 times a day (before meals and at bedtime) through-out the study and to wear their CGM constantly for next 20 day. Carb counting technique will be reviewed and any questions answered on how to use the pump or calculate insulin doses.

Study Visit 2 (Week -1±2days): Patients will be asked to come in the fasting state (no food or calorie intake for 10-12 hours). Their vitals will be taken. Blood sugars and ketone bodies will be measured via a fingerstick blood sample using glucose and ketones meter (Abbott Laboratories) to assess their glycemic control prior to meal challenge. If their blood sugar is below 70mg/dl or more than 250mg/dl the visit will be rescheduled for an

alternative date. Insulin pump will be downloaded and/or CGM/fingerstick blood sugar will be reviewed and insulin adjustment will be made if necessary. An IV port will be placed in order to obtain blood samples throughout the duration of the visit. Patients will undergo a meal challenge test (described below). At the end of the meal test, they will be asked to wear a 24 hour blood pressure monitor (optional). A 24 hr urine container will be dispensed to collect a 24 hour urine sample to bring in at the Week 0 visit. Daily Glucose logs will be reviewed.

Study visit 3 (Week 0±2days): Patients will be asked to come in the fasting state (no food or calorie intake for 10-12 hours). Vitals will be taken. Blood sugars and ketone bodies will be measured via a fingerstick blood sample using glucose and ketones meter (Abbott Laboratories) and daily blood sugars collected and reviewed. The 24 hour urine container will be collected. A fasting blood sample will be taken (45 mL) to evaluate study endpoints, CBC, CMP, HbA1c, and fructosamine. A urine sample will be collected to evaluate ketone bodies and, in females, pregnancy status. Insulin pump will be downloaded and/or CGM/fingerstick blood sugar will be reviewed and the appropriate insulin adjustments will be made by the investigator. In general, insulin dose adjustments should not be >10% from current total insulin dose or >20% from baseline (week 0) total insulin dose. They will discontinue use of the study CGM. An optional fat biopsy will be performed. We will ask them to fill out quality of life questionnaires. Study drug will be dispensed depending on what group they are randomized to. This includes either (1) staying on the insulin regimen given by their doctor, or (2) a combination of their current insulin regimen and a once daily injection of semaglutide. Semaglutide will be started on site at the 0.25 mg dose. The 24 hr blood pressure monitor will be collected.

Study Visit 4 (Week 1±2days): Vitals will be taken. Blood sugars and ketone bodies will be measured via a fingerstick blood sample using glucose and ketones meter (Abbott Laboratories) and daily blood sugars collected and reviewed. Insulin dose will be adjusted if needed as in visit 3. At any time, if HbA1c is <7%, insulin dose adjustment should not be >5%. Semaglutide 0.25mg will be injected on site. Participants will be instructed to call their research center or their doctor directly in case of any problem or untoward side effects. They will be specifically asked to call if they have unmanaged hypoglycemia (blood sugar <70 mg/dl) or hyperglycemia (blood sugar >250 mg/dl) or if occurring in more than one occasion.

Study Visit 5 (Week 2±2days): Vitals will be taken. Blood sugars and ketone bodies will be measured via a fingerstick blood sample using glucose and ketones meter (Abbott Laboratories) and daily blood sugars collected and reviewed. Insulin dose will be adjusted if needed as in visit 3. Semaglutide 0.5mg dose will be injected on site. If they are experiencing mild nausea, they will be continued on the 0.25 mg dose for another week. Supply of the drug will be given to last until the next visit. Patients will be advised to increase the study dose to 0.5 mg a day.

Study Visit 6 (Week 4±1 week): Patients will be asked to bring in used study pens for drug accountability (to be recorded in eCRF). Vitals will be taken. Blood sugars and ketone bodies will be measured via a fingerstick blood sample using glucose and ketones meter

(Abbott Laboratories) and daily blood sugars collected and reviewed. Insulin dose will be adjusted if needed. Supply of the drug will be given to last until the next visit. A urine sample will be collected to evaluate ketone bodies and, in females, pregnancy status. Patients will be advised to increase the study dose to 1.0 mg a day. If they experience mild nausea, they will be dropped from the study. A 24 hr urine container will be dispensed to collect a 24 hour urine sample to bring in at the Week 13 visit.

Study Visit 7 (Week 13±1 week): Patients will be asked to come in the fasting state (10-12 hours fasting), and to bring in your used study pens for drug accountability. Blood sugars and ketone bodies will be measured via a fingerstick blood sample using glucose and ketones meter (Abbott Laboratories) and daily blood sugars collected and reviewed. Insulin dose will be adjusted if needed as in. Supply of the drug will be given to last until visit 26. Vitals will be taken. A urine sample will be collected to evaluate ketone bodies and, in females, pregnancy status. The 24 hour urine container will be collected. A fasting blood sample will be taken (45 mL) to evaluate study endpoints, CBC, CMP, HbA1c, and fructosamine. Patients who agreed to participate in the24 hour blood pressure monitoring will be asked to wear it for the next 24 hr.

Study Visit 8 (Week 23±1 week): Patients will be asked to bring in used study pens for drug accountability. Vitals will be taken. Blood sugars and ketone bodies will be measured via a fingerstick blood sample using glucose and ketones meter (Abbott Laboratories) and daily blood sugars collected and reviewed. A urine sample will be collected to evaluate ketone bodies and, in females, pregnancy status. Insulin dose will be adjusted if needed. All subjects will be reminded to monitor their capillary blood glucose by fingerstick at least 4 times a day (before meals and at bedtime) through-out the study. CGM will be inserted and patients asked to wear their CGM constantly for next 20 day.

Study visit 9 (Week 25±1 week): Patients will be asked to come in the fasting state, and to bring in used study pens for drug accountability. Vitals will be taken. A urine sample will be collected to evaluate ketone bodies. Blood sugars and ketone bodies will be measured via a fingerstick blood sample using glucose and ketones meter (Abbott Laboratories) and daily blood sugars collected and reviewed. Insulin dose will be adjusted if needed. HbA1c will be drawn. Supply of the drug will be given to last until the next visit. A 24 hr urine container will be dispensed to collect a 24 hour urine sample to bring in at the Week 26 visit. A HFHC Meal challenges will be performed as in visit 2. Patients who agreed to participate will be asked to wear a 24 hour blood pressure monitoring for the next 24 hr.

Participants will be provided supplies for urine ketones measurement (KetoStix) and blood ketones meter. Research coordinator will instruct the patients on the use of meter and reading of the measuring strips. Patients will be instructed to start measuring urinary and blood ketones every morning for three consecutive days prior to next visit (starting dapagliflozin treatment) and to report results at the time of their next visit.

Study visit 10 (Week 26±1 week): Patients will be asked to come in the fasting state, and to bring in used study pens for drug accountability. Previous 3 days blood and urine ketones levels will be reviewed. Patients with normal fasting urine (<+, or < small) or blood ketone

(<0.6 mmol) will proceed to next treatment phase included in this visit. Patients who report higher ketone values in at least 1 out of the 3 days will have their glucose levels, insulin dose, carb intake reviewed and managed as necessary. They will be rescheduled for next week (7±1 day) and asked to repeat the 3 days testing before next visit.
Vitals will be taken. A urine sample will be collected to evaluate ketone bodies. Blood sugars and ketone bodies will be reviewed via a fingerstick blood sample using glucose and ketones meter (Abbott Laboratories) and daily blood sugars collected and reviewed. Insulin dose will be adjusted if needed. In general, insulin dose adjustments should **NOT** be >10% from current total insulin dose (or 5% if HbA1c is <7%) or >20% from baseline total insulin dose. The adjusted total insulin dose in this visit will serve as the baseline total insulin dose for the remaining 6 months of the study and future limits of dose adjustments will be based on this new baseline. The CGM will be collected. A urine sample will be collected to evaluate ketone bodies and, in females, pregnancy status. The 24 hour urine container will be collected. A fasting blood sample will be taken (45 mL) to evaluate study endpoints, CBC, CMP, C-peptide, and Fructosamine. An optional fat biopsy will be performed. We will ask patients to fill out quality of life questionnaires. At this point, those patients randomized to receive semaglutide, they will now be randomized to receive an actual dapagliflozin pill or a placebo pill that looks like dapagliflozin. If they are randomized to receive actual dapagliflozin, they will start at the 5 mg dose for one week.
Patients will be asked to perform a daily (preferably early morning or before bedtime) urinary test of ketone bodies using KetoStix and record all outcomes. If results of these scheduled (i.e. not done due to symptoms) ketone measurements, are small or higher (+1 or more), they will be instructed to repeat the measurement after 60 min. If consecutive measurements show moderate or higher ketones in urine, the patient will be instructed to call the research center or their doctor directly. Patients with dual glucose and ketones meters will be instructed to record and report their blood ketones in dedicated study diaries. If blood ketones are 0.6 mM or above, they should repeat the measurement within 60 min. If consecutive measurements show 0.6mm or higher ketones in blood, the patients will be instructed to call the research center or their doctor directly. For any abnormal ketone levels taken due to symptoms, the DKA consensus guidelines (Danne T et al., Diabetes Care. 2019 Feb 6) are to be followed instead (as in section 21).
Participants will be instructed to call their research center or their doctor directly in case of any problem or untoward side effects. They will be specifically asked to call if they have unmanaged hypoglycemia (blood sugar <70 mg/dl) or hyperglycemia (blood sugar >250 mg/dl) or if occurring in more than one occasion. Participants will be instructed to remain hydrated and report any genital mycotic or urinary tract infections.

Study visit 11 (Week 27±2 days): Patients will be asked to bring in used study pens for drug accountability (recorded in eCRF). Vitals will be taken. Daily blood sugars and urinary ketones measurements using KetoStix will be collected and reviewed. Blood sugars and ketone bodies will be measured via a fingerstick blood sample using glucose and ketones meter (Abbott Laboratories). A urine sample will be collected to evaluate urine ketone level. Insulin dose will be adjusted if needed. In general, insulin dose adjustments should not be >10% from current total insulin dose (or 5% if HbA1c is <7%) or >20% from NEW baseline total insulin dose established at visit 10. If at any time during the study total insulin dose reduction is >20% and total insulin dose in <0.40 U/Kg, additional monitoring

protocol (see later) will be implemented. Supply of the drug will be given to last until week 39. If patients are receiving actual dapagliflozin, they will now start at the 10 mg dose for the remainder of the study.

Study Visit 12 (Week 31±1 week): Patients will be asked to bring in used study pens and tablets for drug accountability. Vitals will be taken. Daily blood sugars and urinary ketones measurements using KetoStix will be collected and reviewed. Blood sugars and ketone bodies will be measured via a fingerstick blood sample using glucose and ketones meter (Abbott Laboratories). A urine sample will be collected to evaluate ketone bodies and, in females, pregnancy status. Insulin dose will be adjusted if needed as in visit 11. A blood sample will be taken (15 mL) to evaluate CBC and CMP. A 24 hr urine container will be dispensed to collect a 24 hour urine sample to bring in at the Week 39 visit.

Study visit 13 (Week 39±1 week): Patients will be asked to come in the fasting state, and to bring in used study pens and tablets for drug accountability. Vitals will be taken. Daily blood sugars and urinary ketones measurements using KetoStix will be collected and reviewed. Blood sugars and ketone bodies will be measured via a fingerstick blood sample using glucose and ketones meter (Abbott Laboratories). Insulin dose will be adjusted if needed as in visit 11. Supply of the drug will be given to last until the end of the study. A urine sample will be collected to evaluate ketone bodies and, in females, pregnancy status. The 24 hour urine container will be collected. A fasting blood sample will be taken (45 mL) to evaluate study endpoints, CBC, CMP, HbA1c, and Fructosamine. Patients who have provided consent will be asked to wear a 24 hour blood pressure monitor.

Study visit 14 (Week 49±1 week): Patients will be asked to bring in used study pens and tablets for drug accountability. Vitals will be taken. Daily blood sugars and urinary ketones measurements using KetoStix will be collected and reviewed. Blood sugars and ketone bodies will be measured via a fingerstick blood sample using glucose and ketones meter (Abbott Laboratories). A urine sample will be collected to evaluate ketone bodies and, in females, pregnancy status. Insulin dose will be adjusted if needed as in visit 11. All subjects will be reminded to monitor their capillary blood glucose by fingerstick at least 4 times a day (before meals and at bedtime) through-out the study. CGM will be inserted and patients asked to wear their CGM constantly for the next 20 days.

Study visit 15 (Week 51±1 week): Patients will be asked to come in the fasting state, and to bring in used study pens and tablets for drug accountability. Vitals will be taken. Daily blood sugars and urinary ketones measurements using KetoStix will be collected and reviewed. Blood sugars and ketone bodies will be measured via a fingerstick blood sample using glucose and ketones meter (Abbott Laboratories). A urine sample will be collected to evaluate urine ketone levels. Insulin dose will be adjusted if needed as in visit 11. A 24 hr urine container will be dispensed to collect a 24 hour urine sample to bring in at the Week 52 visit. A HFHC Meal challenges will be performed as in visit 2. Patients will be asked to wear the optional 24 hour blood pressure monitor.

Study visit 16 (Week 52±2 days): Patients will be asked to come in the fasting state, and to bring in used study pens and tablets for drug accountability. Vitals will be taken. Daily

blood sugars and urinary ketones measurements using KetoStix will be collected and reviewed. Blood sugars and ketone bodies will be measured via a fingerstick blood sample using glucose and ketones meter (Abbott Laboratories). Insulin dose will be adjusted if needed. The CGM will be collected. An optional fat biopsy will be performed. A urine sample will be collected to evaluate ketone bodies and, in females, pregnancy status. The 24 hour urine container will be collected. A fasting blood sample will be taken (45 mL) to evaluate study endpoints, CBC, CMP, HbA1c, C-peptide, and Fructosamine. We will ask the patients to fill out quality of life questionnaires. The patient will receive a physical. After this visit, they will be discharged from the study.

Safety phone calls will be conducted at weeks 3, 5, 8, 16, 20, 29, 35, 44 and at two weeks after the end of the study to document any side effects or other changes.

Table 1: Schedule of visits and testing procedures.

Visit #	-	1	2	3	4	5	6	7	8	9	10	11	12	13	14	15	16
Week#(±2-7 days as per text)	-8 to -4	-3	-1	0	1	2	4	13	23	25	26	27	31	39	49	51	52
Study Procedures	Screening	Run-in period		Randomization, start semaglutide							Randomize/Start dapagliflozin/ placebo						End of study
History	X																
Physical	X																X
Vitals	X	X	X	X	X	X	X	X	X	X	X	X	X	X	X	X	X
CBC	X			X				X			X		X	X			X
CMP	X			X				X			X		X	X			X
C-Peptide	X										X						X
Kidney and liver profiles	X			X				X			X		X	X			X
Pregnancy test	X	X		X			X	X	X		X		X	X	X		X
Study drug titration						X	X					X					
Dispense medications				X	X	X	X	X			X	X		X			
HbA1c	X			X				X		X				X			X
Fructosamine				X				X			X			X			X
24 hour BP (optional)			X					X		X				X		X	
CGM		X	X						X	X					X	X	
Daily Fingerstick glucose review		X	X	X	X	X	X	X	X	X	X	X	X	X	X	X	X
Daily urinary ketones review											X	X	X	X	X	X	X
Insulin dose review		X	X	X	X	X	X	X	X	X	X	X	X	X	X	X	X
Ketone bodies (blood/urine)	X	X	X	X	X	X	X	X	X	X	X	X	X	X	X	X	X
Adverse Event reporting		X	X	X	X	X	X	X	X	X	X	X	X	X	X	X	X
Meal Challenge			X							X						X	
Blood endpoints				X				X			X			X			X
Fat biopsy (optional)				X							X						X
24 hr Urine				X				X			X			X			X
Quality of life questionnaires				X							X						X

Drug Blinding:

The randomizations will be performed using an Interactive Web System, hosted by University of Glasgow. One hundred and fourteen (114) patients will be randomized as follows: participants will first be randomized to semaglutide plus usual insulin therapy or insulin therapy only (no placebo for semaglutide or other placebo injection will be provided), in a 2:1 ratio. Those who remain on semaglutide at six months will then be randomized in a 1:1 ratio to receive dapagliflozin or matched placebo. Each randomization will be stratified by study site and the second randomization will also include HbA1c stratification. The randomization schedules will be generated by a statistician with no other involvement in the trial, using the method of randomized permuted blocks. Only those staff responsible for the maintenance of the randomization system will have access to the randomization sequences during the trial. The first randomization (which will be revealed to the researcher at the point of randomization) will use random block sizes to reduce predictability. Researchers at study sites will have no knowledge of the next allocation to be issued. The second randomization (which will be double-blind) will use a different. At the point of randomization, the researcher will be given a medication pack ID number, but will not know whether that medication pack contains Dapagliflozin or placebo. For each randomization, a web-based randomization system (developed and maintained by the Robertson Centre for Biostatistics) will be used. The system will include an emergency unblinding facility for the dapagliflozin/placebo randomization

Astra Zeneca Inc. will supply dapagliflozin and matching placebo tablets in unlabeled and identical packaging; semaglutide pens will be obtained commercially and will be used in an open label fashion.

Breaking of the study blind will be performed only: (i) for SUSARs; and, (ii) where knowledge of the treatment is considered by local health personnel absolutely necessary for further management of the patient. A central unblinding facility based at the Robertson Centre for Biostatistics, University of Glasgow, will be available by telephone. Notification of all unblinding will be sent to the Chief Investigator

During the course of the trial there will be no attempt to identify group assignment for any participant (except for unblinding in the case of safety concerns) and the investigators will execute the protocol in the manner it was approved independent of the group assignment.

Drugs will be stored in a locked cabinet or locked temperature controlled refrigerator at 4C at the research facility of the Diabetes and Endocrinology Center of WNY. IND application for use of all drugs (dapagliflozin and semaglutide) in type 1 diabetes will be submitted to FDA and no study procedure will start before all local and federal approvals are obtained.

Clinical and Laboratory Procedures

1) **CMP, CBC, HbA1c, fructosamine, glucose, glucagon, GLP-1 and FFA measurements:** Blood CMP, CBC, HbA1c, fructosamine and glucose will be measured through Quest diagnostic (US site) and NHS labs (UK site). Free fatty acid levels will be measured by a colorimetric assay (Wako, Richmond, VA). GLP-1 and

glucagon will be measured from samples collected in protease and DPP-IV inhibitors by ELISA kits from Millipore.

2) **Meal Challenge test:** In order to assess the postprandial changes induced by dapagliflozin, semaglutide or the combination of both compared to insulin only, a meal challenge will be performed before starting treatments, at six months and at end of study using a 910 Calorie High fat High carbohydrate meal (HFHC) as in several of our previous studies (HFHC meal includes an egg muffin sandwich, a sausage muffin sandwich and two hash browns which contain 88g carbohydrate, 51 g fat (33% saturated) and 34 g protein). Participants will be asked to attend fasting prior to breakfast insulin bolus; basal insulin will continue during the duration of the meal, however patients will not bolus until the 3 hour mark unless blood glucose values reach above 400 mg/dL. Sequential blood samples will be obtained at 0, 15, 30, 45, 60, 90, 120, 150 and 180 min after consuming the meal. Samples at 15, 30, 45, 90 and 150 min will be 5ml while those at 0, 60, 120 and 180min will be 30 ml (total volume=145 ml). Spot urine will be collected at baseline, 1 and 3hr. Blood will be collected from an indwelling intravenous canula in a superficial forearm vein.
3) **Optional 24 hour Ambulatory BP monitoring:** Participants will be instructed on the use of 24 hour ambulatory BP monitoring device. This device will measure BP and pulse every 30 minutes for 24 hours. In addition to the scheduled measurements there will be an option for additional manual measurements if systolic blood pressure is >160 or <90 mmHg.
4) **Optional Fat aspiration procedure:** Abdominal subcutaneous fat tissue aspiration will be performed at a 10 cm distance from umbilicus. Participants will be asked not to take aspirin or NSAIDS in the previous 72 hours - if they have, the procedure will not be done. Skin will be prepared with povidone-iodine (Betadine) and alcohol. A sterile drape will be placed around the appropriate area. 3cc of 1% lidocaine will then be administered subcutaneously (the dose will not exceed 4.5mg/kg body weight). After adequate anesthesia has been achieved, aspiration of fat tissue will then be with a 3-holed canula (Tulip Instrumentation, length: 15cm, diameter: 2.1mm) fixed to a 10mL syringe. After getting adequate fat tissue (500mg-3g), the puncture site will be pressed for at least ten minutes before the patient rises up from supine position (to minimize bruising). Participants will then be discharged home. Adipose tissue will be centrifuged to remove blood and fluid contaminants. Upper adipose tissue will be collected into a separate sterile tube, washed twice with cold sterile Phosphate Buffered Saline (PBS) and centrifuged to remove the PBS. The adipose tissue sample will be weighed and approximately 500 mg transferred to a separate tube for analysis. Total RNA, nuclear extracts and total cell lysates will be prepared.
5) **Questionnaires:** Participants will be asked to complete the standard Diabetes Specific Quality of Life Scale (DSQOLS) and Problem Areas In Diabetes survey (PAID). Questionnaires attached at the end of this plan.
6) **MNC isolation (for future ancillary studies):** Blood samples will be collected in Na-EDTA as an anticoagulant. Three and a half mL of anticoagulated blood sample are carefully layered over 3.5 mL of PMN medium (Cedarlane Laboratories, Hornby, ON) and then centrifuged. At the end of this process, two bands separate out at the top of the RBC pellet. The top band consists of MNC, while the bottom consists of PMN. The

MNC band is harvested and washed twice with Hank's balanced salt solution (HBSS). This method provides yields greater than 95% pure PMN and MNC suspensions.

7) **NFκB DNA binding activity (for future ancillary studies):** Nuclear NFκB DNA binding activity is measured by EMSA. Nuclear extract is prepared from MNC and adipose tissue by high salt extraction as described by Andrews et al (19). The specificity of the bands is confirmed by supershifting these bands with specific antibodies against Rel-A (p65) and p50 (Santa Cruz Biotechnology, CA) and by competition with cold oligonucleotides. DNA binding activity of NFκB will be adjusted to Oct1 DNA binding activity to correct for any variable.
8) **Plasma concentrations of inflammatory mediators (for future ancillary studies):** Concentrations of TNFα, IL-1β, IL-18, adiponectin, IL-1RA will be measured in plasma using ELISA kits (R&D Systems, MN). CRP and SAA concentrations will be measured using an ELISA kit from Alpha Diagnostics International Inc. (San Antonio, TX).
9) **Plasma vasodilators and vasoconstrictors (for future ancillary studies):** Plasma ANP, NT-proBNP, b-NP, cGMP, cAMP, angiotensinogen, renin and angiotensin II concentrations will be measured by commercially available ELISA kits.
10) **15-isoprostane F_{2t}** (also known as 8-epi-PGF2α or 8-iso-PGF2α) **(for future ancillary studies):** will be measured in urine samples using an ELISA kit from Oxford Biomedical Research (Oxford, MI).
11) **Quantification of mRNA in MNC and adipose tissue (for future ancillary studies).** Total RNA is isolated from MNC and adipose tissue using commercially available RNAqueous®-4PCR Kit (Ambion, Austin, TX). The quality and quantity of the isolated RNA is determined before using the RNA. Real Time RT-PCR is performed using Cepheid Smart Cycler (Sunnyvale, CA) in which 2 µL cDNA, 10 µL Sybergreen Master mix (Qiagen, CA) along with 0.5 µL of 20 µM gene specific primers for IL-1β, IL-RA, TLR-4, TNFα, IL-12, SOCS-3 and JNK-1 (Life Technologies, MD) are used. The specificity and the size of the PCR products are tested by adding a melt curve at the end of the amplifications and by running it on a 2% agarose gel. All values are normalized to 18S expression and β-actin.

10.2 Describe what data will be collected.

NOTE: For studies with multiple data collection points or long-term follow up, consider the addition of a schedule or table in your response.

Response:

Data will be collected as described in table 1 above. The following aspects of the study will be performed and data collected and reported by patients:

1- A CGM device will be provided for use for 3 20-days periods. Detailed instructions on changing the sensor at 10 days will be provided. Patients will be instructed to wear their CGM constantly during the assigned study periods. They will be asked to keep a diary of their food intake to measure their carbohydrate intake during the CGM period. Insulin doses will be titrated aiming for blood glucose between 90-180 mg/dl while avoiding hypoglycemia (<70 mg/dl) or hyperglycemia (>250 mg/dl).

2- Participants who agree to participate in the 24-hr ambulatory blood pressure monitoring will be provided with a 24-hour ambulatory BP monitor to be worn for 24 hours during week -1, 13, 25, 39 and 51.
3- All participants will be asked to monitor capillary blood glucose by finger stick before each meal and at bedtime.
4- For the entire duration of the study, the patients will maintain a diary to record any hypoglycemia and other untoward side effects e.g. nausea, change in appetite.
5- Starting at the 6 months mark, participants will be asked to perform a daily urinary test of ketone bodies using KetoStix and record all outcomes. If results are moderate to large, they will be instructed to repeat the test and to call the research center or their doctor directly if results are confirmed. Patients with dual glucose and ketones meters will be instructed to record and report their blood ketones as well. If blood ketones are above 0.6mM, they should repeat the test and contact the research center of their doctor if levels are confirmed.

10.3 List any instruments or measurement tools used to collect data (e.g. questionnaire, interview guide, validated instrument, data collection form).

Include copies of these documents with your submission.

Response: Source documents will be used to collect patient information. The CGM or insulin pump will be used to collect glucose and insulin data. Glucose and ketone meter will be used to measure glucose and ketone in blood. KetoStix for measuring ketones in urine. Blood pressure monitor will be used for 24 hour blood pressure reading. Glucose, food and urinary ketones diaries will be provided for patients to record their results.

10.4 Describe any source records that will be used to collect data about subjects (e.g. school records, electronic medical records).

Response: electronic medical records and research files.

10.5 Indicate whether or not ***individual*** *subject results, such as results of investigational diagnostic tests, genetic tests, or incidental findings will be shared with subjects or others (e.g., the subject's primary care physician) and if so, describe how these will be shared.*

Response: Individual participant lab results will be disclosed to the participant upon their request. If the participant requests documentation be shared with another physician, physician office or hospital the participant must come to the research center to collect said documentation or the documentation can be mailed to their given home address.

Patients will be assigned a unique study number. All identifiable personal information will be stored locally in locked filing cabinets. Data entered on the

electronic Case Report Form will only be identified by the unique study number. Thus, no personal information will be shared with the data center (Robertson Centre for Biostatistics, University of Glasgow, UK).

*10.6 Indicate whether or not **study** results will be shared with subjects or others, and if so, describe how these will be shared.*

Response: Not Applicable. Study results will not be shared with the subjects. However, unidentifiable study results could be published in the form of a manuscript or abstract and will be reported to JDRF, Astra Zeneca, and to clinicaltrials.gov and other regulatory agencies including FDA.

11.0 Study Timelines

11.1 Describe the anticipated duration needed to enroll all study subjects.

Response: Accrual is expected to be at the rate of 4 subjects/month/site, thus we should complete enrollment of 57 patients/site in approximately 13-15 months. We expect to complete all study clinical activities in 30 months.

11.2 Describe the duration of an individual subject's participation in the study. Include length of study visits, and overall study follow-up time.

Response: 55 Weeks. Study visits will be 60-90 minutes long except for visits 2, 9 and 15 which will be about 6hr long each

11.3 Describe the estimated duration for the investigators to complete this study (i.e. all data is collected and all analyses have been completed).

Response: 36 Months

12.0 Setting

12.1 Describe all facilities/sites where you will be conducting research procedures. Include a description of the security and privacy of the facilities (e.g. locked facility, limited access, privacy barriers). Facility, department, and type of room are relevant. Do not abbreviate facility names.

NOTE: Examples of acceptable response may be: "A classroom setting in the Department of Psychology equipped with a computer with relevant survey administration software," "The angiogram suite at Buffalo General Medical Center, a fully accredited tertiary care institution within New York State with badge access," or, "Community Center meeting hall."

Response:

1) Research will be conducted at the Diabetes Endocrinology Research Center of WNY, located at *1000 Youngs Road, Suite 105, Williamsville NY 14221* and at the CTRC located in 875 Ellicott St. Buffalo NY14203.The Diabetes Research Center has facilities and exam rooms available for insulin pump download, CGM device download, meal and infusion studies and presence of study coordinator and registered nurse for data collection and blood work at all times. One of the investigators will be available at all times to address patients' related issues. CTRC location is a fully equipped laboratory with Equipment include ultra-low freezers for sample storage, centrifuges, microscopes for sample preparation, infusion pumps, ELISA, PCR and immunoblotting instrumentation.

2) Research will also be conducted at the NHS Greater Glasgow and Clyde Clinical Research Facility (CRF, Glasgow Royal Infirmary, UK and at the Diabetes Centre (Clinic D), Stobhill Ambulatory Care Hospital, Glasgow, UK. There are facilities and exam rooms available for insulin pump download, CGM device download, meal and infusion studies and presence of research nursing staff for data collection and blood work at all times. One of the investigators will be available at all times to address patients' related issues. The CRF has equipment for sample preparation, infusion pumps, ultra-low freezers for sample storage and centrifuges. ELISA, PCR and immunoblotting facilities are available in adjacent NHS and University facilities.

12.2 For research conducted outside of UB and its affiliates, describe:

- *Site-specific regulations or customs affecting the research*
- *Local scientific and ethical review structure*

NOTE: This question is referring to UB affiliated research taking place outside UB, i.e. research conducted in the community, school-based research, international research, etc. It is not referring to multi-site research. UB affiliated institutions include Kaleida Health, ECMC, and Roswell Park Cancer Institute.

Response:

☒ **N/A:** This study is not conducted outside of UB or its affiliates.

13.0 Community-Based Participatory Research

13.1 Describe involvement of the community in the design and conduct of the research.

NOTE: Community-Based Participatory Research (CBPR) is a collaborative approach to research that equitably involves all partners in the research process and recognizes the unique strengths that each brings. CBPR begins with a research topic of importance to the community, has the aim of combining knowledge with action and achieving social change to improve health outcomes and eliminate health disparities.

Response:

☒ **N/A:** This study does not utilize CBPR.

13.2 Describe the composition and involvement of a community advisory board.

Response:

☒ **N/A:** This study does not have a community advisory board.

14.0 Resources and Qualifications

14.1 Describe the qualifications (e.g., education, training, experience, expertise, or certifications) of the Principal Investigator ***and*** *staff to perform the research. When applicable describe their knowledge of the local study sites, culture, and society. Provide enough information to convince the IRB that you have qualified staff for the proposed research.*

NOTE: If you specify a person by name, a change to that person will require prior approval by the IRB. If you specify a person by role (e.g., coordinator, research assistant, co-investigator, or pharmacist), a change to that person will not usually require prior approval by the IRB, provided that the person meets the qualifications described to fulfill their roles.

Response: All study personnel are educated, trained, and licensed as required for their delegated role in this study. All study personnel have also received the required university training and will be trained by the PI before the study starts. The PIs, CO-investigators and research team have conducted similar studies in this patient population using similar agents and have the expertise to execute the study protocol and manage any adverse events.

Describe other resources available to conduct the research.

14.2 Describe the time and effort that the Principal Investigator and research staff will devote to conducting and completing the research.

NOTE: Examples include the percentage of Full Time Equivalents (FTE), hours per week. The question will elicit whether there are appropriate resources to conduct the research.

Response: The principal investigator supervises the research project and weekly research meetings are conducted to discuss the recruitment rate, resolve and discuss issues related to the conduct, safety, analysis of the study and related publications. PI is expected to spend 15% of his academic time on this research. The co-investigators, study coordinator and research staff provide coverage to the research related activity for 365 days a year.

14.3 Describe the availability of medical or psychological resources that subjects might need as a result of anticipated consequences of the human research, if applicable.

NOTE: One example includes: on-call availability of a counselor or psychologist for a study that screens subjects for depression.

Response: Available medical literature will be provided as deemed appropriate or requested by patient through UB libraries, PubMed, Google scholar as all the investigators have access to medical literature through listed resources above

The patient will also have access to physician (Investigators and Co-Investigators) who will be available to address any adverse effects or other questions during the course of the study who will be available to address any adverse effects or other questions during the course of the study

14.4 Describe your process to ensure that all persons assisting with the research are adequately informed about the protocol, the research procedures, and their duties and functions.

Response: Before beginning of enrollment all research staff will be asked to review the protocol and a training session will be conducted to provide protocol details, explain technical points and answer staff questions. All training session will have a sign in sheet to document such training. Continuous education through training meetings, conferences and discussions will continue through the study period and when revision are made to protocol.

15.0 Other Approvals

15.1 Describe any approvals that will be obtained prior to commencing the research (e.g., school, external site, funding agency, laboratory, radiation safety, or biosafety).

Response: FDA IND filling is required.

☐ **N/A:** This study does not require any other approvals.

16.0 Provisions to Protect the Privacy Interests of Subjects

16.1 Describe how you will protect subjects' privacy interests during the course of this research.

NOTE: Privacy refers to an individual's right to control access to him or herself. Privacy applies to the person. Confidentiality refers to how data collected about individuals for the research will be protected by the researcher from release. Confidentiality applies to the data.

Examples of appropriate responses include: "participant only meets with a study coordinator in a classroom setting where no one can overhear", or "the participant is reminded that they are free to refuse to answer any questions that they do not feel comfortable answering."

Response: Our clinical providers involved in the study will identify potential patients for recruitment from the Diabetes-Endocrinology Center of WNY Clinics according to the inclusions and exclusion criteria and through advertisements. Patient who qualify will be asked in private during their one on one consultation time with the physician if they wish to participate in the research study. If the patient agrees, the research coordinator will contact them privately for a possible telephone screening and ask if time is appropriate for this discussion. The patients who call for potential participation in the study due to advertisement flyers will be screened over the phone with the research coordinator, using our telephone screening form.

When the patient is being seen at our clinics for the first time they sign the "Consent to use and disclosure of protected health information" form which clearly states that their protected health information (PHI) can be used for review in preparation for possible research. Additionally, a partial HIPAA waiver will be obtained to access medical records when screening for patients.

If the patient passes the telephone screening, they will be asked to make an appointment to review and sign the consent. Patient will do this in a private room in the research unit and will be allowed to discuss the consent in detail with the research coordinator and or study doctor. Patient will be notified that it is completely voluntary to participate in the research study and can withdraw at any time.

We will not be accessing any medical information of the patients for whom the services are not provided by our clinic providers before partial HIPAA is obtained or IC is signed.

16.2 Indicate how the research team is permitted to access any sources of information about the subjects.

*NOTE: Examples of appropriate responses include: school permission for review of records, consent of the subject, HIPAA waiver. This question **does apply** to records reviews.*

Response: Consent of the subject and partial HIPAA waiver.

17.0 Data Management and Analysis

17.1 Describe the data analysis plan, including any statistical procedures. This section applies to both quantitative and qualitative analysis.

Response:

Data (e.g. date of diagnosis, past medical history, drug treatment) extracted from local clinical records will be entered on to the eCRF under the study-specific coded identifier according to structured questions as stipulated in the Study Protocol. Biological samples (blood, urine and adipose tissue aspirates) will be collected from the study participants at the study visits according to the Schedule of Assessments in the Study Protocol.

Measures relating to safety and routine care of the participants (e.g., biochemical measures of glycemic and lipidemic control, kidney function/ fluid balance, hepatic function, hematological variables) will be analysed at local clinical laboratories in order that they are available to study nurses and physicians. The confidentiality of these data will be secure, meeting or exceeding the regulations of the relevant Ethical Review Committees/ Institutional Review Boards. Samples for measurement of key study outcomes will also be measured at the central laboratory at the University of Buffalo. Blood, urine and other samples will be aliquoted into cryovials at site for freezing and storage. Electronic stored material (e.g., patient records, data from blood and urine samples, and data from Continuous Glucose Monitoring) will only be stored using the study specific coded identifier. Data will not be identifiable at the participant level without additional access to the code. The code will be available only to the investigators and the personnel performing the study. Access to any data held electronically will require study specific log-in codes. These are managed and logged by the Robertson Centre for Biostatistics, University of Glasgow, UK

Statistical plan. The primary objective of the proposed research is to evaluate the effect of addition of dapagliflozin to semaglutide on a background of insulin treatment in type 1 diabetes patients on HbA1c at six months following start of dapagliflozin. The similarities between the study groups, baseline values for subject's demographics and study endpoints will be compared using appropriate parametric tests. Transformations of the data in order to meet statistical assumptions may be considered. The primary endpoint of the study is to detect a difference in mean HbA1c change after six months of dapagliflozin or placebo addition to semaglutide and insulin treatment. Statistical analysis will be carried out using t-tests to detect difference between groups or the Wilcoxon test for paired data. Tests will be two-sided and tested at the 0.05 level. The results will be summarized as mean±SE. All randomized patients will be included in the analysis of the primary end point based on an Intent-to-treat model.

The secondary endpoints will be to compare changes in HbA1c between each of the triple therapy and dual therapy groups compared to standard of care group, mean weekly glucose concentrations, percent time spent in different glycemic thresholds, variability as measured by standard deviation, insulin dose, body weight, carbohydrate intake, blood pressure, and 24-hour blood pressure and QOL changes among the groups. A reduction in the glucose and glucagon AUC following the meal will constitute another secondary end point in this setting, ANCOVA analysis will be used. Since the secondary end-points are pre-specified and may as well be correlated, multiplicity control for multiple tests across secondary endpoints may not be necessary. However, Holm-Bonferroni correction

for multiplicity testing will be implemented if additional endpoints are tested or if the pre-specified secondary end-points are found to be not correlated.

17.2 *If applicable, provide a power analysis.*

NOTE: This may not apply to certain types of studies, including chart/records reviews, survey studies, or observational studies. This question is asked to elicit whether the investigator has an adequate sample size to achieve the study objectives and justify a conclusion.

Response:

Sample size determination is based in part on our current data showing that in type 1 diabetes, treatment with a GLP-1 agonist, liraglutide (1.2 and 1.8mg doses) for 3 months leads to HbA1c suppression by an average of 0.6% (SD= 0.5%). Dapagliflozin was recently shown to reduce HbA1c by 0.45% in type 1 diabetes patients (13). Additionally, empagliflozin treatment for 8 weeks was shown to reduce HbA1c by 0.4% in type 1 diabetics (20, 21) and our current data showing additional improvement in HbA1c by an additional 0.66% at 12 weeks when dapagliflozin was added to liraglutide and insulin therapy. Conservatively estimating that the combination therapy will suppress HbA1c by 0.5% (SD =<0.7%) compared to semaglutide alone (t-test), a sample size of 38 (32 evaluable patients) in each group will be sufficient to provide adequate power (β = 0.2) to detect a statistically significant difference (α = 0.05) taking in consideration a 15% dropout rate.

17.3 *Describe any procedures that will be used for quality control of collected data.*

Response:

An electronic case report form (e-CRF) will be used to collect study data at each site. The e-CRF will be developed by the study Data Centre at the Robertson Centre for Biostatistics, University of Glasgow. Access to the e-CRF will be restricted, with only authorised site-specific personnel able to make entries or amendments to their patients' data. It is the investigator's responsibility to ensure completion and to review and approve all data captured in the e-CRF.

Data will be validated at the point of entry into the e-CRF and at regular intervals during the study. Data discrepancies will be flagged to the study site coordinator and any data changes will be recorded in order to maintain a complete audit trail (reason for change, date change made, who made change).

The Robertson Centre for Biostatistics at the University of Glasgow will be responsible for collating and cleaning the data for the study. The Robertson Centre will also be responsible for data back-up and security. This center will also manage the electronic reporting of SUSARS on behalf of the sponsor.

To enable evaluations and/or audits from regulatory authorities, the investigators agree to keep records, including the identity of all participating subjects (sufficient information to link records, all original signed informed consent forms serious adverse event forms, source documents, and detailed records of treatment disposition. The records should be retained by the study country coordinators and investigator according to ICH GCP, local regulations, or as specified in the Clinical Study Agreement, whichever is longer.

Data from the study will be stored by the Chief Investigator for a minimum of 10 years. A final report of the study will be provided to the FDA and the MHRA as per requirements.

- No interim analysis is planned for efficacy so termination of study will be related to safety. Significant increases in hypoglycemic or DKA events as reviewed by DSMB might trigger termination or modification of study.
- Last observation carry forward will be used to fill missing data for the primary end point and some secondary end points. Missing data will not be filled for other endpoints.
- Any deviation(s) from the original statistical plan will be described and justified in the protocol and/or in the final report.
- All subjects entered into treatment will analyzed for primary end point on ITT bases. For secondary endpoints, analysis of completed and evaluable patients will performed per-protocol.

18.0 Confidentiality

A. Confidentiality of Study Data

*Describe the local procedures for maintenance of confidentiality of **study data and any records that will be reviewed for data collection**.*

*18.1 A. Where and how will all data and records be stored? Include information about: password protection, encryption, physical controls, authorization of access, and separation of identifiers and data, as applicable. Include physical (e.g. paper) **and** electronic files.*

Response: All data records will be stored on protected computers with unique passwords issued for each of the study team members and or in locked cabinets within the research center. These files will only be accessible by authorized study personnel.

18.2 A. How long will the data be stored?

Response: De-identified data storage has no expiration date and will be stored for a minimum of 7 years. The researchers may continue to rely on this for future use in research study.

18.3 A. Who will have access to the data?

Response: Those physicians, nurses, and laboratory staff that are on all documentation for the study will have access to the data.

18.4 A. Who is responsible for receipt or transmission of the data?

Response: Those physicians, nurses, and laboratory staff that are on all documentation for the study will have access to the data and can handle transfer of data. Study data will be transferred electronically (through eCRFs) to Robertson Centre for Biostatistics, University of Glasgow, UK

18.5 A. How will the data be transported?

Response: All data are stored at locally and will be transferred to Robertson Centre for Biostatistics, University of Glasgow, UK for data management and analysis. The transfer will be done through electronic CRFs processed on secure network.

Data will also be transported unless when is being archived. At that point files will be transferred to Iron Mountain for storage and archiving.

B. Confidentiality of Study Specimens

Describe the local procedures for maintenance of confidentiality of ***study specimens****.*

☐ **N/A:** No specimens will be collected or analyzed in this research.
(Skip to Section 19.0)

18.6 B. Where and how will all specimens be stored? Include information about: physical controls, authorization of access, and labeling of specimens, as applicable.

Response: The specimens will be stored in the laboratory located at *1000 Youngs Road, Suite 105, Williamsville NY 14221*and at the CTRC located in 875 Ellicott St. Buffalo NY14203. Samples will be stored in a locked -80º C freezer. All specimens collected at the Glasgow site will be stored in NHS -80º C freezers within the eCRF until shipping to Buffalo (World Courier).

18.7 B. How long will the specimens be stored?

Response: Specimens storage has no expiration date and will be stored for a minimum of 7 years. The researchers may continue to rely on this for future use in research study

18.8 B. Who will have access to the specimens?

Response: Those physicians, nurses, and laboratory staff that are on all documentation for the study will have access to the specimens

18.9 B. Who is responsible for receipt or transmission of the specimens?

Response: Those physicians, nurses, and laboratory staff that are on all documentation for the study will have access to the specimens and can handle transfer of samples. Sample/specimens will transported from University of Glasgow site to the university of Buffalo CTRC site (875 Ellicott St, Buffalo, NY 14203) for storage in -80C until analyzed.

18.10 B. How will the specimens be transported?

Response: All study samples will be centrally stored in UB CTRC site. Therefore, samples collected at the UK site will be transported for analysis and final long-term storage. Transport will be done so using dry ice in a properly labeled Styrofoam container by the laboratory technician. Agreement regarding shipping and receiving the specimens form UK site will be part of the subcontract agreement.

19.0 Provisions to Monitor the Data to Ensure the Safety of Subjects

☐ **N/A:** This study is not enrolling subjects, or is limited to records review procedures only. This section does not apply.

NOTE: Minimal risk studies may be required to monitor subject safety if the research procedures include procedures that present unique risks to subjects that require monitoring. Some examples include: exercising to exertion, or instruments that elicit suicidality or substance abuse behavior. In such cases, N/A is not an acceptable response.

19.1 Describe the plan to periodically evaluate the data collected regarding both harms and benefits to determine whether subjects remain safe.

Response:

An independent Data safety and monitoring board (DSMB) will be established for this trial. The board will consist of independent faculty members from UB and other institutions with sufficient knowledge of the field of type 1 diabetes but not

involved in the study. Data Management team at Robertson Centre will periodically provide the data on which to base regular reports to the Data and Safety Monitoring Board. The actual reports will then be generated by the unblinded statistician. The DSMB looks only at safety endpoints and not at futility. The DSMB will convene every six months to review safety end points, side-effects experienced by study subjects and any adverse events reported. The site PIs, CO-Is and study coordinator(s) will also continuously monitor the data and safety of subjects enrolled in the study. The site PIs and/or the DSMB may recommend to terminate the study or specific aspects of the study if clear harm is observed with one treatment option compared to standard of care or placebo. Specific safety endpoints that will be reviewed include increases in hypoglycemic and ketosis/DKA events.

19.2 Describe what data are reviewed, including safety data, untoward events, and efficacy data.

Response:

Assessment of Efficacy

1- glycemic indices including HbA1c, fructosamine, post-prandial glucose excursions and glucagon. CGM data will be collected as efficacy parameter of glucose control. Total insulin dose will be also documented and analyzed.
2- body weight
3- blood pressure and use of blood pressure medications
4- QOL questionnaires.

The timing of recording of the above efficacy parameters is described in table 1.

Assessment of Safety

The following safety assessments assays and measurement will be reviewed:

1- Vitals, CBC, CMP and markers kidney and liver function will be collected at screening, during and at end of the study as in table 1.
2- Assessment of hypoglycemia: Glucose data will be reviewed from CGM and daily finger stick and during visits.
3- Assessment of ketosis: Ketones levels will be reviewed from daily urinary ketone diaries or blood ketone meters and from urine and blood at the time of each visit
4- Reports of side effects by patients.
5- Insulin dose titrations and pump downloads at each visit

The methods and timing for assessing, recording, and analyzing safety parameters.

The DSMB will convene every six months to assess the side-effects experienced by study subjects and any adverse events reported. The PIs, CO-Is and study coordinator(s) will also continuously monitor the safety of subjects enrolled in the study during their visits and during the safety phone calls.

Procedures for eliciting reports of and for recording and reporting adverse event and inter-current illnesses.

All adverse events will be reported at the time of the visit or by telephone when it occurs. Medical events that occur between the signing of the Informed Consent and the first intake of study drug will be documented in the medical history. Participants will be asked to report any adverse events in general, non-directed questioning. For each AE volunteered by the subject, the investigator will obtain all the information required to complete the adverse event page of the eCRF, in accordance with the guidelines that accompany it. All adverse events, regardless of seriousness, severity, or presumed relationship to study therapy, will be recorded using medical terminology in the source document. Whenever possible, diagnoses will be given when signs and symptoms are due to a common etiology (e.g., cough, runny nose, sneezing, sore throat, and head congestion should be reported as "upper respiratory infection). Investigators will record on the CRF their opinion concerning the relationship of the adverse event to study therapy. The principal investigators assumes responsibility for appropriate reporting of AEs to the regulatory authorities. The investigator will report these events to the appropriate Independent Ethics Committee/Institutional Review Board (IEC/IRB) in accordance with local regulations. All measures required for AE management will be recorded in the source document and reported according to the State University of New York at Buffalo instructions. Non-serious events/problems that impacted on the rights and welfare of the subjects will be reported as they occur or at the time of knowledge or notification.

Definition of adverse events

The International Conference on Harmonization (ICH) Guideline for Good Clinical Practice (GCP) E6(R1) defines an AE as:

Any untoward medical occurrence in a patient or clinical investigation patient administered a pharmaceutical product and which does not necessarily have a causal relationship with this treatment. An AE can therefore be any unfavorable and unintended sign (including an abnormal laboratory finding), symptom, or disease temporally associated with the use of a medicinal product, whether or not considered related to the medicinal product.

The term AE is used to include both serious and non-serious AEs.

Definition of serious adverse events

A serious adverse event is an AE occurring during any study phase (i.e., screening, run-in, treatment, wash-out, follow-up), at any dose of the study drugs that fulfils one or more of the following criteria:

- Results in death
- Is immediately life-threatening
- Requires in-patient hospitalization or prolongation of existing hospitalization
- Results in persistent or significant disability or incapacity
- Is a congenital abnormality or birth defect in offspring of the patient
- Is an important medical event that may jeopardize the patient or may require medical intervention to prevent one of the outcomes listed above.

- TO THE AUTHOR: The causality of SAEs (their relationship to all study treatment/procedures) will be assessed by the investigator(s) and communicated to AstraZeneca. This is requested to be on the AE cover sheet.

The following variables are recommended to be collected for each AE:

- AE (verbatim)
- The date when the AE started and stopped
- Whether the AE is serious or not
- Action taken with regard to IPs
- Administration of treatment for the AE
- Outcome

In addition, the following variables are recommended to be collected for SAEs:

- Date the AE met criteria for SAE
- Date the Investigator became aware of the SAE
- Seriousness criteria fulfilled
- Date of hospitalization
- Date of discharge
- Probable cause of death
- Date of death
- Whether an autopsy was performed
- Causality assessment in relation to study procedure(s)
- Causality assessment in relation to other medication, as explained in Section 1.4.2.
- Description of the SAE

Reporting of serious adverse events

All SAEs will be reported, whether or not considered causally related to the investigational product, or to the study procedure(s). The reporting period for SAEs is the period immediately following the time that written informed consent is obtained throughout the treatment period and including the designated follow-up period as per the study protocol. The investigator is responsible for informing the Ethics Committee and/or the Regulatory Authority of the SAE as per local requirements.

Note that all serious or unexpected adverse events must be reported to AstraZeneca regardless of the country where the study is conducted.

The investigator must inform the FDA, via a MedWatch form, of any serious or unexpected adverse events that occur in accordance with the reporting obligations of 21 CFR 312.32 , and will concurrently forward all such reports to AstraZeneca. A copy of the MedWatch report must be emailed to AstraZeneca (TCS vendor) at the time the event is reported to the FDA. It is the responsibility of the investigator to compile all necessary information and ensure that the FDA receives a report according to the FDA reporting requirement timelines and to ensure that these reports are also submitted to AstraZeneca at the same time.

A cover page should accompany the MedWatch form indicating the following:

- “Notification from an Investigator Sponsored Study”

- The investigator IND number assigned by the FDA
- The investigator's name and address
- The trial name/title and AstraZeneca ISS reference number (ESR-##-#####)

Investigator must also indicate, either in the SAE report or the cover page, the causality of events in relation to all study medications and if the SAE is related to disease progression.

Send SAE report and accompanying cover page by way of email to AstraZeneca's designated mailbox: AEMailboxClinicalTrialTCS@astrazeneca.com

If a non-serious AE becomes serious, this and other relevant follow-up information must also be provided to AstraZeneca and the FDA.

Serious adverse events that do not require expedited reporting to the FDA still need to be reported to AstraZeneca preferably using the MedDRA coding language for serious adverse events.

The investigators will either provide a copy of the randomization code/ code break information or unblind those SAEs which require expedited reporting

Overdose

An overdose is defined as a patient receiving a dose of IP in excess of that specified in the Investigator's Brochure, unless otherwise specified in this protocol.

Any overdose of a study patient with the investigational product, with or without associated AEs/SAEs, is required to be reported within 24 hours of knowledge of the event to AstraZeneca Patient Safety using the designated Safety e-mailbox (see Section 1.5 or contact information). If the overdose results in an AE, the AE must also be recorded as an AE. Overdose does not automatically make an AE serious, but if the consequences of the overdose are serious, for example death or hospitalization, the event is serious and must be recorded and reported as an SAE (see Section 1.3). Any specific details around overdose for combination or comparator agents will also be provided as applicable. The investigator will use clinical judgment to treat any overdose.

Hepatic function abnormality

Hepatic function abnormality that fulfills the biochemical criteria of a potential Hy's Law case in a study patient, with or without associated clinical manifestations, is recommended to be reported as "hepatic function abnormal" within 24 hours of knowledge of the event to AstraZeneca Patient Safety using the designated Safety e-mailbox (see Section 1.3), unless a definitive underlying diagnosis for the abnormality (e.g., cholelithiasis or bile duct obstruction) that is unrelated to investigational product has been confirmed. The criteria for a potential Hy's Law case is Aspartate Aminotransferase (AST) or Alanine Aminotransferase (ALT) ≥3x Upper Limit of Normal (ULN) together with Total Bilirubin (TBL) ≥2xULN at any point during the study following the start of study medication irrespective of an increase in Alkaline Phosphatase (ALP).

Pregnancy

If a patient becomes pregnant during the course of the study, the IPs should be discontinued immediately.

Pregnancy itself is not regarded as an AE unless there is a suspicion that the IP under study may have interfered with the effectiveness of a contraceptive medication. Congenital abnormalities or birth defects and spontaneous miscarriages should be reported and handled as SAEs. Elective abortions without complications should not be handled as AEs. The outcome of all pregnancies (spontaneous miscarriage, elective termination, ectopic pregnancy, normal birth, or congenital abnormality) should be followed up and documented even if the patient was discontinued from the study.

If any pregnancy occurs in the course of the study, then the investigator should inform the appropriate AstraZeneca representatives within 1 day, i.e., immediately, but no later than 24 hours of when he or she becomes aware of it.

The investigator will ensure that all relevant information is provided to the AstraZeneca Patient Safety data entry site within 1 to 5 calendar days for SAEs and within 30 days for all other pregnancies.

The same timelines apply when outcome information is available.

19.3 Describe any safety endpoints.

Response: These specific Safety endpoints will be collected:

1- Frequency and severity of hypoglycemia: Hypoglycemia will be assessed throughout the study via multiple daily finger stick glucose measurements, CGM data collected 3 times (about 3 weeks each) during the study and from patient reports during visits and phone calls. Hypoglycemic events will be characterized as in the experts consensus published in *Diabetes Care (2017 Dec; 40(12): 1622-1630):*

- Level 1: Glucose < 70 mg/dL (3.9 mmol/L) and Glucose ≥ 54 mg/dL (3.0 mmol/L)
- Level 2: Glucose < 54 mg/dL (3.0 mmol/L)
- Level 3: A severe event characterized by altered mental and/or physical status requiring assistance.

2- Ketones levels: Urinary ketones measured daily at home and logged in the ketones logbook. Blood and urinary ketones measured during each visit. Ketone levels will be characterized as normal, ketosis and severe ketosis:

-Normal blood ketones <0.6mM or urinary ketones of trace to small (≥+1)

-Ketosis: Blood ketones >0.6mM <2.5mM or urinary ketones of moderate to high (+2 and +3)

-Severe ketosis Blood ketones >2.5mM or urinary ketones of too high (+4)

3- Diabetes ketoacidosis episodes: any confirmed DKA episode (regardless of hospitalization) defined as:

- Elevated serum or urine ketones (greater than the upper limit of the normal range), and
- Serum bicarbonate < 15 mmol/L or Blood pH < 7.3

4- Low blood pressure: <90mmHg SBP and <50 mmHg DBP): Patients will be instructed to measure blood pressure daily and it will be will be assessed in each visit.
5- Nausea, vomiting and other GI side effects. Data will be collected by patients' safety reports, during visits and phone calls.
6- Genital mycotic infections or UTIs
7- Changes in liver and kidney function: CMP and kidney and liver profiles will be assessed as indicated in table 1.

19.4 Describe how the safety information will be collected (e.g., with case report forms, at study visits, by telephone calls with participants).

Response: The safety information will be collected at the time of the visit, and or during preplanned telephone calls with the participant or patient self-reporting (including glucose, food intake and ketones diaries). Data will be entered into eCRFs

19.5 Describe the frequency of safety data collection.

Response: Safety data collection will be done at all study visits and during preplanned phone calls as in table 1. The patient, however, will be asked to report any adverse event or safety related information via phone as soon as it occurs and it will be reviewed the same day.

19.6 Describe who will review the safety data.

Response: DSMB established for this study will review unblended safety data provided by the data management system every 6 months. Additionally, the PIs and coinvestigators will review safety data (listed above) at the completion of each visits by each subject and review any report of side effects or adverse events as they are presented by the patients. The investigators will also review collective blinded data every 3 months to assess the safety and any potential risks to the participants. Furthermore the investigators will assess other risks including the physical, psychological, social, legal and economic harm to these patients. The investigators will carefully watch for any invasion of privacy and breach of confidentiality. The principal investigator will be sharing the results of safety analysis of the study and serious adverse events with the local IRB, DSMB, JDRF, AZ and the FDA. The study groups will remain blinded. If there are a serious safety concerns or an emergency, then, and based on principal investigator discretion, study randomization center in Glasgow University or the unblended statistician will provide information about group assignment of the patient(s) involved. Un-blinded data will be reviewed by the principal investigator and co-investigators and decision to complete, withdraw or adjust dose of drug(s) will be made, documented and shared with IRB and DSMB.

19.7 Describe the frequency or periodicity of review of cumulative safety data.

Response: Safety data will be reviewed every 6 months by DSMB (un-blinded), every 3 months by PIs and CO-Is (blinded) and at every visit by study team.

19.8 Describe the statistical tests for analyzing the safety data to determine whether harm is occurring.

Response: N/A

19.9 Describe any conditions that trigger an immediate suspension of the research.

Response: No interim analysis is planned for efficacy so termination of study will be related to safety. Significant increases in severe hypoglycemic or DKA events as reviewed by DSMB are possible causes that might trigger termination of study.

20.0 Withdrawal of Subjects

☐ **N/A:** This study is not enrolling subjects. This section does not apply.

20.1 Describe ***anticipated*** *circumstances under which subjects may be withdrawn from the research without their consent.*

1. The site PI can stop investigational study treatment or remove participants from the trial without participants approval if for any reason he/she feels is appropriate, including: severe side effect, injury or medical condition which may place the patient at risk of further complications if he/she continue to participate, failure to take the medication as instructed, failure to keep scheduled appointments, cancellation of the study by the sponsor, IRB, FDA or other administrative reasons.
2. If any of the participants become pregnant during the period of study, they will discontinue study treatment.
3. Participants who experience persistent severe nausea or vomiting after starting semaglutide which is not managed by dose reduction will discontinue the investigational study treatment. Those with mild or moderate nausea will continue for at least one more week. If they continue to experience significant nausea (defined as interfering with normal activities), dose will be reduced. If they continue to have these side effects, they will discontinue study medication(s).
4. Developing other conditions included in the exclusion criteria
5. Change in method of insulin delivery between insulin pumps to MDI or vice versa during the study unless pump treatment interruption is temporary for a period of at most 2 weeks. Also, patients who upgrade to the new “artificial pancreas” pump (Medtronic’s MiniMed 670G hybrid) during the study will be withdrawn.
6. Hypoglycemia: Two episodes of severe hypoglycemia (requiring others help).

7. Ketosis (with no other identifiable precipitating factors): Persistent (1week) asymptomatic or symptomatic and unmanaged (using STICH protocol) blood ketone levels >1.5 mM (or ≥+2 in urine).
8. Persistent (>1 week) total insulin dose reduction of >20% from baseline AND total insulin dose <0.4U/Kg.
9. DKA: Two episode of DKA (with no identifiable precipitating factors) as defined by the expert consensus statement (*Diabetes Care (2017 Dec; 40(12): 1622-1630)*)
10. Two episodes of UTI.
11. Two episodes of mycotic genital infection.
12. Significant persistent change in eGFR > 15 ml/min
13. Pancreatitis: One episode of pancreatitis.
14. Other persistent side effects specific to study drugs

20.2 Describe any procedures for orderly termination.

NOTE: Examples may include return of study drug, exit interview with clinician. Include whether additional follow up is recommended for safety reasons for physical or emotional health.

Response: Participation in this study is voluntary. Participants have the right to refuse to participate or to withdraw from participation at any time for any reason. Refusal to participate or to withdraw from the study will involve no penalty or loss of entitled benefits, nor affect ongoing medical care. The site PI can stop investigational study treatment or remove participants from the trial without participants approval. If a patient withdraws consent and decide not to participate in the study, she/he will be asked to undergo a final study visit for his/her safety. If a subject withdraws from the research, the data collected to that point will be used toward the research finding. If applicable the subject will have to bring back any unused research drug and or devices.

20.3 Describe procedures that will be followed when subjects withdraw from the research, including retention of already collected data, and partial withdrawal from procedures with continued data collection, as applicable.

Response: We will use the data collected up to the point of stopping the study. No further information will be collected. Patients who stop study treatment and are interested in continuing in the study will be followed up until the end of the study for the primary end point and select secondary endpoints based on the Intent-To-Treat (ITT) analysis model. Withdrawn participants will not be replaced. The study sample size calculation includes an additional 15% subjects to compensate for drop out.

21.0 Risks to Subjects

21.1 List the reasonably foreseeable risks, discomforts, hazards, or inconveniences to the subjects related to their participation in the research. Consider physical, psychological, social, legal, and economic risks. Include a description of the probability, magnitude, duration, and reversibility of the risks.

NOTE: Breach of confidentiality is always a risk for identifiable subject data.

Response:

- Risks associated with intravenous (IV) line: All participants will be informed of the potential complications of an intravenous (IV) line, which include mild bruising at the site of IV line, usually resolving in a few days. They will also be informed about the possibility of infiltration of the IV line at the time of performing blood draws or during the meal challenge test in which case another IV line at a different site will be required (with risk of bruising at more than one site).
- Risks associated with 24 hour Ambulatory BP monitoring device: The BP cuff inflates every 15 minutes to record blood pressure over 24-hours. Participants will experience a pressure-like sensation over the arm where 24 hour BP device is worn at the time of the BP being measured due to the inflation of the cuff and release of this pressure at the time of deflation of the BP cuff. They will be informed to remove the cuff after 24 hours. If worn for longer periods they may experience mild bruising over the arm and some swelling, usually resolving 1-2 days after discontinuation.
- Risks associated with fat tissue aspiration: will probably lead to a bruise at the site of aspiration. The site may be mildly painful for 1-2 weeks. Participants are advised to call their study site staff if they have more severe pain or swelling at the site after the procedure. Rarely there are side effects such as low BP or heart rate and/or allergic reaction to local anesthetic including swelling of the throat.
- All subjects will be referred to semaglutide and dapagliflozin labeling safety information, and will be given a copy of the package insert of the medication. Discussion of the potential side effects and the different warning and precautions will take place at the time of informed consent. Patients will be informed they must not discontinue their insulin during the study.
- Potential risk of insulin, GLP-1 RA and SGLT-2 inhibitors use in type 1 diabetes patients is hypoglycemia. The combination therapy can possibly increase the magnitude and frequency of this risk.
- Potential side effects of GLP-1 RA and SGLT-2 inhibitors also include hypotension, nausea and other GI side effects, polydipsia, polyuria, dehydration, ketosis, and reduced glomerular filtration rate (GFR).
- In rodents, semaglutide causes dose-dependent and treatment-duration-dependent thyroid C-cell tumors at clinically relevant exposures. However, it received FDA-approval for the treatment of type 2 diabetes in 2017 following large Phase 3 trials including the cardiovascular safety trial SUSTAIN-6.
- On the basis of the above rodent studies, semaglutide is contraindicated in those with a personal or family history of medullary thyroid carcinoma and in patients with Multiple Endocrine Neoplasia syndrome type 2 (MEN 2). However, following review of human data with GLP-1RAs, FDA does not require or suggest routine monitoring of serum calcitonin/ thyroid ultrasound in people treated with semaglutide.
- Injection-site reactions: Serious injection-site reactions, with or without bumps (nodules), have happened in some people who use GLP-1RAs but are less frequent with semaglutide. Injection-site reactions have rarely required surgical

intervention. Participants will be instructed to report any injection site reactions including nodules to study investigators.

- Kidney injury: GLP-1 RA might cause acute kidney injury in patients without underlying renal disease especially in patient who had experienced nausea, vomiting, diarrhea or dehydration. Dapagliflozin also has warnings and precautions against acute kidney injury and impairment in renal function.
- As mentioned above, there is a risk of ketoacidosis, as shown by some studies and by our data, when SGLT2 inhibitors are used in type 1 diabetes. In our own preliminary studies on triple therapy, we observed euglycemic ketoacidosis in participants whose insulin doses had been reduced by > 20% of baseline levels or in patients who are not adequately insulinzed. Thus, this adverse effect is largely attributable to insulinopenia as well as anti-insulin effects of SGLT2 inhibitors at the hepatic level (e.g. increased gluconeogenesis).
- The following other known adverse effects of SGLT-2 inhibitors will be discussed with participants when obtaining informed consent:

  1. Risk of dizziness/ symptomatic hypotension: can occur soon after initiation due to intravascular volume contraction.
  2. Impaired kidney function: As SGLT-2 inhibitors increase serum creatinine and decrease eGFR, this will be monitored closely.
  3. Genital mycotic infections: SGLT-2 inhibitors increase the risk of genital mycotic infections, particularly in women. Those with a prior history are more susceptible. Following topical treatment with anti-fungal medications they rarely re-occur.
  4. Bladder Cancer - Across 22 clinical studies, newly diagnosed cases of bladder cancer were reported in 10/6045 patients (0.17%) treated with Dapagliflozin and 1/3512 patient (0.03%) treated with placebo. After excluding patients in whom exposure to study drug was less than one year at the time of diagnosis, there were 4 cases of bladder cancer with dapagliflozin and no cases with placebo i.e. too few cases to determine causality.
  5. The dapagliflozin tablets and matching placebo contain lactose, which may cause discomfort in lactose-intolerant individuals.

- Severe allergic reaction (anaphylaxis) to study drugs or placebo ingredients or other interventions may accrue in rare cases.
- There might be other unforeseen risks associated with the use of these agents in type 1 diabetes and additional unforeseen risks associated with the combination therapy.
- There is always the risk of breach of confidentiality where patients' identifiable data is compromised.

21.2 Describe procedures performed to lessen the probability or magnitude of risks, including procedures being performed to monitor subjects for safety.

Response:

The following are procedures to lessen the probability and magnitude of risks listed above:

1- Hypoglycemia:

- Patients will be instructed to measure glucose at least 4 times a day (before meals and at bedtime) through-out the study and record all reading.
- Insulin dose adjustments will be done at every visit to avoid hypoglycemia. However, there will be no preemptive insulin dose reduction at start of treatment since all patients will have HbA1c >7.5% and the risk of hypoglycemia will be minimal when semaglutide is added.
- Daily fingerstick glucose, CGM and insulin pump data will be reviewed by the investigators for optimal titration of insulin dose to target glucose levels between 90-180mg/dl.
- In general, insulin dose adjustments should not be >10% from current total insulin dose (or 5% if HbA1c is <7%) or >20% from baseline total insulin dose at week 0 and then at week 26.
- Patients will be trained in CARB counting to avoid inaccurate insulin dose administration.
- Patients will be trained to recognize symptoms of hypoglycemia and how to manage it at home. These symptoms include feeling shaky, being nervous or anxious, sweating, chills and clamminess, confusion, fast heartbeat, feeling lightheaded or dizzy, hunger, nausea and color draining from the skin (pallor) among others. They will be asked to call the research office or the on call clinical services.
- When patients feel symptoms of hypoglycemia they will be asked to measure blood glucose and if <70mg/dl they will be instructed to consume carbohydrate or 4 tablets of glucose (4gm each). If after 15 min hypoglycemia continues, repeat carb intake until sugar levels are above 70 mg/dl. If sugar levels are below 55mg/dl, glucagon injection is permitted as rescue medication for severe hypoglycemia.
- Patients will be instructed to inform people who they are in frequent contact with (for example, friends, family members, and coworkers) on how to give glucagon to treat severe hypoglycemia. If patient have needed glucagon previously, it should be documented during screening visit so more emphasis on ways to prevent severe hypoglycemia in the future can be discussed.
- Patients/family will be instructed not to hesitate to call 911 if someone is unconscious and glucagon is not available or someone does not know how to use it and not to inject insulin (it will lower their blood glucose even more) or provide food or fluids (they can choke). They will be asked to document all details of the incident and report to research staff as soon as possible.
- Study medications dose will be reduced in patients who continue to have more than 2 measurements of glucose levels in the hypoglycemic range (<70mg/dl, regardless of symptoms) after insulin dose is reduced by the maximum allowed in this study.

2- Ketosis and Ketoacidosis:

- Patients will be instructed to measure ketone bodies in urine samples daily starting at 3 days before week 26 with Ketostix (Bayer) and record it in diary provided. Ketones (blood and urine) will be measured at every visit. If ketone bodies in urine measure +1 or greater (>1.5mM in urine), patients are required to notify the research center. They will be monitored closely for insulin dose,

carb intake, ketones and blood sugar and dapagliflozin/semaglutide can be temporarily discontinued, if necessary, for 1-2 weeks.

- Patients will be provided training to recognize the signs and symptoms of DKA and to self-test urine ketones on a PRN basis (in addition to once daily) if they develop any symptoms consistent with DKA, including nausea, vomiting, fatigue, confusion, or shortness of breath. This should be reported regardless of blood sugar levels since in type 1 diabetes on SGLT-2 inhibitors, DKA can occur even in normal glucose levels.
- Insulin dose changes should not be greater than 10% (5% if HbA1c is<7%) from current levels and not reduced by more than 20% from baseline levels at 0 weeks and then at 26 weeks.
- Patients requiring an insulin dose reduction > 20% AND their total insulin dose is <0.4U/Kg will be monitored closely for insulin dose, carb intake, ketones and blood sugar and dapagliflozin/semaglutide can be temporarily discontinued, if necessary, for 1-2 weeks. If insulin requirements remain law, patient will be withdrawn from study.
- We shall not increase dose of dapagliflozin in patients who exhibit significant ketonuria (+2 or more) with the lower dose.
- Urinary ketones (and blood ketones if patients have a dual glucose and ketone meter) will be monitored daily and reported. Dapagliflozin will be temporarily (1 week) discontinued and STICH protocol (STop SGLT-2i, give Insulin bolus, Carbohydrate, and Hydration) activated in events of DKA or if based on investigator discretion if blood ketone levels are persistently (1 week) >1.5 mM (+2 in or more in urine). Dapagliflozin may also be temporarily stopped in clinical situations known to predispose to ketoacidosis (e.g. prolonged fasting due to acute illness or surgery). Patients will be instructed to stay hydrated and not reduce their caloric intake (especially carbohydrates) or start new dietary systems (especially ketogenic diets) during the study.
- If a patient develops symptoms of DKA or ketone levels exceed the allowed limit in this study (persistent (1 week) blood ketones >1.5 mM or +2 in or more in urine), STICH protocol (STop SGLT-2i, give Insulin bolus, Carbohydrate, and Hydration) will be activated. They could be managed for up to a week. If symptoms and ketone levels persist over 1 week, patients will be withdrawn form study. Insulin infusions may be administered by ER or hospital if patients develop DKA. They will be asked to document all details of the incident and report to research staff as soon as possible.

3- Risk of acute kidney injury or renal function impairment: CBC, CMP, including kidney function test and urinary markers will be performed at screening, during and at end of the study (table 1). Additional testing will be performed in patients who suffer from sever nausea or GI symptoms or hypovolemic and during the up titration of investigation drugs. If acute kidney injury occurs dapagliflozin will be discontinued immediately. To lessen the chance of this risk, patients will eGFR<60 ml/min/1.73m^2 will be excluded. Patients will also discontinue study medications if eGFR falls by >15 ml/min in 2 repeated measurements. Also other patients with factors that may predispose patients to acute kidney injury e.g. hypovolemia, chronic renal insufficiency, congestive heart failure will be

excluded. Patients on ACEI, ARB, NSAID will be monitored closely.. Dapagliflozin will be temporarily discontinued in any setting of reduced oral intake such as acute illness or fasting or fluid losses such as gastrointestinal illnesses, or excessive heat exposure.

4- Injection site reactions, mycotic infections and complications of IV and biopsy collection will also be monitored, Physical examination will be performed at screening 26, and 52 weeks visits. Safety vital assessments will be performed at every visit. Pregnancy will be tested at screening, 0, 4 13, 26, 39, and 52 weeks. Phone calls will be made for safety and compliance at least 7 time during the study. The patients, however, will be instructed to report any adverse event or safety related information via phone as soon as it occurs. All safety data will be reviewed by the PI or sub-investigators within 1-2 days of collection.

5- Epinephrine is the primary treatment for anaphylaxis with no absolute contraindication to its use. Our site and staff are trained and equipped to assist patients with such reactions. Severe conditions that are not satisfactory managed in site will be transferred to the hospital emergency department for further assessment and help.

21.3 If applicable, indicate ***which procedures*** *may have risks to the subjects that are currently unforeseeable.*

Response:

There might be additional adverse effects of dapagliflozin not currently known or those unique to their use in Type 1 diabetes. There is no previous studies on the use of semaglutide in type 1 diabetes; therefore, additional unforeseen risk may be encountered in this study. Additionally, unforeseen risks might be associated with the combination therapy that might involve unknown drug-drug interactions.

21.4 If applicable, indicate which research procedures may have risks to an embryo or fetus should the subject be or become pregnant.

Response: Not applicable, we will not be enrolling pregnant participants or who may become pregnant or with in child baring years without signing the consent stating they will use at least two forms of birth control. If a participant becomes pregnant they will be withdrawn from the study immediately. They will be followed until term and any effects on the newborn will be documented.

21.5 If applicable, describe risks to others who are not subjects.

Response: We do not anticipate any additional risk to others not participating in this study.

22.0 Potential Benefits to Subjects

22.1 Describe the potential benefits that individual subjects may experience by taking part in the research. Include the probability, magnitude, and duration of the potential benefits. Indicate if there is no direct benefit.

*NOTE: Compensation **cannot** be stated as a benefit.*

Response: The investigational drugs used in this study are already approved as antidiabetic agents for treating type 2 diabetes. Both agents have demonstrated significant improvement in HbA1c, weight and blood pressure. It is our expectation that patients with type 1 diabetes will experience similar benefits compared to those who are treated with insulin alone. The magnitude and duration of the benefits and the safety profile may however differ and are the focus of this investigation.

23.0 Compensation for Research-Related Injury

☐ **N/A:** The research procedures for this study do not present risk of research related injury (e.g. survey studies, records review studies). This section does not apply.

*23.1 **If the research procedures carry a risk of research related injury,** describe the available compensation to subjects in the event that such injury should occur.*

Response: Routinely, Buffalo General Hospital, Erie County Medical Center, and/or the University at Buffalo, State University of New York, its agents, or its employees do not compensate for or provide free medical care for human subjects/participants in the event that any injury results from participation in a human research project. In the unlikely event that they become ill or injured as a direct result of participating in this study, they may receive medical care. Your study doctor will explain the treatment options to you and tell you where you can get treatment. Generally, this care will be billed to you, your insurance or other third party. Additionally, AstraZeneca shall reimburse the institution for the direct, reasonable, and necessary medical expenses incurred by the institution for the treatment of any personal injury that is a direct result of adverse drug experiences to subjects resulting directly from any manufacturing defects in the study drug to which the subjects would not have been exposed but for their participation in the study if (i) the institutional parties have complied with this agreement, the protocol and any written instructions of AstraZeneca concerning the study drug and (ii) all the requirements of informed consent have been complied with. AstraZeneca will not provide compensation for lost wages or for any other damages, expenses or losses, or for medical expenses have have been covered by a subject’s medical or other insurance.

23.2 Provide a copy of contract language, if any, relevant to compensation for research related injury.

NOTE: If the contract is not yet approved at the time of this submission, submit the current version here. If the contract is later approved with ***different language regarding research related injury****, you must modify your response here and submit an amendment to the IRB for review and approval.*

Response: AstraZeneca shall reimburse the institution for the direct, reasonable, and necessary medical expenses incurred by the institution for the treatment of any personal injury that is a direct result of adverse drug experiences to subjects resulting directly from any manufacturing defects in the study drug to which the subjects would not have been exposed but for their participation in the study if (i) the institutional parties have complied with this agreement, the protocol and any written instructions of AstraZeneca concerning the study drug and (ii) all the requirements of informed consent have been complied with. AstraZeneca will not provide compensation for lost wages or for any other damages, expenses or losses, or for medical expenses have have been covered by a subject's medical or other insurance.

24.0 Economic Burden to Subjects

24.1 Describe any costs that subjects may be responsible for because of participation in the research.

NOTE: Some examples include transportation or parking.

Response: All research expenses will be covered by the study. The background use of insulin is considered as part of standard of care is the responsibility of the patients and his/her insurance. Participants will not be subjected to any other out of pocket cost.

☐ **N/A:** This study is not enrolling subjects, or is limited to records review procedures only. This section does not apply.

25.0 Compensation for Participation

25.1 Describe the amount and timing of any compensation to subjects, including monetary, course credit, or gift card compensation.

Response:
Visit schedule:

- Screen: $0
- Visit 1 (Week -3): Insulin adjustment: $10
- Visit 2 (Week -1): Meal ($75), insulin adjustment, and 24hr blood pressure monitor ($50): $125*
- Visit 3 (Week 0): 24 hr urine ($15), blood draw ($25), fat biopsy ($75), insulin adjustment, questionnaire ($10): $125
- Visit 4 (Week 1): Insulin adjustment: $10
- Visit 5 (Week 2): Insulin adjustment: $10
- Visit 6 (Week 4): Insulin adjustment: $10
- Visit 7 (Week 13): 24hr urine ($15), blood draw ($25), insulin adjustment, 24hr bp monitor ($50): $90*
- Visit 8 (Week 23): Insulin adjustment: $10

- Visit 9 (Week 25): Insulin adjustment, 24bp monitor ($50), Meal ($75): $125*
- Visit 10 (Week 26): Insulin adjustment, 24hr urine ($15), blood draw ($25), fat biopsy ($75), questionnaire ($10): $125*
- Visit 11 (Week 27): Insulin adjustment: $10
- Visit 12 (Weak 31): Insulin adjustment, blood draw: $25
- Visit 13 (Week 39): Insulin adjustment, 24hr urine ($15), blood draw ($25), 24hr bp monitor ($50): $90*
- Visit 14 (Week 49): Insulin adjustment: $10
- Visit 15 (Week 51): Insulin adjustment, 24bp monitor ($50), meal ($75): $125*
- Visit 16 (Week 52): Insulin adjustment, 24hr urine ($15), blood draw ($25), questionnaire ($10), fat biopsy ($75):$125*
  *Total compensation for all completed visits: up to $1,025.00. For those not wishing to participate in the optional blood pressure monitoring and/or fat biopsy procedure, the compensation will decrease by $250 for not completing the bp monitoring, and by yet another $225.00 for not completing the fat biopsies.

☐ **N/A:** This study is not enrolling subjects, or is limited to records review procedures only. This section does not apply.

☐ **N/A:** There is no compensation for participation. This section does not apply.

26.0 Consent Process

26.1 Indicate whether you will be obtaining consent.

NOTE: This does not refer to consent documentation, but rather whether you will be obtaining permission from subjects to participate in a research study. Consent documentation is addressed in Section 27.0.

☒ ***Yes*** *(If yes, Provide responses to each question in this Section)*
☐ ***No*** *(If no, Skip to Section 27.0)*

26.2 Describe where the consent process will take place. Include steps to maximize subjects' privacy.

Response: All participants will come to the research department to be consented. Participants will be placed in a private, screened off area and or room where they can review the consent. Participant questions and or concerns will be address with a member of the study team or research doctor if applicable. The research coordinator will discuss in length the participants requests for privacy of their PHI.

26.3 Describe how you will ensure that subjects are provided with a sufficient period of time to consider taking part in the research study.

NOTE: It is always a requirement that a prospective subject is given sufficient time to have their questions answered and consider their participation. See "SOP: Informed Consent Process for Research (HRP-090)" Sections 5.5 and 5.6.

Response: participants will be made aware that participating in research is completely voluntary, and they may withdraw at any time with no consequence to their routine clinic care. If the patients requires time to decide and or discuss partaking in a research study, the subject will be given said time.

26.4 Describe any process to ensure ongoing consent, defined as a subject's willingness to continue participation for the duration of the research study.

Response: The research coordinator and study team are available to answer any question or concerns with the patient during the duration of the research trial. At each study visit, the patient is asked a series of questions to ensure they are on task with the study visits and feel comfortable. Upon departing from their study visit, the patients are told of their next visit and given detail instruction for their next visit. If study is revised or amendment or new information becomes available about drug safety that may affect patients participation, the patient may be re-consented to ensure patient ongoing consent

26.5 Indicate whether you will be following "SOP: Informed Consent Process for Research (HRP-090)." If not, or if there are any exceptions or additional details to what is covered in the SOP, describe:

- *The role of the individuals listed in the application who are involved in the consent process*
- *The time that will be devoted to the consent discussion*
- *Steps that will be taken to minimize the possibility of coercion or undue influence*
- *Steps that will be taken to ensure the subjects' understanding*

Response:

☒ We have reviewed and will be following "SOP: Informed Consent Process for Research (HRP-090)."

Non-English Speaking Subjects

☒ **N/A:** This study will not enroll Non-English speaking subjects. *(Skip to Section 26.8)*

26.6 Indicate which language(s) other than English are likely to be spoken/understood by your prospective study population or their legally authorized representatives.

NOTE: The response to this Section should correspond with your response to Section 6.4 of this protocol.

Response:

26.7 *If subjects who do not speak English will be enrolled, describe the process to ensure that the oral and written information provided to those subjects will be in that language. Indicate the language that will be used by those obtaining consent.*

NOTE: Guidance is provided on "SOP: Informed Consent Process for Research (HRP-090)."

Response:

Cognitively Impaired Adults

☒ **N/A**: This study will not enroll cognitively impaired adults.
(Skip to Section 26.9)

26.8 *Describe the process to determine whether an individual is capable of consent.*

Response:

Adults Unable to Consent

☒ **N/A**: This study will not enroll adults unable to consent.
(*Skip to Section 26.13)*

When a person is not capable of consent due to cognitive impairment, a legally authorized representative should be used to provide consent (Sections 26.9 and 26.10) ***and, where possible, assent of the individual should also be solicited*** *(Sections 26.11 and 26.12).*

26.9 *Describe how you will identify a Legally Authorized Representative (LAR). Indicate that you have reviewed the "SOP: Legally Authorized Representatives, Children, and Guardians (HRP-013)" for research in New York State.*

NOTE: Examples of acceptable response includes: verifying the electronic medical record to determine if an LAR is recorded.

Response:

☐

26.10 ***For research conducted outside of New York State****, provide information that describes which individuals are authorized under applicable law to consent on behalf of a prospective subject to their participation in the research. One method of obtaining this information is to have a legal counsel or authority review your protocol along with the definition of "legally authorized representative" in "SOP: Legally Authorized Representatives, Children, and Guardians (HRP-013)."*

Response:

26.11 Describe the process for ***assent of the adults****:*

- *Indicate whether assent will be obtained from all, some, or none of the subjects. If some, indicate which adults will be required to assent and which will not.*

  Response:

- *If assent will not be obtained from some or all subjects, provide an explanation of why not.*

  Response:

26.12 Describe whether ***assent of the adult*** *subjects will be documented and the process to document assent.*

NOTE: The IRB allows the person obtaining assent to document assent on the consent document using the "Template Consent Document (HRP-502)" Signature Block for Assent of Adults who are Legally Unable to Consent.

Response:

Subjects who are not yet Adults (Infants, Children, and Teenagers)

☒ **N/A**: This study will not enroll subjects who are not yet adults. *(Skip to Section 27.0)*

26.13 Describe the criteria that will be used to determine ***whether a prospective subject has not attained the legal age for consent to treatments or procedures involved in the research*** *under the applicable law of the jurisdiction in which the research will be conducted* ***(e.g., individuals under the age of 18 years)****. For research conducted in NYS, review "SOP: Legally Authorized Representatives, Children, and Guardians (HRP-013)" to be aware of which individuals in the state meet the definition of "children."*

NOTE: Examples of acceptable responses include: verification via electronic medical record, driver's license or state-issued ID, screening questionnaire.

Response:

26.14 ***For research conducted outside of New York State****, provide information that describes which persons have not attained the legal age for consent to treatments or procedures involved the research, under the applicable law of the jurisdiction in which research will be conducted. One method of obtaining this information is to have a legal counsel or authority review your protocol along the definition of "children" in "SOP: Legally Authorized Representatives, Children, and Guardians (HRP-013)."*

Response:

26.15 Describe whether parental permission will be obtained from:

Response: N/A

☐ One parent even if the other parent is alive, known, competent, reasonably available, and shares legal responsibility for the care and custody of the child.

☐ Both parents unless one parent is deceased, unknown, incompetent, or not reasonably available, or when only one parent has legal responsibility for the care and custody of the child.

☐ Parent permission will not be obtained. A waiver of parent permission is being requested.

NOTE: The requirement for parent permission is a protocol-specific determination made by the IRB based on the risk level of the research. For guidance, review the "CHECKLIST: Children (HRP-416)."

26.16 Describe whether permission will be obtained from individuals ***other than parents****, and if so, who will be allowed to provide permission. Describe your procedure for determining an individual's authority to consent to the child's general medical care.*

Response:

26.17 Indicate whether assent will be obtained from all, some, or none of the ***children****. If assent will be obtained from some children, indicate which children will be required to assent.*

Response:

26.18 When assent of children is obtained, describe how it will be documented.

Response:

27.0 Waiver or Alteration of Consent Process

Consent will not be obtained, required information will not be disclosed, or the research involves deception.

☒ **N/A:** A waiver or alteration of consent is not being requested.

27.1 If the research involves a waiver or alteration of the consent process, please review the "CHECKLIST: Waiver or Alteration of Consent Process (HRP-410)" to ensure that you have provided sufficient information for the IRB to make the determination that a waiver or alteration can be granted.

NOTE: For records review studies, the first set of criteria on the "CHECKLIST: Waiver or Alteration of Consent Process (HRP-410)" applies.

Response:

27.2 If the research involves a waiver of the consent process for planned emergency research, please review the "CHECKLIST: Waiver of Consent for Emergency Research (HRP-419)" to ensure you have provided sufficient information for the IRB to make these determinations. Provide any additional information necessary here:

Response:

28.0 Process to Document Consent

☐ **N/A:** A Waiver of Consent is being requested.
(Skip to Section 29.0)

28.1 Indicate whether you will be following "SOP: Written Documentation of Consent (HRP-091)." If not or if there are any exceptions, describe whether and how consent of the subject will be obtained including whether or not it will be documented in writing.

NOTE: If your research presents no more than minimal risk of harm to subjects and involves no procedures for which written documentation of consent is normally required outside of the research context, the IRB will generally waive the requirement to obtain written documentation of consent. This is sometimes referred to as 'verbal consent.' Review "CHECKLIST: Waiver of Written Documentation of Consent (HRP-411)" to ensure that you have provided sufficient information.

If you will document consent in writing, attach a consent document with your submission. You may use "TEMPLATE CONSENT DOCUMENT (HRP-502)". If you will obtain consent, but not document consent in writing, attach the script of the information to be provided orally or in writing (i.e. consent script or Information Sheet).

Response:

☒ We will be following "SOP: Written Documentation of Consent" (HRP-091).

29.0 Multi-Site Research (Multisite/Multicenter Only)

☐ **N/A:** This study is not an investigator-initiated multi-site study. This section does not apply.

29.1 If this is a multi-site study ***where you are the lead investigator****, describe the processes to ensure communication among sites, such as:*

- *All sites have the most current version of the IRB documents, including the protocol, consent document, and HIPAA authorization.*
- *All required approvals have been obtained at each site (including approval by the site's IRB of record).*
- *All modifications have been communicated to sites, and approved (including approval by the site's IRB of record) before the modification is implemented.*
- *All engaged participating sites will safeguard data as required by local information security policies.*
- *All local site investigators conduct the study appropriately.*
- *All non-compliance with the study protocol or applicable requirements will be reported in accordance with local policy.*

Response:

29.2 Describe the method for communicating to engaged participating sites:

- *Problems*
- *Interim results*
- *Study closure*

Response:

29.3 Indicate the total number of subjects that will be enrolled or records that will be reviewed across all sites.

Response: 114

29.4 If this is a multicenter study for which UB will serve as the IRB of record, and subjects will be recruited by methods not under the control of the local site (e.g., call centers, national advertisements) describe those methods.

Response: N/A

30.0 Banking Data or Specimens for Future Use

☐ **N/A:** This study is not banking data or specimens for future use or research outside the scope of the present protocol. This section does not apply.

*30.1 If data or specimens will be banked (stored) for **future use, that is, use or research outside of the scope of the present protocol**, describe where the data/specimens will be stored, how long they will be stored, how the data/specimens will be accessed, and who will have access to the data/specimens.*

NOTE: Your response here must be consistent with your response at the "What happens if I say yes, I want to be in this research?" Section of the Template Consent Document (HRP-502).

Response: The study data/specimens will be stored in a locked closet or -80 freezer at the research facility of the Diabetes and Endocrinology Center of WNY for at least 7 years.

The research staff (study personnel including coordinator) only will be authorized to access data and or specimens

30.2 List the data to be stored or associated with each specimen.

Response: Patient ID number, study visit information and date of collection will be stored with specimen. Other data stored will include record files of all patients participating in the study, including data collection sheets, lab results, CGM and insulin pump data.

30.3 Describe the procedures to release banked data or specimens for future uses, including: the process to request a release, approvals required for release, who can obtain data or specimens, and the data to be provided with specimens.

Response: The copy of the individual patient data collected during the study period will be provided to these individual patients who can choose to hand carry it to their respective physicians and a copy will be faxed to their respective clinical providers upon verbal request from the patient. The data provided will include the insulin pump or CGM data or any of the lab results obtained during the study period. The results of the completed study will be made available to the patients if requested through published manuscript.

31.0 Drugs or Devices

☐ **N/A:** This study does not involve drugs or devices. This section does not apply.

31.1 If the research involves drugs or devices, list and describe all drugs and devices used in the research, the purpose of their use, and their regulatory approval status.

Response: **Drugs used:**

Description or Identity of investigational product(s)

Investigational product	***Dosage form and strength***	***Manufacturer***	***Description***
Dapagliflozin (Farxiga) 5 mg	*Green, plain, diamond shaped, film coated 5 mg tablet*	*AstraZeneca*	*sodium-glucose cotransporter 2 (SGLT2) inhibitor indicated as an adjunct to diet and exercise to improve glycemic control*
Matching placebo for dapagliflozin 5 mg	*Green, plain, diamond shaped, film coated tablet*	*AstraZeneca*	
Semaglutide (Ozempic) 0.25 mg, 0.5 mg, 1.0 mg	*clear, colorless solution that contains 2 mg of semaglutide in a 1.5 mL (1.34 mg/mL) pre-filled, disposable, single-patient-use pen injector*	*Novo Nordisk*	*glucagon-like peptide 1 (GLP-1) receptor agonist indicated as an adjunct to diet and exercise to improve glycemic control*

- *Dapagliflozin and its matching placebo tablets will be supplied in bottles and are provided by Astra Zeneca.*
- *Semaglutide will be supplied as prefilled injectable pens and will be purchased commercially through the University at Buffalo Research Pharmacy.*

Subjects will receive drugs in their unmodified form. Dapagliflozin is a once daily tablet, while Semaglutide is a weekly subcutaneous injection.

Insulin pumps (CSII) and long and short acting insulin is the only diabetes medication accepted before and during this trial. No other diabetes drug is permitted.
Blood pressure or lipid lowering drugs are permitted if on stable dose for at least three months prior to start of trial. Glucagon injection is permitted as rescue medication for severe hypoglycemia. Insulin infusions may be administered by ER or hospital if patients develop DKA.

31.2 Describe your plans to store, handle, and administer those drugs or devices so that they will be used only on subjects and be used only by authorized investigators.

Response: Dapagliflozin 5mg tablets and matching placebo provided by Astra Zeneca will be stored in a locked cabinet in research pharmacy. Semaglutide provided by University at Buffalo research pharmacy will be stored in temperature controlled refrigerator at 4C at the research facility of the Diabetes and Endocrinology Center of WNY at 1000 Youngs Rd until dispensed to patients. Investigational drugs will be dispensed by PI or CO-Is only to study patients according to randomization plan.

If the drug is investigational (has an IND) or the device has an IDE or a claim of abbreviated IDE (non-significant risk device), include the following information:

31.3 Identify the holder of the IND/IDE/Abbreviated IDE.

Response: IND application for use of all drugs (dapagliflozin and semaglutide) in type 1 diabetes will be submitted to FDA and no study procedure will start before all local and federal approvals are obtained. FDA has granted IND for usage of dapagliflozin in type 1 diabetes. We will seek FDA approval for the use of semaglutide in this study. These IND letters will be submitted to IRB and JDRF along with final version of protocol before initiating any study activities.

31.4 Explain procedures followed to comply with FDA sponsor requirements for the following:

	Applicable to:		
FDA Regulation	***IND Studies***	***IDE studies***	***Abbreviated IDE studies***
21 CFR 11	***X***	***X***	
21 CFR 54	***X***	***X***	
21 CFR 210	***X***		
21 CFR 211	***X***		
21 CFR 312	***X***		
21 CFR 812		***X***	***X***
21 CFR 820		***X***	

Response: All FDA sponsor requirements have been reviewed and will be followed during the study procedures

32.0 Humanitarian Use Devices

☒ **N/A:** This study does not involve humanitarian use devices. This does not apply.

32.1 For Humanitarian Use Device (HUD) uses provide a description of the device, a summary of how you propose to use the device, including a description of any screening procedures, the HUD procedure, and any patient follow-up visits, tests or procedures.

Response:

32.2 For HUD uses provide a description of how the patient will be informed of the potential risks and benefits of the HUD and any procedures associated with its use.

Response:

US TTT1 Protocol Summary of changes V2 to V3

Section	Original	New	Justification	Consent changes
9.1	No statement	Along with this, a radio ad will be on the air of the radio station STAR102.5 at various times during the day during the month of mid January to mid February		NO

University at Buffalo Institutional Review Board (UBIRB)
Office of Research Compliance | Clinical and Translational Research Center Room 5018
875 Ellicott St. | Buffalo, NY 14203
UB Federalwide Assurance ID#: FWA00008824

Complete Research Protocol (HRP-503)

Table of Contents

Template Instructions 2
1.0 Objectives 4
2.0 Scientific Endpoints 5
3.0 Background 6
4.0 Study Design 12
5.0 Local Number of Subjects 13
6.0 Inclusion and Exclusion Criteria 13
7.0 Vulnerable Populations 16
8.0 Eligibility Screening 17
9.0 Recruitment Methods 18
10.0 Procedures Involved 19
11.0 Study Timelines 32
12.0 Setting 32
13.0 Community-Based Participatory Research 33
14.0 Resources and Qualifications 34
15.0 Other Approvals 35
16.0 Provisions to Protect the Privacy Interests of Subjects 35
17.0 Data Management and Analysis 36
18.0 Confidentiality 39
A. Confidentiality of Study Data 39
B. Confidentiality of Study Specimens 40
19.0 Provisions to Monitor the Data to Ensure the Safety of Subjects 41
20.0 Withdrawal of Subjects 48
21.0 Risks to Subjects 49
22.0 Potential Benefits to Subjects 54
23.0 Compensation for Research-Related Injury 55
24.0 Economic Burden to Subjects 56
25.0 Compensation for Participation 56
26.0 Consent Process 57
27.0 Waiver or Alteration of Consent Process 62
28.0 Process to Document Consent 62
29.0 Multi-Site Research (Multisite/Multicenter Only) 63
30.0 Banking Data or Specimens for Future Use 64
31.0 Drugs or Devices 64
32.0 Humanitarian Use Devices 66

Template Instructions

Sections that do not apply:

- *In several sections, the addition of checkboxes for **Not Applicable** have been added to the template as responses.*
    - *If an N/A checkbox is present, select the appropriate justification from the list.*
    - *If an N/A checkbox is not present, or if none of the existing checkboxes apply to your study, you must write in your own justification.*
- *In addition:*
    - *For research where the only study procedures are records/chart review: Sections 19, 20, 22, 23, 24, 25, 31, and 32 do not apply.*
    - *For exempt research: Sections 31 and 32 do not apply.*

Studies with multiple participant groups:

- *If this study involves multiple participant groups (e.g. parents and children), provide information in applicable sections for each participant group. Clearly label responses when they differ. For example:*

  Response:

  <u>Intervention Group:</u>

  <u>Control Group:</u>

Formatting:

- *Do not remove template instructions or section headings when they do not apply to your study.*

If you are pasting information from other documents using the "Merge Formatting" Paste option will maintain the formatting of the response boxes.

Amendments:

- *When making modifications or revisions to this and other documents, use the **Track Changes** function in Microsoft Word.*
- *Update the version date or number **on Page 3.***

PROTOCOL TITLE:

Include the full protocol title.

Response: **Triple Therapy for Type 1 Diabetes with Insulin, Semaglutide, and Dapagliflozin**

PRINCIPAL INVESTIGATOR:

Response:

**Principal Investigator:**	**Paresh Dandona, MBBS, PhD**
**Co- Investigators:**	**Husam Ghanim, PhD**
	**Antoine Makdissi, MD**
	**Manav Batra, MD**
**Co- Principal Investigator:**	**John R Petrie, MBChB, PhD**

Paresh Dandona, M.B.B.S., Ph.D., F.R.C.P, F.A.C.P, F.A.C.C, FACE,
SUNY Distinguished Professor,
Director, Diabetes-Endocrinology Center of Western NY
Chief of Endocrinology, Diabetes, Endocrinology and Metabolism, SUNY at Buffalo
1000 Youngs Road, Suite 105
Williamsville NY 14221
Telephone: (716) 535-1850
Fax: 716-639-1200
E-mail: pdandona@kaleidahealth.org

CO-PI:

Professor John R Petrie
BSc MBChB PhD FRCP(Ed) FRCPSG
Professor of Diabetic Medicine
Institute of Cardiovascular and Medical Sciences
BHF Glasgow Cardiovascular Research Centre
University of Glasgow
126 University Place
Glasgow G12 8TA
Tel: +44(0)141 330 3325
Email: john.petrie@glasgow.ac.uk
Web: http://www.gla.ac.uk/researchinstitutes/icams/staff/johnpetrie/
The University of Glasgow

VERSION:

Include the version date or number.

Response: 12/11/2019, V3

GRANT APPLICABILITY:

Indicate whether this protocol is funded by a grant (e.g. NIH, foundation grant). For a grant with multiple aims, indicate which aims are covered by this research proposal.

NOTE: This question does not apply to studies funded by a sponsor contract.

Include a copy of the grant proposal with your submission.

Response: Research support provided by JDRF and AZ

RESEARCH REPOSITORY:

Indicate where the research files will be kept, including when the study has been closed. The repository should include, at minimum, copies of IRB correspondence (approval, determination letters) as well as signed consent documents. This documentation should be maintained for 3 years after the study has been closed.

Response:
Site 1: Diabetes Endocrinology Research Center of WNY
Address: 1000 Youngs Road, Suite 105, Williamsville NY 14221
Department: Diabetes Endocrinology

Site 2: University of Glasgow. Institute of Cardiovascular and Medical Sciences University of Glasgow 126 University Place Glasgow, G12 8TA

1.0 Objectives

1.1 *Describe the purpose, specific aims, or objectives of this research.*

Response:

Primary Objective: To assess whether the addition of dapagliflozin to semaglutide and insulin (triple therapy) improves glycemic control in patients with type 1 diabetes compared with semaglutide and insulin (dual therapy) and insulin only (standard) treatment.

General hypothesis: The addition of dapagliflozin to semaglutide and insulin treatment improves glycemic control, weight loss and blood pressure in adults with type 1 diabetes.

1.2 *State the hypotheses to be tested, if applicable.*

NOTE: A hypothesis is a specific, testable prediction about what you expect to happen in your study that corresponds with your above listed objectives.

Response:

Specific hypotheses:

Primary: combined dapagliflozin and semaglutide treatment in addition to insulin will improve HbA1c levels compared with semaglutide in addition to insulin.

Secondary 1: semaglutide in addition to insulin treatment over 26 weeks will improve HbA1c levels compared with insulin therapy alone.

Secondary 2: combined dapagliflozin and semaglutide treatment in addition to insulin will improve HbA1c levels compared to insulin therapy alone at 52 weeks.

Secondary 3: combined dapagliflozin and semaglutide treatment in addition to insulin will improve CGM-based weekly mean and fasting blood glucose, glycemic oscillations and percent of time in range (70-180mg/dl) with lower insulin requirements compared with semaglutide and insulin (dual therapy) or insulin only (standard) treatment.

Secondary : combined dapagliflozin and semaglutide in addition to insulin therapy will reduce systolic and diastolic blood pressure compared with semaglutide and insulin (dual therapy) or insulin only (standard) treatment.

Secondary 5: combined dapagliflozin and semaglutide in addition to insulin therapy (triple therapy) will result in weight loss compared to dual and standard therapy.

Secondary 6: combined dapagliflozin and semaglutide in addition to insulin therapy will result in improved scores on the Diabetes Specific Quality of Life Scale (DSQOLS) and the Problem Areas In Diabetes (PAID) survey compared with dual and standard therapy.

Tertiary and Exploratory Objectives (EO) for future ancillary studies (PLEASE NOTE: For EO, samples will be collected to facilitate the measurements of these end points in future ancillary studies once submitted and approved by JDRF so cost of assays is not included in the current budget) : The addition of dapagliflozin to semaglutide and insulin in type 1 diabetes will:

Tertiary 1: combined dapagliflozin and semaglutide treatment in addition to insulin therapy will improve fasting and postprandial glucose excursions and suppress postprandial glucagon, FFA, GLP-1 compared with semaglutide and insulin or insulin therapy alone.

Tertiary 2: semaglutide in addition to insulin treatment over 52 weeks will improve HbA1c levels compared with insulin therapy alone.

EO 1: increase plasma concentrations of vasodilatation mediators and reduce plasma concentrations of vasoconstriction mediators compared to dual and standard therapy groups.

EO 2: exert a systemic anti-inflammatory effect including reducing mRNA expression of inflammatory mediators and NFκB binding in peripheral mononuclear cells (MNC) and adipose tissue (optional) and their circulating levels in plasma while increasing levels of IL-RA and adiponectin.

2.0 Scientific Endpoints

2.1 Describe the scientific endpoint(s), the main result or occurrence under study.

NOTE: Scientific endpoints are outcomes defined before the study begins to determine whether the objectives of the study have been met and to draw conclusions from the data. Include primary and secondary endpoints. Some example endpoints are: reduction of

*symptoms, improvement in quality of life, or survival. Your response should **not** be a date.*

Response:

Primary endpoint: Change in HbA1c at 6 months following dapagliflozin or placebo therapy in addition to combined semaglutide and insulin treatment.

Efficacy Secondary endpoints:

1. Change in HbA1c from baseline at 12 months in triple therapy group compared to insulin only group.
2. Change in HbA1c from baseline at 6 months in dual therapy group compared to insulin only group.
3. Change in percent Time in Range (70– 180 mg/dl), percent time in hyperglycemia Level 1(180-250mg/dl) and Level 2 (>250mg/dl) and percent time in hypoglycemia Level 1 (54-70mg/dl) as assessed by CGM
4. Other indices of glycemic control: fructosamine, weekly mean and fasting glucose, standard deviation, insulin requirements.
5. Change in body weight and quality of life as assessed by DSQOLS and PAID at 6 and 12 months.
6. Anti-hypertensive effects including change in systolic and diastolic BP, numbers of BP medications required.

Safety Secondary endpoints:

1- Differences in rates of hypoglycemic events Level 2 (<54mg/dl) and Level 3 (A severe event characterized by altered mental and/or physical status requiring assistance) between investigative arms and standard therapy arm.

2- *Differences in rates of diabetic ketoacidosis defined as elevated serum or urine ketones (greater than the upper limit of the normal range), and serum bicarbonate <15 mmol/L or Blood pH <7.3 between investigative arms and standard therapy arm.*

Tertiary/ exploratory endpoints: (items 2-4 to be performed in future on collected samples from this study if additional funding is approved by JDRF):

1- Fasting and postprandial (Area Under curve) glucose excursions, FFA, GLP-1 and glucagon and fasting fructosamine.

2- Indices of inflammatory stress. These will be CRP, FFA, SAA, IL-1β, IL-18, IL-1RA, adiponectin and TNFα concentrations in plasma and isoprostane in urine.

3- Expression of IL-1β, TNFα, IL-12, TLR2, TLR4, adiponectin, IκBα as well as NFκB binding in peripheral MNC and adipose tissue (optional).

4- Indices of vasodilatation and vasoconstriction: ANP, b-NP, cGMP, cAMP, angiotensinogen, renin and angiotensin II concentrations.

3.0 Background

3.1 Provide the scientific or scholarly background, rationale, and significance of the research based on the existing literature and how it will contribute to existing knowledge. Describe any gaps in current knowledge. Include relevant preliminary findings or prior research by the investigator.

Response:

The control of glucose homeostasis in type 1 diabetes is fragile since β-cell reserve is negligible. Traditionally, insulin has been the only treatment approach available. This approach, often leads to erratic blood sugar control given the challenge of matching exogenous insulin to mealtime glucose excursions in the face of a lack of any endogenous insulin to compensate for variation in requirements in response to meals and activity levels. Injected insulin boluses rarely match precise insulin requirements either in terms of dose administered or bio-availability. The result often is wide fluctuations in blood glucose readings. Based on our work to date and other published reports, previous attempts to add other glucose-lowering medications to insulin therapy yielded minimal benefits on glycemia (although metformin may have other benefits (1-3)

We have recently shown in two preliminary retrospective studies that the addition of liraglutide, a Glucagon Like Peptide -1 receptor agonist (GLP-1RA), to insulin results in consistent improvements in glycemic control in type 1 diabetes as demonstrated by reductions in HbA1c, glycemic excursions, and insulin requirements accompanied by improvements in body weight and systolic blood pressure (4, 5). We have now also completed the first prospective randomized controlled trial of this drug in 72 people with type 1 diabetes. Our data not only confirmed improvements in glycemic control, HbA1c, body weight and systolic BP, but also demonstrated a reduction in appetite, carbohydrate intake in low grade inflammation as measured by plasma CRP concentrations (6). Importantly, quality of life was also improved. Our recent studies in type 2 diabetes have revealed that systolic blood pressure reduction with GLP-1RA (exenatide) treatment in type 2 diabetes is associated with increased plasma concentrations of ANP, cGMP and cAMP along with reduced concentrations of angiotensinogen, renin and angiotensin II (7). It is possible that these mechanisms play a role in the prevention of cardiovascular complications in type 1 diabetes.

While GLP-1RAs, therefore, have great potential as adjunct therapy for type 1 diabetes, mean HbA1c was still ≥7% after liraglutide treatment (6) and larger pharma-sponsored trials reported increased rates of hypoglycemia (8, 9). In seeking further improvement, the advent of sodium-glucose transport protein-2 (SGLT2) inhibitors is of importance since their mode of action is independent of insulinogenesis. Introduced for use in type 2 diabetes, SGLT2 inhibitors are selective antagonists of the sodium-glucose transporter subtype expressed in the proximal convoluted tubules of the kidneys. They act by inhibiting renal glucose re-absorption and therefore stimulating excretion of 60-100 grams per day of glucose in urine. SGLT2 inhibitors have been studied extensively in type 2 diabetes, including the landmark cardiovascular outcome trial EMPA-REG (10), but data have only quite recently begun to accumulate in type 1(11). Short term effects on urine glucose excretion of remogliflozin and empagliflozin are similar to those seen in type 2 diabetes and these translate into improved glycemic and other indices including HbA1c; fasting blood glucose, rates of hypoglycemia, daily insulin dose and weight (12). Weight loss accompanying increased urinary glucose excretion could translate into reduced cardiovascular complications as in type 2 diabetes, but there are no data so far available to support this hypothesis. More recently, in the Phase 3 trial DEPICT-1 we showed that dapagliflozin improves glycemic control and HbA1c over six months by about 0.45% with no severe hypoglycemia or diabetic ketoacidosis (13). Similarly, sotagliflozin has been shown in a Phase 3 trial to allow a larger proportion of patients to achieve HbA1c lower than 7.0% with no severe hypoglycemia although the rate of diabetic ketoacidosis was

higher in the sotagliflozin group (14). Both products are now being reviewed by regulatory agencies for the type 1 diabetes indication.

On the basis of the above, we hypothesize that adding an SGLT2 inhibitor to a combination of insulin and semaglutide (a weekly and potent GLP-1RA) will improve glycemic control in type 1 diabetes such that HbA1c levels of ≤7% are achieved more consistently. Our protocol is designed to allow an objective assessment of whether this triple therapy combination will work effectively and safely so that a majority of people with type 1 diabetes can reach a glycemic target HbA1c of ≤7%. In addition, since both semaglutide and dapagliflozin inhibitors reduce blood pressure and weight, it will allow an assessment of the combined effect of GLP-1RAs and SGLT2 inhibitors on these outcomes. Since there are no previous data on the effects of once weekly semaglutide or the combination of semaglutide and dapagliflozin compared to standard insulin treatment in type 1 diabetes, the design requires allocation of some participants to insulin therapy only.

Due to the potential ketogenic effect of SGLT-2 inhibitors, data from SGLT-2 inhibitors studies in type 1 diabetes were analyzed for it ketogenic effects and a consensus statement and guidelines on management of patients was recently published (Danne T et al., Diabetes Care. 2019 Feb 6).

Preliminary data

1) Liraglutide treatment in patients with type 1 diabetes (4): We treated 8 type 1 diabetes patients; mean age (38.5 years) and duration of diabetes (20±4 yrs), with liraglutide in addition to insulin for 24 weeks. As shown in table 1, there was a significant reduction in fasting and mean blood glucose concentrations, insulin doses, HbA1c and a reduction in appetite and caloric intake with weight loss. In addition, there was a reduction in the excursions of blood glucose concentrations, i.e., the standard deviations of blood glucose concentrations were markedly reduced (**figure 1**). The changes were observed within 48h and improved marginally thereafter with fine tuning of insulin doses and a further reduction in these doses. These observations are noteworthy in that study participants were well controlled and meticulous users of CSII and CGM. They had all tried hard to improve their diabetic control by adjusting insulin doses but had not succeeded because of hypoglycemia. We then demonstrated that poorly controlled and obese individuals with type 1 diabetes also experienced similar benefits of liraglutide treatment (2).

2) Liraglutide As Additional Treatment to Insulin in Patients with Type 1 Diabetes Mellitus: A Randomized Clinical Trial (6): Following the two above non-randomized studies we recently conducted a randomized clinical trial in which 72 individuals (Placebo=18; liraglutide = 54) with TYPE 1 DIABETES for at least one year, on insulin therapy and with no detectable C-peptide in plasma (mean BMI: 30±1; mean body weight: 84±2 kg ; mean HbA1c: 7.57±0.09%; mean age: 44±2 years; mean age of TYPE 1 DIABETES diagnosis: 20±1 years) were randomized to receive placebo, 0.6, 1.2 or 1.8mg of liraglutide daily for 12 weeks. In the 1.2 mg and 1.8 mg groups, mean change in average blood glucose was -10±2 and -10.0±1mg/dl, respectively, ($p<0.0001$ vs. placebo). In the 1.2mg group, HbA1c fell by 0.78% from 7.84±0.17% to 7.06±0.15% ($p<0.0001$, $p<0.01$ vs. placebo) and in the 1.8mg group fell by 0.42% from 7.41±0.15 to 6.99±0.15 ($p=0.001$, $p=0.39$ vs placebo). Time spent between 70 to 160 mg/dl increased by 5±1% ($p<0.05$ vs

placebo) and time between 160-400 mg/dl decreased by 7±1% ($p<0.01$ vs placebo) in the 1.8 mg group with no additional hypoglycemia. During the 12 week period, the average total daily dose of insulin in the two groups fell by 12.4±3.9 and 10.0±2.3 units respectively ($p<0.05$ vs baseline and placebo). There was a reduction in body weight (from 83±4 to 78±5 kg, $p<0.0001$, in the 1.8mg group, from 96±4 to 91±4 kg, $p<0.001$, in the 1.2 mg group and from 80±4 to 77±4 kg, $p<0.01$ in the 0.6 mg group). A reduction in daily carbohydrate intake (171±17g vs. 127±18g; $p<0.01$ and 153±18g vs 115±16g, $p<0.01$ in the 1.2 mg and 1.8 mg groups, respectively) was also observed over 12 weeks. Systolic BP (SBP) in the 1.8 mg group fell by 3 mmHg (120±2 to 117±3 mmHg, $p=0.01$). CRP fell by 15±6 and 19±8% in the 1.2 and 1.8mg groups, respectively ($p<0.05$). Quality of life improved significantly compared to baseline and placebo (unpublished data).

3) Dapagliflozin as an Additional Treatment to Insulin and Liraglutide in Patients with Type 1 Diabetes Mellitus (15): The objective of this randomized clinical trial was to investigate whether addition of dapagliflozin to insulin and liraglutide results in a significant reduction in glycemia and body weight. 30 participants with TYPE 1 DIABETES on liraglutide therapy for at least last 6 months were randomized (in 2:1 ratio) to receive either dapagliflozin 10 mg or placebo daily for 12 weeks. With 12 weeks of dapagliflozin HbA1c fell by 0.66±0.08% from 7.8±0.21% ($p < .01$ vs placebo) but did not change significantly with placebo (7.40±0.20% to 7.30±0.20%). In one patient within one week of addition of dapagliflozin average glucose fell from 170 to 122 mg/dl while standard deviation was reduced from 71 to 58 (**Figure 2-diminished glycemic excursions)**. Body weight fell by1.9 +/- 0.54kg ($p < 0.05$ vs placebo). There was no additional hypoglycemia (blood glucose < 3.88 mmol/L; $p = 0.52$ vs placebo). In the dapagliflozin group, there were significant increases in the plasma concentrations of glucagon by 35% +/- 13% ($p < 0.05$), hormone-sensitive lipase by 29% +/- 11% ($p < 0.05$), free fatty acids by 74% +/- 32% ($p < 0.05$), acetoacetate by 67% +/- 34% ($p < 0.05$), and beta-hydroxybutyrate by 254% +/- 81% ($p < 0.05$). Urinary ketone levels also increased significantly ($P < .05$). None of these changes was observed in the placebo group. Two individuals in the dapagliflozin group developed ketoacidosis. Overall, addition of dapagliflozin to insulin and liraglutide in patients with TYPE 1 DIABETES resulted in a significant improvement in glycemia and in weight loss but tended to increase ketosis.

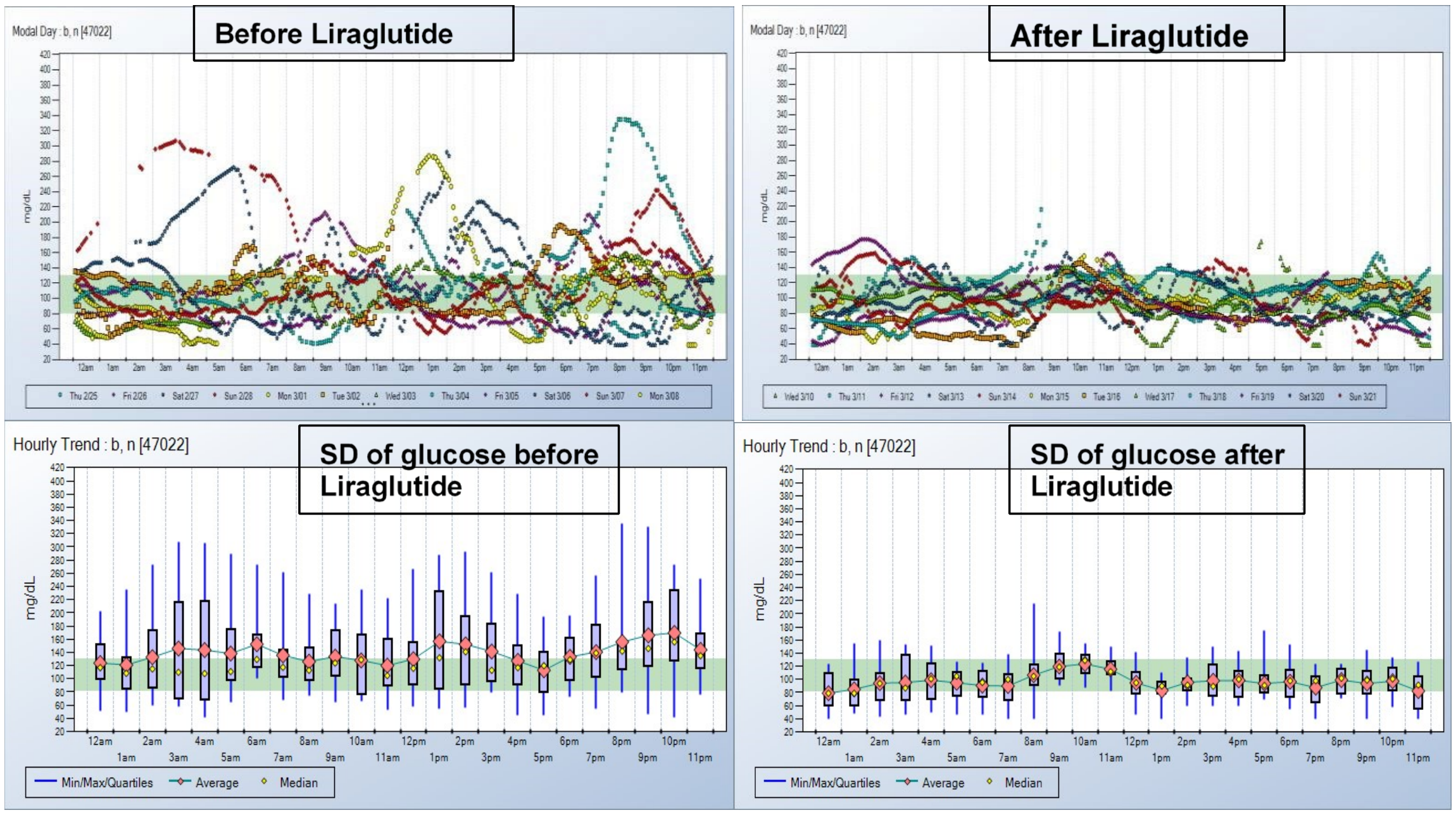

Figure 1: Weekly continuous glucose monitoring record of one patient prior to and following 1 week of liraglutide. Note the glycemic variability (SD) and the frequency of glucose concentrations within the target range prior to and following liraglutide

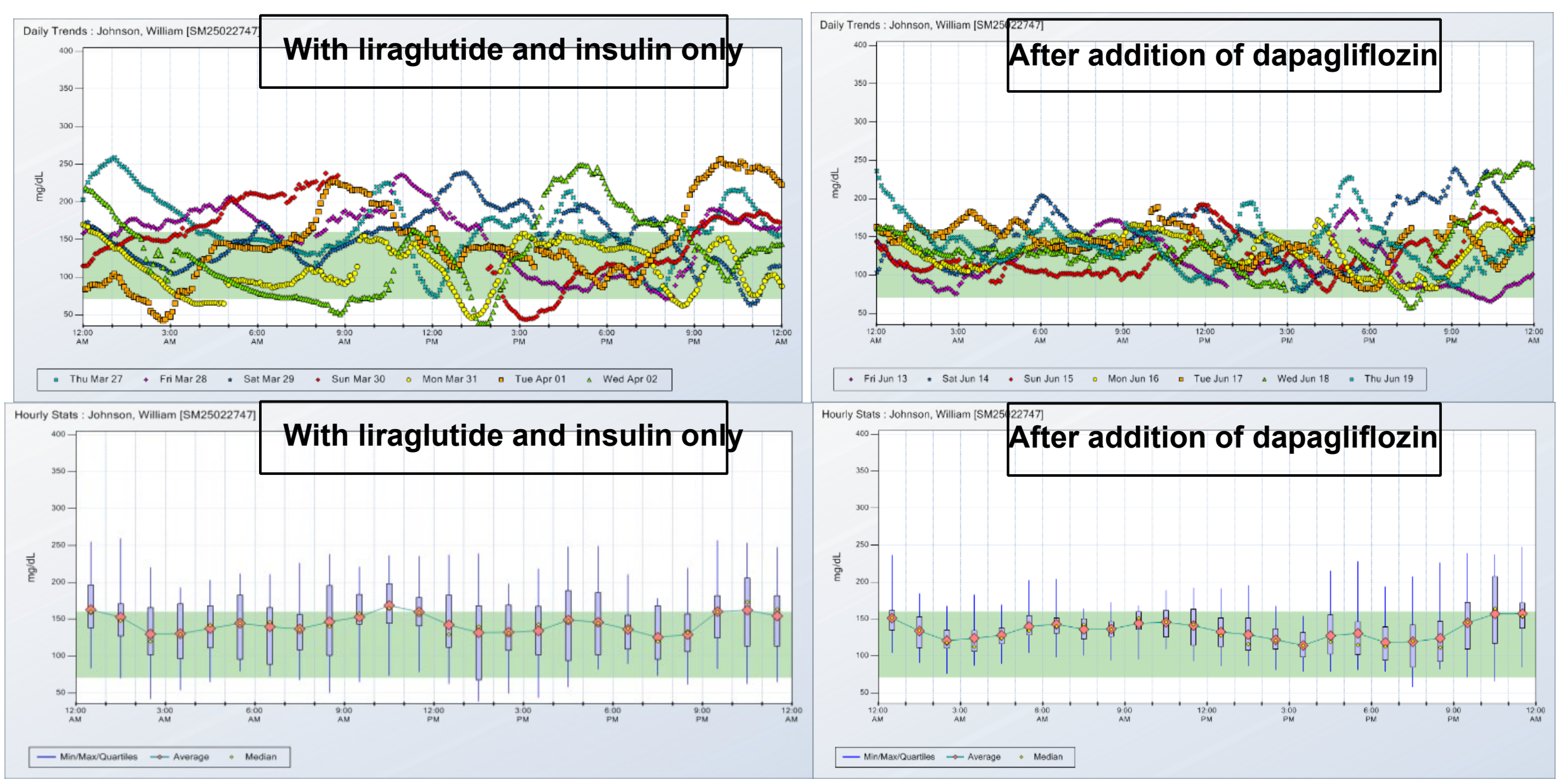

Figure 2: Weekly continuous glucose monitoring record of one patient prior to and following 8 weeks of the triple therapy combination with dapagliflozin and liraglutide. Note the glycemic variability (SD) (bottom) and the frequency of glucose concentrations within the target range prior to and following dapagliflozin addition.

3.2 Include complete citations or references.

Response:

1. Konrad K, Datz N, Engelsberger I, Grulich-Henn J, Hoertenhuber T, Knauth B, et al. Current use of metformin in addition to insulin in pediatric patients with type 1 diabetes mellitus: an analysis based on a large diabetes registry in Germany and Austria. *Pediatr Diabetes.* 2014.
2. Vella S, Buetow L, Royle P, Livingstone S, Colhoun HM, and Petrie JR. The use of metformin in type 1 diabetes: a systematic review of efficacy. *Diabetologia.* 2010;53(5):809-20.
3. Vella S, Buetow L, Royle P, Livingstone S, and Petrie JR. Metformin in type 1 diabetes reduces insulin requirements without significantly improving glycaemic control. Reply to Schatz H [letter]. *Diabetologia.* 2011;54(1):203-4.
4. Varanasi A, Bellini N, Rawal D, Vora M, Makdissi A, Dhindsa S, et al. Liraglutide as additional treatment for type 1 diabetes. *Eur J Endocrinol.* 2011;165(1):77-84.
5. Kuhadiya ND, Malik R, Bellini NJ, Patterson JL, Traina A, Makdissi A, et al. Liraglutide as additional treatment to insulin in obese patients with type 1 diabetes mellitus. *Endocrine practice : official journal of the American College of Endocrinology and the American Association of Clinical Endocrinologists.* 2013;19(6):963-7.
6. Kuhadiya ND, Dhindsa S, Ghanim H, Mehta A, Makdissi A, Batra M, et al. Addition of Liraglutide to Insulin in Patients With Type 1 Diabetes: A Randomized Placebo-Controlled Clinical Trial of 12 Weeks. *Diabetes Care.* 2016;39(6):1027-35.
7. Chaudhuri A, Ghanim H, Makdissi A, Green K, Abuaysheh S, Batra M, et al. Exenatide induces an increase in vasodilatory and a decrease in vasoconstrictive mediators. *Diabetes Obes Metab.* 2017;19(5):729-33.
8. Mathieu C, Zinman B, Hemmingsson JU, Woo V, Colman P, Christiansen E, et al. Efficacy and Safety of Liraglutide Added to Insulin Treatment in Type 1 Diabetes: The ADJUNCT ONE Treat-To-Target Randomized Trial. *Diabetes Care.* 2016;39(10):1702-10.
9. Ahren B, Hirsch IB, Pieber TR, Mathieu C, Gomez-Peralta F, Hansen TK, et al. Efficacy and Safety of Liraglutide Added to Capped Insulin Treatment in Subjects With Type 1 Diabetes: The ADJUNCT TWO Randomized Trial. *Diabetes Care.* 2016;39(10):1693-701.
10. Zinman B, Wanner C, Lachin JM, Fitchett D, Bluhmki E, Hantel S, et al. Empagliflozin, Cardiovascular Outcomes, and Mortality in Type 2 Diabetes. *N Engl J Med.* 2015;373(22):2117-28.
11. Petrie JR. SGLT2 inhibitors in type 1 diabetes: knocked down, but up again? *Lancet Diabetes Endocrinol.* 2017;5(11):841-3.
12. Mudaliar S, Armstrong DA, Mavian AA, O'Connor-Semmes R, Mydlow PK, Ye J, et al. Remogliflozin etabonate, a selective inhibitor of the sodium-glucose transporter 2, improves serum glucose profiles in type 1 diabetes. *Diabetes Care.* 2012;35(11):2198-200.
13. Dandona P, Mathieu C, Phillip M, Hansen L, Griffen SC, Tschope D, et al. Efficacy and safety of dapagliflozin in patients with inadequately controlled type 1 diabetes (DEPICT-1): 24 week results from a multicentre, double-blind, phase 3, randomised controlled trial. *Lancet Diabetes Endocrinol.* 2017;5(11):864-76.

14. Garg SK, Henry RR, Banks P, Buse JB, Davies MJ, Fulcher GR, et al. Effects of Sotagliflozin Added to Insulin in Patients with Type 1 Diabetes. *N Engl J Med.* 2017;377(24):2337-48.
15. Kuhadiya ND, Ghanim H, Mehta A, Garg M, Khan S, Hejna J, et al. Dapagliflozin as Additional Treatment to Liraglutide and Insulin in Patients With Type 1 Diabetes. *J Clin Endocrinol Metab.* 2016;101(9):3506-15.
16. Henry RR, Thakkar P, Tong C, Polidori D, and Alba M. Efficacy and Safety of Canagliflozin, a Sodium-Glucose Cotransporter 2 Inhibitor, as Add-on to Insulin in Patients With Type 1 Diabetes. *Diabetes Care.* 2015;38(12):2258-65.
17. Peters AL, Buschur EO, Buse JB, Cohan P, Diner JC, and Hirsch IB. Euglycemic Diabetic Ketoacidosis: A Potential Complication of Treatment With Sodium-Glucose Cotransporter 2 Inhibition. *Diabetes Care.* 2015;38(9):1687-93.
18. Ahmann AJ, Capehorn M, Charpentier G, Dotta F, Henkel E, Lingvay I, et al. Efficacy and Safety of Once-Weekly Semaglutide Versus Exenatide ER in Subjects With Type 2 Diabetes (SUSTAIN 3): A 56-Week, Open-Label, Randomized Clinical Trial. *Diabetes Care.* 2017.
19. Andrews NC, and Faller DV. A rapid micropreparation technique for extraction of DNA-binding proteins from limiting numbers of mammalian cells. *Nucleic Acids Res.* 1991;19(9):2499.
20. Perkins BA, Cherney DZ, Partridge H, Soleymanlou N, Tschirhart H, Zinman B, et al. Sodium-glucose cotransporter 2 inhibition and glycemic control in type 1 diabetes: results of an 8-week open-label proofof-concept trial. *Diabetes Care.* 2014;37(5):1480-3.
21. Cherney DZ, Perkins BA, Soleymanlou N, Maione M, Lai V, Lee A, et al. Renal hemodynamic effect of sodium-glucose cotransporter 2 inhibition in patients with type 1 diabetes mellitus. *Circulation.* 2014;129(5):587-97.

4.0 Study Design

4.1 Describe and explain the study design (e.g. case-control, cross-sectional, ethnographic, experimental, interventional, longitudinal, observational).

Response: This will be a two-center, randomized, open-label then double-blind, parallel group, placebo and standard of care controlled prospective study in type 1 diabetes. One hundred and (114) patients will be randomized (open label, see below) into three groups (38 each) and will be either maintained on their regular insulin therapy for 12 months (standard therapy group) or started on semaglutide in addition to regular insulin treatment for six month (dual therapy and triple therapy groups). For those on insulin and semaglutide, after six months of stable treatment, dapagliflozin (triple therapy group) or placebo (dual therapy group) treatment will be initiated for an additional six months according to a second randomization (double-blind).

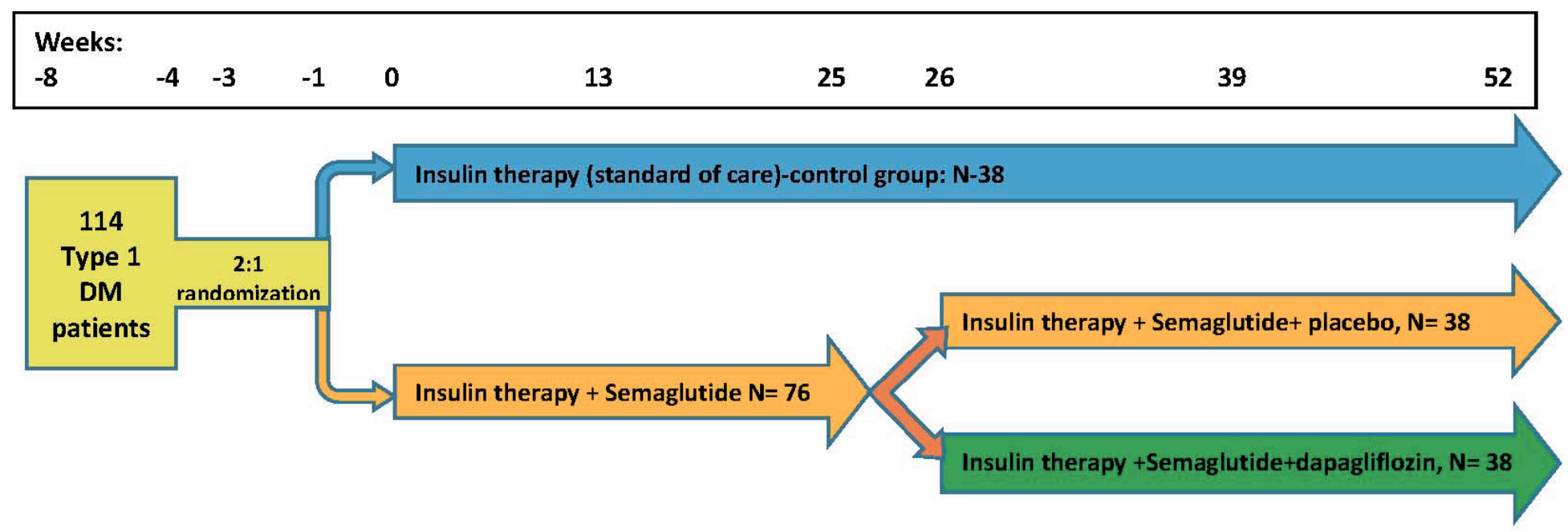

5.0 Local Number of Subjects

5.1 Indicate the total number of subjects that will be enrolled or records that will be reviewed locally.

Response: 57 patients will be enrolled locally with another 57 patients recruited at the University of Glasgow site: a total of 114.

5.2 If applicable, indicate how many subjects you expect to screen to reach your target sample (i.e. your screen failure rate).

Response: All screened and qualified patients will be enrolled and randomized

5.3 Justify the feasibility of recruiting the proposed number of eligible subjects within the anticipated recruitment period. For example, how many potential subjects do you have access to? What percentage of those potential subjects do you need to recruit?

Response: The Diabetes and Endocrinology Center of WNY is the largest Diabetes center in the WNY area, seeing between 60 to 100 type 1 diabetic patient every month. Therefore, the majority of recruited patients are our clinic patients. We also recruit a study participants through advertisement and other physician's references. These sources will suffice to recruit the needed number to subjects. The Glasgow center (Stobhill Ambulatory Care Hospital) reviews more than 300 patients per month.

6.0 Inclusion and Exclusion Criteria

*6.1 Describe the criteria that define who will be **included** in your final study sample.*

NOTE: This may be done in bullet point fashion.

Response:
Inclusion Criteria

1) Type 1 Diabetes for at least 1 year on staple use of continuous subcutaneous insulin infusion (CSII) or multiple daily (four or more) injections (MDI) of insulin for last 3 months.
2) C-peptide <0.23 nM
3) Minimum dose of insulin in Units/kg at entry: 0.5 U/kg for MDI and 0.4 U/kg for CSII
4) Regularly measuring blood sugars four or more times daily or use of CGM.
5) HbA1c between7.5-11% inclusive.
6) Well versed in CHO counting*
7) Age 18-70 years.
8) BMI ≥25 kg/m^2.

*In the opinion of the site Principal Investigator

6.2 Describe the criteria that define who will be ***excluded*** *from your final study sample.*

NOTE: This may be done in bullet point fashion.

Response:

Exclusion Criteria

1) Type 1 diabetes for less than 12 months, type 2 diabetes, chronic pancreatitis, MODY
2) Previous use of any agent other than insulin for treatment of diabetes in the last 3 months.
3) History of diabetic ketoacidosis (DKA) requiring medical intervention (e.g., emergency room visit and/or hospitalization) within 3 months prior to the screening visit
4) Patients on ketogenic or low (<50g/day) carbohydrate diet or who practice intermittent fasting.
5) Frequent episodes of severe hypoglycemia as defined by more than one episode requiring medical assistance, emergency care (paramedics or emergency room care), and/or glucagon therapy administered by a third-party individual within 1 month prior to the screening visit
6) Symptoms of poorly controlled diabetes that would preclude participation in this trial
7) Subjects on a commercial weight loss program with ongoing weight loss, or on an intensive exercise program
8) History of bariatric surgery or lap-band procedure within 12 months prior to screening
9) History of Addison's disease or chronic adrenal insufficiency
10) History of diabetes insipidus
11) Hepatic disease or cirrhosis
12) Aspartate Aminotransferase (AST) > 3X Upper limit of normal (ULN) *and/or* Alanine aminotransferase (ALT) > 3X ULN
13) Serum Total Bilirubin > 2X ULN unless exclusively caused by Gilbert's Syndrome

14) Hemoglobin < 11.0 g/dL (110 g/L) for men; hemoglobin < 10.0 g/dL (100 g/L) for women.
15) Coronary event or procedure (myocardial infarction, unstable angina, coronary artery bypass, surgery or coronary angioplasty) in the previous 3 months or patients with congestive heart failure.
16) ESRD on hemodialysis; and or e-GFR < 60 ml/min/1.73m^2
17) HIV or Hepatitis B/C positive status
18) Any other life-threatening, noncardiac disease
19) History of pancreatitis
20) Women who are pregnant or women of childbearing potential who are not using adequate contraception or who are breast feeding
21) Inability to give informed consent
22) History of gastroparesis
23) History of medullary thyroid carcinoma or MEN 2 syndrome
24) History of serious hypersensitivity reaction to these agents
25) Painful gallstones
26) Alcoholism
27) Hypertriglyceridemia (>500 mg/dl)
28) Recurrent genital mycotic infection.
29) Hypovolemic patients or with chronic renal insufficiency.
30) Patients with any malignancy except treated in situ malignancy and basal cell carcinoma of the skin
31) Unexplained hematuria
32) Patients with a history of diabetic retinopathy
33) Use of an investigational agent or therapeutic regimen within 30 days of study
34) Participation in any other concurrent interventional clinical trial

6.3 Indicate specifically whether you will include any of the following special populations in your study using the checkboxes below.

NOTE: Members of special populations may not be targeted for enrollment in your study unless you indicate this in your inclusion criteria.

Response: NA

☐ Adults unable to consent

☐ Individuals who are not yet adults (infants, children, teenagers)

☐ Pregnant women

☐ Prisoners

6.4 Indicate whether you will include non-English speaking individuals in your study. ***Provide justification if you will exclude non-English speaking individuals.***

*In order to meet one of the primary ethical principles of equitable selection of subjects, non-English speaking individuals may **not** be routinely excluded from research as a matter of convenience.*

In cases where the research is of therapeutic intent or is designed to investigate areas that would necessarily require certain populations who may not speak English, the researcher is required to make efforts to recruit and include non-English speaking individuals. However, there are studies in which it would be reasonable to limit subjects to those who speak English. Some examples include pilot studies, small unfunded studies with validated instruments not available in other languages, studies with numerous questionnaires, and some non-therapeutic studies which offer no direct benefit.

Response:

We have no non-English speaking patients in this population. We have patients that English is a second language, but they are able to read, write and understand it. This population is less than 10% of the total population. No benefit is being withheld from not participating in the study.

7.0 Vulnerable Populations

*If the research involves special populations that are considered vulnerable, **describe the safeguards included to protect their rights and welfare.***

NOTE: You should refer to the appropriate checklists, referenced below, to ensure you have provided adequate detail regarding safeguards and protections. You do not, however, need to provide these checklists to the IRB.

*7.1 For research that involves **pregnant women,** safeguards include:*
NOTE CHECKLIST: Pregnant Women (HRP-412)

Response: We will not be recruiting subjects from vulnerable populations

☒ **N/A**: This research does not involve pregnant women.

*7.2 For research that involves **neonates of uncertain viability or non-viable neonates,** safeguards include:*
NOTE CHECKLISTS: Non-Viable Neonates (HRP-413), or Neonates of Uncertain Viability (HRP-414)

Response:

☒ **N/A:** This research does not involve non-viable neonates or neonates of uncertain viability.

*7.3 For research that involves **prisoners**, safeguards include:*
NOTE CHECKLIST: Prisoners (HRP-415)

Response:

☒ **N/A:** This research does not involve prisoners.

*7.4 For research that involves **persons who have not attained the legal age for consent to treatments or procedures involved in the research ("children")**, safeguards include:*
NOTE CHECKLIST: Children (HRP-416)

Response:

☒ **N/A:** This research does not involve persons who have not attained the legal age for consent to treatments or procedures ("children").

*7.5 For research that involves **cognitively impaired adults**, safeguards include:*
NOTE CHECKLIST: Cognitively Impaired Adults (HRP-417)

Response:

☒ **N/A:** This research does not involve cognitively impaired adults.

*7.6 Consider if other specifically targeted populations such as students, employees of a specific firm, or educationally or economically disadvantaged persons are vulnerable. **Provide information regarding their safeguards and protections, including safeguards to eliminate coercion or undue influence.***

Response: No specific populations or vulnerable groups will be targeted. All subjects enrolled in this study will be of legal adult consenting age with the ability to speak, read and interpret the English language. Patients will have the ability to speak with the research team regarding any questions or concern they have before signing the consent. Research team have the ability to evaluate patient's cognitive abilities through these interactions with patients. Patients are made aware that this study is voluntary and they are able to stop participating at any time they feel uncomfortable. Patients are not be pressured into participating and their clinic standard of care will remain the same if they participate or choose not to participate.

8.0 Eligibility Screening

*8.1 Describe **screening procedures** for determining subjects' eligibility. Screening refers to determining if prospective participants meet inclusion and exclusion criteria.*

Include all relevant screening documents with your submission (e.g. screening protocol, script, questionnaire).

Response:

After obtaining partial HIPAA waiver, patient charts will be screened according to the study inclusion and exclusion criteria by our trained clinical staff and physicians. In additions, the study clinical team will evaluate their clinic patients for possible participation in this study according to the inclusion and exclusion criteria at the Diabetes and Endocrinology Center of WNY clinics. Physicians will

speak to them about their interests in participating in research. If the patient agrees, their information will be given to the research coordinator to be contacted for further evaluation. The research coordinator will contact interested patients and those meeting the inclusion and exclusion criteria based on preliminary phone evaluation will be invited to participate in the study. All personal information will be kept confidential and locked in the coordinator office.

Prospective participants will be asked to read the consent and any questions they may have regarding the protocol will be answered. If the subject wants to participate in the study, they will be asked to sign the informed consent form. The subject's medical history and current medications will be obtained as well as their blood pressure and vitals. A physical examination will also be done. Blood and urine samples will be taken in order to evaluate HbA1c, CBC, CMP and liver and kidney functions and pregnancy status. Females of childbearing age, will have to be on two forms of birth control to prevent pregnancy. Patients meeting all the inclusion and exclusion criteria based on all screening tests will be enrolled in the study.

☐ **N/A:** There is no screening as part of this protocol.

9.0 Recruitment Methods

☐ **N/A:** This is a records review only, and subjects will not be recruited. NOTE: If you select this option, please make sure that all records review procedures and inclusion/exclusion screening are adequately described in other sections.

9.1 Describe when, where, and how potential subjects will be recruited.

NOTE: Recruitment refers to how you are identifying potential participants and introducing them to the study. Include specific methods you will use (e.g. searching charts for specific ICD code numbers, Research Participant Groups, posted advertisements, etc.).

Response:

Participants will be identified by prescreening clinical charts, patient doctor interaction at the time of their visits to the Diabetes Endocrinology Center of WNY Locations include:

1. 1020 Youngs Road, Williamsville NY 14221
2. 705 Maple Road, Williamsville NY 14221
3. 462 Grider Street, Buffalo NY 14215
4. 1000 Youngs Road, Suite 105, Williamsville NY 14221

Patients can be also referred by other physicians and providers

The remainder of the participants (57 patients) will be recruited from NHS Greater Glasgow and Clyde Diabetes clinics (Scotland, UK). Separate local IRB/ Ethical approval will be sought to cover this site (based on discussions with UBIRB director).

The study clinical team will evaluate their clinic patients for possible participation in this study according to the inclusion and exclusion criteria at the Diabetes and Endocrinology Center of WNY clinics. Patients that may qualify for the study are referred to the research team for further eligibility evolution. Patients meeting the inclusion and exclusion criteria based on preliminary phone evaluation will be invited to participate in the study.

Participants will also be identified by flyers, advertisements on Facebook and Craigslist and researchmatch.org. The study will also be put on UB's Study Information Portal (SIP) website. Along with this, a radio ad will be on the air of the radio station STAR102.5 at various times during the day during the month of mid January to mid February

9.2 Describe how you will protect the privacy interests of prospective subjects during the recruitment process.

NOTE: Privacy refers to an individual's right to control access to him or herself.

Response: Patient charts will be screened according to the study inclusion and exclusion criteria by our trained clinical staff and physicians. If the patient qualifies and is of consenting age, the physicians will speak to them about their interests in participating in research in a private room where no one else can hear the conversation. If the patient agrees, their information will be given to the research coordinator to be contacted for further evaluation. No personal information will be shared without patient's approval and will not be used for any purpose except for this research. All personal information will be kept confidential and locked in the coordinator office.

9.3 Identify any materials that will be used to recruit subjects.

NOTE: Examples include scripts for telephone calls, in person announcements / presentations, email invitations.

For advertisements, include the final copy of printed advertisements with your submission. When advertisements are taped for broadcast, attach the final audio/video tape. NOTE: You may submit the wording of the advertisement prior to taping to ensure there will be no IRB-required revisions, provided the IRB also reviews and approves the final version.

Response: In addition to screening clinical charts, participations will be identified through; flyer advertisement, researchmatch.org, craigslist.com, and Facebook. The study will also be put on UB's Study Information Portal (SIP) website.

10.0 Procedures Involved

*10.1 Provide a description of **all research procedures or activities** being performed and when they are performed once a subject is screened and determined to be eligible. Provide as much detail as possible.*

NOTE: This should serve as a blueprint for your study and include enough detail so that another investigator could pick up your protocol and replicate the research. For studies that have multiple or complex visits or procedures, consider the addition of a schedule of events table in in your response.

Response:

Each patient will spend about 55 weeks in the trial comprising three weeks of monitoring to stabilize baselines and ensure adherence and commitment to participation before randomization into an intervention arm for an additional period of 52 weeks. Following a completed screening visit and meeting the inclusion and exclusion criteria, each patient will have the potential for a total of 16 study visits. At weeks 3, 5, 8, 16, 20, 29, 35, 44 and at 2 weeks after the end of the study, each patient will receive a phone call to document any adverse effects or other changes. There will be no long term follow-up following the end of the study.

Participants will be treated with either insulin only (control arm) for the whole duration of the study or with insulin and semaglutide weekly s.c. injection (titrated up to 1 mg or the maximum tolerated dose, see below) for the first six months then either dapagliflozin (Triple therapy arm) or placebo (dual therapy) daily will be added for an additional six months. Semaglutide will be started at a dose 0.25 mg once weekly for two weeks then increased to 0.5 mg dose. If well tolerated it will be increased to 1 mg once weekly at week 4. Dapagliflozin (or placebo) will be started at a dose of 5 mg daily at week 26 and if well tolerated increased after 1 week to 10 mg (2x5mg tablets) daily to ensure drug tolerability and to avoid any hypoglycemia or hypotension. Patients who do not tolerate the increase in dosing of either medications will be maintained at the highest tolerated dose.

As stated before, each patient will have a total of 16 study visits, depending on what group they are randomized to. Those that are randomized to receive insulin have the potential to be excused from coming to the research center at visits 4, 5, 11, and 12, at the discretion of the PI; if they are well controlled and are not experiencing any extreme hypo or hyperglycemic events, the PI has the ability to determine whether or not the patient will come to the research center at the previously mentioned four visits. If the PI decides the patient is in need of an adjustment or there are any other health issues at hand, the PI can determine whether or not the patient will come to the center.

Treatments used

1- Insulin: All patients (114 patients) will continue to self-administer their prescribed insulin as part of their standard of care. Insulin dose will be titrated during the study to control blood glucose average between 90-180 mg/dl and to avoid hypoglycemia (blood sugar <70 mg/dl) or hyperglycemia (blood sugar >250 mg/dl). Among all patients, 38 patients will be randomized (open-label) to remain on insulin therapy only (standard therapy group) while the other 76 will start the investigational therapies.
2- Semaglutide: a once weekly a GLP-1 mimetic injection (pen) indicated for type 2 diabetes. Doses to be used: start at 0.25mg then increase to 0.5mg following 2 weeks

and to 1mg weekly s.c. injection 2 weeks later. Semaglutide will be added to a background of insulin treatment for 12 months in 76 patients who will be randomized (double-blind) into 2 groups (dual and triple therapy groups) at the end of 26 weeks.

3- Farxiga (dapagliflozin): a once daily SGLT-2 inhibitor tablet indicated for type 2 diabetes. Doses used: starting with 5mg and increase after 1 week to 10mg oral administration daily. Dapagliflozin will be started according to the randomization (Interactive Web System, hosted by University of Glasgow) in 38 patients at 26 weeks as an additional treatment on a background of insulin and semaglutide therapy and end at 52 weeks (triple therapy group).

4- Placebo: dapagliflozin matching placebo (supplied by Astra Zeneca) will be started in 38 patients at 26 weeks as an additional treatment on a background of insulin and semaglutide therapy and end at 52 weeks (dual therapy group).

As shown in **Table 1,** fasting blood and urine samples will be obtained prior to drug administration (weeks 0) and at 13, 26, 39 and 52 weeks after the commencement of the study. Optional fat biopsies from the abdominal subcutaneous tissue will be collected at weeks 0, 26 and 52. Continuous Glucose Monitoring (Dexcom G6 with blinded receiver) will be performed for three 21-days periods on weeks -3 to 0, 23 to 26 and from 49 to 52 weeks. An optional 24hr blood pressure monitoring will be performed at baseline (week -1) and at 13, 25, 39 and 51 weeks in order to characterize the time course of antihypertensive effects, which may be early (i.e. independent of weight loss) as indicated by our preliminary data. High fat high caloric (HFHC) Meal challenges will be performed at baseline (week -1) and at 25 and 51 weeks with assessment of glucose excursions and postprandial glucagon (AUC) as well as postprandial inflammation and oxidative stress. Telephone calls will be conducted at weeks 3, 5, 8, 16, 20, 29, 35, 44 and at two weeks after the end of the study to document any side effects or any other patient's reported issues or comments.

Study Visits:

Screening Visit (Screening Visit, Week -8 to -4): Patients will be asked to read and understand the consent and any questions regarding the protocol will be answered. If they want to participate in the study, they will be asked to sign the informed consent form. Their medical history and current medications will be obtained as well as blood pressure and vitals. A physical examination will also be done. Blood samples will be taken in order to evaluate HbA1c, CBC, CMP, C-peptide, kidney and liver functions, ketone bodies, and urine for pregnancy status in females.

Study Visit 1 (Week -3): Participants who meet entry criteria will be scheduled to for their next visit at -3 week. Vitals will be taken including resting (min. 10 minutes) blood pressure measurement (will be repeated from same arm every visit). Insulin pump will be downloaded and/or CGM/fingerstick blood sugar will be reviewed and the appropriate insulin adjustments will be made by the investigator to target fasting blood glucose concentrations between 90-180 mg/dl if possible at all times without having additional hypoglycemia (less than 70 mg/dl). Blood sugars and ketone bodies will be measured via a fingerstick blood sample using glucose and ketones meter (Abbott Laboratories) and

daily blood sugars collected and reviewed. Urine for pregnancy status and ketone bodies assessment. We will instruct patients on how to use and insert the Continuous Glucose Monitoring System (Dexcom G6 with blinded receiver). All subjects will be advised to monitor their capillary blood glucose by CGM or fingerstick at least 4 times a day (before meals and at bedtime) through-out the study and to wear their CGM constantly for next 20 day. Carb counting technique will be reviewed and any questions answered on how to use the pump or calculate insulin doses.

Study Visit 2 (Week -1±2days): Patients will be asked to come in the fasting state (no food or calorie intake for 10-12 hours). Their vitals will be taken. Blood sugars and ketone bodies will be measured via a fingerstick blood sample using glucose and ketones meter (Abbott Laboratories) to assess their glycemic control prior to meal challenge. If their blood sugar is below 70mg/dl or more than 250mg/dl the visit will be rescheduled for an alternative date. Insulin pump will be downloaded and/or CGM/fingerstick blood sugar will be reviewed and insulin adjustment will be made if necessary. An IV port will be placed in order to obtain blood samples throughout the duration of the visit. Patients will undergo a meal challenge test (described below). At the end of the meal test, they will be asked to wear a 24 hour blood pressure monitor (optional). A 24 hr urine container will be dispensed to collect a 24 hour urine sample to bring in at the Week 0 visit. Daily Glucose logs will be reviewed.

Study visit 3 (Week 0±2days): Patients will be asked to come in the fasting state (no food or calorie intake for 10-12 hours). Vitals will be taken. Blood sugars and ketone bodies will be measured via a fingerstick blood sample using glucose and ketones meter (Abbott Laboratories) and daily blood sugars collected and reviewed. The 24 hour urine container will be collected. A fasting blood sample will be taken (45 mL) to evaluate study endpoints, CBC, CMP, HbA1c, and fructosamine. A urine sample will be collected to evaluate ketone bodies and, in females, pregnancy status. Insulin pump will be downloaded and/or CGM/fingerstick blood sugar will be reviewed and the appropriate insulin adjustments will be made by the investigator. In general, insulin dose adjustments should not be >10% from current total insulin dose or >20% from baseline (week 0) total insulin dose. They will discontinue use of the study CGM. An optional fat biopsy will be performed. We will ask them to fill out quality of life questionnaires. Study drug will be dispensed depending on what group they are randomized to. This includes either (1) staying on the insulin regimen given by their doctor, or (2) a combination of their current insulin regimen and a once daily injection of semaglutide. Semaglutide will be started on site at the 0.25 mg dose. The 24 hr blood pressure monitor will be collected.

Study Visit 4 (Week 1±2days): Vitals will be taken. Blood sugars and ketone bodies will be measured via a fingerstick blood sample using glucose and ketones meter (Abbott Laboratories) and daily blood sugars collected and reviewed. Insulin dose will be adjusted if needed as in visit 3. At any time, if HbA1c is <7%, insulin dose adjustment should not be >5%. Semaglutide 0.25mg will be injected on site. Participants will be instructed to call their research center or their doctor directly in case of any problem or untoward side effects. They will be specifically asked to call if they have unmanaged hypoglycemia (blood sugar

<70 mg/dl) or hyperglycemia (blood sugar >250 mg/dl) or if occurring in more than one occasion.

Study Visit 5 (Week 2±2days): Vitals will be taken. Blood sugars and ketone bodies will be measured via a fingerstick blood sample using glucose and ketones meter (Abbott Laboratories) and daily blood sugars collected and reviewed. Insulin dose will be adjusted if needed as in visit 3. Semaglutide 0.5mg dose will be injected on site. If they are experiencing mild nausea, they will be continued on the 0.25 mg dose for another week. Supply of the drug will be given to last until the next visit. Patients will be advised to increase the study dose to 0.5 mg a day.

Study Visit 6 (Week 4±1 week): Patients will be asked to bring in used study pens for drug accountability (to be recorded in eCRF). Vitals will be taken. Blood sugars and ketone bodies will be measured via a fingerstick blood sample using glucose and ketones meter (Abbott Laboratories) and daily blood sugars collected and reviewed. Insulin dose will be adjusted if needed. Supply of the drug will be given to last until the next visit. A urine sample will be collected to evaluate ketone bodies and, in females, pregnancy status. Patients will be advised to increase the study dose to 1.0 mg a day. If they experience mild nausea, they will be dropped from the study. A 24 hr urine container will be dispensed to collect a 24 hour urine sample to bring in at the Week 13 visit.

Study Visit 7 (Week 13±1 week): Patients will be asked to come in the fasting state (10-12 hours fasting), and to bring in your used study pens for drug accountability. Blood sugars and ketone bodies will be measured via a fingerstick blood sample using glucose and ketones meter (Abbott Laboratories) and daily blood sugars collected and reviewed. Insulin dose will be adjusted if needed as in. Supply of the drug will be given to last until visit 26. Vitals will be taken. A urine sample will be collected to evaluate ketone bodies and, in females, pregnancy status. The 24 hour urine container will be collected. A fasting blood sample will be taken (45 mL) to evaluate study endpoints, CBC, CMP, HbA1c, and fructosamine. Patients who agreed to participate in the24 hour blood pressure monitoring will be asked to wear it for the next 24 hr.

Study Visit 8 (Week 23±1 week): Patients will be asked to bring in used study pens for drug accountability. Vitals will be taken. Blood sugars and ketone bodies will be measured via a fingerstick blood sample using glucose and ketones meter (Abbott Laboratories) and daily blood sugars collected and reviewed. A urine sample will be collected to evaluate ketone bodies and, in females, pregnancy status. Insulin dose will be adjusted if needed. All subjects will be reminded to monitor their capillary blood glucose by fingerstick at least 4 times a day (before meals and at bedtime) through-out the study. CGM will be inserted and patients asked to wear their CGM constantly for next 20 day.

Study visit 9 (Week 25±1 week): Patients will be asked to come in the fasting state, and to bring in used study pens for drug accountability. Vitals will be taken. A urine sample will be collected to evaluate ketone bodies. Blood sugars and ketone bodies will be measured via a fingerstick blood sample using glucose and ketones meter (Abbott Laboratories) and daily blood sugars collected and reviewed. Insulin dose will be adjusted

if needed. HbA1c will be drawn. Supply of the drug will be given to last until the next visit. A 24 hr urine container will be dispensed to collect a 24 hour urine sample to bring in at the Week 26 visit. A HFHC Meal challenges will be performed as in visit 2. Patients who agreed to participate will be asked to wear a 24 hour blood pressure monitoring for the next 24 hr.

Participants will be provided supplies for urine ketones measurement (KetoStix) and blood ketones meter. Research coordinator will instruct the patients on the use of meter and reading of the measuring strips. Patients will be instructed to start measuring urinary and blood ketones every morning for three consecutive days prior to next visit (starting dapagliflozin treatment) and to report results at the time of their next visit.

Study visit 10 (Week 26±1 week): Patients will be asked to come in the fasting state, and to bring in used study pens for drug accountability. Previous 3 days blood and urine ketones levels will be reviewed. Patients with normal fasting urine (<+, or < small) or blood ketone (<0.6 mmol) will proceed to next treatment phase included in this visit. Patients who report higher ketone values in at least 1 out of the 3 days will have their glucose levels, insulin dose, carb intake reviewed and managed as necessary. They will be rescheduled for next week (7±1 day) and asked to repeat the 3 days testing before next visit.

Vitals will be taken. A urine sample will be collected to evaluate ketone bodies. Blood sugars and ketone bodies will be reviewed via a fingerstick blood sample using glucose and ketones meter (Abbott Laboratories) and daily blood sugars collected and reviewed. Insulin dose will be adjusted if needed. In general, insulin dose adjustments should **NOT** be >10% from current total insulin dose (or 5% if HbA1c is <7%) or >20% from baseline total insulin dose. The adjusted total insulin dose in this visit will serve as the baseline total insulin dose for the remaining 6 months of the study and future limits of dose adjustments will be based on this new baseline. The CGM will be collected. A urine sample will be collected to evaluate ketone bodies and, in females, pregnancy status. The 24 hour urine container will be collected. A fasting blood sample will be taken (45 mL) to evaluate study endpoints, CBC, CMP, C-peptide, and Fructosamine. An optional fat biopsy will be performed. We will ask patients to fill out quality of life questionnaires. At this point, those patients randomized to receive semaglutide, they will now be randomized to receive an actual dapagliflozin pill or a placebo pill that looks like dapagliflozin. If they are randomized to receive actual dapagliflozin, they will start at the 5 mg dose for one week. Supply of the study drug will be given to last until week 27.

Patients will be asked to perform a daily (preferably early morning or before bedtime) urinary test of ketone bodies using KetoStix and record all outcomes. If results of these scheduled (i.e. not done due to symptoms) ketone measurements, are small or higher (+1 or more), they will be instructed to repeat the measurement after 60 min. If consecutive measurements show moderate or higher ketones in urine, the patient will be instructed to call the research center or their doctor directly. Patients with dual glucose and ketones meters will be instructed to record and report their blood ketones in dedicated study diaries. If blood ketones are 0.6 mM or above, they should repeat the measurement within 60 min. If consecutive measurements show 0.6mm or higher ketones in blood, the patients will be instructed to call the research center or their doctor directly. For any abnormal ketone levels taken due to symptoms, the DKA consensus guidelines (Danne T et al., Diabetes Care. 2019 Feb 6) are to be followed instead (as in section 21).

Participants will be instructed to call their research center or their doctor directly in case of any problem or untoward side effects. They will be specifically asked to call if they have unmanaged hypoglycemia (blood sugar <70 mg/dl) or hyperglycemia (blood sugar >250 mg/dl) or if occurring in more than one occasion. Participants will be instructed to remain hydrated and report any genital mycotic or urinary tract infections.

Study visit 11 (Week 27±2 days): Patients will be asked to bring in used study pens for drug accountability (recorded in eCRF). Vitals will be taken. Daily blood sugars and urinary ketones measurements using KetoStix will be collected and reviewed. Blood sugars and ketone bodies will be measured via a fingerstick blood sample using glucose and ketones meter (Abbott Laboratories). A urine sample will be collected to evaluate urine ketone level. Insulin dose will be adjusted if needed. In general, insulin dose adjustments should not be >10% from current total insulin dose (or 5% if HbA1c is <7%) or >20% from NEW baseline total insulin dose established at visit 10. If at any time during the study total insulin dose reduction is >20% and total insulin dose in <0.40 U/Kg, additional monitoring protocol (see later) will be implemented. Supply of the drug will be given to last until week 39. If patients are receiving actual dapagliflozin, they will now start at the 10 mg dose for the remainder of the study.

Study Visit 12 (Week 31±1 week): Patients will be asked to bring in used study pens and tablets for drug accountability. Vitals will be taken. Daily blood sugars and urinary ketones measurements using KetoStix will be collected and reviewed. Blood sugars and ketone bodies will be measured via a fingerstick blood sample using glucose and ketones meter (Abbott Laboratories). A urine sample will be collected to evaluate ketone bodies and, in females, pregnancy status. Insulin dose will be adjusted if needed as in visit 11. A blood sample will be taken (15 mL) to evaluate CBC and CMP. A 24 hr urine container will be dispensed to collect a 24 hour urine sample to bring in at the Week 39 visit.

Study visit 13 (Week 39±1 week): Patients will be asked to come in the fasting state, and to bring in used study pens and tablets for drug accountability. Vitals will be taken. Daily blood sugars and urinary ketones measurements using KetoStix will be collected and reviewed. Blood sugars and ketone bodies will be measured via a fingerstick blood sample using glucose and ketones meter (Abbott Laboratories). Insulin dose will be adjusted if needed as in visit 11. Supply of the drug will be given to last until the end of the study. A urine sample will be collected to evaluate ketone bodies and, in females, pregnancy status. The 24 hour urine container will be collected. A fasting blood sample will be taken (45 mL) to evaluate study endpoints, CBC, CMP, HbA1c, and Fructosamine. Patients who have provided consent will be asked to wear a 24 hour blood pressure monitor.

Study visit 14 (Week 49±1 week): Patients will be asked to bring in used study pens and tablets for drug accountability. Vitals will be taken. Daily blood sugars and urinary ketones measurements using KetoStix will be collected and reviewed. Blood sugars and ketone bodies will be measured via a fingerstick blood sample using glucose and ketones meter (Abbott Laboratories). A urine sample will be collected to evaluate ketone bodies and, in females, pregnancy status. Insulin dose will be adjusted if needed as in visit 11. All subjects will be reminded to monitor their capillary blood glucose by fingerstick at least 4 times a

day (before meals and at bedtime) through-out the study. CGM will be inserted and patients asked to wear their CGM constantly for the next 20 days.

Study visit 15 (Week 51±1 week): Patients will be asked to come in the fasting state, and to bring in used study pens and tablets for drug accountability. Vitals will be taken. Daily blood sugars and urinary ketones measurements using KetoStix will be collected and reviewed. Blood sugars and ketone bodies will be measured via a fingerstick blood sample using glucose and ketones meter (Abbott Laboratories). A urine sample will be collected to evaluate urine ketone levels. Insulin dose will be adjusted if needed as in visit 11. A 24 hr urine container will be dispensed to collect a 24 hour urine sample to bring in at the Week 52 visit. A HFHC Meal challenges will be performed as in visit 2. Patients will be asked to wear the optional 24 hour blood pressure monitor.

Study visit 16 (Week 52±2 days): Patients will be asked to come in the fasting state, and to bring in used study pens and tablets for drug accountability. Vitals will be taken. Daily blood sugars and urinary ketones measurements using KetoStix will be collected and reviewed. Blood sugars and ketone bodies will be measured via a fingerstick blood sample using glucose and ketones meter (Abbott Laboratories). Insulin dose will be adjusted if needed. The CGM will be collected. An optional fat biopsy will be performed. A urine sample will be collected to evaluate ketone bodies and, in females, pregnancy status. The 24 hour urine container will be collected. A fasting blood sample will be taken (45 mL) to evaluate study endpoints, CBC, CMP, HbA1c, C-peptide, and Fructosamine. We will ask the patients to fill out quality of life questionnaires. The patient will receive a physical. After this visit, they will be discharged from the study.
Safety phone calls will be conducted at weeks 3, 5, 8, 16, 20, 29, 35, 44 and at two weeks after the end of the study to document any side effects or other changes.

Table 1: Schedule of visits and testing procedures.

Visit #	-	1	2	3	4	5	6	7	8	9	10	11	12	13	14	15	16
Week#(±2-7 days as per text)	-8 to -4	-3	-1	0	1	2	4	13	23	25	26	27	31	39	49	51	52
Study Procedures	Screening	Run-in period		Randomization, start semaglutide							Randomize/Start dapagliflozin/ placebo						End of study
History	X																
Physical	X																X
Vitals	X	X	X	X	X	X	X	X	X	X	X	X	X	X	X	X	X
CBC	X			X				X			X		X	X			X
CMP	X			X				X			X		X	X			X
C-Peptide	X										X						X
Kidney and liver profiles	X			X				X			X		X	X			X
Pregnancy test	X	X		X			X	X	X		X		X	X	X		X
Study drug titration						X	X					X					
Dispense medications				X	X	X	X	X			X	X		X			
HbA1c	X			X				X		X				X			X
Fructosamine				X				X			X			X			X
24 hour BP (optional)			X					X		X				X		X	
CGM		X	X						X	X					X	X	
Daily Fingerstick glucose review		X	X	X	X	X	X	X	X	X	X	X	X	X	X	X	X
Daily urinary ketones review											X	X	X	X	X	X	X
Insulin dose review		X	X	X	X	X	X	X	X	X	X	X	X	X	X	X	X
Ketone bodies (blood/urine)	X	X	X	X	X	X	X	X	X	X	X	X	X	X	X	X	X
Adverse Event reporting		X	X	X	X	X	X	X	X	X	X	X	X	X	X	X	X
Meal Challenge			X							X						X	
Blood endpoints				X				X			X			X			X
Fat biopsy (optional)				X							X						X
24 hr Urine				X				X			X			X			X
Quality of life questionnaires				X							X						X

Drug Blinding:
The randomizations will be performed using an Interactive Web System, hosted by University of Glasgow. One hundred and fourteen (114) patients will be randomized as follows: participants will first be randomized to semaglutide plus usual insulin therapy or insulin therapy only (no placebo for semaglutide or other placebo injection will be provided), in a 2:1 ratio. Those who remain on semaglutide at six months will then be randomized in a 1:1 ratio to receive dapagliflozin or matched placebo. Each randomization will be stratified by study site and the second randomization will also include HbA1c stratification. The randomization schedules will be generated by a statistician with no other involvement in the trial, using the method of randomized permuted blocks. Only those staff responsible for the maintenance of the randomization system will have access to the randomization sequences during the trial. The first randomization (which will be revealed to the researcher at the point of randomization) will use random block sizes to reduce predictability. Researchers at study sites will have no knowledge of the next allocation to be issued. The second randomization (which will be double-blind) will use a different. At the point of randomization, the researcher will be given a medication pack ID number, but will not know whether that medication pack contains Dapagliflozin or placebo. For each randomization, a web-based randomization system (developed and maintained by the Robertson Centre for Biostatistics) will be used. The system will include an emergency unblinding facility for the dapagliflozin/placebo randomization
Astra Zeneca Inc. will supply dapagliflozin and matching placebo tablets in unlabeled and identical packaging; semaglutide pens will be obtained commercially and will be used in an open label fashion.

Breaking of the study blind will be performed only: (i) for SUSARs; and, (ii) where knowledge of the treatment is considered by local health personnel absolutely necessary for further management of the patient. A central unblinding facility based at the Robertson Centre for Biostatistics, University of Glasgow, will be available by telephone. Notification of all unblinding will be sent to the Chief Investigator

During the course of the trial there will be no attempt to identify group assignment for any participant (except for unblinding in the case of safety concerns) and the investigators will execute the protocol in the manner it was approved independent of the group assignment.

Drugs will be stored in a locked cabinet or locked temperature controlled refrigerator at 4C at the research facility of the Diabetes and Endocrinology Center of WNY. IND application for use of all drugs (dapagliflozin and semaglutide) in type 1 diabetes will be submitted to FDA and no study procedure will start before all local and federal approvals are obtained.

Clinical and Laboratory Procedures

1) **CMP, CBC, HbA1c, fructosamine, glucose, glucagon, GLP-1 and FFA measurements:** Blood CMP, CBC, HbA1c, fructosamine and glucose will be measured through Quest diagnostic (US site) and NHS labs (UK site). Free fatty acid levels will be measured by a colorimetric assay (Wako, Richmond, VA). GLP-1 and

glucagon will be measured from samples collected in protease and DPP-IV inhibitors by ELISA kits from Millipore.

2) **Meal Challenge test:** In order to assess the postprandial changes induced by dapagliflozin, semaglutide or the combination of both compared to insulin only, a meal challenge will be performed before starting treatments, at six months and at end of study using a 910 Calorie High fat High carbohydrate meal (HFHC) as in several of our previous studies (HFHC meal includes an egg muffin sandwich, a sausage muffin sandwich and two hash browns which contain 88g carbohydrate, 51 g fat (33% saturated) and 34 g protein). Participants will be asked to attend fasting prior to breakfast insulin bolus; basal insulin will continue during the duration of the meal, however patients will not bolus until the 3 hour mark unless blood glucose values reach above 400 mg/dL. Sequential blood samples will be obtained at 0, 15, 30, 45, 60, 90, 120, 150 and 180 min after consuming the meal. Samples at 15, 30, 45, 90 and 150 min will be 5ml while those at 0, 60, 120 and 180min will be 30 ml (total volume=145 ml). Spot urine will be collected at baseline, 1 and 3hr. Blood will be collected from an indwelling intravenous canula in a superficial forearm vein.
3) **Optional 24 hour Ambulatory BP monitoring:** Participants will be instructed on the use of 24 hour ambulatory BP monitoring device. This device will measure BP and pulse every 30 minutes for 24 hours. In addition to the scheduled measurements there will be an option for additional manual measurements if systolic blood pressure is >160 or <90 mmHg.
4) **Optional Fat aspiration procedure:** Abdominal subcutaneous fat tissue aspiration will be performed at a 10 cm distance from umbilicus. Participants will be asked not to take aspirin or NSAIDS in the previous 72 hours - if they have, the procedure will not be done. Skin will be prepared with povidone-iodine (Betadine) and alcohol. A sterile drape will be placed around the appropriate area. 3cc of 1% lidocaine will then be administered subcutaneously (the dose will not exceed 4.5mg/kg body weight). After adequate anesthesia has been achieved, aspiration of fat tissue will then be with a 3-holed canula (Tulip Instrumentation, length: 15cm, diameter: 2.1mm) fixed to a 10mL syringe. After getting adequate fat tissue (500mg-3g), the puncture site will be pressed for at least ten minutes before the patient rises up from supine position (to minimize bruising). Participants will then be discharged home. Adipose tissue will be centrifuged to remove blood and fluid contaminants. Upper adipose tissue will be collected into a separate sterile tube, washed twice with cold sterile Phosphate Buffered Saline (PBS) and centrifuged to remove the PBS. The adipose tissue sample will be weighed and approximately 500 mg transferred to a separate tube for analysis. Total RNA, nuclear extracts and total cell lysates will be prepared.
5) **Questionnaires:** Participants will be asked to complete the standard Diabetes Specific Quality of Life Scale (DSQOLS) and Problem Areas In Diabetes survey (PAID). Questionnaires attached at the end of this plan.
6) **MNC isolation (for future ancillary studies):** Blood samples will be collected in Na-EDTA as an anticoagulant. Three and a half mL of anticoagulated blood sample are carefully layered over 3.5 mL of PMN medium (Cedarlane Laboratories, Hornby, ON) and then centrifuged. At the end of this process, two bands separate out at the top of the RBC pellet. The top band consists of MNC, while the bottom consists of PMN. The

MNC band is harvested and washed twice with Hank's balanced salt solution (HBSS). This method provides yields greater than 95% pure PMN and MNC suspensions.

7) **NFκB DNA binding activity (for future ancillary studies):** Nuclear NFκB DNA binding activity is measured by EMSA. Nuclear extract is prepared from MNC and adipose tissue by high salt extraction as described by Andrews et al (19). The specificity of the bands is confirmed by supershifting these bands with specific antibodies against Rel-A (p65) and p50 (Santa Cruz Biotechnology, CA) and by competition with cold oligonucleotides. DNA binding activity of NFκB will be adjusted to Oct1 DNA binding activity to correct for any variable.
8) **Plasma concentrations of inflammatory mediators (for future ancillary studies):** Concentrations of TNFα, IL-1β, IL-18, adiponectin, IL-1RA will be measured in plasma using ELISA kits (R&D Systems, MN). CRP and SAA concentrations will be measured using an ELISA kit from Alpha Diagnostics International Inc. (San Antonio, TX).
9) **Plasma vasodilators and vasoconstrictors (for future ancillary studies):** Plasma ANP, NT-proBNP, b-NP, cGMP, cAMP, angiotensinogen, renin and angiotensin II concentrations will be measured by commercially available ELISA kits.
10) **15-isoprostane F_{2t}** (also known as 8-epi-PGF2α or 8-iso-PGF2α) **(for future ancillary studies):** will be measured in urine samples using an ELISA kit from Oxford Biomedical Research (Oxford, MI).
11) **Quantification of mRNA in MNC and adipose tissue (for future ancillary studies).** Total RNA is isolated from MNC and adipose tissue using commercially available RNAqueous®-4PCR Kit (Ambion, Austin, TX). The quality and quantity of the isolated RNA is determined before using the RNA. Real Time RT-PCR is performed using Cepheid Smart Cycler (Sunnyvale, CA) in which 2 µL cDNA, 10 µL Sybergreen Master mix (Qiagen, CA) along with 0.5 µL of 20 µM gene specific primers for IL-1β, IL-RA, TLR-4, TNFα, IL-12, SOCS-3 and JNK-1 (Life Technologies, MD) are used. The specificity and the size of the PCR products are tested by adding a melt curve at the end of the amplifications and by running it on a 2% agarose gel. All values are normalized to 18S expression and β-actin.

10.2 Describe what data will be collected.

NOTE: For studies with multiple data collection points or long-term follow up, consider the addition of a schedule or table in your response.

Response:

Data will be collected as described in table 1 above. The following aspects of the study will be performed and data collected and reported by patients:

1- A CGM device will be provided for use for 3 20-days periods. Detailed instructions on changing the sensor at 10 days will be provided. Patients will be instructed to wear their CGM constantly during the assigned study periods. They will be asked to keep a diary of their food intake to measure their carbohydrate intake during the CGM period. Insulin doses will be titrated aiming for blood glucose between 90-180 mg/dl while avoiding hypoglycemia (<70 mg/dl) or hyperglycemia (>250 mg/dl).

2- Participants who agree to participate in the 24-hr ambulatory blood pressure monitoring will be provided with a 24-hour ambulatory BP monitor to be worn for 24 hours during week -1, 13, 25, 39 and 51.
3- All participants will be asked to monitor capillary blood glucose by finger stick before each meal and at bedtime.
4- For the entire duration of the study, the patients will maintain a diary to record any hypoglycemia and other untoward side effects e.g. nausea, change in appetite.
5- Starting at the 6 months mark, participants will be asked to perform a daily urinary test of ketone bodies using KetoStix and record all outcomes. If results are moderate to large, they will be instructed to repeat the test and to call the research center or their doctor directly if results are confirmed. Patients with dual glucose and ketones meters will be instructed to record and report their blood ketones as well. If blood ketones are above 0.6mM, they should repeat the test and contact the research center of their doctor if levels are confirmed.

10.3 List any instruments or measurement tools used to collect data (e.g. questionnaire, interview guide, validated instrument, data collection form).

Include copies of these documents with your submission.

Response: Source documents will be used to collect patient information. The CGM or insulin pump will be used to collect glucose and insulin data. Glucose and ketone meter will be used to measure glucose and ketone in blood. KetoStix for measuring ketones in urine. Blood pressure monitor will be used for 24 hour blood pressure reading. Glucose, food and urinary ketones diaries will be provided for patients to record their results.

10.4 Describe any source records that will be used to collect data about subjects (e.g. school records, electronic medical records).

Response: electronic medical records and research files.

10.5 Indicate whether or not ***individual*** *subject results, such as results of investigational diagnostic tests, genetic tests, or incidental findings will be shared with subjects or others (e.g., the subject's primary care physician) and if so, describe how these will be shared.*

Response: Individual participant lab results will be disclosed to the participant upon their request. If the participant requests documentation be shared with another physician, physician office or hospital the participant must come to the research center to collect said documentation or the documentation can be mailed to their given home address.

Patients will be assigned a unique study number. All identifiable personal information will be stored locally in locked filing cabinets. Data entered on the

electronic Case Report Form will only be identified by the unique study number. Thus, no personal information will be shared with the data center (Robertson Centre for Biostatistics, University of Glasgow, UK).

*10.6 Indicate whether or not **study** results will be shared with subjects or others, and if so, describe how these will be shared.*

Response: Not Applicable. Study results will not be shared with the subjects. However, unidentifiable study results could be published in the form of a manuscript or abstract and will be reported to JDRF, Astra Zeneca, and to clinicaltrials.gov and other regulatory agencies including FDA.

11.0 Study Timelines

11.1 Describe the anticipated duration needed to enroll all study subjects.

Response: Accrual is expected to be at the rate of 4 subjects/month/site, thus we should complete enrollment of 57 patients/site in approximately 13-15 months. We expect to complete all study clinical activities in 30 months.

11.2 Describe the duration of an individual subject's participation in the study. Include length of study visits, and overall study follow-up time.

Response: 55 Weeks. Study visits will be 60-90 minutes long except for visits 2, 9 and 15 which will be about 6hr long each

11.3 Describe the estimated duration for the investigators to complete this study (i.e. all data is collected and all analyses have been completed).

Response: 36 Months

12.0 Setting

12.1 Describe all facilities/sites where you will be conducting research procedures. Include a description of the security and privacy of the facilities (e.g. locked facility, limited access, privacy barriers). Facility, department, and type of room are relevant. Do not abbreviate facility names.

NOTE: Examples of acceptable response may be: "A classroom setting in the Department of Psychology equipped with a computer with relevant survey administration software," "The angiogram suite at Buffalo General Medical Center, a fully accredited tertiary care institution within New York State with badge access," or, "Community Center meeting hall."

Response:

1) Research will be conducted at the Diabetes Endocrinology Research Center of WNY, located at *1000 Youngs Road, Suite 105, Williamsville NY 14221* and at the CTRC located in 875 Ellicott St. Buffalo NY14203.The Diabetes Research Center has facilities and exam rooms available for insulin pump download, CGM device download, meal and infusion studies and presence of study coordinator and registered nurse for data collection and blood work at all times. One of the investigators will be available at all times to address patients' related issues. CTRC location is a fully equipped laboratory with Equipment include ultra-low freezers for sample storage, centrifuges, microscopes for sample preparation, infusion pumps, ELISA, PCR and immunoblotting instrumentation.

2) Research will also be conducted at the NHS Greater Glasgow and Clyde Clinical Research Facility (CRF, Glasgow Royal Infirmary, UK and at the Diabetes Centre (Clinic D), Stobhill Ambulatory Care Hospital, Glasgow, UK. There are facilities and exam rooms available for insulin pump download, CGM device download, meal and infusion studies and presence of research nursing staff for data collection and blood work at all times. One of the investigators will be available at all times to address patients' related issues. The CRF has equipment for sample preparation, infusion pumps, ultra-low freezers for sample storage and centrifuges. ELISA, PCR and immunoblotting facilities are available in adjacent NHS and University facilities.

12.2 For research conducted outside of UB and its affiliates, describe:

- *Site-specific regulations or customs affecting the research*
- *Local scientific and ethical review structure*

NOTE: This question is referring to UB affiliated research taking place outside UB, i.e. research conducted in the community, school-based research, international research, etc. It is not referring to multi-site research. UB affiliated institutions include Kaleida Health, ECMC, and Roswell Park Cancer Institute.

Response:

☒ **N/A:** This study is not conducted outside of UB or its affiliates.

13.0 Community-Based Participatory Research

13.1 Describe involvement of the community in the design and conduct of the research.

NOTE: Community-Based Participatory Research (CBPR) is a collaborative approach to research that equitably involves all partners in the research process and recognizes the unique strengths that each brings. CBPR begins with a research topic of importance to the community, has the aim of combining knowledge with action and achieving social change to improve health outcomes and eliminate health disparities.

Response:

☒ **N/A:** This study does not utilize CBPR.

13.2 Describe the composition and involvement of a community advisory board.

Response:

☒ **N/A:** This study does not have a community advisory board.

14.0 Resources and Qualifications

14.1 Describe the qualifications (e.g., education, training, experience, expertise, or certifications) of the Principal Investigator ***and*** *staff to perform the research. When applicable describe their knowledge of the local study sites, culture, and society. Provide enough information to convince the IRB that you have qualified staff for the proposed research.*

NOTE: If you specify a person by name, a change to that person will require prior approval by the IRB. If you specify a person by role (e.g., coordinator, research assistant, co-investigator, or pharmacist), a change to that person will not usually require prior approval by the IRB, provided that the person meets the qualifications described to fulfill their roles.

Response: All study personnel are educated, trained, and licensed as required for their delegated role in this study. All study personnel have also received the required university training and will be trained by the PI before the study starts. The PIs, CO-investigators and research team have conducted similar studies in this patient population using similar agents and have the expertise to execute the study protocol and manage any adverse events.

Describe other resources available to conduct the research.

14.2 Describe the time and effort that the Principal Investigator and research staff will devote to conducting and completing the research.

NOTE: Examples include the percentage of Full Time Equivalents (FTE), hours per week. The question will elicit whether there are appropriate resources to conduct the research.

Response: The principal investigator supervises the research project and weekly research meetings are conducted to discuss the recruitment rate, resolve and discuss issues related to the conduct, safety, analysis of the study and related publications. PI is expected to spend 15% of his academic time on this research. The co-investigators, study coordinator and research staff provide coverage to the research related activity for 365 days a year.

14.3 Describe the availability of medical or psychological resources that subjects might need as a result of anticipated consequences of the human research, if applicable.

NOTE: One example includes: on-call availability of a counselor or psychologist for a study that screens subjects for depression.

Response: Available medical literature will be provided as deemed appropriate or requested by patient through UB libraries, PubMed, Google scholar as all the investigators have access to medical literature through listed resources above

The patient will also have access to physician (Investigators and Co-Investigators) who will be available to address any adverse effects or other questions during the course of the study who will be available to address any adverse effects or other questions during the course of the study

14.4 Describe your process to ensure that all persons assisting with the research are adequately informed about the protocol, the research procedures, and their duties and functions.

Response: Before beginning of enrollment all research staff will be asked to review the protocol and a training session will be conducted to provide protocol details, explain technical points and answer staff questions. All training session will have a sign in sheet to document such training. Continuous education through training meetings, conferences and discussions will continue through the study period and when revision are made to protocol.

15.0 Other Approvals

15.1 Describe any approvals that will be obtained prior to commencing the research (e.g., school, external site, funding agency, laboratory, radiation safety, or biosafety).

Response: FDA IND filling is required.

☐ **N/A:** This study does not require any other approvals.

16.0 Provisions to Protect the Privacy Interests of Subjects

16.1 Describe how you will protect subjects' privacy interests during the course of this research.

NOTE: Privacy refers to an individual's right to control access to him or herself. Privacy applies to the person. Confidentiality refers to how data collected about individuals for the research will be protected by the researcher from release. Confidentiality applies to the data.

Examples of appropriate responses include: "participant only meets with a study coordinator in a classroom setting where no one can overhear", or "the participant is reminded that they are free to refuse to answer any questions that they do not feel comfortable answering."

Response: Our clinical providers involved in the study will identify potential patients for recruitment from the Diabetes-Endocrinology Center of WNY Clinics according to the inclusions and exclusion criteria and through advertisements. Patient who qualify will be asked in private during their one on one consultation time with the physician if they wish to participate in the research study. If the patient agrees, the research coordinator will contact them privately for a possible telephone screening and ask if time is appropriate for this discussion. The patients who call for potential participation in the study due to advertisement flyers will be screened over the phone with the research coordinator, using our telephone screening form.

When the patient is being seen at our clinics for the first time they sign the "Consent to use and disclosure of protected health information" form which clearly states that their protected health information (PHI) can be used for review in preparation for possible research. Additionally, a partial HIPAA waiver will be obtained to access medical records when screening for patients.

If the patient passes the telephone screening, they will be asked to make an appointment to review and sign the consent. Patient will do this in a private room in the research unit and will be allowed to discuss the consent in detail with the research coordinator and or study doctor. Patient will be notified that it is completely voluntary to participate in the research study and can withdraw at any time.

We will not be accessing any medical information of the patients for whom the services are not provided by our clinic providers before partial HIPAA is obtained or IC is signed.

16.2 Indicate how the research team is permitted to access any sources of information about the subjects.

*NOTE: Examples of appropriate responses include: school permission for review of records, consent of the subject, HIPAA waiver. This question **does apply** to records reviews.*

Response: Consent of the subject and partial HIPAA waiver.

17.0 Data Management and Analysis

17.1 Describe the data analysis plan, including any statistical procedures. This section applies to both quantitative and qualitative analysis.

Response:

Data (e.g. date of diagnosis, past medical history, drug treatment) extracted from local clinical records will be entered on to the eCRF under the study-specific coded identifier according to structured questions as stipulated in the Study Protocol. Biological samples (blood, urine and adipose tissue aspirates) will be collected from the study participants at the study visits according to the Schedule of Assessments in the Study Protocol.

Measures relating to safety and routine care of the participants (e.g., biochemical measures of glycemic and lipidemic control, kidney function/ fluid balance, hepatic function, hematological variables) will be analysed at local clinical laboratories in order that they are available to study nurses and physicians. The confidentiality of these data will be secure, meeting or exceeding the regulations of the relevant Ethical Review Committees/ Institutional Review Boards. Samples for measurement of key study outcomes will also be measured at the central laboratory at the University of Buffalo. Blood, urine and other samples will be aliquoted into cryovials at site for freezing and storage. Electronic stored material (e.g., patient records, data from blood and urine samples, and data from Continuous Glucose Monitoring) will only be stored using the study specific coded identifier. Data will not be identifiable at the participant level without additional access to the code. The code will be available only to the investigators and the personnel performing the study. Access to any data held electronically will require study specific log-in codes. These are managed and logged by the Robertson Centre for Biostatistics, University of Glasgow, UK

Statistical plan. The primary objective of the proposed research is to evaluate the effect of addition of dapagliflozin to semaglutide on a background of insulin treatment in type 1 diabetes patients on HbA1c at six months following start of dapagliflozin. The similarities between the study groups, baseline values for subject's demographics and study endpoints will be compared using appropriate parametric tests. Transformations of the data in order to meet statistical assumptions may be considered. The primary endpoint of the study is to detect a difference in mean HbA1c change after six months of dapagliflozin or placebo addition to semaglutide and insulin treatment. Statistical analysis will be carried out using t-tests to detect difference between groups or the Wilcoxon test for paired data. Tests will be two-sided and tested at the 0.05 level. The results will be summarized as mean±SE. All randomized patients will be included in the analysis of the primary end point based on an Intent-to-treat model.

The secondary endpoints will be to compare changes in HbA1c between each of the triple therapy and dual therapy groups compared to standard of care group, mean weekly glucose concentrations, percent time spent in different glycemic thresholds, variability as measured by standard deviation, insulin dose, body weight, carbohydrate intake, blood pressure, and 24-hour blood pressure and QOL changes among the groups. A reduction in the glucose and glucagon AUC following the meal will constitute another secondary end point in this setting, ANCOVA analysis will be used. Since the secondary end-points are pre-specified and may as well be correlated, multiplicity control for multiple tests across secondary endpoints may not be necessary. However, Holm-Bonferroni correction

for multiplicity testing will be implemented if additional endpoints are tested or if the pre-specified secondary end-points are found to be not correlated.

17.2 If applicable, provide a power analysis.

NOTE: This may not apply to certain types of studies, including chart/records reviews, survey studies, or observational studies. This question is asked to elicit whether the investigator has an adequate sample size to achieve the study objectives and justify a conclusion.

Response:

Sample size determination is based in part on our current data showing that in type 1 diabetes, treatment with a GLP-1 agonist, liraglutide (1.2 and 1.8mg doses) for 3 months leads to HbA1c suppression by an average of 0.6% (SD= 0.5%). Dapagliflozin was recently shown to reduce HbA1c by 0.45% in type 1 diabetes patients (13). Additionally, empagliflozin treatment for 8 weeks was shown to reduce HbA1c by 0.4% in type 1 diabetics (20, 21) and our current data showing additional improvement in HbA1c by an additional 0.66% at 12 weeks when dapagliflozin was added to liraglutide and insulin therapy. Conservatively estimating that the combination therapy will suppress HbA1c by 0.5% (SD =<0.7%) compared to semaglutide alone (t-test), a sample size of 38 (32 evaluable patients) in each group will be sufficient to provide adequate power ($\beta = 0.2$) to detect a statistically significant difference ($\alpha = 0.05$) taking in consideration a 15% dropout rate.

17.3 Describe any procedures that will be used for quality control of collected data.

Response:

An electronic case report form (e-CRF) will be used to collect study data at each site. The e-CRF will be developed by the study Data Centre at the Robertson Centre for Biostatistics, University of Glasgow. Access to the e-CRF will be restricted, with only authorised site-specific personnel able to make entries or amendments to their patients' data. It is the investigator's responsibility to ensure completion and to review and approve all data captured in the e-CRF.

Data will be validated at the point of entry into the e-CRF and at regular intervals during the study. Data discrepancies will be flagged to the study site coordinator and any data changes will be recorded in order to maintain a complete audit trail (reason for change, date change made, who made change).

The Robertson Centre for Biostatistics at the University of Glasgow will be responsible for collating and cleaning the data for the study. The Robertson Centre will also be responsible for data back-up and security. This center will also manage the electronic reporting of SUSARS on behalf of the sponsor.

To enable evaluations and/or audits from regulatory authorities, the investigators agree to keep records, including the identity of all participating subjects (sufficient information to link records, all original signed informed consent forms serious adverse event forms, source documents, and detailed records of treatment disposition. The records should be retained by the study country coordinators and investigator according to ICH GCP, local regulations, or as specified in the Clinical Study Agreement, whichever is longer.

Data from the study will be stored by the Chief Investigator for a minimum of 10 years. A final report of the study will be provided to the FDA and the MHRA as per requirements.

- No interim analysis is planned for efficacy so termination of study will be related to safety. Significant increases in hypoglycemic or DKA events as reviewed by DSMB might trigger termination or modification of study.
- Last observation carry forward will be used to fill missing data for the primary end point and some secondary end points. Missing data will not be filled for other endpoints.
- Any deviation(s) from the original statistical plan will be described and justified in the protocol and/or in the final report.
- All subjects entered into treatment will analyzed for primary end point on ITT bases. For secondary endpoints, analysis of completed and evaluable patients will performed per-protocol.

18.0 Confidentiality

A. Confidentiality of Study Data

Describe the local procedures for maintenance of confidentiality of ***study data and any records that will be reviewed for data collection****.*

18.1 A. Where and how will all data and records be stored? Include information about: password protection, encryption, physical controls, authorization of access, and separation of identifiers and data, as applicable. Include physical (e.g. paper) ***and*** *electronic files.*

Response: All data records will be stored on protected computers with unique passwords issued for each of the study team members and or in locked cabinets within the research center. These files will only be accessible by authorized study personnel.

18.2 A. How long will the data be stored?

Response: De-identified data storage has no expiration date and will be stored for a minimum of 7 years. The researchers may continue to rely on this for future use in research study.

18.3 A. Who will have access to the data?

Response: Those physicians, nurses, and laboratory staff that are on all documentation for the study will have access to the data.

18.4 A. Who is responsible for receipt or transmission of the data?

Response: Those physicians, nurses, and laboratory staff that are on all documentation for the study will have access to the data and can handle transfer of data. Study data will be transferred electronically (through eCRFs) to Robertson Centre for Biostatistics, University of Glasgow, UK

18.5 A. How will the data be transported?

Response: All data are stored at locally and will be transferred to Robertson Centre for Biostatistics, University of Glasgow, UK for data management and analysis. The transfer will be done through electronic CRFs processed on secure network.

Data will also be transported unless when is being archived. At that point files will be transferred to Iron Mountain for storage and archiving.

B. Confidentiality of Study Specimens

*Describe the local procedures for maintenance of confidentiality of **study specimens**.*

☐ **N/A:** No specimens will be collected or analyzed in this research.
(Skip to Section 19.0)

18.6 B. Where and how will all specimens be stored? Include information about: physical controls, authorization of access, and labeling of specimens, as applicable.

Response: The specimens will be stored in the laboratory located at *1000 Youngs Road, Suite 105, Williamsville NY 14221*and at the CTRC located in 875 Ellicott St. Buffalo NY14203. Samples will be stored in a locked -80º C freezer. All specimens collected at the Glasgow site will be stored in NHS -80º C freezers within the eCRF until shipping to Buffalo (World Courier).

18.7 B. How long will the specimens be stored?

Response: Specimens storage has no expiration date and will be stored for a minimum of 7 years. The researchers may continue to rely on this for future use in research study

18.8 B. Who will have access to the specimens?

Response: Those physicians, nurses, and laboratory staff that are on all documentation for the study will have access to the specimens

18.9 B. Who is responsible for receipt or transmission of the specimens?

Response: Those physicians, nurses, and laboratory staff that are on all documentation for the study will have access to the specimens and can handle transfer of samples. Sample/specimens will transported from University of Glasgow site to the university of Buffalo CTRC site (875 Ellicott St, Buffalo, NY 14203) for storage in -80C until analyzed.

18.10 B. How will the specimens be transported?

Response: All study samples will be centrally stored in UB CTRC site. Therefore, samples collected at the UK site will be transported for analysis and final long-term storage. Transport will be done so using dry ice in a properly labeled Styrofoam container by the laboratory technician. Agreement regarding shipping and receiving the specimens form UK site will be part of the subcontract agreement.

19.0 Provisions to Monitor the Data to Ensure the Safety of Subjects

☐ **N/A:** This study is not enrolling subjects, or is limited to records review procedures only. This section does not apply.

NOTE: Minimal risk studies may be required to monitor subject safety if the research procedures include procedures that present unique risks to subjects that require monitoring. Some examples include: exercising to exertion, or instruments that elicit suicidality or substance abuse behavior. In such cases, N/A is not an acceptable response.

19.1 Describe the plan to periodically evaluate the data collected regarding both harms and benefits to determine whether subjects remain safe.

Response:

An independent Data safety and monitoring board (DSMB) will be established for this trial. The board will consist of independent faculty members from UB and other institutions with sufficient knowledge of the field of type 1 diabetes but not

involved in the study. Data Management team at Robertson Centre will periodically provide the data on which to base regular reports to the Data and Safety Monitoring Board. The actual reports will then be generated by the unblinded statistician. The DSMB looks only at safety endpoints and not at futility. The DSMB will convene every six months to review safety end points, side-effects experienced by study subjects and any adverse events reported. The site PIs, CO-Is and study coordinator(s) will also continuously monitor the data and safety of subjects enrolled in the study. The site PIs and/or the DSMB may recommend to terminate the study or specific aspects of the study if clear harm is observed with one treatment option compared to standard of care or placebo. Specific safety endpoints that will be reviewed include increases in hypoglycemic and ketosis/DKA events.

19.2 Describe what data are reviewed, including safety data, untoward events, and efficacy data.

Response:

Assessment of Efficacy

1- glycemic indices including HbA1c, fructosamine, post-prandial glucose excursions and glucagon. CGM data will be collected as efficacy parameter of glucose control. Total insulin dose will be also documented and analyzed.
2- body weight
3- blood pressure and use of blood pressure medications
4- QOL questionnaires.

The timing of recording of the above efficacy parameters is described in table 1.

Assessment of Safety

The following safety assessments assays and measurement will be reviewed:

1- Vitals, CBC, CMP and markers kidney and liver function will be collected at screening, during and at end of the study as in table 1.
2- Assessment of hypoglycemia: Glucose data will be reviewed from CGM and daily finger stick and during visits.
3- Assessment of ketosis: Ketones levels will be reviewed from daily urinary ketone diaries or blood ketone meters and from urine and blood at the time of each visit
4- Reports of side effects by patients.
5- Insulin dose titrations and pump downloads at each visit

The methods and timing for assessing, recording, and analyzing safety parameters.

The DSMB will convene every six months to assess the side-effects experienced by study subjects and any adverse events reported. The PIs, CO-Is and study coordinator(s) will also continuously monitor the safety of subjects enrolled in the study during their visits and during the safety phone calls.

Procedures for eliciting reports of and for recording and reporting adverse event and inter-current illnesses.

All adverse events will be reported at the time of the visit or by telephone when it occurs. Medical events that occur between the signing of the Informed Consent and the first intake of study drug will be documented in the medical history. Participants will be asked to report any adverse events in general, non-directed questioning. For each AE volunteered by the subject, the investigator will obtain all the information required to complete the adverse event page of the eCRF, in accordance with the guidelines that accompany it. All adverse events, regardless of seriousness, severity, or presumed relationship to study therapy, will be recorded using medical terminology in the source document. Whenever possible, diagnoses will be given when signs and symptoms are due to a common etiology (e.g., cough, runny nose, sneezing, sore throat, and head congestion should be reported as "upper respiratory infection). Investigators will record on the CRF their opinion concerning the relationship of the adverse event to study therapy. The principal investigators assumes responsibility for appropriate reporting of AEs to the regulatory authorities. The investigator will report these events to the appropriate Independent Ethics Committee/Institutional Review Board (IEC/IRB) in accordance with local regulations. All measures required for AE management will be recorded in the source document and reported according to the State University of New York at Buffalo instructions. Non-serious events/problems that impacted on the rights and welfare of the subjects will be reported as they occur or at the time of knowledge or notification.

Definition of adverse events

The International Conference on Harmonization (ICH) Guideline for Good Clinical Practice (GCP) E6(R1) defines an AE as:

Any untoward medical occurrence in a patient or clinical investigation patient administered a pharmaceutical product and which does not necessarily have a causal relationship with this treatment. An AE can therefore be any unfavorable and unintended sign (including an abnormal laboratory finding), symptom, or disease temporally associated with the use of a medicinal product, whether or not considered related to the medicinal product.

The term AE is used to include both serious and non-serious AEs.

Definition of serious adverse events

A serious adverse event is an AE occurring during any study phase (i.e., screening, run-in, treatment, wash-out, follow-up), at any dose of the study drugs that fulfils one or more of the following criteria:

- Results in death
- Is immediately life-threatening
- Requires in-patient hospitalization or prolongation of existing hospitalization
- Results in persistent or significant disability or incapacity
- Is a congenital abnormality or birth defect in offspring of the patient
- Is an important medical event that may jeopardize the patient or may require medical intervention to prevent one of the outcomes listed above.

- TO THE AUTHOR: The causality of SAEs (their relationship to all study treatment/procedures) will be assessed by the investigator(s) and communicated to AstraZeneca. This is requested to be on the AE cover sheet.

The following variables are recommended to be collected for each AE:

- AE (verbatim)
- The date when the AE started and stopped
- Whether the AE is serious or not
- Action taken with regard to IPs
- Administration of treatment for the AE
- Outcome

In addition, the following variables are recommended to be collected for SAEs:

- Date the AE met criteria for SAE
- Date the Investigator became aware of the SAE
- Seriousness criteria fulfilled
- Date of hospitalization
- Date of discharge
- Probable cause of death
- Date of death
- Whether an autopsy was performed
- Causality assessment in relation to study procedure(s)
- Causality assessment in relation to other medication, as explained in Section 1.4.2.
- Description of the SAE

Reporting of serious adverse events

All SAEs will be reported, whether or not considered causally related to the investigational product, or to the study procedure(s). The reporting period for SAEs is the period immediately following the time that written informed consent is obtained throughout the treatment period and including the designated follow-up period as per the study protocol. The investigator is responsible for informing the Ethics Committee and/or the Regulatory Authority of the SAE as per local requirements.

Note that all serious or unexpected adverse events must be reported to AstraZeneca regardless of the country where the study is conducted.

The investigator must inform the FDA, via a MedWatch form, of any serious or unexpected adverse events that occur in accordance with the reporting obligations of 21 CFR 312.32 , and will concurrently forward all such reports to AstraZeneca. A copy of the MedWatch report must be emailed to AstraZeneca (TCS vendor) at the time the event is reported to the FDA. It is the responsibility of the investigator to compile all necessary information and ensure that the FDA receives a report according to the FDA reporting requirement timelines and to ensure that these reports are also submitted to AstraZeneca at the same time.

A cover page should accompany the MedWatch form indicating the following:

- "Notification from an Investigator Sponsored Study"

- The investigator IND number assigned by the FDA
- The investigator's name and address
- The trial name/title and AstraZeneca ISS reference number (ESR-##-#####)

Investigator must also indicate, either in the SAE report or the cover page, the causality of events in relation to all study medications and if the SAE is related to disease progression.

Send SAE report and accompanying cover page by way of email to AstraZeneca's designated mailbox: AEMailboxClinicalTrialTCS@astrazeneca.com

If a non-serious AE becomes serious, this and other relevant follow-up information must also be provided to AstraZeneca and the FDA.

Serious adverse events that do not require expedited reporting to the FDA still need to be reported to AstraZeneca preferably using the MedDRA coding language for serious adverse events.

The investigators will either provide a copy of the randomization code/ code break information or unblind those SAEs which require expedited reporting

Overdose

An overdose is defined as a patient receiving a dose of IP in excess of that specified in the Investigator's Brochure, unless otherwise specified in this protocol.

Any overdose of a study patient with the investigational product, with or without associated AEs/SAEs, is required to be reported within 24 hours of knowledge of the event to AstraZeneca Patient Safety using the designated Safety e-mailbox (see Section 1.5 or contact information). If the overdose results in an AE, the AE must also be recorded as an AE. Overdose does not automatically make an AE serious, but if the consequences of the overdose are serious, for example death or hospitalization, the event is serious and must be recorded and reported as an SAE (see Section 1.3). Any specific details around overdose for combination or comparator agents will also be provided as applicable. The investigator will use clinical judgment to treat any overdose.

Hepatic function abnormality

Hepatic function abnormality that fulfills the biochemical criteria of a potential Hy's Law case in a study patient, with or without associated clinical manifestations, is recommended to be reported as "hepatic function abnormal" within 24 hours of knowledge of the event to AstraZeneca Patient Safety using the designated Safety e-mailbox (see Section 1.3), unless a definitive underlying diagnosis for the abnormality (e.g., cholelithiasis or bile duct obstruction) that is unrelated to investigational product has been confirmed. The criteria for a potential Hy's Law case is Aspartate Aminotransferase (AST) or Alanine Aminotransferase (ALT) ≥3x Upper Limit of Normal (ULN) together with Total Bilirubin (TBL) ≥2xULN at any point during the study following the start of study medication irrespective of an increase in Alkaline Phosphatase (ALP).

Pregnancy

If a patient becomes pregnant during the course of the study, the IPs should be discontinued immediately.

Pregnancy itself is not regarded as an AE unless there is a suspicion that the IP under study may have interfered with the effectiveness of a contraceptive medication. Congenital abnormalities or birth defects and spontaneous miscarriages should be reported and handled as SAEs. Elective abortions without complications should not be handled as AEs. The outcome of all pregnancies (spontaneous miscarriage, elective termination, ectopic pregnancy, normal birth, or congenital abnormality) should be followed up and documented even if the patient was discontinued from the study.

If any pregnancy occurs in the course of the study, then the investigator should inform the appropriate AstraZeneca representatives within 1 day, i.e., immediately, but no later than 24 hours of when he or she becomes aware of it.

The investigator will ensure that all relevant information is provided to the AstraZeneca Patient Safety data entry site within 1 to 5 calendar days for SAEs and within 30 days for all other pregnancies.

The same timelines apply when outcome information is available.

19.3 Describe any safety endpoints.

Response: These specific Safety endpoints will be collected:

1- Frequency and severity of hypoglycemia: Hypoglycemia will be assessed throughout the study via multiple daily finger stick glucose measurements, CGM data collected 3 times (about 3 weeks each) during the study and from patient reports during visits and phone calls. Hypoglycemic events will be characterized as in the experts consensus published in *Diabetes Care (2017 Dec; 40(12): 1622-1630):*

- Level 1: Glucose < 70 mg/dL (3.9 mmol/L) and Glucose ≥ 54 mg/dL (3.0 mmol/L)
- Level 2: Glucose < 54 mg/dL (3.0 mmol/L)
- Level 3: A severe event characterized by altered mental and/or physical status requiring assistance.

2- Ketones levels: Urinary ketones measured daily at home and logged in the ketones logbook. Blood and urinary ketones measured during each visit. Ketone levels will be characterized as normal, ketosis and severe ketosis:

-Normal blood ketones <0.6mM or urinary ketones of trace to small (≥+1)

-Ketosis: Blood ketones >0.6mM <2.5mM or urinary ketones of moderate to high (+2 and +3)

-Severe ketosis Blood ketones >2.5mM or urinary ketones of too high (+4)

3- Diabetes ketoacidosis episodes: any confirmed DKA episode (regardless of hospitalization) defined as:

- Elevated serum or urine ketones (greater than the upper limit of the normal range), and
- Serum bicarbonate < 15 mmol/L or Blood pH < 7.3

4- Low blood pressure: <90mmHg SBP and <50 mmHg DBP): Patients will be instructed to measure blood pressure daily and it will be will be assessed in each visit.
5- Nausea, vomiting and other GI side effects. Data will be collected by patients' safety reports, during visits and phone calls.
6- Genital mycotic infections or UTIs
7- Changes in liver and kidney function: CMP and kidney and liver profiles will be assessed as indicated in table 1.

19.4 Describe how the safety information will be collected (e.g., with case report forms, at study visits, by telephone calls with participants).

Response: The safety information will be collected at the time of the visit, and or during preplanned telephone calls with the participant or patient self-reporting (including glucose, food intake and ketones diaries). Data will be entered into eCRFs

19.5 Describe the frequency of safety data collection.

Response: Safety data collection will be done at all study visits and during preplanned phone calls as in table 1. The patient, however, will be asked to report any adverse event or safety related information via phone as soon as it occurs and it will be reviewed the same day.

19.6 Describe who will review the safety data.

Response: DSMB established for this study will review unblended safety data provided by the data management system every 6 months. Additionally, the PIs and coinvestigators will review safety data (listed above) at the completion of each visits by each subject and review any report of side effects or adverse events as they are presented by the patients. The investigators will also review collective blinded data every 3 months to assess the safety and any potential risks to the participants. Furthermore the investigators will assess other risks including the physical, psychological, social, legal and economic harm to these patients. The investigators will carefully watch for any invasion of privacy and breach of confidentiality. The principal investigator will be sharing the results of safety analysis of the study and serious adverse events with the local IRB, DSMB, JDRF, AZ and the FDA. The study groups will remain blinded. If there are a serious safety concerns or an emergency, then, and based on principal investigator discretion, study randomization center in Glasgow University or the unblended statistician will provide information about group assignment of the patient(s) involved. Un-blinded data will be reviewed by the principal investigator and co-investigators and decision to complete, withdraw or adjust dose of drug(s) will be made, documented and shared with IRB and DSMB.

19.7 Describe the frequency or periodicity of review of cumulative safety data.

Response: Safety data will be reviewed every 6 months by DSMB (un-blinded), every 3 months by PIs and CO-Is (blinded) and at every visit by study team.

19.8 Describe the statistical tests for analyzing the safety data to determine whether harm is occurring.

Response: N/A

19.9 Describe any conditions that trigger an immediate suspension of the research.

Response: No interim analysis is planned for efficacy so termination of study will be related to safety. Significant increases in severe hypoglycemic or DKA events as reviewed by DSMB are possible causes that might trigger termination of study.

20.0 Withdrawal of Subjects

☐ **N/A:** This study is not enrolling subjects. This section does not apply.

*20.1 Describe **anticipated** circumstances under which subjects may be withdrawn from the research without their consent.*

1. The site PI can stop investigational study treatment or remove participants from the trial without participants approval if for any reason he/she feels is appropriate, including: severe side effect, injury or medical condition which may place the patient at risk of further complications if he/she continue to participate, failure to take the medication as instructed, failure to keep scheduled appointments, cancellation of the study by the sponsor, IRB, FDA or other administrative reasons.
2. If any of the participants become pregnant during the period of study, they will discontinue study treatment.
3. Participants who experience persistent severe nausea or vomiting after starting semaglutide which is not managed by dose reduction will discontinue the investigational study treatment. Those with mild or moderate nausea will continue for at least one more week. If they continue to experience significant nausea (defined as interfering with normal activities), dose will be reduced. If they continue to have these side effects, they will discontinue study medication(s).
4. Developing other conditions included in the exclusion criteria
5. Change in method of insulin delivery between insulin pumps to MDI or vice versa during the study unless pump treatment interruption is temporary for a period of at most 2 weeks. Also, patients who upgrade to the new "artificial pancreas" pump (Medtronic's MiniMed 670G hybrid) during the study will be withdrawn.
6. Hypoglycemia: Two episodes of severe hypoglycemia (requiring others help).

7. Ketosis (with no other identifiable precipitating factors): Persistent (1week) asymptomatic or symptomatic and unmanaged (using STICH protocol) blood ketone levels >1.5 mM (or ≥+2 in urine).
8. Persistent (>1 week) total insulin dose reduction of >20% from baseline AND total insulin dose <0.4U/Kg.
9. DKA: Two episode of DKA (with no identifiable precipitating factors) as defined by the expert consensus statement (*Diabetes Care (2017 Dec; 40(12): 1622-1630)*)
10. Two episodes of UTI.
11. Two episodes of mycotic genital infection.
12. Significant persistent change in eGFR > 15 ml/min
13. Pancreatitis: One episode of pancreatitis.
14. Other persistent side effects specific to study drugs

20.2 Describe any procedures for orderly termination.

NOTE: Examples may include return of study drug, exit interview with clinician. Include whether additional follow up is recommended for safety reasons for physical or emotional health.

Response: Participation in this study is voluntary. Participants have the right to refuse to participate or to withdraw from participation at any time for any reason. Refusal to participate or to withdraw from the study will involve no penalty or loss of entitled benefits, nor affect ongoing medical care. The site PI can stop investigational study treatment or remove participants from the trial without participants approval. If a patient withdraws consent and decide not to participate in the study, she/he will be asked to undergo a final study visit for his/her safety. If a subject withdraws from the research, the data collected to that point will be used toward the research finding. If applicable the subject will have to bring back any unused research drug and or devices.

20.3 Describe procedures that will be followed when subjects withdraw from the research, including retention of already collected data, and partial withdrawal from procedures with continued data collection, as applicable.

Response: We will use the data collected up to the point of stopping the study. No further information will be collected. Patients who stop study treatment and are interested in continuing in the study will be followed up until the end of the study for the primary end point and select secondary endpoints based on the Intent-To-Treat (ITT) analysis model. Withdrawn participants will not be replaced. The study sample size calculation includes an additional 15% subjects to compensate for drop out.

21.0 Risks to Subjects

21.1 List the reasonably foreseeable risks, discomforts, hazards, or inconveniences to the subjects related to their participation in the research. Consider physical, psychological, social, legal, and economic risks. Include a description of the probability, magnitude, duration, and reversibility of the risks.

NOTE: Breach of confidentiality is always a risk for identifiable subject data.

Response:

- Risks associated with intravenous (IV) line: All participants will be informed of the potential complications of an intravenous (IV) line, which include mild bruising at the site of IV line, usually resolving in a few days. They will also be informed about the possibility of infiltration of the IV line at the time of performing blood draws or during the meal challenge test in which case another IV line at a different site will be required (with risk of bruising at more than one site).
- Risks associated with 24 hour Ambulatory BP monitoring device: The BP cuff inflates every 15 minutes to record blood pressure over 24-hours. Participants will experience a pressure-like sensation over the arm where 24 hour BP device is worn at the time of the BP being measured due to the inflation of the cuff and release of this pressure at the time of deflation of the BP cuff. They will be informed to remove the cuff after 24 hours. If worn for longer periods they may experience mild bruising over the arm and some swelling, usually resolving 1-2 days after discontinuation.
- Risks associated with fat tissue aspiration: will probably lead to a bruise at the site of aspiration. The site may be mildly painful for 1-2 weeks. Participants are advised to call their study site staff if they have more severe pain or swelling at the site after the procedure. Rarely there are side effects such as low BP or heart rate and/or allergic reaction to local anesthetic including swelling of the throat.
- All subjects will be referred to semaglutide and dapagliflozin labeling safety information, and will be given a copy of the package insert of the medication. Discussion of the potential side effects and the different warning and precautions will take place at the time of informed consent. Patients will be informed they must not discontinue their insulin during the study.
- Potential risk of insulin, GLP-1 RA and SGLT-2 inhibitors use in type 1 diabetes patients is hypoglycemia. The combination therapy can possibly increase the magnitude and frequency of this risk.
- Potential side effects of GLP-1 RA and SGLT-2 inhibitors also include hypotension, nausea and other GI side effects, polydipsia, polyuria, dehydration, ketosis, and reduced glomerular filtration rate (GFR).
- In rodents, semaglutide causes dose-dependent and treatment-duration-dependent thyroid C-cell tumors at clinically relevant exposures. However, it received FDA-approval for the treatment of type 2 diabetes in 2017 following large Phase 3 trials including the cardiovascular safety trial SUSTAIN-6.
- On the basis of the above rodent studies, semaglutide is contraindicated in those with a personal or family history of medullary thyroid carcinoma and in patients with Multiple Endocrine Neoplasia syndrome type 2 (MEN 2). However, following review of human data with GLP-1RAs, FDA does not require or suggest routine monitoring of serum calcitonin/ thyroid ultrasound in people treated with semaglutide.
- Injection-site reactions: Serious injection-site reactions, with or without bumps (nodules), have happened in some people who use GLP-1RAs but are less frequent with semaglutide. Injection-site reactions have rarely required surgical

intervention. Participants will be instructed to report any injection site reactions including nodules to study investigators.

- Kidney injury: GLP-1 RA might cause acute kidney injury in patients without underlying renal disease especially in patient who had experienced nausea, vomiting, diarrhea or dehydration. Dapagliflozin also has warnings and precautions against acute kidney injury and impairment in renal function.
- As mentioned above, there is a risk of ketoacidosis, as shown by some studies and by our data, when SGLT2 inhibitors are used in type 1 diabetes. In our own preliminary studies on triple therapy, we observed euglycemic ketoacidosis in participants whose insulin doses had been reduced by > 20% of baseline levels or in patients who are not adequately insulinzed. Thus, this adverse effect is largely attributable to insulinopenia as well as anti-insulin effects of SGLT2 inhibitors at the hepatic level (e.g. increased gluconeogenesis).
- The following other known adverse effects of SGLT-2 inhibitors will be discussed with participants when obtaining informed consent:

1. Risk of dizziness/ symptomatic hypotension: can occur soon after initiation due to intravascular volume contraction.
2. Impaired kidney function: As SGLT-2 inhibitors increase serum creatinine and decrease eGFR, this will be monitored closely.
3. Genital mycotic infections: SGLT-2 inhibitors increase the risk of genital mycotic infections, particularly in women. Those with a prior history are more susceptible. Following topical treatment with anti-fungal medications they rarely re-occur.
4. Bladder Cancer - Across 22 clinical studies, newly diagnosed cases of bladder cancer were reported in 10/6045 patients (0.17%) treated with Dapagliflozin and 1/3512 patient (0.03%) treated with placebo. After excluding patients in whom exposure to study drug was less than one year at the time of diagnosis, there were 4 cases of bladder cancer with dapagliflozin and no cases with placebo i.e. too few cases to determine causality.
5. The dapagliflozin tablets and matching placebo contain lactose, which may cause discomfort in lactose-intolerant individuals.

- Severe allergic reaction (anaphylaxis) to study drugs or placebo ingredients or other interventions may accrue in rare cases.
- There might be other unforeseen risks associated with the use of these agents in type 1 diabetes and additional unforeseen risks associated with the combination therapy.
- There is always the risk of breach of confidentiality where patients' identifiable data is compromised.

21.2 Describe procedures performed to lessen the probability or magnitude of risks, including procedures being performed to monitor subjects for safety.

Response:

The following are procedures to lessen the probability and magnitude of risks listed above:

1- Hypoglycemia:

- Patients will be instructed to measure glucose at least 4 times a day (before meals and at bedtime) through-out the study and record all reading.
- Insulin dose adjustments will be done at every visit to avoid hypoglycemia. However, there will be no preemptive insulin dose reduction at start of treatment since all patients will have HbA1c >7.5% and the risk of hypoglycemia will be minimal when semaglutide is added.
- Daily fingerstick glucose, CGM and insulin pump data will be reviewed by the investigators for optimal titration of insulin dose to target glucose levels between 90-180mg/dl.
- In general, insulin dose adjustments should not be >10% from current total insulin dose (or 5% if HbA1c is <7%) or >20% from baseline total insulin dose at week 0 and then at week 26.
- Patients will be trained in CARB counting to avoid inaccurate insulin dose administration.
- Patients will be trained to recognize symptoms of hypoglycemia and how to manage it at home. These symptoms include feeling shaky, being nervous or anxious, sweating, chills and clamminess, confusion, fast heartbeat, feeling lightheaded or dizzy, hunger, nausea and color draining from the skin (pallor) among others. They will be asked to call the research office or the on call clinical services.
- When patients feel symptoms of hypoglycemia they will be asked to measure blood glucose and if <70mg/dl they will be instructed to consume carbohydrate or 4 tablets of glucose (4gm each). If after 15 min hypoglycemia continues, repeat carb intake until sugar levels are above 70 mg/dl. If sugar levels are below 55mg/dl, glucagon injection is permitted as rescue medication for severe hypoglycemia.
- Patients will be instructed to inform people who they are in frequent contact with (for example, friends, family members, and coworkers) on how to give glucagon to treat severe hypoglycemia. If patient have needed glucagon previously, it should be documented during screening visit so more emphasis on ways to prevent severe hypoglycemia in the future can be discussed.
- Patients/family will be instructed not to hesitate to call 911 if someone is unconscious and glucagon is not available or someone does not know how to use it and not to inject insulin (it will lower their blood glucose even more) or provide food or fluids (they can choke). They will be asked to document all details of the incident and report to research staff as soon as possible.
- Study medications dose will be reduced in patients who continue to have more than 2 measurements of glucose levels in the hypoglycemic range (<70mg/dl, regardless of symptoms) after insulin dose is reduced by the maximum allowed in this study.

2- Ketosis and Ketoacidosis:

- Patients will be instructed to measure ketone bodies in urine samples daily starting at 3 days before week 26 with Ketostix (Bayer) and record it in diary provided. Ketones (blood and urine) will be measured at every visit. If ketone bodies in urine measure +1 or greater (>1.5mM in urine), patients are required to notify the research center. They will be monitored closely for insulin dose,

carb intake, ketones and blood sugar and dapagliflozin/semaglutide can be temporarily discontinued, if necessary, for 1-2 weeks.

- Patients will be provided training to recognize the signs and symptoms of DKA and to self-test urine ketones on a PRN basis (in addition to once daily) if they develop any symptoms consistent with DKA, including nausea, vomiting, fatigue, confusion, or shortness of breath. This should be reported regardless of blood sugar levels since in type 1 diabetes on SGLT-2 inhibitors, DKA can occur even in normal glucose levels.
- Insulin dose changes should not be greater than 10% (5% if HbA1c is<7%) from current levels and not reduced by more than 20% from baseline levels at 0 weeks and then at 26 weeks.
- Patients requiring an insulin dose reduction > 20% AND their total insulin dose is <0.4U/Kg will be monitored closely for insulin dose, carb intake, ketones and blood sugar and dapagliflozin/semaglutide can be temporarily discontinued, if necessary, for 1-2 weeks. If insulin requirements remain law, patient will be withdrawn from study.
- We shall not increase dose of dapagliflozin in patients who exhibit significant ketonuria (+2 or more) with the lower dose.
- Urinary ketones (and blood ketones if patients have a dual glucose and ketone meter) will be monitored daily and reported. Dapagliflozin will be temporarily (1 week) discontinued and STICH protocol (STop SGLT-2i, give Insulin bolus, Carbohydrate, and Hydration) activated in events of DKA or if based on investigator discretion if blood ketone levels are persistently (1 week) >1.5 mM (+2 in or more in urine). Dapagliflozin may also be temporarily stopped in clinical situations known to predispose to ketoacidosis (e.g. prolonged fasting due to acute illness or surgery). Patients will be instructed to stay hydrated and not reduce their caloric intake (especially carbohydrates) or start new dietary systems (especially ketogenic diets) during the study.
- If a patient develops symptoms of DKA or ketone levels exceed the allowed limit in this study (persistent (1 week) blood ketones >1.5 mM or +2 in or more in urine), STICH protocol (STop SGLT-2i, give Insulin bolus, Carbohydrate, and Hydration) will be activated. They could be managed for up to a week. If symptoms and ketone levels persist over 1 week, patients will be withdrawn form study. Insulin infusions may be administered by ER or hospital if patients develop DKA. They will be asked to document all details of the incident and report to research staff as soon as possible.

3- Risk of acute kidney injury or renal function impairment: CBC, CMP, including kidney function test and urinary markers will be performed at screening, during and at end of the study (table 1). Additional testing will be performed in patients who suffer from sever nausea or GI symptoms or hypovolemic and during the up titration of investigation drugs. If acute kidney injury occurs dapagliflozin will be discontinued immediately. To lessen the chance of this risk, patients will eGFR<60 ml/min/1.73m^2 will be excluded. Patients will also discontinue study medications if eGFR falls by >15 ml/min in 2 repeated measurements. Also other patients with factors that may predispose patients to acute kidney injury e.g. hypovolemia, chronic renal insufficiency, congestive heart failure will be

excluded. Patients on ACEI, ARB, NSAID will be monitored closely.. Dapagliflozin will be temporarily discontinued in any setting of reduced oral intake such as acute illness or fasting or fluid losses such as gastrointestinal illnesses, or excessive heat exposure.

4- Injection site reactions, mycotic infections and complications of IV and biopsy collection will also be monitored, Physical examination will be performed at screening 26, and 52 weeks visits. Safety vital assessments will be performed at every visit. Pregnancy will be tested at screening, 0, 4 13, 26, 39, and 52 weeks. Phone calls will be made for safety and compliance at least 7 time during the study. The patients, however, will be instructed to report any adverse event or safety related information via phone as soon as it occurs. All safety data will be reviewed by the PI or sub-investigators within 1-2 days of collection.

5- Epinephrine is the primary treatment for anaphylaxis with no absolute contraindication to its use. Our site and staff are trained and equipped to assist patients with such reactions. Severe conditions that are not satisfactory managed in site will be transferred to the hospital emergency department for further assessment and help.

*21.3 If applicable, indicate **which procedures** may have risks to the subjects that are currently unforeseeable.*

Response:

There might be additional adverse effects of dapagliflozin not currently known or those unique to their use in Type 1 diabetes. There is no previous studies on the use of semaglutide in type 1 diabetes; therefore, additional unforeseen risk may be encountered in this study. Additionally, unforeseen risks might be associated with the combination therapy that might involve unknown drug-drug interactions.

21.4 If applicable, indicate which research procedures may have risks to an embryo or fetus should the subject be or become pregnant.

Response: Not applicable, we will not be enrolling pregnant participants or who may become pregnant or with in child baring years without signing the consent stating they will use at least two forms of birth control. If a participant becomes pregnant they will be withdrawn from the study immediately. They will be followed until term and any effects on the newborn will be documented.

21.5 If applicable, describe risks to others who are not subjects.

Response: We do not anticipate any additional risk to others not participating in this study.

22.0 Potential Benefits to Subjects

22.1 Describe the potential benefits that individual subjects may experience by taking part in the research. Include the probability, magnitude, and duration of the potential benefits. Indicate if there is no direct benefit.

*NOTE: Compensation **cannot** be stated as a benefit.*

Response: The investigational drugs used in this study are already approved as antidiabetic agents for treating type 2 diabetes. Both agents have demonstrated significant improvement in HbA1c, weight and blood pressure. It is our expectation that patients with type 1 diabetes will experience similar benefits compared to those who are treated with insulin alone. The magnitude and duration of the benefits and the safety profile may however differ and are the focus of this investigation.

23.0 Compensation for Research-Related Injury

☐ **N/A:** The research procedures for this study do not present risk of research related injury (e.g. survey studies, records review studies). This section does not apply.

*23.1 **If the research procedures carry a risk of research related injury,** describe the available compensation to subjects in the event that such injury should occur.*

Response: Routinely, Buffalo General Hospital, Erie County Medical Center, and/or the University at Buffalo, State University of New York, its agents, or its employees do not compensate for or provide free medical care for human subjects/participants in the event that any injury results from participation in a human research project. In the unlikely event that they become ill or injured as a direct result of participating in this study, they may receive medical care. Your study doctor will explain the treatment options to you and tell you where you can get treatment. Generally, this care will be billed to you, your insurance or other third party. Additionally, AstraZeneca shall reimburse the institution for the direct, reasonable, and necessary medical expenses incurred by the institution for the treatment of any personal injury that is a direct result of adverse drug experiences to subjects resulting directly from any manufacturing defects in the study drug to which the subjects would not have been exposed but for their participation in the study if (i) the institutional parties have complied with this agreement, the protocol and any written instructions of AstraZeneca concerning the study drug and (ii) all the requirements of informed consent have been complied with. AstraZeneca will not provide compensation for lost wages or for any other damages, expenses or losses, or for medical expenses have have been covered by a subject's medical or other insurance.

23.2 Provide a copy of contract language, if any, relevant to compensation for research related injury.

*NOTE: If the contract is not yet approved at the time of this submission, submit the current version here. If the contract is later approved with **different language regarding research related injury**, you must modify your response here and submit an amendment to the IRB for review and approval.*

Response: AstraZeneca shall reimburse the institution for the direct, reasonable, and necessary medical expenses incurred by the institution for the treatment of any personal injury that is a direct result of adverse drug experiences to subjects resulting directly from any manufacturing defects in the study drug to which the subjects would not have been exposed but for their participation in the study if (i) the institutional parties have complied with this agreement, the protocol and any written instructions of AstraZeneca concerning the study drug and (ii) all the requirements of informed consent have been complied with. AstraZeneca will not provide compensation for lost wages or for any other damages, expenses or losses, or for medical expenses have have been covered by a subject's medical or other insurance.

24.0 Economic Burden to Subjects

24.1 Describe any costs that subjects may be responsible for because of participation in the research.

NOTE: Some examples include transportation or parking.

Response: All research expenses will be covered by the study. The background use of insulin is considered as part of standard of care is the responsibility of the patients and his/her insurance. Participants will not be subjected to any other out of pocket cost.

☐ **N/A:** This study is not enrolling subjects, or is limited to records review procedures only. This section does not apply.

25.0 Compensation for Participation

25.1 Describe the amount and timing of any compensation to subjects, including monetary, course credit, or gift card compensation.

Response:
Visit schedule:

- Screen: $0
- Visit 1 (Week -3): Insulin adjustment: $10
- Visit 2 (Week -1): Meal ($75), insulin adjustment, and 24hr blood pressure monitor ($50): $125*
- Visit 3 (Week 0): 24 hr urine ($15), blood draw ($25), fat biopsy ($75), insulin adjustment, questionnaire ($10): $125
- Visit 4 (Week 1): Insulin adjustment: $10
- Visit 5 (Week 2): Insulin adjustment: $10
- Visit 6 (Week 4): Insulin adjustment: $10
- Visit 7 (Week 13): 24hr urine ($15), blood draw ($25), insulin adjustment, 24hr bp monitor ($50): $90*
- Visit 8 (Week 23): Insulin adjustment: $10

- Visit 9 (Week 25): Insulin adjustment, 24bp monitor ($50), Meal ($75): $125*
- Visit 10 (Week 26): Insulin adjustment, 24hr urine ($15), blood draw ($25), fat biopsy ($75), questionnaire ($10): $125*
- Visit 11 (Week 27): Insulin adjustment: $10
- Visit 12 (Weak 31): Insulin adjustment, blood draw: $25
- Visit 13 (Week 39): Insulin adjustment, 24hr urine ($15), blood draw ($25), 24hr bp monitor ($50): $90*
- Visit 14 (Week 49): Insulin adjustment: $10
- Visit 15 (Week 51): Insulin adjustment, 24bp monitor ($50), meal ($75): $125*
- Visit 16 (Week 52): Insulin adjustment, 24hr urine ($15), blood draw ($25), questionnaire ($10), fat biopsy ($75):$125*
  *Total compensation for all completed visits: up to $1,025.00. For those not wishing to participate in the optional blood pressure monitoring and/or fat biopsy procedure, the compensation will decrease by $250 for not completing the bp monitoring, and by yet another $225.00 for not completing the fat biopsies.

☐ **N/A:** This study is not enrolling subjects, or is limited to records review procedures only. This section does not apply.

☐ **N/A:** There is no compensation for participation. This section does not apply.

26.0 Consent Process

26.1 Indicate whether you will be obtaining consent.

NOTE: This does not refer to consent documentation, but rather whether you will be obtaining permission from subjects to participate in a research study. Consent documentation is addressed in Section 27.0.

☒ ***Yes*** *(If yes, Provide responses to each question in this Section)*
☐ ***No*** *(If no, Skip to Section 27.0)*

26.2 Describe where the consent process will take place. Include steps to maximize subjects' privacy.

Response: All participants will come to the research department to be consented. Participants will be placed in a private, screened off area and or room where they can review the consent. Participant questions and or concerns will be address with a member of the study team or research doctor if applicable. The research coordinator will discuss in length the participants requests for privacy of their PHI.

26.3 Describe how you will ensure that subjects are provided with a sufficient period of time to consider taking part in the research study.

NOTE: It is always a requirement that a prospective subject is given sufficient time to have their questions answered and consider their participation. See "SOP: Informed Consent Process for Research (HRP-090)" Sections 5.5 and 5.6.

Response: participants will be made aware that participating in research is completely voluntary, and they may withdraw at any time with no consequence to their routine clinic care. If the patients requires time to decide and or discuss partaking in a research study, the subject will be given said time.

26.4 Describe any process to ensure ongoing consent, defined as a subject's willingness to continue participation for the duration of the research study.

Response: The research coordinator and study team are available to answer any question or concerns with the patient during the duration of the research trial. At each study visit, the patient is asked a series of questions to ensure they are on task with the study visits and feel comfortable. Upon departing from their study visit, the patients are told of their next visit and given detail instruction for their next visit. If study is revised or amendment or new information becomes available about drug safety that may affect patients participation, the patient may be re-consented to ensure patient ongoing consent

26.5 Indicate whether you will be following "SOP: Informed Consent Process for Research (HRP-090)." If not, or if there are any exceptions or additional details to what is covered in the SOP, describe:

- *The role of the individuals listed in the application who are involved in the consent process*
- *The time that will be devoted to the consent discussion*
- *Steps that will be taken to minimize the possibility of coercion or undue influence*
- *Steps that will be taken to ensure the subjects' understanding*

Response:

☒ We have reviewed and will be following "SOP: Informed Consent Process for Research (HRP-090)."

Non-English Speaking Subjects

☒ **N/A:** This study will not enroll Non-English speaking subjects.
(Skip to Section 26.8)

26.6 Indicate which language(s) other than English are likely to be spoken/understood by your prospective study population or their legally authorized representatives.

NOTE: The response to this Section should correspond with your response to Section 6.4 of this protocol.

Response:

26.7 If subjects who do not speak English will be enrolled, describe the process to ensure that the oral and written information provided to those subjects will be in that language. Indicate the language that will be used by those obtaining consent.

NOTE: Guidance is provided on "SOP: Informed Consent Process for Research (HRP-090)."

Response:

Cognitively Impaired Adults

☒ **N/A**: This study will not enroll cognitively impaired adults.
(Skip to Section 26.9)

26.8 Describe the process to determine whether an individual is capable of consent.

Response:

Adults Unable to Consent

☒ **N/A**: This study will not enroll adults unable to consent.
(*Skip to Section 26.13)*

When a person is not capable of consent due to cognitive impairment, a legally authorized representative should be used to provide consent (Sections 26.9 and 26.10) ***and, where possible, assent of the individual should also be solicited*** *(Sections 26.11 and 26.12).*

26.9 Describe how you will identify a Legally Authorized Representative (LAR). Indicate that you have reviewed the "SOP: Legally Authorized Representatives, Children, and Guardians (HRP-013)" for research in New York State.

NOTE: Examples of acceptable response includes: verifying the electronic medical record to determine if an LAR is recorded.

Response:

☐

26.10 ***For research conducted outside of New York State****, provide information that describes which individuals are authorized under applicable law to consent on behalf of a prospective subject to their participation in the research. One method of obtaining this information is to have a legal counsel or authority review your protocol along with the definition of "legally authorized representative" in "SOP: Legally Authorized Representatives, Children, and Guardians (HRP-013)."*

Response:

26.11 Describe the process for ***assent of the adults****:*

- *Indicate whether assent will be obtained from all, some, or none of the subjects. If some, indicate which adults will be required to assent and which will not.*

  Response:

- *If assent will not be obtained from some or all subjects, provide an explanation of why not.*

  Response:

26.12 Describe whether ***assent of the adult*** *subjects will be documented and the process to document assent.*

NOTE: The IRB allows the person obtaining assent to document assent on the consent document using the "Template Consent Document (HRP-502)" Signature Block for Assent of Adults who are Legally Unable to Consent.

Response:

Subjects who are not yet Adults (Infants, Children, and Teenagers)

☒ **N/A**: This study will not enroll subjects who are not yet adults.
(Skip to Section 27.0)

26.13 Describe the criteria that will be used to determine ***whether a prospective subject has not attained the legal age for consent to treatments or procedures involved in the research*** *under the applicable law of the jurisdiction in which the research will be conducted* ***(e.g., individuals under the age of 18 years)****. For research conducted in NYS, review "SOP: Legally Authorized Representatives, Children, and Guardians (HRP-013)" to be aware of which individuals in the state meet the definition of "children."*

NOTE: Examples of acceptable responses include: verification via electronic medical record, driver's license or state-issued ID, screening questionnaire.

Response:

26.14 ***For research conducted outside of New York State****, provide information that describes which persons have not attained the legal age for consent to treatments or procedures involved the research, under the applicable law of the jurisdiction in which research will be conducted. One method of obtaining this information is to have a legal counsel or authority review your protocol along the definition of "children" in "SOP: Legally Authorized Representatives, Children, and Guardians (HRP-013)."*

Response:

26.15 Describe whether parental permission will be obtained from:

Response: N/A

- ☐ One parent even if the other parent is alive, known, competent, reasonably available, and shares legal responsibility for the care and custody of the child.
- ☐ Both parents unless one parent is deceased, unknown, incompetent, or not reasonably available, or when only one parent has legal responsibility for the care and custody of the child.
- ☐ Parent permission will not be obtained. A waiver of parent permission is being requested.

NOTE: The requirement for parent permission is a protocol-specific determination made by the IRB based on the risk level of the research. For guidance, review the "CHECKLIST: Children (HRP-416)."

26.16 Describe whether permission will be obtained from individuals ***other than parents****, and if so, who will be allowed to provide permission. Describe your procedure for determining an individual's authority to consent to the child's general medical care.*

Response:

26.17 Indicate whether assent will be obtained from all, some, or none of the ***children****. If assent will be obtained from some children, indicate which children will be required to assent.*

Response:

26.18 When assent of children is obtained, describe how it will be documented.

Response:

27.0 Waiver or Alteration of Consent Process

Consent will not be obtained, required information will not be disclosed, or the research involves deception.

☒ **N/A:** A waiver or alteration of consent is not being requested.

27.1 If the research involves a waiver or alteration of the consent process, please review the "CHECKLIST: Waiver or Alteration of Consent Process (HRP-410)" to ensure that you have provided sufficient information for the IRB to make the determination that a waiver or alteration can be granted.

NOTE: For records review studies, the first set of criteria on the "CHECKLIST: Waiver or Alteration of Consent Process (HRP-410)" applies.

Response:

27.2 If the research involves a waiver of the consent process for planned emergency research, please review the "CHECKLIST: Waiver of Consent for Emergency Research (HRP-419)" to ensure you have provided sufficient information for the IRB to make these determinations. Provide any additional information necessary here:

Response:

28.0 Process to Document Consent

☐ **N/A:** A Waiver of Consent is being requested.
(Skip to Section 29.0)

28.1 Indicate whether you will be following "SOP: Written Documentation of Consent (HRP-091)." If not or if there are any exceptions, describe whether and how consent of the subject will be obtained including whether or not it will be documented in writing.

NOTE: If your research presents no more than minimal risk of harm to subjects and involves no procedures for which written documentation of consent is normally required outside of the research context, the IRB will generally waive the requirement to obtain written documentation of consent. This is sometimes referred to as 'verbal consent.' Review "CHECKLIST: Waiver of Written Documentation of Consent (HRP-411)" to ensure that you have provided sufficient information.

📎 *If you will document consent in writing, attach a consent document with your submission. You may use "TEMPLATE CONSENT DOCUMENT (HRP-502)". If you will obtain consent, but not document consent in writing, attach the script of the information to be provided orally or in writing (i.e. consent script or Information Sheet).*

Response:

☒ We will be following "SOP: Written Documentation of Consent" (HRP-091).

29.0 Multi-Site Research (Multisite/Multicenter Only)

☐ **N/A:** This study is not an investigator-initiated multi-site study. This section does not apply.

*29.1 If this is a multi-site study **where you are the lead investigator**, describe the processes to ensure communication among sites, such as:*

- *All sites have the most current version of the IRB documents, including the protocol, consent document, and HIPAA authorization.*
- *All required approvals have been obtained at each site (including approval by the site's IRB of record).*
- *All modifications have been communicated to sites, and approved (including approval by the site's IRB of record) before the modification is implemented.*
- *All engaged participating sites will safeguard data as required by local information security policies.*
- *All local site investigators conduct the study appropriately.*
- *All non-compliance with the study protocol or applicable requirements will be reported in accordance with local policy.*

Response:

29.2 Describe the method for communicating to engaged participating sites:

- *Problems*
- *Interim results*
- *Study closure*

Response:

29.3 Indicate the total number of subjects that will be enrolled or records that will be reviewed across all sites.

Response: 114

29.4 If this is a multicenter study for which UB will serve as the IRB of record, and subjects will be recruited by methods not under the control of the local site (e.g., call centers, national advertisements) describe those methods.

Response: N/A

30.0 Banking Data or Specimens for Future Use

☐ **N/A:** This study is not banking data or specimens for future use or research outside the scope of the present protocol. This section does not apply.

30.1 If data or specimens will be banked (stored) for ***future use, that is, use or research outside of the scope of the present protocol****, describe where the data/specimens will be stored, how long they will be stored, how the data/specimens will be accessed, and who will have access to the data/specimens.*

NOTE: Your response here must be consistent with your response at the "What happens if I say yes, I want to be in this research?" Section of the Template Consent Document (HRP-502).

Response: The study data/specimens will be stored in a locked closet or -80 freezer at the research facility of the Diabetes and Endocrinology Center of WNY for at least 7 years.

The research staff (study personnel including coordinator) only will be authorized to access data and or specimens

30.2 List the data to be stored or associated with each specimen.

Response: Patient ID number, study visit information and date of collection will be stored with specimen. Other data stored will include record files of all patients participating in the study, including data collection sheets, lab results, CGM and insulin pump data.

30.3 Describe the procedures to release banked data or specimens for future uses, including: the process to request a release, approvals required for release, who can obtain data or specimens, and the data to be provided with specimens.

Response: The copy of the individual patient data collected during the study period will be provided to these individual patients who can choose to hand carry it to their respective physicians and a copy will be faxed to their respective clinical providers upon verbal request from the patient. The data provided will include the insulin pump or CGM data or any of the lab results obtained during the study period. The results of the completed study will be made available to the patients if requested through published manuscript.

31.0 Drugs or Devices

☐ **N/A:** This study does not involve drugs or devices. This section does not apply.

31.1 If the research involves drugs or devices, list and describe all drugs and devices used in the research, the purpose of their use, and their regulatory approval status.

Response: **Drugs used:**

Description or Identity of investigational product(s)

Investigational product	***Dosage form and strength***	***Manufacturer***	***Description***
Dapagliflozin (Farxiga) 5 mg	*Green, plain, diamond shaped, film coated 5 mg tablet*	*AstraZeneca*	*sodium-glucose cotransporter 2 (SGLT2) inhibitor indicated as an adjunct to diet and exercise to improve glycemic control*
Matching placebo for dapagliflozin 5 mg	*Green, plain, diamond shaped, film coated tablet*	*AstraZeneca*	
Semaglutide (Ozempic) 0.25 mg, 0.5 mg, 1.0 mg	*clear, colorless solution that contains 2 mg of semaglutide in a 1.5 mL (1.34 mg/mL) pre-filled, disposable, single-patient-use pen injector*	*Novo Nordisk*	*glucagon-like peptide 1 (GLP-1) receptor agonist indicated as an adjunct to diet and exercise to improve glycemic control*

- *Dapagliflozin and its matching placebo tablets will be supplied in bottles and are provided by Astra Zeneca.*
- *Semaglutide will be supplied as prefilled injectable pens and will be purchased commercially through the University at Buffalo Research Pharmacy.*

Subjects will receive drugs in their unmodified form. Dapagliflozin is a once daily tablet, while Semaglutide is a weekly subcutaneous injection.

Insulin pumps (CSII) and long and short acting insulin is the only diabetes medication accepted before and during this trial. No other diabetes drug is permitted.
Blood pressure or lipid lowering drugs are permitted if on stable dose for at least three months prior to start of trial. Glucagon injection is permitted as rescue medication for severe hypoglycemia. Insulin infusions may be administered by ER or hospital if patients develop DKA.

31.2 Describe your plans to store, handle, and administer those drugs or devices so that they will be used only on subjects and be used only by authorized investigators.

Response: Dapagliflozin 5mg tablets and matching placebo provided by Astra Zeneca will be stored in a locked cabinet in research pharmacy. Semaglutide provided by University at Buffalo research pharmacy will be stored in temperature controlled refrigerator at 4C at the research facility of the Diabetes and Endocrinology Center of WNY at 1000 Youngs Rd until dispensed to patients. Investigational drugs will be dispensed by PI or CO-Is only to study patients according to randomization plan.

If the drug is investigational (has an IND) or the device has an IDE or a claim of abbreviated IDE (non-significant risk device), include the following information:

31.3 Identify the holder of the IND/IDE/Abbreviated IDE.

Response: IND application for use of all drugs (dapagliflozin and semaglutide) in type 1 diabetes will be submitted to FDA and no study procedure will start before all local and federal approvals are obtained. FDA has granted IND for usage of dapagliflozin in type 1 diabetes. We will seek FDA approval for the use of semaglutide in this study. These IND letters will be submitted to IRB and JDRF along with final version of protocol before initiating any study activities.

31.4 Explain procedures followed to comply with FDA sponsor requirements for the following:

	Applicable to:		
FDA Regulation	***IND Studies***	***IDE studies***	***Abbreviated IDE studies***
21 CFR 11	***X***	***X***	
21 CFR 54	***X***	***X***	
21 CFR 210	***X***		
21 CFR 211	***X***		
21 CFR 312	***X***		
21 CFR 812		***X***	***X***
21 CFR 820		***X***	

Response: All FDA sponsor requirements have been reviewed and will be followed during the study procedures

32.0 Humanitarian Use Devices

☒ **N/A:** This study does not involve humanitarian use devices. This does not apply.

32.1 For Humanitarian Use Device (HUD) uses provide a description of the device, a summary of how you propose to use the device, including a description of any screening procedures, the HUD procedure, and any patient follow-up visits, tests or procedures.

Response:

32.2 For HUD uses provide a description of how the patient will be informed of the potential risks and benefits of the HUD and any procedures associated with its use.

Response:

US TTT1 Protocol Summary of changes V3 to V4

Section	Original	New	Justification	Consent changes
10.1	No statement	each visit- add CGM to the statement "Blood sugars and ketone bodies will be measured via a CGM/fingerstick blood sample using glucose and ketones meter (Abbott Laboratories) and daily blood sugars collected and reviewed"		yes
10.1	wk 25 "Participants will be provided supplies for urine ketones measurement (KetoStix) and blood ketones meter. "	wk 25 "Participants in the semaglutide group will be provided supplies for urine ketones measurement (KetoStix) and blood ketones meter. "	Defining what group will receive ketone meter	yes
10.1	wk 26 "Patients will be asked to perform a daily (preferably early morning or before bedtime) urinary test of ketone bodies using KetoStix and record all outcomes. "	wk 26 "All patients will be asked to perform a daily (preferably early morning or before bedtime) urinary test of ketone bodies using KetoStix and record all outcomes. "	Defining what group will measure daily urine ketones	yes
21.1	No statement	· Excessive weight loss: Both investigation drugs are known to cause weight loss in the range of 5-10% in type 2 diabetes patients. However, the magnitude of weight loss following the combination of both drugs in type 1 diabetes patients may be significantly more. Excessive weight loss may lead to increased risk of hypoglycemia and ketosis or DKA (especially in the presence of dapagliflozin) and other unforeseen risks. Therefore, weight loss during any phase of the study will be limited to ≤15% baseline body weight or reaching a BMI of ≤22Kg/m^2.		yes

21.2		Continuation of 21.1- explain how we will overcome this risk<br>6- 6- Excessive weight loss: Doses of investigational drugs will be reduced in patients who experience excessive weight loss (≥15% of basal body weight or BMI ≤22Kg/m2). Patients on semaglutide only (first 26 weeks) will be down-titrated to the lower dose. In patients on semaglutide and dapagliflozin/placebo (weeks 26-52), dose of dapagliflozin/placebo will be down-titrated to the lower dose. If weight loss continues, semaglutide dose will be down-titrated as well. Instructions to maintain a minimum of 50g daily carb intake will be also emphasized in patients with significant weight loss (>10% baseline body weight).		yes

University at Buffalo Institutional Review Board (UBIRB)
Office of Research Compliance | Clinical and Translational Research Center Room 5018
875 Ellicott St. | Buffalo, NY 14203
UB Federalwide Assurance ID#: FWA00008824

Complete Research Protocol (HRP-503)

Table of Contents

Template Instructions 2
1.0 Objectives 4
2.0 Scientific Endpoints 5
3.0 Background 6
4.0 Study Design 12
5.0 Local Number of Subjects 13
6.0 Inclusion and Exclusion Criteria 13
7.0 Vulnerable Populations 16
8.0 Eligibility Screening 17
9.0 Recruitment Methods 18
10.0 Procedures Involved 19
11.0 Study Timelines 32
12.0 Setting 32
13.0 Community-Based Participatory Research 33
14.0 Resources and Qualifications 34
15.0 Other Approvals 35
16.0 Provisions to Protect the Privacy Interests of Subjects 35
17.0 Data Management and Analysis 36
18.0 Confidentiality 39
A. Confidentiality of Study Data 39
B. Confidentiality of Study Specimens 40
19.0 Provisions to Monitor the Data to Ensure the Safety of Subjects 41
20.0 Withdrawal of Subjects 48
21.0 Risks to Subjects 49
22.0 Potential Benefits to Subjects 55
23.0 Compensation for Research-Related Injury 55
24.0 Economic Burden to Subjects 56
25.0 Compensation for Participation 56
26.0 Consent Process 57
27.0 Waiver or Alteration of Consent Process 62
28.0 Process to Document Consent 62
29.0 Multi-Site Research (Multisite/Multicenter Only) 63
30.0 Banking Data or Specimens for Future Use 64
31.0 Drugs or Devices 65
32.0 Humanitarian Use Devices 66

Template Instructions

Sections that do not apply:

- *In several sections, the addition of checkboxes for* ***Not Applicable*** *have been added to the template as responses.*
  - *If an N/A checkbox is present, select the appropriate justification from the list.*
  - *If an N/A checkbox is not present, or if none of the existing checkboxes apply to your study, you must write in your own justification.*
- *In addition:*
  - *For research where the only study procedures are records/chart review: Sections 19, 20, 22, 23, 24, 25, 31, and 32 do not apply.*
  - *For exempt research: Sections 31 and 32 do not apply.*

Studies with multiple participant groups:

- *If this study involves multiple participant groups (e.g. parents and children), provide information in applicable sections for each participant group. Clearly label responses when they differ. For example:*

  Response:

  <u>Intervention Group:</u>

  <u>Control Group:</u>

Formatting:

- *Do not remove template instructions or section headings when they do not apply to your study.*

If you are pasting information from other documents using the "Merge Formatting" Paste option will maintain the formatting of the response boxes.

Amendments:

- *When making modifications or revisions to this and other documents, use the* ***Track Changes*** *function in Microsoft Word.*
- *Update the version date or number* ***on Page 3.***

PROTOCOL TITLE:

Include the full protocol title.

Response: **Triple Therapy for Type 1 Diabetes with Insulin, Semaglutide, and Dapagliflozin**

PRINCIPAL INVESTIGATOR:

Response:

**Principal Investigator:**	**Paresh Dandona, MBBS, PhD**
**Co- Investigators:**	**Husam Ghanim, PhD**
	**Antoine Makdissi, MD**
	**Manav Batra, MD**
**Co- Principal Investigator:**	**John R Petrie, MBChB, PhD**

Paresh Dandona, M.B.B.S., Ph.D., F.R.C.P, F.A.C.P, F.A.C.C, FACE,
SUNY Distinguished Professor,
Director, Diabetes-Endocrinology Center of Western NY
Chief of Endocrinology, Diabetes, Endocrinology and Metabolism, SUNY at Buffalo
1000 Youngs Road, Suite 105
Williamsville NY 14221
Telephone: (716) 535-1850
Fax: 716-639-1200
E-mail: pdandona@kaleidahealth.org

CO-PI:

Professor John R Petrie
BSc MBChB PhD FRCP(Ed) FRCPSG
Professor of Diabetic Medicine
Institute of Cardiovascular and Medical Sciences
BHF Glasgow Cardiovascular Research Centre
University of Glasgow
126 University Place
Glasgow G12 8TA
Tel: +44(0)141 330 3325
Email: john.petrie@glasgow.ac.uk
Web: http://www.gla.ac.uk/researchinstitutes/icams/staff/johnpetrie/
The University of Glasgow

VERSION:

Include the version date or number.

Response: 3/10/2020, V4

GRANT APPLICABILITY:

Indicate whether this protocol is funded by a grant (e.g. NIH, foundation grant). For a grant with multiple aims, indicate which aims are covered by this research proposal.

NOTE: This question does not apply to studies funded by a sponsor contract.

Include a copy of the grant proposal with your submission.

Response: Research support provided by JDRF and AZ

RESEARCH REPOSITORY:

Indicate where the research files will be kept, including when the study has been closed. The repository should include, at minimum, copies of IRB correspondence (approval, determination letters) as well as signed consent documents. This documentation should be maintained for 3 years after the study has been closed.

Response:
Site 1: Diabetes Endocrinology Research Center of WNY
Address: 1000 Youngs Road, Suite 105, Williamsville NY 14221
Department: Diabetes Endocrinology

Site 2: University of Glasgow. Institute of Cardiovascular and Medical Sciences
University of Glasgow 126 University Place Glasgow, G12 8TA

1.0 Objectives

1.1 Describe the purpose, specific aims, or objectives of this research.

Response:

Primary Objective: To assess whether the addition of dapagliflozin to semaglutide and insulin (triple therapy) improves glycemic control in patients with type 1 diabetes compared with semaglutide and insulin (dual therapy) and insulin only (standard) treatment.

General hypothesis: The addition of dapagliflozin to semaglutide and insulin treatment improves glycemic control, weight loss and blood pressure in adults with type 1 diabetes.

1.2 State the hypotheses to be tested, if applicable.

NOTE: A hypothesis is a specific, testable prediction about what you expect to happen in your study that corresponds with your above listed objectives.

Response:

Specific hypotheses:

Primary: combined dapagliflozin and semaglutide treatment in addition to insulin will improve HbA1c levels compared with semaglutide in addition to insulin .

Secondary 1: semaglutide in addition to insulin treatment over 26 weeks will improve HbA1c levels compared with insulin therapy alone.

Secondary 2: combined dapagliflozin and semaglutide treatment in addition to insulin will improve HbA1c levels compared to insulin therapy alone at 52 weeks.

Secondary 3: combined dapagliflozin and semaglutide treatment in addition to insulin will improve CGM-based weekly mean and fasting blood glucose, glycemic oscillations and percent of time in range (70-180mg/dl) with lower insulin requirements compared with semaglutide and insulin (dual therapy) or insulin only (standard) treatment.

Secondary : combined dapagliflozin and semaglutide in addition to insulin therapy will reduce systolic and diastolic blood pressure compared with semaglutide and insulin (dual therapy) or insulin only (standard) treatment.

Secondary 5: combined dapagliflozin and semaglutide in addition to insulin therapy (triple therapy) will result in weight loss compared to dual and standard therapy.

Secondary 6: combined dapagliflozin and semaglutide in addition to insulin therapy will result in improved scores on the Diabetes Specific Quality of Life Scale (DSQOLS) and the Problem Areas In Diabetes (PAID) survey compared with dual and standard therapy.

Tertiary and Exploratory Objectives (EO) for future ancillary studies (PLEASE NOTE: For EO, samples will be collected to facilitate the measurements of these end points in future ancillary studies once submitted and approved by JDRF so cost of assays is not included in the current budget) : The addition of dapagliflozin to semaglutide and insulin in type 1 diabetes will:

Tertiary 1: combined dapagliflozin and semaglutide treatment in addition to insulin therapy will improve fasting and postprandial glucose excursions and suppress postprandial glucagon, FFA, GLP-1 compared with semaglutide and insulin or insulin therapy alone.

Tertiary 2: semaglutide in addition to insulin treatment over 52 weeks will improve HbA1c levels compared with insulin therapy alone.

EO 1: increase plasma concentrations of vasodilatation mediators and reduce plasma concentrations of vasoconstriction mediators compared to dual and standard therapy groups.

EO 2: exert a systemic anti-inflammatory effect including reducing mRNA expression of inflammatory mediators and NFκB binding in peripheral mononuclear cells (MNC) and adipose tissue (optional) and their circulating levels in plasma while increasing levels of IL-RA and adiponectin.

2.0 Scientific Endpoints

2.1 Describe the scientific endpoint(s), the main result or occurrence under study.

NOTE: Scientific endpoints are outcomes defined before the study begins to determine whether the objectives of the study have been met and to draw conclusions from the data. Include primary and secondary endpoints. Some example endpoints are: reduction of

*symptoms, improvement in quality of life, or survival. Your response should **not** be a date.*

Response:

Primary endpoint: Change in HbA1c at 6 months following dapagliflozin or placebo therapy in addition to combined semaglutide and insulin treatment.

Efficacy Secondary endpoints:

1. Change in HbA1c from baseline at 12 months in triple therapy group compared to insulin only group.
2. Change in HbA1c from baseline at 6 months in dual therapy group compared to insulin only group.
3. Change in percent Time in Range (70– 180 mg/dl), percent time in hyperglycemia Level 1(180-250mg/dl) and Level 2 (>250mg/dl) and percent time in hypoglycemia Level 1 (54-70mg/dl) as assessed by CGM
4. Other indices of glycemic control: fructosamine, weekly mean and fasting glucose, standard deviation, insulin requirements.
5. Change in body weight and quality of life as assessed by DSQOLS and PAID at 6 and 12 months.
6. Anti-hypertensive effects including change in systolic and diastolic BP, numbers of BP medications required.

Safety Secondary endpoints:

1- Differences in rates of hypoglycemic events Level 2 (<54mg/dl) and Level 3 (A severe event characterized by altered mental and/or physical status requiring assistance) between investigative arms and standard therapy arm.

2- *Differences in rates of diabetic ketoacidosis defined as elevated serum or urine ketones (greater than the upper limit of the normal range), and serum bicarbonate <15 mmol/L or Blood pH <7.3 between investigative arms and standard therapy arm.*

Tertiary/ exploratory endpoints: (items 2-4 to be performed in future on collected samples from this study if additional funding is approved by JDRF):

1- Fasting and postprandial (Area Under curve) glucose excursions, FFA, GLP-1 and glucagon and fasting fructosamine.

2- Indices of inflammatory stress. These will be CRP, FFA, SAA, IL-1β, IL-18, IL-1RA, adiponectin and TNFα concentrations in plasma and isoprostane in urine.

3- Expression of IL-1β, TNFα, IL-12, TLR2, TLR4, adiponectin, IκBα as well as NFκB binding in peripheral MNC and adipose tissue (optional).

4- Indices of vasodilatation and vasoconstriction: ANP, b-NP, cGMP, cAMP, angiotensinogen, renin and angiotensin II concentrations.

3.0 Background

3.1 Provide the scientific or scholarly background, rationale, and significance of the research based on the existing literature and how it will contribute to existing knowledge. Describe any gaps in current knowledge. Include relevant preliminary findings or prior research by the investigator.

Response:

The control of glucose homeostasis in type 1 diabetes is fragile since β-cell reserve is negligible. Traditionally, insulin has been the only treatment approach available. This approach, often leads to erratic blood sugar control given the challenge of matching exogenous insulin to mealtime glucose excursions in the face of a lack of any endogenous insulin to compensate for variation in requirements in response to meals and activity levels. Injected insulin boluses rarely match precise insulin requirements either in terms of dose administered or bio-availability. The result often is wide fluctuations in blood glucose readings. Based on our work to date and other published reports, previous attempts to add other glucose-lowering medications to insulin therapy yielded minimal benefits on glycemia (although metformin may have other benefits (1-3)

We have recently shown in two preliminary retrospective studies that the addition of liraglutide, a Glucagon Like Peptide -1 receptor agonist (GLP-1RA), to insulin results in consistent improvements in glycemic control in type 1 diabetes as demonstrated by reductions in HbA1c, glycemic excursions, and insulin requirements accompanied by improvements in body weight and systolic blood pressure (4, 5). We have now also completed the first prospective randomized controlled trial of this drug in 72 people with type 1 diabetes. Our data not only confirmed improvements in glycemic control, HbA1c, body weight and systolic BP, but also demonstrated a reduction in appetite, carbohydrate intake in low grade inflammation as measured by plasma CRP concentrations (6). Importantly, quality of life was also improved. Our recent studies in type 2 diabetes have revealed that systolic blood pressure reduction with GLP-1RA (exenatide) treatment in type 2 diabetes is associated with increased plasma concentrations of ANP, cGMP and cAMP along with reduced concentrations of angiotensinogen, renin and angiotensin II (7). It is possible that these mechanisms play a role in the prevention of cardiovascular complications in type 1 diabetes.

While GLP-1RAs, therefore, have great potential as adjunct therapy for type 1 diabetes, mean HbA1c was still ≥7% after liraglutide treatment (6) and larger pharma-sponsored trials reported increased rates of hypoglycemia (8, 9). In seeking further improvement, the advent of sodium-glucose transport protein-2 (SGLT2) inhibitors is of importance since their mode of action is independent of insulinogenesis. Introduced for use in type 2 diabetes, SGLT2 inhibitors are selective antagonists of the sodium-glucose transporter subtype expressed in the proximal convoluted tubules of the kidneys. They act by inhibiting renal glucose re-absorption and therefore stimulating excretion of 60-100 grams per day of glucose in urine. SGLT2 inhibitors have been studied extensively in type 2 diabetes, including the landmark cardiovascular outcome trial EMPA-REG (10), but data have only quite recently begun to accumulate in type 1(11). Short term effects on urine glucose excretion of remogliflozin and empagliflozin are similar to those seen in type 2 diabetes and these translate into improved glycemic and other indices including HbA1c; fasting blood glucose, rates of hypoglycemia, daily insulin dose and weight (12). Weight loss accompanying increased urinary glucose excretion could translate into reduced cardiovascular complications as in type 2 diabetes, but there are no data so far available to support this hypothesis. More recently, in the Phase 3 trial DEPICT-1 we showed that dapagliflozin improves glycemic control and HbA1c over six months by about 0.45% with no severe hypoglycemia or diabetic ketoacidosis (13). Similarly, sotagliflozin has been shown in a Phase 3 trial to allow a larger proportion of patients to achieve HbA1c lower than 7.0% with no severe hypoglycemia although the rate of diabetic ketoacidosis was

higher in the sotagliflozin group (14). Both products are now being reviewed by regulatory agencies for the type 1 diabetes indication.

On the basis of the above, we hypothesize that adding an SGLT2 inhibitor to a combination of insulin and semaglutide (a weekly and potent GLP-1RA) will improve glycemic control in type 1 diabetes such that HbA1c levels of ≤7% are achieved more consistently. Our protocol is designed to allow an objective assessment of whether this triple therapy combination will work effectively and safely so that a majority of people with type 1 diabetes can reach a glycemic target HbA1c of ≤7%. In addition, since both semaglutide and dapagliflozin inhibitors reduce blood pressure and weight, it will allow an assessment of the combined effect of GLP-1RAs and SGLT2 inhibitors on these outcomes. Since there are no previous data on the effects of once weekly semaglutide or the combination of semaglutide and dapagliflozin compared to standard insulin treatment in type 1 diabetes, the design requires allocation of some participants to insulin therapy only.

Due to the potential ketogenic effect of SGLT-2 inhibitors, data from SGLT-2 inhibitors studies in type 1 diabetes were analyzed for it ketogenic effects and a consensus statement and guidelines on management of patients was recently published (Danne T et al., Diabetes Care. 2019 Feb 6).

Preliminary data

1) Liraglutide treatment in patients with type 1 diabetes (4): We treated 8 type 1 diabetes patients; mean age (38.5 years) and duration of diabetes (20±4 yrs), with liraglutide in addition to insulin for 24 weeks. As shown in table 1, there was a significant reduction in fasting and mean blood glucose concentrations, insulin doses, HbA1c and a reduction in appetite and caloric intake with weight loss. In addition, there was a reduction in the excursions of blood glucose concentrations, i.e., the standard deviations of blood glucose concentrations were markedly reduced (**figure 1**). The changes were observed within 48h and improved marginally thereafter with fine tuning of insulin doses and a further reduction in these doses. These observations are noteworthy in that study participants were well controlled and meticulous users of CSII and CGM. They had all tried hard to improve their diabetic control by adjusting insulin doses but had not succeeded because of hypoglycemia. We then demonstrated that poorly controlled and obese individuals with type 1 diabetes also experienced similar benefits of liraglutide treatment (2).

2) Liraglutide As Additional Treatment to Insulin in Patients with Type 1 Diabetes Mellitus: A Randomized Clinical Trial (6): Following the two above non-randomized studies we recently conducted a randomized clinical trial in which 72 individuals (Placebo=18; liraglutide = 54) with TYPE 1 DIABETES for at least one year, on insulin therapy and with no detectable C-peptide in plasma (mean BMI: 30±1; mean body weight: 84±2 kg ; mean HbA1c: 7.57±0.09%; mean age: 44±2 years; mean age of TYPE 1 DIABETES diagnosis: 20±1 years) were randomized to receive placebo, 0.6, 1.2 or 1.8mg of liraglutide daily for 12 weeks. In the 1.2 mg and 1.8 mg groups, mean change in average blood glucose was -10±2 and -10.0±1mg/dl, respectively, ($p<0.0001$ vs. placebo). In the 1.2mg group, HbA1c fell by 0.78% from 7.84±0.17% to 7.06±0.15% ($p<0.0001$, $p<0.01$ vs. placebo) and in the 1.8mg group fell by 0.42% from 7.41±0.15 to 6.99±0.15 ($p=0.001$, $p=0.39$ vs placebo). Time spent between 70 to 160 mg/dl increased by 5±1% ($p<0.05$ vs

placebo) and time between 160-400 mg/dl decreased by 7±1% (p<0.01 vs placebo) in the 1.8 mg group with no additional hypoglycemia. During the 12 week period, the average total daily dose of insulin in the two groups fell by 12.4±3.9 and 10.0±2.3 units respectively (p<0.05 vs baseline and placebo). There was a reduction in body weight (from 83±4 to 78±5 kg, p<0.0001, in the 1.8mg group, from 96±4 to 91±4 kg, p<0.001, in the 1.2 mg group and from 80±4 to 77±4 kg, p<0.01 in the 0.6 mg group). A reduction in daily carbohydrate intake (171±17g vs. 127±18g; p<0.01 and 153±18g vs 115±16g, p<0.01 in the 1.2 mg and 1.8 mg groups, respectively) was also observed over 12 weeks. Systolic BP (SBP) in the 1.8 mg group fell by 3 mmHg (120±2 to 117±3 mmHg, p=0.01). CRP fell by 15±6 and 19±8% in the 1.2 and 1.8mg groups, respectively (p<0.05). Quality of life improved significantly compared to baseline and placebo (unpublished data).

3) Dapagliflozin as an Additional Treatment to Insulin and Liraglutide in Patients with Type 1 Diabetes Mellitus (15): The objective of this randomized clinical trial was to investigate whether addition of dapagliflozin to insulin and liraglutide results in a significant reduction in glycemia and body weight. 30 participants with TYPE 1 DIABETES on liraglutide therapy for at least last 6 months were randomized (in 2:1 ratio) to receive either dapagliflozin 10 mg or placebo daily for 12 weeks. With 12 weeks of dapagliflozin HbA1c fell by 0.66±0.08% from 7.8±0.21% (p < .01 vs placebo) but did not change significantly with placebo (7.40±0.20% to 7.30±0.20%). In one patient within one week of addition of dapagliflozin average glucose fell from 170 to 122 mg/dl while standard deviation was reduced from 71 to 58 (**Figure 2-diminished glycemic excursions)**. Body weight fell by1.9 +/- 0.54kg (p < 0.05 vs placebo). There was no additional hypoglycemia (blood glucose < 3.88 mmol/L; p = 0.52 vs placebo). In the dapagliflozin group, there were significant increases in the plasma concentrations of glucagon by 35% +/- 13% (p < 0.05), hormone-sensitive lipase by 29% +/- 11% (p < 0.05), free fatty acids by 74% +/- 32% (p < 0.05), acetoacetate by 67% +/- 34% (p < 0.05), and beta-hydroxybutyrate by 254% +/- 81% (p < 0.05). Urinary ketone levels also increased significantly (P < .05). None of these changes was observed in the placebo group. Two individuals in the dapagliflozin group developed ketoacidosis. Overall, addition of dapagliflozin to insulin and liraglutide in patients with TYPE 1 DIABETES resulted in a significant improvement in glycemia and in weight loss but tended to increase ketosis.

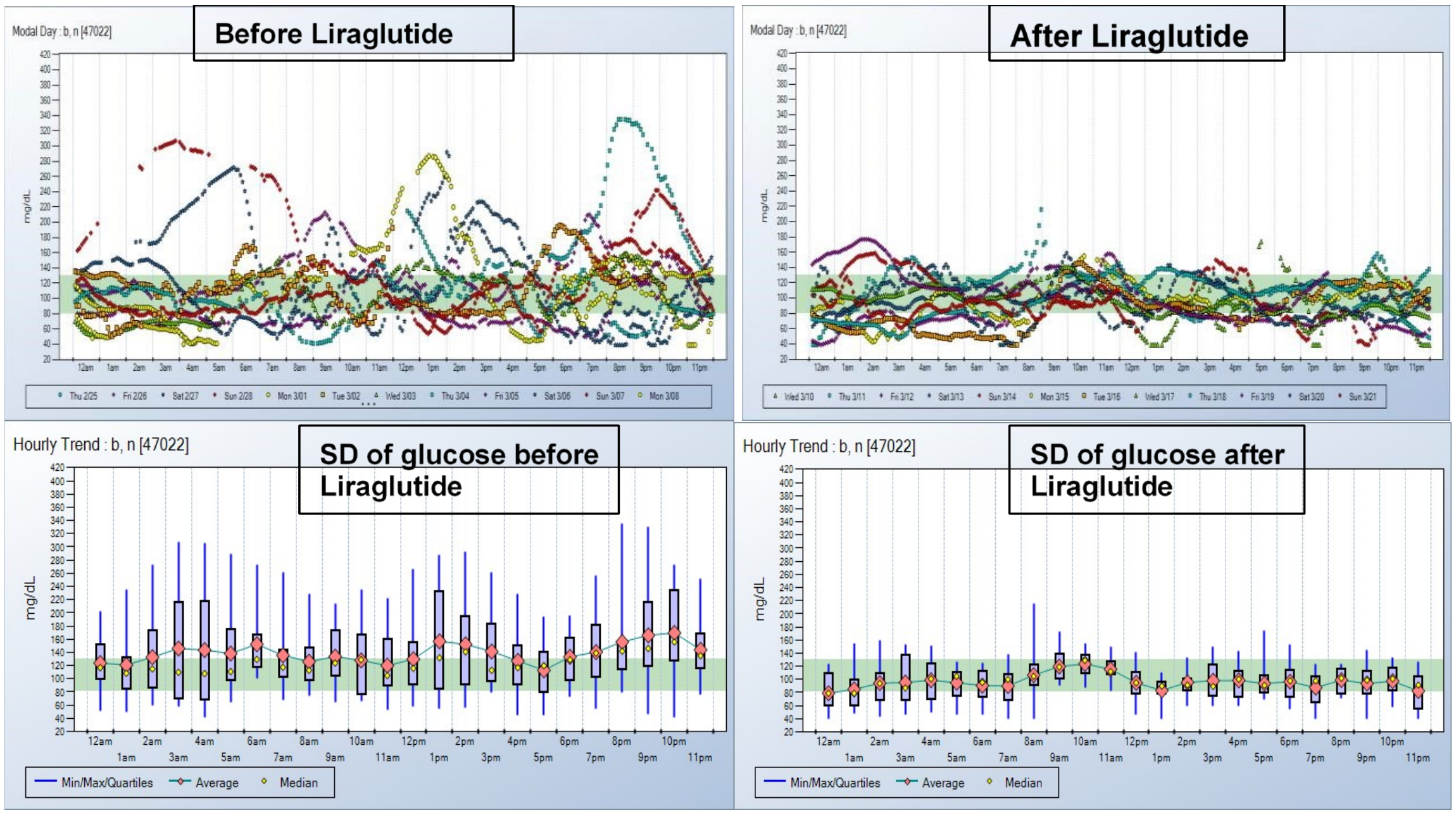

Figure 1: Weekly continuous glucose monitoring record of one patient prior to and following 1 week of liraglutide. Note the glycemic variability (SD) and the frequency of glucose concentrations within the target range prior to and following liraglutide

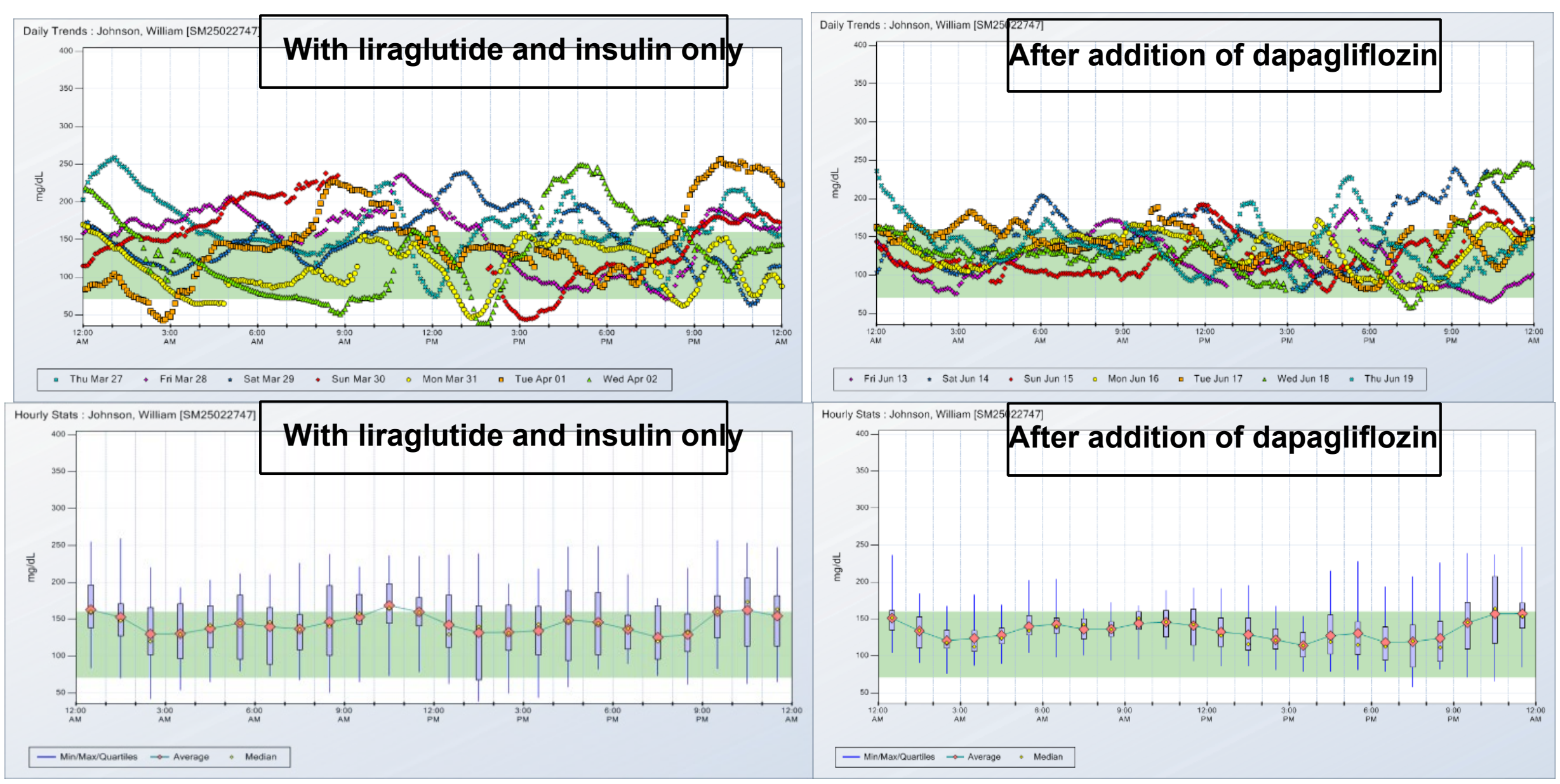

Figure 2: Weekly continuous glucose monitoring record of one patient prior to and following 8 weeks of the triple therapy combination with dapagliflozin and liraglutide. Note the glycemic variability (SD) (bottom) and the frequency of glucose concentrations within the target range prior to and following dapagliflozin addition.

3.2 Include complete citations or references.

Response:

1. Konrad K, Datz N, Engelsberger I, Grulich-Henn J, Hoertenhuber T, Knauth B, et al. Current use of metformin in addition to insulin in pediatric patients with type 1 diabetes mellitus: an analysis based on a large diabetes registry in Germany and Austria. *Pediatr Diabetes.* 2014.
2. Vella S, Buetow L, Royle P, Livingstone S, Colhoun HM, and Petrie JR. The use of metformin in type 1 diabetes: a systematic review of efficacy. *Diabetologia.* 2010;53(5):809-20.
3. Vella S, Buetow L, Royle P, Livingstone S, and Petrie JR. Metformin in type 1 diabetes reduces insulin requirements without significantly improving glycaemic control. Reply to Schatz H [letter]. *Diabetologia.* 2011;54(1):203-4.
4. Varanasi A, Bellini N, Rawal D, Vora M, Makdissi A, Dhindsa S, et al. Liraglutide as additional treatment for type 1 diabetes. *Eur J Endocrinol.* 2011;165(1):77-84.
5. Kuhadiya ND, Malik R, Bellini NJ, Patterson JL, Traina A, Makdissi A, et al. Liraglutide as additional treatment to insulin in obese patients with type 1 diabetes mellitus. *Endocrine practice : official journal of the American College of Endocrinology and the American Association of Clinical Endocrinologists.* 2013;19(6):963-7.
6. Kuhadiya ND, Dhindsa S, Ghanim H, Mehta A, Makdissi A, Batra M, et al. Addition of Liraglutide to Insulin in Patients With Type 1 Diabetes: A Randomized Placebo-Controlled Clinical Trial of 12 Weeks. *Diabetes Care.* 2016;39(6):1027-35.
7. Chaudhuri A, Ghanim H, Makdissi A, Green K, Abuaysheh S, Batra M, et al. Exenatide induces an increase in vasodilatory and a decrease in vasoconstrictive mediators. *Diabetes Obes Metab.* 2017;19(5):729-33.
8. Mathieu C, Zinman B, Hemmingsson JU, Woo V, Colman P, Christiansen E, et al. Efficacy and Safety of Liraglutide Added to Insulin Treatment in Type 1 Diabetes: The ADJUNCT ONE Treat-To-Target Randomized Trial. *Diabetes Care.* 2016;39(10):1702-10.
9. Ahren B, Hirsch IB, Pieber TR, Mathieu C, Gomez-Peralta F, Hansen TK, et al. Efficacy and Safety of Liraglutide Added to Capped Insulin Treatment in Subjects With Type 1 Diabetes: The ADJUNCT TWO Randomized Trial. *Diabetes Care.* 2016;39(10):1693-701.
10. Zinman B, Wanner C, Lachin JM, Fitchett D, Bluhmki E, Hantel S, et al. Empagliflozin, Cardiovascular Outcomes, and Mortality in Type 2 Diabetes. *N Engl J Med.* 2015;373(22):2117-28.
11. Petrie JR. SGLT2 inhibitors in type 1 diabetes: knocked down, but up again? *Lancet Diabetes Endocrinol.* 2017;5(11):841-3.
12. Mudaliar S, Armstrong DA, Mavian AA, O'Connor-Semmes R, Mydlow PK, Ye J, et al. Remogliflozin etabonate, a selective inhibitor of the sodium-glucose transporter 2, improves serum glucose profiles in type 1 diabetes. *Diabetes Care.* 2012;35(11):2198-200.
13. Dandona P, Mathieu C, Phillip M, Hansen L, Griffen SC, Tschope D, et al. Efficacy and safety of dapagliflozin in patients with inadequately controlled type 1 diabetes (DEPICT-1): 24 week results from a multicentre, double-blind, phase 3, randomised controlled trial. *Lancet Diabetes Endocrinol.* 2017;5(11):864-76.

14. Garg SK, Henry RR, Banks P, Buse JB, Davies MJ, Fulcher GR, et al. Effects of Sotagliflozin Added to Insulin in Patients with Type 1 Diabetes. *N Engl J Med.* 2017;377(24):2337-48.
15. Kuhadiya ND, Ghanim H, Mehta A, Garg M, Khan S, Hejna J, et al. Dapagliflozin as Additional Treatment to Liraglutide and Insulin in Patients With Type 1 Diabetes. *J Clin Endocrinol Metab.* 2016;101(9):3506-15.
16. Henry RR, Thakkar P, Tong C, Polidori D, and Alba M. Efficacy and Safety of Canagliflozin, a Sodium-Glucose Cotransporter 2 Inhibitor, as Add-on to Insulin in Patients With Type 1 Diabetes. *Diabetes Care.* 2015;38(12):2258-65.
17. Peters AL, Buschur EO, Buse JB, Cohan P, Diner JC, and Hirsch IB. Euglycemic Diabetic Ketoacidosis: A Potential Complication of Treatment With Sodium-Glucose Cotransporter 2 Inhibition. *Diabetes Care.* 2015;38(9):1687-93.
18. Ahmann AJ, Capehorn M, Charpentier G, Dotta F, Henkel E, Lingvay I, et al. Efficacy and Safety of Once-Weekly Semaglutide Versus Exenatide ER in Subjects With Type 2 Diabetes (SUSTAIN 3): A 56-Week, Open-Label, Randomized Clinical Trial. *Diabetes Care.* 2017.
19. Andrews NC, and Faller DV. A rapid micropreparation technique for extraction of DNA-binding proteins from limiting numbers of mammalian cells. *Nucleic Acids Res.* 1991;19(9):2499.
20. Perkins BA, Cherney DZ, Partridge H, Soleymanlou N, Tschirhart H, Zinman B, et al. Sodium-glucose cotransporter 2 inhibition and glycemic control in type 1 diabetes: results of an 8-week open-label proofof-concept trial. *Diabetes Care.* 2014;37(5):1480-3.
21. Cherney DZ, Perkins BA, Soleymanlou N, Maione M, Lai V, Lee A, et al. Renal hemodynamic effect of sodium-glucose cotransporter 2 inhibition in patients with type 1 diabetes mellitus. *Circulation.* 2014;129(5):587-97.

4.0 Study Design

4.1 Describe and explain the study design (e.g. case-control, cross-sectional, ethnographic, experimental, interventional, longitudinal, observational).

Response: This will be a two-center, randomized, open-label then double-blind, parallel group, placebo and standard of care controlled prospective study in type 1 diabetes. One hundred and (114) patients will be randomized (open label, see below) into three groups (38 each) and will be either maintained on their regular insulin therapy for 12 months (standard therapy group) or started on semaglutide in addition to regular insulin treatment for six month (dual therapy and triple therapy groups). For those on insulin and semaglutide, after six months of stable treatment, dapagliflozin (triple therapy group) or placebo (dual therapy group) treatment will be initiated for an additional six months according to a second randomization (double-blind).

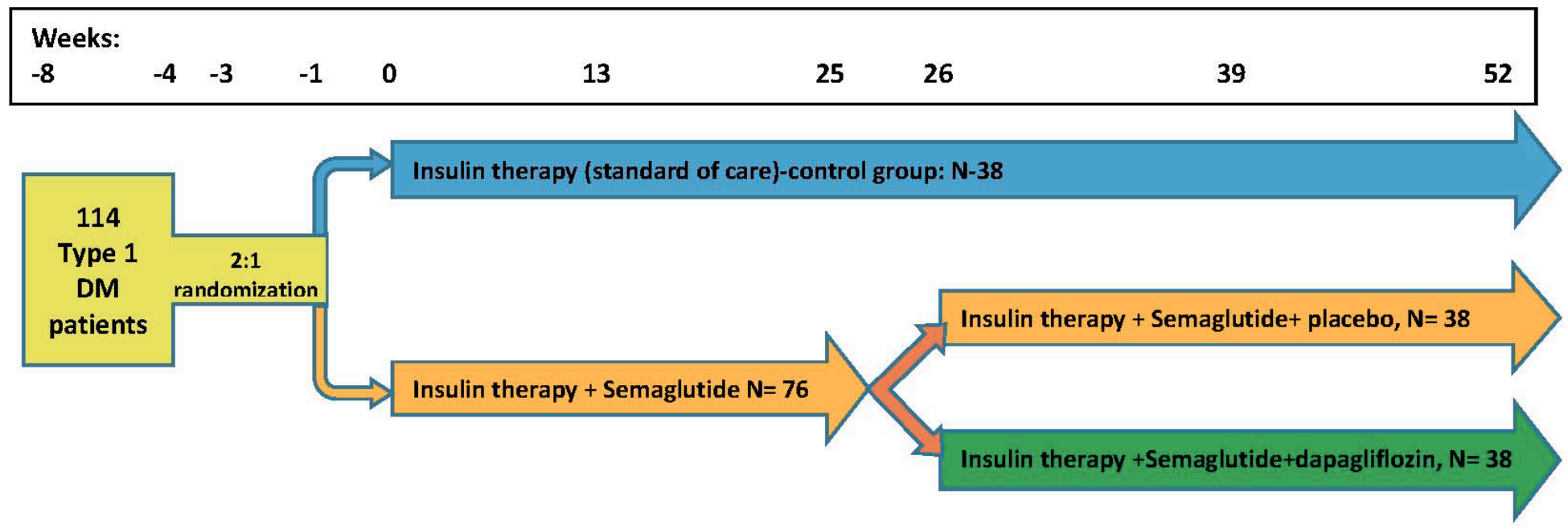

5.0 Local Number of Subjects

5.1 Indicate the total number of subjects that will be enrolled or records that will be reviewed locally.

Response: 57 patients will be enrolled locally with another 57 patients recruited at the University of Glasgow site: a total of 114.

5.2 If applicable, indicate how many subjects you expect to screen to reach your target sample (i.e. your screen failure rate).

Response: All screened and qualified patients will be enrolled and randomized

5.3 Justify the feasibility of recruiting the proposed number of eligible subjects within the anticipated recruitment period. For example, how many potential subjects do you have access to? What percentage of those potential subjects do you need to recruit?

Response: The Diabetes and Endocrinology Center of WNY is the largest Diabetes center in the WNY area, seeing between 60 to 100 type 1 diabetic patient every month. Therefore, the majority of recruited patients are our clinic patients. We also recruit a study participants through advertisement and other physician's references. These sources will suffice to recruit the needed number to subjects. The Glasgow center (Stobhill Ambulatory Care Hospital) reviews more than 300 patients per month.

6.0 Inclusion and Exclusion Criteria

*6.1 Describe the criteria that define who will be **included** in your final study sample.*

NOTE: This may be done in bullet point fashion.

Response:
Inclusion Criteria

1) Type 1 Diabetes for at least 1 year on staple use of continuous subcutaneous insulin infusion (CSII) or multiple daily (four or more) injections (MDI) of insulin for last 3 months.
2) C-peptide <0.23 nM
3) Minimum dose of insulin in Units/kg at entry: 0.5 U/kg for MDI and 0.4 U/kg for CSII
4) Regularly measuring blood sugars four or more times daily or use of CGM.
5) HbA1c between7.5-11% inclusive.
6) Well versed in CHO counting*
7) Age 18-70 years.
8) BMI ≥25 kg/m^2.

*In the opinion of the site Principal Investigator

6.2 Describe the criteria that define who will be ***excluded*** *from your final study sample.*

NOTE: This may be done in bullet point fashion.

Response:

Exclusion Criteria

1) Type 1 diabetes for less than 12 months, type 2 diabetes, chronic pancreatitis, MODY
2) Previous use of any agent other than insulin for treatment of diabetes in the last 3 months.
3) History of diabetic ketoacidosis (DKA) requiring medical intervention (e.g., emergency room visit and/or hospitalization) within 3 months prior to the screening visit
4) Patients on ketogenic or low (<50g/day) carbohydrate diet or who practice intermittent fasting.
5) Frequent episodes of severe hypoglycemia as defined by more than one episode requiring medical assistance, emergency care (paramedics or emergency room care), and/or glucagon therapy administered by a third-party individual within 1 month prior to the screening visit
6) Symptoms of poorly controlled diabetes that would preclude participation in this trial
7) Subjects on a commercial weight loss program with ongoing weight loss, or on an intensive exercise program
8) History of bariatric surgery or lap-band procedure within 12 months prior to screening
9) History of Addison's disease or chronic adrenal insufficiency
10) History of diabetes insipidus
11) Hepatic disease or cirrhosis
12) Aspartate Aminotransferase (AST) > 3X Upper limit of normal (ULN) *and/or* Alanine aminotransferase (ALT) > 3X ULN
13) Serum Total Bilirubin > 2X ULN unless exclusively caused by Gilbert's Syndrome

14) Hemoglobin < 11.0 g/dL (110 g/L) for men; hemoglobin < 10.0 g/dL (100 g/L) for women.
15) Coronary event or procedure (myocardial infarction, unstable angina, coronary artery bypass, surgery or coronary angioplasty) in the previous 3 months or patients with congestive heart failure.
16) ESRD on hemodialysis; and or e-GFR < 60 ml/min/1.73m^2
17) HIV or Hepatitis B/C positive status
18) Any other life-threatening, noncardiac disease
19) History of pancreatitis
20) Women who are pregnant or women of childbearing potential who are not using adequate contraception or who are breast feeding
21) Inability to give informed consent
22) History of gastroparesis
23) History of medullary thyroid carcinoma or MEN 2 syndrome
24) History of serious hypersensitivity reaction to these agents
25) Painful gallstones
26) Alcoholism
27) Hypertriglyceridemia (>500 mg/dl)
28) Recurrent genital mycotic infection.
29) Hypovolemic patients or with chronic renal insufficiency.
30) Patients with any malignancy except treated in situ malignancy and basal cell carcinoma of the skin
31) Unexplained hematuria
32) Patients with a history of diabetic retinopathy
33) Use of an investigational agent or therapeutic regimen within 30 days of study
34) Participation in any other concurrent interventional clinical trial

6.3 Indicate specifically whether you will include any of the following special populations in your study using the checkboxes below.

NOTE: Members of special populations may not be targeted for enrollment in your study unless you indicate this in your inclusion criteria.

Response: NA

☐ Adults unable to consent

☐ Individuals who are not yet adults (infants, children, teenagers)

☐ Pregnant women

☐ Prisoners

6.4 Indicate whether you will include non-English speaking individuals in your study. ***Provide justification if you will exclude non-English speaking individuals.***

*In order to meet one of the primary ethical principles of equitable selection of subjects, non-English speaking individuals may **not** be routinely excluded from research as a matter of convenience.*

In cases where the research is of therapeutic intent or is designed to investigate areas that would necessarily require certain populations who may not speak English, the researcher is required to make efforts to recruit and include non-English speaking individuals. However, there are studies in which it would be reasonable to limit subjects to those who speak English. Some examples include pilot studies, small unfunded studies with validated instruments not available in other languages, studies with numerous questionnaires, and some non-therapeutic studies which offer no direct benefit.

Response:

We have no non-English speaking patients in this population. We have patients that English is a second language, but they are able to read, write and understand it. This population is less than 10% of the total population. No benefit is being withheld from not participating in the study.

7.0 Vulnerable Populations

*If the research involves special populations that are considered vulnerable, **describe the safeguards included to protect their rights and welfare.***

NOTE: You should refer to the appropriate checklists, referenced below, to ensure you have provided adequate detail regarding safeguards and protections. You do not, however, need to provide these checklists to the IRB.

*7.1 For research that involves **pregnant women,** safeguards include:*
NOTE CHECKLIST: Pregnant Women (HRP-412)

Response: We will not be recruiting subjects from vulnerable populations

☒ **N/A**: This research does not involve pregnant women.

*7.2 For research that involves **neonates of uncertain viability or non-viable neonates,** safeguards include:*
NOTE CHECKLISTS: Non-Viable Neonates (HRP-413), or Neonates of Uncertain Viability (HRP-414)

Response:

☒ **N/A:** This research does not involve non-viable neonates or neonates of uncertain viability.

*7.3 For research that involves **prisoners**, safeguards include:*
NOTE CHECKLIST: Prisoners (HRP-415)

Response:

☒ **N/A:** This research does not involve prisoners.

7.4 For research that involves ***persons who have not attained the legal age for consent to treatments or procedures involved in the research ("children")****, safeguards include:*
NOTE CHECKLIST: Children (HRP-416)

Response:

☒ **N/A:** This research does not involve persons who have not attained the legal age for consent to treatments or procedures ("children").

7.5 For research that involves ***cognitively impaired adults****, safeguards include:*
NOTE CHECKLIST: Cognitively Impaired Adults (HRP-417)

Response:

☒ **N/A:** This research does not involve cognitively impaired adults.

7.6 Consider if other specifically targeted populations such as students, employees of a specific firm, or educationally or economically disadvantaged persons are vulnerable. ***Provide information regarding their safeguards and protections, including safeguards to eliminate coercion or undue influence.***

Response: No specific populations or vulnerable groups will be targeted. All subjects enrolled in this study will be of legal adult consenting age with the ability to speak, read and interpret the English language. Patients will have the ability to speak with the research team regarding any questions or concern they have before signing the consent. Research team have the ability to evaluate patient's cognitive abilities through these interactions with patients. Patients are made aware that this study is voluntary and they are able to stop participating at any time they feel uncomfortable. Patients are not be pressured into participating and their clinic standard of care will remain the same if they participate or choose not to participate.

8.0 Eligibility Screening

8.1 Describe ***screening procedures*** *for determining subjects' eligibility.*
Screening refers to determining if prospective participants meet inclusion and exclusion criteria.

Include all relevant screening documents with your submission (e.g. screening protocol, script, questionnaire).

Response:

After obtaining partial HIPAA waiver, patient charts will be screened according to the study inclusion and exclusion criteria by our trained clinical staff and physicians. In additions, the study clinical team will evaluate their clinic patients for possible participation in this study according to the inclusion and exclusion criteria at the Diabetes and Endocrinology Center of WNY clinics. Physicians will

speak to them about their interests in participating in research. If the patient agrees, their information will be given to the research coordinator to be contacted for further evaluation. The research coordinator will contact interested patients and those meeting the inclusion and exclusion criteria based on preliminary phone evaluation will be invited to participate in the study. All personal information will be kept confidential and locked in the coordinator office.

Prospective participants will be asked to read the consent and any questions they may have regarding the protocol will be answered. If the subject wants to participate in the study, they will be asked to sign the informed consent form. The subject's medical history and current medications will be obtained as well as their blood pressure and vitals. A physical examination will also be done. Blood and urine samples will be taken in order to evaluate HbA1c, CBC, CMP and liver and kidney functions and pregnancy status. Females of childbearing age, will have to be on two forms of birth control to prevent pregnancy. Patients meeting all the inclusion and exclusion criteria based on all screening tests will be enrolled in the study.

☐ **N/A:** There is no screening as part of this protocol.

9.0 Recruitment Methods

☐ **N/A:** This is a records review only, and subjects will not be recruited. NOTE: If you select this option, please make sure that all records review procedures and inclusion/exclusion screening are adequately described in other sections.

9.1 Describe when, where, and how potential subjects will be recruited.

NOTE: Recruitment refers to how you are identifying potential participants and introducing them to the study. Include specific methods you will use (e.g. searching charts for specific ICD code numbers, Research Participant Groups, posted advertisements, etc.).

Response:

Participants will be identified by prescreening clinical charts, patient doctor interaction at the time of their visits to the Diabetes Endocrinology Center of WNY Locations include:

1. 1020 Youngs Road, Williamsville NY 14221
2. 705 Maple Road, Williamsville NY 14221
3. 462 Grider Street, Buffalo NY 14215
4. 1000 Youngs Road, Suite 105, Williamsville NY 14221

Patients can be also referred by other physicians and providers

The remainder of the participants (57 patients) will be recruited from NHS Greater Glasgow and Clyde Diabetes clinics (Scotland, UK). Separate local IRB/ Ethical approval will be sought to cover this site (based on discussions with UBIRB director).

The study clinical team will evaluate their clinic patients for possible participation in this study according to the inclusion and exclusion criteria at the Diabetes and Endocrinology Center of WNY clinics. Patients that may qualify for the study are referred to the research team for further eligibility evolution. Patients meeting the inclusion and exclusion criteria based on preliminary phone evaluation will be invited to participate in the study.

Participants will also be identified by flyers, advertisements on Facebook and Craigslist and researchmatch.org. The study will also be put on UB's Study Information Portal (SIP) website. Along with this, a radio ad will be on the air of the radio station STAR102.5 at various times during the day during the month of mid January to mid February

9.2 Describe how you will protect the privacy interests of prospective subjects during the recruitment process.

NOTE: Privacy refers to an individual's right to control access to him or herself.

Response: Patient charts will be screened according to the study inclusion and exclusion criteria by our trained clinical staff and physicians. If the patient qualifies and is of consenting age, the physicians will speak to them about their interests in participating in research in a private room where no one else can hear the conversation. If the patient agrees, their information will be given to the research coordinator to be contacted for further evaluation. No personal information will be shared without patient's approval and will not be used for any purpose except for this research. All personal information will be kept confidential and locked in the coordinator office.

9.3 Identify any materials that will be used to recruit subjects.

NOTE: Examples include scripts for telephone calls, in person announcements / presentations, email invitations.

For advertisements, include the final copy of printed advertisements with your submission. When advertisements are taped for broadcast, attach the final audio/video tape. NOTE: You may submit the wording of the advertisement prior to taping to ensure there will be no IRB-required revisions, provided the IRB also reviews and approves the final version.

Response: In addition to screening clinical charts, participations will be identified through; flyer advertisement, researchmatch.org, craigslist.com, and Facebook. The study will also be put on UB's Study Information Portal (SIP) website.

10.0 Procedures Involved

*10.1 Provide a description of **all research procedures or activities** being performed and when they are performed once a subject is screened and determined to be eligible. Provide as much detail as possible.*

NOTE: This should serve as a blueprint for your study and include enough detail so that another investigator could pick up your protocol and replicate the research. For studies that have multiple or complex visits or procedures, consider the addition of a schedule of events table in in your response.

Response:

Each patient will spend about 55 weeks in the trial comprising three weeks of monitoring to stabilize baselines and ensure adherence and commitment to participation before randomization into an intervention arm for an additional period of 52 weeks. Following a completed screening visit and meeting the inclusion and exclusion criteria, each patient will have a total of 16 study visits. At weeks 3, 5, 8, 16, 20, 29, 35, 44 and at 2 weeks after the end of the study, each patient will receive a phone call to document any adverse effects or other changes. There will be no long term follow-up following the end of the study.

Participants will be treated with either insulin only (control arm) for the whole duration of the study or with insulin and semaglutide weekly s.c. injection (titrated up to 1 mg or the maximum tolerated dose, see below) for the first six months then either dapagliflozin (Triple therapy arm) or placebo (dual therapy) daily will be added for an additional six months. Semaglutide will be started at a dose 0.25 mg once weekly for two weeks then increased to 0.5 mg dose. If well tolerated it will be increased to 1 mg once weekly at week 4. Dapagliflozin (or placebo) will be started at a dose of 5 mg daily at week 26 and if well tolerated increased after 1 week to 10 mg (2x5mg tablets) daily to ensure drug tolerability and to avoid any hypoglycemia or hypotension. Patients who do not tolerate the increase in dosing of either medications will be maintained at the highest tolerated dose.

Treatments used

1- Insulin: All patients (114 patients) will continue to self-administer their prescribed insulin as part of their standard of care. Insulin dose will be titrated during the study to control blood glucose average between 90-180 mg/dl and to avoid hypoglycemia (blood sugar <70 mg/dl) or hyperglycemia (blood sugar >250 mg/dl). Among all patients, 38 patients will be randomized (open-label) to remain on insulin therapy only (standard therapy group) while the other 76 will start the investigational therapies.
2- Semaglutide: a once weekly a GLP-1 mimetic injection (pen) indicated for type 2 diabetes. Doses to be used: start at 0.25mg then increase to 0.5mg following 2 weeks and to 1mg weekly s.c. injection 2 weeks later. Semaglutide will be added to a background of insulin treatment for 12 months in 76 patients who will be randomized (double-blind) into 2 groups (dual and triple therapy groups) at the end of 26 weeks.
3- Farxiga (dapagliflozin): a once daily SGLT-2 inhibitor tablet indicated for type 2 diabetes. Doses used: starting with 5mg and increase after 1 week to 10mg oral administration daily. Dapagliflozin will be started according to the randomization (Interactive Web System, hosted by University of Glasgow) in 38 patients at 26 weeks as an additional treatment on a background of insulin and semaglutide therapy and end at 52 weeks (triple therapy group).

4- Placebo: dapagliflozin matching placebo (supplied by Astra Zeneca) will be started in 38 patients at 26 weeks as an additional treatment on a background of insulin and semaglutide therapy and end at 52 weeks (dual therapy group).

As shown in **Table 1,** fasting blood and urine samples will be obtained prior to drug administration (weeks 0) and at 13, 26, 39 and 52 weeks after the commencement of the study. Optional fat biopsies from the abdominal subcutaneous tissue will be collected at weeks 0, 26 and 52. Continuous Glucose Monitoring (Dexcom G6 with blinded receiver) will be performed for three 21-days periods on weeks -3 to 0, 23 to 26 and from 49 to 52 weeks. An optional 24hr blood pressure monitoring will be performed at baseline (week -1) and at 13, 25, 39 and 51 weeks in order to characterize the time course of antihypertensive effects, which may be early (i.e. independent of weight loss) as indicated by our preliminary data. High fat high caloric (HFHC) Meal challenges will be performed at baseline (week -1) and at 25 and 51 weeks with assessment of glucose excursions and postprandial glucagon (AUC) as well as postprandial inflammation and oxidative stress. Telephone calls will be conducted at weeks 3, 5, 8, 16, 20, 29, 35, 44 and at two weeks after the end of the study to document any side effects or any other patient's reported issues or comments.

Study Visits:

<u>Screening Visit (Screening Visit, Week -8 to -4):</u> Patients will be asked to read and understand the consent and any questions regarding the protocol will be answered. If they want to participate in the study, they will be asked to sign the informed consent form. Their medical history and current medications will be obtained as well as blood pressure and vitals. A physical examination will also be done. Blood samples will be taken in order to evaluate HbA1c, CBC, CMP, C-peptide, kidney and liver functions, ketone bodies, and urine for pregnancy status in females.

<u>Study Visit 1 (Week -3):</u> Participants who meet entry criteria will be scheduled to for their next visit at -3 week. Vitals will be taken including resting (min. 10 minutes) blood pressure measurement (will be repeated from same arm every visit). Insulin pump will be downloaded and/or CGM/fingerstick blood sugar will be reviewed and the appropriate insulin adjustments will be made by the investigator to target fasting blood glucose concentrations between 90-180 mg/dl if possible at all times without having additional hypoglycemia (less than 70 mg/dl). Blood sugars and ketone bodies will be measured via a fingerstick blood sample using glucose and ketones meter (Abbott Laboratories) and daily blood sugars collected and reviewed. Urine for pregnancy status and ketone bodies assessment. We will instruct patients on how to use and insert the Continuous Glucose Monitoring System (Dexcom G6 with blinded receiver). All subjects will be advised to monitor their capillary blood glucose by CGM or fingerstick at least 4 times a day (before meals and at bedtime) through-out the study and to wear their CGM constantly for next 20 day. Carb counting technique will be reviewed and any questions answered on how to use the pump or calculate insulin doses.

<u>Study Visit 2 (Week -1±2days):</u> Patients will be asked to come in the fasting state (no food or calorie intake for 10-12 hours). Their vitals will be taken. Blood sugars and ketone bodies will be measured via a CGM/fingerstick blood sample using glucose and ketones meter (Abbott Laboratories) to assess their glycemic control prior to meal challenge. If their blood sugar is below 70mg/dl or more than 250mg/dl the visit will be rescheduled for an alternative date. Insulin pump will be downloaded and/or CGM/fingerstick blood sugar will be reviewed and insulin adjustment will be made if necessary. An IV port will be placed in order to obtain blood samples throughout the duration of the visit. Patients will undergo a meal challenge test (described below). At the end of the meal test, they will be asked to wear a 24 hour blood pressure monitor (optional). A 24 hr urine container will be dispensed to collect a 24 hour urine sample to bring in at the Week 0 visit. Daily Glucose logs will be reviewed.

<u>Study visit 3 (Week 0±2days):</u> Patients will be asked to come in the fasting state (no food or calorie intake for 10-12 hours). Vitals will be taken. Blood sugars and ketone bodies will be measured via a CGM/fingerstick blood sample using glucose and ketones meter (Abbott Laboratories) and daily blood sugars collected and reviewed. The 24 hour urine container will be collected. A fasting blood sample will be taken (45 mL) to evaluate study endpoints, CBC, CMP, HbA1c, and fructosamine. A urine sample will be collected to evaluate ketone bodies and, in females, pregnancy status. Insulin pump will be downloaded and/or CGM/fingerstick blood sugar will be reviewed and the appropriate insulin adjustments will be made by the investigator. In general, insulin dose adjustments should not be >10% from current total insulin dose or >20% from baseline (week 0) total insulin dose. They will discontinue use of the study CGM. An optional fat biopsy will be performed. We will ask them to fill out quality of life questionnaires. Study drug will be dispensed depending on what group they are randomized to. This includes either (1) staying on the insulin regimen given by their doctor, or (2) a combination of their current insulin regimen and a once daily injection of semaglutide. Semaglutide will be started on site at the 0.25 mg dose. The 24 hr blood pressure monitor will be collected.

<u>Study Visit 4 (Week 1±2days)</u>: Vitals will be taken. Blood sugars and ketone bodies will be measured via a CGM/fingerstick blood sample using glucose and ketones meter (Abbott Laboratories) and daily blood sugars collected and reviewed. Insulin dose will be adjusted if needed as in visit 3. At any time, if HbA1c is <7%, insulin dose adjustment should not be >5%. Semaglutide 0.25mg will be injected on site. Participants will be instructed to call their research center or their doctor directly in case of any problem or untoward side effects. They will be specifically asked to call if they have unmanaged hypoglycemia (blood sugar <70 mg/dl) or hyperglycemia (blood sugar >250 mg/dl) or if occurring in more than one occasion.

<u>Study Visit 5 (Week 2±2days):</u> Vitals will be taken. Blood sugars and ketone bodies will be measured via a CGM/fingerstick blood sample using glucose and ketones meter (Abbott Laboratories) and daily blood sugars collected and reviewed. Insulin dose will be adjusted if needed as in visit 3. Semaglutide 0.5mg dose will be injected on site. If they are experiencing mild nausea, they will be continued on the 0.25 mg dose for another week.

Supply of the drug will be given to last until the next visit. Patients will be advised to increase the study dose to 0.5 mg a day if they are ok.

<u>Study Visit 6 (Week 4±1 week):</u> Patients will be asked to bring in used study pens for drug accountability (to be recorded in eCRF). Vitals will be taken. Blood sugars and ketone bodies will be measured via a CGM/fingerstick blood sample using glucose and ketones meter (Abbott Laboratories) and daily blood sugars collected and reviewed. Insulin dose will be adjusted if needed. Supply of the drug will be given to last until the next visit. A urine sample will be collected to evaluate ketone bodies and, in females, pregnancy status. Patients will be advised to increase the study dose to 1.0 mg a day. If they experience mild nausea, they will be continued on the 0.5 mg dose for another week. At all times, patients will be maintained at the maximum tolerated dose. A 24 hr urine container will be dispensed to collect a 24 hour urine sample to bring in at the Week 13 visit.

<u>Study Visit 7 (Week 13±1 week):</u> Patients will be asked to come in the fasting state (10-12 hours fasting), and to bring in your used study pens for drug accountability. Blood sugars and ketone bodies will be measured via a CGM/fingerstick blood sample using glucose and ketones meter (Abbott Laboratories) and daily blood sugars collected and reviewed. Insulin dose will be adjusted if needed as in. Supply of the drug will be given to last until visit 26. Vitals will be taken. A urine sample will be collected to evaluate ketone bodies and, in females, pregnancy status. The 24 hour urine container will be collected. A fasting blood sample will be taken (45 mL) to evaluate study endpoints, CBC, CMP, HbA1c, and fructosamine. Patients who agreed to participate in the24 hour blood pressure monitoring will be asked to wear it for the next 24 hr.

<u>Study Visit 8 (Week 23±1 week):</u> Patients will be asked to bring in used study pens for drug accountability. Vitals will be taken. Blood sugars and ketone bodies will be measured via a CGM/fingerstick blood sample using glucose and ketones meter (Abbott Laboratories) and daily blood sugars collected and reviewed. A urine sample will be collected to evaluate ketone bodies and, in females, pregnancy status. Insulin dose will be adjusted if needed. All subjects will be reminded to monitor their capillary blood glucose by fingerstick at least 4 times a day (before meals and at bedtime) through-out the study. CGM will be inserted and patients asked to wear their CGM constantly for next 20 day.

<u>Study visit 9 (Week 25±1 week):</u> Patients will be asked to come in the fasting state, and to bring in used study pens for drug accountability. Vitals will be taken. A urine sample will be collected to evaluate ketone bodies. Blood sugars and ketone bodies will be measured via a CGM/fingerstick blood sample using glucose and ketones meter (Abbott Laboratories) and daily blood sugars collected and reviewed. Insulin dose will be adjusted if needed. HbA1c will be drawn. Supply of the drug will be given to last until the next visit. A 24 hr urine container will be dispensed to collect a 24 hour urine sample to bring in at the Week 26 visit. A HFHC Meal challenges will be performed as in visit 2. Patients who agreed to participate will be asked to wear a 24 hour blood pressure monitoring for the next 24 hr.

Participants in the semaglutide group will be provided supplies for urine ketones measurement (KetoStix) and blood ketones meter. Research coordinator will instruct the

patients on the use of meter and reading of the measuring strips. Patients will be instructed to start measuring urinary and blood ketones every morning for three consecutive days prior to next visit (starting dapagliflozin treatment) and to report results at the time of their next visit.

Study visit 10 (Week 26±1 week): Patients will be asked to come in the fasting state, and to bring in used study pens for drug accountability. Previous 3 days blood and urine ketones levels will be reviewed. Patients with normal fasting urine (<+, or < small) or blood ketone (<0.6 mmol) will proceed to next treatment phase included in this visit. Patients who report higher ketone values in at least 1 out of the 3 days will have their glucose levels, insulin dose, carb intake reviewed and managed as necessary. They will be rescheduled for next week (7±1 day) and asked to repeat the 3 days testing before next visit.
Vitals will be taken. A urine sample will be collected to evaluate ketone bodies. Blood sugars and ketone bodies will be reviewed via a CGM/fingerstick blood sample using glucose and ketones meter (Abbott Laboratories) and daily blood sugars collected and reviewed. Insulin dose will be adjusted if needed. In general, insulin dose adjustments should **NOT** be >10% from current total insulin dose (or 5% if HbA1c is <7%) or >20% from baseline total insulin dose. The adjusted total insulin dose in this visit will serve as the baseline total insulin dose for the remaining 6 months of the study and future limits of dose adjustments will be based on this new baseline. The CGM will be collected. A urine sample will be collected to evaluate ketone bodies and, in females, pregnancy status. The 24 hour urine container will be collected. A fasting blood sample will be taken (45 mL) to evaluate study endpoints, CBC, CMP, C-peptide, and Fructosamine. An optional fat biopsy will be performed. We will ask patients to fill out quality of life questionnaires. At this point, those patients randomized to receive semaglutide, they will now be randomized to receive an actual dapagliflozin pill or a placebo pill that looks like dapagliflozin. If they are randomized to receive actual dapagliflozin, they will start at the 5 mg dose for one week. Supply of the study drug will be given to last until week 27.
All patients will be asked to perform a daily (preferably early morning or before bedtime) urinary test of ketone bodies using KetoStix and record all outcomes. If results of these scheduled (i.e. not done due to symptoms) ketone measurements, are small or higher (+1 or more), they will be instructed to repeat the measurement after 60 min. If consecutive measurements show moderate or higher ketones in urine, the patient will be instructed to call the research center or their doctor directly. Patients with dual glucose and ketones meters will be instructed to record and report their blood ketones in dedicated study diaries. If blood ketones are 0.6 mM or above, they should repeat the measurement within 60 min. If consecutive measurements show 0.6mm or higher ketones in blood, the patients will be instructed to call the research center or their doctor directly. For any abnormal ketone levels taken due to symptoms, the DKA consensus guidelines (Danne T et al., Diabetes Care. 2019 Feb 6) are to be followed instead (as in section 21).
Participants will be instructed to call their research center or their doctor directly in case of any problem or untoward side effects. They will be specifically asked to call if they have unmanaged hypoglycemia (blood sugar <70 mg/dl) or hyperglycemia (blood sugar >250 mg/dl) or if occurring in more than one occasion. Participants will be instructed to remain hydrated and report any genital mycotic or urinary tract infections.

Study visit 11 (Week 27±2 days): Patients will be asked to bring in used study pens for drug accountability (recorded in eCRF). Vitals will be taken. Daily blood sugars and urinary ketones measurements using KetoStix will be collected and reviewed. Blood sugars and ketone bodies will be measured via a CGM/fingerstick blood sample using glucose and ketones meter (Abbott Laboratories). A urine sample will be collected to evaluate urine ketone level. Insulin dose will be adjusted if needed. In general, insulin dose adjustments should not be >10% from current total insulin dose (or 5% if HbA1c is <7%) or >20% from NEW baseline total insulin dose established at visit 10. If at any time during the study total insulin dose reduction is >20% and total insulin dose in <0.40 U/Kg, additional monitoring protocol (see later) will be implemented. Supply of the drug will be given to last until week 39. If patients are receiving actual dapagliflozin, they will now start at the 10 mg dose for the remainder of the study.

Study Visit 12 (Week 31±1 week): Patients will be asked to bring in used study pens and tablets for drug accountability. Vitals will be taken. Daily blood sugars and urinary ketones measurements using KetoStix will be collected and reviewed. Blood sugars and ketone bodies will be measured via a CGM/fingerstick blood sample using glucose and ketones meter (Abbott Laboratories). A urine sample will be collected to evaluate ketone bodies and, in females, pregnancy status. Insulin dose will be adjusted if needed as in visit 11. A blood sample will be taken (15 mL) to evaluate CBC and CMP. A 24 hr urine container will be dispensed to collect a 24 hour urine sample to bring in at the Week 39 visit.

Study visit 13 (Week 39±1 week): Patients will be asked to come in the fasting state, and to bring in used study pens and tablets for drug accountability. Vitals will be taken. Daily blood sugars and urinary ketones measurements using KetoStix will be collected and reviewed. Blood sugars and ketone bodies will be measured via a CGM/fingerstick blood sample using glucose and ketones meter (Abbott Laboratories). Insulin dose will be adjusted if needed as in visit 11. Supply of the drug will be given to last until the end of the study. A urine sample will be collected to evaluate ketone bodies and, in females, pregnancy status. The 24 hour urine container will be collected. A fasting blood sample will be taken (45 mL) to evaluate study endpoints, CBC, CMP, HbA1c, and Fructosamine. Patients who have provided consent will be asked to wear a 24 hour blood pressure monitor.

Study visit 14 (Week 49±1 week): Patients will be asked to bring in used study pens and tablets for drug accountability. Vitals will be taken. Daily blood sugars and urinary ketones measurements using KetoStix will be collected and reviewed. Blood sugars and ketone bodies will be measured via a CGM/fingerstick blood sample using glucose and ketones meter (Abbott Laboratories). A urine sample will be collected to evaluate ketone bodies and, in females, pregnancy status. Insulin dose will be adjusted if needed as in visit 11. All subjects will be reminded to monitor their capillary blood glucose by fingerstick at least 4 times a day (before meals and at bedtime) through-out the study. CGM will be inserted and patients asked to wear their CGM constantly for the next 20 days.

Study visit 15 (Week 51±1 week): Patients will be asked to come in the fasting state, and to bring in used study pens and tablets for drug accountability. Vitals will be taken. Daily blood sugars and urinary ketones measurements using KetoStix will be collected and

reviewed. Blood sugars and ketone bodies will be measured via a CGM/fingerstick blood sample using glucose and ketones meter (Abbott Laboratories). A urine sample will be collected to evaluate urine ketone levels. Insulin dose will be adjusted if needed as in visit 11. A 24 hr urine container will be dispensed to collect a 24 hour urine sample to bring in at the Week 52 visit. A HFHC Meal challenges will be performed as in visit 2. Patients will be asked to wear the optional 24 hour blood pressure monitor.

Study visit 16 (Week 52±2 days): Patients will be asked to come in the fasting state, and to bring in used study pens and tablets for drug accountability. Vitals will be taken. Daily blood sugars and urinary ketones measurements using KetoStix will be collected and reviewed. Blood sugars and ketone bodies will be measured via a CGM/fingerstick blood sample using glucose and ketones meter (Abbott Laboratories). Insulin dose will be adjusted if needed. The CGM will be collected. An optional fat biopsy will be performed. A urine sample will be collected to evaluate ketone bodies and, in females, pregnancy status. The 24 hour urine container will be collected. A fasting blood sample will be taken (45 mL) to evaluate study endpoints, CBC, CMP, HbA1c, C-peptide, and Fructosamine. We will ask the patients to fill out quality of life questionnaires. The patient will receive a physical. After this visit, they will be discharged from the study.
Safety phone calls will be conducted at weeks 3, 5, 8, 16, 20, 29, 35, 44 and at two weeks after the end of the study to document any side effects or other changes.

Table 1: Schedule of visits and testing procedures.

Visit #	-	1	2	3	4	5	6	7	8	9	10	11	12	13	14	15	16
Week#(±2-7 days as per text)	-8 to -4	-3	-1	0	1	2	4	13	23	25	26	27	31	39	49	51	52
Study Procedures	Screening	Run-in period		Randomization, start semaglutide							Randomize/Start dapagliflozin/ placebo						End of study
History	X																
Physical	X																X
Vitals	X	X	X	X	X	X	X	X	X	X	X	X	X	X	X	X	X
CBC	X			X				X			X		X	X			X
CMP	X			X				X			X		X	X			X
C-Peptide	X										X						X
Kidney and liver profiles	X			X				X			X		X	X			X
Pregnancy test	X	X		X			X	X	X		X		X	X	X		X
Study drug titration						X	X					X					
Dispense medications				X	X	X	X	X			X	X		X			
HbA1c	X			X				X		X				X			X
Fructosamine				X				X			X			X			X
24 hour BP (optional)			X					X		X				X		X	
CGM		X	X						X	X					X	X	
Daily Fingerstick glucose review		X	X	X	X	X	X	X	X	X	X	X	X	X	X	X	X
Daily urinary ketones review											X	X	X	X	X	X	X
Insulin dose review		X	X	X	X	X	X	X	X	X	X	X	X	X	X	X	X
Ketone bodies (blood/urine)	X	X	X	X	X	X	X	X	X	X	X	X	X	X	X	X	X
Adverse Event reporting		X	X	X	X	X	X	X	X	X	X	X	X	X	X	X	X
Meal Challenge			X							X						X	
Blood endpoints				X				X			X			X			X
Fat biopsy (optional)				X							X						X
24 hr Urine				X				X			X			X			X
Quality of life questionnaires				X							X						X

Drug Blinding:
The randomizations will be performed using an Interactive Web System, hosted by University of Glasgow. One hundred and fourteen (114) patients will be randomized as follows: participants will first be randomized to semaglutide plus usual insulin therapy or insulin therapy only (no placebo for semaglutide or other placebo injection will be provided), in a 2:1 ratio. Those who remain on semaglutide at six months will then be randomized in a 1:1 ratio to receive dapagliflozin or matched placebo. Each randomization will be stratified by study site and the second randomization will also include HbA1c stratification. The randomization schedules will be generated by a statistician with no other involvement in the trial, using the method of randomized permuted blocks. Only those staff responsible for the maintenance of the randomization system will have access to the randomization sequences during the trial. The first randomization (which will be revealed to the researcher at the point of randomization) will use random block sizes to reduce predictability. Researchers at study sites will have no knowledge of the next allocation to be issued. The second randomization (which will be double-blind) will use a different. At the point of randomization, the researcher will be given a medication pack ID number, but will not know whether that medication pack contains Dapagliflozin or placebo. For each randomization, a web-based randomization system (developed and maintained by the Robertson Centre for Biostatistics) will be used. The system will include an emergency unblinding facility for the dapagliflozin/placebo randomization
Astra Zeneca Inc. will supply dapagliflozin and matching placebo tablets in unlabeled and identical packaging; semaglutide pens will be obtained commercially and will be used in an open label fashion.

Breaking of the study blind will be performed only: (i) for SUSARs; and, (ii) where knowledge of the treatment is considered by local health personnel absolutely necessary for further management of the patient. A central unblinding facility based at the Robertson Centre for Biostatistics, University of Glasgow, will be available by telephone. Notification of all unblinding will be sent to the Chief Investigator

During the course of the trial there will be no attempt to identify group assignment for any participant (except for unblinding in the case of safety concerns) and the investigators will execute the protocol in the manner it was approved independent of the group assignment.

Drugs will be stored in a locked cabinet or locked temperature controlled refrigerator at 4C at the research facility of the Diabetes and Endocrinology Center of WNY. IND application for use of all drugs (dapagliflozin and semaglutide) in type 1 diabetes will be submitted to FDA and no study procedure will start before all local and federal approvals are obtained.

Clinical and Laboratory Procedures

1) **CMP, CBC, HbA1c, fructosamine, glucose, glucagon, GLP-1 and FFA measurements:** Blood CMP, CBC, HbA1c, fructosamine and glucose will be measured through Quest diagnostic (US site) and NHS labs (UK site). Free fatty acid levels will be measured by a colorimetric assay (Wako, Richmond, VA). GLP-1 and

glucagon will be measured from samples collected in protease and DPP-IV inhibitors by ELISA kits from Millipore.

2) **Meal Challenge test:** In order to assess the postprandial changes induced by dapagliflozin, semaglutide or the combination of both compared to insulin only, a meal challenge will be performed before starting treatments, at six months and at end of study using a 910 Calorie High fat High carbohydrate meal (HFHC) as in several of our previous studies (HFHC meal includes an egg muffin sandwich, a sausage muffin sandwich and two hash browns which contain 88g carbohydrate, 51 g fat (33% saturated) and 34 g protein). Participants will be asked to attend fasting prior to breakfast insulin bolus; basal insulin will continue during the duration of the meal, however patients will not bolus until the 3 hour mark unless blood glucose values reach above 400 mg/dL. Sequential blood samples will be obtained at 0, 15, 30, 45, 60, 90, 120, 150 and 180 min after consuming the meal. Samples at 15, 30, 45, 90 and 150 min will be 5ml while those at 0, 60, 120 and 180min will be 30 ml (total volume=145 ml). Spot urine will be collected at baseline, 1 and 3hr. Blood will be collected from an indwelling intravenous canula in a superficial forearm vein.

3) **Optional 24 hour Ambulatory BP monitoring:** Participants will be instructed on the use of 24 hour ambulatory BP monitoring device. This device will measure BP and pulse every 30 minutes for 24 hours. In addition to the scheduled measurements there will be an option for additional manual measurements if systolic blood pressure is >160 or <90 mmHg.

4) **Optional Fat aspiration procedure:** Abdominal subcutaneous fat tissue aspiration will be performed at a 10 cm distance from umbilicus. Participants will be asked not to take aspirin or NSAIDS in the previous 72 hours - if they have, the procedure will not be done. Skin will be prepared with povidone-iodine (Betadine) and alcohol. A sterile drape will be placed around the appropriate area. 3cc of 1% lidocaine will then be administered subcutaneously (the dose will not exceed 4.5mg/kg body weight). After adequate anesthesia has been achieved, aspiration of fat tissue will then be with a 3-holed canula (Tulip Instrumentation, length: 15cm, diameter: 2.1mm) fixed to a 10mL syringe. After getting adequate fat tissue (500mg-3g), the puncture site will be pressed for at least ten minutes before the patient rises up from supine position (to minimize bruising). Participants will then be discharged home. Adipose tissue will be centrifuged to remove blood and fluid contaminants. Upper adipose tissue will be collected into a separate sterile tube, washed twice with cold sterile Phosphate Buffered Saline (PBS) and centrifuged to remove the PBS. The adipose tissue sample will be weighed and approximately 500 mg transferred to a separate tube for analysis. Total RNA, nuclear extracts and total cell lysates will be prepared.

5) **Questionnaires:** Participants will be asked to complete the standard Diabetes Specific Quality of Life Scale (DSQOLS) and Problem Areas In Diabetes survey (PAID). Questionnaires attached at the end of this plan.

6) **MNC isolation (for future ancillary studies):** Blood samples will be collected in Na-EDTA as an anticoagulant. Three and a half mL of anticoagulated blood sample are carefully layered over 3.5 mL of PMN medium (Cedarlane Laboratories, Hornby, ON) and then centrifuged. At the end of this process, two bands separate out at the top of the RBC pellet. The top band consists of MNC, while the bottom consists of PMN. The

MNC band is harvested and washed twice with Hank's balanced salt solution (HBSS). This method provides yields greater than 95% pure PMN and MNC suspensions.

7) **NFκB DNA binding activity (for future ancillary studies):** Nuclear NFκB DNA binding activity is measured by EMSA. Nuclear extract is prepared from MNC and adipose tissue by high salt extraction as described by Andrews et al (19). The specificity of the bands is confirmed by supershifting these bands with specific antibodies against Rel-A (p65) and p50 (Santa Cruz Biotechnology, CA) and by competition with cold oligonucleotides. DNA binding activity of NFκB will be adjusted to Oct1 DNA binding activity to correct for any variable.
8) **Plasma concentrations of inflammatory mediators (for future ancillary studies):** Concentrations of TNFα, IL-1β, IL-18, adiponectin, IL-1RA will be measured in plasma using ELISA kits (R&D Systems, MN). CRP and SAA concentrations will be measured using an ELISA kit from Alpha Diagnostics International Inc. (San Antonio, TX).
9) **Plasma vasodilators and vasoconstrictors (for future ancillary studies):** Plasma ANP, NT-proBNP, b-NP, cGMP, cAMP, angiotensinogen, renin and angiotensin II concentrations will be measured by commercially available ELISA kits.
10) **15-isoprostane F_{2t}** (also known as 8-epi-PGF2α or 8-iso-PGF2α) **(for future ancillary studies):** will be measured in urine samples using an ELISA kit from Oxford Biomedical Research (Oxford, MI).
11) **Quantification of mRNA in MNC and adipose tissue (for future ancillary studies).** Total RNA is isolated from MNC and adipose tissue using commercially available RNAqueous®-4PCR Kit (Ambion, Austin, TX). The quality and quantity of the isolated RNA is determined before using the RNA. Real Time RT-PCR is performed using Cepheid Smart Cycler (Sunnyvale, CA) in which 2 µL cDNA, 10 µL Sybergreen Master mix (Qiagen, CA) along with 0.5 µL of 20 µM gene specific primers for IL-1β, IL-RA, TLR-4, TNFα, IL-12, SOCS-3 and JNK-1 (Life Technologies, MD) are used. The specificity and the size of the PCR products are tested by adding a melt curve at the end of the amplifications and by running it on a 2% agarose gel. All values are normalized to 18S expression and β-actin.

10.2 Describe what data will be collected.

NOTE: For studies with multiple data collection points or long-term follow up, consider the addition of a schedule or table in your response.

Response:

Data will be collected as described in table 1 above. The following aspects of the study will be performed and data collected and reported by patients:

1- A CGM device will be provided for use for 3 20-days periods. Detailed instructions on changing the sensor at 10 days will be provided. Patients will be instructed to wear their CGM constantly during the assigned study periods. They will be asked to keep a diary of their food intake to measure their carbohydrate intake during the CGM period. Insulin doses will be titrated aiming for blood glucose between 90-180 mg/dl while avoiding hypoglycemia (<70 mg/dl) or hyperglycemia (>250 mg/dl).

2- Participants who agree to participate in the 24-hr ambulatory blood pressure monitoring will be provided with a 24-hour ambulatory BP monitor to be worn for 24 hours during week -1, 13, 25, 39 and 51.
3- All participants will be asked to monitor capillary blood glucose by finger stick before each meal and at bedtime.
4- For the entire duration of the study, the patients will maintain a diary to record any hypoglycemia and other untoward side effects e.g. nausea, change in appetite.
5- Starting at the 6 months mark, participants will be asked to perform a daily urinary test of ketone bodies using KetoStix and record all outcomes. If results are moderate to large, they will be instructed to repeat the test and to call the research center or their doctor directly if results are confirmed. Patients with dual glucose and ketones meters will be instructed to record and report their blood ketones as well. If blood ketones are above 0.6mM, they should repeat the test and contact the research center of their doctor if levels are confirmed.

10.3 List any instruments or measurement tools used to collect data (e.g. questionnaire, interview guide, validated instrument, data collection form).

Include copies of these documents with your submission.

Response: Source documents will be used to collect patient information. The CGM or insulin pump will be used to collect glucose and insulin data. Glucose and ketone meter will be used to measure glucose and ketone in blood. KetoStix for measuring ketones in urine. Blood pressure monitor will be used for 24 hour blood pressure reading. Glucose, food and urinary ketones diaries will be provided for patients to record their results.

10.4 Describe any source records that will be used to collect data about subjects (e.g. school records, electronic medical records).

Response: electronic medical records and research files.

*10.5 Indicate whether or not **individual** subject results, such as results of investigational diagnostic tests, genetic tests, or incidental findings will be shared with subjects or others (e.g., the subject's primary care physician) and if so, describe how these will be shared.*

Response: Individual participant lab results will be disclosed to the participant upon their request. If the participant requests documentation be shared with another physician, physician office or hospital the participant must come to the research center to collect said documentation or the documentation can be mailed to their given home address.

Patients will be assigned a unique study number. All identifiable personal information will be stored locally in locked filing cabinets. Data entered on the

electronic Case Report Form will only be identified by the unique study number. Thus, no personal information will be shared with the data center (Robertson Centre for Biostatistics, University of Glasgow, UK).

10.6 Indicate whether or not ***study*** *results will be shared with subjects or others, and if so, describe how these will be shared.*

Response: Not Applicable. Study results will not be shared with the subjects. However, unidentifiable study results could be published in the form of a manuscript or abstract and will be reported to JDRF, Astra Zeneca, and to clinicaltrials.gov and other regulatory agencies including FDA.

11.0 Study Timelines

11.1 Describe the anticipated duration needed to enroll all study subjects.

Response: Accrual is expected to be at the rate of 4 subjects/month/site, thus we should complete enrollment of 57 patients/site in approximately 13-15 months. We expect to complete all study clinical activities in 30 months.

11.2 Describe the duration of an individual subject's participation in the study. Include length of study visits, and overall study follow-up time.

Response: 55 Weeks. Study visits will be 60-90 minutes long except for visits 2, 9 and 15 which will be about 6hr long each

11.3 Describe the estimated duration for the investigators to complete this study (i.e. all data is collected and all analyses have been completed).

Response: 36 Months

12.0 Setting

12.1 Describe all facilities/sites where you will be conducting research procedures. Include a description of the security and privacy of the facilities (e.g. locked facility, limited access, privacy barriers). Facility, department, and type of room are relevant. Do not abbreviate facility names.

NOTE: Examples of acceptable response may be: "A classroom setting in the Department of Psychology equipped with a computer with relevant survey administration software," "The angiogram suite at Buffalo General Medical Center, a fully accredited tertiary care institution within New York State with badge access," or, "Community Center meeting hall."

Response:

1) Research will be conducted at the Diabetes Endocrinology Research Center of WNY, located at *1000 Youngs Road, Suite 105, Williamsville NY 14221* and at the CTRC located in 875 Ellicott St. Buffalo NY14203.The Diabetes Research Center has facilities and exam rooms available for insulin pump download, CGM device download, meal and infusion studies and presence of study coordinator and registered nurse for data collection and blood work at all times. One of the investigators will be available at all times to address patients' related issues. CTRC location is a fully equipped laboratory with Equipment include ultra-low freezers for sample storage, centrifuges, microscopes for sample preparation, infusion pumps, ELISA, PCR and immunoblotting instrumentation.

2) Research will also be conducted at the NHS Greater Glasgow and Clyde Clinical Research Facility (CRF, Glasgow Royal Infirmary, UK and at the Diabetes Centre (Clinic D), Stobhill Ambulatory Care Hospital, Glasgow, UK. There are facilities and exam rooms available for insulin pump download, CGM device download, meal and infusion studies and presence of research nursing staff for data collection and blood work at all times. One of the investigators will be available at all times to address patients' related issues. The CRF has equipment for sample preparation, infusion pumps, ultra-low freezers for sample storage and centrifuges. ELISA, PCR and immunoblotting facilities are available in adjacent NHS and University facilities.

12.2 For research conducted outside of UB and its affiliates, describe:

- *Site-specific regulations or customs affecting the research*
- *Local scientific and ethical review structure*

NOTE: This question is referring to UB affiliated research taking place outside UB, i.e. research conducted in the community, school-based research, international research, etc. It is not referring to multi-site research. UB affiliated institutions include Kaleida Health, ECMC, and Roswell Park Cancer Institute.

Response:

☒ **N/A:** This study is not conducted outside of UB or its affiliates.

13.0 Community-Based Participatory Research

13.1 Describe involvement of the community in the design and conduct of the research.

NOTE: Community-Based Participatory Research (CBPR) is a collaborative approach to research that equitably involves all partners in the research process and recognizes the unique strengths that each brings. CBPR begins with a research topic of importance to the community, has the aim of combining knowledge with action and achieving social change to improve health outcomes and eliminate health disparities.

Response:

☒ **N/A:** This study does not utilize CBPR.

13.2 Describe the composition and involvement of a community advisory board.

Response:

☒ **N/A:** This study does not have a community advisory board.

14.0 Resources and Qualifications

14.1 Describe the qualifications (e.g., education, training, experience, expertise, or certifications) of the Principal Investigator ***and*** *staff to perform the research. When applicable describe their knowledge of the local study sites, culture, and society. Provide enough information to convince the IRB that you have qualified staff for the proposed research.*

NOTE: If you specify a person by name, a change to that person will require prior approval by the IRB. If you specify a person by role (e.g., coordinator, research assistant, co-investigator, or pharmacist), a change to that person will not usually require prior approval by the IRB, provided that the person meets the qualifications described to fulfill their roles.

Response: All study personnel are educated, trained, and licensed as required for their delegated role in this study. All study personnel have also received the required university training and will be trained by the PI before the study starts. The PIs, CO-investigators and research team have conducted similar studies in this patient population using similar agents and have the expertise to execute the study protocol and manage any adverse events.

Describe other resources available to conduct the research.

14.2 Describe the time and effort that the Principal Investigator and research staff will devote to conducting and completing the research.

NOTE: Examples include the percentage of Full Time Equivalents (FTE), hours per week. The question will elicit whether there are appropriate resources to conduct the research.

Response: The principal investigator supervises the research project and weekly research meetings are conducted to discuss the recruitment rate, resolve and discuss issues related to the conduct, safety, analysis of the study and related publications. PI is expected to spend 15% of his academic time on this research. The co-investigators, study coordinator and research staff provide coverage to the research related activity for 365 days a year.

14.3 Describe the availability of medical or psychological resources that subjects might need as a result of anticipated consequences of the human research, if applicable.

NOTE: One example includes: on-call availability of a counselor or psychologist for a study that screens subjects for depression.

Response: Available medical literature will be provided as deemed appropriate or requested by patient through UB libraries, PubMed, Google scholar as all the investigators have access to medical literature through listed resources above

The patient will also have access to physician (Investigators and Co-Investigators) who will be available to address any adverse effects or other questions during the course of the study who will be available to address any adverse effects or other questions during the course of the study

14.4 Describe your process to ensure that all persons assisting with the research are adequately informed about the protocol, the research procedures, and their duties and functions.

Response: Before beginning of enrollment all research staff will be asked to review the protocol and a training session will be conducted to provide protocol details, explain technical points and answer staff questions. All training session will have a sign in sheet to document such training. Continuous education through training meetings, conferences and discussions will continue through the study period and when revision are made to protocol.

15.0 Other Approvals

15.1 Describe any approvals that will be obtained prior to commencing the research (e.g., school, external site, funding agency, laboratory, radiation safety, or biosafety).

Response: FDA IND filling is required.

☐ **N/A:** This study does not require any other approvals.

16.0 Provisions to Protect the Privacy Interests of Subjects

16.1 Describe how you will protect subjects' privacy interests during the course of this research.

NOTE: Privacy refers to an individual's right to control access to him or herself. Privacy applies to the person. Confidentiality refers to how data collected about individuals for the research will be protected by the researcher from release. Confidentiality applies to the data.

Examples of appropriate responses include: "participant only meets with a study coordinator in a classroom setting where no one can overhear", or "the participant is reminded that they are free to refuse to answer any questions that they do not feel comfortable answering."

Response: Our clinical providers involved in the study will identify potential patients for recruitment from the Diabetes-Endocrinology Center of WNY Clinics according to the inclusions and exclusion criteria and through advertisements. Patient who qualify will be asked in private during their one on one consultation time with the physician if they wish to participate in the research study. If the patient agrees, the research coordinator will contact them privately for a possible telephone screening and ask if time is appropriate for this discussion. The patients who call for potential participation in the study due to advertisement flyers will be screened over the phone with the research coordinator, using our telephone screening form.

When the patient is being seen at our clinics for the first time they sign the "Consent to use and disclosure of protected health information" form which clearly states that their protected health information (PHI) can be used for review in preparation for possible research. Additionally, a partial HIPAA waiver will be obtained to access medical records when screening for patients.

If the patient passes the telephone screening, they will be asked to make an appointment to review and sign the consent. Patient will do this in a private room in the research unit and will be allowed to discuss the consent in detail with the research coordinator and or study doctor. Patient will be notified that it is completely voluntary to participate in the research study and can withdraw at any time.

We will not be accessing any medical information of the patients for whom the services are not provided by our clinic providers before partial HIPAA is obtained or IC is signed.

16.2 Indicate how the research team is permitted to access any sources of information about the subjects.

*NOTE: Examples of appropriate responses include: school permission for review of records, consent of the subject, HIPAA waiver. This question **does apply** to records reviews.*

Response: Consent of the subject and partial HIPAA waiver.

17.0 Data Management and Analysis

17.1 Describe the data analysis plan, including any statistical procedures. This section applies to both quantitative and qualitative analysis.

Response:

Data (e.g. date of diagnosis, past medical history, drug treatment) extracted from local clinical records will be entered on to the eCRF under the study-specific coded identifier according to structured questions as stipulated in the Study Protocol. Biological samples (blood, urine and adipose tissue aspirates) will be collected from the study participants at the study visits according to the Schedule of Assessments in the Study Protocol.

Measures relating to safety and routine care of the participants (e.g., biochemical measures of glycemic and lipidemic control, kidney function/ fluid balance, hepatic function, hematological variables) will be analysed at local clinical laboratories in order that they are available to study nurses and physicians. The confidentiality of these data will be secure, meeting or exceeding the regulations of the relevant Ethical Review Committees/ Institutional Review Boards. Samples for measurement of key study outcomes will also be measured at the central laboratory at the University of Buffalo. Blood, urine and other samples will be aliquoted into cryovials at site for freezing and storage. Electronic stored material (e.g., patient records, data from blood and urine samples, and data from Continuous Glucose Monitoring) will only be stored using the study specific coded identifier. Data will not be identifiable at the participant level without additional access to the code. The code will be available only to the investigators and the personnel performing the study. Access to any data held electronically will require study specific log-in codes. These are managed and logged by the Robertson Centre for Biostatistics, University of Glasgow, UK

Statistical plan. The primary objective of the proposed research is to evaluate the effect of addition of dapagliflozin to semaglutide on a background of insulin treatment in type 1 diabetes patients on HbA1c at six months following start of dapagliflozin. The similarities between the study groups, baseline values for subject's demographics and study endpoints will be compared using appropriate parametric tests. Transformations of the data in order to meet statistical assumptions may be considered. The primary endpoint of the study is to detect a difference in mean HbA1c change after six months of dapagliflozin or placebo addition to semaglutide and insulin treatment. Statistical analysis will be carried out using t-tests to detect difference between groups or the Wilcoxon test for paired data. Tests will be two-sided and tested at the 0.05 level. The results will be summarized as mean±SE. All randomized patients will be included in the analysis of the primary end point based on an Intent-to-treat model.

The secondary endpoints will be to compare changes in HbA1c between each of the triple therapy and dual therapy groups compared to standard of care group, mean weekly glucose concentrations, percent time spent in different glycemic thresholds, variability as measured by standard deviation, insulin dose, body weight, carbohydrate intake, blood pressure, and 24-hour blood pressure and QOL changes among the groups. A reduction in the glucose and glucagon AUC following the meal will constitute another secondary end point in this setting, ANCOVA analysis will be used. Since the secondary end-points are pre-specified and may as well be correlated, multiplicity control for multiple tests across secondary endpoints may not be necessary. However, Holm-Bonferroni correction

for multiplicity testing will be implemented if additional endpoints are tested or if the pre-specified secondary end-points are found to be not correlated.

17.2 *If applicable, provide a power analysis.*

NOTE: This may not apply to certain types of studies, including chart/records reviews, survey studies, or observational studies. This question is asked to elicit whether the investigator has an adequate sample size to achieve the study objectives and justify a conclusion.

Response:

Sample size determination is based in part on our current data showing that in type 1 diabetes, treatment with a GLP-1 agonist, liraglutide (1.2 and 1.8mg doses) for 3 months leads to HbA1c suppression by an average of 0.6% (SD= 0.5%). Dapagliflozin was recently shown to reduce HbA1c by 0.45% in type 1 diabetes patients (13). Additionally, empagliflozin treatment for 8 weeks was shown to reduce HbA1c by 0.4% in type 1 diabetics (20, 21) and our current data showing additional improvement in HbA1c by an additional 0.66% at 12 weeks when dapagliflozin was added to liraglutide and insulin therapy. Conservatively estimating that the combination therapy will suppress HbA1c by 0.5% (SD =<0.7%) compared to semaglutide alone (t-test), a sample size of 38 (32 evaluable patients) in each group will be sufficient to provide adequate power ($\beta = 0.2$) to detect a statistically significant difference ($\alpha = 0.05$) taking in consideration a 15% dropout rate.

17.3 *Describe any procedures that will be used for quality control of collected data.*

Response:

An electronic case report form (e-CRF) will be used to collect study data at each site. The e-CRF will be developed by the study Data Centre at the Robertson Centre for Biostatistics, University of Glasgow. Access to the e-CRF will be restricted, with only authorised site-specific personnel able to make entries or amendments to their patients' data. It is the investigator's responsibility to ensure completion and to review and approve all data captured in the e-CRF.

Data will be validated at the point of entry into the e-CRF and at regular intervals during the study. Data discrepancies will be flagged to the study site coordinator and any data changes will be recorded in order to maintain a complete audit trail (reason for change, date change made, who made change).

The Robertson Centre for Biostatistics at the University of Glasgow will be responsible for collating and cleaning the data for the study. The Robertson Centre will also be responsible for data back-up and security. This center will also manage the electronic reporting of SUSARS on behalf of the sponsor.

To enable evaluations and/or audits from regulatory authorities, the investigators agree to keep records, including the identity of all participating subjects (sufficient information to link records, all original signed informed consent forms serious adverse event forms, source documents, and detailed records of treatment disposition. The records should be retained by the study country coordinators and investigator according to ICH GCP, local regulations, or as specified in the Clinical Study Agreement, whichever is longer.

Data from the study will be stored by the Chief Investigator for a minimum of 10 years. A final report of the study will be provided to the FDA and the MHRA as per requirements.

- No interim analysis is planned for efficacy so termination of study will be related to safety. Significant increases in hypoglycemic or DKA events as reviewed by DSMB might trigger termination or modification of study.
- Last observation carry forward will be used to fill missing data for the primary end point and some secondary end points. Missing data will not be filled for other endpoints.
- Any deviation(s) from the original statistical plan will be described and justified in the protocol and/or in the final report.
- All subjects entered into treatment will analyzed for primary end point on ITT bases. For secondary endpoints, analysis of completed and evaluable patients will performed per-protocol.

18.0 Confidentiality

A. Confidentiality of Study Data

Describe the local procedures for maintenance of confidentiality of ***study data and any records that will be reviewed for data collection****.*

18.1 A. Where and how will all data and records be stored? Include information about: password protection, encryption, physical controls, authorization of access, and separation of identifiers and data, as applicable. Include physical (e.g. paper) ***and*** *electronic files.*

Response: All data records will be stored on protected computers with unique passwords issued for each of the study team members and or in locked cabinets within the research center. These files will only be accessible by authorized study personnel.

18.2 A. How long will the data be stored?

Response: De-identified data storage has no expiration date and will be stored for a minimum of 7 years. The researchers may continue to rely on this for future use in research study.

18.3 A. Who will have access to the data?

Response: Those physicians, nurses, and laboratory staff that are on all documentation for the study will have access to the data.

18.4 A. Who is responsible for receipt or transmission of the data?

Response: Those physicians, nurses, and laboratory staff that are on all documentation for the study will have access to the data and can handle transfer of data. Study data will be transferred electronically (through eCRFs) to Robertson Centre for Biostatistics, University of Glasgow, UK

18.5 A. How will the data be transported?

Response: All data are stored at locally and will be transferred to Robertson Centre for Biostatistics, University of Glasgow, UK for data management and analysis. The transfer will be done through electronic CRFs processed on secure network.

Data will also be transported unless when is being archived. At that point files will be transferred to Iron Mountain for storage and archiving.

B. Confidentiality of Study Specimens

*Describe the local procedures for maintenance of confidentiality of **study specimens**.*

☐ **N/A:** No specimens will be collected or analyzed in this research. *(Skip to Section 19.0)*

18.6 B. Where and how will all specimens be stored? Include information about: physical controls, authorization of access, and labeling of specimens, as applicable.

Response: The specimens will be stored in the laboratory located at *1000 Youngs Road, Suite 105, Williamsville NY 14221*and at the CTRC located in 875 Ellicott St. Buffalo NY14203. Samples will be stored in a locked -80º C freezer. All specimens collected at the Glasgow site will be stored in NHS -80º C freezers within the eCRF until shipping to Buffalo (World Courier).

18.7 B. How long will the specimens be stored?

Response: Specimens storage has no expiration date and will be stored for a minimum of 7 years. The researchers may continue to rely on this for future use in research study

18.8 B. Who will have access to the specimens?

Response: Those physicians, nurses, and laboratory staff that are on all documentation for the study will have access to the specimens

18.9 B. Who is responsible for receipt or transmission of the specimens?

Response: Those physicians, nurses, and laboratory staff that are on all documentation for the study will have access to the specimens and can handle transfer of samples. Sample/specimens will transported from University of Glasgow site to the university of Buffalo CTRC site (875 Ellicott St, Buffalo, NY 14203) for storage in -80C until analyzed.

18.10 B. How will the specimens be transported?

Response: All study samples will be centrally stored in UB CTRC site. Therefore, samples collected at the UK site will be transported for analysis and final long-term storage. Transport will be done so using dry ice in a properly labeled Styrofoam container by the laboratory technician. Agreement regarding shipping and receiving the specimens form UK site will be part of the subcontract agreement.

19.0 Provisions to Monitor the Data to Ensure the Safety of Subjects

☐ **N/A:** This study is not enrolling subjects, or is limited to records review procedures only. This section does not apply.

NOTE: Minimal risk studies may be required to monitor subject safety if the research procedures include procedures that present unique risks to subjects that require monitoring. Some examples include: exercising to exertion, or instruments that elicit suicidality or substance abuse behavior. In such cases, N/A is not an acceptable response.

19.1 Describe the plan to periodically evaluate the data collected regarding both harms and benefits to determine whether subjects remain safe.

Response:

An independent Data safety and monitoring board (DSMB) will be established for this trial. The board will consist of independent faculty members from UB and other institutions with sufficient knowledge of the field of type 1 diabetes but not

involved in the study. Data Management team at Robertson Centre will periodically provide the data on which to base regular reports to the Data and Safety Monitoring Board. The actual reports will then be generated by the unblinded statistician. The DSMB looks only at safety endpoints and not at futility. The DSMB will convene every six months to review safety end points, side-effects experienced by study subjects and any adverse events reported. The site PIs, CO-Is and study coordinator(s) will also continuously monitor the data and safety of subjects enrolled in the study. The site PIs and/or the DSMB may recommend to terminate the study or specific aspects of the study if clear harm is observed with one treatment option compared to standard of care or placebo. Specific safety endpoints that will be reviewed include increases in hypoglycemic and ketosis/DKA events.

19.2 Describe what data are reviewed, including safety data, untoward events, and efficacy data.

Response:

Assessment of Efficacy

1- glycemic indices including HbA1c, fructosamine, post-prandial glucose excursions and glucagon. CGM data will be collected as efficacy parameter of glucose control. Total insulin dose will be also documented and analyzed.
2- body weight
3- blood pressure and use of blood pressure medications
4- QOL questionnaires.

The timing of recording of the above efficacy parameters is described in table 1.

Assessment of Safety

The following safety assessments assays and measurement will be reviewed:

1- Vitals, CBC, CMP and markers kidney and liver function will be collected at screening, during and at end of the study as in table 1.
2- Assessment of hypoglycemia: Glucose data will be reviewed from CGM and daily finger stick and during visits.
3- Assessment of ketosis: Ketones levels will be reviewed from daily urinary ketone diaries or blood ketone meters and from urine and blood at the time of each visit
4- Reports of side effects by patients.
5- Insulin dose titrations and pump downloads at each visit

The methods and timing for assessing, recording, and analyzing safety parameters.

The DSMB will convene every six months to assess the side-effects experienced by study subjects and any adverse events reported. The PIs, CO-Is and study coordinator(s) will also continuously monitor the safety of subjects enrolled in the study during their visits and during the safety phone calls.

Procedures for eliciting reports of and for recording and reporting adverse event and inter-current illnesses.

All adverse events will be reported at the time of the visit or by telephone when it occurs. Medical events that occur between the signing of the Informed Consent and the first intake of study drug will be documented in the medical history. Participants will be asked to report any adverse events in general, non-directed questioning. For each AE volunteered by the subject, the investigator will obtain all the information required to complete the adverse event page of the eCRF, in accordance with the guidelines that accompany it. All adverse events, regardless of seriousness, severity, or presumed relationship to study therapy, will be recorded using medical terminology in the source document. Whenever possible, diagnoses will be given when signs and symptoms are due to a common etiology (e.g., cough, runny nose, sneezing, sore throat, and head congestion should be reported as "upper respiratory infection). Investigators will record on the CRF their opinion concerning the relationship of the adverse event to study therapy. The principal investigators assumes responsibility for appropriate reporting of AEs to the regulatory authorities. The investigator will report these events to the appropriate Independent Ethics Committee/Institutional Review Board (IEC/IRB) in accordance with local regulations. All measures required for AE management will be recorded in the source document and reported according to the State University of New York at Buffalo instructions. Non-serious events/problems that impacted on the rights and welfare of the subjects will be reported as they occur or at the time of knowledge or notification.

Definition of adverse events

The International Conference on Harmonization (ICH) Guideline for Good Clinical Practice (GCP) E6(R1) defines an AE as:

Any untoward medical occurrence in a patient or clinical investigation patient administered a pharmaceutical product and which does not necessarily have a causal relationship with this treatment. An AE can therefore be any unfavorable and unintended sign (including an abnormal laboratory finding), symptom, or disease temporally associated with the use of a medicinal product, whether or not considered related to the medicinal product.

The term AE is used to include both serious and non-serious AEs.

Definition of serious adverse events

A serious adverse event is an AE occurring during any study phase (i.e., screening, run-in, treatment, wash-out, follow-up), at any dose of the study drugs that fulfils one or more of the following criteria:

- Results in death
- Is immediately life-threatening
- Requires in-patient hospitalization or prolongation of existing hospitalization
- Results in persistent or significant disability or incapacity
- Is a congenital abnormality or birth defect in offspring of the patient
- Is an important medical event that may jeopardize the patient or may require medical intervention to prevent one of the outcomes listed above.

- TO THE AUTHOR: The causality of SAEs (their relationship to all study treatment/procedures) will be assessed by the investigator(s) and communicated to AstraZeneca. This is requested to be on the AE cover sheet.

The following variables are recommended to be collected for each AE:

- AE (verbatim)
- The date when the AE started and stopped
- Whether the AE is serious or not
- Action taken with regard to IPs
- Administration of treatment for the AE
- Outcome

In addition, the following variables are recommended to be collected for SAEs:

- Date the AE met criteria for SAE
- Date the Investigator became aware of the SAE
- Seriousness criteria fulfilled
- Date of hospitalization
- Date of discharge
- Probable cause of death
- Date of death
- Whether an autopsy was performed
- Causality assessment in relation to study procedure(s)
- Causality assessment in relation to other medication, as explained in Section 1.4.2.
- Description of the SAE

Reporting of serious adverse events

All SAEs will be reported, whether or not considered causally related to the investigational product, or to the study procedure(s). The reporting period for SAEs is the period immediately following the time that written informed consent is obtained throughout the treatment period and including the designated follow-up period as per the study protocol. The investigator is responsible for informing the Ethics Committee and/or the Regulatory Authority of the SAE as per local requirements.

Note that all serious or unexpected adverse events must be reported to AstraZeneca regardless of the country where the study is conducted.

The investigator must inform the FDA, via a MedWatch form, of any serious or unexpected adverse events that occur in accordance with the reporting obligations of 21 CFR 312.32 , and will concurrently forward all such reports to AstraZeneca. A copy of the MedWatch report must be emailed to AstraZeneca (TCS vendor) at the time the event is reported to the FDA. It is the responsibility of the investigator to compile all necessary information and ensure that the FDA receives a report according to the FDA reporting requirement timelines and to ensure that these reports are also submitted to AstraZeneca at the same time.

A cover page should accompany the MedWatch form indicating the following:

- “Notification from an Investigator Sponsored Study”

- The investigator IND number assigned by the FDA
- The investigator's name and address
- The trial name/title and AstraZeneca ISS reference number (ESR-##-#####)

Investigator must also indicate, either in the SAE report or the cover page, the causality of events in relation to all study medications and if the SAE is related to disease progression.

Send SAE report and accompanying cover page by way of email to AstraZeneca's designated mailbox: AEMailboxClinicalTrialTCS@astrazeneca.com

If a non-serious AE becomes serious, this and other relevant follow-up information must also be provided to AstraZeneca and the FDA.

Serious adverse events that do not require expedited reporting to the FDA still need to be reported to AstraZeneca preferably using the MedDRA coding language for serious adverse events.

The investigators will either provide a copy of the randomization code/ code break information or unblind those SAEs which require expedited reporting

Overdose

An overdose is defined as a patient receiving a dose of IP in excess of that specified in the Investigator's Brochure, unless otherwise specified in this protocol.

Any overdose of a study patient with the investigational product, with or without associated AEs/SAEs, is required to be reported within 24 hours of knowledge of the event to AstraZeneca Patient Safety using the designated Safety e-mailbox (see Section 1.5 or contact information). If the overdose results in an AE, the AE must also be recorded as an AE. Overdose does not automatically make an AE serious, but if the consequences of the overdose are serious, for example death or hospitalization, the event is serious and must be recorded and reported as an SAE (see Section 1.3). Any specific details around overdose for combination or comparator agents will also be provided as applicable. The investigator will use clinical judgment to treat any overdose.

Hepatic function abnormality

Hepatic function abnormality that fulfills the biochemical criteria of a potential Hy's Law case in a study patient, with or without associated clinical manifestations, is recommended to be reported as "hepatic function abnormal" within 24 hours of knowledge of the event to AstraZeneca Patient Safety using the designated Safety e-mailbox (see Section 1.3), unless a definitive underlying diagnosis for the abnormality (e.g., cholelithiasis or bile duct obstruction) that is unrelated to investigational product has been confirmed. The criteria for a potential Hy's Law case is Aspartate Aminotransferase (AST) or Alanine Aminotransferase (ALT) ≥3x Upper Limit of Normal (ULN) together with Total Bilirubin (TBL) ≥2xULN at any point during the study following the start of study medication irrespective of an increase in Alkaline Phosphatase (ALP).

Pregnancy

If a patient becomes pregnant during the course of the study, the IPs should be discontinued immediately.

Pregnancy itself is not regarded as an AE unless there is a suspicion that the IP under study may have interfered with the effectiveness of a contraceptive medication. Congenital abnormalities or birth defects and spontaneous miscarriages should be reported and handled as SAEs. Elective abortions without complications should not be handled as AEs. The outcome of all pregnancies (spontaneous miscarriage, elective termination, ectopic pregnancy, normal birth, or congenital abnormality) should be followed up and documented even if the patient was discontinued from the study.

If any pregnancy occurs in the course of the study, then the investigator should inform the appropriate AstraZeneca representatives within 1 day, i.e., immediately, but no later than 24 hours of when he or she becomes aware of it.

The investigator will ensure that all relevant information is provided to the AstraZeneca Patient Safety data entry site within 1 to 5 calendar days for SAEs and within 30 days for all other pregnancies.

The same timelines apply when outcome information is available.

19.3 Describe any safety endpoints.

Response: These specific Safety endpoints will be collected:

1- Frequency and severity of hypoglycemia: Hypoglycemia will be assessed throughout the study via multiple daily finger stick glucose measurements, CGM data collected 3 times (about 3 weeks each) during the study and from patient reports during visits and phone calls. Hypoglycemic events will be characterized as in the experts consensus published in *Diabetes Care (2017 Dec; 40(12): 1622-1630):*

- Level 1: Glucose < 70 mg/dL (3.9 mmol/L) and Glucose ≥ 54 mg/dL (3.0 mmol/L)
- Level 2: Glucose < 54 mg/dL (3.0 mmol/L)
- Level 3: A severe event characterized by altered mental and/or physical status requiring assistance.

2- Ketones levels: Urinary ketones measured daily at home and logged in the ketones logbook. Blood and urinary ketones measured during each visit. Ketone levels will be characterized as normal, ketosis and severe ketosis:

-Normal blood ketones <0.6mM or urinary ketones of trace to small (≥+1)

-Ketosis: Blood ketones >0.6mM <2.5mM or urinary ketones of moderate to high (+2 and +3)

-Severe ketosis Blood ketones >2.5mM or urinary ketones of too high (+4)

3- Diabetes ketoacidosis episodes: any confirmed DKA episode (regardless of hospitalization) defined as:

- Elevated serum or urine ketones (greater than the upper limit of the normal range), and
- Serum bicarbonate < 15 mmol/L or Blood pH < 7.3

4- Low blood pressure: <90mmHg SBP and <50 mmHg DBP): Patients will be instructed to measure blood pressure daily and it will be will be assessed in each visit.
5- Nausea, vomiting and other GI side effects. Data will be collected by patients' safety reports, during visits and phone calls.
6- Genital mycotic infections or UTIs
7- Changes in liver and kidney function: CMP and kidney and liver profiles will be assessed as indicated in table 1.

19.4 Describe how the safety information will be collected (e.g., with case report forms, at study visits, by telephone calls with participants).

Response: The safety information will be collected at the time of the visit, and or during preplanned telephone calls with the participant or patient self-reporting (including glucose, food intake and ketones diaries). Data will be entered into eCRFs

19.5 Describe the frequency of safety data collection.

Response: Safety data collection will be done at all study visits and during preplanned phone calls as in table 1. The patient, however, will be asked to report any adverse event or safety related information via phone as soon as it occurs and it will be reviewed the same day.

19.6 Describe who will review the safety data.

Response: DSMB established for this study will review unblended safety data provided by the data management system every 6 months. Additionally, the PIs and coinvestigators will review safety data (listed above) at the completion of each visits by each subject and review any report of side effects or adverse events as they are presented by the patients. The investigators will also review collective blinded data every 3 months to assess the safety and any potential risks to the participants. Furthermore the investigators will assess other risks including the physical, psychological, social, legal and economic harm to these patients. The investigators will carefully watch for any invasion of privacy and breach of confidentiality. The principal investigator will be sharing the results of safety analysis of the study and serious adverse events with the local IRB, DSMB, JDRF, AZ and the FDA. The study groups will remain blinded. If there are a serious safety concerns or an emergency, then, and based on principal investigator discretion, study randomization center in Glasgow University or the unblended statistician will provide information about group assignment of the patient(s) involved. Un-blinded data will be reviewed by the principal investigator and co-investigators and decision to complete, withdraw or adjust dose of drug(s) will be made, documented and shared with IRB and DSMB.

19.7 Describe the frequency or periodicity of review of cumulative safety data.

Response: Safety data will be reviewed every 6 months by DSMB (un-blinded), every 3 months by PIs and CO-Is (blinded) and at every visit by study team.

19.8 Describe the statistical tests for analyzing the safety data to determine whether harm is occurring.

Response: N/A

19.9 Describe any conditions that trigger an immediate suspension of the research.

Response: No interim analysis is planned for efficacy so termination of study will be related to safety. Significant increases in severe hypoglycemic or DKA events as reviewed by DSMB are possible causes that might trigger termination of study.

20.0 Withdrawal of Subjects

☐ **N/A:** This study is not enrolling subjects. This section does not apply.

20.1 Describe ***anticipated*** *circumstances under which subjects may be withdrawn from the research without their consent.*

1. The site PI can stop investigational study treatment or remove participants from the trial without participants approval if for any reason he/she feels is appropriate, including: severe side effect, injury or medical condition which may place the patient at risk of further complications if he/she continue to participate, failure to take the medication as instructed, failure to keep scheduled appointments, cancellation of the study by the sponsor, IRB, FDA or other administrative reasons.
2. If any of the participants become pregnant during the period of study, they will discontinue study treatment.
3. Participants who experience persistent severe nausea or vomiting after starting semaglutide which is not managed by dose reduction will discontinue the investigational study treatment. Those with mild or moderate nausea will continue for at least one more week. If they continue to experience significant nausea (defined as interfering with normal activities), dose will be reduced. If they continue to have these side effects, they will discontinue study medication(s).
4. Persistent weight loss or failure to maintain weight if subject has lost >15% or BMI is < 22 kg/m2 in spite of down-titration of semaglutide and/or dapagliflozin.
5. Developing other conditions included in the exclusion criteria
6. Change in method of insulin delivery between insulin pumps to MDI or vice versa during the study unless pump treatment interruption is temporary for a period of at most 2 weeks. Also, patients who upgrade to the new "artificial pancreas" pump (Medtronic's MiniMed 670G hybrid) during the study will be withdrawn.

7. Hypoglycemia: Two episodes of severe hypoglycemia (requiring others help).
8. Ketosis (with no other identifiable precipitating factors): Persistent (1week) asymptomatic or symptomatic and unmanaged (using STICH protocol) blood ketone levels >1.5 mM (or ≥+2 in urine).
9. Persistent (>1 week) total insulin dose reduction of >20% from baseline AND total insulin dose <0.4U/Kg.
10. DKA: Two episode of DKA (with no identifiable precipitating factors) as defined by the expert consensus statement (*Diabetes Care (2017 Dec; 40(12): 1622-1630)*)
11. Two episodes of UTI.
12. Two episodes of mycotic genital infection.
13. Significant persistent change in eGFR > 15 ml/min
14. Pancreatitis: One episode of pancreatitis.
15. Other persistent side effects specific to study drugs

20.2 Describe any procedures for orderly termination.

NOTE: Examples may include return of study drug, exit interview with clinician. Include whether additional follow up is recommended for safety reasons for physical or emotional health.

Response: Participation in this study is voluntary. Participants have the right to refuse to participate or to withdraw from participation at any time for any reason. Refusal to participate or to withdraw from the study will involve no penalty or loss of entitled benefits, nor affect ongoing medical care. The site PI can stop investigational study treatment or remove participants from the trial without participants approval. If a patient withdraws consent and decide not to participate in the study, she/he will be asked to undergo a final study visit for his/her safety. If a subject withdraws from the research, the data collected to that point will be used toward the research finding. If applicable the subject will have to bring back any unused research drug and or devices.

20.3 Describe procedures that will be followed when subjects withdraw from the research, including retention of already collected data, and partial withdrawal from procedures with continued data collection, as applicable.

Response: We will use the data collected up to the point of stopping the study. No further information will be collected. Patients who stop study treatment and are interested in continuing in the study will be followed up until the end of the study for the primary end point and select secondary endpoints based on the Intent-To-Treat (ITT) analysis model. Withdrawn participants will not be replaced. The study sample size calculation includes an additional 15% subjects to compensate for drop out.

21.0 Risks to Subjects

21.1 List the reasonably foreseeable risks, discomforts, hazards, or inconveniences to the subjects related to their participation in the research. Consider physical, psychological, social, legal, and economic risks. Include a description of the probability, magnitude, duration, and reversibility of the risks.

NOTE: Breach of confidentiality is always a risk for identifiable subject data.

Response:

- Risks associated with intravenous (IV) line: All participants will be informed of the potential complications of an intravenous (IV) line, which include mild bruising at the site of IV line, usually resolving in a few days. They will also be informed about the possibility of infiltration of the IV line at the time of performing blood draws or during the meal challenge test in which case another IV line at a different site will be required (with risk of bruising at more than one site).
- Risks associated with 24 hour Ambulatory BP monitoring device: The BP cuff inflates every 15 minutes to record blood pressure over 24-hours. Participants will experience a pressure-like sensation over the arm where 24 hour BP device is worn at the time of the BP being measured due to the inflation of the cuff and release of this pressure at the time of deflation of the BP cuff. They will be informed to remove the cuff after 24 hours. If worn for longer periods they may experience mild bruising over the arm and some swelling, usually resolving 1-2 days after discontinuation.
- Risks associated with fat tissue aspiration: will probably lead to a bruise at the site of aspiration. The site may be mildly painful for 1-2 weeks. Participants are advised to call their study site staff if they have more severe pain or swelling at the site after the procedure. Rarely there are side effects such as low BP or heart rate and/or allergic reaction to local anesthetic including swelling of the throat.
- All subjects will be referred to semaglutide and dapagliflozin labeling safety information, and will be given a copy of the package insert of the medication. Discussion of the potential side effects and the different warning and precautions will take place at the time of informed consent. Patients will be informed they must not discontinue their insulin during the study.
- Potential risk of insulin, GLP-1 RA and SGLT-2 inhibitors use in type 1 diabetes patients is hypoglycemia. The combination therapy can possibly increase the magnitude and frequency of this risk.
- Potential side effects of GLP-1 RA and SGLT-2 inhibitors also include hypotension, nausea and other GI side effects, polydipsia, polyuria, dehydration, ketosis, and reduced glomerular filtration rate (GFR).
- In rodents, semaglutide causes dose-dependent and treatment-duration-dependent thyroid C-cell tumors at clinically relevant exposures. However, it received FDA-approval for the treatment of type 2 diabetes in 2017 following large Phase 3 trials including the cardiovascular safety trial SUSTAIN-6.
- On the basis of the above rodent studies, semaglutide is contraindicated in those with a personal or family history of medullary thyroid carcinoma and in patients with Multiple Endocrine Neoplasia syndrome type 2 (MEN 2). However, following review of human data with GLP-1RAs, FDA does not require or suggest routine monitoring of serum calcitonin/ thyroid ultrasound in people treated with semaglutide.
- Injection-site reactions: Serious injection-site reactions, with or without bumps (nodules), have happened in some people who use GLP-1RAs but are less frequent with semaglutide. Injection-site reactions have rarely required surgical

intervention. Participants will be instructed to report any injection site reactions including nodules to study investigators.

- Kidney injury: GLP-1 RA might cause acute kidney injury in patients without underlying renal disease especially in patient who had experienced nausea, vomiting, diarrhea or dehydration. Dapagliflozin also has warnings and precautions against acute kidney injury and impairment in renal function.
- As mentioned above, there is a risk of ketoacidosis, as shown by some studies and by our data, when SGLT2 inhibitors are used in type 1 diabetes. In our own preliminary studies on triple therapy, we observed euglycemic ketoacidosis in participants whose insulin doses had been reduced by > 20% of baseline levels or in patients who are not adequately insulinzed. Thus, this adverse effect is largely attributable to insulinopenia as well as anti-insulin effects of SGLT2 inhibitors at the hepatic level (e.g. increased gluconeogenesis).
- The following other known adverse effects of SGLT-2 inhibitors will be discussed with participants when obtaining informed consent:

1. Risk of dizziness/ symptomatic hypotension: can occur soon after initiation due to intravascular volume contraction.
2. Impaired kidney function: As SGLT-2 inhibitors increase serum creatinine and decrease eGFR, this will be monitored closely.
3. Genital mycotic infections: SGLT-2 inhibitors increase the risk of genital mycotic infections, particularly in women. Those with a prior history are more susceptible. Following topical treatment with anti-fungal medications they rarely re-occur.
4. Bladder Cancer - Across 22 clinical studies, newly diagnosed cases of bladder cancer were reported in 10/6045 patients (0.17%) treated with Dapagliflozin and 1/3512 patient (0.03%) treated with placebo. After excluding patients in whom exposure to study drug was less than one year at the time of diagnosis, there were 4 cases of bladder cancer with dapagliflozin and no cases with placebo i.e. too few cases to determine causality.
5. The dapagliflozin tablets and matching placebo contain lactose, which may cause discomfort in lactose-intolerant individuals.

- Severe allergic reaction (anaphylaxis) to study drugs or placebo ingredients or other interventions may accrue in rare cases.
- Excessive weight loss: Both investigation drugs are known to cause weight loss in the range of 5-10% in type 2 diabetes patients. However, the magnitude of weight loss following the combination of both drugs in type 1 diabetes patients may be significantly more. Excessive weight loss may lead to increased risk of hypoglycemia and ketosis or DKA (especially in the presence of dapagliflozin) and other unforeseen risks. Therefore, weight loss during any phase of the study will be limited to ≤15% baseline body weight or reaching a BMI of $\leq 22 Kg/m^2$.
- There might be other unforeseen risks associated with the use of these agents in type 1 diabetes and additional unforeseen risks associated with the combination therapy.
- There is always the risk of breach of confidentiality where patients’ identifiable data is compromised.

21.2 Describe procedures performed to lessen the probability or magnitude of risks, including procedures being performed to monitor subjects for safety.

Response:

The following are procedures to lessen the probability and magnitude of risks listed above:

1- Hypoglycemia:

- Patients will be instructed to measure glucose at least 4 times a day (before meals and at bedtime) through-out the study and record all reading.
- Insulin dose adjustments will be done at every visit to avoid hypoglycemia. However, there will be no preemptive insulin dose reduction at start of treatment since all patients will have HbA1c >7.5% and the risk of hypoglycemia will be minimal when semaglutide is added.
- Daily fingerstick glucose, CGM and insulin pump data will be reviewed by the investigators for optimal titration of insulin dose to target glucose levels between 90-180mg/dl.
- In general, insulin dose adjustments should not be >10% from current total insulin dose (or 5% if HbA1c is <7%) or >20% from baseline total insulin dose at week 0 and then at week 26.
- Patients will be trained in CARB counting to avoid inaccurate insulin dose administration.
- Patients will be trained to recognize symptoms of hypoglycemia and how to manage it at home. These symptoms include feeling shaky, being nervous or anxious, sweating, chills and clamminess, confusion, fast heartbeat, feeling lightheaded or dizzy, hunger, nausea and color draining from the skin (pallor) among others. They will be asked to call the research office or the on call clinical services.
- When patients feel symptoms of hypoglycemia they will be asked to measure blood glucose and if <70mg/dl they will be instructed to consume carbohydrate or 4 tablets of glucose (4gm each). If after 15 min hypoglycemia continues, repeat carb intake until sugar levels are above 70 mg/dl. If sugar levels are below 55mg/dl, glucagon injection is permitted as rescue medication for severe hypoglycemia.
- Patients will be instructed to inform people who they are in frequent contact with (for example, friends, family members, and coworkers) on how to give glucagon to treat severe hypoglycemia. If patient have needed glucagon previously, it should be documented during screening visit so more emphasis on ways to prevent severe hypoglycemia in the future can be discussed.
- Patients/family will be instructed not to hesitate to call 911 if someone is unconscious and glucagon is not available or someone does not know how to use it and not to inject insulin (it will lower their blood glucose even more) or provide food or fluids (they can choke). They will be asked to document all details of the incident and report to research staff as soon as possible.
- Study medications dose will be reduced in patients who continue to have more than 2 measurements of glucose levels in the hypoglycemic range (<70mg/dl, regardless of symptoms) after insulin dose is reduced by the maximum allowed in this study.

2- Ketosis and Ketoacidosis:

- Patients will be instructed to measure ketone bodies in urine samples daily starting at 3 days before week 26 with Ketostix (Bayer) and record it in diary provided. Ketones (blood and urine) will be measured at every visit. If ketone bodies in urine measure +1 or greater (>1.5mM in urine), patients are required to notify the research center. They will be monitored closely for insulin dose, carb intake, ketones and blood sugar and dapagliflozin/semaglutide can be temporarily discontinued, if necessary, for 1-2 weeks.
- Patients will be provided training to recognize the signs and symptoms of DKA and to self-test urine ketones on a PRN basis (in addition to once daily) if they develop any symptoms consistent with DKA, including nausea, vomiting, fatigue, confusion, or shortness of breath. This should be reported regardless of blood sugar levels since in type 1 diabetes on SGLT-2 inhibitors, DKA can occur even in normal glucose levels.
- Insulin dose changes should not be greater than 10% (5% if HbA1c is<7%) from current levels and not reduced by more than 20% from baseline levels at 0 weeks and then at 26 weeks.
- Patients requiring an insulin dose reduction > 20% AND their total insulin dose is <0.4U/Kg will be monitored closely for insulin dose, carb intake, ketones and blood sugar and dapagliflozin/semaglutide can be temporarily discontinued, if necessary, for 1-2 weeks. If insulin requirements remain law, patient will be withdrawn from study.
- We shall not increase dose of dapagliflozin in patients who exhibit significant ketonuria (+2 or more) with the lower dose.
- Urinary ketones (and blood ketones if patients have a dual glucose and ketone meter) will be monitored daily and reported. Dapagliflozin will be temporarily (1 week) discontinued and STICH protocol (STop SGLT-2i, give Insulin bolus, Carbohydrate, and Hydration) activated in events of DKA or if based on investigator discretion if blood ketone levels are persistently (1 week) >1.5 mM (+2 in or more in urine). Dapagliflozin may also be temporarily stopped in clinical situations known to predispose to ketoacidosis (e.g. prolonged fasting due to acute illness or surgery). Patients will be instructed to stay hydrated and not reduce their caloric intake (especially carbohydrates) or start new dietary systems (especially ketogenic diets) during the study.
- If a patient develops symptoms of DKA or ketone levels exceed the allowed limit in this study (persistent (1 week) blood ketones >1.5 mM or +2 in or more in urine), STICH protocol (STop SGLT-2i, give Insulin bolus, Carbohydrate, and Hydration) will be activated. They could be managed for up to a week. If symptoms and ketone levels persist over 1 week, patients will be withdrawn form study. Insulin infusions may be administered by ER or hospital if patients develop DKA. They will be asked to document all details of the incident and report to research staff as soon as possible.

3- Risk of acute kidney injury or renal function impairment: CBC, CMP, including kidney function test and urinary markers will be performed at screening, during and at end of the study (table 1). Additional testing will be performed in patients who suffer from sever nausea or GI symptoms or hypovolemic and during the

up titration of investigation drugs. If acute kidney injury occurs dapagliflozin will be discontinued immediately. To lessen the chance of this risk, patients will eGFR<60 ml/min/1.73m^2 will be excluded. Patients will also discontinue study medications if eGFR falls by >15 ml/min in 2 repeated measurements. Also other patients with factors that may predispose patients to acute kidney injury e.g. hypovolemia, chronic renal insufficiency, congestive heart failure will be excluded. Patients on ACEI, ARB, NSAID will be monitored closely. Dapagliflozin will be temporarily discontinued in any setting of reduced oral intake such as acute illness or fasting or fluid losses such as gastrointestinal illnesses, or excessive heat exposure.

4- Injection site reactions, mycotic infections and complications of IV and biopsy collection will also be monitored, Physical examination will be performed at screening 26, and 52 weeks visits. Safety vital assessments will be performed at every visit. Pregnancy will be tested at screening, 0, 4 13, 26, 39, and 52 weeks. Phone calls will be made for safety and compliance at least 7 time during the study. The patients, however, will be instructed to report any adverse event or safety related information via phone as soon as it occurs. All safety data will be reviewed by the PI or sub-investigators within 1-2 days of collection.

5- Epinephrine is the primary treatment for anaphylaxis with no absolute contraindication to its use. Our site and staff are trained and equipped to assist patients with such reactions. Severe conditions that are not satisfactory managed in site will be transferred to the hospital emergency department for further assessment and help.

6- Excessive weight loss: Doses of investigational drugs will be reduced in patients who experience excessive weight loss (≥15% of basal body weight or BMI ≤22Kg/m^2). Patients on semaglutide only (first 26 weeks) will be down-titrated to the lower dose. In patients on semaglutide and dapagliflozin/placebo (weeks 26-52), dose of dapagliflozin/placebo will be down-titrated to the lower dose. If weight loss continues, semaglutide dose will be down-titrated as well. Instructions to maintain a minimum of 50g daily carb intake will be also emphasized in patients with significant weight loss (>10% baseline body weight).

21.3 If applicable, indicate ***which procedures*** *may have risks to the subjects that are currently unforeseeable.*

Response:

There might be additional adverse effects of dapagliflozin not currently known or those unique to their use in Type 1 diabetes. There is no previous studies on the use of semaglutide in type 1 diabetes; therefore, additional unforeseen risk may be encountered in this study. Additionally, unforeseen risks might be associated with the combination therapy that might involve unknown drug-drug interactions.

21.4 If applicable, indicate which research procedures may have risks to an embryo or fetus should the subject be or become pregnant.

Response: Not applicable, we will not be enrolling pregnant participants or who may become pregnant or with in child baring years without signing the consent stating they will use at least two forms of birth control. If a participant becomes pregnant they will be withdrawn from the study immediately. They will be followed until term and any effects on the newborn will be documented.

21.5 If applicable, describe risks to others who are not subjects.

Response: We do not anticipate any additional risk to others not participating in this study.

22.0 Potential Benefits to Subjects

22.1 Describe the potential benefits that individual subjects may experience by taking part in the research. Include the probability, magnitude, and duration of the potential benefits. Indicate if there is no direct benefit.

*NOTE: Compensation **cannot** be stated as a benefit.*

Response: The investigational drugs used in this study are already approved as antidiabetic agents for treating type 2 diabetes. Both agents have demonstrated significant improvement in HbA1c, weight and blood pressure. It is our expectation that patients with type 1 diabetes will experience similar benefits compared to those who are treated with insulin alone. The magnitude and duration of the benefits and the safety profile may however differ and are the focus of this investigation.

23.0 Compensation for Research-Related Injury

☐ **N/A:** The research procedures for this study do not present risk of research related injury (e.g. survey studies, records review studies). This section does not apply.

23.1 ***If the research procedures carry a risk of research related injury,*** *describe the available compensation to subjects in the event that such injury should occur.*

Response: Routinely, Buffalo General Hospital, Erie County Medical Center, and/or the University at Buffalo, State University of New York, its agents, or its employees do not compensate for or provide free medical care for human subjects/participants in the event that any injury results from participation in a human research project. In the unlikely event that they become ill or injured as a direct result of participating in this study, they may receive medical care. Your study doctor will explain the treatment options to you and tell you where you can get treatment. Generally, this care will be billed to you, your insurance or other third party. Additionally, AstraZeneca shall reimburse the institution for the direct, reasonable, and necessary medical expenses incurred by the institution for

the treatment of any personal injury that is a direct result of adverse drug experiences to subjects resulting directly from any manufacturing defects in the study drug to which the subjects would not have been exposed but for their participation in the study if (i) the institutional parties have complied with this agreement, the protocol and any written instructions of AstraZeneca concerning the study drug and (ii) all the requirements of informed consent have been complied with. AstraZeneca will not provide compensation for lost wages or for any other damages, expenses or losses, or for medical expenses have have been covered by a subject's medical or other insurance.

23.2 Provide a copy of contract language, if any, relevant to compensation for research related injury.

*NOTE: If the contract is not yet approved at the time of this submission, submit the current version here. If the contract is later approved with **different language regarding research related injury**, you must modify your response here and submit an amendment to the IRB for review and approval.*

Response: AstraZeneca shall reimburse the institution for the direct, reasonable, and necessary medical expenses incurred by the institution for the treatment of any personal injury that is a direct result of adverse drug experiences to subjects resulting directly from any manufacturing defects in the study drug to which the subjects would not have been exposed but for their participation in the study if (i) the institutional parties have complied with this agreement, the protocol and any written instructions of AstraZeneca concerning the study drug and (ii) all the requirements of informed consent have been complied with. AstraZeneca will not provide compensation for lost wages or for any other damages, expenses or losses, or for medical expenses have have been covered by a subject's medical or other insurance.

24.0 Economic Burden to Subjects

24.1 Describe any costs that subjects may be responsible for because of participation in the research.

NOTE: Some examples include transportation or parking.

Response: All research expenses will be covered by the study. The background use of insulin is considered as part of standard of care is the responsibility of the patients and his/her insurance. Participants will not be subjected to any other out of pocket cost.

☐ **N/A:** This study is not enrolling subjects, or is limited to records review procedures only. This section does not apply.

25.0 Compensation for Participation

25.1 Describe the amount and timing of any compensation to subjects, including monetary, course credit, or gift card compensation.

Response:

Visit schedule:

- Screen: $0
- Visit 1 (Week -3): Insulin adjustment: $10
- Visit 2 (Week -1): Meal ($75), insulin adjustment, and 24hr blood pressure monitor ($50): $125*
- Visit 3 (Week 0): 24 hr urine ($15), blood draw ($25), fat biopsy ($75), insulin adjustment, questionnaire ($10): $125
- Visit 4 (Week 1): Insulin adjustment: $10
- Visit 5 (Week 2): Insulin adjustment: $10
- Visit 6 (Week 4): Insulin adjustment: $10
- Visit 7 (Week 13): 24hr urine ($15), blood draw ($25), insulin adjustment, 24hr bp monitor ($50): $90*
- Visit 8 (Week 23): Insulin adjustment: $10
- Visit 9 (Week 25): Insulin adjustment, 24bp monitor ($50), Meal ($75): $125*
- Visit 10 (Week 26): Insulin adjustment, 24hr urine ($15), blood draw ($25), fat biopsy ($75), questionnaire ($10): $125*
- Visit 11 (Week 27): Insulin adjustment: $10
- Visit 12 (Weak 31): Insulin adjustment, blood draw: $25
- Visit 13 (Week 39): Insulin adjustment, 24hr urine ($15), blood draw ($25), 24hr bp monitor ($50): $90*
- Visit 14 (Week 49): Insulin adjustment: $10
- Visit 15 (Week 51): Insulin adjustment, 24bp monitor ($50), meal ($75): $125*
- Visit 16 (Week 52): Insulin adjustment, 24hr urine ($15), blood draw ($25), questionnaire ($10), fat biopsy ($75):$125*

*Total compensation for all completed visits: up to $1,025.00. For those not wishing to participate in the optional blood pressure monitoring and/or fat biopsy procedure, the compensation will decrease by $250 for not completing the bp monitoring, and by yet another $225.00 for not completing the fat biopsies.

☐ **N/A:** This study is not enrolling subjects, or is limited to records review procedures only. This section does not apply.

☐ **N/A:** There is no compensation for participation. This section does not apply.

26.0 Consent Process

26.1 Indicate whether you will be obtaining consent.

NOTE: This does not refer to consent documentation, but rather whether you will be obtaining permission from subjects to participate in a research study. Consent documentation is addressed in Section 27.0.

☒ ***Yes*** *(If yes, Provide responses to each question in this Section)*

☐ ***No*** *(If no, Skip to Section 27.0)*

26.2 Describe where the consent process will take place. Include steps to maximize subjects' privacy.

Response: All participants will come to the research department to be consented. Participants will be placed in a private, screened off area and or room where they can review the consent. Participant questions and or concerns will be address with a member of the study team or research doctor if applicable. The research coordinator will discuss in length the participants requests for privacy of their PHI.

26.3 Describe how you will ensure that subjects are provided with a sufficient period of time to consider taking part in the research study.

NOTE: It is always a requirement that a prospective subject is given sufficient time to have their questions answered and consider their participation. See "SOP: Informed Consent Process for Research (HRP-090)" Sections 5.5 and 5.6.

Response: participants will be made aware that participating in research is completely voluntary, and they may withdraw at any time with no consequence to their routine clinic care. If the patients requires time to decide and or discuss partaking in a research study, the subject will be given said time.

26.4 Describe any process to ensure ongoing consent, defined as a subject's willingness to continue participation for the duration of the research study.

Response: The research coordinator and study team are available to answer any question or concerns with the patient during the duration of the research trial. At each study visit, the patient is asked a series of questions to ensure they are on task with the study visits and feel comfortable. Upon departing from their study visit, the patients are told of their next visit and given detail instruction for their next visit. If study is revised or amendment or new information becomes available about drug safety that may affect patients participation, the patient may be re-consented to ensure patient ongoing consent

26.5 Indicate whether you will be following "SOP: Informed Consent Process for Research (HRP-090)." If not, or if there are any exceptions or additional details to what is covered in the SOP, describe:

- *The role of the individuals listed in the application who are involved in the consent process*
- *The time that will be devoted to the consent discussion*
- *Steps that will be taken to minimize the possibility of coercion or undue influence*
- *Steps that will be taken to ensure the subjects' understanding*

Response:

☒ We have reviewed and will be following "SOP: Informed Consent Process for Research (HRP-090)."

Non-English Speaking Subjects

☒ **N/A:** This study will not enroll Non-English speaking subjects.
(Skip to Section 26.8)

26.6 Indicate which language(s) other than English are likely to be spoken/understood by your prospective study population or their legally authorized representatives.

NOTE: The response to this Section should correspond with your response to Section 6.4 of this protocol.

Response:

26.7 If subjects who do not speak English will be enrolled, describe the process to ensure that the oral and written information provided to those subjects will be in that language. Indicate the language that will be used by those obtaining consent.

NOTE: Guidance is provided on "SOP: Informed Consent Process for Research (HRP-090)."

Response:

Cognitively Impaired Adults

☒ **N/A**: This study will not enroll cognitively impaired adults.
(Skip to Section 26.9)

26.8 Describe the process to determine whether an individual is capable of consent.

Response:

Adults Unable to Consent

☒ **N/A**: This study will not enroll adults unable to consent.
(*Skip to Section 26.13)*

When a person is not capable of consent due to cognitive impairment, a legally authorized representative should be used to provide consent (Sections 26.9 and

*26.10) **and, where possible, assent of the individual should also be solicited** (Sections 26.11 and 26.12).*

26.9 Describe how you will identify a Legally Authorized Representative (LAR). Indicate that you have reviewed the "SOP: Legally Authorized Representatives, Children, and Guardians (HRP-013)" for research in New York State.

NOTE: Examples of acceptable response includes: verifying the electronic medical record to determine if an LAR is recorded.

Response:

☐

*26.10 **For research conducted outside of New York State**, provide information that describes which individuals are authorized under applicable law to consent on behalf of a prospective subject to their participation in the research. One method of obtaining this information is to have a legal counsel or authority review your protocol along with the definition of "legally authorized representative" in "SOP: Legally Authorized Representatives, Children, and Guardians (HRP-013)."*

Response:

*26.11 Describe the process for **assent of the adults**:*

- *Indicate whether assent will be obtained from all, some, or none of the subjects. If some, indicate which adults will be required to assent and which will not.*

  Response:

- *If assent will not be obtained from some or all subjects, provide an explanation of why not.*

  Response:

*26.12 Describe whether **assent of the adult** subjects will be documented and the process to document assent.*

NOTE: The IRB allows the person obtaining assent to document assent on the consent document using the "Template Consent Document (HRP-502)" Signature Block for Assent of Adults who are Legally Unable to Consent.

Response:

Subjects who are not yet Adults (Infants, Children, and Teenagers)

☒ **N/A**: This study will not enroll subjects who are not yet adults.
(Skip to Section 27.0)

26.13 Describe the criteria that will be used to determine ***whether a prospective subject has not attained the legal age for consent to treatments or procedures involved in the research*** *under the applicable law of the jurisdiction in which the research will be conducted* ***(e.g., individuals under the age of 18 years)****. For research conducted in NYS, review "SOP: Legally Authorized Representatives, Children, and Guardians (HRP-013)" to be aware of which individuals in the state meet the definition of "children."*

NOTE: Examples of acceptable responses include: verification via electronic medical record, driver's license or state-issued ID, screening questionnaire.

Response:

26.14 ***For research conducted outside of New York State****, provide information that describes which persons have not attained the legal age for consent to treatments or procedures involved the research, under the applicable law of the jurisdiction in which research will be conducted. One method of obtaining this information is to have a legal counsel or authority review your protocol along the definition of "children" in "SOP: Legally Authorized Representatives, Children, and Guardians (HRP-013)."*

Response:

26.15 Describe whether parental permission will be obtained from:

Response: N/A

☐ One parent even if the other parent is alive, known, competent, reasonably available, and shares legal responsibility for the care and custody of the child.

☐ Both parents unless one parent is deceased, unknown, incompetent, or not reasonably available, or when only one parent has legal responsibility for the care and custody of the child.

☐ Parent permission will not be obtained. A waiver of parent permission is being requested.

NOTE: The requirement for parent permission is a protocol-specific determination made by the IRB based on the risk level of the research. For guidance, review the "CHECKLIST: Children (HRP-416)."

26.16 Describe whether permission will be obtained from individuals ***other than parents****, and if so, who will be allowed to provide permission. Describe*

your procedure for determining an individual's authority to consent to the child's general medical care.

Response:

26.17 Indicate whether assent will be obtained from all, some, or none of the ***children****. If assent will be obtained from some children, indicate which children will be required to assent.*

Response:

26.18 When assent of children is obtained, describe how it will be documented.

Response:

27.0 Waiver or Alteration of Consent Process

Consent will not be obtained, required information will not be disclosed, or the research involves deception.

☒ **N/A:** A waiver or alteration of consent is not being requested.

27.1 If the research involves a waiver or alteration of the consent process, please review the "CHECKLIST: Waiver or Alteration of Consent Process (HRP-410)" to ensure that you have provided sufficient information for the IRB to make the determination that a waiver or alteration can be granted.

NOTE: For records review studies, the first set of criteria on the "CHECKLIST: Waiver or Alteration of Consent Process (HRP-410)" applies.

Response:

27.2 If the research involves a waiver of the consent process for planned emergency research, please review the "CHECKLIST: Waiver of Consent for Emergency Research (HRP-419)" to ensure you have provided sufficient information for the IRB to make these determinations. Provide any additional information necessary here:

Response:

28.0 Process to Document Consent

☐ **N/A:** A Waiver of Consent is being requested.
(Skip to Section 29.0)

28.1 Indicate whether you will be following "SOP: Written Documentation of Consent (HRP-091)." If not or if there are any exceptions, describe whether

and how consent of the subject will be obtained including whether or not it will be documented in writing.

NOTE: If your research presents no more than minimal risk of harm to subjects and involves no procedures for which written documentation of consent is normally required outside of the research context, the IRB will generally waive the requirement to obtain written documentation of consent. This is sometimes referred to as 'verbal consent.' Review "CHECKLIST: Waiver of Written Documentation of Consent (HRP-411)" to ensure that you have provided sufficient information.

📎 *If you will document consent in writing, attach a consent document with your submission. You may use "TEMPLATE CONSENT DOCUMENT (HRP-502)". If you will obtain consent, but not document consent in writing, attach the script of the information to be provided orally or in writing (i.e. consent script or Information Sheet).*

Response:

☒ We will be following "SOP: Written Documentation of Consent" (HRP-091).

29.0 Multi-Site Research (Multisite/Multicenter Only)

☐ **N/A:** This study is not an investigator-initiated multi-site study. This section does not apply.

29.1 If this is a multi-site study ***where you are the lead investigator****, describe the processes to ensure communication among sites, such as:*

- *All sites have the most current version of the IRB documents, including the protocol, consent document, and HIPAA authorization.*
- *All required approvals have been obtained at each site (including approval by the site's IRB of record).*
- *All modifications have been communicated to sites, and approved (including approval by the site's IRB of record) before the modification is implemented.*
- *All engaged participating sites will safeguard data as required by local information security policies.*
- *All local site investigators conduct the study appropriately.*
- *All non-compliance with the study protocol or applicable requirements will be reported in accordance with local policy.*

Response:

29.2 Describe the method for communicating to engaged participating sites:

- *Problems*
- *Interim results*
- *Study closure*

Response:

29.3 Indicate the total number of subjects that will be enrolled or records that will be reviewed across all sites.

Response: 114

29.4 If this is a multicenter study for which UB will serve as the IRB of record, and subjects will be recruited by methods not under the control of the local site (e.g., call centers, national advertisements) describe those methods.

Response: N/A

30.0 Banking Data or Specimens for Future Use

☐ **N/A:** This study is not banking data or specimens for future use or research outside the scope of the present protocol. This section does not apply.

*30.1 If data or specimens will be banked (stored) for **future use, that is, use or research outside of the scope of the present protocol**, describe where the data/specimens will be stored, how long they will be stored, how the data/specimens will be accessed, and who will have access to the data/specimens.*

NOTE: Your response here must be consistent with your response at the "What happens if I say yes, I want to be in this research?" Section of the Template Consent Document (HRP-502).

Response: The study data/specimens will be stored in a locked closet or -80 freezer at the research facility of the Diabetes and Endocrinology Center of WNY for at least 7 years.

The research staff (study personnel including coordinator) only will be authorized to access data and or specimens

30.2 List the data to be stored or associated with each specimen.

Response: Patient ID number, study visit information and date of collection will be stored with specimen. Other data stored will include record files of all patients participating in the study, including data collection sheets, lab results, CGM and insulin pump data.

30.3 Describe the procedures to release banked data or specimens for future uses, including: the process to request a release, approvals required for release, who can obtain data or specimens, and the data to be provided with specimens.

Response: The copy of the individual patient data collected during the study period will be provided to these individual patients who can choose to hand

carry it to their respective physicians and a copy will be faxed to their respective clinical providers upon verbal request from the patient. The data provided will include the insulin pump or CGM data or any of the lab results obtained during the study period. The results of the completed study will be made available to the patients if requested through published manuscript.

31.0 Drugs or Devices

☐ **N/A:** This study does not involve drugs or devices. This section does not apply.

31.1 If the research involves drugs or devices, list and describe all drugs and devices used in the research, the purpose of their use, and their regulatory approval status.

Response: **Drugs used:**

Description or Identity of investigational product(s)

Investigational product	***Dosage form and strength***	***Manufacturer***	***Description***
Dapagliflozin (Farxiga) 5 mg	*Green, plain, diamond shaped, film coated 5 mg tablet*	*AstraZeneca*	*sodium-glucose cotransporter 2 (SGLT2) inhibitor indicated as an adjunct to diet and exercise to improve glycemic control*
Matching placebo for dapagliflozin 5 mg	*Green, plain, diamond shaped, film coated tablet*	*AstraZeneca*	
Semaglutide (Ozempic) 0.25 mg, 0.5 mg, 1.0 mg	*clear, colorless solution that contains 2 mg of semaglutide in a 1.5 mL (1.34 mg/mL) pre-filled, disposable, single-patient-use pen injector*	*Novo Nordisk*	*glucagon-like peptide 1 (GLP-1) receptor agonist indicated as an adjunct to diet and exercise to improve glycemic control*

- *Dapagliflozin and its matching placebo tablets will be supplied in bottles and are provided by Astra Zeneca.*
- *Semaglutide will be supplied as prefilled injectable pens and will be purchased commercially through the University at Buffalo Research Pharmacy.*

Subjects will receive drugs in their unmodified form. Dapagliflozin is a once daily tablet, while Semaglutide is a weekly subcutaneous injection.

Insulin pumps (CSII) and long and short acting insulin is the only diabetes medication accepted before and during this trial. No other diabetes drug is permitted.
Blood pressure or lipid lowering drugs are permitted if on stable dose for at least three months prior to start of trial. Glucagon injection is permitted as rescue medication for severe hypoglycemia. Insulin infusions may be administered by ER or hospital if patients develop DKA.

31.2 Describe your plans to store, handle, and administer those drugs or devices so that they will be used only on subjects and be used only by authorized investigators.

Response: Dapagliflozin 5mg tablets and matching placebo provided by Astra Zeneca will be stored in a locked cabinet in research pharmacy. Semaglutide provided by University at Buffalo research pharmacy will be stored in temperature controlled refrigerator at 4C at the research facility of the Diabetes and Endocrinology Center of WNY at 1000 Youngs Rd until dispensed to patients. Investigational drugs will be dispensed by PI or CO-Is only to study patients according to randomization plan.

If the drug is investigational (has an IND) or the device has an IDE or a claim of abbreviated IDE (non-significant risk device), include the following information:

31.3 Identify the holder of the IND/IDE/Abbreviated IDE.

Response: IND application for use of all drugs (dapagliflozin and semaglutide) in type 1 diabetes will be submitted to FDA and no study procedure will start before all local and federal approvals are obtained. FDA has granted IND for usage of dapagliflozin in type 1 diabetes. We will seek FDA approval for the use of semaglutide in this study. These IND letters will be submitted to IRB and JDRF along with final version of protocol before initiating any study activities.

31.4 Explain procedures followed to comply with FDA sponsor requirements for the following:

	Applicable to:		
FDA Regulation	***IND Studies***	***IDE studies***	***Abbreviated IDE studies***
21 CFR 11	***X***	***X***	
21 CFR 54	***X***	***X***	
21 CFR 210	***X***		
21 CFR 211	***X***		
21 CFR 312	***X***		
21 CFR 812		***X***	***X***
21 CFR 820		***X***	

Response: All FDA sponsor requirements have been reviewed and will be followed during the study procedures

32.0 Humanitarian Use Devices

☒ **N/A:** This study does not involve humanitarian use devices. This does not apply.

32.1 For Humanitarian Use Device (HUD) uses provide a description of the device, a summary of how you propose to use the device, including a description of any screening procedures, the HUD procedure, and any patient follow-up visits, tests or procedures.

Response:

32.2 For HUD uses provide a description of how the patient will be informed of the potential risks and benefits of the HUD and any procedures associated with its use.

Response:

US TTT1 Protocol Summary of changes V4 to V5

Section	Original	New	Justification	Consent changes
6.2	ESRD on hemodialysis; and or e-GFR < 60 ml/min/1.73m2	ESRD on hemodialysis; and or e-GFR < 45 ml/min/1.73m2	reducing eGFR limit to be included in the study from 60 to 45. This is based on dapagliflozin prescribing information.	yes
10.1 (visit 10)	No statement	Also, they will be provided with additional blood ketone strips to measure blood ketones when needed. If results of these daily scheduled (i.e. not done due to symptoms) urine ketone measurements, are small or higher (+1 or more), they will be instructed to measure blood ketones and to repeat both measurements after 60 min.	including blood ketone testing at home for all patients if home urine ketones measurement result is +1 or more. This applies during the 2nd phase of randomization (week 26 till end of study).	yes

University at Buffalo
The State University of New York

University at Buffalo Institutional Review Board (UBIRB)
Office of Research Compliance | Clinical and Translational Research Center Room 5018
875 Ellicott St. | Buffalo, NY 14203
UB Federalwide Assurance ID#: FWA00008824

Complete Research Protocol (HRP-503)

Table of Contents

Template Instructions 2
1.0 Objectives 4
2.0 Scientific Endpoints 5
3.0 Background 6
4.0 Study Design 12
5.0 Local Number of Subjects 12
6.0 Inclusion and Exclusion Criteria 13
7.0 Vulnerable Populations 15
8.0 Eligibility Screening 17
9.0 Recruitment Methods 17
10.0 Procedures Involved 19
11.0 Study Timelines 31
12.0 Setting 31
13.0 Community-Based Participatory Research 32
14.0 Resources and Qualifications 33
15.0 Other Approvals 34
16.0 Provisions to Protect the Privacy Interests of Subjects 34
17.0 Data Management and Analysis 35
18.0 Confidentiality 38
A. Confidentiality of Study Data 38
B. Confidentiality of Study Specimens 39
19.0 Provisions to Monitor the Data to Ensure the Safety of Subjects 40
20.0 Withdrawal of Subjects 47
21.0 Risks to Subjects 48
22.0 Potential Benefits to Subjects 54
23.0 Compensation for Research-Related Injury 54
24.0 Economic Burden to Subjects 55
25.0 Compensation for Participation 56
26.0 Consent Process 56
27.0 Waiver or Alteration of Consent Process 61
28.0 Process to Document Consent 62
29.0 Multi-Site Research (Multisite/Multicenter Only) 62
30.0 Banking Data or Specimens for Future Use 63
31.0 Drugs or Devices 64
32.0 Humanitarian Use Devices 66

Template Instructions

Sections that do not apply:

- *In several sections, the addition of checkboxes for* ***Not Applicable*** *have been added to the template as responses.*
  - *If an N/A checkbox is present, select the appropriate justification from the list.*
  - *If an N/A checkbox is not present, or if none of the existing checkboxes apply to your study, you must write in your own justification.*
- *In addition:*
  - *For research where the only study procedures are records/chart review: Sections 19, 20, 22, 23, 24, 25, 31, and 32 do not apply.*
  - *For exempt research: Sections 31 and 32 do not apply.*

Studies with multiple participant groups:

- *If this study involves multiple participant groups (e.g. parents and children), provide information in applicable sections for each participant group. Clearly label responses when they differ. For example:*

  Response:

  Intervention Group:

  Control Group:

Formatting:

- *Do not remove template instructions or section headings when they do not apply to your study.*

If you are pasting information from other documents using the "Merge Formatting" Paste option will maintain the formatting of the response boxes.

Amendments:

- *When making modifications or revisions to this and other documents, use the* ***Track Changes*** *function in Microsoft Word.*
- *Update the version date or number* ***on Page 3.***

PROTOCOL TITLE:

Include the full protocol title.

Response: **Triple Therapy for Type 1 Diabetes with Insulin, Semaglutide, and Dapagliflozin**

PRINCIPAL INVESTIGATOR:

Response:

**Principal Investigator:**	**Paresh Dandona, MBBS, PhD**
**Co- Investigators:**	**Husam Ghanim, PhD**
	**Antoine Makdissi, MD**
	**Manav Batra, MD**
**Co- Principal Investigator:**	**John R Petrie, MBChB, PhD**

Paresh Dandona, M.B.B.S., Ph.D., F.R.C.P, F.A.C.P, F.A.C.C, FACE,
SUNY Distinguished Professor,
Director, Diabetes-Endocrinology Center of Western NY
Chief of Endocrinology, Diabetes, Endocrinology and Metabolism, SUNY at Buffalo
1000 Youngs Road, Suite 105
Williamsville NY 14221
Telephone: (716) 535-1850
Fax: 716-639-1200
E-mail: pdandona@kaleidahealth.org

CO-PI:

Professor John R Petrie
BSc MBChB PhD FRCP(Ed) FRCPSG
Professor of Diabetic Medicine
Institute of Cardiovascular and Medical Sciences
BHF Glasgow Cardiovascular Research Centre
University of Glasgow
126 University Place
Glasgow G12 8TA
Tel: +44(0)141 330 3325
Email: john.petrie@glasgow.ac.uk
Web: http://www.gla.ac.uk/researchinstitutes/icams/staff/johnpetrie/
The University of Glasgow

VERSION:

Include the version date or number.

Response: 2/10/2022, V5

GRANT APPLICABILITY:

Indicate whether this protocol is funded by a grant (e.g. NIH, foundation grant). For a grant with multiple aims, indicate which aims are covered by this research proposal.

NOTE: This question does not apply to studies funded by a sponsor contract.

Include a copy of the grant proposal with your submission.

Response: Research support provided by JDRF and AZ

RESEARCH REPOSITORY:

Indicate where the research files will be kept, including when the study has been closed. The repository should include, at minimum, copies of IRB correspondence (approval, determination letters) as well as signed consent documents. This documentation should be maintained for 3 years after the study has been closed.

Response:
Site 1: Diabetes Endocrinology Research Center of WNY
Address: 1000 Youngs Road, Suite 105, Williamsville NY 14221
Department: Diabetes Endocrinology

Site 2: University of Glasgow. Institute of Cardiovascular and Medical Sciences University of Glasgow 126 University Place Glasgow, G12 8TA

1.0 Objectives

1.1 Describe the purpose, specific aims, or objectives of this research.

Response:

Primary Objective: To assess whether the addition of dapagliflozin to semaglutide and insulin (triple therapy) improves glycemic control in patients with type 1 diabetes compared with semaglutide and insulin (dual therapy) and insulin only (standard) treatment.

General hypothesis: The addition of dapagliflozin to semaglutide and insulin treatment improves glycemic control, weight loss and blood pressure in adults with type 1 diabetes.

1.2 State the hypotheses to be tested, if applicable.

NOTE: A hypothesis is a specific, testable prediction about what you expect to happen in your study that corresponds with your above listed objectives.

Response:

Specific hypotheses:

Primary: combined dapagliflozin and semaglutide treatment in addition to insulin will improve HbA1c levels compared with semaglutide in addition to insulin .

Secondary 1: semaglutide in addition to insulin treatment over 26 weeks will improve HbA1c levels compared with insulin therapy alone.

Secondary 2: combined dapagliflozin and semaglutide treatment in addition to insulin will improve HbA1c levels compared to insulin therapy alone at 52 weeks.

Secondary 3: combined dapagliflozin and semaglutide treatment in addition to insulin will improve CGM-based weekly mean and fasting blood glucose, glycemic oscillations and percent of time in range (70-180mg/dl) with lower insulin requirements compared with semaglutide and insulin (dual therapy) or insulin only (standard) treatment.

Secondary : combined dapagliflozin and semaglutide in addition to insulin therapy will reduce systolic and diastolic blood pressure compared with semaglutide and insulin (dual therapy) or insulin only (standard) treatment.

Secondary 5: combined dapagliflozin and semaglutide in addition to insulin therapy (triple therapy) will result in weight loss compared to dual and standard therapy.

Secondary 6: combined dapagliflozin and semaglutide in addition to insulin therapy will result in improved scores on the Diabetes Specific Quality of Life Scale (DSQOLS) and the Problem Areas In Diabetes (PAID) survey compared with dual and standard therapy.

Tertiary and Exploratory Objectives (EO) for future ancillary studies (PLEASE NOTE: For EO, samples will be collected to facilitate the measurements of these end points in future ancillary studies once submitted and approved by JDRF so cost of assays is not included in the current budget): The addition of dapagliflozin to semaglutide and insulin in type 1 diabetes will:

Tertiary 1: combined dapagliflozin and semaglutide treatment in addition to insulin therapy will improve fasting and postprandial glucose excursions and suppress postprandial glucagon, FFA, GLP-1 compared with semaglutide and insulin or insulin therapy alone.

Tertiary 2: semaglutide in addition to insulin treatment over 52 weeks will improve HbA1c levels compared with insulin therapy alone.

EO 1: increase plasma concentrations of vasodilatation mediators and reduce plasma concentrations of vasoconstriction mediators compared to dual and standard therapy groups.

EO 2: exert a systemic anti-inflammatory effect including reducing mRNA expression of inflammatory mediators and NFκB binding in peripheral mononuclear cells (MNC) and adipose tissue (optional) and their circulating levels in plasma while increasing levels of IL-RA and adiponectin.

2.0 Scientific Endpoints

2.1 Describe the scientific endpoint(s), the main result or occurrence under study.

NOTE: Scientific endpoints are outcomes defined before the study begins to determine whether the objectives of the study have been met and to draw conclusions from the data. Include primary and secondary endpoints. Some example endpoints are: reduction of

symptoms, improvement in quality of life, or survival. Your response should ***not*** *be a date.*

Response:

Primary endpoint: Change in HbA1c at 6 months following dapagliflozin or placebo therapy in addition to combined semaglutide and insulin treatment.

Efficacy Secondary endpoints:

1. Change in HbA1c from baseline at 12 months in triple therapy group compared to insulin only group.
2. Change in HbA1c from baseline at 6 months in dual therapy group compared to insulin only group.
3. Change in percent Time in Range (70– 180 mg/dl), percent time in hyperglycemia Level 1(180-250mg/dl) and Level 2 (>250mg/dl) and percent time in hypoglycemia Level 1 (54-70mg/dl) as assessed by CGM
4. Other indices of glycemic control: fructosamine, weekly mean and fasting glucose, standard deviation, insulin requirements.
5. Change in body weight and quality of life as assessed by DSQOLS and PAID at 6 and 12 months.
6. Anti-hypertensive effects including change in systolic and diastolic BP, numbers of BP medications required.

Safety Secondary endpoints:

1- Differences in rates of hypoglycemic events Level 2 (<54mg/dl) and Level 3 (A severe event characterized by altered mental and/or physical status requiring assistance) between investigative arms and standard therapy arm.

2- *Differences in rates of diabetic ketoacidosis defined as elevated serum or urine ketones (greater than the upper limit of the normal range), and serum bicarbonate <15 mmol/L or Blood pH <7.3 between investigative arms and standard therapy arm.*

Tertiary/ exploratory endpoints: (items 2-4 to be performed in future on collected samples from this study if additional funding is approved by JDRF):

1- Fasting and postprandial (Area Under curve) glucose excursions, FFA, GLP-1 and glucagon and fasting fructosamine.

2- Indices of inflammatory stress. These will be CRP, FFA, SAA, IL-1β, IL-18, IL-1RA, adiponectin and TNFα concentrations in plasma and isoprostane in urine.

3- Expression of IL-1β, TNFα, IL-12, TLR2, TLR4, adiponectin, IκBα as well as NFκB binding in peripheral MNC and adipose tissue (optional).

4- Indices of vasodilatation and vasoconstriction: ANP, b-NP, cGMP, cAMP, angiotensinogen, renin and angiotensin II concentrations.

3.0 Background

3.1 Provide the scientific or scholarly background, rationale, and significance of the research based on the existing literature and how it will contribute to existing knowledge. Describe any gaps in current knowledge. Include relevant preliminary findings or prior research by the investigator.

Response:

The control of glucose homeostasis in type 1 diabetes is fragile since β-cell reserve is negligible. Traditionally, insulin has been the only treatment approach available. This approach, often leads to erratic blood sugar control given the challenge of matching exogenous insulin to mealtime glucose excursions in the face of a lack of any endogenous insulin to compensate for variation in requirements in response to meals and activity levels. Injected insulin boluses rarely match precise insulin requirements either in terms of dose administered or bio-availability. The result often is wide fluctuations in blood glucose readings. Based on our work to date and other published reports, previous attempts to add other glucose-lowering medications to insulin therapy yielded minimal benefits on glycemia (although metformin may have other benefits (1-3)

We have recently shown in two preliminary retrospective studies that the addition of liraglutide, a Glucagon Like Peptide -1 receptor agonist (GLP-1RA), to insulin results in consistent improvements in glycemic control in type 1 diabetes as demonstrated by reductions in HbA1c, glycemic excursions, and insulin requirements accompanied by improvements in body weight and systolic blood pressure (4, 5). We have now also completed the first prospective randomized controlled trial of this drug in 72 people with type 1 diabetes. Our data not only confirmed improvements in glycemic control, HbA1c, body weight and systolic BP, but also demonstrated a reduction in appetite, carbohydrate intake in low grade inflammation as measured by plasma CRP concentrations (6). Importantly, quality of life was also improved. Our recent studies in type 2 diabetes have revealed that systolic blood pressure reduction with GLP-1RA (exenatide) treatment in type 2 diabetes is associated with increased plasma concentrations of ANP, cGMP and cAMP along with reduced concentrations of angiotensinogen, renin and angiotensin II (7). It is possible that these mechanisms play a role in the prevention of cardiovascular complications in type 1 diabetes.

While GLP-1RAs, therefore, have great potential as adjunct therapy for type 1 diabetes, mean HbA1c was still ≥7% after liraglutide treatment (6) and larger pharma-sponsored trials reported increased rates of hypoglycemia (8, 9). In seeking further improvement, the advent of sodium-glucose transport protein-2 (SGLT2) inhibitors is of importance since their mode of action is independent of insulinogenesis. Introduced for use in type 2 diabetes, SGLT2 inhibitors are selective antagonists of the sodium-glucose transporter subtype expressed in the proximal convoluted tubules of the kidneys. They act by inhibiting renal glucose re-absorption and therefore stimulating excretion of 60-100 grams per day of glucose in urine. SGLT2 inhibitors have been studied extensively in type 2 diabetes, including the landmark cardiovascular outcome trial EMPA-REG (10), but data have only quite recently begun to accumulate in type 1(11). Short term effects on urine glucose excretion of remogliflozin and empagliflozin are similar to those seen in type 2 diabetes and these translate into improved glycemic and other indices including HbA1c; fasting blood glucose, rates of hypoglycemia, daily insulin dose and weight (12). Glucose-lowering efficacy is attenuated in individuals with eGFR <45/ml/min/1.73m^2(13). Therefore, use of dapagliflozin is recommended for patients with eGFR>45/ml/min/1.73 m^2 (as per prescribing information). Weight loss accompanying increased urinary glucose excretion could translate into reduced cardiovascular complications as in type 2 diabetes, but there are no data so far available to support this hypothesis. More recently, in the Phase 3 trial DEPICT-1 we showed that dapagliflozin improves glycemic control and HbA1c over six months by about 0.45% with no severe hypoglycemia or diabetic ketoacidosis

(14). Similarly, sotagliflozin has been shown in a Phase 3 trial to allow a larger proportion of patients to achieve HbA1c lower than 7.0% with no severe hypoglycemia although the rate of diabetic ketoacidosis was higher in the sotagliflozin group (15). Both products are now being reviewed by regulatory agencies for the type 1 diabetes indication.

On the basis of the above, we hypothesize that adding an SGLT2 inhibitor to a combination of insulin and semaglutide (a weekly and potent GLP-1RA) will improve glycemic control in type 1 diabetes such that HbA1c levels of ≤7% are achieved more consistently. Our protocol is designed to allow an objective assessment of whether this triple therapy combination will work effectively and safely so that a majority of people with type 1 diabetes can reach a glycemic target HbA1c of ≤7%. In addition, since both semaglutide and dapagliflozin inhibitors reduce blood pressure and weight, it will allow an assessment of the combined effect of GLP-1RAs and SGLT2 inhibitors on these outcomes. Since there are no previous data on the effects of once weekly semaglutide or the combination of semaglutide and dapagliflozin compared to standard insulin treatment in type 1 diabetes, the design requires allocation of some participants to insulin therapy only.

Due to the potential ketogenic effect of SGLT-2 inhibitors, data from SGLT-2 inhibitors studies in type 1 diabetes were analyzed for it ketogenic effects and a consensus statement and guidelines on management of patients was recently published (Danne T et al., Diabetes Care. 2019 Feb 6).

Preliminary data

1) Liraglutide treatment in patients with type 1 diabetes (4): We treated 8 type 1 diabetes patients; mean age (38.5 years) and duration of diabetes (20±4 yrs), with liraglutide in addition to insulin for 24 weeks. As shown in table 1, there was a significant reduction in fasting and mean blood glucose concentrations, insulin doses, HbA1c and a reduction in appetite and caloric intake with weight loss. In addition, there was a reduction in the excursions of blood glucose concentrations, i.e., the standard deviations of blood glucose concentrations were markedly reduced (**figure 1**). The changes were observed within 48h and improved marginally thereafter with fine tuning of insulin doses and a further reduction in these doses. These observations are noteworthy in that study participants were well controlled and meticulous users of CSII and CGM. They had all tried hard to improve their diabetic control by adjusting insulin doses but had not succeeded because of hypoglycemia. We then demonstrated that poorly controlled and obese individuals with type 1 diabetes also experienced similar benefits of liraglutide treatment (2).

2) Liraglutide As Additional Treatment to Insulin in Patients with Type 1 Diabetes Mellitus: A Randomized Clinical Trial (6): Following the two above non-randomized studies we recently conducted a randomized clinical trial in which 72 individuals (Placebo=18; liraglutide = 54) with TYPE 1 DIABETES for at least one year, on insulin therapy and with no detectable C-peptide in plasma (mean BMI: 30±1; mean body weight: 84±2 kg ; mean HbA1c: 7.57±0.09%; mean age: 44±2 years; mean age of TYPE 1 DIABETES diagnosis: 20±1 years) were randomized to receive placebo, 0.6, 1.2 or 1.8mg of liraglutide daily for 12 weeks. In the 1.2 mg and 1.8 mg groups, mean change in average blood glucose was -10±2 and -10.0±1mg/dl, respectively, ($p<0.0001$ vs. placebo). In the 1.2mg group, HbA1c fell by 0.78% from 7.84±0.17% to 7.06±0.15% ($p<0.0001$, $p<0.01$

vs. placebo) and in the 1.8mg group fell by 0.42% from 7.41±0.15 to 6.99±0.15 (p=0.001, p=0.39 vs placebo). Time spent between 70 to 160 mg/dl increased by 5±1% (p<0.05 vs placebo) and time between 160-400 mg/dl decreased by 7±1% (p<0.01 vs placebo) in the 1.8 mg group with no additional hypoglycemia. During the 12 week period, the average total daily dose of insulin in the two groups fell by 12.4±3.9 and 10.0±2.3 units respectively (p<0.05 vs baseline and placebo). There was a reduction in body weight (from 83±4 to 78±5 kg, p<0.0001, in the 1.8mg group, from 96±4 to 91±4 kg, p<0.001, in the 1.2 mg group and from 80±4 to 77±4 kg, p<0.01 in the 0.6 mg group). A reduction in daily carbohydrate intake (171±17g vs. 127±18g; p<0.01 and 153±18g vs 115±16g, p<0.01 in the 1.2 mg and 1.8 mg groups, respectively) was also observed over 12 weeks. Systolic BP (SBP) in the 1.8 mg group fell by 3 mmHg (120±2 to 117±3 mmHg, p=0.01). CRP fell by 15±6 and 19±8% in the 1.2 and 1.8mg groups, respectively (p<0.05). Quality of life improved significantly compared to baseline and placebo (unpublished data).

3) Dapagliflozin as an Additional Treatment to Insulin and Liraglutide in Patients with Type 1 Diabetes Mellitus (16): The objective of this randomized clinical trial was to investigate whether addition of dapagliflozin to insulin and liraglutide results in a significant reduction in glycemia and body weight. 30 participants with TYPE 1 DIABETES on liraglutide therapy for at least last 6 months were randomized (in 2:1 ratio) to receive either dapagliflozin 10 mg or placebo daily for 12 weeks. With 12 weeks of dapagliflozin HbA1c fell by 0.66±0.08% from 7.8±0.21% (p < .01 vs placebo) but did not change significantly with placebo (7.40±0.20% to 7.30±0.20%). In one patient within one week of addition of dapagliflozin average glucose fell from 170 to 122 mg/dl while standard deviation was reduced from 71 to 58 (**Figure 2-diminished glycemic excursions)**. Body weight fell by1.9 +/- 0.54kg (p < 0.05 vs placebo). There was no additional hypoglycemia (blood glucose < 3.88 mmol/L; p = 0.52 vs placebo). In the dapagliflozin group, there were significant increases in the plasma concentrations of glucagon by 35% +/- 13% (p < 0.05), hormone-sensitive lipase by 29% +/- 11% (p < 0.05), free fatty acids by 74% +/- 32% (p < 0.05), acetoacetate by 67% +/- 34% (p < 0.05), and beta-hydroxybutyrate by 254% +/- 81% (p < 0.05). Urinary ketone levels also increased significantly (P < .05). None of these changes was observed in the placebo group. Two individuals in the dapagliflozin group developed ketoacidosis. Overall, addition of dapagliflozin to insulin and liraglutide in patients with TYPE 1 DIABETES resulted in a significant improvement in glycemia and in weight loss but tended to increase ketosis.

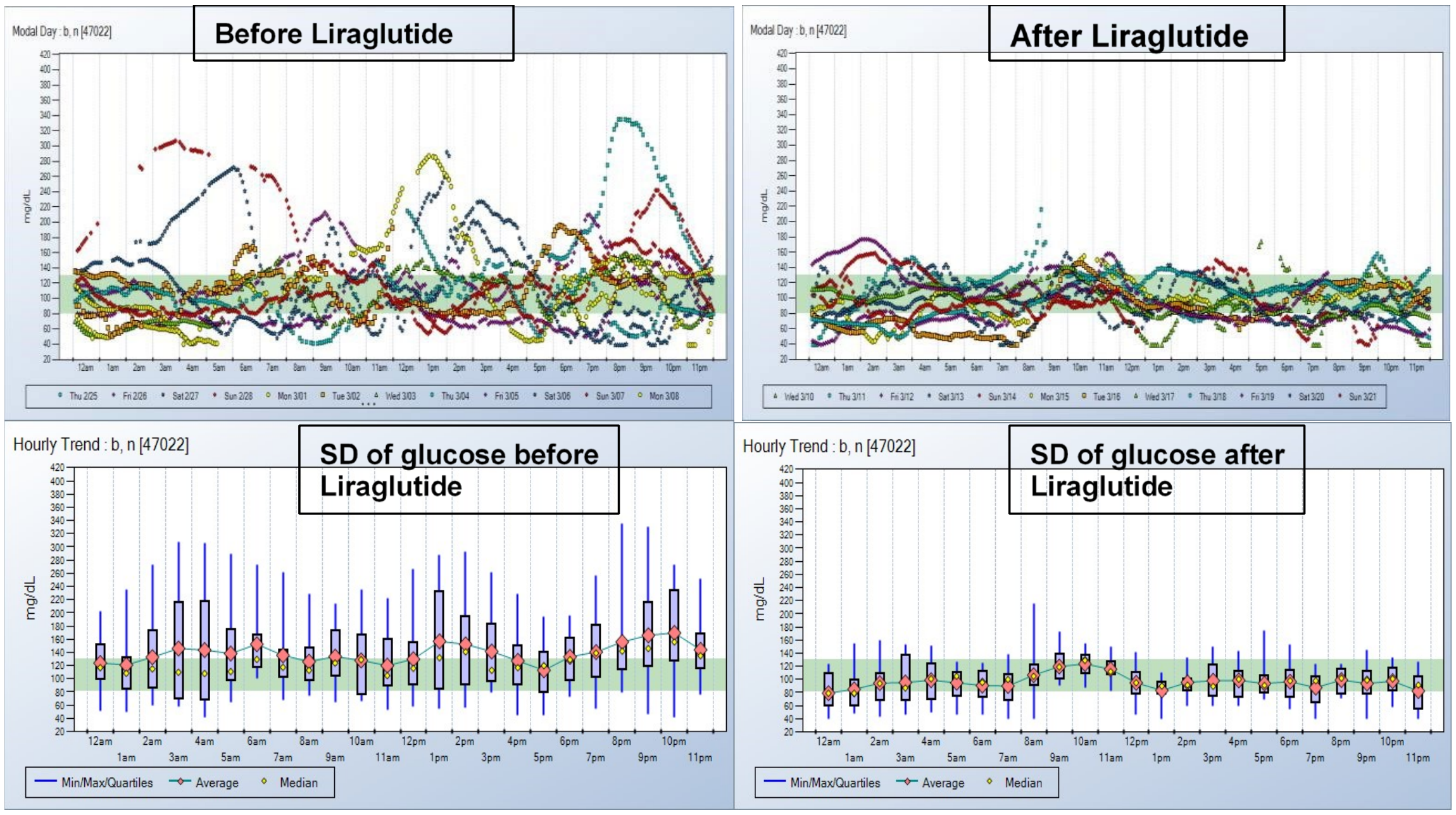

Figure 1: Weekly continuous glucose monitoring record of one patient prior to and following 1 week of liraglutide. Note the glycemic variability (SD) and the frequency of glucose concentrations within the target range prior to and following liraglutide

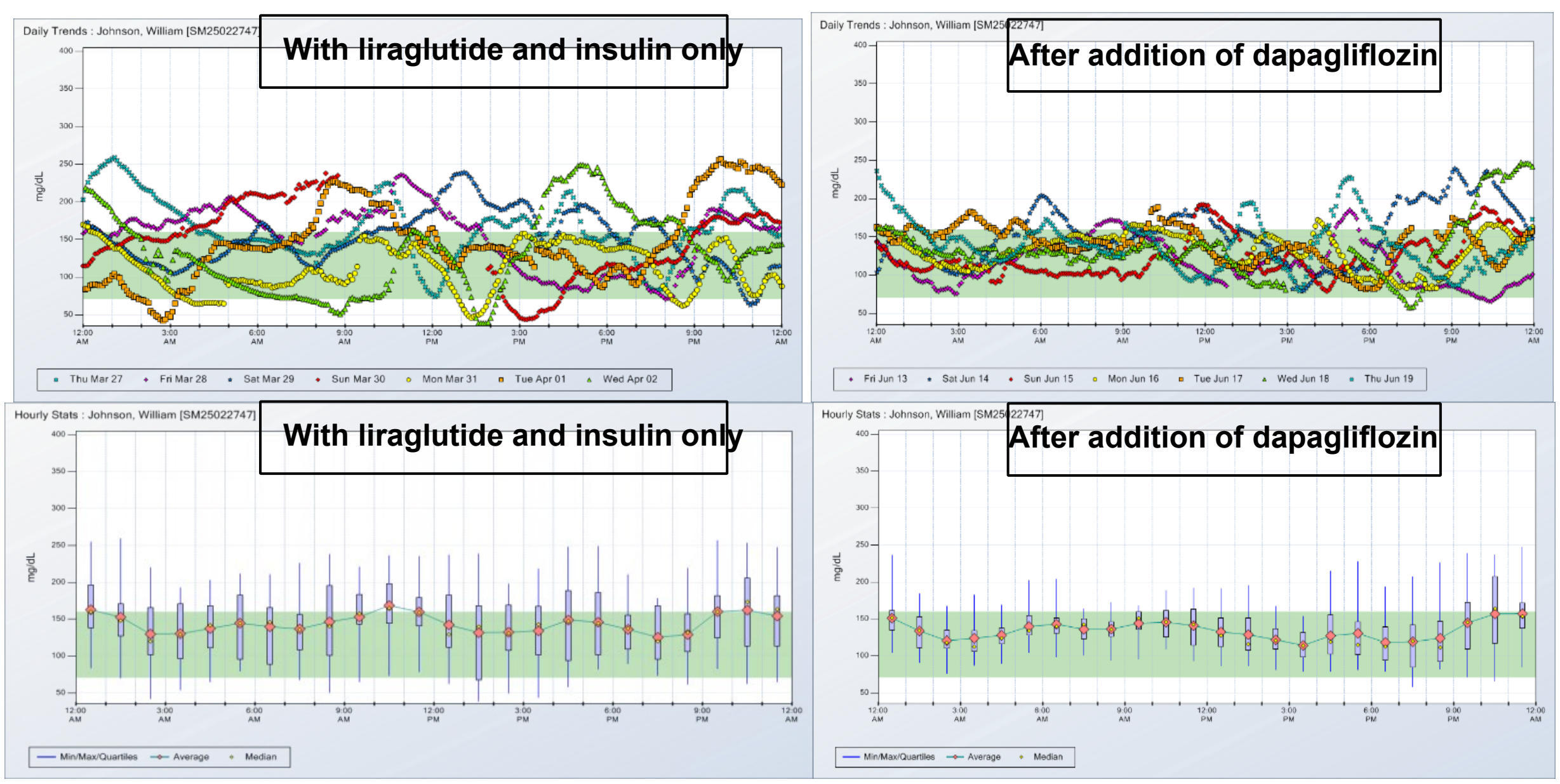

Figure 2: Weekly continuous glucose monitoring record of one patient prior to and following 8 weeks of the triple therapy combination with dapagliflozin and liraglutide. Note the glycemic variability (SD) (bottom) and the frequency of glucose concentrations within the target range prior to and following dapagliflozin addition.

3.2 Include complete citations or references.

Response:

1. Konrad K, Datz N, Engelsberger I, Grulich-Henn J, Hoertenhuber T, Knauth B, et al. Current use of metformin in addition to insulin in pediatric patients with type 1 diabetes mellitus: an analysis based on a large diabetes registry in Germany and Austria. *Pediatr Diabetes.* 2014.
2. Vella S, Buetow L, Royle P, Livingstone S, Colhoun HM, and Petrie JR. The use of metformin in type 1 diabetes: a systematic review of efficacy. *Diabetologia.* 2010;53(5):809-20.
3. Vella S, Buetow L, Royle P, Livingstone S, and Petrie JR. Metformin in type 1 diabetes reduces insulin requirements without significantly improving glycaemic control. Reply to Schatz H [letter]. *Diabetologia.* 2011;54(1):203-4.
4. Varanasi A, Bellini N, Rawal D, Vora M, Makdissi A, Dhindsa S, et al. Liraglutide as additional treatment for type 1 diabetes. *Eur J Endocrinol.* 2011;165(1):77-84.
5. Kuhadiya ND, Malik R, Bellini NJ, Patterson JL, Traina A, Makdissi A, et al. Liraglutide as additional treatment to insulin in obese patients with type 1 diabetes mellitus. *Endocrine practice : official journal of the American College of Endocrinology and the American Association of Clinical Endocrinologists.* 2013;19(6):963-7.
6. Kuhadiya ND, Dhindsa S, Ghanim H, Mehta A, Makdissi A, Batra M, et al. Addition of Liraglutide to Insulin in Patients With Type 1 Diabetes: A Randomized Placebo-Controlled Clinical Trial of 12 Weeks. *Diabetes Care.* 2016;39(6):1027-35.
7. Chaudhuri A, Ghanim H, Makdissi A, Green K, Abuaysheh S, Batra M, et al. Exenatide induces an increase in vasodilatory and a decrease in vasoconstrictive mediators. *Diabetes Obes Metab.* 2017;19(5):729-33.
8. Mathieu C, Zinman B, Hemmingsson JU, Woo V, Colman P, Christiansen E, et al. Efficacy and Safety of Liraglutide Added to Insulin Treatment in Type 1 Diabetes: The ADJUNCT ONE Treat-To-Target Randomized Trial. *Diabetes Care.* 2016;39(10):1702-10.
9. Ahren B, Hirsch IB, Pieber TR, Mathieu C, Gomez-Peralta F, Hansen TK, et al. Efficacy and Safety of Liraglutide Added to Capped Insulin Treatment in Subjects With Type 1 Diabetes: The ADJUNCT TWO Randomized Trial. *Diabetes Care.* 2016;39(10):1693-701.
10. Zinman B, Wanner C, Lachin JM, Fitchett D, Bluhmki E, Hantel S, et al. Empagliflozin, Cardiovascular Outcomes, and Mortality in Type 2 Diabetes. *N Engl J Med.* 2015;373(22):2117-28.
11. Petrie JR. SGLT2 inhibitors in type 1 diabetes: knocked down, but up again? *Lancet Diabetes Endocrinol.* 2017;5(11):841-3.
12. Mudaliar S, Armstrong DA, Mavian AA, O'Connor-Semmes R, Mydlow PK, Ye J, et al. Remogliflozin etabonate, a selective inhibitor of the sodium-glucose transporter 2, improves serum glucose profiles in type 1 diabetes. *Diabetes Care.* 2012;35(11):2198-200.
13. Kohan DE, Fioretto P, Tang W, List JF. Long-term study of patients with type 2 diabetes and moderate renal impairment shows that dapagliflozin reduces weight and blood pressure but does not improve glycemic control. Kidney Int. 2014 Apr;85(4):962-71.

14. Dandona P, Mathieu C, Phillip M, Hansen L, Griffen SC, Tschope D, et al. Efficacy and safety of dapagliflozin in patients with inadequately controlled type 1 diabetes (DEPICT-1): 24 week results from a multicentre, double-blind, phase 3, randomised controlled trial. *Lancet Diabetes Endocrinol.* 2017;5(11):864-76.
15. Garg SK, Henry RR, Banks P, Buse JB, Davies MJ, Fulcher GR, et al. Effects of Sotagliflozin Added to Insulin in Patients with Type 1 Diabetes. *N Engl J Med.* 2017;377(24):2337-48.
16. Kuhadiya ND, Ghanim H, Mehta A, Garg M, Khan S, Hejna J, et al. Dapagliflozin as Additional Treatment to Liraglutide and Insulin in Patients With Type 1 Diabetes. *J Clin Endocrinol Metab.* 2016;101(9):3506-15.

17. Andrews NC, and Faller DV. A rapid micropreparation technique for extraction of DNA-binding proteins from limiting numbers of mammalian cells. *Nucleic Acids Res.* 1991;19(9):2499.

4.0 Study Design

4.1 Describe and explain the study design (e.g. case-control, cross-sectional, ethnographic, experimental, interventional, longitudinal, observational).

Response: This will be a two-center, randomized, open-label then double-blind, parallel group, placebo and standard of care controlled prospective study in type 1 diabetes. One hundred and (114) patients will be randomized (open label, see below) into three groups (38 each) and will be either maintained on their regular insulin therapy for 12 months (standard therapy group) or started on semaglutide in addition to regular insulin treatment for six month (dual therapy and triple therapy groups). For those on insulin and semaglutide, after six months of stable treatment, dapagliflozin (triple therapy group) or placebo (dual therapy group) treatment will be initiated for an additional six months according to a second randomization (double-blind).

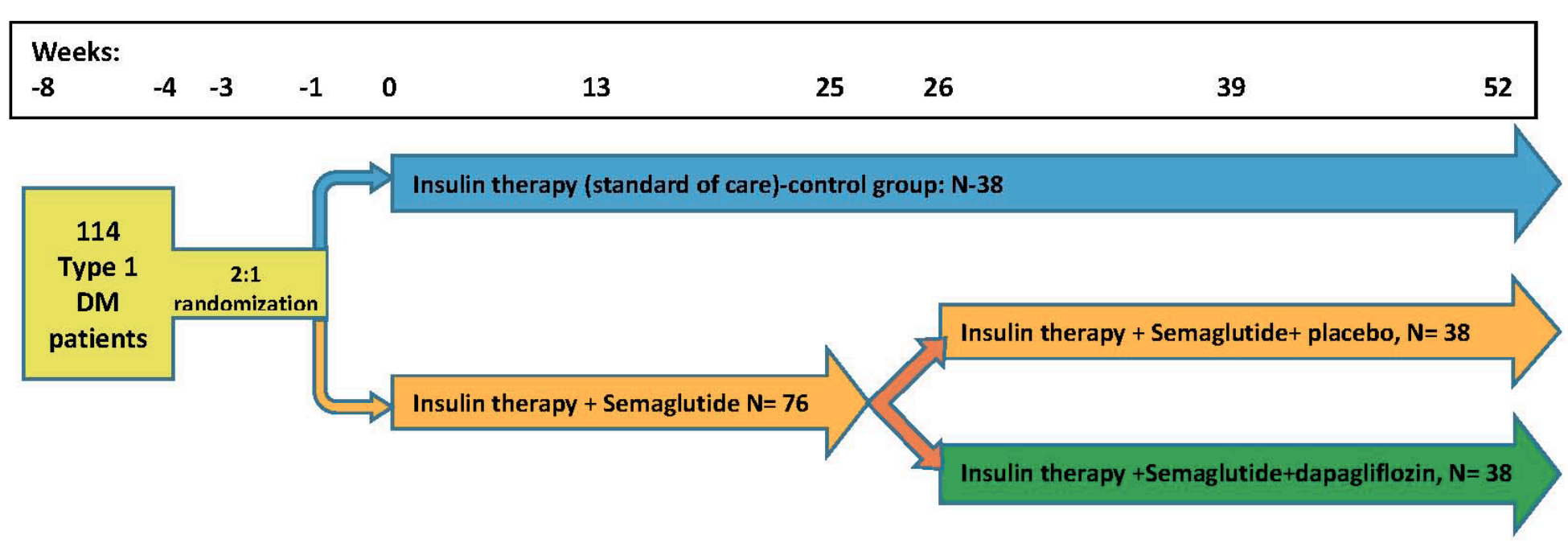

5.0 Local Number of Subjects

5.1 Indicate the total number of subjects that will be enrolled or records that will be reviewed locally.

Response: 57 patients will be enrolled locally with another 57 patients recruited at the University of Glasgow site: a total of 114.

5.2 If applicable, indicate how many subjects you expect to screen to reach your target sample (i.e. your screen failure rate).

Response: All screened and qualified patients will be enrolled and randomized

5.3 Justify the feasibility of recruiting the proposed number of eligible subjects within the anticipated recruitment period. For example, how many potential subjects do you have access to? What percentage of those potential subjects do you need to recruit?

Response: The Diabetes and Endocrinology Center of WNY is the largest Diabetes center in the WNY area, seeing between 60 to 100 type 1 diabetic patient every month. Therefore, the majority of recruited patients are our clinic patients. We also recruit a study participants through advertisement and other physician's references. These sources will suffice to recruit the needed number to subjects. The Glasgow center (Stobhill Ambulatory Care Hospital) reviews more than 300 patients per month.

6.0 Inclusion and Exclusion Criteria

*6.1 Describe the criteria that define who will be **included** in your final study sample.*

NOTE: This may be done in bullet point fashion.

Response:

Inclusion Criteria

1) Type 1 Diabetes for at least 1 year on staple use of continuous subcutaneous insulin infusion (CSII) or multiple daily (four or more) injections (MDI) of insulin for last 3 months.
2) C-peptide <0.23 nM
3) Minimum dose of insulin in Units/kg at entry: 0.5 U/kg for MDI and 0.4 U/kg for CSII
4) Regularly measuring blood sugars four or more times daily or use of CGM.
5) HbA1c between7.5-11% inclusive.
6) Well versed in CHO counting*
7) Age 18-70 years.
8) BMI ≥25 kg/m^2.

*In the opinion of the site Principal Investigator

*6.2 Describe the criteria that define who will be **excluded** from your final study sample.*

NOTE: This may be done in bullet point fashion.

Response:

Exclusion Criteria

1) Type 1 diabetes for less than 12 months, type 2 diabetes, chronic pancreatitis, MODY
2) Previous use of any agent other than insulin for treatment of diabetes in the last 3 months.
3) History of diabetic ketoacidosis (DKA) requiring medical intervention (e.g., emergency room visit and/or hospitalization) within 3 months prior to the screening visit
4) Patients on ketogenic or low (<50g/day) carbohydrate diet or who practice intermittent fasting.
5) Frequent episodes of severe hypoglycemia as defined by more than one episode requiring medical assistance, emergency care (paramedics or emergency room care), and/or glucagon therapy administered by a third-party individual within 1 month prior to the screening visit
6) Symptoms of poorly controlled diabetes that would preclude participation in this trial
7) Subjects on a commercial weight loss program with ongoing weight loss, or on an intensive exercise program
8) History of bariatric surgery or lap-band procedure within 12 months prior to screening
9) History of Addison's disease or chronic adrenal insufficiency
10) History of diabetes insipidus
11) Hepatic disease or cirrhosis
12) Aspartate Aminotransferase (AST) > 3X Upper limit of normal (ULN) *and/or* Alanine aminotransferase (ALT) > 3X ULN
13) Serum Total Bilirubin > 2X ULN unless exclusively caused by Gilbert's Syndrome
14) Hemoglobin < 11.0 g/dL (110 g/L) for men; hemoglobin < 10.0 g/dL (100 g/L) for women.
15) Coronary event or procedure (myocardial infarction, unstable angina, coronary artery bypass, surgery or coronary angioplasty) in the previous 3 months or patients with congestive heart failure.
16) ESRD on hemodialysis; and or e-GFR < 45 ml/min/1.73m^2
17) HIV or Hepatitis B/C positive status
18) Any other life-threatening, noncardiac disease
19) History of pancreatitis
20) Women who are pregnant or women of childbearing potential who are not using adequate contraception or who are breast feeding
21) Inability to give informed consent
22) History of gastroparesis
23) History of medullary thyroid carcinoma or MEN 2 syndrome
24) History of serious hypersensitivity reaction to these agents
25) Painful gallstones
26) Alcoholism
27) Hypertriglyceridemia (>500 mg/dl)

28) Recurrent genital mycotic infection.
29) Hypovolemic patients or with chronic renal insufficiency.
30) Patients with any malignancy except treated in situ malignancy and basal cell carcinoma of the skin
31) Unexplained hematuria
32) Patients with a history of diabetic retinopathy
33) Use of an investigational agent or therapeutic regimen within 30 days of study
34) Participation in any other concurrent interventional clinical trial

6.3 Indicate specifically whether you will include any of the following special populations in your study using the checkboxes below.

NOTE: Members of special populations may not be targeted for enrollment in your study unless you indicate this in your inclusion criteria.

Response: NA

☐ Adults unable to consent

☐ Individuals who are not yet adults (infants, children, teenagers)

☐ Pregnant women

☐ Prisoners

6.4 Indicate whether you will include non-English speaking individuals in your study. ***Provide justification if you will exclude non-English speaking individuals.***

In order to meet one of the primary ethical principles of equitable selection of subjects, non-English speaking individuals may ***not*** *be routinely excluded from research as a matter of convenience.*

In cases where the research is of therapeutic intent or is designed to investigate areas that would necessarily require certain populations who may not speak English, the researcher is required to make efforts to recruit and include non-English speaking individuals. However, there are studies in which it would be reasonable to limit subjects to those who speak English. Some examples include pilot studies, small unfunded studies with validated instruments not available in other languages, studies with numerous questionnaires, and some non-therapeutic studies which offer no direct benefit.

Response:

We have no non-English speaking patients in this population. We have patients that English is a second language, but they are able to read, write and understand it. This population is less than 10% of the total population. No benefit is being withheld from not participating in the study.

7.0 Vulnerable Populations

If the research involves special populations that are considered vulnerable, ***describe the safeguards included to protect their rights and welfare.***

NOTE: You should refer to the appropriate checklists, referenced below, to ensure you have provided adequate detail regarding safeguards and protections. You do not, however, need to provide these checklists to the IRB.

7.1 *For research that involves* ***pregnant women,*** *safeguards include:*
NOTE CHECKLIST: Pregnant Women (HRP-412)

Response: We will not be recruiting subjects from vulnerable populations

☒ **N/A**: This research does not involve pregnant women.

7.2 *For research that involves* ***neonates of uncertain viability or non-viable neonates,*** *safeguards include:*
NOTE CHECKLISTS: Non-Viable Neonates (HRP-413), or Neonates of Uncertain Viability (HRP-414)

Response:

☒ **N/A:** This research does not involve non-viable neonates or neonates of uncertain viability.

7.3 *For research that involves* ***prisoners****, safeguards include:*
NOTE CHECKLIST: Prisoners (HRP-415)

Response:

☒ **N/A:** This research does not involve prisoners.

7.4 *For research that involves* ***persons who have not attained the legal age for consent to treatments or procedures involved in the research ("children")****, safeguards include:*
NOTE CHECKLIST: Children (HRP-416)

Response:

☒ **N/A:** This research does not involve persons who have not attained the legal age for consent to treatments or procedures ("children").

7.5 *For research that involves* ***cognitively impaired adults****, safeguards include:*
NOTE CHECKLIST: Cognitively Impaired Adults (HRP-417)

Response:

☒ **N/A:** This research does not involve cognitively impaired adults.

7.6 *Consider if other specifically targeted populations such as students, employees of a specific firm, or educationally or economically disadvantaged persons are vulnerable.* ***Provide information regarding***

their safeguards and protections, including safeguards to eliminate coercion or undue influence.

Response: No specific populations or vulnerable groups will be targeted. All subjects enrolled in this study will be of legal adult consenting age with the ability to speak, read and interpret the English language. Patients will have the ability to speak with the research team regarding any questions or concern they have before signing the consent. Research team have the ability to evaluate patient's cognitive abilities through these interactions with patients. Patients are made aware that this study is voluntary and they are able to stop participating at any time they feel uncomfortable. Patients are not to be pressured into participating and their clinic standard of care will remain the same if they participate or choose not to participate.

8.0 Eligibility Screening

8.1 Describe ***screening procedures*** *for determining subjects' eligibility. Screening refers to determining if prospective participants meet inclusion and exclusion criteria.*

Include all relevant screening documents with your submission (e.g. screening protocol, script, questionnaire).

Response:

After obtaining partial HIPAA waiver, patient charts will be screened according to the study inclusion and exclusion criteria by our trained clinical staff and physicians. In additions, the study clinical team will evaluate their clinic patients for possible participation in this study according to the inclusion and exclusion criteria at the Diabetes and Endocrinology Center of WNY clinics. Physicians will speak to them about their interests in participating in research. If the patient agrees, their information will be given to the research coordinator to be contacted for further evaluation. The research coordinator will contact interested patients and those meeting the inclusion and exclusion criteria based on preliminary phone evaluation will be invited to participate in the study. All personal information will be kept confidential and locked in the coordinator office.

Prospective participants will be asked to read the consent and any questions they may have regarding the protocol will be answered. If the subject wants to participate in the study, they will be asked to sign the informed consent form. The subject's medical history and current medications will be obtained as well as their blood pressure and vitals. A physical examination will also be done. Blood and urine samples will be taken in order to evaluate HbA1c, CBC, CMP and liver and kidney functions and pregnancy status. Females of childbearing age, will have to be on two forms of birth control to prevent pregnancy. Patients meeting all the inclusion and exclusion criteria based on all screening tests will be enrolled in the study.

☐ **N/A:** There is no screening as part of this protocol.

9.0 Recruitment Methods

☐ **N/A:** This is a records review only, and subjects will not be recruited. NOTE: If you select this option, please make sure that all records review procedures and inclusion/exclusion screening are adequately described in other sections.

9.1 Describe when, where, and how potential subjects will be recruited.

NOTE: Recruitment refers to how you are identifying potential participants and introducing them to the study. Include specific methods you will use (e.g. searching charts for specific ICD code numbers, Research Participant Groups, posted advertisements, etc.).

Response:

Participants will be identified by prescreening clinical charts, patient doctor interaction at the time of their visits to the Diabetes Endocrinology Center of WNY Locations include:

1. 1020 Youngs Road, Williamsville NY 14221
2. 705 Maple Road, Williamsville NY 14221
3. 462 Grider Street, Buffalo NY 14215
4. 1000 Youngs Road, Suite 105, Williamsville NY 14221

Patients can be also referred by other physicians and providers

The remainder of the participants (57 patients) will be recruited from NHS Greater Glasgow and Clyde Diabetes clinics (Scotland, UK). Separate local IRB/ Ethical approval will be sought to cover this site (based on discussions with UBIRB director).

The study clinical team will evaluate their clinic patients for possible participation in this study according to the inclusion and exclusion criteria at the Diabetes and Endocrinology Center of WNY clinics. Patients that may qualify for the study are referred to the research team for further eligibility evolution. Patients meeting the inclusion and exclusion criteria based on preliminary phone evaluation will be invited to participate in the study.

Participants will also be identified by flyers, advertisements on Facebook and Craigslist and researchmatch.org. The study will also be put on UB's Study Information Portal (SIP) website. Along with this, a radio ad will be on the air of the radio station STAR102.5 at various times during the day during the month of mid January to mid February

9.2 Describe how you will protect the privacy interests of prospective subjects during the recruitment process.

NOTE: Privacy refers to an individual's right to control access to him or herself.

Response: Patient charts will be screened according to the study inclusion and exclusion criteria by our trained clinical staff and physicians. If the patient qualifies and is of consenting age, the physicians will speak to them about their interests in participating in research in a private room where no one else can hear

the conversation. If the patient agrees, their information will be given to the research coordinator to be contacted for further evaluation. No personal information will be shared without patient's approval and will not be used for any purpose except for this research. All personal information will be kept confidential and locked in the coordinator office.

9.3 Identify any materials that will be used to recruit subjects.

NOTE: Examples include scripts for telephone calls, in person announcements / presentations, email invitations.

For advertisements, include the final copy of printed advertisements with your submission. When advertisements are taped for broadcast, attach the final audio/video tape. NOTE: You may submit the wording of the advertisement prior to taping to ensure there will be no IRB-required revisions, provided the IRB also reviews and approves the final version.

Response: In addition to screening clinical charts, participations will be identified through; flyer advertisement, researchmatch.org, craigslist.com, and Facebook. The study will also be put on UB's Study Information Portal (SIP) website.

10.0 Procedures Involved

*10.1 Provide a description of **all research procedures or activities** being performed and when they are performed once a subject is screened and determined to be eligible. Provide as much detail as possible.*

NOTE: This should serve as a blueprint for your study and include enough detail so that another investigator could pick up your protocol and replicate the research. For studies that have multiple or complex visits or procedures, consider the addition of a schedule of events table in in your response.

Response:

Each patient will spend about 55 weeks in the trial comprising three weeks of monitoring to stabilize baselines and ensure adherence and commitment to participation before randomization into an intervention arm for an additional period of 52 weeks. Following a completed screening visit and meeting the inclusion and exclusion criteria, each patient will have a total of 16 study visits. At weeks 3, 5, 8, 16, 20, 29, 35, 44 and at 2 weeks after the end of the study, each patient will receive a phone call to document any adverse effects or other changes. There will be no long term follow-up following the end of the study.

Participants will be treated with either insulin only (control arm) for the whole duration of the study or with insulin and semaglutide weekly s.c. injection (titrated up to 1 mg or the maximum tolerated dose, see below) for the first six months then either dapagliflozin (Triple therapy arm) or placebo (dual therapy) daily will be added for an additional six months. Semaglutide will be started at a dose 0.25 mg once weekly for two weeks then increased to 0.5 mg dose. If well tolerated it will be increased to 1 mg once weekly at week 4. Dapagliflozin (or placebo) will be started at a dose of 5 mg daily at week 26 and if well tolerated increased after 1 week to 10 mg (2x5mg tablets) daily to ensure drug

tolerability and to avoid any hypoglycemia or hypotension. Patients who do not tolerate the increase in dosing of either medications will be maintained at the highest tolerated dose.

Treatments used

1- Insulin: All patients (114 patients) will continue to self-administer their prescribed insulin as part of their standard of care. Insulin dose will be titrated during the study to control blood glucose average between 90-180 mg/dl and to avoid hypoglycemia (blood sugar <70 mg/dl) or hyperglycemia (blood sugar >250 mg/dl). Among all patients, 38 patients will be randomized (open-label) to remain on insulin therapy only (standard therapy group) while the other 76 will start the investigational therapies.
2- Semaglutide: a once weekly a GLP-1 mimetic injection (pen) indicated for type 2 diabetes. Doses to be used: start at 0.25mg then increase to 0.5mg following 2 weeks and to 1mg weekly s.c. injection 2 weeks later. Semaglutide will be added to a background of insulin treatment for 12 months in 76 patients who will be randomized (double-blind) into 2 groups (dual and triple therapy groups) at the end of 26 weeks.
3- Farxiga (dapagliflozin): a once daily SGLT-2 inhibitor tablet indicated for type 2 diabetes. Doses used: starting with 5mg and increase after 1 week to 10mg oral administration daily. Dapagliflozin will be started according to the randomization (Interactive Web System, hosted by University of Glasgow) in 38 patients at 26 weeks as an additional treatment on a background of insulin and semaglutide therapy and end at 52 weeks (triple therapy group).
4- Placebo: dapagliflozin matching placebo (supplied by Astra Zeneca) will be started in 38 patients at 26 weeks as an additional treatment on a background of insulin and semaglutide therapy and end at 52 weeks (dual therapy group).

As shown in **Table 1,** fasting blood and urine samples will be obtained prior to drug administration (weeks 0) and at 13, 26, 39 and 52 weeks after the commencement of the study. Optional fat biopsies from the abdominal subcutaneous tissue will be collected at weeks 0, 26 and 52. Continuous Glucose Monitoring (Dexcom G6 with blinded receiver) will be performed for three 21-days periods on weeks -3 to 0, 23 to 26 and from 49 to 52 weeks. An optional 24hr blood pressure monitoring will be performed at baseline (week -1) and at 13, 25, 39 and 51 weeks in order to characterize the time course of antihypertensive effects, which may be early (i.e. independent of weight loss) as indicated by our preliminary data. High fat high caloric (HFHC) Meal challenges will be performed at baseline (week -1) and at 25 and 51 weeks with assessment of glucose excursions and postprandial glucagon (AUC) as well as postprandial inflammation and oxidative stress. Telephone calls will be conducted at weeks 3, 5, 8, 16, 20, 29, 35, 44 and at two weeks after the end of the study to document any side effects or any other patient's reported issues or comments.

Study Visits:

<u>Screening Visit (Screening Visit, Week -8 to -4):</u> Patients will be asked to read and understand the consent and any questions regarding the protocol will be answered. If they want to participate in the study, they will be asked to sign the informed consent form. Their medical history and current medications will be obtained as well as blood pressure and vitals. A physical examination will also be done. Blood samples will be taken in order to evaluate HbA1c, CBC, CMP, C-peptide, kidney and liver functions, ketone bodies, and urine for pregnancy status in females.

<u>Study Visit 1 (Week -3):</u> Participants who meet entry criteria will be scheduled to for their next visit at -3 week. Vitals will be taken including resting (min. 10 minutes) blood pressure measurement (will be repeated from same arm every visit). Insulin pump will be downloaded and/or CGM/fingerstick blood sugar will be reviewed and the appropriate insulin adjustments will be made by the investigator to target fasting blood glucose concentrations between 90-180 mg/dl if possible at all times without having additional hypoglycemia (less than 70 mg/dl). Blood sugars and ketone bodies will be measured via a fingerstick blood sample using glucose and ketones meter (Abbott Laboratories) and daily blood sugars collected and reviewed. Urine for pregnancy status and ketone bodies assessment. We will instruct patients on how to use and insert the Continuous Glucose Monitoring System (Dexcom G6 with blinded receiver). All subjects will be advised to monitor their capillary blood glucose by CGM or fingerstick at least 4 times a day (before meals and at bedtime) through-out the study and to wear their CGM constantly for next 20 day. Carb counting technique will be reviewed and any questions answered on how to use the pump or calculate insulin doses.

<u>Study Visit 2 (Week -1±2days):</u> Patients will be asked to come in the fasting state (no food or calorie intake for 10-12 hours). Their vitals will be taken. Blood sugars and ketone bodies will be measured via a CGM/fingerstick blood sample using glucose and ketones meter (Abbott Laboratories) to assess their glycemic control prior to meal challenge. If their blood sugar is below 70mg/dl or more than 250mg/dl the visit will be rescheduled for an alternative date. Insulin pump will be downloaded and/or CGM/fingerstick blood sugar will be reviewed and insulin adjustment will be made if necessary. An IV port will be placed in order to obtain blood samples throughout the duration of the visit. Patients will undergo a meal challenge test (described below). At the end of the meal test, they will be asked to wear a 24 hour blood pressure monitor (optional). A 24 hr urine container will be dispensed to collect a 24 hour urine sample to bring in at the Week 0 visit. Daily Glucose logs will be reviewed.

<u>Study visit 3 (Week 0±2days):</u> Patients will be asked to come in the fasting state (no food or calorie intake for 10-12 hours). Vitals will be taken. Blood sugars and ketone bodies will be measured via a CGM/fingerstick blood sample using glucose and ketones meter (Abbott Laboratories) and daily blood sugars collected and reviewed. The 24 hour urine container will be collected. A fasting blood sample will be taken (45 mL) to evaluate study endpoints, CBC, CMP, HbA1c, and fructosamine. A urine sample will be collected to evaluate ketone bodies and, in females, pregnancy status. Insulin pump will be downloaded and/or CGM/fingerstick blood sugar will be reviewed and the appropriate insulin adjustments will be made by the investigator. In general, insulin dose adjustments should

not be >10% from current total insulin dose or >20% from baseline (week 0) total insulin dose. They will discontinue use of the study CGM. An optional fat biopsy will be performed. We will ask them to fill out quality of life questionnaires. Study drug will be dispensed depending on what group they are randomized to. This includes either (1) staying on the insulin regimen given by their doctor, or (2) a combination of their current insulin regimen and a once daily injection of semaglutide. Semaglutide will be started on site at the 0.25 mg dose. The 24 hr blood pressure monitor will be collected.

Study Visit 4 (Week 1±2days): Vitals will be taken. Blood sugars and ketone bodies will be measured via a CGM/fingerstick blood sample using glucose and ketones meter (Abbott Laboratories) and daily blood sugars collected and reviewed. Insulin dose will be adjusted if needed as in visit 3. At any time, if HbA1c is <7%, insulin dose adjustment should not be >5%. Semaglutide 0.25mg will be injected on site. Participants will be instructed to call their research center or their doctor directly in case of any problem or untoward side effects. They will be specifically asked to call if they have unmanaged hypoglycemia (blood sugar <70 mg/dl) or hyperglycemia (blood sugar >250 mg/dl) or if occurring in more than one occasion.

Study Visit 5 (Week 2±2days): Vitals will be taken. Blood sugars and ketone bodies will be measured via a CGM/fingerstick blood sample using glucose and ketones meter (Abbott Laboratories) and daily blood sugars collected and reviewed. Insulin dose will be adjusted if needed as in visit 3. Semaglutide 0.5mg dose will be injected on site. If they are experiencing mild nausea, they will be continued on the 0.25 mg dose for another week. Supply of the drug will be given to last until the next visit. Patients will be advised to increase the study dose to 0.5 mg a day if they are ok.

Study Visit 6 (Week 4±1 week): Patients will be asked to bring in used study pens for drug accountability (to be recorded in eCRF). Vitals will be taken. Blood sugars and ketone bodies will be measured via a CGM/fingerstick blood sample using glucose and ketones meter (Abbott Laboratories) and daily blood sugars collected and reviewed. Insulin dose will be adjusted if needed. Supply of the drug will be given to last until the next visit. A urine sample will be collected to evaluate ketone bodies and, in females, pregnancy status. Patients will be advised to increase the study dose to 1.0 mg a day. If they experience mild nausea, they will be continued on the 0.5 mg dose for another week. At all times, patients will be maintained at the maximum tolerated dose. A 24 hr urine container will be dispensed to collect a 24 hour urine sample to bring in at the Week 13 visit.

Study Visit 7 (Week 13±1 week): Patients will be asked to come in the fasting state (10-12 hours fasting), and to bring in your used study pens for drug accountability. Blood sugars and ketone bodies will be measured via a CGM/fingerstick blood sample using glucose and ketones meter (Abbott Laboratories) and daily blood sugars collected and reviewed. Insulin dose will be adjusted if needed as in. Supply of the drug will be given to last until visit 26. Vitals will be taken. A urine sample will be collected to evaluate ketone bodies and, in females, pregnancy status. The 24 hour urine container will be collected. A fasting blood sample will be taken (45 mL) to evaluate study endpoints, CBC, CMP,

HbA1c, and fructosamine. Patients who agreed to participate in the24 hour blood pressure monitoring will be asked to wear it for the next 24 hr.

Study Visit 8 (Week 23±1 week): Patients will be asked to bring in used study pens for drug accountability. Vitals will be taken. Blood sugars and ketone bodies will be measured via a CGM/fingerstick blood sample using glucose and ketones meter (Abbott Laboratories) and daily blood sugars collected and reviewed. A urine sample will be collected to evaluate ketone bodies and, in females, pregnancy status. Insulin dose will be adjusted if needed. All subjects will be reminded to monitor their capillary blood glucose by fingerstick at least 4 times a day (before meals and at bedtime) through-out the study. CGM will be inserted and patients asked to wear their CGM constantly for next 20 day.

Study visit 9 (Week 25±1 week): Patients will be asked to come in the fasting state, and to bring in used study pens for drug accountability. Vitals will be taken. A urine sample will be collected to evaluate ketone bodies. Blood sugars and ketone bodies will be measured via a CGM/fingerstick blood sample using glucose and ketones meter (Abbott Laboratories) and daily blood sugars collected and reviewed. Insulin dose will be adjusted if needed. HbA1c will be drawn. Supply of the drug will be given to last until the next visit. A 24 hr urine container will be dispensed to collect a 24 hour urine sample to bring in at the Week 26 visit. A HFHC Meal challenges will be performed as in visit 2. Patients who agreed to participate will be asked to wear a 24 hour blood pressure monitoring for the next 24 hr.
Participants in the semaglutide group will be provided supplies for urine ketones measurement (KetoStix) and blood ketones meter and strips. Research coordinator will instruct the patients on the use of meter and reading of the measuring strips. Patients will be instructed to start measuring urinary and blood ketones every morning for three consecutive days prior to next visit (starting dapagliflozin treatment) and to report results at the time of their next visit.

Study visit 10 (Week 26±1 week): Patients will be asked to come in the fasting state, and to bring in used study pens for drug accountability. Previous 3 days blood and urine ketones levels will be reviewed. Patients with normal fasting urine (<+, or < small) or blood ketone (<0.6 mmol) will proceed to next treatment phase included in this visit. Patients who report higher ketone values in at least 1 out of the 3 days will have their glucose levels, insulin dose, carb intake reviewed and managed as necessary. They will be rescheduled for next week (7±1 day) and asked to repeat the 3 days testing before next visit.
Vitals will be taken. Blood sugars and ketone bodies will be reviewed via a CGM/fingerstick blood sample using glucose and ketones meter (Abbott Laboratories) and daily blood sugars collected and reviewed. Insulin dose will be adjusted if needed. In general, insulin dose adjustments should **NOT** be >10% from current total insulin dose (or 5% if HbA1c is <7%) or >20% from baseline total insulin dose. The adjusted total insulin dose in this visit will serve as the baseline total insulin dose for the remaining 6 months of the study and future limits of dose adjustments will be based on this new baseline. The CGM will be collected. A urine sample will be collected to evaluate ketone bodies and, in females, pregnancy status. The 24 hour urine container will be collected. A fasting blood sample will be taken (45 mL) to evaluate study endpoints, CBC, CMP, C-peptide, and

Fructosamine. An optional fat biopsy will be performed. We will ask patients to fill out quality of life questionnaires. At this point, those patients randomized to receive semaglutide, they will now be randomized to receive an actual dapagliflozin pill or a placebo pill that looks like dapagliflozin. If they are randomized to receive actual dapagliflozin, they will start at the 5 mg dose for one week. Supply of the study drug will be given to last until week 27.
All patients will be asked to perform a daily (preferably early morning or before bedtime) urinary test of ketone bodies using KetoStix and record all outcomes until the end of the study. Also, they will be provided with additional blood ketone strips to measure blood ketones when needed. If results of these daily scheduled (i.e. not done due to symptoms) urine ketone measurements, are small or higher (+1 or more), they will be instructed to measure blood ketones and to repeat both measurements after 60 min. If consecutive measurements show moderate or higher ketones in urine or blood ketones >0.6mM, the patient will be instructed to call the research center or their doctor directly. For any abnormal ketone levels taken due to symptoms, the DKA consensus guidelines (Danne T et al., Diabetes Care. 2019 Feb 6) are to be followed instead (as in section 21).
Participants will be instructed to call their research center or their doctor directly in case of any problem or untoward side effects. They will be specifically asked to call if they have unmanaged hypoglycemia (blood sugar <70 mg/dl) or hyperglycemia (blood sugar >250 mg/dl) or if occurring in more than one occasion. Participants will be instructed to remain hydrated and report any genital mycotic or urinary tract infections.

Study visit 11 (Week 27±2 days): Patients will be asked to bring in used study pens for drug accountability (recorded in eCRF). Vitals will be taken. Daily blood sugars and urinary ketones measurements using KetoStix will be collected and reviewed. Blood sugars and ketone bodies will be measured via a CGM/fingerstick blood sample using glucose and ketones meter (Abbott Laboratories). A urine sample will be collected to evaluate urine ketone level. Insulin dose will be adjusted if needed. In general, insulin dose adjustments should not be >10% from current total insulin dose (or 5% if HbA1c is <7%) or >20% from NEW baseline total insulin dose established at visit 10. If at any time during the study total insulin dose reduction is >20% and total insulin dose in <0.40 U/Kg, additional monitoring protocol (see later) will be implemented. Supply of the drug will be given to last until week 39. If patients are receiving actual dapagliflozin, they will now start at the 10 mg dose for the remainder of the study.

Study Visit 12 (Week 31±1 week): Patients will be asked to bring in used study pens and tablets for drug accountability. Vitals will be taken. Daily blood sugars and urinary ketones measurements using KetoStix will be collected and reviewed. Blood sugars and ketone bodies will be measured via a CGM/fingerstick blood sample using glucose and ketones meter (Abbott Laboratories). A urine sample will be collected to evaluate ketone bodies and, in females, pregnancy status. Insulin dose will be adjusted if needed as in visit 11. A blood sample will be taken (15 mL) to evaluate CBC and CMP. A 24 hr urine container will be dispensed to collect a 24 hour urine sample to bring in at the Week 39 visit.

Study visit 13 (Week 39±1 week): Patients will be asked to come in the fasting state, and to bring in used study pens and tablets for drug accountability. Vitals will be taken. Daily

blood sugars and urinary ketones measurements using KetoStix will be collected and reviewed. Blood sugars and ketone bodies will be measured via a CGM/fingerstick blood sample using glucose and ketones meter (Abbott Laboratories). Insulin dose will be adjusted if needed as in visit 11. Supply of the drug will be given to last until the end of the study. A urine sample will be collected to evaluate ketone bodies and, in females, pregnancy status. The 24 hour urine container will be collected. A fasting blood sample will be taken (45 mL) to evaluate study endpoints, CBC, CMP, HbA1c, and Fructosamine. Patients who have provided consent will be asked to wear a 24 hour blood pressure monitor.

Study visit 14 (Week 49±1 week): Patients will be asked to bring in used study pens and tablets for drug accountability. Vitals will be taken. Daily blood sugars and urinary ketones measurements using KetoStix will be collected and reviewed. Blood sugars and ketone bodies will be measured via a CGM/fingerstick blood sample using glucose and ketones meter (Abbott Laboratories). A urine sample will be collected to evaluate ketone bodies and, in females, pregnancy status. Insulin dose will be adjusted if needed as in visit 11. All subjects will be reminded to monitor their capillary blood glucose by fingerstick at least 4 times a day (before meals and at bedtime) through-out the study. CGM will be inserted and patients asked to wear their CGM constantly for the next 20 days.

Study visit 15 (Week 51±1 week): Patients will be asked to come in the fasting state, and to bring in used study pens and tablets for drug accountability. Vitals will be taken. Daily blood sugars and urinary ketones measurements using KetoStix will be collected and reviewed. Blood sugars and ketone bodies will be measured via a CGM/fingerstick blood sample using glucose and ketones meter (Abbott Laboratories). A urine sample will be collected to evaluate urine ketone levels. Insulin dose will be adjusted if needed as in visit 11. A 24 hr urine container will be dispensed to collect a 24 hour urine sample to bring in at the Week 52 visit. A HFHC Meal challenges will be performed as in visit 2. Patients will be asked to wear the optional 24 hour blood pressure monitor.

Study visit 16 (Week 52±2 days): Patients will be asked to come in the fasting state, and to bring in used study pens and tablets for drug accountability. Vitals will be taken. Daily blood sugars and urinary ketones measurements using KetoStix will be collected and reviewed. Blood sugars and ketone bodies will be measured via a CGM/fingerstick blood sample using glucose and ketones meter (Abbott Laboratories). Insulin dose will be adjusted if needed. The CGM will be collected. An optional fat biopsy will be performed. A urine sample will be collected to evaluate ketone bodies and, in females, pregnancy status. The 24 hour urine container will be collected. A fasting blood sample will be taken (45 mL) to evaluate study endpoints, CBC, CMP, HbA1c, C-peptide, and Fructosamine. We will ask the patients to fill out quality of life questionnaires. The patient will receive a physical. After this visit, they will be discharged from the study.
Safety phone calls will be conducted at weeks 3, 5, 8, 16, 20, 29, 35, 44 and at two weeks after the end of the study to document any side effects or other changes.

Table 1: Schedule of visits and testing procedures.

Visit #	-	1	2	3	4	5	6	7	8	9	10	11	12	13	14	15	16
Week#(±2-7 days as per text)	-8 to -4	-3	-1	0	1	2	4	13	23	25	26	27	31	39	49	51	52
Study Procedures	Screening	Run-in period		Randomization, start semaglutide							Randomize/Start dapagliflozin/placebo						End of study
History	X																
Physical	X																X
Vitals	X	X	X	X	X	X	X	X	X	X	X	X	X	X	X	X	X
CBC	X			X				X			X		X	X			X
CMP	X			X				X			X		X	X			X
C-Peptide	X										X						X
Kidney and liver profiles	X			X				X			X		X	X			X
Pregnancy test	X	X		X			X	X	X		X		X	X	X		X
Study drug titration						X	X					X					
Dispense medications				X	X	X	X	X			X	X		X			
HbA1c	X			X				X		X				X			X
Fructosamine				X				X			X			X			X
24 hour BP (optional)			X					X		X				X		X	
CGM		X	X						X	X					X	X	
Daily Fingerstick glucose review		X	X	X	X	X	X	X	X	X	X	X	X	X	X	X	X
Daily urinary ketones review											X	X	X	X	X	X	X
Insulin dose review		X	X	X	X	X	X	X	X	X	X	X	X	X	X	X	X
Ketone bodies (blood/urine)	X	X	X	X	X	X	X	X	X	X	X	X	X	X	X	X	X
Adverse Event reporting		X	X	X	X	X	X	X	X	X	X	X	X	X	X	X	X
Meal Challenge			X							X						X	
Blood endpoints				X				X			X			X			X
Fat biopsy (optional)				X							X						X
24 hr Urine				X				X			X			X			X
Quality of life questionnaires				X							X						X

Drug Blinding:
The randomizations will be performed using an Interactive Web System, hosted by University of Glasgow. One hundred and fourteen (114) patients will be randomized as follows: participants will first be randomized to semaglutide plus usual insulin therapy or insulin therapy only (no placebo for semaglutide or other placebo injection will be provided), in a 2:1 ratio. Those who remain on semaglutide at six months will then be randomized in a 1:1 ratio to receive dapagliflozin or matched placebo. Each randomization will be stratified by study site and the second randomization will also include HbA1c stratification. The randomization schedules will be generated by a statistician with no other involvement in the trial, using the method of randomized permuted blocks. Only those staff responsible for the maintenance of the randomization system will have access to the randomization sequences during the trial. The first randomization (which will be revealed to the researcher at the point of randomization) will use random block sizes to reduce predictability. Researchers at study sites will have no knowledge of the next allocation to be issued. The second randomization (which will be double-blind) will use a different. At the point of randomization, the researcher will be given a medication pack ID number, but will not know whether that medication pack contains Dapagliflozin or placebo. For each randomization, a web-based randomization system (developed and maintained by the Robertson Centre for Biostatistics) will be used. The system will include an emergency unblinding facility for the dapagliflozin/placebo randomization
Astra Zeneca Inc. will supply dapagliflozin and matching placebo tablets in unlabeled and identical packaging; semaglutide pens will be obtained commercially and will be used in an open label fashion.

Breaking of the study blind will be performed only: (i) for SUSARs; and, (ii) where knowledge of the treatment is considered by local health personnel absolutely necessary for further management of the patient. A central unblinding facility based at the Robertson Centre for Biostatistics, University of Glasgow, will be available by telephone. Notification of all unblinding will be sent to the Chief Investigator

During the course of the trial there will be no attempt to identify group assignment for any participant (except for unblinding in the case of safety concerns) and the investigators will execute the protocol in the manner it was approved independent of the group assignment.

Drugs will be stored in a locked cabinet or locked temperature controlled refrigerator at 4C at the research facility of the Diabetes and Endocrinology Center of WNY. IND application for use of all drugs (dapagliflozin and semaglutide) in type 1 diabetes will be submitted to FDA and no study procedure will start before all local and federal approvals are obtained.

Clinical and Laboratory Procedures

1) **CMP, CBC, HbA1c, fructosamine, glucose, glucagon, GLP-1 and FFA measurements:** Blood CMP, CBC, HbA1c, fructosamine and glucose will be measured through Quest diagnostic (US site) and NHS labs (UK site). Free fatty acid levels will be measured by a colorimetric assay (Wako, Richmond, VA). GLP-1 and

glucagon will be measured from samples collected in protease and DPP-IV inhibitors by ELISA kits from Millipore.

2) **Meal Challenge test:** In order to assess the postprandial changes induced by dapagliflozin, semaglutide or the combination of both compared to insulin only, a meal challenge will be performed before starting treatments, at six months and at end of study using a 910 Calorie High fat High carbohydrate meal (HFHC) as in several of our previous studies (HFHC meal includes an egg muffin sandwich, a sausage muffin sandwich and two hash browns which contain 88g carbohydrate, 51 g fat (33% saturated) and 34 g protein). Participants will be asked to attend fasting prior to breakfast insulin bolus; basal insulin will continue during the duration of the meal, however patients will not bolus until the 3 hour mark unless blood glucose values reach above 400 mg/dL. Sequential blood samples will be obtained at 0, 15, 30, 45, 60, 90, 120, 150 and 180 min after consuming the meal. Samples at 15, 30, 45, 90 and 150 min will be 5ml while those at 0, 60, 120 and 180min will be 30 ml (total volume=145 ml). Spot urine will be collected at baseline, 1 and 3hr. Blood will be collected from an indwelling intravenous canula in a superficial forearm vein.
3) **Optional 24 hour Ambulatory BP monitoring:** Participants will be instructed on the use of 24 hour ambulatory BP monitoring device. This device will measure BP and pulse every 30 minutes for 24 hours. In addition to the scheduled measurements there will be an option for additional manual measurements if systolic blood pressure is >160 or <90 mmHg.
4) **Optional Fat aspiration procedure:** Abdominal subcutaneous fat tissue aspiration will be performed at a 10 cm distance from umbilicus. Participants will be asked not to take aspirin or NSAIDS in the previous 72 hours - if they have, the procedure will not be done. Skin will be prepared with povidone-iodine (Betadine) and alcohol. A sterile drape will be placed around the appropriate area. 3cc of 1% lidocaine will then be administered subcutaneously (the dose will not exceed 4.5mg/kg body weight). After adequate anesthesia has been achieved, aspiration of fat tissue will then be with a 3-holed canula (Tulip Instrumentation, length: 15cm, diameter: 2.1mm) fixed to a 10mL syringe. After getting adequate fat tissue (500mg-3g), the puncture site will be pressed for at least ten minutes before the patient rises up from supine position (to minimize bruising). Participants will then be discharged home. Adipose tissue will be centrifuged to remove blood and fluid contaminants. Upper adipose tissue will be collected into a separate sterile tube, washed twice with cold sterile Phosphate Buffered Saline (PBS) and centrifuged to remove the PBS. The adipose tissue sample will be weighed and approximately 500 mg transferred to a separate tube for analysis. Total RNA, nuclear extracts and total cell lysates will be prepared.
5) **Questionnaires:** Participants will be asked to complete the standard Diabetes Specific Quality of Life Scale (DSQOLS) and Problem Areas In Diabetes survey (PAID). Questionnaires attached at the end of this plan.
6) **MNC isolation (for future ancillary studies):** Blood samples will be collected in Na-EDTA as an anticoagulant. Three and a half mL of anticoagulated blood sample are carefully layered over 3.5 mL of PMN medium (Cedarlane Laboratories, Hornby, ON) and then centrifuged. At the end of this process, two bands separate out at the top of the RBC pellet. The top band consists of MNC, while the bottom consists of PMN. The

MNC band is harvested and washed twice with Hank's balanced salt solution (HBSS). This method provides yields greater than 95% pure PMN and MNC suspensions.

7) **NFκB DNA binding activity (for future ancillary studies):** Nuclear NFκB DNA binding activity is measured by EMSA. Nuclear extract is prepared from MNC and adipose tissue by high salt extraction as described by Andrews et al (17). The specificity of the bands is confirmed by supershifting these bands with specific antibodies against Rel-A (p65) and p50 (Santa Cruz Biotechnology, CA) and by competition with cold oligonucleotides. DNA binding activity of NFκB will be adjusted to Oct1 DNA binding activity to correct for any variable.
8) **Plasma concentrations of inflammatory mediators (for future ancillary studies):** Concentrations of TNFα, IL-1β, IL-18, adiponectin, IL-1RA will be measured in plasma using ELISA kits (R&D Systems, MN). CRP and SAA concentrations will be measured using an ELISA kit from Alpha Diagnostics International Inc. (San Antonio, TX).
9) **Plasma vasodilators and vasoconstrictors (for future ancillary studies):** Plasma ANP, NT-proBNP, b-NP, cGMP, cAMP, angiotensinogen, renin and angiotensin II concentrations will be measured by commercially available ELISA kits.
10) **15-isoprostane F_{2t}** (also known as 8-epi-PGF2α or 8-iso-PGF2α) **(for future ancillary studies):** will be measured in urine samples using an ELISA kit from Oxford Biomedical Research (Oxford, MI).
11) **Quantification of mRNA in MNC and adipose tissue (for future ancillary studies).** Total RNA is isolated from MNC and adipose tissue using commercially available RNAqueous®-4PCR Kit (Ambion, Austin, TX). The quality and quantity of the isolated RNA is determined before using the RNA. Real Time RT-PCR is performed using Cepheid Smart Cycler (Sunnyvale, CA) in which 2 µL cDNA, 10 µL Sybergreen Master mix (Qiagen, CA) along with 0.5 µL of 20 µM gene specific primers for IL-1β, IL-RA, TLR-4, TNFα, IL-12, SOCS-3 and JNK-1 (Life Technologies, MD) are used. The specificity and the size of the PCR products are tested by adding a melt curve at the end of the amplifications and by running it on a 2% agarose gel. All values are normalized to 18S expression and β-actin.

10.2 Describe what data will be collected.

NOTE: For studies with multiple data collection points or long-term follow up, consider the addition of a schedule or table in your response.

Response:

Data will be collected as described in table 1 above. The following aspects of the study will be performed and data collected and reported by patients:

1- A CGM device will be provided for use for 3 20-days periods. Detailed instructions on changing the sensor at 10 days will be provided. Patients will be instructed to wear their CGM constantly during the assigned study periods. They will be asked to keep a diary of their food intake to measure their carbohydrate intake during the CGM period. Insulin doses will be titrated aiming for blood glucose between 90-180 mg/dl while avoiding hypoglycemia (<70 mg/dl) or hyperglycemia (>250 mg/dl).

2- Participants who agree to participate in the 24-hr ambulatory blood pressure monitoring will be provided with a 24-hour ambulatory BP monitor to be worn for 24 hours during week -1, 13, 25, 39 and 51.
3- All participants will be asked to monitor capillary blood glucose by finger stick before each meal and at bedtime.
4- For the entire duration of the study, the patients will maintain a diary to record any hypoglycemia and other untoward side effects e.g. nausea, change in appetite.
5- Starting at the 6 months mark, participants will be asked to perform a daily urinary test of ketone bodies using KetoStix and record all outcomes. If results are moderate to large, they will be instructed to repeat the test and to call the research center or their doctor directly if results are confirmed. Patients with dual glucose and ketones meters will be instructed to record and report their blood ketones as well. If blood ketones are above 0.6mM, they should repeat the test and contact the research center of their doctor if levels are confirmed.

10.3 List any instruments or measurement tools used to collect data (e.g. questionnaire, interview guide, validated instrument, data collection form).

Include copies of these documents with your submission.

Response: Source documents will be used to collect patient information. The CGM or insulin pump will be used to collect glucose and insulin data. Glucose and ketone meter will be used to measure glucose and ketone in blood. KetoStix for measuring ketones in urine. Blood pressure monitor will be used for 24 hour blood pressure reading. Glucose, food and urinary ketones diaries will be provided for patients to record their results.

10.4 Describe any source records that will be used to collect data about subjects (e.g. school records, electronic medical records).

Response: electronic medical records and research files.

10.5 Indicate whether or not ***individual*** *subject results, such as results of investigational diagnostic tests, genetic tests, or incidental findings will be shared with subjects or others (e.g., the subject's primary care physician) and if so, describe how these will be shared.*

Response: Individual participant lab results will be disclosed to the participant upon their request. If the participant requests documentation be shared with another physician, physician office or hospital the participant must come to the research center to collect said documentation or the documentation can be mailed to their given home address.

Patients will be assigned a unique study number. All identifiable personal information will be stored locally in locked filing cabinets. Data entered on the

electronic Case Report Form will only be identified by the unique study number. Thus, no personal information will be shared with the data center (Robertson Centre for Biostatistics, University of Glasgow, UK).

10.6 Indicate whether or not ***study*** *results will be shared with subjects or others, and if so, describe how these will be shared.*

Response: Not Applicable. Study results will not be shared with the subjects. However, unidentifiable study results could be published in the form of a manuscript or abstract and will be reported to JDRF, Astra Zeneca, and to clinicaltrials.gov and other regulatory agencies including FDA.

11.0 Study Timelines

11.1 Describe the anticipated duration needed to enroll all study subjects.

Response: Accrual is expected to be at the rate of 2 subjects/month/site, thus we should complete enrollment of 57 patients/site in approximately 26-30 months. We expect to complete all study clinical activities in 48 months.

11.2 Describe the duration of an individual subject's participation in the study. Include length of study visits, and overall study follow-up time.

Response: 55 Weeks. Study visits will be 60-90 minutes long except for visits 2, 9 and 15 which will be about 4hr long each

11.3 Describe the estimated duration for the investigators to complete this study (i.e. all data is collected and all analyses have been completed).

Response: 60 Months

12.0 Setting

12.1 Describe all facilities/sites where you will be conducting research procedures. Include a description of the security and privacy of the facilities (e.g. locked facility, limited access, privacy barriers). Facility, department, and type of room are relevant. Do not abbreviate facility names.

NOTE: Examples of acceptable response may be: "A classroom setting in the Department of Psychology equipped with a computer with relevant survey administration software," "The angiogram suite at Buffalo General Medical Center, a fully accredited tertiary care institution within New York State with badge access," or, "Community Center meeting hall."

Response:

1) Research will be conducted at the Diabetes Endocrinology Research Center of WNY, located at *1000 Youngs Road, Suite 105, Williamsville NY 14221* and at the CTRC located in 875 Ellicott St. Buffalo NY14203.The Diabetes Research Center has facilities and exam rooms available for insulin pump download, CGM device download, meal and infusion studies and presence of study coordinator and registered nurse for data collection and blood work at all times. One of the investigators will be available at all times to address patients' related issues. CTRC location is a fully equipped laboratory with Equipment include ultra-low freezers for sample storage, centrifuges, microscopes for sample preparation, infusion pumps, ELISA, PCR and immunoblotting instrumentation.

2) Research will also be conducted at the NHS Greater Glasgow and Clyde Clinical Research Facility (CRF, Glasgow Royal Infirmary, UK and at the Diabetes Centre (Clinic D), Stobhill Ambulatory Care Hospital, Glasgow, UK. There are facilities and exam rooms available for insulin pump download, CGM device download, meal and infusion studies and presence of research nursing staff for data collection and blood work at all times. One of the investigators will be available at all times to address patients' related issues. The CRF has equipment for sample preparation, infusion pumps, ultra-low freezers for sample storage and centrifuges. ELISA, PCR and immunoblotting facilities are available in adjacent NHS and University facilities.

12.2 For research conducted outside of UB and its affiliates, describe:

- *Site-specific regulations or customs affecting the research*
- *Local scientific and ethical review structure*

NOTE: This question is referring to UB affiliated research taking place outside UB, i.e. research conducted in the community, school-based research, international research, etc. It is not referring to multi-site research. UB affiliated institutions include Kaleida Health, ECMC, and Roswell Park Cancer Institute.

Response:

☒ **N/A:** This study is not conducted outside of UB or its affiliates.

13.0 Community-Based Participatory Research

13.1 Describe involvement of the community in the design and conduct of the research.

NOTE: Community-Based Participatory Research (CBPR) is a collaborative approach to research that equitably involves all partners in the research process and recognizes the unique strengths that each brings. CBPR begins with a research topic of importance to the community, has the aim of combining knowledge with action and achieving social change to improve health outcomes and eliminate health disparities.

Response:

☒ **N/A:** This study does not utilize CBPR.

13.2 Describe the composition and involvement of a community advisory board.

Response:

☒ **N/A:** This study does not have a community advisory board.

14.0 Resources and Qualifications

14.1 Describe the qualifications (e.g., education, training, experience, expertise, or certifications) of the Principal Investigator ***and*** *staff to perform the research. When applicable describe their knowledge of the local study sites, culture, and society. Provide enough information to convince the IRB that you have qualified staff for the proposed research.*

NOTE: If you specify a person by name, a change to that person will require prior approval by the IRB. If you specify a person by role (e.g., coordinator, research assistant, co-investigator, or pharmacist), a change to that person will not usually require prior approval by the IRB, provided that the person meets the qualifications described to fulfill their roles.

Response: All study personnel are educated, trained, and licensed as required for their delegated role in this study. All study personnel have also received the required university training and will be trained by the PI before the study starts. The PIs, CO-investigators and research team have conducted similar studies in this patient population using similar agents and have the expertise to execute the study protocol and manage any adverse events.

Describe other resources available to conduct the research.

14.2 Describe the time and effort that the Principal Investigator and research staff will devote to conducting and completing the research.

NOTE: Examples include the percentage of Full Time Equivalents (FTE), hours per week. The question will elicit whether there are appropriate resources to conduct the research.

Response: The principal investigator supervises the research project and weekly research meetings are conducted to discuss the recruitment rate, resolve and discuss issues related to the conduct, safety, analysis of the study and related publications. PI is expected to spend 15% of his academic time on this research. The co-investigators, study coordinator and research staff provide coverage to the research related activity for 365 days a year.

14.3 Describe the availability of medical or psychological resources that subjects might need as a result of anticipated consequences of the human research, if applicable.

NOTE: One example includes: on-call availability of a counselor or psychologist for a study that screens subjects for depression.

Response: Available medical literature will be provided as deemed appropriate or requested by patient through UB libraries, PubMed, Google scholar as all the investigators have access to medical literature through listed resources above

The patient will also have access to physician (Investigators and Co-Investigators) who will be available to address any adverse effects or other questions during the course of the study who will be available to address any adverse effects or other questions during the course of the study

14.4 Describe your process to ensure that all persons assisting with the research are adequately informed about the protocol, the research procedures, and their duties and functions.

Response: Before beginning of enrollment all research staff will be asked to review the protocol and a training session will be conducted to provide protocol details, explain technical points and answer staff questions. All training session will have a sign in sheet to document such training. Continuous education through training meetings, conferences and discussions will continue through the study period and when revision are made to protocol.

15.0 Other Approvals

15.1 Describe any approvals that will be obtained prior to commencing the research (e.g., school, external site, funding agency, laboratory, radiation safety, or biosafety).

Response: FDA IND filling is required.

☐ **N/A:** This study does not require any other approvals.

16.0 Provisions to Protect the Privacy Interests of Subjects

16.1 Describe how you will protect subjects' privacy interests during the course of this research.

NOTE: Privacy refers to an individual's right to control access to him or herself. Privacy applies to the person. Confidentiality refers to how data collected about individuals for the research will be protected by the researcher from release. Confidentiality applies to the data.

Examples of appropriate responses include: "participant only meets with a study coordinator in a classroom setting where no one can overhear", or "the participant is reminded that they are free to refuse to answer any questions that they do not feel comfortable answering."

Response: Our clinical providers involved in the study will identify potential patients for recruitment from the Diabetes-Endocrinology Center of WNY Clinics according to the inclusions and exclusion criteria and through advertisements. Patient who qualify will be asked in private during their one on one consultation time with the physician if they wish to participate in the research study. If the patient agrees, the research coordinator will contact them privately for a possible telephone screening and ask if time is appropriate for this discussion. The patients who call for potential participation in the study due to advertisement flyers will be screened over the phone with the research coordinator, using our telephone screening form.

When the patient is being seen at our clinics for the first time they sign the "Consent to use and disclosure of protected health information" form which clearly states that their protected health information (PHI) can be used for review in preparation for possible research. Additionally, a partial HIPAA waiver will be obtained to access medical records when screening for patients.

If the patient passes the telephone screening, they will be asked to make an appointment to review and sign the consent. Patient will do this in a private room in the research unit and will be allowed to discuss the consent in detail with the research coordinator and or study doctor. Patient will be notified that it is completely voluntary to participate in the research study and can withdraw at any time.

We will not be accessing any medical information of the patients for whom the services are not provided by our clinic providers before partial HIPAA is obtained or IC is signed.

16.2 Indicate how the research team is permitted to access any sources of information about the subjects.

NOTE: Examples of appropriate responses include: school permission for review of records, consent of the subject, HIPAA waiver. This question ***does apply*** *to records reviews.*

Response: Consent of the subject and partial HIPAA waiver.

17.0 Data Management and Analysis

17.1 Describe the data analysis plan, including any statistical procedures. This section applies to both quantitative and qualitative analysis.

Response:

Data (e.g. date of diagnosis, past medical history, drug treatment) extracted from local clinical records will be entered on to the eCRF under the study-specific coded identifier according to structured questions as stipulated in the Study Protocol. Biological samples (blood, urine and adipose tissue aspirates) will be collected from the study participants at the study visits according to the Schedule of Assessments in the Study Protocol.

Measures relating to safety and routine care of the participants (e.g., biochemical measures of glycemic and lipidemic control, kidney function/ fluid balance, hepatic function, hematological variables) will be analysed at local clinical laboratories in order that they are available to study nurses and physicians. The confidentiality of these data will be secure, meeting or exceeding the regulations of the relevant Ethical Review Committees/ Institutional Review Boards. Samples for measurement of key study outcomes will also be measured at the central laboratory at the University of Buffalo. Blood, urine and other samples will be aliquoted into cryovials at site for freezing and storage. Electronic stored material (e.g., patient records, data from blood and urine samples, and data from Continuous Glucose Monitoring) will only be stored using the study specific coded identifier. Data will not be identifiable at the participant level without additional access to the code. The code will be available only to the investigators and the personnel performing the study. Access to any data held electronically will require study specific log-in codes. These are managed and logged by the Robertson Centre for Biostatistics, University of Glasgow, UK

Statistical plan. The primary objective of the proposed research is to evaluate the effect of addition of dapagliflozin to semaglutide on a background of insulin treatment in type 1 diabetes patients on HbA1c at six months following start of dapagliflozin. The similarities between the study groups, baseline values for subject's demographics and study endpoints will be compared using appropriate parametric tests. Transformations of the data in order to meet statistical assumptions may be considered. The primary endpoint of the study is to detect a difference in mean HbA1c change after six months of dapagliflozin or placebo addition to semaglutide and insulin treatment. Statistical analysis will be carried out using t-tests to detect difference between groups or the Wilcoxon test for paired data. Tests will be two-sided and tested at the 0.05 level. The results will be summarized as mean±SE. All randomized patients will be included in the analysis of the primary end point based on an Intent-to-treat model.

The secondary endpoints will be to compare changes in HbA1c between each of the triple therapy and dual therapy groups compared to standard of care group, mean weekly glucose concentrations, percent time spent in different glycemic thresholds, variability as measured by standard deviation, insulin dose, body weight, carbohydrate intake, blood pressure, and 24-hour blood pressure and QOL changes among the groups. A reduction in the glucose and glucagon AUC following the meal will constitute another secondary end point in this setting, ANCOVA analysis will be used. Since the secondary end-points are pre-specified and may as well be correlated, multiplicity control for multiple tests across secondary endpoints may not be necessary. However, Holm-Bonferroni correction

for multiplicity testing will be implemented if additional endpoints are tested or if the pre-specified secondary end-points are found to be not correlated.

17.2 If applicable, provide a power analysis.

NOTE: This may not apply to certain types of studies, including chart/records reviews, survey studies, or observational studies. This question is asked to elicit whether the investigator has an adequate sample size to achieve the study objectives and justify a conclusion.

Response:

Sample size determination is based in part on our current data showing that in type 1 diabetes, treatment with a GLP-1 agonist, liraglutide (1.2 and 1.8mg doses) for 3 months leads to HbA1c suppression by an average of 0.6% (SD= 0.5%). Dapagliflozin was recently shown to reduce HbA1c by 0.45% in type 1 diabetes patients (13). Additionally, empagliflozin treatment for 8 weeks was shown to reduce HbA1c by 0.4% in type 1 diabetics (20, 21) and our current data showing additional improvement in HbA1c by an additional 0.66% at 12 weeks when dapagliflozin was added to liraglutide and insulin therapy. Conservatively estimating that the combination therapy will suppress HbA1c by 0.5% (SD =<0.7%) compared to semaglutide alone (t-test), a sample size of 38 (32 evaluable patients) in each group will be sufficient to provide adequate power ($\beta = 0.2$) to detect a statistically significant difference ($\alpha = 0.05$) taking in consideration a 15% dropout rate.

17.3 Describe any procedures that will be used for quality control of collected data.

Response:

An electronic case report form (e-CRF) will be used to collect study data at each site. The e-CRF will be developed by the study Data Centre at the Robertson Centre for Biostatistics, University of Glasgow. Access to the e-CRF will be restricted, with only authorised site-specific personnel able to make entries or amendments to their patients' data. It is the investigator's responsibility to ensure completion and to review and approve all data captured in the e-CRF.

Data will be validated at the point of entry into the e-CRF and at regular intervals during the study. Data discrepancies will be flagged to the study site coordinator and any data changes will be recorded in order to maintain a complete audit trail (reason for change, date change made, who made change).

The Robertson Centre for Biostatistics at the University of Glasgow will be responsible for collating and cleaning the data for the study. The Robertson Centre will also be responsible for data back-up and security. This center will also manage the electronic reporting of SUSARS on behalf of the sponsor.

To enable evaluations and/or audits from regulatory authorities, the investigators agree to keep records, including the identity of all participating subjects (sufficient information to link records, all original signed informed consent forms serious adverse event forms, source documents, and detailed records of treatment disposition. The records should be retained by the study country coordinators and investigator according to ICH GCP, local regulations, or as specified in the Clinical Study Agreement, whichever is longer.

Data from the study will be stored by the Chief Investigator for a minimum of 10 years. A final report of the study will be provided to the FDA and the MHRA as per requirements.

- No interim analysis is planned for efficacy so termination of study will be related to safety. Significant increases in hypoglycemic or DKA events as reviewed by DSMB might trigger termination or modification of study.
- Last observation carry forward will be used to fill missing data for the primary end point and some secondary end points. Missing data will not be filled for other endpoints.
- Any deviation(s) from the original statistical plan will be described and justified in the protocol and/or in the final report.
- All subjects entered into treatment will analyzed for primary end point on ITT bases. For secondary endpoints, analysis of completed and evaluable patients will performed per-protocol.

18.0 Confidentiality

A. Confidentiality of Study Data

Describe the local procedures for maintenance of confidentiality of ***study data and any records that will be reviewed for data collection****.*

18.1 A. Where and how will all data and records be stored? Include information about: password protection, encryption, physical controls, authorization of access, and separation of identifiers and data, as applicable. Include physical (e.g. paper) ***and*** *electronic files.*

Response: All data records will be stored on protected computers with unique passwords issued for each of the study team members and or in locked cabinets within the research center. These files will only be accessible by authorized study personnel.

18.2 A. How long will the data be stored?

Response: De-identified data storage has no expiration date and will be stored for a minimum of 7 years. The researchers may continue to rely on this for future use in research study.

18.3 A. Who will have access to the data?

Response: Those physicians, nurses, and laboratory staff that are on all documentation for the study will have access to the data.

18.4 A. Who is responsible for receipt or transmission of the data?

Response: Those physicians, nurses, and laboratory staff that are on all documentation for the study will have access to the data and can handle transfer of data. Study data will be transferred electronically (through eCRFs) to Robertson Centre for Biostatistics, University of Glasgow, UK

18.5 A. How will the data be transported?

Response: All data are stored at locally and will be transferred to Robertson Centre for Biostatistics, University of Glasgow, UK for data management and analysis. The transfer will be done through electronic CRFs processed on secure network.

Data will also be transported unless when is being archived. At that point files will be transferred to Iron Mountain for storage and archiving.

B. Confidentiality of Study Specimens

*Describe the local procedures for maintenance of confidentiality of **study specimens**.*

☐ **N/A:** No specimens will be collected or analyzed in this research.
(Skip to Section 19.0)

18.6 B. Where and how will all specimens be stored? Include information about: physical controls, authorization of access, and labeling of specimens, as applicable.

Response: The specimens will be stored in the laboratory located at *1000 Youngs Road, Suite 105, Williamsville NY 14221*and at the CTRC located in 875 Ellicott St. Buffalo NY14203. Samples will be stored in a locked -80º C freezer. All specimens collected at the Glasgow site will be stored in NHS -80º C freezers within the eCRF until shipping to Buffalo (World Courier).

18.7 B. How long will the specimens be stored?

Response: Specimens storage has no expiration date and will be stored for a minimum of 7 years. The researchers may continue to rely on this for future use in research study

18.8 B. Who will have access to the specimens?

Response: Those physicians, nurses, and laboratory staff that are on all documentation for the study will have access to the specimens

18.9 B. Who is responsible for receipt or transmission of the specimens?

Response: Those physicians, nurses, and laboratory staff that are on all documentation for the study will have access to the specimens and can handle transfer of samples. Sample/specimens will transported from University of Glasgow site to the university of Buffalo CTRC site (875 Ellicott St, Buffalo, NY 14203) for storage in -80C until analyzed.

18.10 B. How will the specimens be transported?

Response: All study samples will be centrally stored in UB CTRC site. Therefore, samples collected at the UK site will be transported for analysis and final long-term storage. Transport will be done so using dry ice in a properly labeled Styrofoam container by the laboratory technician. Agreement regarding shipping and receiving the specimens form UK site will be part of the subcontract agreement.

19.0 Provisions to Monitor the Data to Ensure the Safety of Subjects

☐ **N/A:** This study is not enrolling subjects, or is limited to records review procedures only. This section does not apply.

NOTE: Minimal risk studies may be required to monitor subject safety if the research procedures include procedures that present unique risks to subjects that require monitoring. Some examples include: exercising to exertion, or instruments that elicit suicidality or substance abuse behavior. In such cases, N/A is not an acceptable response.

19.1 Describe the plan to periodically evaluate the data collected regarding both harms and benefits to determine whether subjects remain safe.

Response:

An independent Data safety and monitoring board (DSMB) will be established for this trial. The board will consist of independent faculty members from UB and other institutions with sufficient knowledge of the field of type 1 diabetes but not

involved in the study. Data Management team at Robertson Centre will periodically provide the data on which to base regular reports to the Data and Safety Monitoring Board. The actual reports will then be generated by the unblinded statistician. The DSMB looks only at safety endpoints and not at futility. The DSMB will convene every six months to review safety end points, side-effects experienced by study subjects and any adverse events reported. The site PIs, CO-Is and study coordinator(s) will also continuously monitor the data and safety of subjects enrolled in the study. The site PIs and/or the DSMB may recommend to terminate the study or specific aspects of the study if clear harm is observed with one treatment option compared to standard of care or placebo. Specific safety endpoints that will be reviewed include increases in hypoglycemic and ketosis/DKA events.

19.2 Describe what data are reviewed, including safety data, untoward events, and efficacy data.

Response:

Assessment of Efficacy

1- glycemic indices including HbA1c, fructosamine, post-prandial glucose excursions and glucagon. CGM data will be collected as efficacy parameter of glucose control. Total insulin dose will be also documented and analyzed.
2- body weight
3- blood pressure and use of blood pressure medications
4- QOL questionnaires.

The timing of recording of the above efficacy parameters is described in table 1.

Assessment of Safety

The following safety assessments assays and measurement will be reviewed:

1- Vitals, CBC, CMP and markers kidney and liver function will be collected at screening, during and at end of the study as in table 1.
2- Assessment of hypoglycemia: Glucose data will be reviewed from CGM and daily finger stick and during visits.
3- Assessment of ketosis: Ketones levels will be reviewed from daily urinary ketone diaries or blood ketone meters and from urine and blood at the time of each visit
4- Reports of side effects by patients.
5- Insulin dose titrations and pump downloads at each visit

The methods and timing for assessing, recording, and analyzing safety parameters.

The DSMB will convene every six months to assess the side-effects experienced by study subjects and any adverse events reported. The PIs, CO-Is and study coordinator(s) will also continuously monitor the safety of subjects enrolled in the study during their visits and during the safety phone calls.

Procedures for eliciting reports of and for recording and reporting adverse event and inter-current illnesses.

All adverse events will be reported at the time of the visit or by telephone when it occurs. Medical events that occur between the signing of the Informed Consent and the first intake of study drug will be documented in the medical history. Participants will be asked to report any adverse events in general, non-directed questioning. For each AE volunteered by the subject, the investigator will obtain all the information required to complete the adverse event page of the eCRF, in accordance with the guidelines that accompany it. All adverse events, regardless of seriousness, severity, or presumed relationship to study therapy, will be recorded using medical terminology in the source document. Whenever possible, diagnoses will be given when signs and symptoms are due to a common etiology (e.g., cough, runny nose, sneezing, sore throat, and head congestion should be reported as "upper respiratory infection). Investigators will record on the CRF their opinion concerning the relationship of the adverse event to study therapy. The principal investigators assumes responsibility for appropriate reporting of AEs to the regulatory authorities. The investigator will report these events to the appropriate Independent Ethics Committee/Institutional Review Board (IEC/IRB) in accordance with local regulations. All measures required for AE management will be recorded in the source document and reported according to the State University of New York at Buffalo instructions. Non-serious events/problems that impacted on the rights and welfare of the subjects will be reported as they occur or at the time of knowledge or notification.

Definition of adverse events

The International Conference on Harmonization (ICH) Guideline for Good Clinical Practice (GCP) E6(R1) defines an AE as:

Any untoward medical occurrence in a patient or clinical investigation patient administered a pharmaceutical product and which does not necessarily have a causal relationship with this treatment. An AE can therefore be any unfavorable and unintended sign (including an abnormal laboratory finding), symptom, or disease temporally associated with the use of a medicinal product, whether or not considered related to the medicinal product.

The term AE is used to include both serious and non-serious AEs.

Definition of serious adverse events

A serious adverse event is an AE occurring during any study phase (i.e., screening, run-in, treatment, wash-out, follow-up), at any dose of the study drugs that fulfils one or more of the following criteria:

- Results in death
- Is immediately life-threatening
- Requires in-patient hospitalization or prolongation of existing hospitalization
- Results in persistent or significant disability or incapacity
- Is a congenital abnormality or birth defect in offspring of the patient
- Is an important medical event that may jeopardize the patient or may require medical intervention to prevent one of the outcomes listed above.

- TO THE AUTHOR: The causality of SAEs (their relationship to all study treatment/procedures) will be assessed by the investigator(s) and communicated to AstraZeneca. This is requested to be on the AE cover sheet.

The following variables are recommended to be collected for each AE:

- AE (verbatim)
- The date when the AE started and stopped
- Whether the AE is serious or not
- Action taken with regard to IPs
- Administration of treatment for the AE
- Outcome

In addition, the following variables are recommended to be collected for SAEs:

- Date the AE met criteria for SAE
- Date the Investigator became aware of the SAE
- Seriousness criteria fulfilled
- Date of hospitalization
- Date of discharge
- Probable cause of death
- Date of death
- Whether an autopsy was performed
- Causality assessment in relation to study procedure(s)
- Causality assessment in relation to other medication, as explained in Section 1.4.2.
- Description of the SAE

Reporting of serious adverse events

All SAEs will be reported, whether or not considered causally related to the investigational product, or to the study procedure(s). The reporting period for SAEs is the period immediately following the time that written informed consent is obtained throughout the treatment period and including the designated follow-up period as per the study protocol. The investigator is responsible for informing the Ethics Committee and/or the Regulatory Authority of the SAE as per local requirements.

Note that all serious or unexpected adverse events must be reported to AstraZeneca regardless of the country where the study is conducted.

The investigator must inform the FDA, via a MedWatch form, of any serious or unexpected adverse events that occur in accordance with the reporting obligations of 21 CFR 312.32 , and will concurrently forward all such reports to AstraZeneca. A copy of the MedWatch report must be emailed to AstraZeneca (TCS vendor) at the time the event is reported to the FDA. It is the responsibility of the investigator to compile all necessary information and ensure that the FDA receives a report according to the FDA reporting requirement timelines and to ensure that these reports are also submitted to AstraZeneca at the same time.

A cover page should accompany the MedWatch form indicating the following:

- "Notification from an Investigator Sponsored Study"

- The investigator IND number assigned by the FDA
- The investigator's name and address
- The trial name/title and AstraZeneca ISS reference number (ESR-##-#####)

Investigator must also indicate, either in the SAE report or the cover page, the causality of events in relation to all study medications and if the SAE is related to disease progression.

Send SAE report and accompanying cover page by way of email to AstraZeneca's designated mailbox: AEMailboxClinicalTrialTCS@astrazeneca.com

If a non-serious AE becomes serious, this and other relevant follow-up information must also be provided to AstraZeneca and the FDA.

Serious adverse events that do not require expedited reporting to the FDA still need to be reported to AstraZeneca preferably using the MedDRA coding language for serious adverse events.

The investigators will either provide a copy of the randomization code/ code break information or unblind those SAEs which require expedited reporting

Overdose

An overdose is defined as a patient receiving a dose of IP in excess of that specified in the Investigator's Brochure, unless otherwise specified in this protocol.

Any overdose of a study patient with the investigational product, with or without associated AEs/SAEs, is required to be reported within 24 hours of knowledge of the event to AstraZeneca Patient Safety using the designated Safety e-mailbox (see Section 1.5 or contact information). If the overdose results in an AE, the AE must also be recorded as an AE. Overdose does not automatically make an AE serious, but if the consequences of the overdose are serious, for example death or hospitalization, the event is serious and must be recorded and reported as an SAE (see Section 1.3). Any specific details around overdose for combination or comparator agents will also be provided as applicable. The investigator will use clinical judgment to treat any overdose.

Hepatic function abnormality

Hepatic function abnormality that fulfills the biochemical criteria of a potential Hy's Law case in a study patient, with or without associated clinical manifestations, is recommended to be reported as "hepatic function abnormal" within 24 hours of knowledge of the event to AstraZeneca Patient Safety using the designated Safety e-mailbox (see Section 1.3), unless a definitive underlying diagnosis for the abnormality (e.g., cholelithiasis or bile duct obstruction) that is unrelated to investigational product has been confirmed. The criteria for a potential Hy's Law case is Aspartate Aminotransferase (AST) or Alanine Aminotransferase (ALT) ≥3x Upper Limit of Normal (ULN) together with Total Bilirubin (TBL) ≥2xULN at any point during the study following the start of study medication irrespective of an increase in Alkaline Phosphatase (ALP).

Pregnancy

If a patient becomes pregnant during the course of the study, the IPs should be discontinued immediately.

Pregnancy itself is not regarded as an AE unless there is a suspicion that the IP under study may have interfered with the effectiveness of a contraceptive medication. Congenital abnormalities or birth defects and spontaneous miscarriages should be reported and handled as SAEs. Elective abortions without complications should not be handled as AEs. The outcome of all pregnancies (spontaneous miscarriage, elective termination, ectopic pregnancy, normal birth, or congenital abnormality) should be followed up and documented even if the patient was discontinued from the study.

If any pregnancy occurs in the course of the study, then the investigator should inform the appropriate AstraZeneca representatives within 1 day, i.e., immediately, but no later than 24 hours of when he or she becomes aware of it.

The investigator will ensure that all relevant information is provided to the AstraZeneca Patient Safety data entry site within 1 to 5 calendar days for SAEs and within 30 days for all other pregnancies.

The same timelines apply when outcome information is available.

19.3 Describe any safety endpoints.

Response: These specific Safety endpoints will be collected:

1- Frequency and severity of hypoglycemia: Hypoglycemia will be assessed throughout the study via multiple daily finger stick glucose measurements, CGM data collected 3 times (about 3 weeks each) during the study and from patient reports during visits and phone calls. Hypoglycemic events will be characterized as in the experts consensus published in *Diabetes Care (2017 Dec; 40(12): 1622-1630):*

- Level 1: Glucose < 70 mg/dL (3.9 mmol/L) and Glucose ≥ 54 mg/dL (3.0 mmol/L)
- Level 2: Glucose < 54 mg/dL (3.0 mmol/L)
- Level 3: A severe event characterized by altered mental and/or physical status requiring assistance.

2- Ketones levels: Urinary ketones measured daily (and any blood ketones) at home and logged in the ketones logbook. Blood and urinary ketones measured during each visit. Ketone levels will be characterized as normal, ketosis and severe ketosis:

-Normal blood ketones <0.6mM or urinary ketones of trace to small (≥+1)

-Ketosis: Blood ketones >0.6mM <2.5mM or urinary ketones of moderate to high (+2 and +3)

-Severe ketosis Blood ketones >2.5mM or urinary ketones of too high (+4)

3- Diabetes ketoacidosis episodes: any confirmed DKA episode (regardless of hospitalization) defined as:

- Elevated serum or urine ketones (greater than the upper limit of the normal range), and
- Serum bicarbonate < 15 mmol/L or Blood pH < 7.3

4- Low blood pressure: <90mmHg SBP and <50 mmHg DBP): Patients will be instructed to measure blood pressure daily and it will be will be assessed in each visit.
5- Nausea, vomiting and other GI side effects. Data will be collected by patients' safety reports, during visits and phone calls.
6- Genital mycotic infections or UTIs
7- Changes in liver and kidney function: CMP and kidney and liver profiles will be assessed as indicated in table 1.

19.4 Describe how the safety information will be collected (e.g., with case report forms, at study visits, by telephone calls with participants).

Response: The safety information will be collected at the time of the visit, and or during preplanned telephone calls with the participant or patient self-reporting (including glucose, food intake and ketones diaries). Data will be entered into eCRFs

19.5 Describe the frequency of safety data collection.

Response: Safety data collection will be done at all study visits and during preplanned phone calls as in table 1. The patient, however, will be asked to report any adverse event or safety related information via phone as soon as it occurs and it will be reviewed the same day.

19.6 Describe who will review the safety data.

Response: DSMB established for this study will review unblended safety data provided by the data management system every 6 months. Additionally, the PIs and coinvestigators will review safety data (listed above) at the completion of each visits by each subject and review any report of side effects or adverse events as they are presented by the patients. The investigators will also review collective blinded data every 3 months to assess the safety and any potential risks to the participants. Furthermore the investigators will assess other risks including the physical, psychological, social, legal and economic harm to these patients. The investigators will carefully watch for any invasion of privacy and breach of confidentiality. The principal investigator will be sharing the results of safety analysis of the study and serious adverse events with the local IRB, DSMB, JDRF, AZ and the FDA. The study groups will remain blinded. If there are a serious safety concerns or an emergency, then, and based on principal investigator discretion, study randomization center in Glasgow University or the unblended statistician will provide information about group assignment of the patient(s) involved. Un-blinded data will be reviewed by the principal investigator and co-investigators and decision to complete, withdraw or adjust dose of drug(s) will be made, documented and shared with IRB and DSMB.

19.7 Describe the frequency or periodicity of review of cumulative safety data.

Response: Safety data will be reviewed every 6 months by DSMB (un-blinded), every 3 months by PIs and CO-Is (blinded) and at every visit by study team.

19.8 Describe the statistical tests for analyzing the safety data to determine whether harm is occurring.

Response: N/A

19.9 Describe any conditions that trigger an immediate suspension of the research.

Response: No interim analysis is planned for efficacy so termination of study will be related to safety. Significant increases in severe hypoglycemic or DKA events as reviewed by DSMB are possible causes that might trigger termination of study.

20.0 Withdrawal of Subjects

☐ **N/A:** This study is not enrolling subjects. This section does not apply.

20.1 Describe ***anticipated*** *circumstances under which subjects may be withdrawn from the research without their consent.*

1. The site PI can stop investigational study treatment or remove participants from the trial without participants approval if for any reason he/she feels is appropriate, including: severe side effect, injury or medical condition which may place the patient at risk of further complications if he/she continue to participate, failure to take the medication as instructed, failure to keep scheduled appointments, cancellation of the study by the sponsor, IRB, FDA or other administrative reasons.
2. If any of the participants become pregnant during the period of study, they will discontinue study treatment.
3. Participants who experience persistent severe nausea or vomiting after starting semaglutide which is not managed by dose reduction will discontinue the investigational study treatment. Those with mild or moderate nausea will continue for at least one more week. If they continue to experience significant nausea (defined as interfering with normal activities), dose will be reduced. If they continue to have these side effects, they will discontinue study medication(s).
4. Persistent weight loss or failure to maintain weight if subject has lost >15% or BMI is < 22 kg/m2 in spite of down-titration of semaglutide and/or dapagliflozin.
5. Developing other conditions included in the exclusion criteria
6. Change in method of insulin delivery between insulin pumps to MDI or vice versa during the study unless pump treatment interruption is temporary for a period of at most 2 weeks. Also, patients who upgrade to the new "artificial pancreas" pump (Medtronic's MiniMed 670G hybrid) during the study will be withdrawn.

7. Hypoglycemia: Two episodes of severe hypoglycemia (requiring others help).
8. Ketosis (with no other identifiable precipitating factors): Persistent (1week) asymptomatic or symptomatic and unmanaged (using STICH protocol) blood ketone levels >1.5 mM (or ≥+2 in urine).
9. Persistent (>1 week) total insulin dose reduction of >20% from baseline AND total insulin dose <0.4U/Kg.
10. DKA: Two episode of DKA (with no identifiable precipitating factors) as defined by the expert consensus statement (*Diabetes Care (2017 Dec; 40(12): 1622-1630)*)
11. Two episodes of UTI.
12. Two episodes of mycotic genital infection.
13. Significant persistent change in eGFR > 15 ml/min
14. Pancreatitis: One episode of pancreatitis.
15. Other persistent side effects specific to study drugs

20.2 Describe any procedures for orderly termination.

NOTE: Examples may include return of study drug, exit interview with clinician. Include whether additional follow up is recommended for safety reasons for physical or emotional health.

Response: Participation in this study is voluntary. Participants have the right to refuse to participate or to withdraw from participation at any time for any reason. Refusal to participate or to withdraw from the study will involve no penalty or loss of entitled benefits, nor affect ongoing medical care. The site PI can stop investigational study treatment or remove participants from the trial without participants approval. If a patient withdraws consent and decide not to participate in the study, she/he will be asked to undergo a final study visit for his/her safety. If a subject withdraws from the research, the data collected to that point will be used toward the research finding. If applicable the subject will have to bring back any unused research drug and or devices.

20.3 Describe procedures that will be followed when subjects withdraw from the research, including retention of already collected data, and partial withdrawal from procedures with continued data collection, as applicable.

Response: We will use the data collected up to the point of stopping the study. No further information will be collected. Patients who stop study treatment and are interested in continuing in the study will be followed up until the end of the study for the primary end point and select secondary endpoints based on the Intent-To-Treat (ITT) analysis model. Withdrawn participants will not be replaced. The study sample size calculation includes an additional 15% subjects to compensate for drop out.

21.0 Risks to Subjects

21.1 List the reasonably foreseeable risks, discomforts, hazards, or inconveniences to the subjects related to their participation in the research. Consider physical, psychological, social, legal, and economic risks. Include a description of the probability, magnitude, duration, and reversibility of the risks.

NOTE: Breach of confidentiality is always a risk for identifiable subject data.

Response:

- Risks associated with intravenous (IV) line: All participants will be informed of the potential complications of an intravenous (IV) line, which include mild bruising at the site of IV line, usually resolving in a few days. They will also be informed about the possibility of infiltration of the IV line at the time of performing blood draws or during the meal challenge test in which case another IV line at a different site will be required (with risk of bruising at more than one site).
- Risks associated with 24 hour Ambulatory BP monitoring device: The BP cuff inflates every 15 minutes to record blood pressure over 24-hours. Participants will experience a pressure-like sensation over the arm where 24 hour BP device is worn at the time of the BP being measured due to the inflation of the cuff and release of this pressure at the time of deflation of the BP cuff. They will be informed to remove the cuff after 24 hours. If worn for longer periods they may experience mild bruising over the arm and some swelling, usually resolving 1-2 days after discontinuation.
- Risks associated with fat tissue aspiration: will probably lead to a bruise at the site of aspiration. The site may be mildly painful for 1-2 weeks. Participants are advised to call their study site staff if they have more severe pain or swelling at the site after the procedure. Rarely there are side effects such as low BP or heart rate and/or allergic reaction to local anesthetic including swelling of the throat.
- All subjects will be referred to semaglutide and dapagliflozin labeling safety information, and will be given a copy of the package insert of the medication. Discussion of the potential side effects and the different warning and precautions will take place at the time of informed consent. Patients will be informed they must not discontinue their insulin during the study.
- Potential risk of insulin, GLP-1 RA and SGLT-2 inhibitors use in type 1 diabetes patients is hypoglycemia. The combination therapy can possibly increase the magnitude and frequency of this risk.
- Potential side effects of GLP-1 RA and SGLT-2 inhibitors also include hypotension, nausea and other GI side effects, polydipsia, polyuria, dehydration, ketosis, and reduced glomerular filtration rate (GFR).
- In rodents, semaglutide causes dose-dependent and treatment-duration-dependent thyroid C-cell tumors at clinically relevant exposures. However, it received FDA-approval for the treatment of type 2 diabetes in 2017 following large Phase 3 trials including the cardiovascular safety trial SUSTAIN-6.
- On the basis of the above rodent studies, semaglutide is contraindicated in those with a personal or family history of medullary thyroid carcinoma and in patients with Multiple Endocrine Neoplasia syndrome type 2 (MEN 2). However, following review of human data with GLP-1RAs, FDA does not require or suggest routine monitoring of serum calcitonin/ thyroid ultrasound in people treated with semaglutide.
- Injection-site reactions: Serious injection-site reactions, with or without bumps (nodules), have happened in some people who use GLP-1RAs but are less frequent with semaglutide. Injection-site reactions have rarely required surgical

intervention. Participants will be instructed to report any injection site reactions including nodules to study investigators.

- Kidney injury: GLP-1 RA might cause acute kidney injury in patients without underlying renal disease especially in patient who had experienced nausea, vomiting, diarrhea or dehydration. Dapagliflozin also has warnings and precautions against acute kidney injury and impairment in renal function.
- As mentioned above, there is a risk of ketoacidosis, as shown by some studies and by our data, when SGLT2 inhibitors are used in type 1 diabetes. In our own preliminary studies on triple therapy, we observed euglycemic ketoacidosis in participants whose insulin doses had been reduced by > 20% of baseline levels or in patients who are not adequately insulinzed. Thus, this adverse effect is largely attributable to insulinopenia as well as anti-insulin effects of SGLT2 inhibitors at the hepatic level (e.g. increased gluconeogenesis).
- The following other known adverse effects of SGLT-2 inhibitors will be discussed with participants when obtaining informed consent:

1. Risk of dizziness/ symptomatic hypotension: can occur soon after initiation due to intravascular volume contraction.
2. Impaired kidney function: As SGLT-2 inhibitors increase serum creatinine and decrease eGFR, this will be monitored closely.
3. Genital mycotic infections: SGLT-2 inhibitors increase the risk of genital mycotic infections, particularly in women. Those with a prior history are more susceptible. Following topical treatment with anti-fungal medications they rarely re-occur.
4. Bladder Cancer - Across 22 clinical studies, newly diagnosed cases of bladder cancer were reported in 10/6045 patients (0.17%) treated with Dapagliflozin and 1/3512 patient (0.03%) treated with placebo. After excluding patients in whom exposure to study drug was less than one year at the time of diagnosis, there were 4 cases of bladder cancer with dapagliflozin and no cases with placebo i.e. too few cases to determine causality.
5. The dapagliflozin tablets and matching placebo contain lactose, which may cause discomfort in lactose-intolerant individuals.

- Severe allergic reaction (anaphylaxis) to study drugs or placebo ingredients or other interventions may accrue in rare cases.
- Excessive weight loss: Both investigation drugs are known to cause weight loss in the range of 5-10% in type 2 diabetes patients. However, the magnitude of weight loss following the combination of both drugs in type 1 diabetes patients may be significantly more. Excessive weight loss may lead to increased risk of hypoglycemia and ketosis or DKA (especially in the presence of dapagliflozin) and other unforeseen risks. Therefore, weight loss during any phase of the study will be limited to ≤15% baseline body weight or reaching a BMI of $\leq 22 Kg/m^2$.
- There might be other unforeseen risks associated with the use of these agents in type 1 diabetes and additional unforeseen risks associated with the combination therapy.
- There is always the risk of breach of confidentiality where patients' identifiable data is compromised.

21.2 Describe procedures performed to lessen the probability or magnitude of risks, including procedures being performed to monitor subjects for safety.

Response:

The following are procedures to lessen the probability and magnitude of risks listed above:

1- Hypoglycemia:

- Patients will be instructed to measure glucose at least 4 times a day (before meals and at bedtime) through-out the study and record all reading.
- Insulin dose adjustments will be done at every visit to avoid hypoglycemia. However, there will be no preemptive insulin dose reduction at start of treatment since all patients will have HbA1c >7.5% and the risk of hypoglycemia will be minimal when semaglutide is added.
- Daily fingerstick glucose, CGM and insulin pump data will be reviewed by the investigators for optimal titration of insulin dose to target glucose levels between 90-180mg/dl.
- In general, insulin dose adjustments should not be >10% from current total insulin dose (or 5% if HbA1c is <7%) or >20% from baseline total insulin dose at week 0 and then at week 26.
- Patients will be trained in CARB counting to avoid inaccurate insulin dose administration.
- Patients will be trained to recognize symptoms of hypoglycemia and how to manage it at home. These symptoms include feeling shaky, being nervous or anxious, sweating, chills and clamminess, confusion, fast heartbeat, feeling lightheaded or dizzy, hunger, nausea and color draining from the skin (pallor) among others. They will be asked to call the research office or the on call clinical services.
- When patients feel symptoms of hypoglycemia they will be asked to measure blood glucose and if <70mg/dl they will be instructed to consume carbohydrate or 4 tablets of glucose (4gm each). If after 15 min hypoglycemia continues, repeat carb intake until sugar levels are above 70 mg/dl. If sugar levels are below 55mg/dl, glucagon injection is permitted as rescue medication for severe hypoglycemia.
- Patients will be instructed to inform people who they are in frequent contact with (for example, friends, family members, and coworkers) on how to give glucagon to treat severe hypoglycemia. If patient have needed glucagon previously, it should be documented during screening visit so more emphasis on ways to prevent severe hypoglycemia in the future can be discussed.
- Patients/family will be instructed not to hesitate to call 911 if someone is unconscious and glucagon is not available or someone does not know how to use it and not to inject insulin (it will lower their blood glucose even more) or provide food or fluids (they can choke). They will be asked to document all details of the incident and report to research staff as soon as possible.
- Study medications dose will be reduced in patients who continue to have more than 2 measurements of glucose levels in the hypoglycemic range (<70mg/dl, regardless of symptoms) after insulin dose is reduced by the maximum allowed in this study.

2- Ketosis and Ketoacidosis:

- Patients will be instructed to measure ketone bodies in urine samples daily starting at 3 days before week 26 with Ketostix (Bayer) and record it in diary provided. Ketones (blood and urine) will be measured at every visit. Additionally, patients will be provided with a ketone meter and strips to measure blood ketones when needed. If ketone bodies in urine measure +1 or greater (>1.5mM in urine), they will be instructed to measure blood ketones and to repeat both the measurements after 60 min. If consecutive measurements show moderate or higher ketones in urine or blood ketones >0.6mM, the patient will be instructed to call the research center or their doctor directly. They will be monitored closely for insulin dose, carb intake, ketones and blood sugar and dapagliflozin/semaglutide can be temporarily discontinued, if necessary, for 1-2 weeks.
- Patients will be provided training to recognize the signs and symptoms of DKA and to self-test urine ketones on a PRN basis (in addition to once daily) if they develop any symptoms consistent with DKA, including nausea, vomiting, fatigue, confusion, or shortness of breath. This should be reported regardless of blood sugar levels since in type 1 diabetes on SGLT-2 inhibitors, DKA can occur even in normal glucose levels.
- Insulin dose changes should not be greater than 10% (5% if HbA1c is<7%) from current levels and not reduced by more than 20% from baseline levels at 0 weeks and then at 26 weeks.
- Patients requiring an insulin dose reduction > 20% AND their total insulin dose is <0.4U/Kg will be monitored closely for insulin dose, carb intake, ketones and blood sugar and dapagliflozin/semaglutide can be temporarily discontinued, if necessary, for 1-2 weeks. If insulin requirements remain law, patient will be withdrawn from study.
- We shall not increase dose of dapagliflozin in patients who exhibit significant ketonuria (+2 or more) with the lower dose.
- Urinary ketones (and blood ketones if patients have a dual glucose and ketone meter) will be monitored daily and reported. Dapagliflozin will be temporarily (1 week) discontinued and STICH protocol (STop SGLT-2i, give Insulin bolus, Carbohydrate, and Hydration) activated in events of DKA or if based on investigator discretion if blood ketone levels are persistently (1 week) >1.5 mM (+2 in or more in urine). Dapagliflozin may also be temporarily stopped in clinical situations known to predispose to ketoacidosis (e.g. prolonged fasting due to acute illness or surgery). Patients will be instructed to stay hydrated and not reduce their caloric intake (especially carbohydrates) or start new dietary systems (especially ketogenic diets) during the study.
- If a patient develops symptoms of DKA or ketone levels exceed the allowed limit in this study (persistent (1 week) blood ketones >1.5 mM or +2 in or more in urine), STICH protocol (STop SGLT-2i, give Insulin bolus, Carbohydrate, and Hydration) will be activated. They could be managed for up to a week. If symptoms and ketone levels persist over 1 week, patients will be withdrawn form study. Insulin infusions may be administered by ER or hospital if patients

develop DKA. They will be asked to document all details of the incident and report to research staff as soon as possible.

3- Risk of acute kidney injury or renal function impairment: CBC, CMP, including kidney function test and urinary markers will be performed at screening, during and at end of the study (table 1). Additional testing will be performed in patients who suffer from sever nausea or GI symptoms or hypovolemic and during the up titration of investigation drugs. If acute kidney injury occurs dapagliflozin will be discontinued immediately. To lessen the chance of this risk, patients will eGFR<60 ml/min/1.73m^2 will be excluded. Patients will also discontinue study medications if eGFR falls by >15 ml/min in 2 repeated measurements. Also other patients with factors that may predispose patients to acute kidney injury e.g. hypovolemia, chronic renal insufficiency, congestive heart failure will be excluded. Patients on ACEI, ARB, NSAID will be monitored closely. Dapagliflozin will be temporarily discontinued in any setting of reduced oral intake such as acute illness or fasting or fluid losses such as gastrointestinal illnesses, or excessive heat exposure.

4- Injection site reactions, mycotic infections and complications of IV and biopsy collection will also be monitored, Physical examination will be performed at screening 26, and 52 weeks visits. Safety vital assessments will be performed at every visit. Pregnancy will be tested at screening, 0, 4 13, 26, 39, and 52 weeks. Phone calls will be made for safety and compliance at least 7 time during the study. The patients, however, will be instructed to report any adverse event or safety related information via phone as soon as it occurs. All safety data will be reviewed by the PI or sub-investigators within 1-2 days of collection.

5- Epinephrine is the primary treatment for anaphylaxis with no absolute contraindication to its use. Our site and staff are trained and equipped to assist patients with such reactions. Severe conditions that are not satisfactory managed in site will be transferred to the hospital emergency department for further assessment and help.

6- Excessive weight loss: Doses of investigational drugs will be reduced in patients who experience excessive weight loss (≥15% of basal body weight or BMI ≤22Kg/m^2). Patients on semaglutide only (first 26 weeks) will be down-titrated to the lower dose. In patients on semaglutide and dapagliflozin/placebo (weeks 26-52), dose of dapagliflozin/placebo will be down-titrated to the lower dose. If weight loss continues, semaglutide dose will be down-titrated as well. Instructions to maintain a minimum of 50g daily carb intake will be also emphasized in patients with significant weight loss (>10% baseline body weight).

21.3 If applicable, indicate ***which procedures*** *may have risks to the subjects that are currently unforeseeable.*

Response:

There might be additional adverse effects of dapagliflozin not currently known or those unique to their use in Type 1 diabetes. There is no previous studies on the use of semaglutide in type 1 diabetes; therefore, additional unforeseen risk may be encountered in this study. Additionally, unforeseen risks might be associated with the combination therapy that might involve unknown drug-drug interactions.

21.4 If applicable, indicate which research procedures may have risks to an embryo or fetus should the subject be or become pregnant.

Response: Not applicable, we will not be enrolling pregnant participants or who may become pregnant or with in child baring years without signing the consent stating they will use at least two forms of birth control. If a participant becomes pregnant they will be withdrawn from the study immediately. They will be followed until term and any effects on the newborn will be documented.

21.5 If applicable, describe risks to others who are not subjects.

Response: We do not anticipate any additional risk to others not participating in this study.

22.0 Potential Benefits to Subjects

22.1 Describe the potential benefits that individual subjects may experience by taking part in the research. Include the probability, magnitude, and duration of the potential benefits. Indicate if there is no direct benefit.

*NOTE: Compensation **cannot** be stated as a benefit.*

Response: The investigational drugs used in this study are already approved as antidiabetic agents for treating type 2 diabetes. Both agents have demonstrated significant improvement in HbA1c, weight and blood pressure. It is our expectation that patients with type 1 diabetes will experience similar benefits compared to those who are treated with insulin alone. The magnitude and duration of the benefits and the safety profile may however differ and are the focus of this investigation.

23.0 Compensation for Research-Related Injury

☐ **N/A:** The research procedures for this study do not present risk of research related injury (e.g. survey studies, records review studies). This section does not apply.

23.1 ***If the research procedures carry a risk of research related injury,*** *describe the available compensation to subjects in the event that such injury should occur.*

Response: Routinely, Buffalo General Hospital, Erie County Medical Center, and/or the University at Buffalo, State University of New York, its agents, or its employees do not compensate for or provide free medical care for human

subjects/participants in the event that any injury results from participation in a human research project. In the unlikely event that they become ill or injured as a direct result of participating in this study, they may receive medical care. Your study doctor will explain the treatment options to you and tell you where you can get treatment. Generally, this care will be billed to you, your insurance or other third party. Additionally, AstraZeneca shall reimburse the institution for the direct, reasonable, and necessary medical expenses incurred by the institution for the treatment of any personal injury that is a direct result of adverse drug experiences to subjects resulting directly from any manufacturing defects in the study drug to which the subjects would not have been exposed but for their participation in the study if (i) the institutional parties have complied with this agreement, the protocol and any written instructions of AstraZeneca concerning the study drug and (ii) all the requirements of informed consent have been complied with. AstraZeneca will not provide compensation for lost wages or for any other damages, expenses or losses, or for medical expenses have been covered by a subject's medical or other insurance.

23.2 Provide a copy of contract language, if any, relevant to compensation for research related injury.

NOTE: If the contract is not yet approved at the time of this submission, submit the current version here. If the contract is later approved with ***different language regarding research related injury****, you must modify your response here and submit an amendment to the IRB for review and approval.*

Response: AstraZeneca shall reimburse the institution for the direct, reasonable, and necessary medical expenses incurred by the institution for the treatment of any personal injury that is a direct result of adverse drug experiences to subjects resulting directly from any manufacturing defects in the study drug to which the subjects would not have been exposed but for their participation in the study if (i) the institutional parties have complied with this agreement, the protocol and any written instructions of AstraZeneca concerning the study drug and (ii) all the requirements of informed consent have been complied with. AstraZeneca will not provide compensation for lost wages or for any other damages, expenses or losses, or for medical expenses have have been covered by a subject's medical or other insurance.

24.0 Economic Burden to Subjects

24.1 Describe any costs that subjects may be responsible for because of participation in the research.

NOTE: Some examples include transportation or parking.

Response: All research expenses will be covered by the study. The background use of insulin is considered as part of standard of care is the responsibility of the patients and his/her insurance. Participants will not be subjected to any other out of pocket cost.

☐ **N/A:** This study is not enrolling subjects, or is limited to records review procedures only. This section does not apply.

25.0 Compensation for Participation

25.1 Describe the amount and timing of any compensation to subjects, including monetary, course credit, or gift card compensation.

Response:
Visit schedule:

- Screen: $0
- Visit 1 (Week -3): Insulin adjustment: $10
- Visit 2 (Week -1): Meal ($75), insulin adjustment, and 24hr blood pressure monitor ($50): $125*
- Visit 3 (Week 0): 24 hr urine ($15), blood draw ($25), fat biopsy ($75), insulin adjustment, questionnaire ($10): $125
- Visit 4 (Week 1): Insulin adjustment: $10
- Visit 5 (Week 2): Insulin adjustment: $10
- Visit 6 (Week 4): Insulin adjustment: $10
- Visit 7 (Week 13): 24hr urine ($15), blood draw ($25), insulin adjustment, 24hr bp monitor ($50): $90*
- Visit 8 (Week 23): Insulin adjustment: $10
- Visit 9 (Week 25): Insulin adjustment, 24bp monitor ($50), Meal ($75): $125*
- Visit 10 (Week 26): Insulin adjustment, 24hr urine ($15), blood draw ($25), fat biopsy ($75), questionnaire ($10): $125*
- Visit 11 (Week 27): Insulin adjustment: $10
- Visit 12 (Weak 31): Insulin adjustment, blood draw: $25
- Visit 13 (Week 39): Insulin adjustment, 24hr urine ($15), blood draw ($25), 24hr bp monitor ($50): $90*
- Visit 14 (Week 49): Insulin adjustment: $10
- Visit 15 (Week 51): Insulin adjustment, 24bp monitor ($50), meal ($75): $125*
- Visit 16 (Week 52): Insulin adjustment, 24hr urine ($15), blood draw ($25), questionnaire ($10), fat biopsy ($75):$125*

*Total compensation for all completed visits: up to $1,025.00. For those not wishing to participate in the optional blood pressure monitoring and/or fat biopsy procedure, the compensation will decrease by $250 for not completing the bp monitoring, and by yet another $225.00 for not completing the fat biopsies.

☐ **N/A:** This study is not enrolling subjects, or is limited to records review procedures only. This section does not apply.

☐ **N/A:** There is no compensation for participation. This section does not apply.

26.0 Consent Process

26.1 Indicate whether you will be obtaining consent.

NOTE: This does not refer to consent documentation, but rather whether you will be obtaining permission from subjects to participate in a research study. Consent documentation is addressed in Section 27.0.

☒ ***Yes*** *(If yes, Provide responses to each question in this Section)*

☐ ***No*** *(If no, Skip to Section 27.0)*

26.2 Describe where the consent process will take place. Include steps to maximize subjects' privacy.

Response: All participants will come to the research department to be consented. Participants will be placed in a private, screened off area and or room where they can review the consent. Participant questions and or concerns will be address with a member of the study team or research doctor if applicable. The research coordinator will discuss in length the participants requests for privacy of their PHI.

26.3 Describe how you will ensure that subjects are provided with a sufficient period of time to consider taking part in the research study.

NOTE: It is always a requirement that a prospective subject is given sufficient time to have their questions answered and consider their participation. See "SOP: Informed Consent Process for Research (HRP-090)" Sections 5.5 and 5.6.

Response: participants will be made aware that participating in research is completely voluntary, and they may withdraw at any time with no consequence to their routine clinic care. If the patients requires time to decide and or discuss partaking in a research study, the subject will be given said time.

26.4 Describe any process to ensure ongoing consent, defined as a subject's willingness to continue participation for the duration of the research study.

Response: The research coordinator and study team are available to answer any question or concerns with the patient during the duration of the research trial. At each study visit, the patient is asked a series of questions to ensure they are on task with the study visits and feel comfortable. Upon departing from their study visit, the patients are told of their next visit and given detail instruction for their next visit. If study is revised or amendment or new information becomes available about drug safety that may affect patients participation, the patient may be re-consented to ensure patient ongoing consent

26.5 Indicate whether you will be following "SOP: Informed Consent Process for Research (HRP-090)." If not, or if there are any exceptions or additional details to what is covered in the SOP, describe:

- *The role of the individuals listed in the application who are involved in the consent process*
- *The time that will be devoted to the consent discussion*
- *Steps that will be taken to minimize the possibility of coercion or undue influence*
- *Steps that will be taken to ensure the subjects' understanding*

Response:

☒ We have reviewed and will be following "SOP: Informed Consent Process for Research (HRP-090)."

Non-English Speaking Subjects

☒ **N/A:** This study will not enroll Non-English speaking subjects. *(Skip to Section 26.8)*

26.6 Indicate which language(s) other than English are likely to be spoken/understood by your prospective study population or their legally authorized representatives.

NOTE: The response to this Section should correspond with your response to Section 6.4 of this protocol.

Response:

26.7 If subjects who do not speak English will be enrolled, describe the process to ensure that the oral and written information provided to those subjects will be in that language. Indicate the language that will be used by those obtaining consent.

NOTE: Guidance is provided on "SOP: Informed Consent Process for Research (HRP-090)."

Response:

Cognitively Impaired Adults

☒ **N/A**: This study will not enroll cognitively impaired adults. *(Skip to Section 26.9)*

26.8 Describe the process to determine whether an individual is capable of consent.

Response:

Adults Unable to Consent

☒ **N/A**: This study will not enroll adults unable to consent.
(*Skip to Section 26.13)*

When a person is not capable of consent due to cognitive impairment, a legally authorized representative should be used to provide consent (Sections 26.9 and 26.10) ***and, where possible, assent of the individual should also be solicited*** *(Sections 26.11 and 26.12).*

26.9 Describe how you will identify a Legally Authorized Representative (LAR). Indicate that you have reviewed the "SOP: Legally Authorized Representatives, Children, and Guardians (HRP-013)" for research in New York State.

NOTE: Examples of acceptable response includes: verifying the electronic medical record to determine if an LAR is recorded.

Response:

☐

26.10 ***For research conducted outside of New York State****, provide information that describes which individuals are authorized under applicable law to consent on behalf of a prospective subject to their participation in the research. One method of obtaining this information is to have a legal counsel or authority review your protocol along with the definition of "legally authorized representative" in "SOP: Legally Authorized Representatives, Children, and Guardians (HRP-013)."*

Response:

26.11 Describe the process for ***assent of the adults****:*

- *Indicate whether assent will be obtained from all, some, or none of the subjects. If some, indicate which adults will be required to assent and which will not.*

  Response:

- *If assent will not be obtained from some or all subjects, provide an explanation of why not.*

  Response:

26.12 Describe whether ***assent of the adult*** *subjects will be documented and the process to document assent.*

NOTE: The IRB allows the person obtaining assent to document assent on the consent document using the "Template Consent Document (HRP-502)" Signature Block for Assent of Adults who are Legally Unable to Consent.

Response:

Subjects who are not yet Adults (Infants, Children, and Teenagers)

☒ **N/A**: This study will not enroll subjects who are not yet adults. *(Skip to Section 27.0)*

26.13 Describe the criteria that will be used to determine ***whether a prospective subject has not attained the legal age for consent to treatments or procedures involved in the research*** *under the applicable law of the jurisdiction in which the research will be conducted* ***(e.g., individuals under the age of 18 years)****. For research conducted in NYS, review "SOP: Legally Authorized Representatives, Children, and Guardians (HRP-013)" to be aware of which individuals in the state meet the definition of "children."*

NOTE: Examples of acceptable responses include: verification via electronic medical record, driver's license or state-issued ID, screening questionnaire.

Response:

26.14 ***For research conducted outside of New York State****, provide information that describes which persons have not attained the legal age for consent to treatments or procedures involved the research, under the applicable law of the jurisdiction in which research will be conducted. One method of obtaining this information is to have a legal counsel or authority review your protocol along the definition of "children" in "SOP: Legally Authorized Representatives, Children, and Guardians (HRP-013)."*

Response:

26.15 Describe whether parental permission will be obtained from:

Response: N/A

☐ One parent even if the other parent is alive, known, competent, reasonably available, and shares legal responsibility for the care and custody of the child.

☐ Both parents unless one parent is deceased, unknown, incompetent, or not reasonably available, or when only one parent has legal responsibility for the care and custody of the child.

☐ Parent permission will not be obtained. A waiver of parent permission is being requested.

NOTE: The requirement for parent permission is a protocol-specific determination made by the IRB based on the risk level of the research. For guidance, review the "CHECKLIST: Children (HRP-416)."

26.16 Describe whether permission will be obtained from individuals ***other than parents****, and if so, who will be allowed to provide permission. Describe your procedure for determining an individual's authority to consent to the child's general medical care.*

Response:

26.17 Indicate whether assent will be obtained from all, some, or none of the ***children****. If assent will be obtained from some children, indicate which children will be required to assent.*

Response:

26.18 When assent of children is obtained, describe how it will be documented.

Response:

27.0 Waiver or Alteration of Consent Process

Consent will not be obtained, required information will not be disclosed, or the research involves deception.

☒ **N/A:** A waiver or alteration of consent is not being requested.

27.1 If the research involves a waiver or alteration of the consent process, please review the "CHECKLIST: Waiver or Alteration of Consent Process (HRP-410)" to ensure that you have provided sufficient information for the IRB to make the determination that a waiver or alteration can be granted.

NOTE: For records review studies, the first set of criteria on the "CHECKLIST: Waiver or Alteration of Consent Process (HRP-410)" applies.

Response:

27.2 If the research involves a waiver of the consent process for planned emergency research, please review the "CHECKLIST: Waiver of Consent for Emergency Research (HRP-419)" to ensure you have provided sufficient information for the IRB to make these determinations. Provide any additional information necessary here:

Response:

28.0 Process to Document Consent

☐ **N/A:** A Waiver of Consent is being requested.
(Skip to Section 29.0)

28.1 Indicate whether you will be following "SOP: Written Documentation of Consent (HRP-091)." If not or if there are any exceptions, describe whether and how consent of the subject will be obtained including whether or not it will be documented in writing.

NOTE: If your research presents no more than minimal risk of harm to subjects and involves no procedures for which written documentation of consent is normally required outside of the research context, the IRB will generally waive the requirement to obtain written documentation of consent. This is sometimes referred to as 'verbal consent.' Review "CHECKLIST: Waiver of Written Documentation of Consent (HRP-411)" to ensure that you have provided sufficient information.

If you will document consent in writing, attach a consent document with your submission. You may use "TEMPLATE CONSENT DOCUMENT (HRP-502)". If you will obtain consent, but not document consent in writing, attach the script of the information to be provided orally or in writing (i.e. consent script or Information Sheet).

Response:

☒ We will be following "SOP: Written Documentation of Consent" (HRP-091).

29.0 Multi-Site Research (Multisite/Multicenter Only)

☐ **N/A:** This study is not an investigator-initiated multi-site study. This section does not apply.

29.1 If this is a multi-site study ***where you are the lead investigator****, describe the processes to ensure communication among sites, such as:*

- *All sites have the most current version of the IRB documents, including the protocol, consent document, and HIPAA authorization.*
- *All required approvals have been obtained at each site (including approval by the site's IRB of record).*
- *All modifications have been communicated to sites, and approved (including approval by the site's IRB of record) before the modification is implemented.*
- *All engaged participating sites will safeguard data as required by local information security policies.*
- *All local site investigators conduct the study appropriately.*
- *All non-compliance with the study protocol or applicable requirements will be reported in accordance with local policy.*

Response:

29.2 Describe the method for communicating to engaged participating sites:

- *Problems*
- *Interim results*
- *Study closure*

Response:

29.3 Indicate the total number of subjects that will be enrolled or records that will be reviewed across all sites.

Response: 114

29.4 If this is a multicenter study for which UB will serve as the IRB of record, and subjects will be recruited by methods not under the control of the local site (e.g., call centers, national advertisements) describe those methods.

Response: N/A

30.0 Banking Data or Specimens for Future Use

☐ **N/A:** This study is not banking data or specimens for future use or research outside the scope of the present protocol. This section does not apply.

30.1 If data or specimens will be banked (stored) for ***future use, that is, use or research outside of the scope of the present protocol****, describe where the data/specimens will be stored, how long they will be stored, how the data/specimens will be accessed, and who will have access to the data/specimens.*

NOTE: Your response here must be consistent with your response at the "What happens if I say yes, I want to be in this research?" Section of the Template Consent Document (HRP-502).

Response: The study data/specimens will be stored in a locked closet or -80 freezer at the research facility of the Diabetes and Endocrinology Center of WNY for at least 7 years.

The research staff (study personnel including coordinator) only will be authorized to access data and or specimens

30.2 List the data to be stored or associated with each specimen.

Response: Patient ID number, study visit information and date of collection will be stored with specimen. Other data stored will include record files of

all patients participating in the study, including data collection sheets, lab results, CGM and insulin pump data.

30.3 Describe the procedures to release banked data or specimens for future uses, including: the process to request a release, approvals required for release, who can obtain data or specimens, and the data to be provided with specimens.

Response: The copy of the individual patient data collected during the study period will be provided to these individual patients who can choose to hand carry it to their respective physicians and a copy will be faxed to their respective clinical providers upon verbal request from the patient. The data provided will include the insulin pump or CGM data or any of the lab results obtained during the study period. The results of the completed study will be made available to the patients if requested through published manuscript.

31.0 Drugs or Devices

☐ **N/A:** This study does not involve drugs or devices. This section does not apply.

31.1 If the research involves drugs or devices, list and describe all drugs and devices used in the research, the purpose of their use, and their regulatory approval status.

Response: **Drugs used:**

Description or Identity of investigational product(s)

Investigational product	***Dosage form and strength***	***Manufacturer***	***Description***
Dapagliflozin (Farxiga) 5 mg	*Green, plain, diamond shaped, film coated 5 mg tablet*	*AstraZeneca*	*sodium-glucose cotransporter 2 (SGLT2) inhibitor indicated as an adjunct to diet and exercise to improve glycemic control*
Matching placebo for dapagliflozin 5 mg	*Green, plain, diamond shaped, film coated tablet*	*AstraZeneca*	
Semaglutide (Ozempic) 0.25 mg, 0.5 mg, 1.0 mg	*clear, colorless solution that contains 2 mg of semaglutide in a 1.5 mL (1.34 mg/mL) pre-filled, disposable, single-patient-use pen injector*	*Novo Nordisk*	*glucagon-like peptide 1 (GLP-1) receptor agonist indicated as an adjunct to diet and exercise to improve glycemic control*

- *Dapagliflozin and its matching placebo tablets will be supplied in bottles and are provided by Astra Zeneca.*
- *Semaglutide will be supplied as prefilled injectable pens and will be purchased commercially through the University at Buffalo Research Pharmacy.*

Subjects will receive drugs in their unmodified form. Dapagliflozin is a once daily tablet, while Semaglutide is a weekly subcutaneous injection.

Insulin pumps (CSII) and long and short acting insulin is the only diabetes medication accepted before and during this trial. No other diabetes drug is permitted.
Blood pressure or lipid lowering drugs are permitted if on stable dose for at least three months prior to start of trial. Glucagon injection is permitted as rescue medication for severe hypoglycemia. Insulin infusions may be administered by ER or hospital if patients develop DKA.

31.2 Describe your plans to store, handle, and administer those drugs or devices so that they will be used only on subjects and be used only by authorized investigators.

Response: Dapagliflozin 5mg tablets and matching placebo provided by Astra Zeneca will be stored in a locked cabinet in research pharmacy. Semaglutide provided by University at Buffalo research pharmacy will be stored in temperature controlled refrigerator at 4C at the research facility of the Diabetes and Endocrinology Center of WNY at 1000 Youngs Rd until dispensed to patients. Investigational drugs will be dispensed by PI or CO-Is only to study patients according to randomization plan.

If the drug is investigational (has an IND) or the device has an IDE or a claim of abbreviated IDE (non-significant risk device), include the following information:

31.3 Identify the holder of the IND/IDE/Abbreviated IDE.

Response: IND application for use of all drugs (dapagliflozin and semaglutide) in type 1 diabetes will be submitted to FDA and no study procedure will start before all local and federal approvals are obtained. FDA has granted IND for usage of dapagliflozin in type 1 diabetes. We will seek FDA approval for the use of semaglutide in this study. These IND letters will be submitted to IRB and JDRF along with final version of protocol before initiating any study activities.

31.4 Explain procedures followed to comply with FDA sponsor requirements for the following:

	Applicable to:		
FDA Regulation	***IND Studies***	***IDE studies***	***Abbreviated IDE studies***
21 CFR 11	***X***	***X***	
21 CFR 54	***X***	***X***	
21 CFR 210	***X***		
21 CFR 211	***X***		
21 CFR 312	***X***		
21 CFR 812		***X***	***X***
21 CFR 820		***X***	

Response: All FDA sponsor requirements have been reviewed and will be followed during the study procedures

32.0 Humanitarian Use Devices

☒ **N/A:** This study does not involve humanitarian use devices. This does not apply.

32.1 For Humanitarian Use Device (HUD) uses provide a description of the device, a summary of how you propose to use the device, including a description of any screening procedures, the HUD procedure, and any patient follow-up visits, tests or procedures.

Response:

32.2 For HUD uses provide a description of how the patient will be informed of the potential risks and benefits of the HUD and any procedures associated with its use.

Response:

US TTT1 Protocol Summary of changes V5 to V6

Section	Original	New	Justification	Consent changes
9.1,9.2,9.3	No statement	Language to indicate use of researchmatch.org, Buffalo Research Registry, participate in research (PIR) and TriNetX/I2B2 platforms was added to these sections	To improve enrollment into this study we are adding language and attachments to recruit through researchmatch.org, Buffalo Research Registry, participate in research (PIR) and TriNetX/I2B2 platforms.	NO

University at Buffalo
The State University of New York

University at Buffalo Institutional Review Board (UBIRB)
Office of Research Compliance | Clinical and Translational Research Center Room 5018
875 Ellicott St. | Buffalo, NY 14203
UB Federalwide Assurance ID#: FWA00008824

Complete Research Protocol (HRP-503)

Table of Contents

Template Instructions........ 2
1.0 Objectives........ 4
2.0 Scientific Endpoints........ 5
3.0 Background........ 6
4.0 Study Design........ 12
5.0 Local Number of Subjects........ 12
6.0 Inclusion and Exclusion Criteria........ 13
7.0 Vulnerable Populations........ 15
8.0 Eligibility Screening........ 17
9.0 Recruitment Methods........ 17
10.0 Procedures Involved........ 20
11.0 Study Timelines........ 32
12.0 Setting........ 32
13.0 Community-Based Participatory Research........ 33
14.0 Resources and Qualifications........ 34
15.0 Other Approvals........ 35
16.0 Provisions to Protect the Privacy Interests of Subjects........ 35
17.0 Data Management and Analysis........ 36
18.0 Confidentiality........ 39
A. Confidentiality of Study Data........ 39
B. Confidentiality of Study Specimens........ 40
19.0 Provisions to Monitor the Data to Ensure the Safety of Subjects........ 41
20.0 Withdrawal of Subjects........ 48
21.0 Risks to Subjects........ 49
22.0 Potential Benefits to Subjects........ 55
23.0 Compensation for Research-Related Injury........ 55
24.0 Economic Burden to Subjects........ 56
25.0 Compensation for Participation........ 57
26.0 Consent Process........ 57
27.0 Waiver or Alteration of Consent Process........ 62
28.0 Process to Document Consent........ 63
29.0 Multi-Site Research (Multisite/Multicenter Only)........ 63
30.0 Banking Data or Specimens for Future Use........ 64
31.0 Drugs or Devices........ 65
32.0 Humanitarian Use Devices........ 67

Template Instructions

Sections that do not apply:

- *In several sections, the addition of checkboxes for* ***Not Applicable*** *have been added to the template as responses.*
    - *If an N/A checkbox is present, select the appropriate justification from the list.*
    - *If an N/A checkbox is not present, or if none of the existing checkboxes apply to your study, you must write in your own justification.*
- *In addition:*
    - *For research where the only study procedures are records/chart review: Sections 19, 20, 22, 23, 24, 25, 31, and 32 do not apply.*
    - *For exempt research: Sections 31 and 32 do not apply.*

Studies with multiple participant groups:

- *If this study involves multiple participant groups (e.g. parents and children), provide information in applicable sections for each participant group. Clearly label responses when they differ. For example:*

Response:<br><u>Intervention Group:</u><br><u>Control Group:</u>

Formatting:

- *Do not remove template instructions or section headings when they do not apply to your study.*

If you are pasting information from other documents using the "Merge Formatting" Paste option will maintain the formatting of the response boxes.

Amendments:

- *When making modifications or revisions to this and other documents, use the* ***Track Changes*** *function in Microsoft Word.*
- *Update the version date or number* ***on Page 3.***

PROTOCOL TITLE:

Include the full protocol title.

Response: **Triple Therapy for Type 1 Diabetes with Insulin, Semaglutide, and Dapagliflozin**

PRINCIPAL INVESTIGATOR:

Response:

**Principal Investigator:**	**Paresh Dandona, MBBS, PhD**
**Co- Investigators:**	**Husam Ghanim, PhD**
	**Antoine Makdissi, MD**
	**Manav Batra, MD**
**Co- Principal Investigator:**	**John R Petrie, MBChB, PhD**

Paresh Dandona, M.B.B.S., Ph.D., F.R.C.P, F.A.C.P, F.A.C.C, FACE,
SUNY Distinguished Professor,
Director, Diabetes-Endocrinology Center of Western NY
Chief of Endocrinology, Diabetes, Endocrinology and Metabolism, SUNY at Buffalo
1000 Youngs Road, Suite 105
Williamsville NY 14221
Telephone: (716) 535-1850
Fax: 716-639-1200
E-mail: pdandona@kaleidahealth.org

CO-PI:

Professor John R Petrie
BSc MBChB PhD FRCP(Ed) FRCPSG
Professor of Diabetic Medicine
Institute of Cardiovascular and Medical Sciences
BHF Glasgow Cardiovascular Research Centre
University of Glasgow
126 University Place
Glasgow G12 8TA
Tel: +44(0)141 330 3325
Email: john.petrie@glasgow.ac.uk
Web: http://www.gla.ac.uk/researchinstitutes/icams/staff/johnpetrie/
The University of Glasgow

VERSION:

Include the version date or number.

Response: 12/10/2023, V6

GRANT APPLICABILITY:

Indicate whether this protocol is funded by a grant (e.g. NIH, foundation grant). For a grant with multiple aims, indicate which aims are covered by this research proposal.

NOTE: This question does not apply to studies funded by a sponsor contract.

Include a copy of the grant proposal with your submission.

Response: Research support provided by JDRF and AZ

RESEARCH REPOSITORY:

Indicate where the research files will be kept, including when the study has been closed. The repository should include, at minimum, copies of IRB correspondence (approval, determination letters) as well as signed consent documents. This documentation should be maintained for 3 years after the study has been closed.

Response:
Site 1: Diabetes Endocrinology Research Center of WNY
Address: 1000 Youngs Road, Suite 105, Williamsville NY 14221
Department: Diabetes Endocrinology

Site 2: University of Glasgow. Institute of Cardiovascular and Medical Sciences University of Glasgow 126 University Place Glasgow, G12 8TA

1.0 Objectives

1.1 Describe the purpose, specific aims, or objectives of this research.

Response:

Primary Objective: To assess whether the addition of dapagliflozin to semaglutide and insulin (triple therapy) improves glycemic control in patients with type 1 diabetes compared with semaglutide and insulin (dual therapy) and insulin only (standard) treatment.

General hypothesis: The addition of dapagliflozin to semaglutide and insulin treatment improves glycemic control, weight loss and blood pressure in adults with type 1 diabetes.

1.2 State the hypotheses to be tested, if applicable.

NOTE: A hypothesis is a specific, testable prediction about what you expect to happen in your study that corresponds with your above listed objectives.

Response:

Specific hypotheses:

Primary: combined dapagliflozin and semaglutide treatment in addition to insulin will improve HbA1c levels compared with semaglutide in addition to insulin .

Secondary 1: semaglutide in addition to insulin treatment over 26 weeks will improve HbA1c levels compared with insulin therapy alone.

Secondary 2: combined dapagliflozin and semaglutide treatment in addition to insulin will improve HbA1c levels compared to insulin therapy alone at 52 weeks.

Secondary 3: combined dapagliflozin and semaglutide treatment in addition to insulin will improve CGM-based weekly mean and fasting blood glucose, glycemic oscillations and percent of time in range (70-180mg/dl) with lower insulin requirements compared with semaglutide and insulin (dual therapy) or insulin only (standard) treatment.

Secondary : combined dapagliflozin and semaglutide in addition to insulin therapy will reduce systolic and diastolic blood pressure compared with semaglutide and insulin (dual therapy) or insulin only (standard) treatment.

Secondary 5: combined dapagliflozin and semaglutide in addition to insulin therapy (triple therapy) will result in weight loss compared to dual and standard therapy.

Secondary 6: combined dapagliflozin and semaglutide in addition to insulin therapy will result in improved scores on the Diabetes Specific Quality of Life Scale (DSQOLS) and the Problem Areas In Diabetes (PAID) survey compared with dual and standard therapy.

Tertiary and Exploratory Objectives (EO) for future ancillary studies (PLEASE NOTE: For EO, samples will be collected to facilitate the measurements of these end points in future ancillary studies once submitted and approved by JDRF so cost of assays is not included in the current budget): The addition of dapagliflozin to semaglutide and insulin in type 1 diabetes will:

Tertiary 1: combined dapagliflozin and semaglutide treatment in addition to insulin therapy will improve fasting and postprandial glucose excursions and suppress postprandial glucagon, FFA, GLP-1 compared with semaglutide and insulin or insulin therapy alone.

Tertiary 2: semaglutide in addition to insulin treatment over 52 weeks will improve HbA1c levels compared with insulin therapy alone.

EO 1: increase plasma concentrations of vasodilatation mediators and reduce plasma concentrations of vasoconstriction mediators compared to dual and standard therapy groups.

EO 2: exert a systemic anti-inflammatory effect including reducing mRNA expression of inflammatory mediators and NFκB binding in peripheral mononuclear cells (MNC) and adipose tissue (optional) and their circulating levels in plasma while increasing levels of IL-RA and adiponectin.

2.0 Scientific Endpoints

2.1 Describe the scientific endpoint(s), the main result or occurrence under study.

NOTE: Scientific endpoints are outcomes defined before the study begins to determine whether the objectives of the study have been met and to draw conclusions from the data. Include primary and secondary endpoints. Some example endpoints are: reduction of

*symptoms, improvement in quality of life, or survival. Your response should **not** be a date.*

Response:

Primary endpoint: Change in HbA1c at 6 months following dapagliflozin or placebo therapy in addition to combined semaglutide and insulin treatment.

Efficacy Secondary endpoints:

1. Change in HbA1c from baseline at 12 months in triple therapy group compared to insulin only group.
2. Change in HbA1c from baseline at 6 months in dual therapy group compared to insulin only group.
3. Change in percent Time in Range (70– 180 mg/dl), percent time in hyperglycemia Level 1(180-250mg/dl) and Level 2 (>250mg/dl) and percent time in hypoglycemia Level 1 (54-70mg/dl) as assessed by CGM
4. Other indices of glycemic control: fructosamine, weekly mean and fasting glucose, standard deviation, insulin requirements.
5. Change in body weight and quality of life as assessed by DSQOLS and PAID at 6 and 12 months.
6. Anti-hypertensive effects including change in systolic and diastolic BP, numbers of BP medications required.

Safety Secondary endpoints:

1- Differences in rates of hypoglycemic events Level 2 (<54mg/dl) and Level 3 (A severe event characterized by altered mental and/or physical status requiring assistance) between investigative arms and standard therapy arm.

2- *Differences in rates of diabetic ketoacidosis defined as elevated serum or urine ketones (greater than the upper limit of the normal range), and serum bicarbonate <15 mmol/L or Blood pH <7.3 between investigative arms and standard therapy arm.*

Tertiary/ exploratory endpoints: (items 2-4 to be performed in future on collected samples from this study if additional funding is approved by JDRF):

1- Fasting and postprandial (Area Under curve) glucose excursions, FFA, GLP-1 and glucagon and fasting fructosamine.

2- Indices of inflammatory stress. These will be CRP, FFA, SAA, IL-1β, IL-18, IL-1RA, adiponectin and TNFα concentrations in plasma and isoprostane in urine.

3- Expression of IL-1β, TNFα, IL-12, TLR2, TLR4, adiponectin, IκBα as well as NFκB binding in peripheral MNC and adipose tissue (optional).

4- Indices of vasodilatation and vasoconstriction: ANP, b-NP, cGMP, cAMP, angiotensinogen, renin and angiotensin II concentrations.

3.0 Background

3.1 Provide the scientific or scholarly background, rationale, and significance of the research based on the existing literature and how it will contribute to existing knowledge. Describe any gaps in current knowledge. Include relevant preliminary findings or prior research by the investigator.

Response:

The control of glucose homeostasis in type 1 diabetes is fragile since β-cell reserve is negligible. Traditionally, insulin has been the only treatment approach available. This approach, often leads to erratic blood sugar control given the challenge of matching exogenous insulin to mealtime glucose excursions in the face of a lack of any endogenous insulin to compensate for variation in requirements in response to meals and activity levels. Injected insulin boluses rarely match precise insulin requirements either in terms of dose administered or bio-availability. The result often is wide fluctuations in blood glucose readings. Based on our work to date and other published reports, previous attempts to add other glucose-lowering medications to insulin therapy yielded minimal benefits on glycemia (although metformin may have other benefits (1-3)

We have recently shown in two preliminary retrospective studies that the addition of liraglutide, a Glucagon Like Peptide -1 receptor agonist (GLP-1RA), to insulin results in consistent improvements in glycemic control in type 1 diabetes as demonstrated by reductions in HbA1c, glycemic excursions, and insulin requirements accompanied by improvements in body weight and systolic blood pressure (4, 5). We have now also completed the first prospective randomized controlled trial of this drug in 72 people with type 1 diabetes. Our data not only confirmed improvements in glycemic control, HbA1c, body weight and systolic BP, but also demonstrated a reduction in appetite, carbohydrate intake in low grade inflammation as measured by plasma CRP concentrations (6). Importantly, quality of life was also improved. Our recent studies in type 2 diabetes have revealed that systolic blood pressure reduction with GLP-1RA (exenatide) treatment in type 2 diabetes is associated with increased plasma concentrations of ANP, cGMP and cAMP along with reduced concentrations of angiotensinogen, renin and angiotensin II (7). It is possible that these mechanisms play a role in the prevention of cardiovascular complications in type 1 diabetes.

While GLP-1RAs, therefore, have great potential as adjunct therapy for type 1 diabetes, mean HbA1c was still ≥7% after liraglutide treatment (6) and larger pharma-sponsored trials reported increased rates of hypoglycemia (8, 9). In seeking further improvement, the advent of sodium-glucose transport protein-2 (SGLT2) inhibitors is of importance since their mode of action is independent of insulinogenesis. Introduced for use in type 2 diabetes, SGLT2 inhibitors are selective antagonists of the sodium-glucose transporter subtype expressed in the proximal convoluted tubules of the kidneys. They act by inhibiting renal glucose re-absorption and therefore stimulating excretion of 60-100 grams per day of glucose in urine. SGLT2 inhibitors have been studied extensively in type 2 diabetes, including the landmark cardiovascular outcome trial EMPA-REG (10), but data have only quite recently begun to accumulate in type 1(11). Short term effects on urine glucose excretion of remogliflozin and empagliflozin are similar to those seen in type 2 diabetes and these translate into improved glycemic and other indices including HbA1c; fasting blood glucose, rates of hypoglycemia, daily insulin dose and weight (12). Glucose-lowering efficacy is attenuated in individuals with eGFR $<45/ml/min/1.73m^2$(13). Therefore, use of dapagliflozin is recommended for patients with eGFR$>45/ml/min/1.73\ m^2$ (as per prescribing information). Weight loss accompanying increased urinary glucose excretion could translate into reduced cardiovascular complications as in type 2 diabetes, but there are no data so far available to support this hypothesis. More recently, in the Phase 3 trial DEPICT-1 we showed that dapagliflozin improves glycemic control and HbA1c over six months by about 0.45% with no severe hypoglycemia or diabetic ketoacidosis

(14). Similarly, sotagliflozin has been shown in a Phase 3 trial to allow a larger proportion of patients to achieve HbA1c lower than 7.0% with no severe hypoglycemia although the rate of diabetic ketoacidosis was higher in the sotagliflozin group (15). Both products are now being reviewed by regulatory agencies for the type 1 diabetes indication.

On the basis of the above, we hypothesize that adding an SGLT2 inhibitor to a combination of insulin and semaglutide (a weekly and potent GLP-1RA) will improve glycemic control in type 1 diabetes such that HbA1c levels of ≤7% are achieved more consistently. Our protocol is designed to allow an objective assessment of whether this triple therapy combination will work effectively and safely so that a majority of people with type 1 diabetes can reach a glycemic target HbA1c of ≤7%. In addition, since both semaglutide and dapagliflozin inhibitors reduce blood pressure and weight, it will allow an assessment of the combined effect of GLP-1RAs and SGLT2 inhibitors on these outcomes. Since there are no previous data on the effects of once weekly semaglutide or the combination of semaglutide and dapagliflozin compared to standard insulin treatment in type 1 diabetes, the design requires allocation of some participants to insulin therapy only.

Due to the potential ketogenic effect of SGLT-2 inhibitors, data from SGLT-2 inhibitors studies in type 1 diabetes were analyzed for it ketogenic effects and a consensus statement and guidelines on management of patients was recently published (Danne T et al., Diabetes Care. 2019 Feb 6).

Preliminary data

1) Liraglutide treatment in patients with type 1 diabetes (4): We treated 8 type 1 diabetes patients; mean age (38.5 years) and duration of diabetes (20±4 yrs), with liraglutide in addition to insulin for 24 weeks. As shown in table 1, there was a significant reduction in fasting and mean blood glucose concentrations, insulin doses, HbA1c and a reduction in appetite and caloric intake with weight loss. In addition, there was a reduction in the excursions of blood glucose concentrations, i.e., the standard deviations of blood glucose concentrations were markedly reduced (**figure 1**). The changes were observed within 48h and improved marginally thereafter with fine tuning of insulin doses and a further reduction in these doses. These observations are noteworthy in that study participants were well controlled and meticulous users of CSII and CGM. They had all tried hard to improve their diabetic control by adjusting insulin doses but had not succeeded because of hypoglycemia. We then demonstrated that poorly controlled and obese individuals with type 1 diabetes also experienced similar benefits of liraglutide treatment (2).

2) Liraglutide As Additional Treatment to Insulin in Patients with Type 1 Diabetes Mellitus: A Randomized Clinical Trial (6): Following the two above non-randomized studies we recently conducted a randomized clinical trial in which 72 individuals (Placebo=18; liraglutide = 54) with TYPE 1 DIABETES for at least one year, on insulin therapy and with no detectable C-peptide in plasma (mean BMI: 30±1; mean body weight: 84±2 kg ; mean HbA1c: 7.57±0.09%; mean age: 44±2 years; mean age of TYPE 1 DIABETES diagnosis: 20±1 years) were randomized to receive placebo, 0.6, 1.2 or 1.8mg of liraglutide daily for 12 weeks. In the 1.2 mg and 1.8 mg groups, mean change in average blood glucose was -10±2 and -10.0±1mg/dl, respectively, ($p<0.0001$ vs. placebo). In the 1.2mg group, HbA1c fell by 0.78% from 7.84±0.17% to 7.06±0.15% ($p<0.0001$, $p<0.01$

vs. placebo) and in the 1.8mg group fell by 0.42% from 7.41±0.15 to 6.99±0.15 ($p=0.001$, $p=0.39$ vs placebo). Time spent between 70 to 160 mg/dl increased by 5±1% ($p<0.05$ vs placebo) and time between 160-400 mg/dl decreased by 7±1% ($p<0.01$ vs placebo) in the 1.8 mg group with no additional hypoglycemia. During the 12 week period, the average total daily dose of insulin in the two groups fell by 12.4±3.9 and 10.0±2.3 units respectively ($p<0.05$ vs baseline and placebo). There was a reduction in body weight (from 83±4 to 78±5 kg, $p<0.0001$, in the 1.8mg group, from 96±4 to 91±4 kg, $p<0.001$, in the 1.2 mg group and from 80±4 to 77±4 kg, $p<0.01$ in the 0.6 mg group). A reduction in daily carbohydrate intake (171±17g vs. 127±18g; $p<0.01$ and 153±18g vs 115±16g, $p<0.01$ in the 1.2 mg and 1.8 mg groups, respectively) was also observed over 12 weeks. Systolic BP (SBP) in the 1.8 mg group fell by 3 mmHg (120±2 to 117±3 mmHg, $p=0.01$). CRP fell by 15±6 and 19±8% in the 1.2 and 1.8mg groups, respectively ($p<0.05$). Quality of life improved significantly compared to baseline and placebo (unpublished data).

3) Dapagliflozin as an Additional Treatment to Insulin and Liraglutide in Patients with Type 1 Diabetes Mellitus (16): The objective of this randomized clinical trial was to investigate whether addition of dapagliflozin to insulin and liraglutide results in a significant reduction in glycemia and body weight. 30 participants with TYPE 1 DIABETES on liraglutide therapy for at least last 6 months were randomized (in 2:1 ratio) to receive either dapagliflozin 10 mg or placebo daily for 12 weeks. With 12 weeks of dapagliflozin HbA1c fell by 0.66±0.08% from 7.8±0.21% ($p < .01$ vs placebo) but did not change significantly with placebo (7.40±0.20% to 7.30±0.20%). In one patient within one week of addition of dapagliflozin average glucose fell from 170 to 122 mg/dl while standard deviation was reduced from 71 to 58 (**Figure 2-diminished glycemic excursions)**. Body weight fell by1.9 +/- 0.54kg ($p < 0.05$ vs placebo). There was no additional hypoglycemia (blood glucose < 3.88 mmol/L; $p = 0.52$ vs placebo). In the dapagliflozin group, there were significant increases in the plasma concentrations of glucagon by 35% +/- 13% ($p < 0.05$), hormone-sensitive lipase by 29% +/- 11% ($p < 0.05$), free fatty acids by 74% +/- 32% ($p < 0.05$), acetoacetate by 67% +/- 34% ($p < 0.05$), and beta-hydroxybutyrate by 254% +/- 81% ($p < 0.05$). Urinary ketone levels also increased significantly ($P < .05$). None of these changes was observed in the placebo group. Two individuals in the dapagliflozin group developed ketoacidosis. Overall, addition of dapagliflozin to insulin and liraglutide in patients with TYPE 1 DIABETES resulted in a significant improvement in glycemia and in weight loss but tended to increase ketosis.

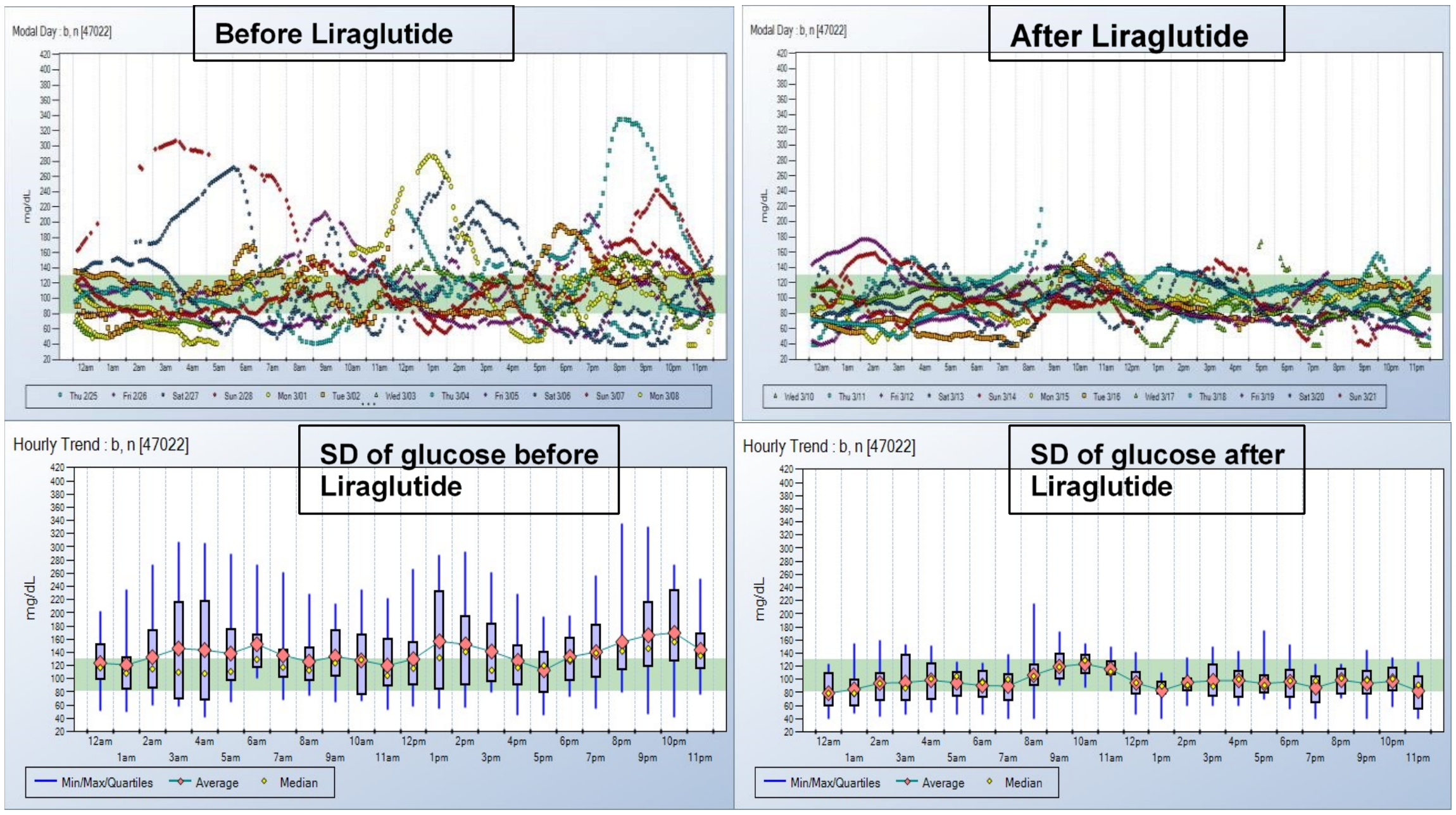

Figure 1: Weekly continuous glucose monitoring record of one patient prior to and following 1 week of liraglutide. Note the glycemic variability (SD) and the frequency of glucose concentrations within the target range prior to and following liraglutide

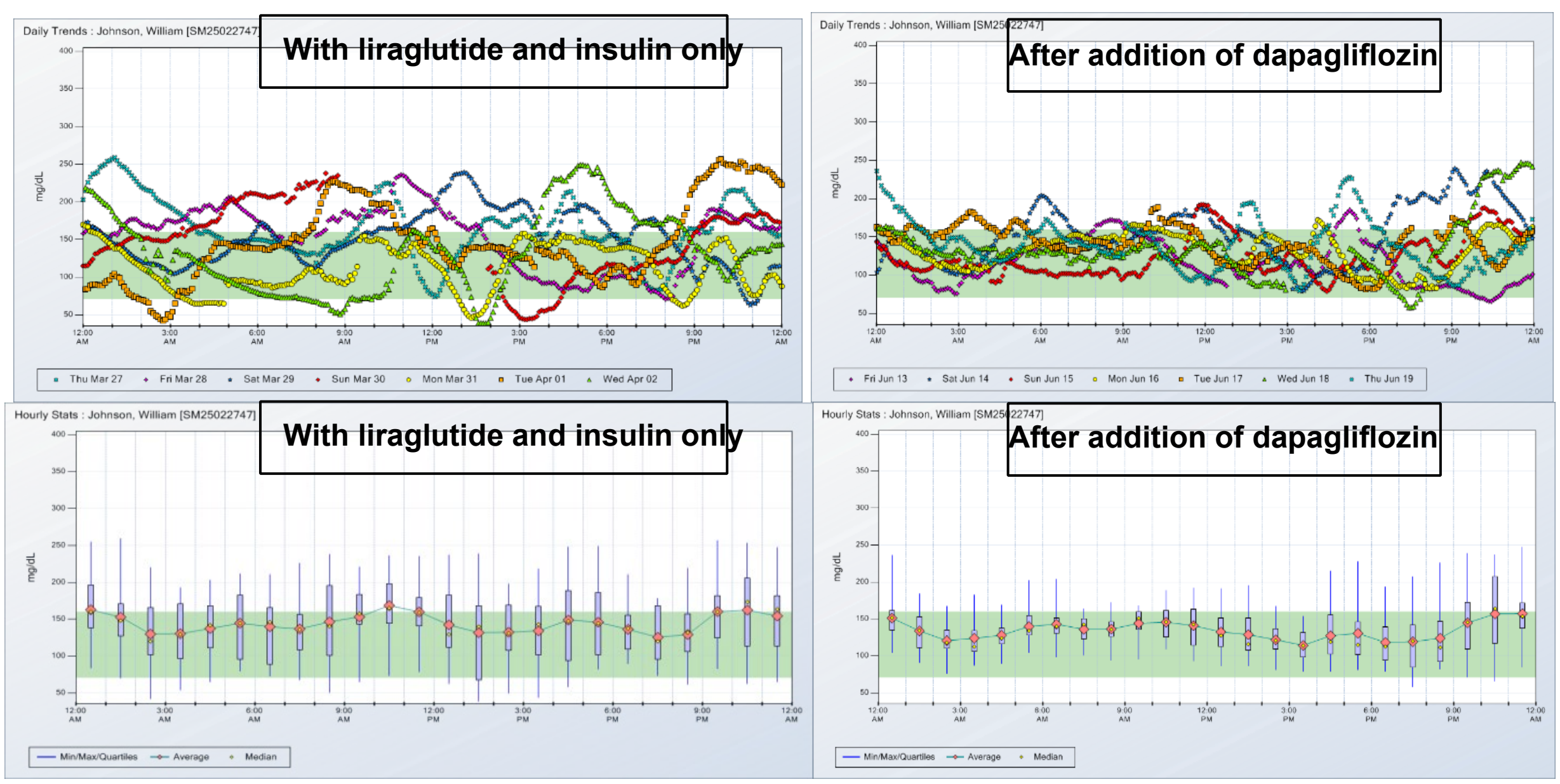

Figure 2: Weekly continuous glucose monitoring record of one patient prior to and following 8 weeks of the triple therapy combination with dapagliflozin and liraglutide. Note the glycemic variability (SD) (bottom) and the frequency of glucose concentrations within the target range prior to and following dapagliflozin addition.

3.2 Include complete citations or references.

Response:

1. Konrad K, Datz N, Engelsberger I, Grulich-Henn J, Hoertenhuber T, Knauth B, et al. Current use of metformin in addition to insulin in pediatric patients with type 1 diabetes mellitus: an analysis based on a large diabetes registry in Germany and Austria. *Pediatr Diabetes.* 2014.
2. Vella S, Buetow L, Royle P, Livingstone S, Colhoun HM, and Petrie JR. The use of metformin in type 1 diabetes: a systematic review of efficacy. *Diabetologia.* 2010;53(5):809-20.
3. Vella S, Buetow L, Royle P, Livingstone S, and Petrie JR. Metformin in type 1 diabetes reduces insulin requirements without significantly improving glycaemic control. Reply to Schatz H [letter]. *Diabetologia.* 2011;54(1):203-4.
4. Varanasi A, Bellini N, Rawal D, Vora M, Makdissi A, Dhindsa S, et al. Liraglutide as additional treatment for type 1 diabetes. *Eur J Endocrinol.* 2011;165(1):77-84.
5. Kuhadiya ND, Malik R, Bellini NJ, Patterson JL, Traina A, Makdissi A, et al. Liraglutide as additional treatment to insulin in obese patients with type 1 diabetes mellitus. *Endocrine practice : official journal of the American College of Endocrinology and the American Association of Clinical Endocrinologists.* 2013;19(6):963-7.
6. Kuhadiya ND, Dhindsa S, Ghanim H, Mehta A, Makdissi A, Batra M, et al. Addition of Liraglutide to Insulin in Patients With Type 1 Diabetes: A Randomized Placebo-Controlled Clinical Trial of 12 Weeks. *Diabetes Care.* 2016;39(6):1027-35.
7. Chaudhuri A, Ghanim H, Makdissi A, Green K, Abuaysheh S, Batra M, et al. Exenatide induces an increase in vasodilatory and a decrease in vasoconstrictive mediators. *Diabetes Obes Metab.* 2017;19(5):729-33.
8. Mathieu C, Zinman B, Hemmingsson JU, Woo V, Colman P, Christiansen E, et al. Efficacy and Safety of Liraglutide Added to Insulin Treatment in Type 1 Diabetes: The ADJUNCT ONE Treat-To-Target Randomized Trial. *Diabetes Care.* 2016;39(10):1702-10.
9. Ahren B, Hirsch IB, Pieber TR, Mathieu C, Gomez-Peralta F, Hansen TK, et al. Efficacy and Safety of Liraglutide Added to Capped Insulin Treatment in Subjects With Type 1 Diabetes: The ADJUNCT TWO Randomized Trial. *Diabetes Care.* 2016;39(10):1693-701.
10. Zinman B, Wanner C, Lachin JM, Fitchett D, Bluhmki E, Hantel S, et al. Empagliflozin, Cardiovascular Outcomes, and Mortality in Type 2 Diabetes. *N Engl J Med.* 2015;373(22):2117-28.
11. Petrie JR. SGLT2 inhibitors in type 1 diabetes: knocked down, but up again? *Lancet Diabetes Endocrinol.* 2017;5(11):841-3.
12. Mudaliar S, Armstrong DA, Mavian AA, O'Connor-Semmes R, Mydlow PK, Ye J, et al. Remogliflozin etabonate, a selective inhibitor of the sodium-glucose transporter 2, improves serum glucose profiles in type 1 diabetes. *Diabetes Care.* 2012;35(11):2198-200.
13. Kohan DE, Fioretto P, Tang W, List JF. Long-term study of patients with type 2 diabetes and moderate renal impairment shows that dapagliflozin reduces weight and blood pressure but does not improve glycemic control. Kidney Int. 2014 Apr;85(4):962-71.

14. Dandona P, Mathieu C, Phillip M, Hansen L, Griffen SC, Tschope D, et al. Efficacy and safety of dapagliflozin in patients with inadequately controlled type 1 diabetes (DEPICT-1): 24 week results from a multicentre, double-blind, phase 3, randomised controlled trial. *Lancet Diabetes Endocrinol.* 2017;5(11):864-76.
15. Garg SK, Henry RR, Banks P, Buse JB, Davies MJ, Fulcher GR, et al. Effects of Sotagliflozin Added to Insulin in Patients with Type 1 Diabetes. *N Engl J Med.* 2017;377(24):2337-48.
16. Kuhadiya ND, Ghanim H, Mehta A, Garg M, Khan S, Hejna J, et al. Dapagliflozin as Additional Treatment to Liraglutide and Insulin in Patients With Type 1 Diabetes. *J Clin Endocrinol Metab.* 2016;101(9):3506-15.

17. Andrews NC, and Faller DV. A rapid micropreparation technique for extraction of DNA-binding proteins from limiting numbers of mammalian cells. *Nucleic Acids Res.* 1991;19(9):2499.

4.0 Study Design

4.1 Describe and explain the study design (e.g. case-control, cross-sectional, ethnographic, experimental, interventional, longitudinal, observational).

Response: This will be a two-center, randomized, open-label then double-blind, parallel group, placebo and standard of care controlled prospective study in type 1 diabetes. One hundred and (114) patients will be randomized (open label, see below) into three groups (38 each) and will be either maintained on their regular insulin therapy for 12 months (standard therapy group) or started on semaglutide in addition to regular insulin treatment for six month (dual therapy and triple therapy groups). For those on insulin and semaglutide, after six months of stable treatment, dapagliflozin (triple therapy group) or placebo (dual therapy group) treatment will be initiated for an additional six months according to a second randomization (double-blind).

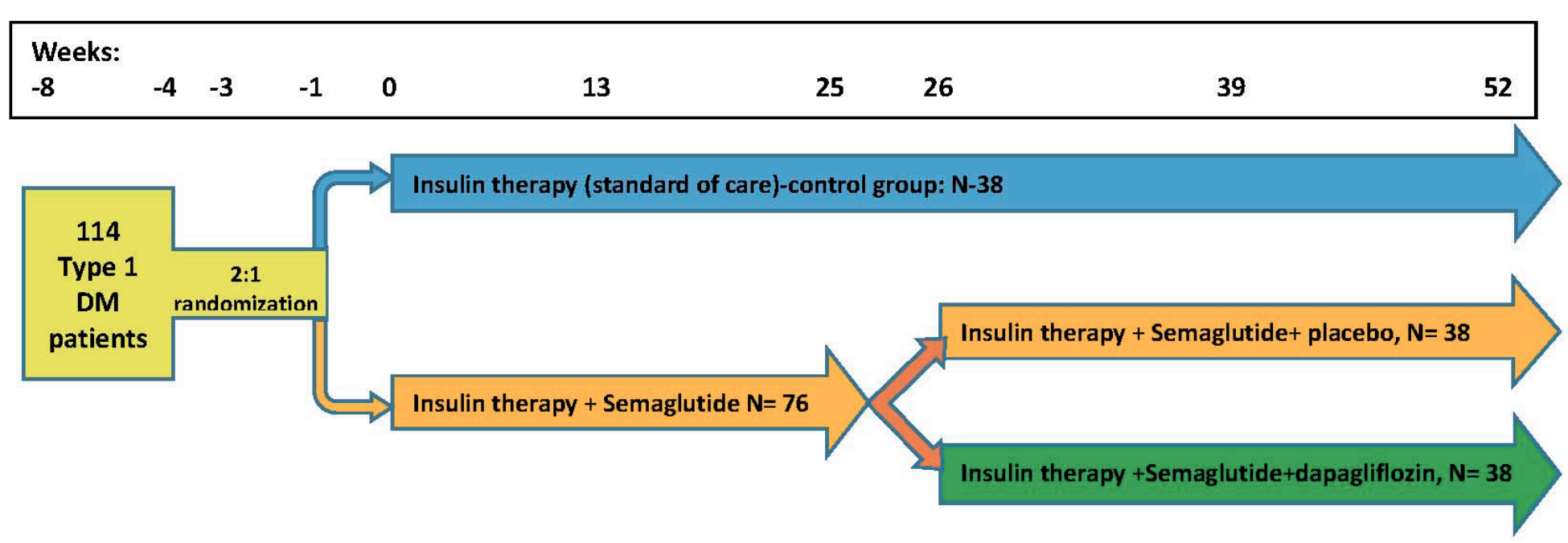

5.0 Local Number of Subjects

5.1 Indicate the total number of subjects that will be enrolled or records that will be reviewed locally.

Response: 57 patients will be enrolled locally with another 57 patients recruited at the University of Glasgow site: a total of 114.

5.2 If applicable, indicate how many subjects you expect to screen to reach your target sample (i.e. your screen failure rate).

Response: All screened and qualified patients will be enrolled and randomized

5.3 Justify the feasibility of recruiting the proposed number of eligible subjects within the anticipated recruitment period. For example, how many potential subjects do you have access to? What percentage of those potential subjects do you need to recruit?

Response: The Diabetes and Endocrinology Center of WNY is the largest Diabetes center in the WNY area, seeing between 60 to 100 type 1 diabetic patient every month. Therefore, the majority of recruited patients are our clinic patients. We also recruit a study participants through advertisement and other physician's references. These sources will suffice to recruit the needed number to subjects. The Glasgow center (Stobhill Ambulatory Care Hospital) reviews more than 300 patients per month.

6.0 Inclusion and Exclusion Criteria

*6.1 Describe the criteria that define who will be **included** in your final study sample.*

NOTE: This may be done in bullet point fashion.

Response:

Inclusion Criteria

1) Type 1 Diabetes for at least 1 year on staple use of continuous subcutaneous insulin infusion (CSII) or multiple daily (four or more) injections (MDI) of insulin for last 3 months.
2) C-peptide <0.23 nM
3) Minimum dose of insulin in Units/kg at entry: 0.5 U/kg for MDI and 0.4 U/kg for CSII
4) Regularly measuring blood sugars four or more times daily or use of CGM.
5) HbA1c between7.5-11% inclusive.
6) Well versed in CHO counting*
7) Age 18-70 years.
8) BMI ≥25 kg/m^2.

*In the opinion of the site Principal Investigator

*6.2 Describe the criteria that define who will be **excluded** from your final study sample.*

NOTE: This may be done in bullet point fashion.

Response:

Exclusion Criteria

1) Type 1 diabetes for less than 12 months, type 2 diabetes, chronic pancreatitis, MODY
2) Previous use of any agent other than insulin for treatment of diabetes in the last 3 months.
3) History of diabetic ketoacidosis (DKA) requiring medical intervention (e.g., emergency room visit and/or hospitalization) within 3 months prior to the screening visit
4) Patients on ketogenic or low (<50g/day) carbohydrate diet or who practice intermittent fasting.
5) Frequent episodes of severe hypoglycemia as defined by more than one episode requiring medical assistance, emergency care (paramedics or emergency room care), and/or glucagon therapy administered by a third-party individual within 1 month prior to the screening visit
6) Symptoms of poorly controlled diabetes that would preclude participation in this trial
7) Subjects on a commercial weight loss program with ongoing weight loss, or on an intensive exercise program
8) History of bariatric surgery or lap-band procedure within 12 months prior to screening
9) History of Addison's disease or chronic adrenal insufficiency
10) History of diabetes insipidus
11) Hepatic disease or cirrhosis
12) Aspartate Aminotransferase (AST) > 3X Upper limit of normal (ULN) *and/or* Alanine aminotransferase (ALT) > 3X ULN
13) Serum Total Bilirubin > 2X ULN unless exclusively caused by Gilbert's Syndrome
14) Hemoglobin < 11.0 g/dL (110 g/L) for men; hemoglobin < 10.0 g/dL (100 g/L) for women.
15) Coronary event or procedure (myocardial infarction, unstable angina, coronary artery bypass, surgery or coronary angioplasty) in the previous 3 months or patients with congestive heart failure.
16) ESRD on hemodialysis; and or e-GFR < 45 ml/min/1.73m^2
17) HIV or Hepatitis B/C positive status
18) Any other life-threatening, noncardiac disease
19) History of pancreatitis
20) Women who are pregnant or women of childbearing potential who are not using adequate contraception or who are breast feeding
21) Inability to give informed consent
22) History of gastroparesis
23) History of medullary thyroid carcinoma or MEN 2 syndrome
24) History of serious hypersensitivity reaction to these agents
25) Painful gallstones
26) Alcoholism
27) Hypertriglyceridemia (>500 mg/dl)

28) Recurrent genital mycotic infection.
29) Hypovolemic patients or with chronic renal insufficiency.
30) Patients with any malignancy except treated in situ malignancy and basal cell carcinoma of the skin
31) Unexplained hematuria
32) Patients with a history of diabetic retinopathy
33) Use of an investigational agent or therapeutic regimen within 30 days of study
34) Participation in any other concurrent interventional clinical trial

6.3 Indicate specifically whether you will include any of the following special populations in your study using the checkboxes below.

NOTE: Members of special populations may not be targeted for enrollment in your study unless you indicate this in your inclusion criteria.

Response: NA

☐ Adults unable to consent

☐ Individuals who are not yet adults (infants, children, teenagers)

☐ Pregnant women

☐ Prisoners

6.4 Indicate whether you will include non-English speaking individuals in your study. ***Provide justification if you will exclude non-English speaking individuals.***

In order to meet one of the primary ethical principles of equitable selection of subjects, non-English speaking individuals may ***not*** *be routinely excluded from research as a matter of convenience.*

In cases where the research is of therapeutic intent or is designed to investigate areas that would necessarily require certain populations who may not speak English, the researcher is required to make efforts to recruit and include non-English speaking individuals. However, there are studies in which it would be reasonable to limit subjects to those who speak English. Some examples include pilot studies, small unfunded studies with validated instruments not available in other languages, studies with numerous questionnaires, and some non-therapeutic studies which offer no direct benefit.

Response:

We have no non-English speaking patients in this population. We have patients that English is a second language, but they are able to read, write and understand it. This population is less than 10% of the total population. No benefit is being withheld from not participating in the study.

7.0 Vulnerable Populations

If the research involves special populations that are considered vulnerable, ***describe the safeguards included to protect their rights and welfare.***

NOTE: You should refer to the appropriate checklists, referenced below, to ensure you have provided adequate detail regarding safeguards and protections. You do not, however, need to provide these checklists to the IRB.

7.1 *For research that involves* ***pregnant women,*** *safeguards include:*
NOTE CHECKLIST: Pregnant Women (HRP-412)

Response: We will not be recruiting subjects from vulnerable populations

☒ **N/A**: This research does not involve pregnant women.

7.2 *For research that involves* ***neonates of uncertain viability or non-viable neonates,*** *safeguards include:*
NOTE CHECKLISTS: Non-Viable Neonates (HRP-413), or Neonates of Uncertain Viability (HRP-414)

Response:

☒ **N/A:** This research does not involve non-viable neonates or neonates of uncertain viability.

7.3 *For research that involves* ***prisoners****, safeguards include:*
NOTE CHECKLIST: Prisoners (HRP-415)

Response:

☒ **N/A:** This research does not involve prisoners.

7.4 *For research that involves* ***persons who have not attained the legal age for consent to treatments or procedures involved in the research ("children")****, safeguards include:*
NOTE CHECKLIST: Children (HRP-416)

Response:

☒ **N/A:** This research does not involve persons who have not attained the legal age for consent to treatments or procedures ("children").

7.5 *For research that involves* ***cognitively impaired adults****, safeguards include:*
NOTE CHECKLIST: Cognitively Impaired Adults (HRP-417)

Response:

☒ **N/A:** This research does not involve cognitively impaired adults.

7.6 *Consider if other specifically targeted populations such as students, employees of a specific firm, or educationally or economically disadvantaged persons are vulnerable.* ***Provide information regarding***

their safeguards and protections, including safeguards to eliminate coercion or undue influence.

Response: No specific populations or vulnerable groups will be targeted. All subjects enrolled in this study will be of legal adult consenting age with the ability to speak, read and interpret the English language. Patients will have the ability to speak with the research team regarding any questions or concern they have before signing the consent. Research team have the ability to evaluate patient's cognitive abilities through these interactions with patients. Patients are made aware that this study is voluntary and they are able to stop participating at any time they feel uncomfortable. Patients are not to be pressured into participating and their clinic standard of care will remain the same if they participate or choose not to participate.

8.0 Eligibility Screening

8.1 Describe ***screening procedures*** *for determining subjects' eligibility. Screening refers to determining if prospective participants meet inclusion and exclusion criteria.*

Include all relevant screening documents with your submission (e.g. screening protocol, script, questionnaire).

Response:

After obtaining partial HIPAA waiver, patient charts will be screened according to the study inclusion and exclusion criteria by our trained clinical staff and physicians. In additions, the study clinical team will evaluate their clinic patients for possible participation in this study according to the inclusion and exclusion criteria at the Diabetes and Endocrinology Center of WNY clinics. Physicians will speak to them about their interests in participating in research. If the patient agrees, their information will be given to the research coordinator to be contacted for further evaluation. The research coordinator will contact interested patients and those meeting the inclusion and exclusion criteria based on preliminary phone evaluation will be invited to participate in the study. All personal information will be kept confidential and locked in the coordinator office.

Prospective participants will be asked to read the consent and any questions they may have regarding the protocol will be answered. If the subject wants to participate in the study, they will be asked to sign the informed consent form. The subject's medical history and current medications will be obtained as well as their blood pressure and vitals. A physical examination will also be done. Blood and urine samples will be taken in order to evaluate HbA1c, CBC, CMP and liver and kidney functions and pregnancy status. Females of childbearing age, will have to be on two forms of birth control to prevent pregnancy. Patients meeting all the inclusion and exclusion criteria based on all screening tests will be enrolled in the study.

☐ **N/A:** There is no screening as part of this protocol.

9.0 Recruitment Methods

☐ **N/A:** This is a records review only, and subjects will not be recruited. NOTE: If you select this option, please make sure that all records review procedures and inclusion/exclusion screening are adequately described in other sections.

9.1 Describe when, where, and how potential subjects will be recruited.

NOTE: Recruitment refers to how you are identifying potential participants and introducing them to the study. Include specific methods you will use (e.g. searching charts for specific ICD code numbers, Research Participant Groups, posted advertisements, etc.).

Response:

Participants will be identified by prescreening clinical charts, patient doctor interaction at the time of their visits to the Diabetes Endocrinology Center of WNY Locations include:

1. 1020 Youngs Road, Williamsville NY 14221
2. 705 Maple Road, Williamsville NY 14221
3. 462 Grider Street, Buffalo NY 14215
4. 1000 Youngs Road, Suite 105, Williamsville NY 14221

Patients can be also referred by other physicians and providers

The remainder of the participants (57 patients) will be recruited from NHS Greater Glasgow and Clyde Diabetes clinics (Scotland, UK). Separate local IRB/ Ethical approval will be sought to cover this site (based on discussions with UBIRB director).

The study clinical team will evaluate their clinic patients for possible participation in this study according to the inclusion and exclusion criteria at the Diabetes and Endocrinology Center of WNY clinics. Patients that may qualify for the study are referred to the research team for further eligibility evolution. Patients meeting the inclusion and exclusion criteria based on preliminary phone evaluation will be invited to participate in the study.

Participants will also be identified by:

-Flyers, advertisements on Facebook, ,

- Researchmatch.org, UB's participate in research (PIR) website, and through Buffalo research registry (BRR).

- We will also use the TriNetX/i2b2 database through UB's Institute for Healthcare Informatics (IHI) to recruit participants from the UBMD medical database.

Along with this, a radio ad will be on the air of the radio station STAR102.5 at various times during the day during the month of mid January to mid February

9.2 Describe how you will protect the privacy interests of prospective subjects during the recruitment process.

NOTE: Privacy refers to an individual's right to control access to him or herself.

Response: Patient charts will be screened according to the study inclusion and exclusion criteria by our trained clinical staff and physicians. If the patient qualifies and is of consenting age, the physicians will speak to them about their interests in participating in research in a private room where no one else can hear the conversation. If the patient agrees, their information will be given to the research coordinator to be contacted for further evaluation. No personal information will be shared without patient's approval and will not be used for any purpose except for this research. All personal information will be kept confidential and locked in the coordinator office.

The privacy of patients recruited using the TriNetX/ i2b2 data will be protected by following the UB IRB-approved procedure. A letter will be sent to the eligible patient's physician (see attached). After one week, if the physician doesn't contact the research team to refuse, a recruitment letter will be sent to participants via mail (see attached). If we don't hear from the patients within one week, one follow-up call will be made (see attached phone script). If at any point the physician or the patient declines, the patient will be removed from the contact list.

The Clinical and Translational Science Institute's (CTSI) Community Engagement Team (CET) hosts the Buffalo Research Registry (BRR, IRB Approved STUDY00000806), a resource that connects researchers looking to recruit participants and community members looking to get involved with research. To participate in the registry, volunteers complete a voluntary intake form. Volunteers have agreed to be contacted about potential research opportunities based on their self-reported information.

As described in the BRR's IRB Approved Protocol (STUDY00000806), key personnel from the CTSI will serve as the conduit between our research team and the registry volunteers. Key personnel will verify our IRB study approval, review inclusion and exclusion criteria and will sort the registry data accordingly. In terms of recruiting for our study, key personnel will pull volunteer reports using the inclusion and exclusion criteria. Key personnel will centrally invite volunteers to participate in the study and provide a warm hand off to our team. There are two ways we are sharing our study. These include:

Electronically- Using the volunteer report from REDCap, key personnel from the CTSI will send initial e-mail invites to introduce our study to registry volunteers.

Post Mail- Key personnel from the CTSI will provide our team with contact information (i.e. first name, last name, post mail address) to prepare a mailing. This information will be shared in a password protected excel spread sheet. The password to the spreadsheet will be sent in a separate follow-up e-mail. We will only use the list for this protocol and we agree to destroy the list once recruitment for the study has closed.

We also plan on conducting follow-up by phone/email after initial contact. This contact information will be provided in a password protected excel spread sheet. The password to the spreadsheet will be sent in a separate follow-up e-mail. We

will only use the list for this protocol and we agree to destroy the list once recruitment for the study has closed.
We will have no access to the health information provided in the registry.

9.3 Identify any materials that will be used to recruit subjects.

NOTE: Examples include scripts for telephone calls, in person announcements / presentations, email invitations.

For advertisements, include the final copy of printed advertisements with your submission. When advertisements are taped for broadcast, attach the final audio/video tape. NOTE: You may submit the wording of the advertisement prior to taping to ensure there will be no IRB-required revisions, provided the IRB also reviews and approves the final version.

Response: In addition to screening clinical charts, participations will be identified through; flyer advertisement, researchmatch.org, and Facebook. The study will also be put on UB's PIR website.

The researchmatch.org contact message and listing is provided as separate attachment to this submission (RM IRB Template Language-JDRF)

Sample letters of physician permission and patient contact and phone prescreen script are also included in this submission.

10.0 Procedures Involved

*10.1 Provide a description of **all research procedures or activities** being performed and when they are performed once a subject is screened and determined to be eligible. Provide as much detail as possible.*

NOTE: This should serve as a blueprint for your study and include enough detail so that another investigator could pick up your protocol and replicate the research. For studies that have multiple or complex visits or procedures, consider the addition of a schedule of events table in in your response.

Response:

Each patient will spend about 55 weeks in the trial comprising three weeks of monitoring to stabilize baselines and ensure adherence and commitment to participation before randomization into an intervention arm for an additional period of 52 weeks. Following a completed screening visit and meeting the inclusion and exclusion criteria, each patient will have a total of 16 study visits. At weeks 3, 5, 8, 16, 20, 29, 35, 44 and at 2 weeks after the end of the study, each patient will receive a phone call to document any adverse effects or other changes. There will be no long term follow-up following the end of the study.

Participants will be treated with either insulin only (control arm) for the whole duration of the study or with insulin and semaglutide weekly s.c. injection (titrated up to 1 mg or the maximum tolerated dose, see below) for the first six months then either dapagliflozin (Triple therapy arm) or placebo (dual therapy) daily will be added for an additional six months. Semaglutide will be started at a dose 0.25 mg once weekly for two weeks then increased to 0.5 mg dose. If well tolerated it will be increased to 1 mg once weekly at week 4. Dapagliflozin (or placebo) will be started at a dose of 5 mg daily at week 26 and if well tolerated increased after 1 week to 10 mg (2x5mg tablets) daily to ensure drug

tolerability and to avoid any hypoglycemia or hypotension. Patients who do not tolerate the increase in dosing of either medications will be maintained at the highest tolerated dose.

Treatments used

1- Insulin: All patients (114 patients) will continue to self-administer their prescribed insulin as part of their standard of care. Insulin dose will be titrated during the study to control blood glucose average between 90-180 mg/dl and to avoid hypoglycemia (blood sugar <70 mg/dl) or hyperglycemia (blood sugar >250 mg/dl). Among all patients, 38 patients will be randomized (open-label) to remain on insulin therapy only (standard therapy group) while the other 76 will start the investigational therapies.
2- Semaglutide: a once weekly a GLP-1 mimetic injection (pen) indicated for type 2 diabetes. Doses to be used: start at 0.25mg then increase to 0.5mg following 2 weeks and to 1mg weekly s.c. injection 2 weeks later. Semaglutide will be added to a background of insulin treatment for 12 months in 76 patients who will be randomized (double-blind) into 2 groups (dual and triple therapy groups) at the end of 26 weeks.
3- Farxiga (dapagliflozin): a once daily SGLT-2 inhibitor tablet indicated for type 2 diabetes. Doses used: starting with 5mg and increase after 1 week to 10mg oral administration daily. Dapagliflozin will be started according to the randomization (Interactive Web System, hosted by University of Glasgow) in 38 patients at 26 weeks as an additional treatment on a background of insulin and semaglutide therapy and end at 52 weeks (triple therapy group).
4- Placebo: dapagliflozin matching placebo (supplied by Astra Zeneca) will be started in 38 patients at 26 weeks as an additional treatment on a background of insulin and semaglutide therapy and end at 52 weeks (dual therapy group).

As shown in **Table 1,** fasting blood and urine samples will be obtained prior to drug administration (weeks 0) and at 13, 26, 39 and 52 weeks after the commencement of the study. Optional fat biopsies from the abdominal subcutaneous tissue will be collected at weeks 0, 26 and 52. Continuous Glucose Monitoring (Dexcom G6 with blinded receiver) will be performed for three 21-days periods on weeks -3 to 0, 23 to 26 and from 49 to 52 weeks. An optional 24hr blood pressure monitoring will be performed at baseline (week -1) and at 13, 25, 39 and 51 weeks in order to characterize the time course of antihypertensive effects, which may be early (i.e. independent of weight loss) as indicated by our preliminary data. High fat high caloric (HFHC) Meal challenges will be performed at baseline (week -1) and at 25 and 51 weeks with assessment of glucose excursions and postprandial glucagon (AUC) as well as postprandial inflammation and oxidative stress. Telephone calls will be conducted at weeks 3, 5, 8, 16, 20, 29, 35, 44 and at two weeks after the end of the study to document any side effects or any other patient's reported issues or comments.

Study Visits:

Screening Visit (Screening Visit, Week -8 to -4): Patients will be asked to read and understand the consent and any questions regarding the protocol will be answered. If they want to participate in the study, they will be asked to sign the informed consent form. Their medical history and current medications will be obtained as well as blood pressure and vitals. A physical examination will also be done. Blood samples will be taken in order to evaluate HbA1c, CBC, CMP, C-peptide, kidney and liver functions, ketone bodies, and urine for pregnancy status in females.

Study Visit 1 (Week -3): Participants who meet entry criteria will be scheduled to for their next visit at -3 week. Vitals will be taken including resting (min. 10 minutes) blood pressure measurement (will be repeated from same arm every visit). Insulin pump will be downloaded and/or CGM/fingerstick blood sugar will be reviewed and the appropriate insulin adjustments will be made by the investigator to target fasting blood glucose concentrations between 90-180 mg/dl if possible at all times without having additional hypoglycemia (less than 70 mg/dl). Blood sugars and ketone bodies will be measured via a fingerstick blood sample using glucose and ketones meter (Abbott Laboratories) and daily blood sugars collected and reviewed. Urine for pregnancy status and ketone bodies assessment. We will instruct patients on how to use and insert the Continuous Glucose Monitoring System (Dexcom G6 with blinded receiver). All subjects will be advised to monitor their capillary blood glucose by CGM or fingerstick at least 4 times a day (before meals and at bedtime) through-out the study and to wear their CGM constantly for next 20 day. Carb counting technique will be reviewed and any questions answered on how to use the pump or calculate insulin doses.

Study Visit 2 (Week -1±2days): Patients will be asked to come in the fasting state (no food or calorie intake for 10-12 hours). Their vitals will be taken. Blood sugars and ketone bodies will be measured via a CGM/fingerstick blood sample using glucose and ketones meter (Abbott Laboratories) to assess their glycemic control prior to meal challenge. If their blood sugar is below 70mg/dl or more than 250mg/dl the visit will be rescheduled for an alternative date. Insulin pump will be downloaded and/or CGM/fingerstick blood sugar will be reviewed and insulin adjustment will be made if necessary. An IV port will be placed in order to obtain blood samples throughout the duration of the visit. Patients will undergo a meal challenge test (described below). At the end of the meal test, they will be asked to wear a 24 hour blood pressure monitor (optional). A 24 hr urine container will be dispensed to collect a 24 hour urine sample to bring in at the Week 0 visit. Daily Glucose logs will be reviewed.

Study visit 3 (Week 0±2days): Patients will be asked to come in the fasting state (no food or calorie intake for 10-12 hours). Vitals will be taken. Blood sugars and ketone bodies will be measured via a CGM/fingerstick blood sample using glucose and ketones meter (Abbott Laboratories) and daily blood sugars collected and reviewed. The 24 hour urine container will be collected. A fasting blood sample will be taken (45 mL) to evaluate study endpoints, CBC, CMP, HbA1c, and fructosamine. A urine sample will be collected to evaluate ketone bodies and, in females, pregnancy status. Insulin pump will be downloaded and/or CGM/fingerstick blood sugar will be reviewed and the appropriate insulin adjustments will be made by the investigator. In general, insulin dose adjustments should

not be >10% from current total insulin dose or >20% from baseline (week 0) total insulin dose. They will discontinue use of the study CGM. An optional fat biopsy will be performed. We will ask them to fill out quality of life questionnaires. Study drug will be dispensed depending on what group they are randomized to. This includes either (1) staying on the insulin regimen given by their doctor, or (2) a combination of their current insulin regimen and a once daily injection of semaglutide. Semaglutide will be started on site at the 0.25 mg dose. The 24 hr blood pressure monitor will be collected.

<u>Study Visit 4 (Week 1±2days)</u>: Vitals will be taken. Blood sugars and ketone bodies will be measured via a CGM/fingerstick blood sample using glucose and ketones meter (Abbott Laboratories) and daily blood sugars collected and reviewed. Insulin dose will be adjusted if needed as in visit 3. At any time, if HbA1c is <7%, insulin dose adjustment should not be >5%. Semaglutide 0.25mg will be injected on site. Participants will be instructed to call their research center or their doctor directly in case of any problem or untoward side effects. They will be specifically asked to call if they have unmanaged hypoglycemia (blood sugar <70 mg/dl) or hyperglycemia (blood sugar >250 mg/dl) or if occurring in more than one occasion.

<u>Study Visit 5 (Week 2±2days):</u> Vitals will be taken. Blood sugars and ketone bodies will be measured via a CGM/fingerstick blood sample using glucose and ketones meter (Abbott Laboratories) and daily blood sugars collected and reviewed. Insulin dose will be adjusted if needed as in visit 3. Semaglutide 0.5mg dose will be injected on site. If they are experiencing mild nausea, they will be continued on the 0.25 mg dose for another week. Supply of the drug will be given to last until the next visit. Patients will be advised to increase the study dose to 0.5 mg a day if they are ok.

<u>Study Visit 6 (Week 4±1 week):</u> Patients will be asked to bring in used study pens for drug accountability (to be recorded in eCRF). Vitals will be taken. Blood sugars and ketone bodies will be measured via a CGM/fingerstick blood sample using glucose and ketones meter (Abbott Laboratories) and daily blood sugars collected and reviewed. Insulin dose will be adjusted if needed. Supply of the drug will be given to last until the next visit. A urine sample will be collected to evaluate ketone bodies and, in females, pregnancy status. Patients will be advised to increase the study dose to 1.0 mg/week. If they experience mild nausea, they will be continued on the 0.5 mg dose for another week. At all times, patients will be maintained at the maximum tolerated dose. A 24 hr urine container will be dispensed to collect a 24 hour urine sample to bring in at the Week 13 visit.

<u>Study Visit 7 (Week 13±1 week):</u> Patients will be asked to come in the fasting state (10-12 hours fasting), and to bring in your used study pens for drug accountability. Blood sugars and ketone bodies will be measured via a CGM/fingerstick blood sample using glucose and ketones meter (Abbott Laboratories) and daily blood sugars collected and reviewed. Insulin dose will be adjusted if needed as in. Supply of the drug will be given to last until visit 26. Vitals will be taken. A urine sample will be collected to evaluate ketone bodies and, in females, pregnancy status. The 24 hour urine container will be collected. A fasting blood sample will be taken (45 mL) to evaluate study endpoints, CBC, CMP,

HbA1c, and fructosamine. Patients who agreed to participate in the24 hour blood pressure monitoring will be asked to wear it for the next 24 hr.

Study Visit 8 (Week 23±1 week): Patients will be asked to bring in used study pens for drug accountability. Vitals will be taken. Blood sugars and ketone bodies will be measured via a CGM/fingerstick blood sample using glucose and ketones meter (Abbott Laboratories) and daily blood sugars collected and reviewed. A urine sample will be collected to evaluate ketone bodies and, in females, pregnancy status. Insulin dose will be adjusted if needed. All subjects will be reminded to monitor their capillary blood glucose by fingerstick at least 4 times a day (before meals and at bedtime) through-out the study. CGM will be inserted and patients asked to wear their CGM constantly for next 20 day.

Study visit 9 (Week 25±1 week): Patients will be asked to come in the fasting state, and to bring in used study pens for drug accountability. Vitals will be taken. A urine sample will be collected to evaluate ketone bodies. Blood sugars and ketone bodies will be measured via a CGM/fingerstick blood sample using glucose and ketones meter (Abbott Laboratories) and daily blood sugars collected and reviewed. Insulin dose will be adjusted if needed. HbA1c will be drawn. Supply of the drug will be given to last until the next visit. A 24 hr urine container will be dispensed to collect a 24 hour urine sample to bring in at the Week 26 visit. A HFHC Meal challenges will be performed as in visit 2. Patients who agreed to participate will be asked to wear a 24 hour blood pressure monitoring for the next 24 hr.
Participants in the semaglutide group will be provided supplies for urine ketones measurement (KetoStix) and blood ketones meter and strips. Research coordinator will instruct the patients on the use of meter and reading of the measuring strips. Patients will be instructed to start measuring urinary and blood ketones every morning for three consecutive days prior to next visit (starting dapagliflozin treatment) and to report results at the time of their next visit.

Study visit 10 (Week 26±1 week): Patients will be asked to come in the fasting state, and to bring in used study pens for drug accountability. Previous 3 days blood and urine ketones levels will be reviewed. Patients with normal fasting urine (<+, or < small) or blood ketone (<0.6 mmol) will proceed to next treatment phase included in this visit. Patients who report higher ketone values in at least 1 out of the 3 days will have their glucose levels, insulin dose, carb intake reviewed and managed as necessary. They will be rescheduled for next week (7±1 day) and asked to repeat the 3 days testing before next visit.
Vitals will be taken. Blood sugars and ketone bodies will be reviewed via a CGM/fingerstick blood sample using glucose and ketones meter (Abbott Laboratories) and daily blood sugars collected and reviewed. Insulin dose will be adjusted if needed. In general, insulin dose adjustments should **NOT** be >10% from current total insulin dose (or 5% if HbA1c is <7%) or >20% from baseline total insulin dose. The adjusted total insulin dose in this visit will serve as the baseline total insulin dose for the remaining 6 months of the study and future limits of dose adjustments will be based on this new baseline. The CGM will be collected. A urine sample will be collected to evaluate ketone bodies and, in females, pregnancy status. The 24 hour urine container will be collected. A fasting blood sample will be taken (45 mL) to evaluate study endpoints, CBC, CMP, C-peptide, and

Fructosamine. An optional fat biopsy will be performed. We will ask patients to fill out quality of life questionnaires. At this point, those patients randomized to receive semaglutide, they will now be randomized to receive an actual dapagliflozin pill or a placebo pill that looks like dapagliflozin. If they are randomized to receive actual dapagliflozin, they will start at the 5 mg dose for one week. Supply of the study drug will be given to last until week 27.

All patients will be asked to perform a daily (preferably early morning or before bedtime) urinary test of ketone bodies using KetoStix and record all outcomes until the end of the study. Also, they will be provided with additional blood ketone strips to measure blood ketones when needed. If results of these daily scheduled (i.e. not done due to symptoms) urine ketone measurements, are small or higher (+1 or more), they will be instructed to measure blood ketones and to repeat both measurements after 60 min. If consecutive measurements show moderate or higher ketones in urine or blood ketones >0.6mM, the patient will be instructed to call the research center or their doctor directly. For any abnormal ketone levels taken due to symptoms, the DKA consensus guidelines (Danne T et al., Diabetes Care. 2019 Feb 6) are to be followed instead (as in section 21).

Participants will be instructed to call their research center or their doctor directly in case of any problem or untoward side effects. They will be specifically asked to call if they have unmanaged hypoglycemia (blood sugar <70 mg/dl) or hyperglycemia (blood sugar >250 mg/dl) or if occurring in more than one occasion. Participants will be instructed to remain hydrated and report any genital mycotic or urinary tract infections.

Study visit 11 (Week 27±2 days): Patients will be asked to bring in used study pens for drug accountability (recorded in eCRF). Vitals will be taken. Daily blood sugars and urinary ketones measurements using KetoStix will be collected and reviewed. Blood sugars and ketone bodies will be measured via a CGM/fingerstick blood sample using glucose and ketones meter (Abbott Laboratories). A urine sample will be collected to evaluate urine ketone level. Insulin dose will be adjusted if needed. In general, insulin dose adjustments should not be >10% from current total insulin dose (or 5% if HbA1c is <7%) or >20% from NEW baseline total insulin dose established at visit 10. If at any time during the study total insulin dose reduction is >20% and total insulin dose in <0.40 U/Kg, additional monitoring protocol (see later) will be implemented. Supply of the drug will be given to last until week 39. If patients are receiving actual dapagliflozin, they will now start at the 10 mg dose for the remainder of the study.

Study Visit 12 (Week 31±1 week): Patients will be asked to bring in used study pens and tablets for drug accountability. Vitals will be taken. Daily blood sugars and urinary ketones measurements using KetoStix will be collected and reviewed. Blood sugars and ketone bodies will be measured via a CGM/fingerstick blood sample using glucose and ketones meter (Abbott Laboratories). A urine sample will be collected to evaluate ketone bodies and, in females, pregnancy status. Insulin dose will be adjusted if needed as in visit 11. A blood sample will be taken (15 mL) to evaluate CBC and CMP. A 24 hr urine container will be dispensed to collect a 24 hour urine sample to bring in at the Week 39 visit.

Study visit 13 (Week 39±1 week): Patients will be asked to come in the fasting state, and to bring in used study pens and tablets for drug accountability. Vitals will be taken. Daily

blood sugars and urinary ketones measurements using KetoStix will be collected and reviewed. Blood sugars and ketone bodies will be measured via a CGM/fingerstick blood sample using glucose and ketones meter (Abbott Laboratories). Insulin dose will be adjusted if needed as in visit 11. Supply of the drug will be given to last until the end of the study. A urine sample will be collected to evaluate ketone bodies and, in females, pregnancy status. The 24 hour urine container will be collected. A fasting blood sample will be taken (45 mL) to evaluate study endpoints, CBC, CMP, HbA1c, and Fructosamine. Patients who have provided consent will be asked to wear a 24 hour blood pressure monitor.

Study visit 14 (Week 49±1 week): Patients will be asked to bring in used study pens and tablets for drug accountability. Vitals will be taken. Daily blood sugars and urinary ketones measurements using KetoStix will be collected and reviewed. Blood sugars and ketone bodies will be measured via a CGM/fingerstick blood sample using glucose and ketones meter (Abbott Laboratories). A urine sample will be collected to evaluate ketone bodies and, in females, pregnancy status. Insulin dose will be adjusted if needed as in visit 11. All subjects will be reminded to monitor their capillary blood glucose by fingerstick at least 4 times a day (before meals and at bedtime) through-out the study. CGM will be inserted and patients asked to wear their CGM constantly for the next 20 days.

Study visit 15 (Week 51±1 week): Patients will be asked to come in the fasting state, and to bring in used study pens and tablets for drug accountability. Vitals will be taken. Daily blood sugars and urinary ketones measurements using KetoStix will be collected and reviewed. Blood sugars and ketone bodies will be measured via a CGM/fingerstick blood sample using glucose and ketones meter (Abbott Laboratories). A urine sample will be collected to evaluate urine ketone levels. Insulin dose will be adjusted if needed as in visit 11. A 24 hr urine container will be dispensed to collect a 24 hour urine sample to bring in at the Week 52 visit. A HFHC Meal challenges will be performed as in visit 2. Patients will be asked to wear the optional 24 hour blood pressure monitor.

Study visit 16 (Week 52±2 days): Patients will be asked to come in the fasting state, and to bring in used study pens and tablets for drug accountability. Vitals will be taken. Daily blood sugars and urinary ketones measurements using KetoStix will be collected and reviewed. Blood sugars and ketone bodies will be measured via a CGM/fingerstick blood sample using glucose and ketones meter (Abbott Laboratories). Insulin dose will be adjusted if needed. The CGM will be collected. An optional fat biopsy will be performed. A urine sample will be collected to evaluate ketone bodies and, in females, pregnancy status. The 24 hour urine container will be collected. A fasting blood sample will be taken (45 mL) to evaluate study endpoints, CBC, CMP, HbA1c, C-peptide, and Fructosamine. We will ask the patients to fill out quality of life questionnaires. The patient will receive a physical. After this visit, they will be discharged from the study.
Safety phone calls will be conducted at weeks 3, 5, 8, 16, 20, 29, 35, 44 and at two weeks after the end of the study to document any side effects or other changes.

Table 1: Schedule of visits and testing procedures.

Visit #	-	1	2	3	4	5	6	7	8	9	10	11	12	13	14	15	16
Week#(±2-7 days as per text)	-8 to -4	-3	-1	0	1	2	4	13	23	25	26	27	31	39	49	51	52
Study Procedures	Screening	Run-in period		Randomization, start semaglutide							Randomize/Start dapagliflozin/ placebo						End of study
History	X																
Physical	X																X
Vitals	X	X	X	X	X	X	X	X	X	X	X	X	X	X	X	X	X
CBC	X			X				X			X		X	X			X
CMP	X			X				X			X		X	X			X
C-Peptide	X										X						X
Kidney and liver profiles	X			X				X			X		X	X			X
Pregnancy test	X	X		X			X	X	X		X		X	X	X		X
Study drug titration						X	X					X					
Dispense medications				X	X	X	X	X			X	X		X			
HbA1c	X			X				X		X				X			X
Fructosamine				X				X			X			X			X
24 hour BP (optional)			X					X		X				X		X	
CGM		X	X						X	X					X	X	
Daily Fingerstick glucose review		X	X	X	X	X	X	X	X	X	X	X	X	X	X	X	X
Daily urinary ketones review											X	X	X	X	X	X	X
Insulin dose review		X	X	X	X	X	X	X	X	X	X	X	X	X	X	X	X
Ketone bodies (blood/urine)	X	X	X	X	X	X	X	X	X	X	X	X	X	X	X	X	X
Adverse Event reporting		X	X	X	X	X	X	X	X	X	X	X	X	X	X	X	X
Meal Challenge			X							X						X	
Blood endpoints				X				X			X			X			X
Fat biopsy (optional)				X							X						X
24 hr Urine				X				X			X			X			X
Quality of life questionnaires				X							X						X

Drug Blinding:
The randomizations will be performed using an Interactive Web System, hosted by University of Glasgow. One hundred and fourteen (114) patients will be randomized as follows: participants will first be randomized to semaglutide plus usual insulin therapy or insulin therapy only (no placebo for semaglutide or other placebo injection will be provided), in a 2:1 ratio. Those who remain on semaglutide at six months will then be randomized in a 1:1 ratio to receive dapagliflozin or matched placebo. Each randomization will be stratified by study site and the second randomization will also include HbA1c stratification. The randomization schedules will be generated by a statistician with no other involvement in the trial, using the method of randomized permuted blocks. Only those staff responsible for the maintenance of the randomization system will have access to the randomization sequences during the trial. The first randomization (which will be revealed to the researcher at the point of randomization) will use random block sizes to reduce predictability. Researchers at study sites will have no knowledge of the next allocation to be issued. The second randomization (which will be double-blind) will use a different. At the point of randomization, the researcher will be given a medication pack ID number, but will not know whether that medication pack contains Dapagliflozin or placebo. For each randomization, a web-based randomization system (developed and maintained by the Robertson Centre for Biostatistics) will be used. The system will include an emergency unblinding facility for the dapagliflozin/placebo randomization
Astra Zeneca Inc. will supply dapagliflozin and matching placebo tablets in unlabeled and identical packaging; semaglutide pens will be obtained commercially and will be used in an open label fashion.

Breaking of the study blind will be performed only: (i) for SUSARs; and, (ii) where knowledge of the treatment is considered by local health personnel absolutely necessary for further management of the patient. A central unblinding facility based at the Robertson Centre for Biostatistics, University of Glasgow, will be available by telephone. Notification of all unblinding will be sent to the Chief Investigator

During the course of the trial there will be no attempt to identify group assignment for any participant (except for unblinding in the case of safety concerns) and the investigators will execute the protocol in the manner it was approved independent of the group assignment.

Drugs will be stored in a locked cabinet or locked temperature controlled refrigerator at 4C at the research facility of the Diabetes and Endocrinology Center of WNY. IND application for use of all drugs (dapagliflozin and semaglutide) in type 1 diabetes will be submitted to FDA and no study procedure will start before all local and federal approvals are obtained.

Clinical and Laboratory Procedures

1) **CMP, CBC, HbA1c, fructosamine, glucose, glucagon, GLP-1 and FFA measurements:** Blood CMP, CBC, HbA1c, fructosamine and glucose will be measured through Quest diagnostic (US site) and NHS labs (UK site). Free fatty acid levels will be measured by a colorimetric assay (Wako, Richmond, VA). GLP-1 and

glucagon will be measured from samples collected in protease and DPP-IV inhibitors by ELISA kits from Millipore.

2) **Meal Challenge test:** In order to assess the postprandial changes induced by dapagliflozin, semaglutide or the combination of both compared to insulin only, a meal challenge will be performed before starting treatments, at six months and at end of study using a 910 Calorie High fat High carbohydrate meal (HFHC) as in several of our previous studies (HFHC meal includes an egg muffin sandwich, a sausage muffin sandwich and two hash browns which contain 88g carbohydrate, 51 g fat (33% saturated) and 34 g protein). Participants will be asked to attend fasting prior to breakfast insulin bolus; basal insulin will continue during the duration of the meal, however patients will not bolus until the 3 hour mark unless blood glucose values reach above 400 mg/dL. Sequential blood samples will be obtained at 0, 15, 30, 45, 60, 90, 120, 150 and 180 min after consuming the meal. Samples at 15, 30, 45, 90 and 150 min will be 5ml while those at 0, 60, 120 and 180min will be 30 ml (total volume=145 ml). Spot urine will be collected at baseline, 1 and 3hr. Blood will be collected from an indwelling intravenous canula in a superficial forearm vein.
3) **Optional 24 hour Ambulatory BP monitoring:** Participants will be instructed on the use of 24 hour ambulatory BP monitoring device. This device will measure BP and pulse every 30 minutes for 24 hours. In addition to the scheduled measurements there will be an option for additional manual measurements if systolic blood pressure is >160 or <90 mmHg.
4) **Optional Fat aspiration procedure:** Abdominal subcutaneous fat tissue aspiration will be performed at a 10 cm distance from umbilicus. Participants will be asked not to take aspirin or NSAIDS in the previous 72 hours - if they have, the procedure will not be done. Skin will be prepared with povidone-iodine (Betadine) and alcohol. A sterile drape will be placed around the appropriate area. 3cc of 1% lidocaine will then be administered subcutaneously (the dose will not exceed 4.5mg/kg body weight). After adequate anesthesia has been achieved, aspiration of fat tissue will then be with a 3-holed canula (Tulip Instrumentation, length: 15cm, diameter: 2.1mm) fixed to a 10mL syringe. After getting adequate fat tissue (500mg-3g), the puncture site will be pressed for at least ten minutes before the patient rises up from supine position (to minimize bruising). Participants will then be discharged home. Adipose tissue will be centrifuged to remove blood and fluid contaminants. Upper adipose tissue will be collected into a separate sterile tube, washed twice with cold sterile Phosphate Buffered Saline (PBS) and centrifuged to remove the PBS. The adipose tissue sample will be weighed and approximately 500 mg transferred to a separate tube for analysis. Total RNA, nuclear extracts and total cell lysates will be prepared.
5) **Questionnaires:** Participants will be asked to complete the standard Diabetes Specific Quality of Life Scale (DSQOLS) and Problem Areas In Diabetes survey (PAID). Questionnaires attached at the end of this plan.
6) **MNC isolation (for future ancillary studies):** Blood samples will be collected in Na-EDTA as an anticoagulant. Three and a half mL of anticoagulated blood sample are carefully layered over 3.5 mL of PMN medium (Cedarlane Laboratories, Hornby, ON) and then centrifuged. At the end of this process, two bands separate out at the top of the RBC pellet. The top band consists of MNC, while the bottom consists of PMN. The

MNC band is harvested and washed twice with Hank's balanced salt solution (HBSS). This method provides yields greater than 95% pure PMN and MNC suspensions.

7) **NFκB DNA binding activity (for future ancillary studies):** Nuclear NFκB DNA binding activity is measured by EMSA. Nuclear extract is prepared from MNC and adipose tissue by high salt extraction as described by Andrews et al (17). The specificity of the bands is confirmed by supershifting these bands with specific antibodies against Rel-A (p65) and p50 (Santa Cruz Biotechnology, CA) and by competition with cold oligonucleotides. DNA binding activity of NFκB will be adjusted to Oct1 DNA binding activity to correct for any variable.

8) **Plasma concentrations of inflammatory mediators (for future ancillary studies):** Concentrations of TNFα, IL-1β, IL-18, adiponectin, IL-1RA will be measured in plasma using ELISA kits (R&D Systems, MN). CRP and SAA concentrations will be measured using an ELISA kit from Alpha Diagnostics International Inc. (San Antonio, TX).

9) **Plasma vasodilators and vasoconstrictors (for future ancillary studies):** Plasma ANP, NT-proBNP, b-NP, cGMP, cAMP, angiotensinogen, renin and angiotensin II concentrations will be measured by commercially available ELISA kits.

10) **15-isoprostane F_{2t}** (also known as 8-epi-PGF2α or 8-iso-PGF2α) **(for future ancillary studies):** will be measured in urine samples using an ELISA kit from Oxford Biomedical Research (Oxford, MI).

11) **Quantification of mRNA in MNC and adipose tissue (for future ancillary studies).** Total RNA is isolated from MNC and adipose tissue using commercially available RNAqueous®-4PCR Kit (Ambion, Austin, TX). The quality and quantity of the isolated RNA is determined before using the RNA. Real Time RT-PCR is performed using Cepheid Smart Cycler (Sunnyvale, CA) in which 2 μL cDNA, 10 μL Sybergreen Master mix (Qiagen, CA) along with 0.5 μL of 20 μM gene specific primers for IL-1β, IL-RA, TLR-4, TNFα, IL-12, SOCS-3 and JNK-1 (Life Technologies, MD) are used. The specificity and the size of the PCR products are tested by adding a melt curve at the end of the amplifications and by running it on a 2% agarose gel. All values are normalized to 18S expression and β-actin.

10.2 Describe what data will be collected.

NOTE: For studies with multiple data collection points or long-term follow up, consider the addition of a schedule or table in your response.

Response:

Data will be collected as described in table 1 above. The following aspects of the study will be performed and data collected and reported by patients:

1- A CGM device will be provided for use for 3 20-days periods. Detailed instructions on changing the sensor at 10 days will be provided. Patients will be instructed to wear their CGM constantly during the assigned study periods. They will be asked to keep a diary of their food intake to measure their carbohydrate intake during the CGM period. Insulin doses will be titrated aiming for blood glucose between 90-180 mg/dl while avoiding hypoglycemia (<70 mg/dl) or hyperglycemia (>250 mg/dl).

2- Participants who agree to participate in the 24-hr ambulatory blood pressure monitoring will be provided with a 24-hour ambulatory BP monitor to be worn for 24 hours during week -1, 13, 25, 39 and 51.
3- All participants will be asked to monitor capillary blood glucose by finger stick before each meal and at bedtime.
4- For the entire duration of the study, the patients will maintain a diary to record any hypoglycemia and other untoward side effects e.g. nausea, change in appetite.
5- Starting at the 6 months mark, participants will be asked to perform a daily urinary test of ketone bodies using KetoStix and record all outcomes. If results are moderate to large, they will be instructed to repeat the test and to call the research center or their doctor directly if results are confirmed. Patients with dual glucose and ketones meters will be instructed to record and report their blood ketones as well. If blood ketones are above 0.6mM, they should repeat the test and contact the research center of their doctor if levels are confirmed.

10.3 List any instruments or measurement tools used to collect data (e.g. questionnaire, interview guide, validated instrument, data collection form).

Include copies of these documents with your submission.

Response: Source documents will be used to collect patient information. The CGM or insulin pump will be used to collect glucose and insulin data. Glucose and ketone meter will be used to measure glucose and ketone in blood. KetoStix for measuring ketones in urine. Blood pressure monitor will be used for 24 hour blood pressure reading. Glucose, food and urinary ketones diaries will be provided for patients to record their results.

10.4 Describe any source records that will be used to collect data about subjects (e.g. school records, electronic medical records).

Response: electronic medical records and research files.

*10.5 Indicate whether or not **individual** subject results, such as results of investigational diagnostic tests, genetic tests, or incidental findings will be shared with subjects or others (e.g., the subject's primary care physician) and if so, describe how these will be shared.*

Response: Individual participant lab results will be disclosed to the participant upon their request. If the participant requests documentation be shared with another physician, physician office or hospital the participant must come to the research center to collect said documentation or the documentation can be mailed to their given home address.

Patients will be assigned a unique study number. All identifiable personal information will be stored locally in locked filing cabinets. Data entered on the

electronic Case Report Form will only be identified by the unique study number. Thus, no personal information will be shared with the data center (Robertson Centre for Biostatistics, University of Glasgow, UK).

*10.6 Indicate whether or not **study** results will be shared with subjects or others, and if so, describe how these will be shared.*

Response: Not Applicable. Study results will not be shared with the subjects. However, unidentifiable study results could be published in the form of a manuscript or abstract and will be reported to JDRF, Astra Zeneca, and to clinicaltrials.gov and other regulatory agencies including FDA.

11.0 Study Timelines

11.1 Describe the anticipated duration needed to enroll all study subjects.

Response: Accrual is expected to be at the rate of 2 subjects/month/site, thus we should complete enrollment of 57 patients/site in approximately 26-30 months. We expect to complete all study clinical activities in 48 months.

11.2 Describe the duration of an individual subject's participation in the study. Include length of study visits, and overall study follow-up time.

Response: 55 Weeks. Study visits will be 60-90 minutes long except for visits 2, 9 and 15 which will be about 4hr long each

11.3 Describe the estimated duration for the investigators to complete this study (i.e. all data is collected and all analyses have been completed).

Response: 60 Months

12.0 Setting

12.1 Describe all facilities/sites where you will be conducting research procedures. Include a description of the security and privacy of the facilities (e.g. locked facility, limited access, privacy barriers). Facility, department, and type of room are relevant. Do not abbreviate facility names.

NOTE: Examples of acceptable response may be: "A classroom setting in the Department of Psychology equipped with a computer with relevant survey administration software," "The angiogram suite at Buffalo General Medical Center, a fully accredited tertiary care institution within New York State with badge access," or, "Community Center meeting hall."

Response:

1) Research will be conducted at the Diabetes Endocrinology Research Center of WNY, located at *1000 Youngs Road, Suite 105, Williamsville NY 14221* and at the CTRC located in 875 Ellicott St. Buffalo NY14203.The Diabetes Research Center has facilities and exam rooms available for insulin pump download, CGM device download, meal and infusion studies and presence of study coordinator and registered nurse for data collection and blood work at all times. One of the investigators will be available at all times to address patients' related issues. CTRC location is a fully equipped laboratory with Equipment include ultra-low freezers for sample storage, centrifuges, microscopes for sample preparation, infusion pumps, ELISA, PCR and immunoblotting instrumentation.

2) Research will also be conducted at the NHS Greater Glasgow and Clyde Clinical Research Facility (CRF, Glasgow Royal Infirmary, UK and at the Diabetes Centre (Clinic D), Stobhill Ambulatory Care Hospital, Glasgow, UK. There are facilities and exam rooms available for insulin pump download, CGM device download, meal and infusion studies and presence of research nursing staff for data collection and blood work at all times. One of the investigators will be available at all times to address patients' related issues. The CRF has equipment for sample preparation, infusion pumps, ultra-low freezers for sample storage and centrifuges. ELISA, PCR and immunoblotting facilities are available in adjacent NHS and University facilities.

12.2 For research conducted outside of UB and its affiliates, describe:

- *Site-specific regulations or customs affecting the research*
- *Local scientific and ethical review structure*

NOTE: This question is referring to UB affiliated research taking place outside UB, i.e. research conducted in the community, school-based research, international research, etc. It is not referring to multi-site research. UB affiliated institutions include Kaleida Health, ECMC, and Roswell Park Cancer Institute.

Response:

☒ **N/A:** This study is not conducted outside of UB or its affiliates.

13.0 Community-Based Participatory Research

13.1 Describe involvement of the community in the design and conduct of the research.

NOTE: Community-Based Participatory Research (CBPR) is a collaborative approach to research that equitably involves all partners in the research process and recognizes the unique strengths that each brings. CBPR begins with a research topic of importance to the community, has the aim of combining knowledge with action and achieving social change to improve health outcomes and eliminate health disparities.

Response:

☒ **N/A:** This study does not utilize CBPR.

13.2 Describe the composition and involvement of a community advisory board.

Response:

☒ **N/A:** This study does not have a community advisory board.

14.0 Resources and Qualifications

14.1 Describe the qualifications (e.g., education, training, experience, expertise, or certifications) of the Principal Investigator ***and*** *staff to perform the research. When applicable describe their knowledge of the local study sites, culture, and society. Provide enough information to convince the IRB that you have qualified staff for the proposed research.*

NOTE: If you specify a person by name, a change to that person will require prior approval by the IRB. If you specify a person by role (e.g., coordinator, research assistant, co-investigator, or pharmacist), a change to that person will not usually require prior approval by the IRB, provided that the person meets the qualifications described to fulfill their roles.

Response: All study personnel are educated, trained, and licensed as required for their delegated role in this study. All study personnel have also received the required university training and will be trained by the PI before the study starts. The PIs, CO-investigators and research team have conducted similar studies in this patient population using similar agents and have the expertise to execute the study protocol and manage any adverse events.

Describe other resources available to conduct the research.

14.2 Describe the time and effort that the Principal Investigator and research staff will devote to conducting and completing the research.

NOTE: Examples include the percentage of Full Time Equivalents (FTE), hours per week. The question will elicit whether there are appropriate resources to conduct the research.

Response: The principal investigator supervises the research project and weekly research meetings are conducted to discuss the recruitment rate, resolve and discuss issues related to the conduct, safety, analysis of the study and related publications. PI is expected to spend 15% of his academic time on this research. The co-investigators, study coordinator and research staff provide coverage to the research related activity for 365 days a year.

14.3 Describe the availability of medical or psychological resources that subjects might need as a result of anticipated consequences of the human research, if applicable.

NOTE: One example includes: on-call availability of a counselor or psychologist for a study that screens subjects for depression.

Response: Available medical literature will be provided as deemed appropriate or requested by patient through UB libraries, PubMed, Google scholar as all the investigators have access to medical literature through listed resources above

The patient will also have access to physician (Investigators and Co-Investigators) who will be available to address any adverse effects or other questions during the course of the study who will be available to address any adverse effects or other questions during the course of the study

14.4 Describe your process to ensure that all persons assisting with the research are adequately informed about the protocol, the research procedures, and their duties and functions.

Response: Before beginning of enrollment all research staff will be asked to review the protocol and a training session will be conducted to provide protocol details, explain technical points and answer staff questions. All training session will have a sign in sheet to document such training. Continuous education through training meetings, conferences and discussions will continue through the study period and when revision are made to protocol.

15.0 Other Approvals

15.1 Describe any approvals that will be obtained prior to commencing the research (e.g., school, external site, funding agency, laboratory, radiation safety, or biosafety).

Response: FDA IND filling is required.

☐ **N/A:** This study does not require any other approvals.

16.0 Provisions to Protect the Privacy Interests of Subjects

16.1 Describe how you will protect subjects' privacy interests during the course of this research.

NOTE: Privacy refers to an individual's right to control access to him or herself. Privacy applies to the person. Confidentiality refers to how data collected about individuals for the research will be protected by the researcher from release. Confidentiality applies to the data.

Examples of appropriate responses include: "participant only meets with a study coordinator in a classroom setting where no one can overhear", or "the participant is reminded that they are free to refuse to answer any questions that they do not feel comfortable answering."

Response: Our clinical providers involved in the study will identify potential patients for recruitment from the Diabetes-Endocrinology Center of WNY Clinics according to the inclusions and exclusion criteria and through advertisements. Patient who qualify will be asked in private during their one on one consultation time with the physician if they wish to participate in the research study. If the patient agrees, the research coordinator will contact them privately for a possible telephone screening and ask if time is appropriate for this discussion. The patients who call for potential participation in the study due to advertisement flyers will be screened over the phone with the research coordinator, using our telephone screening form.

When the patient is being seen at our clinics for the first time they sign the "Consent to use and disclosure of protected health information" form which clearly states that their protected health information (PHI) can be used for review in preparation for possible research. Additionally, a partial HIPAA waiver will be obtained to access medical records when screening for patients.

If the patient passes the telephone screening, they will be asked to make an appointment to review and sign the consent. Patient will do this in a private room in the research unit and will be allowed to discuss the consent in detail with the research coordinator and or study doctor. Patient will be notified that it is completely voluntary to participate in the research study and can withdraw at any time.

We will not be accessing any medical information of the patients for whom the services are not provided by our clinic providers before partial HIPAA is obtained or IC is signed.

16.2 Indicate how the research team is permitted to access any sources of information about the subjects.

*NOTE: Examples of appropriate responses include: school permission for review of records, consent of the subject, HIPAA waiver. This question **does apply** to records reviews.*

Response: Consent of the subject and partial HIPAA waiver.

17.0 Data Management and Analysis

17.1 Describe the data analysis plan, including any statistical procedures. This section applies to both quantitative and qualitative analysis.

Response:

Data (e.g. date of diagnosis, past medical history, drug treatment) extracted from local clinical records will be entered on to the eCRF under the study-specific coded identifier according to structured questions as stipulated in the Study Protocol. Biological samples (blood, urine and adipose tissue aspirates) will be collected from the study participants at the study visits according to the Schedule of Assessments in the Study Protocol.

Measures relating to safety and routine care of the participants (e.g., biochemical measures of glycemic and lipidemic control, kidney function/ fluid balance, hepatic function, hematological variables) will be analysed at local clinical laboratories in order that they are available to study nurses and physicians. The confidentiality of these data will be secure, meeting or exceeding the regulations of the relevant Ethical Review Committees/ Institutional Review Boards. Samples for measurement of key study outcomes will also be measured at the central laboratory at the University of Buffalo. Blood, urine and other samples will be aliquoted into cryovials at site for freezing and storage. Electronic stored material (e.g., patient records, data from blood and urine samples, and data from Continuous Glucose Monitoring) will only be stored using the study specific coded identifier. Data will not be identifiable at the participant level without additional access to the code. The code will be available only to the investigators and the personnel performing the study. Access to any data held electronically will require study specific log-in codes. These are managed and logged by the Robertson Centre for Biostatistics, University of Glasgow, UK

Statistical plan. The primary objective of the proposed research is to evaluate the effect of addition of dapagliflozin to semaglutide on a background of insulin treatment in type 1 diabetes patients on HbA1c at six months following start of dapagliflozin. The similarities between the study groups, baseline values for subject's demographics and study endpoints will be compared using appropriate parametric tests. Transformations of the data in order to meet statistical assumptions may be considered. The primary endpoint of the study is to detect a difference in mean HbA1c change after six months of dapagliflozin or placebo addition to semaglutide and insulin treatment. Statistical analysis will be carried out using t-tests to detect difference between groups or the Wilcoxon test for paired data. Tests will be two-sided and tested at the 0.05 level. The results will be summarized as mean±SE. All randomized patients will be included in the analysis of the primary end point based on an Intent-to-treat model.

The secondary endpoints will be to compare changes in HbA1c between each of the triple therapy and dual therapy groups compared to standard of care group, mean weekly glucose concentrations, percent time spent in different glycemic thresholds, variability as measured by standard deviation, insulin dose, body weight, carbohydrate intake, blood pressure, and 24-hour blood pressure and QOL changes among the groups. A reduction in the glucose and glucagon AUC following the meal will constitute another secondary end point in this setting, ANCOVA analysis will be used. Since the secondary end-points are pre-specified and may as well be correlated, multiplicity control for multiple tests across secondary endpoints may not be necessary. However, Holm-Bonferroni correction

for multiplicity testing will be implemented if additional endpoints are tested or if the pre-specified secondary end-points are found to be not correlated.

17.2 *If applicable, provide a power analysis.*

NOTE: This may not apply to certain types of studies, including chart/records reviews, survey studies, or observational studies. This question is asked to elicit whether the investigator has an adequate sample size to achieve the study objectives and justify a conclusion.

Response:

Sample size determination is based in part on our current data showing that in type 1 diabetes, treatment with a GLP-1 agonist, liraglutide (1.2 and 1.8mg doses) for 3 months leads to HbA1c suppression by an average of 0.6% (SD= 0.5%). Dapagliflozin was recently shown to reduce HbA1c by 0.45% in type 1 diabetes patients (13). Additionally, empagliflozin treatment for 8 weeks was shown to reduce HbA1c by 0.4% in type 1 diabetics (20, 21) and our current data showing additional improvement in HbA1c by an additional 0.66% at 12 weeks when dapagliflozin was added to liraglutide and insulin therapy. Conservatively estimating that the combination therapy will suppress HbA1c by 0.5% (SD =<0.7%) compared to semaglutide alone (t-test), a sample size of 38 (32 evaluable patients) in each group will be sufficient to provide adequate power ($\beta = 0.2$) to detect a statistically significant difference ($\alpha = 0.05$) taking in consideration a 15% dropout rate.

17.3 *Describe any procedures that will be used for quality control of collected data.*

Response:

An electronic case report form (e-CRF) will be used to collect study data at each site. The e-CRF will be developed by the study Data Centre at the Robertson Centre for Biostatistics, University of Glasgow. Access to the e-CRF will be restricted, with only authorised site-specific personnel able to make entries or amendments to their patients' data. It is the investigator's responsibility to ensure completion and to review and approve all data captured in the e-CRF.

Data will be validated at the point of entry into the e-CRF and at regular intervals during the study. Data discrepancies will be flagged to the study site coordinator and any data changes will be recorded in order to maintain a complete audit trail (reason for change, date change made, who made change).

The Robertson Centre for Biostatistics at the University of Glasgow will be responsible for collating and cleaning the data for the study. The Robertson Centre will also be responsible for data back-up and security. This center will also manage the electronic reporting of SUSARS on behalf of the sponsor.

To enable evaluations and/or audits from regulatory authorities, the investigators agree to keep records, including the identity of all participating subjects (sufficient information to link records, all original signed informed consent forms serious adverse event forms, source documents, and detailed records of treatment disposition. The records should be retained by the study country coordinators and investigator according to ICH GCP, local regulations, or as specified in the Clinical Study Agreement, whichever is longer.

Data from the study will be stored by the Chief Investigator for a minimum of 10 years. A final report of the study will be provided to the FDA and the MHRA as per requirements.

- No interim analysis is planned for efficacy so termination of study will be related to safety. Significant increases in hypoglycemic or DKA events as reviewed by DSMB might trigger termination or modification of study.
- Last observation carry forward will be used to fill missing data for the primary end point and some secondary end points. Missing data will not be filled for other endpoints.
- Any deviation(s) from the original statistical plan will be described and justified in the protocol and/or in the final report.
- All subjects entered into treatment will analyzed for primary end point on ITT bases. For secondary endpoints, analysis of completed and evaluable patients will performed per-protocol.

18.0 Confidentiality

A. Confidentiality of Study Data

Describe the local procedures for maintenance of confidentiality of ***study data and any records that will be reviewed for data collection****.*

18.1 A. Where and how will all data and records be stored? Include information about: password protection, encryption, physical controls, authorization of access, and separation of identifiers and data, as applicable. Include physical (e.g. paper) ***and*** *electronic files.*

Response: All data records will be stored on protected computers with unique passwords issued for each of the study team members and or in locked cabinets within the research center. These files will only be accessible by authorized study personnel.

The TriNetX/i2b2 data will be protected by only accessing identifiable contact information remotely on IHI's "virtual machine", so the data never leaves their secure encrypted server. Recruitment letters will be generated in a mail merge behind the firewall on IHI's virtual machine. No patient data from TriNetX/i2b2 will be removed from the IHI server or saved in other locations.

18.2 A. How long will the data be stored?

Response: De-identified data storage has no expiration date and will be stored for a minimum of 7 years. The researchers may continue to rely on this for future use in research study.

18.3 A. Who will have access to the data?

Response: Those physicians, nurses, and laboratory staff that are on all documentation for the study will have access to the data.

18.4 A. Who is responsible for receipt or transmission of the data?

Response: Those physicians, nurses, and laboratory staff that are on all documentation for the study will have access to the data and can handle transfer of data. Study data will be transferred electronically (through eCRFs) to Robertson Centre for Biostatistics, University of Glasgow, UK

18.5 A. How will the data be transported?

Response: All data are stored at locally and will be transferred to Robertson Centre for Biostatistics, University of Glasgow, UK for data management and analysis. The transfer will be done through electronic CRFs processed on secure network.

Data will also be transported unless when is being archived. At that point files will be transferred to Iron Mountain for storage and archiving.

B. Confidentiality of Study Specimens

*Describe the local procedures for maintenance of confidentiality of **study specimens**.*

☐ **N/A:** No specimens will be collected or analyzed in this research. *(Skip to Section 19.0)*

18.6 B. Where and how will all specimens be stored? Include information about: physical controls, authorization of access, and labeling of specimens, as applicable.

Response: The specimens will be stored in the laboratory located at *1000 Youngs Road, Suite 105, Williamsville NY 14221*and at the CTRC located in 875 Ellicott St. Buffalo NY14203. Samples will be stored in a locked -80º C freezer. All

specimens collected at the Glasgow site will be stored in NHS -80º C freezers within the eCRF until shipping to Buffalo (World Courier).

18.7 B. How long will the specimens be stored?

Response: Specimens storage has no expiration date and will be stored for a minimum of 7 years. The researchers may continue to rely on this for future use in research study

18.8 B. Who will have access to the specimens?

Response: Those physicians, nurses, and laboratory staff that are on all documentation for the study will have access to the specimens

18.9 B. Who is responsible for receipt or transmission of the specimens?

Response: Those physicians, nurses, and laboratory staff that are on all documentation for the study will have access to the specimens and can handle transfer of samples. Sample/specimens will transported from University of Glasgow site to the university of Buffalo CTRC site (875 Ellicott St, Buffalo, NY 14203) for storage in -80C until analyzed.

18.10 B. How will the specimens be transported?

Response: All study samples will be centrally stored in UB CTRC site. Therefore, samples collected at the UK site will be transported for analysis and final long-term storage. Transport will be done so using dry ice in a properly labeled Styrofoam container by the laboratory technician. Agreement regarding shipping and receiving the specimens form UK site will be part of the subcontract agreement.

19.0 Provisions to Monitor the Data to Ensure the Safety of Subjects

☐ **N/A:** This study is not enrolling subjects, or is limited to records review procedures only. This section does not apply.

NOTE: Minimal risk studies may be required to monitor subject safety if the research procedures include procedures that present unique risks to subjects that require monitoring. Some examples include: exercising to exertion, or instruments that elicit suicidality or substance abuse behavior. In such cases, N/A is not an acceptable response.

19.1 Describe the plan to periodically evaluate the data collected regarding both harms and benefits to determine whether subjects remain safe.

Response:

An independent Data safety and monitoring board (DSMB) will be established for this trial. The board will consist of independent faculty members from UB and other institutions with sufficient knowledge of the field of type 1 diabetes but not involved in the study. Data Management team at Robertson Centre will periodically provide the data on which to base regular reports to the Data and Safety Monitoring Board. The actual reports will then be generated by the unblinded statistician. The DSMB looks only at safety endpoints and not at futility. The DSMB will convene every six months to review safety end points, side-effects experienced by study subjects and any adverse events reported. The site PIs, CO-Is and study coordinator(s) will also continuously monitor the data and safety of subjects enrolled in the study. The site PIs and/or the DSMB may recommend to terminate the study or specific aspects of the study if clear harm is observed with one treatment option compared to standard of care or placebo. Specific safety endpoints that will be reviewed include increases in hypoglycemic and ketosis/DKA events.

19.2 Describe what data are reviewed, including safety data, untoward events, and efficacy data.

Response:

Assessment of Efficacy

1- glycemic indices including HbA1c, fructosamine, post-prandial glucose excursions and glucagon. CGM data will be collected as efficacy parameter of glucose control. Total insulin dose will be also documented and analyzed.
2- body weight
3- blood pressure and use of blood pressure medications
4- QOL questionnaires.

The timing of recording of the above efficacy parameters is described in table 1.

Assessment of Safety

The following safety assessments assays and measurement will be reviewed:

1- Vitals, CBC, CMP and markers kidney and liver function will be collected at screening, during and at end of the study as in table 1.
2- Assessment of hypoglycemia: Glucose data will be reviewed from CGM and daily finger stick and during visits.
3- Assessment of ketosis: Ketones levels will be reviewed from daily urinary ketone diaries or blood ketone meters and from urine and blood at the time of each visit
4- Reports of side effects by patients.
5- Insulin dose titrations and pump downloads at each visit

The methods and timing for assessing, recording, and analyzing safety parameters.

The DSMB will convene every six months to assess the side-effects experienced by study subjects and any adverse events reported. The PIs, CO-Is and study coordinator(s) will also continuously monitor the safety of subjects enrolled in the study during their visits and during the safety phone calls.

Procedures for eliciting reports of and for recording and reporting adverse event and inter-current illnesses.

All adverse events will be reported at the time of the visit or by telephone when it occurs. Medical events that occur between the signing of the Informed Consent and the first intake of study drug will be documented in the medical history. Participants will be asked to report any adverse events in general, non-directed questioning. For each AE volunteered by the subject, the investigator will obtain all the information required to complete the adverse event page of the eCRF, in accordance with the guidelines that accompany it. All adverse events, regardless of seriousness, severity, or presumed relationship to study therapy, will be recorded using medical terminology in the source document. Whenever possible, diagnoses will be given when signs and symptoms are due to a common etiology (e.g., cough, runny nose, sneezing, sore throat, and head congestion should be reported as "upper respiratory infection). Investigators will record on the CRF their opinion concerning the relationship of the adverse event to study therapy. The principal investigators assumes responsibility for appropriate reporting of AEs to the regulatory authorities. The investigator will report these events to the appropriate Independent Ethics Committee/Institutional Review Board (IEC/IRB) in accordance with local regulations. All measures required for AE management will be recorded in the source document and reported according to the State University of New York at Buffalo instructions. Non-serious events/problems that impacted on the rights and welfare of the subjects will be reported as they occur or at the time of knowledge or notification.

Definition of adverse events

The International Conference on Harmonization (ICH) Guideline for Good Clinical Practice (GCP) E6(R1) defines an AE as:

Any untoward medical occurrence in a patient or clinical investigation patient administered a pharmaceutical product and which does not necessarily have a causal relationship with this treatment. An AE can therefore be any unfavorable and unintended sign (including an abnormal laboratory finding), symptom, or disease temporally associated with the use of a medicinal product, whether or not considered related to the medicinal product.

The term AE is used to include both serious and non-serious AEs.

Definition of serious adverse events

A serious adverse event is an AE occurring during any study phase (i.e., screening, run-in, treatment, wash-out, follow-up), at any dose of the study drugs that fulfils one or more of the following criteria:

- Results in death
- Is immediately life-threatening
- Requires in-patient hospitalization or prolongation of existing hospitalization
- Results in persistent or significant disability or incapacity
- Is a congenital abnormality or birth defect in offspring of the patient

- Is an important medical event that may jeopardize the patient or may require medical intervention to prevent one of the outcomes listed above.
- TO THE AUTHOR: The causality of SAEs (their relationship to all study treatment/procedures) will be assessed by the investigator(s) and communicated to AstraZeneca. This is requested to be on the AE cover sheet.

The following variables are recommended to be collected for each AE:

- AE (verbatim)
- The date when the AE started and stopped
- Whether the AE is serious or not
- Action taken with regard to IPs
- Administration of treatment for the AE
- Outcome

In addition, the following variables are recommended to be collected for SAEs:

- Date the AE met criteria for SAE
- Date the Investigator became aware of the SAE
- Seriousness criteria fulfilled
- Date of hospitalization
- Date of discharge
- Probable cause of death
- Date of death
- Whether an autopsy was performed
- Causality assessment in relation to study procedure(s)
- Causality assessment in relation to other medication, as explained in Section 1.4.2.
- Description of the SAE

Reporting of serious adverse events

All SAEs will be reported, whether or not considered causally related to the investigational product, or to the study procedure(s). The reporting period for SAEs is the period immediately following the time that written informed consent is obtained throughout the treatment period and including the designated follow-up period as per the study protocol. The investigator is responsible for informing the Ethics Committee and/or the Regulatory Authority of the SAE as per local requirements.

Note that all serious or unexpected adverse events must be reported to AstraZeneca regardless of the country where the study is conducted.

The investigator must inform the FDA, via a MedWatch form, of any serious or unexpected adverse events that occur in accordance with the reporting obligations of 21 CFR 312.32 , and will concurrently forward all such reports to AstraZeneca. A copy of the MedWatch report must be emailed to AstraZeneca (TCS vendor) at the time the event is reported to the FDA. It is the responsibility of the investigator to compile all necessary information and ensure that the FDA receives a report according to the FDA reporting requirement timelines and to ensure that these reports are also submitted to AstraZeneca at the same time.

A cover page should accompany the MedWatch form indicating the following:

- "Notification from an Investigator Sponsored Study"
- The investigator IND number assigned by the FDA
- The investigator's name and address
- The trial name/title and AstraZeneca ISS reference number (ESR-##-#####)

Investigator must also indicate, either in the SAE report or the cover page, the causality of events in relation to all study medications and if the SAE is related to disease progression.

Send SAE report and accompanying cover page by way of email to AstraZeneca's designated mailbox: AEMailboxClinicalTrialTCS@astrazeneca.com

If a non-serious AE becomes serious, this and other relevant follow-up information must also be provided to AstraZeneca and the FDA.

Serious adverse events that do not require expedited reporting to the FDA still need to be reported to AstraZeneca preferably using the MedDRA coding language for serious adverse events.

The investigators will either provide a copy of the randomization code/ code break information or unblind those SAEs which require expedited reporting

Overdose

An overdose is defined as a patient receiving a dose of IP in excess of that specified in the Investigator's Brochure, unless otherwise specified in this protocol.

Any overdose of a study patient with the investigational product, with or without associated AEs/SAEs, is required to be reported within 24 hours of knowledge of the event to AstraZeneca Patient Safety using the designated Safety e-mailbox (see Section 1.5 or contact information). If the overdose results in an AE, the AE must also be recorded as an AE. Overdose does not automatically make an AE serious, but if the consequences of the overdose are serious, for example death or hospitalization, the event is serious and must be recorded and reported as an SAE (see Section 1.3). Any specific details around overdose for combination or comparator agents will also be provided as applicable. The investigator will use clinical judgment to treat any overdose.

Hepatic function abnormality

Hepatic function abnormality that fulfills the biochemical criteria of a potential Hy's Law case in a study patient, with or without associated clinical manifestations, is recommended to be reported as "hepatic function abnormal" within 24 hours of knowledge of the event to AstraZeneca Patient Safety using the designated Safety e-mailbox (see Section 1.3), unless a definitive underlying diagnosis for the abnormality (e.g., cholelithiasis or bile duct obstruction) that is unrelated to investigational product has been confirmed. The criteria for a potential Hy's Law case is Aspartate Aminotransferase (AST) or Alanine Aminotransferase (ALT) ≥3x Upper Limit of Normal (ULN) together with Total Bilirubin (TBL) ≥2xULN at any point during the study following the start of study medication irrespective of an increase in Alkaline Phosphatase (ALP).

Pregnancy

If a patient becomes pregnant during the course of the study, the IPs should be discontinued immediately.

Pregnancy itself is not regarded as an AE unless there is a suspicion that the IP under study may have interfered with the effectiveness of a contraceptive medication. Congenital abnormalities or birth defects and spontaneous miscarriages should be reported and handled as SAEs. Elective abortions without complications should not be handled as AEs. The outcome of all pregnancies (spontaneous miscarriage, elective termination, ectopic pregnancy, normal birth, or congenital abnormality) should be followed up and documented even if the patient was discontinued from the study.

If any pregnancy occurs in the course of the study, then the investigator should inform the appropriate AstraZeneca representatives within 1 day, i.e., immediately, but no later than 24 hours of when he or she becomes aware of it.

The investigator will ensure that all relevant information is provided to the AstraZeneca Patient Safety data entry site within 1 to 5 calendar days for SAEs and within 30 days for all other pregnancies.

The same timelines apply when outcome information is available.

19.3 Describe any safety endpoints.

Response: These specific Safety endpoints will be collected:

1- Frequency and severity of hypoglycemia: Hypoglycemia will be assessed throughout the study via multiple daily finger stick glucose measurements, CGM data collected 3 times (about 3 weeks each) during the study and from patient reports during visits and phone calls. Hypoglycemic events will be characterized as in the experts consensus published in *Diabetes Care (2017 Dec; 40(12): 1622-1630):*

- Level 1: Glucose < 70 mg/dL (3.9 mmol/L) and Glucose ≥ 54 mg/dL (3.0 mmol/L)
- Level 2: Glucose < 54 mg/dL (3.0 mmol/L)
- Level 3: A severe event characterized by altered mental and/or physical status requiring assistance.

2- Ketones levels: Urinary ketones measured daily (and any blood ketones) at home and logged in the ketones logbook. Blood and urinary ketones measured during each visit. Ketone levels will be characterized as normal, ketosis and severe ketosis:

-Normal blood ketones <0.6mM or urinary ketones of trace to small (≥+1)

-Ketosis: Blood ketones >0.6mM <2.5mM or urinary ketones of moderate to high (+2 and +3)

-Severe ketosis Blood ketones >2.5mM or urinary ketones of too high (+4)

3- Diabetes ketoacidosis episodes: any confirmed DKA episode (regardless of hospitalization) defined as:

• Elevated serum or urine ketones (greater than the upper limit of the normal range), and

• Serum bicarbonate < 15 mmol/L or Blood pH < 7.3

4- Low blood pressure: <90mmHg SBP and <50 mmHg DBP): Patients will be instructed to measure blood pressure daily and it will be will be assessed in each visit.
5- Nausea, vomiting and other GI side effects. Data will be collected by patients' safety reports, during visits and phone calls.
6- Genital mycotic infections or UTIs
7- Changes in liver and kidney function: CMP and kidney and liver profiles will be assessed as indicated in table 1.

19.4 Describe how the safety information will be collected (e.g., with case report forms, at study visits, by telephone calls with participants).

Response: The safety information will be collected at the time of the visit, and or during preplanned telephone calls with the participant or patient self-reporting (including glucose, food intake and ketones diaries). Data will be entered into eCRFs

19.5 Describe the frequency of safety data collection.

Response: Safety data collection will be done at all study visits and during preplanned phone calls as in table 1. The patient, however, will be asked to report any adverse event or safety related information via phone as soon as it occurs and it will be reviewed the same day.

19.6 Describe who will review the safety data.

Response: DSMB established for this study will review unblended safety data provided by the data management system every 6 months. Additionally, the PIs and coinvestigators will review safety data (listed above) at the completion of each visits by each subject and review any report of side effects or adverse events as they are presented by the patients. The investigators will also review collective blinded data every 3 months to assess the safety and any potential risks to the participants. Furthermore the investigators will assess other risks including the physical, psychological, social, legal and economic harm to these patients. The investigators will carefully watch for any invasion of privacy and breach of confidentiality. The principal investigator will be sharing the results of safety analysis of the study and serious adverse events with the local IRB, DSMB, JDRF, AZ and the FDA. The study groups will remain blinded. If there are a serious safety concerns or an emergency, then, and based on principal investigator discretion, study randomization center in Glasgow University or the unblended statistician will provide information about group assignment of the patient(s) involved. Un-blinded data will be reviewed by the principal investigator and co-investigators and decision to complete, withdraw or adjust dose of drug(s) will be made, documented and shared with IRB and DSMB.

19.7 Describe the frequency or periodicity of review of cumulative safety data.

Response: Safety data will be reviewed every 6 months by DSMB (un-blinded), every 3 months by PIs and CO-Is (blinded) and at every visit by study team.

19.8 Describe the statistical tests for analyzing the safety data to determine whether harm is occurring.

Response: N/A

19.9 Describe any conditions that trigger an immediate suspension of the research.

Response: No interim analysis is planned for efficacy so termination of study will be related to safety. Significant increases in severe hypoglycemic or DKA events as reviewed by DSMB are possible causes that might trigger termination of study.

20.0 Withdrawal of Subjects

☐ **N/A:** This study is not enrolling subjects. This section does not apply.

*20.1 Describe **anticipated** circumstances under which subjects may be withdrawn from the research without their consent.*

1. The site PI can stop investigational study treatment or remove participants from the trial without participants approval if for any reason he/she feels is appropriate, including: severe side effect, injury or medical condition which may place the patient at risk of further complications if he/she continue to participate, failure to take the medication as instructed, failure to keep scheduled appointments, cancellation of the study by the sponsor, IRB, FDA or other administrative reasons.
2. If any of the participants become pregnant during the period of study, they will discontinue study treatment.
3. Participants who experience persistent severe nausea or vomiting after starting semaglutide which is not managed by dose reduction will discontinue the investigational study treatment. Those with mild or moderate nausea will continue for at least one more week. If they continue to experience significant nausea (defined as interfering with normal activities), dose will be reduced. If they continue to have these side effects, they will discontinue study medication(s).
4. Persistent weight loss or failure to maintain weight if subject has lost >15% or BMI is < 22 kg/m2 in spite of down-titration of semaglutide and/or dapagliflozin.
5. Developing other conditions included in the exclusion criteria
6. Change in method of insulin delivery between insulin pumps to MDI or vice versa during the study unless pump treatment interruption is temporary for a period of at

most 2 weeks. Also, patients who upgrade to the new "artificial pancreas" pump (Medtronic's MiniMed 670G hybrid) during the study will be withdrawn.

7. Hypoglycemia: Two episodes of severe hypoglycemia (requiring others help).
8. Ketosis (with no other identifiable precipitating factors): Persistent (1week) asymptomatic or symptomatic and unmanaged (using STICH protocol) blood ketone levels >1.5 mM (or ≥+2 in urine).
9. Persistent (>1 week) total insulin dose reduction of >20% from baseline AND total insulin dose <0.4U/Kg.
10. DKA: Two episode of DKA (with no identifiable precipitating factors) as defined by the expert consensus statement (*Diabetes Care (2017 Dec; 40(12): 1622-1630)*)
11. Two episodes of UTI.
12. Two episodes of mycotic genital infection.
13. Significant persistent change in eGFR > 15 ml/min
14. Pancreatitis: One episode of pancreatitis.
15. Other persistent side effects specific to study drugs

20.2 Describe any procedures for orderly termination.

NOTE: Examples may include return of study drug, exit interview with clinician. Include whether additional follow up is recommended for safety reasons for physical or emotional health.

Response: Participation in this study is voluntary. Participants have the right to refuse to participate or to withdraw from participation at any time for any reason. Refusal to participate or to withdraw from the study will involve no penalty or loss of entitled benefits, nor affect ongoing medical care. The site PI can stop investigational study treatment or remove participants from the trial without participants approval. If a patient withdraws consent and decide not to participate in the study, she/he will be asked to undergo a final study visit for his/her safety. If a subject withdraws from the research, the data collected to that point will be used toward the research finding. If applicable the subject will have to bring back any unused research drug and or devices.

20.3 Describe procedures that will be followed when subjects withdraw from the research, including retention of already collected data, and partial withdrawal from procedures with continued data collection, as applicable.

Response: We will use the data collected up to the point of stopping the study. No further information will be collected. Patients who stop study treatment and are interested in continuing in the study will be followed up until the end of the study for the primary end point and select secondary endpoints based on the Intent-To-Treat (ITT) analysis model. Withdrawn participants will not be replaced. The study sample size calculation includes an additional 15% subjects to compensate for drop out.

21.0 Risks to Subjects

21.1 List the reasonably foreseeable risks, discomforts, hazards, or inconveniences to the subjects related to their participation in the research. Consider physical, psychological, social, legal, and economic risks. Include

a description of the probability, magnitude, duration, and reversibility of the risks.

NOTE: Breach of confidentiality is always a risk for identifiable subject data.

Response:

- Risks associated with intravenous (IV) line: All participants will be informed of the potential complications of an intravenous (IV) line, which include mild bruising at the site of IV line, usually resolving in a few days. They will also be informed about the possibility of infiltration of the IV line at the time of performing blood draws or during the meal challenge test in which case another IV line at a different site will be required (with risk of bruising at more than one site).
- Risks associated with 24 hour Ambulatory BP monitoring device: The BP cuff inflates every 15 minutes to record blood pressure over 24-hours. Participants will experience a pressure-like sensation over the arm where 24 hour BP device is worn at the time of the BP being measured due to the inflation of the cuff and release of this pressure at the time of deflation of the BP cuff. They will be informed to remove the cuff after 24 hours. If worn for longer periods they may experience mild bruising over the arm and some swelling, usually resolving 1-2 days after discontinuation.
- Risks associated with fat tissue aspiration: will probably lead to a bruise at the site of aspiration. The site may be mildly painful for 1-2 weeks. Participants are advised to call their study site staff if they have more severe pain or swelling at the site after the procedure. Rarely there are side effects such as low BP or heart rate and/or allergic reaction to local anesthetic including swelling of the throat.
- All subjects will be referred to semaglutide and dapagliflozin labeling safety information, and will be given a copy of the package insert of the medication. Discussion of the potential side effects and the different warning and precautions will take place at the time of informed consent. Patients will be informed they must not discontinue their insulin during the study.
- Potential risk of insulin, GLP-1 RA and SGLT-2 inhibitors use in type 1 diabetes patients is hypoglycemia. The combination therapy can possibly increase the magnitude and frequency of this risk.
- Potential side effects of GLP-1 RA and SGLT-2 inhibitors also include hypotension, nausea and other GI side effects, polydipsia, polyuria, dehydration, ketosis, and reduced glomerular filtration rate (GFR).
- In rodents, semaglutide causes dose-dependent and treatment-duration-dependent thyroid C-cell tumors at clinically relevant exposures. However, it received FDA-approval for the treatment of type 2 diabetes in 2017 following large Phase 3 trials including the cardiovascular safety trial SUSTAIN-6.
- On the basis of the above rodent studies, semaglutide is contraindicated in those with a personal or family history of medullary thyroid carcinoma and in patients with Multiple Endocrine Neoplasia syndrome type 2 (MEN 2). However, following review of human data with GLP-1RAs, FDA does not require or suggest routine monitoring of serum calcitonin/ thyroid ultrasound in people treated with semaglutide.

- Injection-site reactions: Serious injection-site reactions, with or without bumps (nodules), have happened in some people who use GLP-1RAs but are less frequent with semaglutide. Injection-site reactions have rarely required surgical intervention. Participants will be instructed to report any injection site reactions including nodules to study investigators.
- Kidney injury: GLP-1 RA might cause acute kidney injury in patients without underlying renal disease especially in patient who had experienced nausea, vomiting, diarrhea or dehydration. Dapagliflozin also has warnings and precautions against acute kidney injury and impairment in renal function.
- As mentioned above, there is a risk of ketoacidosis, as shown by some studies and by our data, when SGLT2 inhibitors are used in type 1 diabetes. In our own preliminary studies on triple therapy, we observed euglycemic ketoacidosis in participants whose insulin doses had been reduced by > 20% of baseline levels or in patients who are not adequately insulinzed. Thus, this adverse effect is largely attributable to insulinopenia as well as anti-insulin effects of SGLT2 inhibitors at the hepatic level (e.g. increased gluconeogenesis).
- The following other known adverse effects of SGLT-2 inhibitors will be discussed with participants when obtaining informed consent:

1. Risk of dizziness/ symptomatic hypotension: can occur soon after initiation due to intravascular volume contraction.
2. Impaired kidney function: As SGLT-2 inhibitors increase serum creatinine and decrease eGFR, this will be monitored closely.
3. Genital mycotic infections: SGLT-2 inhibitors increase the risk of genital mycotic infections, particularly in women. Those with a prior history are more susceptible. Following topical treatment with anti-fungal medications they rarely re-occur.
4. Bladder Cancer - Across 22 clinical studies, newly diagnosed cases of bladder cancer were reported in 10/6045 patients (0.17%) treated with Dapagliflozin and 1/3512 patient (0.03%) treated with placebo. After excluding patients in whom exposure to study drug was less than one year at the time of diagnosis, there were 4 cases of bladder cancer with dapagliflozin and no cases with placebo i.e. too few cases to determine causality.
5. The dapagliflozin tablets and matching placebo contain lactose, which may cause discomfort in lactose-intolerant individuals.

- Severe allergic reaction (anaphylaxis) to study drugs or placebo ingredients or other interventions may accrue in rare cases.
- Excessive weight loss: Both investigation drugs are known to cause weight loss in the range of 5-10% in type 2 diabetes patients. However, the magnitude of weight loss following the combination of both drugs in type 1 diabetes patients may be significantly more. Excessive weight loss may lead to increased risk of hypoglycemia and ketosis or DKA (especially in the presence of dapagliflozin) and other unforeseen risks. Therefore, weight loss during any phase of the study will be limited to ≤15% baseline body weight or reaching a BMI of $\leq 22 Kg/m^2$.
- There might be other unforeseen risks associated with the use of these agents in type 1 diabetes and additional unforeseen risks associated with the combination therapy.

- There is always the risk of breach of confidentiality where patients' identifiable data is compromised.

21.2 Describe procedures performed to lessen the probability or magnitude of risks, including procedures being performed to monitor subjects for safety.

Response:

The following are procedures to lessen the probability and magnitude of risks listed above:

1- Hypoglycemia:
- Patients will be instructed to measure glucose at least 4 times a day (before meals and at bedtime) through-out the study and record all reading.
- Insulin dose adjustments will be done at every visit to avoid hypoglycemia. However, there will be no preemptive insulin dose reduction at start of treatment since all patients will have HbA1c >7.5% and the risk of hypoglycemia will be minimal when semaglutide is added.
- Daily fingerstick glucose, CGM and insulin pump data will be reviewed by the investigators for optimal titration of insulin dose to target glucose levels between 90-180mg/dl.
- In general, insulin dose adjustments should not be >10% from current total insulin dose (or 5% if HbA1c is <7%) or >20% from baseline total insulin dose at week 0 and then at week 26.
- Patients will be trained in CARB counting to avoid inaccurate insulin dose administration.
- Patients will be trained to recognize symptoms of hypoglycemia and how to manage it at home. These symptoms include feeling shaky, being nervous or anxious, sweating, chills and clamminess, confusion, fast heartbeat, feeling lightheaded or dizzy, hunger, nausea and color draining from the skin (pallor) among others. They will be asked to call the research office or the on call clinical services.
- When patients feel symptoms of hypoglycemia they will be asked to measure blood glucose and if <70mg/dl they will be instructed to consume carbohydrate or 4 tablets of glucose (4gm each). If after 15 min hypoglycemia continues, repeat carb intake until sugar levels are above 70 mg/dl. If sugar levels are below 55mg/dl, glucagon injection is permitted as rescue medication for severe hypoglycemia.
- Patients will be instructed to inform people who they are in frequent contact with (for example, friends, family members, and coworkers) on how to give glucagon to treat severe hypoglycemia. If patient have needed glucagon previously, it should be documented during screening visit so more emphasis on ways to prevent severe hypoglycemia in the future can be discussed.
- Patients/family will be instructed not to hesitate to call 911 if someone is unconscious and glucagon is not available or someone does not know how to use it and not to inject insulin (it will lower their blood glucose even more) or provide food or fluids (they can choke). They will be asked to document all details of the incident and report to research staff as soon as possible.
- Study medications dose will be reduced in patients who continue to have more than 2 measurements of glucose levels in the hypoglycemic range (<70mg/dl,

regardless of symptoms) after insulin dose is reduced by the maximum allowed in this study.

2- Ketosis and Ketoacidosis:

- Patients will be instructed to measure ketone bodies in urine samples daily starting at 3 days before week 26 with Ketostix (Bayer) and record it in diary provided. Ketones (blood and urine) will be measured at every visit. Additionally, patients will be provided with a ketone meter and strips to measure blood ketones when needed. If ketone bodies in urine measure +1 or greater (>1.5mM in urine), they will be instructed to measure blood ketones and to repeat both the measurements after 60 min. If consecutive measurements show moderate or higher ketones in urine or blood ketones >0.6mM, the patient will be instructed to call the research center or their doctor directly. They will be monitored closely for insulin dose, carb intake, ketones and blood sugar and dapagliflozin/semaglutide can be temporarily discontinued, if necessary, for 1-2 weeks.
- Patients will be provided training to recognize the signs and symptoms of DKA and to self-test urine ketones on a PRN basis (in addition to once daily) if they develop any symptoms consistent with DKA, including nausea, vomiting, fatigue, confusion, or shortness of breath. This should be reported regardless of blood sugar levels since in type 1 diabetes on SGLT-2 inhibitors, DKA can occur even in normal glucose levels.
- Insulin dose changes should not be greater than 10% (5% if HbA1c is<7%) from current levels and not reduced by more than 20% from baseline levels at 0 weeks and then at 26 weeks.
- Patients requiring an insulin dose reduction > 20% AND their total insulin dose is <0.4U/Kg will be monitored closely for insulin dose, carb intake, ketones and blood sugar and dapagliflozin/semaglutide can be temporarily discontinued, if necessary, for 1-2 weeks. If insulin requirements remain law, patient will be withdrawn from study.
- We shall not increase dose of dapagliflozin in patients who exhibit significant ketonuria (+2 or more) with the lower dose.
- Urinary ketones (and blood ketones if patients have a dual glucose and ketone meter) will be monitored daily and reported. Dapagliflozin will be temporarily (1 week) discontinued and STICH protocol (STop SGLT-2i, give Insulin bolus, Carbohydrate, and Hydration) activated in events of DKA or if based on investigator discretion if blood ketone levels are persistently (1 week) >1.5 mM (+2 in or more in urine). Dapagliflozin may also be temporarily stopped in clinical situations known to predispose to ketoacidosis (e.g. prolonged fasting due to acute illness or surgery). Patients will be instructed to stay hydrated and not reduce their caloric intake (especially carbohydrates) or start new dietary systems (especially ketogenic diets) during the study.
- If a patient develops symptoms of DKA or ketone levels exceed the allowed limit in this study (persistent (1 week) blood ketones >1.5 mM or +2 in or more in urine), STICH protocol (STop SGLT-2i, give Insulin bolus, Carbohydrate, and Hydration) will be activated. They could be managed for up to a week. If

symptoms and ketone levels persist over 1 week, patients will be withdrawn form study. Insulin infusions may be administered by ER or hospital if patients develop DKA. They will be asked to document all details of the incident and report to research staff as soon as possible.

3- Risk of acute kidney injury or renal function impairment: CBC, CMP, including kidney function test and urinary markers will be performed at screening, during and at end of the study (table 1). Additional testing will be performed in patients who suffer from sever nausea or GI symptoms or hypovolemic and during the up titration of investigation drugs. If acute kidney injury occurs dapagliflozin will be discontinued immediately. To lessen the chance of this risk, patients will eGFR<60 ml/min/1.73m^2 will be excluded. Patients will also discontinue study medications if eGFR falls by >15 ml/min in 2 repeated measurements. Also other patients with factors that may predispose patients to acute kidney injury e.g. hypovolemia, chronic renal insufficiency, congestive heart failure will be excluded. Patients on ACEI, ARB, NSAID will be monitored closely. Dapagliflozin will be temporarily discontinued in any setting of reduced oral intake such as acute illness or fasting or fluid losses such as gastrointestinal illnesses, or excessive heat exposure.

4- Injection site reactions, mycotic infections and complications of IV and biopsy collection will also be monitored, Physical examination will be performed at screening 26, and 52 weeks visits. Safety vital assessments will be performed at every visit. Pregnancy will be tested at screening, 0, 4 13, 26, 39, and 52 weeks. Phone calls will be made for safety and compliance at least 7 time during the study. The patients, however, will be instructed to report any adverse event or safety related information via phone as soon as it occurs. All safety data will be reviewed by the PI or sub-investigators within 1-2 days of collection.

5- Epinephrine is the primary treatment for anaphylaxis with no absolute contraindication to its use. Our site and staff are trained and equipped to assist patients with such reactions. Severe conditions that are not satisfactory managed in site will be transferred to the hospital emergency department for further assessment and help.

6- Excessive weight loss: Doses of investigational drugs will be reduced in patients who experience excessive weight loss (≥15% of basal body weight or BMI ≤22Kg/m^2). Patients on semaglutide only (first 26 weeks) will be down-titrated to the lower dose. In patients on semaglutide and dapagliflozin/placebo (weeks 26-52), dose of dapagliflozin/placebo will be down-titrated to the lower dose. If weight loss continues, semaglutide dose will be down-titrated as well. Instructions to maintain a minimum of 50g daily carb intake will be also emphasized in patients with significant weight loss (>10% baseline body weight).

*21.3 If applicable, indicate **which procedures** may have risks to the subjects that are currently unforeseeable.*

Response:

There might be additional adverse effects of dapagliflozin not currently known or those unique to their use in Type 1 diabetes. There is no previous studies on the use of semaglutide in type 1 diabetes; therefore, additional unforeseen risk may be encountered in this study. Additionally, unforeseen risks might be associated with the combination therapy that might involve unknown drug-drug interactions.

21.4 If applicable, indicate which research procedures may have risks to an embryo or fetus should the subject be or become pregnant.

Response: Not applicable, we will not be enrolling pregnant participants or who may become pregnant or with in child baring years without signing the consent stating they will use at least two forms of birth control. If a participant becomes pregnant they will be withdrawn from the study immediately. They will be followed until term and any effects on the newborn will be documented.

21.5 If applicable, describe risks to others who are not subjects.

Response: We do not anticipate any additional risk to others not participating in this study.

22.0 Potential Benefits to Subjects

22.1 Describe the potential benefits that individual subjects may experience by taking part in the research. Include the probability, magnitude, and duration of the potential benefits. Indicate if there is no direct benefit.

*NOTE: Compensation **cannot** be stated as a benefit.*

Response: The investigational drugs used in this study are already approved as antidiabetic agents for treating type 2 diabetes. Both agents have demonstrated significant improvement in HbA1c, weight and blood pressure. It is our expectation that patients with type 1 diabetes will experience similar benefits compared to those who are treated with insulin alone. The magnitude and duration of the benefits and the safety profile may however differ and are the focus of this investigation.

23.0 Compensation for Research-Related Injury

☐ **N/A:** The research procedures for this study do not present risk of research related injury (e.g. survey studies, records review studies). This section does not apply.

*23.1 **If the research procedures carry a risk of research related injury,** describe the available compensation to subjects in the event that such injury should occur.*

Response: Routinely, Buffalo General Hospital, Erie County Medical Center, and/or the University at Buffalo, State University of New York, its agents, or its employees do not compensate for or provide free medical care for human

subjects/participants in the event that any injury results from participation in a human research project. In the unlikely event that they become ill or injured as a direct result of participating in this study, they may receive medical care. Your study doctor will explain the treatment options to you and tell you where you can get treatment. Generally, this care will be billed to you, your insurance or other third party. Additionally, AstraZeneca shall reimburse the institution for the direct, reasonable, and necessary medical expenses incurred by the institution for the treatment of any personal injury that is a direct result of adverse drug experiences to subjects resulting directly from any manufacturing defects in the study drug to which the subjects would not have been exposed but for their participation in the study if (i) the institutional parties have complied with this agreement, the protocol and any written instructions of AstraZeneca concerning the study drug and (ii) all the requirements of informed consent have been complied with. AstraZeneca will not provide compensation for lost wages or for any other damages, expenses or losses, or for medical expenses have been covered by a subject's medical or other insurance.

23.2 Provide a copy of contract language, if any, relevant to compensation for research related injury.

NOTE: If the contract is not yet approved at the time of this submission, submit the current version here. If the contract is later approved with ***different language regarding research related injury****, you must modify your response here and submit an amendment to the IRB for review and approval.*

Response: AstraZeneca shall reimburse the institution for the direct, reasonable, and necessary medical expenses incurred by the institution for the treatment of any personal injury that is a direct result of adverse drug experiences to subjects resulting directly from any manufacturing defects in the study drug to which the subjects would not have been exposed but for their participation in the study if (i) the institutional parties have complied with this agreement, the protocol and any written instructions of AstraZeneca concerning the study drug and (ii) all the requirements of informed consent have been complied with. AstraZeneca will not provide compensation for lost wages or for any other damages, expenses or losses, or for medical expenses have have been covered by a subject's medical or other insurance.

24.0 Economic Burden to Subjects

24.1 Describe any costs that subjects may be responsible for because of participation in the research.

NOTE: Some examples include transportation or parking.

Response: All research expenses will be covered by the study. The background use of insulin is considered as part of standard of care is the responsibility of the patients and his/her insurance. Participants will not be subjected to any other out of pocket cost.

☐ **N/A:** This study is not enrolling subjects, or is limited to records review procedures only. This section does not apply.

25.0 Compensation for Participation

25.1 Describe the amount and timing of any compensation to subjects, including monetary, course credit, or gift card compensation.

Response:
Visit schedule:

- Screen: $0
- Visit 1 (Week -3): Insulin adjustment: $10
- Visit 2 (Week -1): Meal ($75), insulin adjustment, and 24hr blood pressure monitor ($50): $125*
- Visit 3 (Week 0): 24 hr urine ($15), blood draw ($25), fat biopsy ($75), insulin adjustment, questionnaire ($10): $125
- Visit 4 (Week 1): Insulin adjustment: $10
- Visit 5 (Week 2): Insulin adjustment: $10
- Visit 6 (Week 4): Insulin adjustment: $10
- Visit 7 (Week 13): 24hr urine ($15), blood draw ($25), insulin adjustment, 24hr bp monitor ($50): $90*
- Visit 8 (Week 23): Insulin adjustment: $10
- Visit 9 (Week 25): Insulin adjustment, 24bp monitor ($50), Meal ($75): $125*
- Visit 10 (Week 26): Insulin adjustment, 24hr urine ($15), blood draw ($25), fat biopsy ($75), questionnaire ($10): $125*
- Visit 11 (Week 27): Insulin adjustment: $10
- Visit 12 (Weak 31): Insulin adjustment, blood draw: $25
- Visit 13 (Week 39): Insulin adjustment, 24hr urine ($15), blood draw ($25), 24hr bp monitor ($50): $90*
- Visit 14 (Week 49): Insulin adjustment: $10
- Visit 15 (Week 51): Insulin adjustment, 24bp monitor ($50), meal ($75): $125*
- Visit 16 (Week 52): Insulin adjustment, 24hr urine ($15), blood draw ($25), questionnaire ($10), fat biopsy ($75):$125*

*Total compensation for all completed visits: up to $1,025.00. For those not wishing to participate in the optional blood pressure monitoring and/or fat biopsy procedure, the compensation will decrease by $250 for not completing the bp monitoring, and by yet another $225.00 for not completing the fat biopsies.

☐ **N/A:** This study is not enrolling subjects, or is limited to records review procedures only. This section does not apply.

☐ **N/A:** There is no compensation for participation. This section does not apply.

26.0 Consent Process

26.1 Indicate whether you will be obtaining consent.

NOTE: This does not refer to consent documentation, but rather whether you will be obtaining permission from subjects to participate in a research study. Consent documentation is addressed in Section 27.0.

☒ ***Yes*** *(If yes, Provide responses to each question in this Section)*

☐ ***No*** *(If no, Skip to Section 27.0)*

26.2 Describe where the consent process will take place. Include steps to maximize subjects' privacy.

Response: All participants will come to the research department to be consented. Participants will be placed in a private, screened off area and or room where they can review the consent. Participant questions and or concerns will be address with a member of the study team or research doctor if applicable. The research coordinator will discuss in length the participants requests for privacy of their PHI.

26.3 Describe how you will ensure that subjects are provided with a sufficient period of time to consider taking part in the research study.

NOTE: It is always a requirement that a prospective subject is given sufficient time to have their questions answered and consider their participation. See "SOP: Informed Consent Process for Research (HRP-090)" Sections 5.5 and 5.6.

Response: participants will be made aware that participating in research is completely voluntary, and they may withdraw at any time with no consequence to their routine clinic care. If the patients requires time to decide and or discuss partaking in a research study, the subject will be given said time.

26.4 Describe any process to ensure ongoing consent, defined as a subject's willingness to continue participation for the duration of the research study.

Response: The research coordinator and study team are available to answer any question or concerns with the patient during the duration of the research trial. At each study visit, the patient is asked a series of questions to ensure they are on task with the study visits and feel comfortable. Upon departing from their study visit, the patients are told of their next visit and given detail instruction for their next visit. If study is revised or amendment or new information becomes available about drug safety that may affect patients participation, the patient may be re-consented to ensure patient ongoing consent

26.5 Indicate whether you will be following "SOP: Informed Consent Process for Research (HRP-090)." If not, or if there are any exceptions or additional details to what is covered in the SOP, describe:

- *The role of the individuals listed in the application who are involved in the consent process*
- *The time that will be devoted to the consent discussion*
- *Steps that will be taken to minimize the possibility of coercion or undue influence*
- *Steps that will be taken to ensure the subjects' understanding*

Response:

☒ We have reviewed and will be following "SOP: Informed Consent Process for Research (HRP-090)."

Non-English Speaking Subjects

☒ **N/A:** This study will not enroll Non-English speaking subjects.
(Skip to Section 26.8)

26.6 Indicate which language(s) other than English are likely to be spoken/understood by your prospective study population or their legally authorized representatives.

NOTE: The response to this Section should correspond with your response to Section 6.4 of this protocol.

Response:

26.7 If subjects who do not speak English will be enrolled, describe the process to ensure that the oral and written information provided to those subjects will be in that language. Indicate the language that will be used by those obtaining consent.

NOTE: Guidance is provided on "SOP: Informed Consent Process for Research (HRP-090)."

Response:

Cognitively Impaired Adults

☒ **N/A**: This study will not enroll cognitively impaired adults.
(Skip to Section 26.9)

26.8 Describe the process to determine whether an individual is capable of consent.

Response:

Adults Unable to Consent

☒ **N/A**: This study will not enroll adults unable to consent. (*Skip to Section 26.13)*

When a person is not capable of consent due to cognitive impairment, a legally authorized representative should be used to provide consent (Sections 26.9 and 26.10) ***and, where possible, assent of the individual should also be solicited*** *(Sections 26.11 and 26.12).*

26.9 Describe how you will identify a Legally Authorized Representative (LAR). Indicate that you have reviewed the "SOP: Legally Authorized Representatives, Children, and Guardians (HRP-013)" for research in New York State.

NOTE: Examples of acceptable response includes: verifying the electronic medical record to determine if an LAR is recorded.

Response:

☐

26.10 ***For research conducted outside of New York State****, provide information that describes which individuals are authorized under applicable law to consent on behalf of a prospective subject to their participation in the research. One method of obtaining this information is to have a legal counsel or authority review your protocol along with the definition of "legally authorized representative" in "SOP: Legally Authorized Representatives, Children, and Guardians (HRP-013)."*

Response:

26.11 Describe the process for ***assent of the adults****:*

- *Indicate whether assent will be obtained from all, some, or none of the subjects. If some, indicate which adults will be required to assent and which will not.*

Response:

- *If assent will not be obtained from some or all subjects, provide an explanation of why not.*

Response:

26.12 Describe whether ***assent of the adult*** *subjects will be documented and the process to document assent.*

NOTE: The IRB allows the person obtaining assent to document assent on the consent document using the "Template Consent Document (HRP-502)" Signature Block for Assent of Adults who are Legally Unable to Consent.

Response:

Subjects who are not yet Adults (Infants, Children, and Teenagers)

☒ **N/A**: This study will not enroll subjects who are not yet adults. *(Skip to Section 27.0)*

26.13 Describe the criteria that will be used to determine ***whether a prospective subject has not attained the legal age for consent to treatments or procedures involved in the research*** *under the applicable law of the jurisdiction in which the research will be conducted* ***(e.g., individuals under the age of 18 years)****. For research conducted in NYS, review "SOP: Legally Authorized Representatives, Children, and Guardians (HRP-013)" to be aware of which individuals in the state meet the definition of "children."*

NOTE: Examples of acceptable responses include: verification via electronic medical record, driver's license or state-issued ID, screening questionnaire.

Response:

26.14 ***For research conducted outside of New York State****, provide information that describes which persons have not attained the legal age for consent to treatments or procedures involved the research, under the applicable law of the jurisdiction in which research will be conducted. One method of obtaining this information is to have a legal counsel or authority review your protocol along the definition of "children" in "SOP: Legally Authorized Representatives, Children, and Guardians (HRP-013)."*

Response:

26.15 Describe whether parental permission will be obtained from:

Response: N/A

☐ One parent even if the other parent is alive, known, competent, reasonably available, and shares legal responsibility for the care and custody of the child.

☐ Both parents unless one parent is deceased, unknown, incompetent, or not reasonably available, or when only one parent has legal responsibility for the care and custody of the child.

☐ Parent permission will not be obtained. A waiver of parent permission is being requested.

NOTE: The requirement for parent permission is a protocol-specific determination made by the IRB based on the risk level of the research. For guidance, review the "CHECKLIST: Children (HRP-416)."

26.16 Describe whether permission will be obtained from individuals ***other than parents****, and if so, who will be allowed to provide permission. Describe your procedure for determining an individual's authority to consent to the child's general medical care.*

Response:

26.17 Indicate whether assent will be obtained from all, some, or none of the ***children****. If assent will be obtained from some children, indicate which children will be required to assent.*

Response:

26.18 When assent of children is obtained, describe how it will be documented.

Response:

27.0 Waiver or Alteration of Consent Process

Consent will not be obtained, required information will not be disclosed, or the research involves deception.

☒ **N/A:** A waiver or alteration of consent is not being requested.

27.1 If the research involves a waiver or alteration of the consent process, please review the "CHECKLIST: Waiver or Alteration of Consent Process (HRP-410)" to ensure that you have provided sufficient information for the IRB to make the determination that a waiver or alteration can be granted.

NOTE: For records review studies, the first set of criteria on the "CHECKLIST: Waiver or Alteration of Consent Process (HRP-410)" applies.

Response:

27.2 If the research involves a waiver of the consent process for planned emergency research, please review the "CHECKLIST: Waiver of Consent for Emergency Research (HRP-419)" to ensure you have provided sufficient information for the IRB to make these determinations. Provide any additional information necessary here:

Response:

28.0 Process to Document Consent

☐ **N/A:** A Waiver of Consent is being requested.
(Skip to Section 29.0)

28.1 Indicate whether you will be following "SOP: Written Documentation of Consent (HRP-091)." If not or if there are any exceptions, describe whether and how consent of the subject will be obtained including whether or not it will be documented in writing.

NOTE: If your research presents no more than minimal risk of harm to subjects and involves no procedures for which written documentation of consent is normally required outside of the research context, the IRB will generally waive the requirement to obtain written documentation of consent. This is sometimes referred to as 'verbal consent.' Review "CHECKLIST: Waiver of Written Documentation of Consent (HRP-411)" to ensure that you have provided sufficient information.

If you will document consent in writing, attach a consent document with your submission. You may use "TEMPLATE CONSENT DOCUMENT (HRP-502)". If you will obtain consent, but not document consent in writing, attach the script of the information to be provided orally or in writing (i.e. consent script or Information Sheet).

Response:

☒ We will be following "SOP: Written Documentation of Consent" (HRP-091).

29.0 Multi-Site Research (Multisite/Multicenter Only)

☐ **N/A:** This study is not an investigator-initiated multi-site study. This section does not apply.

29.1 If this is a multi-site study ***where you are the lead investigator****, describe the processes to ensure communication among sites, such as:*

- *All sites have the most current version of the IRB documents, including the protocol, consent document, and HIPAA authorization.*
- *All required approvals have been obtained at each site (including approval by the site's IRB of record).*
- *All modifications have been communicated to sites, and approved (including approval by the site's IRB of record) before the modification is implemented.*
- *All engaged participating sites will safeguard data as required by local information security policies.*
- *All local site investigators conduct the study appropriately.*
- *All non-compliance with the study protocol or applicable requirements will be reported in accordance with local policy.*

Response:

29.2 Describe the method for communicating to engaged participating sites:

- *Problems*
- *Interim results*
- *Study closure*

Response:

29.3 Indicate the total number of subjects that will be enrolled or records that will be reviewed across all sites.

Response: 114

29.4 If this is a multicenter study for which UB will serve as the IRB of record, and subjects will be recruited by methods not under the control of the local site (e.g., call centers, national advertisements) describe those methods.

Response: N/A

30.0 Banking Data or Specimens for Future Use

☐ **N/A:** This study is not banking data or specimens for future use or research outside the scope of the present protocol. This section does not apply.

30.1 If data or specimens will be banked (stored) for ***future use, that is, use or research outside of the scope of the present protocol****, describe where the data/specimens will be stored, how long they will be stored, how the data/specimens will be accessed, and who will have access to the data/specimens.*

NOTE: Your response here must be consistent with your response at the "What happens if I say yes, I want to be in this research?" Section of the Template Consent Document (HRP-502).

Response: The study data/specimens will be stored in a locked closet or -80 freezer at the research facility of the Diabetes and Endocrinology Center of WNY for at least 7 years.

The research staff (study personnel including coordinator) only will be authorized to access data and or specimens

30.2 List the data to be stored or associated with each specimen.

Response: Patient ID number, study visit information and date of collection will be stored with specimen. Other data stored will include record files of

all patients participating in the study, including data collection sheets, lab results, CGM and insulin pump data.

30.3 Describe the procedures to release banked data or specimens for future uses, including: the process to request a release, approvals required for release, who can obtain data or specimens, and the data to be provided with specimens.

Response: The copy of the individual patient data collected during the study period will be provided to these individual patients who can choose to hand carry it to their respective physicians and a copy will be faxed to their respective clinical providers upon verbal request from the patient. The data provided will include the insulin pump or CGM data or any of the lab results obtained during the study period. The results of the completed study will be made available to the patients if requested through published manuscript.

31.0 Drugs or Devices

☐ **N/A:** This study does not involve drugs or devices. This section does not apply.

31.1 If the research involves drugs or devices, list and describe all drugs and devices used in the research, the purpose of their use, and their regulatory approval status.

Response: **Drugs used:**

Description or Identity of investigational product(s)

Investigational product	***Dosage form and strength***	***Manufacturer***	***Description***
Dapagliflozin (Farxiga) 5 mg	*Green, plain, diamond shaped, film coated 5 mg tablet*	*AstraZeneca*	*sodium-glucose cotransporter 2 (SGLT2) inhibitor indicated as an adjunct to diet and exercise to improve glycemic control*
Matching placebo for dapagliflozin 5 mg	*Green, plain, diamond shaped, film coated tablet*	*AstraZeneca*	
Semaglutide (Ozempic) 0.25 mg, 0.5 mg, 1.0 mg	*clear, colorless solution that contains 2 mg of semaglutide in a 1.5 mL (1.34 mg/mL) pre-filled, disposable, single-patient-use pen injector*	*Novo Nordisk*	*glucagon-like peptide 1 (GLP-1) receptor agonist indicated as an adjunct to diet and exercise to improve glycemic control*

- *Dapagliflozin and its matching placebo tablets will be supplied in bottles and are provided by Astra Zeneca.*
- *Semaglutide will be supplied as prefilled injectable pens and will be purchased commercially through the University at Buffalo Research Pharmacy.*

Subjects will receive drugs in their unmodified form. Dapagliflozin is a once daily tablet, while Semaglutide is a weekly subcutaneous injection.

Insulin pumps (CSII) and long and short acting insulin is the only diabetes medication accepted before and during this trial. No other diabetes drug is permitted.
Blood pressure or lipid lowering drugs are permitted if on stable dose for at least three months prior to start of trial. Glucagon injection is permitted as rescue medication for severe hypoglycemia. Insulin infusions may be administered by ER or hospital if patients develop DKA.

31.2 Describe your plans to store, handle, and administer those drugs or devices so that they will be used only on subjects and be used only by authorized investigators.

Response: Dapagliflozin 5mg tablets and matching placebo provided by Astra Zeneca will be stored in a locked cabinet in research pharmacy. Semaglutide provided by University at Buffalo research pharmacy will be stored in temperature controlled refrigerator at 4C at the research facility of the Diabetes and Endocrinology Center of WNY at 1000 Youngs Rd until dispensed to patients. Investigational drugs will be dispensed by PI or CO-Is only to study patients according to randomization plan.

If the drug is investigational (has an IND) or the device has an IDE or a claim of abbreviated IDE (non-significant risk device), include the following information:

31.3 Identify the holder of the IND/IDE/Abbreviated IDE.

Response: IND application for use of all drugs (dapagliflozin and semaglutide) in type 1 diabetes will be submitted to FDA and no study procedure will start before all local and federal approvals are obtained. FDA has granted IND for usage of dapagliflozin in type 1 diabetes. We will seek FDA approval for the use of semaglutide in this study. These IND letters will be submitted to IRB and JDRF along with final version of protocol before initiating any study activities.

31.4 Explain procedures followed to comply with FDA sponsor requirements for the following:

	Applicable to:		
FDA Regulation	***IND Studies***	***IDE studies***	***Abbreviated IDE studies***
21 CFR 11	***X***	***X***	
21 CFR 54	***X***	***X***	
21 CFR 210	***X***		
21 CFR 211	***X***		
21 CFR 312	***X***		
21 CFR 812		***X***	***X***
21 CFR 820		***X***	

Response: All FDA sponsor requirements have been reviewed and will be followed during the study procedures

32.0 Humanitarian Use Devices

☒ **N/A:** This study does not involve humanitarian use devices. This does not apply.

32.1 For Humanitarian Use Device (HUD) uses provide a description of the device, a summary of how you propose to use the device, including a description of any screening procedures, the HUD procedure, and any patient follow-up visits, tests or procedures.

Response:

32.2 For HUD uses provide a description of how the patient will be informed of the potential risks and benefits of the HUD and any procedures associated with its use.

Response:

US TTT1 Protocol Summary of changes V6 to V7

Section	Original	New	Justification	Consent changes
4.1<br><br>5.1	-One hundred and (114) patients will be randomized (open label, see below) into three groups (38 each).<br><br>- 57 patients will be enrolled locally with another 57 patients recruited at the University of Glasgow site: a total of 114.	-Eighty-one (81) patients will be randomized (open label, see below) into three groups (27 each)<br>-Patients will be enrolled locally at UB site and at the University of Glasgow site for a total of 81. Enrollment will be open to both sites until target enrollment is achieved.	Due to slow enrollment into the study, the IDMC review of current data concluded that total sample size can be reduced from 114 to 81-82 patients without loss of power due to smaller than expected standard deviation of the primary endpoint. Details of this sample size change are included and reflected in the protocol and ICF.	Yes

University at Buffalo Institutional Review Board (UBIRB)
Office of Research Compliance | Clinical and Translational Research Center Room 5018
875 Ellicott St. | Buffalo, NY 14203
UB Federalwide Assurance ID#: FWA00008824

Complete Research Protocol (HRP-503)

Table of Contents

Template Instructions 2
1.0 Objectives 4
2.0 Scientific Endpoints 5
3.0 Background 6
4.0 Study Design 12
5.0 Local Number of Subjects 12
6.0 Inclusion and Exclusion Criteria 13
7.0 Vulnerable Populations 16
8.0 Eligibility Screening 17
9.0 Recruitment Methods 18
10.0 Procedures Involved 20
11.0 Study Timelines 33
12.0 Setting 33
13.0 Community-Based Participatory Research 34
14.0 Resources and Qualifications 35
15.0 Other Approvals 36
16.0 Provisions to Protect the Privacy Interests of Subjects 36
17.0 Data Management and Analysis 37
18.0 Confidentiality 40
A. Confidentiality of Study Data 40
B. Confidentiality of Study Specimens 41
19.0 Provisions to Monitor the Data to Ensure the Safety of Subjects 42
20.0 Withdrawal of Subjects 49
21.0 Risks to Subjects 50
22.0 Potential Benefits to Subjects 56
23.0 Compensation for Research-Related Injury 56
24.0 Economic Burden to Subjects 57
25.0 Compensation for Participation 58
26.0 Consent Process 58
27.0 Waiver or Alteration of Consent Process 63
28.0 Process to Document Consent 64
29.0 Multi-Site Research (Multisite/Multicenter Only) 64
30.0 Banking Data or Specimens for Future Use 65
31.0 Drugs or Devices 66
32.0 Humanitarian Use Devices 68

Template Instructions

Sections that do not apply:

- *In several sections, the addition of checkboxes for **Not Applicable** have been added to the template as responses.*
  - *If an N/A checkbox is present, select the appropriate justification from the list.*
  - *If an N/A checkbox is not present, or if none of the existing checkboxes apply to your study, you must write in your own justification.*
- *In addition:*
  - *For research where the only study procedures are records/chart review: Sections 19, 20, 22, 23, 24, 25, 31, and 32 do not apply.*
  - *For exempt research: Sections 31 and 32 do not apply.*

Studies with multiple participant groups:

- *If this study involves multiple participant groups (e.g. parents and children), provide information in applicable sections for each participant group. Clearly label responses when they differ. For example:*

  Response:

  Intervention Group:

  Control Group:

Formatting:

- *Do not remove template instructions or section headings when they do not apply to your study.*

If you are pasting information from other documents using the "Merge Formatting" Paste option will maintain the formatting of the response boxes.

Amendments:

- *When making modifications or revisions to this and other documents, use the **Track Changes** function in Microsoft Word.*
- *Update the version date or number **on Page 3.***

PROTOCOL TITLE:

Include the full protocol title.

Response: **Triple Therapy for Type 1 Diabetes with Insulin, Semaglutide, and Dapagliflozin**

PRINCIPAL INVESTIGATOR:

Response:

**Principal Investigator:**	**Paresh Dandona, MBBS, PhD**
**Co- Investigators:**	**Husam Ghanim, PhD**
	**Antoine Makdissi, MD**
	**Manav Batra, MD**
**Co- Principal Investigator:**	**John R Petrie, MBChB, PhD**

Paresh Dandona, M.B.B.S., Ph.D., F.R.C.P, F.A.C.P, F.A.C.C, FACE,
SUNY Distinguished Professor,
Director, Diabetes-Endocrinology Center of Western NY
Chief of Endocrinology, Diabetes, Endocrinology and Metabolism, SUNY at Buffalo
1000 Youngs Road, Suite 105
Williamsville NY 14221
Telephone: (716) 535-1850
Fax: 716-639-1200
E-mail: pdandona@kaleidahealth.org

CO-PI:
Professor John R Petrie
BSc MBChB PhD FRCP(Ed) FRCPSG
Professor of Diabetic Medicine
Institute of Cardiovascular and Medical Sciences
BHF Glasgow Cardiovascular Research Centre
University of Glasgow
126 University Place
Glasgow G12 8TA
Tel: +44(0)141 330 3325
Email: john.petrie@glasgow.ac.uk
Web: http://www.gla.ac.uk/researchinstitutes/icams/staff/johnpetrie/
The University of Glasgow

VERSION:

Include the version date or number.

Response: 2/10/2024, V7

GRANT APPLICABILITY:

Indicate whether this protocol is funded by a grant (e.g. NIH, foundation grant). For a grant with multiple aims, indicate which aims are covered by this research proposal.

NOTE: This question does not apply to studies funded by a sponsor contract.

Include a copy of the grant proposal with your submission.

Response: Research support provided by JDRF and AZ

RESEARCH REPOSITORY:

Indicate where the research files will be kept, including when the study has been closed. The repository should include, at minimum, copies of IRB correspondence (approval, determination letters) as well as signed consent documents. This documentation should be maintained for 3 years after the study has been closed.

Response:
Site 1: Diabetes Endocrinology Research Center of WNY
Address: 1000 Youngs Road, Suite 105, Williamsville NY 14221
Department: Diabetes Endocrinology

Site 2: University of Glasgow. Institute of Cardiovascular and Medical Sciences University of Glasgow 126 University Place Glasgow, G12 8TA

1.0 Objectives

1.1 Describe the purpose, specific aims, or objectives of this research.

Response:

Primary Objective: To assess whether the addition of dapagliflozin to semaglutide and insulin (triple therapy) improves glycemic control in patients with type 1 diabetes compared with semaglutide and insulin (dual therapy) and insulin only (standard) treatment.

General hypothesis: The addition of dapagliflozin to semaglutide and insulin treatment improves glycemic control, weight loss and blood pressure in adults with type 1 diabetes.

1.2 State the hypotheses to be tested, if applicable.

NOTE: A hypothesis is a specific, testable prediction about what you expect to happen in your study that corresponds with your above listed objectives.

Response:

Specific hypotheses:

Primary: combined dapagliflozin and semaglutide treatment in addition to insulin will improve HbA1c levels compared with semaglutide in addition to insulin .

Secondary 1: semaglutide in addition to insulin treatment over 26 weeks will improve HbA1c levels compared with insulin therapy alone.

Secondary 2: combined dapagliflozin and semaglutide treatment in addition to insulin will improve HbA1c levels compared to insulin therapy alone at 52 weeks.

Secondary 3: combined dapagliflozin and semaglutide treatment in addition to insulin will improve CGM-based weekly mean and fasting blood glucose, glycemic oscillations and percent of time in range (70-180mg/dl) with lower insulin requirements compared with semaglutide and insulin (dual therapy) or insulin only (standard) treatment.

Secondary : combined dapagliflozin and semaglutide in addition to insulin therapy will reduce systolic and diastolic blood pressure compared with semaglutide and insulin (dual therapy) or insulin only (standard) treatment.

Secondary 5: combined dapagliflozin and semaglutide in addition to insulin therapy (triple therapy) will result in weight loss compared to dual and standard therapy.

Secondary 6: combined dapagliflozin and semaglutide in addition to insulin therapy will result in improved scores on the Diabetes Specific Quality of Life Scale (DSQOLS) and the Problem Areas In Diabetes (PAID) survey compared with dual and standard therapy.

Tertiary and Exploratory Objectives (EO) for future ancillary studies (PLEASE NOTE: For EO, samples will be collected to facilitate the measurements of these end points in future ancillary studies once submitted and approved by JDRF so cost of assays is not included in the current budget): The addition of dapagliflozin to semaglutide and insulin in type 1 diabetes will:

Tertiary 1: combined dapagliflozin and semaglutide treatment in addition to insulin therapy will improve fasting and postprandial glucose excursions and suppress postprandial glucagon, FFA, GLP-1 compared with semaglutide and insulin or insulin therapy alone.

Tertiary 2: semaglutide in addition to insulin treatment over 52 weeks will improve HbA1c levels compared with insulin therapy alone.

EO 1: increase plasma concentrations of vasodilatation mediators and reduce plasma concentrations of vasoconstriction mediators compared to dual and standard therapy groups.

EO 2: exert a systemic anti-inflammatory effect including reducing mRNA expression of inflammatory mediators and NFκB binding in peripheral mononuclear cells (MNC) and adipose tissue (optional) and their circulating levels in plasma while increasing levels of IL-RA and adiponectin.

2.0 Scientific Endpoints

2.1 Describe the scientific endpoint(s), the main result or occurrence under study.

NOTE: Scientific endpoints are outcomes defined before the study begins to determine whether the objectives of the study have been met and to draw conclusions from the data. Include primary and secondary endpoints. Some example endpoints are: reduction of

symptoms, improvement in quality of life, or survival. Your response should ***not*** *be a date.*

Response:

Primary endpoint: Change in HbA1c at 6 months following dapagliflozin or placebo therapy in addition to combined semaglutide and insulin treatment.

Efficacy Secondary endpoints:

1. Change in HbA1c from baseline at 12 months in triple therapy group compared to insulin only group.
2. Change in HbA1c from baseline at 6 months in dual therapy group compared to insulin only group.
3. Change in percent Time in Range (70– 180 mg/dl), percent time in hyperglycemia Level 1(180-250mg/dl) and Level 2 (>250mg/dl) and percent time in hypoglycemia Level 1 (54-70mg/dl) as assessed by CGM
4. Other indices of glycemic control: fructosamine, weekly mean and fasting glucose, standard deviation, insulin requirements.
5. Change in body weight and quality of life as assessed by DSQOLS and PAID at 6 and 12 months.
6. Anti-hypertensive effects including change in systolic and diastolic BP, numbers of BP medications required.

Safety Secondary endpoints:

1- Differences in rates of hypoglycemic events Level 2 (<54mg/dl) and Level 3 (A severe event characterized by altered mental and/or physical status requiring assistance) between investigative arms and standard therapy arm.

2- *Differences in rates of diabetic ketoacidosis defined as elevated serum or urine ketones (greater than the upper limit of the normal range), and serum bicarbonate <15 mmol/L or Blood pH <7.3 between investigative arms and standard therapy arm.*

Tertiary/ exploratory endpoints: (items 2-4 to be performed in future on collected samples from this study if additional funding is approved by JDRF):

1- Fasting and postprandial (Area Under curve) glucose excursions, FFA, GLP-1 and glucagon and fasting fructosamine.

2- Indices of inflammatory stress. These will be CRP, FFA, SAA, IL-1β, IL-18, IL-1RA, adiponectin and TNFα concentrations in plasma and isoprostane in urine.

3- Expression of IL-1β, TNFα, IL-12, TLR2, TLR4, adiponectin, IκBα as well as NFκB binding in peripheral MNC and adipose tissue (optional).

4- Indices of vasodilatation and vasoconstriction: ANP, b-NP, cGMP, cAMP, angiotensinogen, renin and angiotensin II concentrations.

3.0 Background

3.1 Provide the scientific or scholarly background, rationale, and significance of the research based on the existing literature and how it will contribute to existing knowledge. Describe any gaps in current knowledge. Include relevant preliminary findings or prior research by the investigator.

Response:

The control of glucose homeostasis in type 1 diabetes is fragile since β-cell reserve is negligible. Traditionally, insulin has been the only treatment approach available. This approach, often leads to erratic blood sugar control given the challenge of matching exogenous insulin to mealtime glucose excursions in the face of a lack of any endogenous insulin to compensate for variation in requirements in response to meals and activity levels. Injected insulin boluses rarely match precise insulin requirements either in terms of dose administered or bio-availability. The result often is wide fluctuations in blood glucose readings. Based on our work to date and other published reports, previous attempts to add other glucose-lowering medications to insulin therapy yielded minimal benefits on glycemia (although metformin may have other benefits (1-3)

We have recently shown in two preliminary retrospective studies that the addition of liraglutide, a Glucagon Like Peptide -1 receptor agonist (GLP-1RA), to insulin results in consistent improvements in glycemic control in type 1 diabetes as demonstrated by reductions in HbA1c, glycemic excursions, and insulin requirements accompanied by improvements in body weight and systolic blood pressure (4, 5). We have now also completed the first prospective randomized controlled trial of this drug in 72 people with type 1 diabetes. Our data not only confirmed improvements in glycemic control, HbA1c, body weight and systolic BP, but also demonstrated a reduction in appetite, carbohydrate intake in low grade inflammation as measured by plasma CRP concentrations (6). Importantly, quality of life was also improved. Our recent studies in type 2 diabetes have revealed that systolic blood pressure reduction with GLP-1RA (exenatide) treatment in type 2 diabetes is associated with increased plasma concentrations of ANP, cGMP and cAMP along with reduced concentrations of angiotensinogen, renin and angiotensin II (7). It is possible that these mechanisms play a role in the prevention of cardiovascular complications in type 1 diabetes.

While GLP-1RAs, therefore, have great potential as adjunct therapy for type 1 diabetes, mean HbA1c was still ≥7% after liraglutide treatment (6) and larger pharma-sponsored trials reported increased rates of hypoglycemia (8, 9). In seeking further improvement, the advent of sodium-glucose transport protein-2 (SGLT2) inhibitors is of importance since their mode of action is independent of insulinogenesis. Introduced for use in type 2 diabetes, SGLT2 inhibitors are selective antagonists of the sodium-glucose transporter subtype expressed in the proximal convoluted tubules of the kidneys. They act by inhibiting renal glucose re-absorption and therefore stimulating excretion of 60-100 grams per day of glucose in urine. SGLT2 inhibitors have been studied extensively in type 2 diabetes, including the landmark cardiovascular outcome trial EMPA-REG (10), but data have only quite recently begun to accumulate in type 1(11). Short term effects on urine glucose excretion of remogliflozin and empagliflozin are similar to those seen in type 2 diabetes and these translate into improved glycemic and other indices including HbA1c; fasting blood glucose, rates of hypoglycemia, daily insulin dose and weight (12). Glucose-lowering efficacy is attenuated in individuals with eGFR $<45/ml/min/1.73m^2$(13). Therefore, use of dapagliflozin is recommended for patients with eGFR>45/ml/min/1.73 m^2 (as per prescribing information). Weight loss accompanying increased urinary glucose excretion could translate into reduced cardiovascular complications as in type 2 diabetes, but there are no data so far available to support this hypothesis. More recently, in the Phase 3 trial DEPICT-1 we showed that dapagliflozin improves glycemic control and HbA1c over six months by about 0.45% with no severe hypoglycemia or diabetic ketoacidosis

(14). Similarly, sotagliflozin has been shown in a Phase 3 trial to allow a larger proportion of patients to achieve HbA1c lower than 7.0% with no severe hypoglycemia although the rate of diabetic ketoacidosis was higher in the sotagliflozin group (15). Both products are now being reviewed by regulatory agencies for the type 1 diabetes indication.

On the basis of the above, we hypothesize that adding an SGLT2 inhibitor to a combination of insulin and semaglutide (a weekly and potent GLP-1RA) will improve glycemic control in type 1 diabetes such that HbA1c levels of ≤7% are achieved more consistently. Our protocol is designed to allow an objective assessment of whether this triple therapy combination will work effectively and safely so that a majority of people with type 1 diabetes can reach a glycemic target HbA1c of ≤7%. In addition, since both semaglutide and dapagliflozin inhibitors reduce blood pressure and weight, it will allow an assessment of the combined effect of GLP-1RAs and SGLT2 inhibitors on these outcomes. Since there are no previous data on the effects of once weekly semaglutide or the combination of semaglutide and dapagliflozin compared to standard insulin treatment in type 1 diabetes, the design requires allocation of some participants to insulin therapy only.

Due to the potential ketogenic effect of SGLT-2 inhibitors, data from SGLT-2 inhibitors studies in type 1 diabetes were analyzed for it ketogenic effects and a consensus statement and guidelines on management of patients was recently published (Danne T et al., Diabetes Care. 2019 Feb 6).

Preliminary data

1) Liraglutide treatment in patients with type 1 diabetes (4): We treated 8 type 1 diabetes patients; mean age (38.5 years) and duration of diabetes (20±4 yrs), with liraglutide in addition to insulin for 24 weeks. As shown in table 1, there was a significant reduction in fasting and mean blood glucose concentrations, insulin doses, HbA1c and a reduction in appetite and caloric intake with weight loss. In addition, there was a reduction in the excursions of blood glucose concentrations, i.e., the standard deviations of blood glucose concentrations were markedly reduced (**figure 1**). The changes were observed within 48h and improved marginally thereafter with fine tuning of insulin doses and a further reduction in these doses. These observations are noteworthy in that study participants were well controlled and meticulous users of CSII and CGM. They had all tried hard to improve their diabetic control by adjusting insulin doses but had not succeeded because of hypoglycemia. We then demonstrated that poorly controlled and obese individuals with type 1 diabetes also experienced similar benefits of liraglutide treatment (2).

2) Liraglutide As Additional Treatment to Insulin in Patients with Type 1 Diabetes Mellitus: A Randomized Clinical Trial (6): Following the two above non-randomized studies we recently conducted a randomized clinical trial in which 72 individuals (Placebo=18; liraglutide = 54) with TYPE 1 DIABETES for at least one year, on insulin therapy and with no detectable C-peptide in plasma (mean BMI: 30±1; mean body weight: 84±2 kg ; mean HbA1c: 7.57±0.09%; mean age: 44±2 years; mean age of TYPE 1 DIABETES diagnosis: 20±1 years) were randomized to receive placebo, 0.6, 1.2 or 1.8mg of liraglutide daily for 12 weeks. In the 1.2 mg and 1.8 mg groups, mean change in average blood glucose was -10±2 and -10.0±1mg/dl, respectively, ($p<0.0001$ vs. placebo). In the 1.2mg group, HbA1c fell by 0.78% from 7.84±0.17% to 7.06±0.15% ($p<0.0001$, $p<0.01$

vs. placebo) and in the 1.8mg group fell by 0.42% from 7.41±0.15 to 6.99±0.15 ($p=0.001$, $p=0.39$ vs placebo). Time spent between 70 to 160 mg/dl increased by 5±1% ($p<0.05$ vs placebo) and time between 160-400 mg/dl decreased by 7±1% ($p<0.01$ vs placebo) in the 1.8 mg group with no additional hypoglycemia. During the 12 week period, the average total daily dose of insulin in the two groups fell by 12.4±3.9 and 10.0±2.3 units respectively ($p<0.05$ vs baseline and placebo). There was a reduction in body weight (from 83±4 to 78±5 kg, $p<0.0001$, in the 1.8mg group, from 96±4 to 91±4 kg, $p<0.001$, in the 1.2 mg group and from 80±4 to 77±4 kg, $p<0.01$ in the 0.6 mg group). A reduction in daily carbohydrate intake (171±17g vs. 127±18g; $p<0.01$ and 153±18g vs 115±16g, $p<0.01$ in the 1.2 mg and 1.8 mg groups, respectively) was also observed over 12 weeks. Systolic BP (SBP) in the 1.8 mg group fell by 3 mmHg (120±2 to 117±3 mmHg, $p=0.01$). CRP fell by 15±6 and 19±8% in the 1.2 and 1.8mg groups, respectively ($p<0.05$). Quality of life improved significantly compared to baseline and placebo (unpublished data).

3) Dapagliflozin as an Additional Treatment to Insulin and Liraglutide in Patients with Type 1 Diabetes Mellitus (16): The objective of this randomized clinical trial was to investigate whether addition of dapagliflozin to insulin and liraglutide results in a significant reduction in glycemia and body weight. 30 participants with TYPE 1 DIABETES on liraglutide therapy for at least last 6 months were randomized (in 2:1 ratio) to receive either dapagliflozin 10 mg or placebo daily for 12 weeks. With 12 weeks of dapagliflozin HbA1c fell by 0.66±0.08% from 7.8±0.21% ($p < .01$ vs placebo) but did not change significantly with placebo (7.40±0.20% to 7.30±0.20%). In one patient within one week of addition of dapagliflozin average glucose fell from 170 to 122 mg/dl while standard deviation was reduced from 71 to 58 (**Figure 2-diminished glycemic excursions)**. Body weight fell by1.9 +/- 0.54kg ($p < 0.05$ vs placebo). There was no additional hypoglycemia (blood glucose < 3.88 mmol/L; $p = 0.52$ vs placebo). In the dapagliflozin group, there were significant increases in the plasma concentrations of glucagon by 35% +/- 13% ($p < 0.05$), hormone-sensitive lipase by 29% +/- 11% ($p < 0.05$), free fatty acids by 74% +/- 32% ($p < 0.05$), acetoacetate by 67% +/- 34% ($p < 0.05$), and beta-hydroxybutyrate by 254% +/- 81% ($p < 0.05$). Urinary ketone levels also increased significantly ($P < .05$). None of these changes was observed in the placebo group. Two individuals in the dapagliflozin group developed ketoacidosis. Overall, addition of dapagliflozin to insulin and liraglutide in patients with TYPE 1 DIABETES resulted in a significant improvement in glycemia and in weight loss but tended to increase ketosis.

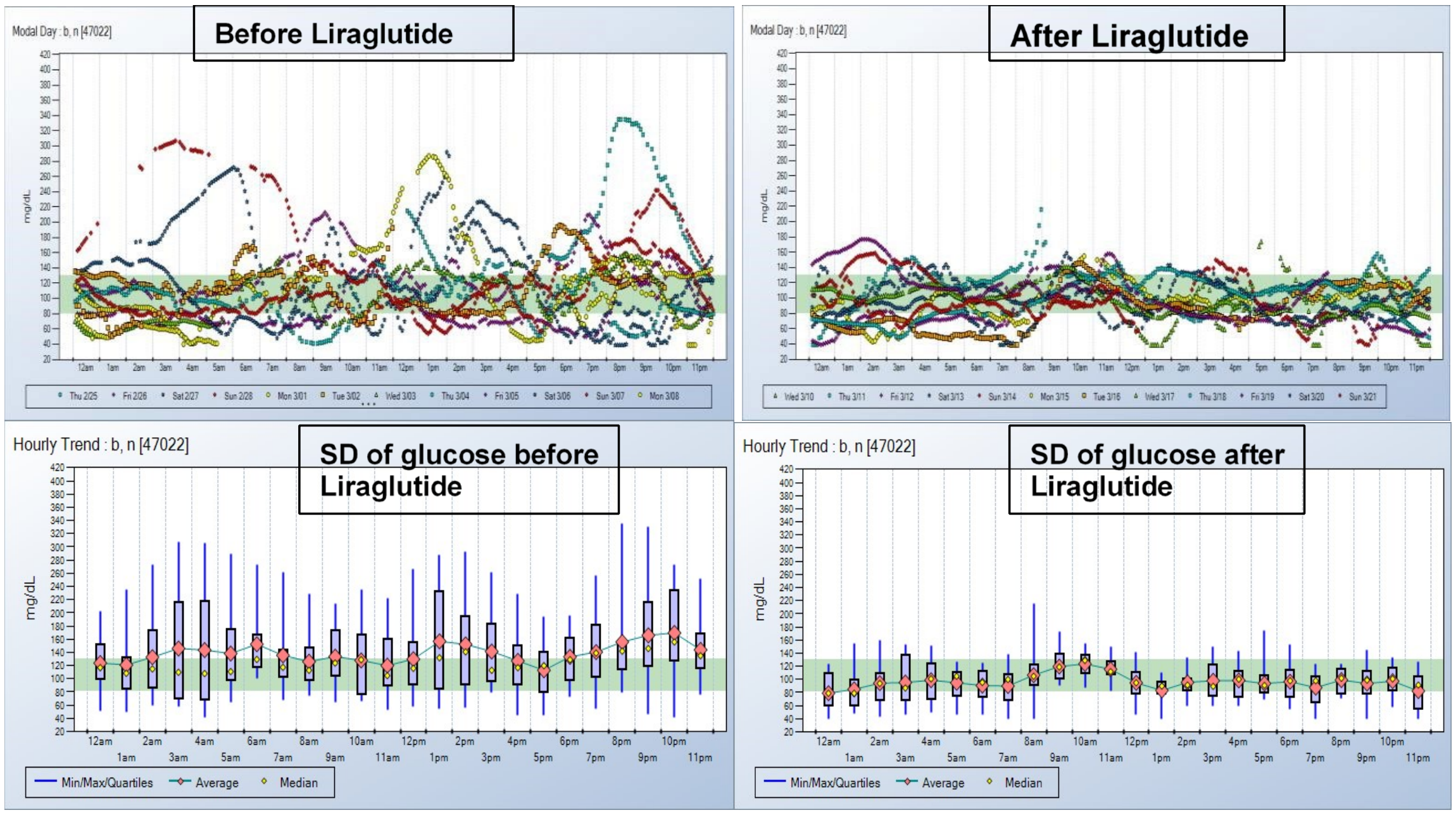

Figure 1: Weekly continuous glucose monitoring record of one patient prior to and following 1 week of liraglutide. Note the glycemic variability (SD) and the frequency of glucose concentrations within the target range prior to and following liraglutide

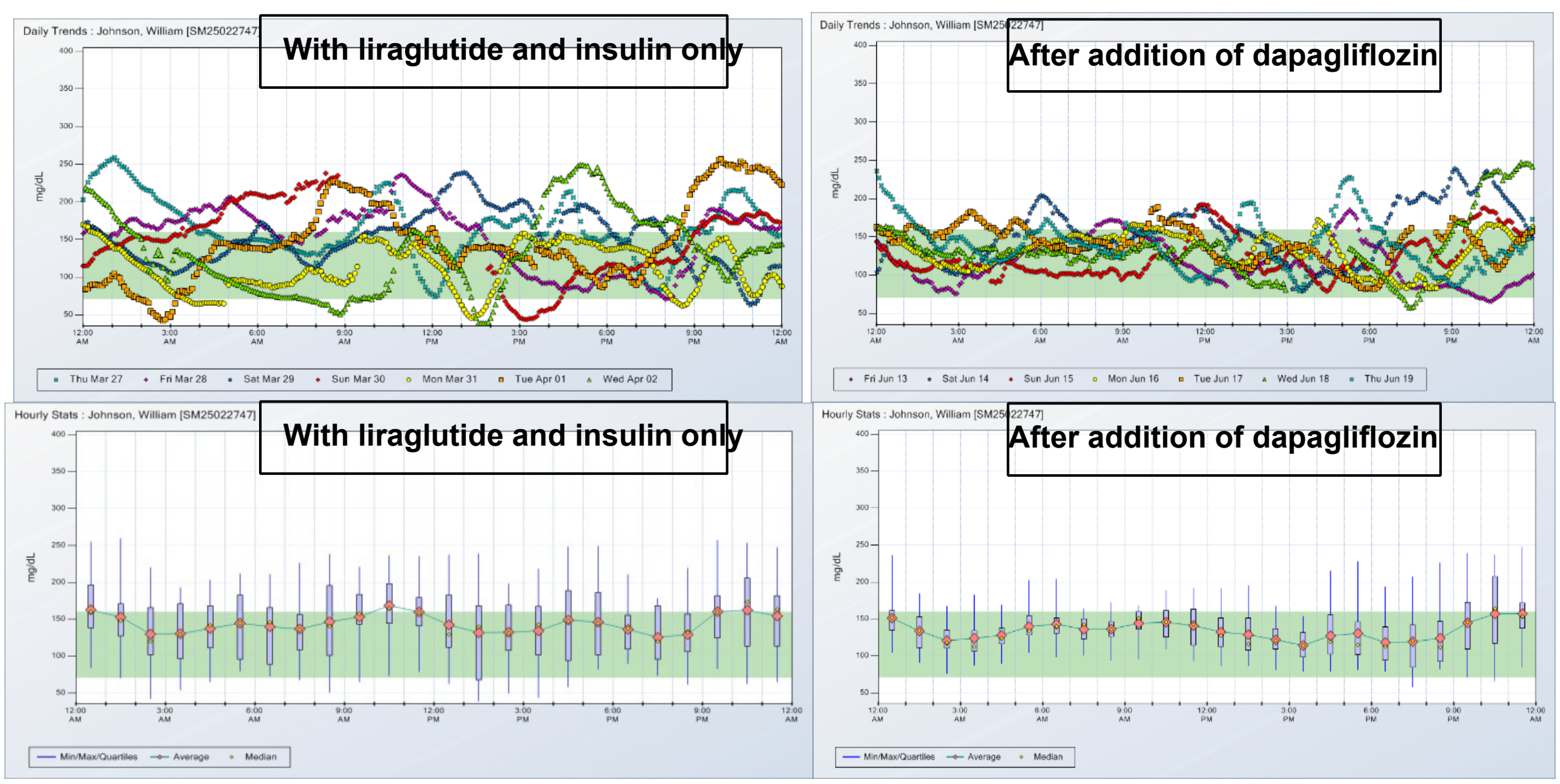

Figure 2: Weekly continuous glucose monitoring record of one patient prior to and following 8 weeks of the triple therapy combination with dapagliflozin and liraglutide. Note the glycemic variability (SD) (bottom) and the frequency of glucose concentrations within the target range prior to and following dapagliflozin addition.

3.2 Include complete citations or references.

Response:

1. Konrad K, Datz N, Engelsberger I, Grulich-Henn J, Hoertenhuber T, Knauth B, et al. Current use of metformin in addition to insulin in pediatric patients with type 1 diabetes mellitus: an analysis based on a large diabetes registry in Germany and Austria. *Pediatr Diabetes.* 2014.
2. Vella S, Buetow L, Royle P, Livingstone S, Colhoun HM, and Petrie JR. The use of metformin in type 1 diabetes: a systematic review of efficacy. *Diabetologia.* 2010;53(5):809-20.
3. Vella S, Buetow L, Royle P, Livingstone S, and Petrie JR. Metformin in type 1 diabetes reduces insulin requirements without significantly improving glycaemic control. Reply to Schatz H [letter]. *Diabetologia.* 2011;54(1):203-4.
4. Varanasi A, Bellini N, Rawal D, Vora M, Makdissi A, Dhindsa S, et al. Liraglutide as additional treatment for type 1 diabetes. *Eur J Endocrinol.* 2011;165(1):77-84.
5. Kuhadiya ND, Malik R, Bellini NJ, Patterson JL, Traina A, Makdissi A, et al. Liraglutide as additional treatment to insulin in obese patients with type 1 diabetes mellitus. *Endocrine practice : official journal of the American College of Endocrinology and the American Association of Clinical Endocrinologists.* 2013;19(6):963-7.
6. Kuhadiya ND, Dhindsa S, Ghanim H, Mehta A, Makdissi A, Batra M, et al. Addition of Liraglutide to Insulin in Patients With Type 1 Diabetes: A Randomized Placebo-Controlled Clinical Trial of 12 Weeks. *Diabetes Care.* 2016;39(6):1027-35.
7. Chaudhuri A, Ghanim H, Makdissi A, Green K, Abuaysheh S, Batra M, et al. Exenatide induces an increase in vasodilatory and a decrease in vasoconstrictive mediators. *Diabetes Obes Metab.* 2017;19(5):729-33.
8. Mathieu C, Zinman B, Hemmingsson JU, Woo V, Colman P, Christiansen E, et al. Efficacy and Safety of Liraglutide Added to Insulin Treatment in Type 1 Diabetes: The ADJUNCT ONE Treat-To-Target Randomized Trial. *Diabetes Care.* 2016;39(10):1702-10.
9. Ahren B, Hirsch IB, Pieber TR, Mathieu C, Gomez-Peralta F, Hansen TK, et al. Efficacy and Safety of Liraglutide Added to Capped Insulin Treatment in Subjects With Type 1 Diabetes: The ADJUNCT TWO Randomized Trial. *Diabetes Care.* 2016;39(10):1693-701.
10. Zinman B, Wanner C, Lachin JM, Fitchett D, Bluhmki E, Hantel S, et al. Empagliflozin, Cardiovascular Outcomes, and Mortality in Type 2 Diabetes. *N Engl J Med.* 2015;373(22):2117-28.
11. Petrie JR. SGLT2 inhibitors in type 1 diabetes: knocked down, but up again? *Lancet Diabetes Endocrinol.* 2017;5(11):841-3.
12. Mudaliar S, Armstrong DA, Mavian AA, O'Connor-Semmes R, Mydlow PK, Ye J, et al. Remogliflozin etabonate, a selective inhibitor of the sodium-glucose transporter 2, improves serum glucose profiles in type 1 diabetes. *Diabetes Care.* 2012;35(11):2198-200.
13. Kohan DE, Fioretto P, Tang W, List JF. Long-term study of patients with type 2 diabetes and moderate renal impairment shows that dapagliflozin reduces weight and blood pressure but does not improve glycemic control. Kidney Int. 2014 Apr;85(4):962-71.

14. Dandona P, Mathieu C, Phillip M, Hansen L, Griffen SC, Tschope D, et al. Efficacy and safety of dapagliflozin in patients with inadequately controlled type 1 diabetes (DEPICT-1): 24 week results from a multicentre, double-blind, phase 3, randomised controlled trial. *Lancet Diabetes Endocrinol.* 2017;5(11):864-76.
15. Garg SK, Henry RR, Banks P, Buse JB, Davies MJ, Fulcher GR, et al. Effects of Sotagliflozin Added to Insulin in Patients with Type 1 Diabetes. *N Engl J Med.* 2017;377(24):2337-48.
16. Kuhadiya ND, Ghanim H, Mehta A, Garg M, Khan S, Hejna J, et al. Dapagliflozin as Additional Treatment to Liraglutide and Insulin in Patients With Type 1 Diabetes. *J Clin Endocrinol Metab.* 2016;101(9):3506-15.

17. Andrews NC, and Faller DV. A rapid micropreparation technique for extraction of DNA-binding proteins from limiting numbers of mammalian cells. *Nucleic Acids Res.* 1991;19(9):2499.

4.0 Study Design

4.1 Describe and explain the study design (e.g. case-control, cross-sectional, ethnographic, experimental, interventional, longitudinal, observational).

Response: This will be a two-center, randomized, open-label then double-blind, parallel group, placebo and standard of care controlled prospective study in type 1 diabetes. Eighty-one (81) patients will be randomized (open label, see below) into three groups (27 each) and will be either maintained on their regular insulin therapy for 12 months (standard therapy group) or started on semaglutide in addition to regular insulin treatment for six month (dual therapy and triple therapy groups). For those on insulin and semaglutide, after six months of stable treatment, dapagliflozin (triple therapy group) or placebo (dual therapy group) treatment will be initiated for an additional six months according to a second randomization (double-blind).

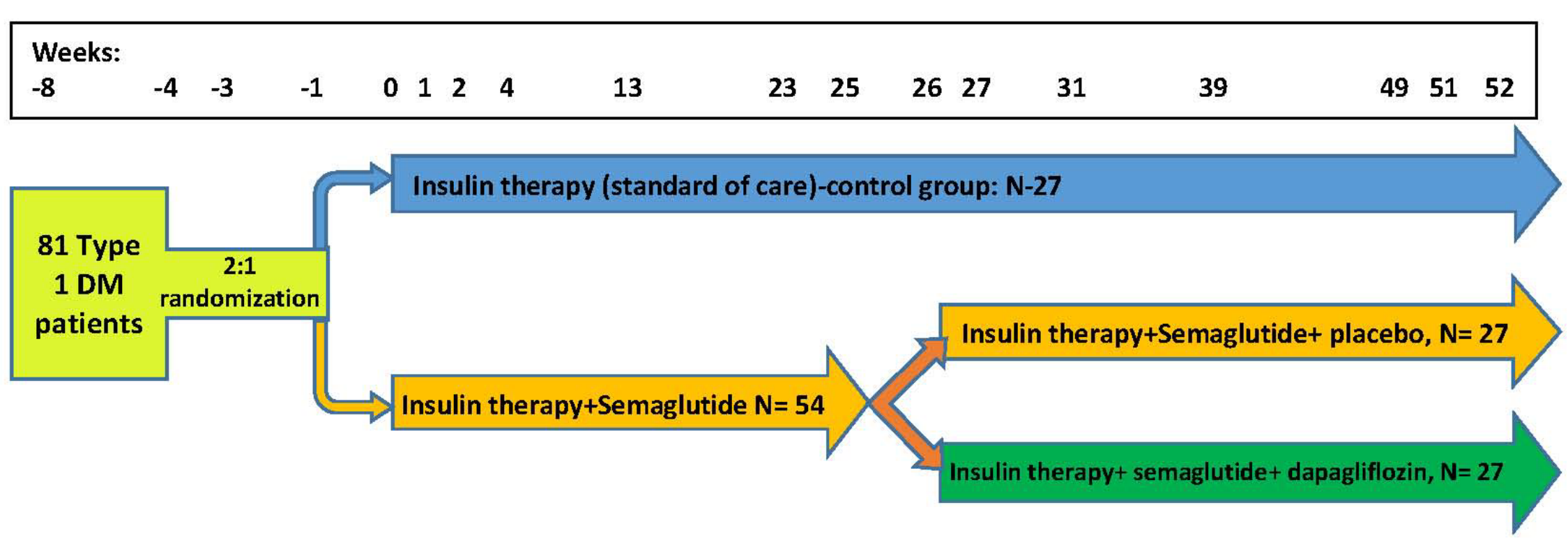

5.0 Local Number of Subjects

5.1 Indicate the total number of subjects that will be enrolled or records that will be reviewed locally.

Response: Patients will be enrolled locally at UB site and at the University of Glasgow site for a total of 81. Enrollment will be open to both sites until target enrollment is achieved.

5.2 If applicable, indicate how many subjects you expect to screen to reach your target sample (i.e. your screen failure rate).

Response: All screened and qualified patients will be enrolled and randomized

5.3 Justify the feasibility of recruiting the proposed number of eligible subjects within the anticipated recruitment period. For example, how many potential subjects do you have access to? What percentage of those potential subjects do you need to recruit?

Response: The Diabetes and Endocrinology Center of WNY is the largest Diabetes center in the WNY area, seeing between 60 to 100 type 1 diabetic patient every month. Therefore, the majority of recruited patients are our clinic patients. We also recruit a study participants through advertisement and other physician's references. These sources will suffice to recruit the needed number to subjects. The Glasgow center (Stobhill Ambulatory Care Hospital) reviews more than 300 patients per month.

6.0 Inclusion and Exclusion Criteria

6.1 Describe the criteria that define who will be ***included*** *in your final study sample.*

NOTE: This may be done in bullet point fashion.

Response:

Inclusion Criteria

1) Type 1 Diabetes for at least 1 year on staple use of continuous subcutaneous insulin infusion (CSII) or multiple daily (four or more) injections (MDI) of insulin for last 3 months.
2) C-peptide <0.23 nM
3) Minimum dose of insulin in Units/kg at entry: 0.5 U/kg for MDI and 0.4 U/kg for CSII
4) Regularly measuring blood sugars four or more times daily or use of CGM.
5) HbA1c between7.5-11% inclusive.
6) Well versed in CHO counting*
7) Age 18-70 years.
8) BMI $\geq$25 kg/m^2.

*In the opinion of the site Principal Investigator

6.2 Describe the criteria that define who will be ***excluded*** *from your final study sample.*

NOTE: This may be done in bullet point fashion.

Response:

Exclusion Criteria

1) Type 1 diabetes for less than 12 months, type 2 diabetes, chronic pancreatitis, MODY
2) Previous use of any agent other than insulin for treatment of diabetes in the last 3 months.
3) History of diabetic ketoacidosis (DKA) requiring medical intervention (e.g., emergency room visit and/or hospitalization) within 3 months prior to the screening visit
4) Patients on ketogenic or low (<50g/day) carbohydrate diet or who practice intermittent fasting.
5) Frequent episodes of severe hypoglycemia as defined by more than one episode requiring medical assistance, emergency care (paramedics or emergency room care), and/or glucagon therapy administered by a third-party individual within 1 month prior to the screening visit
6) Symptoms of poorly controlled diabetes that would preclude participation in this trial
7) Subjects on a commercial weight loss program with ongoing weight loss, or on an intensive exercise program
8) History of bariatric surgery or lap-band procedure within 12 months prior to screening
9) History of Addison's disease or chronic adrenal insufficiency
10) History of diabetes insipidus
11) Hepatic disease or cirrhosis
12) Aspartate Aminotransferase (AST) > 3X Upper limit of normal (ULN) *and/or* Alanine aminotransferase (ALT) > 3X ULN
13) Serum Total Bilirubin > 2X ULN unless exclusively caused by Gilbert's Syndrome
14) Hemoglobin < 11.0 g/dL (110 g/L) for men; hemoglobin < 10.0 g/dL (100 g/L) for women.
15) Coronary event or procedure (myocardial infarction, unstable angina, coronary artery bypass, surgery or coronary angioplasty) in the previous 3 months or patients with congestive heart failure.
16) ESRD on hemodialysis; and or e-GFR < 45 ml/min/1.73m^2
17) HIV or Hepatitis B/C positive status
18) Any other life-threatening, noncardiac disease
19) History of pancreatitis
20) Women who are pregnant or women of childbearing potential who are not using adequate contraception or who are breast feeding
21) Inability to give informed consent
22) History of gastroparesis
23) History of medullary thyroid carcinoma or MEN 2 syndrome
24) History of serious hypersensitivity reaction to these agents
25) Painful gallstones

26) Alcoholism
27) Hypertriglyceridemia (>500 mg/dl)
28) Recurrent genital mycotic infection.
29) Hypovolemic patients or with chronic renal insufficiency.
30) Patients with any malignancy except treated in situ malignancy and basal cell carcinoma of the skin
31) Unexplained hematuria
32) Patients with a history of diabetic retinopathy
33) Use of an investigational agent or therapeutic regimen within 30 days of study
34) Participation in any other concurrent interventional clinical trial

6.3 Indicate specifically whether you will include any of the following special populations in your study using the checkboxes below.

NOTE: Members of special populations may not be targeted for enrollment in your study unless you indicate this in your inclusion criteria.

Response: NA

☐ Adults unable to consent

☐ Individuals who are not yet adults (infants, children, teenagers)

☐ Pregnant women

☐ Prisoners

6.4 Indicate whether you will include non-English speaking individuals in your study. ***Provide justification if you will exclude non-English speaking individuals.***

In order to meet one of the primary ethical principles of equitable selection of subjects, non-English speaking individuals may ***not*** *be routinely excluded from research as a matter of convenience.*

In cases where the research is of therapeutic intent or is designed to investigate areas that would necessarily require certain populations who may not speak English, the researcher is required to make efforts to recruit and include non-English speaking individuals. However, there are studies in which it would be reasonable to limit subjects to those who speak English. Some examples include pilot studies, small unfunded studies with validated instruments not available in other languages, studies with numerous questionnaires, and some non-therapeutic studies which offer no direct benefit.

Response:

We have no non-English speaking patients in this population. We have patients that English is a second language, but they are able to read, write

and understand it. This population is less than 10% of the total population. No benefit is being withheld from not participating in the study.

7.0 Vulnerable Populations

*If the research involves special populations that are considered vulnerable, **describe the safeguards included to protect their rights and welfare.***

NOTE: You should refer to the appropriate checklists, referenced below, to ensure you have provided adequate detail regarding safeguards and protections. You do not, however, need to provide these checklists to the IRB.

*7.1 For research that involves **pregnant women,** safeguards include:*
NOTE CHECKLIST: Pregnant Women (HRP-412)

Response: We will not be recruiting subjects from vulnerable populations

☒ **N/A**: This research does not involve pregnant women.

*7.2 For research that involves **neonates of uncertain viability or non-viable neonates,** safeguards include:*
NOTE CHECKLISTS: Non-Viable Neonates (HRP-413), or Neonates of Uncertain Viability (HRP-414)

Response:

☒ **N/A:** This research does not involve non-viable neonates or neonates of uncertain viability.

*7.3 For research that involves **prisoners**, safeguards include:*
NOTE CHECKLIST: Prisoners (HRP-415)

Response:

☒ **N/A:** This research does not involve prisoners.

*7.4 For research that involves **persons who have not attained the legal age for consent to treatments or procedures involved in the research ("children")**, safeguards include:*
NOTE CHECKLIST: Children (HRP-416)

Response:

☒ **N/A:** This research does not involve persons who have not attained the legal age for consent to treatments or procedures ("children").

*7.5 For research that involves **cognitively impaired adults**, safeguards include:*
NOTE CHECKLIST: Cognitively Impaired Adults (HRP-417)

Response:

☒ **N/A:** This research does not involve cognitively impaired adults.

7.6 Consider if other specifically targeted populations such as students, employees of a specific firm, or educationally or economically disadvantaged persons are vulnerable. ***Provide information regarding their safeguards and protections, including safeguards to eliminate coercion or undue influence.***

Response: No specific populations or vulnerable groups will be targeted. All subjects enrolled in this study will be of legal adult consenting age with the ability to speak, read and interpret the English language. Patients will have the ability to speak with the research team regarding any questions or concern they have before signing the consent. Research team have the ability to evaluate patient's cognitive abilities through these interactions with patients. Patients are made aware that this study is voluntary and they are able to stop participating at any time they feel uncomfortable. Patients are not to be pressured into participating and their clinic standard of care will remain the same if they participate or choose not to participate.

8.0 Eligibility Screening

8.1 Describe ***screening procedures*** *for determining subjects' eligibility. Screening refers to determining if prospective participants meet inclusion and exclusion criteria.*

Include all relevant screening documents with your submission (e.g. screening protocol, script, questionnaire).

Response:

After obtaining partial HIPAA waiver, patient charts will be screened according to the study inclusion and exclusion criteria by our trained clinical staff and physicians. In additions, the study clinical team will evaluate their clinic patients for possible participation in this study according to the inclusion and exclusion criteria at the Diabetes and Endocrinology Center of WNY clinics. Physicians will speak to them about their interests in participating in research. If the patient agrees, their information will be given to the research coordinator to be contacted for further evaluation. The research coordinator will contact interested patients and those meeting the inclusion and exclusion criteria based on preliminary phone evaluation will be invited to participate in the study. All personal information will be kept confidential and locked in the coordinator office.

Prospective participants will be asked to read the consent and any questions they may have regarding the protocol will be answered. If the subject wants to participate in the study, they will be asked to sign the informed consent form. The subject's medical history and current medications will be obtained as well as their blood pressure and vitals. A physical examination will also be done. Blood and urine samples will be taken in order to evaluate HbA1c, CBC, CMP and liver and kidney functions and pregnancy status. Females of childbearing age, will have to be on two forms of birth control to prevent pregnancy. Patients meeting all the

inclusion and exclusion criteria based on all screening tests will be enrolled in the study.

☐ **N/A:** There is no screening as part of this protocol.

9.0 Recruitment Methods

☐ **N/A:** This is a records review only, and subjects will not be recruited. NOTE: If you select this option, please make sure that all records review procedures and inclusion/exclusion screening are adequately described in other sections.

9.1 Describe when, where, and how potential subjects will be recruited.

NOTE: Recruitment refers to how you are identifying potential participants and introducing them to the study. Include specific methods you will use (e.g. searching charts for specific ICD code numbers, Research Participant Groups, posted advertisements, etc.).

Response:

Participants will be identified by prescreening clinical charts, patient doctor interaction at the time of their visits to the Diabetes Endocrinology Center of WNY Locations include:

1. 1020 Youngs Road, Williamsville NY 14221
2. 705 Maple Road, Williamsville NY 14221
3. 462 Grider Street, Buffalo NY 14215
4. 1000 Youngs Road, Suite 105, Williamsville NY 14221

Patients can be also referred by other physicians and providers

The remainder of the participants (57 patients) will be recruited from NHS Greater Glasgow and Clyde Diabetes clinics (Scotland, UK). Separate local IRB/ Ethical approval will be sought to cover this site (based on discussions with UBIRB director).

The study clinical team will evaluate their clinic patients for possible participation in this study according to the inclusion and exclusion criteria at the Diabetes and Endocrinology Center of WNY clinics. Patients that may qualify for the study are referred to the research team for further eligibility evolution. Patients meeting the inclusion and exclusion criteria based on preliminary phone evaluation will be invited to participate in the study.

Participants will also be identified by:

-Flyers, advertisements on Facebook, ,

- Researchmatch.org, UB's participate in research (PIR) website, and through Buffalo research registry (BRR).

- We will also use the TriNetX/i2b2 database through UB's Institute for Healthcare Informatics (IHI) to recruit participants from the UBMD medical database.

Along with this, a radio ad will be on the air of the radio station STAR102.5 at various times during the day during the month of mid January to mid February

9.2 *Describe how you will protect the privacy interests of prospective subjects during the recruitment process.*

NOTE: Privacy refers to an individual's right to control access to him or herself.

Response: Patient charts will be screened according to the study inclusion and exclusion criteria by our trained clinical staff and physicians. If the patient qualifies and is of consenting age, the physicians will speak to them about their interests in participating in research in a private room where no one else can hear the conversation. If the patient agrees, their information will be given to the research coordinator to be contacted for further evaluation. No personal information will be shared without patient's approval and will not be used for any purpose except for this research. All personal information will be kept confidential and locked in the coordinator office.

The privacy of patients recruited using the TriNetX/ i2b2 data will be protected by following the UB IRB-approved procedure. A letter will be sent to the eligible patient's physician (see attached). After one week, if the physician doesn't contact the research team to refuse, a recruitment letter will be sent to participants via mail (see attached). If we don't hear from the patients within one week, one follow-up call will be made (see attached phone script). If at any point the physician or the patient declines, the patient will be removed from the contact list.

The Clinical and Translational Science Institute's (CTSI) Community Engagement Team (CET) hosts the Buffalo Research Registry (BRR, IRB Approved STUDY00000806), a resource that connects researchers looking to recruit participants and community members looking to get involved with research. To participate in the registry, volunteers complete a voluntary intake form. Volunteers have agreed to be contacted about potential research opportunities based on their self-reported information.

As described in the BRR's IRB Approved Protocol (STUDY00000806), key personnel from the CTSI will serve as the conduit between our research team and the registry volunteers. Key personnel will verify our IRB study approval, review inclusion and exclusion criteria and will sort the registry data accordingly. In terms of recruiting for our study, key personnel will pull volunteer reports using the inclusion and exclusion criteria. Key personnel will centrally invite volunteers to participate in the study and provide a warm hand off to our team. There are two ways we are sharing our study. These include:

Electronically- Using the volunteer report from REDCap, key personnel from the CTSI will send initial e-mail invites to introduce our study to registry volunteers.

Post Mail- Key personnel from the CTSI will provide our team with contact information (i.e. first name, last name, post mail address) to prepare a mailing. This information will be shared in a password protected excel spread sheet. The password to the spreadsheet will be sent in a separate follow-up e-mail. We will

only use the list for this protocol and we agree to destroy the list once recruitment for the study has closed.

We also plan on conducting follow-up by phone/email after initial contact. This contact information will be provided in a password protected excel spread sheet. The password to the spreadsheet will be sent in a separate follow-up e-mail. We will only use the list for this protocol and we agree to destroy the list once recruitment for the study has closed.

We will have no access to the health information provided in the registry.

9.3 Identify any materials that will be used to recruit subjects.

NOTE: Examples include scripts for telephone calls, in person announcements / presentations, email invitations.

For advertisements, include the final copy of printed advertisements with your submission. When advertisements are taped for broadcast, attach the final audio/video tape. NOTE: You may submit the wording of the advertisement prior to taping to ensure there will be no IRB-required revisions, provided the IRB also reviews and approves the final version.

Response: In addition to screening clinical charts, participations will be identified through; flyer advertisement, researchmatch.org, and Facebook. The study will also be put on UB's PIR website.

The researchmatch.org contact message and listing is provided as separate attachment to this submission (RM IRB Template Language-JDRF)

Sample letters of physician permission and patient contact and phone prescreen script are also included in this submission.

10.0 Procedures Involved

*10.1 Provide a description of **all research procedures or activities** being performed and when they are performed once a subject is screened and determined to be eligible. Provide as much detail as possible.*

NOTE: This should serve as a blueprint for your study and include enough detail so that another investigator could pick up your protocol and replicate the research. For studies that have multiple or complex visits or procedures, consider the addition of a schedule of events table in in your response.

Response:

Each patient will spend about 55 weeks in the trial comprising three weeks of monitoring to stabilize baselines and ensure adherence and commitment to participation before randomization into an intervention arm for an additional period of 52 weeks. Following a completed screening visit and meeting the inclusion and exclusion criteria, each patient will have a total of 16 study visits. At weeks 3, 5, 8, 16, 20, 29, 35, 44 and at 2 weeks after the end of the study, each patient will receive a phone call to document any adverse effects or other changes. There will be no long term follow-up following the end of the study.

Participants will be treated with either insulin only (control arm) for the whole duration of the study or with insulin and semaglutide weekly s.c. injection (titrated up to 1 mg or the maximum tolerated dose, see below) for the first six months then either dapagliflozin

(Triple therapy arm) or placebo (dual therapy) daily will be added for an additional six months. Semaglutide will be started at a dose 0.25 mg once weekly for two weeks then increased to 0.5 mg dose. If well tolerated it will be increased to 1 mg once weekly at week 4. Dapagliflozin (or placebo) will be started at a dose of 5 mg daily at week 26 and if well tolerated increased after 1 week to 10 mg (2x5mg tablets) daily to ensure drug tolerability and to avoid any hypoglycemia or hypotension. Patients who do not tolerate the increase in dosing of either medications will be maintained at the highest tolerated dose.

Treatments used

1- Insulin: All patients (81 patients) will continue to self-administer their prescribed insulin as part of their standard of care. Insulin dose will be titrated during the study to control blood glucose average between 90-180 mg/dl and to avoid hypoglycemia (blood sugar <70 mg/dl) or hyperglycemia (blood sugar >250 mg/dl). Among all patients, 3827 patients will be randomized (open-label) to remain on insulin therapy only (standard therapy group) while the other 76 will start the investigational therapies.
2- Semaglutide: a once weekly a GLP-1 mimetic injection (pen) indicated for type 2 diabetes. Doses to be used: start at 0.25mg then increase to 0.5mg following 2 weeks and to 1mg weekly s.c. injection 2 weeks later. Semaglutide will be added to a background of insulin treatment for 12 months in 76 patients who will be randomized (double-blind) into 2 groups (dual and triple therapy groups) at the end of 26 weeks.
3- Farxiga (dapagliflozin): a once daily SGLT-2 inhibitor tablet indicated for type 2 diabetes. Doses used: starting with 5mg and increase after 1 week to 10mg oral administration daily. Dapagliflozin will be started according to the randomization (Interactive Web System, hosted by University of Glasgow) in 3827 patients at 26 weeks as an additional treatment on a background of insulin and semaglutide therapy and end at 52 weeks (triple therapy group).
4- Placebo: dapagliflozin matching placebo (supplied by Astra Zeneca) will be started in 27 patients at 26 weeks as an additional treatment on a background of insulin and semaglutide therapy and end at 52 weeks (dual therapy group).

As shown in **Table 1,** fasting blood and urine samples will be obtained prior to drug administration (weeks 0) and at 13, 26, 39 and 52 weeks after the commencement of the study. Optional fat biopsies from the abdominal subcutaneous tissue will be collected at weeks 0, 26 and 52. Continuous Glucose Monitoring (Dexcom G6 with blinded receiver) will be performed for three 21-days periods on weeks -3 to 0, 23 to 26 and from 49 to 52 weeks. An optional 24hr blood pressure monitoring will be performed at baseline (week -1) and at 13, 25, 39 and 51 weeks in order to characterize the time course of antihypertensive effects, which may be early (i.e. independent of weight loss) as indicated by our preliminary data. High fat high caloric (HFHC) Meal challenges will be performed at baseline (week -1) and at 25 and 51 weeks with assessment of glucose excursions and postprandial glucagon (AUC) as well as postprandial inflammation and oxidative stress. Telephone calls will be conducted at weeks 3, 5, 8, 16, 20, 29, 35, 44 and at two weeks

after the end of the study to document any side effects or any other patient's reported issues or comments.

Study Visits:

<u>Screening Visit (Screening Visit, Week -8 to -4):</u> Patients will be asked to read and understand the consent and any questions regarding the protocol will be answered. If they want to participate in the study, they will be asked to sign the informed consent form. Their medical history and current medications will be obtained as well as blood pressure and vitals. A physical examination will also be done. Blood samples will be taken in order to evaluate HbA1c, CBC, CMP, C-peptide, kidney and liver functions, ketone bodies, and urine for pregnancy status in females.

<u>Study Visit 1 (Week -3):</u> Participants who meet entry criteria will be scheduled to for their next visit at -3 week. Vitals will be taken including resting (min. 10 minutes) blood pressure measurement (will be repeated from same arm every visit). Insulin pump will be downloaded and/or CGM/fingerstick blood sugar will be reviewed and the appropriate insulin adjustments will be made by the investigator to target fasting blood glucose concentrations between 90-180 mg/dl if possible at all times without having additional hypoglycemia (less than 70 mg/dl). Blood sugars and ketone bodies will be measured via a fingerstick blood sample using glucose and ketones meter (Abbott Laboratories) and daily blood sugars collected and reviewed. Urine for pregnancy status and ketone bodies assessment. We will instruct patients on how to use and insert the Continuous Glucose Monitoring System (Dexcom G6 with blinded receiver). All subjects will be advised to monitor their capillary blood glucose by CGM or fingerstick at least 4 times a day (before meals and at bedtime) through-out the study and to wear their CGM constantly for next 20 day. Carb counting technique will be reviewed and any questions answered on how to use the pump or calculate insulin doses.

<u>Study Visit 2 (Week -1±2days):</u> Patients will be asked to come in the fasting state (no food or calorie intake for 10-12 hours). Their vitals will be taken. Blood sugars and ketone bodies will be measured via a CGM/fingerstick blood sample using glucose and ketones meter (Abbott Laboratories) to assess their glycemic control prior to meal challenge. If their blood sugar is below 70mg/dl or more than 250mg/dl the visit will be rescheduled for an alternative date. Insulin pump will be downloaded and/or CGM/fingerstick blood sugar will be reviewed and insulin adjustment will be made if necessary. An IV port will be placed in order to obtain blood samples throughout the duration of the visit. Patients will undergo a meal challenge test (described below). At the end of the meal test, they will be asked to wear a 24 hour blood pressure monitor (optional). A 24 hr urine container will be dispensed to collect a 24 hour urine sample to bring in at the Week 0 visit. Daily Glucose logs will be reviewed.

<u>Study visit 3 (Week 0±2days):</u> Patients will be asked to come in the fasting state (no food or calorie intake for 10-12 hours). Vitals will be taken. Blood sugars and ketone bodies will be measured via a CGM/fingerstick blood sample using glucose and ketones meter

(Abbott Laboratories) and daily blood sugars collected and reviewed. The 24 hour urine container will be collected. A fasting blood sample will be taken (45 mL) to evaluate study endpoints, CBC, CMP, HbA1c, and fructosamine. A urine sample will be collected to evaluate ketone bodies and, in females, pregnancy status. Insulin pump will be downloaded and/or CGM/fingerstick blood sugar will be reviewed and the appropriate insulin adjustments will be made by the investigator. In general, insulin dose adjustments should not be >10% from current total insulin dose or >20% from baseline (week 0) total insulin dose. They will discontinue use of the study CGM. An optional fat biopsy will be performed. We will ask them to fill out quality of life questionnaires. Study drug will be dispensed depending on what group they are randomized to. This includes either (1) staying on the insulin regimen given by their doctor, or (2) a combination of their current insulin regimen and a once daily injection of semaglutide. Semaglutide will be started on site at the 0.25 mg dose. The 24 hr blood pressure monitor will be collected.

Study Visit 4 (Week 1±2days): Vitals will be taken. Blood sugars and ketone bodies will be measured via a CGM/fingerstick blood sample using glucose and ketones meter (Abbott Laboratories) and daily blood sugars collected and reviewed. Insulin dose will be adjusted if needed as in visit 3. At any time, if HbA1c is <7%, insulin dose adjustment should not be >5%. Semaglutide 0.25mg will be injected on site. Participants will be instructed to call their research center or their doctor directly in case of any problem or untoward side effects. They will be specifically asked to call if they have unmanaged hypoglycemia (blood sugar <70 mg/dl) or hyperglycemia (blood sugar >250 mg/dl) or if occurring in more than one occasion.

Study Visit 5 (Week 2±2days): Vitals will be taken. Blood sugars and ketone bodies will be measured via a CGM/fingerstick blood sample using glucose and ketones meter (Abbott Laboratories) and daily blood sugars collected and reviewed. Insulin dose will be adjusted if needed as in visit 3. Semaglutide 0.5mg dose will be injected on site. If they are experiencing mild nausea, they will be continued on the 0.25 mg dose for another week. Supply of the drug will be given to last until the next visit. Patients will be advised to increase the study dose to 0.5 mg a day if they are ok.

Study Visit 6 (Week 4±1 week): Patients will be asked to bring in used study pens for drug accountability (to be recorded in eCRF). Vitals will be taken. Blood sugars and ketone bodies will be measured via a CGM/fingerstick blood sample using glucose and ketones meter (Abbott Laboratories) and daily blood sugars collected and reviewed. Insulin dose will be adjusted if needed. Supply of the drug will be given to last until the next visit. A urine sample will be collected to evaluate ketone bodies and, in females, pregnancy status. Patients will be advised to increase the study dose to 1.0 mg/week. If they experience mild nausea, they will be continued on the 0.5 mg dose for another week. At all times, patients will be maintained at the maximum tolerated dose. A 24 hr urine container will be dispensed to collect a 24 hour urine sample to bring in at the Week 13 visit.

Study Visit 7 (Week 13±1 week): Patients will be asked to come in the fasting state (10-12 hours fasting), and to bring in your used study pens for drug accountability. Blood sugars and ketone bodies will be measured via a CGM/fingerstick blood sample using

glucose and ketones meter (Abbott Laboratories) and daily blood sugars collected and reviewed. Insulin dose will be adjusted if needed as in. Supply of the drug will be given to last until visit 26. Vitals will be taken. A urine sample will be collected to evaluate ketone bodies and, in females, pregnancy status. The 24 hour urine container will be collected. A fasting blood sample will be taken (45 mL) to evaluate study endpoints, CBC, CMP, HbA1c, and fructosamine. Patients who agreed to participate in the24 hour blood pressure monitoring will be asked to wear it for the next 24 hr.

Study Visit 8 (Week 23±1 week): Patients will be asked to bring in used study pens for drug accountability. Vitals will be taken. Blood sugars and ketone bodies will be measured via a CGM/fingerstick blood sample using glucose and ketones meter (Abbott Laboratories) and daily blood sugars collected and reviewed. A urine sample will be collected to evaluate ketone bodies and, in females, pregnancy status. Insulin dose will be adjusted if needed. All subjects will be reminded to monitor their capillary blood glucose by fingerstick at least 4 times a day (before meals and at bedtime) through-out the study. CGM will be inserted and patients asked to wear their CGM constantly for next 20 day.

Study visit 9 (Week 25±1 week): Patients will be asked to come in the fasting state, and to bring in used study pens for drug accountability. Vitals will be taken. A urine sample will be collected to evaluate ketone bodies. Blood sugars and ketone bodies will be measured via a CGM/fingerstick blood sample using glucose and ketones meter (Abbott Laboratories) and daily blood sugars collected and reviewed. Insulin dose will be adjusted if needed. HbA1c will be drawn. Supply of the drug will be given to last until the next visit. A 24 hr urine container will be dispensed to collect a 24 hour urine sample to bring in at the Week 26 visit. A HFHC Meal challenges will be performed as in visit 2. Patients who agreed to participate will be asked to wear a 24 hour blood pressure monitoring for the next 24 hr.
Participants in the semaglutide group will be provided supplies for urine ketones measurement (KetoStix) and blood ketones meter and strips. Research coordinator will instruct the patients on the use of meter and reading of the measuring strips. Patients will be instructed to start measuring urinary and blood ketones every morning for three consecutive days prior to next visit (starting dapagliflozin treatment) and to report results at the time of their next visit.

Study visit 10 (Week 26±1 week): Patients will be asked to come in the fasting state, and to bring in used study pens for drug accountability. Previous 3 days blood and urine ketones levels will be reviewed. Patients with normal fasting urine (<+, or < small) or blood ketone (<0.6 mmol) will proceed to next treatment phase included in this visit. Patients who report higher ketone values in at least 1 out of the 3 days will have their glucose levels, insulin dose, carb intake reviewed and managed as necessary. They will be rescheduled for next week (7±1 day) and asked to repeat the 3 days testing before next visit.
Vitals will be taken. Blood sugars and ketone bodies will be reviewed via a CGM/fingerstick blood sample using glucose and ketones meter (Abbott Laboratories) and daily blood sugars collected and reviewed. Insulin dose will be adjusted if needed. In general, insulin dose adjustments should **NOT** be >10% from current total insulin dose (or 5% if HbA1c is <7%) or >20% from baseline total insulin dose. The adjusted total insulin

dose in this visit will serve as the baseline total insulin dose for the remaining 6 months of the study and future limits of dose adjustments will be based on this new baseline. The CGM will be collected. A urine sample will be collected to evaluate ketone bodies and, in females, pregnancy status. The 24 hour urine container will be collected. A fasting blood sample will be taken (45 mL) to evaluate study endpoints, CBC, CMP, C-peptide, and Fructosamine. An optional fat biopsy will be performed. We will ask patients to fill out quality of life questionnaires. At this point, those patients randomized to receive semaglutide, they will now be randomized to receive an actual dapagliflozin pill or a placebo pill that looks like dapagliflozin. If they are randomized to receive actual dapagliflozin, they will start at the 5 mg dose for one week. Supply of the study drug will be given to last until week 27.

All patients will be asked to perform a daily (preferably early morning or before bedtime) urinary test of ketone bodies using KetoStix and record all outcomes until the end of the study. Also, they will be provided with additional blood ketone strips to measure blood ketones when needed. If results of these daily scheduled (i.e. not done due to symptoms) urine ketone measurements, are small or higher (+1 or more), they will be instructed to measure blood ketones and to repeat both measurements after 60 min. If consecutive measurements show moderate or higher ketones in urine or blood ketones >0.6mM, the patient will be instructed to call the research center or their doctor directly. For any abnormal ketone levels taken due to symptoms, the DKA consensus guidelines (Danne T et al., Diabetes Care. 2019 Feb 6) are to be followed instead (as in section 21).

Participants will be instructed to call their research center or their doctor directly in case of any problem or untoward side effects. They will be specifically asked to call if they have unmanaged hypoglycemia (blood sugar <70 mg/dl) or hyperglycemia (blood sugar >250 mg/dl) or if occurring in more than one occasion. Participants will be instructed to remain hydrated and report any genital mycotic or urinary tract infections.

Study visit 11 (Week 27±2 days): Patients will be asked to bring in used study pens for drug accountability (recorded in eCRF). Vitals will be taken. Daily blood sugars and urinary ketones measurements using KetoStix will be collected and reviewed. Blood sugars and ketone bodies will be measured via a CGM/fingerstick blood sample using glucose and ketones meter (Abbott Laboratories). A urine sample will be collected to evaluate urine ketone level. Insulin dose will be adjusted if needed. In general, insulin dose adjustments should not be >10% from current total insulin dose (or 5% if HbA1c is <7%) or >20% from NEW baseline total insulin dose established at visit 10. If at any time during the study total insulin dose reduction is >20% and total insulin dose in <0.40 U/Kg, additional monitoring protocol (see later) will be implemented. Supply of the drug will be given to last until week 39. If patients are receiving actual dapagliflozin, they will now start at the 10 mg dose for the remainder of the study.

Study Visit 12 (Week 31±1 week): Patients will be asked to bring in used study pens and tablets for drug accountability. Vitals will be taken. Daily blood sugars and urinary ketones measurements using KetoStix will be collected and reviewed. Blood sugars and ketone bodies will be measured via a CGM/fingerstick blood sample using glucose and ketones meter (Abbott Laboratories). A urine sample will be collected to evaluate ketone bodies and, in females, pregnancy status. Insulin dose will be adjusted if needed as in visit 11. A

blood sample will be taken (15 mL) to evaluate CBC and CMP. A 24 hr urine container will be dispensed to collect a 24 hour urine sample to bring in at the Week 39 visit.

Study visit 13 (Week 39±1 week): Patients will be asked to come in the fasting state, and to bring in used study pens and tablets for drug accountability. Vitals will be taken. Daily blood sugars and urinary ketones measurements using KetoStix will be collected and reviewed. Blood sugars and ketone bodies will be measured via a CGM/fingerstick blood sample using glucose and ketones meter (Abbott Laboratories). Insulin dose will be adjusted if needed as in visit 11. Supply of the drug will be given to last until the end of the study. A urine sample will be collected to evaluate ketone bodies and, in females, pregnancy status. The 24 hour urine container will be collected. A fasting blood sample will be taken (45 mL) to evaluate study endpoints, CBC, CMP, HbA1c, and Fructosamine. Patients who have provided consent will be asked to wear a 24 hour blood pressure monitor.

Study visit 14 (Week 49±1 week): Patients will be asked to bring in used study pens and tablets for drug accountability. Vitals will be taken. Daily blood sugars and urinary ketones measurements using KetoStix will be collected and reviewed. Blood sugars and ketone bodies will be measured via a CGM/fingerstick blood sample using glucose and ketones meter (Abbott Laboratories). A urine sample will be collected to evaluate ketone bodies and, in females, pregnancy status. Insulin dose will be adjusted if needed as in visit 11. All subjects will be reminded to monitor their capillary blood glucose by fingerstick at least 4 times a day (before meals and at bedtime) through-out the study. CGM will be inserted and patients asked to wear their CGM constantly for the next 20 days.

Study visit 15 (Week 51±1 week): Patients will be asked to come in the fasting state, and to bring in used study pens and tablets for drug accountability. Vitals will be taken. Daily blood sugars and urinary ketones measurements using KetoStix will be collected and reviewed. Blood sugars and ketone bodies will be measured via a CGM/fingerstick blood sample using glucose and ketones meter (Abbott Laboratories). A urine sample will be collected to evaluate urine ketone levels. Insulin dose will be adjusted if needed as in visit 11. A 24 hr urine container will be dispensed to collect a 24 hour urine sample to bring in at the Week 52 visit. A HFHC Meal challenges will be performed as in visit 2. Patients will be asked to wear the optional 24 hour blood pressure monitor.

Study visit 16 (Week 52±2 days): Patients will be asked to come in the fasting state, and to bring in used study pens and tablets for drug accountability. Vitals will be taken. Daily blood sugars and urinary ketones measurements using KetoStix will be collected and reviewed. Blood sugars and ketone bodies will be measured via a CGM/fingerstick blood sample using glucose and ketones meter (Abbott Laboratories). Insulin dose will be adjusted if needed. The CGM will be collected. An optional fat biopsy will be performed. A urine sample will be collected to evaluate ketone bodies and, in females, pregnancy status. The 24 hour urine container will be collected. A fasting blood sample will be taken (45 mL) to evaluate study endpoints, CBC, CMP, HbA1c, C-peptide, and Fructosamine. We will ask the patients to fill out quality of life questionnaires. The patient will receive a physical. After this visit, they will be discharged from the study.

Safety phone calls will be conducted at weeks 3, 5, 8, 16, 20, 29, 35, 44 and at two weeks after the end of the study to document any side effects or other changes.

Table 1: Schedule of visits and testing procedures.

Visit #	-	1	2	3	4	5	6	7	8	9	10	11	12	13	14	15	16
Week#(±2-7 days as per text)	-8 to -4	-3	-1	0	1	2	4	13	23	25	26	27	31	39	49	51	52
Study Procedures	Screening	Run-in period		Randomization, start semaglutide							Randomize/Start dapagliflozin/ placebo						End of study
History	X																
Physical	X																X
Vitals	X	X	X	X	X	X	X	X	X	X	X	X	X	X	X	X	X
CBC	X			X				X			X		X	X			X
CMP	X			X				X			X		X	X			X
C-Peptide	X										X						X
Kidney and liver profiles	X			X				X			X		X	X			X
Pregnancy test	X	X		X			X	X	X		X		X	X	X		X
Study drug titration						X	X					X					
Dispense medications				X	X	X	X	X			X	X		X			
HbA1c	X			X				X		X				X			X
Fructosamine				X				X			X			X			X
24 hour BP (optional)			X					X		X				X		X	
CGM		X	X						X	X					X	X	
Daily Fingerstick glucose review		X	X	X	X	X	X	X	X	X	X	X	X	X	X	X	X
Daily urinary ketones review											X	X	X	X	X	X	X
Insulin dose review		X	X	X	X	X	X	X	X	X	X	X	X	X	X	X	X
Ketone bodies (blood/urine)	X	X	X	X	X	X	X	X	X	X	X	X	X	X	X	X	X
Adverse Event reporting		X	X	X	X	X	X	X	X	X	X	X	X	X	X	X	X
Meal Challenge			X							X						X	
Blood endpoints				X				X			X			X			X
Fat biopsy (optional)				X							X						X
24 hr Urine				X				X			X			X			X
Quality of life questionnaires				X							X						X

Drug Blinding:

The randomizations will be performed using an Interactive Web System, hosted by University of Glasgow. Eighty-one (81) patients will be randomized as follows: participants will first be randomized to semaglutide plus usual insulin therapy or insulin therapy only (no placebo for semaglutide or other placebo injection will be provided), in a 2:1 ratio. Those who remain on semaglutide at six months will then be randomized in a 1:1 ratio to receive dapagliflozin or matched placebo. Each randomization will be stratified by study site and the second randomization will also include HbA1c stratification. The randomization schedules will be generated by a statistician with no other involvement in the trial, using the method of randomized permuted blocks. Only those staff responsible for the maintenance of the randomization system will have access to the randomization sequences during the trial. The first randomization (which will be revealed to the researcher at the point of randomization) will use random block sizes to reduce predictability. Researchers at study sites will have no knowledge of the next allocation to be issued. The second randomization (which will be double-blind) will use a different. At the point of randomization, the researcher will be given a medication pack ID number, but will not know whether that medication pack contains Dapagliflozin or placebo. For each randomization, a web-based randomization system (developed and maintained by the Robertson Centre for Biostatistics) will be used. The system will include an emergency unblinding facility for the dapagliflozin/placebo randomization

Astra Zeneca Inc. will supply dapagliflozin and matching placebo tablets in unlabeled and identical packaging; semaglutide pens will be obtained commercially and will be used in an open label fashion.

Breaking of the study blind will be performed only: (i) for SUSARs; and, (ii) where knowledge of the treatment is considered by local health personnel absolutely necessary for further management of the patient. A central unblinding facility based at the Robertson Centre for Biostatistics, University of Glasgow, will be available by telephone. Notification of all unblinding will be sent to the Chief Investigator

During the course of the trial there will be no attempt to identify group assignment for any participant (except for unblinding in the case of safety concerns) and the investigators will execute the protocol in the manner it was approved independent of the group assignment.

Drugs will be stored in a locked cabinet or locked temperature controlled refrigerator at 4C at the research facility of the Diabetes and Endocrinology Center of WNY. IND application for use of all drugs (dapagliflozin and semaglutide) in type 1 diabetes will be submitted to FDA and no study procedure will start before all local and federal approvals are obtained.

Clinical and Laboratory Procedures

1) **CMP, CBC, HbA1c, fructosamine, glucose, glucagon, GLP-1 and FFA measurements:** Blood CMP, CBC, HbA1c, fructosamine and glucose will be measured through Quest diagnostic (US site) and NHS labs (UK site). Free fatty acid levels will be measured by a colorimetric assay (Wako, Richmond, VA). GLP-1 and

glucagon will be measured from samples collected in protease and DPP-IV inhibitors by ELISA kits from Millipore.

2) **Meal Challenge test:** In order to assess the postprandial changes induced by dapagliflozin, semaglutide or the combination of both compared to insulin only, a meal challenge will be performed before starting treatments, at six months and at end of study using a 910 Calorie High fat High carbohydrate meal (HFHC) as in several of our previous studies (HFHC meal includes an egg muffin sandwich, a sausage muffin sandwich and two hash browns which contain 88g carbohydrate, 51 g fat (33% saturated) and 34 g protein). Participants will be asked to attend fasting prior to breakfast insulin bolus; basal insulin will continue during the duration of the meal, however patients will not bolus until the 3 hour mark unless blood glucose values reach above 400 mg/dL. Sequential blood samples will be obtained at 0, 15, 30, 45, 60, 90, 120, 150 and 180 min after consuming the meal. Samples at 15, 30, 45, 90 and 150 min will be 5ml while those at 0, 60, 120 and 180min will be 30 ml (total volume=145 ml). Spot urine will be collected at baseline, 1 and 3hr. Blood will be collected from an indwelling intravenous canula in a superficial forearm vein.
3) **Optional 24 hour Ambulatory BP monitoring:** Participants will be instructed on the use of 24 hour ambulatory BP monitoring device. This device will measure BP and pulse every 30 minutes for 24 hours. In addition to the scheduled measurements there will be an option for additional manual measurements if systolic blood pressure is >160 or <90 mmHg.
4) **Optional Fat aspiration procedure:** Abdominal subcutaneous fat tissue aspiration will be performed at a 10 cm distance from umbilicus. Participants will be asked not to take aspirin or NSAIDS in the previous 72 hours - if they have, the procedure will not be done. Skin will be prepared with povidone-iodine (Betadine) and alcohol. A sterile drape will be placed around the appropriate area. 3cc of 1% lidocaine will then be administered subcutaneously (the dose will not exceed 4.5mg/kg body weight). After adequate anesthesia has been achieved, aspiration of fat tissue will then be with a 3-holed canula (Tulip Instrumentation, length: 15cm, diameter: 2.1mm) fixed to a 10mL syringe. After getting adequate fat tissue (500mg-3g), the puncture site will be pressed for at least ten minutes before the patient rises up from supine position (to minimize bruising). Participants will then be discharged home. Adipose tissue will be centrifuged to remove blood and fluid contaminants. Upper adipose tissue will be collected into a separate sterile tube, washed twice with cold sterile Phosphate Buffered Saline (PBS) and centrifuged to remove the PBS. The adipose tissue sample will be weighed and approximately 500 mg transferred to a separate tube for analysis. Total RNA, nuclear extracts and total cell lysates will be prepared.
5) **Questionnaires:** Participants will be asked to complete the standard Diabetes Specific Quality of Life Scale (DSQOLS) and Problem Areas In Diabetes survey (PAID). Questionnaires attached at the end of this plan.
6) **MNC isolation (for future ancillary studies):** Blood samples will be collected in Na-EDTA as an anticoagulant. Three and a half mL of anticoagulated blood sample are carefully layered over 3.5 mL of PMN medium (Cedarlane Laboratories, Hornby, ON) and then centrifuged. At the end of this process, two bands separate out at the top of the RBC pellet. The top band consists of MNC, while the bottom consists of PMN. The

MNC band is harvested and washed twice with Hank's balanced salt solution (HBSS). This method provides yields greater than 95% pure PMN and MNC suspensions.

7) **NFκB DNA binding activity (for future ancillary studies):** Nuclear NFκB DNA binding activity is measured by EMSA. Nuclear extract is prepared from MNC and adipose tissue by high salt extraction as described by Andrews et al (17). The specificity of the bands is confirmed by supershifting these bands with specific antibodies against Rel-A (p65) and p50 (Santa Cruz Biotechnology, CA) and by competition with cold oligonucleotides. DNA binding activity of NFκB will be adjusted to Oct1 DNA binding activity to correct for any variable.
8) **Plasma concentrations of inflammatory mediators (for future ancillary studies):** Concentrations of TNFα, IL-1β, IL-18, adiponectin, IL-1RA will be measured in plasma using ELISA kits (R&D Systems, MN). CRP and SAA concentrations will be measured using an ELISA kit from Alpha Diagnostics International Inc. (San Antonio, TX).
9) **Plasma vasodilators and vasoconstrictors (for future ancillary studies):** Plasma ANP, NT-proBNP, b-NP, cGMP, cAMP, angiotensinogen, renin and angiotensin II concentrations will be measured by commercially available ELISA kits.
10) **15-isoprostane F_{2t}** (also known as 8-epi-PGF2α or 8-iso-PGF2α) **(for future ancillary studies):** will be measured in urine samples using an ELISA kit from Oxford Biomedical Research (Oxford, MI).
11) **Quantification of mRNA in MNC and adipose tissue (for future ancillary studies).** Total RNA is isolated from MNC and adipose tissue using commercially available RNAqueous®-4PCR Kit (Ambion, Austin, TX). The quality and quantity of the isolated RNA is determined before using the RNA. Real Time RT-PCR is performed using Cepheid Smart Cycler (Sunnyvale, CA) in which 2 µL cDNA, 10 µL Sybergreen Master mix (Qiagen, CA) along with 0.5 µL of 20 µM gene specific primers for IL-1β, IL-RA, TLR-4, TNFα, IL-12, SOCS-3 and JNK-1 (Life Technologies, MD) are used. The specificity and the size of the PCR products are tested by adding a melt curve at the end of the amplifications and by running it on a 2% agarose gel. All values are normalized to 18S expression and β-actin.

10.2 Describe what data will be collected.

NOTE: For studies with multiple data collection points or long-term follow up, consider the addition of a schedule or table in your response.

Response:

Data will be collected as described in table 1 above. The following aspects of the study will be performed and data collected and reported by patients:

1- A CGM device will be provided for use for 3 20-days periods. Detailed instructions on changing the sensor at 10 days will be provided. Patients will be instructed to wear their CGM constantly during the assigned study periods. They will be asked to keep a diary of their food intake to measure their carbohydrate intake during the CGM period. Insulin doses will be titrated aiming for blood glucose between 90-180 mg/dl while avoiding hypoglycemia (<70 mg/dl) or hyperglycemia (>250 mg/dl).

2- Participants who agree to participate in the 24-hr ambulatory blood pressure monitoring will be provided with a 24-hour ambulatory BP monitor to be worn for 24 hours during week -1, 13, 25, 39 and 51.
3- All participants will be asked to monitor capillary blood glucose by finger stick before each meal and at bedtime.
4- For the entire duration of the study, the patients will maintain a diary to record any hypoglycemia and other untoward side effects e.g. nausea, change in appetite.
5- Starting at the 6 months mark, participants will be asked to perform a daily urinary test of ketone bodies using KetoStix and record all outcomes. If results are moderate to large, they will be instructed to repeat the test and to call the research center or their doctor directly if results are confirmed. Patients with dual glucose and ketones meters will be instructed to record and report their blood ketones as well. If blood ketones are above 0.6mM, they should repeat the test and contact the research center of their doctor if levels are confirmed.

10.3 List any instruments or measurement tools used to collect data (e.g. questionnaire, interview guide, validated instrument, data collection form).

Include copies of these documents with your submission.

Response: Source documents will be used to collect patient information. The CGM or insulin pump will be used to collect glucose and insulin data. Glucose and ketone meter will be used to measure glucose and ketone in blood. KetoStix for measuring ketones in urine. Blood pressure monitor will be used for 24 hour blood pressure reading. Glucose, food and urinary ketones diaries will be provided for patients to record their results.

10.4 Describe any source records that will be used to collect data about subjects (e.g. school records, electronic medical records).

Response: electronic medical records and research files.

10.5 Indicate whether or not ***individual*** *subject results, such as results of investigational diagnostic tests, genetic tests, or incidental findings will be shared with subjects or others (e.g., the subject's primary care physician) and if so, describe how these will be shared.*

Response: Individual participant lab results will be disclosed to the participant upon their request. If the participant requests documentation be shared with another physician, physician office or hospital the participant must come to the research center to collect said documentation or the documentation can be mailed to their given home address.

Patients will be assigned a unique study number. All identifiable personal information will be stored locally in locked filing cabinets. Data entered on the

electronic Case Report Form will only be identified by the unique study number. Thus, no personal information will be shared with the data center (Robertson Centre for Biostatistics, University of Glasgow, UK).

10.6 Indicate whether or not ***study*** *results will be shared with subjects or others, and if so, describe how these will be shared.*

Response: Not Applicable. Study results will not be shared with the subjects. However, unidentifiable study results could be published in the form of a manuscript or abstract and will be reported to JDRF, Astra Zeneca, and to clinicaltrials.gov and other regulatory agencies including FDA.

11.0 Study Timelines

11.1 Describe the anticipated duration needed to enroll all study subjects.

Response: Accrual is expected to be at the rate of 2 subjects/month/site, thus we should complete enrollment of 57 patients/site in approximately 26-30 months. We expect to complete all study clinical activities in 48 months.

11.2 Describe the duration of an individual subject's participation in the study. Include length of study visits, and overall study follow-up time.

Response: 55 Weeks. Study visits will be 60-90 minutes long except for visits 2, 9 and 15 which will be about 4hr long each

11.3 Describe the estimated duration for the investigators to complete this study (i.e. all data is collected and all analyses have been completed).

Response: 60 Months

12.0 Setting

12.1 Describe all facilities/sites where you will be conducting research procedures. Include a description of the security and privacy of the facilities (e.g. locked facility, limited access, privacy barriers). Facility, department, and type of room are relevant. Do not abbreviate facility names.

NOTE: Examples of acceptable response may be: "A classroom setting in the Department of Psychology equipped with a computer with relevant survey administration software," "The angiogram suite at Buffalo General Medical Center, a fully accredited tertiary care institution within New York State with badge access," or, "Community Center meeting hall."

Response:

1) Research will be conducted at the Diabetes Endocrinology Research Center of WNY, located at *1000 Youngs Road, Suite 105, Williamsville NY 14221* and at the CTRC located in 875 Ellicott St. Buffalo NY14203.The Diabetes Research Center has facilities and exam rooms available for insulin pump download, CGM device download, meal and infusion studies and presence of study coordinator and registered nurse for data collection and blood work at all times. One of the investigators will be available at all times to address patients' related issues. CTRC location is a fully equipped laboratory with Equipment include ultra-low freezers for sample storage, centrifuges, microscopes for sample preparation, infusion pumps, ELISA, PCR and immunoblotting instrumentation.

2) Research will also be conducted at the NHS Greater Glasgow and Clyde Clinical Research Facility (CRF, Glasgow Royal Infirmary, UK and at the Diabetes Centre (Clinic D), Stobhill Ambulatory Care Hospital, Glasgow, UK. There are facilities and exam rooms available for insulin pump download, CGM device download, meal and infusion studies and presence of research nursing staff for data collection and blood work at all times. One of the investigators will be available at all times to address patients' related issues. The CRF has equipment for sample preparation, infusion pumps, ultra-low freezers for sample storage and centrifuges. ELISA, PCR and immunoblotting facilities are available in adjacent NHS and University facilities.

12.2 For research conducted outside of UB and its affiliates, describe:

- *Site-specific regulations or customs affecting the research*
- *Local scientific and ethical review structure*

NOTE: This question is referring to UB affiliated research taking place outside UB, i.e. research conducted in the community, school-based research, international research, etc. It is not referring to multi-site research. UB affiliated institutions include Kaleida Health, ECMC, and Roswell Park Cancer Institute.

Response:

☒ **N/A:** This study is not conducted outside of UB or its affiliates.

13.0 Community-Based Participatory Research

13.1 Describe involvement of the community in the design and conduct of the research.

NOTE: Community-Based Participatory Research (CBPR) is a collaborative approach to research that equitably involves all partners in the research process and recognizes the unique strengths that each brings. CBPR begins with a research topic of importance to the community, has the aim of combining knowledge with action and achieving social change to improve health outcomes and eliminate health disparities.

Response:

☒ **N/A:** This study does not utilize CBPR.

13.2 Describe the composition and involvement of a community advisory board.

Response:

☒ **N/A:** This study does not have a community advisory board.

14.0 Resources and Qualifications

14.1 Describe the qualifications (e.g., education, training, experience, expertise, or certifications) of the Principal Investigator ***and*** *staff to perform the research. When applicable describe their knowledge of the local study sites, culture, and society. Provide enough information to convince the IRB that you have qualified staff for the proposed research.*

NOTE: If you specify a person by name, a change to that person will require prior approval by the IRB. If you specify a person by role (e.g., coordinator, research assistant, co-investigator, or pharmacist), a change to that person will not usually require prior approval by the IRB, provided that the person meets the qualifications described to fulfill their roles.

Response: All study personnel are educated, trained, and licensed as required for their delegated role in this study. All study personnel have also received the required university training and will be trained by the PI before the study starts. The PIs, CO-investigators and research team have conducted similar studies in this patient population using similar agents and have the expertise to execute the study protocol and manage any adverse events.

Describe other resources available to conduct the research.

14.2 Describe the time and effort that the Principal Investigator and research staff will devote to conducting and completing the research.

NOTE: Examples include the percentage of Full Time Equivalents (FTE), hours per week. The question will elicit whether there are appropriate resources to conduct the research.

Response: The principal investigator supervises the research project and weekly research meetings are conducted to discuss the recruitment rate, resolve and discuss issues related to the conduct, safety, analysis of the study and related publications. PI is expected to spend 15% of his academic time on this research. The co-investigators, study coordinator and research staff provide coverage to the research related activity for 365 days a year.

14.3 Describe the availability of medical or psychological resources that subjects might need as a result of anticipated consequences of the human research, if applicable.

NOTE: One example includes: on-call availability of a counselor or psychologist for a study that screens subjects for depression.

Response: Available medical literature will be provided as deemed appropriate or requested by patient through UB libraries, PubMed, Google scholar as all the investigators have access to medical literature through listed resources above

The patient will also have access to physician (Investigators and Co-Investigators) who will be available to address any adverse effects or other questions during the course of the study who will be available to address any adverse effects or other questions during the course of the study

14.4 Describe your process to ensure that all persons assisting with the research are adequately informed about the protocol, the research procedures, and their duties and functions.

Response: Before beginning of enrollment all research staff will be asked to review the protocol and a training session will be conducted to provide protocol details, explain technical points and answer staff questions. All training session will have a sign in sheet to document such training. Continuous education through training meetings, conferences and discussions will continue through the study period and when revision are made to protocol.

15.0 Other Approvals

15.1 Describe any approvals that will be obtained prior to commencing the research (e.g., school, external site, funding agency, laboratory, radiation safety, or biosafety).

Response: FDA IND filling is required.

☐ **N/A:** This study does not require any other approvals.

16.0 Provisions to Protect the Privacy Interests of Subjects

16.1 Describe how you will protect subjects' privacy interests during the course of this research.

NOTE: Privacy refers to an individual's right to control access to him or herself. Privacy applies to the person. Confidentiality refers to how data collected about individuals for the research will be protected by the researcher from release. Confidentiality applies to the data.

Examples of appropriate responses include: "participant only meets with a study coordinator in a classroom setting where no one can overhear", or "the participant is reminded that they are free to refuse to answer any questions that they do not feel comfortable answering."

Response: Our clinical providers involved in the study will identify potential patients for recruitment from the Diabetes-Endocrinology Center of WNY Clinics according to the inclusions and exclusion criteria and through advertisements. Patient who qualify will be asked in private during their one on one consultation time with the physician if they wish to participate in the research study. If the patient agrees, the research coordinator will contact them privately for a possible telephone screening and ask if time is appropriate for this discussion. The patients who call for potential participation in the study due to advertisement flyers will be screened over the phone with the research coordinator, using our telephone screening form.

When the patient is being seen at our clinics for the first time they sign the "Consent to use and disclosure of protected health information" form which clearly states that their protected health information (PHI) can be used for review in preparation for possible research. Additionally, a partial HIPAA waiver will be obtained to access medical records when screening for patients.

If the patient passes the telephone screening, they will be asked to make an appointment to review and sign the consent. Patient will do this in a private room in the research unit and will be allowed to discuss the consent in detail with the research coordinator and or study doctor. Patient will be notified that it is completely voluntary to participate in the research study and can withdraw at any time.

We will not be accessing any medical information of the patients for whom the services are not provided by our clinic providers before partial HIPAA is obtained or IC is signed.

16.2 Indicate how the research team is permitted to access any sources of information about the subjects.

*NOTE: Examples of appropriate responses include: school permission for review of records, consent of the subject, HIPAA waiver. This question **does apply** to records reviews.*

Response: Consent of the subject and partial HIPAA waiver.

17.0 Data Management and Analysis

17.1 Describe the data analysis plan, including any statistical procedures. This section applies to both quantitative and qualitative analysis.

Response:

Data (e.g. date of diagnosis, past medical history, drug treatment) extracted from local clinical records will be entered on to the eCRF under the study-specific coded identifier according to structured questions as stipulated in the Study Protocol. Biological samples (blood, urine and adipose tissue aspirates) will be collected from the study participants at the study visits according to the Schedule of Assessments in the Study Protocol.

Measures relating to safety and routine care of the participants (e.g., biochemical measures of glycemic and lipidemic control, kidney function/ fluid balance, hepatic function, hematological variables) will be analysed at local clinical laboratories in order that they are available to study nurses and physicians. The confidentiality of these data will be secure, meeting or exceeding the regulations of the relevant Ethical Review Committees/ Institutional Review Boards. Samples for measurement of key study outcomes will also be measured at the central laboratory at the University of Buffalo. Blood, urine and other samples will be aliquoted into cryovials at site for freezing and storage. Electronic stored material (e.g., patient records, data from blood and urine samples, and data from Continuous Glucose Monitoring) will only be stored using the study specific coded identifier. Data will not be identifiable at the participant level without additional access to the code. The code will be available only to the investigators and the personnel performing the study. Access to any data held electronically will require study specific log-in codes. These are managed and logged by the Robertson Centre for Biostatistics, University of Glasgow, UK

Statistical plan. The primary objective of the proposed research is to evaluate the effect of addition of dapagliflozin to semaglutide on a background of insulin treatment in type 1 diabetes patients on HbA1c at six months following start of dapagliflozin. The similarities between the study groups, baseline values for subject's demographics and study endpoints will be compared using appropriate parametric tests. Transformations of the data in order to meet statistical assumptions may be considered. The primary endpoint of the study is to detect a difference in mean HbA1c change after six months of dapagliflozin or placebo addition to semaglutide and insulin treatment. Statistical analysis will be carried out using t-tests to detect difference between groups or the Wilcoxon test for paired data. Tests will be two-sided and tested at the 0.05 level. The results will be summarized as mean±SE. All randomized patients will be included in the analysis of the primary end point based on an Intent-to-treat model.

The secondary endpoints will be to compare changes in HbA1c between each of the triple therapy and dual therapy groups compared to standard of care group, mean weekly glucose concentrations, percent time spent in different glycemic thresholds, variability as measured by standard deviation, insulin dose, body weight, carbohydrate intake, blood pressure, and 24-hour blood pressure and QOL changes among the groups. A reduction in the glucose and glucagon AUC following the meal will constitute another secondary end point in this setting, ANCOVA analysis will be used. Since the secondary end-points are pre-specified and may as well be correlated, multiplicity control for multiple tests across secondary endpoints may not be necessary. However, Holm-Bonferroni correction

for multiplicity testing will be implemented if additional endpoints are tested or if the pre-specified secondary end-points are found to be not correlated.

17.2 *If applicable, provide a power analysis.*

NOTE: This may not apply to certain types of studies, including chart/records reviews, survey studies, or observational studies. This question is asked to elicit whether the investigator has an adequate sample size to achieve the study objectives and justify a conclusion.

Response:

Sample size determination is based in part on our current data showing that in type 1 diabetes, treatment with a GLP-1 agonist, liraglutide (1.2 and 1.8mg doses) for 3 months leads to HbA1c suppression by an average of 0.6% (SD= 0.5%). Dapagliflozin was recently shown to reduce HbA1c by 0.45% in type 1 diabetes patients (13). Additionally, empagliflozin treatment for 8 weeks was shown to reduce HbA1c by 0.4% in type 1 diabetics (20, 21) and our current data showing additional improvement in HbA1c by an additional 0.66% at 12 weeks when dapagliflozin was added to liraglutide and insulin therapy. Conservatively estimating that the combination therapy will suppress HbA1c by 0.5% (SD =<0.53%) compared to semaglutide alone (t-test), a sample size of 3827 (22-23 evaluable patients) in each group will be sufficient to provide adequate power ($\beta = 0.2$) to detect a statistically significant difference ($\alpha = 0.05$) taking in consideration a 15% dropout rate.

17.3 *Describe any procedures that will be used for quality control of collected data.*

Response:

An electronic case report form (e-CRF) will be used to collect study data at each site. The e-CRF will be developed by the study Data Centre at the Robertson Centre for Biostatistics, University of Glasgow. Access to the e-CRF will be restricted, with only authorised site-specific personnel able to make entries or amendments to their patients' data. It is the investigator's responsibility to ensure completion and to review and approve all data captured in the e-CRF.

Data will be validated at the point of entry into the e-CRF and at regular intervals during the study. Data discrepancies will be flagged to the study site coordinator and any data changes will be recorded in order to maintain a complete audit trail (reason for change, date change made, who made change).

The Robertson Centre for Biostatistics at the University of Glasgow will be responsible for collating and cleaning the data for the study. The Robertson Centre will also be responsible for data back-up and security. This center will also manage the electronic reporting of SUSARS on behalf of the sponsor.

To enable evaluations and/or audits from regulatory authorities, the investigators agree to keep records, including the identity of all participating subjects (sufficient information to link records, all original signed informed consent forms serious adverse event forms, source documents, and detailed records of treatment disposition. The records should be retained by the study country coordinators and investigator according to ICH GCP, local regulations, or as specified in the Clinical Study Agreement, whichever is longer.

Data from the study will be stored by the Chief Investigator for a minimum of 10 years. A final report of the study will be provided to the FDA and the MHRA as per requirements.

- No interim analysis is planned for efficacy so termination of study will be related to safety. Significant increases in hypoglycemic or DKA events as reviewed by DSMB might trigger termination or modification of study.
- Last observation carry forward will be used to fill missing data for the primary end point and some secondary end points. Missing data will not be filled for other endpoints.
- Any deviation(s) from the original statistical plan will be described and justified in the protocol and/or in the final report.
- All subjects entered into treatment will analyzed for primary end point on ITT bases. For secondary endpoints, analysis of completed and evaluable patients will performed per-protocol.

18.0 Confidentiality

A. Confidentiality of Study Data

Describe the local procedures for maintenance of confidentiality of ***study data and any records that will be reviewed for data collection****.*

18.1 A. Where and how will all data and records be stored? Include information about: password protection, encryption, physical controls, authorization of access, and separation of identifiers and data, as applicable. Include physical (e.g. paper) ***and*** *electronic files.*

Response: All data records will be stored on protected computers with unique passwords issued for each of the study team members and or in locked cabinets within the research center. These files will only be accessible by authorized study personnel.

The TriNetX/i2b2 data will be protected by only accessing identifiable contact information remotely on IHI’s “virtual machine”, so the data never leaves their secure encrypted server. Recruitment letters will be generated in a mail merge behind the firewall on IHI’s virtual machine. No patient data from TriNetX/i2b2 will be removed from the IHI server or saved in other locations.

18.2 A. How long will the data be stored?

Response: De-identified data storage has no expiration date and will be stored for a minimum of 7 years. The researchers may continue to rely on this for future use in research study.

18.3 A. Who will have access to the data?

Response: Those physicians, nurses, and laboratory staff that are on all documentation for the study will have access to the data.

18.4 A. Who is responsible for receipt or transmission of the data?

Response: Those physicians, nurses, and laboratory staff that are on all documentation for the study will have access to the data and can handle transfer of data. Study data will be transferred electronically (through eCRFs) to Robertson Centre for Biostatistics, University of Glasgow, UK

18.5 A. How will the data be transported?

Response: All data are stored at locally and will be transferred to Robertson Centre for Biostatistics, University of Glasgow, UK for data management and analysis. The transfer will be done through electronic CRFs processed on secure network.

Data will also be transported unless when is being archived. At that point files will be transferred to Iron Mountain for storage and archiving.

B. Confidentiality of Study Specimens

*Describe the local procedures for maintenance of confidentiality of **study specimens**.*

☐ **N/A:** No specimens will be collected or analyzed in this research. *(Skip to Section 19.0)*

18.6 B. Where and how will all specimens be stored? Include information about: physical controls, authorization of access, and labeling of specimens, as applicable.

Response: The specimens will be stored in the laboratory located at *1000 Youngs Road, Suite 105, Williamsville NY 14221*and at the CTRC located in 875 Ellicott St. Buffalo NY14203. Samples will be stored in a locked -80º C freezer. All

specimens collected at the Glasgow site will be stored in NHS -80º C freezers within the eCRF until shipping to Buffalo (World Courier).

18.7 B. How long will the specimens be stored?

Response: Specimens storage has no expiration date and will be stored for a minimum of 7 years. The researchers may continue to rely on this for future use in research study

18.8 B. Who will have access to the specimens?

Response: Those physicians, nurses, and laboratory staff that are on all documentation for the study will have access to the specimens

18.9 B. Who is responsible for receipt or transmission of the specimens?

Response: Those physicians, nurses, and laboratory staff that are on all documentation for the study will have access to the specimens and can handle transfer of samples. Sample/specimens will transported from University of Glasgow site to the university of Buffalo CTRC site (875 Ellicott St, Buffalo, NY 14203) for storage in -80C until analyzed.

18.10 B. How will the specimens be transported?

Response: All study samples will be centrally stored in UB CTRC site. Therefore, samples collected at the UK site will be transported for analysis and final long-term storage. Transport will be done so using dry ice in a properly labeled Styrofoam container by the laboratory technician. Agreement regarding shipping and receiving the specimens form UK site will be part of the subcontract agreement.

19.0 Provisions to Monitor the Data to Ensure the Safety of Subjects

☐ **N/A:** This study is not enrolling subjects, or is limited to records review procedures only. This section does not apply.

NOTE: Minimal risk studies may be required to monitor subject safety if the research procedures include procedures that present unique risks to subjects that require monitoring. Some examples include: exercising to exertion, or instruments that elicit suicidality or substance abuse behavior. In such cases, N/A is not an acceptable response.

19.1 Describe the plan to periodically evaluate the data collected regarding both harms and benefits to determine whether subjects remain safe.

Response:

An independent Data safety and monitoring board (DSMB) will be established for this trial. The board will consist of independent faculty members from UB and other institutions with sufficient knowledge of the field of type 1 diabetes but not involved in the study. Data Management team at Robertson Centre will periodically provide the data on which to base regular reports to the Data and Safety Monitoring Board. The actual reports will then be generated by the unblinded statistician. The DSMB looks only at safety endpoints and not at futility. The DSMB will convene every six months to review safety end points, side-effects experienced by study subjects and any adverse events reported. The site PIs, CO-Is and study coordinator(s) will also continuously monitor the data and safety of subjects enrolled in the study. The site PIs and/or the DSMB may recommend to terminate the study or specific aspects of the study if clear harm is observed with one treatment option compared to standard of care or placebo. Specific safety endpoints that will be reviewed include increases in hypoglycemic and ketosis/DKA events.

19.2 Describe what data are reviewed, including safety data, untoward events, and efficacy data.

Response:

Assessment of Efficacy

1- glycemic indices including HbA1c, fructosamine, post-prandial glucose excursions and glucagon. CGM data will be collected as efficacy parameter of glucose control. Total insulin dose will be also documented and analyzed.
2- body weight
3- blood pressure and use of blood pressure medications
4- QOL questionnaires.

The timing of recording of the above efficacy parameters is described in table 1.

Assessment of Safety

The following safety assessments assays and measurement will be reviewed:
1- Vitals, CBC, CMP and markers kidney and liver function will be collected at screening, during and at end of the study as in table 1.
2- Assessment of hypoglycemia: Glucose data will be reviewed from CGM and daily finger stick and during visits.
3- Assessment of ketosis: Ketones levels will be reviewed from daily urinary ketone diaries or blood ketone meters and from urine and blood at the time of each visit
4- Reports of side effects by patients.
5- Insulin dose titrations and pump downloads at each visit

The methods and timing for assessing, recording, and analyzing safety parameters.

The DSMB will convene every six months to assess the side-effects experienced by study subjects and any adverse events reported. The PIs, CO-Is and study coordinator(s) will also continuously monitor the safety of subjects enrolled in the study during their visits and during the safety phone calls.

Procedures for eliciting reports of and for recording and reporting adverse event and inter-current illnesses.

All adverse events will be reported at the time of the visit or by telephone when it occurs. Medical events that occur between the signing of the Informed Consent and the first intake of study drug will be documented in the medical history. Participants will be asked to report any adverse events in general, non-directed questioning. For each AE volunteered by the subject, the investigator will obtain all the information required to complete the adverse event page of the eCRF, in accordance with the guidelines that accompany it. All adverse events, regardless of seriousness, severity, or presumed relationship to study therapy, will be recorded using medical terminology in the source document. Whenever possible, diagnoses will be given when signs and symptoms are due to a common etiology (e.g., cough, runny nose, sneezing, sore throat, and head congestion should be reported as "upper respiratory infection). Investigators will record on the CRF their opinion concerning the relationship of the adverse event to study therapy. The principal investigators assumes responsibility for appropriate reporting of AEs to the regulatory authorities. The investigator will report these events to the appropriate Independent Ethics Committee/Institutional Review Board (IEC/IRB) in accordance with local regulations. All measures required for AE management will be recorded in the source document and reported according to the State University of New York at Buffalo instructions. Non-serious events/problems that impacted on the rights and welfare of the subjects will be reported as they occur or at the time of knowledge or notification.

Definition of adverse events

The International Conference on Harmonization (ICH) Guideline for Good Clinical Practice (GCP) E6(R1) defines an AE as:

Any untoward medical occurrence in a patient or clinical investigation patient administered a pharmaceutical product and which does not necessarily have a causal relationship with this treatment. An AE can therefore be any unfavorable and unintended sign (including an abnormal laboratory finding), symptom, or disease temporally associated with the use of a medicinal product, whether or not considered related to the medicinal product.

The term AE is used to include both serious and non-serious AEs.

Definition of serious adverse events

A serious adverse event is an AE occurring during any study phase (i.e., screening, run-in, treatment, wash-out, follow-up), at any dose of the study drugs that fulfils one or more of the following criteria:

- Results in death
- Is immediately life-threatening
- Requires in-patient hospitalization or prolongation of existing hospitalization
- Results in persistent or significant disability or incapacity
- Is a congenital abnormality or birth defect in offspring of the patient

- Is an important medical event that may jeopardize the patient or may require medical intervention to prevent one of the outcomes listed above.
- TO THE AUTHOR: The causality of SAEs (their relationship to all study treatment/procedures) will be assessed by the investigator(s) and communicated to AstraZeneca. This is requested to be on the AE cover sheet.

The following variables are recommended to be collected for each AE:

- AE (verbatim)
- The date when the AE started and stopped
- Whether the AE is serious or not
- Action taken with regard to IPs
- Administration of treatment for the AE
- Outcome

In addition, the following variables are recommended to be collected for SAEs:

- Date the AE met criteria for SAE
- Date the Investigator became aware of the SAE
- Seriousness criteria fulfilled
- Date of hospitalization
- Date of discharge
- Probable cause of death
- Date of death
- Whether an autopsy was performed
- Causality assessment in relation to study procedure(s)
- Causality assessment in relation to other medication, as explained in Section 1.4.2.
- Description of the SAE

Reporting of serious adverse events

All SAEs will be reported, whether or not considered causally related to the investigational product, or to the study procedure(s). The reporting period for SAEs is the period immediately following the time that written informed consent is obtained throughout the treatment period and including the designated follow-up period as per the study protocol. The investigator is responsible for informing the Ethics Committee and/or the Regulatory Authority of the SAE as per local requirements.

Note that all serious or unexpected adverse events must be reported to AstraZeneca regardless of the country where the study is conducted.

The investigator must inform the FDA, via a MedWatch form, of any serious or unexpected adverse events that occur in accordance with the reporting obligations of 21 CFR 312.32 , and will concurrently forward all such reports to AstraZeneca. A copy of the MedWatch report must be emailed to AstraZeneca (TCS vendor) at the time the event is reported to the FDA. It is the responsibility of the investigator to compile all necessary information and ensure that the FDA receives a report according to the FDA reporting requirement timelines and to ensure that these reports are also submitted to AstraZeneca at the same time.

A cover page should accompany the MedWatch form indicating the following:

- "Notification from an Investigator Sponsored Study"
- The investigator IND number assigned by the FDA
- The investigator's name and address
- The trial name/title and AstraZeneca ISS reference number (ESR-##-#####)

Investigator must also indicate, either in the SAE report or the cover page, the causality of events in relation to all study medications and if the SAE is related to disease progression.

Send SAE report and accompanying cover page by way of email to AstraZeneca's designated mailbox: AEMailboxClinicalTrialTCS@astrazeneca.com

If a non-serious AE becomes serious, this and other relevant follow-up information must also be provided to AstraZeneca and the FDA.

Serious adverse events that do not require expedited reporting to the FDA still need to be reported to AstraZeneca preferably using the MedDRA coding language for serious adverse events.

The investigators will either provide a copy of the randomization code/ code break information or unblind those SAEs which require expedited reporting

Overdose

An overdose is defined as a patient receiving a dose of IP in excess of that specified in the Investigator's Brochure, unless otherwise specified in this protocol.

Any overdose of a study patient with the investigational product, with or without associated AEs/SAEs, is required to be reported within 24 hours of knowledge of the event to AstraZeneca Patient Safety using the designated Safety e-mailbox (see Section 1.5 or contact information). If the overdose results in an AE, the AE must also be recorded as an AE. Overdose does not automatically make an AE serious, but if the consequences of the overdose are serious, for example death or hospitalization, the event is serious and must be recorded and reported as an SAE (see Section 1.3). Any specific details around overdose for combination or comparator agents will also be provided as applicable. The investigator will use clinical judgment to treat any overdose.

Hepatic function abnormality

Hepatic function abnormality that fulfills the biochemical criteria of a potential Hy's Law case in a study patient, with or without associated clinical manifestations, is recommended to be reported as "hepatic function abnormal" within 24 hours of knowledge of the event to AstraZeneca Patient Safety using the designated Safety e-mailbox (see Section 1.3), unless a definitive underlying diagnosis for the abnormality (e.g., cholelithiasis or bile duct obstruction) that is unrelated to investigational product has been confirmed. The criteria for a potential Hy's Law case is Aspartate Aminotransferase (AST) or Alanine Aminotransferase (ALT) ≥3x Upper Limit of Normal (ULN) together with Total Bilirubin (TBL) ≥2xULN at any point during the study following the start of study medication irrespective of an increase in Alkaline Phosphatase (ALP).

Pregnancy

If a patient becomes pregnant during the course of the study, the IPs should be discontinued immediately.
Pregnancy itself is not regarded as an AE unless there is a suspicion that the IP under study may have interfered with the effectiveness of a contraceptive medication. Congenital abnormalities or birth defects and spontaneous miscarriages should be reported and handled as SAEs. Elective abortions without complications should not be handled as AEs. The outcome of all pregnancies (spontaneous miscarriage, elective termination, ectopic pregnancy, normal birth, or congenital abnormality) should be followed up and documented even if the patient was discontinued from the study.
If any pregnancy occurs in the course of the study, then the investigator should inform the appropriate AstraZeneca representatives within 1 day, i.e., immediately, but no later than 24 hours of when he or she becomes aware of it.
The investigator will ensure that all relevant information is provided to the AstraZeneca Patient Safety data entry site within 1 to 5 calendar days for SAEs and within 30 days for all other pregnancies.
The same timelines apply when outcome information is available.

19.3 Describe any safety endpoints.

Response: These specific Safety endpoints will be collected:

1- Frequency and severity of hypoglycemia: Hypoglycemia will be assessed throughout the study via multiple daily finger stick glucose measurements, CGM data collected 3 times (about 3 weeks each) during the study and from patient reports during visits and phone calls. Hypoglycemic events will be characterized as in the experts consensus published in *Diabetes Care (2017 Dec; 40(12): 1622-1630):*

- Level 1: Glucose < 70 mg/dL (3.9 mmol/L) and Glucose ≥ 54 mg/dL (3.0 mmol/L)
- Level 2: Glucose < 54 mg/dL (3.0 mmol/L)
- Level 3: A severe event characterized by altered mental and/or physical status requiring assistance.

2- Ketones levels: Urinary ketones measured daily (and any blood ketones) at home and logged in the ketones logbook. Blood and urinary ketones measured during each visit. Ketone levels will be characterized as normal, ketosis and severe ketosis:
-Normal blood ketones <0.6mM or urinary ketones of trace to small (≥+1)
-Ketosis: Blood ketones >0.6mM <2.5mM or urinary ketones of moderate to high (+2 and +3)
-Severe ketosis Blood ketones >2.5mM or urinary ketones of too high (+4)
3- Diabetes ketoacidosis episodes: any confirmed DKA episode (regardless of hospitalization) defined as:
• Elevated serum or urine ketones (greater than the upper limit of the normal range), and

• Serum bicarbonate < 15 mmol/L or Blood pH < 7.3

4- Low blood pressure: <90mmHg SBP and <50 mmHg DBP): Patients will be instructed to measure blood pressure daily and it will be will be assessed in each visit.

5- Nausea, vomiting and other GI side effects. Data will be collected by patients' safety reports, during visits and phone calls.

6- Genital mycotic infections or UTIs

7- Changes in liver and kidney function: CMP and kidney and liver profiles will be assessed as indicated in table 1.

19.4 Describe how the safety information will be collected (e.g., with case report forms, at study visits, by telephone calls with participants).

Response: The safety information will be collected at the time of the visit, and or during preplanned telephone calls with the participant or patient self-reporting (including glucose, food intake and ketones diaries). Data will be entered into eCRFs

19.5 Describe the frequency of safety data collection.

Response: Safety data collection will be done at all study visits and during preplanned phone calls as in table 1. The patient, however, will be asked to report any adverse event or safety related information via phone as soon as it occurs and it will be reviewed the same day.

19.6 Describe who will review the safety data.

Response: DSMB established for this study will review unblended safety data provided by the data management system every 6 months. Additionally, the PIs and coinvestigators will review safety data (listed above) at the completion of each visits by each subject and review any report of side effects or adverse events as they are presented by the patients. The investigators will also review collective blinded data every 3 months to assess the safety and any potential risks to the participants. Furthermore the investigators will assess other risks including the physical, psychological, social, legal and economic harm to these patients. The investigators will carefully watch for any invasion of privacy and breach of confidentiality. The principal investigator will be sharing the results of safety analysis of the study and serious adverse events with the local IRB, DSMB, JDRF, AZ and the FDA. The study groups will remain blinded. If there are a serious safety concerns or an emergency, then, and based on principal investigator discretion, study randomization center in Glasgow University or the unblended statistician will provide information about group assignment of the patient(s) involved. Un-blinded data will be reviewed by the principal investigator and co-investigators and decision to complete, withdraw or adjust dose of drug(s) will be made, documented and shared with IRB and DSMB.

19.7 Describe the frequency or periodicity of review of cumulative safety data.

Response: Safety data will be reviewed every 6 months by DSMB (un-blinded), every 3 months by PIs and CO-Is (blinded) and at every visit by study team.

19.8 Describe the statistical tests for analyzing the safety data to determine whether harm is occurring.

Response: N/A

19.9 Describe any conditions that trigger an immediate suspension of the research.

Response: No interim analysis is planned for efficacy so termination of study will be related to safety. Significant increases in severe hypoglycemic or DKA events as reviewed by DSMB are possible causes that might trigger termination of study.

20.0 Withdrawal of Subjects

☐ **N/A:** This study is not enrolling subjects. This section does not apply.

*20.1 Describe **anticipated** circumstances under which subjects may be withdrawn from the research without their consent.*

1. The site PI can stop investigational study treatment or remove participants from the trial without participants approval if for any reason he/she feels is appropriate, including: severe side effect, injury or medical condition which may place the patient at risk of further complications if he/she continue to participate, failure to take the medication as instructed, failure to keep scheduled appointments, cancellation of the study by the sponsor, IRB, FDA or other administrative reasons.
2. If any of the participants become pregnant during the period of study, they will discontinue study treatment.
3. Participants who experience persistent severe nausea or vomiting after starting semaglutide which is not managed by dose reduction will discontinue the investigational study treatment. Those with mild or moderate nausea will continue for at least one more week. If they continue to experience significant nausea (defined as interfering with normal activities), dose will be reduced. If they continue to have these side effects, they will discontinue study medication(s).
4. Persistent weight loss or failure to maintain weight if subject has lost >15% or BMI is < 22 kg/m2 in spite of down-titration of semaglutide and/or dapagliflozin.
5. Developing other conditions included in the exclusion criteria
6. Change in method of insulin delivery between insulin pumps to MDI or vice versa during the study unless pump treatment interruption is temporary for a period of at

most 2 weeks. Also, patients who upgrade to the new "artificial pancreas" pump (Medtronic's MiniMed 670G hybrid) during the study will be withdrawn.

7. Hypoglycemia: Two episodes of severe hypoglycemia (requiring others help).
8. Ketosis (with no other identifiable precipitating factors): Persistent (1week) asymptomatic or symptomatic and unmanaged (using STICH protocol) blood ketone levels >1.5 mM (or ≥+2 in urine).
9. Persistent (>1 week) total insulin dose reduction of >20% from baseline AND total insulin dose <0.4U/Kg.
10. DKA: Two episode of DKA (with no identifiable precipitating factors) as defined by the expert consensus statement (*Diabetes Care (2017 Dec; 40(12): 1622-1630)*)
11. Two episodes of UTI.
12. Two episodes of mycotic genital infection.
13. Significant persistent change in eGFR > 15 ml/min
14. Pancreatitis: One episode of pancreatitis.
15. Other persistent side effects specific to study drugs

20.2 Describe any procedures for orderly termination.

NOTE: Examples may include return of study drug, exit interview with clinician. Include whether additional follow up is recommended for safety reasons for physical or emotional health.

Response: Participation in this study is voluntary. Participants have the right to refuse to participate or to withdraw from participation at any time for any reason. Refusal to participate or to withdraw from the study will involve no penalty or loss of entitled benefits, nor affect ongoing medical care. The site PI can stop investigational study treatment or remove participants from the trial without participants approval. If a patient withdraws consent and decide not to participate in the study, she/he will be asked to undergo a final study visit for his/her safety. If a subject withdraws from the research, the data collected to that point will be used toward the research finding. If applicable the subject will have to bring back any unused research drug and or devices.

20.3 Describe procedures that will be followed when subjects withdraw from the research, including retention of already collected data, and partial withdrawal from procedures with continued data collection, as applicable.

Response: We will use the data collected up to the point of stopping the study. No further information will be collected. Patients who stop study treatment and are interested in continuing in the study will be followed up until the end of the study for the primary end point and select secondary endpoints based on the Intent-To-Treat (ITT) analysis model. Withdrawn participants will not be replaced. The study sample size calculation includes an additional 15% subjects to compensate for drop out.

21.0 Risks to Subjects

21.1 List the reasonably foreseeable risks, discomforts, hazards, or inconveniences to the subjects related to their participation in the research. Consider physical, psychological, social, legal, and economic risks. Include

a description of the probability, magnitude, duration, and reversibility of the risks.

NOTE: Breach of confidentiality is always a risk for identifiable subject data.

Response:

- Risks associated with intravenous (IV) line: All participants will be informed of the potential complications of an intravenous (IV) line, which include mild bruising at the site of IV line, usually resolving in a few days. They will also be informed about the possibility of infiltration of the IV line at the time of performing blood draws or during the meal challenge test in which case another IV line at a different site will be required (with risk of bruising at more than one site).
- Risks associated with 24 hour Ambulatory BP monitoring device: The BP cuff inflates every 15 minutes to record blood pressure over 24-hours. Participants will experience a pressure-like sensation over the arm where 24 hour BP device is worn at the time of the BP being measured due to the inflation of the cuff and release of this pressure at the time of deflation of the BP cuff. They will be informed to remove the cuff after 24 hours. If worn for longer periods they may experience mild bruising over the arm and some swelling, usually resolving 1-2 days after discontinuation.
- Risks associated with fat tissue aspiration: will probably lead to a bruise at the site of aspiration. The site may be mildly painful for 1-2 weeks. Participants are advised to call their study site staff if they have more severe pain or swelling at the site after the procedure. Rarely there are side effects such as low BP or heart rate and/or allergic reaction to local anesthetic including swelling of the throat.
- All subjects will be referred to semaglutide and dapagliflozin labeling safety information, and will be given a copy of the package insert of the medication. Discussion of the potential side effects and the different warning and precautions will take place at the time of informed consent. Patients will be informed they must not discontinue their insulin during the study.
- Potential risk of insulin, GLP-1 RA and SGLT-2 inhibitors use in type 1 diabetes patients is hypoglycemia. The combination therapy can possibly increase the magnitude and frequency of this risk.
- Potential side effects of GLP-1 RA and SGLT-2 inhibitors also include hypotension, nausea and other GI side effects, polydipsia, polyuria, dehydration, ketosis, and reduced glomerular filtration rate (GFR).
- In rodents, semaglutide causes dose-dependent and treatment-duration-dependent thyroid C-cell tumors at clinically relevant exposures. However, it received FDA-approval for the treatment of type 2 diabetes in 2017 following large Phase 3 trials including the cardiovascular safety trial SUSTAIN-6.
- On the basis of the above rodent studies, semaglutide is contraindicated in those with a personal or family history of medullary thyroid carcinoma and in patients with Multiple Endocrine Neoplasia syndrome type 2 (MEN 2). However, following review of human data with GLP-1RAs, FDA does not require or suggest routine monitoring of serum calcitonin/ thyroid ultrasound in people treated with semaglutide.

- Injection-site reactions: Serious injection-site reactions, with or without bumps (nodules), have happened in some people who use GLP-1RAs but are less frequent with semaglutide. Injection-site reactions have rarely required surgical intervention. Participants will be instructed to report any injection site reactions including nodules to study investigators.
- Kidney injury: GLP-1 RA might cause acute kidney injury in patients without underlying renal disease especially in patient who had experienced nausea, vomiting, diarrhea or dehydration. Dapagliflozin also has warnings and precautions against acute kidney injury and impairment in renal function.
- As mentioned above, there is a risk of ketoacidosis, as shown by some studies and by our data, when SGLT2 inhibitors are used in type 1 diabetes. In our own preliminary studies on triple therapy, we observed euglycemic ketoacidosis in participants whose insulin doses had been reduced by > 20% of baseline levels or in patients who are not adequately insulinzed. Thus, this adverse effect is largely attributable to insulinopenia as well as anti-insulin effects of SGLT2 inhibitors at the hepatic level (e.g. increased gluconeogenesis).
- The following other known adverse effects of SGLT-2 inhibitors will be discussed with participants when obtaining informed consent:

1. Risk of dizziness/ symptomatic hypotension: can occur soon after initiation due to intravascular volume contraction.
2. Impaired kidney function: As SGLT-2 inhibitors increase serum creatinine and decrease eGFR, this will be monitored closely.
3. Genital mycotic infections: SGLT-2 inhibitors increase the risk of genital mycotic infections, particularly in women. Those with a prior history are more susceptible. Following topical treatment with anti-fungal medications they rarely re-occur.
4. Bladder Cancer - Across 22 clinical studies, newly diagnosed cases of bladder cancer were reported in 10/6045 patients (0.17%) treated with Dapagliflozin and 1/3512 patient (0.03%) treated with placebo. After excluding patients in whom exposure to study drug was less than one year at the time of diagnosis, there were 4 cases of bladder cancer with dapagliflozin and no cases with placebo i.e. too few cases to determine causality.
5. The dapagliflozin tablets and matching placebo contain lactose, which may cause discomfort in lactose-intolerant individuals.

- Severe allergic reaction (anaphylaxis) to study drugs or placebo ingredients or other interventions may accrue in rare cases.
- Excessive weight loss: Both investigation drugs are known to cause weight loss in the range of 5-10% in type 2 diabetes patients. However, the magnitude of weight loss following the combination of both drugs in type 1 diabetes patients may be significantly more. Excessive weight loss may lead to increased risk of hypoglycemia and ketosis or DKA (especially in the presence of dapagliflozin) and other unforeseen risks. Therefore, weight loss during any phase of the study will be limited to ≤15% baseline body weight or reaching a BMI of ≤22Kg/m^2.
- There might be other unforeseen risks associated with the use of these agents in type 1 diabetes and additional unforeseen risks associated with the combination therapy.

- There is always the risk of breach of confidentiality where patients' identifiable data is compromised.

21.2 Describe procedures performed to lessen the probability or magnitude of risks, including procedures being performed to monitor subjects for safety.

Response:

The following are procedures to lessen the probability and magnitude of risks listed above:

1- Hypoglycemia:
- Patients will be instructed to measure glucose at least 4 times a day (before meals and at bedtime) through-out the study and record all reading.
- Insulin dose adjustments will be done at every visit to avoid hypoglycemia. However, there will be no preemptive insulin dose reduction at start of treatment since all patients will have HbA1c >7.5% and the risk of hypoglycemia will be minimal when semaglutide is added.
- Daily fingerstick glucose, CGM and insulin pump data will be reviewed by the investigators for optimal titration of insulin dose to target glucose levels between 90-180mg/dl.
- In general, insulin dose adjustments should not be >10% from current total insulin dose (or 5% if HbA1c is <7%) or >20% from baseline total insulin dose at week 0 and then at week 26.
- Patients will be trained in CARB counting to avoid inaccurate insulin dose administration.
- Patients will be trained to recognize symptoms of hypoglycemia and how to manage it at home. These symptoms include feeling shaky, being nervous or anxious, sweating, chills and clamminess, confusion, fast heartbeat, feeling lightheaded or dizzy, hunger, nausea and color draining from the skin (pallor) among others. They will be asked to call the research office or the on call clinical services.
- When patients feel symptoms of hypoglycemia they will be asked to measure blood glucose and if <70mg/dl they will be instructed to consume carbohydrate or 4 tablets of glucose (4gm each). If after 15 min hypoglycemia continues, repeat carb intake until sugar levels are above 70 mg/dl. If sugar levels are below 55mg/dl, glucagon injection is permitted as rescue medication for severe hypoglycemia.
- Patients will be instructed to inform people who they are in frequent contact with (for example, friends, family members, and coworkers) on how to give glucagon to treat severe hypoglycemia. If patient have needed glucagon previously, it should be documented during screening visit so more emphasis on ways to prevent severe hypoglycemia in the future can be discussed.
- Patients/family will be instructed not to hesitate to call 911 if someone is unconscious and glucagon is not available or someone does not know how to use it and not to inject insulin (it will lower their blood glucose even more) or provide food or fluids (they can choke). They will be asked to document all details of the incident and report to research staff as soon as possible.
- Study medications dose will be reduced in patients who continue to have more than 2 measurements of glucose levels in the hypoglycemic range (<70mg/dl,

regardless of symptoms) after insulin dose is reduced by the maximum allowed in this study.

2- Ketosis and Ketoacidosis:

- Patients will be instructed to measure ketone bodies in urine samples daily starting at 3 days before week 26 with Ketostix (Bayer) and record it in diary provided. Ketones (blood and urine) will be measured at every visit. Additionally, patients will be provided with a ketone meter and strips to measure blood ketones when needed. If ketone bodies in urine measure +1 or greater (>1.5mM in urine), they will be instructed to measure blood ketones and to repeat both the measurements after 60 min. If consecutive measurements show moderate or higher ketones in urine or blood ketones >0.6mM, the patient will be instructed to call the research center or their doctor directly. They will be monitored closely for insulin dose, carb intake, ketones and blood sugar and dapagliflozin/semaglutide can be temporarily discontinued, if necessary, for 1-2 weeks.
- Patients will be provided training to recognize the signs and symptoms of DKA and to self-test urine ketones on a PRN basis (in addition to once daily) if they develop any symptoms consistent with DKA, including nausea, vomiting, fatigue, confusion, or shortness of breath. This should be reported regardless of blood sugar levels since in type 1 diabetes on SGLT-2 inhibitors, DKA can occur even in normal glucose levels.
- Insulin dose changes should not be greater than 10% (5% if HbA1c is<7%) from current levels and not reduced by more than 20% from baseline levels at 0 weeks and then at 26 weeks.
- Patients requiring an insulin dose reduction > 20% AND their total insulin dose is <0.4U/Kg will be monitored closely for insulin dose, carb intake, ketones and blood sugar and dapagliflozin/semaglutide can be temporarily discontinued, if necessary, for 1-2 weeks. If insulin requirements remain law, patient will be withdrawn from study.
- We shall not increase dose of dapagliflozin in patients who exhibit significant ketonuria (+2 or more) with the lower dose.
- Urinary ketones (and blood ketones if patients have a dual glucose and ketone meter) will be monitored daily and reported. Dapagliflozin will be temporarily (1 week) discontinued and STICH protocol (STop SGLT-2i, give Insulin bolus, Carbohydrate, and Hydration) activated in events of DKA or if based on investigator discretion if blood ketone levels are persistently (1 week) >1.5 mM (+2 in or more in urine). Dapagliflozin may also be temporarily stopped in clinical situations known to predispose to ketoacidosis (e.g. prolonged fasting due to acute illness or surgery). Patients will be instructed to stay hydrated and not reduce their caloric intake (especially carbohydrates) or start new dietary systems (especially ketogenic diets) during the study.
- If a patient develops symptoms of DKA or ketone levels exceed the allowed limit in this study (persistent (1 week) blood ketones >1.5 mM or +2 in or more in urine), STICH protocol (STop SGLT-2i, give Insulin bolus, Carbohydrate, and Hydration) will be activated. They could be managed for up to a week. If

symptoms and ketone levels persist over 1 week, patients will be withdrawn form study. Insulin infusions may be administered by ER or hospital if patients develop DKA. They will be asked to document all details of the incident and report to research staff as soon as possible.

3- Risk of acute kidney injury or renal function impairment: CBC, CMP, including kidney function test and urinary markers will be performed at screening, during and at end of the study (table 1). Additional testing will be performed in patients who suffer from sever nausea or GI symptoms or hypovolemic and during the up titration of investigation drugs. If acute kidney injury occurs dapagliflozin will be discontinued immediately. To lessen the chance of this risk, patients will eGFR<60 ml/min/1.73m^2 will be excluded. Patients will also discontinue study medications if eGFR falls by >15 ml/min in 2 repeated measurements. Also other patients with factors that may predispose patients to acute kidney injury e.g. hypovolemia, chronic renal insufficiency, congestive heart failure will be excluded. Patients on ACEI, ARB, NSAID will be monitored closely. Dapagliflozin will be temporarily discontinued in any setting of reduced oral intake such as acute illness or fasting or fluid losses such as gastrointestinal illnesses, or excessive heat exposure.

4- Injection site reactions, mycotic infections and complications of IV and biopsy collection will also be monitored, Physical examination will be performed at screening 26, and 52 weeks visits. Safety vital assessments will be performed at every visit. Pregnancy will be tested at screening, 0, 4 13, 26, 39, and 52 weeks. Phone calls will be made for safety and compliance at least 7 time during the study. The patients, however, will be instructed to report any adverse event or safety related information via phone as soon as it occurs. All safety data will be reviewed by the PI or sub-investigators within 1-2 days of collection.

5- Epinephrine is the primary treatment for anaphylaxis with no absolute contraindication to its use. Our site and staff are trained and equipped to assist patients with such reactions. Severe conditions that are not satisfactory managed in site will be transferred to the hospital emergency department for further assessment and help.

6- Excessive weight loss: Doses of investigational drugs will be reduced in patients who experience excessive weight loss (≥15% of basal body weight or BMI ≤22Kg/m^2). Patients on semaglutide only (first 26 weeks) will be down-titrated to the lower dose. In patients on semaglutide and dapagliflozin/placebo (weeks 26-52), dose of dapagliflozin/placebo will be down-titrated to the lower dose. If weight loss continues, semaglutide dose will be down-titrated as well. Instructions to maintain a minimum of 50g daily carb intake will be also emphasized in patients with significant weight loss (>10% baseline body weight).

*21.3 If applicable, indicate **which procedures** may have risks to the subjects that are currently unforeseeable.*

Response:

There might be additional adverse effects of dapagliflozin not currently known or those unique to their use in Type 1 diabetes. There is no previous studies on the use of semaglutide in type 1 diabetes; therefore, additional unforeseen risk may be encountered in this study. Additionally, unforeseen risks might be associated with the combination therapy that might involve unknown drug-drug interactions.

21.4 If applicable, indicate which research procedures may have risks to an embryo or fetus should the subject be or become pregnant.

Response: Not applicable, we will not be enrolling pregnant participants or who may become pregnant or with in child baring years without signing the consent stating they will use at least two forms of birth control. If a participant becomes pregnant they will be withdrawn from the study immediately. They will be followed until term and any effects on the newborn will be documented.

21.5 If applicable, describe risks to others who are not subjects.

Response: We do not anticipate any additional risk to others not participating in this study.

22.0 Potential Benefits to Subjects

22.1 Describe the potential benefits that individual subjects may experience by taking part in the research. Include the probability, magnitude, and duration of the potential benefits. Indicate if there is no direct benefit.

*NOTE: Compensation **cannot** be stated as a benefit.*

Response: The investigational drugs used in this study are already approved as antidiabetic agents for treating type 2 diabetes. Both agents have demonstrated significant improvement in HbA1c, weight and blood pressure. It is our expectation that patients with type 1 diabetes will experience similar benefits compared to those who are treated with insulin alone. The magnitude and duration of the benefits and the safety profile may however differ and are the focus of this investigation.

23.0 Compensation for Research-Related Injury

☐ **N/A:** The research procedures for this study do not present risk of research related injury (e.g. survey studies, records review studies). This section does not apply.

*23.1 **If the research procedures carry a risk of research related injury,** describe the available compensation to subjects in the event that such injury should occur.*

Response: Routinely, Buffalo General Hospital, Erie County Medical Center, and/or the University at Buffalo, State University of New York, its agents, or its employees do not compensate for or provide free medical care for human

subjects/participants in the event that any injury results from participation in a human research project. In the unlikely event that they become ill or injured as a direct result of participating in this study, they may receive medical care. Your study doctor will explain the treatment options to you and tell you where you can get treatment. Generally, this care will be billed to you, your insurance or other third party. Additionally, AstraZeneca shall reimburse the institution for the direct, reasonable, and necessary medical expenses incurred by the institution for the treatment of any personal injury that is a direct result of adverse drug experiences to subjects resulting directly from any manufacturing defects in the study drug to which the subjects would not have been exposed but for their participation in the study if (i) the institutional parties have complied with this agreement, the protocol and any written instructions of AstraZeneca concerning the study drug and (ii) all the requirements of informed consent have been complied with. AstraZeneca will not provide compensation for lost wages or for any other damages, expenses or losses, or for medical expenses have been covered by a subject's medical or other insurance.

23.2 Provide a copy of contract language, if any, relevant to compensation for research related injury.

NOTE: If the contract is not yet approved at the time of this submission, submit the current version here. If the contract is later approved with ***different language regarding research related injury****, you must modify your response here and submit an amendment to the IRB for review and approval.*

Response: AstraZeneca shall reimburse the institution for the direct, reasonable, and necessary medical expenses incurred by the institution for the treatment of any personal injury that is a direct result of adverse drug experiences to subjects resulting directly from any manufacturing defects in the study drug to which the subjects would not have been exposed but for their participation in the study if (i) the institutional parties have complied with this agreement, the protocol and any written instructions of AstraZeneca concerning the study drug and (ii) all the requirements of informed consent have been complied with. AstraZeneca will not provide compensation for lost wages or for any other damages, expenses or losses, or for medical expenses have have been covered by a subject's medical or other insurance.

24.0 Economic Burden to Subjects

24.1 Describe any costs that subjects may be responsible for because of participation in the research.

NOTE: Some examples include transportation or parking.

Response: All research expenses will be covered by the study. The background use of insulin is considered as part of standard of care is the responsibility of the patients and his/her insurance. Participants will not be subjected to any other out of pocket cost.

☐ **N/A:** This study is not enrolling subjects, or is limited to records review procedures only. This section does not apply.

25.0 Compensation for Participation

25.1 Describe the amount and timing of any compensation to subjects, including monetary, course credit, or gift card compensation.

Response:
Visit schedule:

- Screen: $0
- Visit 1 (Week -3): Insulin adjustment: $10
- Visit 2 (Week -1): Meal ($75), insulin adjustment, and 24hr blood pressure monitor ($50): $125*
- Visit 3 (Week 0): 24 hr urine ($15), blood draw ($25), fat biopsy ($75), insulin adjustment, questionnaire ($10): $125
- Visit 4 (Week 1): Insulin adjustment: $10
- Visit 5 (Week 2): Insulin adjustment: $10
- Visit 6 (Week 4): Insulin adjustment: $10
- Visit 7 (Week 13): 24hr urine ($15), blood draw ($25), insulin adjustment, 24hr bp monitor ($50): $90*
- Visit 8 (Week 23): Insulin adjustment: $10
- Visit 9 (Week 25): Insulin adjustment, 24bp monitor ($50), Meal ($75): $125*
- Visit 10 (Week 26): Insulin adjustment, 24hr urine ($15), blood draw ($25), fat biopsy ($75), questionnaire ($10): $125*
- Visit 11 (Week 27): Insulin adjustment: $10
- Visit 12 (Weak 31): Insulin adjustment, blood draw: $25
- Visit 13 (Week 39): Insulin adjustment, 24hr urine ($15), blood draw ($25), 24hr bp monitor ($50): $90*
- Visit 14 (Week 49): Insulin adjustment: $10
- Visit 15 (Week 51): Insulin adjustment, 24bp monitor ($50), meal ($75): $125*
- Visit 16 (Week 52): Insulin adjustment, 24hr urine ($15), blood draw ($25), questionnaire ($10), fat biopsy ($75):$125*
  *Total compensation for all completed visits: up to $1,025.00. For those not wishing to participate in the optional blood pressure monitoring and/or fat biopsy procedure, the compensation will decrease by $250 for not completing the bp monitoring, and by yet another $225.00 for not completing the fat biopsies.

☐ **N/A:** This study is not enrolling subjects, or is limited to records review procedures only. This section does not apply.

☐ **N/A:** There is no compensation for participation. This section does not apply.

26.0 Consent Process

26.1 *Indicate whether you will be obtaining consent.*

NOTE: This does not refer to consent documentation, but rather whether you will be obtaining permission from subjects to participate in a research study. Consent documentation is addressed in Section 27.0.

☒ ***Yes*** *(If yes, Provide responses to each question in this Section)*

☐ ***No*** *(If no, Skip to Section 27.0)*

26.2 *Describe where the consent process will take place. Include steps to maximize subjects' privacy.*

Response: All participants will come to the research department to be consented. Participants will be placed in a private, screened off area and or room where they can review the consent. Participant questions and or concerns will be address with a member of the study team or research doctor if applicable. The research coordinator will discuss in length the participants requests for privacy of their PHI.

26.3 *Describe how you will ensure that subjects are provided with a sufficient period of time to consider taking part in the research study.*

NOTE: It is always a requirement that a prospective subject is given sufficient time to have their questions answered and consider their participation. See "SOP: Informed Consent Process for Research (HRP-090)" Sections 5.5 and 5.6.

Response: participants will be made aware that participating in research is completely voluntary, and they may withdraw at any time with no consequence to their routine clinic care. If the patients requires time to decide and or discuss partaking in a research study, the subject will be given said time.

26.4 *Describe any process to ensure ongoing consent, defined as a subject's willingness to continue participation for the duration of the research study.*

Response: The research coordinator and study team are available to answer any question or concerns with the patient during the duration of the research trial. At each study visit, the patient is asked a series of questions to ensure they are on task with the study visits and feel comfortable. Upon departing from their study visit, the patients are told of their next visit and given detail instruction for their next visit. If study is revised or amendment or new information becomes available about drug safety that may affect patients participation, the patient may be re-consented to ensure patient ongoing consent

26.5 *Indicate whether you will be following "SOP: Informed Consent Process for Research (HRP-090)." If not, or if there are any exceptions or additional details to what is covered in the SOP, describe:*

- *The role of the individuals listed in the application who are involved in the consent process*
- *The time that will be devoted to the consent discussion*
- *Steps that will be taken to minimize the possibility of coercion or undue influence*
- *Steps that will be taken to ensure the subjects' understanding*

Response:

☒ We have reviewed and will be following "SOP: Informed Consent Process for Research (HRP-090)."

Non-English Speaking Subjects

☒ **N/A:** This study will not enroll Non-English speaking subjects. *(Skip to Section 26.8)*

26.6 Indicate which language(s) other than English are likely to be spoken/understood by your prospective study population or their legally authorized representatives.

NOTE: The response to this Section should correspond with your response to Section 6.4 of this protocol.

Response:

26.7 If subjects who do not speak English will be enrolled, describe the process to ensure that the oral and written information provided to those subjects will be in that language. Indicate the language that will be used by those obtaining consent.

NOTE: Guidance is provided on "SOP: Informed Consent Process for Research (HRP-090)."

Response:

Cognitively Impaired Adults

☒ **N/A**: This study will not enroll cognitively impaired adults. *(Skip to Section 26.9)*

26.8 Describe the process to determine whether an individual is capable of consent.

Response:

Adults Unable to Consent

☒ **N/A**: This study will not enroll adults unable to consent.
(*Skip to Section 26.13)*

When a person is not capable of consent due to cognitive impairment, a legally authorized representative should be used to provide consent (Sections 26.9 and 26.10) ***and, where possible, assent of the individual should also be solicited*** *(Sections 26.11 and 26.12).*

26.9 Describe how you will identify a Legally Authorized Representative (LAR). Indicate that you have reviewed the "SOP: Legally Authorized Representatives, Children, and Guardians (HRP-013)" for research in New York State.

NOTE: Examples of acceptable response includes: verifying the electronic medical record to determine if an LAR is recorded.

Response:

☐

26.10 ***For research conducted outside of New York State****, provide information that describes which individuals are authorized under applicable law to consent on behalf of a prospective subject to their participation in the research. One method of obtaining this information is to have a legal counsel or authority review your protocol along with the definition of "legally authorized representative" in "SOP: Legally Authorized Representatives, Children, and Guardians (HRP-013)."*

Response:

26.11 Describe the process for ***assent of the adults****:*

- *Indicate whether assent will be obtained from all, some, or none of the subjects. If some, indicate which adults will be required to assent and which will not.*

  Response:

- *If assent will not be obtained from some or all subjects, provide an explanation of why not.*

  Response:

26.12 Describe whether ***assent of the adult*** *subjects will be documented and the process to document assent.*

NOTE: The IRB allows the person obtaining assent to document assent on the consent document using the "Template Consent Document (HRP-502)" Signature Block for Assent of Adults who are Legally Unable to Consent.

Response:

Subjects who are not yet Adults (Infants, Children, and Teenagers)

☒ **N/A**: This study will not enroll subjects who are not yet adults.
(Skip to Section 27.0)

26.13 Describe the criteria that will be used to determine ***whether a prospective subject has not attained the legal age for consent to treatments or procedures involved in the research*** *under the applicable law of the jurisdiction in which the research will be conducted* ***(e.g., individuals under the age of 18 years)****. For research conducted in NYS, review "SOP: Legally Authorized Representatives, Children, and Guardians (HRP-013)" to be aware of which individuals in the state meet the definition of "children."*

NOTE: Examples of acceptable responses include: verification via electronic medical record, driver's license or state-issued ID, screening questionnaire.

Response:

26.14 ***For research conducted outside of New York State****, provide information that describes which persons have not attained the legal age for consent to treatments or procedures involved the research, under the applicable law of the jurisdiction in which research will be conducted. One method of obtaining this information is to have a legal counsel or authority review your protocol along the definition of "children" in "SOP: Legally Authorized Representatives, Children, and Guardians (HRP-013)."*

Response:

26.15 Describe whether parental permission will be obtained from:

Response: N/A

☐ One parent even if the other parent is alive, known, competent, reasonably available, and shares legal responsibility for the care and custody of the child.

☐ Both parents unless one parent is deceased, unknown, incompetent, or not reasonably available, or when only one parent has legal responsibility for the care and custody of the child.

☐ Parent permission will not be obtained. A waiver of parent permission is being requested.

NOTE: The requirement for parent permission is a protocol-specific determination made by the IRB based on the risk level of the research. For guidance, review the "CHECKLIST: Children (HRP-416)."

*26.16 Describe whether permission will be obtained from individuals **other than parents**, and if so, who will be allowed to provide permission. Describe your procedure for determining an individual's authority to consent to the child's general medical care.*

Response:

*26.17 Indicate whether assent will be obtained from all, some, or none of the **children**. If assent will be obtained from some children, indicate which children will be required to assent.*

Response:

26.18 When assent of children is obtained, describe how it will be documented.

Response:

27.0 Waiver or Alteration of Consent Process

Consent will not be obtained, required information will not be disclosed, or the research involves deception.

☒ **N/A:** A waiver or alteration of consent is not being requested.

27.1 If the research involves a waiver or alteration of the consent process, please review the "CHECKLIST: Waiver or Alteration of Consent Process (HRP-410)" to ensure that you have provided sufficient information for the IRB to make the determination that a waiver or alteration can be granted.

NOTE: For records review studies, the first set of criteria on the "CHECKLIST: Waiver or Alteration of Consent Process (HRP-410)" applies.

Response:

27.2 If the research involves a waiver of the consent process for planned emergency research, please review the "CHECKLIST: Waiver of Consent for Emergency Research (HRP-419)" to ensure you have provided sufficient information for the IRB to make these determinations. Provide any additional information necessary here:

Response:

28.0 Process to Document Consent

☐ **N/A:** A Waiver of Consent is being requested.
(Skip to Section 29.0)

28.1 Indicate whether you will be following "SOP: Written Documentation of Consent (HRP-091)." If not or if there are any exceptions, describe whether and how consent of the subject will be obtained including whether or not it will be documented in writing.

NOTE: If your research presents no more than minimal risk of harm to subjects and involves no procedures for which written documentation of consent is normally required outside of the research context, the IRB will generally waive the requirement to obtain written documentation of consent. This is sometimes referred to as 'verbal consent.' Review "CHECKLIST: Waiver of Written Documentation of Consent (HRP-411)" to ensure that you have provided sufficient information.

If you will document consent in writing, attach a consent document with your submission. You may use "TEMPLATE CONSENT DOCUMENT (HRP-502)". If you will obtain consent, but not document consent in writing, attach the script of the information to be provided orally or in writing (i.e. consent script or Information Sheet).

Response:

☒ We will be following "SOP: Written Documentation of Consent" (HRP-091).

29.0 Multi-Site Research (Multisite/Multicenter Only)

☐ **N/A:** This study is not an investigator-initiated multi-site study. This section does not apply.

29.1 If this is a multi-site study ***where you are the lead investigator****, describe the processes to ensure communication among sites, such as:*

- *All sites have the most current version of the IRB documents, including the protocol, consent document, and HIPAA authorization.*
- *All required approvals have been obtained at each site (including approval by the site's IRB of record).*
- *All modifications have been communicated to sites, and approved (including approval by the site's IRB of record) before the modification is implemented.*
- *All engaged participating sites will safeguard data as required by local information security policies.*
- *All local site investigators conduct the study appropriately.*
- *All non-compliance with the study protocol or applicable requirements will be reported in accordance with local policy.*

Response:

29.2 Describe the method for communicating to engaged participating sites:

- *Problems*
- *Interim results*
- *Study closure*

Response:

29.3 Indicate the total number of subjects that will be enrolled or records that will be reviewed across all sites.

Response: 81

29.4 If this is a multicenter study for which UB will serve as the IRB of record, and subjects will be recruited by methods not under the control of the local site (e.g., call centers, national advertisements) describe those methods.

Response: N/A

30.0 Banking Data or Specimens for Future Use

☐ **N/A:** This study is not banking data or specimens for future use or research outside the scope of the present protocol. This section does not apply.

30.1 If data or specimens will be banked (stored) for ***future use, that is, use or research outside of the scope of the present protocol****, describe where the data/specimens will be stored, how long they will be stored, how the data/specimens will be accessed, and who will have access to the data/specimens.*

NOTE: Your response here must be consistent with your response at the "What happens if I say yes, I want to be in this research?" Section of the Template Consent Document (HRP-502).

Response: The study data/specimens will be stored in a locked closet or -80 freezer at the research facility of the Diabetes and Endocrinology Center of WNY for at least 7 years.

The research staff (study personnel including coordinator) only will be authorized to access data and or specimens

30.2 List the data to be stored or associated with each specimen.

Response: Patient ID number, study visit information and date of collection will be stored with specimen. Other data stored will include record files of

all patients participating in the study, including data collection sheets, lab results, CGM and insulin pump data.

30.3 Describe the procedures to release banked data or specimens for future uses, including: the process to request a release, approvals required for release, who can obtain data or specimens, and the data to be provided with specimens.

Response: The copy of the individual patient data collected during the study period will be provided to these individual patients who can choose to hand carry it to their respective physicians and a copy will be faxed to their respective clinical providers upon verbal request from the patient. The data provided will include the insulin pump or CGM data or any of the lab results obtained during the study period. The results of the completed study will be made available to the patients if requested through published manuscript.

31.0 Drugs or Devices

☐ **N/A:** This study does not involve drugs or devices. This section does not apply.

31.1 If the research involves drugs or devices, list and describe all drugs and devices used in the research, the purpose of their use, and their regulatory approval status.

Response: **Drugs used:**

Description or Identity of investigational product(s)

Investigational product	***Dosage form and strength***	***Manufacturer***	***Description***
Dapagliflozin (Farxiga) 5 mg	*Green, plain, diamond shaped, film coated 5 mg tablet*	*AstraZeneca*	*sodium-glucose cotransporter 2 (SGLT2) inhibitor indicated as an adjunct to diet and exercise to improve glycemic control*
Matching placebo for dapagliflozin 5 mg	*Green, plain, diamond shaped, film coated tablet*	*AstraZeneca*	
Semaglutide (Ozempic) 0.25 mg, 0.5 mg, 1.0 mg	*clear, colorless solution that contains 2 mg of semaglutide in a 1.5 mL (1.34 mg/mL) pre-filled, disposable, single-patient-use pen injector*	*Novo Nordisk*	*glucagon-like peptide 1 (GLP-1) receptor agonist indicated as an adjunct to diet and exercise to improve glycemic control*

- *Dapagliflozin and its matching placebo tablets will be supplied in bottles and are provided by Astra Zeneca.*
- *Semaglutide will be supplied as prefilled injectable pens and will be purchased commercially through the University at Buffalo Research Pharmacy.*

Subjects will receive drugs in their unmodified form. Dapagliflozin is a once daily tablet, while Semaglutide is a weekly subcutaneous injection.

Insulin pumps (CSII) and long and short acting insulin is the only diabetes medication accepted before and during this trial. No other diabetes drug is permitted.
Blood pressure or lipid lowering drugs are permitted if on stable dose for at least three months prior to start of trial. Glucagon injection is permitted as rescue medication for severe hypoglycemia. Insulin infusions may be administered by ER or hospital if patients develop DKA.

31.2 Describe your plans to store, handle, and administer those drugs or devices so that they will be used only on subjects and be used only by authorized investigators.

Response: Dapagliflozin 5mg tablets and matching placebo provided by Astra Zeneca will be stored in a locked cabinet in research pharmacy. Semaglutide provided by University at Buffalo research pharmacy will be stored in temperature controlled refrigerator at 4C at the research facility of the Diabetes and Endocrinology Center of WNY at 1000 Youngs Rd until dispensed to patients. Investigational drugs will be dispensed by PI or CO-Is only to study patients according to randomization plan.

If the drug is investigational (has an IND) or the device has an IDE or a claim of abbreviated IDE (non-significant risk device), include the following information:

31.3 Identify the holder of the IND/IDE/Abbreviated IDE.

Response: IND application for use of all drugs (dapagliflozin and semaglutide) in type 1 diabetes will be submitted to FDA and no study procedure will start before all local and federal approvals are obtained. FDA has granted IND for usage of dapagliflozin in type 1 diabetes. We will seek FDA approval for the use of semaglutide in this study. These IND letters will be submitted to IRB and JDRF along with final version of protocol before initiating any study activities.

31.4 Explain procedures followed to comply with FDA sponsor requirements for the following:

	Applicable to:		
FDA Regulation	***IND Studies***	***IDE studies***	***Abbreviated IDE studies***
21 CFR 11	***X***	***X***	
21 CFR 54	***X***	***X***	
21 CFR 210	***X***		
21 CFR 211	***X***		
21 CFR 312	***X***		
21 CFR 812		***X***	***X***
21 CFR 820		***X***	

Response: All FDA sponsor requirements have been reviewed and will be followed during the study procedures

32.0 Humanitarian Use Devices

☒ **N/A:** This study does not involve humanitarian use devices. This does not apply.

32.1 For Humanitarian Use Device (HUD) uses provide a description of the device, a summary of how you propose to use the device, including a description of any screening procedures, the HUD procedure, and any patient follow-up visits, tests or procedures.

Response:

32.2 For HUD uses provide a description of how the patient will be informed of the potential risks and benefits of the HUD and any procedures associated with its use.

Response:

University at Buffalo Institutional Review Board (UBIRB)
Office of Research Compliance | Clinical and Translational Research Center Room 5018
875 Ellicott St. | Buffalo, NY 14203
UB Federalwide Assurance ID#: FWA00008824

Complete Research Protocol (HRP-503)

Table of Contents

Template Instructions 2
1.0 Objectives 4
2.0 Scientific Endpoints 5
3.0 Background 6
4.0 Study Design 12
5.0 Local Number of Subjects 13
6.0 Inclusion and Exclusion Criteria 13
7.0 Vulnerable Populations 16
8.0 Eligibility Screening 17
9.0 Recruitment Methods 18
10.0 Procedures Involved 20
11.0 Study Timelines 33
12.0 Setting 34
13.0 Community-Based Participatory Research 35
14.0 Resources and Qualifications 35
15.0 Other Approvals 36
16.0 Provisions to Protect the Privacy Interests of Subjects 37
17.0 Data Management and Analysis 38
18.0 Confidentiality 40
A. Confidentiality of Study Data 40
B. Confidentiality of Study Specimens 41
19.0 Provisions to Monitor the Data to Ensure the Safety of Subjects 42
20.0 Withdrawal of Subjects 49
21.0 Risks to Subjects 51
22.0 Potential Benefits to Subjects 56
23.0 Compensation for Research-Related Injury 57
24.0 Economic Burden to Subjects 58
25.0 Compensation for Participation 58
26.0 Consent Process 59
27.0 Waiver or Alteration of Consent Process 63
28.0 Process to Document Consent 64
29.0 Multi-Site Research (Multisite/Multicenter Only) 64
30.0 Banking Data or Specimens for Future Use 65
31.0 Drugs or Devices 66
32.0 Humanitarian Use Devices 68

Template Instructions

Sections that do not apply:

- *In several sections, the addition of checkboxes for **Not Applicable** have been added to the template as responses.*
    - *If an N/A checkbox is present, select the appropriate justification from the list.*
    - *If an N/A checkbox is not present, or if none of the existing checkboxes apply to your study, you must write in your own justification.*
- *In addition:*
    - *For research where the only study procedures are records/chart review: Sections 19, 20, 22, 23, 24, 25, 31, and 32 do not apply.*
    - *For exempt research: Sections 31 and 32 do not apply.*

Studies with multiple participant groups:

- *If this study involves multiple participant groups (e.g. parents and children), provide information in applicable sections for each participant group. Clearly label responses when they differ. For example:*

  Response:

  Intervention Group:

  Control Group:

Formatting:

- *Do not remove template instructions or section headings when they do not apply to your study.*

If you are pasting information from other documents using the "Merge Formatting" Paste option will maintain the formatting of the response boxes.

Amendments:

- *When making modifications or revisions to this and other documents, use the **Track Changes** function in Microsoft Word.*
- *Update the version date or number **on Page 3.***

PROTOCOL TITLE:

Include the full protocol title.

Response: **Triple Therapy for Type 1 Diabetes with Insulin, Semaglutide, and Dapagliflozin**

PRINCIPAL INVESTIGATOR:

Response:

**Principal Investigator:**	**Paresh Dandona, MBBS, PhD**
**Co- Investigators:**	**Husam Ghanim, PhD**
	**Antoine Makdissi, MD**
	**Manav Batra, MD**
**Co- Principal Investigator:**	**John R Petrie, MBChB, PhD**

Paresh Dandona, M.B.B.S., Ph.D., F.R.C.P, F.A.C.P, F.A.C.C, FACE,
SUNY Distinguished Professor,
Director, Diabetes-Endocrinology Center of Western NY
Chief of Endocrinology, Diabetes, Endocrinology and Metabolism, SUNY at Buffalo
1000 Youngs Road, Suite 105
Williamsville NY 14221
Telephone: (716) 535-1850
Fax: 716-639-1200
E-mail: pdandona@kaleidahealth.org

CO-PI:
Professor John R Petrie
BSc MBChB PhD FRCP(Ed) FRCPSG
Professor of Diabetic Medicine
Institute of Cardiovascular and Medical Sciences
BHF Glasgow Cardiovascular Research Centre
University of Glasgow
126 University Place
Glasgow G12 8TA
Tel: +44(0)141 330 3325
Email: john.petrie@glasgow.ac.uk
Web: http://www.gla.ac.uk/researchinstitutes/icams/staff/johnpetrie/
The University of Glasgow

VERSION:

Include the version date or number.

Response: 2/09/2026, V8

GRANT APPLICABILITY:

Indicate whether this protocol is funded by a grant (e.g. NIH, foundation grant). For a grant with multiple aims, indicate which aims are covered by this research proposal.

NOTE: This question does not apply to studies funded by a sponsor contract.

Include a copy of the grant proposal with your submission.

Response: Research support provided by JDRF and AZ

RESEARCH REPOSITORY:

Indicate where the research files will be kept, including when the study has been closed. The repository should include, at minimum, copies of IRB correspondence (approval, determination letters) as well as signed consent documents. This documentation should be maintained for 3 years after the study has been closed.

Response:
Site 1: Diabetes Endocrinology Research Center of WNY
Address: 1000 Youngs Road, Suite 105, Williamsville NY 14221
Department: Diabetes Endocrinology

Site 2: University of Glasgow. Institute of Cardiovascular and Medical Sciences University of Glasgow 126 University Place Glasgow, G12 8TA

1.0 Objectives

1.1 Describe the purpose, specific aims, or objectives of this research.

Response:

Primary Objective: To assess whether the addition of dapagliflozin to semaglutide and insulin (triple therapy) improves glycemic control in patients with type 1 diabetes compared with semaglutide and insulin (dual therapy) and insulin only (standard) treatment.

General hypothesis: The addition of dapagliflozin to semaglutide and insulin treatment improves glycemic control, weight loss and blood pressure in adults with type 1 diabetes.

1.2 State the hypotheses to be tested, if applicable.

NOTE: A hypothesis is a specific, testable prediction about what you expect to happen in your study that corresponds with your above listed objectives.

Response:

Specific hypotheses:

Primary: combined dapagliflozin and semaglutide treatment in addition to insulin will improve HbA1c levels compared with semaglutide in addition to insulin .

Secondary 1: semaglutide in addition to insulin treatment over 26 weeks will improve HbA1c levels compared with insulin therapy alone.

Secondary 2: combined dapagliflozin and semaglutide treatment in addition to insulin will improve HbA1c levels compared to insulin therapy alone at 52 weeks.

Secondary 3: combined dapagliflozin and semaglutide treatment in addition to insulin will improve CGM-based weekly mean and fasting blood glucose, glycemic oscillations and percent of time in range (70-180mg/dl) with lower insulin requirements compared with semaglutide and insulin (dual therapy) or insulin only (standard) treatment.

Secondary : combined dapagliflozin and semaglutide in addition to insulin therapy will reduce systolic and diastolic blood pressure compared with semaglutide and insulin (dual therapy) or insulin only (standard) treatment.

Secondary 5: combined dapagliflozin and semaglutide in addition to insulin therapy (triple therapy) will result in weight loss compared to dual and standard therapy.

Secondary 6: combined dapagliflozin and semaglutide in addition to insulin therapy will result in improved scores on the Diabetes Specific Quality of Life Scale (DSQOLS) and the Problem Areas In Diabetes (PAID) survey compared with dual and standard therapy.

Tertiary and Exploratory Objectives (EO) for future ancillary studies (PLEASE NOTE: For EO, samples will be collected to facilitate the measurements of these end points in future ancillary studies once submitted and approved by JDRF so cost of assays is not included in the current budget): The addition of dapagliflozin to semaglutide and insulin in type 1 diabetes will:

Tertiary 1: combined dapagliflozin and semaglutide treatment in addition to insulin therapy will improve fasting and postprandial glucose excursions and suppress postprandial glucagon, FFA, GLP-1 compared with semaglutide and insulin or insulin therapy alone.

Tertiary 2: semaglutide in addition to insulin treatment over 52 weeks will improve HbA1c levels compared with insulin therapy alone.

EO 1: increase plasma concentrations of vasodilatation mediators and reduce plasma concentrations of vasoconstriction mediators compared to dual and standard therapy groups.

EO 2: exert a systemic anti-inflammatory effect including reducing mRNA expression of inflammatory mediators and NFκB binding in peripheral mononuclear cells (MNC) and adipose tissue (optional) and their circulating levels in plasma while increasing levels of IL-RA and adiponectin.

2.0 Scientific Endpoints

2.1 Describe the scientific endpoint(s), the main result or occurrence under study.

NOTE: Scientific endpoints are outcomes defined before the study begins to determine whether the objectives of the study have been met and to draw conclusions from the data. Include primary and secondary endpoints. Some example endpoints are:

*reduction of symptoms, improvement in quality of life, or survival. Your response should **not** be a date.*

Response:

Primary endpoint: Change in HbA1c at 6 months following dapagliflozin or placebo therapy in addition to combined semaglutide and insulin treatment.

Efficacy Secondary endpoints:

1. Change in HbA1c from baseline at 12 months in triple therapy group compared to insulin only group.
2. Change in HbA1c from baseline at 6 months in dual therapy group compared to insulin only group.
3. Change in percent Time in Range (70– 180 mg/dl), percent time in hyperglycemia Level 1(180-250mg/dl) and Level 2 (>250mg/dl) and percent time in hypoglycemia Level 1 (54-70mg/dl) as assessed by CGM
4. Other indices of glycemic control: weekly mean and fasting glucose, standard deviation, insulin requirements.
5. Change in body weight and quality of life as assessed by DSQOLS and PAID at 6 and 12 months.
6. Anti-hypertensive effects including change in systolic and diastolic BP, numbers of BP medications required.

Safety Secondary endpoints:

1- Differences in rates of hypoglycemic events Level 2 (<54mg/dl) and Level 3 (A severe event characterized by altered mental and/or physical status requiring assistance) between investigative arms and standard therapy arm.

2- *Differences in rates of diabetic ketoacidosis defined as elevated serum or urine ketones (greater than the upper limit of the normal range), and serum bicarbonate <15 mmol/L or Blood pH <7.3 between investigative arms and standard therapy arm.*

Tertiary/ exploratory endpoints: (items 2-4 to be performed in future on collected samples from this study if additional funding is approved by JDRF):

1- Fasting and postprandial (Area Under curve) glucose excursions, FFA, GLP-1 and glucagon.

2- Indices of inflammatory stress. These will be CRP, FFA, SAA, IL-1β, IL-18, IL-1RA, adiponectin and TNFα concentrations in plasma and isoprostane in urine.

3- Expression of IL-1β, TNFα, IL-12, TLR2, TLR4, adiponectin, IκBα as well as NFκB binding in peripheral MNC and adipose tissue (optional).

4- Indices of vasodilatation and vasoconstriction: ANP, b-NP, cGMP, cAMP, angiotensinogen, renin and angiotensin II concentrations.

3.0 Background

3.1 Provide the scientific or scholarly background, rationale, and significance of the research based on the existing literature and how it will contribute to existing knowledge. Describe any gaps in current knowledge. Include relevant preliminary findings or prior research by the investigator.

Response:

The control of glucose homeostasis in type 1 diabetes is fragile since β-cell reserve is negligible. Traditionally, insulin has been the only treatment approach available. This approach, often leads to erratic blood sugar control given the challenge of matching exogenous insulin to mealtime glucose excursions in the face of a lack of any endogenous insulin to compensate for variation in requirements in response to meals and activity levels. Injected insulin boluses rarely match precise insulin requirements either in terms of dose administered or bio-availability. The result often is wide fluctuations in blood glucose readings. Based on our work to date and other published reports, previous attempts to add other glucose-lowering medications to insulin therapy yielded minimal benefits on glycemia (although metformin may have other benefits (1-3)

We have recently shown in two preliminary retrospective studies that the addition of liraglutide, a Glucagon Like Peptide -1 receptor agonist (GLP-1RA), to insulin results in consistent improvements in glycemic control in type 1 diabetes as demonstrated by reductions in HbA1c, glycemic excursions, and insulin requirements accompanied by improvements in body weight and systolic blood pressure (4, 5). We have now also completed the first prospective randomized controlled trial of this drug in 72 people with type 1 diabetes. Our data not only confirmed improvements in glycemic control, HbA1c, body weight and systolic BP, but also demonstrated a reduction in appetite, carbohydrate intake in low grade inflammation as measured by plasma CRP concentrations (6). Importantly, quality of life was also improved. Our recent studies in type 2 diabetes have revealed that systolic blood pressure reduction with GLP-1RA (exenatide) treatment in type 2 diabetes is associated with increased plasma concentrations of ANP, cGMP and cAMP along with reduced concentrations of angiotensinogen, renin and angiotensin II (7). It is possible that these mechanisms play a role in the prevention of cardiovascular complications in type 1 diabetes.

While GLP-1RAs, therefore, have great potential as adjunct therapy for type 1 diabetes, mean HbA1c was still ≥7% after liraglutide treatment (6) and larger pharma-sponsored trials reported increased rates of hypoglycemia (8, 9). In seeking further improvement, the advent of sodium-glucose transport protein-2 (SGLT2) inhibitors is of importance since their mode of action is independent of insulinogenesis. Introduced for use in type 2 diabetes, SGLT2 inhibitors are selective antagonists of the sodium-glucose transporter subtype expressed in the proximal convoluted tubules of the kidneys. They act by inhibiting renal glucose re-absorption and therefore stimulating excretion of 60-100 grams per day of glucose in urine. SGLT2 inhibitors have been studied extensively in type 2 diabetes, including the landmark cardiovascular outcome trial EMPA-REG (10), but data have only quite recently begun to accumulate in type 1(11). Short term effects on urine glucose excretion of remogliflozin and empagliflozin are similar to those seen in type 2 diabetes and these translate into improved glycemic and other indices including HbA1c; fasting blood glucose, rates of hypoglycemia, daily insulin dose and weight (12). Glucose-lowering efficacy is attenuated in individuals with eGFR <45/ml/min/1.73m^2(13). Therefore, use of dapagliflozin is recommended for patients with eGFR>45/ml/min/1.73 m^2 (as per prescribing information). Weight loss accompanying increased urinary glucose excretion could translate into reduced cardiovascular complications as in type 2 diabetes, but there are no data so far available to support this hypothesis. More recently, in the Phase 3 trial DEPICT-1 we showed that dapagliflozin improves glycemic control and HbA1c over six months by about 0.45% with no severe hypoglycemia or diabetic ketoacidosis

(14). Similarly, sotagliflozin has been shown in a Phase 3 trial to allow a larger proportion of patients to achieve HbA1c lower than 7.0% with no severe hypoglycemia although the rate of diabetic ketoacidosis was higher in the sotagliflozin group (15). Both products are now being reviewed by regulatory agencies for the type 1 diabetes indication.

On the basis of the above, we hypothesize that adding an SGLT2 inhibitor to a combination of insulin and semaglutide (a weekly and potent GLP-1RA) will improve glycemic control in type 1 diabetes such that HbA1c levels of ≤7% are achieved more consistently. Our protocol is designed to allow an objective assessment of whether this triple therapy combination will work effectively and safely so that a majority of people with type 1 diabetes can reach a glycemic target HbA1c of ≤7%. In addition, since both semaglutide and dapagliflozin inhibitors reduce blood pressure and weight, it will allow an assessment of the combined effect of GLP-1RAs and SGLT2 inhibitors on these outcomes. Since there are no previous data on the effects of once weekly semaglutide or the combination of semaglutide and dapagliflozin compared to standard insulin treatment in type 1 diabetes, the design requires allocation of some participants to insulin therapy only.

Due to the potential ketogenic effect of SGLT-2 inhibitors, data from SGLT-2 inhibitors studies in type 1 diabetes were analyzed for it ketogenic effects and a consensus statement and guidelines on management of patients was recently published (Danne T et al., Diabetes Care. 2019 Feb 6).

Preliminary data

1) Liraglutide treatment in patients with type 1 diabetes (4): We treated 8 type 1 diabetes patients; mean age (38.5 years) and duration of diabetes (20±4 yrs), with liraglutide in addition to insulin for 24 weeks. As shown in table 1, there was a significant reduction in fasting and mean blood glucose concentrations, insulin doses, HbA1c and a reduction in appetite and caloric intake with weight loss. In addition, there was a reduction in the excursions of blood glucose concentrations, i.e., the standard deviations of blood glucose concentrations were markedly reduced (**figure 1**). The changes were observed within 48h and improved marginally thereafter with fine tuning of insulin doses and a further reduction in these doses. These observations are noteworthy in that study participants were well controlled and meticulous users of CSII and CGM. They had all tried hard to improve their diabetic control by adjusting insulin doses but had not succeeded because of hypoglycemia. We then demonstrated that poorly controlled and obese individuals with type 1 diabetes also experienced similar benefits of liraglutide treatment (2).

2) Liraglutide As Additional Treatment to Insulin in Patients with Type 1 Diabetes Mellitus: A Randomized Clinical Trial (6): Following the two above non-randomized studies we recently conducted a randomized clinical trial in which 72 individuals (Placebo=18; liraglutide = 54) with TYPE 1 DIABETES for at least one year, on insulin therapy and with no detectable C-peptide in plasma (mean BMI: 30±1; mean body weight: 84±2 kg ; mean HbA1c: 7.57±0.09%; mean age: 44±2 years; mean age of TYPE 1 DIABETES diagnosis: 20±1 years) were randomized to receive placebo, 0.6, 1.2 or 1.8mg of liraglutide daily for 12 weeks. In the 1.2 mg and 1.8 mg groups, mean change in average blood glucose was -10±2 and -10.0±1mg/dl, respectively, ($p<0.0001$ vs. placebo). In the 1.2mg group, HbA1c fell by 0.78% from 7.84±0.17% to 7.06±0.15% ($p<0.0001$, $p<0.01$

vs. placebo) and in the 1.8mg group fell by 0.42% from 7.41±0.15 to 6.99±0.15 ($p=0.001$, $p=0.39$ vs placebo). Time spent between 70 to 160 mg/dl increased by 5±1% ($p<0.05$ vs placebo) and time between 160-400 mg/dl decreased by 7±1% ($p<0.01$ vs placebo) in the 1.8 mg group with no additional hypoglycemia. During the 12 week period, the average total daily dose of insulin in the two groups fell by 12.4±3.9 and 10.0±2.3 units respectively ($p<0.05$ vs baseline and placebo). There was a reduction in body weight (from 83±4 to 78±5 kg, $p<0.0001$, in the 1.8mg group, from 96±4 to 91±4 kg, $p<0.001$, in the 1.2 mg group and from 80±4 to 77±4 kg, $p<0.01$ in the 0.6 mg group). A reduction in daily carbohydrate intake (171±17g vs. 127±18g; $p<0.01$ and 153±18g vs 115±16g, $p<0.01$ in the 1.2 mg and 1.8 mg groups, respectively) was also observed over 12 weeks. Systolic BP (SBP) in the 1.8 mg group fell by 3 mmHg (120±2 to 117±3 mmHg, $p=0.01$). CRP fell by 15±6 and 19±8% in the 1.2 and 1.8mg groups, respectively ($p<0.05$). Quality of life improved significantly compared to baseline and placebo (unpublished data).

3) Dapagliflozin as an Additional Treatment to Insulin and Liraglutide in Patients with Type 1 Diabetes Mellitus (16): The objective of this randomized clinical trial was to investigate whether addition of dapagliflozin to insulin and liraglutide results in a significant reduction in glycemia and body weight. 30 participants with TYPE 1 DIABETES on liraglutide therapy for at least last 6 months were randomized (in 2:1 ratio) to receive either dapagliflozin 10 mg or placebo daily for 12 weeks. With 12 weeks of dapagliflozin HbA1c fell by 0.66±0.08% from 7.8±0.21% ($p < .01$ vs placebo) but did not change significantly with placebo (7.40±0.20% to 7.30±0.20%). In one patient within one week of addition of dapagliflozin average glucose fell from 170 to 122 mg/dl while standard deviation was reduced from 71 to 58 (**Figure 2-diminished glycemic excursions)**. Body weight fell by1.9 +/- 0.54kg ($p < 0.05$ vs placebo). There was no additional hypoglycemia (blood glucose < 3.88 mmol/L; $p = 0.52$ vs placebo). In the dapagliflozin group, there were significant increases in the plasma concentrations of glucagon by 35% +/- 13% ($p < 0.05$), hormone-sensitive lipase by 29% +/- 11% ($p < 0.05$), free fatty acids by 74% +/- 32% ($p < 0.05$), acetoacetate by 67% +/- 34% ($p < 0.05$), and beta-hydroxybutyrate by 254% +/- 81% ($p < 0.05$). Urinary ketone levels also increased significantly ($P < .05$). None of these changes was observed in the placebo group. Two individuals in the dapagliflozin group developed ketoacidosis. Overall, addition of dapagliflozin to insulin and liraglutide in patients with TYPE 1 DIABETES resulted in a significant improvement in glycemia and in weight loss but tended to increase ketosis.

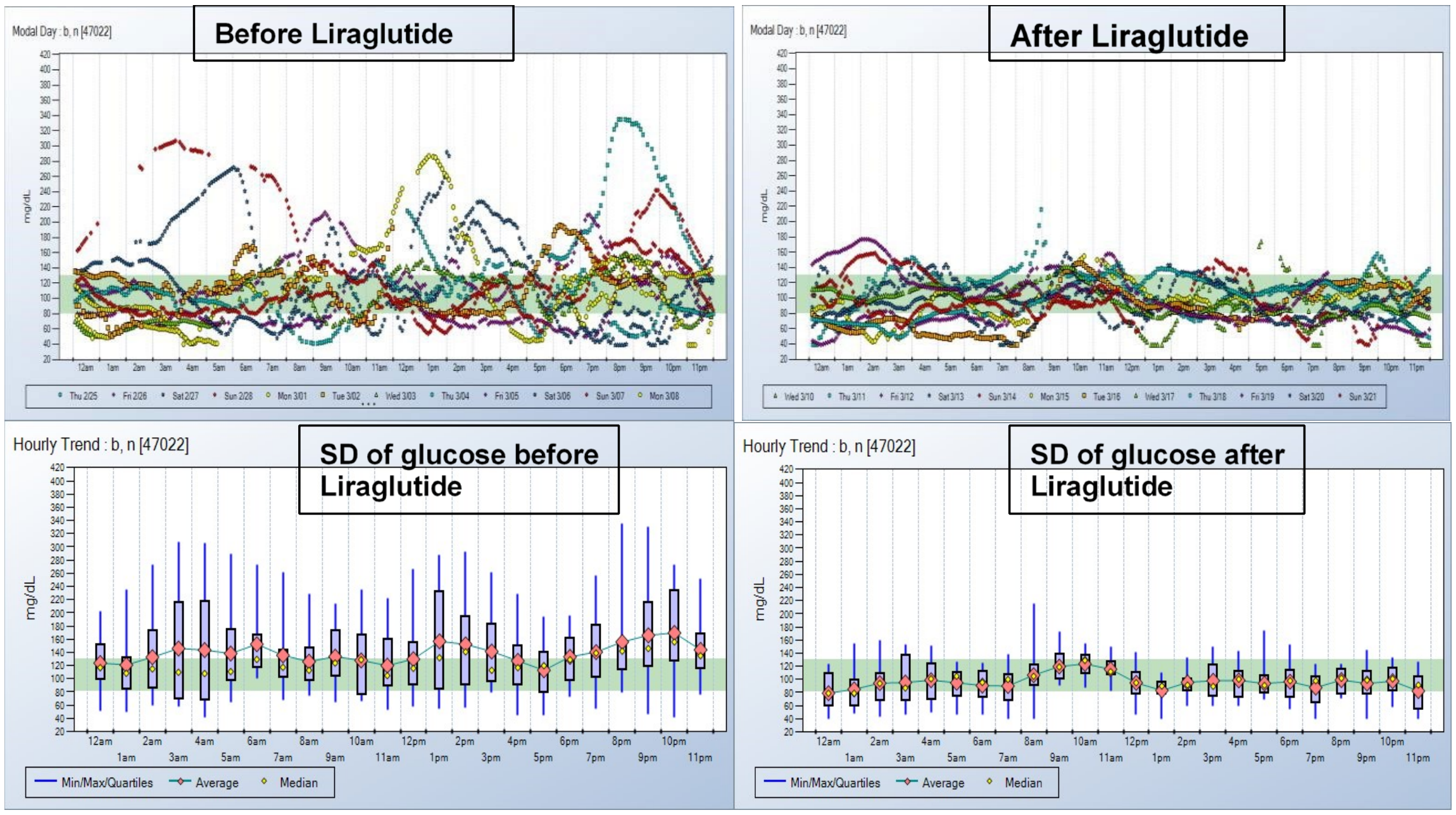

Figure 1: Weekly continuous glucose monitoring record of one patient prior to and following 1 week of liraglutide. Note the glycemic variability (SD) and the frequency of glucose concentrations within the target range prior to and following liraglutide

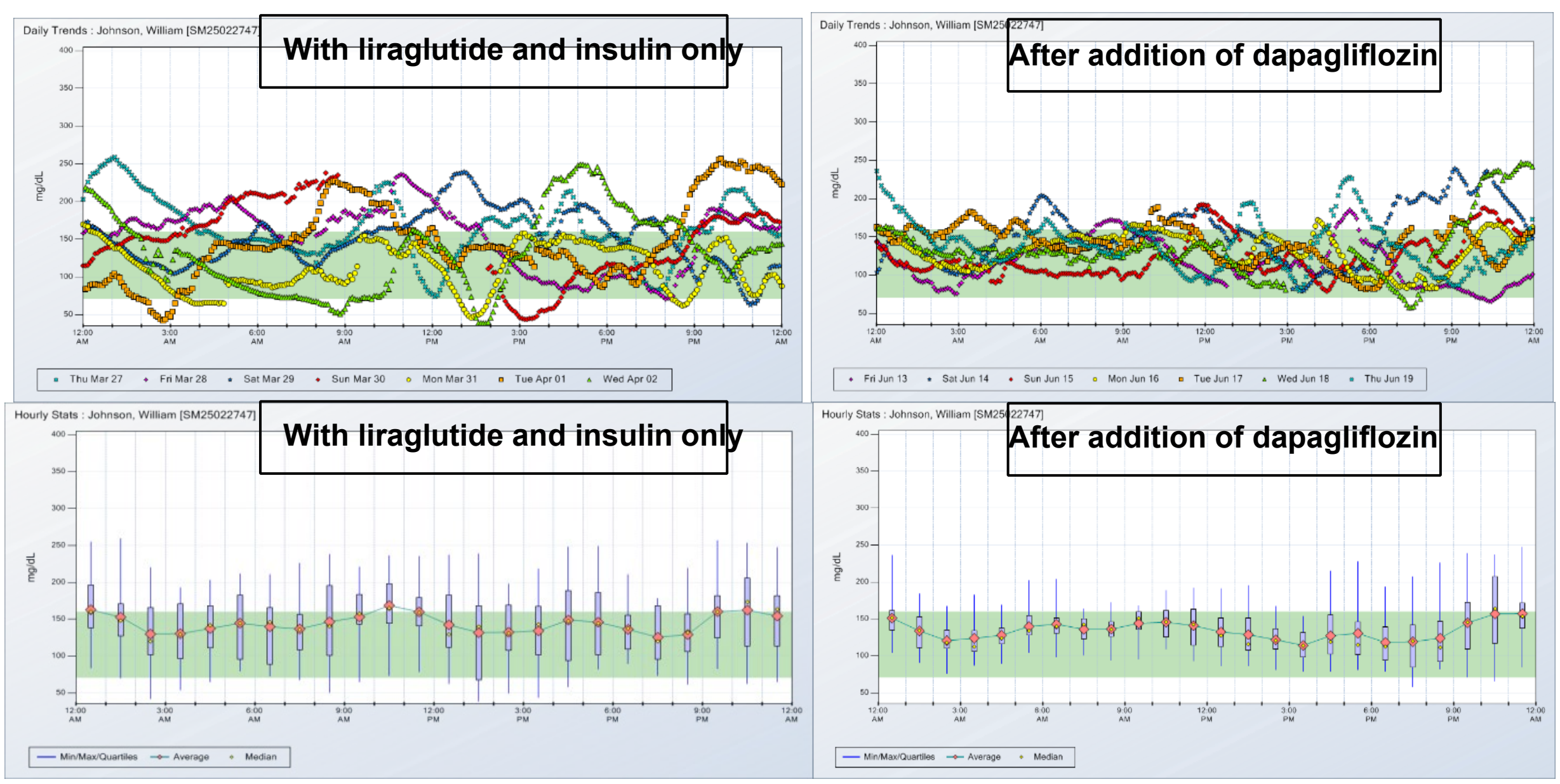

Figure 2: Weekly continuous glucose monitoring record of one patient prior to and following 8 weeks of the triple therapy combination with dapagliflozin and liraglutide. Note the glycemic variability (SD) (bottom) and the frequency of glucose concentrations within the target range prior to and following dapagliflozin addition.

3.2 Include complete citations or references.

Response:

1. Konrad K, Datz N, Engelsberger I, Grulich-Henn J, Hoertenhuber T, Knauth B, et al. Current use of metformin in addition to insulin in pediatric patients with type 1 diabetes mellitus: an analysis based on a large diabetes registry in Germany and Austria. *Pediatr Diabetes.* 2014.
2. Vella S, Buetow L, Royle P, Livingstone S, Colhoun HM, and Petrie JR. The use of metformin in type 1 diabetes: a systematic review of efficacy. *Diabetologia.* 2010;53(5):809-20.
3. Vella S, Buetow L, Royle P, Livingstone S, and Petrie JR. Metformin in type 1 diabetes reduces insulin requirements without significantly improving glycaemic control. Reply to Schatz H [letter]. *Diabetologia.* 2011;54(1):203-4.
4. Varanasi A, Bellini N, Rawal D, Vora M, Makdissi A, Dhindsa S, et al. Liraglutide as additional treatment for type 1 diabetes. *Eur J Endocrinol.* 2011;165(1):77-84.
5. Kuhadiya ND, Malik R, Bellini NJ, Patterson JL, Traina A, Makdissi A, et al. Liraglutide as additional treatment to insulin in obese patients with type 1 diabetes mellitus. *Endocrine practice : official journal of the American College of Endocrinology and the American Association of Clinical Endocrinologists.* 2013;19(6):963-7.
6. Kuhadiya ND, Dhindsa S, Ghanim H, Mehta A, Makdissi A, Batra M, et al. Addition of Liraglutide to Insulin in Patients With Type 1 Diabetes: A Randomized Placebo-Controlled Clinical Trial of 12 Weeks. *Diabetes Care.* 2016;39(6):1027-35.
7. Chaudhuri A, Ghanim H, Makdissi A, Green K, Abuaysheh S, Batra M, et al. Exenatide induces an increase in vasodilatory and a decrease in vasoconstrictive mediators. *Diabetes Obes Metab.* 2017;19(5):729-33.
8. Mathieu C, Zinman B, Hemmingsson JU, Woo V, Colman P, Christiansen E, et al. Efficacy and Safety of Liraglutide Added to Insulin Treatment in Type 1 Diabetes: The ADJUNCT ONE Treat-To-Target Randomized Trial. *Diabetes Care.* 2016;39(10):1702-10.
9. Ahren B, Hirsch IB, Pieber TR, Mathieu C, Gomez-Peralta F, Hansen TK, et al. Efficacy and Safety of Liraglutide Added to Capped Insulin Treatment in Subjects With Type 1 Diabetes: The ADJUNCT TWO Randomized Trial. *Diabetes Care.* 2016;39(10):1693-701.
10. Zinman B, Wanner C, Lachin JM, Fitchett D, Bluhmki E, Hantel S, et al. Empagliflozin, Cardiovascular Outcomes, and Mortality in Type 2 Diabetes. *N Engl J Med.* 2015;373(22):2117-28.
11. Petrie JR. SGLT2 inhibitors in type 1 diabetes: knocked down, but up again? *Lancet Diabetes Endocrinol.* 2017;5(11):841-3.
12. Mudaliar S, Armstrong DA, Mavian AA, O'Connor-Semmes R, Mydlow PK, Ye J, et al. Remogliflozin etabonate, a selective inhibitor of the sodium-glucose transporter 2, improves serum glucose profiles in type 1 diabetes. *Diabetes Care.* 2012;35(11):2198-200.
13. Kohan DE, Fioretto P, Tang W, List JF. Long-term study of patients with type 2 diabetes and moderate renal impairment shows that dapagliflozin reduces weight

and blood pressure but does not improve glycemic control. Kidney Int. 2014 Apr;85(4):962-71.
14. Dandona P, Mathieu C, Phillip M, Hansen L, Griffen SC, Tschope D, et al. Efficacy and safety of dapagliflozin in patients with inadequately controlled type 1 diabetes (DEPICT-1): 24 week results from a multicentre, double-blind, phase 3, randomised controlled trial. *Lancet Diabetes Endocrinol.* 2017;5(11):864-76.
15. Garg SK, Henry RR, Banks P, Buse JB, Davies MJ, Fulcher GR, et al. Effects of Sotagliflozin Added to Insulin in Patients with Type 1 Diabetes. *N Engl J Med.* 2017;377(24):2337-48.
16. Kuhadiya ND, Ghanim H, Mehta A, Garg M, Khan S, Hejna J, et al. Dapagliflozin as Additional Treatment to Liraglutide and Insulin in Patients With Type 1 Diabetes. *J Clin Endocrinol Metab.* 2016;101(9):3506-15.

17. Andrews NC, and Faller DV. A rapid micropreparation technique for extraction of DNA-binding proteins from limiting numbers of mammalian cells. *Nucleic Acids Res.* 1991;19(9):2499.

4.0 Study Design

4.1 Describe and explain the study design (e.g. case-control, cross-sectional, ethnographic, experimental, interventional, longitudinal, observational).

Response: This will be a two-center, randomized, open-label then double-blind, parallel group, placebo and standard of care controlled prospective study in type 1 diabetes. Eighty-one (81) patients will be randomized (open label, see below) into three groups (27 each) and will be either maintained on their regular insulin therapy for 12 months (standard therapy group) or started on semaglutide in addition to regular insulin treatment for six month (dual therapy and triple therapy groups). For those on insulin and semaglutide, after six months of stable treatment, dapagliflozin (triple therapy group) or placebo (dual therapy group) treatment will be initiated for an additional six months according to a second randomization (double-blind).

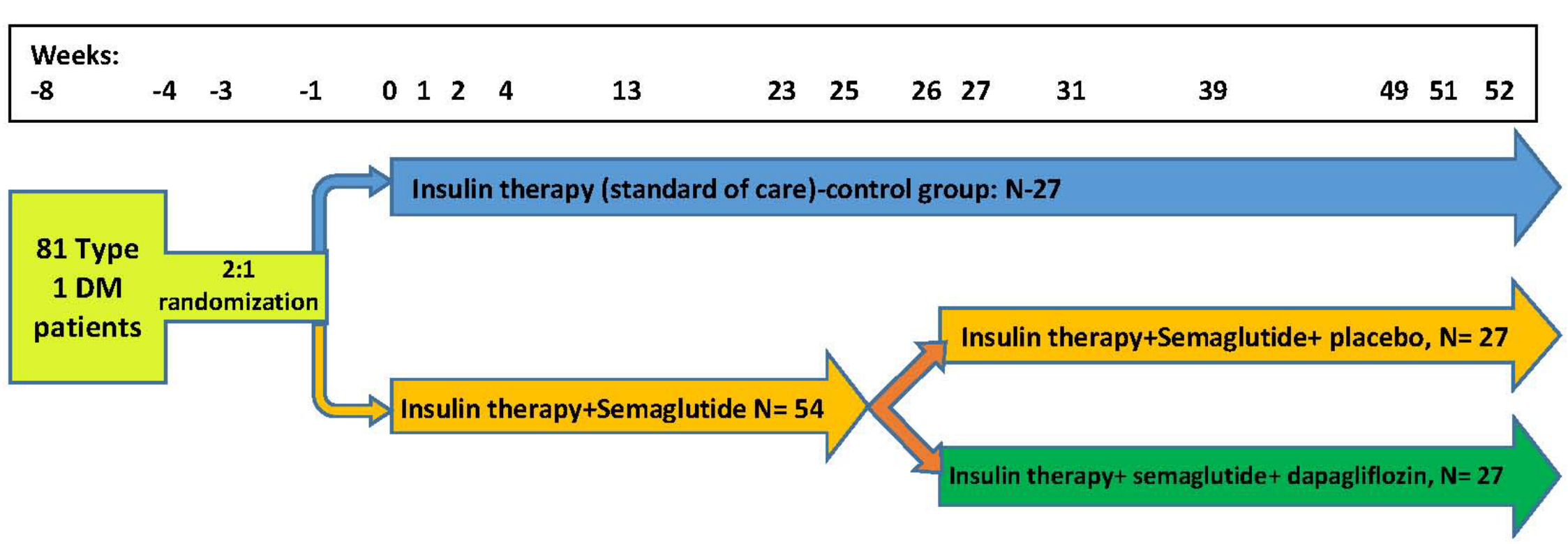

5.0 Local Number of Subjects

5.1 Indicate the total number of subjects that will be enrolled or records that will be reviewed locally.

Response: Patients will be enrolled locally at UB site and at the University of Glasgow site for a total of 81. Enrollment will be open to both sites until target enrollment is achieved.

5.2 If applicable, indicate how many subjects you expect to screen to reach your target sample (i.e. your screen failure rate).

Response: All screened and qualified patients will be enrolled and randomized

5.3 Justify the feasibility of recruiting the proposed number of eligible subjects within the anticipated recruitment period. For example, how many potential subjects do you have access to? What percentage of those potential subjects do you need to recruit?

Response: The Diabetes and Endocrinology Center of WNY is the largest Diabetes center in the WNY area, seeing between 60 to 100 type 1 diabetic patient every month. Therefore, the majority of recruited patients are our clinic patients. We also recruit a study participants through advertisement and other physician's references. These sources will suffice to recruit the needed number to subjects. The Glasgow center (Stobhill Ambulatory Care Hospital) reviews more than 300 patients per month.

6.0 Inclusion and Exclusion Criteria

*6.1 Describe the criteria that define who will be **included** in your final study sample.*

NOTE: This may be done in bullet point fashion.

Response:

Inclusion Criteria

1) Type 1 Diabetes for at least 1 year on staple use of continuous subcutaneous insulin infusion (CSII) or multiple daily (four or more) injections (MDI) of insulin for last 3 months.
2) C-peptide <0.23 nM
3) Minimum dose of insulin in Units/kg at entry: 0.5 U/kg for MDI and 0.4 U/kg for CSII
4) Regularly measuring blood sugars four or more times daily or use of CGM.
5) HbA1c between7.5-11% inclusive.
6) Well versed in CHO counting*
7) Age 18-70 years.
8) BMI ≥25 kg/m^2.

*In the opinion of the site Principal Investigator

6.2 *Describe the criteria that define who will be* ***excluded*** *from your final study sample.*

NOTE: This may be done in bullet point fashion.

Response:

Exclusion Criteria

1) Type 1 diabetes for less than 12 months, type 2 diabetes, chronic pancreatitis, MODY
2) Previous use of any agent other than insulin for treatment of diabetes in the last 3 months.
3) History of diabetic ketoacidosis (DKA) requiring medical intervention (e.g., emergency room visit and/or hospitalization) within 3 months prior to the screening visit
4) Patients on ketogenic or low (<50g/day) carbohydrate diet or who practice intermittent fasting.
5) Frequent episodes of severe hypoglycemia as defined by more than one episode requiring medical assistance, emergency care (paramedics or emergency room care), and/or glucagon therapy administered by a third-party individual within 1 month prior to the screening visit
6) Symptoms of poorly controlled diabetes that would preclude participation in this trial
7) Subjects on a commercial weight loss program with ongoing weight loss, or on an intensive exercise program
8) History of bariatric surgery or lap-band procedure within 12 months prior to screening
9) History of Addison's disease or chronic adrenal insufficiency
10) History of diabetes insipidus
11) Hepatic disease or cirrhosis
12) Aspartate Aminotransferase (AST) > 3X Upper limit of normal (ULN) *and/or* Alanine aminotransferase (ALT) > 3X ULN
13) Serum Total Bilirubin > 2X ULN unless exclusively caused by Gilbert's Syndrome
14) Hemoglobin < 11.0 g/dL (110 g/L) for men; hemoglobin < 10.0 g/dL (100 g/L) for women.
15) Coronary event or procedure (myocardial infarction, unstable angina, coronary artery bypass, surgery or coronary angioplasty) in the previous 3 months or patients with congestive heart failure.
16) ESRD on hemodialysis; and or e-GFR < 45 ml/min/$1.73m^2$
17) HIV or Hepatitis B/C positive status
18) Any other life-threatening, noncardiac disease
19) History of pancreatitis
20) Women who are pregnant or women of childbearing potential who are not using adequate contraception or who are breast feeding
21) Inability to give informed consent
22) History of gastroparesis
23) History of medullary thyroid carcinoma or MEN 2 syndrome

24) History of serious hypersensitivity reaction to these agents
25) Painful gallstones
26) Alcoholism
27) Hypertriglyceridemia (>500 mg/dl)
28) Recurrent genital mycotic infection.
29) Hypovolemic patients or with chronic renal insufficiency.
30) Patients with any malignancy except treated in situ malignancy and basal cell carcinoma of the skin
31) Unexplained hematuria
32) Patients with a history of diabetic retinopathy
33) Use of an investigational agent or therapeutic regimen within 30 days of study
34) Participation in any other concurrent interventional clinical trial

6.3 *Indicate specifically whether you will include any of the following special populations in your study using the checkboxes below.*

NOTE: Members of special populations may not be targeted for enrollment in your study unless you indicate this in your inclusion criteria.

Response: NA

☐ Adults unable to consent

☐ Individuals who are not yet adults (infants, children, teenagers)

☐ Pregnant women

☐ Prisoners

6.4 *Indicate whether you will include non-English speaking individuals in your study.* ***Provide justification if you will exclude non-English speaking individuals.***

In order to meet one of the primary ethical principles of equitable selection of subjects, non-English speaking individuals may ***not*** *be routinely excluded from research as a matter of convenience.*

In cases where the research is of therapeutic intent or is designed to investigate areas that would necessarily require certain populations who may not speak English, the researcher is required to make efforts to recruit and include non-English speaking individuals. However, there are studies in which it would be reasonable to limit subjects to those who speak English. Some examples include pilot studies, small unfunded studies with validated instruments not available in other languages, studies with numerous questionnaires, and some non-therapeutic studies which offer no direct benefit.

Response:

We have no non-English speaking patients in this population. We have patients that English is a second language, but they are able to read, write and understand it. This population is less than 10% of the total population. No benefit is being withheld from not participating in the study.

7.0 Vulnerable Populations

If the research involves special populations that are considered vulnerable, ***describe the safeguards included to protect their rights and welfare.***

NOTE: You should refer to the appropriate checklists, referenced below, to ensure you have provided adequate detail regarding safeguards and protections. You do not, however, need to provide these checklists to the IRB.

7.1 For research that involves ***pregnant women,*** *safeguards include:*
NOTE CHECKLIST: Pregnant Women (HRP-412)

Response: We will not be recruiting subjects from vulnerable populations

☒ **N/A**: This research does not involve pregnant women.

7.2 For research that involves ***neonates of uncertain viability or non-viable neonates,*** *safeguards include:*
NOTE CHECKLISTS: Non-Viable Neonates (HRP-413), or Neonates of Uncertain Viability (HRP-414)

Response:

☒ **N/A:** This research does not involve non-viable neonates or neonates of uncertain viability.

7.3 For research that involves ***prisoners****, safeguards include:*
NOTE CHECKLIST: Prisoners (HRP-415)

Response:

☒ **N/A:** This research does not involve prisoners.

7.4 For research that involves ***persons who have not attained the legal age for consent to treatments or procedures involved in the research ("children")****, safeguards include:*
NOTE CHECKLIST: Children (HRP-416)

Response:

☒ **N/A:** This research does not involve persons who have not attained the legal age for consent to treatments or procedures ("children").

7.5 For research that involves ***cognitively impaired adults****, safeguards include:*
NOTE CHECKLIST: Cognitively Impaired Adults (HRP-417)

Response:

☒ **N/A:** This research does not involve cognitively impaired adults.

7.6 *Consider if other specifically targeted populations such as students, employees of a specific firm, or educationally or economically disadvantaged persons are vulnerable.* ***Provide information regarding their safeguards and protections, including safeguards to eliminate coercion or undue influence.***

Response: No specific populations or vulnerable groups will be targeted. All subjects enrolled in this study will be of legal adult consenting age with the ability to speak, read and interpret the English language. Patients will have the ability to speak with the research team regarding any questions or concern they have before signing the consent. Research team have the ability to evaluate patient's cognitive abilities through these interactions with patients. Patients are made aware that this study is voluntary and they are able to stop participating at any time they feel uncomfortable. Patients are not to be pressured into participating and their clinic standard of care will remain the same if they participate or choose not to participate.

8.0 Eligibility Screening

8.1 *Describe* ***screening procedures*** *for determining subjects' eligibility. Screening refers to determining if prospective participants meet inclusion and exclusion criteria.*

Include all relevant screening documents with your submission (e.g. screening protocol, script, questionnaire).

Response:

After obtaining partial HIPAA waiver, patient charts will be screened according to the study inclusion and exclusion criteria by our trained clinical staff and physicians. In additions, the study clinical team will evaluate their clinic patients for possible participation in this study according to the inclusion and exclusion criteria at the Diabetes and Endocrinology Center of WNY clinics. Physicians will speak to them about their interests in participating in research. If the patient agrees, their information will be given to the research coordinator to be contacted for further evaluation. The research coordinator will contact interested patients and those meeting the inclusion and exclusion criteria based on preliminary phone evaluation will be invited to participate in the study. All personal information will be kept confidential and locked in the coordinator office.

Prospective participants will be asked to read the consent and any questions they may have regarding the protocol will be answered. If the subject wants to participate in the study, they will be asked to sign the informed consent form. The subject's medical history and current medications will be obtained as well as their blood pressure and vitals. A physical examination will also be done. Blood and urine samples will be taken in order to evaluate HbA1c, CBC, CMP and liver and kidney functions and pregnancy status. Females of childbearing age, will have to be on two forms of birth control to prevent pregnancy. Patients meeting all the

inclusion and exclusion criteria based on all screening tests will be enrolled in the study.

☐ **N/A:** There is no screening as part of this protocol.

9.0 Recruitment Methods

☐ **N/A:** This is a records review only, and subjects will not be recruited. NOTE: If you select this option, please make sure that all records review procedures and inclusion/exclusion screening are adequately described in other sections.

9.1 Describe when, where, and how potential subjects will be recruited.

NOTE: Recruitment refers to how you are identifying potential participants and introducing them to the study. Include specific methods you will use (e.g. searching charts for specific ICD code numbers, Research Participant Groups, posted advertisements, etc.).

Response:

Participants will be identified by prescreening clinical charts, patient doctor interaction at the time of their visits to the Diabetes Endocrinology Center of WNY Locations include:

1. 1020 Youngs Road, Williamsville NY 14221
2. 705 Maple Road, Williamsville NY 14221
3. 462 Grider Street, Buffalo NY 14215
4. 1000 Youngs Road, Suite 105, Williamsville NY 14221

Patients can be also referred by other physicians and providers

The remainder of the participants (57 patients) will be recruited from NHS Greater Glasgow and Clyde Diabetes clinics (Scotland, UK). Separate local IRB/Ethical approval will be sought to cover this site (based on discussions with UBIRB director).

The study clinical team will evaluate their clinic patients for possible participation in this study according to the inclusion and exclusion criteria at the Diabetes and Endocrinology Center of WNY clinics. Patients that may qualify for the study are referred to the research team for further eligibility evolution. Patients meeting the inclusion and exclusion criteria based on preliminary phone evaluation will be invited to participate in the study.

Participants will also be identified by:

-Flyers, advertisements on Facebook, ,

- Researchmatch.org, UB's participate in research (PIR) website, and through Buffalo research registry (BRR).

- We will also use the TriNetX/i2b2 database through UB's Institute for Healthcare Informatics (IHI) to recruit participants from the UBMD medical database.

Along with this, a radio ad will be on the air of the radio station STAR102.5 at various times during the day during the month of mid January to mid February

9.2 Describe how you will protect the privacy interests of prospective subjects during the recruitment process.

NOTE: Privacy refers to an individual's right to control access to him or herself.

Response: Patient charts will be screened according to the study inclusion and exclusion criteria by our trained clinical staff and physicians. If the patient qualifies and is of consenting age, the physicians will speak to them about their interests in participating in research in a private room where no one else can hear the conversation. If the patient agrees, their information will be given to the research coordinator to be contacted for further evaluation. No personal information will be shared without patient's approval and will not be used for any purpose except for this research. All personal information will be kept confidential and locked in the coordinator office.

The privacy of patients recruited using the TriNetX/ i2b2 data will be protected by following the UB IRB-approved procedure. A letter will be sent to the eligible patient's physician (see attached). After one week, if the physician doesn't contact the research team to refuse, a recruitment letter will be sent to participants via mail (see attached). If we don't hear from the patients within one week, one follow-up call will be made (see attached phone script). If at any point the physician or the patient declines, the patient will be removed from the contact list.

The Clinical and Translational Science Institute's (CTSI) Community Engagement Team (CET) hosts the Buffalo Research Registry (BRR, IRB Approved STUDY00000806), a resource that connects researchers looking to recruit participants and community members looking to get involved with research. To participate in the registry, volunteers complete a voluntary intake form. Volunteers have agreed to be contacted about potential research opportunities based on their self-reported information.

As described in the BRR's IRB Approved Protocol (STUDY00000806), key personnel from the CTSI will serve as the conduit between our research team and the registry volunteers. Key personnel will verify our IRB study approval, review inclusion and exclusion criteria and will sort the registry data accordingly. In terms of recruiting for our study, key personnel will pull volunteer reports using the inclusion and exclusion criteria. Key personnel will centrally invite volunteers to participate in the study and provide a warm hand off to our team. There are two ways we are sharing our study. These include:

Electronically- Using the volunteer report from REDCap, key personnel from the CTSI will send initial e-mail invites to introduce our study to registry volunteers.

Post Mail- Key personnel from the CTSI will provide our team with contact information (i.e. first name, last name, post mail address) to prepare a mailing. This information will be shared in a password protected excel spread sheet. The password to the spreadsheet will be sent in a separate follow-up e-mail. We will

only use the list for this protocol and we agree to destroy the list once recruitment for the study has closed.

We also plan on conducting follow-up by phone/email after initial contact. This contact information will be provided in a password protected excel spread sheet. The password to the spreadsheet will be sent in a separate follow-up e-mail. We will only use the list for this protocol and we agree to destroy the list once recruitment for the study has closed.

We will have no access to the health information provided in the registry.

9.3 Identify any materials that will be used to recruit subjects.

NOTE: Examples include scripts for telephone calls, in person announcements / presentations, email invitations.

For advertisements, include the final copy of printed advertisements with your submission. When advertisements are taped for broadcast, attach the final audio/video tape. NOTE: You may submit the wording of the advertisement prior to taping to ensure there will be no IRB-required revisions, provided the IRB also reviews and approves the final version.

Response: In addition to screening clinical charts, participations will be identified through; flyer advertisement, researchmatch.org, and Facebook. The study will also be put on UB's PIR website.

The researchmatch.org contact message and listing is provided as separate attachment to this submission (RM IRB Template Language-JDRF)

Sample letters of physician permission and patient contact and phone prescreen script are also included in this submission.

10.0 Procedures Involved

*10.1 Provide a description of **all research procedures or activities** being performed and when they are performed once a subject is screened and determined to be eligible. Provide as much detail as possible.*

NOTE: This should serve as a blueprint for your study and include enough detail so that another investigator could pick up your protocol and replicate the research. For studies that have multiple or complex visits or procedures, consider the addition of a schedule of events table in in your response.

Response:

Each patient will spend about 55 weeks in the trial comprising three weeks of monitoring to stabilize baselines and ensure adherence and commitment to participation before randomization into an intervention arm for an additional period of 52 weeks. Following a completed screening visit and meeting the inclusion and exclusion criteria, each patient will have a total of 16 study visits. At weeks 3, 5, 8, 16, 20, 29, 35, 44 and at 2 weeks after the end of the study, each patient will receive a phone call to document any adverse effects or other changes. There will be no long term follow-up following the end of the study.

Participants will be treated with either insulin only (control arm) for the whole duration of the study or with insulin and semaglutide weekly s.c. injection (titrated

up to 1 mg or the maximum tolerated dose, see below) for the first six months then either dapagliflozin (Triple therapy arm) or placebo (dual therapy) daily will be added for an additional six months. Semaglutide will be started at a dose 0.25 mg once weekly for two weeks then increased to 0.5 mg dose. If well tolerated it will be increased to 1 mg once weekly at week 4. Dapagliflozin (or placebo) will be started at a dose of 5 mg daily at week 26 and if well tolerated increased after 1 week to 10 mg (2x5mg tablets) daily to ensure drug tolerability and to avoid any hypoglycemia or hypotension. Patients who do not tolerate the increase in dosing of either medications will be maintained at the highest tolerated dose.

Treatments used

1- Insulin: All patients (81 patients) will continue to self-administer their prescribed insulin as part of their standard of care. Insulin dose will be titrated during the study to control blood glucose average between 90-180 mg/dl and to avoid hypoglycemia (blood sugar <70 mg/dl) or hyperglycemia (blood sugar >250 mg/dl). Among all patients, 3827 patients will be randomized (open-label) to remain on insulin therapy only (standard therapy group) while the other 76 will start the investigational therapies.
2- Semaglutide: a once weekly a GLP-1 mimetic injection (pen) indicated for type 2 diabetes. Doses to be used: start at 0.25mg then increase to 0.5mg following 2 weeks and to 1mg weekly s.c. injection 2 weeks later. Semaglutide will be added to a background of insulin treatment for 12 months in 76 patients who will be randomized (double-blind) into 2 groups (dual and triple therapy groups) at the end of 26 weeks.
3- Farxiga (dapagliflozin): a once daily SGLT-2 inhibitor tablet indicated for type 2 diabetes. Doses used: starting with 5mg and increase after 1 week to 10mg oral administration daily. Dapagliflozin will be started according to the randomization (Interactive Web System, hosted by University of Glasgow) in 3827 patients at 26 weeks as an additional treatment on a background of insulin and semaglutide therapy and end at 52 weeks (triple therapy group).
4- Placebo: dapagliflozin matching placebo (supplied by Astra Zeneca) will be started in 27 patients at 26 weeks as an additional treatment on a background of insulin and semaglutide therapy and end at 52 weeks (dual therapy group).

As shown in **Table 1,** fasting blood and urine samples will be obtained prior to drug administration (weeks 0) and at 13, 26, 39 and 52 weeks after the commencement of the study. Optional fat biopsies from the abdominal subcutaneous tissue will be collected at weeks 0, 26 and 52. Continuous Glucose Monitoring (Dexcom G6 with blinded receiver) will be performed for three 21-days periods on weeks -3 to 0, 23 to 26 and from 49 to 52 weeks. An optional 24hr blood pressure monitoring will be performed at baseline (week -1) and at 13, 25, 39 and 51 weeks in order to characterize the time course of antihypertensive effects, which may be early (i.e. independent of weight loss) as indicated by our preliminary data. High fat high caloric

(HFHC) Meal challenges will be performed at baseline (week -1) and at 25 and 51 weeks with assessment of glucose excursions and postprandial glucagon (AUC) as well as postprandial inflammation and oxidative stress. Telephone calls will be conducted at weeks 3, 5, 8, 16, 20, 29, 35, 44 and at two weeks after the end of the study to document any side effects or any other patient's reported issues or comments.

Study Visits:

Screening Visit (Screening Visit, Week -8 to -4): Patients will be asked to read and understand the consent and any questions regarding the protocol will be answered. If they want to participate in the study, they will be asked to sign the informed consent form. Their medical history and current medications will be obtained as well as blood pressure and vitals. A physical examination will also be done. Blood samples will be taken in order to evaluate HbA1c, CBC, CMP, C-peptide, kidney and liver functions, ketone bodies, and urine for pregnancy status in females.

Study Visit 1 (Week -3): Participants who meet entry criteria will be scheduled to for their next visit at -3 week. Vitals will be taken including resting (min. 10 minutes) blood pressure measurement (will be repeated from same arm every visit). Insulin pump will be downloaded and/or CGM/fingerstick blood sugar will be reviewed and the appropriate insulin adjustments will be made by the investigator to target fasting blood glucose concentrations between 90-180 mg/dl if possible at all times without having additional hypoglycemia (less than 70 mg/dl). Blood sugars and ketone bodies will be measured via a fingerstick blood sample using glucose and ketones meter (Abbott Laboratories) and daily blood sugars collected and reviewed. Urine for pregnancy status and ketone bodies assessment. We will instruct patients on how to use and insert the Continuous Glucose Monitoring System (Dexcom G6 with blinded receiver). All subjects will be advised to monitor their capillary blood glucose by CGM or fingerstick at least 4 times a day (before meals and at bedtime) through-out the study and to wear their CGM constantly for next 20 day. Carb counting technique will be reviewed and any questions answered on how to use the pump or calculate insulin doses.

Study Visit 2 (Week -1±2days): Patients will be asked to come in the fasting state (no food or calorie intake for 10-12 hours). Their vitals will be taken. Blood sugars and ketone bodies will be measured via a CGM/fingerstick blood sample using glucose and ketones meter (Abbott Laboratories) to assess their glycemic control prior to meal challenge. If their blood sugar is below 70mg/dl or more than 250mg/dl the visit will be rescheduled for an alternative date. Insulin pump will be downloaded and/or CGM/fingerstick blood sugar will be reviewed and insulin adjustment will be made if necessary. An IV port will be placed in order to obtain blood samples throughout the duration of the visit. Patients will undergo a meal challenge test (described below). At the end of the meal test, they will be asked to wear a 24 hour blood pressure monitor (optional). A 24 hr urine container will be dispensed to collect a 24 hour urine sample to bring in at the Week 0 visit. Daily Glucose logs will be reviewed.

Study visit 3 (Week 0±2days): Patients will be asked to come in the fasting state (no food or calorie intake for 10-12 hours). Vitals will be taken. Blood sugars and ketone bodies will be measured via a CGM/fingerstick blood sample using glucose and ketones meter (Abbott Laboratories) and daily blood sugars collected and reviewed. The 24 hour urine container will be collected. A fasting blood sample will be taken (45 mL) to evaluate study endpoints, CBC, CMP and HbA1c. A urine sample will be collected to evaluate ketone bodies and, in females, pregnancy status. Insulin pump will be downloaded and/or CGM/fingerstick blood sugar will be reviewed and the appropriate insulin adjustments will be made by the investigator. In general, insulin dose adjustments should not be >10% from current total insulin dose or >20% from baseline (week 0) total insulin dose. They will discontinue use of the study CGM. An optional fat biopsy will be performed. We will ask them to fill out quality of life questionnaires. Study drug will be dispensed depending on what group they are randomized to. This includes either (1) staying on the insulin regimen given by their doctor, or (2) a combination of their current insulin regimen and a once daily injection of semaglutide. Semaglutide will be started on site at the 0.25 mg dose. The 24 hr blood pressure monitor will be collected.

Study Visit 4 (Week 1±2days): Vitals will be taken. Blood sugars and ketone bodies will be measured via a CGM/fingerstick blood sample using glucose and ketones meter (Abbott Laboratories) and daily blood sugars collected and reviewed. Insulin dose will be adjusted if needed as in visit 3. At any time, if HbA1c is <7%, insulin dose adjustment should not be >5%. Semaglutide 0.25mg will be injected on site. Participants will be instructed to call their research center or their doctor directly in case of any problem or untoward side effects. They will be specifically asked to call if they have unmanaged hypoglycemia (blood sugar <70 mg/dl) or hyperglycemia (blood sugar >250 mg/dl) or if occurring in more than one occasion.

Study Visit 5 (Week 2±2days): Vitals will be taken. Blood sugars and ketone bodies will be measured via a CGM/fingerstick blood sample using glucose and ketones meter (Abbott Laboratories) and daily blood sugars collected and reviewed. Insulin dose will be adjusted if needed as in visit 3. Semaglutide 0.5mg dose will be injected on site. If they are experiencing mild nausea, they will be continued on the 0.25 mg dose for another week. Supply of the drug will be given to last until the next visit. Patients will be advised to increase the study dose to 0.5 mg a day if they are ok.

Study Visit 6 (Week 4±1 week): Patients will be asked to bring in used study pens for drug accountability (to be recorded in eCRF). Vitals will be taken. Blood sugars and ketone bodies will be measured via a CGM/fingerstick blood sample using glucose and ketones meter (Abbott Laboratories) and daily blood sugars collected and reviewed. Insulin dose will be adjusted if needed. Supply of the drug will be given to last until the next visit. A urine sample will be collected to evaluate ketone bodies and, in females, pregnancy status. Patients will be advised to increase the study dose to 1.0 mg/week. If they experience mild nausea, they will be continued on the 0.5 mg dose for another week. At all times, patients will be maintained at the maximum tolerated dose. A 24 hr urine container will be dispensed to collect a 24 hour urine sample to bring in at the Week 13 visit.

Study Visit 7 (Week 13±1 week): Patients will be asked to come in the fasting state (10-12 hours fasting), and to bring in your used study pens for drug accountability. Blood sugars and ketone bodies will be measured via a CGM/fingerstick blood sample using glucose and ketones meter (Abbott Laboratories) and daily blood sugars collected and reviewed. Insulin dose will be adjusted if needed as in. Supply of the drug will be given to last until visit 26. Vitals will be taken. A urine sample will be collected to evaluate ketone bodies and, in females, pregnancy status. The 24 hour urine container will be collected. A fasting blood sample will be taken (45 mL) to evaluate study endpoints, CBC, CMP and HbA1c. Patients who agreed to participate in the24 hour blood pressure monitoring will be asked to wear it for the next 24 hr.

Study Visit 8 (Week 23±1 week): Patients will be asked to bring in used study pens for drug accountability. Vitals will be taken. Blood sugars and ketone bodies will be measured via a CGM/fingerstick blood sample using glucose and ketones meter (Abbott Laboratories) and daily blood sugars collected and reviewed. A urine sample will be collected to evaluate ketone bodies and, in females, pregnancy status. Insulin dose will be adjusted if needed. All subjects will be reminded to monitor their capillary blood glucose by fingerstick at least 4 times a day (before meals and at bedtime) through-out the study. CGM will be inserted and patients asked to wear their CGM constantly for next 20 day.

Study visit 9 (Week 25±1 week): Patients will be asked to come in the fasting state, and to bring in used study pens for drug accountability. Vitals will be taken. A urine sample will be collected to evaluate ketone bodies. Blood sugars and ketone bodies will be measured via a CGM/fingerstick blood sample using glucose and ketones meter (Abbott Laboratories) and daily blood sugars collected and reviewed. Insulin dose will be adjusted if needed. HbA1c will be drawn. Supply of the drug will be given to last until the next visit. A 24 hr urine container will be dispensed to collect a 24 hour urine sample to bring in at the Week 26 visit. A HFHC Meal challenges will be performed as in visit 2. Patients who agreed to participate will be asked to wear a 24 hour blood pressure monitoring for the next 24 hr.
Participants in the semaglutide group will be provided supplies for urine ketones measurement (KetoStix) and blood ketones meter and strips. Research coordinator will instruct the patients on the use of meter and reading of the measuring strips. Patients will be instructed to start measuring urinary and blood ketones every morning for three consecutive days prior to next visit (starting dapagliflozin treatment) and to report results at the time of their next visit.

Study visit 10 (Week 26±1 week): Patients will be asked to come in the fasting state, and to bring in used study pens for drug accountability. Previous 3 days blood and urine ketones levels will be reviewed. Patients with normal fasting urine (<+, or < small) or blood ketone (<0.6 mmol) will proceed to next treatment phase included in this visit. Patients who report higher ketone values in at least 1 out of the 3 days will have their glucose levels, insulin dose, carb intake reviewed and managed as necessary. They will be rescheduled for next week (7±1 day) and asked to repeat the 3 days testing before next visit.

Vitals will be taken. Blood sugars and ketone bodies will be reviewed via a CGM/fingerstick blood sample using glucose and ketones meter (Abbott Laboratories) and daily blood sugars collected and reviewed. Insulin dose will be adjusted if needed. In general, insulin dose adjustments should **NOT** be >10% from current total insulin dose (or 5% if HbA1c is <7%) or >20% from baseline total insulin dose. The adjusted total insulin dose in this visit will serve as the baseline total insulin dose for the remaining 6 months of the study and future limits of dose adjustments will be based on this new baseline. The CGM will be collected. A urine sample will be collected to evaluate ketone bodies and, in females, pregnancy status. The 24 hour urine container will be collected. A fasting blood sample will be taken (45 mL) to evaluate study endpoints, CBC, CMP and C-peptide. An optional fat biopsy will be performed. We will ask patients to fill out quality of life questionnaires. At this point, those patients randomized to receive semaglutide, they will now be randomized to receive an actual dapagliflozin pill or a placebo pill that looks like dapagliflozin. If they are randomized to receive actual dapagliflozin, they will start at the 5 mg dose for one week. Supply of the study drug will be given to last until week 27.

All patients will be asked to perform a daily (preferably early morning or before bedtime) urinary test of ketone bodies using KetoStix and record all outcomes until the end of the study. Also, they will be provided with additional blood ketone strips to measure blood ketones when needed. If results of these daily scheduled (i.e. not done due to symptoms) urine ketone measurements, are small or higher (+1 or more), they will be instructed to measure blood ketones and to repeat both measurements after 60 min. If consecutive measurements show moderate or higher ketones in urine or blood ketones >0.6mM, the patient will be instructed to call the research center or their doctor directly. For any abnormal ketone levels taken due to symptoms, the DKA consensus guidelines (Danne T et al., Diabetes Care. 2019 Feb 6) are to be followed instead (as in section 21).

Participants will be instructed to call their research center or their doctor directly in case of any problem or untoward side effects. They will be specifically asked to call if they have unmanaged hypoglycemia (blood sugar <70 mg/dl) or hyperglycemia (blood sugar >250 mg/dl) or if occurring in more than one occasion. Participants will be instructed to remain hydrated and report any genital mycotic or urinary tract infections.

Study visit 11 (Week 27±2 days): Patients will be asked to bring in used study pens for drug accountability (recorded in eCRF). Vitals will be taken. Daily blood sugars and urinary ketones measurements using KetoStix will be collected and reviewed. Blood sugars and ketone bodies will be measured via a CGM/fingerstick blood sample using glucose and ketones meter (Abbott Laboratories). A urine sample will be collected to evaluate urine ketone level. Insulin dose will be adjusted if needed. In general, insulin dose adjustments should not be >10% from current total insulin dose (or 5% if HbA1c is <7%) or >20% from NEW baseline total insulin dose established at visit 10. If at any time during the study total insulin dose reduction is >20% and total insulin dose in <0.40 U/Kg, additional monitoring protocol (see later) will be implemented. Supply of the drug will be given to last until week 39. If patients are receiving actual dapagliflozin, they will now start at the 10 mg dose for the remainder of the study.

Study Visit 12 (Week 31±1 week): Patients will be asked to bring in used study pens and tablets for drug accountability. Vitals will be taken. Daily blood sugars and urinary ketones

measurements using KetoStix will be collected and reviewed. Blood sugars and ketone bodies will be measured via a CGM/fingerstick blood sample using glucose and ketones meter (Abbott Laboratories). A urine sample will be collected to evaluate ketone bodies and, in females, pregnancy status. Insulin dose will be adjusted if needed as in visit 11. A blood sample will be taken (15 mL) to evaluate CBC and CMP. A 24 hr urine container will be dispensed to collect a 24 hour urine sample to bring in at the Week 39 visit.

Study visit 13 (Week 39±1 week): Patients will be asked to come in the fasting state, and to bring in used study pens and tablets for drug accountability. Vitals will be taken. Daily blood sugars and urinary ketones measurements using KetoStix will be collected and reviewed. Blood sugars and ketone bodies will be measured via a CGM/fingerstick blood sample using glucose and ketones meter (Abbott Laboratories). Insulin dose will be adjusted if needed as in visit 11. Supply of the drug will be given to last until the end of the study. A urine sample will be collected to evaluate ketone bodies and, in females, pregnancy status. The 24 hour urine container will be collected. A fasting blood sample will be taken (45 mL) to evaluate study endpoints, CBC, CMP and HbA1c. Patients who have provided consent will be asked to wear a 24 hour blood pressure monitor.

Study visit 14 (Week 49±1 week): Patients will be asked to bring in used study pens and tablets for drug accountability. Vitals will be taken. Daily blood sugars and urinary ketones measurements using KetoStix will be collected and reviewed. Blood sugars and ketone bodies will be measured via a CGM/fingerstick blood sample using glucose and ketones meter (Abbott Laboratories). A urine sample will be collected to evaluate ketone bodies and, in females, pregnancy status. Insulin dose will be adjusted if needed as in visit 11. All subjects will be reminded to monitor their capillary blood glucose by fingerstick at least 4 times a day (before meals and at bedtime) through-out the study. CGM will be inserted and patients asked to wear their CGM constantly for the next 20 days.

Study visit 15 (Week 51±1 week): Patients will be asked to come in the fasting state, and to bring in used study pens and tablets for drug accountability. Vitals will be taken. Daily blood sugars and urinary ketones measurements using KetoStix will be collected and reviewed. Blood sugars and ketone bodies will be measured via a CGM/fingerstick blood sample using glucose and ketones meter (Abbott Laboratories). A urine sample will be collected to evaluate urine ketone levels. Insulin dose will be adjusted if needed as in visit 11. A 24 hr urine container will be dispensed to collect a 24 hour urine sample to bring in at the Week 52 visit. A HFHC Meal challenges will be performed as in visit 2. Patients will be asked to wear the optional 24 hour blood pressure monitor.

Study visit 16 (Week 52±2 days): Patients will be asked to come in the fasting state, and to bring in used study pens and tablets for drug accountability. Vitals will be taken. Daily blood sugars and urinary ketones measurements using KetoStix will be collected and reviewed. Blood sugars and ketone bodies will be measured via a CGM/fingerstick blood sample using glucose and ketones meter (Abbott Laboratories). Insulin dose will be adjusted if needed. The CGM will be collected. An optional fat biopsy will be performed. A urine sample will be collected to evaluate ketone bodies and, in females, pregnancy status. The 24 hour urine container will be collected. A fasting blood sample will be taken

(45 mL) to evaluate study endpoints, CBC, CMP, HbA1c and C-peptide. We will ask the patients to fill out quality of life questionnaires. The patient will receive a physical. After this visit, they will be discharged from the study.

Safety phone calls will be conducted at weeks 3, 5, 8, 16, 20, 29, 35, 44 and at two weeks after the end of the study to document any side effects or other changes.

Visit #	-	1	2	3	4	5	6	7	8	9	10	11	12	13	14	15	16
Week#(±2-7 days as per text)	-8 to -4	-3	-1	0	1	2	4	13	23	25	26	27	31	39	49	51	52
Study Procedures	Screening	Run-in period		Randomization, start semaglutide							Randomize/Start dapagliflozin/ placebo						End of study
History	X																
Physical	X																X
Vitals	X	X	X	X	X	X	X	X	X	X	X	X	X	X	X	X	X
CBC	X			X				X			X		X	X			X
CMP	X			X				X			X		X	X			X
C-Peptide	X										X						X
Kidney and liver profiles	X			X				X			X		X	X			X
Pregnancy test	X	X		X			X	X	X		X		X	X	X		X
Study drug titration						X	X					X					
Dispense medications				X	X	X	X	X			X	X		X			
HbA1c	X			X				X		X				X			X
24 hour BP (optional)			X					X		X				X		X	
CGM		X	X						X	X					X	X	
Daily Fingerstick glucose review		X	X	X	X	X	X	X	X	X	X	X	X	X	X	X	X
Daily urinary ketones review											X	X	X	X	X	X	X
Insulin dose review		X	X	X	X	X	X	X	X	X	X	X	X	X	X	X	X
Ketone bodies (blood/urine)	X	X	X	X	X	X	X	X	X	X	X	X	X	X	X	X	X
Adverse Event reporting		X	X	X	X	X	X	X	X	X	X	X	X	X	X	X	X
Meal Challenge			X							X						X	
Blood endpoints				X				X			X			X			X
Fat biopsy (optional)				X							X						X
24 hr Urine				X				X			X			X			X

Quality of life questionnaires				X							X						X

Table 1: Schedule of visits and testing procedures.

Drug Blinding:
The randomizations will be performed using an Interactive Web System, hosted by University of Glasgow. Eighty-one (81) patients will be randomized as follows: participants will first be randomized to semaglutide plus usual insulin therapy or insulin therapy only (no placebo for semaglutide or other placebo injection will be provided), in a 2:1 ratio. Those who remain on semaglutide at six months will then be randomized in a 1:1 ratio to receive dapagliflozin or matched placebo. Each randomization will be stratified by study site and the second randomization will also include HbA1c stratification. The randomization schedules will be generated by a statistician with no other involvement in the trial, using the method of randomized permuted blocks. Only those staff responsible for the maintenance of the randomization system will have access to the randomization sequences during the trial. The first randomization (which will be revealed to the researcher at the point of randomization) will use random block sizes to reduce predictability. Researchers at study sites will have no knowledge of the next allocation to be issued. The second randomization (which will be double-blind) will use a different. At the point of randomization, the researcher will be given a medication pack ID number, but will not know whether that medication pack contains Dapagliflozin or placebo. For each randomization, a web-based randomization system (developed and maintained by the Robertson Centre for Biostatistics) will be used. The system will include an emergency unblinding facility for the dapagliflozin/placebo randomization
Astra Zeneca Inc. will supply dapagliflozin and matching placebo tablets in unlabeled and identical packaging; semaglutide pens will be obtained commercially and will be used in an open label fashion.

Breaking of the study blind will be performed only: (i) for SUSARs; and, (ii) where knowledge of the treatment is considered by local health personnel absolutely necessary for further management of the patient. A central unblinding facility based at the Robertson Centre for Biostatistics, University of Glasgow, will be available by telephone. Notification of all unblinding will be sent to the Chief Investigator

During the course of the trial there will be no attempt to identify group assignment for any participant (except for unblinding in the case of safety concerns) and the investigators will execute the protocol in the manner it was approved independent of the group assignment.

Drugs will be stored in a locked cabinet or locked temperature controlled refrigerator at 4C at the research facility of the Diabetes and Endocrinology Center of WNY. IND application for use of all drugs (dapagliflozin and semaglutide) in type 1 diabetes will be submitted to FDA and no study procedure will start before all local and federal approvals are obtained.

Clinical and Laboratory Procedures

1) **CMP, CBC, HbA1c, , glucose, glucagon, GLP-1 and FFA measurements:** Blood CMP, CBC, HbA1c, and glucose will be measured through Quest diagnostic (US site) and NHS labs (UK site). Free fatty acid levels will be measured by a colorimetric assay (Wako, Richmond, VA). GLP-1 and glucagon will be measured from samples collected in protease and DPP-IV inhibitors by ELISA kits from Millipore.
2) **Meal Challenge test:** In order to assess the postprandial changes induced by dapagliflozin, semaglutide or the combination of both compared to insulin only, a meal challenge will be performed before starting treatments, at six months and at end of study using a 910 Calorie High fat High carbohydrate meal (HFHC) as in several of our previous studies (HFHC meal includes an egg muffin sandwich, a sausage muffin sandwich and two hash browns which contain 88g carbohydrate, 51 g fat (33% saturated) and 34 g protein). Participants will be asked to attend fasting prior to breakfast insulin bolus; basal insulin will continue during the duration of the meal, however patients will not bolus until the 3 hour mark unless blood glucose values reach above 400 mg/dL. Sequential blood samples will be obtained at 0, 15, 30, 45, 60, 90, 120, 150 and 180 min after consuming the meal. Samples at 15, 30, 45, 90 and 150 min will be 5ml while those at 0, 60, 120 and 180min will be 30 ml (total volume=145 ml). Spot urine will be collected at baseline, 1 and 3hr. Blood will be collected from an indwelling intravenous canula in a superficial forearm vein.
3) **Optional 24 hour Ambulatory BP monitoring:** Participants will be instructed on the use of 24 hour ambulatory BP monitoring device. This device will measure BP and pulse every 30 minutes for 24 hours. In addition to the scheduled measurements there will be an option for additional manual measurements if systolic blood pressure is >160 or <90 mmHg.
4) **Optional Fat aspiration procedure:** Abdominal subcutaneous fat tissue aspiration will be performed at a 10 cm distance from umbilicus. Participants will be asked not to take aspirin or NSAIDS in the previous 72 hours - if they have, the procedure will not be done. Skin will be prepared with povidone-iodine (Betadine) and alcohol. A sterile drape will be placed around the appropriate area. 3cc of 1% lidocaine will then be administered subcutaneously (the dose will not exceed 4.5mg/kg body weight). After adequate anesthesia has been achieved, aspiration of fat tissue will then be with a 3-holed canula (Tulip Instrumentation, length: 15cm, diameter: 2.1mm) fixed to a 10mL syringe. After getting adequate fat tissue (500mg-3g), the puncture site will be pressed for at least ten minutes before the patient rises up from supine position (to minimize bruising). Participants will then be discharged home. Adipose tissue will be centrifuged to remove blood and fluid contaminants. Upper adipose tissue will be collected into a separate sterile tube, washed twice with cold sterile Phosphate Buffered Saline (PBS) and centrifuged to remove the PBS. The adipose tissue sample will be

weighed and approximately 500 mg transferred to a separate tube for analysis. Total RNA, nuclear extracts and total cell lysates will be prepared.

5) **Questionnaires:** Participants will be asked to complete the standard Diabetes Specific Quality of Life Scale (DSQOLS) and Problem Areas In Diabetes survey (PAID). Questionnaires attached at the end of this plan.

6) **MNC isolation (for future ancillary studies):** Blood samples will be collected in Na-EDTA as an anticoagulant. Three and a half mL of anticoagulated blood sample are carefully layered over 3.5 mL of PMN medium (Cedarlane Laboratories, Hornby, ON) and then centrifuged. At the end of this process, two bands separate out at the top of the RBC pellet. The top band consists of MNC, while the bottom consists of PMN. The MNC band is harvested and washed twice with Hank's balanced salt solution (HBSS). This method provides yields greater than 95% pure PMN and MNC suspensions.

7) **NFκB DNA binding activity (for future ancillary studies):** Nuclear NFκB DNA binding activity is measured by EMSA. Nuclear extract is prepared from MNC and adipose tissue by high salt extraction as described by Andrews et al (17). The specificity of the bands is confirmed by supershifting these bands with specific antibodies against Rel-A (p65) and p50 (Santa Cruz Biotechnology, CA) and by competition with cold oligonucleotides. DNA binding activity of NFκB will be adjusted to Oct1 DNA binding activity to correct for any variable.

8) **Plasma concentrations of inflammatory mediators (for future ancillary studies):** Concentrations of TNFα, IL-1β, IL-18, adiponectin, IL-1RA will be measured in plasma using ELISA kits (R&D Systems, MN). CRP and SAA concentrations will be measured using an ELISA kit from Alpha Diagnostics International Inc. (San Antonio, TX).

9) **Plasma vasodilators and vasoconstrictors (for future ancillary studies):** Plasma ANP, NT-proBNP, b-NP, cGMP, cAMP, angiotensinogen, renin and angiotensin II concentrations will be measured by commercially available ELISA kits.

10) **15-isoprostane F_{2t}** (also known as 8-epi-PGF2α or 8-iso-PGF2α) **(for future ancillary studies):** will be measured in urine samples using an ELISA kit from Oxford Biomedical Research (Oxford, MI).

11) **Quantification of mRNA in MNC and adipose tissue (for future ancillary studies).** Total RNA is isolated from MNC and adipose tissue using commercially available RNAqueous®-4PCR Kit (Ambion, Austin, TX). The quality and quantity of the isolated RNA is determined before using the RNA. Real Time RT-PCR is performed using Cepheid Smart Cycler (Sunnyvale, CA) in which 2 µL cDNA, 10 µL Sybergreen Master mix (Qiagen, CA) along with 0.5 µL of 20 µM gene specific primers for

IL-1β, IL-RA, TLR-4, TNFα, IL-12, SOCS-3 and JNK-1 (Life Technologies, MD) are used. The specificity and the size of the PCR products are tested by adding a melt curve at the end of the amplifications and by running it on a 2% agarose gel. All values are normalized to 18S expression and β-actin.

10.2 *Describe what data will be collected.*

NOTE: For studies with multiple data collection points or long-term follow up, consider the addition of a schedule or table in your response.

Response:

Data will be collected as described in table 1 above. The following aspects of the study will be performed and data collected and reported by patients:

1- A CGM device will be provided for use for 3 20-days periods. Detailed instructions on changing the sensor at 10 days will be provided. Patients will be instructed to wear their CGM constantly during the assigned study periods. They will be asked to keep a diary of their food intake to measure their carbohydrate intake during the CGM period. Insulin doses will be titrated aiming for blood glucose between 90-180 mg/dl while avoiding hypoglycemia (<70 mg/dl) or hyperglycemia (>250 mg/dl).
2- Participants who agree to participate in the 24-hr ambulatory blood pressure monitoring will be provided with a 24-hour ambulatory BP monitor to be worn for 24 hours during week -1, 13, 25, 39 and 51.
3- All participants will be asked to monitor capillary blood glucose by finger stick before each meal and at bedtime.
4- For the entire duration of the study, the patients will maintain a diary to record any hypoglycemia and other untoward side effects e.g. nausea, change in appetite.
5- Starting at the 6 months mark, participants will be asked to perform a daily urinary test of ketone bodies using KetoStix and record all outcomes. If results are moderate to large, they will be instructed to repeat the test and to call the research center or their doctor directly if results are confirmed. Patients with dual glucose and ketones meters will be instructed to record and report their blood ketones as well. If blood ketones are above 0.6mM, they should repeat the test and contact the research center of their doctor if levels are confirmed.

10.3 *List any instruments or measurement tools used to collect data (e.g. questionnaire, interview guide, validated instrument, data collection form).*

Include copies of these documents with your submission.

Response: Source documents will be used to collect patient information. The CGM or insulin pump will be used to collect glucose and insulin data. Glucose and ketone meter will be used to measure glucose and ketone in blood. KetoStix for measuring ketones in urine. Blood pressure monitor will be used for 24 hour blood pressure reading. Glucose, food and urinary ketones diaries will be provided for patients to record their results.

10.4 *Describe any source records that will be used to collect data about subjects (e.g. school records, electronic medical records).*

Response: electronic medical records and research files.

*10.5 Indicate whether or not **individual** subject results, such as results of investigational diagnostic tests, genetic tests, or incidental findings will be shared with subjects or others (e.g., the subject's primary care physician) and if so, describe how these will be shared.*

Response: Individual participant lab results will be disclosed to the participant upon their request. If the participant requests documentation be shared with another physician, physician office or hospital the participant must come to the research center to collect said documentation or the documentation can be mailed to their given home address.

Patients will be assigned a unique study number. All identifiable personal information will be stored locally in locked filing cabinets. Data entered on the electronic Case Report Form will only be identified by the unique study number. Thus, no personal information will be shared with the data center (Robertson Centre for Biostatistics, University of Glasgow, UK).

*10.6 Indicate whether or not **study** results will be shared with subjects or others, and if so, describe how these will be shared.*

Response: Not Applicable. Study results will not be shared with the subjects. However, unidentifiable study results could be published in the form of a manuscript or abstract and will be reported to JDRF, Astra Zeneca, and to clinicaltrials.gov and other regulatory agencies including FDA.

11.0 Study Timelines

11.1 Describe the anticipated duration needed to enroll all study subjects.

Response: Accrual is expected to be at the rate of 2 subjects/month/site, thus we should complete enrollment of 57 patients/site in approximately 26-30 months. We expect to complete all study clinical activities in 48 months.

11.2 Describe the duration of an individual subject's participation in the study. Include length of study visits, and overall study follow-up time.

Response: 55 Weeks. Study visits will be 60-90 minutes long except for visits 2, 9 and 15 which will be about 4hr long each

11.3 Describe the estimated duration for the investigators to complete this study (i.e. all data is collected and all analyses have been completed).

Response: 60 Months

12.0 Setting

12.1 Describe all facilities/sites where you will be conducting research procedures. Include a description of the security and privacy of the facilities (e.g. locked facility, limited access, privacy barriers). Facility, department, and type of room are relevant. Do not abbreviate facility names.

NOTE: Examples of acceptable response may be: "A classroom setting in the Department of Psychology equipped with a computer with relevant survey administration software," "The angiogram suite at Buffalo General Medical Center, a fully accredited tertiary care institution within New York State with badge access," or, "Community Center meeting hall."

Response:

1) Research will be conducted at the Diabetes Endocrinology Research Center of WNY, located at *1000 Youngs Road, Suite 105, Williamsville NY 14221* and at the CTRC located in 875 Ellicott St. Buffalo NY14203.The Diabetes Research Center has facilities and exam rooms available for insulin pump download, CGM device download, meal and infusion studies and presence of study coordinator and registered nurse for data collection and blood work at all times. One of the investigators will be available at all times to address patients' related issues. CTRC location is a fully equipped laboratory with Equipment include ultra-low freezers for sample storage, centrifuges, microscopes for sample preparation, infusion pumps, ELISA, PCR and immunoblotting instrumentation.

2) Research will also be conducted at the NHS Greater Glasgow and Clyde Clinical Research Facility (CRF, Glasgow Royal Infirmary, UK and at the Diabetes Centre (Clinic D), Stobhill Ambulatory Care Hospital, Glasgow, UK. There are facilities and exam rooms available for insulin pump download, CGM device download, meal and infusion studies and presence of research nursing staff for data collection and blood work at all times. One of the investigators will be available at all times to address patients' related issues. The CRF has equipment for sample preparation, infusion pumps, ultra-low freezers for sample storage and centrifuges. ELISA, PCR and immunoblotting facilities are available in adjacent NHS and University facilities.

12.2 For research conducted outside of UB and its affiliates, describe:

- *Site-specific regulations or customs affecting the research*
- *Local scientific and ethical review structure*

NOTE: This question is referring to UB affiliated research taking place outside UB, i.e. research conducted in the community, school-based research, international research, etc. It is not referring to multi-site research. UB affiliated institutions include Kaleida Health, ECMC, and Roswell Park Cancer Institute.

Response:

☒ **N/A:** This study is not conducted outside of UB or its affiliates.

13.0 Community-Based Participatory Research

13.1 Describe involvement of the community in the design and conduct of the research.

NOTE: Community-Based Participatory Research (CBPR) is a collaborative approach to research that equitably involves all partners in the research process and recognizes the unique strengths that each brings. CBPR begins with a research topic of importance to the community, has the aim of combining knowledge with action and achieving social change to improve health outcomes and eliminate health disparities.

Response:

☒ **N/A:** This study does not utilize CBPR.

13.2 Describe the composition and involvement of a community advisory board.

Response:

☒ **N/A:** This study does not have a community advisory board.

14.0 Resources and Qualifications

14.1 Describe the qualifications (e.g., education, training, experience, expertise, or certifications) of the Principal Investigator ***and*** *staff to perform the research. When applicable describe their knowledge of the local study sites, culture, and society. Provide enough information to convince the IRB that you have qualified staff for the proposed research.*

NOTE: If you specify a person by name, a change to that person will require prior approval by the IRB. If you specify a person by role (e.g., coordinator, research assistant, co-investigator, or pharmacist), a change to that person will not usually require prior approval by the IRB, provided that the person meets the qualifications described to fulfill their roles.

Response: All study personnel are educated, trained, and licensed as required for their delegated role in this study. All study personnel have also received the required university training and will be trained by the PI before the study starts. The PIs, CO-investigators and research team have conducted similar studies in this patient population using similar agents and have the expertise to execute the study protocol and manage any adverse events.

Describe other resources available to conduct the research.

14.2 Describe the time and effort that the Principal Investigator and research staff will devote to conducting and completing the research.

NOTE: Examples include the percentage of Full Time Equivalents (FTE), hours per week. The question will elicit whether there are appropriate resources to conduct the research.

Response: The principal investigator supervises the research project and weekly research meetings are conducted to discuss the recruitment rate, resolve and discuss issues related to the conduct, safety, analysis of the study and related publications. PI is expected to spend 15% of his academic time on this research. The co-investigators, study coordinator and research staff provide coverage to the research related activity for 365 days a year.

14.3 Describe the availability of medical or psychological resources that subjects might need as a result of anticipated consequences of the human research, if applicable.

NOTE: One example includes: on-call availability of a counselor or psychologist for a study that screens subjects for depression.

Response: Available medical literature will be provided as deemed appropriate or requested by patient through UB libraries, PubMed, Google scholar as all the investigators have access to medical literature through listed resources above

The patient will also have access to physician (Investigators and Co-Investigators) who will be available to address any adverse effects or other questions during the course of the study who will be available to address any adverse effects or other questions during the course of the study

14.4 Describe your process to ensure that all persons assisting with the research are adequately informed about the protocol, the research procedures, and their duties and functions.

Response: Before beginning of enrollment all research staff will be asked to review the protocol and a training session will be conducted to provide protocol details, explain technical points and answer staff questions. All training session will have a sign in sheet to document such training. Continuous education through training meetings, conferences and discussions will continue through the study period and when revision are made to protocol.

15.0 Other Approvals

15.1 Describe any approvals that will be obtained prior to commencing the research (e.g., school, external site, funding agency, laboratory, radiation safety, or biosafety).

Response: FDA IND filling is required.

☐ **N/A:** This study does not require any other approvals.

16.0 Provisions to Protect the Privacy Interests of Subjects

16.1 Describe how you will protect subjects' privacy interests during the course of this research.

NOTE: Privacy refers to an individual's right to control access to him or herself. Privacy applies to the person. Confidentiality refers to how data collected about individuals for the research will be protected by the researcher from release. Confidentiality applies to the data.

Examples of appropriate responses include: "participant only meets with a study coordinator in a classroom setting where no one can overhear", or "the participant is reminded that they are free to refuse to answer any questions that they do not feel comfortable answering."

Response: Our clinical providers involved in the study will identify potential patients for recruitment from the Diabetes-Endocrinology Center of WNY Clinics according to the inclusions and exclusion criteria and through advertisements. Patient who qualify will be asked in private during their one on one consultation time with the physician if they wish to participate in the research study. If the patient agrees, the research coordinator will contact them privately for a possible telephone screening and ask if time is appropriate for this discussion. The patients who call for potential participation in the study due to advertisement flyers will be screened over the phone with the research coordinator, using our telephone screening form.

When the patient is being seen at our clinics for the first time they sign the "Consent to use and disclosure of protected health information" form which clearly states that their protected health information (PHI) can be used for review in preparation for possible research. Additionally, a partial HIPAA waiver will be obtained to access medical records when screening for patients.

If the patient passes the telephone screening, they will be asked to make an appointment to review and sign the consent. Patient will do this in a private room in the research unit and will be allowed to discuss the consent in detail with the research coordinator and or study doctor. Patient will be notified that it is completely voluntary to participate in the research study and can withdraw at any time.

We will not be accessing any medical information of the patients for whom the services are not provided by our clinic providers before partial HIPAA is obtained or IC is signed.

16.2 Indicate how the research team is permitted to access any sources of information about the subjects.

*NOTE: Examples of appropriate responses include: school permission for review of records, consent of the subject, HIPAA waiver. This question **does apply** to records reviews.*

Response: Consent of the subject and partial HIPAA waiver.

17.0 Data Management and Analysis

17.1 Describe the data analysis plan, including any statistical procedures. This section applies to both quantitative and qualitative analysis.

Response:

Data (e.g. date of diagnosis, past medical history, drug treatment) extracted from local clinical records will be entered on to the eCRF under the study-specific coded identifier according to structured questions as stipulated in the Study Protocol. Biological samples (blood, urine and adipose tissue aspirates) will be collected from the study participants at the study visits according to the Schedule of Assessments in the Study Protocol.

Measures relating to safety and routine care of the participants (e.g., biochemical measures of glycemic and lipidemic control, kidney function/ fluid balance, hepatic function, hematological variables) will be analysed at local clinical laboratories in order that they are available to study nurses and physicians. The confidentiality of these data will be secure, meeting or exceeding the regulations of the relevant Ethical Review Committees/ Institutional Review Boards. Samples for measurement of key study outcomes will also be measured at the central laboratory at the University of Buffalo. Blood, urine and other samples will be aliquoted into cryovials at site for freezing and storage. Electronic stored material (e.g., patient records, data from blood and urine samples, and data from Continuous Glucose Monitoring) will only be stored using the study specific coded identifier. Data will not be identifiable at the participant level without additional access to the code. The code will be available only to the investigators and the personnel performing the study. Access to any data held electronically will require study specific log-in codes. These are managed and logged by the Robertson Centre for Biostatistics, University of Glasgow, UK

Statistical plan. The primary objective of the proposed research is to evaluate the effect of addition of dapagliflozin to semaglutide on a background of insulin treatment in type 1 diabetes patients on HbA1c at six months following start of dapagliflozin. The similarities between the study groups, baseline values for subject's demographics and study endpoints will be compared using appropriate parametric tests. Transformations of the data in order to meet statistical assumptions may be considered. The primary endpoint of the study is to detect a difference in mean HbA1c change after six months of dapagliflozin or placebo addition to semaglutide and insulin treatment. Statistical analysis will be carried out using t-tests to detect difference between groups or the Wilcoxon test for paired data. Tests will be two-sided and tested at the 0.05 level. The results will

be summarized as mean±SE. All randomized patients will be included in the analysis of the primary end point based on an Intent-to-treat model.

The secondary endpoints will be to compare changes in HbA1c between each of the triple therapy and dual therapy groups compared to standard of care group, mean weekly glucose concentrations, percent time spent in different glycemic thresholds, variability as measured by standard deviation, insulin dose, body weight, carbohydrate intake, blood pressure, and 24-hour blood pressure and QOL changes among the groups. A reduction in the glucose and glucagon AUC following the meal will constitute another secondary end point in this setting, ANCOVA analysis will be used. Since the secondary end-points are pre-specified and may as well be correlated, multiplicity control for multiple tests across secondary endpoints may not be necessary. However, Holm-Bonferroni correction for multiplicity testing will be implemented if additional endpoints are tested or if the pre-specified secondary end-points are found to be not correlated.

17.2 *If applicable, provide a power analysis.*

NOTE: This may not apply to certain types of studies, including chart/records reviews, survey studies, or observational studies. This question is asked to elicit whether the investigator has an adequate sample size to achieve the study objectives and justify a conclusion.

Response:

Sample size determination is based in part on our current data showing that in type 1 diabetes, treatment with a GLP-1 agonist, liraglutide (1.2 and 1.8mg doses) for 3 months leads to HbA1c suppression by an average of 0.6% (SD= 0.5%). Dapagliflozin was recently shown to reduce HbA1c by 0.45% in type 1 diabetes patients (13). Additionally, empagliflozin treatment for 8 weeks was shown to reduce HbA1c by 0.4% in type 1 diabetics (20, 21) and our current data showing additional improvement in HbA1c by an additional 0.66% at 12 weeks when dapagliflozin was added to liraglutide and insulin therapy. Conservatively estimating that the combination therapy will suppress HbA1c by 0.5% (SD =<0.53%) compared to semaglutide alone (t-test), a sample size of 3827 (22-23 evaluable patients) in each group will be sufficient to provide adequate power ($\beta = 0.2$) to detect a statistically significant difference ($\alpha = 0.05$) taking in consideration a 15% dropout rate.

17.3 *Describe any procedures that will be used for quality control of collected data.*

Response:

An electronic case report form (e-CRF) will be used to collect study data at each site. The e-CRF will be developed by the study Data Centre at the Robertson Centre for Biostatistics, University of Glasgow. Access to the e-CRF will be restricted, with only authorised site-specific personnel able to make entries or amendments to

their patients' data. It is the investigator's responsibility to ensure completion and to review and approve all data captured in the e-CRF.

Data will be validated at the point of entry into the e-CRF and at regular intervals during the study. Data discrepancies will be flagged to the study site coordinator and any data changes will be recorded in order to maintain a complete audit trail (reason for change, date change made, who made change).

The Robertson Centre for Biostatistics at the University of Glasgow will be responsible for collating and cleaning the data for the study. The Robertson Centre will also be responsible for data back-up and security. This center will also manage the electronic reporting of SUSARS on behalf of the sponsor.

To enable evaluations and/or audits from regulatory authorities, the investigators agree to keep records, including the identity of all participating subjects (sufficient information to link records, all original signed informed consent forms serious adverse event forms, source documents, and detailed records of treatment disposition. The records should be retained by the study country coordinators and investigator according to ICH GCP, local regulations, or as specified in the Clinical Study Agreement, whichever is longer.

Data from the study will be stored by the Chief Investigator for a minimum of 10 years. A final report of the study will be provided to the FDA and the MHRA as per requirements.

- No interim analysis is planned for efficacy so termination of study will be related to safety. Significant increases in hypoglycemic or DKA events as reviewed by DSMB might trigger termination or modification of study.
- Last observation carry forward will be used to fill missing data for the primary end point and some secondary end points. Missing data will not be filled for other endpoints.
- Any deviation(s) from the original statistical plan will be described and justified in the protocol and/or in the final report.
- All subjects entered into treatment will analyzed for primary end point on ITT bases. For secondary endpoints, analysis of completed and evaluable patients will performed per-protocol.

18.0 Confidentiality

A. Confidentiality of Study Data

Describe the local procedures for maintenance of confidentiality of ***study data and any records that will be reviewed for data collection****.*

18.1 A. Where and how will all data and records be stored? Include information about: password protection, encryption, physical controls,

authorization of access, and separation of identifiers and data, as applicable. Include physical (e.g. paper) ***and*** *electronic files.*

Response: All data records will be stored on protected computers with unique passwords issued for each of the study team members and or in locked cabinets within the research center. These files will only be accessible by authorized study personnel.

The TriNetX/i2b2 data will be protected by only accessing identifiable contact information remotely on IHI's "virtual machine", so the data never leaves their secure encrypted server. Recruitment letters will be generated in a mail merge behind the firewall on IHI's virtual machine. No patient data from TriNetX/i2b2 will be removed from the IHI server or saved in other locations.

18.2 A. How long will the data be stored?

Response: De-identified data storage has no expiration date and will be stored for a minimum of 7 years. The researchers may continue to rely on this for future use in research study.

18.3 A. Who will have access to the data?

Response: Those physicians, nurses, and laboratory staff that are on all documentation for the study will have access to the data.

18.4 A. Who is responsible for receipt or transmission of the data?

Response: Those physicians, nurses, and laboratory staff that are on all documentation for the study will have access to the data and can handle transfer of data. Study data will be transferred electronically (through eCRFs) to Robertson Centre for Biostatistics, University of Glasgow, UK

18.5 A. How will the data be transported?

Response: All data are stored at locally and will be transferred to Robertson Centre for Biostatistics, University of Glasgow, UK for data management and analysis. The transfer will be done through electronic CRFs processed on secure network.

Data will also be transported unless when is being archived. At that point files will be transferred to Iron Mountain for storage and archiving.

B. Confidentiality of Study Specimens

Describe the local procedures for maintenance of confidentiality of ***study specimens****.*

☐ **N/A:** No specimens will be collected or analyzed in this research. *(Skip to Section 19.0)*

18.6 B. Where and how will all specimens be stored? Include information about: physical controls, authorization of access, and labeling of specimens, as applicable.

Response: The specimens will be stored in the laboratory located at *1000 Youngs Road, Suite 105, Williamsville NY 14221*and at the CTRC located in 875 Ellicott St. Buffalo NY14203. Samples will be stored in a locked -80º C freezer. All specimens collected at the Glasgow site will be stored in NHS -80º C freezers within the eCRF until shipping to Buffalo (World Courier).

18.7 B. How long will the specimens be stored?

Response: Specimens storage has no expiration date and will be stored for a minimum of 7 years. The researchers may continue to rely on this for future use in research study

18.8 B. Who will have access to the specimens?

Response: Those physicians, nurses, and laboratory staff that are on all documentation for the study will have access to the specimens

18.9 B. Who is responsible for receipt or transmission of the specimens?

Response: Those physicians, nurses, and laboratory staff that are on all documentation for the study will have access to the specimens and can handle transfer of samples. Sample/specimens will transported from University of Glasgow site to the university of Buffalo CTRC site (875 Ellicott St, Buffalo, NY 14203) for storage in -80C until analyzed.

18.10 B. How will the specimens be transported?

Response: All study samples will be centrally stored in UB CTRC site. Therefore, samples collected at the UK site will be transported for analysis and final long-term storage. Transport will be done so using dry ice in a properly labeled Styrofoam container by the laboratory technician. Agreement regarding shipping and receiving the specimens form UK site will be part of the subcontract agreement.

19.0 Provisions to Monitor the Data to Ensure the Safety of Subjects

☐ **N/A:** This study is not enrolling subjects, or is limited to records review procedures only. This section does not apply.

NOTE: Minimal risk studies may be required to monitor subject safety if the research procedures include procedures that present unique risks to subjects that require monitoring. Some examples include: exercising to exertion, or instruments that elicit suicidality or substance abuse behavior. In such cases, N/A is not an acceptable response.

19.1 Describe the plan to periodically evaluate the data collected regarding both harms and benefits to determine whether subjects remain safe.

Response:

An independent Data safety and monitoring board (DSMB) will be established for this trial. The board will consist of independent faculty members from UB and other institutions with sufficient knowledge of the field of type 1 diabetes but not involved in the study. Data Management team at Robertson Centre will periodically provide the data on which to base regular reports to the Data and Safety Monitoring Board. The actual reports will then be generated by the unblinded statistician. The DSMB looks only at safety endpoints and not at futility. The DSMB will convene every six months to review safety end points, side-effects experienced by study subjects and any adverse events reported. The site PIs, CO-Is and study coordinator(s) will also continuously monitor the data and safety of subjects enrolled in the study. The site PIs and/or the DSMB may recommend to terminate the study or specific aspects of the study if clear harm is observed with one treatment option compared to standard of care or placebo. Specific safety endpoints that will be reviewed include increases in hypoglycemic and ketosis/DKA events.

19.2 Describe what data are reviewed, including safety data, untoward events, and efficacy data.

Response:

Assessment of Efficacy

1- glycemic indices including HbA1c, post-prandial glucose excursions and glucagon. CGM data will be collected as efficacy parameter of glucose control. Total insulin dose will be also documented and analyzed.
2- body weight
3- blood pressure and use of blood pressure medications
4- QOL questionnaires.

The timing of recording of the above efficacy parameters is described in table 1.

Assessment of Safety

The following safety assessments assays and measurement will be reviewed:

1- Vitals, CBC, CMP and markers kidney and liver function will be collected at screening, during and at end of the study as in table 1.

2- Assessment of hypoglycemia: Glucose data will be reviewed from CGM and daily finger stick and during visits.
3- Assessment of ketosis: Ketones levels will be reviewed from daily urinary ketone diaries or blood ketone meters and from urine and blood at the time of each visit
4- Reports of side effects by patients.
5- Insulin dose titrations and pump downloads at each visit

The methods and timing for assessing, recording, and analyzing safety parameters.

The DSMB will convene every six months to assess the side-effects experienced by study subjects and any adverse events reported. The PIs, CO-Is and study coordinator(s) will also continuously monitor the safety of subjects enrolled in the study during their visits and during the safety phone calls.

Procedures for eliciting reports of and for recording and reporting adverse event and inter-current illnesses.

All adverse events will be reported at the time of the visit or by telephone when it occurs. Medical events that occur between the signing of the Informed Consent and the first intake of study drug will be documented in the medical history. Participants will be asked to report any adverse events in general, non-directed questioning. For each AE volunteered by the subject, the investigator will obtain all the information required to complete the adverse event page of the eCRF, in accordance with the guidelines that accompany it. All adverse events, regardless of seriousness, severity, or presumed relationship to study therapy, will be recorded using medical terminology in the source document. Whenever possible, diagnoses will be given when signs and symptoms are due to a common etiology (e.g., cough, runny nose, sneezing, sore throat, and head congestion should be reported as "upper respiratory infection). Investigators will record on the CRF their opinion concerning the relationship of the adverse event to study therapy. The principal investigators assumes responsibility for appropriate reporting of AEs to the regulatory authorities. The investigator will report these events to the appropriate Independent Ethics Committee/Institutional Review Board (IEC/IRB) in accordance with local regulations. All measures required for AE management will be recorded in the source document and reported according to the State University of New York at Buffalo instructions. Non-serious events/problems that impacted on the rights and welfare of the subjects will be reported as they occur or at the time of knowledge or notification.

Definition of adverse events

The International Conference on Harmonization (ICH) Guideline for Good Clinical Practice (GCP) E6(R1) defines an AE as:

Any untoward medical occurrence in a patient or clinical investigation patient administered a pharmaceutical product and which does not necessarily have a causal relationship with this treatment. An AE can therefore be any unfavorable and unintended sign (including an abnormal laboratory finding), symptom, or

disease temporally associated with the use of a medicinal product, whether or not considered related to the medicinal product.

The term AE is used to include both serious and non-serious AEs.

Definition of serious adverse events

A serious adverse event is an AE occurring during any study phase (i.e., screening, run-in, treatment, wash-out, follow-up), at any dose of the study drugs that fulfils one or more of the following criteria:

- Results in death
- Is immediately life-threatening
- Requires in-patient hospitalization or prolongation of existing hospitalization
- Results in persistent or significant disability or incapacity
- Is a congenital abnormality or birth defect in offspring of the patient
- Is an important medical event that may jeopardize the patient or may require medical intervention to prevent one of the outcomes listed above.
- TO THE AUTHOR: The causality of SAEs (their relationship to all study treatment/procedures) will be assessed by the investigator(s) and communicated to AstraZeneca. This is requested to be on the AE cover sheet.

The following variables are recommended to be collected for each AE:

- AE (verbatim)
- The date when the AE started and stopped
- Whether the AE is serious or not
- Action taken with regard to IPs
- Administration of treatment for the AE
- Outcome

In addition, the following variables are recommended to be collected for SAEs:

- Date the AE met criteria for SAE
- Date the Investigator became aware of the SAE
- Seriousness criteria fulfilled
- Date of hospitalization
- Date of discharge
- Probable cause of death
- Date of death
- Whether an autopsy was performed
- Causality assessment in relation to study procedure(s)
- Causality assessment in relation to other medication, as explained in Section 1.4.2.
- Description of the SAE

Reporting of serious adverse events

All SAEs will be reported, whether or not considered causally related to the investigational product, or to the study procedure(s). The reporting period for SAEs is the period immediately following the time that written informed consent is obtained throughout the treatment period and including the designated follow-up period as per the study protocol. The investigator is responsible for informing the

Ethics Committee and/or the Regulatory Authority of the SAE as per local requirements.

Note that all serious or unexpected adverse events must be reported to AstraZeneca regardless of the country where the study is conducted.

The investigator must inform the FDA, via a MedWatch form, of any serious or unexpected adverse events that occur in accordance with the reporting obligations of 21 CFR 312.32 , and will concurrently forward all such reports to AstraZeneca. A copy of the MedWatch report must be emailed to AstraZeneca (TCS vendor) at the time the event is reported to the FDA. It is the responsibility of the investigator to compile all necessary information and ensure that the FDA receives a report according to the FDA reporting requirement timelines and to ensure that these reports are also submitted to AstraZeneca at the same time.

A cover page should accompany the MedWatch form indicating the following:

- "Notification from an Investigator Sponsored Study"
- The investigator IND number assigned by the FDA
- The investigator's name and address
- The trial name/title and AstraZeneca ISS reference number (ESR-##-#####)

Investigator must also indicate, either in the SAE report or the cover page, the causality of events in relation to all study medications and if the SAE is related to disease progression.

Send SAE report and accompanying cover page by way of email to AstraZeneca's designated mailbox: AEMailboxClinicalTrialTCS@astrazeneca.com

If a non-serious AE becomes serious, this and other relevant follow-up information must also be provided to AstraZeneca and the FDA.

Serious adverse events that do not require expedited reporting to the FDA still need to be reported to AstraZeneca preferably using the MedDRA coding language for serious adverse events.

The investigators will either provide a copy of the randomization code/ code break information or unblind those SAEs which require expedited reporting

Overdose

An overdose is defined as a patient receiving a dose of IP in excess of that specified in the Investigator's Brochure, unless otherwise specified in this protocol.

Any overdose of a study patient with the investigational product, with or without associated AEs/SAEs, is required to be reported within 24 hours of knowledge of the event to AstraZeneca Patient Safety using the designated Safety e-mailbox (see Section 1.5 or contact information). If the overdose results in an AE, the AE must also be recorded as an AE. Overdose does not automatically make an AE serious, but if the consequences of the overdose are serious, for example death or hospitalization, the event is serious and must be recorded and reported as an SAE (see Section 1.3). Any specific details around overdose for combination or

comparator agents will also be provided as applicable. The investigator will use clinical judgment to treat any overdose.

Hepatic function abnormality

Hepatic function abnormality that fulfills the biochemical criteria of a potential Hy's Law case in a study patient, with or without associated clinical manifestations, is recommended to be reported as "hepatic function abnormal" within 24 hours of knowledge of the event to AstraZeneca Patient Safety using the designated Safety e-mailbox (see Section 1.3), unless a definitive underlying diagnosis for the abnormality (e.g., cholelithiasis or bile duct obstruction) that is unrelated to investigational product has been confirmed. The criteria for a potential Hy's Law case is Aspartate Aminotransferase (AST) or Alanine Aminotransferase (ALT) ≥3x Upper Limit of Normal (ULN) together with Total Bilirubin (TBL) ≥2xULN at any point during the study following the start of study medication irrespective of an increase in Alkaline Phosphatase (ALP).

Pregnancy

If a patient becomes pregnant during the course of the study, the IPs should be discontinued immediately.

Pregnancy itself is not regarded as an AE unless there is a suspicion that the IP under study may have interfered with the effectiveness of a contraceptive medication. Congenital abnormalities or birth defects and spontaneous miscarriages should be reported and handled as SAEs. Elective abortions without complications should not be handled as AEs. The outcome of all pregnancies (spontaneous miscarriage, elective termination, ectopic pregnancy, normal birth, or congenital abnormality) should be followed up and documented even if the patient was discontinued from the study.

If any pregnancy occurs in the course of the study, then the investigator should inform the appropriate AstraZeneca representatives within 1 day, i.e., immediately, but no later than 24 hours of when he or she becomes aware of it.

The investigator will ensure that all relevant information is provided to the AstraZeneca Patient Safety data entry site within 1 to 5 calendar days for SAEs and within 30 days for all other pregnancies.

The same timelines apply when outcome information is available.

19.3 Describe any safety endpoints.

Response: These specific Safety endpoints will be collected:

1- Frequency and severity of hypoglycemia: Hypoglycemia will be assessed throughout the study via multiple daily finger stick glucose measurements, CGM data collected 3 times (about 3 weeks each) during the study and from patient reports during visits and phone calls. Hypoglycemic events will be characterized as in the experts consensus published in *Diabetes Care (2017 Dec; 40(12): 1622-1630):*

- Level 1: Glucose < 70 mg/dL (3.9 mmol/L) and Glucose ≥ 54 mg/dL (3.0 mmol/L)
- Level 2: Glucose < 54 mg/dL (3.0 mmol/L)

• Level 3: A severe event characterized by altered mental and/or physical status requiring assistance.

2- Ketones levels: Urinary ketones measured daily (and any blood ketones) at home and logged in the ketones logbook. Blood and urinary ketones measured during each visit. Ketone levels will be characterized as normal, ketosis and severe ketosis:
-Normal blood ketones <0.6mM or urinary ketones of trace to small (≥+1)
-Ketosis: Blood ketones >0.6mM <2.5mM or urinary ketones of moderate to high (+2 and +3)
-Severe ketosis Blood ketones >2.5mM or urinary ketones of too high (+4)
3- Diabetes ketoacidosis episodes: any confirmed DKA episode (regardless of hospitalization) defined as:
• Elevated serum or urine ketones (greater than the upper limit of the normal range), and
• Serum bicarbonate < 15 mmol/L or Blood pH < 7.3

4- Low blood pressure: <90mmHg SBP and <50 mmHg DBP): Patients will be instructed to measure blood pressure daily and it will be will be assessed in each visit.
5- Nausea, vomiting and other GI side effects. Data will be collected by patients' safety reports, during visits and phone calls.
6- Genital mycotic infections or UTIs
7- Changes in liver and kidney function: CMP and kidney and liver profiles will be assessed as indicated in table 1.

19.4 Describe how the safety information will be collected (e.g., with case report forms, at study visits, by telephone calls with participants).

Response: The safety information will be collected at the time of the visit, and or during preplanned telephone calls with the participant or patient self-reporting (including glucose, food intake and ketones diaries). Data will be entered into eCRFs

19.5 Describe the frequency of safety data collection.

Response: Safety data collection will be done at all study visits and during preplanned phone calls as in table 1. The patient, however, will be asked to report any adverse event or safety related information via phone as soon as it occurs and it will be reviewed the same day.

19.6 Describe who will review the safety data.

Response: DSMB established for this study will review unblended safety data provided by the data management system every 6 months. Additionally, the PIs and coinvestigators will review safety data (listed above) at the completion of each visits by each subject and review any report of side effects or adverse events as they are presented by the patients. The investigators will also review collective blinded

data every 3 months to assess the safety and any potential risks to the participants. Furthermore the investigators will assess other risks including the physical, psychological, social, legal and economic harm to these patients. The investigators will carefully watch for any invasion of privacy and breach of confidentiality. The principal investigator will be sharing the results of safety analysis of the study and serious adverse events with the local IRB, DSMB, JDRF, AZ and the FDA. The study groups will remain blinded. If there are a serious safety concerns or an emergency, then, and based on principal investigator discretion, study randomization center in Glasgow University or the unblended statistician will provide information about group assignment of the patient(s) involved. Un-blinded data will be reviewed by the principal investigator and co-investigators and decision to complete, withdraw or adjust dose of drug(s) will be made, documented and shared with IRB and DSMB.

19.7 Describe the frequency or periodicity of review of cumulative safety data.

Response: Safety data will be reviewed every 6 months by DSMB (un-blinded), every 3 months by PIs and CO-Is (blinded) and at every visit by study team.

19.8 Describe the statistical tests for analyzing the safety data to determine whether harm is occurring.

Response: N/A

19.9 Describe any conditions that trigger an immediate suspension of the research.

Response: No interim analysis is planned for efficacy so termination of study will be related to safety. Significant increases in severe hypoglycemic or DKA events as reviewed by DSMB are possible causes that might trigger termination of study.

20.0 Withdrawal of Subjects

☐ **N/A:** This study is not enrolling subjects. This section does not apply.

*20.1 Describe **anticipated** circumstances under which subjects may be withdrawn from the research without their consent.*

1. The site PI can stop investigational study treatment or remove participants from the trial without participants approval if for any reason he/she feels is appropriate, including: severe side effect, injury or medical condition which may place the patient at risk of further complications if he/she continue to participate, failure to take the

medication as instructed, failure to keep scheduled appointments, cancellation of the study by the sponsor, IRB, FDA or other administrative reasons.

2. If any of the participants become pregnant during the period of study, they will discontinue study treatment.
3. Participants who experience persistent severe nausea or vomiting after starting semaglutide which is not managed by dose reduction will discontinue the investigational study treatment. Those with mild or moderate nausea will continue for at least one more week. If they continue to experience significant nausea (defined as interfering with normal activities), dose will be reduced. If they continue to have these side effects, they will discontinue study medication(s).
4. Persistent weight loss or failure to maintain weight if subject has lost >15% or BMI is < 22 kg/m2 in spite of down-titration of semaglutide and/or dapagliflozin.
5. Developing other conditions included in the exclusion criteria
6. Change in method of insulin delivery between insulin pumps to MDI or vice versa during the study unless pump treatment interruption is temporary for a period of at most 2 weeks. Also, patients who upgrade to the new “artificial pancreas” pump (Medtronic’s MiniMed 670G hybrid) during the study will be withdrawn.
7. Hypoglycemia: Two episodes of severe hypoglycemia (requiring others help).
8. Ketosis (with no other identifiable precipitating factors): Persistent (1week) asymptomatic or symptomatic and unmanaged (using STICH protocol) blood ketone levels >1.5 mM (or ≥+2 in urine).
9. Persistent (>1 week) total insulin dose reduction of >20% from baseline AND total insulin dose <0.4U/Kg.
10. DKA: Two episode of DKA (with no identifiable precipitating factors) as defined by the expert consensus statement (*Diabetes Care (2017 Dec; 40(12): 1622-1630)*)
11. Two episodes of UTI.
12. Two episodes of mycotic genital infection.
13. Significant persistent change in eGFR > 15 ml/min
14. Pancreatitis: One episode of pancreatitis.
15. Other persistent side effects specific to study drugs

20.2 Describe any procedures for orderly termination.

NOTE: Examples may include return of study drug, exit interview with clinician. Include whether additional follow up is recommended for safety reasons for physical or emotional health.

Response: Participation in this study is voluntary. Participants have the right to refuse to participate or to withdraw from participation at any time for any reason. Refusal to participate or to withdraw from the study will involve no penalty or loss of entitled benefits, nor affect ongoing medical care. The site PI can stop investigational study treatment or remove participants from the trial without participants approval. If a patient withdraws consent and decide not to participate in the study, she/he will be asked to undergo a final study visit for his/her safety. If a subject withdraws from the research, the data collected to that point will be used toward the research finding. If applicable the subject will have to bring back any unused research drug and or devices.

20.3 Describe procedures that will be followed when subjects withdraw from the research, including retention of already collected data, and partial withdrawal from procedures with continued data collection, as applicable.

Response: We will use the data collected up to the point of stopping the study. No further information will be collected. Patients who stop study treatment and are interested in continuing in the study will be followed up until the end of the study for the primary end point and select secondary endpoints based on the Intent-To-Treat (ITT) analysis model. Withdrawn participants will not be replaced. The study sample size calculation includes an additional 15% subjects to compensate for drop out.

21.0 Risks to Subjects

21.1 List the reasonably foreseeable risks, discomforts, hazards, or inconveniences to the subjects related to their participation in the research. Consider physical, psychological, social, legal, and economic risks. Include a description of the probability, magnitude, duration, and reversibility of the risks.

NOTE: Breach of confidentiality is always a risk for identifiable subject data.

Response:

- Risks associated with intravenous (IV) line: All participants will be informed of the potential complications of an intravenous (IV) line, which include mild bruising at the site of IV line, usually resolving in a few days. They will also be informed about the possibility of infiltration of the IV line at the time of performing blood draws or during the meal challenge test in which case another IV line at a different site will be required (with risk of bruising at more than one site).
- Risks associated with 24 hour Ambulatory BP monitoring device: The BP cuff inflates every 15 minutes to record blood pressure over 24-hours. Participants will experience a pressure-like sensation over the arm where 24 hour BP device is worn at the time of the BP being measured due to the inflation of the cuff and release of this pressure at the time of deflation of the BP cuff. They will be informed to remove the cuff after 24 hours. If worn for longer periods they may experience mild bruising over the arm and some swelling, usually resolving 1-2 days after discontinuation.
- Risks associated with fat tissue aspiration: will probably lead to a bruise at the site of aspiration. The site may be mildly painful for 1-2 weeks. Participants are advised to call their study site staff if they have more severe pain or swelling at the site after the procedure. Rarely there are side effects such as low BP or heart rate and/or allergic reaction to local anesthetic including swelling of the throat.
- All subjects will be referred to semaglutide and dapagliflozin labeling safety information, and will be given a copy of the package insert of the medication. Discussion of the potential side effects and the different warning and precautions will take place at the time of informed consent. Patients will be informed they must not discontinue their insulin during the study.

- Potential risk of insulin, GLP-1 RA and SGLT-2 inhibitors use in type 1 diabetes patients is hypoglycemia. The combination therapy can possibly increase the magnitude and frequency of this risk.
- Potential side effects of GLP-1 RA and SGLT-2 inhibitors also include hypotension, nausea and other GI side effects, polydipsia, polyuria, dehydration, ketosis, and reduced glomerular filtration rate (GFR).
- In rodents, semaglutide causes dose-dependent and treatment-duration-dependent thyroid C-cell tumors at clinically relevant exposures. However, it received FDA-approval for the treatment of type 2 diabetes in 2017 following large Phase 3 trials including the cardiovascular safety trial SUSTAIN-6.
- On the basis of the above rodent studies, semaglutide is contraindicated in those with a personal or family history of medullary thyroid carcinoma and in patients with Multiple Endocrine Neoplasia syndrome type 2 (MEN 2). However, following review of human data with GLP-1RAs, FDA does not require or suggest routine monitoring of serum calcitonin/ thyroid ultrasound in people treated with semaglutide.
- Injection-site reactions: Serious injection-site reactions, with or without bumps (nodules), have happened in some people who use GLP-1RAs but are less frequent with semaglutide. Injection-site reactions have rarely required surgical intervention. Participants will be instructed to report any injection site reactions including nodules to study investigators.
- Kidney injury: GLP-1 RA might cause acute kidney injury in patients without underlying renal disease especially in patient who had experienced nausea, vomiting, diarrhea or dehydration. Dapagliflozin also has warnings and precautions against acute kidney injury and impairment in renal function.
- As mentioned above, there is a risk of ketoacidosis, as shown by some studies and by our data, when SGLT2 inhibitors are used in type 1 diabetes. In our own preliminary studies on triple therapy, we observed euglycemic ketoacidosis in participants whose insulin doses had been reduced by > 20% of baseline levels or in patients who are not adequately insulinzed. Thus, this adverse effect is largely attributable to insulinopenia as well as anti-insulin effects of SGLT2 inhibitors at the hepatic level (e.g. increased gluconeogenesis).
- The following other known adverse effects of SGLT-2 inhibitors will be discussed with participants when obtaining informed consent:

1. Risk of dizziness/ symptomatic hypotension: can occur soon after initiation due to intravascular volume contraction.
2. Impaired kidney function: As SGLT-2 inhibitors increase serum creatinine and decrease eGFR, this will be monitored closely.
3. Genital mycotic infections: SGLT-2 inhibitors increase the risk of genital mycotic infections, particularly in women. Those with a prior history are more susceptible. Following topical treatment with anti-fungal medications they rarely re-occur.
4. Bladder Cancer - Across 22 clinical studies, newly diagnosed cases of bladder cancer were reported in 10/6045 patients (0.17%) treated with Dapagliflozin and 1/3512 patient (0.03%) treated with placebo. After excluding patients in whom exposure to study drug was less than one year at the time of

diagnosis, there were 4 cases of bladder cancer with dapagliflozin and no cases with placebo i.e. too few cases to determine causality.

5. The dapagliflozin tablets and matching placebo contain lactose, which may cause discomfort in lactose-intolerant individuals.

- Severe allergic reaction (anaphylaxis) to study drugs or placebo ingredients or other interventions may accrue in rare cases.
- Excessive weight loss: Both investigation drugs are known to cause weight loss in the range of 5-10% in type 2 diabetes patients. However, the magnitude of weight loss following the combination of both drugs in type 1 diabetes patients may be significantly more. Excessive weight loss may lead to increased risk of hypoglycemia and ketosis or DKA (especially in the presence of dapagliflozin) and other unforeseen risks. Therefore, weight loss during any phase of the study will be limited to ≤15% baseline body weight or reaching a BMI of ≤22Kg/m^2.
- There might be other unforeseen risks associated with the use of these agents in type 1 diabetes and additional unforeseen risks associated with the combination therapy.
- There is always the risk of breach of confidentiality where patients' identifiable data is compromised.

21.2 Describe procedures performed to lessen the probability or magnitude of risks, including procedures being performed to monitor subjects for safety.

Response:

The following are procedures to lessen the probability and magnitude of risks listed above:

1- Hypoglycemia:

- Patients will be instructed to measure glucose at least 4 times a day (before meals and at bedtime) through-out the study and record all reading.
- Insulin dose adjustments will be done at every visit to avoid hypoglycemia. However, there will be no preemptive insulin dose reduction at start of treatment since all patients will have HbA1c >7.5% and the risk of hypoglycemia will be minimal when semaglutide is added.
- Daily fingerstick glucose, CGM and insulin pump data will be reviewed by the investigators for optimal titration of insulin dose to target glucose levels between 90-180mg/dl.
- In general, insulin dose adjustments should not be >10% from current total insulin dose (or 5% if HbA1c is <7%) or >20% from baseline total insulin dose at week 0 and then at week 26.
- Patients will be trained in CARB counting to avoid inaccurate insulin dose administration.
- Patients will be trained to recognize symptoms of hypoglycemia and how to manage it at home. These symptoms include feeling shaky, being nervous or anxious, sweating, chills and clamminess, confusion, fast heartbeat, feeling lightheaded or dizzy, hunger, nausea and color draining from the skin (pallor) among others. They will be asked to call the research office or the on call clinical services.

- When patients feel symptoms of hypoglycemia they will be asked to measure blood glucose and if <70mg/dl they will be instructed to consume carbohydrate or 4 tablets of glucose (4gm each). If after 15 min hypoglycemia continues, repeat carb intake until sugar levels are above 70 mg/dl. If sugar levels are below 55mg/dl, glucagon injection is permitted as rescue medication for severe hypoglycemia.
- Patients will be instructed to inform people who they are in frequent contact with (for example, friends, family members, and coworkers) on how to give glucagon to treat severe hypoglycemia. If patient have needed glucagon previously, it should be documented during screening visit so more emphasis on ways to prevent severe hypoglycemia in the future can be discussed.
- Patients/family will be instructed not to hesitate to call 911 if someone is unconscious and glucagon is not available or someone does not know how to use it and not to inject insulin (it will lower their blood glucose even more) or provide food or fluids (they can choke). They will be asked to document all details of the incident and report to research staff as soon as possible.
- Study medications dose will be reduced in patients who continue to have more than 2 measurements of glucose levels in the hypoglycemic range (<70mg/dl, regardless of symptoms) after insulin dose is reduced by the maximum allowed in this study.

2- Ketosis and Ketoacidosis:

- Patients will be instructed to measure ketone bodies in urine samples daily starting at 3 days before week 26 with Ketostix (Bayer) and record it in diary provided. Ketones (blood and urine) will be measured at every visit. Additionally, patients will be provided with a ketone meter and strips to measure blood ketones when needed. If ketone bodies in urine measure +1 or greater (>1.5mM in urine), they will be instructed to measure blood ketones and to repeat both the measurements after 60 min. If consecutive measurements show moderate or higher ketones in urine or blood ketones >0.6mM, the patient will be instructed to call the research center or their doctor directly. They will be monitored closely for insulin dose, carb intake, ketones and blood sugar and dapagliflozin/semaglutide can be temporarily discontinued, if necessary, for 1-2 weeks.
- Patients will be provided training to recognize the signs and symptoms of DKA and to self-test urine ketones on a PRN basis (in addition to once daily) if they develop any symptoms consistent with DKA, including nausea, vomiting, fatigue, confusion, or shortness of breath. This should be reported regardless of blood sugar levels since in type 1 diabetes on SGLT-2 inhibitors, DKA can occur even in normal glucose levels.
- Insulin dose changes should not be greater than 10% (5% if HbA1c is<7%) from current levels and not reduced by more than 20% from baseline levels at 0 weeks and then at 26 weeks.
- Patients requiring an insulin dose reduction > 20% AND their total insulin dose is <0.4U/Kg will be monitored closely for insulin dose, carb intake, ketones and blood sugar and dapagliflozin/semaglutide can be temporarily discontinued, if

necessary, for 1-2 weeks. If insulin requirements remain law, patient will be withdrawn from study.

- We shall not increase dose of dapagliflozin in patients who exhibit significant ketonuria (+2 or more) with the lower dose.
- Urinary ketones (and blood ketones if patients have a dual glucose and ketone meter) will be monitored daily and reported. Dapagliflozin will be temporarily (1 week) discontinued and STICH protocol (STop SGLT-2i, give Insulin bolus, Carbohydrate, and Hydration) activated in events of DKA or if based on investigator discretion if blood ketone levels are persistently (1 week) >1.5 mM (+2 in or more in urine). Dapagliflozin may also be temporarily stopped in clinical situations known to predispose to ketoacidosis (e.g. prolonged fasting due to acute illness or surgery). Patients will be instructed to stay hydrated and not reduce their caloric intake (especially carbohydrates) or start new dietary systems (especially ketogenic diets) during the study.
- If a patient develops symptoms of DKA or ketone levels exceed the allowed limit in this study (persistent (1 week) blood ketones >1.5 mM or +2 in or more in urine), STICH protocol (STop SGLT-2i, give Insulin bolus, Carbohydrate, and Hydration) will be activated. They could be managed for up to a week. If symptoms and ketone levels persist over 1 week, patients will be withdrawn form study. Insulin infusions may be administered by ER or hospital if patients develop DKA. They will be asked to document all details of the incident and report to research staff as soon as possible.

3- Risk of acute kidney injury or renal function impairment: CBC, CMP, including kidney function test and urinary markers will be performed at screening, during and at end of the study (table 1). Additional testing will be performed in patients who suffer from sever nausea or GI symptoms or hypovolemic and during the up titration of investigation drugs. If acute kidney injury occurs dapagliflozin will be discontinued immediately. To lessen the chance of this risk, patients will eGFR<60 ml/min/1.73m^2 will be excluded. Patients will also discontinue study medications if eGFR falls by >15 ml/min in 2 repeated measurements. Also other patients with factors that may predispose patients to acute kidney injury e.g. hypovolemia, chronic renal insufficiency, congestive heart failure will be excluded. Patients on ACEI, ARB, NSAID will be monitored closely. Dapagliflozin will be temporarily discontinued in any setting of reduced oral intake such as acute illness or fasting or fluid losses such as gastrointestinal illnesses, or excessive heat exposure.

4- Injection site reactions, mycotic infections and complications of IV and biopsy collection will also be monitored, Physical examination will be performed at screening 26, and 52 weeks visits. Safety vital assessments will be performed at every visit. Pregnancy will be tested at screening, 0, 4 13, 26, 39, and 52 weeks. Phone calls will be made for safety and compliance at least 7 time during the study. The patients, however, will be instructed to report any adverse event or safety related information via phone as soon as it occurs. All safety data will be reviewed by the PI or sub-investigators within 1-2 days of collection.

5- Epinephrine is the primary treatment for anaphylaxis with no absolute contraindication to its use. Our site and staff are trained and equipped to assist patients with such reactions. Severe conditions that are not satisfactory managed in site will be transferred to the hospital emergency department for further assessment and help.
6- Excessive weight loss: Doses of investigational drugs will be reduced in patients who experience excessive weight loss (≥15% of basal body weight or BMI ≤22Kg/m^2). Patients on semaglutide only (first 26 weeks) will be down-titrated to the lower dose. In patients on semaglutide and dapagliflozin/placebo (weeks 26-52), dose of dapagliflozin/placebo will be down-titrated to the lower dose. If weight loss continues, semaglutide dose will be down-titrated as well. Instructions to maintain a minimum of 50g daily carb intake will be also emphasized in patients with significant weight loss (>10% baseline body weight).

21.3 If applicable, indicate ***which procedures*** *may have risks to the subjects that are currently unforeseeable.*

Response:

There might be additional adverse effects of dapagliflozin not currently known or those unique to their use in Type 1 diabetes. There is no previous studies on the use of semaglutide in type 1 diabetes; therefore, additional unforeseen risk may be encountered in this study. Additionally, unforeseen risks might be associated with the combination therapy that might involve unknown drug-drug interactions.

21.4 If applicable, indicate which research procedures may have risks to an embryo or fetus should the subject be or become pregnant.

Response: Not applicable, we will not be enrolling pregnant participants or who may become pregnant or with in child baring years without signing the consent stating they will use at least two forms of birth control. If a participant becomes pregnant they will be withdrawn from the study immediately. They will be followed until term and any effects on the newborn will be documented.

21.5 If applicable, describe risks to others who are not subjects.

Response: We do not anticipate any additional risk to others not participating in this study.

22.0 Potential Benefits to Subjects

22.1 Describe the potential benefits that individual subjects may experience by taking part in the research. Include the probability, magnitude, and duration of the potential benefits. Indicate if there is no direct benefit.

NOTE: Compensation ***cannot*** *be stated as a benefit.*

Response: The investigational drugs used in this study are already approved as antidiabetic agents for treating type 2 diabetes. Both agents have demonstrated significant improvement in HbA1c, weight and blood pressure. It is our expectation that patients with type 1 diabetes will experience similar benefits compared to those who are treated with insulin alone. The magnitude and duration of the benefits and the safety profile may however differ and are the focus of this investigation.

23.0 Compensation for Research-Related Injury

☐ **N/A:** The research procedures for this study do not present risk of research related injury (e.g. survey studies, records review studies). This section does not apply.

*23.1 **If the research procedures carry a risk of research related injury,** describe the available compensation to subjects in the event that such injury should occur.*

Response: Routinely, Buffalo General Hospital, Erie County Medical Center, and/or the University at Buffalo, State University of New York, its agents, or its employees do not compensate for or provide free medical care for human subjects/participants in the event that any injury results from participation in a human research project. In the unlikely event that they become ill or injured as a direct result of participating in this study, they may receive medical care. Your study doctor will explain the treatment options to you and tell you where you can get treatment. Generally, this care will be billed to you, your insurance or other third party. Additionally, AstraZeneca shall reimburse the institution for the direct, reasonable, and necessary medical expenses incurred by the institution for the treatment of any personal injury that is a direct result of adverse drug experiences to subjects resulting directly from any manufacturing defects in the study drug to which the subjects would not have been exposed but for their participation in the study if (i) the institutional parties have complied with this agreement, the protocol and any written instructions of AstraZeneca concerning the study drug and (ii) all the requirements of informed consent have been complied with. AstraZeneca will not provide compensation for lost wages or for any other damages, expenses or losses, or for medical expenses have been covered by a subject's medical or other insurance.

23.2 Provide a copy of contract language, if any, relevant to compensation for research related injury.

*NOTE: If the contract is not yet approved at the time of this submission, submit the current version here. If the contract is later approved with **different language regarding research related injury**, you must modify your response here and submit an amendment to the IRB for review and approval.*

Response: AstraZeneca shall reimburse the institution for the direct, reasonable, and necessary medical expenses incurred by the institution for the treatment of any

personal injury that is a direct result of adverse drug experiences to subjects resulting directly from any manufacturing defects in the study drug to which the subjects would not have been exposed but for their participation in the study if (i) the institutional parties have complied with this agreement, the protocol and any written instructions of AstraZeneca concerning the study drug and (ii) all the requirements of informed consent have been complied with. AstraZeneca will not provide compensation for lost wages or for any other damages, expenses or losses, or for medical expenses have have been covered by a subject's medical or other insurance.

24.0 Economic Burden to Subjects

24.1 Describe any costs that subjects may be responsible for because of participation in the research.

NOTE: Some examples include transportation or parking.

Response: All research expenses will be covered by the study. The background use of insulin is considered as part of standard of care is the responsibility of the patients and his/her insurance. Participants will not be subjected to any other out of pocket cost.

☐ **N/A:** This study is not enrolling subjects, or is limited to records review procedures only. This section does not apply.

25.0 Compensation for Participation

25.1 Describe the amount and timing of any compensation to subjects, including monetary, course credit, or gift card compensation.

Response:
Visit schedule:

- Screen: $0
- Visit 1 (Week -3): Insulin adjustment: $10
- Visit 2 (Week -1): Meal ($75), insulin adjustment, and 24hr blood pressure monitor ($50): $125*
- Visit 3 (Week 0): 24 hr urine ($15), blood draw ($25), fat biopsy ($75), insulin adjustment, questionnaire ($10): $125
- Visit 4 (Week 1): Insulin adjustment: $10
- Visit 5 (Week 2): Insulin adjustment: $10
- Visit 6 (Week 4): Insulin adjustment: $10
- Visit 7 (Week 13): 24hr urine ($15), blood draw ($25), insulin adjustment, 24hr bp monitor ($50): $90*
- Visit 8 (Week 23): Insulin adjustment: $10
- Visit 9 (Week 25): Insulin adjustment, 24bp monitor ($50), Meal ($75): $125*
- Visit 10 (Week 26): Insulin adjustment, 24hr urine ($15), blood draw ($25), fat biopsy ($75), questionnaire ($10): $125*
- Visit 11 (Week 27): Insulin adjustment: $10
- Visit 12 (Weak 31): Insulin adjustment, blood draw: $25

- Visit 13 (Week 39): Insulin adjustment, 24hr urine ($15), blood draw ($25), 24hr bp monitor ($50): $90*
- Visit 14 (Week 49): Insulin adjustment: $10
- Visit 15 (Week 51): Insulin adjustment, 24bp monitor ($50), meal ($75): $125*
- Visit 16 (Week 52): Insulin adjustment, 24hr urine ($15), blood draw ($25), questionnaire ($10), fat biopsy ($75):$125*
  *Total compensation for all completed visits: up to $1,025.00. For those not wishing to participate in the optional blood pressure monitoring and/or fat biopsy procedure, the compensation will decrease by $250 for not completing the bp monitoring, and by yet another $225.00 for not completing the fat biopsies.

☐ **N/A:** This study is not enrolling subjects, or is limited to records review procedures only. This section does not apply.

☐ **N/A:** There is no compensation for participation. This section does not apply.

26.0 Consent Process

26.1 Indicate whether you will be obtaining consent.

NOTE: This does not refer to consent documentation, but rather whether you will be obtaining permission from subjects to participate in a research study. Consent documentation is addressed in Section 27.0.

☒ ***Yes*** *(If yes, Provide responses to each question in this Section)*
☐ ***No*** *(If no, Skip to Section 27.0)*

26.2 Describe where the consent process will take place. Include steps to maximize subjects' privacy.

Response: All participants will come to the research department to be consented. Participants will be placed in a private, screened off area and or room where they can review the consent. Participant questions and or concerns will be address with a member of the study team or research doctor if applicable. The research coordinator will discuss in length the participants requests for privacy of their PHI.

26.3 Describe how you will ensure that subjects are provided with a sufficient period of time to consider taking part in the research study.

NOTE: It is always a requirement that a prospective subject is given sufficient time to have their questions answered and consider their participation. See "SOP: Informed Consent Process for Research (HRP-090)" Sections 5.5 and 5.6.

Response: participants will be made aware that participating in research is completely voluntary, and they may withdraw at any time with no consequence to their routine clinic care. If the patients requires time to decide and or discuss partaking in a research study, the subject will be given said time.

26.4 Describe any process to ensure ongoing consent, defined as a subject's willingness to continue participation for the duration of the research study.

Response: The research coordinator and study team are available to answer any question or concerns with the patient during the duration of the research trial. At each study visit, the patient is asked a series of questions to ensure they are on task with the study visits and feel comfortable. Upon departing from their study visit, the patients are told of their next visit and given detail instruction for their next visit. If study is revised or amendment or new information becomes available about drug safety that may affect patients participation, the patient may be re-consented to ensure patient ongoing consent

26.5 Indicate whether you will be following "SOP: Informed Consent Process for Research (HRP-090)." If not, or if there are any exceptions or additional details to what is covered in the SOP, describe:

- *The role of the individuals listed in the application who are involved in the consent process*
- *The time that will be devoted to the consent discussion*
- *Steps that will be taken to minimize the possibility of coercion or undue influence*
- *Steps that will be taken to ensure the subjects' understanding*

Response:

☒ We have reviewed and will be following "SOP: Informed Consent Process for Research (HRP-090)."

Non-English Speaking Subjects

☒ **N/A:** This study will not enroll Non-English speaking subjects.
(Skip to Section 26.8)

26.6 Indicate which language(s) other than English are likely to be spoken/understood by your prospective study population or their legally authorized representatives.

NOTE: The response to this Section should correspond with your response to Section 6.4 of this protocol.

Response:

26.7 If subjects who do not speak English will be enrolled, describe the process to ensure that the oral and written information provided to those subjects will be in that language. Indicate the language that will be used by those obtaining consent.

NOTE: Guidance is provided on "SOP: Informed Consent Process for Research (HRP-090)."

Response:

Cognitively Impaired Adults

☒ **N/A**: This study will not enroll cognitively impaired adults.
(Skip to Section 26.9)

26.8 Describe the process to determine whether an individual is capable of consent.

Response:

Adults Unable to Consent

☒ **N/A**: This study will not enroll adults unable to consent.
(*Skip to Section 26.13)*

When a person is not capable of consent due to cognitive impairment, a legally authorized representative should be used to provide consent (Sections 26.9 and 26.10) ***and, where possible, assent of the individual should also be solicited*** *(Sections 26.11 and 26.12).*

26.9 Describe how you will identify a Legally Authorized Representative (LAR). Indicate that you have reviewed the "SOP: Legally Authorized Representatives, Children, and Guardians (HRP-013)" for research in New York State.

NOTE: Examples of acceptable response includes: verifying the electronic medical record to determine if an LAR is recorded.

Response:

☐

26.10 ***For research conducted outside of New York State****, provide information that describes which individuals are authorized under applicable law to consent on behalf of a prospective subject to their participation in the research. One method of obtaining this information is to have a legal counsel or authority review your protocol along with the definition of "legally authorized representative" in "SOP: Legally Authorized Representatives, Children, and Guardians (HRP-013)."*

Response:

*26.11 Describe the process for **assent of the adults**:*

- *Indicate whether assent will be obtained from all, some, or none of the subjects. If some, indicate which adults will be required to assent and which will not.*

  Response:

- *If assent will not be obtained from some or all subjects, provide an explanation of why not.*

  Response:

*26.12 Describe whether **assent of the adult** subjects will be documented and the process to document assent.*

NOTE: The IRB allows the person obtaining assent to document assent on the consent document using the "Template Consent Document (HRP-502)" Signature Block for Assent of Adults who are Legally Unable to Consent.

Response:

Subjects who are not yet Adults (Infants, Children, and Teenagers)

☒ **N/A**: This study will not enroll subjects who are not yet adults.
(Skip to Section 27.0)

*26.13 Describe the criteria that will be used to determine **whether a prospective subject has not attained the legal age for consent to treatments or procedures involved in the research** under the applicable law of the jurisdiction in which the research will be conducted **(e.g., individuals under the age of 18 years)**. For research conducted in NYS, review "SOP: Legally Authorized Representatives, Children, and Guardians (HRP-013)" to be aware of which individuals in the state meet the definition of "children."*

NOTE: Examples of acceptable responses include: verification via electronic medical record, driver's license or state-issued ID, screening questionnaire.

Response:

*26.14 **For research conducted outside of New York State**, provide information that describes which persons have not attained the legal age for consent to treatments or procedures involved the research, under the applicable law of*

the jurisdiction in which research will be conducted. One method of obtaining this information is to have a legal counsel or authority review your protocol along the definition of "children" in "SOP: Legally Authorized Representatives, Children, and Guardians (HRP-013)."

Response:

26.15 Describe whether parental permission will be obtained from:

Response: N/A

- ☐ One parent even if the other parent is alive, known, competent, reasonably available, and shares legal responsibility for the care and custody of the child.
- ☐ Both parents unless one parent is deceased, unknown, incompetent, or not reasonably available, or when only one parent has legal responsibility for the care and custody of the child.
- ☐ Parent permission will not be obtained. A waiver of parent permission is being requested.

NOTE: The requirement for parent permission is a protocol-specific determination made by the IRB based on the risk level of the research. For guidance, review the "CHECKLIST: Children (HRP-416)."

*26.16Describe whether permission will be obtained from individuals **other than parents**, and if so, who will be allowed to provide permission. Describe your procedure for determining an individual's authority to consent to the child's general medical care.*

Response:

*26.17 Indicate whether assent will be obtained from all, some, or none of the **children**. If assent will be obtained from some children, indicate which children will be required to assent.*

Response:

26.18 When assent of children is obtained, describe how it will be documented.

Response:

27.0 Waiver or Alteration of Consent Process

Consent will not be obtained, required information will not be disclosed, or the research involves deception.

☒ **N/A:** A waiver or alteration of consent is not being requested.

27.1 If the research involves a waiver or alteration of the consent process, please review the "CHECKLIST: Waiver or Alteration of Consent Process (HRP-410)" to ensure that you have provided sufficient information for the IRB to make the determination that a waiver or alteration can be granted.

NOTE: For records review studies, the first set of criteria on the "CHECKLIST: Waiver or Alteration of Consent Process (HRP-410)" applies.

Response:

27.2 If the research involves a waiver of the consent process for planned emergency research, please review the "CHECKLIST: Waiver of Consent for Emergency Research (HRP-419)" to ensure you have provided sufficient information for the IRB to make these determinations. Provide any additional information necessary here:

Response:

28.0 Process to Document Consent

☐ **N/A:** A Waiver of Consent is being requested.
(Skip to Section 29.0)

28.1 Indicate whether you will be following "SOP: Written Documentation of Consent (HRP-091)." If not or if there are any exceptions, describe whether and how consent of the subject will be obtained including whether or not it will be documented in writing.

NOTE: If your research presents no more than minimal risk of harm to subjects and involves no procedures for which written documentation of consent is normally required outside of the research context, the IRB will generally waive the requirement to obtain written documentation of consent. This is sometimes referred to as 'verbal consent.' Review "CHECKLIST: Waiver of Written Documentation of Consent (HRP-411)" to ensure that you have provided sufficient information.

If you will document consent in writing, attach a consent document with your submission. You may use "TEMPLATE CONSENT DOCUMENT (HRP-502)". If you will obtain consent, but not document consent in writing, attach the script of the information to be provided orally or in writing (i.e. consent script or Information Sheet).

Response:

☒ We will be following "SOP: Written Documentation of Consent" (HRP-091).

29.0 Multi-Site Research (Multisite/Multicenter Only)

☐ **N/A:** This study is not an investigator-initiated multi-site study. This section does not apply.

29.1 If this is a multi-site study ***where you are the lead investigator****, describe the processes to ensure communication among sites, such as:*

- *All sites have the most current version of the IRB documents, including the protocol, consent document, and HIPAA authorization.*
- *All required approvals have been obtained at each site (including approval by the site's IRB of record).*
- *All modifications have been communicated to sites, and approved (including approval by the site's IRB of record) before the modification is implemented.*
- *All engaged participating sites will safeguard data as required by local information security policies.*
- *All local site investigators conduct the study appropriately.*
- *All non-compliance with the study protocol or applicable requirements will be reported in accordance with local policy.*

Response:

29.2 Describe the method for communicating to engaged participating sites:

- *Problems*
- *Interim results*
- *Study closure*

Response:

29.3 Indicate the total number of subjects that will be enrolled or records that will be reviewed across all sites.

Response: 81

29.4 If this is a multicenter study for which UB will serve as the IRB of record, and subjects will be recruited by methods not under the control of the local site (e.g., call centers, national advertisements) describe those methods.

Response: N/A

30.0 Banking Data or Specimens for Future Use

☐ **N/A:** This study is not banking data or specimens for future use or research outside the scope of the present protocol. This section does not apply.

30.1 If data or specimens will be banked (stored) for ***future use, that is, use or research outside of the scope of the present protocol****, describe where the data/specimens will be stored, how long they will be stored, how the data/specimens will be accessed, and who will have access to the data/specimens.*

NOTE: Your response here must be consistent with your response at the "What happens if I say yes, I want to be in this research?" Section of the Template Consent Document (HRP-502).

Response: The study data/specimens will be stored in a locked closet or -80 freezer at the research facility of the Diabetes and Endocrinology Center of WNY for at least 7 years.

The research staff (study personnel including coordinator) only will be authorized to access data and or specimens

30.2 List the data to be stored or associated with each specimen.

Response: Patient ID number, study visit information and date of collection will be stored with specimen. Other data stored will include record files of all patients participating in the study, including data collection sheets, lab results, CGM and insulin pump data.

30.3 Describe the procedures to release banked data or specimens for future uses, including: the process to request a release, approvals required for release, who can obtain data or specimens, and the data to be provided with specimens.

Response: The copy of the individual patient data collected during the study period will be provided to these individual patients who can choose to hand carry it to their respective physicians and a copy will be faxed to their respective clinical providers upon verbal request from the patient. The data provided will include the insulin pump or CGM data or any of the lab results obtained during the study period. The results of the completed study will be made available to the patients if requested through published manuscript.

31.0 Drugs or Devices

☐ **N/A:** This study does not involve drugs or devices. This section does not apply.

31.1 If the research involves drugs or devices, list and describe all drugs and devices used in the research, the purpose of their use, and their regulatory approval status.

Response: **Drugs used:**

Description or Identity of investigational product(s)

Investigational product	***Dosage form and strength***	***Manufacturer***	***Description***

Dapagliflozin (Farxiga) 5 mg	*Green, plain, diamond shaped, film coated 5 mg tablet*	*AstraZeneca*	*sodium-glucose cotransporter 2 (SGLT2) inhibitor indicated as an adjunct to diet and exercise to improve glycemic control*
Matching placebo for dapagliflozin 5 mg	*Green, plain, diamond shaped, film coated tablet*	*AstraZeneca*	
Semaglutide (Ozempic) 0.25 mg, 0.5 mg, 1.0 mg	*clear, colorless solution that contains 2 mg of semaglutide in a 1.5 mL (1.34 mg/mL) pre-filled, disposable, single-patient-use pen injector*	*Novo Nordisk*	*glucagon-like peptide 1 (GLP-1) receptor agonist indicated as an adjunct to diet and exercise to improve glycemic control*

- *Dapagliflozin and its matching placebo tablets will be supplied in bottles and are provided by Astra Zeneca.*
- *Semaglutide will be supplied as prefilled injectable pens and will be purchased commercially through the University at Buffalo Research Pharmacy.*

Subjects will receive drugs in their unmodified form. Dapagliflozin is a once daily tablet, while Semaglutide is a weekly subcutaneous injection.

Insulin pumps (CSII) and long and short acting insulin is the only diabetes medication accepted before and during this trial. No other diabetes drug is permitted.
Blood pressure or lipid lowering drugs are permitted if on stable dose for at least three months prior to start of trial. Glucagon injection is permitted as rescue medication for severe hypoglycemia. Insulin infusions may be administered by ER or hospital if patients develop DKA.

31.2 Describe your plans to store, handle, and administer those drugs or devices so that they will be used only on subjects and be used only by authorized investigators.

Response: Dapagliflozin 5mg tablets and matching placebo provided by Astra Zeneca will be stored in a locked cabinet in research pharmacy. Semaglutide provided by University at Buffalo research pharmacy will be stored in temperature controlled refrigerator at 4C at the research facility of the Diabetes and Endocrinology Center of WNY at 1000 Youngs Rd until dispensed to patients. Investigational drugs will be dispensed by PI or CO-Is only to study patients according to randomization plan.

If the drug is investigational (has an IND) or the device has an IDE or a claim of abbreviated IDE (non-significant risk device), include the following information:

31.3 Identify the holder of the IND/IDE/Abbreviated IDE.

Response: IND application for use of all drugs (dapagliflozin and semaglutide) in type 1 diabetes will be submitted to FDA and no study procedure will start before all local and

federal approvals are obtained. FDA has granted IND for usage of dapagliflozin in type 1 diabetes. We will seek FDA approval for the use of semaglutide in this study. These IND letters will be submitted to IRB and JDRF along with final version of protocol before initiating any study activities.

31.4 Explain procedures followed to comply with FDA sponsor requirements for the following:

	**Applicable to:**		
FDA Regulation	***IND Studies***	***IDE studies***	***Abbreviated IDE studies***
21 CFR 11	***X***	***X***	
21 CFR 54	***X***	***X***	
21 CFR 210	***X***		
21 CFR 211	***X***		
21 CFR 312	***X***		
21 CFR 812		***X***	***X***
21 CFR 820		***X***	

Response: All FDA sponsor requirements have been reviewed and will be followed during the study procedures

32.0 Humanitarian Use Devices

☒ **N/A:** This study does not involve humanitarian use devices. This does not apply.

32.1 For Humanitarian Use Device (HUD) uses provide a description of the device, a summary of how you propose to use the device, including a description of any screening procedures, the HUD procedure, and any patient follow-up visits, tests or procedures.

Response:

32.2 For HUD uses provide a description of how the patient will be informed of the potential risks and benefits of the HUD and any procedures associated with its use.

Response:

University at Buffalo Institutional Review Board (UBIRB)
Office of Research Compliance | Clinical and Translational Research Center Room 5018
875 Ellicott St. | Buffalo, NY 14203
UB Federalwide Assurance ID#: FWA00008824

Complete Research Protocol (HRP-503)

Table of Contents

Template Instructions ... 2
1.0 Objectives ... 4
2.0 Scientific Endpoints ... 5
3.0 Background ... 6
4.0 Study Design ... 12
5.0 Local Number of Subjects ... 12
6.0 Inclusion and Exclusion Criteria ... 13
7.0 Vulnerable Populations ... 16
8.0 Eligibility Screening ... 17
9.0 Recruitment Methods ... 18
10.0 Procedures Involved ... 20
11.0 Study Timelines ... 33
12.0 Setting ... 33
13.0 Community-Based Participatory Research ... 34
14.0 Resources and Qualifications ... 35
15.0 Other Approvals ... 36
16.0 Provisions to Protect the Privacy Interests of Subjects ... 36
17.0 Data Management and Analysis ... 37
18.0 Confidentiality ... 40
A. Confidentiality of Study Data ... 40
B. Confidentiality of Study Specimens ... 41
19.0 Provisions to Monitor the Data to Ensure the Safety of Subjects ... 42
20.0 Withdrawal of Subjects ... 49
21.0 Risks to Subjects ... 50
22.0 Potential Benefits to Subjects ... 56
23.0 Compensation for Research-Related Injury ... 56
24.0 Economic Burden to Subjects ... 57
25.0 Compensation for Participation ... 58
26.0 Consent Process ... 58
27.0 Waiver or Alteration of Consent Process ... 63
28.0 Process to Document Consent ... 64
29.0 Multi-Site Research (Multisite/Multicenter Only) ... 64
30.0 Banking Data or Specimens for Future Use ... 65
31.0 Drugs or Devices ... 66
32.0 Humanitarian Use Devices ... 68

Template Instructions

Sections that do not apply:

- *In several sections, the addition of checkboxes for* ***Not Applicable*** *have been added to the template as responses.*
    - *If an N/A checkbox is present, select the appropriate justification from the list.*
    - *If an N/A checkbox is not present, or if none of the existing checkboxes apply to your study, you must write in your own justification.*
- *In addition:*
    - *For research where the only study procedures are records/chart review: Sections 19, 20, 22, 23, 24, 25, 31, and 32 do not apply.*
    - *For exempt research: Sections 31 and 32 do not apply.*

Studies with multiple participant groups:

- *If this study involves multiple participant groups (e.g. parents and children), provide information in applicable sections for each participant group. Clearly label responses when they differ. For example:*

  Response:

  Intervention Group:

  Control Group:

Formatting:

- *Do not remove template instructions or section headings when they do not apply to your study.*

If you are pasting information from other documents using the "Merge Formatting" Paste option will maintain the formatting of the response boxes.

Amendments:

- *When making modifications or revisions to this and other documents, use the* ***Track Changes*** *function in Microsoft Word.*
- *Update the version date or number* ***on Page 3.***

PROTOCOL TITLE:

Include the full protocol title.

Response: **Triple Therapy for Type 1 Diabetes with Insulin, Semaglutide, and Dapagliflozin**

PRINCIPAL INVESTIGATOR:

Response:

**Principal Investigator:**	**Paresh Dandona, MBBS, PhD**
**Co- Investigators:**	**Husam Ghanim, PhD**
	**Antoine Makdissi, MD**
	**Manav Batra, MD**
**Co- Principal Investigator:**	**John R Petrie, MBChB, PhD**

Paresh Dandona, M.B.B.S., Ph.D., F.R.C.P, F.A.C.P, F.A.C.C, FACE,
SUNY Distinguished Professor,
Director, Diabetes-Endocrinology Center of Western NY
Chief of Endocrinology, Diabetes, Endocrinology and Metabolism, SUNY at Buffalo
1000 Youngs Road, Suite 105
Williamsville NY 14221
Telephone: (716) 535-1850
Fax: 716-639-1200
E-mail: pdandona@kaleidahealth.org

CO-PI:

Professor John R Petrie
BSc MBChB PhD FRCP(Ed) FRCPSG
Professor of Diabetic Medicine
Institute of Cardiovascular and Medical Sciences
BHF Glasgow Cardiovascular Research Centre
University of Glasgow
126 University Place
Glasgow G12 8TA
Tel: +44(0)141 330 3325
Email: john.petrie@glasgow.ac.uk
Web: http://www.gla.ac.uk/researchinstitutes/icams/staff/johnpetrie/
The University of Glasgow

VERSION:

Include the version date or number.

Response: 2/09/2026, V8

GRANT APPLICABILITY:

Indicate whether this protocol is funded by a grant (e.g. NIH, foundation grant). For a grant with multiple aims, indicate which aims are covered by this research proposal.

NOTE: This question does not apply to studies funded by a sponsor contract.

Include a copy of the grant proposal with your submission.

Response: Research support provided by JDRF and AZ

RESEARCH REPOSITORY:

Indicate where the research files will be kept, including when the study has been closed. The repository should include, at minimum, copies of IRB correspondence (approval, determination letters) as well as signed consent documents. This documentation should be maintained for 3 years after the study has been closed.

Response:
Site 1: Diabetes Endocrinology Research Center of WNY
Address: 1000 Youngs Road, Suite 105, Williamsville NY 14221
Department: Diabetes Endocrinology

Site 2: University of Glasgow. Institute of Cardiovascular and Medical Sciences University of Glasgow 126 University Place Glasgow, G12 8TA

1.0 Objectives

1.1 Describe the purpose, specific aims, or objectives of this research.

Response:

Primary Objective: To assess whether the addition of dapagliflozin to semaglutide and insulin (triple therapy) improves glycemic control in patients with type 1 diabetes compared with semaglutide and insulin (dual therapy) and insulin only (standard) treatment.

General hypothesis: The addition of dapagliflozin to semaglutide and insulin treatment improves glycemic control, weight loss and blood pressure in adults with type 1 diabetes.

1.2 State the hypotheses to be tested, if applicable.

NOTE: A hypothesis is a specific, testable prediction about what you expect to happen in your study that corresponds with your above listed objectives.

Response:

Specific hypotheses:

Primary: combined dapagliflozin and semaglutide treatment in addition to insulin will improve HbA1c levels compared with semaglutide in addition to insulin .

Secondary 1: semaglutide in addition to insulin treatment over 26 weeks will improve HbA1c levels compared with insulin therapy alone.

Secondary 2: combined dapagliflozin and semaglutide treatment in addition to insulin will improve HbA1c levels compared to insulin therapy alone at 52 weeks.

Secondary 3: combined dapagliflozin and semaglutide treatment in addition to insulin will improve CGM-based weekly mean and fasting blood glucose, glycemic oscillations and percent of time in range (70-180mg/dl) with lower insulin requirements compared with semaglutide and insulin (dual therapy) or insulin only (standard) treatment.

Secondary : combined dapagliflozin and semaglutide in addition to insulin therapy will reduce systolic and diastolic blood pressure compared with semaglutide and insulin (dual therapy) or insulin only (standard) treatment.

Secondary 5: combined dapagliflozin and semaglutide in addition to insulin therapy (triple therapy) will result in weight loss compared to dual and standard therapy.

Secondary 6: combined dapagliflozin and semaglutide in addition to insulin therapy will result in improved scores on the Diabetes Specific Quality of Life Scale (DSQOLS) and the Problem Areas In Diabetes (PAID) survey compared with dual and standard therapy.

Tertiary and Exploratory Objectives (EO) for future ancillary studies (PLEASE NOTE: For EO, samples will be collected to facilitate the measurements of these end points in future ancillary studies once submitted and approved by JDRF so cost of assays is not included in the current budget): The addition of dapagliflozin to semaglutide and insulin in type 1 diabetes will:

Tertiary 1: combined dapagliflozin and semaglutide treatment in addition to insulin therapy will improve fasting and postprandial glucose excursions and suppress postprandial glucagon, FFA, GLP-1 compared with semaglutide and insulin or insulin therapy alone.

Tertiary 2: semaglutide in addition to insulin treatment over 52 weeks will improve HbA1c levels compared with insulin therapy alone.

EO 1: increase plasma concentrations of vasodilatation mediators and reduce plasma concentrations of vasoconstriction mediators compared to dual and standard therapy groups.

EO 2: exert a systemic anti-inflammatory effect including reducing mRNA expression of inflammatory mediators and NFκB binding in peripheral mononuclear cells (MNC) and adipose tissue (optional) and their circulating levels in plasma while increasing levels of IL-RA and adiponectin.

2.0 Scientific Endpoints

2.1 Describe the scientific endpoint(s), the main result or occurrence under study.

NOTE: Scientific endpoints are outcomes defined before the study begins to determine whether the objectives of the study have been met and to draw conclusions from the data. Include primary and secondary endpoints. Some example endpoints are: reduction of

*symptoms, improvement in quality of life, or survival. Your response should **not** be a date.*

Response:

Primary endpoint: Change in HbA1c at 6 months following dapagliflozin or placebo therapy in addition to combined semaglutide and insulin treatment.

Efficacy Secondary endpoints:

1. Change in HbA1c from baseline at 12 months in triple therapy group compared to insulin only group.
2. Change in HbA1c from baseline at 6 months in dual therapy group compared to insulin only group.
3. Change in percent Time in Range (70– 180 mg/dl), percent time in hyperglycemia Level 1(180-250mg/dl) and Level 2 (>250mg/dl) and percent time in hypoglycemia Level 1 (54-70mg/dl) as assessed by CGM
4. Other indices of glycemic control: weekly mean and fasting glucose, standard deviation, insulin requirements.
5. Change in body weight and quality of life as assessed by DSQOLS and PAID at 6 and 12 months.
6. Anti-hypertensive effects including change in systolic and diastolic BP, numbers of BP medications required.

Safety Secondary endpoints:

1- Differences in rates of hypoglycemic events Level 2 (<54mg/dl) and Level 3 (A severe event characterized by altered mental and/or physical status requiring assistance) between investigative arms and standard therapy arm.

2- *Differences in rates of diabetic ketoacidosis defined as elevated serum or urine ketones (greater than the upper limit of the normal range), and serum bicarbonate <15 mmol/L or Blood pH <7.3 between investigative arms and standard therapy arm.*

Tertiary/ exploratory endpoints: (items 2-4 to be performed in future on collected samples from this study if additional funding is approved by JDRF):

1- Fasting and postprandial (Area Under curve) glucose excursions, FFA, GLP-1 and glucagon.

2- Indices of inflammatory stress. These will be CRP, FFA, SAA, IL-1β, IL-18, IL-1RA, adiponectin and TNFα concentrations in plasma and isoprostane in urine.

3- Expression of IL-1β, TNFα, IL-12, TLR2, TLR4, adiponectin, IκBα as well as NFκB binding in peripheral MNC and adipose tissue (optional).

4- Indices of vasodilatation and vasoconstriction: ANP, b-NP, cGMP, cAMP, angiotensinogen, renin and angiotensin II concentrations.

3.0 Background

3.1 Provide the scientific or scholarly background, rationale, and significance of the research based on the existing literature and how it will contribute to existing knowledge. Describe any gaps in current knowledge. Include relevant preliminary findings or prior research by the investigator.

Response:

The control of glucose homeostasis in type 1 diabetes is fragile since β-cell reserve is negligible. Traditionally, insulin has been the only treatment approach available. This approach, often leads to erratic blood sugar control given the challenge of matching exogenous insulin to mealtime glucose excursions in the face of a lack of any endogenous insulin to compensate for variation in requirements in response to meals and activity levels. Injected insulin boluses rarely match precise insulin requirements either in terms of dose administered or bio-availability. The result often is wide fluctuations in blood glucose readings. Based on our work to date and other published reports, previous attempts to add other glucose-lowering medications to insulin therapy yielded minimal benefits on glycemia (although metformin may have other benefits (1-3)

We have recently shown in two preliminary retrospective studies that the addition of liraglutide, a Glucagon Like Peptide -1 receptor agonist (GLP-1RA), to insulin results in consistent improvements in glycemic control in type 1 diabetes as demonstrated by reductions in HbA1c, glycemic excursions, and insulin requirements accompanied by improvements in body weight and systolic blood pressure (4, 5). We have now also completed the first prospective randomized controlled trial of this drug in 72 people with type 1 diabetes. Our data not only confirmed improvements in glycemic control, HbA1c, body weight and systolic BP, but also demonstrated a reduction in appetite, carbohydrate intake in low grade inflammation as measured by plasma CRP concentrations (6). Importantly, quality of life was also improved. Our recent studies in type 2 diabetes have revealed that systolic blood pressure reduction with GLP-1RA (exenatide) treatment in type 2 diabetes is associated with increased plasma concentrations of ANP, cGMP and cAMP along with reduced concentrations of angiotensinogen, renin and angiotensin II (7). It is possible that these mechanisms play a role in the prevention of cardiovascular complications in type 1 diabetes.

While GLP-1RAs, therefore, have great potential as adjunct therapy for type 1 diabetes, mean HbA1c was still ≥7% after liraglutide treatment (6) and larger pharma-sponsored trials reported increased rates of hypoglycemia (8, 9). In seeking further improvement, the advent of sodium-glucose transport protein-2 (SGLT2) inhibitors is of importance since their mode of action is independent of insulinogenesis. Introduced for use in type 2 diabetes, SGLT2 inhibitors are selective antagonists of the sodium-glucose transporter subtype expressed in the proximal convoluted tubules of the kidneys. They act by inhibiting renal glucose re-absorption and therefore stimulating excretion of 60-100 grams per day of glucose in urine. SGLT2 inhibitors have been studied extensively in type 2 diabetes, including the landmark cardiovascular outcome trial EMPA-REG (10), but data have only quite recently begun to accumulate in type 1(11). Short term effects on urine glucose excretion of remogliflozin and empagliflozin are similar to those seen in type 2 diabetes and these translate into improved glycemic and other indices including HbA1c; fasting blood glucose, rates of hypoglycemia, daily insulin dose and weight (12). Glucose-lowering efficacy is attenuated in individuals with eGFR <45/ml/min/1.73m^2(13). Therefore, use of dapagliflozin is recommended for patients with eGFR>45/ml/min/1.73 m^2 (as per prescribing information). Weight loss accompanying increased urinary glucose excretion could translate into reduced cardiovascular complications as in type 2 diabetes, but there are no data so far available to support this hypothesis. More recently, in the Phase 3 trial DEPICT-1 we showed that dapagliflozin improves glycemic control and HbA1c over six months by about 0.45% with no severe hypoglycemia or diabetic ketoacidosis

(14). Similarly, sotagliflozin has been shown in a Phase 3 trial to allow a larger proportion of patients to achieve HbA1c lower than 7.0% with no severe hypoglycemia although the rate of diabetic ketoacidosis was higher in the sotagliflozin group (15). Both products are now being reviewed by regulatory agencies for the type 1 diabetes indication.

On the basis of the above, we hypothesize that adding an SGLT2 inhibitor to a combination of insulin and semaglutide (a weekly and potent GLP-1RA) will improve glycemic control in type 1 diabetes such that HbA1c levels of ≤7% are achieved more consistently. Our protocol is designed to allow an objective assessment of whether this triple therapy combination will work effectively and safely so that a majority of people with type 1 diabetes can reach a glycemic target HbA1c of ≤7%. In addition, since both semaglutide and dapagliflozin inhibitors reduce blood pressure and weight, it will allow an assessment of the combined effect of GLP-1RAs and SGLT2 inhibitors on these outcomes. Since there are no previous data on the effects of once weekly semaglutide or the combination of semaglutide and dapagliflozin compared to standard insulin treatment in type 1 diabetes, the design requires allocation of some participants to insulin therapy only.

Due to the potential ketogenic effect of SGLT-2 inhibitors, data from SGLT-2 inhibitors studies in type 1 diabetes were analyzed for it ketogenic effects and a consensus statement and guidelines on management of patients was recently published (Danne T et al., Diabetes Care. 2019 Feb 6).

Preliminary data

1) Liraglutide treatment in patients with type 1 diabetes (4): We treated 8 type 1 diabetes patients; mean age (38.5 years) and duration of diabetes (20±4 yrs), with liraglutide in addition to insulin for 24 weeks. As shown in table 1, there was a significant reduction in fasting and mean blood glucose concentrations, insulin doses, HbA1c and a reduction in appetite and caloric intake with weight loss. In addition, there was a reduction in the excursions of blood glucose concentrations, i.e., the standard deviations of blood glucose concentrations were markedly reduced (**figure 1**). The changes were observed within 48h and improved marginally thereafter with fine tuning of insulin doses and a further reduction in these doses. These observations are noteworthy in that study participants were well controlled and meticulous users of CSII and CGM. They had all tried hard to improve their diabetic control by adjusting insulin doses but had not succeeded because of hypoglycemia. We then demonstrated that poorly controlled and obese individuals with type 1 diabetes also experienced similar benefits of liraglutide treatment (2).

2) Liraglutide As Additional Treatment to Insulin in Patients with Type 1 Diabetes Mellitus: A Randomized Clinical Trial (6): Following the two above non-randomized studies we recently conducted a randomized clinical trial in which 72 individuals (Placebo=18; liraglutide = 54) with TYPE 1 DIABETES for at least one year, on insulin therapy and with no detectable C-peptide in plasma (mean BMI: 30±1; mean body weight: 84±2 kg ; mean HbA1c: 7.57±0.09%; mean age: 44±2 years; mean age of TYPE 1 DIABETES diagnosis: 20±1 years) were randomized to receive placebo, 0.6, 1.2 or 1.8mg of liraglutide daily for 12 weeks. In the 1.2 mg and 1.8 mg groups, mean change in average blood glucose was -10±2 and -10.0±1mg/dl, respectively, ($p<0.0001$ vs. placebo). In the 1.2mg group, HbA1c fell by 0.78% from 7.84±0.17% to 7.06±0.15% ($p<0.0001$, $p<0.01$

vs. placebo) and in the 1.8mg group fell by 0.42% from 7.41±0.15 to 6.99±0.15 ($p=0.001$, $p=0.39$ vs placebo). Time spent between 70 to 160 mg/dl increased by 5±1% ($p<0.05$ vs placebo) and time between 160-400 mg/dl decreased by 7±1% ($p<0.01$ vs placebo) in the 1.8 mg group with no additional hypoglycemia. During the 12 week period, the average total daily dose of insulin in the two groups fell by 12.4±3.9 and 10.0±2.3 units respectively ($p<0.05$ vs baseline and placebo). There was a reduction in body weight (from 83±4 to 78±5 kg, $p<0.0001$, in the 1.8mg group, from 96±4 to 91±4 kg, $p<0.001$, in the 1.2 mg group and from 80±4 to 77±4 kg, $p<0.01$ in the 0.6 mg group). A reduction in daily carbohydrate intake (171±17g vs. 127±18g; $p<0.01$ and 153±18g vs 115±16g, $p<0.01$ in the 1.2 mg and 1.8 mg groups, respectively) was also observed over 12 weeks. Systolic BP (SBP) in the 1.8 mg group fell by 3 mmHg (120±2 to 117±3 mmHg, $p=0.01$). CRP fell by 15±6 and 19±8% in the 1.2 and 1.8mg groups, respectively ($p<0.05$). Quality of life improved significantly compared to baseline and placebo (unpublished data).

3) Dapagliflozin as an Additional Treatment to Insulin and Liraglutide in Patients with Type 1 Diabetes Mellitus (16): The objective of this randomized clinical trial was to investigate whether addition of dapagliflozin to insulin and liraglutide results in a significant reduction in glycemia and body weight. 30 participants with TYPE 1 DIABETES on liraglutide therapy for at least last 6 months were randomized (in 2:1 ratio) to receive either dapagliflozin 10 mg or placebo daily for 12 weeks. With 12 weeks of dapagliflozin HbA1c fell by 0.66±0.08% from 7.8±0.21% ($p < .01$ vs placebo) but did not change significantly with placebo (7.40±0.20% to 7.30±0.20%). In one patient within one week of addition of dapagliflozin average glucose fell from 170 to 122 mg/dl while standard deviation was reduced from 71 to 58 (**Figure 2-diminished glycemic excursions)**. Body weight fell by1.9 +/- 0.54kg ($p < 0.05$ vs placebo). There was no additional hypoglycemia (blood glucose < 3.88 mmol/L; $p = 0.52$ vs placebo). In the dapagliflozin group, there were significant increases in the plasma concentrations of glucagon by 35% +/- 13% ($p < 0.05$), hormone-sensitive lipase by 29% +/- 11% ($p < 0.05$), free fatty acids by 74% +/- 32% ($p < 0.05$), acetoacetate by 67% +/- 34% ($p < 0.05$), and beta-hydroxybutyrate by 254% +/- 81% ($p < 0.05$). Urinary ketone levels also increased significantly ($P < .05$). None of these changes was observed in the placebo group. Two individuals in the dapagliflozin group developed ketoacidosis. Overall, addition of dapagliflozin to insulin and liraglutide in patients with TYPE 1 DIABETES resulted in a significant improvement in glycemia and in weight loss but tended to increase ketosis.

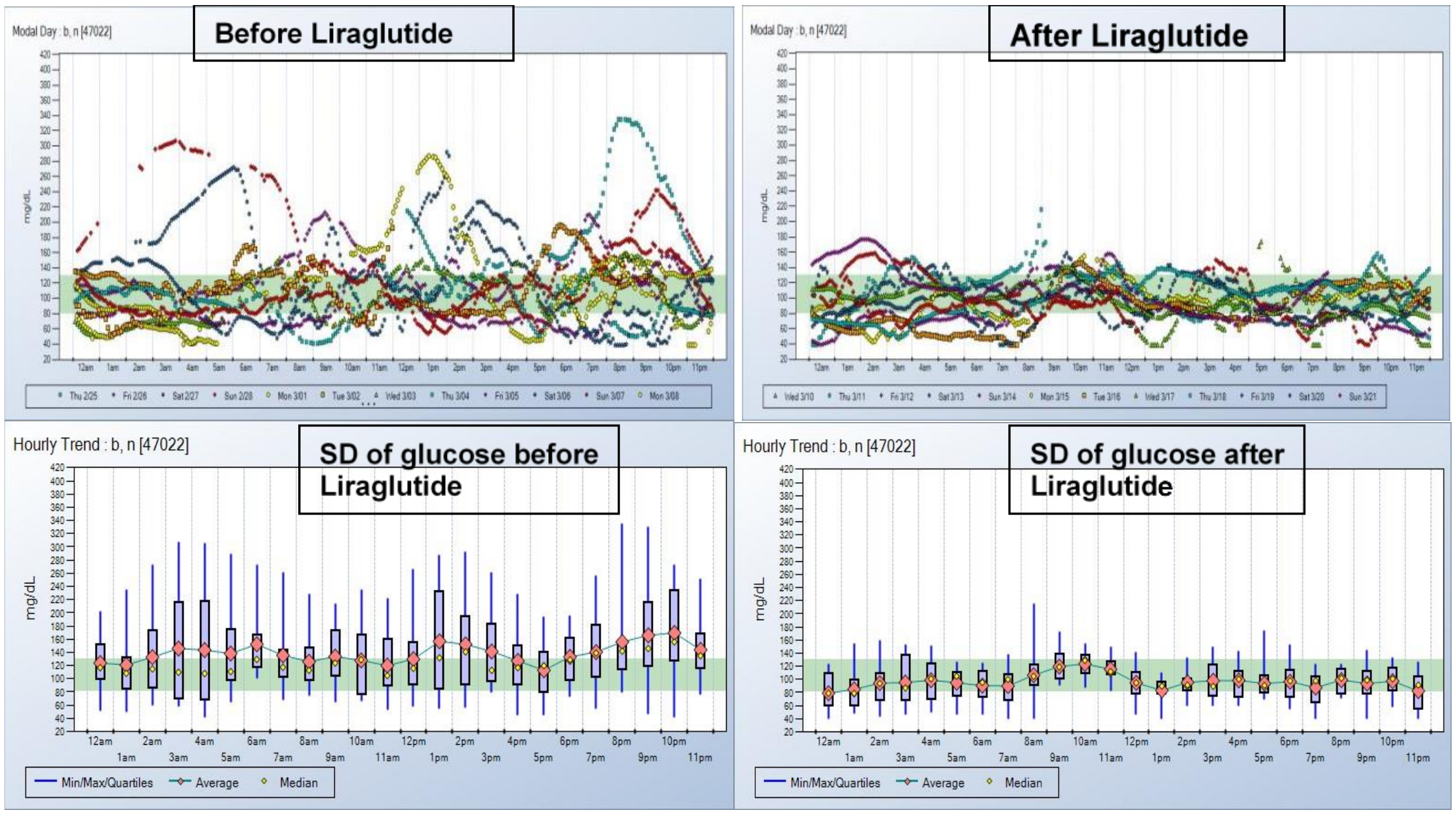

Figure 1: Weekly continuous glucose monitoring record of one patient prior to and following 1 week of liraglutide. Note the glycemic variability (SD) and the frequency of glucose concentrations within the target range prior to and following liraglutide

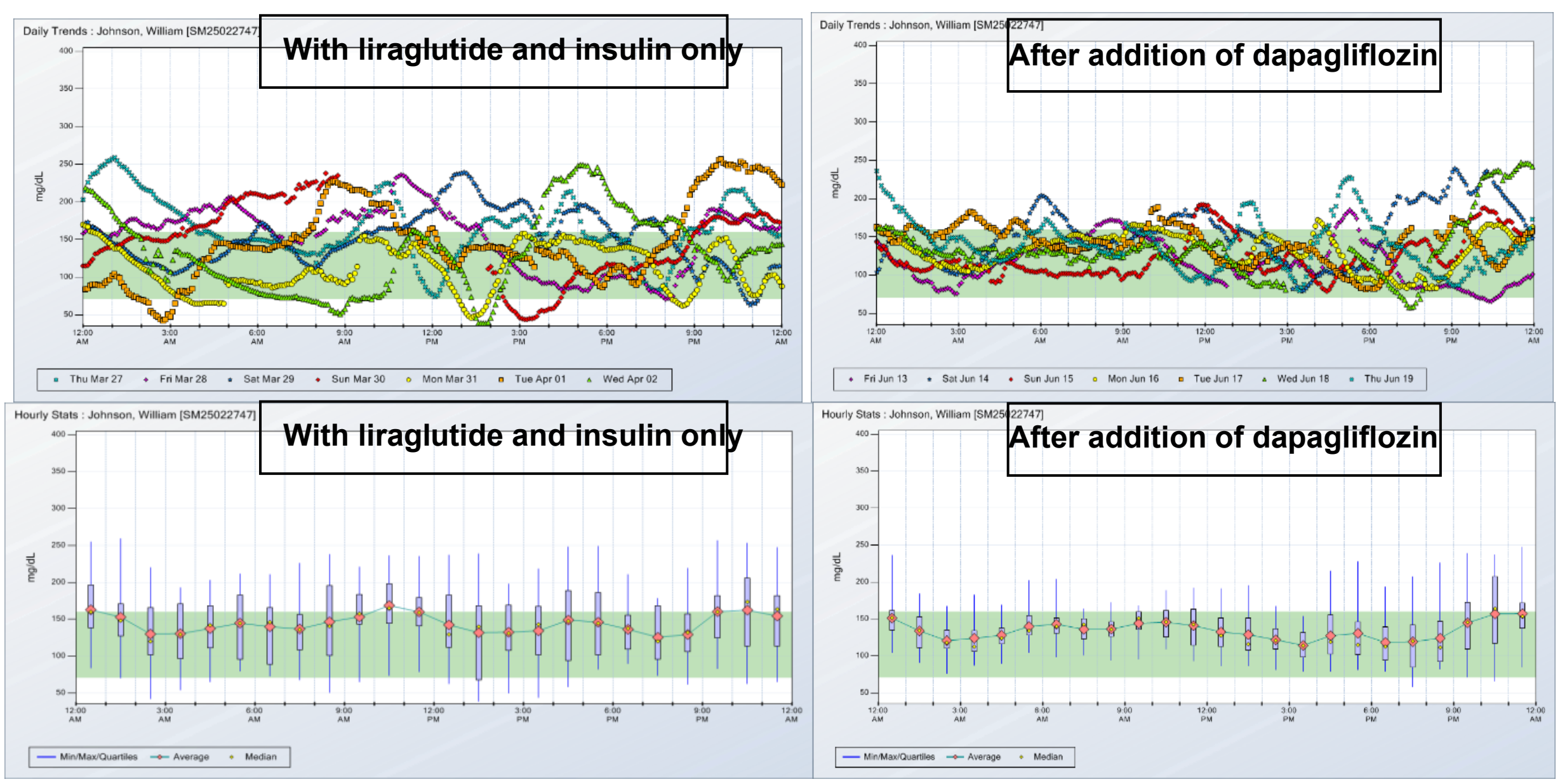

Figure 2: Weekly continuous glucose monitoring record of one patient prior to and following 8 weeks of the triple therapy combination with dapagliflozin and liraglutide. Note the glycemic variability (SD) (bottom) and the frequency of glucose concentrations within the target range prior to and following dapagliflozin addition.

3.2 Include complete citations or references.

Response:

1. Konrad K, Datz N, Engelsberger I, Grulich-Henn J, Hoertenhuber T, Knauth B, et al. Current use of metformin in addition to insulin in pediatric patients with type 1 diabetes mellitus: an analysis based on a large diabetes registry in Germany and Austria. *Pediatr Diabetes.* 2014.
2. Vella S, Buetow L, Royle P, Livingstone S, Colhoun HM, and Petrie JR. The use of metformin in type 1 diabetes: a systematic review of efficacy. *Diabetologia.* 2010;53(5):809-20.
3. Vella S, Buetow L, Royle P, Livingstone S, and Petrie JR. Metformin in type 1 diabetes reduces insulin requirements without significantly improving glycaemic control. Reply to Schatz H [letter]. *Diabetologia.* 2011;54(1):203-4.
4. Varanasi A, Bellini N, Rawal D, Vora M, Makdissi A, Dhindsa S, et al. Liraglutide as additional treatment for type 1 diabetes. *Eur J Endocrinol.* 2011;165(1):77-84.
5. Kuhadiya ND, Malik R, Bellini NJ, Patterson JL, Traina A, Makdissi A, et al. Liraglutide as additional treatment to insulin in obese patients with type 1 diabetes mellitus. *Endocrine practice : official journal of the American College of Endocrinology and the American Association of Clinical Endocrinologists.* 2013;19(6):963-7.
6. Kuhadiya ND, Dhindsa S, Ghanim H, Mehta A, Makdissi A, Batra M, et al. Addition of Liraglutide to Insulin in Patients With Type 1 Diabetes: A Randomized Placebo-Controlled Clinical Trial of 12 Weeks. *Diabetes Care.* 2016;39(6):1027-35.
7. Chaudhuri A, Ghanim H, Makdissi A, Green K, Abuaysheh S, Batra M, et al. Exenatide induces an increase in vasodilatory and a decrease in vasoconstrictive mediators. *Diabetes Obes Metab.* 2017;19(5):729-33.
8. Mathieu C, Zinman B, Hemmingsson JU, Woo V, Colman P, Christiansen E, et al. Efficacy and Safety of Liraglutide Added to Insulin Treatment in Type 1 Diabetes: The ADJUNCT ONE Treat-To-Target Randomized Trial. *Diabetes Care.* 2016;39(10):1702-10.
9. Ahren B, Hirsch IB, Pieber TR, Mathieu C, Gomez-Peralta F, Hansen TK, et al. Efficacy and Safety of Liraglutide Added to Capped Insulin Treatment in Subjects With Type 1 Diabetes: The ADJUNCT TWO Randomized Trial. *Diabetes Care.* 2016;39(10):1693-701.
10. Zinman B, Wanner C, Lachin JM, Fitchett D, Bluhmki E, Hantel S, et al. Empagliflozin, Cardiovascular Outcomes, and Mortality in Type 2 Diabetes. *N Engl J Med.* 2015;373(22):2117-28.
11. Petrie JR. SGLT2 inhibitors in type 1 diabetes: knocked down, but up again? *Lancet Diabetes Endocrinol.* 2017;5(11):841-3.
12. Mudaliar S, Armstrong DA, Mavian AA, O'Connor-Semmes R, Mydlow PK, Ye J, et al. Remogliflozin etabonate, a selective inhibitor of the sodium-glucose transporter 2, improves serum glucose profiles in type 1 diabetes. *Diabetes Care.* 2012;35(11):2198-200.
13. Kohan DE, Fioretto P, Tang W, List JF. Long-term study of patients with type 2 diabetes and moderate renal impairment shows that dapagliflozin reduces weight and blood pressure but does not improve glycemic control. Kidney Int. 2014 Apr;85(4):962-71.

14. Dandona P, Mathieu C, Phillip M, Hansen L, Griffen SC, Tschope D, et al. Efficacy and safety of dapagliflozin in patients with inadequately controlled type 1 diabetes (DEPICT-1): 24 week results from a multicentre, double-blind, phase 3, randomised controlled trial. *Lancet Diabetes Endocrinol.* 2017;5(11):864-76.
15. Garg SK, Henry RR, Banks P, Buse JB, Davies MJ, Fulcher GR, et al. Effects of Sotagliflozin Added to Insulin in Patients with Type 1 Diabetes. *N Engl J Med.* 2017;377(24):2337-48.
16. Kuhadiya ND, Ghanim H, Mehta A, Garg M, Khan S, Hejna J, et al. Dapagliflozin as Additional Treatment to Liraglutide and Insulin in Patients With Type 1 Diabetes. *J Clin Endocrinol Metab.* 2016;101(9):3506-15.

17. Andrews NC, and Faller DV. A rapid micropreparation technique for extraction of DNA-binding proteins from limiting numbers of mammalian cells. *Nucleic Acids Res.* 1991;19(9):2499.

4.0 Study Design

4.1 Describe and explain the study design (e.g. case-control, cross-sectional, ethnographic, experimental, interventional, longitudinal, observational).

Response: This will be a two-center, randomized, open-label then double-blind, parallel group, placebo and standard of care controlled prospective study in type 1 diabetes. Eighty-one (81) patients will be randomized (open label, see below) into three groups (27 each) and will be either maintained on their regular insulin therapy for 12 months (standard therapy group) or started on semaglutide in addition to regular insulin treatment for six month (dual therapy and triple therapy groups). For those on insulin and semaglutide, after six months of stable treatment, dapagliflozin (triple therapy group) or placebo (dual therapy group) treatment will be initiated for an additional six months according to a second randomization (double-blind).

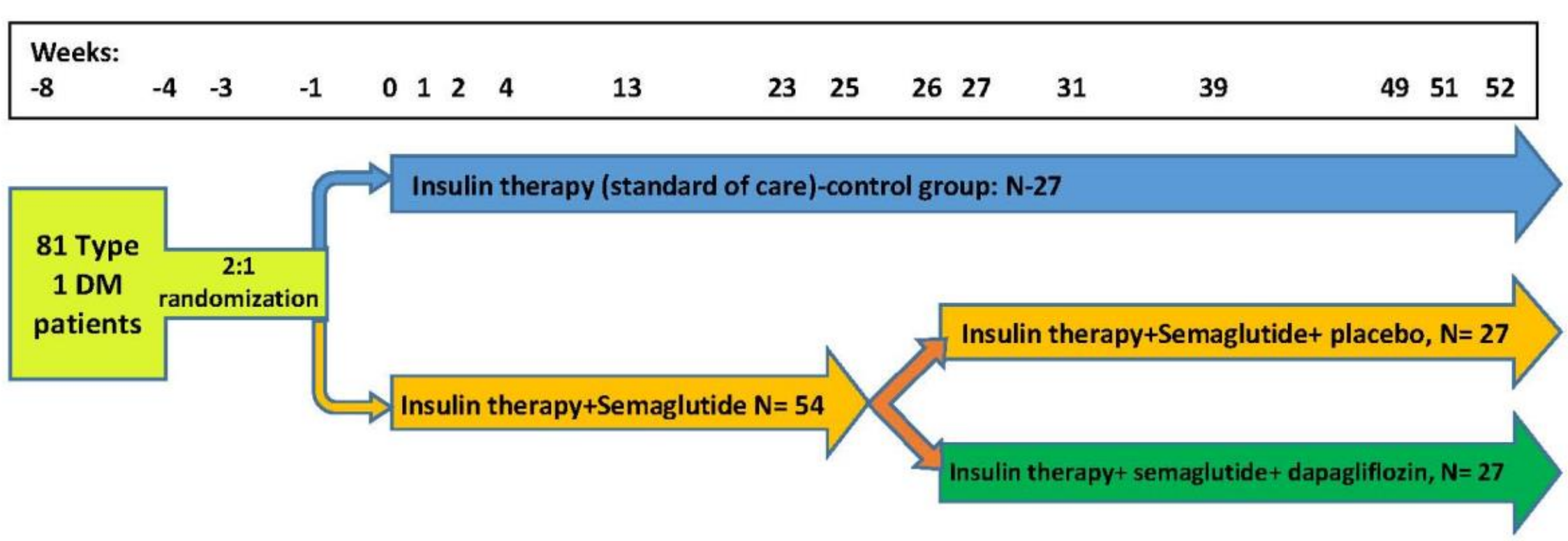

5.0 Local Number of Subjects

5.1 Indicate the total number of subjects that will be enrolled or records that will be reviewed locally.

Response: Patients will be enrolled locally at UB site and at the University of Glasgow site for a total of 81. Enrollment will be open to both sites until target enrollment is achieved.

5.2 If applicable, indicate how many subjects you expect to screen to reach your target sample (i.e. your screen failure rate).

Response: All screened and qualified patients will be enrolled and randomized

5.3 Justify the feasibility of recruiting the proposed number of eligible subjects within the anticipated recruitment period. For example, how many potential subjects do you have access to? What percentage of those potential subjects do you need to recruit?

Response: The Diabetes and Endocrinology Center of WNY is the largest Diabetes center in the WNY area, seeing between 60 to 100 type 1 diabetic patient every month. Therefore, the majority of recruited patients are our clinic patients. We also recruit a study participants through advertisement and other physician's references. These sources will suffice to recruit the needed number to subjects. The Glasgow center (Stobhill Ambulatory Care Hospital) reviews more than 300 patients per month.

6.0 Inclusion and Exclusion Criteria

*6.1 Describe the criteria that define who will be **included** in your final study sample.*

NOTE: This may be done in bullet point fashion.

Response:

Inclusion Criteria

1) Type 1 Diabetes for at least 1 year on staple use of continuous subcutaneous insulin infusion (CSII) or multiple daily (four or more) injections (MDI) of insulin for last 3 months.
2) C-peptide <0.23 nM
3) Minimum dose of insulin in Units/kg at entry: 0.5 U/kg for MDI and 0.4 U/kg for CSII
4) Regularly measuring blood sugars four or more times daily or use of CGM.
5) HbA1c between7.5-11% inclusive.
6) Well versed in CHO counting*
7) Age 18-70 years.
8) BMI ≥25 kg/m^2.

*In the opinion of the site Principal Investigator

*6.2 Describe the criteria that define who will be **excluded** from your final study sample.*

NOTE: This may be done in bullet point fashion.

Response:

Exclusion Criteria

1) Type 1 diabetes for less than 12 months, type 2 diabetes, chronic pancreatitis, MODY
2) Previous use of any agent other than insulin for treatment of diabetes in the last 3 months.
3) History of diabetic ketoacidosis (DKA) requiring medical intervention (e.g., emergency room visit and/or hospitalization) within 3 months prior to the screening visit
4) Patients on ketogenic or low (<50g/day) carbohydrate diet or who practice intermittent fasting.
5) Frequent episodes of severe hypoglycemia as defined by more than one episode requiring medical assistance, emergency care (paramedics or emergency room care), and/or glucagon therapy administered by a third-party individual within 1 month prior to the screening visit
6) Symptoms of poorly controlled diabetes that would preclude participation in this trial
7) Subjects on a commercial weight loss program with ongoing weight loss, or on an intensive exercise program
8) History of bariatric surgery or lap-band procedure within 12 months prior to screening
9) History of Addison's disease or chronic adrenal insufficiency
10) History of diabetes insipidus
11) Hepatic disease or cirrhosis
12) Aspartate Aminotransferase (AST) > 3X Upper limit of normal (ULN) *and/or* Alanine aminotransferase (ALT) > 3X ULN
13) Serum Total Bilirubin > 2X ULN unless exclusively caused by Gilbert's Syndrome
14) Hemoglobin < 11.0 g/dL (110 g/L) for men; hemoglobin < 10.0 g/dL (100 g/L) for women.
15) Coronary event or procedure (myocardial infarction, unstable angina, coronary artery bypass, surgery or coronary angioplasty) in the previous 3 months or patients with congestive heart failure.
16) ESRD on hemodialysis; and or e-GFR < 45 ml/min/1.73m^2
17) HIV or Hepatitis B/C positive status
18) Any other life-threatening, noncardiac disease
19) History of pancreatitis
20) Women who are pregnant or women of childbearing potential who are not using adequate contraception or who are breast feeding
21) Inability to give informed consent
22) History of gastroparesis
23) History of medullary thyroid carcinoma or MEN 2 syndrome
24) History of serious hypersensitivity reaction to these agents
25) Painful gallstones

26) Alcoholism
27) Hypertriglyceridemia (>500 mg/dl)
28) Recurrent genital mycotic infection.
29) Hypovolemic patients or with chronic renal insufficiency.
30) Patients with any malignancy except treated in situ malignancy and basal cell carcinoma of the skin
31) Unexplained hematuria
32) Patients with a history of diabetic retinopathy
33) Use of an investigational agent or therapeutic regimen within 30 days of study
34) Participation in any other concurrent interventional clinical trial

6.3 Indicate specifically whether you will include any of the following special populations in your study using the checkboxes below.

NOTE: Members of special populations may not be targeted for enrollment in your study unless you indicate this in your inclusion criteria.

Response: NA

☐ Adults unable to consent

☐ Individuals who are not yet adults (infants, children, teenagers)

☐ Pregnant women

☐ Prisoners

6.4 Indicate whether you will include non-English speaking individuals in your study. ***Provide justification if you will exclude non-English speaking individuals.***

In order to meet one of the primary ethical principles of equitable selection of subjects, non-English speaking individuals may ***not*** *be routinely excluded from research as a matter of convenience.*

In cases where the research is of therapeutic intent or is designed to investigate areas that would necessarily require certain populations who may not speak English, the researcher is required to make efforts to recruit and include non-English speaking individuals. However, there are studies in which it would be reasonable to limit subjects to those who speak English. Some examples include pilot studies, small unfunded studies with validated instruments not available in other languages, studies with numerous questionnaires, and some non-therapeutic studies which offer no direct benefit.

Response:

We have no non-English speaking patients in this population. We have patients that English is a second language, but they are able to read, write

and understand it. This population is less than 10% of the total population. No benefit is being withheld from not participating in the study.

7.0 Vulnerable Populations

If the research involves special populations that are considered vulnerable, ***describe the safeguards included to protect their rights and welfare.***

NOTE: You should refer to the appropriate checklists, referenced below, to ensure you have provided adequate detail regarding safeguards and protections. You do not, however, need to provide these checklists to the IRB.

7.1 For research that involves ***pregnant women,*** *safeguards include:*
NOTE CHECKLIST: Pregnant Women (HRP-412)

Response: We will not be recruiting subjects from vulnerable populations

☒ **N/A**: This research does not involve pregnant women.

7.2 For research that involves ***neonates of uncertain viability or non-viable neonates,*** *safeguards include:*
NOTE CHECKLISTS: Non-Viable Neonates (HRP-413), or Neonates of Uncertain Viability (HRP-414)

Response:

☒ **N/A:** This research does not involve non-viable neonates or neonates of uncertain viability.

7.3 For research that involves ***prisoners****, safeguards include:*
NOTE CHECKLIST: Prisoners (HRP-415)

Response:

☒ **N/A:** This research does not involve prisoners.

7.4 For research that involves ***persons who have not attained the legal age for consent to treatments or procedures involved in the research ("children")****, safeguards include:*
NOTE CHECKLIST: Children (HRP-416)

Response:

☒ **N/A:** This research does not involve persons who have not attained the legal age for consent to treatments or procedures ("children").

7.5 For research that involves ***cognitively impaired adults****, safeguards include:*
NOTE CHECKLIST: Cognitively Impaired Adults (HRP-417)

Response:

☒ **N/A:** This research does not involve cognitively impaired adults.

7.6 Consider if other specifically targeted populations such as students, employees of a specific firm, or educationally or economically disadvantaged persons are vulnerable. ***Provide information regarding their safeguards and protections, including safeguards to eliminate coercion or undue influence.***

Response: No specific populations or vulnerable groups will be targeted. All subjects enrolled in this study will be of legal adult consenting age with the ability to speak, read and interpret the English language. Patients will have the ability to speak with the research team regarding any questions or concern they have before signing the consent. Research team have the ability to evaluate patient's cognitive abilities through these interactions with patients. Patients are made aware that this study is voluntary and they are able to stop participating at any time they feel uncomfortable. Patients are not to be pressured into participating and their clinic standard of care will remain the same if they participate or choose not to participate.

8.0 Eligibility Screening

8.1 Describe ***screening procedures*** *for determining subjects' eligibility. Screening refers to determining if prospective participants meet inclusion and exclusion criteria.*

Include all relevant screening documents with your submission (e.g. screening protocol, script, questionnaire).

Response:

After obtaining partial HIPAA waiver, patient charts will be screened according to the study inclusion and exclusion criteria by our trained clinical staff and physicians. In additions, the study clinical team will evaluate their clinic patients for possible participation in this study according to the inclusion and exclusion criteria at the Diabetes and Endocrinology Center of WNY clinics. Physicians will speak to them about their interests in participating in research. If the patient agrees, their information will be given to the research coordinator to be contacted for further evaluation. The research coordinator will contact interested patients and those meeting the inclusion and exclusion criteria based on preliminary phone evaluation will be invited to participate in the study. All personal information will be kept confidential and locked in the coordinator office.

Prospective participants will be asked to read the consent and any questions they may have regarding the protocol will be answered. If the subject wants to participate in the study, they will be asked to sign the informed consent form. The subject's medical history and current medications will be obtained as well as their blood pressure and vitals. A physical examination will also be done. Blood and urine samples will be taken in order to evaluate HbA1c, CBC, CMP and liver and kidney functions and pregnancy status. Females of childbearing age, will have to be on two forms of birth control to prevent pregnancy. Patients meeting all the

inclusion and exclusion criteria based on all screening tests will be enrolled in the study.

☐ **N/A:** There is no screening as part of this protocol.

9.0 Recruitment Methods

☐ **N/A:** This is a records review only, and subjects will not be recruited. NOTE: If you select this option, please make sure that all records review procedures and inclusion/exclusion screening are adequately described in other sections.

9.1 Describe when, where, and how potential subjects will be recruited.

NOTE: Recruitment refers to how you are identifying potential participants and introducing them to the study. Include specific methods you will use (e.g. searching charts for specific ICD code numbers, Research Participant Groups, posted advertisements, etc.).

Response:

Participants will be identified by prescreening clinical charts, patient doctor interaction at the time of their visits to the Diabetes Endocrinology Center of WNY Locations include:

1. 1020 Youngs Road, Williamsville NY 14221
2. 705 Maple Road, Williamsville NY 14221
3. 462 Grider Street, Buffalo NY 14215
4. 1000 Youngs Road, Suite 105, Williamsville NY 14221

Patients can be also referred by other physicians and providers

The remainder of the participants (57 patients) will be recruited from NHS Greater Glasgow and Clyde Diabetes clinics (Scotland, UK). Separate local IRB/Ethical approval will be sought to cover this site (based on discussions with UBIRB director).

The study clinical team will evaluate their clinic patients for possible participation in this study according to the inclusion and exclusion criteria at the Diabetes and Endocrinology Center of WNY clinics. Patients that may qualify for the study are referred to the research team for further eligibility evolution. Patients meeting the inclusion and exclusion criteria based on preliminary phone evaluation will be invited to participate in the study.

Participants will also be identified by:

-Flyers, advertisements on Facebook, ,

- Researchmatch.org, UB's participate in research (PIR) website, and through Buffalo research registry (BRR).

- We will also use the TriNetX/i2b2 database through UB's Institute for Healthcare Informatics (IHI) to recruit participants from the UBMD medical database.

Along with this, a radio ad will be on the air of the radio station STAR102.5 at various times during the day during the month of mid January to mid February

9.2 Describe how you will protect the privacy interests of prospective subjects during the recruitment process.

NOTE: Privacy refers to an individual's right to control access to him or herself.

Response: Patient charts will be screened according to the study inclusion and exclusion criteria by our trained clinical staff and physicians. If the patient qualifies and is of consenting age, the physicians will speak to them about their interests in participating in research in a private room where no one else can hear the conversation. If the patient agrees, their information will be given to the research coordinator to be contacted for further evaluation. No personal information will be shared without patient's approval and will not be used for any purpose except for this research. All personal information will be kept confidential and locked in the coordinator office.

The privacy of patients recruited using the TriNetX/ i2b2 data will be protected by following the UB IRB-approved procedure. A letter will be sent to the eligible patient's physician (see attached). After one week, if the physician doesn't contact the research team to refuse, a recruitment letter will be sent to participants via mail (see attached). If we don't hear from the patients within one week, one follow-up call will be made (see attached phone script). If at any point the physician or the patient declines, the patient will be removed from the contact list.

The Clinical and Translational Science Institute's (CTSI) Community Engagement Team (CET) hosts the Buffalo Research Registry (BRR, IRB Approved STUDY00000806), a resource that connects researchers looking to recruit participants and community members looking to get involved with research. To participate in the registry, volunteers complete a voluntary intake form. Volunteers have agreed to be contacted about potential research opportunities based on their self-reported information.

As described in the BRR's IRB Approved Protocol (STUDY00000806), key personnel from the CTSI will serve as the conduit between our research team and the registry volunteers. Key personnel will verify our IRB study approval, review inclusion and exclusion criteria and will sort the registry data accordingly. In terms of recruiting for our study, key personnel will pull volunteer reports using the inclusion and exclusion criteria. Key personnel will centrally invite volunteers to participate in the study and provide a warm hand off to our team. There are two ways we are sharing our study. These include:

Electronically- Using the volunteer report from REDCap, key personnel from the CTSI will send initial e-mail invites to introduce our study to registry volunteers.

Post Mail- Key personnel from the CTSI will provide our team with contact information (i.e. first name, last name, post mail address) to prepare a mailing. This information will be shared in a password protected excel spread sheet. The password to the spreadsheet will be sent in a separate follow-up e-mail. We will

only use the list for this protocol and we agree to destroy the list once recruitment for the study has closed.
We also plan on conducting follow-up by phone/email after initial contact. This contact information will be provided in a password protected excel spread sheet. The password to the spreadsheet will be sent in a separate follow-up e-mail. We will only use the list for this protocol and we agree to destroy the list once recruitment for the study has closed.
We will have no access to the health information provided in the registry.

9.3 Identify any materials that will be used to recruit subjects.

NOTE: Examples include scripts for telephone calls, in person announcements / presentations, email invitations.

For advertisements, include the final copy of printed advertisements with your submission. When advertisements are taped for broadcast, attach the final audio/video tape. NOTE: You may submit the wording of the advertisement prior to taping to ensure there will be no IRB-required revisions, provided the IRB also reviews and approves the final version.

Response: In addition to screening clinical charts, participations will be identified through; flyer advertisement, researchmatch.org, and Facebook. The study will also be put on UB's PIR website.

The researchmatch.org contact message and listing is provided as separate attachment to this submission (RM IRB Template Language-JDRF)

Sample letters of physician permission and patient contact and phone prescreen script are also included in this submission.

10.0 Procedures Involved

10.1 Provide a description of ***all research procedures or activities*** *being performed and when they are performed once a subject is screened and determined to be eligible. Provide as much detail as possible.*

NOTE: This should serve as a blueprint for your study and include enough detail so that another investigator could pick up your protocol and replicate the research. For studies that have multiple or complex visits or procedures, consider the addition of a schedule of events table in in your response.

Response:

Each patient will spend about 55 weeks in the trial comprising three weeks of monitoring to stabilize baselines and ensure adherence and commitment to participation before randomization into an intervention arm for an additional period of 52 weeks. Following a completed screening visit and meeting the inclusion and exclusion criteria, each patient will have a total of 16 study visits. At weeks 3, 5, 8, 16, 20, 29, 35, 44 and at 2 weeks after the end of the study, each patient will receive a phone call to document any adverse effects or other changes. There will be no long term follow-up following the end of the study.

Participants will be treated with either insulin only (control arm) for the whole duration of the study or with insulin and semaglutide weekly s.c. injection (titrated up to 1 mg or the maximum tolerated dose, see below) for the first six months then either dapagliflozin

(Triple therapy arm) or placebo (dual therapy) daily will be added for an additional six months. Semaglutide will be started at a dose 0.25 mg once weekly for two weeks then increased to 0.5 mg dose. If well tolerated it will be increased to 1 mg once weekly at week 4. Dapagliflozin (or placebo) will be started at a dose of 5 mg daily at week 26 and if well tolerated increased after 1 week to 10 mg (2x5mg tablets) daily to ensure drug tolerability and to avoid any hypoglycemia or hypotension. Patients who do not tolerate the increase in dosing of either medications will be maintained at the highest tolerated dose.

Treatments used

1- Insulin: All patients (81 patients) will continue to self-administer their prescribed insulin as part of their standard of care. Insulin dose will be titrated during the study to control blood glucose average between 90-180 mg/dl and to avoid hypoglycemia (blood sugar <70 mg/dl) or hyperglycemia (blood sugar >250 mg/dl). Among all patients, 3827 patients will be randomized (open-label) to remain on insulin therapy only (standard therapy group) while the other 76 will start the investigational therapies.
2- Semaglutide: a once weekly a GLP-1 mimetic injection (pen) indicated for type 2 diabetes. Doses to be used: start at 0.25mg then increase to 0.5mg following 2 weeks and to 1mg weekly s.c. injection 2 weeks later. Semaglutide will be added to a background of insulin treatment for 12 months in 76 patients who will be randomized (double-blind) into 2 groups (dual and triple therapy groups) at the end of 26 weeks.
3- Farxiga (dapagliflozin): a once daily SGLT-2 inhibitor tablet indicated for type 2 diabetes. Doses used: starting with 5mg and increase after 1 week to 10mg oral administration daily. Dapagliflozin will be started according to the randomization (Interactive Web System, hosted by University of Glasgow) in 3827 patients at 26 weeks as an additional treatment on a background of insulin and semaglutide therapy and end at 52 weeks (triple therapy group).
4- Placebo: dapagliflozin matching placebo (supplied by Astra Zeneca) will be started in 27 patients at 26 weeks as an additional treatment on a background of insulin and semaglutide therapy and end at 52 weeks (dual therapy group).

As shown in **Table 1,** fasting blood and urine samples will be obtained prior to drug administration (weeks 0) and at 13, 26, 39 and 52 weeks after the commencement of the study. Optional fat biopsies from the abdominal subcutaneous tissue will be collected at weeks 0, 26 and 52. Continuous Glucose Monitoring (Dexcom G6 with blinded receiver) will be performed for three 21-days periods on weeks -3 to 0, 23 to 26 and from 49 to 52 weeks. An optional 24hr blood pressure monitoring will be performed at baseline (week -1) and at 13, 25, 39 and 51 weeks in order to characterize the time course of antihypertensive effects, which may be early (i.e. independent of weight loss) as indicated by our preliminary data. High fat high caloric (HFHC) Meal challenges will be performed at baseline (week -1) and at 25 and 51 weeks with assessment of glucose excursions and postprandial glucagon (AUC) as well as postprandial inflammation and oxidative stress. Telephone calls will be conducted at weeks 3, 5, 8, 16, 20, 29, 35, 44 and at two weeks

after the end of the study to document any side effects or any other patient's reported issues or comments.

Study Visits:

Screening Visit (Screening Visit, Week -8 to -4): Patients will be asked to read and understand the consent and any questions regarding the protocol will be answered. If they want to participate in the study, they will be asked to sign the informed consent form. Their medical history and current medications will be obtained as well as blood pressure and vitals. A physical examination will also be done. Blood samples will be taken in order to evaluate HbA1c, CBC, CMP, C-peptide, kidney and liver functions, ketone bodies, and urine for pregnancy status in females.

Study Visit 1 (Week -3): Participants who meet entry criteria will be scheduled to for their next visit at -3 week. Vitals will be taken including resting (min. 10 minutes) blood pressure measurement (will be repeated from same arm every visit). Insulin pump will be downloaded and/or CGM/fingerstick blood sugar will be reviewed and the appropriate insulin adjustments will be made by the investigator to target fasting blood glucose concentrations between 90-180 mg/dl if possible at all times without having additional hypoglycemia (less than 70 mg/dl). Blood sugars and ketone bodies will be measured via a fingerstick blood sample using glucose and ketones meter (Abbott Laboratories) and daily blood sugars collected and reviewed. Urine for pregnancy status and ketone bodies assessment. We will instruct patients on how to use and insert the Continuous Glucose Monitoring System (Dexcom G6 with blinded receiver). All subjects will be advised to monitor their capillary blood glucose by CGM or fingerstick at least 4 times a day (before meals and at bedtime) through-out the study and to wear their CGM constantly for next 20 day. Carb counting technique will be reviewed and any questions answered on how to use the pump or calculate insulin doses.

Study Visit 2 (Week -1±2days): Patients will be asked to come in the fasting state (no food or calorie intake for 10-12 hours). Their vitals will be taken. Blood sugars and ketone bodies will be measured via a CGM/fingerstick blood sample using glucose and ketones meter (Abbott Laboratories) to assess their glycemic control prior to meal challenge. If their blood sugar is below 70mg/dl or more than 250mg/dl the visit will be rescheduled for an alternative date. Insulin pump will be downloaded and/or CGM/fingerstick blood sugar will be reviewed and insulin adjustment will be made if necessary. An IV port will be placed in order to obtain blood samples throughout the duration of the visit. Patients will undergo a meal challenge test (described below). At the end of the meal test, they will be asked to wear a 24 hour blood pressure monitor (optional). A 24 hr urine container will be dispensed to collect a 24 hour urine sample to bring in at the Week 0 visit. Daily Glucose logs will be reviewed.

Study visit 3 (Week 0±2days): Patients will be asked to come in the fasting state (no food or calorie intake for 10-12 hours). Vitals will be taken. Blood sugars and ketone bodies will be measured via a CGM/fingerstick blood sample using glucose and ketones meter

(Abbott Laboratories) and daily blood sugars collected and reviewed. The 24 hour urine container will be collected. A fasting blood sample will be taken (45 mL) to evaluate study endpoints, CBC, CMP and HbA1c. A urine sample will be collected to evaluate ketone bodies and, in females, pregnancy status. Insulin pump will be downloaded and/or CGM/fingerstick blood sugar will be reviewed and the appropriate insulin adjustments will be made by the investigator. In general, insulin dose adjustments should not be >10% from current total insulin dose or >20% from baseline (week 0) total insulin dose. They will discontinue use of the study CGM. An optional fat biopsy will be performed. We will ask them to fill out quality of life questionnaires. Study drug will be dispensed depending on what group they are randomized to. This includes either (1) staying on the insulin regimen given by their doctor, or (2) a combination of their current insulin regimen and a once daily injection of semaglutide. Semaglutide will be started on site at the 0.25 mg dose. The 24 hr blood pressure monitor will be collected.

Study Visit 4 (Week 1±2days): Vitals will be taken. Blood sugars and ketone bodies will be measured via a CGM/fingerstick blood sample using glucose and ketones meter (Abbott Laboratories) and daily blood sugars collected and reviewed. Insulin dose will be adjusted if needed as in visit 3. At any time, if HbA1c is <7%, insulin dose adjustment should not be >5%. Semaglutide 0.25mg will be injected on site. Participants will be instructed to call their research center or their doctor directly in case of any problem or untoward side effects. They will be specifically asked to call if they have unmanaged hypoglycemia (blood sugar <70 mg/dl) or hyperglycemia (blood sugar >250 mg/dl) or if occurring in more than one occasion.

Study Visit 5 (Week 2±2days): Vitals will be taken. Blood sugars and ketone bodies will be measured via a CGM/fingerstick blood sample using glucose and ketones meter (Abbott Laboratories) and daily blood sugars collected and reviewed. Insulin dose will be adjusted if needed as in visit 3. Semaglutide 0.5mg dose will be injected on site. If they are experiencing mild nausea, they will be continued on the 0.25 mg dose for another week. Supply of the drug will be given to last until the next visit. Patients will be advised to increase the study dose to 0.5 mg a day if they are ok.

Study Visit 6 (Week 4±1 week): Patients will be asked to bring in used study pens for drug accountability (to be recorded in eCRF). Vitals will be taken. Blood sugars and ketone bodies will be measured via a CGM/fingerstick blood sample using glucose and ketones meter (Abbott Laboratories) and daily blood sugars collected and reviewed. Insulin dose will be adjusted if needed. Supply of the drug will be given to last until the next visit. A urine sample will be collected to evaluate ketone bodies and, in females, pregnancy status. Patients will be advised to increase the study dose to 1.0 mg/week. If they experience mild nausea, they will be continued on the 0.5 mg dose for another week. At all times, patients will be maintained at the maximum tolerated dose. A 24 hr urine container will be dispensed to collect a 24 hour urine sample to bring in at the Week 13 visit.

Study Visit 7 (Week 13±1 week): Patients will be asked to come in the fasting state (10-12 hours fasting), and to bring in your used study pens for drug accountability. Blood sugars and ketone bodies will be measured via a CGM/fingerstick blood sample using

glucose and ketones meter (Abbott Laboratories) and daily blood sugars collected and reviewed. Insulin dose will be adjusted if needed as in. Supply of the drug will be given to last until visit 26. Vitals will be taken. A urine sample will be collected to evaluate ketone bodies and, in females, pregnancy status. The 24 hour urine container will be collected. A fasting blood sample will be taken (45 mL) to evaluate study endpoints, CBC, CMP and HbA1c. Patients who agreed to participate in the24 hour blood pressure monitoring will be asked to wear it for the next 24 hr.

Study Visit 8 (Week 23±1 week): Patients will be asked to bring in used study pens for drug accountability. Vitals will be taken. Blood sugars and ketone bodies will be measured via a CGM/fingerstick blood sample using glucose and ketones meter (Abbott Laboratories) and daily blood sugars collected and reviewed. A urine sample will be collected to evaluate ketone bodies and, in females, pregnancy status. Insulin dose will be adjusted if needed. All subjects will be reminded to monitor their capillary blood glucose by fingerstick at least 4 times a day (before meals and at bedtime) through-out the study. CGM will be inserted and patients asked to wear their CGM constantly for next 20 day.

Study visit 9 (Week 25±1 week): Patients will be asked to come in the fasting state, and to bring in used study pens for drug accountability. Vitals will be taken. A urine sample will be collected to evaluate ketone bodies. Blood sugars and ketone bodies will be measured via a CGM/fingerstick blood sample using glucose and ketones meter (Abbott Laboratories) and daily blood sugars collected and reviewed. Insulin dose will be adjusted if needed. HbA1c will be drawn. Supply of the drug will be given to last until the next visit. A 24 hr urine container will be dispensed to collect a 24 hour urine sample to bring in at the Week 26 visit. A HFHC Meal challenges will be performed as in visit 2. Patients who agreed to participate will be asked to wear a 24 hour blood pressure monitoring for the next 24 hr.
Participants in the semaglutide group will be provided supplies for urine ketones measurement (KetoStix) and blood ketones meter and strips. Research coordinator will instruct the patients on the use of meter and reading of the measuring strips. Patients will be instructed to start measuring urinary and blood ketones every morning for three consecutive days prior to next visit (starting dapagliflozin treatment) and to report results at the time of their next visit.

Study visit 10 (Week 26±1 week): Patients will be asked to come in the fasting state, and to bring in used study pens for drug accountability. Previous 3 days blood and urine ketones levels will be reviewed. Patients with normal fasting urine (<+, or < small) or blood ketone (<0.6 mmol) will proceed to next treatment phase included in this visit. Patients who report higher ketone values in at least 1 out of the 3 days will have their glucose levels, insulin dose, carb intake reviewed and managed as necessary. They will be rescheduled for next week (7±1 day) and asked to repeat the 3 days testing before next visit.
Vitals will be taken. Blood sugars and ketone bodies will be reviewed via a CGM/fingerstick blood sample using glucose and ketones meter (Abbott Laboratories) and daily blood sugars collected and reviewed. Insulin dose will be adjusted if needed. In general, insulin dose adjustments should **NOT** be >10% from current total insulin dose (or 5% if HbA1c is <7%) or >20% from baseline total insulin dose. The adjusted total insulin

dose in this visit will serve as the baseline total insulin dose for the remaining 6 months of the study and future limits of dose adjustments will be based on this new baseline. The CGM will be collected. A urine sample will be collected to evaluate ketone bodies and, in females, pregnancy status. The 24 hour urine container will be collected. A fasting blood sample will be taken (45 mL) to evaluate study endpoints, CBC, CMP and C-peptide. An optional fat biopsy will be performed. We will ask patients to fill out quality of life questionnaires. At this point, those patients randomized to receive semaglutide, they will now be randomized to receive an actual dapagliflozin pill or a placebo pill that looks like dapagliflozin. If they are randomized to receive actual dapagliflozin, they will start at the 5 mg dose for one week. Supply of the study drug will be given to last until week 27.
All patients will be asked to perform a daily (preferably early morning or before bedtime) urinary test of ketone bodies using KetoStix and record all outcomes until the end of the study. Also, they will be provided with additional blood ketone strips to measure blood ketones when needed. If results of these daily scheduled (i.e. not done due to symptoms) urine ketone measurements, are small or higher (+1 or more), they will be instructed to measure blood ketones and to repeat both measurements after 60 min. If consecutive measurements show moderate or higher ketones in urine or blood ketones >0.6mM, the patient will be instructed to call the research center or their doctor directly. For any abnormal ketone levels taken due to symptoms, the DKA consensus guidelines (Danne T et al., Diabetes Care. 2019 Feb 6) are to be followed instead (as in section 21).
Participants will be instructed to call their research center or their doctor directly in case of any problem or untoward side effects. They will be specifically asked to call if they have unmanaged hypoglycemia (blood sugar <70 mg/dl) or hyperglycemia (blood sugar >250 mg/dl) or if occurring in more than one occasion. Participants will be instructed to remain hydrated and report any genital mycotic or urinary tract infections.

Study visit 11 (Week 27±2 days): Patients will be asked to bring in used study pens for drug accountability (recorded in eCRF). Vitals will be taken. Daily blood sugars and urinary ketones measurements using KetoStix will be collected and reviewed. Blood sugars and ketone bodies will be measured via a CGM/fingerstick blood sample using glucose and ketones meter (Abbott Laboratories). A urine sample will be collected to evaluate urine ketone level. Insulin dose will be adjusted if needed. In general, insulin dose adjustments should not be >10% from current total insulin dose (or 5% if HbA1c is <7%) or >20% from NEW baseline total insulin dose established at visit 10. If at any time during the study total insulin dose reduction is >20% and total insulin dose in <0.40 U/Kg, additional monitoring protocol (see later) will be implemented. Supply of the drug will be given to last until week 39. If patients are receiving actual dapagliflozin, they will now start at the 10 mg dose for the remainder of the study.

Study Visit 12 (Week 31±1 week): Patients will be asked to bring in used study pens and tablets for drug accountability. Vitals will be taken. Daily blood sugars and urinary ketones measurements using KetoStix will be collected and reviewed. Blood sugars and ketone bodies will be measured via a CGM/fingerstick blood sample using glucose and ketones meter (Abbott Laboratories). A urine sample will be collected to evaluate ketone bodies and, in females, pregnancy status. Insulin dose will be adjusted if needed as in visit 11. A

blood sample will be taken (15 mL) to evaluate CBC and CMP. A 24 hr urine container will be dispensed to collect a 24 hour urine sample to bring in at the Week 39 visit.

Study visit 13 (Week 39±1 week): Patients will be asked to come in the fasting state, and to bring in used study pens and tablets for drug accountability. Vitals will be taken. Daily blood sugars and urinary ketones measurements using KetoStix will be collected and reviewed. Blood sugars and ketone bodies will be measured via a CGM/fingerstick blood sample using glucose and ketones meter (Abbott Laboratories). Insulin dose will be adjusted if needed as in visit 11. Supply of the drug will be given to last until the end of the study. A urine sample will be collected to evaluate ketone bodies and, in females, pregnancy status. The 24 hour urine container will be collected. A fasting blood sample will be taken (45 mL) to evaluate study endpoints, CBC, CMP and HbA1c. Patients who have provided consent will be asked to wear a 24 hour blood pressure monitor.

Study visit 14 (Week 49±1 week): Patients will be asked to bring in used study pens and tablets for drug accountability. Vitals will be taken. Daily blood sugars and urinary ketones measurements using KetoStix will be collected and reviewed. Blood sugars and ketone bodies will be measured via a CGM/fingerstick blood sample using glucose and ketones meter (Abbott Laboratories). A urine sample will be collected to evaluate ketone bodies and, in females, pregnancy status. Insulin dose will be adjusted if needed as in visit 11. All subjects will be reminded to monitor their capillary blood glucose by fingerstick at least 4 times a day (before meals and at bedtime) through-out the study. CGM will be inserted and patients asked to wear their CGM constantly for the next 20 days.

Study visit 15 (Week 51±1 week): Patients will be asked to come in the fasting state, and to bring in used study pens and tablets for drug accountability. Vitals will be taken. Daily blood sugars and urinary ketones measurements using KetoStix will be collected and reviewed. Blood sugars and ketone bodies will be measured via a CGM/fingerstick blood sample using glucose and ketones meter (Abbott Laboratories). A urine sample will be collected to evaluate urine ketone levels. Insulin dose will be adjusted if needed as in visit 11. A 24 hr urine container will be dispensed to collect a 24 hour urine sample to bring in at the Week 52 visit. A HFHC Meal challenges will be performed as in visit 2. Patients will be asked to wear the optional 24 hour blood pressure monitor.

Study visit 16 (Week 52±2 days): Patients will be asked to come in the fasting state, and to bring in used study pens and tablets for drug accountability. Vitals will be taken. Daily blood sugars and urinary ketones measurements using KetoStix will be collected and reviewed. Blood sugars and ketone bodies will be measured via a CGM/fingerstick blood sample using glucose and ketones meter (Abbott Laboratories). Insulin dose will be adjusted if needed. The CGM will be collected. An optional fat biopsy will be performed. A urine sample will be collected to evaluate ketone bodies and, in females, pregnancy status. The 24 hour urine container will be collected. A fasting blood sample will be taken (45 mL) to evaluate study endpoints, CBC, CMP, HbA1c and C-peptide. We will ask the patients to fill out quality of life questionnaires. The patient will receive a physical. After this visit, they will be discharged from the study.

Safety phone calls will be conducted at weeks 3, 5, 8, 16, 20, 29, 35, 44 and at two weeks after the end of the study to document any side effects or other changes.

Table 1: Schedule of visits and testing procedures.

Visit #	-	1	2	3	4	5	6	7	8	9	10	11	12	13	14	15	16
Week#(±2-7 days as per text)	-8 to -4	-3	-1	0	1	2	4	13	23	25	26	27	31	39	49	51	52
Study Procedures	Screening	Run-in period		Randomization, start semaglutide							Randomize/Start dapagliflozin/ placebo						End of study
History	X																
Physical	X																X
Vitals	X	X	X	X	X	X	X	X	X	X	X	X	X	X	X	X	X
CBC	X			X				X			X		X	X			X
CMP	X			X				X			X		X	X			X
C-Peptide	X										X						X
Kidney and liver profiles	X			X				X			X		X	X			X
Pregnancy test	X	X		X			X	X	X		X		X	X	X		X
Study drug titration						X	X					X					
Dispense medications				X	X	X	X	X			X	X		X			
HbA1c	X			X				X		X				X			X
24 hour BP (optional)			X					X		X				X		X	
CGM		X	X						X	X					X	X	
Daily Fingerstick glucose review		X	X	X	X	X	X	X	X	X	X	X	X	X	X	X	X
Daily urinary ketones review											X	X	X	X	X	X	X
Insulin dose review		X	X	X	X	X	X	X	X	X	X	X	X	X	X	X	X
Ketone bodies (blood/urine)	X	X	X	X	X	X	X	X	X	X	X	X	X	X	X	X	X
Adverse Event reporting		X	X	X	X	X	X	X	X	X	X	X	X	X	X	X	X
Meal Challenge			X							X						X	
Blood endpoints				X				X			X			X			X
Fat biopsy (optional)				X							X						X
24 hr Urine				X				X			X			X			X
Quality of life questionnaires				X							X						X

Drug Blinding:
The randomizations will be performed using an Interactive Web System, hosted by University of Glasgow. Eighty-one (81) patients will be randomized as follows: participants will first be randomized to semaglutide plus usual insulin therapy or insulin therapy only (no placebo for semaglutide or other placebo injection will be provided), in a 2:1 ratio. Those who remain on semaglutide at six months will then be randomized in a 1:1 ratio to receive dapagliflozin or matched placebo. Each randomization will be stratified by study site and the second randomization will also include HbA1c stratification. The randomization schedules will be generated by a statistician with no other involvement in the trial, using the method of randomized permuted blocks. Only those staff responsible for the maintenance of the randomization system will have access to the randomization sequences during the trial. The first randomization (which will be revealed to the researcher at the point of randomization) will use random block sizes to reduce predictability. Researchers at study sites will have no knowledge of the next allocation to be issued. The second randomization (which will be double-blind) will use a different. At the point of randomization, the researcher will be given a medication pack ID number, but will not know whether that medication pack contains Dapagliflozin or placebo. For each randomization, a web-based randomization system (developed and maintained by the Robertson Centre for Biostatistics) will be used. The system will include an emergency unblinding facility for the dapagliflozin/placebo randomization
Astra Zeneca Inc. will supply dapagliflozin and matching placebo tablets in unlabeled and identical packaging; semaglutide pens will be obtained commercially and will be used in an open label fashion.

Breaking of the study blind will be performed only: (i) for SUSARs; and, (ii) where knowledge of the treatment is considered by local health personnel absolutely necessary for further management of the patient. A central unblinding facility based at the Robertson Centre for Biostatistics, University of Glasgow, will be available by telephone. Notification of all unblinding will be sent to the Chief Investigator

During the course of the trial there will be no attempt to identify group assignment for any participant (except for unblinding in the case of safety concerns) and the investigators will execute the protocol in the manner it was approved independent of the group assignment.

Drugs will be stored in a locked cabinet or locked temperature controlled refrigerator at 4C at the research facility of the Diabetes and Endocrinology Center of WNY. IND application for use of all drugs (dapagliflozin and semaglutide) in type 1 diabetes will be submitted to FDA and no study procedure will start before all local and federal approvals are obtained.

Clinical and Laboratory Procedures

1) **CMP, CBC, HbA1c, , glucose, glucagon, GLP-1 and FFA measurements:** Blood CMP, CBC, HbA1c, and glucose will be measured through Quest diagnostic (US site) and NHS labs (UK site). Free fatty acid levels will be measured by a colorimetric assay (Wako, Richmond, VA). GLP-1 and glucagon will be measured from samples collected in protease and DPP-IV inhibitors by ELISA kits from Millipore.

2) **Meal Challenge test:** In order to assess the postprandial changes induced by dapagliflozin, semaglutide or the combination of both compared to insulin only, a meal challenge will be performed before starting treatments, at six months and at end of study using a 910 Calorie High fat High carbohydrate meal (HFHC) as in several of our previous studies (HFHC meal includes an egg muffin sandwich, a sausage muffin sandwich and two hash browns which contain 88g carbohydrate, 51 g fat (33% saturated) and 34 g protein). Participants will be asked to attend fasting prior to breakfast insulin bolus; basal insulin will continue during the duration of the meal, however patients will not bolus until the 3 hour mark unless blood glucose values reach above 400 mg/dL. Sequential blood samples will be obtained at 0, 15, 30, 45, 60, 90, 120, 150 and 180 min after consuming the meal. Samples at 15, 30, 45, 90 and 150 min will be 5ml while those at 0, 60, 120 and 180min will be 30 ml (total volume=145 ml). Spot urine will be collected at baseline, 1 and 3hr. Blood will be collected from an indwelling intravenous canula in a superficial forearm vein.
3) **Optional 24 hour Ambulatory BP monitoring:** Participants will be instructed on the use of 24 hour ambulatory BP monitoring device. This device will measure BP and pulse every 30 minutes for 24 hours. In addition to the scheduled measurements there will be an option for additional manual measurements if systolic blood pressure is >160 or <90 mmHg.
4) **Optional Fat aspiration procedure:** Abdominal subcutaneous fat tissue aspiration will be performed at a 10 cm distance from umbilicus. Participants will be asked not to take aspirin or NSAIDS in the previous 72 hours - if they have, the procedure will not be done. Skin will be prepared with povidone-iodine (Betadine) and alcohol. A sterile drape will be placed around the appropriate area. 3cc of 1% lidocaine will then be administered subcutaneously (the dose will not exceed 4.5mg/kg body weight). After adequate anesthesia has been achieved, aspiration of fat tissue will then be with a 3-holed canula (Tulip Instrumentation, length: 15cm, diameter: 2.1mm) fixed to a 10mL syringe. After getting adequate fat tissue (500mg-3g), the puncture site will be pressed for at least ten minutes before the patient rises up from supine position (to minimize bruising). Participants will then be discharged home. Adipose tissue will be centrifuged to remove blood and fluid contaminants. Upper adipose tissue will be collected into a separate sterile tube, washed twice with cold sterile Phosphate Buffered Saline (PBS) and centrifuged to remove the PBS. The adipose tissue sample will be weighed and approximately 500 mg transferred to a separate tube for analysis. Total RNA, nuclear extracts and total cell lysates will be prepared.
5) **Questionnaires:** Participants will be asked to complete the standard Diabetes Specific Quality of Life Scale (DSQOLS) and Problem Areas In Diabetes survey (PAID). Questionnaires attached at the end of this plan.
6) **MNC isolation (for future ancillary studies):** Blood samples will be collected in Na-EDTA as an anticoagulant. Three and a half mL of anticoagulated blood sample are carefully layered over 3.5 mL of PMN medium (Cedarlane Laboratories, Hornby, ON) and then centrifuged. At the end of this process, two bands separate out at the top of the RBC pellet. The top band consists of MNC, while the bottom consists of PMN. The MNC band is harvested and washed twice with Hank's balanced salt solution (HBSS). This method provides yields greater than 95% pure PMN and MNC suspensions.

7) **NFκB DNA binding activity (for future ancillary studies):** Nuclear NFκB DNA binding activity is measured by EMSA. Nuclear extract is prepared from MNC and adipose tissue by high salt extraction as described by Andrews et al (17). The specificity of the bands is confirmed by supershifting these bands with specific antibodies against Rel-A (p65) and p50 (Santa Cruz Biotechnology, CA) and by competition with cold oligonucleotides. DNA binding activity of NFκB will be adjusted to Oct1 DNA binding activity to correct for any variable.

8) **Plasma concentrations of inflammatory mediators (for future ancillary studies):** Concentrations of TNFα, IL-1β, IL-18, adiponectin, IL-1RA will be measured in plasma using ELISA kits (R&D Systems, MN). CRP and SAA concentrations will be measured using an ELISA kit from Alpha Diagnostics International Inc. (San Antonio, TX).

9) **Plasma vasodilators and vasoconstrictors (for future ancillary studies):** Plasma ANP, NT-proBNP, b-NP, cGMP, cAMP, angiotensinogen, renin and angiotensin II concentrations will be measured by commercially available ELISA kits.

10) **15-isoprostane F_{2t}** (also known as 8-epi-PGF2α or 8-iso-PGF2α) **(for future ancillary studies):** will be measured in urine samples using an ELISA kit from Oxford Biomedical Research (Oxford, MI).

11) **Quantification of mRNA in MNC and adipose tissue (for future ancillary studies).** Total RNA is isolated from MNC and adipose tissue using commercially available RNAqueous®-4PCR Kit (Ambion, Austin, TX). The quality and quantity of the isolated RNA is determined before using the RNA. Real Time RT-PCR is performed using Cepheid Smart Cycler (Sunnyvale, CA) in which 2 µL cDNA, 10 µL Sybergreen Master mix (Qiagen, CA) along with 0.5 µL of 20 µM gene specific primers for IL-1β, IL-RA, TLR-4, TNFα, IL-12, SOCS-3 and JNK-1 (Life Technologies, MD) are used. The specificity and the size of the PCR products are tested by adding a melt curve at the end of the amplifications and by running it on a 2% agarose gel. All values are normalized to 18S expression and β-actin.

10.2 Describe what data will be collected.

NOTE: For studies with multiple data collection points or long-term follow up, consider the addition of a schedule or table in your response.

Response:

Data will be collected as described in table 1 above. The following aspects of the study will be performed and data collected and reported by patients:

1- A CGM device will be provided for use for 3 20-days periods. Detailed instructions on changing the sensor at 10 days will be provided. Patients will be instructed to wear their CGM constantly during the assigned study periods. They will be asked to keep a diary of their food intake to measure their carbohydrate intake during the CGM period. Insulin doses will be titrated aiming for blood glucose between 90-180 mg/dl while avoiding hypoglycemia (<70 mg/dl) or hyperglycemia (>250 mg/dl).

2- Participants who agree to participate in the 24-hr ambulatory blood pressure monitoring will be provided with a 24-hour ambulatory BP monitor to be worn for 24 hours during week -1, 13, 25, 39 and 51.
3- All participants will be asked to monitor capillary blood glucose by finger stick before each meal and at bedtime.
4- For the entire duration of the study, the patients will maintain a diary to record any hypoglycemia and other untoward side effects e.g. nausea, change in appetite.
5- Starting at the 6 months mark, participants will be asked to perform a daily urinary test of ketone bodies using KetoStix and record all outcomes. If results are moderate to large, they will be instructed to repeat the test and to call the research center or their doctor directly if results are confirmed. Patients with dual glucose and ketones meters will be instructed to record and report their blood ketones as well. If blood ketones are above 0.6mM, they should repeat the test and contact the research center of their doctor if levels are confirmed.

10.3 List any instruments or measurement tools used to collect data (e.g. questionnaire, interview guide, validated instrument, data collection form).

Include copies of these documents with your submission.

Response: Source documents will be used to collect patient information. The CGM or insulin pump will be used to collect glucose and insulin data. Glucose and ketone meter will be used to measure glucose and ketone in blood. KetoStix for measuring ketones in urine. Blood pressure monitor will be used for 24 hour blood pressure reading. Glucose, food and urinary ketones diaries will be provided for patients to record their results.

10.4 Describe any source records that will be used to collect data about subjects (e.g. school records, electronic medical records).

Response: electronic medical records and research files.

10.5 Indicate whether or not ***individual*** *subject results, such as results of investigational diagnostic tests, genetic tests, or incidental findings will be shared with subjects or others (e.g., the subject's primary care physician) and if so, describe how these will be shared.*

Response: Individual participant lab results will be disclosed to the participant upon their request. If the participant requests documentation be shared with another physician, physician office or hospital the participant must come to the research center to collect said documentation or the documentation can be mailed to their given home address.

Patients will be assigned a unique study number. All identifiable personal information will be stored locally in locked filing cabinets. Data entered on the

electronic Case Report Form will only be identified by the unique study number. Thus, no personal information will be shared with the data center (Robertson Centre for Biostatistics, University of Glasgow, UK).

10.6 Indicate whether or not ***study*** *results will be shared with subjects or others, and if so, describe how these will be shared.*

Response: Not Applicable. Study results will not be shared with the subjects. However, unidentifiable study results could be published in the form of a manuscript or abstract and will be reported to JDRF, Astra Zeneca, and to clinicaltrials.gov and other regulatory agencies including FDA.

11.0 Study Timelines

11.1 Describe the anticipated duration needed to enroll all study subjects.

Response: Accrual is expected to be at the rate of 2 subjects/month/site, thus we should complete enrollment of 57 patients/site in approximately 26-30 months. We expect to complete all study clinical activities in 48 months.

11.2 Describe the duration of an individual subject's participation in the study. Include length of study visits, and overall study follow-up time.

Response: 55 Weeks. Study visits will be 60-90 minutes long except for visits 2, 9 and 15 which will be about 4hr long each

11.3 Describe the estimated duration for the investigators to complete this study (i.e. all data is collected and all analyses have been completed).

Response: 60 Months

12.0 Setting

12.1 Describe all facilities/sites where you will be conducting research procedures. Include a description of the security and privacy of the facilities (e.g. locked facility, limited access, privacy barriers). Facility, department, and type of room are relevant. Do not abbreviate facility names.

NOTE: Examples of acceptable response may be: "A classroom setting in the Department of Psychology equipped with a computer with relevant survey administration software," "The angiogram suite at Buffalo General Medical Center, a fully accredited tertiary care institution within New York State with badge access," or, "Community Center meeting hall."

Response:

1) Research will be conducted at the Diabetes Endocrinology Research Center of WNY, located at *1000 Youngs Road, Suite 105, Williamsville NY 14221* and at the CTRC located in 875 Ellicott St. Buffalo NY14203.The Diabetes Research Center has facilities and exam rooms available for insulin pump download, CGM device download, meal and infusion studies and presence of study coordinator and registered nurse for data collection and blood work at all times. One of the investigators will be available at all times to address patients' related issues. CTRC location is a fully equipped laboratory with Equipment include ultra-low freezers for sample storage, centrifuges, microscopes for sample preparation, infusion pumps, ELISA, PCR and immunoblotting instrumentation.

2) Research will also be conducted at the NHS Greater Glasgow and Clyde Clinical Research Facility (CRF, Glasgow Royal Infirmary, UK and at the Diabetes Centre (Clinic D), Stobhill Ambulatory Care Hospital, Glasgow, UK. There are facilities and exam rooms available for insulin pump download, CGM device download, meal and infusion studies and presence of research nursing staff for data collection and blood work at all times. One of the investigators will be available at all times to address patients' related issues. The CRF has equipment for sample preparation, infusion pumps, ultra-low freezers for sample storage and centrifuges. ELISA, PCR and immunoblotting facilities are available in adjacent NHS and University facilities.

12.2 For research conducted outside of UB and its affiliates, describe:

- *Site-specific regulations or customs affecting the research*
- *Local scientific and ethical review structure*

NOTE: This question is referring to UB affiliated research taking place outside UB, i.e. research conducted in the community, school-based research, international research, etc. It is not referring to multi-site research. UB affiliated institutions include Kaleida Health, ECMC, and Roswell Park Cancer Institute.

Response:

☒ **N/A:** This study is not conducted outside of UB or its affiliates.

13.0 Community-Based Participatory Research

13.1 Describe involvement of the community in the design and conduct of the research.

NOTE: Community-Based Participatory Research (CBPR) is a collaborative approach to research that equitably involves all partners in the research process and recognizes the unique strengths that each brings. CBPR begins with a research topic of importance to the community, has the aim of combining knowledge with action and achieving social change to improve health outcomes and eliminate health disparities.

Response:

☒ **N/A:** This study does not utilize CBPR.

13.2 Describe the composition and involvement of a community advisory board.

Response:

☒ **N/A:** This study does not have a community advisory board.

14.0 Resources and Qualifications

14.1 Describe the qualifications (e.g., education, training, experience, expertise, or certifications) of the Principal Investigator ***and*** *staff to perform the research. When applicable describe their knowledge of the local study sites, culture, and society. Provide enough information to convince the IRB that you have qualified staff for the proposed research.*

NOTE: If you specify a person by name, a change to that person will require prior approval by the IRB. If you specify a person by role (e.g., coordinator, research assistant, co-investigator, or pharmacist), a change to that person will not usually require prior approval by the IRB, provided that the person meets the qualifications described to fulfill their roles.

Response: All study personnel are educated, trained, and licensed as required for their delegated role in this study. All study personnel have also received the required university training and will be trained by the PI before the study starts. The PIs, CO-investigators and research team have conducted similar studies in this patient population using similar agents and have the expertise to execute the study protocol and manage any adverse events.

Describe other resources available to conduct the research.

14.2 Describe the time and effort that the Principal Investigator and research staff will devote to conducting and completing the research.

NOTE: Examples include the percentage of Full Time Equivalents (FTE), hours per week. The question will elicit whether there are appropriate resources to conduct the research.

Response: The principal investigator supervises the research project and weekly research meetings are conducted to discuss the recruitment rate, resolve and discuss issues related to the conduct, safety, analysis of the study and related publications. PI is expected to spend 15% of his academic time on this research. The co-investigators, study coordinator and research staff provide coverage to the research related activity for 365 days a year.

14.3 Describe the availability of medical or psychological resources that subjects might need as a result of anticipated consequences of the human research, if applicable.

NOTE: One example includes: on-call availability of a counselor or psychologist for a study that screens subjects for depression.

Response: Available medical literature will be provided as deemed appropriate or requested by patient through UB libraries, PubMed, Google scholar as all the investigators have access to medical literature through listed resources above

The patient will also have access to physician (Investigators and Co-Investigators) who will be available to address any adverse effects or other questions during the course of the study who will be available to address any adverse effects or other questions during the course of the study

14.4 Describe your process to ensure that all persons assisting with the research are adequately informed about the protocol, the research procedures, and their duties and functions.

Response: Before beginning of enrollment all research staff will be asked to review the protocol and a training session will be conducted to provide protocol details, explain technical points and answer staff questions. All training session will have a sign in sheet to document such training. Continuous education through training meetings, conferences and discussions will continue through the study period and when revision are made to protocol.

15.0 Other Approvals

15.1 Describe any approvals that will be obtained prior to commencing the research (e.g., school, external site, funding agency, laboratory, radiation safety, or biosafety).

Response: FDA IND filling is required.

☐ **N/A:** This study does not require any other approvals.

16.0 Provisions to Protect the Privacy Interests of Subjects

16.1 Describe how you will protect subjects' privacy interests during the course of this research.

NOTE: Privacy refers to an individual's right to control access to him or herself. Privacy applies to the person. Confidentiality refers to how data collected about individuals for the research will be protected by the researcher from release. Confidentiality applies to the data.

Examples of appropriate responses include: "participant only meets with a study coordinator in a classroom setting where no one can overhear", or "the participant is reminded that they are free to refuse to answer any questions that they do not feel comfortable answering."

Response: Our clinical providers involved in the study will identify potential patients for recruitment from the Diabetes-Endocrinology Center of WNY Clinics according to the inclusions and exclusion criteria and through advertisements. Patient who qualify will be asked in private during their one on one consultation time with the physician if they wish to participate in the research study. If the patient agrees, the research coordinator will contact them privately for a possible telephone screening and ask if time is appropriate for this discussion. The patients who call for potential participation in the study due to advertisement flyers will be screened over the phone with the research coordinator, using our telephone screening form.

When the patient is being seen at our clinics for the first time they sign the "Consent to use and disclosure of protected health information" form which clearly states that their protected health information (PHI) can be used for review in preparation for possible research. Additionally, a partial HIPAA waiver will be obtained to access medical records when screening for patients.

If the patient passes the telephone screening, they will be asked to make an appointment to review and sign the consent. Patient will do this in a private room in the research unit and will be allowed to discuss the consent in detail with the research coordinator and or study doctor. Patient will be notified that it is completely voluntary to participate in the research study and can withdraw at any time.

We will not be accessing any medical information of the patients for whom the services are not provided by our clinic providers before partial HIPAA is obtained or IC is signed.

16.2 Indicate how the research team is permitted to access any sources of information about the subjects.

NOTE: Examples of appropriate responses include: school permission for review of records, consent of the subject, HIPAA waiver. This question ***does apply*** *to records reviews.*

Response: Consent of the subject and partial HIPAA waiver.

17.0 Data Management and Analysis

17.1 Describe the data analysis plan, including any statistical procedures. This section applies to both quantitative and qualitative analysis.

Response:

Data (e.g. date of diagnosis, past medical history, drug treatment) extracted from local clinical records will be entered on to the eCRF under the study-specific coded identifier according to structured questions as stipulated in the Study Protocol. Biological samples (blood, urine and adipose tissue aspirates) will be collected from the study participants at the study visits according to the Schedule of Assessments in the Study Protocol.

Measures relating to safety and routine care of the participants (e.g., biochemical measures of glycemic and lipidemic control, kidney function/ fluid balance, hepatic function, hematological variables) will be analysed at local clinical laboratories in order that they are available to study nurses and physicians. The confidentiality of these data will be secure, meeting or exceeding the regulations of the relevant Ethical Review Committees/ Institutional Review Boards. Samples for measurement of key study outcomes will also be measured at the central laboratory at the University of Buffalo. Blood, urine and other samples will be aliquoted into cryovials at site for freezing and storage. Electronic stored material (e.g., patient records, data from blood and urine samples, and data from Continuous Glucose Monitoring) will only be stored using the study specific coded identifier. Data will not be identifiable at the participant level without additional access to the code. The code will be available only to the investigators and the personnel performing the study. Access to any data held electronically will require study specific log-in codes. These are managed and logged by the Robertson Centre for Biostatistics, University of Glasgow, UK

Statistical plan. The primary objective of the proposed research is to evaluate the effect of addition of dapagliflozin to semaglutide on a background of insulin treatment in type 1 diabetes patients on HbA1c at six months following start of dapagliflozin. The similarities between the study groups, baseline values for subject's demographics and study endpoints will be compared using appropriate parametric tests. Transformations of the data in order to meet statistical assumptions may be considered. The primary endpoint of the study is to detect a difference in mean HbA1c change after six months of dapagliflozin or placebo addition to semaglutide and insulin treatment. Statistical analysis will be carried out using t-tests to detect difference between groups or the Wilcoxon test for paired data. Tests will be two-sided and tested at the 0.05 level. The results will be summarized as mean±SE. All randomized patients will be included in the analysis of the primary end point based on an Intent-to-treat model.

The secondary endpoints will be to compare changes in HbA1c between each of the triple therapy and dual therapy groups compared to standard of care group, mean weekly glucose concentrations, percent time spent in different glycemic thresholds, variability as measured by standard deviation, insulin dose, body weight, carbohydrate intake, blood pressure, and 24-hour blood pressure and QOL changes among the groups. A reduction in the glucose and glucagon AUC following the meal will constitute another secondary end point in this setting, ANCOVA analysis will be used. Since the secondary end-points are pre-specified and may as well be correlated, multiplicity control for multiple tests across secondary endpoints may not be necessary. However, Holm-Bonferroni correction

for multiplicity testing will be implemented if additional endpoints are tested or if the pre-specified secondary end-points are found to be not correlated.

17.2 *If applicable, provide a power analysis.*

NOTE: This may not apply to certain types of studies, including chart/records reviews, survey studies, or observational studies. This question is asked to elicit whether the investigator has an adequate sample size to achieve the study objectives and justify a conclusion.

Response:

Sample size determination is based in part on our current data showing that in type 1 diabetes, treatment with a GLP-1 agonist, liraglutide (1.2 and 1.8mg doses) for 3 months leads to HbA1c suppression by an average of 0.6% (SD= 0.5%). Dapagliflozin was recently shown to reduce HbA1c by 0.45% in type 1 diabetes patients (13). Additionally, empagliflozin treatment for 8 weeks was shown to reduce HbA1c by 0.4% in type 1 diabetics (20, 21) and our current data showing additional improvement in HbA1c by an additional 0.66% at 12 weeks when dapagliflozin was added to liraglutide and insulin therapy. Conservatively estimating that the combination therapy will suppress HbA1c by 0.5% (SD =<0.53%) compared to semaglutide alone (t-test), a sample size of 3827 (22-23 evaluable patients) in each group will be sufficient to provide adequate power (β = 0.2) to detect a statistically significant difference (α = 0.05) taking in consideration a 15% dropout rate.

17.3 *Describe any procedures that will be used for quality control of collected data.*

Response:

An electronic case report form (e-CRF) will be used to collect study data at each site. The e-CRF will be developed by the study Data Centre at the Robertson Centre for Biostatistics, University of Glasgow. Access to the e-CRF will be restricted, with only authorised site-specific personnel able to make entries or amendments to their patients' data. It is the investigator's responsibility to ensure completion and to review and approve all data captured in the e-CRF.

Data will be validated at the point of entry into the e-CRF and at regular intervals during the study. Data discrepancies will be flagged to the study site coordinator and any data changes will be recorded in order to maintain a complete audit trail (reason for change, date change made, who made change).

The Robertson Centre for Biostatistics at the University of Glasgow will be responsible for collating and cleaning the data for the study. The Robertson Centre will also be responsible for data back-up and security. This center will also manage the electronic reporting of SUSARS on behalf of the sponsor.

To enable evaluations and/or audits from regulatory authorities, the investigators agree to keep records, including the identity of all participating subjects (sufficient information to link records, all original signed informed consent forms serious adverse event forms, source documents, and detailed records of treatment disposition. The records should be retained by the study country coordinators and investigator according to ICH GCP, local regulations, or as specified in the Clinical Study Agreement, whichever is longer.

Data from the study will be stored by the Chief Investigator for a minimum of 10 years. A final report of the study will be provided to the FDA and the MHRA as per requirements.

- No interim analysis is planned for efficacy so termination of study will be related to safety. Significant increases in hypoglycemic or DKA events as reviewed by DSMB might trigger termination or modification of study.
- Last observation carry forward will be used to fill missing data for the primary end point and some secondary end points. Missing data will not be filled for other endpoints.
- Any deviation(s) from the original statistical plan will be described and justified in the protocol and/or in the final report.
- All subjects entered into treatment will analyzed for primary end point on ITT bases. For secondary endpoints, analysis of completed and evaluable patients will performed per-protocol.

18.0 Confidentiality

A. Confidentiality of Study Data

Describe the local procedures for maintenance of confidentiality of ***study data and any records that will be reviewed for data collection****.*

18.1 A. Where and how will all data and records be stored? Include information about: password protection, encryption, physical controls, authorization of access, and separation of identifiers and data, as applicable. Include physical (e.g. paper) ***and*** *electronic files.*

Response: All data records will be stored on protected computers with unique passwords issued for each of the study team members and or in locked cabinets within the research center. These files will only be accessible by authorized study personnel.

The TriNetX/i2b2 data will be protected by only accessing identifiable contact information remotely on IHI’s “virtual machine”, so the data never leaves their secure encrypted server. Recruitment letters will be generated in a mail merge behind the firewall on IHI’s virtual machine. No patient data from TriNetX/i2b2 will be removed from the IHI server or saved in other locations.

18.2 A. How long will the data be stored?

Response: De-identified data storage has no expiration date and will be stored for a minimum of 7 years. The researchers may continue to rely on this for future use in research study.

18.3 A. Who will have access to the data?

Response: Those physicians, nurses, and laboratory staff that are on all documentation for the study will have access to the data.

18.4 A. Who is responsible for receipt or transmission of the data?

Response: Those physicians, nurses, and laboratory staff that are on all documentation for the study will have access to the data and can handle transfer of data. Study data will be transferred electronically (through eCRFs) to Robertson Centre for Biostatistics, University of Glasgow, UK

18.5 A. How will the data be transported?

Response: All data are stored at locally and will be transferred to Robertson Centre for Biostatistics, University of Glasgow, UK for data management and analysis. The transfer will be done through electronic CRFs processed on secure network.

Data will also be transported unless when is being archived. At that point files will be transferred to Iron Mountain for storage and archiving.

B. Confidentiality of Study Specimens

Describe the local procedures for maintenance of confidentiality of ***study specimens****.*

☐ **N/A:** No specimens will be collected or analyzed in this research. *(Skip to Section 19.0)*

18.6 B. Where and how will all specimens be stored? Include information about: physical controls, authorization of access, and labeling of specimens, as applicable.

Response: The specimens will be stored in the laboratory located at *1000 Youngs Road, Suite 105, Williamsville NY 14221*and at the CTRC located in 875 Ellicott St. Buffalo NY14203. Samples will be stored in a locked -80º C freezer. All

specimens collected at the Glasgow site will be stored in NHS -80º C freezers within the eCRF until shipping to Buffalo (World Courier).

18.7 B. How long will the specimens be stored?

Response: Specimens storage has no expiration date and will be stored for a minimum of 7 years. The researchers may continue to rely on this for future use in research study

18.8 B. Who will have access to the specimens?

Response: Those physicians, nurses, and laboratory staff that are on all documentation for the study will have access to the specimens

18.9 B. Who is responsible for receipt or transmission of the specimens?

Response: Those physicians, nurses, and laboratory staff that are on all documentation for the study will have access to the specimens and can handle transfer of samples. Sample/specimens will transported from University of Glasgow site to the university of Buffalo CTRC site (875 Ellicott St, Buffalo, NY 14203) for storage in -80C until analyzed.

18.10 B. How will the specimens be transported?

Response: All study samples will be centrally stored in UB CTRC site. Therefore, samples collected at the UK site will be transported for analysis and final long-term storage. Transport will be done so using dry ice in a properly labeled Styrofoam container by the laboratory technician. Agreement regarding shipping and receiving the specimens form UK site will be part of the subcontract agreement.

19.0 Provisions to Monitor the Data to Ensure the Safety of Subjects

☐ **N/A:** This study is not enrolling subjects, or is limited to records review procedures only. This section does not apply.

NOTE: Minimal risk studies may be required to monitor subject safety if the research procedures include procedures that present unique risks to subjects that require monitoring. Some examples include: exercising to exertion, or instruments that elicit suicidality or substance abuse behavior. In such cases, N/A is not an acceptable response.

19.1 Describe the plan to periodically evaluate the data collected regarding both harms and benefits to determine whether subjects remain safe.

Response:

An independent Data safety and monitoring board (DSMB) will be established for this trial. The board will consist of independent faculty members from UB and other institutions with sufficient knowledge of the field of type 1 diabetes but not involved in the study. Data Management team at Robertson Centre will periodically provide the data on which to base regular reports to the Data and Safety Monitoring Board. The actual reports will then be generated by the unblinded statistician. The DSMB looks only at safety endpoints and not at futility. The DSMB will convene every six months to review safety end points, side-effects experienced by study subjects and any adverse events reported. The site PIs, CO-Is and study coordinator(s) will also continuously monitor the data and safety of subjects enrolled in the study. The site PIs and/or the DSMB may recommend to terminate the study or specific aspects of the study if clear harm is observed with one treatment option compared to standard of care or placebo. Specific safety endpoints that will be reviewed include increases in hypoglycemic and ketosis/DKA events.

19.2 Describe what data are reviewed, including safety data, untoward events, and efficacy data.

Response:

Assessment of Efficacy

1- glycemic indices including HbA1c, post-prandial glucose excursions and glucagon. CGM data will be collected as efficacy parameter of glucose control. Total insulin dose will be also documented and analyzed.
2- body weight
3- blood pressure and use of blood pressure medications
4- QOL questionnaires.

The timing of recording of the above efficacy parameters is described in table 1.

Assessment of Safety

The following safety assessments assays and measurement will be reviewed:

1- Vitals, CBC, CMP and markers kidney and liver function will be collected at screening, during and at end of the study as in table 1.
2- Assessment of hypoglycemia: Glucose data will be reviewed from CGM and daily finger stick and during visits.
3- Assessment of ketosis: Ketones levels will be reviewed from daily urinary ketone diaries or blood ketone meters and from urine and blood at the time of each visit
4- Reports of side effects by patients.
5- Insulin dose titrations and pump downloads at each visit

The methods and timing for assessing, recording, and analyzing safety parameters.

The DSMB will convene every six months to assess the side-effects experienced by study subjects and any adverse events reported. The PIs, CO-Is and study coordinator(s) will also continuously monitor the safety of subjects enrolled in the study during their visits and during the safety phone calls.

Procedures for eliciting reports of and for recording and reporting adverse event and inter-current illnesses.

All adverse events will be reported at the time of the visit or by telephone when it occurs. Medical events that occur between the signing of the Informed Consent and the first intake of study drug will be documented in the medical history. Participants will be asked to report any adverse events in general, non-directed questioning. For each AE volunteered by the subject, the investigator will obtain all the information required to complete the adverse event page of the eCRF, in accordance with the guidelines that accompany it. All adverse events, regardless of seriousness, severity, or presumed relationship to study therapy, will be recorded using medical terminology in the source document. Whenever possible, diagnoses will be given when signs and symptoms are due to a common etiology (e.g., cough, runny nose, sneezing, sore throat, and head congestion should be reported as "upper respiratory infection). Investigators will record on the CRF their opinion concerning the relationship of the adverse event to study therapy. The principal investigators assumes responsibility for appropriate reporting of AEs to the regulatory authorities. The investigator will report these events to the appropriate Independent Ethics Committee/Institutional Review Board (IEC/IRB) in accordance with local regulations. All measures required for AE management will be recorded in the source document and reported according to the State University of New York at Buffalo instructions. Non-serious events/problems that impacted on the rights and welfare of the subjects will be reported as they occur or at the time of knowledge or notification.

Definition of adverse events

The International Conference on Harmonization (ICH) Guideline for Good Clinical Practice (GCP) E6(R1) defines an AE as:

Any untoward medical occurrence in a patient or clinical investigation patient administered a pharmaceutical product and which does not necessarily have a causal relationship with this treatment. An AE can therefore be any unfavorable and unintended sign (including an abnormal laboratory finding), symptom, or disease temporally associated with the use of a medicinal product, whether or not considered related to the medicinal product.

The term AE is used to include both serious and non-serious AEs.

Definition of serious adverse events

A serious adverse event is an AE occurring during any study phase (i.e., screening, run-in, treatment, wash-out, follow-up), at any dose of the study drugs that fulfils one or more of the following criteria:

- Results in death
- Is immediately life-threatening
- Requires in-patient hospitalization or prolongation of existing hospitalization
- Results in persistent or significant disability or incapacity
- Is a congenital abnormality or birth defect in offspring of the patient

- Is an important medical event that may jeopardize the patient or may require medical intervention to prevent one of the outcomes listed above.
- TO THE AUTHOR: The causality of SAEs (their relationship to all study treatment/procedures) will be assessed by the investigator(s) and communicated to AstraZeneca. This is requested to be on the AE cover sheet.

The following variables are recommended to be collected for each AE:

- AE (verbatim)
- The date when the AE started and stopped
- Whether the AE is serious or not
- Action taken with regard to IPs
- Administration of treatment for the AE
- Outcome

In addition, the following variables are recommended to be collected for SAEs:

- Date the AE met criteria for SAE
- Date the Investigator became aware of the SAE
- Seriousness criteria fulfilled
- Date of hospitalization
- Date of discharge
- Probable cause of death
- Date of death
- Whether an autopsy was performed
- Causality assessment in relation to study procedure(s)
- Causality assessment in relation to other medication, as explained in Section 1.4.2.
- Description of the SAE

Reporting of serious adverse events

All SAEs will be reported, whether or not considered causally related to the investigational product, or to the study procedure(s). The reporting period for SAEs is the period immediately following the time that written informed consent is obtained throughout the treatment period and including the designated follow-up period as per the study protocol. The investigator is responsible for informing the Ethics Committee and/or the Regulatory Authority of the SAE as per local requirements.

Note that all serious or unexpected adverse events must be reported to AstraZeneca regardless of the country where the study is conducted.

The investigator must inform the FDA, via a MedWatch form, of any serious or unexpected adverse events that occur in accordance with the reporting obligations of 21 CFR 312.32 , and will concurrently forward all such reports to AstraZeneca. A copy of the MedWatch report must be emailed to AstraZeneca (TCS vendor) at the time the event is reported to the FDA. It is the responsibility of the investigator to compile all necessary information and ensure that the FDA receives a report according to the FDA reporting requirement timelines and to ensure that these reports are also submitted to AstraZeneca at the same time.

A cover page should accompany the MedWatch form indicating the following:

- "Notification from an Investigator Sponsored Study"
- The investigator IND number assigned by the FDA
- The investigator's name and address
- The trial name/title and AstraZeneca ISS reference number (ESR-##-#####)

Investigator must also indicate, either in the SAE report or the cover page, the causality of events in relation to all study medications and if the SAE is related to disease progression.

Send SAE report and accompanying cover page by way of email to AstraZeneca's designated mailbox: AEMailboxClinicalTrialTCS@astrazeneca.com

If a non-serious AE becomes serious, this and other relevant follow-up information must also be provided to AstraZeneca and the FDA.

Serious adverse events that do not require expedited reporting to the FDA still need to be reported to AstraZeneca preferably using the MedDRA coding language for serious adverse events.

The investigators will either provide a copy of the randomization code/ code break information or unblind those SAEs which require expedited reporting

Overdose

An overdose is defined as a patient receiving a dose of IP in excess of that specified in the Investigator's Brochure, unless otherwise specified in this protocol.

Any overdose of a study patient with the investigational product, with or without associated AEs/SAEs, is required to be reported within 24 hours of knowledge of the event to AstraZeneca Patient Safety using the designated Safety e-mailbox (see Section 1.5 or contact information). If the overdose results in an AE, the AE must also be recorded as an AE. Overdose does not automatically make an AE serious, but if the consequences of the overdose are serious, for example death or hospitalization, the event is serious and must be recorded and reported as an SAE (see Section 1.3). Any specific details around overdose for combination or comparator agents will also be provided as applicable. The investigator will use clinical judgment to treat any overdose.

Hepatic function abnormality

Hepatic function abnormality that fulfills the biochemical criteria of a potential Hy's Law case in a study patient, with or without associated clinical manifestations, is recommended to be reported as "hepatic function abnormal" within 24 hours of knowledge of the event to AstraZeneca Patient Safety using the designated Safety e-mailbox (see Section 1.3), unless a definitive underlying diagnosis for the abnormality (e.g., cholelithiasis or bile duct obstruction) that is unrelated to investigational product has been confirmed. The criteria for a potential Hy's Law case is Aspartate Aminotransferase (AST) or Alanine Aminotransferase (ALT) ≥3x Upper Limit of Normal (ULN) together with Total Bilirubin (TBL) ≥2xULN at any point during the study following the start of study medication irrespective of an increase in Alkaline Phosphatase (ALP).

Pregnancy

If a patient becomes pregnant during the course of the study, the IPs should be discontinued immediately.
Pregnancy itself is not regarded as an AE unless there is a suspicion that the IP under study may have interfered with the effectiveness of a contraceptive medication. Congenital abnormalities or birth defects and spontaneous miscarriages should be reported and handled as SAEs. Elective abortions without complications should not be handled as AEs. The outcome of all pregnancies (spontaneous miscarriage, elective termination, ectopic pregnancy, normal birth, or congenital abnormality) should be followed up and documented even if the patient was discontinued from the study.
If any pregnancy occurs in the course of the study, then the investigator should inform the appropriate AstraZeneca representatives within 1 day, i.e., immediately, but no later than 24 hours of when he or she becomes aware of it.
The investigator will ensure that all relevant information is provided to the AstraZeneca Patient Safety data entry site within 1 to 5 calendar days for SAEs and within 30 days for all other pregnancies.
The same timelines apply when outcome information is available.

19.3 Describe any safety endpoints.

Response: These specific Safety endpoints will be collected:

1- Frequency and severity of hypoglycemia: Hypoglycemia will be assessed throughout the study via multiple daily finger stick glucose measurements, CGM data collected 3 times (about 3 weeks each) during the study and from patient reports during visits and phone calls. Hypoglycemic events will be characterized as in the experts consensus published in *Diabetes Care (2017 Dec; 40(12): 1622-1630):*

- Level 1: Glucose < 70 mg/dL (3.9 mmol/L) and Glucose ≥ 54 mg/dL (3.0 mmol/L)
- Level 2: Glucose < 54 mg/dL (3.0 mmol/L)
- Level 3: A severe event characterized by altered mental and/or physical status requiring assistance.

2- Ketones levels: Urinary ketones measured daily (and any blood ketones) at home and logged in the ketones logbook. Blood and urinary ketones measured during each visit. Ketone levels will be characterized as normal, ketosis and severe ketosis:
-Normal blood ketones <0.6mM or urinary ketones of trace to small (≥+1)
-Ketosis: Blood ketones >0.6mM <2.5mM or urinary ketones of moderate to high (+2 and +3)
-Severe ketosis Blood ketones >2.5mM or urinary ketones of too high (+4)
3- Diabetes ketoacidosis episodes: any confirmed DKA episode (regardless of hospitalization) defined as:
• Elevated serum or urine ketones (greater than the upper limit of the normal range), and

• Serum bicarbonate < 15 mmol/L or Blood pH < 7.3

4- Low blood pressure: <90mmHg SBP and <50 mmHg DBP): Patients will be instructed to measure blood pressure daily and it will be will be assessed in each visit.

5- Nausea, vomiting and other GI side effects. Data will be collected by patients' safety reports, during visits and phone calls.

6- Genital mycotic infections or UTIs

7- Changes in liver and kidney function: CMP and kidney and liver profiles will be assessed as indicated in table 1.

19.4 Describe how the safety information will be collected (e.g., with case report forms, at study visits, by telephone calls with participants).

Response: The safety information will be collected at the time of the visit, and or during preplanned telephone calls with the participant or patient self-reporting (including glucose, food intake and ketones diaries). Data will be entered into eCRFs

19.5 Describe the frequency of safety data collection.

Response: Safety data collection will be done at all study visits and during preplanned phone calls as in table 1. The patient, however, will be asked to report any adverse event or safety related information via phone as soon as it occurs and it will be reviewed the same day.

19.6 Describe who will review the safety data.

Response: DSMB established for this study will review unblended safety data provided by the data management system every 6 months. Additionally, the PIs and coinvestigators will review safety data (listed above) at the completion of each visits by each subject and review any report of side effects or adverse events as they are presented by the patients. The investigators will also review collective blinded data every 3 months to assess the safety and any potential risks to the participants. Furthermore the investigators will assess other risks including the physical, psychological, social, legal and economic harm to these patients. The investigators will carefully watch for any invasion of privacy and breach of confidentiality. The principal investigator will be sharing the results of safety analysis of the study and serious adverse events with the local IRB, DSMB, JDRF, AZ and the FDA. The study groups will remain blinded. If there are a serious safety concerns or an emergency, then, and based on principal investigator discretion, study randomization center in Glasgow University or the unblended statistician will provide information about group assignment of the patient(s) involved. Un-blinded data will be reviewed by the principal investigator and co-investigators and decision to complete, withdraw or adjust dose of drug(s) will be made, documented and shared with IRB and DSMB.

19.7 Describe the frequency or periodicity of review of cumulative safety data.

Response: Safety data will be reviewed every 6 months by DSMB (un-blinded), every 3 months by PIs and CO-Is (blinded) and at every visit by study team.

19.8 Describe the statistical tests for analyzing the safety data to determine whether harm is occurring.

Response: N/A

19.9 Describe any conditions that trigger an immediate suspension of the research.

Response: No interim analysis is planned for efficacy so termination of study will be related to safety. Significant increases in severe hypoglycemic or DKA events as reviewed by DSMB are possible causes that might trigger termination of study.

20.0 Withdrawal of Subjects

☐ **N/A:** This study is not enrolling subjects. This section does not apply.

*20.1 Describe **anticipated** circumstances under which subjects may be withdrawn from the research without their consent.*

1. The site PI can stop investigational study treatment or remove participants from the trial without participants approval if for any reason he/she feels is appropriate, including: severe side effect, injury or medical condition which may place the patient at risk of further complications if he/she continue to participate, failure to take the medication as instructed, failure to keep scheduled appointments, cancellation of the study by the sponsor, IRB, FDA or other administrative reasons.
2. If any of the participants become pregnant during the period of study, they will discontinue study treatment.
3. Participants who experience persistent severe nausea or vomiting after starting semaglutide which is not managed by dose reduction will discontinue the investigational study treatment. Those with mild or moderate nausea will continue for at least one more week. If they continue to experience significant nausea (defined as interfering with normal activities), dose will be reduced. If they continue to have these side effects, they will discontinue study medication(s).
4. Persistent weight loss or failure to maintain weight if subject has lost >15% or BMI is < 22 kg/m2 in spite of down-titration of semaglutide and/or dapagliflozin.
5. Developing other conditions included in the exclusion criteria
6. Change in method of insulin delivery between insulin pumps to MDI or vice versa during the study unless pump treatment interruption is temporary for a period of at

most 2 weeks. Also, patients who upgrade to the new "artificial pancreas" pump (Medtronic's MiniMed 670G hybrid) during the study will be withdrawn.

7. Hypoglycemia: Two episodes of severe hypoglycemia (requiring others help).
8. Ketosis (with no other identifiable precipitating factors): Persistent (1week) asymptomatic or symptomatic and unmanaged (using STICH protocol) blood ketone levels >1.5 mM (or ≥+2 in urine).
9. Persistent (>1 week) total insulin dose reduction of >20% from baseline AND total insulin dose <0.4U/Kg.
10. DKA: Two episode of DKA (with no identifiable precipitating factors) as defined by the expert consensus statement (*Diabetes Care (2017 Dec; 40(12): 1622-1630)*)
11. Two episodes of UTI.
12. Two episodes of mycotic genital infection.
13. Significant persistent change in eGFR > 15 ml/min
14. Pancreatitis: One episode of pancreatitis.
15. Other persistent side effects specific to study drugs

20.2 Describe any procedures for orderly termination.

NOTE: Examples may include return of study drug, exit interview with clinician. Include whether additional follow up is recommended for safety reasons for physical or emotional health.

Response: Participation in this study is voluntary. Participants have the right to refuse to participate or to withdraw from participation at any time for any reason. Refusal to participate or to withdraw from the study will involve no penalty or loss of entitled benefits, nor affect ongoing medical care. The site PI can stop investigational study treatment or remove participants from the trial without participants approval. If a patient withdraws consent and decide not to participate in the study, she/he will be asked to undergo a final study visit for his/her safety. If a subject withdraws from the research, the data collected to that point will be used toward the research finding. If applicable the subject will have to bring back any unused research drug and or devices.

20.3 Describe procedures that will be followed when subjects withdraw from the research, including retention of already collected data, and partial withdrawal from procedures with continued data collection, as applicable.

Response: We will use the data collected up to the point of stopping the study. No further information will be collected. Patients who stop study treatment and are interested in continuing in the study will be followed up until the end of the study for the primary end point and select secondary endpoints based on the Intent-To-Treat (ITT) analysis model. Withdrawn participants will not be replaced. The study sample size calculation includes an additional 15% subjects to compensate for drop out.

21.0 Risks to Subjects

21.1 List the reasonably foreseeable risks, discomforts, hazards, or inconveniences to the subjects related to their participation in the research. Consider physical, psychological, social, legal, and economic risks. Include

a description of the probability, magnitude, duration, and reversibility of the risks.

NOTE: Breach of confidentiality is always a risk for identifiable subject data.

Response:

- Risks associated with intravenous (IV) line: All participants will be informed of the potential complications of an intravenous (IV) line, which include mild bruising at the site of IV line, usually resolving in a few days. They will also be informed about the possibility of infiltration of the IV line at the time of performing blood draws or during the meal challenge test in which case another IV line at a different site will be required (with risk of bruising at more than one site).
- Risks associated with 24 hour Ambulatory BP monitoring device: The BP cuff inflates every 15 minutes to record blood pressure over 24-hours. Participants will experience a pressure-like sensation over the arm where 24 hour BP device is worn at the time of the BP being measured due to the inflation of the cuff and release of this pressure at the time of deflation of the BP cuff. They will be informed to remove the cuff after 24 hours. If worn for longer periods they may experience mild bruising over the arm and some swelling, usually resolving 1-2 days after discontinuation.
- Risks associated with fat tissue aspiration: will probably lead to a bruise at the site of aspiration. The site may be mildly painful for 1-2 weeks. Participants are advised to call their study site staff if they have more severe pain or swelling at the site after the procedure. Rarely there are side effects such as low BP or heart rate and/or allergic reaction to local anesthetic including swelling of the throat.
- All subjects will be referred to semaglutide and dapagliflozin labeling safety information, and will be given a copy of the package insert of the medication. Discussion of the potential side effects and the different warning and precautions will take place at the time of informed consent. Patients will be informed they must not discontinue their insulin during the study.
- Potential risk of insulin, GLP-1 RA and SGLT-2 inhibitors use in type 1 diabetes patients is hypoglycemia. The combination therapy can possibly increase the magnitude and frequency of this risk.
- Potential side effects of GLP-1 RA and SGLT-2 inhibitors also include hypotension, nausea and other GI side effects, polydipsia, polyuria, dehydration, ketosis, and reduced glomerular filtration rate (GFR).
- In rodents, semaglutide causes dose-dependent and treatment-duration-dependent thyroid C-cell tumors at clinically relevant exposures. However, it received FDA-approval for the treatment of type 2 diabetes in 2017 following large Phase 3 trials including the cardiovascular safety trial SUSTAIN-6.
- On the basis of the above rodent studies, semaglutide is contraindicated in those with a personal or family history of medullary thyroid carcinoma and in patients with Multiple Endocrine Neoplasia syndrome type 2 (MEN 2). However, following review of human data with GLP-1RAs, FDA does not require or suggest routine monitoring of serum calcitonin/ thyroid ultrasound in people treated with semaglutide.

- Injection-site reactions: Serious injection-site reactions, with or without bumps (nodules), have happened in some people who use GLP-1RAs but are less frequent with semaglutide. Injection-site reactions have rarely required surgical intervention. Participants will be instructed to report any injection site reactions including nodules to study investigators.
- Kidney injury: GLP-1 RA might cause acute kidney injury in patients without underlying renal disease especially in patient who had experienced nausea, vomiting, diarrhea or dehydration. Dapagliflozin also has warnings and precautions against acute kidney injury and impairment in renal function.
- As mentioned above, there is a risk of ketoacidosis, as shown by some studies and by our data, when SGLT2 inhibitors are used in type 1 diabetes. In our own preliminary studies on triple therapy, we observed euglycemic ketoacidosis in participants whose insulin doses had been reduced by > 20% of baseline levels or in patients who are not adequately insulinzed. Thus, this adverse effect is largely attributable to insulinopenia as well as anti-insulin effects of SGLT2 inhibitors at the hepatic level (e.g. increased gluconeogenesis).
- The following other known adverse effects of SGLT-2 inhibitors will be discussed with participants when obtaining informed consent:

1. Risk of dizziness/ symptomatic hypotension: can occur soon after initiation due to intravascular volume contraction.
2. Impaired kidney function: As SGLT-2 inhibitors increase serum creatinine and decrease eGFR, this will be monitored closely.
3. Genital mycotic infections: SGLT-2 inhibitors increase the risk of genital mycotic infections, particularly in women. Those with a prior history are more susceptible. Following topical treatment with anti-fungal medications they rarely re-occur.
4. Bladder Cancer - Across 22 clinical studies, newly diagnosed cases of bladder cancer were reported in 10/6045 patients (0.17%) treated with Dapagliflozin and 1/3512 patient (0.03%) treated with placebo. After excluding patients in whom exposure to study drug was less than one year at the time of diagnosis, there were 4 cases of bladder cancer with dapagliflozin and no cases with placebo i.e. too few cases to determine causality.
5. The dapagliflozin tablets and matching placebo contain lactose, which may cause discomfort in lactose-intolerant individuals.

- Severe allergic reaction (anaphylaxis) to study drugs or placebo ingredients or other interventions may accrue in rare cases.
- Excessive weight loss: Both investigation drugs are known to cause weight loss in the range of 5-10% in type 2 diabetes patients. However, the magnitude of weight loss following the combination of both drugs in type 1 diabetes patients may be significantly more. Excessive weight loss may lead to increased risk of hypoglycemia and ketosis or DKA (especially in the presence of dapagliflozin) and other unforeseen risks. Therefore, weight loss during any phase of the study will be limited to ≤15% baseline body weight or reaching a BMI of ≤22Kg/m^2.
- There might be other unforeseen risks associated with the use of these agents in type 1 diabetes and additional unforeseen risks associated with the combination therapy.

- There is always the risk of breach of confidentiality where patients' identifiable data is compromised.

21.2 Describe procedures performed to lessen the probability or magnitude of risks, including procedures being performed to monitor subjects for safety.

Response:

The following are procedures to lessen the probability and magnitude of risks listed above:

1- Hypoglycemia:
- Patients will be instructed to measure glucose at least 4 times a day (before meals and at bedtime) through-out the study and record all reading.
- Insulin dose adjustments will be done at every visit to avoid hypoglycemia. However, there will be no preemptive insulin dose reduction at start of treatment since all patients will have HbA1c >7.5% and the risk of hypoglycemia will be minimal when semaglutide is added.
- Daily fingerstick glucose, CGM and insulin pump data will be reviewed by the investigators for optimal titration of insulin dose to target glucose levels between 90-180mg/dl.
- In general, insulin dose adjustments should not be >10% from current total insulin dose (or 5% if HbA1c is <7%) or >20% from baseline total insulin dose at week 0 and then at week 26.
- Patients will be trained in CARB counting to avoid inaccurate insulin dose administration.
- Patients will be trained to recognize symptoms of hypoglycemia and how to manage it at home. These symptoms include feeling shaky, being nervous or anxious, sweating, chills and clamminess, confusion, fast heartbeat, feeling lightheaded or dizzy, hunger, nausea and color draining from the skin (pallor) among others. They will be asked to call the research office or the on call clinical services.
- When patients feel symptoms of hypoglycemia they will be asked to measure blood glucose and if <70mg/dl they will be instructed to consume carbohydrate or 4 tablets of glucose (4gm each). If after 15 min hypoglycemia continues, repeat carb intake until sugar levels are above 70 mg/dl. If sugar levels are below 55mg/dl, glucagon injection is permitted as rescue medication for severe hypoglycemia.
- Patients will be instructed to inform people who they are in frequent contact with (for example, friends, family members, and coworkers) on how to give glucagon to treat severe hypoglycemia. If patient have needed glucagon previously, it should be documented during screening visit so more emphasis on ways to prevent severe hypoglycemia in the future can be discussed.
- Patients/family will be instructed not to hesitate to call 911 if someone is unconscious and glucagon is not available or someone does not know how to use it and not to inject insulin (it will lower their blood glucose even more) or provide food or fluids (they can choke). They will be asked to document all details of the incident and report to research staff as soon as possible.
- Study medications dose will be reduced in patients who continue to have more than 2 measurements of glucose levels in the hypoglycemic range (<70mg/dl,

regardless of symptoms) after insulin dose is reduced by the maximum allowed in this study.

2- Ketosis and Ketoacidosis:

- Patients will be instructed to measure ketone bodies in urine samples daily starting at 3 days before week 26 with Ketostix (Bayer) and record it in diary provided. Ketones (blood and urine) will be measured at every visit. Additionally, patients will be provided with a ketone meter and strips to measure blood ketones when needed. If ketone bodies in urine measure +1 or greater (>1.5mM in urine), they will be instructed to measure blood ketones and to repeat both the measurements after 60 min. If consecutive measurements show moderate or higher ketones in urine or blood ketones >0.6mM, the patient will be instructed to call the research center or their doctor directly. They will be monitored closely for insulin dose, carb intake, ketones and blood sugar and dapagliflozin/semaglutide can be temporarily discontinued, if necessary, for 1-2 weeks.
- Patients will be provided training to recognize the signs and symptoms of DKA and to self-test urine ketones on a PRN basis (in addition to once daily) if they develop any symptoms consistent with DKA, including nausea, vomiting, fatigue, confusion, or shortness of breath. This should be reported regardless of blood sugar levels since in type 1 diabetes on SGLT-2 inhibitors, DKA can occur even in normal glucose levels.
- Insulin dose changes should not be greater than 10% (5% if HbA1c is<7%) from current levels and not reduced by more than 20% from baseline levels at 0 weeks and then at 26 weeks.
- Patients requiring an insulin dose reduction > 20% AND their total insulin dose is <0.4U/Kg will be monitored closely for insulin dose, carb intake, ketones and blood sugar and dapagliflozin/semaglutide can be temporarily discontinued, if necessary, for 1-2 weeks. If insulin requirements remain law, patient will be withdrawn from study.
- We shall not increase dose of dapagliflozin in patients who exhibit significant ketonuria (+2 or more) with the lower dose.
- Urinary ketones (and blood ketones if patients have a dual glucose and ketone meter) will be monitored daily and reported. Dapagliflozin will be temporarily (1 week) discontinued and STICH protocol (STop SGLT-2i, give Insulin bolus, Carbohydrate, and Hydration) activated in events of DKA or if based on investigator discretion if blood ketone levels are persistently (1 week) >1.5 mM (+2 in or more in urine). Dapagliflozin may also be temporarily stopped in clinical situations known to predispose to ketoacidosis (e.g. prolonged fasting due to acute illness or surgery). Patients will be instructed to stay hydrated and not reduce their caloric intake (especially carbohydrates) or start new dietary systems (especially ketogenic diets) during the study.
- If a patient develops symptoms of DKA or ketone levels exceed the allowed limit in this study (persistent (1 week) blood ketones >1.5 mM or +2 in or more in urine), STICH protocol (STop SGLT-2i, give Insulin bolus, Carbohydrate, and Hydration) will be activated. They could be managed for up to a week. If

symptoms and ketone levels persist over 1 week, patients will be withdrawn form study. Insulin infusions may be administered by ER or hospital if patients develop DKA. They will be asked to document all details of the incident and report to research staff as soon as possible.

3- Risk of acute kidney injury or renal function impairment: CBC, CMP, including kidney function test and urinary markers will be performed at screening, during and at end of the study (table 1). Additional testing will be performed in patients who suffer from sever nausea or GI symptoms or hypovolemic and during the up titration of investigation drugs. If acute kidney injury occurs dapagliflozin will be discontinued immediately. To lessen the chance of this risk, patients will eGFR<60 ml/min/1.73m^2 will be excluded. Patients will also discontinue study medications if eGFR falls by >15 ml/min in 2 repeated measurements. Also other patients with factors that may predispose patients to acute kidney injury e.g. hypovolemia, chronic renal insufficiency, congestive heart failure will be excluded. Patients on ACEI, ARB, NSAID will be monitored closely. Dapagliflozin will be temporarily discontinued in any setting of reduced oral intake such as acute illness or fasting or fluid losses such as gastrointestinal illnesses, or excessive heat exposure.

4- Injection site reactions, mycotic infections and complications of IV and biopsy collection will also be monitored, Physical examination will be performed at screening 26, and 52 weeks visits. Safety vital assessments will be performed at every visit. Pregnancy will be tested at screening, 0, 4 13, 26, 39, and 52 weeks. Phone calls will be made for safety and compliance at least 7 time during the study. The patients, however, will be instructed to report any adverse event or safety related information via phone as soon as it occurs. All safety data will be reviewed by the PI or sub-investigators within 1-2 days of collection.

5- Epinephrine is the primary treatment for anaphylaxis with no absolute contraindication to its use. Our site and staff are trained and equipped to assist patients with such reactions. Severe conditions that are not satisfactory managed in site will be transferred to the hospital emergency department for further assessment and help.

6- Excessive weight loss: Doses of investigational drugs will be reduced in patients who experience excessive weight loss (≥15% of basal body weight or BMI ≤22Kg/m^2). Patients on semaglutide only (first 26 weeks) will be down-titrated to the lower dose. In patients on semaglutide and dapagliflozin/placebo (weeks 26-52), dose of dapagliflozin/placebo will be down-titrated to the lower dose. If weight loss continues, semaglutide dose will be down-titrated as well. Instructions to maintain a minimum of 50g daily carb intake will be also emphasized in patients with significant weight loss (>10% baseline body weight).

21.3 If applicable, indicate ***which procedures*** *may have risks to the subjects that are currently unforeseeable.*

Response:

There might be additional adverse effects of dapagliflozin not currently known or those unique to their use in Type 1 diabetes. There is no previous studies on the use of semaglutide in type 1 diabetes; therefore, additional unforeseen risk may be encountered in this study. Additionally, unforeseen risks might be associated with the combination therapy that might involve unknown drug-drug interactions.

21.4 If applicable, indicate which research procedures may have risks to an embryo or fetus should the subject be or become pregnant.

Response: Not applicable, we will not be enrolling pregnant participants or who may become pregnant or with in child baring years without signing the consent stating they will use at least two forms of birth control. If a participant becomes pregnant they will be withdrawn from the study immediately. They will be followed until term and any effects on the newborn will be documented.

21.5 If applicable, describe risks to others who are not subjects.

Response: We do not anticipate any additional risk to others not participating in this study.

22.0 Potential Benefits to Subjects

22.1 Describe the potential benefits that individual subjects may experience by taking part in the research. Include the probability, magnitude, and duration of the potential benefits. Indicate if there is no direct benefit.

*NOTE: Compensation **cannot** be stated as a benefit.*

Response: The investigational drugs used in this study are already approved as antidiabetic agents for treating type 2 diabetes. Both agents have demonstrated significant improvement in HbA1c, weight and blood pressure. It is our expectation that patients with type 1 diabetes will experience similar benefits compared to those who are treated with insulin alone. The magnitude and duration of the benefits and the safety profile may however differ and are the focus of this investigation.

23.0 Compensation for Research-Related Injury

☐ **N/A:** The research procedures for this study do not present risk of research related injury (e.g. survey studies, records review studies). This section does not apply.

*23.1 **If the research procedures carry a risk of research related injury,** describe the available compensation to subjects in the event that such injury should occur.*

Response: Routinely, Buffalo General Hospital, Erie County Medical Center, and/or the University at Buffalo, State University of New York, its agents, or its employees do not compensate for or provide free medical care for human

subjects/participants in the event that any injury results from participation in a human research project. In the unlikely event that they become ill or injured as a direct result of participating in this study, they may receive medical care. Your study doctor will explain the treatment options to you and tell you where you can get treatment. Generally, this care will be billed to you, your insurance or other third party. Additionally, AstraZeneca shall reimburse the institution for the direct, reasonable, and necessary medical expenses incurred by the institution for the treatment of any personal injury that is a direct result of adverse drug experiences to subjects resulting directly from any manufacturing defects in the study drug to which the subjects would not have been exposed but for their participation in the study if (i) the institutional parties have complied with this agreement, the protocol and any written instructions of AstraZeneca concerning the study drug and (ii) all the requirements of informed consent have been complied with. AstraZeneca will not provide compensation for lost wages or for any other damages, expenses or losses, or for medical expenses have been covered by a subject's medical or other insurance.

23.2 Provide a copy of contract language, if any, relevant to compensation for research related injury.

NOTE: If the contract is not yet approved at the time of this submission, submit the current version here. If the contract is later approved with ***different language regarding research related injury****, you must modify your response here and submit an amendment to the IRB for review and approval.*

Response: AstraZeneca shall reimburse the institution for the direct, reasonable, and necessary medical expenses incurred by the institution for the treatment of any personal injury that is a direct result of adverse drug experiences to subjects resulting directly from any manufacturing defects in the study drug to which the subjects would not have been exposed but for their participation in the study if (i) the institutional parties have complied with this agreement, the protocol and any written instructions of AstraZeneca concerning the study drug and (ii) all the requirements of informed consent have been complied with. AstraZeneca will not provide compensation for lost wages or for any other damages, expenses or losses, or for medical expenses have have been covered by a subject's medical or other insurance.

24.0 Economic Burden to Subjects

24.1 Describe any costs that subjects may be responsible for because of participation in the research.

NOTE: Some examples include transportation or parking.

Response: All research expenses will be covered by the study. The background use of insulin is considered as part of standard of care is the responsibility of the patients and his/her insurance. Participants will not be subjected to any other out of pocket cost.

☐ **N/A:** This study is not enrolling subjects, or is limited to records review procedures only. This section does not apply.

25.0 Compensation for Participation

25.1 Describe the amount and timing of any compensation to subjects, including monetary, course credit, or gift card compensation.

Response:
Visit schedule:

- Screen: $0
- Visit 1 (Week -3): Insulin adjustment: $10
- Visit 2 (Week -1): Meal ($75), insulin adjustment, and 24hr blood pressure monitor ($50): $125*
- Visit 3 (Week 0): 24 hr urine ($15), blood draw ($25), fat biopsy ($75), insulin adjustment, questionnaire ($10): $125
- Visit 4 (Week 1): Insulin adjustment: $10
- Visit 5 (Week 2): Insulin adjustment: $10
- Visit 6 (Week 4): Insulin adjustment: $10
- Visit 7 (Week 13): 24hr urine ($15), blood draw ($25), insulin adjustment, 24hr bp monitor ($50): $90*
- Visit 8 (Week 23): Insulin adjustment: $10
- Visit 9 (Week 25): Insulin adjustment, 24bp monitor ($50), Meal ($75): $125*
- Visit 10 (Week 26): Insulin adjustment, 24hr urine ($15), blood draw ($25), fat biopsy ($75), questionnaire ($10): $125*
- Visit 11 (Week 27): Insulin adjustment: $10
- Visit 12 (Weak 31): Insulin adjustment, blood draw: $25
- Visit 13 (Week 39): Insulin adjustment, 24hr urine ($15), blood draw ($25), 24hr bp monitor ($50): $90*
- Visit 14 (Week 49): Insulin adjustment: $10
- Visit 15 (Week 51): Insulin adjustment, 24bp monitor ($50), meal ($75): $125*
- Visit 16 (Week 52): Insulin adjustment, 24hr urine ($15), blood draw ($25), questionnaire ($10), fat biopsy ($75):$125*
  *Total compensation for all completed visits: up to $1,025.00. For those not wishing to participate in the optional blood pressure monitoring and/or fat biopsy procedure, the compensation will decrease by $250 for not completing the bp monitoring, and by yet another $225.00 for not completing the fat biopsies.

☐ **N/A:** This study is not enrolling subjects, or is limited to records review procedures only. This section does not apply.

☐ **N/A:** There is no compensation for participation. This section does not apply.

26.0 Consent Process

26.1 *Indicate whether you will be obtaining consent.*

NOTE: This does not refer to consent documentation, but rather whether you will be obtaining permission from subjects to participate in a research study. Consent documentation is addressed in Section 27.0.

☒ ***Yes*** *(If yes, Provide responses to each question in this Section)*
☐ ***No*** *(If no, Skip to Section 27.0)*

26.2 *Describe where the consent process will take place. Include steps to maximize subjects' privacy.*

Response: All participants will come to the research department to be consented. Participants will be placed in a private, screened off area and or room where they can review the consent. Participant questions and or concerns will be address with a member of the study team or research doctor if applicable. The research coordinator will discuss in length the participants requests for privacy of their PHI.

26.3 *Describe how you will ensure that subjects are provided with a sufficient period of time to consider taking part in the research study.*

NOTE: It is always a requirement that a prospective subject is given sufficient time to have their questions answered and consider their participation. See "SOP: Informed Consent Process for Research (HRP-090)" Sections 5.5 and 5.6.

Response: participants will be made aware that participating in research is completely voluntary, and they may withdraw at any time with no consequence to their routine clinic care. If the patients requires time to decide and or discuss partaking in a research study, the subject will be given said time.

26.4 *Describe any process to ensure ongoing consent, defined as a subject's willingness to continue participation for the duration of the research study.*

Response: The research coordinator and study team are available to answer any question or concerns with the patient during the duration of the research trial. At each study visit, the patient is asked a series of questions to ensure they are on task with the study visits and feel comfortable. Upon departing from their study visit, the patients are told of their next visit and given detail instruction for their next visit. If study is revised or amendment or new information becomes available about drug safety that may affect patients participation, the patient may be re-consented to ensure patient ongoing consent

26.5 *Indicate whether you will be following "SOP: Informed Consent Process for Research (HRP-090)." If not, or if there are any exceptions or additional details to what is covered in the SOP, describe:*

- *The role of the individuals listed in the application who are involved in the consent process*
- *The time that will be devoted to the consent discussion*
- *Steps that will be taken to minimize the possibility of coercion or undue influence*
- *Steps that will be taken to ensure the subjects' understanding*

Response:

☒ We have reviewed and will be following "SOP: Informed Consent Process for Research (HRP-090)."

Non-English Speaking Subjects

☒ **N/A:** This study will not enroll Non-English speaking subjects. *(Skip to Section 26.8)*

26.6 Indicate which language(s) other than English are likely to be spoken/understood by your prospective study population or their legally authorized representatives.

NOTE: The response to this Section should correspond with your response to Section 6.4 of this protocol.

Response:

26.7 If subjects who do not speak English will be enrolled, describe the process to ensure that the oral and written information provided to those subjects will be in that language. Indicate the language that will be used by those obtaining consent.

NOTE: Guidance is provided on "SOP: Informed Consent Process for Research (HRP-090)."

Response:

Cognitively Impaired Adults

☒ **N/A**: This study will not enroll cognitively impaired adults. *(Skip to Section 26.9)*

26.8 Describe the process to determine whether an individual is capable of consent.

Response:

Adults Unable to Consent

☒ **N/A**: This study will not enroll adults unable to consent. (*Skip to Section 26.13)*

When a person is not capable of consent due to cognitive impairment, a legally authorized representative should be used to provide consent (Sections 26.9 and 26.10) ***and, where possible, assent of the individual should also be solicited*** *(Sections 26.11 and 26.12).*

26.9 Describe how you will identify a Legally Authorized Representative (LAR). Indicate that you have reviewed the "SOP: Legally Authorized Representatives, Children, and Guardians (HRP-013)" for research in New York State.

NOTE: Examples of acceptable response includes: verifying the electronic medical record to determine if an LAR is recorded.

Response:

☐

26.10 ***For research conducted outside of New York State****, provide information that describes which individuals are authorized under applicable law to consent on behalf of a prospective subject to their participation in the research. One method of obtaining this information is to have a legal counsel or authority review your protocol along with the definition of "legally authorized representative" in "SOP: Legally Authorized Representatives, Children, and Guardians (HRP-013)."*

Response:

26.11 Describe the process for ***assent of the adults****:*

- *Indicate whether assent will be obtained from all, some, or none of the subjects. If some, indicate which adults will be required to assent and which will not.*

  Response:

- *If assent will not be obtained from some or all subjects, provide an explanation of why not.*

  Response:

26.12 Describe whether ***assent of the adult*** *subjects will be documented and the process to document assent.*

NOTE: The IRB allows the person obtaining assent to document assent on the consent document using the "Template Consent Document (HRP-502)" Signature Block for Assent of Adults who are Legally Unable to Consent.

Response:

Subjects who are not yet Adults (Infants, Children, and Teenagers)

☒ **N/A**: This study will not enroll subjects who are not yet adults. *(Skip to Section 27.0)*

26.13 Describe the criteria that will be used to determine ***whether a prospective subject has not attained the legal age for consent to treatments or procedures involved in the research*** *under the applicable law of the jurisdiction in which the research will be conducted* ***(e.g., individuals under the age of 18 years)****. For research conducted in NYS, review "SOP: Legally Authorized Representatives, Children, and Guardians (HRP-013)" to be aware of which individuals in the state meet the definition of "children."*

NOTE: Examples of acceptable responses include: verification via electronic medical record, driver's license or state-issued ID, screening questionnaire.

Response:

26.14 ***For research conducted outside of New York State****, provide information that describes which persons have not attained the legal age for consent to treatments or procedures involved the research, under the applicable law of the jurisdiction in which research will be conducted. One method of obtaining this information is to have a legal counsel or authority review your protocol along the definition of "children" in "SOP: Legally Authorized Representatives, Children, and Guardians (HRP-013)."*

Response:

26.15 Describe whether parental permission will be obtained from:

Response: N/A

☐ One parent even if the other parent is alive, known, competent, reasonably available, and shares legal responsibility for the care and custody of the child.

☐ Both parents unless one parent is deceased, unknown, incompetent, or not reasonably available, or when only one parent has legal responsibility for the care and custody of the child.

☐ Parent permission will not be obtained. A waiver of parent permission is being requested.

NOTE: The requirement for parent permission is a protocol-specific determination made by the IRB based on the risk level of the research. For guidance, review the "CHECKLIST: Children (HRP-416)."

26.16 Describe whether permission will be obtained from individuals ***other than parents****, and if so, who will be allowed to provide permission. Describe your procedure for determining an individual's authority to consent to the child's general medical care.*

Response:

26.17 Indicate whether assent will be obtained from all, some, or none of the ***children****. If assent will be obtained from some children, indicate which children will be required to assent.*

Response:

26.18 When assent of children is obtained, describe how it will be documented.

Response:

27.0 Waiver or Alteration of Consent Process

Consent will not be obtained, required information will not be disclosed, or the research involves deception.

☒ **N/A:** A waiver or alteration of consent is not being requested.

27.1 If the research involves a waiver or alteration of the consent process, please review the "CHECKLIST: Waiver or Alteration of Consent Process (HRP-410)" to ensure that you have provided sufficient information for the IRB to make the determination that a waiver or alteration can be granted.

NOTE: For records review studies, the first set of criteria on the "CHECKLIST: Waiver or Alteration of Consent Process (HRP-410)" applies.

Response:

27.2 If the research involves a waiver of the consent process for planned emergency research, please review the "CHECKLIST: Waiver of Consent for Emergency Research (HRP-419)" to ensure you have provided sufficient information for the IRB to make these determinations. Provide any additional information necessary here:

Response:

28.0 Process to Document Consent

☐ **N/A:** A Waiver of Consent is being requested.
(Skip to Section 29.0)

28.1 Indicate whether you will be following "SOP: Written Documentation of Consent (HRP-091)." If not or if there are any exceptions, describe whether and how consent of the subject will be obtained including whether or not it will be documented in writing.

NOTE: If your research presents no more than minimal risk of harm to subjects and involves no procedures for which written documentation of consent is normally required outside of the research context, the IRB will generally waive the requirement to obtain written documentation of consent. This is sometimes referred to as 'verbal consent.' Review "CHECKLIST: Waiver of Written Documentation of Consent (HRP-411)" to ensure that you have provided sufficient information.

If you will document consent in writing, attach a consent document with your submission. You may use "TEMPLATE CONSENT DOCUMENT (HRP-502)". If you will obtain consent, but not document consent in writing, attach the script of the information to be provided orally or in writing (i.e. consent script or Information Sheet).

Response:

☒ We will be following "SOP: Written Documentation of Consent" (HRP-091).

29.0 Multi-Site Research (Multisite/Multicenter Only)

☐ **N/A:** This study is not an investigator-initiated multi-site study. This section does not apply.

29.1 If this is a multi-site study ***where you are the lead investigator****, describe the processes to ensure communication among sites, such as:*

- *All sites have the most current version of the IRB documents, including the protocol, consent document, and HIPAA authorization.*
- *All required approvals have been obtained at each site (including approval by the site's IRB of record).*
- *All modifications have been communicated to sites, and approved (including approval by the site's IRB of record) before the modification is implemented.*
- *All engaged participating sites will safeguard data as required by local information security policies.*
- *All local site investigators conduct the study appropriately.*
- *All non-compliance with the study protocol or applicable requirements will be reported in accordance with local policy.*

Response:

29.2 Describe the method for communicating to engaged participating sites:

- *Problems*
- *Interim results*
- *Study closure*

Response:

29.3 Indicate the total number of subjects that will be enrolled or records that will be reviewed across all sites.

Response: 81

29.4 If this is a multicenter study for which UB will serve as the IRB of record, and subjects will be recruited by methods not under the control of the local site (e.g., call centers, national advertisements) describe those methods.

Response: N/A

30.0 Banking Data or Specimens for Future Use

☐ **N/A:** This study is not banking data or specimens for future use or research outside the scope of the present protocol. This section does not apply.

30.1 If data or specimens will be banked (stored) for ***future use, that is, use or research outside of the scope of the present protocol****, describe where the data/specimens will be stored, how long they will be stored, how the data/specimens will be accessed, and who will have access to the data/specimens.*

NOTE: Your response here must be consistent with your response at the "What happens if I say yes, I want to be in this research?" Section of the Template Consent Document (HRP-502).

Response: The study data/specimens will be stored in a locked closet or -80 freezer at the research facility of the Diabetes and Endocrinology Center of WNY for at least 7 years.

The research staff (study personnel including coordinator) only will be authorized to access data and or specimens

30.2 List the data to be stored or associated with each specimen.

Response: Patient ID number, study visit information and date of collection will be stored with specimen. Other data stored will include record files of

all patients participating in the study, including data collection sheets, lab results, CGM and insulin pump data.

30.3 Describe the procedures to release banked data or specimens for future uses, including: the process to request a release, approvals required for release, who can obtain data or specimens, and the data to be provided with specimens.

Response: The copy of the individual patient data collected during the study period will be provided to these individual patients who can choose to hand carry it to their respective physicians and a copy will be faxed to their respective clinical providers upon verbal request from the patient. The data provided will include the insulin pump or CGM data or any of the lab results obtained during the study period. The results of the completed study will be made available to the patients if requested through published manuscript.

31.0 Drugs or Devices

☐ **N/A:** This study does not involve drugs or devices. This section does not apply.

31.1 If the research involves drugs or devices, list and describe all drugs and devices used in the research, the purpose of their use, and their regulatory approval status.

Response: **Drugs used:**

Description or Identity of investigational product(s)

Investigational product	***Dosage form and strength***	***Manufacturer***	***Description***
Dapagliflozin (Farxiga) 5 mg	*Green, plain, diamond shaped, film coated 5 mg tablet*	*AstraZeneca*	*sodium-glucose cotransporter 2 (SGLT2) inhibitor indicated as an adjunct to diet and exercise to improve glycemic control*
Matching placebo for dapagliflozin 5 mg	*Green, plain, diamond shaped, film coated tablet*	*AstraZeneca*	
Semaglutide (Ozempic) 0.25 mg, 0.5 mg, 1.0 mg	*clear, colorless solution that contains 2 mg of semaglutide in a 1.5 mL (1.34 mg/mL) pre-filled, disposable, single-patient-use pen injector*	*Novo Nordisk*	*glucagon-like peptide 1 (GLP-1) receptor agonist indicated as an adjunct to diet and exercise to improve glycemic control*

- *Dapagliflozin and its matching placebo tablets will be supplied in bottles and are provided by Astra Zeneca.*
- *Semaglutide will be supplied as prefilled injectable pens and will be purchased commercially through the University at Buffalo Research Pharmacy.*

Subjects will receive drugs in their unmodified form. Dapagliflozin is a once daily tablet, while Semaglutide is a weekly subcutaneous injection.

Insulin pumps (CSII) and long and short acting insulin is the only diabetes medication accepted before and during this trial. No other diabetes drug is permitted.
Blood pressure or lipid lowering drugs are permitted if on stable dose for at least three months prior to start of trial. Glucagon injection is permitted as rescue medication for severe hypoglycemia. Insulin infusions may be administered by ER or hospital if patients develop DKA.

31.2 Describe your plans to store, handle, and administer those drugs or devices so that they will be used only on subjects and be used only by authorized investigators.

Response: Dapagliflozin 5mg tablets and matching placebo provided by Astra Zeneca will be stored in a locked cabinet in research pharmacy. Semaglutide provided by University at Buffalo research pharmacy will be stored in temperature controlled refrigerator at 4C at the research facility of the Diabetes and Endocrinology Center of WNY at 1000 Youngs Rd until dispensed to patients. Investigational drugs will be dispensed by PI or CO-Is only to study patients according to randomization plan.

If the drug is investigational (has an IND) or the device has an IDE or a claim of abbreviated IDE (non-significant risk device), include the following information:

31.3 Identify the holder of the IND/IDE/Abbreviated IDE.

Response: IND application for use of all drugs (dapagliflozin and semaglutide) in type 1 diabetes will be submitted to FDA and no study procedure will start before all local and federal approvals are obtained. FDA has granted IND for usage of dapagliflozin in type 1 diabetes. We will seek FDA approval for the use of semaglutide in this study. These IND letters will be submitted to IRB and JDRF along with final version of protocol before initiating any study activities.

31.4 Explain procedures followed to comply with FDA sponsor requirements for the following:

	Applicable to:		
FDA Regulation	***IND Studies***	***IDE studies***	***Abbreviated IDE studies***
21 CFR 11	***X***	***X***	
21 CFR 54	***X***	***X***	
21 CFR 210	***X***		
21 CFR 211	***X***		
21 CFR 312	***X***		
21 CFR 812		***X***	***X***
21 CFR 820		***X***	

Response: All FDA sponsor requirements have been reviewed and will be followed during the study procedures

32.0 Humanitarian Use Devices

☒ **N/A:** This study does not involve humanitarian use devices. This does not apply.

32.1 For Humanitarian Use Device (HUD) uses provide a description of the device, a summary of how you propose to use the device, including a description of any screening procedures, the HUD procedure, and any patient follow-up visits, tests or procedures.

Response:

32.2 For HUD uses provide a description of how the patient will be informed of the potential risks and benefits of the HUD and any procedures associated with its use.

Response:
