# Supplementary material for: Insulin, Semaglutide and Dapagliflozin in Adults With Type 1 Diabetes: Design and Methods of Triple Therapy for Type 1 Diabetes (TTT1)—An International Phase 3 Clinical Trial
Source: Diabetes Obes Metab. 2026 Jul 19;28(10):8812–24. doi: 10.1111/dom.71076 (PMC13538731; doi:10.1111/dom.71076)
Supplement: Supplementary file 2 [file DOM-28-8812-s003.pdf]

FULL/LONG TITLE OF THE TRIAL
Triple Therapy for Type 1 Diabetes with Insulin, Semaglutide and Dapagliflozin (TTT1) - UK

SHORT STUDY TITLE / ACRONYM
TTT1 (UK)

PROTOCOL VERSION NUMBER AND DATE
Version 1.0 (2020/01/21)

RESEARCH REFERENCE NUMBERS

**IRAS Number:**	254431
**EudraCT Number:**	2018-004120-11
**ISRCTN Number / Clinical trials.gov Number:**	TBC
**SPONSOR Number:**	GN17DI706
**FUNDER Number:**	3-SRA-2019-669-M-B

SIGNATURE PAGE

The undersigned confirm that the following protocol has been agreed and accepted and that the Chief Investigator agrees to conduct the trial in compliance with the approved protocol and will adhere to the principles outlined in the Medicines for Human Use (Clinical Trials) Regulations 2004 (SI 2004/1031), amended regulations (SI 2006/1928) and any subsequent amendments of the clinical trial regulations, Good Clinical Practice (GCP) guidelines, the Sponsor's (Standard Operating Procedures (SOPs), and other regulatory requirements as amended.
I agree to ensure that the confidential information contained in this document will not be used for any other purpose other than the evaluation or conduct of the clinical investigation without the prior written consent of the Sponsor
I also confirm that I will make the findings of the study publicly available through publication or other dissemination tools without any unnecessary delay and that an honest accurate and transparent account of the study will be given; and that any discrepancies from the study as planned in this protocol will be explained.

For and on behalf of the Study Co-Sponsor (University of Glasgow):
Signature: .... Date: ....../....../......
Name (please print): ....
Position: ....

For and on behalf of the Study Co-Sponsor (NHS Greater Glasgow and Clyde):
Signature: .... Date: ....../....../......
Name (please print): ....
Position: ....

UK Chief Investigator:
Signature: .... Date: ....../....../......
Name: (please print): John Petrie ....
Position: Professor of Diabetic Medicine..

Statistician:
Signature: .... Date: ....../....../......
Name: (please print): ....

Position: ....

KEY TRIAL CONTACTS

UK Chief Investigator	Professor John R Petrie BSc<br>Professor of Diabetic Medicine<br>Institute of Cardiovascular and Medical Sciences<br>BHF Glasgow Cardiovascular Research Centre<br>University of Glasgow<br>126 University Place<br>Glasgow G12 8TA<br>Secretary +44(0)141 330 3325<br>john.petrie@glasgow.ac.uk
UK Co- Investigator	Dr James Boyle<br>Consultant Diabetologist<br>Clinical Lead NHS GG&C Diabetes MCN<br>Diabetes UK Clinical Champion<br>Glasgow Royal Infirmary<br>84 Castle Street, Glasgow, G4 0SF<br>Tel: : +44(0)141 211 5996<br>james.boyle@glasgow.ac.uk
Trial Co-ordination	Glasgow Clinical Trials Unit<br>Robertson Centre for Biostatistics, 11th Floor,<br>Boyd Orr Building, University of Glasgow<br>Glasgow, G12 8QQ<br>Tel: +44 (0) 141 330 4744
Co-Sponsor	NHS Greater Glasgow & Clyde<br><br>Contact: Dr Maureen Travers<br>Research and Development Management Office<br>Clinical Research & Development<br>NHS Greater Glasgow & Clyde<br>Ward 11, Dykebar Hospital<br>Grahamston Road<br>Paisley, PA2 7DE<br>Tel: +44 (0) 0141 314 4012<br>maureen.travers@ggc.scot.nhs.uk
Co-sponsor	University of Glasgow<br><br>Contact: Dr Debra Stuart<br>College of Medical, Veterinary & Life Sciences<br>Room 327 Wolfson Medical School Building<br>University Avenue<br>Glasgow G12 8QQ<br>Tel: +44 (0)141 330 4539<br>Debra.Stuart@glasgow.ac.uk

Funder	JDRF<br>26 Broadway, 15th floor, New York, NY 10004, USA
Clinical Trials Unit	Glasgow Clinical Trials Unit<br>Robertson Centre for Biostatistics, 11th Floor,<br>Boyd Orr Building, University of Glasgow<br>G12 8QQ<br>Tel: 0141 330 4744<br>liz.anderson@glasgow.ac.uk
Statistician	Professor Alex McConnachie<br>Robertson Centre for Biostatistics, 11th Floor, Boyd Orr Building, University of Glasgow, Glasgow, G12 8QQ<br>Telephone: 0141 330 4744<br>Fax: 0141-330-5094
Trials pharmacist	Dr Elizabeth Douglas<br>Clinical Trials Pharmacist<br>Clinical Research and Development<br>Ward 11, Dykebar Hospital,<br>Grahamston Road | Paisley PA2 7DE<br>Tel: 0141 314 4073<br>E-mail: elizabeth.douglas@ggc.scot.nhs.uk

US Principal Investigator	Dr Paresh Dandona<br>Director, Diabetes-Endocrinology Center of Western NY<br>Chief of Endocrinology, Diabetes, Endocrinology and Metabolism, SUNY at Buffalo<br>1000 Youngs Road, Suite 105<br>Williamsville NY 14221<br>Telephone: +1(716) 535-1850<br>Fax: +1(716)-639-1200<br>pdandona@kaleidahealth.org
US Co- Investigator	Dr Husam Ghanim, PhD.<br>Director, Diabetes and Endocrinology Research Lab<br>Research Associate Professor,<br>School of Medicine, State University of NY at Buffalo<br>Clinical and Translational Research Center<br>875 Ellicott St<br>Buffalo, NY 14203<br>Tel: (+1)716-881-8924<br>ghanim@buffalo.edu

Key Protocol Contributors	Professor John Petrie<br>john.petrie@glasgow.acuk<br><br>Dr Paresh Dandona<br>pdandona@kaleidahealth.org<br><br>Husam Ghanim, PhD.<br>ghanim@buffalo.edu<br><br>Dr James Boyle<br>james.boyle@glasgow.ac.uk<br><br>Dr Robert Lindsay<br>robert.lindsay@glasgow.ac.uk<br><br>Dr Joseph Timmons<br>joseph.timmons@glasgow.ac.uk<br><br>Professor Ian Ford<br>ian.ford@glasgow.ac.uk<br><br>Professor Alex McConnachie<br>Alex.McConnachie@glasgow.ac.uk

TRIAL SUMMARY

Trial Title	Triple Therapy for Type 1 Diabetes with Insulin, Semaglutide, and Dapagliflozin (UK)	
Internal ref. no. (or short title)	TTT1	
Clinical Phase	Phase 3	
Trial Design	Two six-month periods: (i) open-label randomised (2:1) parallel group semaglutide vs standard of care; (ii) double-blind, randomised (1:1) parallel group, dapagliflozin vs placebo for those participants initially randomised to semaglutide; ongoing standard of care for all participants.	
Trial Participants	Adults with type 1 diabetes for at least one year with HbA1c 7.5-11.0% on stable multiple daily injections (MDI) (four or more) of insulin or continuous subcutaneous insulin infusion (CSII) for the previous three months.	
Planned Sample Size	114 (57 in UK)	
Treatment duration	Approximately 1 year	
Follow up duration	Approximately 1 year	
Planned Trial Period	Approximately 3 years	
	Objectives	Outcome Measures
Primary	To assess in people with type 1 diabetes whether triple therapy (dapagliflozin, semaglutide and insulin) reduces HbA1c in comparison with dual therapy (placebo, semaglutide, and insulin).	HbA1c
Secondary	To assess in people with type 1 diabetes whether triple therapy (dapagliflozin, semaglutide and insulin) reduces HbA1c in comparison with standard therapy (insulin alone)	HbA1c
	To assess in people with type 1 diabetes whether dual therapy (semaglutide and insulin) reduces HbA1c in comparison with standard therapy (insulin alone)	HbA1c
	To assess in people with type 1 diabetes whether triple therapy (dapagliflozin, semaglutide and insulin) improves glucose control as assessed by CGM, insulin requirement, body weight, blood	- Mean fasting blood glucose<br><br>- Mean blood glucose*<br>- Percentage time in range<br>- Glycaemic variability*<br>- Percentage time in

	pressure and quality of life in comparison with (a) dual therapy (placebo, semaglutide, and and placebo) and (b) standard therapy (insulin alone)	hyperglycaemia Level 1 (10.0-13.9 mmol/)*<br>- Percentage time in hyperglycaemia Level 2 (>13.9 mmol/L)*<br>-Percentage time in hypoglycaemia Level 1 (3.0 – 3.8 mmol/L)*<br>** CGM (by masked Dexcom G6)*<br><br>Insulin requirement<br>Body weight<br>Systolic/ diastolic blood pressure<br>Number of antihypertensive agents<br>Diabetes Specific Quality of Life Scale (DSQOLS)<br>Problem Areas In Diabetes (PAID) questionnaire
Safety Endpoint 1	a) Triple therapy (dapagliflozin, semaglutide and insulin) in comparison with dual therapy (semaglutide, insulin)<br><br>b) Triple therapy (dapagliflozin, semaglutide and insulin) in comparison with standard therapy (insulin alone)	Difference between treatment arms in:<br>(i) Rate of Level 2 (<3.0 mmol/L) hypoglycaemic events (by Dexcom G6 CGM)<br>(ii) Rate of Level 3 hypoglycaemic events (severe event with altered mental and/or physical status requiring assistance)
Safety Endpoint 2	a) Triple therapy (dapagliflozin, semaglutide and insulin) in comparison with dual therapy (placebo, semaglutide, and insulin)<br><br>b) Triple therapy (dapagliflozin, semaglutide and insulin) in comparison with standard therapy (insulin alone)	Difference between treatment arms in:<br>Rate of diabetic ketoacidosis (serum ketones or urinary ketones above normal limits AND serum bicarbonate < 15 mmol/L OR blood pH < 7.3)
Investigational Medicinal Product(s)	Semaglutide<br>Dapagliflozin<br>Placebo matching dapagliflozin	
Non-investigational Medicinal Product(s)	Insulin	
Formulation, Dose, Route of Administration	Semaglutide (subcutaneous injection, once weekly) – open label by pen device (0.25 mg/ 0.5 mg/ 1.0 mg)<br>Dapagliflozin 5 mg (oral) – one then two capsules daily (uptitration)<br>Placebo matching dapagliflozin	

FUNDING AND SUPPORT IN KIND

FUNDER(S)	FINANCIAL AND NON FINANCIALSUPPORT GIVEN
JDRF New York<br>26 Broadway, 15th floor, New York, NY 10004, United States	$1.6M (to University at Buffalo, the State University of New York, NY (SUNY Buffalo), USA; and University of Glasgow, UK)
AstraZeneca<br>1800 Concord Pike, Wilmington, DE 19803, USA	Provision of dapagliflozin and matched placebo investigational medicinal products to US site
Dexcom; Suite 6, 1 Tanfield, Edinburgh, EH3 5DA	Staff training and discounted supply of CGM sensors

ROLE OF STUDY SPONSOR AND FUNDER

NHS Greater Glasgow & Clyde and The University of Glasgow will be Co-sponsors of the UK arm of the trial. Prior to study initiation, a non-commercially funded clinical trial co-sponsorship agreement will be put in place between NHS Greater Glasgow & Clyde and The University of Glasgow. The roles and liabilities each organisation will take under The Medicines for Human Use (Clinical Trials) Regulations, 2004 SI 2001:1031 are laid out in this agreement signed by both organisations. The University of Glasgow shall be responsible for carrying out the obligations and responsibilities set out in the aforementioned agreement, and shall be deemed “sponsor” for the purposes of, Part 3 of the regulations in relation to the study. NHS Greater Glasgow & Clyde shall be responsible for carrying out the responsibilities set out in the agreement, and shall be deemed “sponsor” for the purposes of, Parts 4, 5, 6 and 7 of the Regulations in relation to the study.

The Co-Sponsors will delegate specific roles to the Chief Investigator, Glasgow CTU and other third parties. These arrangements will be clearly documented in agreements and/or the Sponsor Delegated Roles and Responsibilities Matrix.

The US arm of the trial has the same inclusion and exclusion criteria and the same pre-specified outcomes but an individualised Protocol. It will collect the same data via a common eCRF. Adverse event data from each site will be available to the trial Data and Safety Monitoring Board. The UK Co-Sponsors will have no responsibility for (and carry no liability for) the conduct of the US arm of the trial.

<u>JDRF</u>
The study has been funded by a grant from JDRF. JDRF does not have a designated role or responsibility in trial design, conduct, data analysis and interpretation, manuscript writing, and dissemination of results. Periodic reports (at least annual) in relation to progress of the trial will be submitted to the JDRF. Support from JDRF will be acknowledged in any publications related to the study.

<u>Astra Zeneca</u>
This is an investigator-initiated study. Astra Zeneca provided support by supplying investigational medicinal products (IMPs) dapagliflozin and matched placebo to the US site only. Representatives of Astra Zeneca reviewed and provided comments on drafts of the Protocol but the company does not have a designated role or responsibility in trial design, conduct, data analysis and interpretation, manuscript writing, and dissemination of results.

ROLES AND RESPONSIBILITIES OF TRIAL MANAGEMENT COMMITTEES/GROUPS & INDIVIDUALS

Trial Steering Committee (TSC)

The role of the TSC is to provide overall supervision of the trial and ensure that it is being conducted in accordance with the principles of GCP and the relevant regulations. The TSC will:

1. agree the trial protocol and any protocol amendments
2. ensure that conduct of the trial is fully harmonised between the two sites
3. provide advice to the investigators on all aspects of the trial

It will have an independent Chairperson and include the US and UK investigators and key protocol contributors listed above.

Decisions about continuation or termination of the trial or substantial amendments to the protocol will be the responsibility of the TSC who will advise the co-sponsors. The TSC will meet at the start of the study, and six monthly thereafter. The TSC will have its own Charter outlining the role and responsibilities of its members. The TSC may invite other attendees from the trial team to present or participate in discussions on particular topics. These attendees will be non-voting members.

Independent data monitoring committee (IDMC)

An IDMC will be established to include two independent medical experts (covering the domains of type 1 diabetes and metabolic disease; one of the academic clinicians will act as chair) and an independent biostatistician. The Glasgow CTU will liaise with the committee and ensure that the Committee is provided with adequate information about study progress and results.

The IDMC will have a formal Charter outlining the responsibilities of the IDMC members, Glasgow CTU and the co-sponsors. Responsibilities include:

- To protect the safety of participants recruited to the trial.
- Advising the TSC and co-sponsors if it is safe and appropriate to continue with the study
- Examining information provided by the Glasgow CTU on study recruitment, adverse events and outcomes
- Providing reports for the Project Office to forward to the TSC, ethics committees, regulatory bodies, study co-sponsors, and the BHF.

The IDMC may recommend to the TSC to terminate the study or specific aspects of the study if clear harm is observed with one treatment option compared to standard of care or placebo. Specific safety endpoints that will be reviewed include rates of hypoglycaemia and ketosis/ ketoacidosis. The IDMC will not formally review efficacy or futility.

The IDMC will meet approximately every six months.

Study Management Group (SMG)

Trial activity at the UK/ Glasgow site will be coordinated from NHS Greater Glasgow & Clyde (NHS GG&C) by the TTT1 Study Management Group (SMG). The SMG will consist of the UK chief investigator, co-principal investigator other co-applicants, project manager and representatives from the Glasgow CTU, NHS GG&C and The University of Glasgow. The role of the group is to monitor all aspects

of the conduct and progress of the trial in the UK, ensuring adherence to the protocol, and take appropriate action to safeguard participants and the quality of the trial itself.

Joint Study Management Group

Coordination of trial activity between the UK/ Glasgow and the US/ Buffalo site will be facilitated by the Joint TTT1 Study Management Group. This will consist of the US and UK chief investigators, co-principal investigators, other co-applicants, study coordinators, project managers and governance/ administrative representatives as appropriate from State University of New York (SUNY), the Glasgow CTU, NHS GG&C and The University of Glasgow. It will meet approximately every 4-6 weeks. The role of the group is to coordinate all aspects of the conduct and progress of the trial between the US and UK sites, ensuring protocol adherence, and taking appropriate action to safeguard participants and the quality of the trial itself.

Protocol contributors

The protocol has been developed by a group with extensive clinical and research experience in type 1 diabetes and clinical trials including Professor John Petrie and Professor Paresh Dandona. Professor Ian Ford and Dr Alex McConnachie have research expertise in design, conduct, analysis and interpretation of clinical trials and biostatistical methods.

KEY WORDS:

Adjunct therapy
Dapagliflozin
Glycated haemoglobin (HbA1c)
Semaglutide
Type 1 diabetes

LIST of CONTENTS

RESEARCH REFERENCE NUMBERS 2
SIGNATURE PAGE 3
KEY TRIAL CONTACTS 4
TRIAL SUMMARY 7
FUNDING AND SUPPORT IN KIND 9
ROLE OF STUDY SPONSOR AND FUNDER 9
ROLES AND RESPONSIBILITIES OF TRIAL MANAGEMENT COMMITTEES/GROUPS & INDIVIDUALS 10
LIST of CONTENTS 13
LIST OF ABBREVIATIONS 14
TRIAL FLOW CHART 16
SCHEDULE OF ASSESSMENTS ....17**Error! Bookmark not defined.**
1 BACKGROUND 18
2 RATIONALE 19
3 OBJECTIVES AND OUTCOME MEASURES/ENDPOINTS 22
4 TRIAL DESIGN 25
5 STUDY SETTING 25
6 ELIGIBILITY CRITERIA 25
7 TRIAL PROCEDURES 27
8 TRIAL MEDICATION ....**Error! Bookmark not defined.**
9 PHARMACOVIGILANCE 556
10 STATISTICS AND DATA ANALYSIS 63
11 DATA HANDLING 65
12 MONITORING, AUDIT & INSPECTION 66
13 ETHICAL AND REGULATORY CONSIDERATIONS 66
14 DISSEMINIATION POLICY 69
15 REFERENCES 701
16 APPENDICES 73

LIST OF ABBREVIATIONS

ACS	Acute Coronary Syndrome
AUC	Area Under the Curve
AE	Adverse Event
AR	Adverse Reaction
CA	Competent Authority
CABG	Coronary Artery Bypass Graft
CGM	Continuous Glucose Monitoring
CHI	Community Health Index
CI	Chief Investigator
CRO	Contract Research Organisation
CRP	C-Reactive Protein
CSII	Continuous Subcutaneous Insulin Infusion
CTA	Clinical Trial Authorisation
CTU	Clinical Trials Unit
CTIMP	Clinical Trial of Investigational Medicinal Product
CV	Cardiovascular
CVA	Cerebrovascular Accident
DKA	Diabetic Ketoacidosis
DSUR	Development Safety Update Report
EC	European Commission
ECG	Electrocardiogram
eCRF	Electronic Case Report Form
EDTA	Ethylenediaminetetraacetic acid
eGFR	Estimated Glomerular Filtration Rate
EMEA	European Medicines Agency
EU	European Union
EUCTD	European Clinical Trials Directive
EudraCT	European Clinical Trials Database
EudraVIGILANCE	European database for Pharmacovigilance
FBC	Full Blood Count
FGM	Flash Glucose Monitoring
GCP	Good Clinical Practice
GDG	Guideline Development Group
GDPR	General Data Protection Regulation
GLP-1	Glucagon Like Peptide-1
GLP-1RA	Glucagon Like Peptide-1 Receptor Agonist
GMP	Good Manufacturing Practice
HbA1c	Glycated haemoglobin
IB	Investigator Brochure
ICF	Informed Consent Form
ICH	International Conference on Harmonisation of technical requirements for registration of pharmaceuticals for human use.
IDMC	Independent Data Monitoring Committee
IMP	Investigational Medicinal Product
IMPD	Investigational Medicinal Product Dossier
ISF	Investigator Site File
ISRCTN	International Standard Randomised Controlled Trials Number

IVRS	Interactive Voice Randomisation System
IWRS	Interactive Web-based Randomisation System
LPLV	Last Patient Last Visit
MA	Marketing Authorisation
MDRD	Modification of Diet in Renal Disease
MODY	Maturity Onset Diabetes of the Young
MHRA	Medicines and Healthcare products Regulatory Agency
MI	Myocardial Infarction
MDI	Multiple Daily Injections
NHS GG&C	National Health Service Greater Glasgow & Clyde
NHS R&D	National Health Service Research & Development
NICE	The National Institute for Health and Care Excellence
NIMP	Non-Investigational Medicinal Product
PI	Principal Investigator
PIC	Participant Identification Centre
PIS	Participant Information Sheet
PV	Pharmacovigilance
QA	Quality Assurance
QC	Quality Control
QoL	Quality of Life
QP	Qualified Person
RCT	Randomised Control Trial
REC	Research Ethics Committee
SAE	Serious Adverse Event
SAP	Statistical Analysis Plan
SAR	Serious Adverse Reaction
SDV	Source Data Verification
SGLT2	Sodium Glucose Transporter 2
SOP	Standard Operating Procedure
SUNY	State University of New York
SmPC	Summary of Product Characteristics
SSI	Site Specific Information
SUSAR	Suspected Unexpected Serious Adverse Reaction
TIA	Transient Ischaemic Attack
TMG	Trial Management Group
TSC	Trial Steering Committee
TMF	Trial Master File
UB	University of Buffalo
ULN	Upper Limit of Normal reference range
WOCBP	Women Of Childbearing Potential

TRIAL FLOW CHART

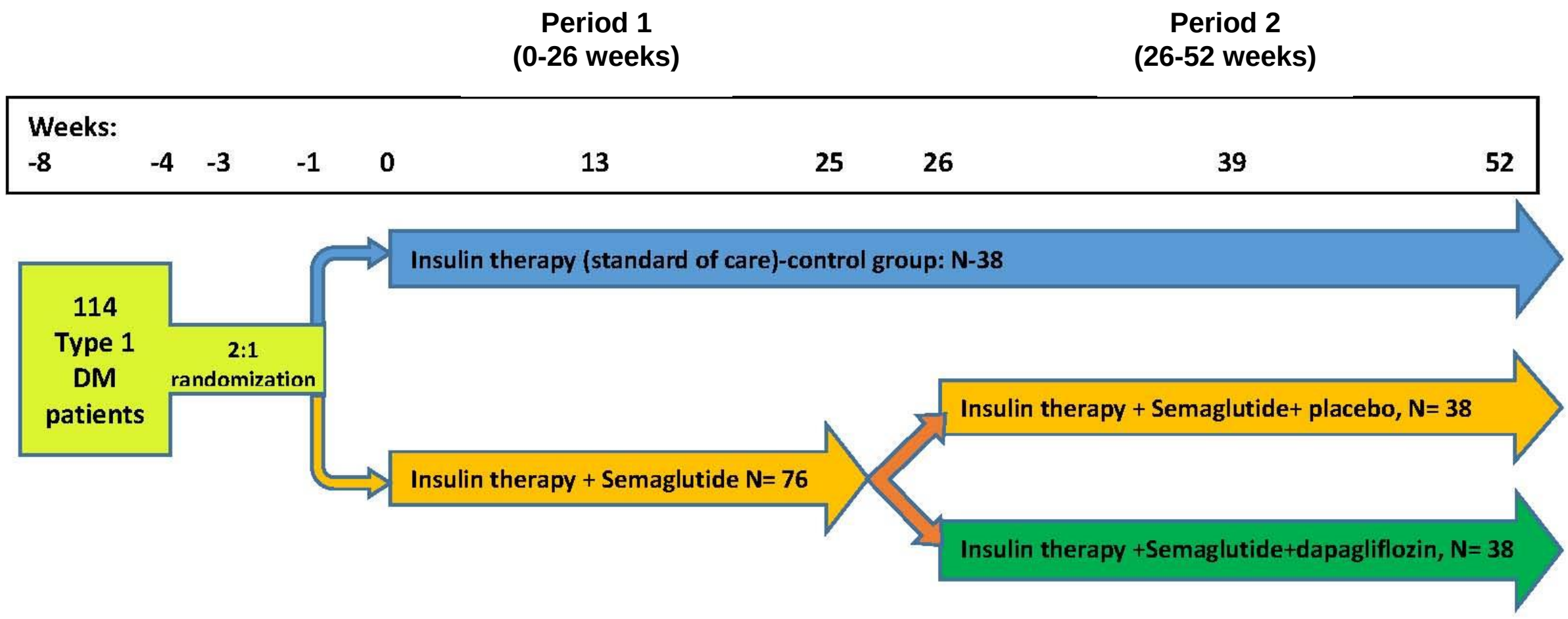

SCHEDULE OF ASSESSMENTS

**Visit #**	**-**	**1**	**2**	**3**	**4**	**5**	**6**	**7**	**8**	**9**	**10**	**11**	**12**	**13**	**14**	**15**	**16**
**Week# (±7 days; see below)**	**-8 to -4**	**-3**	**-1**	**0**	**1**	**2**	**4**	**13**	**23**	**25**	**26**	**27**	**31**	**39**	**49**	**51**	**52**
Study Procedures	Screening	Run-in period		Randomise							Randomise						End
Informed Consent	X																
Fasting visit			X	X				X		X	X			X		X	X
History and demographics	X																
Physical	X																X
Education on hypoglycaemia and ketosis avoidance)				X							X						
BP, heart rate, weight	X	X	X	X	X	X	X	X	X	X	X	X	X	X	X	X	X
Bloods (FBC, kidney and liver profiles)	X			X				X			X		X	X			X
C-peptide	X										X						X
Pregnancy test	X	X		X			X[1]	X[1]	X[1]		X[1]		X[1]	X[1]			X[1]
Dispense/ titrate open-label semaglutide[2]				X	X[2]	X[2]	X[2]	X[2]			X[2]			X[2]			
Dispense dapagliflozin/placebo											X	X		X			
Dapagliflozin/placebo return capsule counts												X		X			X
HbA1c	X			X				X		X				X			X
Blood endpoints (including fructosamine)				X				X			X			X			X
Masked CGM fit (enter data this visit)		X							X						X		
Masked CGM read				X							X						X
Daily fingerstick glucose review		X	X	X	X	X	X	X	X	X	X	X	X	X	X	X	X
Daily urinary ketones review												X	X	X	X	X	X
Insulin dose review		X	X	X	X	X	X	X	X	X	X	X	X	X	X	X	X
Ketone bodies (blood/urine)	X	X	X	X	X	X	X	X	X	X	X	X	X	X	X	X	X
Adverse Event reporting		X	X	X	X	X	X	X	X	X	X	X	X	X	X	X	X
Meal Challenge Test			X							X						X	
24 hr Urine dispensed			X				X			X			X			X	
24 hr Urine collected				X				X			X			X			X
Quality of life questionnaires				X							X						X

N.B. Safety phone calls will be conducted at weeks 3, 5, 8, 16, 20, 29, 35, 44 and at two weeks after the end of the study to document any side effects or other changes. **Abbreviations:** see pages 14-15

Visit dates should be within seven days of the visit time; however, a longer or shorter interval does not constitute a protocol deviation

[1]Not required in women randomised to "Insulin therapy (standard of care)

[2]According to randomisation schedule: resupply of open-label semaglutide after Visit 3 will be according to individual requirements as tolerated (see Section 8.3)

STUDY PROTOCOL
Triple Therapy for Type 1 Diabetes with Insulin, Semaglutide, and Dapagliflozin

1 BACKGROUND

The control of glucose homeostasis in type 1 diabetes is fragile since β-cell reserve is negligible. Traditionally, insulin has been the only treatment approach available. This often leads to erratic blood glucose control given the challenge of matching exogenous insulin to mealtime glucose excursions in the face of a lack of significant endogenous insulin to compensate for variation in requirements in response to meals and activity levels. Injected insulin boluses rarely match precise insulin requirements either in terms of dose administered or bio-availability. The result often is wide fluctuations in blood glucose including episodes when it is too low (hypoglycaemia). Based on our work to date and other published reports, previous attempts to add other glucose-lowering medications to insulin therapy yielded minimal benefits on glycaemia (although metformin may have other benefits) (1-3).

Dandona et al. have shown in two preliminary retrospective studies that the addition of liraglutide, a Glucagon Like Peptide -1 receptor agonist (GLP-1RA), to insulin results in consistent improvements in glycemic control in type 1 diabetes as demonstrated by reductions in HbA1c, glycemic excursions, and insulin requirements accompanied by improvements in body weight and systolic blood pressure (4, 5). As collaborators in the present trial, they also completed the first prospective randomised controlled trial of this drug in 72 people with type 1 diabetes. The data not only confirmed improvements in glycemic control (HbA1c), body weight and systolic BP, but also demonstrated a reduction in appetite, carbohydrate intake and low-grade inflammation as measured by plasma CRP concentrations (6). Importantly, quality of life was also improved. Their recent studies in type 2 diabetes have revealed that systolic blood pressure reduction with GLP-1RA (exenatide) treatment in type 2 diabetes is associated with increased plasma concentrations of ANP, cGMP and cAMP along with reduced concentrations of angiotensinogen, renin and angiotensin II (7). It is possible that these mechanisms play a role in the prevention of cardiovascular complications in type 1 diabetes. There have been no trials to date of semaglutide (a weekly and potent GLP-1RA) in type 1 diabetes.

GLP-1RAs, therefore, have great potential as adjunct therapy for type 1 diabetes. However, mean HbA1c was still ≥7.0% after liraglutide treatment (6) and larger pharma-sponsored trials (ADJUNCT-1 and ADJUNCT-2) reported increased rates of hypoglycaemia (8, 9). In seeking further improvement, the advent of Sodium-GLucose coTransporter 2 (SGLT2) inhibitors is of importance since their mode of action is independent of insulin secretion. Introduced for use in type 2 diabetes, SGLT2 inhibitors are selective antagonists of the sodium-glucose transporter subtype expressed in the proximal convoluted tubules of the kidneys. They act by inhibiting renal glucose re-absorption and therefore stimulating excretion of 60-100 grams per day of glucose in urine. SGLT2 inhibitors have been studied extensively in type 2 diabetes, including the landmark cardiovascular outcome trial EMPA-REG (10), but data have only more recently begun to accumulate in type 1 (11). Short term effects on urine glucose excretion of remogliflozin and empagliflozin are similar to those seen in type 2 diabetes and these translate into improved glycemic and other metabolic indices including HbA1c; fasting blood glucose, rates of hypoglycaemia, daily insulin dose and weight (12). Weight loss accompanying increased urinary glucose excretion could translate into reduced cardiovascular complications as in type 2 diabetes, but there are no data so far available to support this hypothesis. More recently, in the Phase 3 trials DEPICT-1 and DEPICT-2 Dandona et al (PI of the US arm of this trial) showed that dapagliflozin improves glycemic control and HbA1c over six months by about 0.45% without increasing hypoglycaemia. (13, 14, 15). In comparison to other SGLT2 inhibitors studied for use in type 1 diabetes [sotagliflozin and empagliflozin (16, 17)] the risk of diabetic ketoacidosis was relatively modest (see below). Both dapagliflozin and sotagliflozin were approved early in 2019 by the EU Committee for Medicinal Products for Human Use (CHMP) as adjunct therapy in type 1 diabetes for individuals with BMI≥27 kg/m^2.In August 2019, dapagliflozin (5 mg/ day) was recommended by the National Institute for Health and Care Excellence as cost-effective for this indication in NHS England (17, 18, 19).

2 RATIONALE

General hypothesis: adding an SGLT2 inhibitor (dapagliflozin) to a combination of insulin and semaglutide will improve glycaemic control, weight and blood pressure in adults with type 1 diabetes.
Our protocol is designed to allow an objective assessment of whether this triple therapy combination will work effectively and safely so that a majority of people with type 1 diabetes can reach a glycemic target HbA1c of ≤7.0%. In addition, since both semaglutide and dapagliflozin inhibitors reduce blood pressure and weight, it will allow an assessment of the combined effect of GLP-1RAs and SGLT2 inhibitors on these outcomes. Since there are no previous data on the effects of once weekly semaglutide in type 1 diabetes, or the combination of semaglutide and dapagliflozin compared to standard insulin treatment, the design requires allocation of some participants to insulin therapy only (the current standard of care).

As part of the process by which this trial was awarded funding by JDRF, this research question was considered by lay members of the funding panel and deemed important, relevant and timely. We consider that use of placebo matching dapagliflozin in Period 2 of the trial is justified as although it has full approval as adjunct therapy for type 1 diabetes, it remains uncertain which adults with type 1 diabetes are suitable for this treatment. In their review, NICE considered it likely that fewer than 10% of adults with type 1 diabetes will be initiated on SGLT2 inhibitor therapy over the first five years after their favourable decision; this is the period of time during which this trial is being conducted. As individuals with a slightly lower BMI threshold will be included than recommended by the SmPC, trial participants will be more closely monitored for ketosis than is the case in routine clinical care. Dapagliflozin (or placebo) will be administered orally once daily as per current licensed doses in type 2 diabetes (5 mg/day and 10 mg/day: the same doses as used in DEPICT-1 and DEPICT-2) (13,14,15, 20). We have not incorporated blinding for semaglutide in the design as the effects of the closely-related daily GLP-1RA liraglutide have been well-studied in double-blind trials in type 1 diabetes by ourselves and others, and individuals in both arms compared for the primary outcome comparison will be treated with this agent. (6, 8, 9). Semaglutide will be administered subcutaneously once weekly.

2.1 Assessment and management of risk

a) Benefits

Semaglutide (Ozempic): is a once weekly injectable (subcutaneous) GLP1-RA licensed for reducing HbA1c in type 2 diabetes. The licensed doses are 0.25 mg weekly, 0.5 mg weekly and 1.0 mg weekly. In the trial, the starting dose is 0.25 mg weekly uptitrated if tolerated to 0.5 mg weekly after two weeks and then (again if tolerated) after a further two weeks to the maximum dose of 1.0 mg weekly. Semaglutide differs from other weekly GLP-1 receptor agonists in that its half-life is extended by binding to serum albumin i.e. it is not macromolecular. In type 2 diabetes, semaglutide therapy is associated with weight reduction and a reduction in rates of cardiovascular events (21). The licensed dose escalation schedule for routine treatment of type 2 diabetes is at four weekly intervals but in a Phase 2 trial in type 2 diabetes authored by one of this trial's investigators (JP) doses were uptitrated at one-to-two weekly intervals and to higher doses (up to 1.6 mg weekly) (22). Phase 3 trials are currently in progress (for the indication of obesity) using a 2.4 mg semaglutide weekly. It should be noted that semaglutide 0.25 mg is considered too low a dose for maintenance therapy in type 2 diabetes.

While semaglutide once weekly has not been investigated previously in type 1 diabetes, liraglutide (a GLP-1 RA for daily administration with a similar molecular stucture that binds less avidly to serum albumin) has previously been investigated (see above) in the ADJUNCT 1 and ADJUNCT 2 trials (8,9) in which, compared with placebo it resulted in reductions in HbA1c, weight and insulin requirement, albeit with a small increase in rates of hypoglycaemia (see below).

Dapagliflozin (Forxiga): is a once daily oral SGLT2 inhibitor licensed in Europe for reducing HbA1c in people with type 1 diabetes and BMI ≥ 27 kg/m^2 when insulin alone does not provide adequate glycemic control despite optimal insulin therapy (23, 24). The licensed dose for type 1 diabetes is 5 mg once daily but both 5 mg and 10 mg daily are licensed for type 2 diabetes. In the Phase 3 trials of dapagliflozin in type 1 diabetes (DEPICT-1 and DEPICT-2) both doses were used. In both DEPICT-1 and DEPICT-2 there were modest additional benefits with 10mg compared to 5mg dapagliflozin. For example, in DEPICT-1, insulin dose requirement was reduced by 13.2% vs placebo on 10mg daily compared with 8.8% on 5mg daily (13). Weight reduction vs placebo in DEPICT-1 at 52 weeks was by 4.5% on dapagliflozin 10mg daily compared with 2.95% in those receiving 5mg daily. Given the propensity of insulin to encourage weight gain over time and the life-long insulin requirement in T1D, sustained weight reduction and insulin dose reduction - even when modest - may be clinically important. There was no increase in the rate of hypoglycaemia in either the 5mg or 10mg groups compared with placebo. (13,14) Dapagliflozin therapy in type 2 diabetes has been demonstrated to be associated with cardiovascular risk reduction: in both DECLARE-TIMI and DAPA-HF the 10mg dose was used. Such cardiovascular benefits may translate to type 1 diabetes. Given the potential glycaemic and cardiovascular benefits with relatively low increased risk, 10mg rather than 5mg dose was selected for steady state treatment in this protocol. (25,26)

b) Risks/ side effects Please also refer to the respective UK SmPCs (21, 24)

Semaglutide:
Very common: nausea, diarrhoea; hypoglycaemia (when used with insulin).
Common: vomiting, abdominal pain, constipation, gastro-oesophageal reflux, cholelithiasis, weight loss.
Uncommon: injection site reactions.

Hypoglycaemia/ ketosis: Semaglutide once weekly has not been studied previously in type 1 diabetes; however, the ADJUNCT-1 and ADJUNCT-2 trials examined the role of liraglutide once daily (a similar molecule in the GLP-RA class that binds less avidly to serum albumin). Although HbA1c and weight were reduced by both 1.2 mg and 1.8 mg liraglutide, rates of symptomatic hypoglycaemia increased by 20-30%. In ADJUNCT-1, insulin doses were re-uptitrated quite rapidly towards baseline levels after an initial dose reduction, but in ADJUNCT-2 were “capped” (and therefore remained lower). Perhaps for that reason, an increase in hypoglycaemia was less consistent in ADJUNCT -2 but there was an excess of ketosis the higher (1.8 mg) dose, presumably due to under-insulinisation (no lower limit for insulin dose reduction was set). It should be noted that monitoring of participants by site investigators in the ADJUNCT trials was less intensive than will be the case in the present trial with less frequent opportunities for insulin dose titration (see Section 7). Importantly, participants at high risk of hypoglycaemia, (including those with a history of severe hypoglycaemia and those with hypoglycaemia unawareness) were not excluded (8,9).

Retinopathy: In the SUSTAIN-6 cardiovascular outcome trial of semaglutide in type 2 diabetes, rapid reduction of HbA1c was associated with progression of existing proliferative retinopathy in those with a high baseline HbA1c (“early worsening”/ “normoglycaemic re-entry”). However, there was no evidence of worsening of retinopathy on treatment in the trials SUSTAIN 1-5 in which those with proliferative retinopathy were excluded (22). In clinical trials of duration up to one year involving 4,807 patients with type 2 diabetes, adverse events related to diabetic retinopathy were reported in similar proportions of subjects treated with semaglutide (1.7%) and comparators (2.0%) (21, 27).

Other adverse effects: In type 2 diabetes, gastrointestinal adverse effects of semaglutide can be prevented in the majority of cases by careful dose uptitration (21,22) although up to 5% are ultimately unable to tolerate it. In our previous trial with liraglutide in T1D, adverse effects occurred with a similar

frequency to that observed in T2D (in which there is extensive clinical experience); on this basis it is expected that this will also be the case with semaglutide (28).

__Dapagliflozin__:
Very common: hypoglycaemia (when used with insulin).

Common: ketoacidosis, genital infections (vulvovaginitis, balanitis), urinary tract infection, diabetic ketoacidosis,.

In the DEPICT-1 study, ketoacidosis occurred in 4 of 277 participants on 5mg dapagliflozin, 5 of 296 participants on 10 mg and 3 of 260 participants in the placebo group (summarised as 1%, 2% and 1% respectively) with one severe episode in each group (13). In pooled analyses of DEPICT-1 and DEPICT-2 (13,14) there was, however, an approximate threefold elevated rate of DKA adjudicated as "definite" over placebo on active therapy: 2.0% on 5mg dapagliflozin, 1.9% on 10mg and 0.6% on placebo – i.e. rates of DKA were similar with the two doses (13,14,15). Rates of DKA with sotagliflozin, the other SGLT2 inhibitor licensed for type 1 diabetes, were somewhat higher (2.9% on 200 mg, 3.8% on 400 mg, 0.2% on placebo) in pooled analyses of Phase 3 trials (29,30,31).

Uncommon: fungal infection, volume depletion, constipation.

Not known (several case reports): Fournier's gangrene.

Fournier's gangrene is a very rare clinical entity characterized by necrotizing fasciitis of the perineal area. This has been infrequently reported in association with SGLT-2 inhibitor use in both men and women. However, there was no evidence of increased risk in a meta-analysis of 84 clinical trials involving 42415 participants. Nevertheless, participants will be advised to seek urgent medical assistance if they experience erythema, pain, swelling of the perineal area associated with fever and malaise (this will be reinforced in the Patient Information Sheet). (32)

Amputation: An increased incidence of lower limb amputation has been reported in type 2 diabetes with the SGLT-2 inhibitor canagliflozin. However this has not been demonstrated with dapagliflozin (not included in the UK SmPC). People with diabetes are at increased risk of amputation and good foot care is a key component of good diabetes care regardless of use of SGLT-2 Inhibitors (24).

__Placebo__:
Placebo capsules contain microcrystalline cellulose.
This trial is therefore categorised by the Co-Sponsors as:

- Type B = Somewhat higher than the risk of standard medical care

See Appendix 1 (Risk Assessment)

3 OBJECTIVES AND OUTCOME MEASURES/ ENDPOINTS

3.1 Primary objective

- To assess in people with type 1 diabetes whether triple therapy (dapagliflozin, semaglutide and insulin) for six months improves glycaemic control (as measured by HbA1c) compared with dual therapy (placebo, semaglutide and insulin).

3.2 Secondary objectives

To assess in people with type 1 diabetes whether:

- Triple therapy (dapagliflozin, semaglutide and insulin) improves glycaemic control (as measured by HbA1c) compared with standard therapy (placebo and insulin)
- Dual therapy (semaglutide and insulin) improves glycaemic control (as measured by HbA1c) in comparison with standard treatment (insulin only)
- Triple therapy (dapagliflozin, semaglutide and insulin) improves glycaemic control as assessed by a range of continuous glucose monitoring (CGM) parameters, lowers body weight, reduces insulin requirement, improves blood pressure and improves quality of life (as assessed by validated patient questionnaires) compared with dual therapy and standard therapy.

3.3 Outcome measures/endpoints

3.4 Primary endpoint/outcome

Change in HbA1c (week 26 to week 52)

3.5 Secondary endpoints/outcomes

Efficacy:

Change in HbA1c

Mean fasting blood glucose

Mean blood glucose*
Percentage time in range (3.9 - 10.0 mmol/)*
Standard deviation of blood glucose*
Percentage time in hyperglycaemia Level 1 (10.1 - 13.9 mmol/L/)*
Percentage time in hyperglycaemia Level 2 (>13.9 mmol/L) *
Percent time in hypoglycaemia Level 1 (3.0 - 3.8 mmol/)*
(by masked DEXCOM G6 CGM)*

Insulin requirement
Bodyweight
Systolic and diastolic BP
Number of antihypertensive medications
Diabetes Specific Quality of Life Scale (DSQOLS)
Problem Areas In Diabetes (PAID) questionnaire

Safety:

Rate of hypoglycemic events Level 2 (<3.0 mmol/L/ <54mg/dl)
Rate of hypoglycemic events Level 3 (severe event with altered mental and/or physical status requiring assistance)
Rate of diabetic ketoacidosis defined as elevated serum or urine ketones (greater than the upper limit of the normal range), and serum bicarbonate < 15 mmol/L or blood pH < 7.3

3.6 Tertiary endpoints/outcomes

Postprandial glucose (AUC) – meal tolerance test
Postprandial glucagon (AUC) – meal tolerance test
Postprandial FFA (AUC) – meal tolerance test
Postprandial GLP-1 (AUC) – meal tolerance test
Fructosamine

3.7 Exploratory endpoints/outcomes

Plasma and urine samples will be collected and frozen for later analysis for indices of inflammatory stress and other relevant measures.

3.8 Table of endpoints/outcomes

**Endpoints**	**Outcome Measures**	**Timepoint(s) of evaluation of this outcome measure**
**Primary endpoint**<br>Triple therapy (dapagliflozin, semaglutide and insulin) in comparison with dual therapy (placebo, semaglutide and insulin)	Change in HbA1c	Week 26 to Week 52
**Secondary endpoint 1**<br><br>Triple therapy (dapagliflozin, semaglutide and insulin) in comparison with standard therapy (insulin alone)	Change in HbA1c	Week 0 to Week 52
**Secondary endpoint 2**<br><br>Dual therapy (semaglutide and insulin) in comparison with standard therapy (insulin only)	Change in HbA1c	Week 0 to Week 26
**Other secondary endpoints**<br><br>a) Triple therapy (dapagliflozin, semaglutide and insulin) in comparison with semaglutide, insulin and placebo (dual therapy)<br><br>b) Dual therapy (semaglutide and insulin) in comparison with standard therapy (insulin only)	Change in:<br>- Mean fasting blood glucose (4 days)<br><br>- Mean blood glucose*<br>- Percentage time in range (3.9–10 mmol/L)*<br>- Glycaemic variability (SD)*<br>- Percent time in hyperglycaemia Level 1 (10-13.9 mmol/L)*<br>- Percent time in hyperglycaemia Level 2 (>13.9 mmol/L)*	Week 26 to Week 52<br><br>Week 0 to Week 26

	- Percent time in hypoglycaemia Level 1* (3.0-3.8 mmol/L)<br>*(*by masked Dexcom G6 CGM)*<br><br>Insulin requirement<br>Body Weight<br>Systolic and diastolic blood pressure<br>Number of antihypertensive medications<br>Diabetes Specific Quality of Life Scale (DSQOLS)<br>Problem Areas In Diabetes (PAID) questionnaire	
**Tertiary endpoints**<br>a) Triple therapy (dapagliflozin, semaglutide and insulin) in comparison with dual therapy (semaglutide, insulin and placebo)<br><br>b) Dual therapy (semaglutide and insulin) in comparison with standard therapy (insulin only)	Change in:<br>Postprandial glucose (AUC)<br>Postprandial FFA (AUC),<br>Postprandial GLP-1 (AUC)<br>Postprandial glucagon (AUC)	Week 26 to Week 52<br><br>Week 0 to Week 26
**Safety endpoint 1**<br>a) Triple therapy (dapagliflozin, semaglutide and insulin) in comparison with dual therapy (semaglutide, insulin)<br><br>b) Dual therapy (semaglutide and insulin) in comparison with standard therapy (insulin only)	Difference between treatment arms in:<br>(i) Rate of Level 2 (<3.0 mmol/L/ <54mg/dL) hypoglycemic events (by Dexcom G6 CGM)<br>(ii) Rate of Level 3 hypoglycemic events (severe event with altered mental and/or physical status requiring assistance)	Week 26 to Week 52<br><br>Week 0 to Week 26
**Safety endpoint 2**<br>a) Triple therapy (dapagliflozin, semaglutide and insulin) in comparison with dual therapy (placebo, semaglutide, insulin)<br><br>b) Dual therapy (semaglutide and insulin) in comparison with standard therapy (insulin only)	Difference between treatment arms in:<br><br>Rate of diabetic ketoacidosis (serum or urine ketones above normal limits) AND serum bicarbonate < 15 mmol/L OR blood pH < 7.3	Week 26 to Week 52<br><br>Week 0 to Week 26

4 TRIAL DESIGN

The trial has two six-month periods:

Period 1 open-label randomised (2:1) parallel group semaglutide once weekly vs standard of care
Period 2 double-blind, randomised (1:1) parallel group, dapagliflozin vs placebo for those participants initially randomised to semaglutide (with ongoing standard of care for all participants)

Thus, during the first six months, approximately 114 participants with type 1 diabetes (57 in the UK) will be randomised (open label, see below) into three groups (approximately 38 each; i.e. 19 each in the UK) one of which will be maintained on their regular insulin therapy for 12 months (standard therapy group) and two of which will be started on semaglutide in addition to regular insulin treatment for six months (dual therapy). Following six months of stable treatment, those initially randomised to insulin and semaglutide will be additionally randomised (double-blind) to dapagliflozin (triple therapy) or matching placebo (dual therapy) for a further six months. The dose of dapagliflozin will not be increased to 10 mg in those who have confirmed ketonuria (+1) or ketonaemia (≥0.6 mM) after the first week of treatment.

In keeping with the intention-to-treat principle, participants in whom therapy is discontinued or downtitrated due to adverse effects will be encouraged to continue to undertake all study assessments and attend study visits.

5 STUDY SETTING

The study will be conducted at two sites: State University of New York (SUNY) at Buffalo, USA and Glasgow, UK. In Glasgow, n=57 participants (50% of the total) will be identified from secondary care sites and via the Scottish Diabetes Research Network research register. Both randomisations will be stratified by site.

6 ELIGIBILITY CRITERIA

6.1 Inclusion criteria

1) Type 1 Diabetes for at least one year on stable multiple daily (four or more) injections (MDI) of insulin or continuous subcutaneous insulin infusion (CSII) for the previous three months.
2) C-peptide < 0.23 nM
3) Minimum dose of insulin: 0.5 U/kg for MDI and 0.4 U/kg for CSII
4) Regularly measuring blood glucose: four or more times daily either by fingerstick, testing or by "flash"/ continuous glucose monitoring)
5) HbA1c 7.5 – 11.0 % inclusive
6) Well versed in carbohydrate1 counting*
7) Age 18-70 years.
8) BMI ≥25 kg/m^2.

** In the opinion of the site Principal Investigator*

6.2 Exclusion criteria

1) Type 1 diabetes for < 12 months, type 2 diabetes, chronic pancreatitis, MODY
2) Previous use of any agent other than insulin for glycaemic control in the last three months.
3) History of diabetic ketoacidosis (DKA) requiring medical intervention (e.g. emergency room visit and/or hospitalisation) within three months prior to the screening visit.
4) Following ketogenic diet, low (<50g/day) carbohydrate diet or intermittent fasting.

5) Hypoglycaemia unawareness* or frequent episodes of severe hypoglycaemia as defined by more than one episode requiring medical assistance, emergency care (paramedics or emergency room care), or glucagon therapy administered by a third-party individual within three months prior to the screening visit
6) Symptoms of poorly controlled diabetes
7) Participation in a weight loss program with ongoing weight loss, or in an intensive exercise program
8) History of bariatric or other weight-loss surgery within 12 months prior to the screening visit
9) History of Addison's disease or chronic adrenal insufficiency
10) History of diabetes insipidus
11) Hepatic disease or cirrhosis
12) Aspartate Aminotransferase (AST) > 3 X Upper limit of normal (ULN) and/or Alanine aminotransferase (ALT) > 3 X ULN
13) Serum Total Bilirubin > 2 X ULN unless exclusively caused by Gilbert's Syndrome
14) Haemoglobin < 110 g/L (11.0 g/dL) for men; haemoglobin < 100 g/L (10.0 g/dL) for women
15) Coronary event or procedure (myocardial infarction, unstable angina, coronary artery bypass, surgery or coronary angioplasty) in the previous three months or history of congestive heart failure
16) End stage renal disease on haemodialysis and/or eGFR < 60 ml/min/1.73m^2
17) HIV or Hepatitis B/C positive status
18) Any other life-threatening, non-cardiac disease
19) History of pancreatitis or cystic fibrosis
20) Women who are pregnant, planning pregnancy or breast-feeding and women of childbearing potential (WOCBP) who are not established on a method of contraception considered highly effective (see Appendix 2)and willing to continue it for the duration of the study and for at least two months after last dose of IMP.
21) Inability to give informed consent
22) History of gastroparesis
23) History of medullary thyroid carcinoma or MEN 2 syndrome
24) History of hypersensitivity reaction to GLP-1 agonists or SGLT2 inhibitors or known hypersensitivity to any of the excipients
25) Known gallstones (history of cholecystectomy is not an exclusion criterion)
26) History of alcohol problem or drug abuse
27) Hypertriglyceridemia (>500 mg/dl)
28) Recurrent genital mycotic infection (more than one episode in last six months)
29) Hypovolaemia
30) Any malignancy except treated *in situ* malignancy and basal cell carcinoma of the ski
31) Unexplained haematuria (dipstick positive on two occasions one week apart) and/or active bladder cancer
32) Proliferative retinopathy (on basis of photographic retinal screening which must have been performed within the last 12 months
33) Use of an investigational agent or therapeutic regimen within 90 days of study
34) Participation in any other concurrent interventional clinical trial
35) Unlikely to be able to comply with study requirements*

** In the opinion of the site Principal Investigator*

7 TRIAL PROCEDURES

Also see *Schedule of Assessments*

Each participant will be enrolled in the trial for approximately 55 weeks, comprising three weeks of monitoring to stabilise treatment and collect baseline data before randomisation into the intervention or standard of care/ placebo arms for 52 weeks. Following a completed screening visit and meeting the Inclusion and Exclusion criteria, each participant will have a total of 16 scheduled study visits. In addition, at weeks 3, 5, 8, 16, 20, 29, 35, 44 and at two weeks after the end of the study, each participant will receive a phone call to document any adverse effects or other changes. No long-term follow-up is planned following completion of participation in the study.

Participants will be treated with either insulin only (standard treatment) for the whole duration of the study or with insulin and semaglutide weekly s.c. injection (titrated where possible up to 1.0 mg, see below) for the first six month period and then randomised to either oral dapagliflozin (triple therapy) or placebo (dual therapy) daily added for the second six month period. Semaglutide will be started (Visit 4) at a dose of 0.25 mg once weekly for two weeks then increased if tolerated to 0.5 mg (Visit 5). If well tolerated at week 4, it will be increased to 1.0 mg once weekly (Visit 6) or at a subsequent visit at the discretion of the PI. Dapagliflozin (or placebo) will be started at a dose of 5 mg daily at week 26 and increased at week 27 to 10 mg (2 x 5mg capsules) daily to ensure drug tolerability and minimise the risk of insulin-induced hypoglycaemia or hypotension.

Gastrointestinal adverse effects:
During the initial weeks of participation, those participants who report nausea and/or vomiting during uptitration of semaglutide dose that is not sufficiently severe to require treatment discontinuation will remain on their current dose until they are symptom-free prior to discussing a further dose escalation (i.e. uptitration will be to the highest dose tolerated rather than a “forced titration”). Dose will be downtitrated to the maximum tolerated in those who experience gastrointestinal adverse effects.

Retinopathy:
As there was no excess of retinopathy with semaglutide treatment in Phase 3 trials with semaglutide, in which proliferative retinopathy was an exclusion criterion (as in the current protocol), additional retinal imaging beyond annual retinal photography (the standard of care) is not proposed (27).

Insulin dose adjustment:
Minimising hypoglycaemia: All participants will be made aware of the potential for increased hypoglycaemia with addition of adjunct therapy and supported and encouraged to monitor blood glucose at least four times daily in order to minimize the risk of hypoglycaemia. Participants will have ready access to advice from specialist medical and nursing staff at face-to-face and telephone visits. Those who have ongoing access to wearable continuous glucose monitoring [or "flash" glucose monitoring, (FGM)] will continue to use these devices. Information on management of hypoglycaemia will be included within participant diaries, reinforcing the pre-existing knowledge of participants, the majority of whom will have lived with type 1 diabetes for many years.

Reductions in insulin dose will not usually be greater than 10% (5% if HbA1c is < 7.0 %) from current levels. At randomisation, daily insulin dose will not be reduced by more than 20% from the baseline level and then from the new baseline levels at 26 weeks. Participants requiring an insulin dose reduction > 20% to a total insulin dose < 0.4 U/kg (current weight) will be monitored closely for insulin dose, carbohydrate intake, ketones and blood glucose. Following the second randomisation at 26 weeks, dapagliflozin/ placebo will be temporarily discontinued if insulin dose falls below this threshold and permanently discontinued if it remains so at review in one week.

Minimising ketosis: Participants will be instructed to stay hydrated and not to reduce their caloric intake (especially carbohydrates) or start new dietary approaches (especially ketogenic diets) during the study. They will be educated to recognize the signs and symptoms of diabetic ketoacidosis (DKA) and to undertake additional self-testing of urine and/or blood ketones if they develop nausea, vomiting, or anorexia. In addition, they will be given education and a written ketosis management plan (STICH Protocol – i.e. STop SGLT-2 inhibitor or placebo (temporarily), give Insulin bolus, take additional Carbohydrate, and additional Hydration - see Appendix 3).

From Visit 10 (26 weeks), those participants undergoing the second randomisation will be supported and encouraged to monitor urine or blood ketones at home at least once daily: three days before visit 10 (week 26), they will be asked to start daily measurements of urine ketones [CombiScreen® 10 SYS Plus urine testing strips, Sterilab, UK (or equivalent)] and blood ketones [CareSens™ Dual, i-SENS, Koreal; GlucoMen® Areo 2K, Menarini, UK (or equivalent]]. They will be asked to record these in the diary/ log provided. In addition, ketones (blood and urine) will be measured at every visit. If ketone bodies in urine are +1 or greater (≥0.6 mM in blood), participants will be reminded to keep well hydrated and notify the study team as soon as possible (office hours) as diabetic ketoacidosis (DKA) can occur even at glucose levels < 14 mmol/L (34).

If ketones are +2 or more in urine (> 1.5 mmol/L) participants will be advised to initiate the STICH Protocol immediately and contact the study team in office hours (or telephone NHS 24 or attend their Accident & Emergency if out of hours). This information will be reinforced verbally at study visits and also in the form of a Patient Alert Card that participants will be asked to carry. This will also contain details of the investigational study drug, the study number, the investigator's name, and a contact number (for in hours contact). The study team will assess for symptoms or test results indicating a risk of ketoacidosis immediately on reporting and if blood ketone levels are persistently (one week) at these levels dapagliflozin/placebo will be permanently discontinued (24).

If blood ketone readings are confirmed as > 3.0 mmol/L (or urine ketones reading +++) participants will be advised to stop dapagliflozin/ placebo and attend the Accident and Emergency department without delay.

Participants will be advised that dapagliflozin/placebo may be temporarily stopped in other clinical situations known to predispose to ketoacidosis (e.g. acute serious medical illnesses or hospitalisation for major surgical procedures). Any requirement for intravenous insulin infusion (whether in Accident and Emergency or as an in-patient admission) will be recorded as a Serious Adverse Event. For further detailed procedures see section 9.31 (25).

Volume depletion:
There is a theoretical risk of volume depletion and low blood pressure when dapagliflozin is used in combination with other anti-hypertensive drugs. All pre-existing concomitant medication will be reviewed at the screening visit as part of the medical history and examination (see Section 7.1.3 for further details). Participants undergoing the second randomisation will be educated on symptoms suggestive of low blood pressure (e.g. light-headedness, dizziness, feeling faint) and asked to contact the study team if they experience these symptoms. In clinical practice however SGLT-2 inhibitors are commonly used with anti-hypertensive drugs with no significant problems (27).

Genital mycotic infections:
Individuals who report symptoms suggestive of genital mycotic infection will be advised to self-treat with topical antifungal preparations. If a second episode occurs, a decision on treatment discontinuation will

be considered in discussion between the participant and the site Principal Investigator, taking account of the severity of the symptoms.

Foot care:
Annual foot checks will be conducted for all participants as per standard of care.

7.1 Recruitment

7.1.1 Patient identification

All personal details will be managed according to ISO 27001:2013 compliant Standard Operating Procedures.

Potentially eligible participants will be identified from the Scottish Diabetes Research Network Research Register (a managed and searchable database of people with diabetes who have agreed to be contacted about clinical trial participation) and at secondary care diabetes clinics in Greater Glasgow and Clyde. The principal site for recruitment will be Stobhill Ambulatory Care Hospital but other sites may be opened if necessary as Patient Identification Centre sites. The majority of study visits (and all visits involving drug dispensing) will be conducted at the Clinical Research Facility, Glasgow Royal Infirmary. Information posters with the contact details of the research team will be displayed in recruiting clinics.

Clinic lists will be pre-reviewed by members of the usual care team in order that potentially eligible individuals can be sent an abbreviated Participant Information Sheet by post one week before their routine scheduled review visit. Eligibility criteria of those indicating agreement to be approached will then be checked at the routine visit, and the study procedures explained. Potential participants will be given a Patient Information Sheet and an Expression of Interest Form (with prepaid envelope) at this time and will be asked for permission to contact again to discuss further and (if appropriate) arrange a screening visit.

7.1.2 Screening

A separate non-fasting visit will be arranged at which potential participants will have further time to discuss with the study nurse and doctor.

Prospective participants will be asked to read and understand the Participant Information Sheet. Any questions regarding the Protocol will be answered. Risks and side-effects of the active trial medication will be explained. Eligibility for trial enrolment will be confirmed by a medical practitioner.

Individual wishing to participate in the study will then be invited to sign to document their informed consent (see Section 7.2 below).

7.1.3 Screening assessments
Items from the medical history relevant to the Inclusion and Exclusion criteria will be recorded. Demographic data, medical history and current medications (including current insulin doses) will be documented. All participants will have access to a dual blood glucose and ketone meter as per the contemporary standard of care in type 1 diabetes. A physical examination will be conducted. Clinical observations will be recorded and blood and urine samples obtained (see below):
<u>Demographics</u>
- Date of birth
- Sex

- Ethnicity
- Date of diagnosis
- Ethnic group: white/black/Asian/other
- Height

Clinical assessments
- Systolic and diastolic blood pressure (after 5 minutes rest)
- Heart rate
- Body weight (clothed without coat and shoes)
- Capillary blood glucose

Medical History
- Smoking status: current/ex/never
- Alcohol consumption: units/week
- Hypertension: Y/N
- Microalbuminuria: Y/N
- Proteinuria: Y/N
- Neuropathy: Y/N
- Previous major cardiovascular event(s): Y/N (date)
- Existing CV disease of any type*: Y/N
- Amputation above forefoot: Y/N
- Hospitalisation for DKA in last year
- Level of educational attainment: University (college) educated Y/N

* Existing CV Disease is defined as any of: MI, stroke, ACS/unstable angina, coronary stent/angioplasty, CABG, TIA, heart failure, peripheral vascular disease

Insulin treatment
- Regimen: MDI, pump,
- Total dose (all); Basal (longer acting) dose/ Bolus (shorter acting) dose (for MDI only)

Other treatment (drug classes and names)

Current use Y/N. If no, has there been use in last three months?: Y/N

- ACE inhibitors
- Angiotensin receptor blocker
- Calcium channel blocker
- Alpha-blocker
- Beta-blocker
- Loop diuretic
- Thiazide like diuretic
- Statin
- Aspirin
- Clopidogrel
- Novel anticoagulant
- Warfarin

Blood assessments:

- Blood ketones (from participant's own ketone meter)
- HbA1c
- FBC
- Urea and electrolytes
- Creatinine
- Liver function tests (AST/ ALT/ Alkaline Phosphatase)
- Bone profile (Calcium/ Phosphate)
- C-peptide (capillary blood glucose must be > 4.0 mmol/L at time of sampling)

If blood tests have been assessed for clinical purposes within the last three weeks, these values will be used.

Urine sample:

- Urine ketones
- For all WOCBP, a urine pregnancy test will be checked at screening

Potential participants meeting all the inclusion and exclusion criteria based on history, examination and screening tests will be enrolled in the study.

Pregnancy: WOCBP will be asked about pregnancy status and contraceptive usage (see Appendix 2) and a pregnancy test will be conducted (following informed consent) prior to randomisation. We will not recruit those who are pregnant, wanting to become pregnant, or using birth control methods not considered highly effective. Every four weeks following randomisation, WOCBP randomised to IMP (as opposed to standard of care) will be asked about their contraception status, and a urine pregnancy test carried out. Study medication will be permanently discontinued in women who become pregnant or considered to be on contraception that is not highly effective. As the study assessments *per se* do not carry an additional risk over standard of care to either mother or unborn child, further pregnancy tests will not be routinely offered following randomisation to those women allocated to "Insulin therapy (standard of care)" (i.e. who are not exposed to IMP). All pregnancies will be notified to the Sponsor Pharmacovigilance Officer using the standard pregnancy notification form and the pregnancy followed to outcome).

7.1.4 Payment

Participants will be offered reasonable travel expenses for visits additional to usual care.

7.2 Consent

Potential participants will be identified and screened by the Inclusion and Exclusion criteria listed above. Medical staff or appropriately trained support staff will seek consent for screening and participation in the trial from individuals that fulfil the criteria. All potential participants will have at least 24 hours to review the Patient Information Sheet before being approached (see Section 7.1.2 above). Following written consent, each signature will be dated by the signatory, the original retained in the site file, a copy provided to the participant and a copy scanned into the participant's Clinical Portal record.

Data collected for routine clinical care will be used for clinical trial documentation (e.g. blood results within the previous three weeks). In the absence of routine blood results, sampling of blood for study specific laboratory measurements can only be performed following documentation of informed consent.

7.2.1 Re-screening post-consent

Where inclusion (e.g. HbA1c ≤7.5%) or exclusion (e.g. DKA within the last three months) criteria are not met (e.g. blood results falling outside the study parameters) potential participants can be re-screened with additional blood tests on one further occasion. If the initial consent was signed within two months of the re-testing, new consent is not required unless the consent form template has been updated in this period. If the initial consent was not signed within two months of re-testing then consent should be sought again. Rescreened participants will keep the same 5-digit Patient ID and data will be amended on the web portal as appropriate.

7.3 Run-in period

7.3.1 Visit 1 (≅3 weeks prior to randomisation)

Participants who meet entry criteria will be scheduled for their next visit at approximately three weeks prior to randomisation. Daily fingerstick blood glucose levels (study diaries), CGM/ FGM data or insulin pump download data (as applicable) will be reviewed and appropriate insulin dose adjustments made by the medical team to target fasting blood glucose concentrations between 5 -10.0 mmol/L if possible at all times without having additional hypoglycaemia (< 3.9 mmol/L). Clinical observations will be recorded including resting BP measurement. Blood glucose and ketone bodies will be measured via a fingerstick blood sample (see Section 7). Urine for pregnancy status and ketone bodies will be assessed.

Participants will be instructed on the use of the Continuous Glucose Monitoring System (Dexcom G6 with blinded receiver). All subjects will be advised to monitor their capillary blood glucose by CGM or fingerstick at least 4 times a day (before meals and at bedtime) throughout the study and to wear their CGM constantly for next 20 days (changing the sensor at 10 days). CHO counting technique will be reviewed and appropriate support given with calculating bolus insulin doses.

Visit procedures:

Monitoring and treatment optimisation

- Check for adverse events
- Review blood glucose diary
- Blood ketones (from participant's own ketone meter
- Adjust insulin doses (if necessary)

Clinical assessments

- Blood pressure
- Heart rate
- Weight

Urine sample:

- Check urine ketones

Other

- *Start* Continuous Glucose Monitoring with Dexcom G6

7.3.2 Visit 2 (1 ≅ weeks prior to randomisation)

Fasting visit* - Participants will be asked to fast (no food or calorie intake) for 10 hours prior to the visit overnight. Those who plan to drive to fasting visits will be reminded of the increased risk of hypoglycaemia and DVLA guidelines. This will be repeated prior to each fasting visit and reiterated within the patient diaries. This visit will be scheduled for a morning appointment and participants will be advised not to take their breakfast bolus insulin dose prior to arrival. If participants arrive at the study visit in a non-fasted state, the visit will be terminated and a further fasting visit rearranged as soon as practicably possible.

Daily fingerstick blood glucose levels (diaries), CGM/ FGM data or insulin pump download data (as applicable) will be reviewed and appropriate insulin dose adjustments made. Clinical observations will be recorded as detailed below. Blood glucose and ketone bodies will be measured via a fingerstick blood sample (see Section 7). If blood glucose is less than 3.9 mmol/L, or more than 13.9 mmol/L, the visit will be rescheduled for an alternative date. Otherwise, an intravenous cannula will be placed in order to obtain blood samples for the duration of the visit. A Meal Challenge Test will be performed (described below). A 24 hr urine container will be dispensed to collect a 24 hour urine sample to bring in at the Week 0 visit. For this collection and all subsequent 24 hour urine collections, urine will be tested via dipstick testing and an aliquot retained for assay of albumin, creatinine, ketones, and 15-isoprostane.

Visit Procedures

<u>Monitoring and treatment optimisation</u>

- Check for adverse events
- Review blood glucose diary
- Blood ketones (from participant's own ketone meter
- Adjust insulin doses (if necessary)

<u>Clinical assessments</u>

- Blood pressure
- Heart rate
- Weight

<u>Urine sample</u>:

- Check urine ketones
- 24 hour urine container dispensed

<u>Other assessments</u>

- Download Dexcom G6 CGM
- High fat high calorie Meal Challenge: in order to assess postprandial metabolism a Meal Challenge Test will be performed using a 910 kilocalorie High Fat High Carbohydrate (HFHC) meal including an egg muffin sandwich, a sausage muffin sandwich and two hash browns [containing approximately 88g carbohydrate, 51 g fat (33% saturated) and 34 g protein]. Participants will be asked to attend fasting prior to breakfast insulin bolus (with continuing basal insulin); participants will be asked not to take bolus insulin until the 3 hour time point unless blood glucose values reach above 22.2 mmol/L. Blood samples will be collected from an indwelling intravenous canula in a superficial forearm vein for glucose, glucagon, FFA, GLP-1 (AUC). Sequential blood samples will be obtained at 0, 15, 30, 45, 60, 90, 120, 150 and 180 min after consuming the meal. Volume of samples at 15, 30, 45, 90 and 150 min will be 5ml while those

at 0, 60, 120 and 180min will be 30 ml (total volume = 145 ml). A spot urine sample will be collected at baseline, 1 and 3hr for standard urinalysis and an aliquot retained for assay of ketones and 15-isoprostane.

7.4 Randomisation

Participants who are being randomised will be required to have undergone all screening assessments.

At the randomisation visit, study participants will be provided with a Patient Alert Card. There are two Versions: (1) for the intervention arm i.e. those allocated at first randomisation to open-label semaglutide who are also eligible for the second double-blind randomisation (dapagliflozin or placebo); and (2) for those allocated to standard of care. These cards supplement the study diaries and include contact information for the study team. Version 1 contains unblinding information. Participants will be asked to carry their card at all times. These will be collected at the end of participation in the study.

7.4.3 First Randomisation Scheme

For the first randomisation (Period 1, open label), eligible and consenting participants will be randomised (2:1, stratified by site) to the two groups (open-label semaglutide or standard of care) using permuted blocks of variable size to minimize predictability.

7.4.4 Method of implementing the allocation sequence

Randomisation will be achieved by accessing an interactive web-based randomisation system (IWRS). The investigator will provide the participant identifier and the system will check the participant's eligibility from information already entered in the electronic Case Report Form (eCRF): if appropriate the randomisation group will be allocated.

7.5 Visit 3 (First Randomisation Visit)

Participants will be asked to attend in the fasting state (no food or calorie intake for 10 hours; see 7.3.2 above). Blood glucose levels and ketone bodies will be measured via a fingerstick blood sample using a glucose and ketone meter (see Section 7) and daily fingerstick blood glucose levels (diaries) collected and reviewed. The particpant's own CGM/ FGM data or insulin pump download data will also be reviewed. Daily insulin dose will be adjusted following clinical review of study diaries: if reduced, this should not be by more than 20% from the baseline level Participants will discontinue use of the study masked CGM (Dexcom G6). Clinical observations will be recorded. Blood glucose and ketone bodies will be measured via a fingerstick blood sample (see Section 7). The 24 hour urine container will be collected. A fasting blood sample will be taken (45 mL) to evaluate study endpoints, FBC, U&E, LFT, bone profile, lipids, HbA1c, and fructosamine (detailed below). A urine sample will be collected to evaluate ketone bodies and, in females, pregnancy status.

Participants will be asked to complete Quality of Life questionnaires (see Appendices).

IMP will be dispensed for those randomised to open-label semaglutide (once weekly). Semaglutide will be started on site at the 0.25 mg dose. Participants will be instructed to call site staff directly in case of any problem or untoward side effects. An office hours phone number will be provided for advice. Outside these hours, provision of emergency medical care if required will be via NHS 24 and/or Accident & Emergency. Participants will be specifically asked to call if they have hypoglycaemia (blood glucose < 3.9 mmol/L) or Level 2 hyperglycaemia (blood glucose >13.9 mmol/L), particularly if occurring on more than one occasion. This will apply for the remainder of the trial.

Visit Procedures

Fasting visit* as per visit 2.

Monitoring and treatment optimisation

- Check for adverse events
- Check adequacy of contraception (WOCBP)
- Review blood glucose diary
- Adjust insulin doses (if necessary)
- Blood ketones (from participant's own ketone meter)

Clinical assessments

- Blood pressure
- Heart rate
- Body weight

Quality of life assessments

- Diabetes Specific Quality of Life Scale (DSQOLS)
- Problem Areas In Diabetes (PAID) questionnaire

Blood assessments

- Na, K, urea, creatinine, eGFR (MDRD)
- AST/ ALT/ Alkaline Phosphatase
- Calcium/ Phosphate
- CRP
- HbA1c
- Fructosamine
- Haemoglobin
- White cell count
- Platelets
- Bilirubin
- Albumin
- Random glucose

Urine

- For WOCBP, a urine pregnancy test will be checked prior to first administration of IMP.
- Urine ketones
- 24 hour urine sample collected

Medication

- Administer open label semaglutide 0.25 mg if randomised to active treatment
- Issue Patient Alert Card (appropriate version according to randomisation)

Bloods for storage
Approximately 45 mls of venous blood will be withdrawn and collected in tubes containing EDTA. Blood will be centrifuged at 1500g for 20 mins at 4ºC. Plasma will be siphoned, aliquoted and stored locally at -80° ± 10°C until transport in batches to the central laboratory on dry ice.

7.5.1 Visit 4 (≅1 week after first randomisation)

Clinical observations will be recorded. Blood glucose levels and ketone bodies will be measured via a fingerstick blood sample using the participant's own glucose and ketone meter (see Section 7)) and daily blood glucose levels collected and reviewed. nsulin doses adjusted as in visit 3. If HbA1c is <7.0 %, insulin dose reduction should not be >5% from baseline total dose. Semaglutide 0.25mg will again be injected on site for those randomised to active therapy.

Visit Procedures

Monitoring and treatment optimisation

- Check for adverse events
- Review blood glucose diary
- Adjust insulin doses (if necessary)
- Blood ketones (from participant's own ketone meter

Assessments

- Blood pressure
- Heart rate
- Weight

Urine sample:

- Urine ketones

Medication

- Support participant's first self-injection of open label semaglutide 0.25 mg if randomised to active treatment

7.5.2 Visit 5 (≅2 weeks after first randomisation)

Clinical observations will be recorded. Blood glucose levels and ketone bodies will be measured via a fingerstick blood sample using glucose and ketones meter (see Section 7) and daily blood glucose levels collected and reviewed. Daily blood glucose data will be reviewed and insulin doses adjusted as per Visit 3. If participants randomised to semaglutide are experiencing any nausea, they will continue on the 0.25 mg dose for another week (or more as required) before increasing the dose. Otherwise semaglutide 0.5mg dose will be injected on site. Supply of IMP will be dispensed as appropriate to last until the next visit.

Visit Procedures

Monitoring and treatment optimisation

- Check for adverse events
- Review blood glucose diary
- Adjust insulin doses (if necessary)
- Blood ketones (from participant's own ketone meter)

Assessments

- Blood pressure
- Heart rate
- Weight

Urine sample:

- Urine ketones

Medication

- Dose titration (see above) if randomised to open-label semaglutide
- Return used study pen
- Dispense medication if randomised to open-label semaglutide

7.5.3 Visit 6 (≅4 weeks after first randomisation)

For those randomised to open-label semaglutide, doses administered will be recorded (self-report). Clinical observations will be recorded. Daily blood glucose data will be reviewed and insulin doses adjusted as per visit 3.. Supply of IMP will be given to last until the next visit. A urine sample will be collected to evaluate ketone bodies and, in females randomised to IMP, pregnancy status. Unless experiencing nausea, the recommended dose will be increased to 1.0 mg a day. A 24 hr urine container will be dispensed to collect a 24 hour urine sample to bring in at the Week 13 visit.

Visit Procedures

Monitoring and treatment optimisation

- Check for adverse events
- Check adequacy of contraception (WOCBP)
- Review blood glucose diary
- Adjust insulin doses (if necessary)
- Blood ketones (from participant's own ketone meter)

Assessments

- Blood pressure
- Heart rate
- Weight

Urine sample:

- Urine ketones
- Urine pregnancy test (WOCBP randomised to IMP only)
- 24 hour urine container dispensed

Medication

- Record doses of semaglutide self-injected since last visit (self-report)
- Dose titration if randomised to open-label semaglutide (see above)
- Return used study pens
- Dispense medication if randomised to open-label semaglutide

7.5.4 Visit 7 (≅13 weeks after first randomisation)

Participants will be asked to attend in the fasting state (no food or calorie intake for 10 hours). For those randomised to open-label semaglutide, doses administered will be recorded (self-report). Daily fingerstick blood glucose levels (diaries), CGM/ FGM data or insulin pump download data will be reviewed and appropriate insulin dose adjustments made. Participants will discontinue use of the study CGM (Dexcom G6). Supply of IMP will be given to last until visit 10. Observations will be recorded. A urine sample will be collected to evaluate ketone bodies and, in females randomised to IMP, pregnancy status. The 24 hour urine container will be collected. A fasting blood sample will be taken (45 mL) to evaluate study endpoints.

Visit Procedures

Fasting visit*

Monitoring and treatment optimisation

- Check adequacy of contraception (WOCBP)
- Check for adverse events
- Review blood glucose diary
- Adjust insulin doses (if necessary)
- Blood ketones (from participant's own ketone meter)

Clinical assessments

- Blood pressure
- Heart rate
- Weight

Blood assessments

- Na, K, urea, creatinine, eGFR (MDRD)
- AST/ ALT/ Alkaline Phosphatase
- Calcium/ Phosphate
- CRP
- HbA1c
- Fructosamine
- Haemoglobin
- White cell count
- Platelets
- Bilirubin
- Albumin
- Random glucose

Urine

- Pregnancy test (WOCBP randomised to IMP only)
- Urine ketones
- 24 hour urine bottle collected

Medication

- Record doses of semaglutide self-injected since last visit (self-report)
- Return used study pens
- Dispense medication if randomised to open-label semaglutide

7.5.5 Visit 8 (≅23 weeks after first randomisation)

For those randomised to open-label semaglutide, doses administered will be recorded (self-report). Clinical observations will be recorded. Blood glucose levels and ketone bodies will be measured via a fingerstick blood sample. A urine sample will be collected to evaluate ketone bodies and, in females randomised to IMP, pregnancy status. Insulin dose will be adjusted if needed. All subjects will be reminded to monitor their capillary blood glucose by fingerstick at least 4 times a day (before meals and at bedtime) throughout the study. CGM sensor will be fitted and participants asked to wear their CGM constantly for next 20 days.

Visit Procedures

Monitoring and treatment optimisation
- Check adequacy of contraception (WOCBP)
- Check for adverse events
- Review blood glucose diary
- Blood ketones (from participant's own ketone meter)
- Adjust insulin doses (if necessary)

Clinical assessments
- Blood pressure
- Heart rate
- Weight

Urine sample:
- Pregnancy test (WOCBP randomised to IMP only)
- Urine ketones

Other assessments
- *Start* Continuous Glucose Monitoring with Dexcom G6

7.5.6 Visit 9 (≅25 weeks after first randomisation)

Participants will be asked to attend in the fasting state (no food or calorie intake for 10 hours). Daily fingerstick blood glucose levels (diaries), CGM/ FGM data or insulin pump download data will be reviewed and appropriate insulin dose adjustments made. Participants will discontinue use of the study CGM (Dexcom G6). Clinical observations will be recorded. Blood glucose levels and ketone bodies will be measured via a fingerstick blood sample (see Section 7) Supply of IMP will be given to last until the next visit. A 24 hr urine container will be dispensed to collect a 24 hour urine sample to bring in at visit 10 (39pprox.. Week 26) visit. A Meal Challenge Test will be performed as per visit 2. Participants will be provided with strips for urine and blood ketone measurement at home and instructed or reminded as appropriate on how to use these. They will be asked to measure urinary and blood ketones every morning for three consecutive days prior to the next visit (second randomisation: dapagliflozin or placebo) and to record the results in their diary.

Visit Procedures

Fasting visit*

Monitoring and treatment optimisation
- Check adequacy of contraception (WOCBP)
- Check for adverse events
- Review blood glucose diary
- Adjust insulin doses (if necessary) - **this insulin dose is baseline for Period 2**
- Blood ketones (from participant's own ketone meter)

Clinical assessments
- Blood pressure
- Heart rate
- Weight

Urine sample:
- Urine ketones
- Pregnancy test (WOCBP randomised to IMP only)
- 24 hour urine container dispensed

Other assessments
- Download Dexcom G6
- Meal Challenge Test

7.6 Second Randomisation Scheme

For the second randomisation (Period 2, double-blind), eligible and consenting participants previously randomized to open-label semaglutide will be randomised (2:1) to two groups (dapagliflozin or matching placebo) using permuted blocks of variable size to minimize predictability with stratification for HbA1c (above vs below 7.0 %). Those participants previously randomised to standard of care will continue as before.

7.6.1 Blinding (period 2 only)

For the double-blind treatment period, dapagliflozin and placebo will be packed in matching packs to maintain the blind. Each pack will contain sufficient supplies for 30 days' treatment with a small overage (excess). Packs will be labelled with a unique number that will be used to assign treatment to the participant via the IWRS system whilst maintaining the blind. Packs will be labelled in accordance with Good Manufacturing Practice and local regulatory requirements.

Unblinding. Treatment discontinuation (of dapagliflozin or placebo), rather than unblinding, will be carried out as far as possible. Participants will be advised in any case of hospitalisation with acute illness to discontinue study medication and inform the relevant clinician. These instructions will be reinforced in Study Diary Version 1. However, where knowledge of treatment may assist emergency treatment, unblinding will be supported. Information on how to unblind in an emergency (the investigational study drug, the study number, the investigator's name, a contact number, and a freephone number) will be provided on the Patient Alert Card. Several prompts on the telephone menu system warn the user that they require to be a health professional and to record their name and other pertinent information. For each unblinding an email alert is generated to the Study Coordinator and Chief Investigator. The most likely scenarios for unblinding will be: confirmed pregnancy, overdose/ accidental ingestion, or development of acute renal failure.

7.6.2 Visit 10 (Second Randomisation Visit; ≅26 weeks after First Randomisation)

Participants will be asked to attend in the fasting state (no food or calorie intake for 10 hours). Participants with normal fasting urine (<+) or blood ketone (<0.6 mmol) in the three days prior to randomisation will proceed to the next treatment phase as per this visit. The principles of the STICH Protocol (see Appendix 3) will be discussed. Participants with higher ketone values in at least one of the previous three days will have their glucose levels, insulin dose, and carbohydrate intake reviewed and advised on hydration, adequate CHO intake, and adequate insulin dosing. They will be rescheduled for one week and asked to repeat three days testing before the next visit.

Clinical observations will be recorded. A urine sample will be collected for ketones. Blood glucose and ketones will be measured via a fingerstick blood sample (see Section 7.2.1). Daily fingerstick blood

glucose levels (diaries), CGM/ FGM data or insulin pump download data will be reviewed and appropriate insulin dose adjustments made (following the guidance in Section 7, page 35). The previous three days blood and urine ketones levels will be reviewed. The adjusted total insulin dose at this visit will serve as the baseline total insulin dose for the remaining six months of the study and future limits of dose adjustments will be based on this new baseline. The blinded study CGM will be collected. The 24 hour urine container will be collected. A fasting blood sample will be taken (45 mL) to evaluate study endpoints. Participants will be asked to complete quality of life questionnaires.

At this point, those participants previously randomised to receive open-label semaglutide will be randomised to receive dapagliflozin 5 mg daily or matching placebo. They will be educated on symptoms suggestive of low blood pressure (e.g. light-headedness, dizziness, feeling faint).

Participants will be asked to perform a daily (preferably early morning) urine (using CombiScreen® 10 SYS Plus urine testing strips or equivalent) and blood tests for ketone bodies (using the particpant's own meters as per Section 7, "Minimising ketosis": page 28) and record all outcomes in dedicated study diaries. If results of these scheduled (i.e. not done due to symptoms) ketone measurements, are +1 or higher (urine) or > 0.6 mmol/L (blood), participants will be instructed to repeat the measurement after 60 min. Participants will be instructed to call site staff directly as soon as possible within office hours if these results are persistent, . For any abnormal ketone levels taken due to symptoms, the DKA consensus guidelines are to be followed instead (see Section 9.3).

Participants will be instructed to call site staff directly in case of any problem or untoward side effects. They will be specifically asked to call if they have confirmed hypoglycaemia (blood glucose levels < 3.9 mmol/L) or hyperglycaemia (blood glucose > 13.9 mmol/L) occurring on more than one occasion. Participants will be advised to remain well hydrated and report any genital mycotic or urinary tract infections

Visit Procedures

Fasting visit* - Requirements and arrangements as per visits 2 and 3.

Monitoring and treatment optimisation

- Check adequacy of contraception (WOCBP)
- Check for adverse events
- Review blood glucose diary
- Adjust insulin doses (if necessary)
- Blood ketones (from participant's own ketone meter)

Clinical assessments
- Blood pressure
- Heart rate
- Body weight

Quality of life assessments
- Diabetes Specific Quality of Life Scale (DSQOLS)
- Problem Areas In Diabetes (PAID) questionnaire

Blood assessments
- Na, K, urea, creatinine, eGFR (MDRD)

- AST/ ALT/ Alkaline Phosphatase
- Calcium/ Phosphate
- CRP
- HbA1c
- Fructosamine
- Haemoglobin
- White cell count
- Platelets
- Bilirubin
- Albumin
- Random glucose

Urine
- Pregnancy test (WOCBP randomised to IMP only)
- Urine ketones
- 24 hour urine bottle collected

Medication
- Education on symptoms suggestive of low blood pressure (e.g. light-headedness, dizziness, feeling faint) for those undergoing second randomisation
- Record doses of semaglutide self-injected since Visit 7 (self-report)
- Return used study pens
- Dispense open label semaglutide for participants randomised to active treatment at Period 1 (if required).
- Dispense dapagliflozin or placebo if randomised to active treatment

7.6.3 Visit 11 (≅1 week after Second Randomisation)

For those randomised to open-label semaglutide, doses administered will be recorded (self-report). Clinical observations will be recorded. Daily blood glucose levels and urinary ketone measurements will be reviewed. A urine sample will be collected to evaluate for ketones bodies. Blood glucose levels and ketones bodies will be reviewed measured via a fingerstick blood sample (see Section 7.2.1). Blood glucose levels and ketone bodies will be measured via a fingerstick blood sample using glucose and ketones meter. A urine sample will be collected to evaluate urine ketone level. Daily fingerstick blood glucose levels (diaries), CGM/ FGM data or insulin pump download data will be reviewed and appropriate insulin dose adjustments made using the new baseline from Visit 10 (following the guidance in Section 7, page 35). If at any time total insulin dose reduction is >20% and total insulin dose is <0.40 U/Kg, additional monitoring (see Appendix 2) will be implemented. Unless blood (>0.6 mmol/L) or urine ketones (+ or more) have been elevated within the previous three days, dapagliflozin or placebo dose will be uptitrated to 10 mg daily (two capsules of either active or placebo medication) for the remainder of the study. Supply of IMPs will be given to last until week 39.

Visit Procedures

Monitoring and treatment optimisation
- Check adequacy of contraception.
- Check for adverse events
- Review blood glucose diary
- Adjust insulin doses (if necessary)
- Blood ketones (from participant’s own ketone meter)
- Daily urine ketones review

Urine sample:

- Urine ketones
- Pregnancy test (WOCBP randomised to IMP only)
- Collect 24 hour urine container

Medication

- Count returned dapagliflozin or placebo capsules
- Increase dose of dapagliflozin or placebo to 10mg (see above – review ketones)
- Dispense dapagliflozin or placebo

7.6.4 Visit 12 (≅5 weeks after Second Randomisation)

For those randomised to open-label semaglutide, doses administered will be recorded (self-report). Clinical observations will be recorded. Daily blood glucose levels and urinary ketones measurements will be collected and reviewed. Blood glucose levels and ketone bodies will be measured via a fingerstick blood sample. A urine sample will be collected to evaluate ketone bodies and if appropriate pregnancy status. Insulin dose will be adjusted if as per Visit 11. A blood sample will be taken (15 mL) to evaluate study endpoints. A 24 hr urine container will be dispensed to collect a 24 hour urine sample to bring in at the Week 39 visit

Visit Procedures

Monitoring and treatment optimisation

- Check adequacy of contraception (WOCBP)
- Check for adverse events
- Review blood glucose diary
- Adjust insulin doses (if necessary)
- Blood ketones (from participant's own ketone meter)

Clinical assessments

- Blood pressure
- Heart rate
- Weight

Blood assessments

- Na, K, urea, creatinine, eGFR (MDRD)
- AST/ ALT/ Alkaline Phosphatase
- Calcium/ Phosphate
- CRP
- Haemoglobin
- White cell count
- Platelets
- Bilirubin
- Albumin
- Random glucose

Urine

- Pregnancy test (WOCBP randomised to IMP only)
- Urine ketones
- 24 hour urine container dispensed

7.6.5 Visit 13 (≅13 weeks after Second Randomisation)

For those randomised to open-label semaglutide, doses administered will be recorded (self-report). Participants will be asked to attend in the fasting state. Clinical observations will be recorded. Daily blood glucose levels and urinary ketones measurements will be collected and reviewed. Blood glucose levels and ketone bodies will be measured via a fingerstick blood sample. A urine sample will be collected to evaluate ketone bodies and if appropriate pregnancy status. The 24 hour urine container will be collected. Insulin dose will be adjusted if needed as in as per Visit 11.. Sufficient supply of IMPs will be given until the end of the study. A fasting blood sample will be taken (45 mL) to evaluate study endpoints.

Visit Procedures

Fasting visit* - as per previous fasting visits

Monitoring and treatment optimisation

- Check adequacy of contraception (WOCBP)
- Check for adverse events
- Review blood glucose diary
- Adjust insulin doses (if necessary)
- Blood ketones (from participant's own ketone meter)
- Daily urine ketones review

Clinical assessments

- Blood pressure
- Heart rate
- Weight

Quality of life assessments

- Diabetes Specific Quality of Life Scale (DSQOLS)
- Problem Areas In Diabetes (PAID) questionnaire

Blood assessments

- Na, K, urea, creatinine, eGFR (MDRD)
- AST/ ALT/ Alkaline Phosphatase
- Calcium/ Phosphate
- CRP
- HbA1c
- Fructosamine
- Haemoglobin
- White cell count
- Platelets
- Bilirubin

- Albumin
- Random glucose

Urine

- Pregnancy test (WOCBP randomised to IMP only)
- Urine ketones
- 24 hour urine bottle collected

Medication

- Record doses of semaglutide administered since Visit 10 (self-report)
- Dispense open label semaglutide for participants randomised to active treatment at Period 1 (if required).
- Count returned dapagliflozin or placebo capsules
- Dispense dapagliflozin or placebo as required

7.6.6 Visit 14 (≅23 weeks after Second Randomisation)

For those randomised to open-label semaglutide, doses administered will be recorded (self-report). Clinical observations will be recorded. Daily blood glucose levels and urinary ketones measurements will be collected and reviewed. Blood glucose levels and ketone bodies will be measured via a fingerstick blood sample. A urine sample will be collected to evaluate ketone bodies and if appropriate pregnancy status. Insulin dose will be adjusted if as per Visit 11. All subjects will be reminded to monitor their capillary blood glucose by fingerstick at least four times a day (before meals and at bedtime). CGM (Dexcom G6) sensor will be fitted and patients asked to wear their CGM constantly for the next 20 days.

Visit Procedures

Monitoring and treatment optimisation

- Check adequacy of contraception (WOCBP)
- Check for adverse events
- Review blood glucose diary
- Adjust insulin doses (if necessary)
- Blood ketones (from participant's own ketone meter)
- Daily urine ketones review

Clinical assessments

- Blood pressure
- Heart rate
- Weight

Urine sample:

- Pregnancy test (WOCBP randomised to IMP only)
- Urine ketones

Clinical and functional assessment

- *Start* Continuous Glucose Monitoring sensor (mean fasting blood glucose, mean blood glucose, percentage time in range, glycaemic variability)

7.6.7 Visit 15 (≅25 weeks after Second Randomisation)

Participants will be asked to attend in the fasting state. For those randomised to open-label semaglutide, doses administered will be recorded (self-report). Observations will be taken. Daily blood and urinary ketones measurements will be collected and reviewed. Blood glucose levels and ketone bodies will be measured via a fingerstick blood sample. A urine sample will be collected to evaluate urine ketone levels. Insulin dose will be adjusted if needed as per visit 11. A 24 hr urine container will be dispensed to collect a 24 hour urine sample to bring in at the Week 52 visit. A HFHC Meal Challenge Test will be performed as in Visit 2.

Visit Procedures

Fasting visit* - Procedure as per previous fasting visits

Monitoring and treatment optimisation
- Check adequacy of contraception (WOCBP)
- Check for adverse events
- Review blood glucose diary
- Adjust insulin doses (if necessary)
- Daily urine ketones review
- Blood ketones (from participant's own ketone meter)

Assessments
- Blood pressure
- Heart rate
- Weight

Urine sample:
- Pregnancy test (WOCBP randomised to IMP only)
- 24 hour urine container dispensed

Other assessments
- *Read* Continuous Glucose Monitoring sensor (mean fasting blood glucose, mean blood glucose, percentage time in range, glycaemic variability)
- Meal Challenge Test

7.6.8 Visit 16 (End of study; ≅26 weeks after Second Randomisation)

Participants will be asked to attend in the fasting state. Clinical observations will be recorded. Daily blood glucose levels and urinary ketones measurements will be collected and reviewed. Blood glucose levels and ketone bodies will be measured via a fingerstick blood sample. Insulin doses will be reviewed and likely requirement for an increase following discontinuation of IMP discussed with those taking open-label semaglutide. A urine sample will be collected to evaluate ketone bodies and, if appropriate pregnancy status. The CGM will be collected. The 24 hour urine container will be collected. A fasting blood sample will be taken (45 mL) to evaluate study endpoints. We will ask the participants to complete quality of life questionnaires. A physical examination will be performed as per the screening visit. This will be the final face-to-face study visit. A safety phone call will be conducted two weeks to review blood

glucose data, provide further insulin dosing advice, to document any adverse events, and to ensure that follow-up in usual clinical care has been arranged.

<u>Visit Procedures</u>

Fasting visit*

<u>Assessments</u>
- Blood pressure
- Heart rate
- Weight

<u>Monitoring and treatment optimisation</u>
- Check for adverse events
- Review blood glucose diary
- Adjust insulin doses (if necessary)
- Blood ketones (from participant's own ketone meter)
- Daily urine ketones review

<u>Clinical assessments</u>
- Blood pressure
- Weight
- Heart rate
- Physical examination

<u>Quality of life assessments</u>
- Diabetes Specific Quality of Life Scale (DSQOLS)
- Problem Areas In Diabetes (PAID) questionnaire

<u>Blood assessments</u>
- Na, K, urea, creatinine, eGFR (MDRD)
- AST/ ALT/ Alkaline Phosphatase
- Calcium/ Phosphate
- CRP
- HbA1c
- Fructosamine
- Haemoglobin
- White cell count
- Platelets
- Bilirubin
- Albumin
- Random glucose

<u>Urine</u>
- Pregnancy test (WOCBP randomised to IMP only)
- Urine ketones
- 24 hour collection

Other

- Record doses of semaglutide administered since Visit 13 (self-report)
- Return used study pens
- Count returned dapagliflozin or placebo capsules
- Collect Patient Alert Card

7.7 Retention and strategies for maximizing follow-up

Participants will be encouraged to attend all study visits. Prior to commencing the trial, all potential participants will have the opportunity to read the detailed Patient Information Sheet which will outline the level of participation requested. Potential participants will be made aware of the need for frequent study visits and asked to commit to these.

Study visits can be rearranged flexibly within a period around the planned visit as specified in the timeline of visits above (Section 7) and actual dates recorded in the eCRF. This is to maximise participant ability to attend all visits. A longer or shorter interval does not constitute a protocol deviation. Where possible, visits will be arranged to coincide with other appointments to further maximise attendance and participant convenience. Telephone contact will be frequent and will help to maintain contact with participants and ensure timely reminders of upcoming study visits can be given.

Even in the scenario of treatment discontinuation (of either IMP), participants will be encouraged to continue to attend further study visits as scheduled.

7.8 Treatment Interruptions and Withdrawal criteria

Withdrawal from study drug

Treatment interruption from study IMPs may be necessary in some participants. In the case of dapagliflozin, this will occur in any acute illness or hospitalisation which increases the risk of ketosis and ketoacidosis. Circumstances in which temporary discontinuation of dapagliflozin or placebo should occur will be discussed with all study participants. Written information will be provided in the study diaries. An emergency phone line to the study team will be available to all participants for the duration of their participation in the trial. This will be operational during office hours (Monday to Friday 9am – 5pm). Patient Alert Card Version 1 will also contain unblinding information in the event of acute intercurrent illness and a requirement for unblinding in the opinion of the acute medical team treating the participant. If any participant develops diabetic ketoacidosis (DKA) as defined as per the International Consensus guidelines on two occasions with no identifiable precipitant (section 2.1) they will be withdrawn from study medication and the UK Chief and US Principal Investigators informed.

Any other intolerable side effects or acute illness may require temporary discontinuation of the study drugs (dapagliflozin or semaglutide). In these circumstances, if the study drugs cannot be safely restarted in the judgement of the trial physician or if participants are unable to tolerate side-effects, they may be withdrawn from study treatment.

Treatment withdrawal may be necessary during the trial prior to the planned end at 52 weeks following first randomisation for any of the following reasons:

1. The site PI can stop investigational study treatment or remove participants from the trial without participants approval if for any reason he/she feels is appropriate, including: severe side effects, injury or medical condition which may place the patient at risk of further complications if he/she continue to participate, failure to take the medication as instructed, failure to keep scheduled appointments, insufficient level of self-monitoring, cancellation of the study or other administrative reasons.

2. Any woman who becomes pregnant during the period of study will be withdrawn from study treatment.

3. Participants who experience persistent severe nausea or vomiting after starting semaglutide which is not managed by dose reduction will discontinue the investigational study treatment. Those with mild or moderate nausea will continue for at least one more week. For those who continue to experience significant nausea (defined as interfering with normal activities), dose will be reduced: if these side effects persist, they will discontinue study medication(s). Participants for whom this occurs in Period 1 will remain eligible for randomisation in Period 2.

4. New diagnosis of other conditions included in the Exclusion Criteria

5. Change in method of insulin delivery between insulin pumps to MDI or vice versa during the study unless pump treatment interruption is temporary for a period of at most 2 weeks. Participants who upgrade to “artificial pancreas” (e.g. Medtronic’s MiniMed 670G hybrid) during the study will be withdrawn.

6. Hypoglycaemia: Two episodes of severe hypoglycaemia (requiring external intervention).

7. Ketosis (with no other identifiable precipitating factors): Persistent (1 week) asymptomatic or symptomatic and unmanaged (using STICH protocol – see section 2.1 b) blood ketone levels >1.5 mmol (or ≥+2 in urine).

8. Persistent (>1 week) total insulin dose reduction of >20% from baseline AND total insulin dose <0.4U/Kg.

9. DKA: Two episode of DKA (with no identifiable precipitating factors) as defined by the expert consensus statement (34)

10. Two episodes of UTI.

11. Two episodes of mycotic genital infection.

12. Significant persistent reduction in eGFR > 15 ml/min

13. Pancreatitis: any episode judged to be due to pancreatitis by the site PI.

14. Other persistent side effects specific to study drugs

Participation in this study is voluntary. Participants have the right to refuse to participate or to withdraw from participation at any time for any reason. The site PI can stop investigational study treatment or remove participants from the trial without participants approval. If a patient withdraws consent and decide not to participate in the study, she/he will be asked to undergo a final study visit for his/her safety.

If a subject withdraws from the research, the data collected to that point will be used toward the research finding. If applicable the subject will be asked to return any unused research drug and or devices.

Withdrawal from the study

If participants are unable or unwilling to attend all study visits they will be given an option of attending less frequently or only at the end of the study. We will request participants' consent to be contacted by telephone and for contact with their general practitioner or other health care provider to check on their current health status. No participant will be labelled as lost to follow-up. However, participants will have the right to withdraw consent for further participation and for further data collection: all other participants will be followed up for clinical events until study completion.

7.9 Storage and analysis of samples

Blood samples will be undertaken at the beginning and at various points during the study as outlined in detail in the schedule of visits. If blood testing has recently been undertaken in the 3 weeks prior to the study commencing, some of these blood tests may be used as baseline. Blood tests will include analysis of the full blood count, renal function (urea and electrolytes), liver function tests, bone profile, lipid profile and several specialised blood tests for biomarker assays (at central laboratory) following a high fat high caloric meal challenges to examine study endpoints.

7.9.1 Sample collection and processing

- Samples will be appropriately labelled in accordance with the trial procedures to comply with the General Data Protection Regulation (GDPR). Biological samples collected from participants as part of this trial will be transported, stored, accessed and processed in accordance with national legislation relating to the use and storage of human tissue for research purposes and such activities shall at least meet the requirements as set out in the 2004 Human Tissue Act and the 2006 Human Tissue (Scotland) Act.
- Blood will be taken according to the Schedule of Assessments (page 17). Plasma will be siphoned, aliquoted and stored locally at -80° ± 10°C until transport in batches to the central laboratory on dry ice. according to the Sample Handling Procedure as agreed by the Joint Study Management Group
- Individual patient samples will be identified with a unique, anonymised study number.

7.9.2 Sample transport to central laboratory and analysis

- Samples will be transferred to State University of New York in a single batch at the end of recruitment to the study. More frequent transfer can be organised if there are local storage limitations.
- Samples will be transported on dry ice and stored at the central laboratory at -80° ± 10° until analysis. Transport by courier will coordinated by the Trial Manager.
- At the time of analysis individual aliquots will be defrosted at room temperature and analysed in a single batch for each biomarker of interest.
- Samples will be stored at -80° ± 10° in the central laboratory for possible future analysis for novel biomarkers.

7.10 End of trial

At the end of the specified trial period (52 weeks following first randomisation) all participants will discontinue study medications and revert to standard of care (insulin therapy only). This process will be supervised by the trial physicians, research nurses, and research pharmacists under the supervision of the site Principal Investigator. The trial team will liaise with the usual care team to ensure a safe transition back to standard therapy. Following the final scheduled trial visit there will be a safety phone call two weeks after the last visit. This will act as a further opportunity to advise on insulin dosing and ensure there are no ongoing problems or concerns related to the study.

As discussed in section 7.8, there may be circumstances in which trial drugs need to be stopped before the specified end of the trial. If this is the case, the reason(s) for discontinuation will be recorded and the trial clinical team will facilitate safe transition back to usual standard of care. Participants will however if eligible (i.e non-exclusion criteria reason for IMP discontinuation) be asked to continue with trial visits to enable ongoing close clinical observation. If consent is withdrawn and participants voluntarily chose to leave the trial before the specified end date, all efforts by the clinical trial team will be made to safely facilitate transition back to ordinary standard of care. A follow up safety visit will be offered to all such participants and ongoing contact details will be made available should there be any further unforeseen issues relating to the clinical trial.

The trial may be stopped at an earlier stage than anticipated on the advice of the independent data monitoring committee (IDMC) to the trial steering committee (TSC) for the safety reasons detailed on page 12 and in the study safety endpoints. If this occurs, all participants will be informed and safe discontinuation of study medications will be undertaken under the supervision of the site Principal Investigator.

8 TRIAL MEDICATION

8.1 Name and description of investigational medicinal product(s)

a) Semaglutide (Ozempic®): a once weekly GLP-1 agonist injection indicated for type 2 diabetes (approved by the Scottish Medicines Consortium, January 2019).

Doses to be used: 0.25mg increased to 0.5mg at two weeks and then to 1mg weekly s.c. injection after a further two weeks. Open label semaglutide will be added to a background of insulin treatment at week 0 for 12 months in 76 participants (38 in the UK) according to the first randomisation; at week 26 they will be further randomised (double-blind) as below into two groups: dual (placebo, semaglutide and insulin) and triple (dapagliflozin, semaglutide and insulin) therapy.

b) Dapagliflozin (as dapagliflozin propanediol monohydrate Forxiga®): a once daily SGLT-2 inhibitor that will be administered orally in a blinded fashion.

Doses to be used: 5 mg daily increased after one week to 10 mg daily (orally). Dapagliflozin will be started according to the randomisation (Interactive Web System, hosted by University of Glasgow) in 38 participants (19 in the UK) at 26 weeks as an additional treatment on a background of insulin and semaglutide therapy and end at 52 weeks (triple therapy group). Doses to be used: 5 mg increased to 10 mg after one week.

c) Placebo to dapagliflozin: Dapagliflozin matched placebo will be started in 38 participants (19 in the UK) participants at 26 weeks as an additional treatment on a background of insulin and semaglutide therapy and end at 52 weeks (dual therapy group). Doses to be used: as for active dapagliflozin.

8.2 Legal status of Semaglutide and Dapagliflozin

Semaglutide is currently licensed in the UK for those with adult type 2 diabetes which is insufficiently controlled.

Dapagliflozin is currently licensed in the UK for insufficiently controlled type 1 and type 2 diabetes.

8.3 Drug storage and supply

Semaglutide and dapagliflozin/placebo supplies must be stored in the original containers in a locked, secure area with access limited to authorised site staff. Study supplies should be used as directed in the study protocol and not be supplied to any persons other than study participants. All IMPs will be dispensed to participants from the study site pharmacy.

Semaglutide: Supplies of semaglutide pre-filled pens for subcutaneous administration will be sourced from routine hospital stocks. The following semaglutide presentations will be required:

- Semaglutide 0.25 mg: Each pre-filled pen contains 2mg semaglutide in 1.5ml. Each dose contains 0.25mg of semaglutide in 0.19ml solution.
- Semaglutide 0.5 mg: Each pre-filled pen contains 2mg semaglutide in 1.5ml solution. Each dose contains 0.5 mg of semaglutide in 0.37ml solution.

- Semaglutide 1 mg: Each pre-filled pen contains 4 mg semaglutide in 3.0ml solution. Each dose contains 1mg semaglutide in 0.74ml solution.

For all presentations each pre-filled pen is capable of delivering 4 doses which is sufficient for 4 weeks treatment. Pens are for individual participant use only. In the event of a pen-failure, a new pen should be supplied to the participant.

Prior to first use, semaglutide pens must be stored at 2-8 °C in the original container and must not be frozen. Supplies which are frozen either prior to first use or during use must be discard.

Semaglutide pre-filled pens have an in-use shelf life of 6 weeks. After first use the pre-filled pens may be stored below 30oC or in a refrigerator at 2-8 °C. Participants should be advised to store packs away from the cooling element. The pen cap should be on when the pen is not in use in order to protect the contents from light.

Wherever possible, supplies of semaglutide pre-filled pens should be utilised in a pragmatic way so as to minimise unnecessary wastage. Participants should be provided with an appropriate number of pen(s) to last until the next planned visit. A small overage may be given as necessary to accommodate visit windows etc. The correct strength of pen must be supplied for each dose titration i.e. 0.25mg, 0.5mg and 1.0 mg as tolerated.

A web-based system will be used to randomise participants to semaglutide treatment. At randomisation and all subsequent visits (planned or unplanned), the system will notify pharmacy staff at study visits of the requirement for semaglutide supplies.

Dapagliflozin 5mg or placebo capsules: Supplies of dapagliflozin 5mg and matched placebo capsules will be manufactured by Tayside Pharmaceuticals in accordance with Good Manufacturing Practice requirements. Capsules will be packed into HDPE bottles with a tamper evident seal. Each bottle will be labelled in a double-blind fashion to meet local regulatory requirements and will be assigned a unique number. Bottles containing dapagliflozin 5 mg or placebo must be stored at room temperature at 15-25°C.

Dapagliflozin 5mg and placebo capsule supplies will be provided to study site and will only be released by the sponsor once all the appropriate regulatory and governance approvals are in place. Further information on storage requirements and supply arrangements is provided in the study specific IMP Management and Accountability Manual.

A web-based system will be used for the assignment of dapagliflozin 5mg or placebo bottles at randomisation and at study visits where there is planned dispensing of the IMP or in the event that a participant requires replacement medicines.

Other medicines: Supplies of all other medicines and associated consumables will via usual NHS supply mechanisms.

8.4 Drug accountability requirements

The Investigator or designee must maintain accurate records of all study IMP movements for accountability purposes. They should include dates, quantities, batch numbers and expiry. Records must document adequately that:

- the patients were provided the doses specified by the protocol/amendment(s)
- all study drug provided was fully reconciled.

Unused study drug must not be discarded or used for any purpose other than the present study. Further information is provided in the study specific IMP Management and Accountability Manual.

8.5 Preparation and administration of semaglutide and dapagliflozin

Semaglutide (Ozempic®) is a solution for subcutaneous injection. It is administered once weekly, on the same day each week, into the subcutaneous tissues of the abdomen, thigh or upper arm. A participant may change the day of their weekly injection as long as it has been at least three days since their last injection. Thereafter, all doses should be administered once weekly on the new dosing day. The injection can be administered at any time of the day regardless of meals.

Participants must be advised to discard the injection needle after each injection and store the pen without an injection needle attached. This may help prevent blocked needles, contamination, infection, leakage of solution and inaccurate dosing. Semaglutide should not be used if the solution does not appear clear and colourless or almost colourless. The pen is designed to be used with NovoFine or NovoTwist disposable needles and pens are packed with an appropriate quantity of NovoFine Plus needles.

Detailed information on administration of semaglutide is provided in the current Summary of Product Characteristics and product Patient Information Leaflet.

Dapagliflozin (Forxiga®)) is presented as a capsule for once daily oral administration.

8.6 Dosage schedules

Semaglutide will be administered and dosed as described. Participants will be commenced on 0.25 mg s.c once weekly before up-titration as tolerated to 1.0 mg s.c once weekly. Up-titration is quicker than is ordinarily the case in clinical practice in type 2 diabetes as discussed in Section 2.1 (22) There will be increased observation and clinical access for participants in the event of side-effects. Participants randomised to receive semaglutide will continue on the 1.0 mg s.c once weekly dose for the remainder of the study following uptitration provided they tolerate the dose as detailed in Section 7. If necessary, the dose may be adjusted.

If a participant misses a dose of semaglutide it should be administered as soon as possible and within 5 days after the missed dose. If more than 5 days have passed, the missed dose should be skipped, and the next dose should be administered on the regularly scheduled day. In both cases participants, should then resume their regular once weekly dosing schedule.

Dapagliflozin will be commenced at 5mg orally once daily and uptitrated one week after commencement to a dose of 10mg which will thereafter be continued for the remainder of the trial provided they tolerate the dose. If necessary, the dose may be adjusted.

8.7 Dosage modifications

Dapagliflozin/placebo will be commenced at one capsule (5mg or placebo) orally once daily and uptitrated one week after commencement to a dose of two capsules (10mg or placebo) which will thereafter be continued for the remainder of the trial provided they tolerate the dose. If necessary, the dose may be adjusted. Dapagliflozin or placebo capsules can be taken at any time of day with or without food. Capsules should be swallowed whole. If participants forget to take a dose they should be advised as follows:

- If it is 12 hours or more until the next dose, the dose should be taken as soon as remembered and the next dose taken at the usual time.
- If it is less than 12 hours until the next dose, the missed dose should be skipped. The next dose should be taken at the usual time.
- Participants should not take a double dose of dapagliflozin/placebo to make up for a forgotten dose.

8.8 Known drug reactions and interaction with other therapies

Participants with hypersensitivity to either active study medication are excluded from the trial. There are no significant interactions between semaglutide and other prescribed medications other than with agents that can lower blood glucose levels. Participants on any other oral agents which can lower blood glucose levels meet Exclusion Criteria and will not be included in this trial. In combination with insulin and dapagliflozin, semaglutide may be associated with an increased risk of hypoglycaemia. Participants will be advised of the increased risk of hypoglycaemia and will be required to closely monitor their blood glucose levels for the duration of the trial. No interactions are currently listed between warfarin and semaglutide in the British National Formulary but in view of a theoretical interaction (based on protein binding), existing arrangements for INR testing will be reviewed for any participants prescribed warfarin and additional tests conducted if indicated according to the judgement of the investigators.

Dapagliflozin is known to increase the risk of hypoglycaemia when combined with other medications which also act to lower blood glucose levels. Dapagliflozin has a mild diuretic effect and therefore there is an increased risk of hypotension when dapagliflozin is combined with either medications which deplete circulating volume (e.g diuretics) or antihypertensive drugs. Dapagliflozin is however commonly prescribed with such medications in general medical practice. All participants will have their medications reviewed at the screening visit and educated regarding symptoms that may indicate dehydration/ hypovolaemia. If there is any concern of interaction with any of the IMPs, this will be discussed with the PI before a decision regarding their ability to participate is made.

8.9 Concomitant medication

Participants should not receive any adjunct glucose-lowering therapy other than as per the randomisation schedule (e.g. with either of the active study medications).

8.10 Trial restrictions

Contraindications will follow SmPC information for Ozempic® and Forxiga. Acceptable forms of contraception for WOCBP participating in the trial are listed in Appendix 2. There is no requirement for contraception for male participants.

8.11 Assessment of compliance

Treatment adherence will be assessed by the trial medical and nursing team when participants attend for scheduled study visits.

8.12 Name and description of each Non-Investigational Medicinal Product (NIMP)

Insulin therapy is a non-investigational medicinal product in this trial. The trial is in a population who have had a diagnosis of type 1 diabetes for at least one year: therefore all participants will already be established on insulin therapy. The regimen and dosage required will by differ between participants. Participants will be using a combination of long acting (basal) insulin and shorter acting (bolus) insulin or CSII (insulin pump). As in standard of care, dose titration will depend on diet, blood glucose levels and exercise. It is anticipated that dosage adjustments will be required to achieve optimal glycaemic control and maintain participant safety.

There are pre-defined guidelines for insulin titration in this study. Insulin dose reductions should not be greater than 10% (5% if HbA1c is<7%) from current total insulin dose or more than 20% from the baseline level at randomisation and then from the new baseline levels at 26 weeks following second randomisation. Participants requiring an insulin dose reduction > 20% to a total insulin <0.4 U/kg will be monitored closely for insulin dose, carbohydrate intake, ketones and blood glucose. Study medication can be temporarily discontinued, if necessary, for one-to-two weeks. If low insulin requirements remain, the participant will be withdrawn from study (see Section 7.8).

9 PHARMACOVIGILANCE

9.1 Definitions

Term	Definition
**Adverse Event (AE)**	Any untoward medical occurrence in a participant to whom a medicinal product has been administered, including occurrences which are not necessarily caused by or related to that product.
**Adverse Reaction (AR)**	An untoward and unintended response in a participant to an investigational medicinal product which is related to any dose administered to that participant.<br>The phrase "response to an investigational medicinal product" means that a causal relationship between a trial medication and an AE is at least a reasonable possibility, i.e. the relationship cannot be ruled out.<br>All cases judged by either the reporting medically qualified professional or the Sponsor as having a reasonable suspected causal relationship to the trial medication qualify as adverse reactions.
**Serious Adverse Event (SAE)**	A serious adverse event is any untoward medical occurrence that:<br>1. results in death<br>2. is life-threatening<br>3. requires inpatient hospitalisation or prolongation of existing hospitalisation<br>4. results in persistent or significant disability/incapacity<br>5. consists of a congenital anomaly or birth defect<br><br>Other ‘important medical events’ may also be considered serious if they jeopardise the participant or require an intervention to prevent one of the above consequences.<br>NOTE: The term "life-threatening" in the definition of "serious" refers to an event in which the participant was at risk of death at the time of the event; it does not refer to an event which hypothetically might have caused death if it were more severe*.
**Serious Adverse Reaction (SAR)**	An adverse event that is both serious and, in the opinion of the reporting Investigator, believed with reasonable probability to be due to one of the trial treatments, based on the information provided.
**Suspected Unexpected Serious Adverse Reaction (SUSAR)**	A serious adverse reaction, the nature and severity of which is not consistent with the information about the medicinal product in question set out:<br>• in the case of a product with a marketing authorisation, in the summary of product characteristics (SmPC) for that product<br>• in the case of any other investigational medicinal product, in the investigator’s brochure (IB) relating to the trial in question
**Reference Safety Information**	The information used for assessing whether an adverse reaction is expected. This is contained in either the investigator’s brochure or the summary of product characteristics

*Note: “Severe” is often used to describe intensity of a specific event, which may be of relatively minor medical significance. “Seriousness” is the regulatory definition supplied above.

9.2 Operational definitions for (S)AEs

Adverse events (AEs) will be recorded, notified, assessed, reported, analysed and managed in accordance with the Medicines for Human Use (Clinical Trials) Regulations 2004 (as amended) and this protocol.

All Serious Adverse Events (SAEs) occurring during the trial will be recorded within the eCRF. Non-serious AEs occurring during the trial will be recorded within the eCRF if they are considered to be related to trial medication, i.e. adverse reactions (ARs).

Exclusions from SAE reporting

Hospitalisation for the following reasons will not be considered to be SAEs:

- Treatment which was elective or pre-planned, for a pre-existing condition not associated with any deterioration in condition, e.g. pre-planned hip replacement operation which does not lead to further complications.
- Any admission to hospital or other institution for general care where there was no deterioration in condition.
- Treatment on an emergency, outpatient basis for an event **not** fulfilling any of the definitions of serious as given above and not resulting in hospital admission.

9.3 Recording and reporting of AEs, Adverse Events of Special Interest, SAEs and SUSARs

All AEs occurring during the trial that are observed by the Investigator or reported by the participant will be recorded in the participant's medical records whether or not attributed to trial IMP. AEs will be recorded from consent until the later of 30 days post cessation of trial treatment or the end of the study.

Adverse Events of Special Interest, SAEs and SUSARs will be recorded in the participant's medical records and on the eCRF and will be recorded from the point of randomisation until the later of 30 days post cessation of trial treatment or the end of the study.

Additional events identified only through record linkage will generate SAE records in the eCRF and should be recorded in the participant's medical records in the same way as AEs.

9.3.1 Adverse Events of Special Interest

Adverse Events of Special Interest as defined in detail below should be recorded within the relevant section of the eCRF but do not require reporting in an expedited manner (unless the event also meets the regulatory definition of an SAE). The following information will be recorded for all events:

- Nature of the event
- Event duration (start and end dates, if applicable)
- Relationship to study drug (if applicable)
- Outcome (if applicable)

Adverse Events of Special Interest will be monitored and followed up (if applicable) until the event has resolved or a final outcome has been reached.

All Adverse Events of Special Interest must be assessed for seriousness prior to recording within the eCRF. Those meeting the definition of an SAE should also be reported accordingly as per Section 9.3.2.

(i) Ketosis and diabetic ketoacidosis

This trial will be conducted in participants who have type 1 diabetes. All participants will be taking insulin. In this condition, due to the near absence of endogenous insulin, at a cellular level there is an inability to incorporate glucose into normal intra-cellular energy yielding pathways. In this context, alternative energy yielding pathways are sought including ketone body production from fatty acids in the liver. Ketones are clinically important as they easily dissociate hydrogen ions upsetting physiologically regulated acid base balance. As ketone levels rise, there is initially a state of high blood ketone levels without significant acidosis (ketosis); if this develops further, diabetic ketoacidosis (DKA) may develop. DKA requires urgent medical input. Ketones can be easily measured via a simple urine test or fingerstick capillary blood measurement. Participants eligible for this trial will already be trained in how to do this. Although dapagliflozin, one of the IMPs in this trial, has a license for use in type 1 diabetes, it is known to increase the risk of ketosis and ketoacidosis and this can occur at lower blood glucose levels than in those who are on insulin treatment only [euglycaemic DKA] (13,14). Given this known risk, ketosis and DKA are included in the safety endpoints and as such will be recorded in the eCRF as Adverse Events of Special Interest.

DKA is defined clinically and biochemically as a combination of a state of elevated ketone levels (urine ++ or higher, and/or blood > 1.5 mmol/L) in combination with biochemical evidence of a state of metabolic acidosis (pH ≤7.30 or serum bicarbonate < 15 mmol/L). This is as defined by International Consensus Guidelines (34). DKA also varies in severity. In clinical practice the diagnosis is made when a person with type 1 diabetes has elevated ketone levels and usually – although not exclusively – elevated blood glucose levels. The diagnosis is made biochemically by assessing acid base balance through blood testing. This corresponds to the following severity classifications:

- mild DKA: pH 7.25-7.30 (H^+ 50 – 56) or bicarbonate (15 -17.9 mmol/L)
- moderate DKA: pH 7.00-7.24 (H^+ 57 – 100) or bicarbonate 10 – 14.9 mmol/L)
- severe DKA: pH <7.00 (H^+ > 100) or bicarbonate < 10 mmol/L (33)

Given the known risk of DKA, all participants will be made aware of this condition. Patient diaries and Patient Alert Cards will summarise advice for management of high ketone levels including an emergency ketosis management protocol (STICH Protocol – see Appendix 3). All participants will be educated as to the scenarios when this should be activated and this will also be included on the Patient Alert Card which will have the contact details of the study team and participants will be asked to carry at all times.

(ii) Hypoglycaemia

Hypoglycaemia is a low blood glucose level. Hypoglycaemia occurs in diabetes owing to difficulty in precisely matching insulin dosing with the complex factors associated with carbohydrate intake and physical activity. When too much insulin relative to carbohydrate intake is administered, blood glucose levels can drop to a level which is too low. Most people with type 1 diabetes can recognize this phenomenon due to a combination of symptoms which alert them and prompt self-checking of their capillary blood glucose level. Symptoms include weakness, malaise, headache, tremulousness, diaphoresis, palpitations and mild confusion. These are known as autonomic symptoms as they are mediated by activation of the autonomic nervous system as a response to the biological stress of low serum blood glucose. As hypoglycaemia progresses, however, symptoms progress to include confusion and agitation. If untreated this can lead to coma. These symptoms are known as neuroglycopenic symptoms as they are mediated by a lack of glucose in neural cells leading to a confused state.

Hypoglycaemia is also well defined by International Consensus (34). Like DKA, it can be classified in terms of severity. This can be based either on the absolute capillary glucose value and/or clinical features and is classed into stages:

- Level 1 (mild) hypoglycaemia: Blood glucose is 3.0 – 3.8 mmol/L inclusive.
- Level 2 (moderate) hypoglycaemia: Blood glucose is < 3.0 mmol/L
- Level 3 (severe) hypoglycaemia: A person is unable to function because of mental or physical changes due to hypoglycaemia and requires assistance from another person to recover.

The IMPs in this trial are licensed clinically in type 2 diabetes to lower blood glucose levels. It therefore follows that when used in combination with insulin there may be an increased risk of low blood glucose levels. This was the case with the once daily GLP-1 analogue liraglutide in phase 3 trials (8,9), but not in the trials with dapagliflozin (13,14). All participants will have this risk discussed with them, will be performing increased blood glucose monitoring than is the case in usual care, and will be frequently reviewed to adjust insulin dosing in order to mitigate the risk of low blood glucose levels.

9.3.2 Site Reported Serious Adverse Events (SAE)

SAEs will be recorded within the eCRF from the date of randomisation until the later of 30 days post cessation of trial treatment or the end of the study.
Full details of SAEs will be recorded in the electronic Case Report Form. The following information will be collected:

- full details in medical terms and a case description
- seriousness criteria
- event duration (start and end dates, if applicable)
- action taken
- outcome
- causality (i.e. relatedness to trial drug / investigation), in the opinion of the investigator
- if related, whether the reaction would be considered expected or unexpected.

Any change of condition or other follow-up information should be added to the eCRF and forwarded to the Sponsor (if reportable SAE) as soon as it is available or at least within 24 hours of the information becoming available. Events will be followed up until the event has resolved or a final outcome has been reached.

Assessment of Adverse Events

All adverse events must be assessed by the reporting site for seriousness**.** All SAEs must also be assessed for causality and expectedness with reference to this protocol and the Reference Safety Information (RSI).

Assessment of seriousness

An adverse event will be considered serious if it:

1. Results in death
2. Is life threatening
3. Requires hospitalisation or prolongation of existing hospitalisation
4. Results in persistent or significant disability or incapacity
5. Consists of a congenital anomaly or birth defect

6. Required intervention to prevent one of the above.
7. Is a serious Adverse Event of Special Interest.

Assessment of causality i.e. does the event have a "reasonable causal relationship" with trial IMPs. The following categories are used:

- **Yes** - The causal and temporal relationship between the event and the absence of a more likely explanation suggest that the trial IMP is the most likely cause.
- **No** - The event is not considered related to the trial IMPs.

Causality will be assigned for each IMP the participant is randomised to receive.

Should an SAE be considered unrelated to the administration of either trial drug then the causality of the event will be collected taking into consideration other factors, for example concomitant medications or co-morbidities, etc.

Assessment of causality is the responsibility of the Principal Investigator or medically-qualified designee(s) at each site. The CI, their delegate(s), or the Sponsor may provide an opinion regarding causality but cannot downgrade the PI's assessment.

Assessment of expectedness

Should the event be considered to be related to the trial IMP(s), an assessment should be made regarding the expectedness of the reaction, i.e. whether the reaction is a recognised adverse effect of the IMP(s).

.
Expected: consistent with the relevant product information documented in the RSI(s).
Unexpected: not consistent with the relevant product information documented in the RSI(s).

The CI, their delegate(s) or the Sponsor have overall responsibility for assigning expectedness as per the RSI(s) and provide the final assessment of the event. The reporting investigator may provide an opinion on the expectedness of an event and this may be taken into account.

SAEs occurring within the USA and the UK

The US Sponsor is responsible for assigning causality and expectedness for any AEs, SAEs and SUSARs that occur within the USA. Where a SUSAR is identified by the US Sponsor this will be forwarded to the UK Sponsor for review within one working day. Following review by the UK Sponsor, appropriate US SUSARs will be subject to expedited reporting to the MHRA as per UK regulatory requirements. Similarly, where a SUSAR is identified by the UK sponsor, this will forwarded to the US Sponsor for review within two working days.

Recording and reporting of SAEs
All SAEs arising during the clinical trial will be recorded in the eCRF soon as reasonably practicable and in any event within 24hours of first becoming aware of the event. Any follow-up information should also be reported.
If recording in the eCRF is not possible a paper SAE form should be completed:

1. The SAE form is downloaded from www.glasgowctu.org, printed off, completed and signed. The form is then faxed to the Glasgow Clinical Trials Unit Pharmacovigilance (PV) Office on +44(0)141 357 5588. As an alternative, a scanned copy of the SAE form may be emailed to:

pharmacovig@glasgowctu.org. If this website is unavailable a paper copy of the SAE form should be filed in the Investigator Site File at each site pending resolution.

2. If necessary a verbal report can be given by contacting the PV Office on +44(0)141 330 4744. This must be followed up as soon as possible with an electronic or written report.

Reporting to sponsor

All SAEs will be considered reportable to the Sponsor PV office from the time of randomisation until 30 days following permanent discontinuation of all trial IMP(s). SAEs occurring following 30 days post cessation of treatment are not reportable to the Sponsor unless an SAE is considered related to IMP(s)

9.3.3 Suspected Unexpected Serious Adverse Reactions (SUSARs)

Any SAE assessed as related to trial IMP(s) by the PI or delegate, and unexpected (not documented as an expected reaction to the IMP(s) within the RSI(s)) by the CI, their delegate, or the Sponsor, will be classified as a SUSAR and therefore subject to expedited reporting to the Medicines and Healthcare products Regulatory Agency (MHRA) and the Research Ethics Committee (REC). Should the CI disagree with the PI's assessment of causality, both opinions will be provided on the report. Where the CI or UK Sponsor disagrees with the US Sponsor's assessment of expectedness both opinions will also be provided on the report.

The Sponsor will inform the MHRA of all SUSARs that occur within the trial and the REC of all SUSARs that occur within the UK within the required expedited reporting timescales:

- **Fatal or life threatening SUSARs** - no later than 7 days of Sponsor awareness that the case fulfils the criteria for a fatal or life threatening SUSAR, and any follow up information within a further 8 days.
- **All other SUSARs** - no later than 15 days of Sponsor awareness that the case fulfils the criteria for a SUSAR.

The sponsor will report SUSARs to the MHRA via the MHRA eSUSAR reporting system and to REC by email with accompanying CTIMP Safety Report Form.

9.3.4 Unblinding for SUSAR reporting purposes

In the event of a potential SUSAR, the sponsor (but not the investigators) will be unblinded to facilitate appropriate reporting to the MHRA and REC.

9.4 Responsibilities for Safety Reporting and Review

This section details the responsibilities for reporting and reviewing safety information arising from the trial.

Principal Investigator (PI):

- Attendance at the initiation meeting/teleconference
- Training of new members of the trial team in the protocol and its procedures
- Ensuring that the ISF is accurately maintained
- Dissemination of important safety or trial-related information to all stakeholders at their site
- Checking for AEs and ARs when participants attend for treatment/follow-up

- Ensuring that AEs are recorded and reported in line with the requirements of the protocol
- Ensuring that all SAEs are recorded and reported to the Sponsor within 24 hours of becoming aware of the event, and providing further follow-up information as soon as available
- Using medical judgement in assigning seriousness, causality, and severity of SAEs
- Providing an opinion on the expectedness of an SAE with reference to the trial protocol and Reference Safety Information
- Using definitions in this protocol, flagging events of special interest.

Chief Investigator (CI)

- Providing clinical oversight of the safety of patients participating in the trial, including an ongoing review of the risk/benefit
- Providing clinical review of section 4.8 of the representative SmPCs on an annual basis to define the Reference Safety Information for the trial
- Using medical judgement to confirm seriousness and causality of all SAEs
- Reviewing expectedness of related SAEs with reference to the trial protocol and Reference Safety Information
- Preparing the clinical sections and final sign-off of the Development Safety Update Report (DSUR)Using definitions in this protocol, confirming events of special interest

Sponsor:

- Central data collection and verification of AEs, SAEs, SARs and SUSARs according to the trial protocol
- Reporting safety information to the CI or delegate for the ongoing assessment of the risk / benefit
- Reporting safety information to the independent oversight committees identified for the trial (Independent Data Monitoring Committee (IDMC) and / or Trial Steering Committee (TSC)) according to the Trial Monitoring Plan.
- Expedited reporting of SUSARs to the Competent Authority (MHRA in UK) and REC within required timelines.
- Notifying Investigators of SUSARs that occur within the trial.
- Checking for (annually) and notifying PIs of updates to the Reference Safety Information for the trial.
- Preparing standard tables and other relevant information for the DSUR in collaboration with the CI and ensuring timely submission to the MHRA and REC.

Trial Steering Committee:

In accordance with the Charter for the TSC, periodically reviewing recruitment and the overall progress of the trial and liaising with the IDMC and sponsor regarding safety issues.

Independent Data Monitoring Committee:

In accordance with the Charter for the IDMC, periodically reviewing unblinded safety data in individual cases and to determine patterns and trends of events, or to identify safety issues, which would not be apparent on an individual case basis, reporting concerns to the TSC and sponsor.

9.5 Pregnancy reporting

Pregnancy is not considered an AE unless a negative or consequential outcome is recorded for the mother or child/foetus. If the outcome meets the serious criteria, this would be considered an SAE and must be reported as per SAE reporting procedure above.

Any **pregnancy** occurring in a female trial participant or female partner of a male trial participant who becomes pregnant while participating in the Trial will be reported by the PI (or designee) to the Chief Investigator and the sponsor using the sponsor Pregnancy Reporting Form (available at http://www.glasgowctu.org/) within two weeks of the PI first becoming aware of the pregnancy.
The trial participant will also be followed up to determine the outcome of the pregnancy and follow-up information forwarded to the PV office. Any resulting SAEs should be reported as per SAE reporting procedure above.

9.6 Overdose

Any IMP dose which is not administered in accordance with the Protocol should be reported to the sponsor.
If an SAE is associated with an overdose ensure that the overdose if fully described in the SAE report form.

9.7 Reporting urgent safety measures

If any urgent safety measures are taken the CI/Sponsor will phone the MHRA's Clinical Trial Unit on 020 3080 6456, ideally within 24 hours. This will be followed up no later than 3 days from the date the measures are taken, giving written notice to the MHRA (who will advise the format required) and the relevant REC of the measures taken and the circumstances giving rise to those measures. A substantial amendment must also be submitted to the MHRA.

9.8 The type and duration of the follow-up of participants after adverse events.

Adverse events and reactions will be recorded, reported and followed up in line with this protocol until study completion or for a minimum of 30 days after participant's last dose of the IMP, whichever is later. Any SUSAR identified will be reported to the Sponsor and to the Regulatory Authorities irrespective of how long after IMP administration the reaction has occurred.

9.9 Development safety update reports

A Development Safety Update Report (DSUR) will be submitted once a year, or on request, to MHRA and REC until the trial is declared ended. The report will be submitted within 60 days of the anniversary of the issue of the Clinical Trials Authorisation for the trial. The DSUR will be prepared by the sponsor (PV Office) in liaison with the CI and submitted by the sponsor (PV Office). The DSUR will include only those SAEs reported to the co-sponsors; that is: SAEs and SUSARs occurring within the UK and SUSARs originating in the USA.

10 STATISTICS AND DATA ANALYSIS

10.1 Sample size calculation

This is based on our current data showing that in type 1 diabetes, treatment with a GLP-1 agonist, liraglutide (1.2 and 1.8mg doses) for three months leads to HbA1c suppression by an average of 0.6% (SD= 0.5%). Dapagliflozin was recently shown to reduce HbA1c by 0.45% in type 1 diabetes patients (13). Additionally, empagliflozin treatment for 8 weeks was shown to reduce HbA1c by 0.4% in type 1 diabetics (20, 21) and our current data showing additional improvement in HbA1c by an additional 0.66% at 12 weeks when dapagliflozin was added to liraglutide and insulin therapy. Conservatively estimating that the combination therapy will suppress HbA1c by 0.5% (SD≤0.7%) compared to semaglutide alone, a sample size of 38 (32 evaluable patients) in each group will be sufficient to provide 80% power at a

5% significance level, taking in consideration a 15% dropout rate. 50% of participants will be recruited in Glasgow, UK and 50% at State University of New York at Buffalo, USA.

10.2 Anticipated recruitment rate

One year (one participant per week).

10.3 Statistical analysis

The primary objective of the proposed research is to evaluate the effect of addition of dapagliflozin to semaglutide on a background of insulin treatment in type 1 diabetes patients on HbA1c at six months following start of dapagliflozin. Transformations of the data in order to meet statistical assumptions will be considered. Statistical analyses will use linear regression models to compare treatment groups, with adjustment for baseline values of study outcomes, as well as factors used to stratify the randomisation scheme. Tests will be two-sided and tested at the 5% significance level. Analyses will be carried out under the intention to treat principle, that is, according to randomised treatment allocation, regardless of treatment adherence.

A detailed Statistical Analysis Plan will be completed and signed off before database lock. Study statisticians will develop and validate the analysis programs without access to randomised treatment allocations. After database lock, the treatment codes will be released and the final analysis will be carried out.

10.4 Subgroup analyses

These will be specified in the Statistical Analysis Plan. Subgroup analyses will be implemented by extending regression models to include interactions between treatment and potential treatment effect moderators.

10.5 Interim analysis and criteria for the premature termination of the trial

No interim analysis is planned. Unblinded trial data will be reviewed on an ongoing basis by the IDMC. The primary role of the IDMC will be to protect the interests of the patients. The IDMC may recommend to the TSC and Co-Sponsors that the study should stop prematurely because of concerns about patient safety. The IDMC will meet approximately every six months. The IDMC will take into account all results and the consistency and biological plausibility of the findings in making any recommendation. The final decision on continuing or stopping the trial will lie with the TSC/Co-Sponsors.

10.6 Subject population

All analyses will be carried out on an intention to treat basis, using the randomised treatment allocation.

10.7 Procedure(s) to account for missing or spurious data

Data will be analysed without imputation in the first instance. Multiple imputation procedures will be used to assess the sensitivity of selected results to missing data, according to the Statistical Analysis Plan.

10.8 Other statistical considerations.

The Statistical Analysis Plan (SAP) will be maintained as a version controlled document and will be signed off before database lock. The SAP and other statistical documentation (according to Glasgow Clinical Trials Unit Standard Operating Procedures) will contain full details of all analyses along with assumptions and procedures for handling problematic or incomplete data (e.g. incomplete dates).

11 DATA HANDLING

11.1 Source Documentation

ICH GCP defines source data as: ‘All information in original records and certified copies of original records of clinical findings, observations or other activities in a clinical trial necessary for the reconstruction and evaluation of the trial’. In this study, the location of the majority of the source data will be the hospital's medical records including subject case notes, laboratory records and ECGs. The source data transcribed into the eCRF from the medical records must be accurate and verifiable. For questionnaires completed by trial subjects, the completed questionnaires will be regarded as the source data location. In cases where data is transcribed directly into the eCRF and no other paper or electronic source exists, then the eCRF will be considered the source record. In these cases, these data should be prospectively documented in the medical records to ensure a full record of the trial is available at site.

11.2 Data collection

An eCRF, developed by the Robertson Centre for Biostatistics, will capture all data required to meet this protocol’s requirements. Access to the eCRF will be restricted, via a study-specific web portal, and only authorised site-specific personnel will be able to make entries to their patients’ data via the web portal. The Investigator, or his/her designee will be responsible for all entries into the eCRF and will confirm that the data are accurate, complete and verifiable. Data will be stored in a MS SQL Server database. Direct access to the web portal will be granted, on request, to authorised representatives from the Sponsor, host institution and the regulatory authorities to permit trial-related monitoring, audits and inspections.

11.3 Data Validation

Where it is practical, data will be validated at the point of entry into the eCRF. Any additional data discrepancies will be flagged to the investigator and any data changes will be recorded to maintain a complete audit trail (reason for change, date change made, who made change).

11.4 Data Security

The Robertson Centre for Biostatistics systems are fully validated in accordance with industry and regulatory standards, and incorporate controlled access security. High volume servers are firewall protected and preventative system maintenance policies are in place to ensure no loss of service or data. Web servers are secured by digital certificates. Data integrity is assured by strictly controlled procedures, including secure data transfer procedures. Data are backed up on-site nightly and off-site to a commercial data vault weekly. The Robertson Centre for Biostatistics has an ISO 9001:2008 quality management system and ISO 27001:2013 for Information Security, and is regularly inspected against the standards by the British Standards Institution.

11.5 Archiving

The Trial Master File will be archived by the Co-Sponsors at the end of the trial for a minimum period of five years.

Archiving of Site Files will also be for a minimum of five years form completion of the trial, and this action will be delegated to the sites in the Clinical Trial Site Agreement that will be put in place between Co-Sponsors and Sites. Sites will be notified by the Co-Sponsors when Site files can be archived.

Destruction of site files can only take place with the approval of the Co-Sponsors.

12 MONITORING, AUDIT & INSPECTION

Monitoring will be conducted by NHS Greater Glasgow and Clyde (GG&C) Monitor (s) in accordance with local Standard Operating Procedures. The level, frequency and priorities of monitoring will be based on the outcome of the completed Risk Assessment,and clearly documented in the Monitoring Plan which will be approved by the NHS GG&C Research Governance Manager.

13 ETHICAL AND REGULATORY CONSIDERATIONS

13.1 Research Ethics Committee (REC) review & reports

Before the start of the trial, approval will be sought from a REC for the trial protocol, informed consent forms and other relevant documents e.g. advertisements and GP information letters.
Substantial amendments that require review by REC will not be implemented until the REC grants a favourable opinion for the study (it is noted that amendments may also need to be reviewed and accepted by the MHRA and/or NHS R&D departments before they can be implemented in practice at sites).
All correspondence with the REC will be retained in the Trial Master File/Investigator Site File.
An annual progress report (APR) will be submitted to the REC within 30 days of the anniversary date on which the favourable opinion was given, and annually until the trial is declared ended (this is the Chief Investigator's responsibility).
The Chief Investigator will notify the REC of the end of the study.
If the study is ended prematurely, the Chief Investigator will notify the REC, including the reasons for the premature termination
Within one year after the end of the study, the Chief Investigator will submit a final report with the results, including any publications/abstracts, to the REC.

13.2 Peer review

The study protocol has been developed with expert and independent feedback from the JDRF (New York) and during application for funding underwent peer review by independent experts.

13.3 Public and Patient Involvement

The study was designed with input from people with type 1 diabetes via the funder, JDRF.

13.4 Regulatory Compliance

The trial will not commence until a Clinical Trial Authorisation (CTA) is obtained from the MHRA.
The protocol and trial conduct will comply with the Medicines for Human Use (Clinical Trials) Regulations 2004 and any relevant amendments.
Before any site can enrol patients into the trial, the Chief Investigator/Principal Investigator or designee will apply for NHS permission from the site's Research & Development (R&D) department.

For any amendment that will potentially affect a site's NHS permission, the Chief Investigator/ Principal Investigator or designee will confirm with that site's R&D department that NHS permission is ongoing (note that both substantial amendments, and amendments considered to be non-substantial for the purposes of REC and/or MHRA may still need to be notified to NHS R&D).

13.5 Protocol compliance

Prospective, planned deviations or waivers to the protocol are not allowed under the UK regulations on Clinical Trials and must not be used e.g. it is not acceptable to enrol a participant if they do not meet the eligibility criteria or restrictions specified in the trial protocol. Accidental protocol deviations can happen at any time. They must be adequately documented on the relevant forms and reported to the Chief

Investigator, Sponsor and GCTU immediately. Deviations from the protocol which are found to frequently recur are not acceptable, will require immediate action and could potentially be classified as a serious breach.

13.6 Notification of Serious Breaches to GCP and/or the protocol

A "serious breach" is a breach which is likely to affect to a significant degree –

1. the safety or physical or mental integrity of the subjects of the trial; or
2. the scientific value of the trial

If any of the above occurs then the CI and Sponsor will be notified. The sponsor will notify the appropriate authorities in writing of any serious breach in accordance with their standard operating procedures.

13.7 Data protection and patient confidentiality

All investigators and trial site staff must comply with the requirements of the GDPR with regards to the collection, storage, processing and disclosure of personal information and will uphold the Act's core principles.

- Personal information will be collected via the eCRF to enable record linkage to be carried out and to provide electronic access to study monitors to a copy of the signed informed consent document. These data items will be encrypted and only those individuals who require to see these data i.e. the person performing the record linkage and site research team staff or the study monitor, as appropriate, will be able to view them. All electronic data will be held securely in accordance with ISO 27001:2013 at the Robertson Centre for Biostatistics, part of the Glasgow Clinical Trials Unit. All Centre staff are required to sign confidentiality agreements and to follow Standard Operating Procedures in accordance with Good Clinical Practice and ISO certification.
- The trial data managers, statisticians, health economists or any other staff who will perform data related tasks will only be able to access depersonalised data where the participant's identifying information is replaced by a unique study identifier.

- Only those that have been trained and approved will be able to enter or view any data via the web portal. Each site can only see their own patients' data. Patient consent forms will be stored at the study site in a secure location accessible only to study teams.

13.8 Indemnity

The Co-Sponsors (University of Glasgow and Greater Glasgow Health Board) will ensure that provision has been made for insurance or indemnity to cover the liability of the investigator and sponsor which may arise in relation to the clinical trial in accordance with Part 2 (14) of Schedule 1 to SI 2004/1031.

13.9 Amendments

Any change in the study protocol will require an amendment. Any proposed protocol amendments will be initiated by the CI following discussion with the Sponsor and TSC and any required amendment forms will be submitted to the regulatory authority, ethics committee and Sponsor. The Sponsor will determine whether an amendment is non-substantial or substantial. All amended versions of the protocol will be signed by the CI and Sponsor representative. Following a substantial amendment, favourable opinion/approval must be sought from the original reviewing REC, MHRA (where appropriate) and Research and Development (R&D) office prior to implementation. The Chief Investigator will be responsible for informing the Trial Management Group of all protocol amendments.

13.10 Post trial care

At the end of the trial, participants will be returned to usual care as defined by local and national guidelines at that time. The results of the trial may of course have an impact on these guidelines and the future care of patients with type 1 diabetes.

13.11 Access to the final trial dataset

During the trial and in the period prior to publication of the main study results as described in the protocol, only the Glasgow CTU and Professor Paresh Dandona, US Principal Investigator, University of Buffalo will have access to the full dataset. After that period, the trial Steering Committee will conduct further data analyses for a period of three years. After that time the Trial Steering Committee will consider requests from external parties for further analyses of the study data. Proposals that are scientifically well founded and have an academic basis and where relevant data extractions and analyses are appropriately funded will not be refused. These will be considered as collaborative exercises where the contributions related to study design, conduct, database creation and maintenance and data analysis will be recognised in authorship of any scientific publication. The approach we will take will be to minimise any possibility of breach of participant confidentiality. Normally this will be achieved by minimising data travel. However, for the purposes of individual patient meta-analysis and other reasons, data may be transferred to other sites. Such transfer will require assurances on information security systems at the sites that data are to be transferred to and will involve a legal data transfer agreement. A log of all data requests and subsequent data transfers will be held at the Glasgow CTU.

14 DISSEMINATION POLICY

14.1 Dissemination policy

The study database will be owned by the University of Glasgow and maintained on behalf of the Study investigators, represented by the Trial Steering Committee as it is constituted during and after the trial.

The study protocol and a description of the recruitment experience and participant baseline characteristics will be published before study completion. On completion of the trial, the database will be locked and analysed by staff of the Robertson Centre for Biostatistics, University of Glasgow. A final study report will be prepared and the results will be published in a major medical journal.

After the main study publications, study investigators will be invited to submit requests for further analyses of the study database. These will be reviewed and prioritised by a Publications Committee made up of the study grant holders and convened by the study co-CIs.

As funder of the trial, JDRF will have the right to see and comment on any results being submitted for publication. A maximum of 28 days will be given for review of major papers and 14 days for abstracts. Such comments will be considered by the Trial Steering Committee.

A lay summary of the main results of the trial will be prepared and provided to all participants via their study site investigators. Investigators may request a copy of the study data for their participants. Providing some or all of a patient's data to that individual is at their discretion.

14.2 Authorship eligibility guidelines and any intended use of professional writers

The main results of the study will be compiled, written up and published by the study grant holders and others taking responsibility for the study results (e.g. the statistician conducting the final analysis) on behalf of the TTT1 investigators. The TTT1 investigators will be listed in an Appendix and will include all site PIs, all committee members and key members of relevant study coordinating groups (including the Sponsor and Glasgow CTU).

15 REFERENCES

1. Konrad K, Datz N, Engelsberger I, Grulich-Henn J, Hoertenhuber T, Knauth B, et al. Current use of metformin in addition to insulin in pediatric patients with type 1 diabetes mellitus: an analysis based on a large diabetes registry in Germany and Austria. *Pediatr Diabetes.* 2014.
2. Vella S, Buetow L, Royle P, Livingstone S, Colhoun HM, and Petrie JR. The use of metformin in type 1 diabetes: a systematic review of efficacy. *Diabetologia.* 2010;53(5):809-20.
3. Vella S, Buetow L, Royle P, Livingstone S, and Petrie JR. Metformin in type 1 diabetes reduces insulin requirements without significantly improving glycaemic control. Reply to Schatz H [letter]. *Diabetologia.* 2011;54(1):203-4.
4. Varanasi A, Bellini N, Rawal D, Vora M, Makdissi A, Dhindsa S, et al. Liraglutide as additional treatment for type 1 diabetes. *Eur J Endocrinol.* 2011;165(1):77-84.
5. Kuhadiya ND, Malik R, Bellini NJ, Patterson JL, Traina A, Makdissi A, et al. Liraglutide as additional treatment to insulin in obese patients with type 1 diabetes mellitus. *Endocrine practice : official journal of the American College of Endocrinology and the American Association of Clinical Endocrinologists.* 2013;19(6):963-7.
6. Kuhadiya ND, Dhindsa S, Ghanim H, Mehta A, Makdissi A, Batra M, et al. Addition of Liraglutide to Insulin in Patients With Type 1 Diabetes: A Randomized Placebo-Controlled Clinical Trial of 12 Weeks. *Diabetes Care.* 2016;39(6):1027-35.
7. Chaudhuri A, Ghanim H, Makdissi A, Green K, Abuaysheh S, Batra M, et al. Exenatide induces an increase in vasodilatory and a decrease in vasoconstrictive mediators. *Diabetes Obes Metab.* 2017;19(5):729-33.
8. Mathieu C, Zinman B, Hemmingsson JU, Woo V, Colman P, Christiansen E, et al. Efficacy and Safety of Liraglutide Added to Insulin Treatment in Type 1 Diabetes: The ADJUNCT ONE Treat-To-Target Randomized Trial. *Diabetes Care.* 2016;39(10):1702-10.
9. Ahren B, Hirsch IB, Pieber TR, Mathieu C, Gomez-Peralta F, Hansen TK, et al. Efficacy and Safety of Liraglutide Added to Capped Insulin Treatment in Subjects With Type 1 Diabetes: The ADJUNCT TWO Randomized Trial. *Diabetes Care.* 2016;39(10):1693-701.
10. Zinman B, Wanner C, Lachin JM, Fitchett D, Bluhmki E, Hantel S, et al. Empagliflozin, Cardiovascular Outcomes, and Mortality in Type 2 Diabetes. *N Engl J Med.* 2015;373(22):2117-28.
11. Petrie JR. SGLT2 inhibitors in type 1 diabetes: knocked down, but up again? *Lancet Diabetes Endocrinol.* 2017;5(11):841-3.
12. Mudaliar S, Armstrong DA, Mavian AA, O'Connor-Semmes R, Mydlow PK, Ye J, et al. Remogliflozin etabonate, a selective inhibitor of the sodium-glucose transporter 2, improves serum glucose profiles in type 1 diabetes. *Diabetes Care.* 2012;35(11):2198-200.
13. Dandona P, Mathieu C, Phillip M, Hansen L, Griffen SC, Tschope D, et al. Efficacy and safety of dapagliflozin in patients with inadequately controlled type 1 diabetes (DEPICT-1): 24 week results from a multicentre, double-blind, phase 3, randomised controlled trial. *Lancet Diabetes Endocrinol.* 2017;5(11):864-76.
14. Mathieu C, Dandona P, Gillard P, Senior P, Hasslacher C, Araki E, Lind M, Bain SC, Jabbour S, Arya N, Hansen L, Thorén F, Langkilde AM; DEPICT-2 Investigators. Efficacy and Safety of Dapagliflozin in Patients With Inadequately Controlled Type 1 Diabetes (the DEPICT-2 Study): 24-Week Results From a Randomized Controlled Trial. Diabetes Care. 2018 Sep;41(9):1938-1946.

15. European Medicines Agency. Assessment Report Dapagliflozin. https://www.ema.europa.eu/en/documents/variation-report/forxiga-h-c-2322-ws-1344-epar-assessment-report-variation_ar.pdf
16. Rosenstock J, Marquard J, Laffel LM, et al. Empagliflozin as adjunctive to insulin therapy in type 1 diabetes: the EASE trials. *Diabetes Care*. 2018;41(12):2560–2569.
17. Garg SK, Henry RR, Banks P, Buse JB, Davies MJ, Fulcher GR, et al. Effects of Sotagliflozin Added to Insulin in Patients with Type 1 Diabetes. *N Engl J Med.* 2017;377(24):2337-48.
18. European Medicines Agency. First oral add-on treatment to insulin for treatment of certain patients with type 1 diabetes. https://www.ema.europa.eu/en/news/first-oral-add-treatment-insulin-treatment-certain-patients-type-1-diabetes
19. An overview of Zynquista and why it is authorised in the EU. 16th May 2019. https://www.ema.europa.eu/en/documents/overview/zynquista-epar-medicine-overview_en.pdf
20. National Institute for Health and Care Excellence. Dapagliflozin with insulin for treating type 1 diabetes.
21. Ahrén B, Hirsch IB, Pieber TR, Mathieu C, Gómez-Peralta F, Hansen TK, Philotheou A, Birch S, Christiansen E, Jensen TJ, Buse JB; ADJUNCT TWO Investigators. Efficacy and Safety of Liraglutide Added to Capped Insulin Treatment in Subjects With Type 1 Diabetes: The ADJUNCT TWO Randomized Trial. Diabetes Care. 2016 Oct;39(10):1693-701.
22. Ozempic Summary of product characteristics. https://www.medicines.org.uk/emc/product/9728/smpc
23. Nauck MA, Petrie JR, Sesti G, Mannucci E, Courrèges JP, Lindegaard ML, Jensen CB, Atkin SL; Study 1821 Investigators. A Phase 2, Randomized, Dose-Finding Study of the Novel Once-Weekly Human GLP-1 Analog, Semaglutide, Compared With Placebo and Open-Label Liraglutide in Patients With Type 2 Diabetes. Diabetes Care. 2016 Feb;39(2):231-41.
24. Technology appraisal guidance [TA597] https://www.nice.org.uk/guidance/ta597
25. Dapagliflozin Summary of Product Characteristics: https://www.medicines.org.uk/emc/product/2865/smpc
26. Wiviott SD, Raz I, Bonaca MP, Mosenzon O, Kato ET, Cahn A, Silverman MG, Zelniker TA, Kuder JF, Murphy SA, Bhatt DL, Leiter LA, McGuire DK, Wilding JPH, Ruff CT, Gause-Nilsson IAM, Fredriksson M, Johansson PA, Langkilde AM, Sabatine MS; DECLARE–TIMI 58 Investigators. Dapagliflozin and Cardiovascular Outcomes inType 2 Diabetes. N Engl J Med. 2019 Jan 24;380(4):347-357.
27. McMurray JJV, Solomon SD, Inzucchi SE, Køber L, Kosiborod MN, Martinez FA et al. DAPA-HF Trial Committees and Investigators Dapagliflozin in patients with heart failure and reduced ejection fraction. N Engl J Med. 2019. Nov 21;381(21):1995-2008.
28. Vilsbøll T, Bain SC, Leiter LA, et al. Semaglutide, reduction in glycated haemoglobin and the risk of diabetic retinopathy. *Diabetes Obes Metab*. 2018;20(4):889–897.
29. Lingvay I, Desouza CV, Lalic KS et al. A 25 week randomized controlled trial of semaglutide once daily versus liraglutide and placebo in patients with type 2 diabetes suboptimally controlled on diet nad exercise with or without metformin. Diabetes Care. 2018;41(9): 1926-1937.

30. European Medicines Agency. Assessment Report Sotagliflozin. https://www.ema.europa.eu/en/documents/assessment-report/zynquista-epar-public-assessment-report_en.pdf
31. Peters AL, Buschur EO, Buse JB, Cohan P, Diner JC, and Hirsch IB. Euglycemic Diabetic Ketoacidosis: A Potential Complication of Treatment With Sodium-Glucose Cotransporter 2 Inhibition. Diabetes Care. 2015;38(9):1687-93.
32. Silverii GA, Decembrini L, Monami M, Mannucci E. Fournier's gangrene and sodium glucose co-transporter 2 inhibitors: A metanalysis of randomized controlled trials. Diabetes Obes Metab. 2019; 1-4.
33. Agiostratidou G, Anhalt H, Ball D, Blonde L, Gourgari E, Harriman KN, Kowalski AJ, Madden P, McAuliffe-Fogarty AH, McElwee-Malloy M, Peters A, Raman S, Reifschneider K, Rubin K, Weinzimer SA. Standardizing Clinically Meaningful Outcome Measures Beyond HbA(1c) for Type 1 Diabetes: A Consensus Report of the American Association of Clinical Endocrinologists, the American Association of Diabetes Educators, the American Diabetes Association, the Endocrine Society, JDRF International, The Leona M. and Harry B. Helmsley Charitable Trust, the Pediatric Endocrine Society, and the T1D Exchange. Diabetes Care. 2017 Dec;40(12):1622-1630.
34. Garg SK, Peters AL, Buse JB, Danne T. Strategy for mitigating DKA risk in patients with Type 1 diabetes on adjunctive treatment with SGLT-2 inhibitors: A STICH Protocol. Diabetes Technol Ther. 2018;20(9):571-575.

16. APPENDICES

16.1 Appendix 1 – Risk

Risks associated with trial interventions<br>☐ LOW ≡ Comparable to the risk of standard medical care<br>☒ MODERATE ≡ Somewhat higher than the risk of standard medical care<br>☐ HIGH ≡ Markedly higher than the risk of standard medical care				
Justification: Briefly justify the risk category selected and your conclusions below (where the table is completed in detail the detail need not be repeated, however a summary should be given):<br>An IDMC will be convened to monitor all SAEs.<br>Risks and mitigations associated with the intervention are outlined in more detail in the Protocol section 2.1.<br>Co-Sponsors will also carry out a detailed risk assessment of all aspects of the study as part of the approval process (SOP 04.013)				
What are the key risks related to therapeutic interventions you plan to monitor in this trial?		How will these risks be minimised?		
IMP/Intervention	Body system/Hazard	Activity	Frequency	Comments
S/C administration of semaglutide<br>Oral admistration of dapagliflozin	Immune: Hypersensitivity/ana-phylactic reactions	Patients with known hypersensitivity to any GLP-1 agonist or SGLT-2 inhibitor have a contra-indication to the IMP according to the SmPC will not be recruited to the study	Uncommon/ Rare	
PO administration of dapagliflozin	Ketosis and/or Diabetic Ketoacidosis (DKA)	All participants will have access to blood and urine ketone monitoring equipment. An emergency ketosis management plan (STICH Protocol – see above) will be discussed with all participants. Patient alert cards will be provided to all participants. Potential participants with recent ketoacidosis will be	Common	

		excluded from the trial (section 6.2)		
S/C administration of semaglutide (+/-) PO administration of dapagliflozin in addition to insulin therapy	Increased risk of hypoglycaemia	Participant awareness of risk and frequent testing of capillary blood glucose. Patients with pre-existing frequent episodes of hypoglycaemia or problems with hypoglycaemia awareness will be excluded from trial participation (section 6.2)	Common	

Outline any other processes that have been put in place to mitigate risks to participant safety (e.g. IDMC, independent data review, etc.)
See above

16.2 Appendix 2: Contraception

In line with recommendations related to contraception and pregnancy testing in clinical trials from the Clinical Trial Facilitation Group (CTFG), for women of childbearing potential* (WOCBP) participating in TTT1, methods of contraception considered as highly effective are defined as those that can achieve a failure rate of less than 1% per year when used consistently and correctly.

Such methods include:

- combined (oestrogen and progestogen containing) hormonal contraception associated with inhibition of ovulation[1]
  - oral
  - intravaginal
  - transdermal
- progestogen-only hormonal contraception associated with inhibition of ovulation[1]
  - oral
  - injectable
  - implantable[2]
- intrauterine device (IUD) [2]
- intrauterine hormone-releasing system (IUS)[2]
- bilateral tubal occlusion[2]
- vasectomised partner[2,3]
- true sexual abstinence[4]

Footnotes

1. An interaction between dapagliflozin and semaglutide with contraception is not anticipated.
2. Contraception methods that in the context of this guidance are considered to have low user dependency.
3. Vasectomised partner is a highly effective birth control method provided that partner is the sole sexual partner of the WOCBP trial participant and that the vasectomised partner has received medical assessment of the surgical success.
4. In the context of this guidance true sexual abstinence refers to: When this is in line with the preferred and usual lifestyle of the patient. Periodic abstinence (e.g. calendar, ovulation, symptothermal, post-ovulation methods), declaration of abstinence for the duration of a trial, and withdrawal are not acceptable methods of contraception.
The reliability of sexual abstinence needs to be evaluated in relation to the duration of the clinical trial and the preferred and usual lifestyle of the subject.

*<u>Child bearing potential</u> = experienced menarche; AND not undergone successful surgical sterilisation (hysterectomy, bilateral tubal ligation, or bilateral oophorectomy); AND not post-menopausal i.e. amenorrhea for ≥12 consecutive months (without another medical cause)

Source: Recommendations related to contraception and pregnancy testing in clinical trials. Clinical Trial Facilitation Group (CTFG), 15th September 2014.
See: https://www.hma.eu/fileadmin/dateien/Human_Medicines/01-About_HMA/Working_Groups/CTFG/2014_09_HMA_CTFG_Contraception.pdf (Accessed 21st January 2020)

16.3 Appendix 3

STICH Protocol *(also provided in Period 2 Participant Diary)*

As explained above, participants in this trial randomised to dapagliflozin are at an increased risk of ketosis and ketoacidosis. All participants will have access to a ketone meter as standard of care. It will be ascertained that each participant has access to a ketone meter; if there are any issues with their current ketone meter it will be replaced as per standard of care. There are mechanisms designed to mitigate the risk of early ketosis developing further into diabetic ketoacidosis. In this trial, we will be using a protocol called STICH (34). This is a mnemonic designed to act as a reminder for participants as to what to do in the event of elevated ketones following exposure to an SGLT-2 drug such as dapagliflozin:

1. STop dapagliflozin or placebo temporarily
2. Insulin – take a bolus of rapid acting insulin
3. Carbohydrate – take approximately 30g of carbohydrate
4. Hydrate – make sure you are taking sips of water aiming to maintain hydration.

Further guidance will be provided in both the Patient Diaries supplied to all participants and in the Patient Alert Cards that participants will be asked to carry at all times. All participants will also be advised that in the event of acute intercurrent illness or hospitalisation, dapagliflozin or placebo should be temporarily stopped. This is because intercurrent illness may additionally increase the risk of ketosis. Participants will likely already be familiar with this concept of "sick-day rules" as this is a key component of basic education for people with type 1 diabetes in clinical practice. These rules will however be re-iterated to participants.

More detailed guidance on actions participants should take for any given level of ketosis will be provided during face-to-face and telephone contact during the trial. In addition, however, the following written protocol will be provided to participants to advise them explicitly of the safest and most appropriate course of action to be taken in any given circumstance. This will take the form of a traffic light system to aid understanding and compliance.

1. Ketones 0.6 mmol/L or less: Continue - no need for additional bolus insulin.
2. Ketones 0.6 – 1.4 mmol/L: consider taking additional fast acting insulin. Follow sick day rules and monitor ketones 2 hourly until they are 0.6 mmol/L or less.
3. Ketones 1.5 – 2.9 mmol/L – Take 10% of your total daily dose of insulin (this is found by adding up all the long acting and short acting insulin for the previous day - only take a maximum of 10 units) as a bolus of fast acting insulin. Keep hydrated - aim for 100ml water per hour. Re-test your glucose and ketones in 2 hours. If not falling contact the study team during office hours. If you need urgent advice at other times please contact NHS 24 (telephone number 111) or attend your nearest Accident & Emergency Department.
4. Ketones above 3 mmol/L – Take 20% of your total daily insulin as fast acting insulin (Maximum 10 units). Hydrate with water, aim for 500 ml per hour. Attend A&E – High risk of DKA

If you develop DKA or are unwell it is important that you let the TTT1 nurse or doctor know about this as soon as you can. By following this protocol, the risk of ketosis and developing ketoacidosis will be mitigated.

16.4 Appendix 4: Amendment History

Amendment No.	Protocol version no.	Date issued	Author(s) of changes	Details of changes made

List details of all protocol amendments here whenever a new version of the protocol is produced. Protocol amendments must be submitted to the Sponsor for approval prior to submission to the REC committee or MHRA.

FULL/LONG TITLE OF THE TRIAL
Triple Therapy for Type 1 Diabetes with Insulin, Semaglutide and Dapagliflozin (TTT1) - UK

SHORT STUDY TITLE / ACRONYM
TTT1 (UK)

PROTOCOL VERSION NUMBER AND DATE
Version 2.0 7th Aug 2020

RESEARCH REFERENCE NUMBERS

**IRAS Number:**	254431
**EudraCT Number:**	2018-004120-11
**ISRCTN Number / Clinical trials.gov Number:**	TBC
**SPONSOR Number:**	GN17DI706
**FUNDER Number:**	3-SRA-2019-669-M-B

SIGNATURE PAGE

The undersigned confirm that the following protocol has been agreed and accepted and that the Chief Investigator agrees to conduct the trial in compliance with the approved protocol and will adhere to the principles outlined in the Medicines for Human Use (Clinical Trials) Regulations 2004 (SI 2004/1031), amended regulations (SI 2006/1928) and any subsequent amendments of the clinical trial regulations, Good Clinical Practice (GCP) guidelines, the Sponsor's (Standard Operating Procedures (SOPs), and other regulatory requirements as amended.
I agree to ensure that the confidential information contained in this document will not be used for any other purpose other than the evaluation or conduct of the clinical investigation without the prior written consent of the Sponsor
I also confirm that I will make the findings of the study publicly available through publication or other dissemination tools without any unnecessary delay and that an honest accurate and transparent account of the study will be given; and that any discrepancies from the study as planned in this protocol will be explained.

For and on behalf of the Study Co-Sponsor (University of Glasgow):
Signature: .... Date: ....../....../......
Name (please print): ....
Position: ....

For and on behalf of the Study Co-Sponsor (NHS Greater Glasgow and Clyde):
Signature: .... Date: ....../....../......
Name (please print): ....
Position: ....

UK Chief Investigator:
Signature: .... Date: ....../....../......
Name: (please print): John Petrie ....
Position: Professor of Diabetic Medicine..

Statistician:
Signature: .... Date: ....../....../......
Name: (please print): ....

Position: ....

KEY TRIAL CONTACTS

UK Chief Investigator	Professor John R Petrie BSc<br>Professor of Diabetic Medicine<br>Institute of Cardiovascular and Medical Sciences<br>BHF Glasgow Cardiovascular Research Centre<br>University of Glasgow<br>126 University Place<br>Glasgow G12 8TA<br>Secretary +44(0)141 330 3325<br>john.petrie@glasgow.ac.uk
UK Co- Investigator	Dr James Boyle<br>Consultant Diabetologist<br>Clinical Lead NHS GG&C Diabetes MCN<br>Diabetes UK Clinical Champion<br>Glasgow Royal Infirmary<br>84 Castle Street, Glasgow, G4 0SF<br>Tel: : +44(0)141 211 5996<br>james.boyle@glasgow.ac.uk
Trial Co-ordination	Miss Lisa Jolly<br>Research and Innovation<br>NHS Greater Glasgow & Clyde<br>Ward 11, Dykebar Hospital<br>Grahamston Road<br>Paisley, PA2 7DE<br>Lisa.Jolly@ggc.scot.nhs.uk
Co-Sponsor	NHS Greater Glasgow & Clyde<br><br>Contact: Dr Maureen Travers<br>Research and Development Management Office<br>Research and Innovation<br>NHS Greater Glasgow & Clyde<br>Ward 11, Dykebar Hospital<br>Grahamston Road<br>Paisley, PA2 7DE<br>Tel: +44 (0) 0141 314 4012<br>maureen.travers@ggc.scot.nhs.uk
Co-sponsor	University of Glasgow<br><br>Contact: Dr Debra Stuart<br>College of Medical, Veterinary & Life Sciences<br>Room 327 Wolfson Medical School Building<br>University Avenue<br>Glasgow G12 8QQ<br>Tel: +44 (0)141 330 4539<br>Debra.Stuart@glasgow.ac.uk

Funder	JDRF<br>26 Broadway, 15th floor, New York, NY 10004, USA
Clinical Trials Unit	Glasgow Clinical Trials Unit<br>Robertson Centre for Biostatistics, 11th Floor,<br>Boyd Orr Building, University of Glasgow<br>G12 8QQ<br>Tel: 0141 330 4744<br>liz.anderson@glasgow.ac.uk
Statistician	Professor Alex McConnachie<br>Robertson Centre for Biostatistics, 11th Floor, Boyd Orr Building, University of Glasgow, Glasgow, G12 8QQ<br>Telephone: 0141 330 4744<br>Fax: 0141-330-5094
Trials pharmacist	Dr Elizabeth Douglas<br>Clinical Trials Pharmacist<br>Clinical Research and Development<br>Ward 11, Dykebar Hospital,<br>Grahamston Road | Paisley PA2 7DE<br>Tel: 0141 314 4073<br>E-mail: elizabeth.douglas@ggc.scot.nhs.uk

US Principal Investigator	Dr Paresh Dandona<br>Director, Diabetes-Endocrinology Center of Western NY<br>Chief of Endocrinology, Diabetes, Endocrinology and Metabolism, SUNY at Buffalo<br>1000 Youngs Road, Suite 105<br>Williamsville NY 14221<br>Telephone: +1(716) 535-1850<br>Fax: +1(716)-639-1200<br>pdandona@kaleidahealth.org
US Co- Investigator	Dr Husam Ghanim, PhD.<br>Director, Diabetes and Endocrinology Research Lab<br>Research Associate Professor,<br>School of Medicine, State University of NY at Buffalo<br>Clinical and Translational Research Center<br>875 Ellicott St<br>Buffalo, NY 14203<br>Tel: (+1)716-881-8924

	ghanim@buffalo.edu
Key Protocol Contributors	Professor John Petrie<br>john.petrie@glasgow.acuk<br><br>Dr Paresh Dandona<br>pdandona@kaleidahealth.org<br><br>Husam Ghanim, PhD.<br>ghanim@buffalo.edu<br><br>Dr James Boyle<br>james.boyle@glasgow.ac.uk<br><br>Dr Robert Lindsay<br>robert.lindsay@glasgow.ac.uk<br><br>Dr Joseph Timmons<br>joseph.timmons@glasgow.ac.uk<br><br>Professor Ian Ford<br>ian.ford@glasgow.ac.uk<br><br>Professor Alex McConnachie<br>Alex.McConnachie@glasgow.ac.uk

TRIAL SUMMARY

Trial Title	Triple Therapy for Type 1 Diabetes with Insulin, Semaglutide, and Dapagliflozin (UK)	
Internal ref. no. (or short title)	TTT1	
Clinical Phase	Phase 3	
Trial Design	Two six-month periods: (i) open-label randomised (2:1) parallel group semaglutide vs standard of care; (ii) additional double-blind, randomised (1:1) parallel group, dapagliflozin vs placebo for those participants initially randomised to semaglutide.<br>Regardless of each randomisation, ongoing standard of care (insulin therapy) will be provided for all participants throughout the study.	
Trial Participants	Adults with type 1 diabetes for at least one year with HbA1c 7.5-11.0% on stable multiple daily injections (MDI) (four or more) of insulin or continuous subcutaneous insulin infusion (CSII) for the previous three months.	
Planned Sample Size	114 (57 in UK)	
Treatment duration	Approximately 1 year	
Follow up duration	Approximately 1 year	
Planned Trial Period	Approximately 3 years	
	Objectives	Outcome Measures
Primary	To assess in people with type 1 diabetes whether triple therapy (dapagliflozin, semaglutide and insulin) reduces HbA1c in comparison with dual therapy (placebo, semaglutide, and insulin – period 2).	HbA1c
Secondary	To assess in people with type 1 diabetes whether triple therapy (dapagliflozin, semaglutide and insulin) reduces HbA1c in comparison with standard therapy (insulin alone)	HbA1c
	To assess in people with type 1 diabetes whether dual therapy (semaglutide and insulin - period 1) reduces HbA1c in comparison with standard therapy (insulin alone)	HbA1c
	To assess in people with type 1 diabetes whether triple therapy (dapagliflozin, semaglutide and insulin) improves glucose control	- Mean fasting blood glucose<br><br>- Mean blood glucose*<br>- Percentage time in range

	as assessed by CGM, insulin requirement, body weight, blood pressure and quality of life in comparison with (a) dual therapy (placebo, semaglutide, and insulin – period 2) and (b) standard therapy (insulin alone)	- Glycaemic variability*<br>- Percentage time in hyperglycaemia Level 1 (10.0-13.9 mmol/)*<br>- Percentage time in hyperglycaemia Level 2 (>13.9 mmol/L)*<br>-Percentage time in hypoglycaemia Level 1 (3.0 – 3.8 mmol/L)*<br>** CGM (by masked Dexcom G6)*<br><br>Insulin requirement<br>Body weight<br>Systolic/ diastolic blood pressure<br>Number of antihypertensive agents<br>Diabetes Specific Quality of Life Scale (DSQOLS)<br>Problem Areas In Diabetes (PAID) questionnaire
Safety Endpoint 1	a) Triple therapy (dapagliflozin, semaglutide and insulin) in comparison with dual therapy (placebo, semaglutide, insulin – period 2)<br><br>b) Dual therapy (semaglutide and insulin - period 1) in comparison with standard therapy (insulin only)	Difference between treatment arms in:<br>(i) Rate of Level 2 (<3.0 mmol/L) hypoglycaemic events (by Dexcom G6 CGM)<br>(ii) Rate of Level 3 hypoglycaemic events (severe event with altered mental and/or physical status requiring assistance)
Safety Endpoint 2	a) Triple therapy (dapagliflozin, semaglutide and insulin) in comparison with dual therapy (placebo, semaglutide, and insulin – period 2)<br><br>b) Dual therapy (semaglutide and insulin –period 1) in comparison with standard therapy (insulin only)	Difference between treatment arms in:<br>Rate of diabetic ketoacidosis (serum ketones or urinary ketones above normal limits AND serum bicarbonate < 15 mmol/L OR blood pH < 7.3)
Investigational Medicinal Product(s)	Semaglutide<br>Dapagliflozin<br>Placebo matching dapagliflozin	
Non-investigational Medicinal Product(s)	Insulin	
Formulation, Dose, Route of Administration	Semaglutide (subcutaneous injection, once weekly) – open label by pen device (0.25 mg/ 0.5 mg/ 1.0 mg)<br>Dapagliflozin 5 mg (oral) – one then two capsules daily (uptitration)<br>Placebo matching dapagliflozin	

FUNDING AND SUPPORT IN KIND

FUNDER(S)	FINANCIAL AND NON FINANCIALSUPPORT GIVEN
JDRF New York<br>26 Broadway, 15th floor, New York, NY 10004, United States	$1.6M (to University at Buffalo, the State University of New York, NY (SUNY Buffalo), USA; and University of Glasgow, UK)
AstraZeneca<br>1800 Concord Pike, Wilmington, DE 19803, USA	Provision of dapagliflozin and matched placebo investigational medicinal products to US site
Dexcom; Suite 6, 1 Tanfield, Edinburgh, EH3 5DA	Staff training and discounted supply of CGM sensors

ROLE OF STUDY SPONSOR AND FUNDER

NHS Greater Glasgow & Clyde and The University of Glasgow will be Co-sponsors of the UK arm of the trial. Prior to study initiation, a non-commercially funded clinical trial co-sponsorship agreement will be put in place between NHS Greater Glasgow & Clyde and The University of Glasgow. The roles and liabilities each organisation will take under The Medicines for Human Use (Clinical Trials) Regulations, 2004 SI 2001:1031 are laid out in this agreement signed by both organisations. The University of Glasgow shall be responsible for carrying out the obligations and responsibilities set out in the aforementioned agreement, and shall be deemed "sponsor" for the purposes of, Part 3 of the regulations in relation to the study. NHS Greater Glasgow & Clyde shall be responsible for carrying out the responsibilities set out in the agreement, and shall be deemed "sponsor" for the purposes of, Parts 4, 5, 6 and 7 of the Regulations in relation to the study.

The Co-Sponsors will delegate specific roles to the Chief Investigator, Glasgow CTU and other third parties. These arrangements will be clearly documented in agreements and/or the Sponsor Delegated Roles and Responsibilities Matrix.

The US arm of the trial has the same inclusion and exclusion criteria and the same pre-specified outcomes but an individualised Protocol. It will collect the same data via a common eCRF. Adverse event data from each site will be available to the trial Data and Safety Monitoring Board. The UK Co-Sponsors will have no responsibility for (and carry no liability for) the conduct of the US arm of the trial.

<u>JDRF</u>
The study has been funded by a grant from JDRF. JDRF does not have a designated role or responsibility in trial design, conduct, data analysis and interpretation, manuscript writing, and dissemination of results. Periodic reports (at least annual) in relation to progress of the trial will be submitted to the JDRF. Support from JDRF will be acknowledged in any publications related to the study.

<u>Astra Zeneca</u>
This is an investigator-initiated study. Astra Zeneca provided support by supplying investigational medicinal products (IMPs) dapagliflozin and matched placebo to the US site only. Representatives of Astra Zeneca reviewed and provided comments on drafts of the Protocol but the company does not have a designated role or responsibility in trial design, conduct, data analysis and interpretation, manuscript writing, and dissemination of results.

ROLES AND RESPONSIBILITIES OF TRIAL MANAGEMENT COMMITTEES/GROUPS & INDIVIDUALS

Trial Steering Committee (TSC)

The role of the TSC is to provide overall supervision of the trial and ensure that it is being conducted in accordance with the principles of GCP and the relevant regulations. The TSC will:

1. agree the trial protocol and any protocol amendments
2. ensure that conduct of the trial is fully harmonised between the two sites
3. provide advice to the investigators on all aspects of the trial

It will have an independent Chairperson and include the US and UK investigators and key protocol contributors listed above.

Decisions about continuation or termination of the trial or substantial amendments to the protocol will be the responsibility of the TSC who will advise the co-sponsors. The TSC will meet at the start of the study, and six monthly thereafter. The TSC will have its own Charter outlining the role and responsibilities of its members. The TSC may invite other attendees from the trial team to present or participate in discussions on particular topics. These attendees will be non-voting members.

Independent data monitoring committee (IDMC)

An IDMC will be established to include two independent medical experts (covering the domains of type 1 diabetes and metabolic disease; one of the academic clinicians will act as chair) and an independent biostatistician. The Glasgow CTU will liaise with the committee and ensure that the Committee is provided with adequate information about study progress and results.

The IDMC will have a formal Charter outlining the responsibilities of the IDMC members, Glasgow CTU and the co-sponsors. Responsibilities include:

- To protect the safety of participants recruited to the trial.
- Advising the TSC and co-sponsors if it is safe and appropriate to continue with the study
- Examining information provided by the Glasgow CTU on study recruitment, adverse events and outcomes
- Providing reports for the Project Office to forward to the TSC, ethics committees, regulatory bodies, study co-sponsors, and the BHF.

The IDMC may recommend to the TSC to terminate the study or specific aspects of the study if clear harm is observed with one treatment option compared to standard of care or placebo. Specific safety endpoints that will be reviewed include rates of hypoglycaemia and ketosis/ ketoacidosis. The IDMC will not formally review efficacy or futility.

The IDMC will meet approximately every six months.

Study Management Group (SMG)

Trial activity at the UK/ Glasgow site will be coordinated from NHS Greater Glasgow & Clyde (NHS GG&C) by the TTT1 Study Management Group (SMG). The SMG will consist of the UK chief investigator, co-principal investigator other co-applicants, project manager and representatives from the Glasgow CTU, NHS GG&C and The University of Glasgow. The role of the group is to monitor all aspects

of the conduct and progress of the trial in the UK, ensuring adherence to the protocol, and take appropriate action to safeguard participants and the quality of the trial itself.

Joint Study Management Group

Coordination of trial activity between the UK/ Glasgow and the US/ Buffalo site will be facilitated by the Joint TTT1 Study Management Group. This will consist of the US and UK chief investigators, co-principal investigators, other co-applicants, study coordinators, project managers and governance/ administrative representatives as appropriate from State University of New York (SUNY), the Glasgow CTU, NHS GG&C and The University of Glasgow. It will meet approximately every 4-6 weeks. The role of the group is to coordinate all aspects of the conduct and progress of the trial between the US and UK sites, ensuring protocol adherence, and taking appropriate action to safeguard participants and the quality of the trial itself.

Protocol contributors

The protocol has been developed by a group with extensive clinical and research experience in type 1 diabetes and clinical trials including Professor John Petrie and Professor Paresh Dandona. Professor Ian Ford and Professor Alex McConnachie have research expertise in design, conduct, analysis and interpretation of clinical trials and biostatistical methods.

KEY WORDS:

Adjunct therapy
Dapagliflozin
Glycated haemoglobin (HbA1c)
Semaglutide
Type 1 diabetes

LIST of CONTENTS

RESEARCH REFERENCE NUMBERS 2
SIGNATURE PAGE 3
KEY TRIAL CONTACTS 4
TRIAL SUMMARY 7
FUNDING AND SUPPORT IN KIND 10
ROLE OF STUDY SPONSOR AND FUNDER 10
ROLES AND RESPONSIBILITIES OF TRIAL MANAGEMENT COMMITTEES/GROUPS & INDIVIDUALS 11
LIST of CONTENTS 14
LIST OF ABBREVIATIONS 15
TRIAL FLOW CHART 17
SCHEDULE OF ASSESSMENTS .... 17**Error! Bookmark not defined.**
1 BACKGROUND 19
2 RATIONALE 20
3 OBJECTIVES AND OUTCOME MEASURES/ENDPOINTS 23
4 TRIAL DESIGN 26
5 STUDY SETTING 26
6 ELIGIBILITY CRITERIA 26
7 TRIAL PROCEDURES 29
8 TRIAL MEDICATION .... **Error! Bookmark not defined.**
9 PHARMACOVIGILANCE 586
10 STATISTICS AND DATA ANALYSIS 66
11 DATA HANDLING 68
12 MONITORING, AUDIT & INSPECTION 69
13 ETHICAL AND REGULATORY CONSIDERATIONS 69
14 DISSEMINIATION POLICY 72
15 REFERENCES 731
16 APPENDICES 76

LIST OF ABBREVIATIONS

ACS	Acute Coronary Syndrome
AUC	Area Under the Curve
AE	Adverse Event
AR	Adverse Reaction
CA	Competent Authority
CABG	Coronary Artery Bypass Graft
CGM	Continuous Glucose Monitoring
CHI	Community Health Index
CI	Chief Investigator
CRO	Contract Research Organisation
CRP	C-Reactive Protein
CSII	Continuous Subcutaneous Insulin Infusion
CTA	Clinical Trial Authorisation
CTU	Clinical Trials Unit
CTIMP	Clinical Trial of Investigational Medicinal Product
CV	Cardiovascular
CVA	Cerebrovascular Accident
DKA	Diabetic Ketoacidosis
DSUR	Development Safety Update Report
EC	European Commission
ECG	Electrocardiogram
eCRF	Electronic Case Report Form
EDTA	Ethylenediaminetetraacetic acid
eGFR	Estimated Glomerular Filtration Rate
EMEA	European Medicines Agency
EU	European Union
EUCTD	European Clinical Trials Directive
EudraCT	European Clinical Trials Database
EudraVIGILANCE	European database for Pharmacovigilance
FBC	Full Blood Count
FGM	Flash Glucose Monitoring
GCP	Good Clinical Practice
GDG	Guideline Development Group
GDPR	General Data Protection Regulation
GLP-1	Glucagon Like Peptide-1
GLP-1RA	Glucagon Like Peptide-1 Receptor Agonist
GMP	Good Manufacturing Practice
HbA1c	Glycated haemoglobin
IB	Investigator Brochure
ICF	Informed Consent Form
ICH	International Conference on Harmonisation of technical requirements for registration of pharmaceuticals for human use.
IDMC	Independent Data Monitoring Committee
IMP	Investigational Medicinal Product
IMPD	Investigational Medicinal Product Dossier
ISF	Investigator Site File
ISRCTN	International Standard Randomised Controlled Trials Number

IWRS	Interactive Web-based Randomisation System
LPLV	Last Patient Last Visit
MA	Marketing Authorisation
MDRD	Modification of Diet in Renal Disease
MODY	Maturity Onset Diabetes of the Young
MHRA	Medicines and Healthcare products Regulatory Agency
MI	Myocardial Infarction
MDI	Multiple Daily Injections
NHS GG&C	National Health Service Greater Glasgow & Clyde
NHS R&D	National Health Service Research & Development
NICE	The National Institute for Health and Care Excellence
NIMP	Non-Investigational Medicinal Product
PI	Principal Investigator
PIC	Participant Identification Centre
PIS	Participant Information Sheet
PV	Pharmacovigilance
QA	Quality Assurance
QC	Quality Control
QoL	Quality of Life
QP	Qualified Person
RCT	Randomised Control Trial
REC	Research Ethics Committee
SAE	Serious Adverse Event
SAP	Statistical Analysis Plan
SAR	Serious Adverse Reaction
SDV	Source Data Verification
SGLT2	Sodium Glucose Transporter 2
SOP	Standard Operating Procedure
SUNY	State University of New York
SmPC	Summary of Product Characteristics
SSI	Site Specific Information
SUSAR	Suspected Unexpected Serious Adverse Reaction
TIA	Transient Ischaemic Attack
TMG	Trial Management Group
TSC	Trial Steering Committee
TMF	Trial Master File
UB	University of Buffalo
ULN	Upper Limit of Normal reference range
WOCBP	Women Of Childbearing Potential

TRIAL FLOW CHART

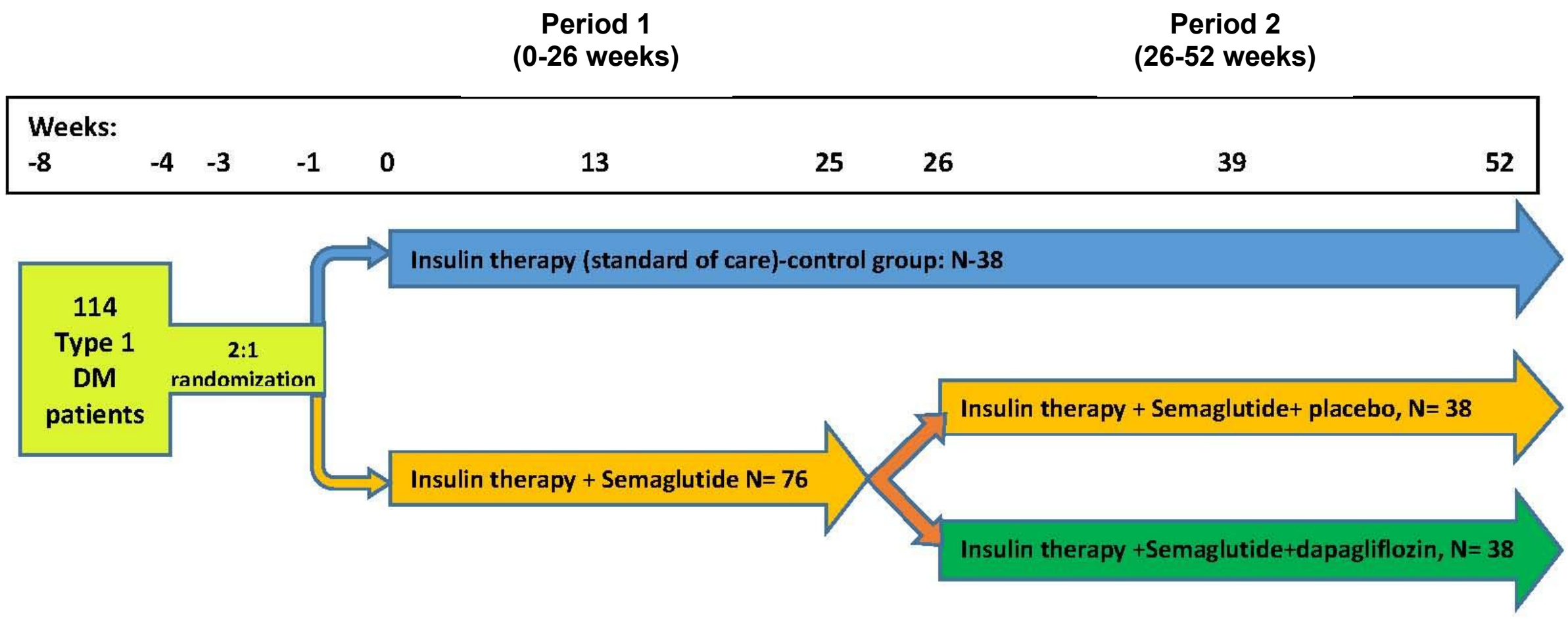

SCHEDULE OF ASSESSMENTS

**Visit #**	**-**	**1**	**2**	**3**	**4**	**5**	**6**	**7**	**8**	**9**	**10**	**11**	**12**	**13**	**14**	**15**	**16**
**Week# (±7 days; see below)**	**-8 to -4**	**-3**	**-1**	**0**	**1**	**2**	**4**	**13**	**23**	**25**	**26**	**27**	**31**	**39**	**49**	**51**	**52**
Study Procedures	Screening	Run-in period		Randomise							Randomise						End
Informed Consent	X																
Fasting visit			X	X				X		X	X			X		X	X
History and demographics	X																
Physical	X																X
Education on hypoglycaemia and ketosis avoidance)				X							X						
BP, heart rate, weight	X	X	X	X	X	X	X	X	X	X	X	X	X	X	X	X	X
Bloods (FBC, kidney and liver profiles)	X			X				X			X		X	X			X
C-peptide	X										X						X
Pregnancy test	X	X		X			X[1]	X[1]	X[1]		X[1]		X[1]	X[1]			X[1]
Dispense/ titrate open-label semaglutide[2]				X	X[2]	X[2]	X[2]	X[2]			X[2]			X[2]			
Dispense dapagliflozin/placebo											X	X		X			
Dapagliflozin/placebo return capsule counts												X		X			X
HbA1c	X			X				X			X			X			X
Blood endpoints (including fructosamine)				X				X			X			X			X
Masked CGM fit (enter data this visit)		X							X						X		
Masked CGM read				X							X						X
Daily fingerstick glucose review		X	X	X	X	X	X	X	X	X	X	X	X	X	X	X	X
Daily urinary ketones review												X	X	X	X	X	X
Insulin dose review		X	X	X	X	X	X	X	X	X	X	X	X	X	X	X	X
Ketone bodies (blood/urine)	X	X	X	X	X	X	X	X	X	X	X	X	X	X	X	X	X
Adverse Event reporting		X	X	X	X	X	X	X	X	X	X	X	X	X	X	X	X
Meal Challenge Test			X							X						X	
24 hr Urine dispensed			X				X			X			X			X	
24 hr Urine collected				X				X			X			X			X
Quality of life questionnaires				X							X						X

N.B. Safety phone calls will be conducted at weeks 3, 5, 8, 16, 20, 29, 35, 44 and at two weeks after the end of the study to document any side effects or other changes. **Abbreviations:** see pages 14-15

Visit dates should be within seven days of the visit time; however, a longer or shorter interval does not constitute a protocol deviation

[1]Not required in women randomised to "Insulin therapy (standard of care)

[2]According to randomisation schedule: resupply of open-label semaglutide after Visit 3 will be according to individual requirements as tolerated (see Section 8.3)

STUDY PROTOCOL
Triple Therapy for Type 1 Diabetes with Insulin, Semaglutide, and Dapagliflozin

1 BACKGROUND

The control of glucose homeostasis in type 1 diabetes is fragile since β-cell reserve is negligible. Traditionally, insulin has been the only treatment approach available. This often leads to erratic blood glucose control given the challenge of matching exogenous insulin to mealtime glucose excursions in the face of a lack of significant endogenous insulin to compensate for variation in requirements in response to meals and activity levels. Injected insulin boluses rarely match precise insulin requirements either in terms of dose administered or bio-availability. The result often is wide fluctuations in blood glucose including episodes when it is too low (hypoglycaemia). Based on our work to date and other published reports, previous attempts to add other glucose-lowering medications to insulin therapy yielded minimal benefits on glycaemia (although metformin may have other benefits) (1-3).

Dandona et al. have shown in two preliminary retrospective studies that the addition of liraglutide, a Glucagon Like Peptide -1 receptor agonist (GLP-1RA), to insulin results in consistent improvements in glycemic control in type 1 diabetes as demonstrated by reductions in HbA1c, glycemic excursions, and insulin requirements accompanied by improvements in body weight and systolic blood pressure (4, 5). As collaborators in the present trial, they also completed the first prospective randomised controlled trial of this drug in 72 people with type 1 diabetes. The data not only confirmed improvements in glycemic control (HbA1c), body weight and systolic BP, but also demonstrated a reduction in appetite, carbohydrate intake and low-grade inflammation as measured by plasma CRP concentrations (6). Importantly, quality of life was also improved. Their recent studies in type 2 diabetes have revealed that systolic blood pressure reduction with GLP-1RA (exenatide) treatment in type 2 diabetes is associated with increased plasma concentrations of ANP, cGMP and cAMP along with reduced concentrations of angiotensinogen, renin and angiotensin II (7). It is possible that these mechanisms play a role in the prevention of cardiovascular complications in type 1 diabetes. There have been no trials to date of semaglutide (a weekly and potent GLP-1RA) in type 1 diabetes.

GLP-1RAs, therefore, have great potential as adjunct therapy for type 1 diabetes. However, mean HbA1c was still ≥7.0% after liraglutide treatment (6) and larger pharma-sponsored trials (ADJUNCT-1 and ADJUNCT-2) reported increased rates of hypoglycaemia (8, 9). In seeking further improvement, the advent of Sodium-GLucose coTransporter 2 (SGLT2) inhibitors is of importance since their mode of action is independent of insulin secretion. Introduced for use in type 2 diabetes, SGLT2 inhibitors are selective antagonists of the sodium-glucose transporter subtype expressed in the proximal convoluted tubules of the kidneys. They act by inhibiting renal glucose re-absorption and therefore stimulating excretion of 60-100 grams per day of glucose in urine. SGLT2 inhibitors have been studied extensively in type 2 diabetes, including the landmark cardiovascular outcome trial EMPA-REG (10), but data have only more recently begun to accumulate in type 1 (11). Short term effects on urine glucose excretion of remogliflozin and empagliflozin are similar to those seen in type 2 diabetes and these translate into improved glycemic and other metabolic indices including HbA1c; fasting blood glucose, rates of hypoglycaemia, daily insulin dose and weight (12). Weight loss accompanying increased urinary glucose excretion could translate into reduced cardiovascular complications as in type 2 diabetes, but there are no data so far available to support this hypothesis. More recently, in the Phase 3 trials DEPICT-1 and DEPICT-2 Dandona et al (PI of the US arm of this trial) showed that dapagliflozin improves glycemic control and HbA1c over six months by about 0.45% without increasing hypoglycaemia. (13, 14, 15). In comparison to other SGLT2 inhibitors studied for use in type 1 diabetes [sotagliflozin and empagliflozin (16, 17)] the risk of diabetic ketoacidosis was relatively modest (see below). Both dapagliflozin and sotagliflozin were approved early in 2019 by the EU Committee for Medicinal Products for Human Use (CHMP) as adjunct therapy in type 1 diabetes for individuals with BMI≥27 kg/m^2.In August 2019, dapagliflozin (5 mg/ day) was recommended by the National Institute for Health and Care Excellence as cost-effective for this indication in NHS England (17, 18, 19).

2 RATIONALE

General hypothesis: adding an SGLT2 inhibitor (dapagliflozin) to a combination of insulin and semaglutide will improve glycaemic control, weight and blood pressure in adults with type 1 diabetes. Our protocol is designed to allow an objective assessment of whether this triple therapy combination will work effectively and safely so that a majority of people with type 1 diabetes can reach a glycemic target HbA1c of ≤7.0%. In addition, since both semaglutide and dapagliflozin inhibitors reduce blood pressure and weight, it will allow an assessment of the combined effect of GLP-1RAs and SGLT2 inhibitors on these outcomes. Since there are no previous data on the effects of once weekly semaglutide in type 1 diabetes, or the combination of semaglutide and dapagliflozin compared to standard insulin treatment, the design requires allocation of some participants to insulin therapy only (the current standard of care).

As part of the process by which this trial was awarded funding by JDRF, this research question was considered by lay members of the funding panel and deemed important, relevant and timely. We consider that use of placebo matching dapagliflozin in Period 2 of the trial is justified as although it has full approval as adjunct therapy for type 1 diabetes, it remains uncertain which adults with type 1 diabetes are suitable for this treatment. In their review, NICE considered it likely that fewer than 10% of adults with type 1 diabetes will be initiated on SGLT2 inhibitor therapy over the first five years after their favourable decision; this is the period of time during which this trial is being conducted. As individuals with a slightly lower BMI threshold will be included than recommended by the SmPC, trial participants will be more closely monitored for ketosis than is the case in routine clinical care. Dapagliflozin (or placebo) will be administered orally once daily as per current licensed doses in type 2 diabetes (5 mg/day and 10 mg/day: the same doses as used in DEPICT-1 and DEPICT-2) (13,14,15, 20). We have not incorporated blinding for semaglutide in the design as the effects of the closely-related daily GLP-1RA liraglutide have been well-studied in double-blind trials in type 1 diabetes by ourselves and others, and individuals in both arms compared for the primary outcome comparison will be treated with this agent. (6, 8, 9). Semaglutide will be administered subcutaneously once weekly.

2.1 Assessment and management of risk

a) Benefits

Semaglutide (Ozempic): is a once weekly injectable (subcutaneous) GLP1-RA licensed for reducing HbA1c in type 2 diabetes. The licensed doses are 0.25 mg weekly, 0.5 mg weekly and 1.0 mg weekly. In the trial, the starting dose is 0.25 mg weekly uptitrated if tolerated to 0.5 mg weekly after two weeks and then (again if tolerated) after a further two weeks to the maximum dose of 1.0 mg weekly. Semaglutide differs from other weekly GLP-1 receptor agonists in that its half-life is extended by binding to serum albumin i.e. it is not macromolecular. In type 2 diabetes, semaglutide therapy is associated with weight reduction and a reduction in rates of cardiovascular events (21). The licensed dose escalation schedule for routine treatment of type 2 diabetes is at four weekly intervals but in a Phase 2 trial in type 2 diabetes authored by one of this trial's investigators (JP) doses were uptitrated at one-to-two weekly intervals and to higher doses (up to 1.6 mg weekly) (22). Phase 3 trials are currently in progress (for the indication of obesity) using a 2.4 mg semaglutide weekly. It should be noted that semaglutide 0.25 mg is considered too low a dose for maintenance therapy in type 2 diabetes but it is unclear whether this may have a beneficial effect in type 1 diabetes at this dose.

While semaglutide once weekly has not been investigated previously in type 1 diabetes, liraglutide (a GLP-1 RA for daily administration with a similar molecular stucture that binds less avidly to serum albumin) has previously been investigated (see above) in the ADJUNCT 1 and ADJUNCT 2 trials (8,9) in which, compared with placebo it resulted in reductions in HbA1c, weight and insulin requirement, albeit with a small increase in rates of hypoglycaemia (see below).

Dapagliflozin (Forxiga): is a once daily oral SGLT2 inhibitor licensed in Europe for reducing HbA1c in people with type 1 diabetes and BMI ≥ 27 kg/m^2 when insulin alone does not provide adequate glycemic control despite optimal insulin therapy (23, 24). The licensed dose for type 1 diabetes is 5 mg once daily but both 5 mg and 10 mg daily are licensed for type 2 diabetes. In the Phase 3 trials of dapagliflozin in type 1 diabetes (DEPICT-1 and DEPICT-2) both doses were used. In both DEPICT-1 and DEPICT-2 there were modest additional benefits with 10mg compared to 5mg dapagliflozin. For example, in DEPICT-1, insulin dose requirement was reduced by 13.2% vs placebo on 10mg daily compared with 8.8% on 5mg daily (13). Weight reduction vs placebo in DEPICT-1 at 52 weeks was by 4.5% on dapagliflozin 10mg daily compared with 2.95% in those receiving 5mg daily. Given the propensity of insulin to encourage weight gain over time and the life-long insulin requirement in T1D, sustained weight reduction and insulin dose reduction - even when modest - may be clinically important. There was no increase in the rate of hypoglycaemia in either the 5mg or 10mg groups compared with placebo. (13,14) Dapagliflozin therapy in type 2 diabetes has been demonstrated to be associated with cardiovascular risk reduction: in both DECLARE-TIMI and DAPA-HF the 10mg dose was used. Such cardiovascular benefits may translate to type 1 diabetes. Given the potential glycaemic and cardiovascular benefits with relatively low increased risk, 10mg rather than 5mg dose was selected for steady state treatment in this protocol. (25,26)

b) Risks/ side effects Please also refer to the respective UK SmPCs (21, 24)

Semaglutide:
Very common: nausea, diarrhoea; hypoglycaemia (when used with insulin).
Common: vomiting, abdominal pain, constipation, gastro-oesophageal reflux, cholelithiasis, weight loss.
Uncommon: injection site reactions.

Hypoglycaemia/ ketosis: Semaglutide once weekly has not been studied previously in type 1 diabetes; however, the ADJUNCT-1 and ADJUNCT-2 trials examined the role of liraglutide once daily (a similar molecule in the GLP-RA class that binds less avidly to serum albumin). Although HbA1c and weight were reduced by both 1.2 mg and 1.8 mg liraglutide, rates of symptomatic hypoglycaemia increased by 20-30%. In ADJUNCT-1, insulin doses were re-uptitrated quite rapidly towards baseline levels after an initial dose reduction, but in ADJUNCT-2 were “capped” (and therefore remained lower). Perhaps for that reason, an increase in hypoglycaemia was less consistent in ADJUNCT -2 but there was an excess of ketosis the higher (1.8 mg) dose, presumably due to under-insulinisation (no lower limit for insulin dose reduction was set). It should be noted that monitoring of participants by site investigators in the ADJUNCT trials was less intensive than will be the case in the present trial with less frequent opportunities for insulin dose titration (see Section 7). Importantly, participants at high risk of hypoglycaemia, (including those with a history of severe hypoglycaemia and those with hypoglycaemia unawareness) were not excluded (8,9).

Retinopathy: In the SUSTAIN-6 cardiovascular outcome trial of semaglutide in type 2 diabetes, rapid reduction of HbA1c was associated with progression of existing proliferative retinopathy in those with a high baseline HbA1c (“early worsening”/ “normoglycaemic re-entry”). However, there was no evidence of worsening of retinopathy on treatment in the trials SUSTAIN 1-5 in which those with proliferative retinopathy were excluded (22). In clinical trials of duration up to one year involving 4,807 patients with type 2 diabetes, adverse events related to diabetic retinopathy were reported in similar proportions of subjects treated with semaglutide (1.7%) and comparators (2.0%) (21, 27).

Other adverse effects: In type 2 diabetes, gastrointestinal adverse effects of semaglutide can be prevented in the majority of cases by careful dose uptitration (21,22) although up to 5% are ultimately

unable to tolerate it. In our previous trial with liraglutide in T1D, adverse effects occurred with a similar frequency to that observed in T2D (in which there is extensive clinical experience); on this basis it is expected that this will also be the case with semaglutide (28).

Dapagliflozin:
Very common: hypoglycaemia (when used with insulin).

Common: ketoacidosis, genital infections (vulvovaginitis, balanitis), urinary tract infection, diabetic ketoacidosis,.

In the DEPICT-1 study, ketoacidosis occurred in 4 of 277 participants on 5mg dapagliflozin, 5 of 296 participants on 10 mg and 3 of 260 participants in the placebo group (summarised as 1%, 2% and 1% respectively) with one severe episode in each group (13). In pooled analyses of DEPICT-1 and DEPICT-2 (13,14) there was, however, an approximate threefold elevated rate of DKA adjudicated as "definite" over placebo on active therapy: 2.0% on 5mg dapagliflozin, 1.9% on 10mg and 0.6% on placebo – i.e. rates of DKA were similar with the two doses (13,14,15). Rates of DKA with sotagliflozin, the other SGLT2 inhibitor licensed for type 1 diabetes, were somewhat higher (2.9% on 200 mg, 3.8% on 400 mg, 0.2% on placebo) in pooled analyses of Phase 3 trials (29,30,31).

Uncommon: fungal infection, volume depletion, constipation.

Not known (several case reports): Fournier's gangrene.

Fournier's gangrene is a very rare clinical entity characterized by necrotizing fasciitis of the perineal area. This has been infrequently reported in association with SGLT-2 inhibitor use in both men and women. However, there was no evidence of increased risk in a meta-analysis of 84 clinical trials involving 42415 participants. Nevertheless, participants will be advised to seek urgent medical assistance if they experience erythema, pain, swelling of the perineal area associated with fever and malaise (this will be reinforced in the Patient Information Sheet). (32)

Amputation: An increased incidence of lower limb amputation has been reported in type 2 diabetes with the SGLT-2 inhibitor canagliflozin. However this has not been demonstrated with dapagliflozin (not included in the UK SmPC). People with diabetes are at increased risk of amputation and good foot care is a key component of good diabetes care regardless of use of SGLT-2 Inhibitors (24).

Placebo:
Placebo capsules contain microcrystalline cellulose.
This trial is therefore categorised by the Co-Sponsors as:

• Type B = Somewhat higher than the risk of standard medical care
See Appendix 1 (Risk Assessment)

3 OBJECTIVES AND OUTCOME MEASURES/ ENDPOINTS

3.1 Primary objective

- To assess in people with type 1 diabetes whether triple therapy (dapagliflozin, semaglutide and insulin) for six months improves glycaemic control (as measured by HbA1c) compared with dual therapy (placebo, semaglutide and insulin –phase 2).

3.2 Secondary objectives

To assess in people with type 1 diabetes whether:

- Triple therapy (dapagliflozin, semaglutide and insulin) improves glycaemic control (as measured by HbA1c) compared with standard therapy (insulin)
- Dual therapy (semaglutide and insulin – period 1) improves glycaemic control (as measured by HbA1c) in comparison with standard therapy (insulin only)
- Triple therapy (dapagliflozin, semaglutide and insulin) improves glycaemic control as assessed by a range of continuous glucose monitoring (CGM) parameters, lowers body weight, reduces insulin requirement, improves blood pressure and improves quality of life (as assessed by validated patient questionnaires) compared with dual therapy and standard therapy.

3.3 Outcome measures/endpoints

3.4 Primary endpoint/outcome

Change in HbA1c (week 26 to week 52)

3.5 Secondary endpoints/outcomes

Efficacy:
Change in HbA1c

Mean fasting blood glucose

Mean blood glucose*
Percentage time in range (3.9 - 10.0 mmol/)*
Standard deviation of blood glucose*
Percentage time in hyperglycaemia Level 1 (10.1 - 13.9 mmol/L/)*
Percentage time in hyperglycaemia Level 2 (>13.9 mmol/L) *
Percent time in hypoglycaemia Level 1 (3.0 - 3.8 mmol/)*
(by masked DEXCOM G6 CGM)*

Insulin requirement
Bodyweight
Systolic and diastolic BP
Number of antihypertensive medications
Diabetes Specific Quality of Life Scale (DSQOLS)
Problem Areas In Diabetes (PAID) questionnaire

Safety:
Rate of hypoglycemic events Level 2 (<3.0 mmol/L/ <54mg/dl)
Rate of hypoglycemic events Level 3 (severe event with altered mental and/or physical status requiring assistance)
Rate of diabetic ketoacidosis defined as elevated serum or urine ketones (greater than the upper limit of the normal range), and serum bicarbonate < 15 mmol/L or blood pH < 7.3

3.6 Tertiary endpoints/outcomes

Postprandial glucose (AUC) – meal tolerance test
Postprandial glucagon (AUC) – meal tolerance test
Postprandial FFA (AUC) – meal tolerance test
Postprandial GLP-1 (AUC) – meal tolerance test
Fructosamine

3.7 Exploratory endpoints/outcomes

Plasma and urine samples will be collected and frozen for later analysis for indices of inflammatory stress and other relevant measures.

3.8 Table of endpoints/outcomes

**Endpoints**	**Outcome Measures**	**Timepoint(s) of evaluation of this outcome measure**
**Primary endpoint**<br>Triple therapy (dapagliflozin, semaglutide and insulin) in comparison with dual therapy (placebo, semaglutide and insulin – period 2)	Change in HbA1c	Week 26 to Week 52
**Secondary endpoint 1**<br><br>Triple therapy (dapagliflozin, semaglutide and insulin) in comparison with standard therapy (insulin alone)	Change in HbA1c	Week 0 to Week 52
**Secondary endpoint 2**<br><br>Dual therapy (semaglutide and insulin – period 1) in comparison with standard therapy (insulin only)	Change in HbA1c	Week 0 to Week 26
**Other secondary endpoints**<br><br>a) Triple therapy (dapagliflozin, semaglutide and insulin) in comparison with dual therapy (semaglutide, insulin and placebo – period 2)<br><br>b) Dual therapy (semaglutide and insulin – period 1) in comparison with standard therapy (insulin only)	Change in:<br>- Mean fasting blood glucose (4 days)<br><br>- Mean blood glucose*<br>- Percentage time in range (3.9–10 mmol/L)*<br>- Glycaemic variability (SD)*<br>- Percent time in hyperglycaemia Level 1 (10-13.9 mmol/L)*<br>- Percent time in hyperglycaemia Level 2 (>13.9	Week 26 to Week 52<br><br>Week 0 to Week 26

	mmol/L)*<br>- Percent time in hypoglycaemia Level 1* (3.0-3.8 mmol/L)<br>*(*by masked Dexcom G6 CGM)*<br><br>Insulin requirement<br>Body Weight<br>Systolic and diastolic blood pressure<br>Number of antihypertensive medications<br>Diabetes Specific Quality of Life Scale (DSQOLS)<br>Problem Areas In Diabetes (PAID) questionnaire	
**Tertiary endpoints**<br>a) Triple therapy (dapagliflozin, semaglutide and insulin) in comparison with dual therapy (semaglutide, insulin and placebo –period 2)<br><br>b) Dual therapy (semaglutide and insulin – period 1) in comparison with standard therapy (insulin only)	Change in:<br>Postprandial glucose (AUC)<br>Postprandial FFA (AUC),<br>Postprandial GLP-1 (AUC)<br>Postprandial glucagon (AUC)	Week 26 to Week 52<br><br>Week 0 to Week 26
**Safety endpoint 1**<br>a) Triple therapy (dapagliflozin, semaglutide and insulin) in comparison with dual therapy (semaglutideinsulin and placebo – period 2)<br><br>b) Dual therapy (semaglutide and insulin – period 1) in comparison with standard therapy (insulin only)	Difference between treatment arms in:<br>(i) Rate of Level 2 (<3.0 mmol/L/ <54mg/dL) hypoglycemic events (by Dexcom G6 CGM)<br>(ii) Rate of Level 3 hypoglycemic events (severe event with altered mental and/or physical status requiring assistance)	Week 26 to Week 52<br><br>Week 0 to Week 26
**Safety endpoint 2**<br>a) Triple therapy (dapagliflozin, semaglutide and insulin) in comparison with dual therapy (semaglutide, insulin and placebo – period 2)	Difference between treatment arms in:<br><br>Rate of diabetic ketoacidosis (serum or urine ketones above normal limits) AND serum bicarbonate < 15 mmol/L OR blood pH < 7.3	Week 26 to Week 52

b) Dual therapy (semaglutide and insulin – period 1) in comparison with standard therapy (insulin only)		Week 0 to Week 26

4 TRIAL DESIGN

The trial has two six-month periods:

Period 1 open-label randomised (2:1) parallel group semaglutide once weekly + insulin (standard treatment) vs insulin alone (standard treatment).
Period 2 double-blind, randomised (1:1) parallel group, dapagliflozin vs placebo for those participants initially randomised to semaglutide (with ongoing standard of care for all participants)

Thus, during the first six months, approximately 114 participants with type 1 diabetes (57 in the UK) will be randomised (open label, see below) into three groups (approximately 38 each; i.e. 19 each in the UK) one of which will be maintained on their regular insulin therapy for 12 months (standard therapy group) and two of which will be started on semaglutide in addition to regular insulin treatment for six months (dual therapy). Following six months of stable treatment, those initially randomised to insulin and semaglutide will be additionally randomised (double-blind) to dapagliflozin (triple therapy) or matching placebo (dual therapy) for a further six months. The dose of dapagliflozin will not be increased to 10 mg in those who have confirmed ketonuria (+1) or ketonaemia (≥0.6 mM) after the first week of treatment.

In keeping with the intention-to-treat principle, participants in whom therapy is discontinued or downtitrated due to adverse effects will be encouraged to continue to undertake all study assessments and attend study visits.

5 STUDY SETTING

The study will be conducted at two sites: State University of New York (SUNY) at Buffalo, USA and Glasgow, UK. In Glasgow, n=57 participants (50% of the total) will be identified from secondary care sites and via the Scottish Diabetes Research Network research register. Both randomisations will be stratified by site.

6 ELIGIBILITY CRITERIA

6.1 Inclusion criteria

1) Type 1 Diabetes for at least one year on stable multiple daily (four or more) injections (MDI) of insulin or continuous subcutaneous insulin infusion (CSII) for the previous three months.
2) C-peptide < 0.23 nM
3) Minimum dose of insulin: 0.5 U/kg for MDI and 0.4 U/kg for CSII
4) Regularly measuring blood glucose: four or more times daily either by fingerstick, testing or by "flash"/ continuous glucose monitoring)
5) HbA1c 7.5 – 11.0 % inclusive
6) Well versed in carbohydrate1 counting*

7) Age 18-70 years.
8) BMI ≥25 kg/m^2.
** In the opinion of the site Principal Investigator*

6.2 Exclusion criteria

1) Type 1 diabetes for < 12 months, type 2 diabetes, chronic pancreatitis, MODY
2) Previous use of any agent other than insulin for glycaemic control in the last three months.
3) History of diabetic ketoacidosis (DKA) requiring medical intervention (e.g. emergency room visit and/or hospitalisation) within three months prior to the screening visit.
4) Following ketogenic diet, low (<50g/day) carbohydrate diet or intermittent fasting.
5) Hypoglycaemia unawareness* or frequent episodes of severe hypoglycaemia as defined by more than one episode requiring medical assistance, emergency care (paramedics or emergency room care), or glucagon therapy administered by a third-party individual within three months prior to the screening visit
6) Symptoms of poorly controlled diabetes
7) Participation in a weight loss program with ongoing weight loss, or in an intensive exercise program
8) History of bariatric or other weight-loss surgery within 12 months prior to the screening visit
9) History of Addison's disease or chronic adrenal insufficiency
10) History of diabetes insipidus
11) Hepatic disease or cirrhosis
12) Aspartate Aminotransferase (AST) > 3 X Upper limit of normal (ULN) and/or Alanine aminotransferase (ALT) > 3 X ULN
13) Serum Total Bilirubin > 2 X ULN unless exclusively caused by Gilbert's Syndrome
14) Haemoglobin < 110 g/L (11.0 g/dL) for men; haemoglobin < 100 g/L (10.0 g/dL) for women
15) Coronary event or procedure (myocardial infarction, unstable angina, coronary artery bypass, surgery or coronary angioplasty) in the previous three months or history of congestive heart failure
16) End stage renal disease on haemodialysis and/or eGFR < 60 ml/min/1.73m^2
17) HIV or Hepatitis B/C positive status
18) Any other life-threatening, non-cardiac disease
19) History of pancreatitis or cystic fibrosis
20) Women who are pregnant, planning pregnancy or breast-feeding and women of childbearing potential (WOCBP) who are not established on a method of contraception considered highly effective (see Appendix 2)and willing to continue it for the duration of the study and for at least two months after last dose of IMP.
21) Inability to give informed consent
22) History of gastroparesis
23) History of medullary thyroid carcinoma or MEN 2 syndrome
24) History of hypersensitivity reaction to GLP-1 agonists or SGLT2 inhibitors or known hypersensitivity to any of the excipients
25) Known gallstones (history of cholecystectomy is not an exclusion criterion)
26) History of alcohol problem or drug abuse
27) Hypertriglyceridemia (>500 mg/dl)
28) Recurrent genital mycotic infection (more than one episode in last six months)

29) Hypovolaemia
30) Any malignancy except treated *in situ* malignancy and basal cell carcinoma of the skin
31) Unexplained haematuria (dipstick positive on two occasions one week apart) and/or active bladder cancer
32) Proliferative retinopathy (on basis of photographic retinal screening which must have been performed within the last 12 months
33) Use of an investigational agent or therapeutic regimen within 90 days of study
34) Participation in any other concurrent interventional clinical trial
35) Unlikely to be able to comply with study requirements*

** In the opinion of the site Principal Investigator*

7 TRIAL PROCEDURES

Also see *Schedule of Assessments*

Each participant will be enrolled in the trial for approximately 55 weeks, comprising three weeks of monitoring to stabilise treatment and collect baseline data before randomisation into the intervention or standard of care/ placebo arms for 52 weeks. Following a completed screening visit and meeting the Inclusion and Exclusion criteria, each participant will have a total of 16 scheduled study visits. In addition, at weeks 3, 5, 8, 16, 20, 29, 35, 44 and at two weeks after the end of the study, each participant will receive a phone call to document any adverse effects or other changes. No long-term follow-up is planned following completion of participation in the study.

Participants will be treated with either insulin only (standard treatment) for the whole duration of the study or with insulin and semaglutide weekly s.c. injection (titrated where possible up to 1.0 mg, see below) for the first six month period and then randomised to either oral dapagliflozin (triple therapy) or placebo (dual therapy) daily added for the second six month period. Semaglutide will be started (Visit 4) at a dose of 0.25 mg once weekly for two weeks then increased if tolerated to 0.5 mg (Visit 5). If well tolerated at week 4, it will be increased to 1.0 mg once weekly (Visit 6) or at a subsequent visit at the discretion of the PI.Dapagliflozin (or placebo) will be started at a dose of 5 mg daily at week 26 and increased at week 27 to 10 mg (2 x 5mg capsules) daily to ensure drug tolerability and minimise the risk of insulin-induced hypoglycaemia or hypotension.

Excessive weight loss: Both investigation drugs are known to cause weight loss in the range of 5-10% in type 2 diabetes patients. However, the magnitude of weight loss following the combination of both drugs in type 1 diabetes patients may be significantly more. Excessive weight loss may lead to increased risk of hypoglycemia and ketosis or DKA (especially in the presence of dapagliflozin) and other unforeseen risks. Therefore, weight loss during any phase of the study will be limited to ≤15% baseline body weight or reaching a BMI of ≤22Kg/m^2. If participants are approaching this threshold, IMP will be sequentially reduced to avoid this outcome. Semaglutide may be reduced to 0.25mg or 0.5mg at the discretion of the PI. If weight loss continues despite this, dose reduction of dapagliflozin may be considered again at the discretion of the PI.

Covid-19 Mitigation

In view of the Covid-19 pandemic, certain trial procedures may be changed where strictly necessary to reduce in person contact with participants. This will be consistently reviewed in view of the prevailing circumstances at the time, in line with local and national guidance and with the principle of protecting participants and study personnel. In the event of a second wave of infection this would be particularly important. In this context, consideration would be given to rearranging visits, changing to virtual visits using video technology or using additional phone calls to ensure patient safety. Consideration will also be given to how we can minimise face to face interaction with participants when dispensing study medication to ensure their safety as best we can. If required, study medication to last for longer periods may be dispensed to participants to minimise risk. Courier services may additionally be used to deliver study medications to participants.

All visits in person will be subject to local and national guidance around the use of personal protective equipment including face masks and where appropriate when conducting procedures, the use of gloves and aprons.

Gastrointestinal adverse effects:

During the initial weeks of participation, those participants who report nausea and/or vomiting during uptitration of semaglutide dose that is not sufficiently severe to require treatment discontinuation will

remain on their current dose until they are symptom-free prior to discussing a further dose escalation (i.e. uptitration will be to the highest dose tolerated rather than a “forced titration”). Dose will be downtitrated to the maximum tolerated in those who experience gastrointestinal adverse effects.

Retinopathy:
As there was no excess of retinopathy with semaglutide treatment in Phase 3 trials with semaglutide, in which proliferative retinopathy was an exclusion criterion (as in the current protocol), additional retinal imaging beyond annual retinal photography (the standard of care) is not proposed (27).

Insulin dose adjustment:
Minimising hypoglycaemia: All participants will be made aware of the potential for increased hypoglycaemia with addition of adjunct therapy and supported and encouraged to monitor blood glucose at least four times daily in order to minimize the risk of hypoglycaemia. Participants will have ready access to advice from specialist medical and nursing staff at face-to-face and telephone visits. Those who have ongoing access to wearable continuous glucose monitoring [or "flash" glucose monitoring, (FGM)] will continue to use these devices. Information on management of hypoglycaemia will be included within participant diaries, reinforcing the pre-existing knowledge of participants, the majority of whom will have lived with type 1 diabetes for many years.

Reductions in insulin dose will not usually be greater than 10% (5% if HbA1c is < 7.0 %) from current levels. At randomisation, daily insulin dose will not be reduced by more than 20% from the baseline level and then from the new baseline levels at 26 weeks. Participants requiring an insulin dose reduction > 20% to a total insulin dose < 0.4 U/kg (current weight) will be monitored closely for insulin dose, carbohydrate intake, ketones and blood glucose. Following the second randomisation at 26 weeks, dapagliflozin/ placebo will be temporarily discontinued if insulin dose falls below this threshold and permanently discontinued if it remains so at review in one week.

Minimising ketosis: Participants will be instructed to stay hydrated and not to reduce their caloric intake (especially carbohydrates) or start new dietary approaches (especially ketogenic diets) during the study. They will be educated to recognize the signs and symptoms of diabetic ketoacidosis (DKA) and to undertake additional self-testing of urine and/or blood ketones if they develop nausea, vomiting, or anorexia. In addition, they will be given education and a written ketosis management plan (STICH Protocol – i.e. STop SGLT-2 inhibitor or placebo (temporarily), give Insulin bolus, take additional Carbohydrate, and additional Hydration - see Appendix 3).

From Visit 10 (26 weeks), those participants undergoing the second randomisation will be supported and encouraged to monitor urine or blood ketones at home at least once daily: three days before visit 10 (week 26), they will be asked to start daily measurements of urine ketones [CombiScreen® 10 SYS Plus urine testing strips, Sterilab, UK (or equivalent)] and blood ketones [CareSens™ Dual, i-SENS, Koreal; GlucoMen® Areo 2K, Menarini, UK (or equivalent]]. They will be asked to record these in the diary/ log provided. In addition, ketones (blood and urine) will be measured at every visit. If ketone bodies in urine are +1 or greater (≥0.6 mM in blood), participants will be reminded to keep well hydrated and notify the study team as soon as possible (office hours) as diabetic ketoacidosis (DKA) can occur even at glucose levels < 14 mmol/L (34).

If ketones are +2 or more in urine (> 1.5 mmol/L) participants will be advised to initiate the STICH Protocol immediately and contact the study team in office hours (or telephone NHS 24 or attend their Accident & Emergency if out of hours). This information will be reinforced verbally at study visits and also in the form of a Patient Alert Card that participants will be asked to carry. This will also contain details of the investigational study drug, the study number, the investigator’s name, and a contact number

(for in hours contact). The study team will assess for symptoms or test results indicating a risk of ketoacidosis immediately on reporting and if blood ketone levels are persistently (one week) at these levels dapagliflozin/placebo will be permanently discontinued (24).

If blood ketone readings are confirmed as > 3.0 mmol/L (or urine ketones reading +++) participants will be advised to stop dapagliflozin/ placebo and attend the Accident and Emergency department without delay.

Participants will be advised that dapagliflozin/placebo may be temporarily stopped in other clinical situations known to predispose to ketoacidosis (e.g. acute serious medical illnesses or hospitalisation for major surgical procedures). Any requirement for intravenous insulin infusion (whether in Accident and Emergency or as an in-patient admission) will be recorded as a Serious Adverse Event. For further detailed procedures see section 9.31 (25).

Volume depletion:
There is a theoretical risk of volume depletion and low blood pressure when dapagliflozin is used in combination with other anti-hypertensive drugs. All pre-existing concomitant medication will be reviewed at the screening visit as part of the medical history and examination (see Section 7.1.3 for further details). Participants undergoing the second randomisation will be educated on symptoms suggestive of low blood pressure (e.g. light-headedness, dizziness, feeling faint) and asked to contact the study team if they experience these symptoms. In clinical practice however SGLT-2 inhibitors are commonly used with anti-hypertensive drugs with no significant problems (27).

Genital mycotic infections:
Individuals who report symptoms suggestive of genital mycotic infection will be advised to self-treat with topical antifungal preparations. If a second episode occurs, a decision on treatment discontinuation will be considered in discussion between the participant and the site Principal Investigator, taking account of the severity of the symptoms.

Foot care:
Annual foot checks will be conducted for all participants as per standard of care.

7.1 Recruitment

7.1.1 Patient identification

All personal details will be managed according to ISO 27001:2013 compliant Standard Operating Procedures.

Potentially eligible participants will be identified from the Scottish Diabetes Research Network Research Register (a managed and searchable database of people with diabetes who have agreed to be contacted about clinical trial participation) and at secondary care diabetes clinics in Greater Glasgow and Clyde. The principal site for recruitment will be Stobhill Ambulatory Care Hospital but other sites may be opened if necessary as Patient Identification Centre sites. The majority of study visits (and all visits involving drug dispensing) will be conducted at the Clinical Research Facility, Glasgow Royal Infirmary. Information posters with the contact details of the research team will be displayed in recruiting clinics.

Clinic lists will be pre-reviewed by members of the usual care team in order that potentially eligible individuals can be sent an abbreviated Participant Information Sheet by post one week before their routine scheduled review visit. Eligibility criteria of those indicating agreement to be approached will

then be checked at the routine visit, and the study procedures explained. Potential participants will be given a Patient Information Sheet and an Expression of Interest Form (with prepaid envelope) at this time and will be asked for permission to contact again to discuss further and (if appropriate) arrange a screening visit.

Covid-19 Mitigation
The delivery of outpatient clinics has been substantially changed due to the pandemic. The use of telephone clinics is now standard and video technologies will soon also be used. Recruitment by necessity will therefore have to be done via telephone clinics and remotely using video technology. This may be subject to change depending on the situation with Covid-19. Other principles of recruitment will be as described above.

7.1.2 Screening

A separate non-fasting visit will be arranged at which potential participants will have further time to discuss with the study nurse and doctor.

Prospective participants will be asked to read and understand the Participant Information Sheet. Any questions regarding the Protocol will be answered. Risks and side-effects of the active trial medication will be explained. Eligibility for trial enrolment will be confirmed by a medical practitioner.

Individual wishing to participate in the study will then be invited to sign to document their informed consent (see Section 7.2 below).

7.1.3 Screening assessments

Items from the medical history relevant to the Inclusion and Exclusion criteria will be recorded. Demographic data, medical history and current medications (including current insulin doses) will be documented. All participants will have access to a dual blood glucose and ketone meter as per the contemporary standard of care in type 1 diabetes. A physical examination will be conducted. Clinical observations will be recorded and blood and urine samples obtained (see below):

<u>Demographics</u>

- Date of birth
- Sex
- Ethnicity
- Date of diagnosis
- Ethnic group: white/black/Asian/other
- Height

<u>Clinical assessments</u>

- Systolic and diastolic blood pressure (after 5 minutes rest)
- Heart rate
- Body weight (clothed without coat and shoes)
- Capillary blood glucose

<u>Medical History</u>

- Smoking status: current/ex/never
- Alcohol consumption: units/week
- Hypertension: Y/N
- Microalbuminuria: Y/N
- Proteinuria: Y/N
- Neuropathy: Y/N

- Previous major cardiovascular event(s): Y/N (date)
- Existing CV disease of any type*: Y/N
- Amputation above forefoot: Y/N
- Hospitalisation for DKA in last year
- Level of educational attainment: University (college) educated Y/N

* Existing CV Disease is defined as any of: MI, stroke, ACS/unstable angina, coronary stent/angioplasty, CABG, TIA, heart failure, peripheral vascular disease

<u>Insulin treatment</u>

- Regimen: MDI, pump,
- Total dose (all); Basal (longer acting) dose/ Bolus (shorter acting) dose (for MDI only)

<u>Other treatment (drug classes and names)</u>

Current use Y/N. If no, has there been use in last three months?: Y/N

- ACE inhibitors
- Angiotensin receptor blocker
- Calcium channel blocker
- Alpha-blocker
- Beta-blocker
- Loop diuretic
- Thiazide like diuretic
- Statin
- Aspirin
- Clopidogrel
- Novel anticoagulant
- Warfarin

<u>Blood assessments</u>:

- Blood ketones (from participant's own ketone meter)
- HbA1c
- FBC
- Urea and electrolytes
- Creatinine
- Liver function tests (AST/ ALT/ Alkaline Phosphatase)
- Bone profile (Calcium/ Phosphate)
- C-peptide (capillary blood glucose must be > 4.0 mmol/L at time of sampling)

If blood tests have been assessed for clinical purposes within the last three weeks, these values will be used.

<u>Urine sample</u>:

- Urine ketones
- For all WOCBP, a urine pregnancy test will be checked at screening

Potential participants meeting all the inclusion and exclusion criteria based on history, examination and screening tests will be enrolled in the study.

Pregnancy: WOCBP will be asked about pregnancy status and contraceptive usage (see Appendix 2) and a pregnancy test will be conducted (following informed consent) prior to randomisation. We will not recruit those who are pregnant, wanting to become pregnant, or using birth control methods not considered highly effective. Every four weeks following randomisation, WOCBP randomised to IMP (as opposed to standard of care) will be asked about their contraception status, and a urine pregnancy test carried out. Study medication will be permanently discontinued in women who become pregnant or considered to be on contraception that is not highly effective. As the study assessments *per se* do not carry an additional risk over standard of care to either mother or unborn child, further pregnancy tests will not be routinely offered following randomisation to those women allocated to "Insulin therapy (standard of care)" (i.e. who are not exposed to IMP). All pregnancies will be notified to the Sponsor Pharmacovigilance Officer using the standard pregnancy notification form and the pregnancy followed to outcome).

7.1.4 Payment

Participants will be offered reasonable travel expenses for visits additional to usual care.

7.2 Consent

Potential participants will be identified and screened by the Inclusion and Exclusion criteria listed above. Medical staff or appropriately trained support staff will seek consent for screening and participation in the trial from individuals that fulfil the criteria. All potential participants will have at least 24 hours to review the Patient Information Sheet before being approached (see Section 7.1.2 above). Following written consent, each signature will be dated by the signatory, the original retained in the site file, a copy provided to the participant and a copy scanned into the participant's Clinical Portal record.

Data collected for routine clinical care will be used for clinical trial documentation (e.g. blood results within the previous three weeks). In the absence of routine blood results, sampling of blood for study specific laboratory measurements can only be performed following documentation of informed consent.

7.2.1 Re-screening post-consent

Where inclusion (e.g. HbA1c ≤7.5%) or exclusion (e.g. DKA within the last three months) criteria are not met (e.g. blood results falling outside the study parameters) potential participants can be re-screened with additional blood tests on one further occasion. If the initial consent was signed within two months of the re-testing, new consent is not required unless the consent form template has been updated in this period. If the initial consent was not signed within two months of re-testing then consent should be sought again. Rescreened participants will keep the same 5-digit Patient ID and data will be amended on the web portal as appropriate.

7.3 Run-in period

7.3.1 Visit 1 (@3 weeks prior to randomisation)

Participants who meet entry criteria will be scheduled for their next visit at approximately three weeks prior to randomisation. Daily fingerstick blood glucose levels (study diaries), CGM/ FGM data or insulin pump download data (as applicable) will be reviewed and appropriate insulin dose adjustments made by the

medical team to target fasting blood glucose concentrations between 5 -10.0 mmol/L if possible at all times without having additional hypoglycaemia (< 3.9 mmol/L). Clinical observations will be recorded including resting BP measurement. Blood glucose and ketone bodies will be measured via a fingerstick blood sample (see Section 7). Urine for pregnancy status and ketone bodies will be assessed.

Participants will be instructed on the use of the Continuous Glucose Monitoring System (Dexcom G6 with blinded receiver). All subjects will be advised to monitor their capillary blood glucose by CGM or fingerstick at least 4 times a day (before meals and at bedtime) throughout the study and to wear their CGM constantly for next 20 days (changing the sensor at 10 days). CHO counting technique will be reviewed and appropriate support given with calculating bolus insulin doses.

Visit procedures:

Monitoring and treatment optimisation

- Check for adverse events
- Review blood glucose diary
- Blood ketones (from participant's own ketone meter
- Adjust insulin doses (if necessary)

Clinical assessments

- Blood pressure
- Heart rate
- Weight

Urine sample:

- Check urine ketones

Other

- *Start* Continuous Glucose Monitoring with Dexcom G6

7.3.2 Visit 2 (1 @ weeks prior to randomisation)

Fasting visit* - Participants will be asked to fast (no food or calorie intake) for 10 hours prior to the visit overnight. Those who plan to drive to fasting visits will be reminded of the increased risk of hypoglycaemia and DVLA guidelines. This will be repeated prior to each fasting visit and reiterated within the patient diaries. This visit will be scheduled for a morning appointment and participants will be advised not to take their breakfast bolus insulin dose prior to arrival. If participants arrive at the study visit in a non-fasted state, the visit will be terminated and a further fasting visit rearranged as soon as practicably possible.

Daily fingerstick blood glucose levels (diaries), CGM/ FGM data or insulin pump download data (as applicable) will be reviewed and appropriate insulin dose adjustments made. Clinical observations will be recorded as detailed below. Blood glucose and ketone bodies will be measured via a fingerstick blood sample (see Section 7). If blood glucose is less than 3.9 mmol/L, or more than 13.9 mmol/L, the visit will be rescheduled for an alternative date. Otherwise, an intravenous cannula will be placed in order to obtain blood samples for the duration of the visit. A Meal Challenge Test will be performed (described below). A 24 hr urine container will be dispensed to collect a 24 hour urine sample to bring

in at the Week 0 visit. For this collection and all subsequent 24 hour urine collections, urine will be tested via dipstick testing and an aliquot retained for assay of albumin, creatinine, ketones, and 15-isoprostane.

Visit Procedures

Monitoring and treatment optimisation

- Check for adverse events
- Review blood glucose diary
- Blood ketones (from participant's own ketone meter
- Adjust insulin doses (if necessary)

Clinical assessments

- Blood pressure
- Heart rate
- Weight

Urine sample:

- Check urine ketones
- 24 hour urine container dispensed

Other assessments

- Download Dexcom G6 CGM
- High fat high calorie Meal Challenge: in order to assess postprandial metabolism a Meal Challenge Test will be performed using an approximately 900 kilocalorie High Fat High Carbohydrate (HFHC) meal including an egg muffin sandwich, a sausage muffin sandwich and two hash browns [containing approximately 88g carbohydrate, 51 g fat (33% saturated) and 34 g protein]. Participants will be asked to attend fasting prior to breakfast insulin bolus (with continuing basal insulin); participants will be asked not to take bolus insulin until the 3 hour time point unless blood glucose values reach above 22.2 mmol/L. Blood samples will be collected from an indwelling intravenous canula in a superficial forearm vein for glucose, glucagon, FFA, GLP-1 (AUC). Sequential blood samples will be obtained at 0, 15, 30, 45, 60, 90, 120, 150 and 180 min after consuming the meal. Volume of samples at 15, 30, 45, 90 and 150 min will be 5ml while those at 0, 60, 120 and 180min will be 30 ml (total volume = 145 ml). A spot urine sample will be collected at baseline, 1 and 3hr for standard urinalysis and an aliquot retained for assay of ketones and 15-isoprostane.

7.4 Randomisation

Participants who are being randomised will be required to have undergone all screening assessments.

At the randomisation visit, study participants will be provided with a Patient Alert Card. There are two Versions: (1) for the intervention arm i.e. those allocated at first randomisation to open-label semaglutide who are also eligible for the second double-blind randomisation (dapagliflozin or placebo); and (2) for those allocated to standard of care. These cards supplement the study diaries and include contact information for the study team. Version 1 contains unblinding information. Participants will be asked to carry their card at all times. These will be collected at the end of participation in the study.

7.4.3 First Randomisation Scheme

For the first randomisation (Period 1, open label), eligible and consenting participants will be randomised (2:1, stratified by site) to the two groups (open-label semaglutide or standard of care) using permuted blocks of variable size to minimize predictability.

7.4.4 Method of implementing the allocation sequence

Randomisation will be achieved by accessing an interactive web-based randomisation system (IWRS). The investigator will provide the participant identifier and the system will check the participant's eligibility from information already entered in the electronic Case Report Form (eCRF): if appropriate the randomisation group will be allocated.

7.5 Visit 3 (First Randomisation Visit)

Participants will be asked to attend in the fasting state (no food or calorie intake for 10 hours; see 7.3.2 above). Blood glucose levels and ketone bodies will be measured via a fingerstick blood sample using a glucose and ketone meter (see Section 7) and daily fingerstick blood glucose levels (diaries) collected and reviewed. The particpant's own CGM/ FGM data or insulin pump download data will also be reviewed. Daily insulin dose will be adjusted following clinical review of study diaries: if reduced, this should not be by more than 20% from the baseline level Participants will discontinue use of the study masked CGM (Dexcom G6). Clinical observations will be recorded. Blood glucose and ketone bodies will be measured via a fingerstick blood sample (see Section 7). The 24 hour urine container will be collected. A fasting blood sample will be taken (45 mL) to evaluate study endpoints, FBC, U&E, LFT, bone profile, lipids, HbA1c, and fructosamine (detailed below). A urine sample will be collected to evaluate ketone bodies and, in females, pregnancy status.

Participants will be asked to complete Quality of Life questionnaires (see Appendices).

IMP will be dispensed for those randomised to open-label semaglutide (once weekly). Semaglutide will be started on site at the 0.25 mg dose. Participants will be instructed to call site staff directly in case of any problem or untoward side effects. An office hours phone number will be provided for advice. Outside these hours, provision of emergency medical care if required will be via NHS 24 and/or Accident & Emergency. Participants will be specifically asked to call if they have hypoglycaemia (blood glucose < 3.9 mmol/L) or Level 2 hyperglycaemia (blood glucose >13.9 mmol/L), particularly if occurring on more than one occasion. This will apply for the remainder of the trial.

Visit Procedures

Fasting visit* as per visit 2.

<u>Monitoring and treatment optimisation</u>

- Check for adverse events
- Check adequacy of contraception (WOCBP)
- Review blood glucose diary
- Adjust insulin doses (if necessary)
- Blood ketones (from participant's own ketone meter)

<u>Clinical assessments</u>

- Blood pressure
- Heart rate
- Body weight

Quality of life assessments

- Diabetes Specific Quality of Life Scale (DSQOLS)
- Problem Areas In Diabetes (PAID) questionnaire

Blood assessments

- Na, K, urea, creatinine, eGFR (MDRD)
- AST/ ALT/ Alkaline Phosphatase
- Calcium/ Phosphate
- CRP
- HbA1c
- Fructosamine
- Haemoglobin
- White cell count
- Platelets
- Bilirubin
- Albumin
- Random glucose

Urine

- For WOCBP, a urine pregnancy test will be checked prior to first administration of IMP.
- Urine ketones
- 24 hour urine sample collected

Medication

- Administer open label semaglutide 0.25 mg if randomised to active treatment
- Issue Patient Alert Card (appropriate version according to randomisation)

Bloods for storage

Approximately 45 mls of venous blood will be withdrawn and collected in tubes containing EDTA. Blood will be centrifuged at 1500g for 20 mins at 4°C. Plasma will be siphoned, aliquoted and stored locally at -80° ± 10°C until transport in batches to the central laboratory on dry ice.

7.5.1 Visit 4 (@1 week after first randomisation)

Clinical observations will be recorded. Blood glucose levels and ketone bodies will be measured via a fingerstick blood sample using the participant's own glucose and ketone meter (see Section 7)) and daily blood glucose levels collected and reviewed. Insulin doses adjusted as in visit 3. If HbA1c is <7.0 %, insulin dose reduction should not be >5% from baseline total dose. Semaglutide 0.25mg will again be injected on site for those randomised to active therapy.

Visit Procedures

Monitoring and treatment optimisation

- Check for adverse events
- Review blood glucose diary
- Adjust insulin doses (if necessary)
- Blood ketones (from participant's own ketone meter

Assessments

- Blood pressure
- Heart rate
- Weight

Urine sample:
- Urine ketones

Medication
- Support participant's first self-injection of open label semaglutide 0.25 mg if randomised to active treatment

7.5.2 Visit 5 (@2 weeks after first randomisation)

Clinical observations will be recorded. Blood glucose levels and ketone bodies will be measured via a fingerstick blood sample using glucose and ketones meter (see Section 7) and daily blood glucose levels collected and reviewed. Daily blood glucose data will be reviewed and insulin doses adjusted as per Visit 3. If participants randomised to semaglutide are experiencing any nausea, they will continue on the 0.25 mg dose for another week (or more as required) before increasing the dose. Otherwise semaglutide 0.5mg dose will be injected on site. Supply of IMP will be dispensed as appropriate to last until the next visit.

Visit Procedures

Monitoring and treatment optimisation
- Check for adverse events
- Review blood glucose diary
- Adjust insulin doses (if necessary)
- Blood ketones (from participant's own ketone meter)

Assessments
- Blood pressure
- Heart rate
- Weight

Urine sample:
- Urine ketones

Medication
- Dose titration (see above) if randomised to open-label semaglutide
- Return used study pen
- Dispense medication if randomised to open-label semaglutide

7.5.3 Visit 6 (@4 weeks after first randomisation)

For those randomised to open-label semaglutide, doses administered will be recorded (self-report). Clinical observations will be recorded. Daily blood glucose data will be reviewed and insulin doses adjusted as per visit 3. Supply of IMP will be given to last until the next visit. A urine sample will be collected to evaluate ketone bodies and, in females randomised to IMP, pregnancy status. Unless experiencing nausea, the recommended dose will be increased to 1.0 mg a day. A 24 hr urine container will be dispensed to collect a 24 hour urine sample to bring in at the Week 13 visit.

Visit Procedures

Monitoring and treatment optimisation

- Check for adverse events
- Check adequacy of contraception (WOCBP)
- Review blood glucose diary
- Adjust insulin doses (if necessary)
- Blood ketones (from participant's own ketone meter)

Assessments

- Blood pressure
- Heart rate
- Weight

Urine sample:

- Urine ketones
- Urine pregnancy test (WOCBP randomised to IMP only)
- 24 hour urine container dispensed

Medication

- Record doses of semaglutide self-injected since last visit (self-report)
- Dose titration if randomised to open-label semaglutide (see above)
- Return used study pens
- Dispense medication if randomised to open-label semaglutide

7.5.4 Visit 7 (@13 weeks after first randomisation)

Participants will be asked to attend in the fasting state (no food or calorie intake for 10 hours). For those randomised to open-label semaglutide, doses administered will be recorded (self-report). Daily fingerstick blood glucose levels (diaries), CGM/ FGM data or insulin pump download data will be reviewed and appropriate insulin dose adjustments made. Participants will discontinue use of the study CGM (Dexcom G6). Supply of IMP will be given to last until visit 10. Observations will be recorded. A urine sample will be collected to evaluate ketone bodies and, in females randomised to IMP, pregnancy status. The 24 hour urine container will be collected. A fasting blood sample will be taken (45 mL) to evaluate study endpoints.

Visit Procedures

Fasting visit*

Monitoring and treatment optimisation

- Check adequacy of contraception (WOCBP)
- Check for adverse events
- Review blood glucose diary
- Adjust insulin doses (if necessary)
- Blood ketones (from participant's own ketone meter)

Clinical assessments

- Blood pressure
- Heart rate
- Weight

Blood assessments

- Na, K, urea, creatinine, eGFR (MDRD)
- AST/ ALT/ Alkaline Phosphatase
- Calcium/ Phosphate
- CRP
- HbA1c
- Fructosamine
- Haemoglobin
- White cell count
- Platelets
- Bilirubin
- Albumin
- Random glucose

Urine

- Pregnancy test (WOCBP randomised to IMP only)
- Urine ketones
- 24 hour urine bottle collected

Medication

- Record doses of semaglutide self-injected since last visit (self-report)
- Return used study pens
- Dispense medication if randomised to open-label semaglutide

7.5.5 Visit 8 (@23 weeks after first randomisation)

For those randomised to open-label semaglutide, doses administered will be recorded (self-report). Clinical observations will be recorded. Blood glucose levels and ketone bodies will be measured via a fingerstick blood sample. A urine sample will be collected to evaluate ketone bodies and, in females randomised to IMP, pregnancy status. Insulin dose will be adjusted if needed. All subjects will be reminded to monitor their capillary blood glucose by fingerstick at least 4 times a day (before meals and at bedtime) throughout the study. CGM sensor will be fitted and participants asked to wear their CGM constantly for next 20 days.

Visit Procedures

Monitoring and treatment optimisation

- Check adequacy of contraception (WOCBP)
- Check for adverse events
- Review blood glucose diary
- Blood ketones (from participant's own ketone meter)
- Adjust insulin doses (if necessary)

Clinical assessments

- Blood pressure
- Heart rate
- Weight

Urine sample:

- Pregnancy test (WOCBP randomised to IMP only)

- Urine ketones

Other assessments
- *Start* Continuous Glucose Monitoring with Dexcom G6

7.5.6 Visit 9 (@25 weeks after first randomisation)

Participants will be asked to attend in the fasting state (no food or calorie intake for 10 hours). Daily fingerstick blood glucose levels (diaries), CGM/ FGM data or insulin pump download data will be reviewed and appropriate insulin dose adjustments made. Participants will discontinue use of the study CGM (Dexcom G6). Clinical observations will be recorded. Blood glucose levels and ketone bodies will be measured via a fingerstick blood sample (see Section 7) Supply of IMP will be given to last until the next visit. A 24 hr urine container will be dispensed to collect a 24 hour urine sample to bring in at visit 10 (42pprox.. Week 26) visit. A Meal Challenge Test will be performed as per visit 2. Participants will be provided with strips for urine and blood ketone measurement at home and instructed or reminded as appropriate on how to use these. They will be asked to measure urinary and blood ketones every morning for three consecutive days prior to the next visit (second randomisation: dapagliflozin or placebo) and to record the results in their diary.

Visit Procedures

Fasting visit*

Monitoring and treatment optimisation
- Check adequacy of contraception (WOCBP)
- Check for adverse events
- Review blood glucose diary
- Adjust insulin doses (if necessary) - **this insulin dose is baseline for Period 2**
- Blood ketones (from participant's own ketone meter)

Clinical assessments
- Blood pressure
- Heart rate
- Weight

Urine sample:
- Urine ketones
- 24 hour urine container dispensed

Other assessments
- Download Dexcom G6
- Meal Challenge Test

7.6 Second Randomisation Scheme

For the second randomisation (Period 2, double-blind), eligible and consenting participants previously randomized to open-label semaglutide will be randomised (2:1) to two groups (dapagliflozin or matching placebo) using permuted blocks of variable size to minimize predictability with stratification for HbA1c (above vs below 7.0 %). Those participants previously randomised to standard of care will continue as before.

7.6.1 Blinding (period 2 only)

For the double-blind treatment period, dapagliflozin and placebo will be packed in matching packs to maintain the blind. Each pack will contain sufficient supplies for 30 days' treatment with a small overage (excess). Packs will be labelled with a unique number that will be used to assign treatment to the participant via the IWRS system whilst maintaining the blind. Packs will be labelled in accordance with Good Manufacturing Practice and local regulatory requirements.

Unblinding. Treatment discontinuation (of dapagliflozin or placebo), rather than unblinding, will be carried out as far as possible. Participants will be advised in any case of hospitalisation with acute illness to discontinue study medication and inform the relevant clinician. These instructions will be reinforced in Study Diary Version 1. However, where knowledge of treatment may assist emergency treatment, unblinding will be supported. Information on how to unblind in an emergency (the investigational study drug, the study number, the investigator's name, a contact number, and a freephone number) will be provided on the Patient Alert Card. Several prompts on the telephone menu system warn the user that they require to be a health professional and to record their name and other pertinent information. For each unblinding an email alert is generated to the Study Coordinator and Chief Investigator. The most likely scenarios for unblinding will be: confirmed pregnancy, overdose/ accidental ingestion, or development of acute renal failure.

7.6.2 Visit 10 (Second Randomisation Visit; @26 weeks after First Randomisation)

Participants will be asked to attend in the fasting state (no food or calorie intake for 10 hours). Participants with normal fasting urine (<+) or blood ketone (<0.6 mmol) in the three days prior to randomisation will proceed to the next treatment phase as per this visit. The principles of the STICH Protocol (see Appendix 3) will be discussed. Participants with higher ketone values in at least one of the previous three days will have their glucose levels, insulin dose, and carbohydrate intake reviewed and advised on hydration, adequate CHO intake, and adequate insulin dosing. They will be rescheduled for one week and asked to repeat three days testing before the next visit.

Clinical observations will be recorded. A urine sample will be collected for ketones. Blood glucose and ketones will be measured via a fingerstick blood sample (see Section 7.2.1). Daily fingerstick blood glucose levels (diaries), CGM/ FGM data or insulin pump download data will be reviewed and appropriate insulin dose adjustments made (following the guidance in Section 7, page 35). The previous three days blood and urine ketones levels will be reviewed. The adjusted total insulin dose at this visit will serve as the baseline total insulin dose for the remaining six months of the study and future limits of dose adjustments will be based on this new baseline. The blinded study CGM will be collected. The 24 hour urine container will be collected. A fasting blood sample will be taken (45 mL) to evaluate study endpoints. Participants will be asked to complete quality of life questionnaires.

At this point, those participants previously randomised to receive open-label semaglutide will be randomised to receive dapagliflozin 5 mg daily or matching placebo. They will be educated on symptoms suggestive of low blood pressure (e.g. light-headedness, dizziness, feeling faint).

Participants will be asked to perform a daily (preferably early morning) urine (using CombiScreen® 10 SYS Plus urine testing strips or equivalent) and blood tests for ketone bodies (using the particpant's own meters as per Section 7, "Minimising ketosis": page 28) and record all outcomes in dedicated study diaries. If results of these scheduled (i.e. not done due to symptoms) ketone measurements, are +1 or higher (urine) or > 0.6 mmol/L (blood), participants will be instructed to repeat the measurement after 60 min. Participants will be instructed to call site staff directly as soon as possible within office hours if

these results are persistent, . For any abnormal ketone levels taken due to symptoms, the DKA consensus guidelines are to be followed instead (see Section 9.3).

Participants will be instructed to call site staff directly in case of any problem or untoward side effects. They will be specifically asked to call if they have confirmed hypoglycaemia (blood glucose levels < 3.9 mmol/L) or hyperglycaemia (blood glucose > 13.9 mmol/L) occurring on more than one occasion. Participants will be advised to remain well hydrated and report any genital mycotic or urinary tract infections

Visit Procedures

Fasting visit* - Requirements and arrangements as per visits 2 and 3.

Monitoring and treatment optimisation

- Check adequacy of contraception (WOCBP)
- Check for adverse events
- Review blood glucose diary
- Adjust insulin doses (if necessary)
- Blood ketones (from participant's own ketone meter)

Clinical assessments
- Blood pressure
- Heart rate
- Body weight

Quality of life assessments
- Diabetes Specific Quality of Life Scale (DSQOLS)
- Problem Areas In Diabetes (PAID) questionnaire

Blood assessments
- Na, K, urea, creatinine, eGFR (MDRD)
- AST/ ALT/ Alkaline Phosphatase
- Calcium/ Phosphate
- CRP
- HbA1c
- Fructosamine
- Haemoglobin
- White cell count
- Platelets
- Bilirubin
- Albumin
- Random glucose

Urine
- Pregnancy test (WOCBP randomised to IMP only)
- Urine ketones
- 24 hour urine bottle collected

Medication

- Education on symptoms suggestive of low blood pressure (e.g. light-headedness, dizziness, feeling faint) for those undergoing second randomisation
- Record doses of semaglutide self-injected since Visit 7 (self-report)
- Return used study pens
- Dispense open label semaglutide for participants randomised to active treatment at Period 1 (if required).
- Dispense dapagliflozin or placebo if randomised to active treatment

<u>Other assessments</u>

- *Read* Continuous Glucose Monitoring sensor (mean fasting blood glucose, mean blood glucose, percentage time in range, glycaemic variability)
-

7.6.3 Visit 11 (@1 week after Second Randomisation)

For those randomised to open-label semaglutide, doses administered will be recorded (self-report). Clinical observations will be recorded. Daily blood glucose levels and urinary ketone measurements will be reviewed. A urine sample will be collected to evaluate for ketones bodies. Blood glucose levels and ketones bodies will be reviewed measured via a fingerstick blood sample (see Section 7.2.1). . Daily fingerstick blood glucose levels (diaries), CGM/ FGM data or insulin pump download data will be reviewed and appropriate insulin dose adjustments made using the new baseline from Visit 10 (following the guidance in Section 7, page 35). If at any time total insulin dose reduction is >20% and total insulin dose is <0.40 U/Kg, additional monitoring (see Appendix 2) will be implemented. Unless blood (>0.6 mmol/L) or urine ketones (+ or more) have been elevated within the previous three days, dapagliflozin or placebo dose will be uptitrated to 10 mg daily (two capsules of either active or placebo medication) for the remainder of the study. Supply of IMPs will be given to last until week 39.

<u>Visit Procedures</u>

<u>Monitoring and treatment optimisation</u>

- Check adequacy of contraception.
- Check for adverse events
- Review blood glucose diary
- Adjust insulin doses (if necessary)
- Blood ketones (from participant's own ketone meter)
- Daily urine ketones review

<u>Urine sample</u>:

- Urine ketones
- Pregnancy test (WOCBP randomised to IMP only)

<u>Medication</u>

- <u>Count returned dapagliflozin or placebo capsules</u>
- Increase dose of dapagliflozin or placebo to 10mg (see above – review ketones)
- <u>Dispense dapagliflozin or placebo</u>

7.6.4 Visit 12 (@5 weeks after Second Randomisation)

For those randomised to open-label semaglutide, doses administered will be recorded (self-report). Clinical observations will be recorded. Daily blood glucose levels and urinary ketones measurements will be

collected and reviewed. Blood glucose levels and ketone bodies will be measured via a fingerstick blood sample. A urine sample will be collected to evaluate ketone bodies and if appropriate pregnancy status. Insulin dose will be adjusted if as per Visit 11. A blood sample will be taken (15 mL) to evaluate study endpoints. A 24 hr urine container will be dispensed to collect a 24 hour urine sample to bring in at the Week 39 visit

Visit Procedures

Monitoring and treatment optimisation

- Check adequacy of contraception (WOCBP)
- Check for adverse events
- Review blood glucose diary
- Adjust insulin doses (if necessary)
- Blood ketones (from participant's own ketone meter)

Clinical assessments

- Blood pressure
- Heart rate
- Weight

Blood assessments

- Na, K, urea, creatinine, eGFR (MDRD)
- AST/ ALT/ Alkaline Phosphatase
- Calcium/ Phosphate
- CRP
- Haemoglobin
- White cell count
- Platelets
- Bilirubin
- Albumin
- Random glucose

Urine

- Pregnancy test (WOCBP randomised to IMP only)
- Urine ketones
- 24 hour urine container dispensed

7.6.5 Visit 13 (@13 weeks after Second Randomisation)

For those randomised to open-label semaglutide, doses administered will be recorded (self-report). Participants will be asked to attend in the fasting state. Clinical observations will be recorded. Daily blood glucose levels and urinary ketones measurements will be collected and reviewed. Blood glucose levels and ketone bodies will be measured via a fingerstick blood sample. A urine sample will be collected to evaluate ketone bodies and if appropriate pregnancy status. The 24 hour urine container will be collected. Insulin dose will be adjusted if needed as in as per Visit 11.. Sufficient supply of IMPs will be given until the end of the study. A fasting blood sample will be taken (45 mL) to evaluate study endpoints.

Visit Procedures

Fasting visit* - as per previous fasting visits

Monitoring and treatment optimisation

- Check adequacy of contraception (WOCBP)
- Check for adverse events
- Review blood glucose diary
- Adjust insulin doses (if necessary)
- Blood ketones (from participant's own ketone meter)
- Daily urine ketones review

Clinical assessments

- Blood pressure
- Heart rate
- Weight

Quality of life assessments

- Diabetes Specific Quality of Life Scale (DSQOLS)
- Problem Areas In Diabetes (PAID) questionnaire

Blood assessments

- Na, K, urea, creatinine, eGFR (MDRD)
- AST/ ALT/ Alkaline Phosphatase
- Calcium/ Phosphate
- CRP
- HbA1c
- Fructosamine
- Haemoglobin
- White cell count
- Platelets
- Bilirubin
- Albumin
- Random glucose

Urine

- Pregnancy test (WOCBP randomised to IMP only)
- Urine ketones
- 24 hour urine bottle collected

Medication

- Record doses of semaglutide administered since Visit 10 (self-report)
- Dispense open label semaglutide for participants randomised to active treatment at Period 1 (if required).
- Count returned dapagliflozin or placebo capsules
- Dispense dapagliflozin or placebo as required

7.6.6 Visit 14 (@23 weeks after Second Randomisation)

For those randomised to open-label semaglutide, doses administered will be recorded (self-report). Clinical observations will be recorded. Daily blood glucose levels and urinary ketones measurements will be collected and reviewed. Blood glucose levels and ketone bodies will be measured via a fingerstick blood sample. A urine sample will be collected to evaluate ketone bodies and if appropriate pregnancy status. Insulin dose will be adjusted if as per Visit 11. All subjects will be reminded to monitor their capillary blood glucose by fingerstick at least four times a day (before meals and at bedtime). CGM (Dexcom G6) sensor will be fitted and patients asked to wear their CGM constantly for the next 20 days.

Visit Procedures

Monitoring and treatment optimisation
- Check adequacy of contraception (WOCBP)
- Check for adverse events
- Review blood glucose diary
- Adjust insulin doses (if necessary)
- Blood ketones (from participant's own ketone meter)
- Daily urine ketones review

Clinical assessments
- Blood pressure
- Heart rate
- Weight

Urine sample:
- Pregnancy test (WOCBP randomised to IMP only)
- Urine ketones

Clinical and functional assessment
- *Start* Continuous Glucose Monitoring sensor (mean fasting blood glucose, mean blood glucose, percentage time in range, glycaemic variability)

7.6.7 Visit 15 (@25 weeks after Second Randomisation)

Participants will be asked to attend in the fasting state. For those randomised to open-label semaglutide, doses administered will be recorded (self-report). Observations will be taken. Daily blood and urinary ketones measurements will be collected and reviewed. Blood glucose levels and ketone bodies will be measured via a fingerstick blood sample. A urine sample will be collected to evaluate urine ketone levels. Insulin dose will be adjusted if needed as per visit 11. A 24 hr urine container will be dispensed to collect a 24 hour urine sample to bring in at the Week 52 visit. A HFHC Meal Challenge Test will be performed as in Visit 2.

Visit Procedures

Fasting visit* - Procedure as per previous fasting visits

Monitoring and treatment optimisation
- Check adequacy of contraception (WOCBP)

- Check for adverse events
- Review blood glucose diary
- Adjust insulin doses (if necessary)
- Daily urine ketones review
- Blood ketones (from participant's own ketone meter)

Assessments
- Blood pressure
- Heart rate
- Weight

Urine sample:
- Pregnancy test (WOCBP randomised to IMP only)
- 24 hour urine container dispensed

Other assessments
- Meal Challenge Test

7.6.8 Visit 16 (End of study; @26 weeks after Second Randomisation)

Participants will be asked to attend in the fasting state. Clinical observations will be recorded. Daily blood glucose levels and urinary ketones measurements will be collected and reviewed. Blood glucose levels and ketone bodies will be measured via a fingerstick blood sample. Insulin doses will be reviewed and likely requirement for an increase following discontinuation of IMP discussed with those taking open-label semaglutide. A urine sample will be collected to evaluate ketone bodies and, if appropriate pregnancy status. The CGM will be collected. The 24 hour urine container will be collected. A fasting blood sample will be taken (45 mL) to evaluate study endpoints. We will ask the participants to complete quality of life questionnaires. A physical examination will be performed as per the screening visit. This will be the final face-to-face study visit. A safety phone call will be conducted two weeks to review blood glucose data, provide further insulin dosing advice, to document any adverse events, and to ensure that follow-up in usual clinical care has been arranged.

Visit Procedures

Fasting visit*

Monitoring and treatment optimisation
- Check for adverse events
- Review blood glucose diary
- Adjust insulin doses (if necessary)
- Blood ketones (from participant's own ketone meter)
- Daily urine ketones review

Clinical assessments
- Blood pressure
- Weight

- Heart rate
- Physical examination

Quality of life assessments

- Diabetes Specific Quality of Life Scale (DSQOLS)
- Problem Areas In Diabetes (PAID) questionnaire

Blood assessments

- Na, K, urea, creatinine, eGFR (MDRD)
- AST/ ALT/ Alkaline Phosphatase
- Calcium/ Phosphate
- CRP
- HbA1c
- Fructosamine
- Haemoglobin
- White cell count
- Platelets
- Bilirubin
- Albumin
- Random glucose

Urine

- Pregnancy test (WOCBP randomised to IMP only)
- Urine ketones
- 24 hour collection

Other

- Record doses of semaglutide administered since Visit 13 (self-report)
- Return used study pens
- Count returned dapagliflozin or placebo capsules
- Collect Patient Alert Card

7.7 Retention and strategies for maximizing follow-up

Participants will be encouraged to attend all study visits. Prior to commencing the trial, all potential participants will have the opportunity to read the detailed Patient Information Sheet which will outline the level of participation requested. Potential participants will be made aware of the need for frequent study visits and asked to commit to these.

Study visits can be rearranged flexibly within a period around the planned visit as specified in the timeline of visits above (Section 7) and actual dates recorded in the eCRF. This is to maximise participant ability to attend all visits. A longer or shorter interval does not constitute a protocol deviation. Where possible, visits will be arranged to coincide with other appointments to further maximise attendance and participant

convenience. Telephone contact will be frequent and will help to maintain contact with participants and ensure timely reminders of upcoming study visits can be given.

Even in the scenario of treatment discontinuation (of either IMP), participants will be encouraged to continue to attend further study visits as scheduled.

7.8 Treatment Interruptions and Withdrawal criteria

Withdrawal from study drug

Treatment interruption from study IMPs may be necessary in some participants. In the case of dapagliflozin, this will occur in any acute illness or hospitalisation which increases the risk of ketosis and ketoacidosis. Circumstances in which temporary discontinuation of dapagliflozin or placebo should occur will be discussed with all study participants. Written information will be provided in the study diaries. An emergency phone line to the study team will be available to all participants for the duration of their participation in the trial. This will be operational during office hours (Monday to Friday 9am – 5pm). Patient Alert Card Version 1 will also contain unblinding information in the event of acute intercurrent illness and a requirement for unblinding in the opinion of the acute medical team treating the participant. If any participant develops diabetic ketoacidosis (DKA) as defined as per the International Consensus guidelines on two occasions with no identifiable precipitant (section 2.1) they will be withdrawn from study medication and the UK Chief and US Principal Investigators informed.

Any other intolerable side effects or acute illness may require temporary discontinuation of the study drugs (dapagliflozin or semaglutide). In these circumstances, if the study drugs cannot be safely restarted in the judgement of the trial physician or if participants are unable to tolerate side-effects, they may be withdrawn from study treatment.

Treatment withdrawal may be necessary during the trial prior to the planned end at 52 weeks following first randomisation for any of the following reasons:

1. The site PI can stop investigational study treatment or remove participants from the trial without participants approval if for any reason he/she feels is appropriate, including: severe side effects, injury or medical condition which may place the patient at risk of further complications if he/she continue to participate, failure to take the medication as instructed, failure to keep scheduled appointments, insufficient level of self-monitoring, cancellation of the study or other administrative reasons.

2. Any woman who becomes pregnant during the period of study will be withdrawn from study treatment.

3. Participants who experience persistent severe nausea or vomiting after starting semaglutide which is not managed by dose reduction will discontinue the investigational study treatment. Those with mild or moderate nausea will continue for at least one more week. For those who continue to experience significant nausea (defined as interfering with normal activities), dose will be reduced: if these side effects persist, they will discontinue study medication(s). Participants for whom this occurs in Period 1 will remain eligible for randomisation in Period 2.

4. New diagnosis of other conditions included in the Exclusion Criteria

5. Change in method of insulin delivery between insulin pumps to MDI or vice versa during the study unless pump treatment interruption is temporary for a period of at most 2 weeks. Participants who

upgrade to “artificial pancreas” (e.g. Medtronic’s MiniMed 670G hybrid) during the study will be withdrawn.

6. Hypoglycaemia: Two episodes of severe hypoglycaemia (requiring external intervention).

7. Ketosis (with no other identifiable precipitating factors): Persistent (1 week) asymptomatic or symptomatic and unmanaged (using STICH protocol – see section 2.1 b) blood ketone levels >1.5 mmol (or ≥+2 in urine).

8. Persistent (>1 week) total insulin dose reduction of >20% from baseline AND total insulin dose <0.4U/Kg.

9. DKA: Two episode of DKA (with no identifiable precipitating factors) as defined by the expert consensus statement (34)

10. Two episodes of UTI.

11. Two episodes of mycotic genital infection.

12. Significant persistent reduction in eGFR > 15 ml/min

13. Pancreatitis: any episode judged to be due to pancreatitis by the site PI.

14. Other persistent side effects specific to study drugs

Participation in this study is voluntary. Participants have the right to refuse to participate or to withdraw from participation at any time for any reason. The site PI can stop investigational study treatment or remove participants from the trial without participants approval. If a patient withdraws consent and decide not to participate in the study, she/he will be asked to undergo a final study visit for his/her safety. If a subject withdraws from the research, the data collected to that point will be used toward the research finding. If applicable the subject will be asked to return any unused research drug and or devices.

Withdrawal from the study

If participants are unable or unwilling to attend all study visits they will be given an option of attending less frequently or only at the end of the study. We will request participants’ consent to be contacted by telephone and for contact with their general practitioner or other health care provider to check on their current health status. No participant will be labelled as lost to follow-up. However, participants will have the right to withdraw consent for further participation and for further data collection: all other participants will be followed up for clinical events until study completion.

7.9 Storage and analysis of samples

Blood samples will be undertaken at the beginning and at various points during the study as outlined in detail in the schedule of visits. If blood testing has recently been undertaken in the 3 weeks prior to the study commencing, some of these blood tests may be used as baseline. Blood tests will include analysis of the full blood count, renal function (urea and electrolytes), liver function tests, bone profile, lipid profile and several specialised blood tests for biomarker assays (at central laboratory) following a high fat high caloric meal challenges to examine study endpoints.

Covid-19 Mitigation

All blood sampling or trial procedures will be undertaken following local and national guidance around personal protective equipment. Mask, gloves and aprons will be used as per current guidelines.

7.9.1 Sample collection and processing

- Samples will be appropriately labelled in accordance with the trial procedures to comply with the General Data Protection Regulation (GDPR). Biological samples collected from participants as part of this trial will be transported, stored, accessed and processed in accordance with national legislation relating to the use and storage of human tissue for research purposes and such activities shall at least meet the requirements as set out in the 2004 Human Tissue Act and the 2006 Human Tissue (Scotland) Act.
- Blood will be taken according to the Schedule of Assessments (page 17). Plasma will be siphoned, aliquoted and stored locally at -80° ± 10°C until transport in batches to the central laboratory on dry ice. according to the Sample Handling Procedure as agreed by the Joint Study Management Group
- Individual patient samples will be identified with a unique, anonymised study number.

7.9.2 Sample transport to central laboratory and analysis

- Samples will be transferred to State University of New York in a single batch at the end of recruitment to the study. More frequent transfer can be organised if there are local storage limitations.
- Samples will be transported on dry ice and stored at the central laboratory at -80° ± 10° until analysis. Transport by courier will coordinated by the Trial Manager.
- At the time of analysis individual aliquots will be defrosted at room temperature and analysed in a single batch for each biomarker of interest.
- Samples will be stored at -80° ± 10° in the central laboratory for possible future analysis for novel biomarkers.

7.10 End of trial

The end of the trial will be following the last study visit of the last subject.
At the end of the specified trial period (52 weeks following first randomisation) all participants will discontinue study medications and revert to standard of care (insulin therapy only). This process will be supervised by the trial physicians, research nurses, and research pharmacists under the supervision of the site Principal Investigator. The trial team will liaise with the usual care team to ensure a safe transition back to standard therapy. Following the final scheduled trial visit there will be a safety phone call two weeks after the last visit. This will act as a further opportunity to advise on insulin dosing and ensure there are no ongoing problems or concerns related to the study.

As discussed in section 7.8, there may be circumstances in which trial drugs need to be stopped before the specified end of the trial. If this is the case, the reason(s) for discontinuation will be recorded and the trial clinical team will facilitate safe transition back to usual standard of care. Participants will however

if eligible (i.e non-exclusion criteria reason for IMP discontinuation) be asked to continue with trial visits to enable ongoing close clinical observation. If consent is withdrawn and participants voluntarily chose to leave the trial before the specified end date, all efforts by the clinical trial team will be made to safely facilitate transition back to ordinary standard of care. A follow up safety visit will be offered to all such participants and ongoing contact details will be made available should there be any further unforeseen issues relating to the clinical trial.

The trial may be stopped at an earlier stage than anticipated on the advice of the independent data monitoring committee (IDMC) to the trial steering committee (TSC) for the safety reasons detailed on page 12 and in the study safety endpoints. If this occurs, all participants will be informed and safe discontinuation of study medications will be undertaken under the supervision of the site Principal Investigator.

8 TRIAL MEDICATION

8.1 Name and description of investigational medicinal product(s)

a) Semaglutide (Ozempic®): a once weekly GLP-1 agonist injection indicated for type 2 diabetes (approved by the Scottish Medicines Consortium, January 2019).

   Doses to be used: 0.25mg increased to 0.5mg at two weeks and then to 1mg weekly s.c. injection after a further two weeks. Open label semaglutide will be added to a background of insulin treatment at week 0 for 12 months in 76 participants (38 in the UK) according to the first randomisation; at week 26 they will be further randomised (double-blind) as below into two groups: dual (placebo, semaglutide and insulin) and triple (dapagliflozin, semaglutide and insulin) therapy.

b) Dapagliflozin (as dapagliflozin propanediol monohydrate Forxiga®): a once daily SGLT-2 inhibitor that will be administered orally in a blinded fashion.

   Doses to be used: 5 mg daily increased after one week to 10 mg daily (orally). Dapagliflozin will be started according to the randomisation (Interactive Web System, hosted by University of Glasgow) in 38 participants (19 in the UK) at 26 weeks as an additional treatment on a background of insulin and semaglutide therapy and end at 52 weeks (triple therapy group). Doses to be used: 5 mg increased to 10 mg after one week.

c) Placebo to dapagliflozin: Dapagliflozin matched placebo will be started in 38 participants (19 in the UK) participants at 26 weeks as an additional treatment on a background of insulin and semaglutide therapy and end at 52 weeks (dual therapy group). Doses to be used: as for active dapagliflozin.

8.2 Legal status of Semaglutide and Dapagliflozin

Semaglutide is currently licensed in the UK for those with adult type 2 diabetes which is insufficiently controlled.

Dapagliflozin is currently licensed in the UK for insufficiently controlled type 1 and type 2 diabetes.

8.3 Drug storage and supply

Semaglutide and dapagliflozin/placebo supplies must be stored in the original containers in a locked, secure area with access limited to authorised site staff. Study supplies should be used as directed in the study protocol and not be supplied to any persons other than study participants. All IMPs will be dispensed to participants from the study site pharmacy.

Covid-19 Mitigation

As detailed above in section 7, the supply of IMP may be altered to reduce risk of Covid-19 transmission in the event of a second wave of infection. This may involve providing increased quantities of IMP to participants to last longer and therefore minimise physical contact with trial personnel. Use of a courier delivery service to facilitate this may also be considered.

Semaglutide: Supplies of semaglutide pre-filled pens for subcutaneous administration will be sourced from routine hospital stocks. The following semaglutide presentations will be required:

- Semaglutide 0.25 mg: Each pre-filled pen contains 2mg semaglutide in 1.5ml. Each dose contains 0.25mg of semaglutide in 0.19ml solution.
- Semaglutide 0.5 mg: Each pre-filled pen contains 2mg semaglutide in 1.5ml solution. Each dose contains 0.5 mg of semaglutide in 0.37ml solution.
- Semaglutide 1 mg: Each pre-filled pen contains 4 mg semaglutide in 3.0ml solution. Each dose contains 1mg semaglutide in 0.74ml solution.

For all presentations each pre-filled pen is capable of delivering 4 doses which is sufficient for 4 weeks treatment. Pens are for individual participant use only. In the event of a pen-failure, a new pen should be supplied to the participant.

Prior to first use, semaglutide pens must be stored at 2-8 °C in the original container and must not be frozen. Supplies which are frozen either prior to first use or during use must be discard.

Semaglutide pre-filled pens have an in-use shelf life of 6 weeks. After first use the pre-filled pens may be stored below 30oC or in a refrigerator at 2-8 °C. Participants should be advised to store packs away from the cooling element. The pen cap should be on when the pen is not in use in order to protect the contents from light.

Wherever possible, supplies of semaglutide pre-filled pens should be utilised in a pragmatic way so as to minimise unnecessary wastage. Participants should be provided with an appropriate number of pen(s) to last until the next planned visit. A small overage may be given as necessary to accommodate visit windows etc. The correct strength of pen must be supplied for each dose titration i.e. 0.25mg, 0.5mg and 1.0 mg as tolerated.

A web-based system will be used to randomise participants to semaglutide treatment. At randomisation and all subsequent visits (planned or unplanned), the system will notify pharmacy staff at study visits of the requirement for semaglutide supplies.

Dapagliflozin 5mg or placebo capsules: Supplies of dapagliflozin 5mg and matched placebo capsules will be manufactured by NHS Scotland Pharmaceuticals Specials Service in accordance with Good Manufacturing Practice requirements. Capsules will be packed into HDPE bottles with a tamper evident seal. Each bottle will be labelled in a double-blind fashion to meet local regulatory requirements and will

be assigned a unique number. Bottles containing dapagliflozin 5 mg or placebo must be stored at room temperature at 15-25°C.

Dapagliflozin 5mg and placebo capsule supplies will be provided to study site and will only be released by the sponsor once all the appropriate regulatory and governance approvals are in place. Further information on storage requirements and supply arrangements is provided in the study specific IMP Management and Accountability Manual.

A web-based system will be used for the assignment of dapagliflozin 5mg or placebo bottles at randomisation and at study visits where there is planned dispensing of the IMP or in the event that a participant requires replacement medicines.

Other medicines: Supplies of all other medicines and associated consumables will via usual NHS supply mechanisms.

8.4 Drug accountability requirements

The Investigator or designee must maintain accurate records of all study IMP movements for accountability purposes. They should include dates, quantities, batch numbers and expiry. Records must document adequately that:

- the patients were provided the doses specified by the protocol/amendment(s)
- all study drug provided was fully reconciled.

Unused study drug must not be discarded or used for any purpose other than the present study. Further information is provided in the study specific IMP Management and Accountability Manual.

8.5 Preparation and administration of semaglutide and dapagliflozin

Semaglutide (Ozempic®) is a solution for subcutaneous injection. It is administered once weekly, on the same day each week, into the subcutaneous tissues of the abdomen, thigh or upper arm. A participant may change the day of their weekly injection as long as it has been at least three days since their last injection. Thereafter, all doses should be administered once weekly on the new dosing day. The injection can be administered at any time of the day regardless of meals.

Participants must be advised to discard the injection needle after each injection and store the pen without an injection needle attached. This may help prevent blocked needles, contamination, infection, leakage of solution and inaccurate dosing. Semaglutide should not be used if the solution does not appear clear and colourless or almost colourless. The pen is designed to be used with NovoFine or NovoTwist disposable needles and pens are packed with an appropriate quantity of NovoFine Plus needles.

Detailed information on administration of semaglutide is provided in the current Summary of Product Characteristics and product Patient Information Leaflet.

Dapagliflozin (Forxiga®)) is presented as a capsule for once daily oral administration.

8.6 Dosage schedules

Semaglutide will be administered and dosed as described. Participants will be commenced on 0.25 mg s.c once weekly before up-titration as tolerated to 1.0 mg s.c once weekly. Up-titration is quicker than is ordinarily the case in clinical practice in type 2 diabetes as discussed in Section 2.1 (22) There will be increased observation and clinical access for participants in the event of side-effects. Participants randomised to receive semaglutide will continue on the 1.0 mg s.c once weekly dose for the remainder of the study following uptitration provided they tolerate the dose as detailed in Section 7. If necessary, the dose may be adjusted.

If a participant misses a dose of semaglutide it should be administered as soon as possible and within 5 days after the missed dose. If more than 5 days have passed, the missed dose should be skipped, and the next dose should be administered on the regularly scheduled day. In both cases participants, should then resume their regular once weekly dosing schedule.

Dapagliflozin will be commenced at 5mg orally once daily and uptitrated one week after commencement to a dose of 10mg which will thereafter be continued for the remainder of the trial provided they tolerate the dose. If necessary, the dose may be adjusted.

8.7 Dosage modifications

Dapagliflozin/placebo will be commenced at one capsule (5mg or placebo) orally once daily and uptitrated one week after commencement to a dose of two capsules (10mg or placebo) which will thereafter be continued for the remainder of the trial provided they tolerate the dose. If necessary, the dose may be adjusted. Dapagliflozin or placebo capsules can be taken at any time of day with or without food. Capsules should be swallowed whole. If participants forget to take a dose they should be advised as follows:

- If it is 12 hours or more until the next dose, the dose should be taken as soon as remembered and the next dose taken at the usual time.
- If it is less than 12 hours until the next dose, the missed dose should be skipped. The next dose should be taken at the usual time.
- Participants should not take a double dose of dapagliflozin/placebo to make up for a forgotten dose.

8.8 Known drug reactions and interaction with other therapies

Participants with hypersensitivity to either active study medication are excluded from the trial. There are no significant interactions between semaglutide and other prescribed medications other than with agents that can lower blood glucose levels. Participants on any other oral agents which can lower blood glucose levels meet Exclusion Criteria and will not be included in this trial. In combination with insulin and dapagliflozin, semaglutide may be associated with an increased risk of hypoglycaemia. Participants will be advised of the increased risk of hypoglycaemia and will be required to closely monitor their blood glucose levels for the duration of the trial. No interactions are currently listed between warfarin and semaglutide in the British National Formulary but in view of a theoretical interaction (based on protein binding), existing arrangements for INR testing will be reviewed for any participants prescribed warfarin and additional tests conducted if indicated according to the judgement of the investigators.

Dapagliflozin is known to increase the risk of hypoglycaemia when combined with other medications which also act to lower blood glucose levels. Dapagliflozin has a mild diuretic effect and therefore there is an increased risk of hypotension when dapagliflozin is combined with either medications which deplete circulating volume (e.g diuretics) or antihypertensive drugs. Dapagliflozin is however commonly prescribed with such medications in general medical practice. All participants will have their medications reviewed at the screening visit and educated regarding symptoms that may indicate dehydration/ hypovolaemia. If there is any concern of interaction with any of the IMPs, this will be discussed with the PI before a decision regarding their ability to participate is made.

8.9 Concomitant medication

Participants should not receive any adjunct glucose-lowering therapy other than as per the randomisation schedule (e.g. with either of the active study medications).

8.10 Trial restrictions

Contraindications will follow SmPC information for Ozempic® and Forxiga. Acceptable forms of contraception for WOCBP participating in the trial are listed in Appendix 2. There is no requirement for contraception for male participants.

8.11 Assessment of compliance

Treatment adherence will be assessed by the trial medical and nursing team when participants attend for scheduled study visits.

8.12 Name and description of each Non-Investigational Medicinal Product (NIMP)

Insulin therapy is a non-investigational medicinal product in this trial. The trial is in a population who have had a diagnosis of type 1 diabetes for at least one year: therefore all participants will already be established on insulin therapy. The regimen and dosage required will by differ between participants. Participants will be using a combination of long acting (basal) insulin and shorter acting (bolus) insulin or CSII (insulin pump). As in standard of care, dose titration will depend on diet, blood glucose levels and exercise. It is anticipated that dosage adjustments will be required to achieve optimal glycaemic control and maintain participant safety.

There are pre-defined guidelines for insulin titration in this study. Insulin dose reductions should not be greater than 10% (5% if HbA1c is<7%) from current total insulin dose or more than 20% from the baseline level at randomisation and then from the new baseline levels at 26 weeks following second randomisation. Participants requiring an insulin dose reduction > 20% to a total insulin <0.4 U/kg will be monitored closely for insulin dose, carbohydrate intake, ketones and blood glucose. Study medication can be temporarily discontinued, if necessary, for one-to-two weeks. If low insulin requirements remain, the participant will be withdrawn from study (see Section 7.8).

9 PHARMACOVIGILANCE

9.1 Definitions

Term	Definition
**Adverse Event (AE)**	Any untoward medical occurrence in a participant to whom a medicinal product has been administered, including occurrences which are not necessarily caused by or related to that product.
**Adverse Reaction (AR)**	An untoward and unintended response in a participant to an investigational medicinal product which is related to any dose administered to that participant.<br>The phrase "response to an investigational medicinal product" means that a causal relationship between a trial medication and an AE is at least a reasonable possibility, i.e. the relationship cannot be ruled out.<br>All cases judged by either the reporting medically qualified professional or the Sponsor as having a reasonable suspected causal relationship to the trial medication qualify as adverse reactions.
**Serious Adverse Event (SAE)**	A serious adverse event is any untoward medical occurrence that:<br>1. results in death<br>2. is life-threatening<br>3. requires inpatient hospitalisation or prolongation of existing hospitalisation<br>4. results in persistent or significant disability/incapacity

	5. consists of a congenital anomaly or birth defect<br><br>Other ‘important medical events’ may also be considered serious if they jeopardise the participant or require an intervention to prevent one of the above consequences.<br>NOTE: The term "life-threatening" in the definition of "serious" refers to an event in which the participant was at risk of death at the time of the event; it does not refer to an event which hypothetically might have caused death if it were more severe*.
**Serious Adverse Reaction (SAR)**	An adverse event that is both serious and, in the opinion of the reporting Investigator, believed with reasonable probability to be due to one of the trial treatments, based on the information provided.
**Suspected Unexpected Serious Adverse Reaction (SUSAR)**	A serious adverse reaction, the nature and severity of which is not consistent with the information about the medicinal product in question set out:<br>• in the case of a product with a marketing authorisation, in the summary of product characteristics (SmPC) for that product<br>• in the case of any other investigational medicinal product, in the investigator’s brochure (IB) relating to the trial in question
**Reference Safety Information**	The information used for assessing whether an adverse reaction is expected. This is contained in either the investigator’s brochure or the summary of product characteristics

*Note: “Severe” is often used to describe intensity of a specific event, which may be of relatively minor medical significance. “Seriousness” is the regulatory definition supplied above.

9.2 Operational definitions for (S)AEs

Adverse events (AEs) will be recorded, notified, assessed, reported, analysed and managed in accordance with the Medicines for Human Use (Clinical Trials) Regulations 2004 (as amended) and this protocol.

All Serious Adverse Events (SAEs) occurring during the trial will be recorded within the eCRF. Non-serious AEs occurring during the trial will be recorded within the eCRF if they are considered to be related to trial medication, i.e. adverse reactions (ARs).

Exclusions from SAE reporting

Hospitalisation for the following reasons will not be considered to be SAEs:

- Treatment which was elective or pre-planned, for a pre-existing condition not associated with any deterioration in condition, e.g. pre-planned hip replacement operation which does not lead to further complications.
- Any admission to hospital or other institution for general care where there was no deterioration in condition.
- Treatment on an emergency, outpatient basis for an event **not** fulfilling any of the definitions of serious as given above and not resulting in hospital admission.
- COVID-19 infection unless results in severe illness with hospital admission.

•
•

9.3 Recording and reporting of AEs, Adverse Events of Special Interest, SAEs and SUSARs

All AEs occurring during the trial that are observed by the Investigator or reported by the participant will be recorded in the participant's medical records whether or not attributed to trial IMP. AEs will be recorded from randomisation until the later of 30 days post cessation of trial treatment or the end of the study.

Adverse Events of Special Interest, SAEs and SUSARs will be recorded in the participant's medical records and on the eCRF and will be recorded from the point of randomisation until the later of 30 days post cessation of trial treatment or the end of the study.

Additional events identified only through record linkage will generate SAE records in the eCRF and should be recorded in the participant's medical records in the same way as AEs.

9.3.1 Adverse Events of Special Interest

Adverse Events of Special Interest as defined in detail below should be recorded within the relevant section of the eCRF but do not require reporting in an expedited manner (unless the event also meets the regulatory definition of an SAE). The following information will be recorded for all events:

- Nature of the event
- Event duration (start and end dates, if applicable)
- Relationship to study drug (if applicable)
- Outcome (if applicable)

Adverse Events of Special Interest will be monitored and followed up (if applicable) until the event has resolved or a final outcome has been reached.

All Adverse Events of Special Interest must be assessed for seriousness prior to recording within the eCRF. Those meeting the definition of an SAE should also be reported accordingly as per Section 9.3.2.

(i) Ketosis and diabetic ketoacidosis

This trial will be conducted in participants who have type 1 diabetes. All participants will be taking insulin. In this condition, due to the near absence of endogenous insulin, at a cellular level there is an inability to incorporate glucose into normal intra-cellular energy yielding pathways. In this context, alternative energy yielding pathways are sought including ketone body production from fatty acids in the liver. Ketones are clinically important as they easily dissociate hydrogen ions upsetting physiologically regulated acid base balance. As ketone levels rise, there is initially a state of high blood ketone levels without significant acidosis (ketosis); if this develops further, diabetic ketoacidosis (DKA) may develop. DKA requires urgent medical input. Ketones can be easily measured via a simple urine test or fingerstick capillary blood measurement. Participants eligible for this trial will already be trained in how to do this. Although dapagliflozin, one of the IMPs in this trial, has a license for use in type 1 diabetes, it is known to increase the risk of ketosis and ketoacidosis and this can occur at lower blood glucose levels than in those who are on insulin treatment only [euglycaemic DKA] (13,14). Given this known risk, ketosis and

DKA are included in the safety endpoints and as such will be recorded in the eCRF as Adverse Events of Special Interest.

DKA is defined clinically and biochemically as a combination of a state of elevated ketone levels (urine ++ or higher, and/or blood > 1.5 mmol/L) in combination with biochemical evidence of a state of metabolic acidosis (pH ≤7.30 or serum bicarbonate < 15 mmol/L). This is as defined by International Consensus Guidelines (34). DKA also varies in severity. In clinical practice the diagnosis is made when a person with type 1 diabetes has elevated ketone levels and usually – although not exclusively – elevated blood glucose levels. The diagnosis is made biochemically by assessing acid base balance through blood testing. This corresponds to the following severity classifications:

- mild DKA: pH 7.25-7.30 (H^+ 50 – 56) or bicarbonate (15 -17.9 mmol/L)
- moderate DKA: pH 7.00-7.24 (H^+ 57 – 100) or bicarbonate 10 – 14.9 mmol/L)
- severe DKA: pH <7.00 (H^+ > 100) or bicarbonate < 10 mmol/L (33)

Given the known risk of DKA, all participants will be made aware of this condition. Patient diaries and Patient Alert Cards will summarise advice for management of high ketone levels including an emergency ketosis management protocol (STICH Protocol – see Appendix 3). All participants will be educated as to the scenarios when this should be activated and this will also be included on the Patient Alert Card which will have the contact details of the study team and participants will be asked to carry at all times.

(ii) Hypoglycaemia

Hypoglycaemia is a low blood glucose level. Hypoglycaemia occurs in diabetes owing to difficulty in precisely matching insulin dosing with the complex factors associated with carbohydrate intake and physical activity. When too much insulin relative to carbohydrate intake is administered, blood glucose levels can drop to a level which is too low. Most people with type 1 diabetes can recognize this phenomenon due to a combination of symptoms which alert them and prompt self-checking of their capillary blood glucose level. Symptoms include weakness, malaise, headache, tremulousness, diaphoresis, palpitations and mild confusion. These are known as autonomic symptoms as they are mediated by activation of the autonomic nervous system as a response to the biological stress of low serum blood glucose. As hypoglycaemia progresses, however, symptoms progress to include confusion and agitation. If untreated this can lead to coma. These symptoms are known as neuroglycopenic symptoms as they are mediated by a lack of glucose in neural cells leading to a confused state.

Hypoglycaemia is also well defined by International Consensus (34). Like DKA, it can be classified in terms of severity. This can be based either on the absolute capillary glucose value and/or clinical features and is classed into stages:

- Level 1 (mild) hypoglycaemia: Blood glucose is 3.0 – 3.8 mmol/L inclusive.
- Level 2 (moderate) hypoglycaemia: Blood glucose is < 3.0 mmol/L
- Level 3 (severe) hypoglycaemia: A person is unable to function because of mental or physical changes due to hypoglycaemia and requires assistance from another person to recover.

The IMPs in this trial are licensed clinically in type 2 diabetes to lower blood glucose levels. It therefore follows that when used in combination with insulin there may be an increased risk of low blood glucose levels. This was the case with the once daily GLP-1 analogue liraglutide in phase 3 trials (8,9), but not in the trials with dapagliflozin (13,14). All participants will have this risk discussed with them, will be performing increased blood glucose monitoring than is the case in usual care, and will be frequently reviewed to adjust insulin dosing in order to mitigate the risk of low blood glucose levels.

9.3.2 Site Reported Serious Adverse Events (SAE)

SAEs will be recorded within the eCRF from the date of randomisation until the later of 30 days post cessation of trial treatment or the end of the study.
Full details of SAEs will be recorded in the electronic Case Report Form. The following information will be collected:

- full details in medical terms and a case description
- seriousness criteria
- event duration (start and end dates, if applicable)
- action taken
- outcome
- causality (i.e. relatedness to trial drug / investigation), in the opinion of the investigator
- if related, whether the reaction would be considered expected or unexpected.

Any change of condition or other follow-up information should be added to the eCRF and forwarded to the Sponsor (if reportable SAE) as soon as it is available or at least within 24 hours of the information becoming available. Events will be followed up until the event has resolved or a final outcome has been reached.

Reporting of suspected/confirmed COVID 19 infection

If a patient presents with clinical signs and symptoms consistent with COVID-19 and a test has been performed or requested:

- If test is positive, record "COVID-19 confirmed" in the diagnosis field
- If test is negative, record the SAE signs and symptoms and/or other diagnosis in the diagnosis field.

If a test has not been performed or result is not available and signs and symptoms, as judged by the investigator, are highly suspicious of COVID-19 infection, record "COVID-19 suspected" in the diagnosis field

If other concurrent diagnoses, e.g. pneumonia, please record within the narrative of the SAE.

Assessment of Adverse Events

All adverse events must be assessed by the reporting site for seriousness**.** All SAEs must also be assessed for causality and expectedness with reference to this protocol and the Reference Safety Information (RSI).

Assessment of seriousness

An adverse event will be considered serious if it:

1. Results in death
2. Is life threatening
3. Requires hospitalisation or prolongation of existing hospitalisation
4. Results in persistent or significant disability or incapacity
5. Consists of a congenital anomaly or birth defect
1. Required intervention to prevent one of the above.

2. Is a serious Adverse Event of Special Interest.

Assessment of causality i.e. does the event have a "reasonable causal relationship" with trial IMPs. The following categories are used:

- **Yes** - The causal and temporal relationship between the event and the absence of a more likely explanation suggest that the trial IMP is the most likely cause.
- **No** - The event is not considered related to the trial IMPs.

Causality will be assigned for each IMP the participant is randomised to receive.

Should an SAE be considered unrelated to the administration of either trial drug then the causality of the event will be collected taking into consideration other factors, for example concomitant medications or co-morbidities, etc.

Assessment of causality is the responsibility of the Principal Investigator or medically-qualified designee(s) at each site. The CI, their delegate(s), or the Sponsor may provide an opinion regarding causality but cannot downgrade the PI's assessment.

Assessment of expectedness

Should the event be considered to be related to the trial IMP(s), an assessment should be made regarding the expectedness of the reaction, i.e. whether the reaction is a recognised adverse effect of the IMP(s).

.
Expected: consistent with the relevant product information documented in the RSI(s).
Unexpected: not consistent with the relevant product information documented in the RSI(s).

The CI, their delegate(s), or the Sponsor have overall responsibility for assigning expectedness as per the RSI(s) and provide the final assessment of the event. The reporting investigator may provide an opinion on the expectedness of an event and this may be taken into account.

SAEs occurring within the USA and the UK

The US Sponsor is responsible for assigning causality and expectedness for any AEs, SAEs and SUSARs that occur within the USA. Where a SUSAR is identified by the US Sponsor this will be forwarded to the UK Sponsor for review within one working day. Following review by the UK Sponsor, appropriate US SUSARs will be subject to expedited reporting to the MHRA as per UK regulatory requirements. Similarly, where a SUSAR is identified by the UK sponsor, this will forwarded to the US Sponsor for review within two working days.

Recording and reporting of SAEs
All SAEs arising during the clinical trial will be recorded in the eCRF soon as reasonably practicable and in any event within 24hours of first becoming aware of the event. Any follow-up information should also be reported.

If recording in the eCRF is not possible a paper SAE form should be completed:

1. The SAE form is downloaded from www.glasgowctu.org, printed off, completed and signed. The form is then faxed to the Glasgow Clinical Trials Unit Pharmacovigilance (PV) Office on +44(0)141 357 5588. As an alternative, a scanned copy of the SAE form may be emailed to:

pharmacovig@glasgowctu.org. If this website is unavailable a paper copy of the SAE form should be filed in the Investigator Site File at each site pending resolution.

2. If necessary a verbal report can be given by contacting the PV Office on +44(0)141 330 4744. This must be followed up as soon as possible with an electronic or written report.

Reporting to sponsor

All SAEs will be considered reportable to the Sponsor PV office from the time of randomisation until 30 days following permanent discontinuation of all trial IMP(s). SAEs occurring following 30 days post cessation of treatment are not reportable to the Sponsor unless an SAE is considered related to IMP(s)

9.3.3 Suspected Unexpected Serious Adverse Reactions (SUSARs)

Any SAE assessed as related to trial IMP(s) by the PI or delegate, and unexpected (not documented as an expected reaction to the IMP(s) within the RSI(s)) by the CI, their delegate, or the Sponsor, will be classified as a SUSAR and therefore subject to expedited reporting to the Medicines and Healthcare products Regulatory Agency (MHRA) and the Research Ethics Committee (REC). Should the CI disagree with the PI's assessment of causality, both opinions will be provided on the report. Where the CI or UK Sponsor disagrees with the US Sponsor's assessment of expectedness both opinions will also be provided on the report.

The Sponsor will inform the MHRA of all SUSARs that occur within the trial and the REC of all SUSARs that occur within the UK within the required expedited reporting timescales:

- **Fatal or life threatening SUSARs** - no later than 7 days of Sponsor awareness that the case fulfils the criteria for a fatal or life threatening SUSAR, and any follow up information within a further 8 days.
- **All other SUSARs** - no later than 15 days of Sponsor awareness that the case fulfils the criteria for a SUSAR.

The sponsor will report SUSARs to the MHRA via the MHRA eSUSAR reporting system and to REC by email with accompanying CTIMP Safety Report Form.

9.3.4 Unblinding for SUSAR reporting purposes

In the event of a potential SUSAR, the sponsor (but not the investigators) will be unblinded to facilitate appropriate reporting to the MHRA and REC.

9.4 Responsibilities for Safety Reporting and Review

This section details the responsibilities for reporting and reviewing safety information arising from the trial.

<u>Principal Investigator (PI)</u>:

- Attendance at the initiation meeting/teleconference
- Training of new members of the trial team in the protocol and its procedures
- Ensuring that the ISF is accurately maintained
- Dissemination of important safety or trial-related information to all stakeholders at their site
- Checking for AEs and ARs when participants attend for treatment/follow-up

- Ensuring that AEs are recorded and reported in line with the requirements of the protocol
- Ensuring that all SAEs are recorded and reported to the Sponsor within 24 hours of becoming aware of the event, and providing further follow-up information as soon as available
- Using medical judgement in assigning causality of SAEs
- Providing an opinion on the expectedness of an SAE with reference to the trial protocol and Reference Safety Information
- Using definitions in this protocol, flagging events of special interest.

Chief Investigator (CI)

- Providing clinical oversight of the safety of patients participating in the trial, including an ongoing review of the risk/benefit
- Providing clinical review of section 4.8 of the representative SmPCs on an annual basis to define the Reference Safety Information for the trial
- Using medical judgement to confirm seriousness and causality of all SARs
- Reviewing expectedness of related SAEs with reference to the trial protocol and Reference Safety Information
- Preparing the clinical sections and final sign-off of the Development Safety Update Report (DSUR)
- Using definitions in this protocol, confirming events of special interest

Sponsor:

- Central data collection and verification of AEs, SAEs, SARs and SUSARs according to the trial protocol
- Reporting safety information to the CI or delegate for the ongoing assessment of the risk / benefit
- Reporting safety information to the independent oversight committees identified for the trial (Independent Data Monitoring Committee (IDMC) and / or Trial Steering Committee (TSC)) according to the Trial Monitoring Plan.
- Expedited reporting of SUSARs to the Competent Authority (MHRA in UK) and REC within required timelines.
- Notifying Investigators of SUSARs that occur within the trial.
- Checking for (annually) and notifying PIs of updates to the Reference Safety Information for the trial.
- Preparing standard tables and other relevant information for the DSUR in collaboration with the CI and ensuring timely submission to the MHRA and REC.

Trial Steering Committee:

In accordance with the Charter for the TSC, periodically reviewing recruitment and the overall progress of the trial and liaising with the IDMC and sponsor regarding safety issues.

Independent Data Monitoring Committee:

In accordance with the Charter for the IDMC, periodically reviewing unblinded safety data in individual cases and to determine patterns and trends of events, or to identify safety issues, which would not be apparent on an individual case basis, reporting concerns to the TSC and sponsor.

9.5 Pregnancy reporting

Pregnancy is not considered an AE unless a negative or consequential outcome is recorded for the mother or child/foetus. If the outcome meets the serious criteria, this would be considered an SAE and must be reported as per SAE reporting procedure above.

Any **pregnancy** occurring in a female trial participant or female partner of a male trial participant who becomes pregnant while participating in the Trial will be reported by the PI (or designee) to the Chief Investigator and the sponsor using the sponsor Pregnancy Reporting Form (available at http://www.glasgowctu.org/) within two weeks of the PI first becoming aware of the pregnancy.
The trial participant will also be followed up to determine the outcome of the pregnancy and follow-up information forwarded to the PV office. Any resulting SAEs should be reported as per SAE reporting procedure above.

9.6 Overdose

Any IMP dose which is not administered in accordance with the Protocol should be reported to the sponsor.
If an SAE is associated with an overdose ensure that the overdose if fully described in the SAE report form.

9.7 Reporting urgent safety measures

If any urgent safety measures are taken the CI/Sponsor will phone the MHRA's Clinical Trial Unit on 020 3080 6456, ideally within 24 hours. This will be followed up no later than 3 days from the date the measures are taken, giving written notice to the MHRA (who will advise the format required) and the relevant REC of the measures taken and the circumstances giving rise to those measures. A substantial amendment must also be submitted to the MHRA.

9.8 The type and duration of the follow-up of participants after adverse events.

Adverse events and reactions will be recorded, reported and followed up in line with this protocol until study completion or for a minimum of 30 days after participant's last dose of the IMP, whichever is later. Any SUSAR identified will be reported to the Sponsor and to the Regulatory Authorities irrespective of how long after IMP administration the reaction has occurred.

9.9 Development safety update reports

A Development Safety Update Report (DSUR) will be submitted once a year, or on request, to MHRA and REC until the trial is declared ended. The report will be submitted within 60 days of the anniversary of the issue of the Clinical Trials Authorisation for the trial. The DSUR will be prepared by the sponsor (PV Office) in liaison with the CI and submitted by the sponsor (PV Office). The DSUR will include only those SAEs reported to the co-sponsors; that is: SAEs and SUSARs occurring within the UK and SUSARs originating in the USA.

10 STATISTICS AND DATA ANALYSIS

10.1 Sample size calculation

This is based on our current data showing that in type 1 diabetes, treatment with a GLP-1 agonist, liraglutide (1.2 and 1.8mg doses) for three months leads to HbA1c suppression by an average of 0.6% (SD= 0.5%). Dapagliflozin was recently shown to reduce HbA1c by 0.45% in type 1 diabetes patients (13). Additionally, empagliflozin treatment for 8 weeks was shown to reduce HbA1c by 0.4% in type 1 diabetics (20, 21) and our current data showing additional improvement in HbA1c by an additional 0.66% at 12 weeks when dapagliflozin was added to liraglutide and insulin therapy. Conservatively estimating that the combination therapy will suppress HbA1c by 0.5% (SD≤0.7%) compared to semaglutide alone, a sample size of 38 (32 evaluable patients) in each group will be sufficient to provide 80% power at a

5% significance level, taking in consideration a 15% dropout rate. 50% of participants will be recruited in Glasgow, UK and 50% at State University of New York at Buffalo, USA.

10.2 Anticipated recruitment rate

One year (one participant per week).

10.3 Statistical analysis

The primary objective of the proposed research is to evaluate the effect of addition of dapagliflozin to semaglutide on a background of insulin treatment in type 1 diabetes patients on HbA1c at six months following start of dapagliflozin. Transformations of the data in order to meet statistical assumptions will be considered. Statistical analyses will use linear regression models to compare treatment groups, with adjustment for baseline values of study outcomes, as well as factors used to stratify the randomisation scheme. Tests will be two-sided and tested at the 5% significance level. Analyses will be carried out under the intention to treat principle, that is, according to randomised treatment allocation, regardless of treatment adherence.

A detailed Statistical Analysis Plan will be completed and signed off before database lock. Study statisticians will develop and validate the analysis programs without access to randomised treatment allocations. After database lock, the treatment codes will be released and the final analysis will be carried out.

10.4 Subgroup analyses

These will be specified in the Statistical Analysis Plan. Subgroup analyses will be implemented by extending regression models to include interactions between treatment and potential treatment effect moderators.

10.5 Interim analysis and criteria for the premature termination of the trial

No interim analysis is planned. Unblinded trial data will be reviewed on an ongoing basis by the IDMC. The primary role of the IDMC will be to protect the interests of the patients. The IDMC may recommend to the TSC and Co-Sponsors that the study should stop prematurely because of concerns about patient safety. The IDMC will meet approximately every six months. The IDMC will take into account all results and the consistency and biological plausibility of the findings in making any recommendation. The final decision on continuing or stopping the trial will lie with the TSC/Co-Sponsors.

10.6 Subject population

All analyses will be carried out on an intention to treat basis, using the randomised treatment allocation.

10.7 Procedure(s) to account for missing or spurious data

Data will be analysed without imputation in the first instance. Multiple imputation procedures will be used to assess the sensitivity of selected results to missing data, according to the Statistical Analysis Plan.

10.8 Other statistical considerations.

The Statistical Analysis Plan (SAP) will be maintained as a version controlled document and will be signed off before database lock. The SAP and other statistical documentation (according to Glasgow Clinical Trials Unit Standard Operating Procedures) will contain full details of all analyses along with assumptions and procedures for handling problematic or incomplete data (e.g. incomplete dates).

11 DATA HANDLING

11.1 Source Documentation

ICH GCP defines source data as: 'All information in original records and certified copies of original records of clinical findings, observations or other activities in a clinical trial necessary for the reconstruction and evaluation of the trial'. In this study, the location of the majority of the source data will be the hospital's medical records including subject case notes, laboratory records and ECGs. The source data transcribed into the eCRF from the medical records must be accurate and verifiable. For questionnaires completed by trial subjects, the completed questionnaires will be regarded as the source data location. In cases where data is transcribed directly into the eCRF and no other paper or electronic source exists, then the eCRF will be considered the source record. In these cases, these data should be prospectively documented in the medical records to ensure a full record of the trial is available at site.

11.2 Data collection

An eCRF, developed by the Robertson Centre for Biostatistics, will capture all data required to meet this protocol's requirements. Access to the eCRF will be restricted, via a study-specific web portal, and only authorised site-specific personnel will be able to make entries to their patients' data via the web portal. The Investigator, or his/her designee will be responsible for all entries into the eCRF and will confirm that the data are accurate, complete and verifiable. Data will be stored in a MS SQL Server database. Direct access to the web portal will be granted, on request, to authorised representatives from the Sponsor, host institution and the regulatory authorities to permit trial-related monitoring, audits and inspections.

11.3 Data Validation

Where it is practical, data will be validated at the point of entry into the eCRF. Any additional data discrepancies will be flagged to the investigator and any data changes will be recorded to maintain a complete audit trail (reason for change, date change made, who made change).

11.4 Data Security

The Robertson Centre for Biostatistics systems are fully validated in accordance with industry and regulatory standards, and incorporate controlled access security. High volume servers are firewall protected and preventative system maintenance policies are in place to ensure no loss of service or data. Web servers are secured by digital certificates. Data integrity is assured by strictly controlled procedures, including secure data transfer procedures. Data are backed up on-site nightly and off-site to a commercial data vault weekly. The Robertson Centre for Biostatistics has an ISO 9001:2008 quality management system and ISO 27001:2013 for Information Security, and is regularly inspected against the standards by the British Standards Institution.

11.5 Archiving

The Trial Master File will be archived by the Co-Sponsors at the end of the trial for a minimum period of five years.

Archiving of Site Files will also be for a minimum of five years form completion of the trial, and this action will be delegated to the sites in the Clinical Trial Site Agreement that will be put in place between Co-Sponsors and Sites. Sites will be notified by the Co-Sponsors when Site files can be archived.

Destruction of site files can only take place with the approval of the Co-Sponsors.

12 MONITORING, AUDIT & INSPECTION

Monitoring will be conducted by NHS Greater Glasgow and Clyde (GG&C) Monitor (s) in accordance with local Standard Operating Procedures. The level, frequency and priorities of monitoring will be based on the outcome of the completed Risk Assessment, and clearly documented in the Monitoring Plan which will be approved by the NHS GG&C Lead Monitor and/or Research Governance Manager.

13 ETHICAL AND REGULATORY CONSIDERATIONS

13.1 Research Ethics Committee (REC) review & reports

Before the start of the trial, approval will be sought from a REC for the trial protocol, informed consent forms and other relevant documents e.g. advertisements and GP information letters.
Substantial amendments that require review by REC will not be implemented until the REC grants a favourable opinion for the study (it is noted that amendments may also need to be reviewed and accepted by the MHRA and/or NHS R&D departments before they can be implemented in practice at sites).
All correspondence with the REC will be retained in the Trial Master File/Investigator Site File.
An annual progress report (APR) will be submitted to the REC within 30 days of the anniversary date on which the favourable opinion was given, and annually until the trial is declared ended (this is the Chief Investigator's responsibility).
The Chief Investigator will notify the REC of the end of the study.
If the study is ended prematurely, the Chief Investigator will notify the REC, including the reasons for the premature termination
Within one year after the end of the study, the Chief Investigator will submit a final report with the results, including any publications/abstracts, to the REC.

13.2 Peer review

The study protocol has been developed with expert and independent feedback from the JDRF (New York) and during application for funding underwent peer review by independent experts.

13.3 Public and Patient Involvement

The study was designed with input from people with type 1 diabetes via the funder, JDRF.

13.4 Regulatory Compliance

The trial will not commence until a Clinical Trial Authorisation (CTA) is obtained from the MHRA.
The protocol and trial conduct will comply with the Medicines for Human Use (Clinical Trials) Regulations 2004 and any relevant amendments.
Before any site can enrol patients into the trial, the Chief Investigator/Principal Investigator or designee will apply for NHS permission from the site's Research & Development (R&D) department.

For any amendment that will potentially affect a site's NHS permission, the Chief Investigator/ Principal Investigator or designee will confirm with that site's R&D department that NHS permission is ongoing (note that both substantial amendments, and amendments considered to be non-substantial for the purposes of REC and/or MHRA may still need to be notified to NHS R&D).

13.5 Protocol compliance

Prospective, planned deviations or waivers to the protocol are not allowed under the UK regulations on Clinical Trials and must not be used e.g. it is not acceptable to enrol a participant if they do not meet the eligibility criteria or restrictions specified in the trial protocol. Accidental protocol deviations can happen

at any time. They must be adequately documented on the relevant forms and reported to the Chief Investigator, Sponsor and GCTU immediately. Deviations from the protocol which are found to frequently recur are not acceptable, will require immediate action and could potentially be classified as a serious breach.

13.6 Notification of Serious Breaches to GCP and/or the protocol

A "serious breach" is a breach which is likely to affect to a significant degree –

1. the safety or physical or mental integrity of the subjects of the trial; or
2. the scientific value of the trial

If any of the above occurs then the CI and Sponsor will be notified. The sponsor will notify the appropriate authorities in writing of any serious breach in accordance with their standard operating procedures.

13.7 Data protection and patient confidentiality

All investigators and trial site staff must comply with the requirements of the GDPR with regards to the collection, storage, processing and disclosure of personal information and will uphold the Act's core principles.

- Personal information will be collected via the eCRF to enable record linkage to be carried out and to provide electronic access to study monitors to a copy of the signed informed consent document. These data items will be encrypted and only those individuals who require to see these data i.e. the person performing the record linkage and site research team staff or the study monitor, as appropriate, will be able to view them. All electronic data will be held securely in accordance with ISO 27001:2013 at the Robertson Centre for Biostatistics, part of the Glasgow Clinical Trials Unit. All Centre staff are required to sign confidentiality agreements and to follow Standard Operating Procedures in accordance with Good Clinical Practice and ISO certification.
- The trial data managers, statisticians, health economists or any other staff who will perform data related tasks will only be able to access depersonalised data where the participant's identifying information is replaced by a unique study identifier.

- Only those that have been trained and approved will be able to enter or view any data via the web portal. Each site can only see their own patients' data. Patient consent forms will be stored at the study site in a secure location accessible only to study teams.

13.8 Indemnity

The Co-Sponsors (University of Glasgow and Greater Glasgow Health Board) will ensure that provision has been made for insurance or indemnity to cover the liability of the investigator and sponsor which may arise in relation to the clinical trial in accordance with Part 2 (14) of Schedule 1 to SI 2004/1031.

13.9 Amendments

Any change in the study protocol will require an amendment. Any proposed protocol amendments will be initiated by the CI following discussion with the Sponsor and TSC and any required amendment forms will be submitted to the regulatory authority, ethics committee and Sponsor. The Sponsor will determine whether an amendment is non-substantial or substantial. All amended versions of the protocol will be signed by the CI and Sponsor representative. Following a substantial amendment, favourable opinion/approval must be sought from the original reviewing REC, MHRA (where appropriate) and Research and Development (R&D) office prior to implementation. The Chief Investigator will be responsible for informing the Trial Management Group of all protocol amendments.

13.10 Post trial care

At the end of the trial, participants will be returned to usual care as defined by local and national guidelines at that time. The results of the trial may of course have an impact on these guidelines and the future care of patients with type 1 diabetes.

13.11 Access to the final trial dataset

During the trial and in the period prior to publication of the main study results as described in the protocol, only the Glasgow CTU and Professor Paresh Dandona, US Principal Investigator, University of Buffalo will have access to the full dataset. After that period, the trial Steering Committee will conduct further data analyses for a period of three years. After that time the Trial Steering Committee will consider requests from external parties for further analyses of the study data. Proposals that are scientifically well founded and have an academic basis and where relevant data extractions and analyses are appropriately funded will not be refused. These will be considered as collaborative exercises where the contributions related to study design, conduct, database creation and maintenance and data analysis will be recognised in authorship of any scientific publication. The approach we will take will be to minimise any possibility of breach of participant confidentiality. Normally this will be achieved by minimising data travel. However, for the purposes of individual patient meta-analysis and other reasons, data may be transferred to other sites. Such transfer will require assurances on information security systems at the sites that data are to be transferred to and will involve a legal data transfer agreement. A log of all data requests and subsequent data transfers will be held at the Glasgow CTU.

14 DISSEMINATION POLICY

14.1 Dissemination policy

The study database will be owned by the University of Glasgow and maintained on behalf of the Study investigators, represented by the Trial Steering Committee as it is constituted during and after the trial.

The study protocol and a description of the recruitment experience and participant baseline characteristics will be published before study completion. On completion of the trial, the database will be locked and analysed by staff of the Robertson Centre for Biostatistics, University of Glasgow. A final study report will be prepared and the results will be published in a major medical journal.

After the main study publications, study investigators will be invited to submit requests for further analyses of the study database. These will be reviewed and prioritised by a Publications Committee made up of the study grant holders and convened by the study co-CIs.

As funder of the trial, JDRF will have the right to see and comment on any results being submitted for publication. A maximum of 28 days will be given for review of major papers and 14 days for abstracts. Such comments will be considered by the Trial Steering Committee.

A lay summary of the main results of the trial will be prepared and provided to all participants via their study site investigators. Investigators may request a copy of the study data for their participants. Providing some or all of a patient's data to that individual is at their discretion.

14.2 Authorship eligibility guidelines and any intended use of professional writers

The main results of the study will be compiled, written up and published by the study grant holders and others taking responsibility for the study results (e.g. the statistician conducting the final analysis) on behalf of the TTT1 investigators. The TTT1 investigators will be listed in an Appendix and will include all site PIs, all committee members and key members of relevant study coordinating groups (including the Sponsor and Glasgow CTU).

15 REFERENCES

1. Konrad K, Datz N, Engelsberger I, Grulich-Henn J, Hoertenhuber T, Knauth B, et al. Current use of metformin in addition to insulin in pediatric patients with type 1 diabetes mellitus: an analysis based on a large diabetes registry in Germany and Austria. *Pediatr Diabetes.* 2014.
2. Vella S, Buetow L, Royle P, Livingstone S, Colhoun HM, and Petrie JR. The use of metformin in type 1 diabetes: a systematic review of efficacy. *Diabetologia.* 2010;53(5):809-20.
3. Vella S, Buetow L, Royle P, Livingstone S, and Petrie JR. Metformin in type 1 diabetes reduces insulin requirements without significantly improving glycaemic control. Reply to Schatz H [letter]. *Diabetologia.* 2011;54(1):203-4.
4. Varanasi A, Bellini N, Rawal D, Vora M, Makdissi A, Dhindsa S, et al. Liraglutide as additional treatment for type 1 diabetes. *Eur J Endocrinol.* 2011;165(1):77-84.
5. Kuhadiya ND, Malik R, Bellini NJ, Patterson JL, Traina A, Makdissi A, et al. Liraglutide as additional treatment to insulin in obese patients with type 1 diabetes mellitus. *Endocrine practice : official journal of the American College of Endocrinology and the American Association of Clinical Endocrinologists.* 2013;19(6):963-7.
6. Kuhadiya ND, Dhindsa S, Ghanim H, Mehta A, Makdissi A, Batra M, et al. Addition of Liraglutide to Insulin in Patients With Type 1 Diabetes: A Randomized Placebo-Controlled Clinical Trial of 12 Weeks. *Diabetes Care.* 2016;39(6):1027-35.
7. Chaudhuri A, Ghanim H, Makdissi A, Green K, Abuaysheh S, Batra M, et al. Exenatide induces an increase in vasodilatory and a decrease in vasoconstrictive mediators. *Diabetes Obes Metab.* 2017;19(5):729-33.
8. Mathieu C, Zinman B, Hemmingsson JU, Woo V, Colman P, Christiansen E, et al. Efficacy and Safety of Liraglutide Added to Insulin Treatment in Type 1 Diabetes: The ADJUNCT ONE Treat-To-Target Randomized Trial. *Diabetes Care.* 2016;39(10):1702-10.
9. Ahren B, Hirsch IB, Pieber TR, Mathieu C, Gomez-Peralta F, Hansen TK, et al. Efficacy and Safety of Liraglutide Added to Capped Insulin Treatment in Subjects With Type 1 Diabetes: The ADJUNCT TWO Randomized Trial. *Diabetes Care.* 2016;39(10):1693-701.
10. Zinman B, Wanner C, Lachin JM, Fitchett D, Bluhmki E, Hantel S, et al. Empagliflozin, Cardiovascular Outcomes, and Mortality in Type 2 Diabetes. *N Engl J Med.* 2015;373(22):2117-28.
11. Petrie JR. SGLT2 inhibitors in type 1 diabetes: knocked down, but up again? *Lancet Diabetes Endocrinol.* 2017;5(11):841-3.
12. Mudaliar S, Armstrong DA, Mavian AA, O'Connor-Semmes R, Mydlow PK, Ye J, et al. Remogliflozin etabonate, a selective inhibitor of the sodium-glucose transporter 2, improves serum glucose profiles in type 1 diabetes. *Diabetes Care.* 2012;35(11):2198-200.
13. Dandona P, Mathieu C, Phillip M, Hansen L, Griffen SC, Tschope D, et al. Efficacy and safety of dapagliflozin in patients with inadequately controlled type 1 diabetes (DEPICT-1): 24 week results from a multicentre, double-blind, phase 3, randomised controlled trial. *Lancet Diabetes Endocrinol.* 2017;5(11):864-76.
14. Mathieu C, Dandona P, Gillard P, Senior P, Hasslacher C, Araki E, Lind M, Bain SC, Jabbour S, Arya N, Hansen L, Thorén F, Langkilde AM; DEPICT-2 Investigators. Efficacy and Safety of Dapagliflozin in Patients With Inadequately Controlled Type 1 Diabetes (the DEPICT-2 Study): 24-Week Results From a Randomized Controlled Trial. Diabetes Care. 2018 Sep;41(9):1938-1946.

15. European Medicines Agency. Assessment Report Dapagliflozin. https://www.ema.europa.eu/en/documents/variation-report/forxiga-h-c-2322-ws-1344-epar-assessment-report-variation_ar.pdf
16. Rosenstock J, Marquard J, Laffel LM, et al. Empagliflozin as adjunctive to insulin therapy in type 1 diabetes: the EASE trials. *Diabetes Care*. 2018;41(12):2560–2569.
17. Garg SK, Henry RR, Banks P, Buse JB, Davies MJ, Fulcher GR, et al. Effects of Sotagliflozin Added to Insulin in Patients with Type 1 Diabetes. *N Engl J Med.* 2017;377(24):2337-48.
18. European Medicines Agency. First oral add-on treatment to insulin for treatment of certain patients with type 1 diabetes. https://www.ema.europa.eu/en/news/first-oral-add-treatment-insulin-treatment-certain-patients-type-1-diabetes
19. An overview of Zynquista and why it is authorised in the EU. 16th May 2019. https://www.ema.europa.eu/en/documents/overview/zynquista-epar-medicine-overview_en.pdf
20. National Institute for Health and Care Excellence. Dapagliflozin with insulin for treating type 1 diabetes.
21. Ahrén B, Hirsch IB, Pieber TR, Mathieu C, Gómez-Peralta F, Hansen TK, Philotheou A, Birch S, Christiansen E, Jensen TJ, Buse JB; ADJUNCT TWO Investigators. Efficacy and Safety of Liraglutide Added to Capped Insulin Treatment in Subjects With Type 1 Diabetes: The ADJUNCT TWO Randomized Trial. Diabetes Care. 2016 Oct;39(10):1693-701.
22. Ozempic Summary of product characteristics. https://www.medicines.org.uk/emc/product/9728/smpc
23. Nauck MA, Petrie JR, Sesti G, Mannucci E, Courrèges JP, Lindegaard ML, Jensen CB, Atkin SL; Study 1821 Investigators. A Phase 2, Randomized, Dose-Finding Study of the Novel Once-Weekly Human GLP-1 Analog, Semaglutide, Compared With Placebo and Open-Label Liraglutide in Patients With Type 2 Diabetes. Diabetes Care. 2016 Feb;39(2):231-41.
24. Technology appraisal guidance [TA597] https://www.nice.org.uk/guidance/ta597
25. Dapagliflozin Summary of Product Characteristics: https://www.medicines.org.uk/emc/product/2865/smpc
26. Wiviott SD, Raz I, Bonaca MP, Mosenzon O, Kato ET, Cahn A, Silverman MG, Zelniker TA, Kuder JF, Murphy SA, Bhatt DL, Leiter LA, McGuire DK, Wilding JPH, Ruff CT, Gause-Nilsson IAM, Fredriksson M, Johansson PA, Langkilde AM, Sabatine MS; DECLARE–TIMI 58 Investigators. Dapagliflozin and Cardiovascular Outcomes inType 2 Diabetes. N Engl J Med. 2019 Jan 24;380(4):347-357.
27. McMurray JJV, Solomon SD, Inzucchi SE, Køber L, Kosiborod MN, Martinez FA et al. DAPA-HF Trial Committees and Investigators Dapagliflozin in patients with heart failure and reduced ejection fraction. N Engl J Med. 2019. Nov 21;381(21):1995-2008.
28. Vilsbøll T, Bain SC, Leiter LA, et al. Semaglutide, reduction in glycated haemoglobin and the risk of diabetic retinopathy. *Diabetes Obes Metab*. 2018;20(4):889–897.
29. Lingvay I, Desouza CV, Lalic KS et al. A 25 week randomized controlled trial of semaglutide once daily versus liraglutide and placebo in patients with type 2 diabetes suboptimally controlled on diet nad exercise with or without metformin. Diabetes Care. 2018;41(9): 1926-1937.

30. European Medicines Agency. Assessment Report Sotagliflozin. https://www.ema.europa.eu/en/documents/assessment-report/zynquista-epar-public-assessment-report_en.pdf
31. Peters AL, Buschur EO, Buse JB, Cohan P, Diner JC, and Hirsch IB. Euglycemic Diabetic Ketoacidosis: A Potential Complication of Treatment With Sodium-Glucose Cotransporter 2 Inhibition. Diabetes Care. 2015;38(9):1687-93.
32. Silverii GA, Decembrini L, Monami M, Mannucci E. Fournier's gangrene and sodium glucose co-transporter 2 inhibitors: A metanalysis of randomized controlled trials. Diabetes Obes Metab. 2019; 1-4.
33. Agiostratidou G, Anhalt H, Ball D, Blonde L, Gourgari E, Harriman KN, Kowalski AJ, Madden P, McAuliffe-Fogarty AH, McElwee-Malloy M, Peters A, Raman S, Reifschneider K, Rubin K, Weinzimer SA. Standardizing Clinically Meaningful Outcome Measures Beyond HbA(1c) for Type 1 Diabetes: A Consensus Report of the American Association of Clinical Endocrinologists, the American Association of Diabetes Educators, the American Diabetes Association, the Endocrine Society, JDRF International, The Leona M. and Harry B. Helmsley Charitable Trust, the Pediatric Endocrine Society, and the T1D Exchange. Diabetes Care. 2017 Dec;40(12):1622-1630.
34. Garg SK, Peters AL, Buse JB, Danne T. Strategy for mitigating DKA risk in patients with Type 1 diabetes on adjunctive treatment with SGLT-2 inhibitors: A STICH Protocol. Diabetes Technol Ther. 2018;20(9):571-575.

16. APPENDICES

16.1 Appendix 1 – Risk

<table>
<tr><td colspan="5">Risks associated with trial interventions
☐ LOW ≡ Comparable to the risk of standard medical care
☒ MODERATE ≡ Somewhat higher than the risk of standard medical care
☐ HIGH ≡ Markedly higher than the risk of standard medical care</td></tr>
<tr><td colspan="5">Justification: Briefly justify the risk category selected and your conclusions below (where the table is completed in detail the detail need not be repeated, however a summary should be given):
An IDMC will be convened to monitor all SAEs.
Risks and mitigations associated with the intervention are outlined in more detail in the Protocol section 2.1.
Co-Sponsors will also carry out a detailed risk assessment of all aspects of the study as part of the approval process (SOP 04.013)</td></tr>
<tr><td colspan="2">What are the key risks related to therapeutic interventions you plan to monitor in this trial?</td><td colspan="3">How will these risks be minimised?</td></tr>
<tr><td>IMP/Intervention</td><td>Body system/Hazard</td><td>Activity</td><td>Frequency</td><td>Comments</td></tr>
<tr><td>S/C administration of semaglutide
Oral admistration of dapagliflozin</td><td>Immune: Hypersensitivity/ana-phylactic reactions</td><td>Patients with known hypersensitivity to any GLP-1 agonist or SGLT-2 inhibitor have a contra-indication to the IMP according to the SmPC will not be recruited to the study</td><td>Uncommon/ Rare</td><td></td></tr>
<tr><td>PO administration of dapagliflozin</td><td>Ketosis and/or Diabetic Ketoacidosis (DKA)</td><td>All participants will have access to blood and urine ketone monitoring equipment. An emergency ketosis management plan (STICH Protocol – see above) will be discussed with all participants. Patient alert cards will be provided to all participants. Potential participants with recent ketoacidosis will be</td><td>Common</td><td></td></tr>
</table>

		excluded from the trial (section 6.2)		
S/C administration of semaglutide (+/-) PO administration of dapagliflozin in addition to insulin therapy	Increased risk of hypoglycaemia	Participant awareness of risk and frequent testing of capillary blood glucose. Patients with pre-existing frequent episodes of hypoglycaemia or problems with hypoglycaemia awareness will be excluded from trial participation (section 6.2)	Common	
Outline any other processes that have been put in place to mitigate risks to participant safety (e.g. IDMC, independent data review, etc.)<br>See above				

16.2 Appendix 2: Contraception

In line with recommendations related to contraception and pregnancy testing in clinical trials from the Clinical Trial Facilitation Group (CTFG), for women of childbearing potential* (WOCBP) participating in TTT1, methods of contraception considered as highly effective are defined as those that can achieve a failure rate of less than 1% per year when used consistently and correctly.

Such methods include:

- combined (oestrogen and progestogen containing) hormonal contraception associated with inhibition of ovulation[1]
  - oral
  - intravaginal
  - transdermal
- progestogen-only hormonal contraception associated with inhibition of ovulation[1]
  - oral
  - injectable
  - implantable[2]
- intrauterine device (IUD)[2]
- intrauterine hormone-releasing system (IUS)[2]
- bilateral tubal occlusion[2]
- vasectomised partner[2,3]
- true sexual abstinence[4]

Footnotes

1. An interaction between dapagliflozin and semaglutide with contraception is not anticipated.
2. Contraception methods that in the context of this guidance are considered to have low user dependency.
3. Vasectomised partner is a highly effective birth control method provided that partner is the sole sexual partner of the WOCBP trial participant and that the vasectomised partner has received medical assessment of the surgical success.
4. In the context of this guidance true sexual abstinence refers to: When this is in line with the preferred and usual lifestyle of the patient. Periodic abstinence (e.g. calendar, ovulation, symptothermal, post-ovulation methods), declaration of abstinence for the duration of a trial, and withdrawal are not acceptable methods of contraception.
The reliability of sexual abstinence needs to be evaluated in relation to the duration of the clinical trial and the preferred and usual lifestyle of the subject.

*Child bearing potential = experienced menarche; AND not undergone successful surgical sterilisation (hysterectomy, bilateral tubal ligation, or bilateral oophorectomy); AND not post-menopausal i.e. amenorrhea for ≥12 consecutive months (without another medical cause)

Source: Recommendations related to contraception and pregnancy testing in clinical trials. Clinical Trial Facilitation Group (CTFG), 15th September 2014.
See: https://www.hma.eu/fileadmin/dateien/Human_Medicines/01-About_HMA/Working_Groups/CTFG/2014_09_HMA_CTFG_Contraception.pdf (Accessed 21st January 2020)

16.3 Appendix 3

STICH Protocol *(also provided in Period 2 Participant Diary)*

As explained above, participants in this trial randomised to dapagliflozin are at an increased risk of ketosis and ketoacidosis. All participants will have access to a ketone meter as standard of care. It will be ascertained that each participant has access to a ketone meter; if there are any issues with their current ketone meter it will be replaced as per standard of care. There are mechanisms designed to mitigate the risk of early ketosis developing further into diabetic ketoacidosis. In this trial, we will be using a protocol called STICH (34). This is a mnemonic designed to act as a reminder for participants as to what to do in the event of elevated ketones following exposure to an SGLT-2 drug such as dapagliflozin:

1. STop dapagliflozin or placebo temporarily
2. Insulin – take a bolus of rapid acting insulin
3. Carbohydrate – take approximately 30g of carbohydrate
4. Hydrate – make sure you are taking sips of water aiming to maintain hydration.

Further guidance will be provided in both the Patient Diaries supplied to all participants and in the Patient Alert Cards that participants will be asked to carry at all times. All participants will also be advised that in the event of acute intercurrent illness or hospitalisation, dapagliflozin or placebo should be temporarily stopped. This is because intercurrent illness may additionally increase the risk of ketosis. Participants will likely already be familiar with this concept of "sick-day rules" as this is a key component of basic education for people with type 1 diabetes in clinical practice. These rules will however be re-iterated to participants.

More detailed guidance on actions participants should take for any given level of ketosis will be provided during face-to-face and telephone contact during the trial. In addition, however, the following written protocol will be provided to participants to advise them explicitly of the safest and most appropriate course of action to be taken in any given circumstance. This will take the form of a traffic light system to aid understanding and compliance.

1. Ketones 0.6 mmol/L or less: Continue - no need for additional bolus insulin.
2. Ketones 0.6 – 1.4 mmol/L: consider taking additional fast acting insulin. Follow sick day rules and monitor ketones 2 hourly until they are 0.6 mmol/L or less.
3. Ketones 1.5 – 2.9 mmol/L – Take 10% of your total daily dose of insulin (this is found by adding up all the long acting and short acting insulin for the previous day - only take a maximum of 10 units) as a bolus of fast acting insulin. Keep hydrated - aim for 100ml water per hour. Re-test your glucose and ketones in 2 hours. If not falling contact the study team during office hours. If you need urgent advice at other times please contact NHS 24 (telephone number 111) or attend your nearest Accident & Emergency Department.
4. Ketones above 3 mmol/L – Take 20% of your total daily insulin as fast acting insulin (Maximum 10 units). Hydrate with water, aim for 500 ml per hour. Attend A&E – High risk of DKA

If you develop DKA or are unwell it is important that you let the TTT1 nurse or doctor know about this as soon as you can. By following this protocol, the risk of ketosis and developing ketoacidosis will be mitigated.

16.4 Appendix 4: Amendment History

Amendment No.	Protocol version no.	Date issued	Author(s) of changes	Details of changes made
AM02	2.0			Protocol Changes: Weight loss mitigation, COVID19 Risk Mitigation and visit activity corrections

List details of all protocol amendments here whenever a new version of the protocol is produced. Protocol amendments must be submitted to the Sponsor for approval prior to submission to the REC committee or MHRA.

16.5 Appendix 5: PAID Questionnaire

Problem Areas In Diabetes (PAID) scale

Instructions: Which of the following diabetes issues are **currently** a problem for you? Tick the box that gives the best answer for you. Please provide an answer for each question.

		Not a problem	Minor problem	Moderate problem	Somewhat serious problem	Serious problem
1	Not having clear and concrete goals for your diabetes care?	☐ 0	☐ 1	☐ 2	☐ 3	☐ 4
2	Feeling discouraged with your diabetes treatment plan?	☐ 0	☐ 1	☐ 2	☐ 3	☐ 4
3	Feeling scared when you think about living with diabetes?	☐ 0	☐ 1	☐ 2	☐ 3	☐ 4
4	Uncomfortable social situations related to your diabetes care (e.g. people telling you what to eat)?	☐ 0	☐ 1	☐ 2	☐ 3	☐ 4
5	Feelings of deprivation regarding food and meals?	☐ 0	☐ 1	☐ 2	☐ 3	☐ 4
6	Feeling depressed when you think about living with diabetes?	☐ 0	☐ 1	☐ 2	☐ 3	☐ 4
7	Not knowing if your mood or feelings are related to your diabetes?	☐ 0	☐ 1	☐ 2	☐ 3	☐ 4
8	Feeling overwhelmed by your diabetes?	☐ 0	☐ 1	☐ 2	☐ 3	☐ 4
9	Worrying about low blood glucose reactions?	☐ 0	☐ 1	☐ 2	☐ 3	☐ 4
10	Feeling angry when you think about living with diabetes?	☐ 0	☐ 1	☐ 2	☐ 3	☐ 4
11	Feeling constantly concerned about food and eating?	☐ 0	☐ 1	☐ 2	☐ 3	☐ 4
12	Worrying about the future and the possibility of serious complications?	☐ 0	☐ 1	☐ 2	☐ 3	☐ 4
13	Feelings of guilt or anxiety when you get off track with your diabetes management?	☐ 0	☐ 1	☐ 2	☐ 3	☐ 4
14	Not 'accepting' your diabetes?	☐ 0	☐ 1	☐ 2	☐ 3	☐ 4
15	Feeling unsatisfied with your diabetes physician?	☐ 0	☐ 1	☐ 2	☐ 3	☐ 4
16	Feeling that diabetes is taking up too much of your mental and physical energy every day?	☐ 0	☐ 1	☐ 2	☐ 3	☐ 4
17	Feeling alone with your diabetes?	☐ 0	☐ 1	☐ 2	☐ 3	☐ 4
18	Feeling that your friends and family are not supportive of your diabetes management efforts?	☐ 0	☐ 1	☐ 2	☐ 3	☐ 4
19	Coping with complications of diabetes?	☐ 0	☐ 1	☐ 2	☐ 3	☐ 4
20	Feeling 'burned out' by the constant effort needed to manage diabetes?	☐ 0	☐ 1	☐ 2	☐ 3	☐ 4

© Joslin Diabetes Center, 1999 (www.joslin.org). All rights reserved. The copyright holder/developer has given permission for the questionnaire to be reproduced in this toolkit. Readers of the toolkit are permitted to reproduce the questionnaire for clinical use and non-commercial research purposes. Readers of the toolkit are not permitted to use the questionnaire for commercial research purposes and must seek permission from the copyright holder/developer to do so.

16.6 Appendix 6: DSQOLS Questionnaire

Last Name: ____________________ **First Name:** ______________ **MI:** ____

Date: (month. day. year): I___I___I . I___I___I . I___I___I___I___I

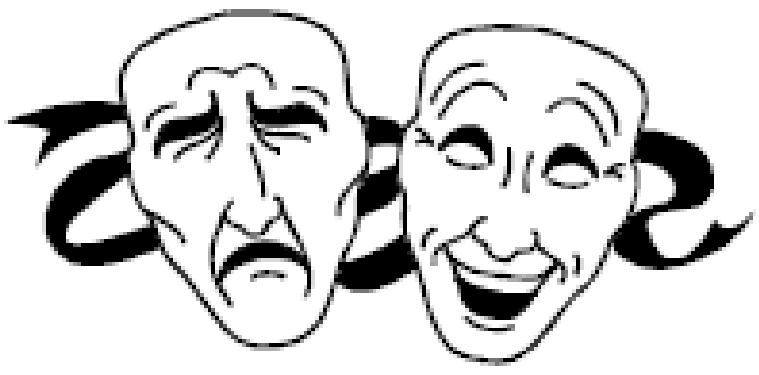

Questionnaire regarding

- **Treatment goals**
- **Treatment satisfaction and**
- **Daily Burdens**

of Patients with Type 1 Diabetes

Diabetes is associated with different restrictions and burdens for most people.

This questionnaire asks what your treatment goals are, how much diabetes represents a burden in your day to day living, and how satisfied you are with your current treatment.

Please rate each statement on how applicable it is to your personal situation.
Fill in ONLY one square per question.
Please answer the questions in the given order without skipping any.

For diabetes treatment it is especially important to me, or it would be my goal that ...	extremely impor-tant	pretty impor-tant	rather impor-tant	rather unim-portant	pretty unim-portant	totally unim-portant
1. my blood sugar values are always below 7 mmol/l (126 mg%).	❑	❑	❑	❑	❑	❑
2. my blood sugar values are as stable as possible.	❑	❑	❑	❑	❑	❑
3. I can arrange my free time with flexibility.	❑	❑	❑	❑	❑	❑
4. I avoid even mild episodes of low blood sugar.	❑	❑	❑	❑	❑	❑
5. I can definitely prevent the long-term complications of diabetes, or slow down their progression.	❑	❑	❑	❑	❑	❑
6. I can eat flexibly by choosing what food I want, how much I want, and when I want my meals.	❑	❑	❑	❑	❑	❑
7. I am as physically fit as possible.	❑	❑	❑	❑	❑	❑
8. I can definitely avoid severe episodes of low blood sugar involving loss of consciousness.	❑	❑	❑	❑	❑	❑
9. I have to measure my blood glucose as rarely as possible.	❑	❑	❑	❑	❑	❑
10. other people don't find out that I have diabetes.	❑	❑	❑	❑	❑	❑

How satisfied have you been over the <u>last 4 weeks</u> with ...	extremely satis-fied	pretty satis-fied	rather satis-fied	rather dissatis-fied	pretty dissatis-fied	totally dissatis-fied
1. your blood sugar levels?	❑	❑	❑	❑	❑	❑
2. the stability of your blood sugar values?	❑	❑	❑	❑	❑	❑
3. the flexibility in arranging your free time?	❑	❑	❑	❑	❑	❑
4. the frequency of mild episodes of low blood sugar?	❑	❑	❑	❑	❑	❑
5. your protection from the long-term complications, or your ability to slow down their progression?	❑	❑	❑	❑	❑	❑
6. the flexibility of your diet?	❑	❑	❑	❑	❑	❑
7. your physical strength?	❑	❑	❑	❑	❑	❑
8. your protection from severe episodes of low blood sugar involving loss of consciousness?	❑	❑	❑	❑	❑	❑
9. the frequency of your blood sugar self-monitoring?	❑	❑	❑	❑	❑	❑
10. the extent to which other people are informed about your diabetes?	❑	❑	❑	❑	❑	❑

How much have you felt burdened and restricted by diabetes and its treatment over the last 4 weeks?

This statement meets my point of view ...

	per-fectly	quite good	some-what	not much	hardly	not at all
1. It bothers me that I have to measure my blood sugar so often	☐	☐	☐	☐	☐	☐
2. It is a burden for me that I need to constantly think about my food plan	☐	☐	☐	☐	☐	☐
3. I suffer from pain because of diabetes	☐	☐	☐	☐	☐	☐
4. Because of diabetes my relationship with my partner has become worse	☐	☐	☐	☐	☐	☐
5. I am worried that my life could be shorter because of diabetes	☐	☐	☐	☐	☐	☐
6. I feel as if I am less attractive to others because of diabetes.	☐	☐	☐	☐	☐	☐
7. Because of diabetes I feel sad or depressed	☐	☐	☐	☐	☐	☐
8. I am worried about my future health	☐	☐	☐	☐	☐	☐
9. It is a burden for me how other people react to my diabetes	☐	☐	☐	☐	☐	☐
10. I feel nervous and restless when I think about episodes of low blood sugar	☐	☐	☐	☐	☐	☐
11. I feel tired and exhausted	☐	☐	☐	☐	☐	☐
12. It bothers me that I have to spend so much time on my diabetes treatment	☐	☐	☐	☐	☐	☐
13. Because of diabetes traveling is complicated and troublesome	☐	☐	☐	☐	☐	☐
14. I get anxious and nervous when I think about the dangers of episodes of low blood sugar	☐	☐	☐	☐	☐	☐
15. Diabetes prevents me from spontaneous physical activities	☐	☐	☐	☐	☐	☐
16. I suffer from frequent urination	☐	☐	☐	☐	☐	☐
17. Because of high blood sugar values after a meal I often feel unwell or less efficient	☐	☐	☐	☐	☐	☐
18. Because of diabetes I feel anxious and threatened	☐	☐	☐	☐	☐	☐
19. I suffer from thirst or having a dry mouth	☐	☐	☐	☐	☐	☐
20. Other people find it hard to understand my problems with diabetes treatment	☐	☐	☐	☐	☐	☐
21. I feel physically unwell	☐	☐	☐	☐	☐	☐
22. I unnecessarily worry too much about episodes of low blood sugar	☐	☐	☐	☐	☐	☐
23. I have to give up good-tasting foods	☐	☐	☐	☐	☐	☐

	This statement meets my point of view ...					
	per-fectly	quite good	some-what	not much	hardly	not at all
24. I cannot eat as much as I want of certain foods........................	❑	❑	❑	❑	❑	❑
25. Because of diabetes I often have physical complaints.............	❑	❑	❑	❑	❑	❑
26. Diabetes interferes with my future plans..................................	❑	❑	❑	❑	❑	❑
27. I get an uncomfortable feeling when I think about the dangers of a severe episode of low blood sugar.....................................	❑	❑	❑	❑	❑	❑
28. Because of diabetes I have less contact with friends or acquaintances..	❑	❑	❑	❑	❑	❑
29. I wish I could eat more the way I want to, without having to plan everything beforehand..	❑	❑	❑	❑	❑	❑
30. I suffer from frequent infections, itching or skin problems........	❑	❑	❑	❑	❑	❑
31. I am concerned about getting into embarrassing situations because of episodes of low blood sugar..................................	❑	❑	❑	❑	❑	❑
32. I feel weak or lazy..	❑	❑	❑	❑	❑	❑
33. I am dissatisfied with the amount of time I have to spend for medical consultations..	❑	❑	❑	❑	❑	❑
34. Because of diabetes I cannot spend my free time the way I would like..	❑	❑	❑	❑	❑	❑
35. I feel like a disabled person..	❑	❑	❑	❑	❑	❑
36. I am worried that I could sustain physical injury in the event of a severe episode of low blood sugar	❑	❑	❑	❑	❑	❑
37. It bothers me that I cannot eat like other people......................	❑	❑	❑	❑	❑	❑
38. I am often worried about the long-term complications of diabetes..	❑	❑	❑	❑	❑	❑
39. Because of diabetes other people treat me like a "sick person"	❑	❑	❑	❑	❑	❑
40. Because of diabetes I cannot pursue my hobbies as I wish.....	❑	❑	❑	❑	❑	❑
41. I have to frequently think about diabetes and its consequences..	❑	❑	❑	❑	❑	❑
42. It bothers me that I cannot eat as spontaneously as people who do not have diabetes..	❑	❑	❑	❑	❑	❑
43. It upsets my stomach when I think about the dangers of severe episodes of low blood sugar..	❑	❑	❑	❑	❑	❑
44. Diabetes constantly causes problems while dealing with other people..	❑	❑	❑	❑	❑	❑
45. It bothers me that I have to take my diabetes supplies (for example syringes) with me whatever I do................................	❑	❑	❑	❑	❑	❑
46. I frequently worry that I may become helpless and may need constant care later on..	❑	❑	❑	❑	❑	❑

	This statement meets my point of view ...					
	per-fectly	quite good	some-what	not much	hardly	not at all
47. I often cannot eat as much as I would like................................	❑	❑	❑	❑	❑	❑
48. I am worried about having a severe episode of low blood sugar at night..	❑	❑	❑	❑	❑	❑
49. Because of diabetes my family-life is affected..........................	❑	❑	❑	❑	❑	❑
50. Because of diabetes my physical strength is restricted............	❑	❑	❑	❑	❑	❑
51. Because of diabetes it is much harder to make friends............	❑	❑	❑	❑	❑	❑
52. It bothers me that I have to frequently think about possible causes and mistakes after episodes of low blood sugar..........	❑	❑	❑	❑	❑	❑
53. It bothers me how much diabetes controls my daily life...........	❑	❑	❑	❑	❑	❑
54. It bothers me that I have to inject insulin before I know how much I would really like to eat or I really can eat......................	❑	❑	❑	❑	❑	❑
55. I would like to eat a greater amount of certain foods which increase my blood sugar strongly..	❑	❑	❑	❑	❑	❑
56. I am worried that I could easily panic in the event of an episode of low blood sugar...	❑	❑	❑	❑	❑	❑
57. When I think about the dangers associated with severe episodes of low blood sugar I wonder how often I will remain unharmed..	❑	❑	❑	❑	❑	❑

FULL/LONG TITLE OF THE TRIAL
Triple Therapy for Type 1 Diabetes with Insulin, Semaglutide and Dapagliflozin (TTT1) - UK

SHORT STUDY TITLE / ACRONYM
TTT1 (UK)

PROTOCOL VERSION NUMBER AND DATE
Version 2.1(14.11.2020)

RESEARCH REFERENCE NUMBERS

**IRAS Number:**	254431
**EudraCT Number:**	2018-004120-11
**ISRCTN Number / Clinical trials.gov Number:**	TBC
**SPONSOR Number:**	GN17DI706
**FUNDER Number:**	3-SRA-2019-669-M-B

SIGNATURE PAGE

The undersigned confirm that the following protocol has been agreed and accepted and that the Chief Investigator agrees to conduct the trial in compliance with the approved protocol and will adhere to the principles outlined in the Medicines for Human Use (Clinical Trials) Regulations 2004 (SI 2004/1031), amended regulations (SI 2006/1928) and any subsequent amendments of the clinical trial regulations, Good Clinical Practice (GCP) guidelines, the Sponsor's (Standard Operating Procedures (SOPs), and other regulatory requirements as amended.
I agree to ensure that the confidential information contained in this document will not be used for any other purpose other than the evaluation or conduct of the clinical investigation without the prior written consent of the Sponsor
I also confirm that I will make the findings of the study publicly available through publication or other dissemination tools without any unnecessary delay and that an honest accurate and transparent account of the study will be given; and that any discrepancies from the study as planned in this protocol will be explained.

For and on behalf of the Study Co-Sponsor (University of Glasgow):

Signature: .... Date: ....../....../......

Name (please print): ....

Position: ....

For and on behalf of the Study Co-Sponsor (NHS Greater Glasgow and Clyde):

Signature: .... Date: ....../....../......

Name (please print): ....

Position: ....

UK Chief Investigator:

Signature: .... Date: ....../....../......

Name: (please print): John Petrie ....

Position: Professor of Diabetic Medicine..

Statistician:

Signature: .... Date: ....../....../......

Name: (please print): ....

Position: ....

KEY TRIAL CONTACTS

UK Chief Investigator	Professor John R Petrie BSc<br>Professor of Diabetic Medicine<br>Institute of Cardiovascular and Medical Sciences<br>BHF Glasgow Cardiovascular Research Centre<br>University of Glasgow<br>126 University Place<br>Glasgow G12 8TA<br>Secretary +44(0)141 330 3325<br>john.petrie@glasgow.ac.uk
UK Co- Investigator	Dr James Boyle<br>Consultant Diabetologist<br>Clinical Lead NHS GG&C Diabetes MCN<br>Diabetes UK Clinical Champion<br>Glasgow Royal Infirmary<br>84 Castle Street, Glasgow, G4 0SF<br>Tel: : +44(0)141 211 5996<br>james.boyle@glasgow.ac.uk
Trial Co-ordination	Miss Lisa Jolly<br>Research and Innovation<br>NHS Greater Glasgow & Clyde<br>Ward 11, Dykebar Hospital<br>Grahamston Road<br>Paisley, PA2 7DE<br>Lisa.Jolly@ggc.scot.nhs.uk
Co-Sponsor	NHS Greater Glasgow & Clyde<br><br>Contact: Dr Maureen Travers<br>Research and Development Management Office<br>Research and Innovation<br>NHS Greater Glasgow & Clyde<br>Ward 11, Dykebar Hospital<br>Grahamston Road<br>Paisley, PA2 7DE<br>Tel: +44 (0) 0141 314 4012<br>maureen.travers@ggc.scot.nhs.uk
Co-sponsor	University of Glasgow<br><br>Contact: Dr Debra Stuart<br>College of Medical, Veterinary & Life Sciences<br>Room 327 Wolfson Medical School Building<br>University Avenue<br>Glasgow G12 8QQ<br>Tel: +44 (0)141 330 4539<br>Debra.Stuart@glasgow.ac.uk

Funder	JDRF<br>26 Broadway, 15th floor, New York, NY 10004, USA
Clinical Trials Unit	Glasgow Clinical Trials Unit<br>Robertson Centre for Biostatistics, 11th Floor,<br>Boyd Orr Building, University of Glasgow<br>G12 8QQ<br>Tel: 0141 330 4744<br>liz.anderson@glasgow.ac.uk
Statistician	Professor Alex McConnachie<br>Robertson Centre for Biostatistics, 11th Floor, Boyd Orr Building, University of Glasgow, Glasgow, G12 8QQ<br>Telephone: 0141 330 4744<br>Fax: 0141-330-5094
Trials pharmacist	Dr Elizabeth Douglas<br>Clinical Trials Pharmacist<br>Clinical Research and Development<br>Ward 11, Dykebar Hospital,<br>Grahamston Road | Paisley PA2 7DE<br>Tel: 0141 314 4073<br>E-mail: elizabeth.douglas@ggc.scot.nhs.uk

US Principal Investigator	Dr Paresh Dandona<br>Director, Diabetes-Endocrinology Center of Western NY<br>Chief of Endocrinology, Diabetes, Endocrinology and Metabolism, SUNY at Buffalo<br>1000 Youngs Road, Suite 105<br>Williamsville NY 14221<br>Telephone: +1(716) 535-1850<br>Fax: +1(716)-639-1200<br>pdandona@kaleidahealth.org
US Co- Investigator	Dr Husam Ghanim, PhD.<br>Director, Diabetes and Endocrinology Research Lab<br>Research Associate Professor,<br>School of Medicine, State University of NY at Buffalo<br>Clinical and Translational Research Center<br>875 Ellicott St<br>Buffalo, NY 14203<br>Tel: (+1)716-881-8924

	ghanim@buffalo.edu
Key Protocol Contributors	Professor John Petrie<br>john.petrie@glasgow.acuk<br><br>Dr Paresh Dandona<br>pdandona@kaleidahealth.org<br><br>Husam Ghanim, PhD.<br>ghanim@buffalo.edu<br><br>Dr James Boyle<br>james.boyle@glasgow.ac.uk<br><br>Dr Robert Lindsay<br>robert.lindsay@glasgow.ac.uk<br><br>Dr Joseph Timmons<br>joseph.timmons@glasgow.ac.uk<br><br>Professor Ian Ford<br>ian.ford@glasgow.ac.uk<br><br>Professor Alex McConnachie<br>Alex.McConnachie@glasgow.ac.uk

TRIAL SUMMARY

Trial Title	Triple Therapy for Type 1 Diabetes with Insulin, Semaglutide, and Dapagliflozin (UK)	
Internal ref. no. (or short title)	TTT1	
Clinical Phase	Phase 3	
Trial Design	Two six-month periods: (i) open-label randomised (2:1) parallel group semaglutide vs standard of care; (ii) additional double-blind, randomised (1:1) parallel group, dapagliflozin vs placebo for those participants initially randomised to semaglutide.<br>Regardless of each randomisation, ongoing standard of care (insulin therapy) will be provided for all participants throughout the study.	
Trial Participants	Adults with type 1 diabetes for at least one year with HbA1c 7.5-11.0% on stable multiple daily injections (MDI) (four or more) of insulin or continuous subcutaneous insulin infusion (CSII) for the previous three months.	
Planned Sample Size	114 (57 in UK)	
Treatment duration	Approximately 1 year	
Follow up duration	Approximately 1 year	
Planned Trial Period	Approximately 3 years	
	Objectives	Outcome Measures
Primary	To assess in people with type 1 diabetes whether triple therapy (dapagliflozin, semaglutide and insulin) reduces HbA1c in comparison with dual therapy (placebo, semaglutide, and insulin – period 2).	HbA1c
Secondary	To assess in people with type 1 diabetes whether triple therapy (dapagliflozin, semaglutide and insulin) reduces HbA1c in comparison with standard therapy (insulin alone)	HbA1c
	To assess in people with type 1 diabetes whether dual therapy (semaglutide and insulin - period 1) reduces HbA1c in comparison with standard therapy (insulin alone)	HbA1c
	To assess in people with type 1 diabetes whether triple therapy (dapagliflozin, semaglutide and insulin) improves glucose control	- Mean fasting blood glucose<br><br>- Mean blood glucose*<br>- Percentage time in range

	as assessed by CGM, insulin requirement, body weight, blood pressure and quality of life in comparison with (a) dual therapy (placebo, semaglutide, and insulin – period 2) and (b) standard therapy (insulin alone)	- Glycaemic variability*<br>- Percentage time in hyperglycaemia Level 1 (10.0-13.9 mmol/)*<br>- Percentage time in hyperglycaemia Level 2 (>13.9 mmol/L)*<br>-Percentage time in hypoglycaemia Level 1 (3.0 – 3.8 mmol/L)*<br>** CGM (by masked Dexcom G6)*<br><br>Insulin requirement<br>Body weight<br>Systolic/ diastolic blood pressure<br>Number of antihypertensive agents<br>Diabetes Specific Quality of Life Scale (DSQOLS)<br>Problem Areas In Diabetes (PAID) questionnaire
Safety Endpoint 1	a) Triple therapy (dapagliflozin, semaglutide and insulin) in comparison with dual therapy (placebo, semaglutide, insulin – period 2)<br><br>b) Dual therapy (semaglutide and insulin - period 1) in comparison with standard therapy (insulin only)	Difference between treatment arms in:<br>(i) Rate of Level 2 (<3.0 mmol/L) hypoglycaemic events (by Dexcom G6 CGM)<br>(ii) Rate of Level 3 hypoglycaemic events (severe event with altered mental and/or physical status requiring assistance)
Safety Endpoint 2	a) Triple therapy (dapagliflozin, semaglutide and insulin) in comparison with dual therapy (placebo, semaglutide, and insulin – period 2)<br><br>b) Dual therapy (semaglutide and insulin –period 1) in comparison with standard therapy (insulin only)	Difference between treatment arms in:<br>Rate of diabetic ketoacidosis (serum ketones or urinary ketones above normal limits AND serum bicarbonate < 15 mmol/L OR blood pH < 7.3)
Investigational Medicinal Product(s)	Semaglutide<br>Dapagliflozin<br>Placebo matching dapagliflozin	
Non-investigational Medicinal Product(s)	Insulin	
Formulation, Dose, Route of Administration	Semaglutide (subcutaneous injection, once weekly) – open label by pen device (0.25 mg/ 0.5 mg/ 1.0 mg)<br>Dapagliflozin 5 mg (oral) – one then two capsules daily (uptitration)<br>Placebo matching dapagliflozin	

FUNDING AND SUPPORT IN KIND

FUNDER(S)	FINANCIAL AND NON FINANCIALSUPPORT GIVEN
JDRF New York<br>26 Broadway, 15th floor, New York, NY 10004, United States	$1.6M (to University at Buffalo, the State University of New York, NY (SUNY Buffalo), USA; and University of Glasgow, UK)
AstraZeneca<br>1800 Concord Pike, Wilmington, DE 19803, USA	Provision of dapagliflozin and matched placebo investigational medicinal products to US site
Dexcom; Suite 6, 1 Tanfield, Edinburgh, EH3 5DA	Staff training and discounted supply of CGM sensors

ROLE OF STUDY SPONSOR AND FUNDER

NHS Greater Glasgow & Clyde and The University of Glasgow will be Co-sponsors of the UK arm of the trial. Prior to study initiation, a non-commercially funded clinical trial co-sponsorship agreement will be put in place between NHS Greater Glasgow & Clyde and The University of Glasgow. The roles and liabilities each organisation will take under The Medicines for Human Use (Clinical Trials) Regulations, 2004 SI 2001:1031 are laid out in this agreement signed by both organisations. The University of Glasgow shall be responsible for carrying out the obligations and responsibilities set out in the aforementioned agreement, and shall be deemed “sponsor” for the purposes of, Part 3 of the regulations in relation to the study. NHS Greater Glasgow & Clyde shall be responsible for carrying out the responsibilities set out in the agreement, and shall be deemed “sponsor” for the purposes of, Parts 4, 5, 6 and 7 of the Regulations in relation to the study.

The Co-Sponsors will delegate specific roles to the Chief Investigator, Glasgow CTU and other third parties. These arrangements will be clearly documented in agreements and/or the Sponsor Delegated Roles and Responsibilities Matrix.

The US arm of the trial has the same inclusion and exclusion criteria and the same pre-specified outcomes but an individualised Protocol. It will collect the same data via a common eCRF. Adverse event data from each site will be available to the trial Data and Safety Monitoring Board. The UK Co-Sponsors will have no responsibility for (and carry no liability for) the conduct of the US arm of the trial.

JDRF

The study has been funded by a grant from JDRF. JDRF does not have a designated role or responsibility in trial design, conduct, data analysis and interpretation, manuscript writing, and dissemination of results. Periodic reports (at least annual) in relation to progress of the trial will be submitted to the JDRF. Support from JDRF will be acknowledged in any publications related to the study.

Astra Zeneca

This is an investigator-initiated study. Astra Zeneca provided support by supplying investigational medicinal products (IMPs) dapagliflozin and matched placebo to the US site only. Representatives of Astra Zeneca reviewed and provided comments on drafts of the Protocol but the company does not have a designated role or responsibility in trial design, conduct, data analysis and interpretation, manuscript writing, and dissemination of results.

ROLES AND RESPONSIBILITIES OF TRIAL MANAGEMENT COMMITTEES/GROUPS & INDIVIDUALS

Trial Steering Committee (TSC)

The role of the TSC is to provide overall supervision of the trial and ensure that it is being conducted in accordance with the principles of GCP and the relevant regulations. The TSC will:

1. agree the trial protocol and any protocol amendments
2. ensure that conduct of the trial is fully harmonised between the two sites
3. provide advice to the investigators on all aspects of the trial

It will have an independent Chairperson and include the US and UK investigators and key protocol contributors listed above.

Decisions about continuation or termination of the trial or substantial amendments to the protocol will be the responsibility of the TSC who will advise the co-sponsors. The TSC will meet at the start of the study, and six monthly thereafter. The TSC will have its own Charter outlining the role and responsibilities of its members. The TSC may invite other attendees from the trial team to present or participate in discussions on particular topics. These attendees will be non-voting members.

Independent data monitoring committee (IDMC)

An IDMC will be established to include two independent medical experts (covering the domains of type 1 diabetes and metabolic disease; one of the academic clinicians will act as chair) and an independent biostatistician. The Glasgow CTU will liaise with the committee and ensure that the Committee is provided with adequate information about study progress and results.

The IDMC will have a formal Charter outlining the responsibilities of the IDMC members, Glasgow CTU and the co-sponsors. Responsibilities include:

- To protect the safety of participants recruited to the trial.
- Advising the TSC and co-sponsors if it is safe and appropriate to continue with the study
- Examining information provided by the Glasgow CTU on study recruitment, adverse events and outcomes
- Providing reports for the Project Office to forward to the TSC, ethics committees, regulatory bodies, study co-sponsors, and the BHF.

The IDMC may recommend to the TSC to terminate the study or specific aspects of the study if clear harm is observed with one treatment option compared to standard of care or placebo. Specific safety endpoints that will be reviewed include rates of hypoglycaemia and ketosis/ ketoacidosis. The IDMC will not formally review efficacy or futility.

The IDMC will meet approximately every six months.

Study Management Group (SMG)

Trial activity at the UK/ Glasgow site will be coordinated from NHS Greater Glasgow & Clyde (NHS GG&C) by the TTT1 Study Management Group (SMG). The SMG will consist of the UK chief investigator, co-principal investigator other co-applicants, project manager and representatives from the Glasgow CTU, NHS GG&C and The University of Glasgow. The role of the group is to monitor all

aspects of the conduct and progress of the trial in the UK, ensuring adherence to the protocol, and take appropriate action to safeguard participants and the quality of the trial itself.

Joint Study Management Group

Coordination of trial activity between the UK/ Glasgow and the US/ Buffalo site will be facilitated by the Joint TTT1 Study Management Group. This will consist of the US and UK chief investigators, co-principal investigators, other co-applicants, study coordinators, project managers and governance/ administrative representatives as appropriate from State University of New York (SUNY), the Glasgow CTU, NHS GG&C and The University of Glasgow. It will meet approximately every 4-6 weeks. The role of the group is to coordinate all aspects of the conduct and progress of the trial between the US and UK sites, ensuring protocol adherence, and taking appropriate action to safeguard participants and the quality of the trial itself.

Protocol contributors

The protocol has been developed by a group with extensive clinical and research experience in type 1 diabetes and clinical trials including Professor John Petrie and Professor Paresh Dandona. Professor Ian Ford and Professor Alex McConnachie have research expertise in design, conduct, analysis and interpretation of clinical trials and biostatistical methods.

KEY WORDS:

Adjunct therapy
Dapagliflozin
Glycated haemoglobin (HbA1c)
Semaglutide
Type 1 diabetes

LIST of CONTENTS

RESEARCH REFERENCE NUMBERS 2
SIGNATURE PAGE 3
KEY TRIAL CONTACTS 4
TRIAL SUMMARY 7
FUNDING AND SUPPORT IN KIND ....9~~10~~
ROLE OF STUDY SPONSOR AND FUNDER ....9~~10~~
ROLES AND RESPONSIBILITIES OF TRIAL MANAGEMENT COMMITTEES/GROUPS & INDIVIDUALS ....10~~11~~
LIST of CONTENTS ....13~~14~~
LIST OF ABBREVIATIONS ....14~~15~~
TRIAL FLOW CHART ....16~~17~~
SCHEDULE OF ASSESSMENTS ....17**Error! Bookmark not defined.**
1 BACKGROUND ....18~~19~~
2 RATIONALE ....19~~20~~
3 OBJECTIVES AND OUTCOME MEASURES/ENDPOINTS ....22~~23~~
4 TRIAL DESIGN ....25~~26~~
5 STUDY SETTING ....25~~26~~
6 ELIGIBILITY CRITERIA ....25~~26~~
7 TRIAL PROCEDURES ....28~~30~~
8 TRIAL MEDICATION ....**Error! Bookmark not defined.**
9 PHARMACOVIGILANCE ....57~~60~~6
10 STATISTICS AND DATA ANALYSIS ....66~~68~~
11 DATA HANDLING ....67~~70~~
12 MONITORING, AUDIT & INSPECTION ....68~~71~~
13 ETHICAL AND REGULATORY CONSIDERATIONS ....68~~71~~
14 DISSEMINIATION POLICY ....72~~74~~
15 REFERENCES ....73~~75~~1
16 APPENDICES ....76~~78~~

LIST OF ABBREVIATIONS

ACS	Acute Coronary Syndrome
AUC	Area Under the Curve
AE	Adverse Event
AR	Adverse Reaction
CA	Competent Authority
CABG	Coronary Artery Bypass Graft
CGM	Continuous Glucose Monitoring
CHI	Community Health Index
CI	Chief Investigator
CRO	Contract Research Organisation
CRP	C-Reactive Protein
CSII	Continuous Subcutaneous Insulin Infusion
CTA	Clinical Trial Authorisation
CTU	Clinical Trials Unit
CTIMP	Clinical Trial of Investigational Medicinal Product
CV	Cardiovascular
CVA	Cerebrovascular Accident
DKA	Diabetic Ketoacidosis
DSUR	Development Safety Update Report
EC	European Commission
ECG	Electrocardiogram
eCRF	Electronic Case Report Form
EDTA	Ethylenediaminetetraacetic acid
eGFR	Estimated Glomerular Filtration Rate
EMEA	European Medicines Agency
EU	European Union
EUCTD	European Clinical Trials Directive
EudraCT	European Clinical Trials Database
EudraVIGILANCE	European database for Pharmacovigilance
FBC	Full Blood Count
FGM	Flash Glucose Monitoring
GCP	Good Clinical Practice
GDG	Guideline Development Group
GDPR	General Data Protection Regulation
GLP-1	Glucagon Like Peptide-1
GLP-1RA	Glucagon Like Peptide-1 Receptor Agonist
GMP	Good Manufacturing Practice
HbA1c	Glycated haemoglobin
IB	Investigator Brochure
ICF	Informed Consent Form
ICH	International Conference on Harmonisation of technical requirements for registration of pharmaceuticals for human use.
IDMC	Independent Data Monitoring Committee
IMP	Investigational Medicinal Product
IMPD	Investigational Medicinal Product Dossier
ISF	Investigator Site File
ISRCTN	International Standard Randomised Controlled Trials Number

IWRS	Interactive Web-based Randomisation System
LPLV	Last Patient Last Visit
MA	Marketing Authorisation
MDRD	Modification of Diet in Renal Disease
MODY	Maturity Onset Diabetes of the Young
MHRA	Medicines and Healthcare products Regulatory Agency
MI	Myocardial Infarction
MDI	Multiple Daily Injections
NHS GG&C	National Health Service Greater Glasgow & Clyde
NHS R&D	National Health Service Research & Development
NICE	The National Institute for Health and Care Excellence
NIMP	Non-Investigational Medicinal Product
PI	Principal Investigator
PIC	Participant Identification Centre
PIS	Participant Information Sheet
PV	Pharmacovigilance
QA	Quality Assurance
QC	Quality Control
QoL	Quality of Life
QP	Qualified Person
RCT	Randomised Control Trial
REC	Research Ethics Committee
SAE	Serious Adverse Event
SAP	Statistical Analysis Plan
SAR	Serious Adverse Reaction
SDV	Source Data Verification
SGLT2	Sodium Glucose Transporter 2
SOP	Standard Operating Procedure
SUNY	State University of New York
SmPC	Summary of Product Characteristics
SSI	Site Specific Information
SUSAR	Suspected Unexpected Serious Adverse Reaction
TIA	Transient Ischaemic Attack
TMG	Trial Management Group
TSC	Trial Steering Committee
TMF	Trial Master File
UB	University of Buffalo
ULN	Upper Limit of Normal reference range
WOCBP	Women Of Childbearing Potential

TRIAL FLOW CHART

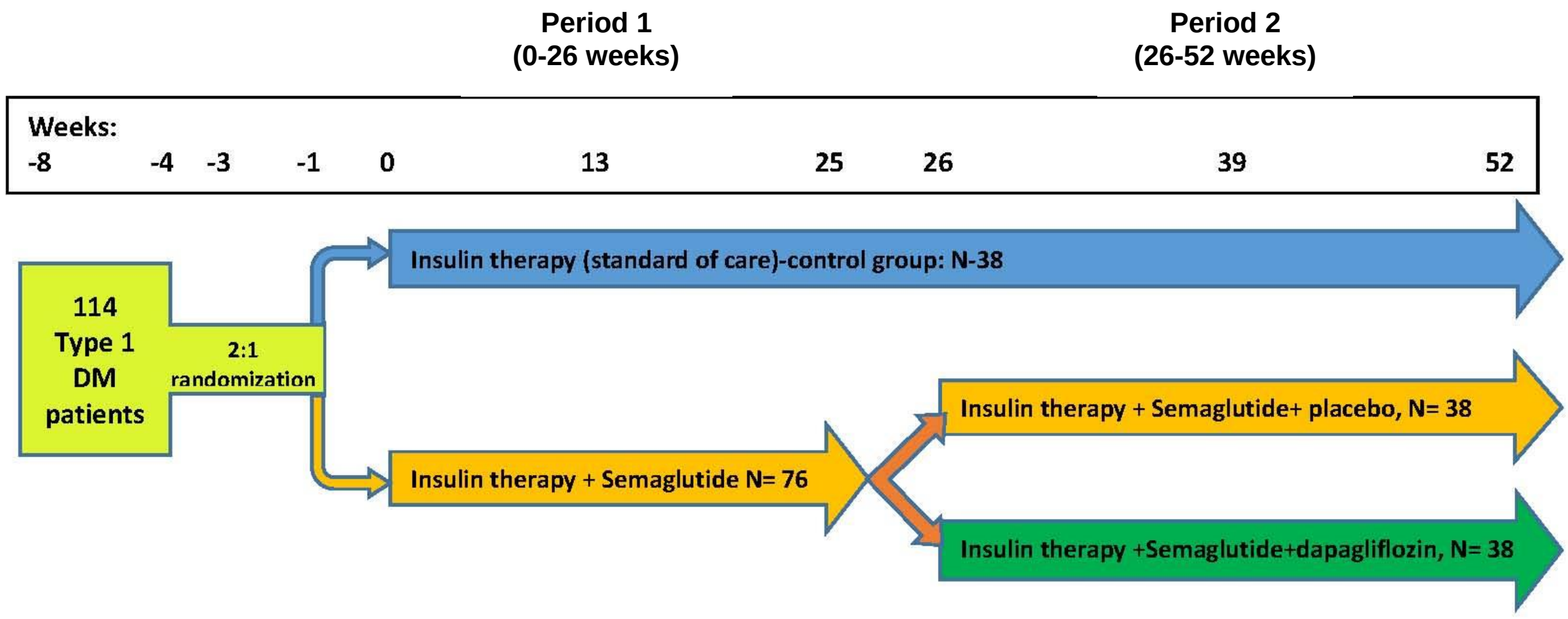

SCHEDULE OF ASSESSMENTS

**Visit #**	**-**	**1**	**2**	**3**	**4**	**5**	**6**	**7**	**8**	**9**	**10**	**11**	**12**	**13**	**14**	**15**	**16**
**Week# (±7 days; see below)**	**-8 to -4**	**-3**	**-1**	**0**	**1**	**2**	**4**	**13**	**23**	**25**	**26**	**27**	**31**	**39**	**49**	**51**	**52**
Study Procedures	Screening	Run-in period		Randomise							Randomise						End
Informed Consent	X																
Fasting visit			X	X				X		X	X			X		X	X
History and demographics	X																
Physical	X																X
Education on hypoglycaemia and ketosis avoidance)				X							X						
BP, heart rate, weight	X	X	X	X	X	X	X	X	X	X	X	X	X	X	X	X	X
Bloods (FBC, kidney and liver profiles)	X			X				X			X		X	X			X
C-peptide	X										X						X
Pregnancy test	X			X			X[1]	X[1]	X[1]		X[1]		X[1]	X[1]	X[1]	X[1]	X[1]
Dispense/ titrate open-label semaglutide[2]				X	X[2]	X[2]	X[2]	X[2]			X[2]			X[2]			
Dispense dapagliflozin/placebo											X	X		X			
Dapagliflozin/placebo return capsule counts												X		X			X
HbA1c	X			X				X		X				X			X
Blood endpoints (including fructosamine)				X				X			X			X			X
Masked CGM fit (enter data this visit)		X							X						X		
Masked CGM read				X							X						X
Daily fingerstick glucose review		X	X	X	X	X	X	X	X	X	X	X	X	X	X	X	X
Daily urinary ketones review												X	X	X	X	X	X
Insulin dose review		X	X	X	X	X	X	X	X	X	X	X	X	X	X	X	X
Ketone bodies (blood/urine)	X	X	X	X	X	X	X	X	X	X	X	X	X	X	X	X	X
Adverse Event reporting		X	X	X	X	X	X	X	X	X	X	X	X	X	X	X	X
Meal Challenge Test			X							X						X	
24 hr Urine dispensed			X				X			X			X			X	
24 hr Urine collected				X				X			X			X			X
Quality of life questionnaires				X							X						X

N.B. Safety phone calls will be conducted at weeks 3, 5, 8, 16, 20, 29, 35, 44 and at two weeks after the end of the study to document any side effects or other changes. **Abbreviations:** see pages 14-15

Visit dates should be within seven days of the visit time; however, a longer or shorter interval does not constitute a protocol deviation

[1]Not required in women randomised to "Insulin therapy (standard of care)

[2]According to randomisation schedule: resupply of open-label semaglutide after Visit 3 will be according to individual requirements as tolerated (see Section 8.3)

STUDY PROTOCOL
Triple Therapy for Type 1 Diabetes with Insulin, Semaglutide, and Dapagliflozin

1 BACKGROUND

The control of glucose homeostasis in type 1 diabetes is fragile since β-cell reserve is negligible. Traditionally, insulin has been the only treatment approach available. This often leads to erratic blood glucose control given the challenge of matching exogenous insulin to mealtime glucose excursions in the face of a lack of significant endogenous insulin to compensate for variation in requirements in response to meals and activity levels. Injected insulin boluses rarely match precise insulin requirements either in terms of dose administered or bio-availability. The result often is wide fluctuations in blood glucose including episodes when it is too low (hypoglycaemia). Based on our work to date and other published reports, previous attempts to add other glucose-lowering medications to insulin therapy yielded minimal benefits on glycaemia (although metformin may have other benefits) (1-3).

Dandona et al. have shown in two preliminary retrospective studies that the addition of liraglutide, a Glucagon Like Peptide -1 receptor agonist (GLP-1RA), to insulin results in consistent improvements in glycemic control in type 1 diabetes as demonstrated by reductions in HbA1c, glycemic excursions, and insulin requirements accompanied by improvements in body weight and systolic blood pressure (4, 5). As collaborators in the present trial, they also completed the first prospective randomised controlled trial of this drug in 72 people with type 1 diabetes. The data not only confirmed improvements in glycemic control (HbA1c), body weight and systolic BP, but also demonstrated a reduction in appetite, carbohydrate intake and low-grade inflammation as measured by plasma CRP concentrations (6). Importantly, quality of life was also improved. Their recent studies in type 2 diabetes have revealed that systolic blood pressure reduction with GLP-1RA (exenatide) treatment in type 2 diabetes is associated with increased plasma concentrations of ANP, cGMP and cAMP along with reduced concentrations of angiotensinogen, renin and angiotensin II (7). It is possible that these mechanisms play a role in the prevention of cardiovascular complications in type 1 diabetes. There have been no trials to date of semaglutide (a weekly and potent GLP-1RA) in type 1 diabetes.

GLP-1RAs, therefore, have great potential as adjunct therapy for type 1 diabetes. However, mean HbA1c was still ≥7.0% after liraglutide treatment (6) and larger pharma-sponsored trials (ADJUNCT-1 and ADJUNCT-2) reported increased rates of hypoglycaemia (8, 9). In seeking further improvement, the advent of Sodium-GLucose coTransporter 2 (SGLT2) inhibitors is of importance since their mode of action is independent of insulin secretion. Introduced for use in type 2 diabetes, SGLT2 inhibitors are selective antagonists of the sodium-glucose transporter subtype expressed in the proximal convoluted tubules of the kidneys. They act by inhibiting renal glucose re-absorption and therefore stimulating excretion of 60-100 grams per day of glucose in urine. SGLT2 inhibitors have been studied extensively in type 2 diabetes, including the landmark cardiovascular outcome trial EMPA-REG (10), but data have only more recently begun to accumulate in type 1 (11). Short term effects on urine glucose excretion of remogliflozin and empagliflozin are similar to those seen in type 2 diabetes and these translate into improved glycemic and other metabolic indices including HbA1c; fasting blood glucose, rates of hypoglycaemia, daily insulin dose and weight (12). Weight loss accompanying increased urinary glucose excretion could translate into reduced cardiovascular complications as in type 2 diabetes, but there are no data so far available to support this hypothesis. More recently, in the Phase 3 trials DEPICT-1 and DEPICT-2 Dandona et al (PI of the US arm of this trial) showed that dapagliflozin improves glycemic control and HbA1c over six months by about 0.45% without increasing hypoglycaemia. (13, 14, 15). In comparison to other SGLT2 inhibitors studied for use in type 1 diabetes [sotagliflozin and empagliflozin (16, 17)] the risk of diabetic ketoacidosis was relatively modest (see below). Both dapagliflozin and sotagliflozin were approved early in 2019 by the EU Committee for Medicinal Products for Human Use (CHMP) as adjunct therapy in type 1 diabetes for individuals with BMI≥27 kg/m^2.In August 2019, dapagliflozin (5 mg/ day) was recommended by the

National Institute for Health and Care Excellence as cost-effective for this indication in NHS England (17, 18, 19).

2 RATIONALE

General hypothesis: adding an SGLT2 inhibitor (dapagliflozin) to a combination of insulin and semaglutide will improve glycaemic control, weight and blood pressure in adults with type 1 diabetes. Our protocol is designed to allow an objective assessment of whether this triple therapy combination will work effectively and safely so that a majority of people with type 1 diabetes can reach a glycemic target HbA1c of ≤7.0%. In addition, since both semaglutide and dapagliflozin inhibitors reduce blood pressure and weight, it will allow an assessment of the combined effect of GLP-1RAs and SGLT2 inhibitors on these outcomes. Since there are no previous data on the effects of once weekly semaglutide in type 1 diabetes, or the combination of semaglutide and dapagliflozin compared to standard insulin treatment, the design requires allocation of some participants to insulin therapy only (the current standard of care).

As part of the process by which this trial was awarded funding by JDRF, this research question was considered by lay members of the funding panel and deemed important, relevant and timely. We consider that use of placebo matching dapagliflozin in Period 2 of the trial is justified as although it has full approval as adjunct therapy for type 1 diabetes, it remains uncertain which adults with type 1 diabetes are suitable for this treatment. In their review, NICE considered it likely that fewer than 10% of adults with type 1 diabetes will be initiated on SGLT2 inhibitor therapy over the first five years after their favourable decision; this is the period of time during which this trial is being conducted. As individuals with a slightly lower BMI threshold will be included than recommended by the SmPC, trial participants will be more closely monitored for ketosis than is the case in routine clinical care. Dapagliflozin (or placebo) will be administered orally once daily as per current licensed doses in type 2 diabetes (5 mg/day and 10 mg/day: the same doses as used in DEPICT-1 and DEPICT-2) (13,14,15, 20). We have not incorporated blinding for semaglutide in the design as the effects of the closely-related daily GLP-1RA liraglutide have been well-studied in double-blind trials in type 1 diabetes by ourselves and others, and individuals in both arms compared for the primary outcome comparison will be treated with this agent. (6, 8, 9). Semaglutide will be administered subcutaneously once weekly.

2.1 Assessment and management of risk

a) Benefits

Semaglutide (Ozempic): is a once weekly injectable (subcutaneous) GLP1-RA licensed for reducing HbA1c in type 2 diabetes. The licensed doses are 0.25 mg weekly, 0.5 mg weekly and 1.0 mg weekly. In the trial, the starting dose is 0.25 mg weekly uptitrated if tolerated to 0.5 mg weekly after two weeks and then (again if tolerated) after a further two weeks to the maximum dose of 1.0 mg weekly. Semaglutide differs from other weekly GLP-1 receptor agonists in that its half-life is extended by binding to serum albumin i.e. it is not macromolecular. In type 2 diabetes, semaglutide therapy is associated with weight reduction and a reduction in rates of cardiovascular events (21). The licensed dose escalation schedule for routine treatment of type 2 diabetes is at four weekly intervals but in a Phase 2 trial in type 2 diabetes authored by one of this trial's investigators (JP) doses were uptitrated at one-to-two weekly intervals and to higher doses (up to 1.6 mg weekly) (22). Phase 3 trials are currently in progress (for the indication of obesity) using a 2.4 mg semaglutide weekly. It should be noted that semaglutide 0.25 mg is considered too low a dose for maintenance therapy in type 2 diabetes but it is unclear whether this may have a beneficial effect in type 1 diabetes at this dose.

While semaglutide once weekly has not been investigated previously in type 1 diabetes, liraglutide (a GLP-1 RA for daily administration with a similar molecular stucture that binds less avidly to serum albumin) has previously been investigated (see above) in the ADJUNCT 1 and ADJUNCT 2 trials (8,9)

in which, compared with placebo it resulted in reductions in HbA1c, weight and insulin requirement, albeit with a small increase in rates of hypoglycaemia (see below).

Dapagliflozin (Forxiga): is a once daily oral SGLT2 inhibitor licensed in Europe for reducing HbA1c in people with type 1 diabetes and BMI ≥ 27 kg/m^2 when insulin alone does not provide adequate glycemic control despite optimal insulin therapy (23, 24). The licensed dose for type 1 diabetes is 5 mg once daily but both 5 mg and 10 mg daily are licensed for type 2 diabetes. In the Phase 3 trials of dapagliflozin in type 1 diabetes (DEPICT-1 and DEPICT-2) both doses were used. In both DEPICT-1 and DEPICT-2 there were modest additional benefits with 10mg compared to 5mg dapagliflozin. For example, in DEPICT-1, insulin dose requirement was reduced by 13.2% vs placebo on 10mg daily compared with 8.8% on 5mg daily (13). Weight reduction vs placebo in DEPICT-1 at 52 weeks was by 4.5% on dapagliflozin 10mg daily compared with 2.95% in those receiving 5mg daily. Given the propensity of insulin to encourage weight gain over time and the life-long insulin requirement in T1D, sustained weight reduction and insulin dose reduction - even when modest - may be clinically important. There was no increase in the rate of hypoglycaemia in either the 5mg or 10mg groups compared with placebo. (13,14)
Dapagliflozin therapy in type 2 diabetes has been demonstrated to be associated with cardiovascular risk reduction: in both DECLARE-TIMI and DAPA-HF the 10mg dose was used. Such cardiovascular benefits may translate to type 1 diabetes. Given the potential glycaemic and cardiovascular benefits with relatively low increased risk, 10mg rather than 5mg dose was selected for steady state treatment in this protocol. (25,26)

b) Risks/ side effects Please also refer to the respective UK SmPCs (21, 24)

<u>Semaglutide</u>:
Very common: nausea, diarrhoea; hypoglycaemia (when used with insulin).
Common: vomiting, abdominal pain, constipation, gastro-oesophageal reflux, cholelithiasis, weight loss.
Uncommon: injection site reactions, hypersensitivity
Rare: Analphylactic reaction.

Hypoglycaemia/ ketosis: Semaglutide once weekly has not been studied previously in type 1 diabetes; however, the ADJUNCT-1 and ADJUNCT-2 trials examined the role of liraglutide once daily (a similar molecule in the GLP-RA class that binds less avidly to serum albumin). Although HbA1c and weight were reduced by both 1.2 mg and 1.8 mg liraglutide, rates of symptomatic hypoglycaemia increased by 20-30%. In ADJUNCT-1, insulin doses were re-uptitrated quite rapidly towards baseline levels after an initial dose reduction, but in ADJUNCT-2 were "capped" (and therefore remained lower). Perhaps for that reason, an increase in hypoglycaemia was less consistent in ADJUNCT -2 but there was an excess of ketosis the higher (1.8 mg) dose, presumably due to under-insulinisation (no lower limit for insulin dose reduction was set). It should be noted that monitoring of participants by site investigators in the ADJUNCT trials was less intensive than will be the case in the present trial with less frequent opportunities for insulin dose titration (see Section 7). Importantly, participants at high risk of hypoglycaemia, (including those with a history of severe hypoglycaemia and those with hypoglycaemia unawareness) were not excluded (8,9).

Retinopathy: In the SUSTAIN-6 cardiovascular outcome trial of semaglutide in type 2 diabetes, rapid reduction of HbA1c was associated with progression of existing proliferative retinopathy in those with a high baseline HbA1c ("early worsening"/ "normoglycaemic re-entry"). However, there was no evidence of worsening of retinopathy on treatment in the trials SUSTAIN 1-5 in which those with proliferative retinopathy were excluded (22). In clinical trials of duration up to one year involving 4,807 patients with

type 2 diabetes, adverse events related to diabetic retinopathy were reported in similar proportions of subjects treated with semaglutide (1.7%) and comparators (2.0%) (21, 27).

Other adverse effects: In type 2 diabetes, gastrointestinal adverse effects of semaglutide can be prevented in the majority of cases by careful dose uptitration (21,22) although up to 5% are ultimately unable to tolerate it. In our previous trial with liraglutide in T1D, adverse effects occurred with a similar frequency to that observed in T2D (in which there is extensive clinical experience); on this basis it is expected that this will also be the case with semaglutide (28).

Dapagliflozin:
Very common: hypoglycaemia (when used with insulin).

Common: ketoacidosis, genital infections (vulvovaginitis, balanitis), urinary tract infection, diabetic ketoacidosis,.

In the DEPICT-1 study, ketoacidosis occurred in 4 of 277 participants on 5mg dapagliflozin, 5 of 296 participants on 10 mg and 3 of 260 participants in the placebo group (summarised as 1%, 2% and 1% respectively) with one severe episode in each group (13). In pooled analyses of DEPICT-1 and DEPICT-2 (13,14) there was, however, an approximate threefold elevated rate of DKA adjudicated as "definite" over placebo on active therapy: 2.0% on 5mg dapagliflozin, 1.9% on 10mg and 0.6% on placebo – i.e. rates of DKA were similar with the two doses (13,14,15). Rates of DKA with sotagliflozin, the other SGLT2 inhibitor licensed for type 1 diabetes, were somewhat higher (2.9% on 200 mg, 3.8% on 400 mg, 0.2% on placebo) in pooled analyses of Phase 3 trials (29,30,31).

Uncommon: fungal infection, volume depletion, constipation.

Not known (several case reports): Fournier's gangrene.

Fournier's gangrene is a very rare clinical entity characterized by necrotizing fasciitis of the perineal area. This has been infrequently reported in association with SGLT-2 inhibitor use in both men and women. However, there was no evidence of increased risk in a meta-analysis of 84 clinical trials involving 42415 participants. Nevertheless, participants will be advised to seek urgent medical assistance if they experience erythema, pain, swelling of the perineal area associated with fever and malaise (this will be reinforced in the Patient Information Sheet). (32)

Amputation: An increased incidence of lower limb amputation has been reported in type 2 diabetes with the SGLT-2 inhibitor canagliflozin. However this has not been demonstrated with dapagliflozin (not included in the UK SmPC). People with diabetes are at increased risk of amputation and good foot care is a key component of good diabetes care regardless of use of SGLT-2 Inhibitors (24).

Placebo:
Placebo capsules contain microcrystalline cellulose.
This trial is therefore categorised by the Co-Sponsors as:

- Type B = Somewhat higher than the risk of standard medical care

See Appendix 1 (Risk Assessment)

3 OBJECTIVES AND OUTCOME MEASURES/ ENDPOINTS

3.1 Primary objective

- To assess in people with type 1 diabetes whether triple therapy (dapagliflozin, semaglutide and insulin) for six months improves glycaemic control (as measured by HbA1c) compared with dual therapy (placebo, semaglutide and insulin – Period 2).

3.2 Secondary objectives

To assess in people with type 1 diabetes whether:

- T7riple therapy (dapagliflozin, semaglutide and insulin) improves glycaemic control (as measured by HbA1c) compared with standard therapy (insulin)
- Dual therapy (semaglutide and insulin – period 1) improves glycaemic control (as measured by HbA1c) in comparison with standard therapy (insulin only)
- Triple therapy (dapagliflozin, semaglutide and insulin) improves glycaemic control as assessed by a range of continuous glucose monitoring (CGM) parameters, lowers body weight, reduces insulin requirement, improves blood pressure and improves quality of life (as assessed by validated patient questionnaires) compared with dual therapy and standard therapy.

3.3 Outcome measures/endpoints

3.4 Primary endpoint/outcome

Change in HbA1c (week 26 to week 52)

3.5 Secondary endpoints/outcomes

Efficacy:
Change in HbA1c

Mean fasting blood glucose

Mean blood glucose*
Percentage time in range (3.9 - 10.0 mmol/)*
Standard deviation of blood glucose*
Percentage time in hyperglycaemia Level 1 (10.1 - 13.9 mmol/L/)*
Percentage time in hyperglycaemia Level 2 (>13.9 mmol/L) *
Percent time in hypoglycaemia Level 1 (3.0 - 3.8 mmol/)*
(by masked DEXCOM G6 CGM)*

Insulin requirement
Bodyweight
Systolic and diastolic BP
Number of antihypertensive medications
Diabetes Specific Quality of Life Scale (DSQOLS)
Problem Areas In Diabetes (PAID) questionnaire

Safety:
Rate of hypoglycemic events Level 2 (<3.0 mmol/L/ <54mg/dl)
Rate of hypoglycemic events Level 3 (severe event with altered mental and/or physical status requiring assistance)
Rate of diabetic ketoacidosis defined as elevated serum or urine ketones (greater than the upper limit of the normal range), and serum bicarbonate < 15 mmol/L or blood pH < 7.3

3.6 Tertiary endpoints/outcomes

Postprandial glucose (AUC) – meal tolerance test
Postprandial glucagon (AUC) – meal tolerance test
Postprandial FFA (AUC) – meal tolerance test
Postprandial GLP-1 (AUC) – meal tolerance test
Fructosamine

3.7 Exploratory endpoints/outcomes

Plasma and urine samples will be collected and frozen for later analysis for indices of inflammatory stress and other relevant measures.

3.8 Table of endpoints/outcomes

Endpoints	Outcome Measures	Timepoint(s) of evaluation of this outcome measure
**Primary endpoint**<br>Triple therapy (dapagliflozin, semaglutide and insulin) in comparison with dual therapy (placebo, semaglutide and insulin – period 2)	Change in HbA1c	Week 26 to Week 52
**Secondary endpoint 1**<br><br>Triple therapy (dapagliflozin, semaglutide and insulin) in comparison with standard therapy (insulin alone)	Change in HbA1c	Week 0 to Week 52
**Secondary endpoint 2**<br><br>Dual therapy (semaglutide and insulin – period 1) in comparison with standard therapy (insulin only)	Change in HbA1c	Week 0 to Week 26
**Other secondary endpoints**<br><br>a) Triple therapy (dapagliflozin, semaglutide and insulin) in comparison with dual therapy (semaglutide, insulin and placebo – period 2)<br><br>b) Dual therapy (semaglutide and insulin – period 1) in comparison with standard therapy (insulin only)	Change in:<br>- Mean fasting blood glucose (4 days)<br><br>- Mean blood glucose*<br>- Percentage time in range (3.9–10 mmol/L)*<br>- Glycaemic variability (SD)*<br>- Percent time in hyperglycaemia Level 1 (10-13.9 mmol/L)*<br>- Percent time in hyperglycaemia Level 2 (>13.9	Week 26 to Week 52<br><br>Week 0 to Week 26

	mmol/L)*<br>- Percent time in hypoglycaemia Level 1* (3.0-3.8 mmol/L)<br>*(*by masked Dexcom G6 CGM)*<br><br>Insulin requirement<br>Body Weight<br>Systolic and diastolic blood pressure<br>Number of antihypertensive medications<br>Diabetes Specific Quality of Life Scale (DSQOLS)<br>Problem Areas In Diabetes (PAID) questionnaire	
**Tertiary endpoints**<br>a) Triple therapy (dapagliflozin, semaglutide and insulin) in comparison with dual therapy (semaglutide, insulin and placebo –period 2)<br><br>b) Dual therapy (semaglutide and insulin – period 1) in comparison with standard therapy (insulin only)	Change in:<br>Postprandial glucose (AUC)<br>Postprandial FFA (AUC),<br>Postprandial GLP-1 (AUC)<br>Postprandial glucagon (AUC)	Week 26 to Week 52<br><br>Week 0 to Week 26
**Safety endpoint 1**<br>a) Triple therapy (dapagliflozin, semaglutide and insulin) in comparison with dual therapy (semaglutide, insulin and placebo – period 2)<br><br>b) Dual therapy (semaglutide and insulin – period 1) in comparison with standard therapy (insulin only)	Difference between treatment arms in:<br>(i) Rate of Level 2 (<3.0 mmol/L/ <54mg/dL) hypoglycemic events (by Dexcom G6 CGM)<br>(ii) Rate of Level 3 hypoglycemic events (severe event with altered mental and/or physical status requiring assistance)	Week 26 to Week 52<br><br>Week 0 to Week 26
**Safety endpoint 2**<br>a) Triple therapy (dapagliflozin, semaglutide and insulin) in comparison with dual therapy (semaglutide, insulin and placebo – period 2)	Difference between treatment arms in:<br><br>Rate of diabetic ketoacidosis (serum or urine ketones above normal limits) AND serum bicarbonate < 15 mmol/L OR blood pH < 7.3	Week 26 to Week 52

b) Dual therapy (semaglutide and insulin – period 1) in comparison with standard therapy (insulin only)		Week 0 to Week 26

4 TRIAL DESIGN

The trial has two six-month periods:

Period 1 open-label randomised (2:1) parallel group semaglutide once weekly + insulin (standard treatment) vs insulin alone (standard treatment).
Period 2 double-blind, randomised (1:1) parallel group, dapagliflozin vs placebo for those participants initially randomised to semaglutide (with ongoing standard of care for all participants)

Thus, during the first six months, approximately 114 participants with type 1 diabetes (57 in the UK) will be randomised (open label, see below) into three groups (approximately 38 each; i.e. 19 each in the UK) one of which will be maintained on their regular insulin therapy for 12 months (standard therapy group) and two of which will be started on semaglutide in addition to regular insulin treatment for six months (dual therapy). Following six months of stable treatment, those initially randomised to insulin and semaglutide will be additionally randomised (double-blind) to dapagliflozin (triple therapy) or matching placebo (dual therapy) for a further six months. The dose of dapagliflozin will not be increased to 10 mg in those who have confirmed ketonuria (+1) or ketonaemia (≥0.6 mM) after the first week of treatment.

In keeping with the intention-to-treat principle, participants in whom therapy is discontinued or downtitrated due to adverse effects will be encouraged to continue to undertake all study assessments and attend study visits.

5 STUDY SETTING

The study will be conducted at two sites: State University of New York (SUNY) at Buffalo, USA and Glasgow, UK. In Glasgow, n=57 participants (50% of the total) will be identified from secondary care sites and via the Scottish Diabetes Research Network research register. Both randomisations will be stratified by site.

6 ELIGIBILITY CRITERIA

6.1 Inclusion criteria

1) Type 1 Diabetes for at least one year on stable multiple daily (four or more) injections (MDI) of insulin or continuous subcutaneous insulin infusion (CSII) for the previous three months.
2) C-peptide < 0.23 nM
3) Minimum dose of insulin: 0.5 U/kg for MDI and 0.4 U/kg for CSII
4) Regularly measuring blood glucose: four or more times daily either by fingerstick, testing or by "flash"/ continuous glucose monitoring)
5) HbA1c 7.5 – 11.0 % inclusive
6) Well versed in carbohydrate1 counting*

7) Age 18-70 years.
8) BMI ≥25 kg/m^2.

** In the opinion of the site Principal Investigator*

6.2 Exclusion criteria

1) Type 1 diabetes for < 12 months, type 2 diabetes, chronic pancreatitis, MODY
2) Previous use of any agent other than insulin for glycaemic control in the last three months.
3) History of diabetic ketoacidosis (DKA) requiring medical intervention (e.g. emergency room visit and/or hospitalisation) within three months prior to the screening visit.
4) Following ketogenic diet, low (<50g/day) carbohydrate diet or intermittent fasting.
5) Hypoglycaemia unawareness* or frequent episodes of severe hypoglycaemia as defined by more than one episode requiring medical assistance, emergency care (paramedics or emergency room care), or glucagon therapy administered by a third-party individual within three months prior to the screening visit
6) Symptoms of poorly controlled diabetes
7) Participation in a weight loss program with ongoing weight loss, or in an intensive exercise program
8) History of bariatric or other weight-loss surgery within 12 months prior to the screening visit
9) History of Addison's disease or chronic adrenal insufficiency
10) History of diabetes insipidus
11) Hepatic disease or cirrhosis
12) Aspartate Aminotransferase (AST) > 3 X Upper limit of normal (ULN) and/or Alanine aminotransferase (ALT) > 3 X ULN
13) Serum Total Bilirubin > 2 X ULN unless exclusively caused by Gilbert's Syndrome
14) Haemoglobin < 110 g/L (11.0 g/dL) for men; haemoglobin < 100 g/L (10.0 g/dL) for women
15) Coronary event or procedure (myocardial infarction, unstable angina, coronary artery bypass, surgery or coronary angioplasty) in the previous three months or history of congestive heart failure
16) End stage renal disease on haemodialysis and/or eGFR < 60 ml/min/1.73m^2
17) HIV or Hepatitis B/C positive status
18) Any other life-threatening, non-cardiac disease
19) History of pancreatitis or cystic fibrosis
20) Women who are pregnant, planning pregnancy or breast-feeding and women of childbearing potential (WOCBP) who are not established on a method of contraception considered highly effective (see Appendix 2)and willing to continue it for the duration of the study and for at least two months after last dose of IMP.
21) Inability to give informed consent
22) History of gastroparesis
23) History of medullary thyroid carcinoma or MEN 2 syndrome
24) History of hypersensitivity reaction to GLP-1 agonists or SGLT2 inhibitors or known hypersensitivity to any of the excipients
25) Known gallstones (history of cholecystectomy is not an exclusion criterion)
26) History of alcohol problem or drug abuse
27) Hypertriglyceridemia (>500 mg/dl)
28) Recurrent genital mycotic infection (more than one episode in last six months)

29) Hypovolaemia
30) Any malignancy except treated *in situ* malignancy and basal cell carcinoma of the skin
31) Unexplained haematuria (dipstick positive on two occasions one week apart) and/or active bladder cancer
32) Proliferative retinopathy (on basis of photographic retinal screening which must have been performed within the last 12 months
33) Use of an investigational agent or therapeutic regimen within 90 days of study
34) Participation in any other concurrent interventional clinical trial
35) Unlikely to be able to comply with study requirements*

** In the opinion of the site Principal Investigator*

36) Unable to undergo meal challenge for dietary reasons (e.g. vegetarian/ vegan diet)

7 TRIAL PROCEDURES

Also see *Schedule of Assessments*

Each participant will be enrolled in the trial for approximately 55 weeks, comprising three weeks of monitoring to stabilise treatment and collect baseline data before randomisation into the intervention or standard of care/ placebo arms for 52 weeks. Following a completed screening visit and meeting the Inclusion and Exclusion criteria, each participant will have a total of 16 scheduled study visits. In addition, at weeks 3, 5, 8, 16, 20, 29, 35, 44 and at two weeks after the end of the study, each participant will receive a phone call to document any adverse effects or other changes. No long-term follow-up is planned following completion of participation in the study.

Participants will be treated with either insulin only (standard treatment) for the whole duration of the study or with insulin and semaglutide weekly s.c. injection (titrated where possible up to 1.0 mg, see below) for the first six month period and then randomised to either oral dapagliflozin (triple therapy) or placebo (dual therapy) daily added for the second six month period. Semaglutide will be started (Visit 4) at a dose of 0.25 mg once weekly for two weeks then increased if tolerated to 0.5 mg (Visit 5). If well tolerated at week 4, it will be increased to 1.0 mg once weekly (Visit 6) or at a subsequent visit at the discretion of the PI. Dapagliflozin (or placebo) will be started at a dose of 5 mg daily at week 26 and increased at week 27 to 10 mg (2 x 5mg capsules) daily to ensure drug tolerability and minimise the risk of insulin-induced hypoglycaemia or hypotension.

Excessive weight loss: Both semaglutide and dapagliflozin are known to cause weight loss in the range of 5-10% in type 2 diabetes. However, the magnitude of weight loss when these are combined in type 1 diabetes is uncertain. Excessive weight loss may lead to increased risk of hypoglycemia or decreased insulin requirement increasing the risk of ketosis or DKA (see Section 2.1b) and other unforeseen risks. Therefore, weight loss during any phase of the study will be limited to ≤ 15% of body weight at randomisation or reaching a BMI ≤ 22Kg/m^2. If participants are approaching this threshold, IMP will be sequentially reduced at the discretion of the PI. Semaglutide may be reduced to 0.25mg or 0.5mg and dapagliflozin to 5 mg. See also Section 7.8 regarding treatment discontinuation.

Covid-19 Mitigation

In view of the Covid-19 pandemic, certain trial procedures may be changed if necessary to reduce in person contact with participants. This will be continuously reviewed in view of the prevailing circumstances in line with local and national guidance and with the principle of protecting participants and study personnel. In the event of future waves of infection this would be particularly important. In this context, consideration would be given to rearranging visits, changing to virtual visits using video technology or using additional phone calls to ensure patient safety. Consideration will also be given to how minimising face-to-face interaction with participants when dispensing study medication. If required, supplies of study medication sufficient to last for longer periods may be dispensed to minimise contact and visits. Courier services may additionally be used to deliver study medications to participants.

All in person visits will be subject to local and national guidance around the use of personal protective equipment including face masks and where appropriate when conducting procedures, the use of gloves and aprons.

Gastrointestinal adverse effects:

During the initial weeks of participation, those participants who report nausea and/or vomiting during uptitration of semaglutide dose that is not sufficiently severe to require treatment discontinuation will

remain on their current dose until they are symptom-free prior to discussing a further dose escalation (i.e. uptitration will be to the highest dose tolerated rather than a "forced titration"). Dose will be downtitrated to the maximum tolerated in those who experience gastrointestinal adverse effects.

Retinopathy:
As there was no excess of retinopathy with semaglutide treatment in Phase 3 trials with semaglutide, in which proliferative retinopathy was an exclusion criterion (as in the current protocol), additional retinal imaging beyond annual retinal photography (the standard of care) is not proposed (27).

Insulin dose adjustment:
Minimising hypoglycaemia: All participants will be made aware of the potential for increased hypoglycaemia with addition of adjunct therapy and supported and encouraged to monitor blood glucose at least four times daily in order to minimize the risk of hypoglycaemia. Participants will have ready access to advice from specialist medical and nursing staff at face-to-face and telephone visits. Those who have ongoing access to wearable continuous glucose monitoring [or "flash" glucose monitoring, (FGM)] will continue to use these devices. Information on management of hypoglycaemia will be included within participant diaries, reinforcing the pre-existing knowledge of participants, the majority of whom will have lived with type 1 diabetes for many years.

Reductions in insulin dose will not usually be greater than 10% (5% if HbA1c is < 7.0 %) from current levels. At randomisation, daily insulin dose will not be reduced by more than 20% from the baseline level and then from the new baseline levels at 26 weeks. Participants requiring an insulin dose reduction > 20% to a total insulin dose < 0.4 U/kg (current weight) will be monitored closely for insulin dose, carbohydrate intake, ketones and blood glucose. Following the second randomisation at 26 weeks, dapagliflozin/ placebo will be temporarily discontinued if insulin dose falls below this threshold and permanently discontinued if it remains so at review in one week.

Minimising ketosis: Participants will be instructed to stay hydrated and not to reduce their caloric intake (especially carbohydrates) or start new dietary approaches (especially ketogenic diets) during the study. They will be educated to recognize the signs and symptoms of diabetic ketoacidosis (DKA) and to undertake additional self-testing of urine and/or blood ketones if they develop nausea, vomiting, or anorexia. In addition, they will be given education and a written ketosis management plan (STICH Protocol – i.e. STop SGLT-2 inhibitor or placebo (temporarily), give Insulin bolus, take additional Carbohydrate, and additional Hydration - see Appendix 3).

From Visit 10 (26 weeks), those participants undergoing the second randomisation will be supported and encouraged to monitor urine or blood ketones at home at least once daily: three days before visit 10 (week 26), they will be asked to start daily measurements of urine ketones [CombiScreen® 10 SYS Plus urine testing strips, Sterilab, UK (or equivalent)] and blood ketones [CareSens™ Dual, i-SENS, Koreal; GlucoMen® Areo 2K, Menarini, UK (or equivalent]]. They will be asked to record these in the diary/ log provided. In addition, ketones (blood and urine) will be measured at every visit. If ketone bodies in urine are +1 or greater (≥0.6 mM in blood), participants will be reminded to keep well hydrated and notify the study team as soon as possible (office hours) as diabetic ketoacidosis (DKA) can occur even at glucose levels < 14 mmol/L (34).

If ketones are +2 or more in urine (> 1.5 mmol/L) participants will be advised to initiate the STICH Protocol immediately and contact the study team in office hours (or telephone NHS 24 or attend their Accident & Emergency if out of hours). This information will be reinforced verbally at study visits and also in the form of a Patient Alert Card that participants will be asked to carry. This will also contain details of the investigational study drug, the study number, the investigator's name, and a contact number

(for in hours contact). The study team will assess for symptoms or test results indicating a risk of ketoacidosis immediately on reporting and if blood ketone levels are persistently (one week) at these levels dapagliflozin/placebo will be permanently discontinued (24).

If blood ketone readings are confirmed as > 3.0 mmol/L (or urine ketones reading +++) participants will be advised to stop dapagliflozin/ placebo and attend the Accident and Emergency department without delay.

Participants will be advised that dapagliflozin/placebo may be temporarily stopped in other clinical situations known to predispose to ketoacidosis (e.g. acute serious medical illnesses or hospitalisation for major surgical procedures). Any requirement for intravenous insulin infusion (whether in Accident and Emergency or as an in-patient admission) will be recorded as a Serious Adverse Event. For further detailed procedures see section 9.31 (25).

Volume depletion:
There is a theoretical risk of volume depletion and low blood pressure when dapagliflozin is used in combination with other anti-hypertensive drugs. All pre-existing concomitant medication will be reviewed at the screening visit as part of the medical history and examination (see Section 7.1.3 for further details). Participants undergoing the second randomisation will be educated on symptoms suggestive of low blood pressure (e.g. light-headedness, dizziness, feeling faint) and asked to contact the study team if they experience these symptoms. In clinical practice however SGLT-2 inhibitors are commonly used with anti-hypertensive drugs with no significant problems (27).

Genital mycotic infections:
Individuals who report symptoms suggestive of genital mycotic infection will be advised to self-treat with topical antifungal preparations. If a second episode occurs, a decision on treatment discontinuation will be considered in discussion between the participant and the site Principal Investigator, taking account of the severity of the symptoms.

Foot care:
Annual foot checks will be conducted for all participants as per standard of care.

7.1 Recruitment

7.1.1 Patient identification

All personal details will be managed according to ISO 27001:2013 compliant Standard Operating Procedures.

Potentially eligible participants will be identified from the Scottish Diabetes Research Network Research Register (a managed and searchable database of people with diabetes who have agreed to be contacted about clinical trial participation) and at secondary care diabetes clinics in Greater Glasgow and Clyde. The principal site for recruitment will be Stobhill Ambulatory Care Hospital but other sites may be opened if necessary as Patient Identification Centre sites. The majority of study visits (and all visits involving drug dispensing) will be conducted at the Clinical Research Facility, Glasgow Royal Infirmary. Information posters with the contact details of the research team will be displayed in recruiting clinics.

Clinic lists will be pre-reviewed by members of the usual care team in order that potentially eligible individuals can be sent an abbreviated Participant Information Sheet by post one week before their routine scheduled review visit. Eligibility criteria of those indicating agreement to be approached will

then be checked at the routine visit, and the study procedures explained. Potential participants will be given a Patient Information Sheet and an Expression of Interest Form (with prepaid envelope) at this time and will be asked for permission to contact again to discuss further and (if appropriate) arrange a screening visit.

Covid-19 Mitigation: The delivery of outpatient clinics has been substantially changed due to the pandemic. The use of telephone clinics is now standard for many visits and video technologies will soon also be used. These mediums will therefore be used for inviting participants to take part when in person appointments are not taking place. This may be subject to change depending on the situation with Covid-19. Other principles of recruitment will be as described above.

7.1.2 Screening

A separate non-fasting visit will be arranged at which potential participants will have further time to discuss with the study nurse and doctor.

Prospective participants will be asked to read and understand the Participant Information Sheet. Any questions regarding the Protocol will be answered. Risks and side-effects of the active trial medication will be explained. Eligibility for trial enrolment will be confirmed by a medical practitioner.

Individual wishing to participate in the study will then be invited to sign to document their informed consent (see Section 7.2 below).

7.1.3 Screening assessments

Items from the medical history relevant to the Inclusion and Exclusion criteria will be recorded. Demographic data, medical history and current medications (including current insulin doses) will be documented. All participants will have access to a dual blood glucose and ketone meter as per the contemporary standard of care in type 1 diabetes. A physical examination will be conducted. Clinical observations will be recorded and blood and urine samples obtained (see below):

Demographics

- Date of birth
- Sex
- Ethnicity
- Date of diagnosis
- Ethnic group: white/black/Asian/other
- Height

Clinical assessments

- Systolic and diastolic blood pressure (after 5 minutes rest)
- Heart rate
- Body weight (clothed without coat and shoes)
- Capillary blood glucose

Medical History

- Smoking status: current/ex/never
- Alcohol consumption: units/week
- Hypertension: Y/N
- Microalbuminuria: Y/N
- Proteinuria: Y/N
- Neuropathy: Y/N
- Previous major cardiovascular event(s): Y/N (date)

- Existing CV disease of any type*: Y/N
- Amputation above forefoot: Y/N
- Hospitalisation for DKA in last year
- Level of educational attainment: University (college) educated Y/N

* Existing CV Disease is defined as any of: MI, stroke, ACS/unstable angina, coronary stent/angioplasty, CABG, TIA, heart failure, peripheral vascular disease

<u>Insulin treatment</u>

- Regimen: MDI, pump,
- Total dose (all); Basal (longer acting) dose/ Bolus (shorter acting) dose (for MDI only)

<u>Other treatment (drug classes and names)</u>

Current use Y/N. If no, has there been use in last three months?: Y/N

- ACE inhibitors
- Angiotensin receptor blocker
- Calcium channel blocker
- Alpha-blocker
- Beta-blocker
- Loop diuretic
- Thiazide like diuretic
- Statin
- Aspirin
- Clopidogrel
- Novel anticoagulant
- Warfarin

<u>Blood assessments</u>:

- Blood ketones (from participant's own ketone meter)
- HbA1c
- FBC
- Urea and electrolytes
- Creatinine
- Liver function tests (AST/ ALT/ Alkaline Phosphatase)
- Bone profile (Calcium/ Phosphate)
- C-peptide (capillary blood glucose must be > 4.0 mmol/L at time of sampling)

If blood tests have been assessed for clinical purposes within the last three weeks, these values will be used.

<u>Urine sample</u>:

- Urine ketones
- For all WOCBP, a urine pregnancy test will be checked at screening

Potential participants meeting all the inclusion and exclusion criteria based on history, examination and screening tests will be enrolled in the study.

Pregnancy: WOCBP will be asked about pregnancy status and contraceptive usage (see Appendix 2) and a pregnancy test will be conducted (following informed consent) prior to randomisation. We will not recruit those who are pregnant, wanting to become pregnant, or using birth control methods not considered highly effective. Every four weeks following randomisation, WOCBP randomised to IMP (as opposed to standard of care) will be asked about their contraception status, and a urine pregnancy test carried out. Study medication will be permanently discontinued in women who become pregnant or considered to be on contraception that is not highly effective. As the study assessments *per se* do not carry an additional risk over standard of care to either mother or unborn child, further pregnancy tests will not be routinely offered following randomisation to those women allocated to “Insulin therapy (standard of care)” (i.e. who are not exposed to IMP). All pregnancies will be notified to the Sponsor Pharmacovigilance Officer using the standard pregnancy notification form and the pregnancy followed to outcome).

7.1.4 Payment

Participants will be offered reasonable travel expenses for visits additional to usual care.

7.2 Consent

Potential participants will be identified and screened by the Inclusion and Exclusion criteria listed above. Medical staff or appropriately trained support staff will seek consent for screening and participation in the trial from individuals that fulfil the criteria. All potential participants will have at least 24 hours to review the Patient Information Sheet before being approached (see Section 7.1.2 above). Following written consent, each signature will be dated by the signatory, the original retained in the site file, a copy provided to the participant and a copy scanned into the participant's Clinical Portal record.

Data collected for routine clinical care will be used for clinical trial documentation (e.g. blood results within the previous three weeks). In the absence of routine blood results, sampling of blood for study specific laboratory measurements can only be performed following documentation of informed consent.

7.2.1 Re-screening post-consent

Where inclusion (e.g. HbA1c ≤7.5%) or exclusion (e.g. DKA within the last three months) criteria are not met (e.g. blood results falling outside the study parameters) potential participants can be re-screened with additional blood tests on one further occasion. If the initial consent was signed within two months of the re-testing, new consent is not required unless the consent form template has been updated in this period. If the initial consent was not signed within two months of re-testing then consent should be sought again. Rescreened participants will keep the same Patient ID and data will be amended on the web portal as appropriate.

7.3 Run-in period

7.3.1 Visit 1 (@3 weeks prior to randomisation)

Participants who meet entry criteria will be scheduled for their next visit at approximately three weeks prior to randomisation. Daily fingerstick blood glucose levels (study diaries), CGM/ FGM data or insulin

pump download data (as applicable) will be reviewed and appropriate insulin dose adjustments made by the medical team to target fasting blood glucose concentrations between 5 -10.0 mmol/L if possible at all times without having additional hypoglycaemia (< 3.9 mmol/L). Clinical observations will be recorded including resting BP measurement. Blood glucose and ketone bodies will be measured via a fingerstick blood sample (see Section 7). Urine for pregnancy status and ketone bodies will be assessed.

Participants will be instructed on the use of the Continuous Glucose Monitoring System (Dexcom G6 with blinded receiver). All subjects will be advised to monitor their capillary blood glucose by CGM or fingerstick at least 4 times a day (before meals and at bedtime) throughout the study and to wear their CGM constantly for next 20 days (changing the sensor at 10 days). CHO counting technique will be reviewed and appropriate support given with calculating bolus insulin doses.

<u>Visit procedures:</u>

<u>Monitoring and treatment optimisation</u>

- Check for adverse events
- Review blood glucose diary
- Blood ketones (from participant's own ketone meter
- Adjust insulin doses (if necessary)

<u>Clinical assessments</u>

- Blood pressure
- Heart rate
- Weight

<u>Urine sample</u>:

- Check urine ketones

<u>Other</u>

- *Start* Continuous Glucose Monitoring with Dexcom G6

7.3.2 Visit 2 (1 @ weeks prior to randomisation)

Fasting visit* - Participants will be asked to fast (no food or calorie intake) for 10 hours prior to the visit overnight. Those who plan to drive to fasting visits will be reminded of the increased risk of hypoglycaemia and DVLA guidelines. This will be repeated prior to each fasting visit and reiterated within the patient diaries. This visit will be scheduled for a morning appointment and participants will be advised not to take their breakfast bolus insulin dose prior to arrival. If participants arrive at the study visit in a non-fasted state, the visit will be terminated and a further fasting visit rearranged as soon as practicably possible.

Daily fingerstick blood glucose levels (diaries), CGM/ FGM data or insulin pump download data (as applicable) will be reviewed and appropriate insulin dose adjustments made. Clinical observations will be recorded as detailed below. Blood glucose and ketone bodies will be measured via a fingerstick blood sample (see Section 7). If blood glucose is less than 3.9 mmol/L, or more than 13.9 mmol/L, the visit will be rescheduled for an alternative date. Otherwise, an intravenous cannula will be placed in order to obtain blood samples for the duration of the visit. A Meal Challenge Test will be performed (described below). A 24 hr urine container will be dispensed to collect a 24 hour urine sample to bring

in at the Week 0 visit. For this collection and all subsequent 24 hour urine collections, urine will be tested via dipstick testing and an aliquot retained for assay of albumin, creatinine, ketones, and 15-isoprostane.

Visit Procedures

Monitoring and treatment optimisation

- Check for adverse events
- Review blood glucose diary
- Blood ketones (from participant's own ketone meter
- Adjust insulin doses (if necessary)

Clinical assessments

- Blood pressure
- Heart rate
- Weight

Urine sample:

- Check urine ketones
- 24 hour urine container dispensed

Other assessments

- Download Dexcom G6 CGM
- High fat high calorie Meal Challenge: in order to assess postprandial metabolism a Meal Challenge Test will be performed using an approximately 900 kilocalorie High Fat High Carbohydrate (HFHC) meal including an egg muffin sandwich, a sausage muffin sandwich and two hash browns [containing approximately 88g carbohydrate, 51 g fat (33% saturated) and 34 g protein]. Participants will be asked to attend fasting prior to breakfast insulin bolus (with continuing basal insulin); participants will be asked not to take bolus insulin until the 3 hour time point unless blood glucose values reach above 22.2 mmol/L. Blood samples will be collected from an indwelling intravenous canula in a superficial forearm vein for glucose, glucagon, FFA, GLP-1 (AUC). Sequential blood samples will be obtained at 0, 15, 30, 45, 60, 90, 120, 150 and 180 min after consuming the meal. Volume of samples at 15, 30, 45, 90 and 150 min will be 5ml while those at 0, 60, 120 and 180min will be 30 ml (total volume = 145 ml). A spot urine sample will be collected at baseline, 1 and 3hr for standard urinalysis and an aliquot retained for assay of ketones and 15-isoprostane.

7.4 Randomisation

Participants who are being randomised will be required to have undergone all screening assessments.

At the randomisation visit, study participants will be provided with a Patient Alert Card. There are two Versions: (1) for the intervention arm i.e. those allocated at first randomisation to open-label semaglutide who are also eligible for the second double-blind randomisation (dapagliflozin or placebo); and (2) for those allocated to standard of care. These cards supplement the study diaries and include contact information for the study team. Version 1 contains unblinding information. Participants will be asked to carry their card at all times. These will be collected at the end of participation in the study.

7.4.3 First Randomisation Scheme

For the first randomisation (Period 1, open label), eligible and consenting participants will be randomised (2:1, stratified by site) to the two groups (open-label semaglutide or standard of care) using permuted blocks of variable size to minimize predictability.

7.4.4 Method of implementing the allocation sequence

Randomisation will be achieved by accessing an interactive web-based randomisation system (IWRS). The investigator will provide the participant identifier and the system will check the participant's eligibility from information already entered in the electronic Case Report Form (eCRF): if appropriate the randomisation group will be allocated.

7.5 Visit 3 (First Randomisation Visit)

Participants will be asked to attend in the fasting state (no food or calorie intake for 10 hours; see 7.3.2 above). Blood glucose levels and ketone bodies will be measured via a fingerstick blood sample using a glucose and ketone meter (see Section 7) and daily fingerstick blood glucose levels (diaries) collected and reviewed. The particpant's own CGM/ FGM data or insulin pump download data will also be reviewed. Daily insulin dose will be adjusted following clinical review of study diaries: if reduced, this should not be by more than 20% from the baseline level Participants will discontinue use of the study masked CGM (Dexcom G6). Clinical observations will be recorded. Blood glucose and ketone bodies will be measured via a fingerstick blood sample (see Section 7). The 24 hour urine container will be collected. A fasting blood sample will be taken (45 mL) to evaluate study endpoints, FBC, U&E, LFT, bone profile, lipids, HbA1c, and fructosamine (detailed below). A urine sample will be collected to evaluate ketone bodies and, in females, pregnancy status.

Participants will be asked to complete Quality of Life questionnaires (see Appendices).

IMP will be dispensed for those randomised to open-label semaglutide (once weekly). Semaglutide will be started on site at the 0.25 mg dose. Participants will be instructed to call site staff directly in case of any problem or untoward side effects. An office hours phone number will be provided for advice. Outside these hours, provision of emergency medical care if required will be via NHS 24 and/or Accident & Emergency. Participants will be specifically asked to call if they have hypoglycaemia (blood glucose < 3.9 mmol/L) or Level 2 hyperglycaemia (blood glucose >13.9 mmol/L), particularly if occurring on more than one occasion. This will apply for the remainder of the trial.

Visit Procedures

Fasting visit* as per visit 2.

<u>Monitoring and treatment optimisation</u>

- Check for adverse events
- Check adequacy of contraception (WOCBP)
- Review blood glucose diary
- Adjust insulin doses (if necessary)
- Blood ketones (from participant's own ketone meter)

<u>Clinical assessments</u>

- Blood pressure
- Heart rate
- Body weight

Quality of life assessments

- Diabetes Specific Quality of Life Scale (DSQOLS)
- Problem Areas In Diabetes (PAID) questionnaire

Blood assessments

- Na, K, urea, creatinine, eGFR (MDRD)
- AST/ ALT/ Alkaline Phosphatase
- Calcium/ Phosphate
- CRP
- HbA1c
- Fructosamine
- Haemoglobin
- White cell count
- Platelets
- Bilirubin
- Albumin
- Random glucose

Urine

- For WOCBP, a urine pregnancy test will be checked prior to first administration of IMP.
- Urine ketones
- 24 hour urine sample collected

Medication

- Administer open label semaglutide 0.25 mg if randomised to active treatment
- Issue Patient Alert Card (appropriate version according to randomisation)

Bloods for storage

Approximately 45 mls of venous blood will be withdrawn and collected in tubes containing EDTA. Blood will be centrifuged at 1500g for 20 mins at 4ºC. Plasma will be siphoned, aliquoted and stored locally at -80° ± 10°C until transport in batches to the central laboratory on dry ice.

7.5.1 Visit 4 (@1 week after first randomisation)

Clinical observations will be recorded. Blood glucose levels and ketone bodies will be measured via a fingerstick blood sample using the participant's own glucose and ketone meter (see Section 7)) and daily blood glucose levels collected and reviewed. Insulin doses adjusted as in visit 3. If HbA1c is <7.0 %, insulin dose reduction should not be >5% from baseline total dose. Semaglutide 0.25mg will again be injected on site for those randomised to active therapy.

Visit Procedures

Monitoring and treatment optimisation

- Check for adverse events
- Review blood glucose diary
- Adjust insulin doses (if necessary)
- Blood ketones (from participant's own ketone meter

Assessments

- Blood pressure
- Heart rate
- Weight

Urine sample:
- Urine ketones

Medication
- Support participant's first self-injection of open label semaglutide 0.25 mg if randomised to active treatment

7.5.2 Visit 5 (@2 weeks after first randomisation)

Clinical observations will be recorded. Blood glucose levels and ketone bodies will be measured via a fingerstick blood sample using glucose and ketones meter (see Section 7) and daily blood glucose levels collected and reviewed. Daily blood glucose data will be reviewed and insulin doses adjusted as per Visit 3. If participants randomised to semaglutide are experiencing any nausea, they will continue on the 0.25 mg dose for another week (or more as required) before increasing the dose. Otherwise semaglutide 0.5mg dose will be injected on site. Supply of IMP will be dispensed as appropriate to last until the next visit.

Visit Procedures

Monitoring and treatment optimisation
- Check for adverse events
- Review blood glucose diary
- Adjust insulin doses (if necessary)
- Blood ketones (from participant's own ketone meter)

Assessments
- Blood pressure
- Heart rate
- Weight

Urine sample:
- Urine ketones

Medication
- Dose titration (see above) if randomised to open-label semaglutide
- Return used study pen
- Dispense medication if randomised to open-label semaglutide

7.5.3 Visit 6 (@4 weeks after first randomisation)

For those randomised to open-label semaglutide, doses administered will be recorded (self-report). Clinical observations will be recorded. Daily blood glucose data will be reviewed and insulin doses adjusted as per visit 3. Supply of IMP will be given to last until the next visit. A urine sample will be collected to evaluate ketone bodies and, in females randomised to IMP, pregnancy status. Unless experiencing nausea, the recommended dose will be increased to 1.0 mg a day. A 24 hr urine container will be dispensed to collect a 24 hour urine sample to bring in at the Week 13 visit.

Visit Procedures

Monitoring and treatment optimisation

- Check for adverse events
- Check adequacy of contraception (WOCBP)
- Review blood glucose diary
- Adjust insulin doses (if necessary)
- Blood ketones (from participant's own ketone meter)

Assessments

- Blood pressure
- Heart rate
- Weight

Urine sample:

- Urine ketones
- Urine pregnancy test (WOCBP randomised to IMP only)
- 24 hour urine container dispensed

Medication

- Record doses of semaglutide self-injected since last visit (self-report)
- Dose titration if randomised to open-label semaglutide (see above)
- Return used study pens
- Dispense medication if randomised to open-label semaglutide

7.5.4 Visit 7 (@13 weeks after first randomisation)

Participants will be asked to attend in the fasting state (no food or calorie intake for 10 hours). For those randomised to open-label semaglutide, doses administered will be recorded (self-report). Daily fingerstick blood glucose levels (diaries), CGM/ FGM data or insulin pump download data will be reviewed and appropriate insulin dose adjustments made. Participants will discontinue use of the study CGM (Dexcom G6). Supply of IMP will be given to last until visit 10. Observations will be recorded. A urine sample will be collected to evaluate ketone bodies and, in females randomised to IMP, pregnancy status. The 24 hour urine container will be collected. A fasting blood sample will be taken (45 mL) to evaluate study endpoints.

Visit Procedures

Fasting visit*

Monitoring and treatment optimisation

- Check adequacy of contraception (WOCBP)
- Check for adverse events
- Review blood glucose diary
- Adjust insulin doses (if necessary)
- Blood ketones (from participant's own ketone meter)

Clinical assessments

- Blood pressure

- Heart rate
- Weight

Blood assessments

- Na, K, urea, creatinine, eGFR (MDRD)
- AST/ ALT/ Alkaline Phosphatase
- Calcium/ Phosphate
- CRP
- HbA1c
- Fructosamine
- Haemoglobin
- White cell count
- Platelets
- Bilirubin
- Albumin
- Random glucose

Urine

- Pregnancy test (WOCBP randomised to IMP only)
- Urine ketones
- 24 hour urine bottle collected

Medication

- Record doses of semaglutide self-injected since last visit (self-report)
- Return used study pens
- Dispense medication if randomised to open-label semaglutide

7.5.5 Visit 8 (@23 weeks after first randomisation)

For those randomised to open-label semaglutide, doses administered will be recorded (self-report). Clinical observations will be recorded. Blood glucose levels and ketone bodies will be measured via a fingerstick blood sample. A urine sample will be collected to evaluate ketone bodies and, in females randomised to IMP, pregnancy status. Insulin dose will be adjusted if needed. All subjects will be reminded to monitor their capillary blood glucose by fingerstick at least 4 times a day (before meals and at bedtime) throughout the study. CGM sensor will be fitted and participants asked to wear their CGM constantly for next 20 days.

Visit Procedures

Monitoring and treatment optimisation

- Check adequacy of contraception (WOCBP)
- Check for adverse events
- Review blood glucose diary
- Blood ketones (from participant's own ketone meter)
- Adjust insulin doses (if necessary)

Clinical assessments

- Blood pressure
- Heart rate

- Weight

Urine sample:
- Pregnancy test (WOCBP randomised to IMP only)
- Urine ketones

Other assessments
- *Start* Continuous Glucose Monitoring with Dexcom G6

7.5.6 Visit 9 (@25 weeks after first randomisation)

Participants will be asked to attend in the fasting state (no food or calorie intake for 10 hours). Daily fingerstick blood glucose levels (diaries), CGM/ FGM data or insulin pump download data will be reviewed and appropriate insulin dose adjustments made. Participants will discontinue use of the study CGM (Dexcom G6). Clinical observations will be recorded. Blood glucose levels and ketone bodies will be measured via a fingerstick blood sample (see Section 7) Supply of IMP will be given to last until the next visit. A 24 hr urine container will be dispensed to collect a 24 hour urine sample to bring in at visit 10 (41pprox.. Week 26) visit. A Meal Challenge Test will be performed as per visit 2. Participants will be provided with strips for urine and blood ketone measurement at home and instructed or reminded as appropriate on how to use these. They will be asked to measure urinary and blood ketones every morning for three consecutive days prior to the next visit (second randomisation: dapagliflozin or placebo) and to record the results in their diary.

Visit Procedures

Fasting visit*

Monitoring and treatment optimisation
- Check adequacy of contraception (WOCBP)
- Check for adverse events
- Review blood glucose diary
- Adjust insulin doses (if necessary) - **this insulin dose is baseline for Period 2**
- Blood ketones (from participant's own ketone meter)

Clinical assessments
- Blood pressure
- Heart rate
- Weight

Urine sample:
- Urine ketones
- 24 hour urine container dispensed

Other assessments
- Download Dexcom G6
- Meal Challenge Test

Blood assessments
- HbA1c

7.6 Second Randomisation Scheme

For the second randomisation (Period 2, double-blind), eligible and consenting participants previously randomized to open-label semaglutide will be randomised (2:1) to two groups (dapagliflozin or matching placebo) using permuted blocks of variable size to minimize predictability with stratification for HbA1c (above vs below 7.0 %). Those participants previously randomised to standard of care will continue as before.

7.6.1 Blinding (period 2 only)

For the double-blind treatment period, dapagliflozin and placebo will be packed in matching packs to maintain the blind. Each pack will contain sufficient supplies for 30 days' treatment with a small overage (excess). Packs will be labelled with a unique number that will be used to assign treatment to the participant via the IWRS system whilst maintaining the blind. Packs will be labelled in accordance with Good Manufacturing Practice and local regulatory requirements.

Unblinding. Treatment discontinuation (of dapagliflozin or placebo), rather than unblinding, will be carried out as far as possible. Participants will be advised in any case of hospitalisation with acute illness to discontinue study medication and inform the relevant clinician. These instructions will be reinforced in Study Diary These instructions will be reinforced in Study Diary However, where knowledge of treatment may assist emergency treatment, the investigator (or designee) may unblind using the IWRS system. Information on emergency unblinding (the investigational study drug, the study number, the investigator's name and a contact number) will be provided on the Patient Alert Card. For each unblinding an email alert is generated to the Study Coordinator and Chief Investigator. The most likely scenarios for unblinding will be: confirmed pregnancy, overdose/ accidental ingestion, or development of acute renal failure

7.6.2 Visit 10 (Second Randomisation Visit; @26 weeks after First Randomisation)

Participants will be asked to attend in the fasting state (no food or calorie intake for 10 hours). Participants with normal fasting urine (<+) or blood ketone (<0.6 mmol) in the three days prior to randomisation will proceed to the next treatment phase as per this visit. The principles of the STICH Protocol (see Appendix 3) will be discussed. Participants with higher ketone values in at least one of the previous three days will have their glucose levels, insulin dose, and carbohydrate intake reviewed and advised on hydration, adequate CHO intake, and adequate insulin dosing. They will be rescheduled for one week and asked to repeat three days testing before the next visit.

Clinical observations will be recorded. A urine sample will be collected for ketones. Blood glucose and ketones will be measured via a fingerstick blood sample (see Section 7.2.1). Daily fingerstick blood glucose levels (diaries), CGM/ FGM data or insulin pump download data will be reviewed and appropriate insulin dose adjustments made (following the guidance in Section 7, page 35). The previous three days blood and urine ketones levels will be reviewed. The adjusted total insulin dose at this visit will serve as the baseline total insulin dose for the remaining six months of the study and future limits of dose adjustments will be based on this new baseline. The blinded study CGM will be collected. The 24 hour urine container will be collected. A fasting blood sample will be taken (45 mL) to evaluate study endpoints. Participants will be asked to complete quality of life questionnaires.

At this point, those participants previously randomised to receive open-label semaglutide will be randomised to receive dapagliflozin 5 mg daily or matching placebo. They will be educated on symptoms suggestive of low blood pressure (e.g. light-headedness, dizziness, feeling faint).

Participants will be asked to perform a daily (preferably early morning) urine (using CombiScreen® 10 SYS Plus urine testing strips or equivalent) and blood tests for ketone bodies (using the particpant's own meters as per Section 7, "Minimising ketosis": page 28) and record all outcomes in dedicated study diaries. If results of these scheduled (i.e. not done due to symptoms) ketone measurements, are +1 or higher (urine) or > 0.6 mmol/L (blood), participants will be instructed to repeat the measurement after 60 min. Participants will be instructed to call site staff directly as soon as possible within office hours if these results are persistent, . For any abnormal ketone levels taken due to symptoms, the DKA consensus guidelines are to be followed instead (see Section 9.3).

Participants will be instructed to call site staff directly in case of any problem or untoward side effects. They will be specifically asked to call if they have confirmed hypoglycaemia (blood glucose levels < 3.9 mmol/L) or hyperglycaemia (blood glucose > 13.9 mmol/L) occurring on more than one occasion. Participants will be advised to remain well hydrated and report any genital mycotic or urinary tract infections

Visit Procedures

Fasting visit* - Requirements and arrangements as per visits 2 and 3.

Monitoring and treatment optimisation

- Check adequacy of contraception (WOCBP)
- Check for adverse events
- Review blood glucose diary
- Adjust insulin doses (if necessary)
- Blood ketones (from participant's own ketone meter)

Clinical assessments
- Blood pressure
- Heart rate
- Body weight

Quality of life assessments
- Diabetes Specific Quality of Life Scale (DSQOLS)
- Problem Areas In Diabetes (PAID) questionnaire

Blood assessments
- Na, K, urea, creatinine, eGFR (MDRD)
- AST/ ALT/ Alkaline Phosphatase
- Calcium/ Phosphate
- CRP
- Fructosamine
- Haemoglobin
- White cell count
- Platelets
- Bilirubin

- Albumin
- Random glucose

Urine

- Pregnancy test (WOCBP randomised to IMP only)
- Urine ketones
- 24 hour urine bottle collected

Medication

- Education on symptoms suggestive of low blood pressure (e.g. light-headedness, dizziness, feeling faint) for those undergoing second randomisation
- Record doses of semaglutide self-injected since Visit 7 (self-report)
- Return used study pens
- Dispense open label semaglutide for participants randomised to active treatment at Period 1 (if required).
- Dispense dapagliflozin or placebo if randomised to active treatment

Other assessments

- *Read* Continuous Glucose Monitoring sensor (mean fasting blood glucose, mean blood glucose, percentage time in range, glycaemic variability)
-

7.6.3 Visit 11 (@1 week after Second Randomisation)

For those randomised to open-label semaglutide, doses administered will be recorded (self-report). Clinical observations will be recorded. Daily blood glucose levels and urinary ketone measurements will be reviewed. A urine sample will be collected to evaluate for ketones bodies. Blood glucose levels and ketones bodies will be reviewed measured via a fingerstick blood sample (see Section 7.2.1). . Daily fingerstick blood glucose levels (diaries), CGM/ FGM data or insulin pump download data will be reviewed and appropriate insulin dose adjustments made using the new baseline from Visit 10 (following the guidance in Section 7, page 35). If at any time total insulin dose reduction is >20% and total insulin dose is <0.40 U/Kg, additional monitoring (see Appendix 2) will be implemented. Unless blood (>0.6 mmol/L) or urine ketones (+ or more) have been elevated within the previous three days, dapagliflozin or placebo dose will be uptitrated to 10 mg daily (two capsules of either active or placebo medication) for the remainder of the study. Supply of IMPs will be given to last until week 39.

Visit Procedures

Monitoring and treatment optimisation

- Check adequacy of contraception.
- Check for adverse events
- Review blood glucose diary
- Adjust insulin doses (if necessary)
- Blood ketones (from participant's own ketone meter)
- Daily urine ketones review

Urine sample:

- Urine ketones

Medication

- Count returned dapagliflozin or placebo capsules

- Increase dose of dapagliflozin or placebo to 10mg (see above – review ketones)
- Dispense dapagliflozin or placebo

7.6.4 Visit 12 (@5 weeks after Second Randomisation)

For those randomised to open-label semaglutide, doses administered will be recorded (self-report). Clinical observations will be recorded. Daily blood glucose levels and urinary ketones measurements will be collected and reviewed. Blood glucose levels and ketone bodies will be measured via a fingerstick blood sample. A urine sample will be collected to evaluate ketone bodies and if appropriate pregnancy status. Insulin dose will be adjusted if as per Visit 11. A blood sample will be taken (15 mL) to evaluate study endpoints. A 24 hr urine container will be dispensed to collect a 24 hour urine sample to bring in at the Week 39 visit

Visit Procedures

Monitoring and treatment optimisation

- Check adequacy of contraception (WOCBP)
- Check for adverse events
- Review blood glucose diary
- Adjust insulin doses (if necessary)
- Blood ketones (from participant's own ketone meter)

Clinical assessments

- Blood pressure
- Heart rate
- Weight

Blood assessments

- Na, K, urea, creatinine, eGFR (MDRD)
- AST/ ALT/ Alkaline Phosphatase
- Calcium/ Phosphate
- CRP
- Haemoglobin
- White cell count
- Platelets
- Bilirubin
- Albumin
- Random glucose

Urine

- Pregnancy test (WOCBP randomised to IMP only)
- Urine ketones
- 24 hour urine container dispensed

7.6.5 Visit 13 (@13 weeks after Second Randomisation)

For those randomised to open-label semaglutide, doses administered will be recorded (self-

report). Participants will be asked to attend in the fasting state. Clinical observations will be recorded. Daily blood glucose levels and urinary ketones measurements will be collected and reviewed. Blood glucose levels and ketone bodies will be measured via a fingerstick blood sample. A urine sample will be collected to evaluate ketone bodies and if appropriate pregnancy status. The 24 hour urine container will be collected. Insulin dose will be adjusted if needed as in as per Visit 11.. Sufficient supply of IMPs will be given until the end of the study. A fasting blood sample will be taken (45 mL) to evaluate study endpoints.

Visit Procedures

Fasting visit* - as per previous fasting visits

Monitoring and treatment optimisation

- Check adequacy of contraception (WOCBP)
- Check for adverse events
- Review blood glucose diary
- Adjust insulin doses (if necessary)
- Blood ketones (from participant's own ketone meter)
- Daily urine ketones review

Clinical assessments

- Blood pressure
- Heart rate
- Weight

Quality of life assessments

- Diabetes Specific Quality of Life Scale (DSQOLS)
- Problem Areas In Diabetes (PAID) questionnaire

Blood assessments

- Na, K, urea, creatinine, eGFR (MDRD)
- AST/ ALT/ Alkaline Phosphatase
- Calcium/ Phosphate
- CRP
- HbA1c
- Fructosamine
- Haemoglobin
- White cell count
- Platelets
- Bilirubin
- Albumin
- Random glucose

Urine

- Pregnancy test (WOCBP randomised to IMP only)
- Urine ketones
- 24 hour urine bottle collected

Medication

- Record doses of semaglutide administered since Visit 10 (self-report)
- Dispense open label semaglutide for participants randomised to active treatment at Period 1 (if required).
- Count returned dapagliflozin or placebo capsules
- Dispense dapagliflozin or placebo as required

7.6.6 Visit 14 (@23 weeks after Second Randomisation)

For those randomised to open-label semaglutide, doses administered will be recorded (self-report). Clinical observations will be recorded. Daily blood glucose levels and urinary ketones measurements will be collected and reviewed. Blood glucose levels and ketone bodies will be measured via a fingerstick blood sample. A urine sample will be collected to evaluate ketone bodies and if appropriate pregnancy status. Insulin dose will be adjusted if as per Visit 11. All subjects will be reminded to monitor their capillary blood glucose by fingerstick at least four times a day (before meals and at bedtime). CGM (Dexcom G6) sensor will be fitted and patients asked to wear their CGM constantly for the next 20 days.

Visit Procedures

Monitoring and treatment optimisation

- Check adequacy of contraception (WOCBP)
- Check for adverse events
- Review blood glucose diary
- Adjust insulin doses (if necessary)
- Blood ketones (from participant's own ketone meter)
- Daily urine ketones review

Clinical assessments

- Blood pressure
- Heart rate
- Weight

Urine sample:

- Pregnancy test (WOCBP randomised to IMP only)
- Urine ketones

Clinical and functional assessment

- *Start* Continuous Glucose Monitoring sensor (mean fasting blood glucose, mean blood glucose, percentage time in range, glycaemic variability)

7.6.7 Visit 15 (@25 weeks after Second Randomisation)

Participants will be asked to attend in the fasting state. For those randomised to open-label semaglutide, doses administered will be recorded (self-report). Observations will be taken. Daily blood and urinary ketones measurements will be collected and reviewed. Blood glucose levels and ketone bodies will be measured via a fingerstick blood sample. A urine sample will be collected to evaluate urine ketone levels. Insulin dose will be adjusted if needed as per visit 11. A 24 hr urine container will be dispensed to collect a 24 hour urine sample to bring in at the Week 52 visit. A HFHC Meal Challenge Test will be performed as in Visit 2.

Visit Procedures

Fasting visit* - Procedure as per previous fasting visits

Monitoring and treatment optimisation
- Check adequacy of contraception (WOCBP)
- Check for adverse events
- Review blood glucose diary
- Adjust insulin doses (if necessary)
- Daily urine ketones review
- Blood ketones (from participant's own ketone meter)

Assessments
- Blood pressure
- Heart rate
- Weight

Urine sample:
- Pregnancy test (WOCBP randomised to IMP only)
- 24 hour urine container dispensed

Other assessments
- Meal Challenge Test

7.6.8 Visit 16 (End of study; @26 weeks after Second Randomisation)

Participants will be asked to attend in the fasting state. Clinical observations will be recorded. Daily blood glucose levels and urinary ketones measurements will be collected and reviewed. Blood glucose levels and ketone bodies will be measured via a fingerstick blood sample. Insulin doses will be reviewed and likely requirement for an increase following discontinuation of IMP discussed with those taking open-label semaglutide. A urine sample will be collected to evaluate ketone bodies and, if appropriate pregnancy status. The CGM will be collected. The 24 hour urine container will be collected. A fasting blood sample will be taken (45 mL) to evaluate study endpoints. We will ask the participants to complete quality of life questionnaires. A physical examination will be performed as per the screening visit. This will be the final face-to-face study visit. A safety phone call will be conducted two weeks to review blood glucose data, provide further insulin dosing advice, to document any adverse events, and to ensure that follow-up in usual clinical care has been arranged.

Visit Procedures

Fasting visit*

Monitoring and treatment optimisation
- Check for adverse events
- Review blood glucose diary

- Adjust insulin doses (if necessary)
- Blood ketones (from participant's own ketone meter)
- Daily urine ketones review

Clinical assessments
- Blood pressure
- Weight
- Heart rate
- Physical examination

Quality of life assessments
- Diabetes Specific Quality of Life Scale (DSQOLS)
- Problem Areas In Diabetes (PAID) questionnaire

Blood assessments
- Na, K, urea, creatinine, eGFR (MDRD)
- AST/ ALT/ Alkaline Phosphatase
- Calcium/ Phosphate
- CRP
- HbA1c
- Fructosamine
- Haemoglobin
- White cell count
- Platelets
- Bilirubin
- Albumin
- Random glucose

Urine
- Pregnancy test (WOCBP randomised to IMP only)
- Urine ketones
- 24 hour collection

Other

- Record doses of semaglutide administered since Visit 13 (self-report)
- Return used study pens
- Count returned dapagliflozin or placebo capsules
- Collect Patient Alert Card

7.7 Retention and strategies for maximizing follow-up

Participants will be encouraged to attend all study visits. Prior to commencing the trial, all potential participants will have the opportunity to read the detailed Patient Information Sheet which will outline the

level of participation requested. Potential participants will be made aware of the need for frequent study visits and asked to commit to these.

Study visits can be rearranged flexibly within a period around the planned visit as specified in the timeline of visits above (Section 7) and actual dates recorded in the eCRF. This is to maximise participant ability to attend all visits. A longer or shorter interval does not constitute a protocol deviation. Where possible, visits will be arranged to coincide with other appointments to further maximise attendance and participant convenience. Telephone contact will be frequent and will help to maintain contact with participants and ensure timely reminders of upcoming study visits can be given.

Even in the scenario of treatment discontinuation (of either IMP), participants will be encouraged to continue to attend further study visits as scheduled.

7.8 Treatment Interruptions and Withdrawal criteria

Withdrawal from study drug

Treatment interruption from study IMPs may be necessary in some participants. In the case of dapagliflozin, this will occur in any acute illness or hospitalisation which increases the risk of ketosis and ketoacidosis. Circumstances in which temporary discontinuation of dapagliflozin or placebo should occur will be discussed with all study participants. Written information will be provided in the study diaries. An emergency phone line to the study team will be available to all participants for the duration of their participation in the trial. This will be operational during office hours (Monday to Friday 9am – 5pm). Patient Alert Card Version 1 will also contain unblinding information in the event of acute intercurrent illness and a requirement for unblinding in the opinion of the acute medical team treating the participant. If any participant develops diabetic ketoacidosis (DKA) as defined as per the International Consensus guidelines on two occasions with no identifiable precipitant (section 2.1) they will be withdrawn from study medication and the UK Chief and US Principal Investigators informed.

Any other intolerable side effects or acute illness may require temporary discontinuation of the study drugs (dapagliflozin or semaglutide). In these circumstances, if the study drugs cannot be safely restarted in the judgement of the trial physician or if participants are unable to tolerate side-effects, they may be withdrawn from study treatment.

Treatment withdrawal may be necessary during the trial prior to the planned end at 52 weeks following first randomisation for any of the following reasons:

1. The site PI can stop investigational study treatment or remove participants from the trial without participants approval if for any reason he/she feels is appropriate, including: severe side effects, injury or medical condition which may place the patient at risk of further complications if he/she continue to participate, failure to take the medication as instructed, failure to keep scheduled appointments, insufficient level of self-monitoring, cancellation of the study or other administrative reasons.

2. Any woman who becomes pregnant during the period of study will be withdrawn from study treatment.

3. Participants who experience persistent severe nausea or vomiting after starting semaglutide which is not managed by dose reduction will discontinue the investigational study treatment. Those with mild or moderate nausea will continue for at least one more week. For those who continue to experience significant nausea (defined as interfering with normal activities), dose will be reduced: if these side

effects persist, they will discontinue study medication(s). Participants for whom this occurs in Period 1 will remain eligible for randomisation in Period 2.

4. Excessive weight loss during either period of the study despite treatment dose reduction (> 15% body weight or BMI < 22 kg/m2 on repeated measurements more than four weeks apart).

5. New diagnosis of other conditions included in the Exclusion Criteria

6. Change in method of insulin delivery between insulin pumps to MDI or vice versa during the study unless pump treatment interruption is temporary for a period of at most 2 weeks. Participants who upgrade to "artificial pancreas" (e.g. Medtronic's MiniMed 670G hybrid) during the study will be withdrawn.

7. Hypoglycaemia: Two episodes of severe hypoglycaemia (requiring external intervention).

7. Ketosis (with no other identifiable precipitating factors): Persistent (1 week) asymptomatic or symptomatic and unmanaged (using STICH protocol – see section 2.1 b) blood ketone levels >1.5 mmol (or ≥+2 in urine).

9. Persistent (>1 week) total insulin dose reduction of >20% from baseline AND total insulin dose <0.4U/Kg.

10. DKA: Two episode of DKA (with no identifiable precipitating factors) as defined by the expert consensus statement (34)

11. Two episodes of UTI.

12. Two episodes of mycotic genital infection.

13. Significant persistent reduction in eGFR > 15 ml/min

14. Pancreatitis: any episode judged to be due to pancreatitis by the site PI.

15. Other persistent side effects specific to study drugs

Participation in this study is voluntary. Participants have the right to refuse to participate or to withdraw from participation at any time for any reason. The site PI can stop investigational study treatment or remove participants from the trial without participants approval. If a patient withdraws consent and decide not to participate in the study, she/he will be asked to undergo a final study visit for his/her safety. If a subject withdraws from the research, the data collected to that point will be used toward the research finding. If applicable the subject will be asked to return any unused research drug and or devices.

Withdrawal from the study

If participants are unable or unwilling to attend all study visits they will be given an option of attending less frequently or only at the end of the study. We will request participants' consent to be contacted by telephone and for contact with their general practitioner or other health care provider to check on their current health status. No participant will be labelled as lost to follow-up. However, participants will have

the right to withdraw consent for further participation and for further data collection: all other participants will be followed up for clinical events until study completion.

7.9 Storage and analysis of samples

Blood samples will be undertaken at the beginning and at various points during the study as outlined in detail in the schedule of visits. If blood testing has recently been undertaken in the 3 weeks prior to the study commencing, some of these blood tests may be used as baseline. Blood tests will include analysis of the full blood count, renal function (urea and electrolytes), liver function tests, bone profile, lipid profile and several specialised blood tests for biomarker assays (at central laboratory) following a high fat high caloric meal challenges to examine study endpoints.

Covid-19 Mitigation

All blood sampling or trial procedures will be undertaken following local and national guidance around personal protective equipment. Mask, gloves and aprons will be used as per current guidelines.

7.9.1 Sample collection and processing

- Samples will be appropriately labelled in accordance with the trial procedures to comply with the General Data Protection Regulation (GDPR). Biological samples collected from participants as part of this trial will be transported, stored, accessed and processed in accordance with national legislation relating to the use and storage of human tissue for research purposes and such activities shall at least meet the requirements as set out in the 2004 Human Tissue Act and the 2006 Human Tissue (Scotland) Act.
- Blood will be taken according to the Schedule of Assessments (page 17). Plasma will be siphoned, aliquoted and stored locally at -80° ± 10°C until transport in batches to the central laboratory on dry ice. according to the Sample Handling Procedure as agreed by the Joint Study Management Group
- Individual patient samples will be identified with a unique, anonymised study number.

7.9.2 Sample transport to central laboratory and analysis

- Samples will be transferred to State University of New York in a single batch at the end of recruitment to the study. More frequent transfer can be organised if there are local storage limitations.
- Samples will be transported on dry ice and stored at the central laboratory at -80° ± 10° until analysis. Transport by courier will coordinated by the Trial Manager.
- At the time of analysis individual aliquots will be defrosted at room temperature and analysed in a single batch for each biomarker of interest.
- Samples will be stored at -80° ± 10° in the central laboratory for possible future analysis for novel biomarkers.

7.10 End of trial

The end of the trial will be following the last study visit of the last subject.
At the end of the specified trial period (52 weeks following first randomisation) all participants will discontinue study medications and revert to standard of care (insulin therapy only). This process will be supervised by the trial physicians, research nurses, and research pharmacists under the supervision of the site Principal Investigator. The trial team will liaise with the usual care team to ensure a safe transition back to standard therapy. Following the final scheduled trial visit there will be a safety phone call two weeks after the last visit. This will act as a further opportunity to advise on insulin dosing and ensure there are no ongoing problems or concerns related to the study.

As discussed in section 7.8, there may be circumstances in which trial drugs need to be stopped before the specified end of the trial. If this is the case, the reason(s) for discontinuation will be recorded and the trial clinical team will facilitate safe transition back to usual standard of care. Participants will however if eligible (i.e non-exclusion criteria reason for IMP discontinuation) be asked to continue with trial visits to enable ongoing close clinical observation. If consent is withdrawn and participants voluntarily chose to leave the trial before the specified end date, all efforts by the clinical trial team will be made to safely facilitate transition back to ordinary standard of care. A follow up safety visit will be offered to all such participants and ongoing contact details will be made available should there be any further unforeseen issues relating to the clinical trial.

The trial may be stopped at an earlier stage than anticipated on the advice of the independent data monitoring committee (IDMC) to the trial steering committee (TSC) for the safety reasons detailed on page 12 and in the study safety endpoints. If this occurs, all participants will be informed and safe discontinuation of study medications will be undertaken under the supervision of the site Principal Investigator.

8 TRIAL MEDICATION

8.1 Name and description of investigational medicinal product(s)

a) Semaglutide (Ozempic®): a once weekly GLP-1 agonist injection indicated for type 2 diabetes (approved by the Scottish Medicines Consortium, January 2019).

   Doses to be used: 0.25mg increased to 0.5mg at two weeks and then to 1mg weekly s.c. injection after a further two weeks. Open label semaglutide will be added to a background of insulin treatment at week 0 for 12 months in 76 participants (38 in the UK) according to the first randomisation; at week 26 they will be further randomised (double-blind) as below into two groups: dual (placebo, semaglutide and insulin) and triple (dapagliflozin, semaglutide and insulin) therapy.

b) Dapagliflozin (as dapagliflozin propanediol monohydrate Forxiga®): a once daily SGLT-2 inhibitor that will be administered orally in a blinded fashion.

   Doses to be used: 5 mg daily increased after one week to 10 mg daily (orally). Dapagliflozin will be started according to the randomisation (Interactive Web System, hosted by University of Glasgow) in 38 participants (19 in the UK) at 26 weeks as an

additional treatment on a background of insulin and semaglutide therapy and end at 52 weeks (triple therapy group). Doses to be used: 5 mg increased to 10 mg after one week.

c) Placebo to dapagliflozin: Dapagliflozin matched placebo will be started in 38 participants (19 in the UK) participants at 26 weeks as an additional treatment on a background of insulin and semaglutide therapy and end at 52 weeks (dual therapy group). Doses to be used: as for active dapagliflozin.

8.2 Legal status of Semaglutide and Dapagliflozin

Semaglutide is currently licensed in the UK for those with adult type 2 diabetes which is insufficiently controlled.

Dapagliflozin is currently licensed in the UK for insufficiently controlled type 1 and type 2 diabetes.

8.3 Drug storage and supply

Semaglutide and dapagliflozin/placebo supplies must be stored in the original containers in a locked, secure area with access limited to authorised site staff. Study supplies should be used as directed in the study protocol and not be supplied to any persons other than study participants. All IMPs will be dispensed to participants from the study site pharmacy.

Covid-19 Mitigation

As detailed above in section 7, the supply of IMP may be altered to reduce risk of Covid-19 transmission in the event of future waves of infection. This may involve providing increased quantities of IMP to participants to last longer and therefore minimise physical contact with trial personnel. Use of a courier delivery service to facilitate this may also be considered.

Semaglutide: Supplies of semaglutide pre-filled pens for subcutaneous administration will be sourced from routine hospital stocks. The following semaglutide presentations will be required:

- Semaglutide 0.25 mg: Each pre-filled pen contains 2mg semaglutide in 1.5ml. Each dose contains 0.25mg of semaglutide in 0.19ml solution.
- Semaglutide 0.5 mg: Each pre-filled pen contains 2mg semaglutide in 1.5ml solution. Each dose contains 0.5 mg of semaglutide in 0.37ml solution.
- Semaglutide 1 mg: Each pre-filled pen contains 4 mg semaglutide in 3.0ml solution. Each dose contains 1mg semaglutide in 0.74ml solution.

For all presentations each pre-filled pen is capable of delivering 4 doses which is sufficient for 4 weeks treatment. Pens are for individual participant use only. In the event of a pen-failure, a new pen should be supplied to the participant.

Prior to first use, semaglutide pens must be stored at 2-8 °C in the original container and must not be frozen. Supplies which are frozen either prior to first use or during use must be discard.

Semaglutide pre-filled pens have an in-use shelf life of 6 weeks. After first use the pre-filled pens may be stored below 30oC or in a refrigerator at 2-8 °C. Participants should be advised to store packs away

from the cooling element. The pen cap should be on when the pen is not in use in order to protect the contents from light.

Wherever possible, supplies of semaglutide pre-filled pens should be utilised in a pragmatic way so as to minimise unnecessary wastage. Participants should be provided with an appropriate number of pen(s) to last until the next planned visit. A small overage may be given as necessary to accommodate visit windows etc. The correct strength of pen must be supplied for each dose titration i.e. 0.25mg, 0.5mg and 1.0 mg as tolerated.

A web-based system will be used to randomise participants to semaglutide treatment. At randomisation and all subsequent visits (planned or unplanned), the system will notify pharmacy staff at study visits of the requirement for semaglutide supplies.

Dapagliflozin 5mg or placebo capsules: Supplies of dapagliflozin 5mg and matched placebo capsules will be manufactured by NHS Scotland Pharmaceuticals Specials Service/NHS Tayside Pharmaceuticals in accordance with Good Manufacturing Practice requirements. Capsules will be packed into HDPE bottles with a tamper evident seal. Each bottle will be labelled in a double-blind fashion to meet local regulatory requirements and will be assigned a unique number. Bottles containing dapagliflozin 5 mg or placebo must be stored at room temperature at 15-25°C.

Dapagliflozin 5mg and placebo capsule supplies will be provided to study site and will only be released by the sponsor once all the appropriate regulatory and governance approvals are in place. Further information on storage requirements and supply arrangements is provided in the study specific IMP Management and Accountability Manual.

A web-based system will be used for the assignment of dapagliflozin 5mg or placebo bottles at randomisation and at study visits where there is planned dispensing of the IMP or in the event that a participant requires replacement medicines.

Other medicines: Supplies of all other medicines and associated consumables will via usual NHS supply mechanisms.

8.4 Drug accountability requirements

The Investigator or designee must maintain accurate records of all study IMP movements for accountability purposes. They should include dates, quantities, batch numbers and expiry. Records must document adequately that:

- the patients were provided the doses specified by the protocol/amendment(s)
- all study drug provided was fully reconciled.

Unused study drug must not be discarded or used for any purpose other than the present study. Further information is provided in the study specific IMP Management and Accountability Manual.

8.5 Preparation and administration of semaglutide and dapagliflozin

Semaglutide (Ozempic®) is a solution for subcutaneous injection. It is administered once weekly, on the same day each week, into the subcutaneous tissues of the abdomen, thigh or upper arm. A participant may change the day of their weekly injection as long as it has been at least three days since their last injection. Thereafter, all doses should be administered once weekly on the new dosing day. The injection can be administered at any time of the day regardless of meals.

Participants must be advised to discard the injection needle after each injection and store the pen without an injection needle attached. This may help prevent blocked needles, contamination, infection, leakage of

solution and inaccurate dosing. Semaglutide should not be used if the solution does not appear clear and colourless or almost colourless. The pen is designed to be used with NovoFine or NovoTwist disposable needles and pens are packed with an appropriate quantity of NovoFine Plus needles.

Detailed information on administration of semaglutide is provided in the current Summary of Product Characteristics and product Patient Information Leaflet.

Dapagliflozin (Forxiga®)) is presented as a capsule for once daily oral administration.

8.6 Dosage schedules

Semaglutide will be administered and dosed as described. Participants will be commenced on 0.25 mg s.c once weekly before up-titration as tolerated to 1.0 mg s.c once weekly. Up-titration is quicker than is ordinarily the case in clinical practice in type 2 diabetes as discussed in Section 2.1 (22) There will be increased observation and clinical access for participants in the event of side-effects. Participants randomised to receive semaglutide will continue on the 1.0 mg s.c once weekly dose for the remainder of the study following uptitration provided they tolerate the dose as detailed in Section 7. If necessary, the dose may be adjusted.
If a participant misses a dose of semaglutide it should be administered as soon as possible and within 5 days after the missed dose. If more than 5 days have passed, the missed dose should be skipped, and the next dose should be administered on the regularly scheduled day. In both cases participants, should then resume their regular once weekly dosing schedule.

Dapagliflozin will be commenced at 5mg orally once daily and uptitrated one week after commencement to a dose of 10mg which will thereafter be continued for the remainder of the trial provided they tolerate the dose. If necessary, the dose may be adjusted.

8.7 Dosage modifications

Dapagliflozin/placebo will be commenced at one capsule (5mg or placebo) orally once daily and uptitrated one week after commencement to a dose of two capsules (10mg or placebo) which will thereafter be continued for the remainder of the trial provided they tolerate the dose. If necessary, the dose may be adjusted. (See also Section 7.8 regarding treatment discontinuation) Dapagliflozin or placebo capsules can be taken at any time of day with or without food. Capsules should be swallowed whole. If participants forget to take a dose they should be advised as follows:

- If it is 12 hours or more until the next dose, the dose should be taken as soon as remembered and the next dose taken at the usual time.
- If it is less than 12 hours until the next dose, the missed dose should be skipped. The next dose should be taken at the usual time.
- Participants should not take a double dose of dapagliflozin/placebo to make up for a forgotten dose.

8.8 Known drug reactions and interaction with other therapies

Participants with hypersensitivity to either active study medication are excluded from the trial. There are no significant interactions between semaglutide and other prescribed medications other than with agents that can lower blood glucose levels. Participants on any other oral agents which can lower blood glucose levels meet Exclusion Criteria and will not be included in this trial. In combination with insulin and dapagliflozin, semaglutide may be associated with an increased risk of hypoglycaemia. Participants will be advised of the increased risk of hypoglycaemia and will be required to closely monitor their blood glucose levels for the duration of the trial. No interactions are currently listed between warfarin and semaglutide in the British National Formulary but in view of a theoretical interaction (based on protein binding), existing arrangements for INR testing will be reviewed for any

participants prescribed warfarin and additional tests conducted if indicated according to the judgement of the investigators.

Dapagliflozin is known to increase the risk of hypoglycaemia when combined with other medications which also act to lower blood glucose levels. Dapagliflozin has a mild diuretic effect and therefore there is an increased risk of hypotension when dapagliflozin is combined with either medications which deplete circulating volume (e.g diuretics) or antihypertensive drugs. Dapagliflozin is however commonly prescribed with such medications in general medical practice. All participants will have their medications reviewed at the screening visit and educated regarding symptoms that may indicate dehydration/ hypovolaemia. If there is any concern of interaction with any of the IMPs, this will be discussed with the PI before a decision regarding their ability to participate is made.

8.9 Concomitant medication

Participants should not receive any adjunct glucose-lowering therapy other than as per the randomisation schedule (e.g. with either of the active study medications).

8.10 Trial restrictions

Contraindications will follow SmPC information for Ozempic® and Forxiga. Acceptable forms of contraception for WOCBP participating in the trial are listed in Appendix 2. There is no requirement for contraception for male participants.

8.11 Assessment of compliance

Treatment adherence will be assessed by the trial medical and nursing team when participants attend for scheduled study visits.

8.12 Name and description of each Non-Investigational Medicinal Product (NIMP)

Insulin therapy is a non-investigational medicinal product in this trial. The trial is in a population who have had a diagnosis of type 1 diabetes for at least one year: therefore all participants will already be established on insulin therapy. The regimen and dosage required will by differ between participants. Participants will be using a combination of long acting (basal) insulin and shorter acting (bolus) insulin or CSII (insulin pump). As in standard of care, dose titration will depend on diet, blood glucose levels and exercise. It is anticipated that dosage adjustments will be required to achieve optimal glycaemic control and maintain participant safety.

There are pre-defined guidelines for insulin titration in this study. Insulin dose reductions should not be greater than 10% (5% if HbA1c is<7%) from current total insulin dose or more than 20% from the baseline level at randomisation and then from the new baseline levels at 26 weeks following second randomisation. Participants requiring an insulin dose reduction > 20% to a total insulin <0.4 U/kg will be monitored closely for insulin dose, carbohydrate intake, ketones and blood glucose. Study medication can be temporarily discontinued, if necessary, for one-to-two weeks. If low insulin requirements remain, the participant will be withdrawn from study (see Section 7.8).

9 PHARMACOVIGILANCE

9.1 Definitions

Term	Definition
**Adverse Event (AE)**	Any untoward medical occurrence in a participant to whom a

	medicinal product has been administered, including occurrences which are not necessarily caused by or related to that product.
**Adverse Reaction (AR)**	An untoward and unintended response in a participant to an investigational medicinal product which is related to any dose administered to that participant.<br>The phrase "response to an investigational medicinal product" means that a causal relationship between a trial medication and an AE is at least a reasonable possibility, i.e. the relationship cannot be ruled out.<br>All cases judged by either the reporting medically qualified professional or the Sponsor as having a reasonable suspected causal relationship to the trial medication qualify as adverse reactions.
**Serious Adverse Event (SAE)**	A serious adverse event is any untoward medical occurrence that:<br>1. results in death<br>2. is life-threatening<br>3. requires inpatient hospitalisation or prolongation of existing hospitalisation<br>4. results in persistent or significant disability/incapacity<br>5. consists of a congenital anomaly or birth defect<br><br>Other ‘important medical events’ may also be considered serious if they jeopardise the participant or require an intervention to prevent one of the above consequences.<br>NOTE: The term "life-threatening" in the definition of "serious" refers to an event in which the participant was at risk of death at the time of the event; it does not refer to an event which hypothetically might have caused death if it were more severe*.
**Serious Adverse Reaction (SAR)**	An adverse event that is both serious and, in the opinion of the reporting Investigator, believed with reasonable probability to be due to one of the trial treatments, based on the information provided.
**Suspected Unexpected Serious Adverse Reaction (SUSAR)**	A serious adverse reaction, the nature and severity of which is not consistent with the information about the medicinal product in question set out:<br>• in the case of a product with a marketing authorisation, in the summary of product characteristics (SmPC) for that product<br>• in the case of any other investigational medicinal product, in the investigator’s brochure (IB) relating to the trial in question
**Reference Safety Information**	The information used for assessing whether an adverse reaction is expected. This is contained in either the investigator’s brochure or the summary of product characteristics

*Note: “Severe” is often used to describe intensity of a specific event, which may be of relatively minor medical significance. “Seriousness” is the regulatory definition supplied above.

9.2 Operational definitions for (S)AEs

Adverse events (AEs) will be recorded, notified, assessed, reported, analysed and managed in accordance with the Medicines for Human Use (Clinical Trials) Regulations 2004 (as amended) and this protocol.

All Serious Adverse Events (SAEs) occurring during the trial will be recorded within the eCRF. Non-serious AEs occurring during the trial will be recorded within the eCRF if they are considered to be related to trial medication, i.e. adverse reactions (ARs).

Exclusions from SAE reporting

Hospitalisation for the following reasons will not be considered to be SAEs:

- Treatment which was elective or pre-planned, for a pre-existing condition not associated with any deterioration in condition, e.g. pre-planned hip replacement operation which does not lead to further complications.
- Any admission to hospital or other institution for general care where there was no deterioration in condition.
- Treatment on an emergency, outpatient basis for an event **not** fulfilling any of the definitions of serious as given above and not resulting in hospital admission.
- COVID-19 infection unless results in severe illness with hospital admission.

9.3 Recording and reporting of AEs, Adverse Events of Special Interest, SAEs and SUSARs

All AEs occurring during the trial that are observed by the Investigator or reported by the participant will be recorded in the participant's medical records whether or not attributed to trial IMP. AEs will be recorded from randomisation until the later of 30 days post cessation of trial treatment or the end of the study.

Adverse Events of Special Interest, SAEs and SUSARs will be recorded in the participant's medical records and on the eCRF and will be recorded from the point of randomisation until the later of 30 days post cessation of trial treatment or the end of the study.

Additional events identified only through record linkage will generate SAE records in the eCRF and should be recorded in the participant's medical records in the same way as AEs.

9.3.1 Adverse Events of Special Interest

Adverse Events of Special Interest as defined in detail below should be recorded within the relevant section of the eCRF but do not require reporting in an expedited manner (unless the event also meets the regulatory definition of an SAE). The following information will be recorded for all events:

- Nature of the event
- Event duration (start and end dates, if applicable)
- Relationship to study drug (if applicable)
- Outcome (if applicable)

Adverse Events of Special Interest will be monitored and followed up (if applicable) until the event has resolved or a final outcome has been reached.

All Adverse Events of Special Interest must be assessed for seriousness prior to recording within the eCRF. Those meeting the definition of an SAE should also be reported accordingly as per Section 9.3.2.

(i) Ketosis and diabetic ketoacidosis

This trial will be conducted in participants who have type 1 diabetes. All participants will be taking insulin. In this condition, due to the near absence of endogenous insulin, at a cellular level there is an inability to incorporate glucose into normal intra-cellular energy yielding pathways. In this context, alternative energy yielding pathways are sought including ketone body production from fatty acids in the liver. Ketones are clinically important as they easily dissociate hydrogen ions upsetting physiologically regulated acid base balance. As ketone levels rise, there is initially a state of high blood ketone levels without significant acidosis (ketosis); if this develops further, diabetic ketoacidosis (DKA) may develop. DKA requires urgent medical input. Ketones can be easily measured via a simple urine test or fingerstick capillary blood measurement. Participants eligible for this trial will already be trained in how to do this. Although dapagliflozin, one of the IMPs in this trial, has a license for use in type 1 diabetes, it is known to increase the risk of ketosis and ketoacidosis and this can occur at lower blood glucose levels than in those who are on insulin treatment only [euglycaemic DKA] (13,14). Given this known risk, ketosis and DKA are included in the safety endpoints and as such will be recorded in the eCRF as Adverse Events of Special Interest.

DKA is defined clinically and biochemically as a combination of a state of elevated ketone levels (urine ++ or higher, and/or blood > 1.5 mmol/L) in combination with biochemical evidence of a state of metabolic acidosis (pH ≤7.30 or serum bicarbonate < 15 mmol/L). This is as defined by International Consensus Guidelines (34). DKA also varies in severity. In clinical practice the diagnosis is made when a person with type 1 diabetes has elevated ketone levels and usually – although not exclusively – elevated blood glucose levels. The diagnosis is made biochemically by assessing acid base balance through blood testing. This corresponds to the following severity classifications:

- mild DKA: pH 7.25-7.30 (H^+ 50 – 56) or bicarbonate (15 -17.9 mmol/L)
- moderate DKA: pH 7.00-7.24 (H^+ 57 – 100) or bicarbonate 10 – 14.9 mmol/L)
- severe DKA: pH <7.00 (H^+ > 100) or bicarbonate < 10 mmol/L (33)

Given the known risk of DKA, all participants will be made aware of this condition. Patient diaries and Patient Alert Cards will summarise advice for management of high ketone levels including an emergency ketosis management protocol (STICH Protocol – see Appendix 3). All participants will be educated as to the scenarios when this should be activated and this will also be included on the Patient Alert Card which will have the contact details of the study team and participants will be asked to carry at all times.

(ii) Hypoglycaemia

Hypoglycaemia is a low blood glucose level. Hypoglycaemia occurs in diabetes owing to difficulty in precisely matching insulin dosing with the complex factors associated with carbohydrate intake and physical activity. When too much insulin relative to carbohydrate intake is administered, blood glucose levels can drop to a level which is too low. Most people with type 1 diabetes can recognize this phenomenon due to a combination of symptoms which alert them and prompt self-checking of their capillary blood glucose level. Symptoms include weakness, malaise, headache, tremulousness, diaphoresis, palpitations and mild confusion. These are known as autonomic symptoms as they are mediated by activation of the autonomic nervous system as a response to the biological stress of low serum blood glucose. As hypoglycaemia progresses, however, symptoms progress to include confusion and agitation. If untreated this can lead to coma. These symptoms are known as

neuroglycopenic symptoms as they are mediated by a lack of glucose in neural cells leading to a confused state.

Hypoglycaemia is also well defined by International Consensus (34). Like DKA, it can be classified in terms of severity. This can be based either on the absolute capillary glucose value and/or clinical features and is classed into stages:

- Level 1 (mild) hypoglycaemia: Blood glucose is 3.0 – 3.8 mmol/L inclusive.
- Level 2 (moderate) hypoglycaemia: Blood glucose is < 3.0 mmol/L
- Level 3 (severe) hypoglycaemia: A person is unable to function because of mental or physical changes due to hypoglycaemia and requires assistance from another person to recover.

The IMPs in this trial are licensed clinically in type 2 diabetes to lower blood glucose levels. It therefore follows that when used in combination with insulin there may be an increased risk of low blood glucose levels. This was the case with the once daily GLP-1 analogue liraglutide in phase 3 trials (8,9), but not in the trials with dapagliflozin (13,14). All participants will have this risk discussed with them, will be performing increased blood glucose monitoring than is the case in usual care, and will be frequently reviewed to adjust insulin dosing in order to mitigate the risk of low blood glucose levels.

9.3.2 Site Reported Serious Adverse Events (SAE)

SAEs will be recorded within the eCRF from the date of randomisation until the later of 30 days post cessation of trial treatment or the end of the study.
Full details of SAEs will be recorded in the electronic Case Report Form. The following information will be collected:

- full details in medical terms and a case description
- seriousness criteria
- event duration (start and end dates, if applicable)
- action taken
- outcome
- causality (i.e. relatedness to trial drug / investigation), in the opinion of the investigator
- if related, whether the reaction would be considered expected or unexpected.

Any change of condition or other follow-up information should be added to the eCRF and forwarded to the Sponsor (if reportable SAE) as soon as it is available or at least within 24 hours of the information becoming available. Events will be followed up until the event has resolved or a final outcome has been reached.

Reporting of suspected/confirmed COVID 19 infection

If a patient presents with clinical signs and symptoms consistent with COVID-19 and a test has been performed or requested:

- If test is positive, record “COVID-19 confirmed” in the diagnosis field
- If test is negative, record the SAE signs and symptoms and/or other diagnosis in the diagnosis field.

If a test has not been performed or result is not available and signs and symptoms, as judged by the investigator, are highly suspicious of COVID-19 infection, record “COVID-19 suspected” in the diagnosis field

If other concurrent diagnoses, e.g. pneumonia, please record within the narrative of the SAE.

Assessment of Adverse Events

All adverse events must be assessed by the reporting site for seriousness**.** All SAEs must also be assessed for causality and expectedness with reference to this protocol and the Reference Safety Information (RSI).

Assessment of seriousness

An adverse event will be considered serious if it:

1. Results in death
2. Is life threatening
3. Requires hospitalisation or prolongation of existing hospitalisation
4. Results in persistent or significant disability or incapacity
5. Consists of a congenital anomaly or birth defect
6. Required intervention to prevent one of the above.
7. Is a serious Adverse Event of Special Interest.

Assessment of causality i.e. does the event have a “reasonable causal relationship” with trial IMPs. The following categories are used:

- **Yes** - The causal and temporal relationship between the event and the absence of a more likely explanation suggest that the trial IMP is the most likely cause.
- **No** - The event is not considered related to the trial IMPs.

Causality will be assigned for each IMP the participant is randomised to receive.

Should an SAE be considered unrelated to the administration of either trial drug then the causality of the event will be collected taking into consideration other factors, for example concomitant medications or co-morbidities, etc.

Assessment of causality is the responsibility of the Principal Investigator or medically-qualified designee(s) at each site. The CI, their delegate(s), or the Sponsor may provide an opinion regarding causality but cannot downgrade the PI’s assessment.

Assessment of expectedness

Should the event be considered to be related to the trial IMP(s), an assessment should be made regarding the expectedness of the reaction, i.e. whether the reaction is a recognised adverse effect of the IMP(s).

.

Expected: consistent with the relevant product information documented in the RSI(s).
Unexpected: not consistent with the relevant product information documented in the RSI(s).

The CI, their delegate(s), or the Sponsor have overall responsibility for assigning expectedness as per the RSI(s) and provide the final assessment of the event. The reporting investigator may provide an opinion on the expectedness of an event and this may be taken into account.

SAEs occurring within the USA and the UK

The US Sponsor is responsible for assigning causality and expectedness for any AEs, SAEs and SUSARs that occur within the USA. Where a SUSAR is identified by the US Sponsor this will be forwarded to the UK Sponsor for review within one working day. Following review by the UK Sponsor, appropriate US SUSARs will be subject to expedited reporting to the MHRA as per UK regulatory requirements. Similarly, where a SUSAR is identified by the UK sponsor, this will forwarded to the US Sponsor for review within two working days.

Recording and reporting of SAEs

All SAEs arising during the clinical trial will be recorded in the eCRF soon as reasonably practicable and in any event within 24hours of first becoming aware of the event. Any follow-up information should also be reported.

If recording in the eCRF is not possible a paper SAE form should be completed:

1. The SAE form is downloaded from www.glasgowctu.org, printed off, completed and signed. The form is then faxed to the Glasgow Clinical Trials Unit Pharmacovigilance (PV) Office on +44(0)141 357 5588. As an alternative, a scanned copy of the SAE form may be emailed to: pharmacovig@glasgowctu.org. If this website is unavailable a paper copy of the SAE form should be filed in the Investigator Site File at each site pending resolution.
2. If necessary a verbal report can be given by contacting the PV Office on +44(0)141 330 4744. This must be followed up as soon as possible with an electronic or written report.

Reporting to sponsor

All SAEs will be considered reportable to the Sponsor PV office from the time of randomisation until 30 days following permanent discontinuation of all trial IMP(s). SAEs occurring following 30 days post cessation of treatment are not reportable to the Sponsor unless an SAE is considered related to IMP(s)

9.3.3 Suspected Unexpected Serious Adverse Reactions (SUSARs)

Any SAE assessed as related to trial IMP(s) by the PI or delegate, and unexpected (not documented as an expected reaction to the IMP(s) within the RSI(s)) by the CI, their delegate, or the Sponsor, will be classified as a SUSAR and therefore subject to expedited reporting to the Medicines and Healthcare products Regulatory Agency (MHRA) and the Research Ethics Committee (REC). Should the CI disagree with the PI's assessment of causality, both opinions will be provided on the report. Where the CI or UK Sponsor disagrees with the US Sponsor's assessment of expectedness both opinions will also be provided on the report.

The Sponsor will inform the MHRA of all SUSARs that occur within the trial and the REC of all SUSARs that occur within the UK within the required expedited reporting timescales:

- **Fatal or life threatening SUSARs** - no later than 7 days of Sponsor awareness that the case fulfils the criteria for a fatal or life threatening SUSAR, and any follow up information within a further 8 days.

- **All other SUSARs** - no later than 15 days of Sponsor awareness that the case fulfils the criteria for a SUSAR.

The sponsor will report SUSARs to the MHRA via the MHRA eSUSAR reporting system and to REC by email with accompanying CTIMP Safety Report Form.

9.3.4 Unblinding for SUSAR reporting purposes

In the event of a potential SUSAR, the sponsor (but not the investigators) will be unblinded to facilitate appropriate reporting to the MHRA and REC.

9.4 Responsibilities for Safety Reporting and Review

This section details the responsibilities for reporting and reviewing safety information arising from the trial.

Principal Investigator (PI):

- Attendance at the initiation meeting/teleconference
- Training of new members of the trial team in the protocol and its procedures
- Ensuring that the ISF is accurately maintained
- Dissemination of important safety or trial-related information to all stakeholders at their site
- Checking for AEs and ARs when participants attend for treatment/follow-up
- Ensuring that AEs are recorded and reported in line with the requirements of the protocol
- Ensuring that all SAEs are recorded and reported to the Sponsor within 24 hours of becoming aware of the event, and providing further follow-up information as soon as available
- Using medical judgement in assigning causality of SAEs
- Providing an opinion on the expectedness of an SAE with reference to the trial protocol and Reference Safety Information
- Using definitions in this protocol, flagging events of special interest.

Chief Investigator (CI)

- Providing clinical oversight of the safety of patients participating in the trial, including an ongoing review of the risk/benefit
- Providing clinical review of section 4.8 of the representative SmPCs on an annual basis to define the Reference Safety Information for the trial
- Using medical judgement to confirm seriousness and causality of all SARs
- Reviewing expectedness of related SAEs with reference to the trial protocol and Reference Safety Information
- Preparing the clinical sections and final sign-off of the Development Safety Update Report (DSUR)
- Using definitions in this protocol, confirming events of special interest

Sponsor:

- Central data collection and verification of AEs, SAEs, SARs and SUSARs according to the trial protocol
- Reporting safety information to the CI or delegate for the ongoing assessment of the risk / benefit

- Reporting safety information to the independent oversight committees identified for the trial (Independent Data Monitoring Committee (IDMC) and / or Trial Steering Committee (TSC)) according to the Trial Monitoring Plan.
- Expedited reporting of SUSARs to the Competent Authority (MHRA in UK) and REC within required timelines.
- Notifying Investigators of SUSARs that occur within the trial.
- Checking for (annually) and notifying PIs of updates to the Reference Safety Information for the trial.
- Preparing standard tables and other relevant information for the DSUR in collaboration with the CI and ensuring timely submission to the MHRA and REC.

Trial Steering Committee:
In accordance with the Charter for the TSC, periodically reviewing recruitment and the overall progress of the trial and liaising with the IDMC and sponsor regarding safety issues.

Independent Data Monitoring Committee:
In accordance with the Charter for the IDMC, periodically reviewing unblinded safety data in individual cases and to determine patterns and trends of events, or to identify safety issues, which would not be apparent on an individual case basis, reporting concerns to the TSC and sponsor.

9.5 Pregnancy reporting

Pregnancy is not considered an AE unless a negative or consequential outcome is recorded for the mother or child/foetus. If the outcome meets the serious criteria, this would be considered an SAE and must be reported as per SAE reporting procedure above.
Any **pregnancy** occurring in a female trial participant or female partner of a male trial participant who becomes pregnant while participating in the Trial will be reported by the PI (or designee) to the Chief Investigator and the sponsor using the sponsor Pregnancy Reporting Form (available at http://www.glasgowctu.org/) within two weeks of the PI first becoming aware of the pregnancy.
The trial participant will also be followed up to determine the outcome of the pregnancy and follow-up information forwarded to the PV office. Any resulting SAEs should be reported as per SAE reporting procedure above.

9.6 Overdose

Any IMP dose which is not administered in accordance with the Protocol should be reported to the sponsor.
If an SAE is associated with an overdose ensure that the overdose if fully described in the SAE report form.

9.7 Reporting urgent safety measures

If any urgent safety measures are taken the CI/Sponsor will phone the MHRA's Clinical Trial Unit on 020 3080 6456, ideally within 24 hours. This will be followed up no later than 3 days from the date the measures are taken, giving written notice to the MHRA (who will advise the format required) and the relevant REC of the measures taken and the circumstances giving rise to those measures. A substantial amendment must also be submitted to the MHRA.

9.8 The type and duration of the follow-up of participants after adverse events.

Adverse events and reactions will be recorded, reported and followed up in line with this protocol until study completion or for a minimum of 30 days after participant's last dose of the IMP, whichever is later.
Any SUSAR identified will be reported to the Sponsor and to the Regulatory Authorities irrespective of how long after IMP administration the reaction has occurred.

9.9 Development safety update reports

A Development Safety Update Report (DSUR) will be submitted once a year, or on request, to MHRA and REC until the trial is declared ended. The report will be submitted within 60 days of the anniversary of the issue of the Clinical Trials Authorisation for the trial. The DSUR will be prepared by the sponsor (PV Office) in liaison with the CI and submitted by the sponsor (PV Office). The DSUR will include only those SAEs reported to the co-sponsors; that is: SAEs and SUSARs occurring within the UK and SUSARs originating in the USA.

10 STATISTICS AND DATA ANALYSIS

10.1 Sample size calculation

This is based on our current data showing that in type 1 diabetes, treatment with a GLP-1 agonist, liraglutide (1.2 and 1.8mg doses) for three months leads to HbA1c suppression by an average of 0.6% (SD= 0.5%). Dapagliflozin was recently shown to reduce HbA1c by 0.45% in type 1 diabetes patients (13). Additionally, empagliflozin treatment for 8 weeks was shown to reduce HbA1c by 0.4% in type 1 diabetics (20, 21) and our current data showing additional improvement in HbA1c by an additional 0.66% at 12 weeks when dapagliflozin was added to liraglutide and insulin therapy. Conservatively estimating that the combination therapy will suppress HbA1c by 0.5% (SD≤0.7%) compared to semaglutide alone, a sample size of 38 (32 evaluable patients) in each group will be sufficient to provide 80% power at a 5% significance level, taking in consideration a 15% dropout rate. 50% of participants will be recruited in Glasgow, UK and 50% at State University of New York at Buffalo, USA.

10.2 Anticipated recruitment rate

One year (one participant per week).

10.3 Statistical analysis

The primary objective of the proposed research is to evaluate the effect of addition of dapagliflozin to semaglutide on a background of insulin treatment in type 1 diabetes patients on HbA1c at six months following start of dapagliflozin. Transformations of the data in order to meet statistical assumptions will be considered. Statistical analyses will use linear regression models to compare treatment groups, with adjustment for baseline values of study outcomes, as well as factors used to stratify the randomisation scheme. Tests will be two-sided and tested at the 5% significance level. Analyses will be carried out under the intention to treat principle, that is, according to randomised treatment allocation, regardless of treatment adherence.

A detailed Statistical Analysis Plan will be completed and signed off before database lock. Study statisticians will develop and validate the analysis programs without access to randomised treatment allocations. After database lock, the treatment codes will be released and the final analysis will be carried out.

10.4 Subgroup analyses

These will be specified in the Statistical Analysis Plan. Subgroup analyses will be implemented by extending regression models to include interactions between treatment and potential treatment effect moderators.

10.5 Interim analysis and criteria for the premature termination of the trial

No interim analysis is planned. Unblinded trial data will be reviewed on an ongoing basis by the IDMC. The primary role of the IDMC will be to protect the interests of the patients. The IDMC may recommend to the TSC and Co-Sponsors that the study should stop prematurely because of concerns about patient safety. The IDMC will meet approximately every six months. The IDMC will take into account all results and the consistency and biological plausibility of the findings in making any recommendation. The final decision on continuing or stopping the trial will lie with the TSC/Co-Sponsors.

10.6 Subject population

All analyses will be carried out on an intention to treat basis, using the randomised treatment allocation.

10.7 Procedure(s) to account for missing or spurious data

Data will be analysed without imputation in the first instance. Multiple imputation procedures will be used to assess the sensitivity of selected results to missing data, according to the Statistical Analysis Plan.

10.8 Other statistical considerations.

The Statistical Analysis Plan (SAP) will be maintained as a version controlled document and will be signed off before database lock. The SAP and other statistical documentation (according to Glasgow Clinical Trials Unit Standard Operating Procedures) will contain full details of all analyses along with assumptions and procedures for handling problematic or incomplete data (e.g. incomplete dates).

11 DATA HANDLING

11.1 Source Documentation

ICH GCP defines source data as: ‘All information in original records and certified copies of original records of clinical findings, observations or other activities in a clinical trial necessary for the reconstruction and evaluation of the trial’. In this study, the location of the majority of the source data will be the hospital's medical records including subject case notes, laboratory records and ECGs. The source data transcribed into the eCRF from the medical records must be accurate and verifiable. For questionnaires completed by trial subjects, the completed questionnaires will be regarded as the source data location. In cases where data is transcribed directly into the eCRF and no other paper or electronic source exists, then the eCRF will be considered the source record. In these cases, these data should be prospectively documented in the medical records to ensure a full record of the trial is available at site.

11.2 Data collection

An eCRF, developed by the Robertson Centre for Biostatistics, will capture all data required to meet this protocol’s requirements. Access to the eCRF will be restricted, via a study-specific web portal, and only authorised site-specific personnel will be able to make entries to their patients’ data via the web portal. The Investigator, or his/her designee will be responsible for all entries into the eCRF and will

confirm that the data are accurate, complete and verifiable. Data will be stored in a MS SQL Server database.
Direct access to the web portal will be granted, on request, to authorised representatives from the Sponsor, host institution and the regulatory authorities to permit trial-related monitoring, audits and inspections.

11.3 Data Validation

Where it is practical, data will be validated at the point of entry into the eCRF. Any additional data discrepancies will be flagged to the investigator and any data changes will be recorded to maintain a complete audit trail (reason for change, date change made, who made change).

11.4 Data Security

The Robertson Centre for Biostatistics systems are fully validated in accordance with industry and regulatory standards, and incorporate controlled access security. High volume servers are firewall protected and preventative system maintenance policies are in place to ensure no loss of service or data. Web servers are secured by digital certificates. Data integrity is assured by strictly controlled procedures, including secure data transfer procedures. Data are backed up on-site nightly and off-site to a commercial data vault weekly. The Robertson Centre for Biostatistics has an ISO 9001:2008 quality management system and ISO 27001:2013 for Information Security, and is regularly inspected against the standards by the British Standards Institution.

11.5 Archiving

The Trial Master File will be archived by the Co-Sponsors at the end of the trial for a minimum period of five years.

Archiving of Site Files will also be for a minimum of five years form completion of the trial, and this action will be delegated to the sites in the Clinical Trial Site Agreement that will be put in place between Co-Sponsors and Sites. Sites will be notified by the Co-Sponsors when Site files can be archived.

Destruction of site files can only take place with the approval of the Co-Sponsors.

12 MONITORING, AUDIT & INSPECTION

Monitoring will be conducted by NHS Greater Glasgow and Clyde (GG&C) Monitor (s) in accordance with local Standard Operating Procedures. The level, frequency and priorities of monitoring will be based on the outcome of the completed Risk Assessment, and clearly documented in the Monitoring Plan which will be approved by the NHS GG&C Lead Monitor and/or Research Governance Manager.

13 ETHICAL AND REGULATORY CONSIDERATIONS

13.1 Research Ethics Committee (REC) review & reports

Before the start of the trial, approval will be sought from a REC for the trial protocol, informed consent forms and other relevant documents e.g. advertisements and GP information letters.
Substantial amendments that require review by REC will not be implemented until the REC grants a favourable opinion for the study (it is noted that amendments may also need to be reviewed and accepted by the MHRA and/or NHS R&D departments before they can be implemented in practice at sites).

All correspondence with the REC will be retained in the Trial Master File/Investigator Site File.
An annual progress report (APR) will be submitted to the REC within 30 days of the anniversary date on which the favourable opinion was given, and annually until the trial is declared ended (this is the Chief Investigator's responsibility).
The Chief Investigator will notify the REC of the end of the study.
If the study is ended prematurely, the Chief Investigator will notify the REC, including the reasons for the premature termination
Within one year after the end of the study, the Chief Investigator will submit a final report with the results, including any publications/abstracts, to the REC.

13.2 Peer review

The study protocol has been developed with expert and independent feedback from the JDRF (New York) and during application for funding underwent peer review by independent experts.

13.3 Public and Patient Involvement

The study was designed with input from people with type 1 diabetes via the funder, JDRF.

13.4 Regulatory Compliance

The trial will not commence until a Clinical Trial Authorisation (CTA) is obtained from the MHRA.
The protocol and trial conduct will comply with the Medicines for Human Use (Clinical Trials) Regulations 2004 and any relevant amendments.
Before any site can enrol patients into the trial, the Chief Investigator/Principal Investigator or designee will apply for NHS permission from the site's Research & Development (R&D) department.

For any amendment that will potentially affect a site's NHS permission, the Chief Investigator/ Principal Investigator or designee will confirm with that site's R&D department that NHS permission is ongoing (note that both substantial amendments, and amendments considered to be non-substantial for the purposes of REC and/or MHRA may still need to be notified to NHS R&D).

13.5 Protocol compliance

Prospective, planned deviations or waivers to the protocol are not allowed under the UK regulations on Clinical Trials and must not be used e.g. it is not acceptable to enrol a participant if they do not meet the eligibility criteria or restrictions specified in the trial protocol. Accidental protocol deviations can happen at any time. They must be adequately documented on the relevant forms and reported to the Chief Investigator, Sponsor and GCTU immediately. Deviations from the protocol which are found to frequently recur are not acceptable, will require immediate action and could potentially be classified as a serious breach.

13.6 Notification of Serious Breaches to GCP and/or the protocol

A "serious breach" is a breach which is likely to affect to a significant degree –

1. the safety or physical or mental integrity of the subjects of the trial; or
2. the scientific value of the trial

If any of the above occurs then the CI and Sponsor will be notified. The sponsor will notify the appropriate authorities in writing of any serious breach in accordance with their standard operating procedures.

13.7 Data protection and patient confidentiality

All investigators and trial site staff must comply with the requirements of the GDPR with regards to the collection, storage, processing and disclosure of personal information and will uphold the Act's core principles.

- Personal information will be collected via the eCRF to enable record linkage to be carried out and to provide electronic access to study monitors to a copy of the signed informed consent document. These data items will be encrypted and only those individuals who require to see these data i.e. the person performing the record linkage and site research team staff or the study monitor, as appropriate, will be able to view them. All electronic data will be held securely in accordance with ISO 27001:2013 at the Robertson Centre for Biostatistics, part of the Glasgow Clinical Trials Unit. All Centre staff are required to sign confidentiality agreements and to follow Standard Operating Procedures in accordance with Good Clinical Practice and ISO certification.
- The trial data managers, statisticians, health economists or any other staff who will perform data related tasks will only be able to access depersonalised data where the participant's identifying information is replaced by a unique study identifier.
- Only those that have been trained and approved will be able to enter or view any data via the web portal. Each site can only see their own patients' data. Patient consent forms will be stored at the study site in a secure location accessible only to study teams.

13.8 Indemnity

The Co-Sponsors (University of Glasgow and Greater Glasgow Health Board) will ensure that provision has been made for insurance or indemnity to cover the liability of the investigator and sponsor which may arise in relation to the clinical trial in accordance with Part 2 (14) of Schedule 1 to SI 2004/1031.

13.9 Amendments

Any change in the study protocol will require an amendment. Any proposed protocol amendments will be initiated by the CI following discussion with the Sponsor and TSC and any required amendment forms will be submitted to the regulatory authority, ethics committee and Sponsor. The Sponsor will determine whether an amendment is non-substantial or substantial. All amended versions of the protocol will be signed by the CI and Sponsor representative. Following a substantial amendment, favourable opinion/approval must be sought from the original reviewing REC, MHRA (where appropriate) and Research and Development (R&D) office prior to implementation. The Chief Investigator will be responsible for informing the Trial Management Group of all protocol amendments.

13.10 Post trial care

At the end of the trial, participants will be returned to usual care as defined by local and national guidelines at that time. The results of the trial may of course have an impact on these guidelines and the future care of patients with type 1 diabetes.

13.11 Access to the final trial dataset

During the trial and in the period prior to publication of the main study results as described in the protocol, only the Glasgow CTU and Professor Paresh Dandona, US Principal Investigator, University of Buffalo will have access to the full dataset. After that period, the trial Steering Committee will conduct further data analyses for a period of three years. After that time the Trial Steering Committee will consider requests from external parties for further analyses of the study data. Proposals that are scientifically well founded and have an academic basis and where relevant data extractions and analyses are appropriately funded

will not be refused. These will be considered as collaborative exercises where the contributions related to study design, conduct, database creation and maintenance and data analysis will be recognised in authorship of any scientific publication. The approach we will take will be to minimise any possibility of breach of participant confidentiality. Normally this will be achieved by minimising data travel. However, for the purposes of individual patient meta-analysis and other reasons, data may be transferred to other sites. Such transfer will require assurances on information security systems at the sites that data are to be transferred to and will involve a legal data transfer agreement. A log of all data requests and subsequent data transfers will be held at the Glasgow CTU.

14 DISSEMINATION POLICY

14.1 Dissemination policy

The study database will be owned by the University of Glasgow and maintained on behalf of the Study investigators, represented by the Trial Steering Committee as it is constituted during and after the trial.

The study protocol and a description of the recruitment experience and participant baseline characteristics will be published before study completion. On completion of the trial, the database will be locked and analysed by staff of the Robertson Centre for Biostatistics, University of Glasgow. A final study report will be prepared and the results will be published in a major medical journal.

After the main study publications, study investigators will be invited to submit requests for further analyses of the study database. These will be reviewed and prioritised by a Publications Committee made up of the study grant holders and convened by the study co-CIs.

As funder of the trial, JDRF will have the right to see and comment on any results being submitted for publication. A maximum of 28 days will be given for review of major papers and 14 days for abstracts. Such comments will be considered by the Trial Steering Committee.

A lay summary of the main results of the trial will be prepared and provided to all participants via their study site investigators. Investigators may request a copy of the study data for their participants. Providing some or all of a patient's data to that individual is at their discretion.

14.2 Authorship eligibility guidelines and any intended use of professional writers

The main results of the study will be compiled, written up and published by the study grant holders and others taking responsibility for the study results (e.g. the statistician conducting the final analysis) on behalf of the TTT1 investigators. The TTT1 investigators will be listed in an Appendix and will include all site PIs, all committee members and key members of relevant study coordinating groups (including the Sponsor and Glasgow CTU).

15 REFERENCES

1. Konrad K, Datz N, Engelsberger I, Grulich-Henn J, Hoertenhuber T, Knauth B, et al. Current use of metformin in addition to insulin in pediatric patients with type 1 diabetes mellitus: an analysis based on a large diabetes registry in Germany and Austria. *Pediatr Diabetes.* 2014.
2. Vella S, Buetow L, Royle P, Livingstone S, Colhoun HM, and Petrie JR. The use of metformin in type 1 diabetes: a systematic review of efficacy. *Diabetologia.* 2010;53(5):809-20.
3. Vella S, Buetow L, Royle P, Livingstone S, and Petrie JR. Metformin in type 1 diabetes reduces insulin requirements without significantly improving glycaemic control. Reply to Schatz H [letter]. *Diabetologia.* 2011;54(1):203-4.
4. Varanasi A, Bellini N, Rawal D, Vora M, Makdissi A, Dhindsa S, et al. Liraglutide as additional treatment for type 1 diabetes. *Eur J Endocrinol.* 2011;165(1):77-84.
5. Kuhadiya ND, Malik R, Bellini NJ, Patterson JL, Traina A, Makdissi A, et al. Liraglutide as additional treatment to insulin in obese patients with type 1 diabetes mellitus. *Endocrine practice : official journal of the American College of Endocrinology and the American Association of Clinical Endocrinologists.* 2013;19(6):963-7.
6. Kuhadiya ND, Dhindsa S, Ghanim H, Mehta A, Makdissi A, Batra M, et al. Addition of Liraglutide to Insulin in Patients With Type 1 Diabetes: A Randomized Placebo-Controlled Clinical Trial of 12 Weeks. *Diabetes Care.* 2016;39(6):1027-35.
7. Chaudhuri A, Ghanim H, Makdissi A, Green K, Abuaysheh S, Batra M, et al. Exenatide induces an increase in vasodilatory and a decrease in vasoconstrictive mediators. *Diabetes Obes Metab.* 2017;19(5):729-33.
8. Mathieu C, Zinman B, Hemmingsson JU, Woo V, Colman P, Christiansen E, et al. Efficacy and Safety of Liraglutide Added to Insulin Treatment in Type 1 Diabetes: The ADJUNCT ONE Treat-To-Target Randomized Trial. *Diabetes Care.* 2016;39(10):1702-10.
9. Ahren B, Hirsch IB, Pieber TR, Mathieu C, Gomez-Peralta F, Hansen TK, et al. Efficacy and Safety of Liraglutide Added to Capped Insulin Treatment in Subjects With Type 1 Diabetes: The ADJUNCT TWO Randomized Trial. *Diabetes Care.* 2016;39(10):1693-701.
10. Zinman B, Wanner C, Lachin JM, Fitchett D, Bluhmki E, Hantel S, et al. Empagliflozin, Cardiovascular Outcomes, and Mortality in Type 2 Diabetes. *N Engl J Med.* 2015;373(22):2117-28.
11. Petrie JR. SGLT2 inhibitors in type 1 diabetes: knocked down, but up again? *Lancet Diabetes Endocrinol.* 2017;5(11):841-3.
12. Mudaliar S, Armstrong DA, Mavian AA, O'Connor-Semmes R, Mydlow PK, Ye J, et al. Remogliflozin etabonate, a selective inhibitor of the sodium-glucose transporter 2, improves serum glucose profiles in type 1 diabetes. *Diabetes Care.* 2012;35(11):2198-200.
13. Dandona P, Mathieu C, Phillip M, Hansen L, Griffen SC, Tschope D, et al. Efficacy and safety of dapagliflozin in patients with inadequately controlled type 1 diabetes (DEPICT-1): 24 week results from a multicentre, double-blind, phase 3, randomised controlled trial. *Lancet Diabetes Endocrinol.* 2017;5(11):864-76.
14. Mathieu C, Dandona P, Gillard P, Senior P, Hasslacher C, Araki E, Lind M, Bain SC, Jabbour S, Arya N, Hansen L, Thorén F, Langkilde AM; DEPICT-2 Investigators. Efficacy and Safety of Dapagliflozin in Patients With Inadequately Controlled Type 1 Diabetes (the

DEPICT-2 Study): 24-Week Results From a Randomized Controlled Trial. Diabetes Care. 2018 Sep;41(9):1938-1946.

15. European Medicines Agency. Assessment Report Dapagliflozin. https://www.ema.europa.eu/en/documents/variation-report/forxiga-h-c-2322-ws-1344-epar-assessment-report-variation_ar.pdf
16. Rosenstock J, Marquard J, Laffel LM, et al. Empagliflozin as adjunctive to insulin therapy in type 1 diabetes: the EASE trials. *Diabetes Care*. 2018;41(12):2560–2569.
17. Garg SK, Henry RR, Banks P, Buse JB, Davies MJ, Fulcher GR, et al. Effects of Sotagliflozin Added to Insulin in Patients with Type 1 Diabetes. *N Engl J Med.* 2017;377(24):2337-48.
18. European Medicines Agency. First oral add-on treatment to insulin for treatment of certain patients with type 1 diabetes. https://www.ema.europa.eu/en/news/first-oral-add-treatment-insulin-treatment-certain-patients-type-1-diabetes
19. An overview of Zynquista and why it is authorised in the EU. 16th May 2019. https://www.ema.europa.eu/en/documents/overview/zynquista-epar-medicine-overview_en.pdf
20. National Institute for Health and Care Excellence. Dapagliflozin with insulin for treating type 1 diabetes.
21. Ahrén B, Hirsch IB, Pieber TR, Mathieu C, Gómez-Peralta F, Hansen TK, Philotheou A, Birch S, Christiansen E, Jensen TJ, Buse JB; ADJUNCT TWO Investigators. Efficacy and Safety of Liraglutide Added to Capped Insulin Treatment in Subjects With Type 1 Diabetes: The ADJUNCT TWO Randomized Trial. Diabetes Care. 2016 Oct;39(10):1693-701.
22. Ozempic Summary of product characteristics. https://www.medicines.org.uk/emc/product/9728/smpc
23. Nauck MA, Petrie JR, Sesti G, Mannucci E, Courrèges JP, Lindegaard ML, Jensen CB, Atkin SL; Study 1821 Investigators. A Phase 2, Randomized, Dose-Finding Study of the Novel Once-Weekly Human GLP-1 Analog, Semaglutide, Compared With Placebo and Open-Label Liraglutide in Patients With Type 2 Diabetes. Diabetes Care. 2016 Feb;39(2):231-41.
24. Technology appraisal guidance [TA597] https://www.nice.org.uk/guidance/ta597
25. Dapagliflozin Summary of Product Characteristics: https://www.medicines.org.uk/emc/product/2865/smpc
26. Wiviott SD, Raz I, Bonaca MP, Mosenzon O, Kato ET, Cahn A, Silverman MG, Zelniker TA, Kuder JF, Murphy SA, Bhatt DL, Leiter LA, McGuire DK, Wilding JPH, Ruff CT, Gause-Nilsson IAM, Fredriksson M, Johansson PA, Langkilde AM, Sabatine MS; DECLARE–TIMI 58 Investigators. Dapagliflozin and Cardiovascular Outcomes inType 2 Diabetes. N Engl J Med. 2019 Jan 24;380(4):347-357.
27. McMurray JJV, Solomon SD, Inzucchi SE, Køber L, Kosiborod MN, Martinez FA et al. DAPA-HF Trial Committees and Investigators Dapagliflozin in patients with heart failure and reduced ejection fraction. N Engl J Med. 2019. Nov 21;381(21):1995-2008.
28. Vilsbøll T, Bain SC, Leiter LA, et al. Semaglutide, reduction in glycated haemoglobin and the risk of diabetic retinopathy. *Diabetes Obes Metab*. 2018;20(4):889–897.
29. Lingvay I, Desouza CV, Lalic KS et al. A 25 week randomized controlled trial of semaglutide once daily versus liraglutide and placebo in patients with type 2 diabetes

suboptimally controlled on diet nad exercise with or without metformin. Diabetes Care. 2018;41(9): 1926-1937.

30. European Medicines Agency. Assessment Report Sotagliflozin. https://www.ema.europa.eu/en/documents/assessment-report/zynquista-epar-public-assessment-report_en.pdf
31. Peters AL, Buschur EO, Buse JB, Cohan P, Diner JC, and Hirsch IB. Euglycemic Diabetic Ketoacidosis: A Potential Complication of Treatment With Sodium-Glucose Cotransporter 2 Inhibition. Diabetes Care. 2015;38(9):1687-93.
32. Silverii GA, Decembrini L, Monami M, Mannucci E. Fournier's gangrene and sodium glucose co-transporter 2 inhibitors: A metanalysis of randomized controlled trials. Diabetes Obes Metab. 2019; 1-4.
33. Agiostratidou G, Anhalt H, Ball D, Blonde L, Gourgari E, Harriman KN, Kowalski AJ, Madden P, McAuliffe-Fogarty AH, McElwee-Malloy M, Peters A, Raman S, Reifschneider K, Rubin K, Weinzimer SA. Standardizing Clinically Meaningful Outcome Measures Beyond HbA(1c) for Type 1 Diabetes: A Consensus Report of the American Association of Clinical Endocrinologists, the American Association of Diabetes Educators, the American Diabetes Association, the Endocrine Society, JDRF International, The Leona M. and Harry B. Helmsley Charitable Trust, the Pediatric Endocrine Society, and the T1D Exchange. Diabetes Care. 2017 Dec;40(12):1622-1630.
34. Garg SK, Peters AL, Buse JB, Danne T. Strategy for mitigating DKA risk in patients with Type 1 diabetes on adjunctive treatment with SGLT-2 inhibitors: A STICH Protocol. Diabetes Technol Ther. 2018;20(9):571-575.

16. APPENDICES

16.1 Appendix 1 – Risk

Risks associated with trial interventions<br>☐ LOW ≡ Comparable to the risk of standard medical care<br>☒ MODERATE ≡ Somewhat higher than the risk of standard medical care<br>☐ HIGH ≡ Markedly higher than the risk of standard medical care				
Justification: Briefly justify the risk category selected and your conclusions below (where the table is completed in detail the detail need not be repeated, however a summary should be given):<br>An IDMC will be convened to monitor all SAEs.<br>Risks and mitigations associated with the intervention are outlined in more detail in the Protocol section 2.1.<br>Co-Sponsors will also carry out a detailed risk assessment of all aspects of the study as part of the approval process (SOP 04.013)				
What are the key risks related to therapeutic interventions you plan to monitor in this trial?		How will these risks be minimised?		
IMP/Intervention	Body system/Hazard	Activity	Frequency	Comments
S/C administration of semaglutide<br>Oral admistration of dapagliflozin	Immune: Hypersensitivity/ana-phylactic reactions	Patients with known hypersensitivity to any GLP-1 agonist or SGLT-2 inhibitor have a contra-indication to the IMP according to the SmPC will not be recruited to the study	Uncommon/ Rare	
PO administration of dapagliflozin	Ketosis and/or Diabetic Ketoacidosis (DKA)	All participants will have access to blood and urine ketone monitoring equipment. An emergency ketosis management plan (STICH Protocol – see above) will be discussed with all participants. Patient alert cards will be provided to all participants. Potential participants with recent ketoacidosis will be excluded	Common	

		from the trial (section 6.2)		
S/C administration of semaglutide (+/-) PO administration of dapagliflozin in addition to insulin therapy	Increased risk of hypoglycaemia	Participant awareness of risk and frequent testing of capillary blood glucose. Patients with pre-existing frequent episodes of hypoglycaemia or problems with hypoglycaemia awareness will be excluded from trial participation (section 6.2)	Common	
Outline any other processes that have been put in place to mitigate risks to participant safety (e.g. IDMC, independent data review, etc.)<br>See above				

16.2 Appendix 2: Contraception

In line with recommendations related to contraception and pregnancy testing in clinical trials from the Clinical Trial Facilitation Group (CTFG), for women of childbearing potential* (WOCBP) participating in TTT1, methods of contraception considered as highly effective are defined as those that can achieve a failure rate of less than 1% per year when used consistently and correctly.

Such methods include:

- combined (oestrogen and progestogen containing) hormonal contraception associated with inhibition of ovulation[1]
    - oral
    - intravaginal
    - transdermal
- progestogen-only hormonal contraception associated with inhibition of ovulation[1]
    - oral
    - injectable
    - implantable[2]
- intrauterine device (IUD) [2]
- intrauterine hormone-releasing system (IUS)[2]
- bilateral tubal occlusion[2]
- vasectomised partner[2,3]
- true sexual abstinence[4]

Footnotes

1. An interaction between dapagliflozin and semaglutide with contraception is not anticipated.
2. Contraception methods that in the context of this guidance are considered to have low user dependency.
3. Vasectomised partner is a highly effective birth control method provided that partner is the sole sexual partner of the WOCBP trial participant and that the vasectomised partner has received medical assessment of the surgical success.
4. In the context of this guidance true sexual abstinence refers to: When this is in line with the preferred and usual lifestyle of the patient. Periodic abstinence (e.g. calendar, ovulation, symptothermal, post-ovulation methods), declaration of abstinence for the duration of a trial, and withdrawal are not acceptable methods of contraception.
The reliability of sexual abstinence needs to be evaluated in relation to the duration of the clinical trial and the preferred and usual lifestyle of the subject.

*<u>Child bearing potential</u> = experienced menarche; AND not undergone successful surgical sterilisation (hysterectomy, bilateral tubal ligation, or bilateral oophorectomy); AND not post-menopausal i.e. amenorrhea for ≥12 consecutive months (without another medical cause)

Source: Recommendations related to contraception and pregnancy testing in clinical trials. Clinical Trial Facilitation Group (CTFG), 15th September 2014.
See: https://www.hma.eu/fileadmin/dateien/Human_Medicines/01-About_HMA/Working_Groups/CTFG/2014_09_HMA_CTFG_Contraception.pdf (Accessed 21st January 2020)

16.3 Appendix 3

STICH Protocol *(also provided in Period 2 Participant Diary)*

As explained above, participants in this trial randomised to dapagliflozin are at an increased risk of ketosis and ketoacidosis. All participants will have access to a ketone meter as standard of care. It will be ascertained that each participant has access to a ketone meter; if there are any issues with their current ketone meter it will be replaced as per standard of care. There are mechanisms designed to mitigate the risk of early ketosis developing further into diabetic ketoacidosis. In this trial, we will be using a protocol called STICH (34). This is a mnemonic designed to act as a reminder for participants as to what to do in the event of elevated ketones following exposure to an SGLT-2 drug such as dapagliflozin:

1. STop dapagliflozin or placebo temporarily
2. Insulin – take a bolus of rapid acting insulin
3. Carbohydrate – take approximately 30g of carbohydrate
4. Hydrate – make sure you are taking sips of water aiming to maintain hydration.

Further guidance will be provided in both the Patient Diaries supplied to all participants and in the Patient Alert Cards that participants will be asked to carry at all times. All participants will also be advised that in the event of acute intercurrent illness or hospitalisation, dapagliflozin or placebo should be temporarily stopped. This is because intercurrent illness may additionally increase the risk of ketosis. Participants will likely already be familiar with this concept of "sick-day rules" as this is a key component of basic education for people with type 1 diabetes in clinical practice. These rules will however be re-iterated to participants.

More detailed guidance on actions participants should take for any given level of ketosis will be provided during face-to-face and telephone contact during the trial. In addition, however, the following written protocol will be provided to participants to advise them explicitly of the safest and most appropriate course of action to be taken in any given circumstance. This will take the form of a traffic light system to aid understanding and compliance.

1. Ketones 0.6 mmol/L or less: Continue - no need for additional bolus insulin.
2. Ketones 0.6 – 1.4 mmol/L: consider taking additional fast acting insulin. Follow sick day rules and monitor ketones 2 hourly until they are 0.6 mmol/L or less.
3. Ketones 1.5 – 2.9 mmol/L – Take 10% of your total daily dose of insulin (this is found by adding up all the long acting and short acting insulin for the previous day - only take a maximum of 10 units) as a bolus of fast acting insulin. Keep hydrated - aim for 100ml water per hour. Re-test your glucose and ketones in 2 hours. If not falling contact the study team during office hours. If you need urgent advice at other times please contact NHS 24 (telephone number 111) or attend your nearest Accident & Emergency Department.
4. Ketones above 3 mmol/L – Take 20% of your total daily insulin as fast acting insulin (Maximum 10 units). Hydrate with water, aim for 500 ml per hour. Attend A&E – High risk of DKA

If you develop DKA or are unwell it is important that you let the TTT1 nurse or doctor know about this as soon as you can. By following this protocol, the risk of ketosis and developing ketoacidosis will be mitigated.

16.4 Appendix 4: Amendment History

Amendment No.	Protocol version no.	Date issued	Author(s) of changes	Details of changes made
AM02	2.0			Protocol Changes: Weight loss mitigation, COVID19 Risk Mitigation and visit activity corrections

List details of all protocol amendments here whenever a new version of the protocol is produced. Protocol amendments must be submitted to the Sponsor for approval prior to submission to the REC committee or MHRA.

16.5 Appendix 5: PAID Questionnaire

Problem Areas In Diabetes (PAID) scale

Instructions: Which of the following diabetes issues are **currently** a problem for you? Tick the box that gives the best answer for you. Please provide an answer for each question.

		Not a problem	Minor problem	Moderate problem	Somewhat serious problem	Serious problem
1	Not having clear and concrete goals for your diabetes care?	☐ 0	☐ 1	☐ 2	☐ 3	☐ 4
2	Feeling discouraged with your diabetes treatment plan?	☐ 0	☐ 1	☐ 2	☐ 3	☐ 4
3	Feeling scared when you think about living with diabetes?	☐ 0	☐ 1	☐ 2	☐ 3	☐ 4
4	Uncomfortable social situations related to your diabetes care (e.g. people telling you what to eat)?	☐ 0	☐ 1	☐ 2	☐ 3	☐ 4
5	Feelings of deprivation regarding food and meals?	☐ 0	☐ 1	☐ 2	☐ 3	☐ 4
6	Feeling depressed when you think about living with diabetes?	☐ 0	☐ 1	☐ 2	☐ 3	☐ 4
7	Not knowing if your mood or feelings are related to your diabetes?	☐ 0	☐ 1	☐ 2	☐ 3	☐ 4
8	Feeling overwhelmed by your diabetes?	☐ 0	☐ 1	☐ 2	☐ 3	☐ 4
9	Worrying about low blood glucose reactions?	☐ 0	☐ 1	☐ 2	☐ 3	☐ 4
10	Feeling angry when you think about living with diabetes?	☐ 0	☐ 1	☐ 2	☐ 3	☐ 4
11	Feeling constantly concerned about food and eating?	☐ 0	☐ 1	☐ 2	☐ 3	☐ 4
12	Worrying about the future and the possibility of serious complications?	☐ 0	☐ 1	☐ 2	☐ 3	☐ 4
13	Feelings of guilt or anxiety when you get off track with your diabetes management?	☐ 0	☐ 1	☐ 2	☐ 3	☐ 4
14	Not 'accepting' your diabetes?	☐ 0	☐ 1	☐ 2	☐ 3	☐ 4
15	Feeling unsatisfied with your diabetes physician?	☐ 0	☐ 1	☐ 2	☐ 3	☐ 4
16	Feeling that diabetes is taking up too much of your mental and physical energy every day?	☐ 0	☐ 1	☐ 2	☐ 3	☐ 4
17	Feeling alone with your diabetes?	☐ 0	☐ 1	☐ 2	☐ 3	☐ 4
18	Feeling that your friends and family are not supportive of your diabetes management efforts?	☐ 0	☐ 1	☐ 2	☐ 3	☐ 4
19	Coping with complications of diabetes?	☐ 0	☐ 1	☐ 2	☐ 3	☐ 4
20	Feeling 'burned out' by the constant effort needed to manage diabetes?	☐ 0	☐ 1	☐ 2	☐ 3	☐ 4

© Joslin Diabetes Center, 1999 (www.joslin.org). All rights reserved. The copyright holder/developer has given permission for the questionnaire to be reproduced in this toolkit. Readers of the toolkit are permitted to reproduce the questionnaire for clinical use and non-commercial research purposes. Readers of the toolkit are not permitted to use the questionnaire for commercial research purposes and must seek permission from the copyright holder/developer to do so.

16.6 Appendix 6: DSQOLS Questionnaire

Last Name: ____________________ **First Name:** ______________ **MI:** ____

Date: (month. day. year): I___I___I . I___I___I . I___I___I___I___I

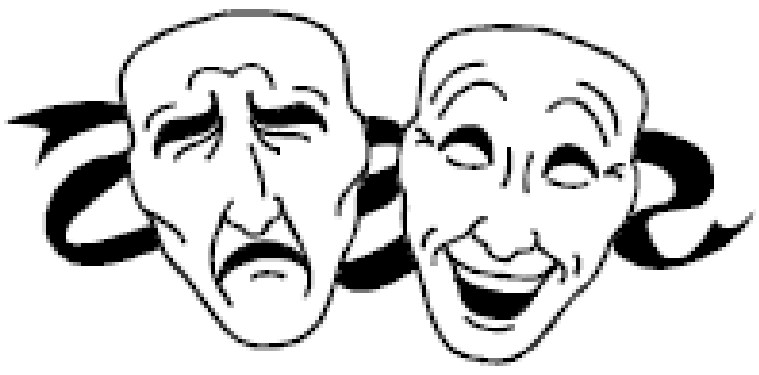

Questionnaire regarding

- **Treatment goals**
- **Treatment satisfaction and**
- **Daily Burdens**

of Patients with Type 1 Diabetes

Diabetes is associated with different restrictions and burdens for most people.

This questionnaire asks what your treatment goals are, how much diabetes represents a burden in your day to day living, and how satisfied you are with your current treatment.

Please rate each statement on how applicable it is to your personal situation.
Fill in ONLY one square per question.
Please answer the questions in the given order without skipping any.

For diabetes treatment it is especially important to me, or it would be my goal that ...	extremely impor-tant	pretty impor-tant	rather impor-tant	rather unim-portant	pretty unim-portant	totally unim-portant
1. my blood sugar values are always below 7 mmol/l (126 mg%).	❑	❑	❑	❑	❑	❑
2. my blood sugar values are as stable as possible.	❑	❑	❑	❑	❑	❑
3. I can arrange my free time with flexibility.	❑	❑	❑	❑	❑	❑
4. I avoid even mild episodes of low blood sugar.	❑	❑	❑	❑	❑	❑
5. I can definitely prevent the long-term complications of diabetes, or slow down their progression.	❑	❑	❑	❑	❑	❑
6. I can eat flexibly by choosing what food I want, how much I want, and when I want my meals.	❑	❑	❑	❑	❑	❑
7. I am as physically fit as possible.	❑	❑	❑	❑	❑	❑
8. I can definitely avoid severe episodes of low blood sugar involving loss of consciousness.	❑	❑	❑	❑	❑	❑
9. I have to measure my blood glucose as rarely as possible.	❑	❑	❑	❑	❑	❑
10. other people don't find out that I have diabetes.	❑	❑	❑	❑	❑	❑

How satisfied have you been over the last 4 weeks with ...	extremely satis-fied	pretty satis-fied	rather satis-fied	rather dissatis-fied	pretty dissatis-fied	totally dissatis-fied
1. your blood sugar levels?	❑	❑	❑	❑	❑	❑
2. the stability of your blood sugar values?	❑	❑	❑	❑	❑	❑
3. the flexibility in arranging your free time?	❑	❑	❑	❑	❑	❑
4. the frequency of mild episodes of low blood sugar?	❑	❑	❑	❑	❑	❑
5. your protection from the long-term complications, or your ability to slow down their progression?	❑	❑	❑	❑	❑	❑
6. the flexibility of your diet?	❑	❑	❑	❑	❑	❑
7. your physical strength?	❑	❑	❑	❑	❑	❑
8. your protection from severe episodes of low blood sugar involving loss of consciousness?	❑	❑	❑	❑	❑	❑
9. the frequency of your blood sugar self-monitoring?	❑	❑	❑	❑	❑	❑
10. the extent to which other people are informed about your diabetes?	❑	❑	❑	❑	❑	❑

How much have you felt burdened and restricted by diabetes and its treatment over the last 4 weeks?

This statement meets my point of view ...

	per-fectly	quite good	some-what	not much	hardly	not at all
1. It bothers me that I have to measure my blood sugar so often	❑	❑	❑	❑	❑	❑
2. It is a burden for me that I need to constantly think about my food plan	❑	❑	❑	❑	❑	❑
3. I suffer from pain because of diabetes	❑	❑	❑	❑	❑	❑
4. Because of diabetes my relationship with my partner has become worse	❑	❑	❑	❑	❑	❑
5. I am worried that my life could be shorter because of diabetes	❑	❑	❑	❑	❑	❑
6. I feel as if I am less attractive to others because of diabetes.	❑	❑	❑	❑	❑	❑
7. Because of diabetes I feel sad or depressed	❑	❑	❑	❑	❑	❑
8. I am worried about my future health	❑	❑	❑	❑	❑	❑
9. It is a burden for me how other people react to my diabetes	❑	❑	❑	❑	❑	❑
10. I feel nervous and restless when I think about episodes of low blood sugar	❑	❑	❑	❑	❑	❑
11. I feel tired and exhausted	❑	❑	❑	❑	❑	❑
12. It bothers me that I have to spend so much time on my diabetes treatment	❑	❑	❑	❑	❑	❑
13. Because of diabetes traveling is complicated and troublesome	❑	❑	❑	❑	❑	❑
14. I get anxious and nervous when I think about the dangers of episodes of low blood sugar	❑	❑	❑	❑	❑	❑
15. Diabetes prevents me from spontaneous physical activities	❑	❑	❑	❑	❑	❑
16. I suffer from frequent urination	❑	❑	❑	❑	❑	❑
17. Because of high blood sugar values after a meal I often feel unwell or less efficient	❑	❑	❑	❑	❑	❑
18. Because of diabetes I feel anxious and threatened	❑	❑	❑	❑	❑	❑
19. I suffer from thirst or having a dry mouth	❑	❑	❑	❑	❑	❑
20. Other people find it hard to understand my problems with diabetes treatment	❑	❑	❑	❑	❑	❑
21. I feel physically unwell	❑	❑	❑	❑	❑	❑
22. I unnecessarily worry too much about episodes of low blood sugar	❑	❑	❑	❑	❑	❑
23. I have to give up good-tasting foods	❑	❑	❑	❑	❑	❑

	This statement meets my point of view ...					
	per-fectly	quite good	some-what	not much	hardly	not at all
24. I cannot eat as much as I want of certain foods........................	❑	❑	❑	❑	❑	❑
25. Because of diabetes I often have physical complaints.............	❑	❑	❑	❑	❑	❑
26. Diabetes interferes with my future plans...................................	❑	❑	❑	❑	❑	❑
27. I get an uncomfortable feeling when I think about the dangers of a severe episode of low blood sugar.....................................	❑	❑	❑	❑	❑	❑
28. Because of diabetes I have less contact with friends or acquaintances...	❑	❑	❑	❑	❑	❑
29. I wish I could eat more the way I want to, without having to plan everything beforehand..	❑	❑	❑	❑	❑	❑
30. I suffer from frequent infections, itching or skin problems........	❑	❑	❑	❑	❑	❑
31. I am concerned about getting into embarrassing situations because of episodes of low blood sugar..................................	❑	❑	❑	❑	❑	❑
32. I feel weak or lazy..	❑	❑	❑	❑	❑	❑
33. I am dissatisfied with the amount of time I have to spend for medical consultations..	❑	❑	❑	❑	❑	❑
34. Because of diabetes I cannot spend my free time the way I would like...	❑	❑	❑	❑	❑	❑
35. I feel like a disabled person..	❑	❑	❑	❑	❑	❑
36. I am worried that I could sustain physical injury in the event of a severe episode of low blood sugar	❑	❑	❑	❑	❑	❑
37. It bothers me that I cannot eat like other people......................	❑	❑	❑	❑	❑	❑
38. I am often worried about the long-term complications of diabetes..	❑	❑	❑	❑	❑	❑
39. Because of diabetes other people treat me like a "sick person"	❑	❑	❑	❑	❑	❑
40. Because of diabetes I cannot pursue my hobbies as I wish.....	❑	❑	❑	❑	❑	❑
41. I have to frequently think about diabetes and its consequences...	❑	❑	❑	❑	❑	❑
42. It bothers me that I cannot eat as spontaneously as people who do not have diabetes...	❑	❑	❑	❑	❑	❑
43. It upsets my stomach when I think about the dangers of severe episodes of low blood sugar..	❑	❑	❑	❑	❑	❑
44. Diabetes constantly causes problems while dealing with other people...	❑	❑	❑	❑	❑	❑
45. It bothers me that I have to take my diabetes supplies (for example syringes) with me whatever I do................................	❑	❑	❑	❑	❑	❑
46. I frequently worry that I may become helpless and may need constant care later on..	❑	❑	❑	❑	❑	❑

	This statement meets my point of view ...					
	per-fectly	quite good	some-what	not much	hardly	not at all
47. I often cannot eat as much as I would like................................	❑	❑	❑	❑	❑	❑
48. I am worried about having a severe episode of low blood sugar at night..	❑	❑	❑	❑	❑	❑
49. Because of diabetes my family-life is affected..........................	❑	❑	❑	❑	❑	❑
50. Because of diabetes my physical strength is restricted...........	❑	❑	❑	❑	❑	❑
51. Because of diabetes it is much harder to make friends...........	❑	❑	❑	❑	❑	❑
52. It bothers me that I have to frequently think about possible causes and mistakes after episodes of low blood sugar..........	❑	❑	❑	❑	❑	❑
53. It bothers me how much diabetes controls my daily life...........	❑	❑	❑	❑	❑	❑
54. It bothers me that I have to inject insulin before I know how much I would really like to eat or I really can eat.....................	❑	❑	❑	❑	❑	❑
55. I would like to eat a greater amount of certain foods which increase my blood sugar strongly...	❑	❑	❑	❑	❑	❑
56. I am worried that I could easily panic in the event of an episode of low blood sugar..	❑	❑	❑	❑	❑	❑
57. When I think about the dangers associated with severe episodes of low blood sugar I wonder how often I will remain unharmed..	❑	❑	❑	❑	❑	❑

FULL/LONG TITLE OF THE TRIAL
Triple Therapy for Type 1 Diabetes with Insulin, Semaglutide and Dapagliflozin (TTT1) - UK

SHORT STUDY TITLE / ACRONYM
TTT1 (UK)

PROTOCOL VERSION NUMBER AND DATE
Version 3.0 11th January 2022

RESEARCH REFERENCE NUMBERS

**IRAS Number:**	254431
**EudraCT Number:**	2018-004120-11
**ISRCTN Number / Clinical trials.gov Number:**	TBC
**SPONSOR Number:**	GN17DI706
**FUNDER Number:**	3-SRA-2019-669-M-B

SIGNATURE PAGE

The undersigned confirm that the following protocol has been agreed and accepted and that the Chief Investigator agrees to conduct the trial in compliance with the approved protocol and will adhere to the principles outlined in the Medicines for Human Use (Clinical Trials) Regulations 2004 (SI 2004/1031), amended regulations (SI 2006/1928) and any subsequent amendments of the clinical trial regulations, Good Clinical Practice (GCP) guidelines, the Sponsor's (Standard Operating Procedures (SOPs), and other regulatory requirements as amended.
I agree to ensure that the confidential information contained in this document will not be used for any other purpose other than the evaluation or conduct of the clinical investigation without the prior written consent of the Sponsor
I also confirm that I will make the findings of the study publicly available through publication or other dissemination tools without any unnecessary delay and that an honest accurate and transparent account of the study will be given; and that any discrepancies from the study as planned in this protocol will be explained.

For and on behalf of the Study Co-Sponsor (University of Glasgow):
Signature: .... Date: ....../....../......
Name (please print): ....
Position: ....

For and on behalf of the Study Co-Sponsor (NHS Greater Glasgow and Clyde):
Signature: .... Date: ....../....../......
Name (please print): ....
Position: ....

UK Chief Investigator:
Signature: .... Date: ....../....../......
Name: (please print): John Petrie ....
Position: Professor of Diabetic Medicine..

Statistician:
Signature: .... Date: ....../....../......
Name: (please print): ....

Position: ....

KEY TRIAL CONTACTS

UK Chief Investigator	Professor John R Petrie<br>Professor of Diabetic Medicine<br>Institute of Cardiovascular and Medical Sciences<br>BHF Glasgow Cardiovascular Research Centre<br>University of Glasgow<br>126 University Place<br>Glasgow G12 8TA<br>Secretary +44(0)141 330 3325<br>john.petrie@glasgow.ac.uk
UK Co- Investigator	Dr James Boyle<br>Consultant Diabetologist<br>Clinical Lead NHS GG&C Diabetes MCN<br>Diabetes UK Clinical Champion<br>Glasgow Royal Infirmary<br>84 Castle Street, Glasgow, G4 0SF<br>Tel: : +44(0)141 211 5996<br>james.boyle@glasgow.ac.uk
Trial Co-ordination	Miss Lisa Jolly<br>Research and Innovation<br>NHS Greater Glasgow & Clyde<br>Ward 11, Dykebar Hospital<br>Grahamston Road<br>Paisley, PA2 7DE<br>Lisa.Jolly@ggc.scot.nhs.uk
Co-Sponsor	NHS Greater Glasgow & Clyde<br><br>Contact: Dr Maureen Travers<br>Research and Development Management Office<br>Research and Innovation<br>NHS Greater Glasgow & Clyde<br>Ward 11, Dykebar Hospital<br>Grahamston Road<br>Paisley, PA2 7DE<br>Tel: +44 (0) 0141 314 4012<br>maureen.travers@ggc.scot.nhs.uk
Co-sponsor	University of Glasgow<br><br>Contact: Dr Debra Stuart<br>College of Medical, Veterinary & Life Sciences<br>Room 327 Wolfson Medical School Building<br>University Avenue<br>Glasgow G12 8QQ<br>Tel: +44 (0)141 330 4539<br>Debra.Stuart@glasgow.ac.uk

Funder	JDRF<br>26 Broadway, 15th floor, New York, NY 10004, USA
Clinical Trials Unit	Glasgow Clinical Trials Unit<br>Robertson Centre for Biostatistics, 11th Floor,<br>Boyd Orr Building, University of Glasgow<br>G12 8QQ<br>Tel: 0141 330 4744<br>liz.anderson@glasgow.ac.uk
Statistician	Professor Alex McConnachie<br>Robertson Centre for Biostatistics, 11th Floor, Boyd Orr Building, University of Glasgow, Glasgow, G12 8QQ<br>Telephone: 0141 330 4744<br>Fax: 0141-330-5094
Trials pharmacist	Dr Elizabeth Douglas<br>Clinical Trials Pharmacist<br>Clinical Research and Development<br>Ward 11, Dykebar Hospital,<br>Grahamston Road | Paisley PA2 7DE<br>Tel: 0141 314 4073<br>E-mail: elizabeth.douglas@ggc.scot.nhs.uk

US Principal Investigator	Dr Paresh Dandona<br>Director, Diabetes-Endocrinology Center of Western NY<br>Chief of Endocrinology, Diabetes, Endocrinology and Metabolism, SUNY at Buffalo<br>1000 Youngs Road, Suite 105<br>Williamsville NY 14221<br>Telephone: +1(716) 535-1850<br>Fax: +1(716)-639-1200<br>pdandona@kaleidahealth.org
US Co- Investigator	Dr Husam Ghanim, PhD.<br>Director, Diabetes and Endocrinology Research Lab<br>Research Associate Professor,<br>School of Medicine, State University of NY at Buffalo<br>Clinical and Translational Research Center<br>875 Ellicott St<br>Buffalo, NY 14203<br>Tel: (+1)716-881-8924<br>ghanim@buffalo.edu

Key Protocol Contributors	Professor John Petrie<br>john.petrie@glasgow.acuk<br><br>Dr Paresh Dandona<br>pdandona@kaleidahealth.org<br><br>Husam Ghanim, PhD.<br>ghanim@buffalo.edu<br><br>Dr James Boyle<br>james.boyle@glasgow.ac.uk<br><br>Dr Robert Lindsay<br>robert.lindsay@glasgow.ac.uk<br><br>Dr Joseph Timmons<br>joseph.timmons@glasgow.ac.uk<br><br>Professor Ian Ford<br>ian.ford@glasgow.ac.uk<br><br>Professor Alex McConnachie<br>Alex.McConnachie@glasgow.ac.uk

TRIAL SUMMARY

Trial Title	Triple Therapy for Type 1 Diabetes with Insulin, Semaglutide, and Dapagliflozin (UK)	
Internal ref. no. (or short title)	TTT1	
Clinical Phase	Phase 3	
Trial Design	Two six-month periods: (i) open-label randomised (2:1) parallel group semaglutide vs standard of care; (ii) additional double-blind, randomised (1:1) parallel group, dapagliflozin vs placebo for those participants initially randomised to semaglutide.<br>Regardless of each randomisation, ongoing standard of care (insulin therapy) will be provided for all participants throughout the study.	
Trial Participants	Adults with type 1 diabetes for at least one year with HbA1c 7.5-11.0% on stable multiple daily injections (MDI) (four or more) of insulin or continuous subcutaneous insulin infusion (CSII) for the previous three months.	
Planned Sample Size	114 (57 in UK)	
Treatment duration	Approximately 1 year	
Follow up duration	Approximately 1 year	
Planned Trial Period	Approximately 3 years	
	Objectives	Outcome Measures
Primary	To assess in people with type 1 diabetes whether triple therapy (dapagliflozin, semaglutide and insulin) reduces HbA1c in comparison with dual therapy (placebo, semaglutide, and insulin – period 2).	HbA1c
Secondary	To assess in people with type 1 diabetes whether triple therapy (dapagliflozin, semaglutide and insulin) reduces HbA1c in comparison with standard therapy (insulin alone)	HbA1c
	To assess in people with type 1 diabetes whether dual therapy (semaglutide and insulin - period 1) reduces HbA1c in comparison with standard therapy (insulin alone)	HbA1c
	To assess in people with type 1 diabetes whether triple therapy (dapagliflozin, semaglutide and insulin) improves glucose control	- Mean fasting blood glucose<br><br>- Mean blood glucose*<br>- Percentage time in range

	as assessed by CGM, insulin requirement, body weight, blood pressure and quality of life in comparison with (a) dual therapy (placebo, semaglutide, and insulin – period 2) and (b) standard therapy (insulin alone)	- Glycaemic variability*<br>- Percentage time in hyperglycaemia Level 1 (10.1-13.9 mmol/)*<br>- Percentage time in hyperglycaemia Level 2 (>13.9 mmol/L)*<br>-Percentage time in hypoglycaemia Level 1 (3.0 – 3.8 mmol/L)*<br>** CGM (by masked Dexcom G6)*<br><br>Insulin requirement<br>Body weight<br>Systolic/ diastolic blood pressure<br>Number of antihypertensive agents<br>Diabetes Specific Quality of Life Scale (DSQOLS)<br>Problem Areas In Diabetes (PAID) questionnaire
Safety Endpoint 1	a) Triple therapy (dapagliflozin, semaglutide and insulin) in comparison with dual therapy (placebo, semaglutide, insulin – period 2)<br><br>b) Dual therapy (semaglutide and insulin - period 1) in comparison with standard therapy (insulin only)	Difference between treatment arms in:<br>(i) Rate of Level 2 (<3.0 mmol/L) hypoglycaemic events (by Dexcom G6 CGM)<br>(ii) Rate of Level 3 hypoglycaemic events (severe event with altered mental and/or physical status requiring assistance)
Safety Endpoint 2	a) Triple therapy (dapagliflozin, semaglutide and insulin) in comparison with dual therapy (placebo, semaglutide, and insulin – period 2)<br><br>b) Dual therapy (semaglutide and insulin –period 1) in comparison with standard therapy (insulin only)	Difference between treatment arms in:<br>Rate of diabetic ketoacidosis (serum ketones or urinary ketones above normal limits AND serum bicarbonate < 15 mmol/L OR blood pH < 7.3)
Investigational Medicinal Product(s)	Semaglutide<br>Dapagliflozin<br>Placebo matching dapagliflozin	
Non-investigational Medicinal Product(s)	Insulin	
Formulation, Dose, Route of Administration	Semaglutide (subcutaneous injection, once weekly) – open label by pen device (0.25 mg/ 0.5 mg/ 1.0 mg)<br>Dapagliflozin 5 mg (oral) – one then two capsules daily (uptitration)<br>Placebo matching dapagliflozin	

FUNDING AND SUPPORT IN KIND

FUNDER(S)	FINANCIAL AND NON FINANCIALSUPPORT GIVEN
JDRF New York<br>26 Broadway, 15th floor, New York, NY 10004, United States	$1,440,000 (to University at Buffalo, the State University of New York, NY (SUNY Buffalo), USA; and University of Glasgow, UK)
Novo Nordisk, 800 Scudders Mill Road, Princeton Landing, NJ, Plainsboro, 08536.	$160,000 (as above)
AstraZeneca<br>1800 Concord Pike, Wilmington, DE 19803, USA	Provision of dapagliflozin and matched placebo investigational medicinal products to US site
Dexcom; Suite 6, 1 Tanfield, Edinburgh, EH3 5DA	Staff training and discounted supply of CGM sensors

ROLE OF STUDY SPONSOR AND FUNDER

NHS Greater Glasgow & Clyde and The University of Glasgow will be Co-sponsors of the UK arm of the trial. Prior to study initiation, a non-commercially funded clinical trial co-sponsorship agreement will be put in place between NHS Greater Glasgow & Clyde and The University of Glasgow. The roles and liabilities each organisation will take under The Medicines for Human Use (Clinical Trials) Regulations, 2004 SI 2001:1031 are laid out in this agreement signed by both organisations. The University of Glasgow shall be responsible for carrying out the obligations and responsibilities set out in the aforementioned agreement, and shall be deemed "sponsor" for the purposes of, Part 3 of the regulations in relation to the study. NHS Greater Glasgow & Clyde shall be responsible for carrying out the responsibilities set out in the agreement, and shall be deemed "sponsor" for the purposes of, Parts 4, 5, 6 and 7 of the Regulations in relation to the study.

The Co-Sponsors will delegate specific roles to the Chief Investigator, Glasgow CTU and other third parties. These arrangements will be clearly documented in agreements and/or the Sponsor Delegated Roles and Responsibilities Matrix.

The US arm of the trial has the same inclusion and exclusion criteria and the same pre-specified outcomes but an individualised Protocol. It will collect the same data via a common eCRF. Adverse event data from each site will be available to the trial Data and Safety Monitoring Board. The UK Co-Sponsors will have no responsibility for (and carry no liability for) the conduct of the US arm of the trial.

<u>JDRF</u>
The study has been funded by a grant from JDRF. JDRF does not have a designated role or responsibility in trial design, conduct, data analysis and interpretation, manuscript writing, and dissemination of results. Periodic reports (at least annual) in relation to progress of the trial will be submitted to the JDRF. Support from JDRF will be acknowledged in any publications related to the study.

<u>Astra Zeneca</u>
This is an investigator-initiated study. Astra Zeneca provided support by supplying investigational medicinal products (IMPs) dapagliflozin and matched placebo to the US site only. Representatives of Astra Zeneca reviewed and provided comments on drafts of the Protocol but the company does not

have a designated role or responsibility in trial design, conduct, data analysis and interpretation, manuscript writing, and dissemination of results.

In November 2020, Novo Nordisk made up a shortfall of funding that resulted from a cut back by 10% of the main grant by the funder JDRF due to the COVID-19 pandemic. By this time, the Protocol had been finalised. The company does not have a designated role or responsibility in trial design, conduct, data analysis and interpretation, manuscript writing, or dissemination of results.

ROLES AND RESPONSIBILITIES OF TRIAL MANAGEMENT COMMITTEES/GROUPS & INDIVIDUALS

Trial Steering Committee (TSC)

The role of the TSC is to provide overall supervision of the trial and ensure that it is being conducted in accordance with the principles of GCP and the relevant regulations. The TSC will:

1. agree the trial protocol and any protocol amendments
2. ensure that conduct of the trial is fully harmonised between the two sites
3. provide advice to the investigators on all aspects of the trial

It will have an independent Chairperson and include the US and UK investigators and key protocol contributors listed above.

Decisions about continuation or termination of the trial or substantial amendments to the protocol will be the responsibility of the TSC who will advise the co-sponsors. The TSC will meet at the start of the study, and four to six monthly thereafter. The TSC will have its own Charter outlining the role and responsibilities of its members. The TSC may invite other attendees from the trial team to present or participate in discussions on particular topics. These attendees will be non-voting members.

Independent data monitoring committee (IDMC)

An IDMC will be established to include two independent medical experts (covering the domains of type 1 diabetes and metabolic disease; one of the academic clinicians will act as chair) and an independent biostatistician. The Glasgow CTU will liaise with the committee and ensure that the Committee is provided with adequate information about study progress and results.

The IDMC will have a formal Charter outlining the responsibilities of the IDMC members, Glasgow CTU and the co-sponsors. Responsibilities include:

- To protect the safety of participants recruited to the trial.
- Advising the TSC and co-sponsors if it is safe and appropriate to continue with the study
- Examining information provided by the Glasgow CTU on study recruitment, adverse events and outcomes
- Providing reports for the Project Office to forward to the TSC, ethics committees, regulatory bodies, study co-sponsors, and the BHF.

The IDMC may recommend to the TSC to terminate the study or specific aspects of the study if clear harm is observed with one treatment option compared to standard of care or placebo. Specific safety endpoints that will be reviewed include rates of hypoglycaemia and ketosis/ ketoacidosis. The IDMC will not formally review efficacy or futility.

The IDMC will meet approximately every six months.

Study Management Group (SMG)

Trial activity at the UK/ Glasgow site will be coordinated from NHS Greater Glasgow & Clyde (NHS GG&C) by the TTT1 Study Management Group (SMG). The SMG will consist of the UK chief investigator, co-principal investigator other co-applicants, project manager and representatives from the Glasgow CTU, NHS GG&C and The University of Glasgow. The role of the group is to monitor all

aspects of the conduct and progress of the trial in the UK, ensuring adherence to the protocol, and take appropriate action to safeguard participants and the quality of the trial itself.

Joint Study Management Group

Coordination of trial activity between the UK/ Glasgow and the US/ Buffalo site will be facilitated by the Joint TTT1 Study Management Group. This will consist of the US and UK chief investigators, co-principal investigators, other co-applicants, study coordinators, project managers and governance/ administrative representatives as appropriate from State University of New York (SUNY), the Glasgow CTU, NHS GG&C and The University of Glasgow. It will meet approximately every 4-6 weeks. The role of the group is to coordinate all aspects of the conduct and progress of the trial between the US and UK sites, ensuring protocol adherence, and taking appropriate action to safeguard participants and the quality of the trial itself.

Protocol contributors

The protocol has been developed by a group with extensive clinical and research experience in type 1 diabetes and clinical trials including Professor John Petrie and Professor Paresh Dandona. Professor Ian Ford and Professor Alex McConnachie have research expertise in design, conduct, analysis and interpretation of clinical trials and biostatistical methods.

KEY WORDS:

Adjunct therapy
Dapagliflozin
Glycated haemoglobin (HbA1c)
Semaglutide
Type 1 diabetes

LIST of CONTENTS

RESEARCH REFERENCE NUMBERS 2
SIGNATURE PAGE 3
KEY TRIAL CONTACTS 4
LIST of CONTENTS....................14
LIST OF ABBREVIATIONS....................15
TRIAL FLOW CHART....................17
1 BACKGROUND 20
2 RATIONALE....................21
3 OBJECTIVES AND OUTCOME MEASURES/ ENDPOINTS....................25
4 TRIAL DESIGN 28
5 STUDY SETTING 28
6 ELIGIBILITY CRITERIA 28
7 TRIAL PROCEDURES....................30
8 TRIAL MEDICATION....................58
9 PHARMACOVIGILANCE....................63
10 STATISTICS AND DATA ANALYSIS....................69
11 DATA HANDLING 71
12 MONITORING, AUDIT & INSPECTION 72
13 ETHICAL AND REGULATORY CONSIDERATIONS....................72
14 DISSEMINATION POLICY....................77
15 REFERENCES....................78
16. APPENDICES....................80

LIST OF ABBREVIATIONS

ACS	Acute Coronary Syndrome
AUC	Area Under the Curve
AE	Adverse Event
AR	Adverse Reaction
CA	Competent Authority
CABG	Coronary Artery Bypass Graft
CGM	Continuous Glucose Monitoring
CHI	Community Health Index
CI	Chief Investigator
CRO	Contract Research Organisation
CRP	C-Reactive Protein
CSII	Continuous Subcutaneous Insulin Infusion
CTA	Clinical Trial Authorisation
CTU	Clinical Trials Unit
CTIMP	Clinical Trial of Investigational Medicinal Product
CV	Cardiovascular
CVA	Cerebrovascular Accident
DKA	Diabetic Ketoacidosis
DSUR	Development Safety Update Report
EC	European Commission
ECG	Electrocardiogram
eCRF	Electronic Case Report Form
EDTA	Ethylenediaminetetraacetic acid
eGFR	Estimated Glomerular Filtration Rate
EMEA	European Medicines Agency
EU	European Union
EUCTD	European Clinical Trials Directive
EudraCT	European Clinical Trials Database
EudraVIGILANCE	European database for Pharmacovigilance
FBC	Full Blood Count
FGM	Flash Glucose Monitoring
GCP	Good Clinical Practice
GDG	Guideline Development Group
GDPR	General Data Protection Regulation
GLP-1	Glucagon Like Peptide-1
GLP-1RA	Glucagon Like Peptide-1 Receptor Agonist
GMP	Good Manufacturing Practice
HbA1c	Glycated haemoglobin
IB	Investigator Brochure
ICF	Informed Consent Form
ICH	International Conference on Harmonisation of technical requirements for registration of pharmaceuticals for human use.
IDMC	Independent Data Monitoring Committee
IMP	Investigational Medicinal Product
IMPD	Investigational Medicinal Product Dossier

ISF	Investigator Site File
ISRCTN	International Standard Randomised Controlled Trials Number
IWRS	Interactive Web-based Randomisation System
LPLV	Last Patient Last Visit
MA	Marketing Authorisation
MDRD	Modification of Diet in Renal Disease
MODY	Maturity Onset Diabetes of the Young
MHRA	Medicines and Healthcare products Regulatory Agency
MI	Myocardial Infarction
MDI	Multiple Daily Injections
NHS GG&C	National Health Service Greater Glasgow & Clyde
NHS R&D	National Health Service Research & Development
NICE	The National Institute for Health and Care Excellence
NIMP	Non-Investigational Medicinal Product
PI	Principal Investigator
PIC	Participant Identification Centre
PIS	Participant Information Sheet
PV	Pharmacovigilance
QA	Quality Assurance
QC	Quality Control
QoL	Quality of Life
QP	Qualified Person
RCT	Randomised Control Trial
REC	Research Ethics Committee
SAE	Serious Adverse Event
SAP	Statistical Analysis Plan
SAR	Serious Adverse Reaction
SDV	Source Data Verification
SGLT2	Sodium Glucose Transporter 2
SOP	Standard Operating Procedure
SUNY	State University of New York
SmPC	Summary of Product Characteristics
SSI	Site Specific Information
SUSAR	Suspected Unexpected Serious Adverse Reaction
TIA	Transient Ischaemic Attack
TMG	Trial Management Group
TSC	Trial Steering Committee
TMF	Trial Master File
UB	University of Buffalo
ULN	Upper Limit of Normal reference range
WOCBP	Women Of Childbearing Potential

TRIAL FLOW CHART

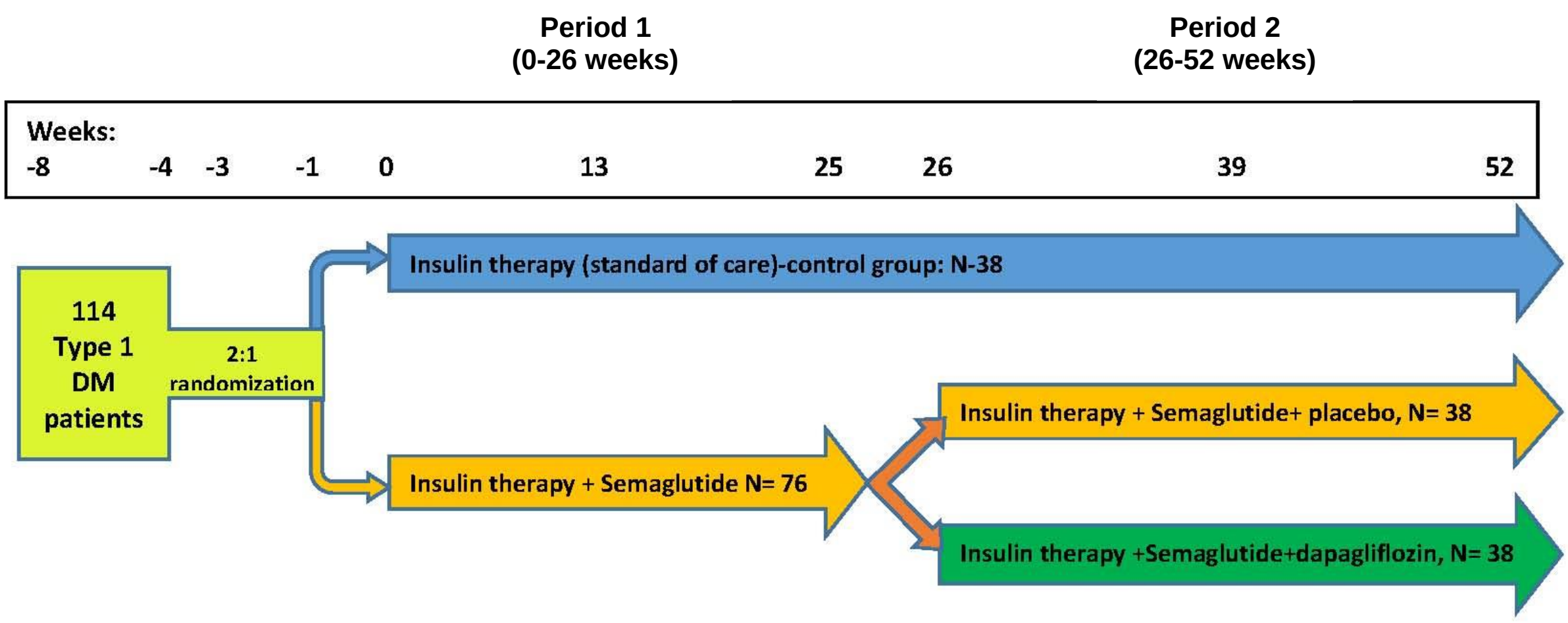

SCHEDULE OF ASSESSMENTS

**Visit #**	**-**	**1**	**2**	**3**	**4[3]**	**5[3]**	**6**	**7**	**8**	**9**	**10**	**11[3]**	**12**	**13**	**14**	**15**	**16**
**Week# (±7 days; see below)**	**-8 to -4**	**-3**	**-1**	**0**	**1**	**2**	**4**	**13**	**23**	**25**	**26**	**27**	**31**	**39**	**49**	**51**	**52**
Study Procedures	Screening	Run-in period		Randomise							Randomise						End
Informed Consent	X																
Fasting visit			X	X				X		X	X			X		X	X
History and demographics	X																
Physical	X																X
Education on hypoglycaemia and ketosis avoidance)				X							X						
BP, heart rate, weight	X	X	X	X	X	X	X	X	X	X	X	X	X	X	X	X	X
Bloods (FBC, kidney and liver profiles)	X			X				X			X		X	X			X
C-peptide	X										X						X
Pregnancy test	X			X			X[1]	X[1]	X[1]		X[1]		X[1]	X[1]	X[1]	X[1]	X[1]
Dispense/ titrate open-label semaglutide[2]				X	X[2]	X[2]	X[2]	X[2]			X[2]			X[2]			
Dispense dapagliflozin/placebo											X	X		X			
Dapagliflozin/placebo return capsule counts												X		X			X
HbA1c	X			X				X		X				X			X
Blood endpoints (including fructosamine)				X				X			X			X			X
Masked CGM fit (enter data this visit)		X							X						X		
Masked CGM read				X							X						X
Daily fingerstick glucose review		X	X	X	X	X	X	X	X	X	X	X	X	X	X	X	X
Daily urinary ketones review												X	X	X	X	X	X
Insulin dose review		X	X	X	X	X	X	X	X	X	X	X	X	X	X	X	X
Ketone bodies (blood/urine)	X	X	X	X	X	X	X	X	X	X	X	X	X	X	X	X	X
Adverse Event reporting		X	X	X	X	X	X	X	X	X	X	X	X	X	X	X	X
Meal Challenge Test			X							X						X	
24 hr Urine dispensed			X				X			X			X			X	
24 hr Urine collected				X				X			X			X			X
Quality of life questionnaires				X							X						X

N.B. Safety phone calls will be conducted at weeks 3, 5, 8, 16, 20, 29, 35, 44 and at two weeks after the end of the study to document any side effects or other changes. **Abbreviations:** see pages 14-15

Visit dates should be within seven days of the visit time; however, a longer or shorter interval does not constitute a protocol deviation

[1]Not required in women randomised to "Insulin therapy (standard of care)

[2]According to randomisation schedule: resupply of open-label semaglutide after Visit 3 will be according to individual requirements as tolerated (see Section 8.3)

[3] Visits 4, 5 and 11 have the option of being carried out by telephone for those participants allocated to the standard care arm only.

STUDY PROTOCOL

Triple Therapy for Type 1 Diabetes with Insulin, Semaglutide, and Dapagliflozin

1 BACKGROUND

The control of glucose homeostasis in type 1 diabetes is fragile since β-cell reserve is negligible. Traditionally, insulin has been the only treatment approach available. This often leads to erratic blood glucose control given the challenge of matching exogenous insulin to mealtime glucose excursions in the face of a lack of significant endogenous insulin to compensate for variation in requirements in response to meals and activity levels. Injected insulin boluses rarely match precise insulin requirements either in terms of dose administered or bio-availability. The result often is wide fluctuations in blood glucose including episodes when it is too low (hypoglycaemia). Based on our work to date and other published reports, previous attempts to add other glucose-lowering medications to insulin therapy yielded minimal benefits on glycaemia (although metformin may have other benefits) (1-3).

Dandona et al. have shown in two preliminary retrospective studies that the addition of liraglutide, a Glucagon Like Peptide -1 receptor agonist (GLP-1RA), to insulin results in consistent improvements in glycemic control in type 1 diabetes as demonstrated by reductions in HbA1c, glycemic excursions, and insulin requirements accompanied by improvements in body weight and systolic blood pressure (4, 5). As collaborators in the present trial, they also completed the first prospective randomised controlled trial of this drug in 72 people with type 1 diabetes. The data not only confirmed improvements in glycemic control (HbA1c), body weight and systolic BP, but also demonstrated a reduction in appetite, carbohydrate intake and low-grade inflammation (6). Importantly, quality of life was also improved. Their recent studies in type 2 diabetes have revealed that systolic blood pressure reduction with GLP-1RA (exenatide) treatment in type 2 diabetes is associated with increased plasma concentrations of ANP, cGMP and cAMP along with reduced concentrations of angiotensinogen, renin and angiotensin II (7). It is possible that these mechanisms play a role in the prevention of cardiovascular complications in type 1 diabetes. There have been no trials to date of semaglutide (a weekly and potent GLP-1RA) in type 1 diabetes.

GLP-1RAs, therefore, have great potential as adjunct therapy for type 1 diabetes. However, mean HbA1c was still ≥7.0% after liraglutide treatment (6) and larger pharma-sponsored trials (ADJUNCT-1 and ADJUNCT-2) reported increased rates of hypoglycaemia (8, 9). In seeking further improvement, the advent of Sodium-GLucose coTransporter 2 (SGLT2) inhibitors is of importance since their mode of action is independent of insulin secretion. Introduced for use in type 2 diabetes, SGLT2 inhibitors are selective antagonists of the sodium-glucose transporter subtype expressed in the proximal convoluted tubules of the kidneys. They act by inhibiting renal glucose re-absorption and therefore stimulating excretion of 60-100 grams per day of glucose in urine. SGLT2 inhibitors have been studied extensively in type 2 diabetes, including the landmark cardiovascular outcome trial EMPA-REG (10), but data have only more recently begun to accumulate in type 1 (11). Short term effects on urine glucose excretion of remogliflozin and empagliflozin are similar to those seen in type 2 diabetes and these translate into improved glycaemic and other metabolic indices including HbA1c, fasting blood glucose, rates of hypoglycaemia, daily insulin dose and weight (12). Glucose-lowering efficacy is attenuated in individuals with eGFR < 45/ml/min/1.73 m^2.(13) Weight loss accompanying increased urinary glucose excretion could translate into reduced cardiovascular complications as in type 2 diabetes, but there are no data so far available to support this hypothesis. More recently, in the Phase 3 trials DEPICT-1 and DEPICT-2 Dandona et al (PI of the US arm of this trial) showed that dapagliflozin improves glycaemic control and HbA1c over six months by about 0.45% without increasing hypoglycaemia and as in other conditions reduces urinary albumin excretion (14,15,16,17). In comparison to other SGLT2 inhibitors studied for use in type 1 diabetes [sotagliflozin and empagliflozin (18, 19)] the risk of diabetic ketoacidosis was relatively modest (see below). Both dapagliflozin and sotagliflozin were approved early in 2019 by the EU Committee for Medicinal Products for Human Use (CHMP) as adjunct therapy in type 1 diabetes for individuals with BMI≥27 kg/m (20,21)2. In August 2019, dapagliflozin (5 mg/ day) was recommended by the National Institute

for Health and Care Excellence as cost-effective for this indication in NHS England (22 However, dapagliflozin did not gain approval for this indication in the USA from the FDA (23). In November 2021, the manufacturer AstraZeneca voluntarily removed this Europe- and UK-specific indication (24) explicitly “not due to any safety concern for dapagliflozin in any condition, including type 1 diabetes” (25).

2 RATIONALE

General hypothesis: adding an SGLT2 inhibitor (dapagliflozin) to a combination of insulin and semaglutide will improve glycaemic control, weight and blood pressure in adults with type 1 diabetes.
Our protocol is designed to allow an objective assessment of whether this triple therapy combination will work effectively and safely so that a majority of people with type 1 diabetes can reach a glycaemic target HbA1c of ≤7.0%. In addition, since both semaglutide and dapagliflozin inhibitors reduce blood pressure and weight, it will allow an assessment of the combined effect of GLP-1RAs and SGLT2 inhibitors on these outcomes. Since there are no previous data on the effects of once weekly semaglutide in type 1 diabetes, or the combination of semaglutide and dapagliflozin compared to standard insulin treatment, the design requires allocation of some participants to insulin therapy only (the current standard of care).

As part of the process by which this trial was awarded funding by JDRF, this research question was considered by lay members of the funding panel and deemed important, relevant and timely. We considered that use of placebo matching dapagliflozin in Period 2 of the trial was justified when dapagliflozin had full approval as adjunct therapy for type 1 diabetes in Europe (although not the USA) as it remained uncertain which adults with type 1 diabetes were suitable for this treatment. In their review, NICE considered it likely that fewer than 10% of adults with type 1 diabetes would be initiated on SGLT2 inhibitor therapy over the first five years after their favourable decision. As individuals with a slightly lower BMI threshold will be included than recommended by the SmPC, trial participants will be more closely monitored for ketosis than was the case when dapagliflozin was approved for routine clinical care. Dapagliflozin (or placebo) will be administered orally once daily as per current licensed doses in type 2 diabetes (5 mg/day and 10 mg/day: the same doses as used in DEPICT-1 and DEPICT-2) (14,15,16,). We have not incorporated blinding for semaglutide in the design as the effects of the closely-related daily GLP-1RA liraglutide have been well-studied in double-blind trials in type 1 diabetes by ourselves and others, and individuals in both arms compared for the primary outcome comparison will be treated with this agent. (6, 8, 9). Semaglutide will be administered subcutaneously once weekly. The need to study combination adjunct therapy with SGLT 2 inhibitors and GLP-1 RAs is greater than ever following removal of the type 1 diabetes indication for SGLT2 inhibitor monotherapy in the UK and Europe.

2.1 Assessment and management of risk

a) Benefits

Semaglutide (Ozempic): is a once weekly injectable (subcutaneous) GLP1-RA licensed for reducing HbA1c in type 2 diabetes. The licensed doses are 0.25 mg weekly, 0.5 mg weekly and 1.0 mg weekly. In the trial, the starting dose is 0.25 mg weekly uptitrated if tolerated to 0.5 mg weekly after two weeks and then (again if tolerated) after a further two weeks to the maximum dose of 1.0 mg weekly. Semaglutide differs from other weekly GLP-1 receptor agonists in that its half-life is extended by binding to serum albumin i.e. it is not macromolecular. In type 2 diabetes, semaglutide therapy is associated with weight reduction and a reduction in rates of cardiovascular events (26). The licensed dose escalation schedule for routine treatment of type 2 diabetes is at four weekly intervals (27) but in a Phase 2 trial in type 2 diabetes authored by one of this trial's investigators (JP) doses were uptitrated at one-to-two weekly intervals and to higher doses (up to 1.6 mg weekly) (28). Phase 3 trials are currently in progress (for the indication of obesity) using a 2.4 mg semaglutide weekly. It

should be noted that semaglutide 0.25 mg is considered too low a dose for maintenance therapy in type 2 diabetes but it is unclear whether this may have a beneficial effect in type 1 diabetes at this dose.

While semaglutide once weekly has not been investigated previously in type 1 diabetes, liraglutide (a GLP-1 RA for daily administration with a similar molecular structure that binds less avidly to serum albumin) has previously been investigated (see above) in the ADJUNCT 1 and ADJUNCT 2 trials (8,9) in which, compared with placebo it resulted in reductions in HbA1c, weight and insulin requirement, albeit with a small increase in rates of hypoglycaemia (see below).

Dapagliflozin (Forxiga): is a once daily oral SGLT2 inhibitor previously licensed in Europe for reducing HbA1c in people with type 1 diabetes and BMI ≥ 27 kg/m^2 when insulin alone does not provide adequate glycemic control despite optimal insulin therapy (23). Although only the 5 mg dose was licensed for type 1 diabetes,both 5 mg and 10 mg daily are licensed for type 2 diabetes (29). As mentioned above, the removal of the type 1 diabetes indication by AstraZeneca was not due to any safety concern for any indication, including type 1 diabetes. In the Phase 3 trials of dapagliflozin in type 1 diabetes (DEPICT-1 and DEPICT-2) both doses were used. In both DEPICT-1 and DEPICT-2 there were modest additional benefits with 10 mg compared to 5 mg dapagliflozin. For example, in DEPICT-1, insulin dose requirement was reduced by 13.2% vs placebo on 10mg daily compared with 8.8% on 5mg daily (14). Weight reduction vs placebo in DEPICT-1 at 52 weeks was by 4.5% on dapagliflozin 10mg daily compared with 2.95% in those receiving 5 mg daily. Given the propensity of insulin to encourage weight gain over time and the life-long insulin requirement in T1D, sustained weight reduction and insulin dose reduction - even when modest - may be clinically important. There was no increase in the rate of hypoglycaemia in either the 5 mg or 10 mg groups compared with placebo. (14,15)

Dapagliflozin therapy in type 2 diabetes has been demonstrated to be associated with cardiovascular and renal risk reduction: in DECLARE-TIMI, DAPA-HF and DAPA-CKD the 10 mg dose was used (30, 31, 32). Such cardiovascular and renal benefits may translate to type 1 diabetes with appropriate monitoring and risk mitigation, although support for such measures by the manufacturer were withdrawn in the UK at the time of the removal of the type 1 indication . Given these potential benefits with relatively low increased risk, the 10 mg rather than 5 mg dose was selected for steady state treatment in this protocol.

b) Risks/ side effects Please also refer to the respective UK SmPCs (27, 29)

<u>Semaglutide</u>:

Very common: nausea, diarrhoea; hypoglycaemia (when used with insulin).
Common: vomiting, abdominal pain, constipation, gastro-oesophageal reflux, cholelithiasis, weight loss.
Uncommon: injection site reactions, hypersensitivity
Rare: Anaphylactic reaction.

Hypoglycaemia/ ketosis: Semaglutide once weekly has not been studied previously in type 1 diabetes; however, the ADJUNCT-1 and ADJUNCT-2 trials examined the role of liraglutide once daily (a similar molecule in the GLP-RA class that binds less avidly to serum albumin). Although HbA1c and weight were reduced by both 1.2 mg and 1.8 mg liraglutide, rates of symptomatic hypoglycaemia increased by 20-30%. In ADJUNCT-1, insulin doses were re-uptitrated quite rapidly towards baseline levels after an initial dose reduction, but in ADJUNCT-2 were "capped" (and therefore remained lower). Perhaps for that reason, an increase in hypoglycaemia was less consistent in ADJUNCT -2 but there was an excess of ketosis the higher (1.8 mg) dose, presumably due to under-insulinisation (no lower limit for insulin dose reduction was set). It should be noted that monitoring of participants by site investigators in the ADJUNCT trials was less intensive than will be the case in the present trial with less frequent

opportunities for insulin dose titration (see Section 7). Importantly, participants at high risk of hypoglycaemia, (including those with a history of severe hypoglycaemia and those with hypoglycaemia unawareness) were not excluded (8,9).

Retinopathy: In the SUSTAIN-6 cardiovascular outcome trial of semaglutide in type 2 diabetes, rapid reduction of HbA1c was associated with progression of existing proliferative retinopathy in those with a high baseline HbA1c ("early worsening"/ "normoglycaemic re-entry"). However, there was no evidence of worsening of retinopathy on treatment in the trials SUSTAIN 1-5 in which those with proliferative retinopathy were excluded (33, 3). In clinical trials of duration up to one year involving 4,807 patients with type 2 diabetes, adverse events related to diabetic retinopathy were reported in similar proportions of subjects treated with semaglutide (1.7%) and comparators (2.0%) (33).

Other adverse effects: In type 2 diabetes, gastrointestinal adverse effects of semaglutide can be prevented in the majority of cases by careful dose uptitration (28, 34) although up to 5% are ultimately unable to tolerate it. In our previous trial with liraglutide in T1D, adverse effects occurred with a similar frequency to that observed in T2D (in which there is extensive clinical experience); on this basis it is expected that this will also be the case with semaglutide (8,9).

Dapagliflozin:
Very common: hypoglycaemia (when used with insulin).

Common: ketoacidosis, genital infections (vulvovaginitis, balanitis), urinary tract infection, diabetic ketoacidosis, increased creatinine.

In the DEPICT-1 study, ketoacidosis occurred in 4 of 277 participants on 5mg dapagliflozin, 5 of 296 participants on 10 mg and 3 of 260 participants in the placebo group (summarised as 1%, 2% and 1% respectively) with one severe episode in each group (14). In pooled analyses of DEPICT-1 and DEPICT-2 (14,15) there was, however, an approximate threefold elevated rate of DKA adjudicated as "definite" over placebo on active therapy: 2.0% on 5mg dapagliflozin, 1.9% on 10mg and 0.6% on placebo – i.e. rates of DKA were similar with the two doses (14,15,16). Rates of DKA with dapagliflozin were described as "occurring in at least 1 per 100 patients" by the manufacturer at the time the indication was removed (24). Rates of DKA with sotagliflozin, the other SGLT2 inhibitor licensed for type 1 diabetes, were somewhat higher (2.9% on 200 mg, 3.8% on 400 mg, 0.2% on placebo) in pooled analyses of Phase 3 trials (19,21).

Increased creatinine: in 13 studies summarized in the Summary of Product Characteristics increases in serum creatinine (generally transient) occurred in 3.2% vs 1.8% of individuals with eGFR ≥ 60 ml/min/1.73 m^2 who received dapagliflozin 10 mg vs placebo rising to 18.5% vs 9.3% in those with eGFR <30 ml/min/1.73 m^2 (16,29). These changes are usually observed 4 weeks after SGLT2i initiation, recovering by week 12. Participants with eGFR < 45 ml ml/min/1.73 m^2 will not be included in the TTT1 trial and creatinine/ eGFR will be monitored at 5 weeks (Visit 12) and 13 weeks (Visit 13) after randomisation to dapagliflozin or placebo (Visit 10). Any participant who has persistent reduction in eGFR of 15 ml/min or more will meet withdrawal criteria from the trial (see Section 7.8).

Uncommon: fungal infection, volume depletion, constipation.
Not known (several case reports): Fournier's gangrene.

Fournier's gangrene is a very rare clinical entity characterized by necrotizing fasciitis of the perineal area. This has been infrequently reported in association with SGLT-2 inhibitor use in both men and women. However, there was no evidence of increased risk in a meta-analysis of 84 clinical trials

involving 42415 participants. Nevertheless, participants will be advised to seek urgent medical assistance if they experience erythema, pain, swelling of the perineal area associated with fever and malaise (this will be reinforced in the Patient Information Sheet). (36)

Amputation: An increased incidence of lower limb amputation has been reported in type 2 diabetes with the SGLT-2 inhibitor canagliflozin. However, this has not been demonstrated with dapagliflozin (not included in the UK SmPC). People with diabetes are at increased risk of amputation and good foot care is a key component of good diabetes care regardless of use of SGLT-2 inhibitors.

Placebo:
Placebo capsules contain microcrystalline cellulose.
This trial is therefore categorised by the Co-Sponsors as:

- Type B = Somewhat higher than the risk of standard medical care

See Appendix 1 (Risk Assessment)

3 OBJECTIVES AND OUTCOME MEASURES/ ENDPOINTS

3.1 Primary objective

- To assess in people with type 1 diabetes whether triple therapy (dapagliflozin, semaglutide and insulin) for six months improves glycaemic control (as measured by HbA1c) compared with dual therapy (placebo, semaglutide and insulin –Period 2).

3.2 Secondary objectives

To assess in people with type 1 diabetes whether:

- Triple therapy (dapagliflozin, semaglutide and insulin) improves glycaemic control (as measured by HbA1c) compared with standard therapy (insulin)
- Dual therapy (semaglutide and insulin – period 1) improves glycaemic control (as measured by HbA1c) in comparison with standard therapy (insulin only)
- Triple therapy (dapagliflozin, semaglutide and insulin) improves glycaemic control as assessed by a range of continuous glucose monitoring (CGM) parameters, lowers body weight, reduces insulin requirement, improves blood pressure and improves quality of life (as assessed by validated patient questionnaires) compared with dual therapy and standard therapy.

3.3 Outcome measures/endpoints

3.4 Primary endpoint/outcome

Change in HbA1c (week 26 to week 52)

3.5 Secondary endpoints/outcomes

Efficacy:

Change in HbA1c

Mean fasting blood glucose

Mean blood glucose*
Percentage time in range (3.9 - 10.0 mmol/)*
Standard deviation of blood glucose*
Percentage time in hyperglycaemia Level 1 (10.1 - 13.9 mmol/L/)*
Percentage time in hyperglycaemia Level 2 (>13.9 mmol/L) *
Percent time in hypoglycaemia Level 1 (3.0 - 3.8 mmol/)*
(by masked DEXCOM G6 CGM)*

Insulin requirement
Bodyweight
Systolic and diastolic BP
Number of antihypertensive medications
Diabetes Specific Quality of Life Scale (DSQOLS)
Problem Areas In Diabetes (PAID) questionnaire

Safety:

Rate of hypoglycemic events Level 2 (<3.0 mmol/L/ <54mg/dl)
Rate of hypoglycemic events Level 3 (severe event with altered mental and/or physical status requiring assistance)
Rate of diabetic ketoacidosis defined as elevated serum or urine ketones (greater than the upper limit of the normal range), and serum bicarbonate < 15 mmol/L or blood pH < 7.3

3.6 Tertiary endpoints/outcomes

Postprandial glucose (AUC) – meal tolerance test
Postprandial glucagon (AUC) – meal tolerance test
Postprandial FFA (AUC) – meal tolerance test
Postprandial GLP-1 (AUC) – meal tolerance test
Fructosamine

3.7 Exploratory endpoints/outcomes

Plasma and urine samples will be collected and frozen for later analysis for indices of inflammatory stress and other relevant measures.

3.8 Table of endpoints/outcomes

Endpoints	Outcome Measures	Timepoint(s) of evaluation of this outcome measure
**Primary endpoint**<br>Triple therapy (dapagliflozin, semaglutide and insulin) in comparison with dual therapy (placebo, semaglutide and insulin – period 2)	Change in HbA1c	Week 26 to Week 52
**Secondary endpoint 1**<br><br>Triple therapy (dapagliflozin, semaglutide and insulin) in comparison with standard therapy (insulin alone)	Change in HbA1c	Week 0 to Week 52
**Secondary endpoint 2**<br><br>Dual therapy (semaglutide and insulin – period 1) in comparison with standard therapy (insulin only)	Change in HbA1c	Week 0 to Week 26
**Other secondary endpoints**<br><br>a) Triple therapy (dapagliflozin, semaglutide and insulin) in comparison with dual therapy (semaglutide, insulin and placebo – period 2)<br><br>b) Dual therapy (semaglutide and insulin – period 1) in comparison with standard therapy (insulin only)	Change in:<br>- Mean fasting blood glucose (4 days)<br><br>- Mean blood glucose*<br>- Percentage time in range (3.9–10 mmol/L)*<br>- Glycaemic variability (SD)*<br>- Percent time in hyperglycaemia Level 1 (10.1-13.9 mmol/L)*<br>- Percent time in	Week 26 to Week 52<br><br>Week 0 to Week 26

	hyperglycaemia Level 2 (>13.9 mmol/L)*<br>- Percent time in hypoglycaemia Level 1* (3.0-3.8 mmol/L)<br>*(*by masked Dexcom G6 CGM)*<br><br>Insulin requirement<br>Body Weight<br>Systolic and diastolic blood pressure<br>Number of antihypertensive medications<br>Diabetes Specific Quality of Life Scale (DSQOLS)<br>Problem Areas In Diabetes (PAID) questionnaire	
**Tertiary endpoints**<br>a) Triple therapy (dapagliflozin, semaglutide and insulin) in comparison with dual therapy (semaglutide, insulin and placebo –period 2)<br><br>b) Dual therapy (semaglutide and insulin – period 1) in comparison with standard therapy (insulin only)	Change in:<br>Postprandial glucose (AUC)<br>Postprandial FFA (AUC),<br>Postprandial GLP-1 (AUC)<br>Postprandial glucagon (AUC)	Week 26 to Week 52<br><br>Week 0 to Week 26
**Safety endpoint 1**<br>a) Triple therapy (dapagliflozin, semaglutide and insulin) in comparison with dual therapy (semaglutide, insulin and placebo – period 2)<br><br>b) Dual therapy (semaglutide and insulin – period 1) in comparison with standard therapy (insulin only)	Difference between treatment arms in:<br>(i) Rate of Level 2 (<3.0 mmol/L/ <54mg/dL) hypoglycemic events<br>(ii) Rate of Level 3 hypoglycemic events (severe event with altered mental and/or physical status requiring assistance)	Week 26 to Week 52<br><br>Week 0 to Week 26
**Safety endpoint 2**<br>a) Triple therapy (dapagliflozin, semaglutide and insulin) in comparison with dual therapy (semaglutide, insulin and placebo – period 2)	Difference between treatment arms in:<br><br>Rate of diabetic ketoacidosis (serum or urine ketones above normal limits) AND serum bicarbonate < 15 mmol/L OR	Week 26 to Week 52

b) Dual therapy (semaglutide and insulin – period 1) in comparison with standard therapy (insulin only)	blood pH < 7.3	Week 0 to Week 26

4 TRIAL DESIGN

The trial has two six-month periods:

Period 1 open-label randomised (2:1) parallel group semaglutide once weekly + insulin (standard treatment) vs insulin alone (standard treatment).
Period 2 double-blind, randomised (1:1) parallel group, dapagliflozin vs placebo for those participants initially randomised to semaglutide (with ongoing standard of care for all participants)

Thus, during the first six months, approximately 114 participants with type 1 diabetes (57 in the UK) will be randomised (open label, see below) into three groups (approximately 38 each; i.e. 19 each in the UK) one of which will be maintained on their regular insulin therapy for 12 months (standard therapy group) and two of which will be started on semaglutide in addition to regular insulin treatment for six months (dual therapy). Following six months of stable treatment, those initially randomised to insulin and semaglutide will be additionally randomised (double-blind) to dapagliflozin (triple therapy) or matching placebo (dual therapy) for a further six months. The dose of dapagliflozin will not be increased to 10 mg in those who have confirmed ketonuria (+1) or ketonaemia (≥0.6 mM) after the first week of treatment.

In keeping with the intention-to-treat principle, participants in whom therapy is discontinued or downtitrated due to adverse effects will be encouraged to continue to undertake all study assessments and attend study visits.

5 STUDY SETTING

The study will be conducted at two sites: State University of New York (SUNY) at Buffalo, USA and Glasgow, UK. In Glasgow, n=57 participants (50% of the total) will be identified from secondary care sites and via the Scottish Diabetes Research Network research register. Both randomisations will be stratified by site.

6 ELIGIBILITY CRITERIA

6.1 Inclusion criteria

1) Type 1 Diabetes for at least one year on stable multiple daily (four or more) injections (MDI) of insulin or continuous subcutaneous insulin infusion (CSII) for the previous three months.
2) C-peptide < 0.23 nM
3) Minimum dose of insulin: 0.5 U/kg for MDI and 0.4 U/kg for CSII
4) Regularly measuring blood glucose: four or more times daily either by fingerstick, testing or by "flash"/ continuous glucose monitoring)
5) HbA1c 7.5 – 11.0 % inclusive
6) Well versed in carbohydrate1 counting*
7) Age 18-70 years.

8) BMI ≥25 kg/m^2.

** In the opinion of the site Principal Investigator*

6.2 Exclusion criteria

1) Type 1 diabetes for < 12 months, type 2 diabetes, chronic pancreatitis, MODY
2) Previous use of any agent other than insulin for glycaemic control in the last three months.
3) History of diabetic ketoacidosis (DKA) requiring medical intervention (e.g. emergency room visit and/or hospitalisation) within three months prior to the screening visit.
4) Following ketogenic diet, low (<50g/day) carbohydrate diet or intermittent fasting.
5) Hypoglycaemia unawareness* or frequent episodes of severe hypoglycaemia as defined by more than one episode requiring medical assistance, emergency care (paramedics or emergency room care), or glucagon therapy administered by a third-party individual within three months prior to the screening visit
6) Symptoms of poorly controlled diabetes
7) Participation in a weight loss program with ongoing weight loss, or in an intensive exercise program
8) History of bariatric or other weight-loss surgery within 12 months prior to the screening visit
9) History of Addison's disease or chronic adrenal insufficiency
10) History of diabetes insipidus
11) Hepatic disease or cirrhosis
12) Aspartate Aminotransferase (AST) > 3 X Upper limit of normal (ULN) and/or Alanine aminotransferase (ALT) > 3 X ULN
13) Serum Total Bilirubin > 2 X ULN unless exclusively caused by Gilbert's Syndrome
14) Haemoglobin < 110 g/L (11.0 g/dL) for men; haemoglobin < 100 g/L (10.0 g/dL) for women
15) Coronary event or procedure (myocardial infarction, unstable angina, coronary artery bypass, surgery or coronary angioplasty) in the previous three months or history of congestive heart failure
16) End stage renal disease on haemodialysis and/or eGFR < 45 ml/min/1.73m^2
17) HIV or Hepatitis B/C positive status
18) Any other life-threatening, non-cardiac disease
19) History of pancreatitis or cystic fibrosis
20) Women who are pregnant, planning pregnancy or breast-feeding and women of childbearing potential (WOCBP) who are not established on a method of contraception considered highly effective (see Appendix 2) and willing to continue it for the duration of the study and for at least two months after last dose of IMP.
21) Inability to give informed consent
22) History of gastroparesis
23) History of medullary thyroid carcinoma or MEN 2 syndrome
24) History of hypersensitivity reaction to GLP-1 agonists or SGLT2 inhibitors or known hypersensitivity to any of the excipients
25) Known gallstones (history of cholecystectomy is not an exclusion criterion)
26) History of alcohol problem or drug abuse
27) Hypertriglyceridemia (>500 mg/dl)
28) Recurrent genital mycotic infection (more than one episode in last six months)
29) Hypovolaemia

30) Any malignancy except treated *in situ* malignancy and basal cell carcinoma of the skin
31) Unexplained haematuria (dipstick positive on two occasions one week apart) and/or active bladder cancer
32) Proliferative retinopathy (on basis of photographic retinal screening which must have been performed within the last 12 months)
33) Use of an investigational agent or therapeutic regimen within 90 days of study
34) Participation in any other concurrent interventional clinical trial
35) Unlikely to be able to comply with study requirements*
36) Unable to undergo meal challenge for dietary reasons (e.g. vegetarian/ vegan diet)

** In the opinion of the site Principal Investigator*

7 TRIAL PROCEDURES

Also see *Schedule of Assessments*

Each participant will be enrolled in the trial for approximately 55 weeks, comprising three weeks of monitoring to stabilise treatment and collect baseline data before randomisation into the intervention or standard of care/ placebo arms for 52 weeks. Following a completed screening visit and meeting the Inclusion and Exclusion criteria, each participant will have a total of 16 scheduled study visits. In addition, at weeks 3, 5, 8, 16, 20, 29, 35, 44 and at two weeks after the end of the study, each participant will receive a phone call to document any adverse effects or other changes. No long-term follow-up is planned following completion of participation in the study.

Participants will be treated with either insulin only (standard treatment) for the whole duration of the study or with insulin and semaglutide weekly s.c. injection (titrated where possible up to 1.0 mg, see below) for the first six month period and then randomised to either oral dapagliflozin (triple therapy) or placebo (dual therapy) daily added for the second six month period. Semaglutide will be started (Visit 4) at a dose of 0.25 mg once weekly for two weeks then increased if tolerated to 0.5 mg (Visit 5). If well tolerated at week 4, it will be increased to 1.0 mg once weekly (Visit 6) or at a subsequent visit at the discretion of the PI. Dapagliflozin (or placebo) will be started at a dose of 5 mg daily at week 26 and increased at week 27 to 10 mg (2 x 5mg capsules) daily to ensure drug tolerability and minimise the risk of insulin-induced hypoglycaemia or hypotension.

Excessive weight loss: Both semaglutide and dapagliflozin are known to cause weight loss in the range of 5-10% in type 2 diabetes. However, the magnitude of weight loss when these are combined in type 1 diabetes is uncertain. Excessive weight loss may lead to increased risk of hypoglycemia or decreased insulin requirement increasing the risk of ketosis or DKA (see Section 2.1b) and other unforeseen risks. Therefore, weight loss during any phase of the study will be limited to ≤ 15% of body weight at randomisation or reaching a BMI ≤ 22 kg/m^2. If participants are approaching this threshold, IMP will be sequentially reduced at the discretion of the PI. Semaglutide may be reduced to 0.25mg or 0.5 mg and dapagliflozin to 5 mg. See also Section 7.8 regarding treatment discontinuation.

Covid-19 Mitigation

In view of the Covid-19 pandemic, certain trial procedures may be changed if necessary to reduce in person contact with participants. This will be continuously reviewed in view of the prevailing circumstances in line with local and national guidance and with the principle of protecting participants and study personnel. In the event of future waves of infection this would be particularly important. In

this context, consideration would be given to rearranging visits, changing to virtual visits using video technology or using additional phone calls to ensure patient safety. Consideration will also be given to how minimising face-to-face interaction with participants when dispensing study medication. If required, supplies of study medication sufficient to last for longer periods may be dispensed to minimise contact and visits. Courier services may additionally be used to deliver study medications to participants.

All in person visits will be subject to local and national guidance around the use of personal protective equipment including face masks and where appropriate when conducting procedures, the use of gloves and aprons.

Gastrointestinal adverse effects:

During the initial weeks of participation, those participants who report nausea and/or vomiting during uptitration of semaglutide dose that is not sufficiently severe to require treatment discontinuation will remain on their current dose until they are symptom-free prior to discussing a further dose escalation (i.e. uptitration will be to the highest dose tolerated rather than a "forced titration"). Dose will be downtitrated to the maximum tolerated in those who experience gastrointestinal adverse effects.

Retinopathy:

As there was no excess of retinopathy with semaglutide treatment in Phase 3 trials with semaglutide, in which proliferative retinopathy was an exclusion criterion (as in the current protocol), additional retinal imaging beyond annual retinal photography (the standard of care) is not proposed (30).

Insulin dose adjustment:

Minimising hypoglycaemia: All participants will be made aware of the potential for increased hypoglycaemia with addition of adjunct therapy and supported and encouraged to monitor blood glucose at least four times daily in order to minimize the risk of hypoglycaemia. Participants will have ready access to advice from specialist medical and nursing staff at face-to-face and telephone visits. Those who have ongoing access to wearable continuous glucose monitoring [or "flash" glucose monitoring, (FGM)] will continue to use these devices. Information on management of hypoglycaemia will be included within participant diaries, reinforcing the pre-existing knowledge of participants, the majority of whom will have lived with type 1 diabetes for many years.

Reductions in insulin dose will not usually be greater than 10% (5% if HbA1c is < 7.0 %) from current levels. At randomisation, daily insulin dose will not be reduced by more than 20% from the baseline level and then from the new baseline levels at 26 weeks. Participants requiring an insulin dose reduction > 20% to a total insulin dose < 0.4 U/kg (current weight) will be monitored closely for insulin dose, carbohydrate intake, ketones and blood glucose. Following the second randomisation at 26 weeks, dapagliflozin/ placebo will be temporarily discontinued if insulin dose falls below this threshold and permanently discontinued if it remains so at review in one week.

Minimising ketosis: Participants will be instructed to stay hydrated and not to reduce their caloric intake (especially carbohydrates) or start new dietary approaches (especially ketogenic diets) during the study. They will be educated to recognize the signs and symptoms of diabetic ketoacidosis (DKA) and to undertake additional self-testing of urine and/or blood ketones if they develop nausea, vomiting, or anorexia. In addition, they will be given education and a written ketosis management plan (STICH Protocol – i.e. STop SGLT-2 inhibitor or placebo (temporarily), give Insulin bolus, take additional Carbohydrate, and additional Hydration - see Appendix 3) (37).

From Visit 10 (26 weeks), those participants undergoing the second randomisation will be supported and encouraged to monitor urine or blood ketones at home at least once daily: three days before visit 10 (week 26), they will be asked to start daily measurements of urine ketones [CombiScreen® 10 SYS Plus urine testing strips, Sterilab, UK (or equivalent)] or blood ketones [CareSens™ Dual, i-SENS, Koreal; GlucoMen® Areo 2K, Menarini, UK (or equivalent]]. They will be asked to record these in the diary/ log provided. In addition, ketones (blood and urine) will be measured at every visit. If ketone bodies in urine are +1 or greater (≥0.6 mM in blood), participants will be reminded to keep well hydrated and notify the study team as soon as possible (office hours) as diabetic ketoacidosis (DKA) can occur even at glucose levels < 14 mmol/L (38).

If ketones are +2 or more in urine (> 1.5 mmol/L) participants will be advised to initiate the STICH Protocol immediately and contact the study team in office hours (or telephone NHS 24 or attend their Accident & Emergency if out of hours) (37). This information will be reinforced verbally at study visits and also in the form of a Patient Alert Card that participants will be asked to carry. This will also contain details of the investigational study drug, the study number, the investigator's name, and a contact number (for in hours contact). The study team will assess for symptoms or test results indicating a risk of ketoacidosis immediately on reporting and if blood ketone levels are persistently (one week) at these levels dapagliflozin/placebo will be permanently discontinued.

If blood ketone readings are confirmed as > 3.0 mmol/L (or urine ketones reading +++) participants will be advised to stop dapagliflozin/ placebo and attend the Accident and Emergency department without delay.

Participants will be advised that dapagliflozin/placebo may be temporarily stopped in other clinical situations known to predispose to ketoacidosis (e.g. acute serious medical illnesses or hospitalisation for major surgical procedures). Any requirement for intravenous insulin infusion (whether in Accident and Emergency or as an in-patient admission) will be recorded as a Serious Adverse Event. For further detailed procedures see section 9.31.

Volume depletion:
There is a theoretical risk of volume depletion and low blood pressure when dapagliflozin is used in combination with other anti-hypertensive drugs. All pre-existing concomitant medication will be reviewed at the screening visit as part of the medical history and examination (see Section 7.1.3 for further details). Participants undergoing the second randomisation will be educated on symptoms suggestive of low blood pressure (e.g. light-headedness, dizziness, feeling faint) and asked to contact the study team if they experience these symptoms. In clinical practice however SGLT-2 inhibitors are commonly used with anti-hypertensive drugs with no significant problems (31).

Genital mycotic infections:
Individuals who report symptoms suggestive of genital mycotic infection will be advised to self-treat with topical antifungal preparations. If a second episode occurs, a decision on treatment discontinuation will be considered in discussion between the participant and the site Principal Investigator, taking account of the severity of the symptoms.

Foot care:
Annual foot checks will be conducted for all participants as per standard of care.

7.1 Recruitment

7.1.1 Patient identification

All personal details will be managed according to ISO 27001:2013 compliant Standard Operating Procedures.

Potentially eligible participants will be identified from the Scottish Diabetes Research Network Research Register (a managed and searchable database of people with diabetes who have agreed to be contacted about clinical trial participation) and at secondary care diabetes clinics in Greater Glasgow and Clyde. The principal site for recruitment will be Stobhill Ambulatory Care Hospital but other sites may be opened if necessary as Patient Identification Centre sites. The majority of study visits (and all visits involving drug dispensing) will be conducted at the Clinical Research Facility, Glasgow Royal Infirmary. Information posters with the contact details of the research team will be displayed in recruiting clinics.

Clinic lists will be pre-reviewed by members of the usual care team in order that potentially eligible individuals can be sent an abbreviated Participant Information Sheet by post one week before their routine scheduled review visit. Eligibility criteria of those indicating agreement to be approached will then be checked at the routine visit, and the study procedures explained. Potential participants will be given a Patient Information Sheet and an Expression of Interest Form (with prepaid envelope) at this time and will be asked for permission to contact again to discuss further and (if appropriate) arrange a screening visit.

Covid-19 Mitigation: The delivery of outpatient clinics has been substantially changed due to the pandemic. The use of telephone clinics is now standard for many visits and video technologies will soon also be used. These mediums will therefore be used for inviting participants to take part when in person appointments are not taking place. This may be subject to change depending on the situation with Covid-19. Other principles of recruitment will be as described above.

7.1.2 Screening

A separate non-fasting visit will be arranged at which potential participants will have further time to discuss with the study nurse and doctor.

Prospective participants will be asked to read and understand the Participant Information Sheet. Any questions regarding the Protocol will be answered. Risks and side-effects of the active trial medication will be explained. Eligibility for trial enrolment will be confirmed by a medical practitioner.

Individual wishing to participate in the study will then be invited to sign to document their informed consent (see Section 7.2 below).

7.1.3 Screening assessments

Items from the medical history relevant to the Inclusion and Exclusion criteria will be recorded. Demographic data, medical history and current medications (including current insulin doses) will be documented. All participants will have access to a dual blood glucose and ketone meter as per the contemporary standard of care in type 1 diabetes. A physical examination will be conducted. Clinical observations will be recorded and blood and urine samples obtained (see below):

<u>Demographics</u>

- Date of birth
- Sex
- Ethnicity
- Date of diagnosis
- Ethnic group: white/black/Asian/other

- Height

Clinical assessments

- Systolic and diastolic blood pressure (after 5 minutes rest)
- Heart rate
- Body weight (clothed without coat and shoes)
- Capillary blood glucose

Medical History

- Smoking status: current/ex/never
- Alcohol consumption: units/week
- Hypertension: Y/N
- Microalbuminuria: Y/N
- Proteinuria: Y/N
- Neuropathy: Y/N
- Previous major cardiovascular event(s): Y/N (date)
- Existing CV disease of any type*: Y/N
- Amputation above forefoot: Y/N
- Hospitalisation for DKA in last year
- Level of educational attainment: University (college) educated Y/N

* Existing CV Disease is defined as any of: MI, stroke, ACS/unstable angina, coronary stent/angioplasty, CABG, TIA, heart failure, peripheral vascular disease

Insulin treatment

- Regimen: MDI, pump,
- Total dose (all); Basal (longer acting) dose/ Bolus (shorter acting) dose (for MDI only)

Other treatment (drug classes and names)

Current use Y/N. If no, has there been use in last three months?: Y/N

- ACE inhibitors
- Angiotensin receptor blocker
- Calcium channel blocker
- Alpha-blocker
- Beta-blocker
- Loop diuretic
- Thiazide like diuretic
- Statin
- Aspirin
- Clopidogrel
- Novel anticoagulant
- Warfarin

Blood assessments:

- Blood ketones
- HbA1c
- FBC
- Urea and electrolytes

- Creatinine
- Liver function tests (AST/ ALT/ Alkaline Phosphatase)
- Bone profile (Calcium/ Phosphate)
- C-peptide (capillary blood glucose must be > 4.0 mmol/L at time of sampling)

If blood tests have been assessed for clinical purposes within the last three weeks, these values will be used.

Urine sample:
- Urine ketones
- For all WOCBP, a urine pregnancy test will be checked at screening

Potential participants meeting all the inclusion and exclusion criteria based on history, examination and screening tests will be enrolled in the study.

Pregnancy: WOCBP will be asked about pregnancy status and contraceptive usage (see Appendix 2) and a pregnancy test will be conducted (following informed consent) prior to randomisation. We will not recruit those who are pregnant, wanting to become pregnant, or using birth control methods not considered highly effective. Every four weeks following randomisation, WOCBP randomised to IMP (as opposed to standard of care) will be asked about their contraception status, and a urine pregnancy test carried out. Study medication will be permanently discontinued in women who become pregnant or considered to be on contraception that is not highly effective. As the study assessments *per se* do not carry an additional risk over standard of care to either mother or unborn child, further pregnancy tests will not be routinely offered following randomisation to those women allocated to "Insulin therapy (standard of care)" (i.e. who are not exposed to IMP). All pregnancies will be notified to the Sponsor Pharmacovigilance Officer using the standard pregnancy notification form and the pregnancy followed to outcome).

7.1.4 Payment

Participants will be offered reasonable travel expenses for visits additional to usual care.

7.2 Consent

Potential participants will be identified and screened by the Inclusion and Exclusion criteria listed above. Medical staff or appropriately trained support staff will seek consent for screening and participation in the trial from individuals that fulfil the criteria. All potential participants will have at least 24 hours to review the Patient Information Sheet before being approached (see Section 7.1.2 above). Following written consent, each signature will be dated by the signatory, the original retained in the site file, a copy provided to the participant and a copy scanned into the participant's Clinical Portal record.

Data collected for routine clinical care will be used for clinical trial documentation (e.g. blood results within the previous three weeks). In the absence of routine blood results, sampling of blood for study specific laboratory measurements can only be performed following documentation of informed consent.

7.2.1 Re-screening post-consent

Where inclusion (e.g. HbA1c ≤7.5%) or exclusion (e.g. DKA within the last three months) criteria are not met (e.g. blood results falling outside the study parameters) potential participants can be re-screened with additional blood tests on one further occasion. If the initial consent was signed within two months of the re-testing, new consent is not required unless the consent form template has been updated in this period. If the initial consent was not signed within two months of re-testing then consent should be sought again. Rescreened participants will keep the same Patient ID and data will be amended on the web portal as appropriate.

7.3 Run-in period

7.3.1 Visit 1 (@3 weeks prior to randomisation)

Participants who meet entry criteria will be scheduled for their next visit at approximately three weeks prior to randomisation. Daily fingerstick blood glucose levels (study diaries), CGM/ FGM data or insulin pump download data (as applicable) will be collected and reviewed and appropriate insulin dose adjustments made by the medical team to target fasting blood glucose concentrations between 5 -10.0 mmol/L if possible at all times without having additional hypoglycaemia (< 3.9 mmol/L). Clinical observations will be recorded including resting BP measurement. Blood glucose and ketone bodies will be measured via a fingerstick blood sample (see Section 7). Urine for pregnancy status and ketone bodies will be assessed.

Participants will be instructed on the use of the Continuous Glucose Monitoring System (Dexcom G6 with blinded receiver). All subjects will be advised to monitor their capillary blood glucose by CGM or fingerstick at least 4 times a day (before meals and at bedtime) throughout the study and to wear their CGM constantly for next 20 days (changing the sensor at 10 days). CHO counting technique will be reviewed and appropriate support given with calculating bolus insulin doses.

Visit procedures:

Monitoring and treatment optimisation

- Check for adverse events
- Review blood glucose diary
- Blood ketones
- Adjust insulin doses (if necessary)

Clinical assessments

- Blood pressure
- Heart rate
- Weight

Urine sample:

- Check urine ketones

Other

- *Start* Continuous Glucose Monitoring with Dexcom G6

7.3.2 Visit 2 (1 @ weeks prior to randomisation)

Fasting visit* - Participants will be asked to fast (no food or calorie intake) for 10 hours prior to the visit overnight. Those who plan to drive to fasting visits will be reminded of the increased risk of hypoglycaemia and DVLA guidelines. This will be repeated prior to each fasting visit and reiterated

within the patient diaries. This visit will be scheduled for a morning appointment and participants will be advised not to take their breakfast bolus insulin dose prior to arrival. If participants arrive at the study visit in a non-fasted state, the visit will be terminated and a further fasting visit rearranged as soon as practicably possible.

Daily fingerstick blood glucose levels (diaries), CGM/ FGM data or insulin pump download data (as applicable) will be collected and reviewed and appropriate insulin dose adjustments made. Clinical observations will be recorded as detailed below. Blood glucose and ketone bodies will be measured via a fingerstick blood sample (see Section 7). If blood glucose is less than 3.9 mmol/L, or more than 13.9 mmol/L, the visit will be rescheduled for an alternative date. Otherwise, an intravenous cannula will be placed in order to obtain blood samples for the duration of the visit. A Meal Challenge Test will be performed (described below). A 24 hr urine container will be dispensed to collect a 24 hour urine sample to bring in at the Week 0 visit. For this collection and all subsequent 24 hour urine collections, urine will be tested via dipstick testing and an aliquot retained for assay of albumin, creatinine, ketones, and 15-isoprostane.

Visit Procedures

Monitoring and treatment optimisation

- Check for adverse events
- Review blood glucose diary
- Blood ketones
- Adjust insulin doses (if necessary)

Clinical assessments

- Blood pressure
- Heart rate
- Weight

Urine sample:

- Check urine ketones
- 24 hour urine container dispensed

Other assessments

- Download Dexcom G6 CGM
- High fat high calorie Meal Challenge: in order to assess postprandial metabolism a Meal Challenge Test will be performed using an approximately 900 kilocalorie High Fat High Carbohydrate (HFHC) meal including an egg muffin sandwich, a sausage muffin sandwich and two hash browns [containing approximately 88g carbohydrate, 51 g fat (33% saturated) and 34 g protein]. Participants will be asked to attend fasting prior to breakfast insulin bolus (with continuing basal insulin); participants will be asked not to take bolus insulin until the 3 hour time point unless blood glucose values reach above 22.2 mmol/L. Blood samples will be collected from an indwelling intravenous canula in a superficial forearm vein for glucose, glucagon, FFA, GLP-1 (AUC). Sequential blood samples will be obtained at 0, 15, 30, 45, 60, 90, 120, 150 and 180 min after consuming the meal. Volume of samples at 15, 30, 45, 90 and 150 min will be 5ml while those at 0, 60, 120 and 180min will be 30 ml (total volume = 145 ml). A spot urine sample will be collected at baseline, 1 and 3hr for standard urinalysis and an aliquot retained for assay of ketones and 15-isoprostane.

7.4 Randomisation

Participants who are being randomised will be required to have undergone all screening assessments.

At the randomisation visit, study participants will be provided with a Patient Alert Card. There are two Versions: (1) for the intervention arm i.e. those allocated at first randomisation to open-label semaglutide who are also eligible for the second double-blind randomisation (dapagliflozin or placebo); and (2) for those allocated to standard of care. These cards supplement the study diaries and include contact information for the study team. Version 1 contains unblinding information. Participants will be asked to carry their card at all times. These will be collected at the end of participation in the study.

7.4.3 First Randomisation Scheme

For the first randomisation (Period 1, open label), eligible and consenting participants will be randomised (2:1, stratified by site) to the two groups (open-label semaglutide or standard of care) using permuted blocks of variable size to minimize predictability.

7.4.4 Method of implementing the allocation sequence

Randomisation will be achieved by accessing an interactive web-based randomisation system (IWRS). The investigator will provide the participant identifier and the system will check the participant's eligibility from information already entered in the electronic Case Report Form (eCRF): if appropriate the randomisation group will be allocated.

7.5 Visit 3 (First Randomisation Visit)

Participants will be asked to attend in the fasting state (no food or calorie intake for 10 hours; see 7.3.2 above). Blood glucose levels and ketone bodies will be measured via a fingerstick blood sample using a glucose and ketone meter (see Section 7) and daily fingerstick blood glucose levels (diaries) collected and reviewed. The participant's own CGM/ FGM blood glucose data or insulin pump download data will also be collected and reviewed. Daily insulin dose will be adjusted following clinical review of study diaries: if reduced, this should not be by more than 20% from the baseline level Participants will discontinue use of the study masked CGM (Dexcom G6). Clinical observations will be recorded. Blood glucose and ketone bodies will be measured via a fingerstick blood sample (see Section 7). The 24 hour urine container will be collected. A fasting blood sample will be taken (45 mL) to evaluate study endpoints, FBC, U&E, LFT, bone profile, lipids, HbA1c, and fructosamine (detailed below). A urine sample will be collected to evaluate ketone bodies and, in females, pregnancy status.

Participants will be asked to complete Quality of Life questionnaires (see Appendices).

IMP will be dispensed for those randomised to open-label semaglutide (once weekly). Semaglutide will be started on site at the 0.25 mg dose. Participants will be instructed to call site staff directly in case of any problem or untoward side effects. An office hours phone number will be provided for advice. Outside these hours, provision of emergency medical care if required will be via NHS 24 and/or Accident & Emergency. Participants will be specifically asked to call if they have hypoglycaemia (blood glucose < 3.9 mmol/L) or Level 2 hyperglycaemia (blood glucose >13.9 mmol/L), particularly if occurring on more than one occasion. This will apply for the remainder of the trial.

Visit Procedures

Fasting visit* as per visit 2.

Monitoring and treatment optimisation

- Check for adverse events
- Check adequacy of contraception (WOCBP)
- Review blood glucose diary
- Adjust insulin doses (if necessary)
- Blood ketones

Clinical assessments

- Blood pressure
- Heart rate
- Body weight

Quality of life assessments

- Diabetes Specific Quality of Life Scale (DSQOLS)
- Problem Areas In Diabetes (PAID) questionnaire

Blood assessments

- Na, K, urea, creatinine, eGFR (MDRD)
- AST/ ALT/ Alkaline Phosphatase
- Calcium/ Phosphate
- HbA1c
- Fructosamine
- Haemoglobin
- White cell count
- Platelets
- Bilirubin
- Random glucose

Urine

- For WOCBP, a urine pregnancy test will be checked prior to first administration of IMP.
- Urine ketones
- 24 hour urine sample collected

Medication

- Administer open label semaglutide 0.25 mg if randomised to active treatment
- Issue Patient Alert Card (appropriate version according to randomisation)

Bloods for storage

Approximately 45 mls of venous blood will be withdrawn and collected in tubes containing EDTA. Blood will be centrifuged at 1500g for 20 mins at 4ºC. Plasma will be siphoned, aliquoted and stored locally at -80° ± 10°C until transport in batches to the central laboratory on dry ice.

7.5.1 Visit 4 (@1 week after first randomisation)

Clinical observations will be recorded. Blood glucose levels and ketone bodies will be measured via a fingerstick blood sample using the participant's own glucose and ketone meter (see Section 7)) and daily blood glucose levels collected and reviewed. Insulin doses will be adjusted as in visit 3. If HbA1c is <7.0 %, insulin dose reduction should not be >5% from baseline total dose. Semaglutide 0.25mg will again be injected on site for those randomised to active therapy.

Visit Procedures

Monitoring and treatment optimisation

- Check for adverse events
- Review blood glucose diary
- Adjust insulin doses (if necessary)
- Blood ketones

Assessments

- Blood pressure
- Heart rate
- Weight

Urine sample:

- Urine ketones

Medication

- Support participant's first self-injection of open label semaglutide 0.25 mg if randomised to active treatment

7.5.2 Visit 5 (@2 weeks after first randomisation)

Clinical observations will be recorded. Blood glucose levels and ketone bodies will be measured via a fingerstick blood sample using glucose and ketones meter (see Section 7) and daily blood glucose levels collected and reviewed. Daily blood glucose data collected and reviewed and insulin doses adjusted as per Visit 3. If participants randomised to semaglutide are experiencing any nausea, they will continue on the 0.25 mg dose for another week (or more as required) before increasing the dose. Otherwise semaglutide 0.5mg dose will be injected on site. Supply of IMP will be dispensed as appropriate to last until the next visit.

Visit Procedures

Monitoring and treatment optimisation

- Check for adverse events
- Review blood glucose diary
- Adjust insulin doses (if necessary)
- Blood ketones

Assessments

- Blood pressure
- Heart rate
- Weight

Urine sample:

- Urine ketones

Medication

- Dose titration (see above) if randomised to open-label semaglutide
- Return used study pen
- Dispense medication if randomised to open-label semaglutide

7.5.3 Visit 6 (@4 weeks after first randomisation)

For those randomised to open-label semaglutide, doses administered will be recorded (self-report). Clinical observations will be recorded. Daily blood glucose data will be collected and reviewed and insulin doses adjusted as per visit 3. Supply of IMP will be given to last until the next visit. A urine sample will be collected to evaluate ketone bodies and, in females randomised to IMP, pregnancy status. Unless experiencing nausea, the recommended dose will be increased to 1.0 mg a day. A 24 hr urine container will be dispensed to collect a 24 hour urine sample to bring in at the Week 13 visit.

Visit Procedures

Monitoring and treatment optimisation

- Check for adverse events
- Check adequacy of contraception (WOCBP)
- Review blood glucose diary
- Adjust insulin doses (if necessary)
- Blood ketones

Assessments

- Blood pressure
- Heart rate
- Weight

Urine sample:

- Urine ketones
- Urine pregnancy test (WOCBP randomised to IMP only)
- 24 hour urine container dispensed

Medication

- Record doses of semaglutide self-injected since last visit (self-report)
- Dose titration if randomised to open-label semaglutide (see above)
- Return used study pens
- Dispense medication if randomised to open-label semaglutide

7.5.4 Visit 7 (@13 weeks after first randomisation)

Participants will be asked to attend in the fasting state (no food or calorie intake for 10 hours). For those randomised to open-label semaglutide, doses administered will be recorded (self-report). Daily fingerstick blood glucose levels (diaries), CGM/ FGM data or insulin pump download data will be collected and reviewed and appropriate insulin dose adjustments made. Participants will discontinue use of the study CGM (Dexcom G6). Supply of IMP will be given to last until visit 10. Observations will be recorded. A urine sample will be collected to evaluate ketone bodies and, in females randomised to IMP, pregnancy status. The 24 hour urine container will be collected. A fasting blood sample will be taken (45 mL) to evaluate study endpoints.

Visit Procedures

Fasting visit*

Monitoring and treatment optimisation

- Check adequacy of contraception (WOCBP)
- Check for adverse events
- Review blood glucose diary
- Adjust insulin doses (if necessary)
- Blood ketones

Clinical assessments
- Blood pressure
- Heart rate
- Weight

Blood assessments
- Na, K, urea, creatinine, eGFR (MDRD)
- AST/ ALT/ Alkaline Phosphatase
- Calcium/ Phosphate
- HbA1c
- Fructosamine
- Haemoglobin
- White cell count
- Platelets
- Bilirubin
- Random glucose

Urine
- Pregnancy test (WOCBP randomised to IMP only)
- Urine ketones
- 24 hour urine bottle collected

Medication

- Record doses of semaglutide self-injected since last visit (self-report)
- Return used study pens
- Dispense medication if randomised to open-label semaglutide

7.5.5 Visit 8 (@23 weeks after first randomisation)

For those randomised to open-label semaglutide, doses administered will be recorded (self-report). Clinical observations will be recorded. Daily blood glucose data (fingerstick or FGM) will be collected and reviewed as per visit 3. Blood glucose levels and ketone bodies will be measured via a fingerstick blood sample. A urine sample will be collected to evaluate ketone bodies and, in females randomised to IMP, pregnancy status. Insulin doses will be adjusted if needed. All subjects will be reminded to monitor their capillary blood glucose by fingerstick at least 4 times a day (before meals and at bedtime) throughout the study. CGM sensor will be fitted and participants asked to wear their CGM constantly for next 20 days.

Visit Procedures

Monitoring and treatment optimisation
- Check adequacy of contraception (WOCBP)
- Check for adverse events
- Review blood glucose diary

- Blood ketones
- Adjust insulin doses (if necessary)

Clinical assessments
- Blood pressure
- Heart rate
- Weight

Urine sample:
- Pregnancy test (WOCBP randomised to IMP only)
- Urine ketones

Other assessments
- *Start* Continuous Glucose Monitoring with Dexcom G6

7.5.6 Visit 9 (@25 weeks after first randomisation)

Participants will be asked to attend in the fasting state (no food or calorie intake for 10 hours). Daily fingerstick blood glucose levels (diaries), CGM/ FGM data or insulin pump download data will be collected and reviewed and appropriate insulin dose adjustments made. Participants will discontinue use of the study CGM (Dexcom G6). Clinical observations will be recorded. Blood glucose levels and ketone bodies will be measured via a fingerstick blood sample (see Section 7). Supply of IMP will be given to last until the next visit. A 24 hr urine container will be dispensed to collect a 24 hour urine sample to bring in at visit 10 (Week 26) visit. A Meal Challenge Test will be performed as per visit 2. Participants will be provided with strips for urine and blood ketone measurement at home and instructed or reminded as appropriate on how to use these. They will be asked to measure urinary and blood ketones every morning for three consecutive days prior to the next visit (second randomisation: dapagliflozin or placebo) and to record the results in their diary.

Visit Procedures

Fasting visit*

Monitoring and treatment optimisation
- Check adequacy of contraception (WOCBP)
- Check for adverse events
- Review blood glucose diary
- Adjust insulin doses (if necessary) - **this insulin dose is baseline for Period 2**
- Blood ketones

Clinical assessments
- Blood pressure
- Heart rate
- Weight

Urine sample:
- Urine ketones
- 24 hour urine container dispensed

Other assessments
- Download Dexcom G6

- Meal Challenge Test

Blood assessments
- HbA1c

7.6 Second Randomisation Scheme

For the second randomisation (Period 2, double-blind), eligible and consenting participants previously randomized to open-label semaglutide will be randomised (1:1) to two groups (dapagliflozin or matching placebo) using permuted blocks of variable size to minimize predictability with stratification for HbA1c (≤ 7.5% vs >7.5% HbA1c) Those participants previously randomised to standard of care will continue as before.

7.6.1 Blinding (period 2 only)

For the double-blind treatment period, dapagliflozin and placebo will be packed in matching packs to maintain the blind. Each pack will contain sufficient supplies for 30 days' treatment with a small overage (excess). Packs will be labelled with a unique number that will be used to assign treatment to the participant via the IWRS system whilst maintaining the blind. Packs will be labelled in accordance with Good Manufacturing Practice and local regulatory requirements.

Unblinding. Treatment discontinuation (of dapagliflozin or placebo), rather than unblinding, will be carried out as far as possible. Participants will be advised in any case of hospitalisation with acute illness to discontinue study medication and inform the relevant clinician. These instructions will be reinforced in Study Diary These instructions will be reinforced in Study Diary However, where knowledge of treatment may assist emergency treatment, the investigator (or designee) may unblind using the IWRS system. Information on emergency unblinding (the investigational study drug, the study number, the investigator's name and a contact number) will be provided on the Patient Alert Card. For each unblinding an email alert is generated to the Study Coordinator and Chief Investigator. The most likely scenarios for unblinding will be: confirmed pregnancy, overdose/ accidental ingestion, or development of acute renal failure

7.6.2 Visit 10 (Second Randomisation Visit; @26 weeks after First Randomisation)

Participants will be asked to attend in the fasting state (no food or calorie intake for 10 hours). Participants with normal fasting urine (<+) or blood ketone (<0.6 mmol) in the three days prior to randomisation will proceed to the next treatment phase as per this visit. The principles of the STICH Protocol (see Appendix 3) will be discussed (37). Participants with higher ketone values in at least one of the previous three days will have their glucose levels, insulin dose, and carbohydrate intake reviewed and advised on hydration, adequate CHO intake, and adequate insulin dosing. They will be rescheduled for one week and asked to repeat three days testing before the next visit.

Clinical observations will be recorded. A urine sample will be collected for ketones. Blood glucose and ketones will be measured via a fingerstick blood sample (see Section 7.2.1). Daily fingerstick blood glucose levels (diaries), CGM/ FGM data or insulin pump download data will be collected and reviewed and appropriate insulin dose adjustments made (following the guidance in Section 7, page 35). The previous three days blood or urine ketones levels will be reviewed. The adjusted total insulin dose at this visit will serve as the baseline total insulin dose for the remaining six months of the study

and future limits of dose adjustments will be based on this new baseline. The blinded study CGM will be collected. The 24 hour urine container will be collected. A fasting blood sample will be taken (45 mL) to evaluate study endpoints. Participants will be asked to complete quality of life questionnaires.

At this point, those participants previously randomised to receive open-label semaglutide will be randomised to receive dapagliflozin 5 mg daily or matching placebo. They will be educated on symptoms suggestive of low blood pressure (e.g. light-headedness, dizziness, feeling faint).

Participants will be asked to perform a daily (preferably early morning) urine (using CombiScreen® 10 SYS Plus urine testing strips or equivalent) or blood tests for ketone bodies (using the particpant's own meters as per Section 7, "Minimising ketosis": page 28) and record all outcomes in dedicated study diaries. If results of these scheduled (i.e. not done due to symptoms) ketone measurements, are +1 or higher (urine) or > 0.6 mmol/L (blood), participants will be instructed to repeat the measurement after 60 min. Participants will be instructed to call site staff directly as soon as possible within office hours if these results are persistent, . For any abnormal ketone levels taken due to symptoms, the DKA consensus guidelines are to be followed instead (see Section 9.3).

Participants will be instructed to call site staff directly in case of any problem or untoward side effects. They will be specifically asked to call if they have confirmed hypoglycaemia (blood glucose levels < 3.9 mmol/L) or hyperglycaemia (blood glucose > 13.9 mmol/L) occurring on more than one occasion. Participants will be advised to remain well hydrated and report any genital mycotic or urinary tract infections

Visit Procedures

Fasting visit* - Requirements and arrangements as per visits 2 and 3.

Monitoring and treatment optimisation

- Check adequacy of contraception (WOCBP)
- Check for adverse events
- Review blood glucose diary
- Adjust insulin doses (if necessary)
- Blood ketones

Clinical assessments
- Blood pressure
- Heart rate
- Body weight

Quality of life assessments
- Diabetes Specific Quality of Life Scale (DSQOLS)
- Problem Areas In Diabetes (PAID) questionnaire

Blood assessments
- Na, K, urea, creatinine, eGFR (MDRD)
- AST/ ALT/ Alkaline Phosphatase
- Calcium/ Phosphate
- Fructosamine
- Haemoglobin

- White cell count
- Platelets
- Bilirubin
- Random glucose
- C-peptide (capillary blood glucose must be > 4.0 mmol/L at time of sampling)
-

Urine
- Pregnancy test (WOCBP randomised to IMP only)
- Urine ketones
- 24 hour urine bottle collected

Medication
- Education on symptoms suggestive of low blood pressure (e.g. light-headedness, dizziness, feeling faint) for those undergoing second randomisation
- Record doses of semaglutide self-injected since Visit 7 (self-report)
- Return used study pens
- Dispense open label semaglutide for participants randomised to active treatment at Period 1 (if required).
- Dispense dapagliflozin or placebo if randomised to active treatment

Other assessments
- *Read* Continuous Glucose Monitoring sensor (mean fasting blood glucose, mean blood glucose, percentage time in range, glycaemic variability)
-

7.6.3 Visit 11 (@1 week after Second Randomisation)

For those randomised to open-label semaglutide, doses administered will be recorded (self-report). Clinical observations will be recorded. Daily blood glucose and blood/ urine ketone levels will be reviewed and glucose levels collected. A urine sample will be collected to evaluate for ketones bodies. Blood glucose levels and ketones bodies will be reviewed measured via a fingerstick blood sample (see Section 7.2.1). . Daily fingerstick blood glucose levels (diaries), CGM/ FGM data or insulin pump download data will be reviewed and appropriate insulin dose adjustments made using the new baseline from Visit 10 (following the guidance in Section 7, page 35). If at any time total insulin dose reduction is >20% and total insulin dose is <0.40 U/Kg, additional monitoring (see Appendix 2) will be implemented. Unless blood (>0.6 mmol/L) or urine ketones (+ or more) have been elevated within the previous three days, dapagliflozin or placebo dose will be uptitrated to 10 mg daily (two capsules of either active or placebo medication) for the remainder of the study. Supply of IMPs will be given to last until week 39.

Visit Procedures

Monitoring and treatment optimisation
- Check adequacy of contraception.
- Check for adverse events
- Review blood glucose diary
- Adjust insulin doses (if necessary)
- Blood ketones
- Daily urine ketones review

Urine sample:

- Urine ketones

Medication

- Count returned dapagliflozin or placebo capsules
- Increase dose of dapagliflozin or placebo to 10mg (see above – review ketones)
- Dispense dapagliflozin or placebo

7.6.4 Visit 12 (@5 weeks after Second Randomisation)

For those randomised to open-label semaglutide, doses administered will be recorded (self-report). Clinical observations will be recorded. Daily blood glucose and blood/ urine ketone levels will be reviewed and glucose levels collected. Blood glucose levels and ketone bodies will be measured via a fingerstick blood sample. A urine sample will be collected to evaluate ketone bodies and if appropriate pregnancy status. Insulin dose will be adjusted if as per Visit 11. A blood sample will be taken (15 mL) to evaluate study endpoints. A 24 hr urine container will be dispensed to collect a 24 hour urine sample to bring in at the Week 39 visit

Visit Procedures

Monitoring and treatment optimisation

- Check adequacy of contraception (WOCBP)
- Check for adverse events
- Review blood glucose diary
- Adjust insulin doses (if necessary)
- Blood ketones

Clinical assessments

- Blood pressure
- Heart rate
- Weight

Blood assessments

- Na, K, urea, creatinine, eGFR (MDRD)
- AST/ ALT/ Alkaline Phosphatase
- Calcium/ Phosphate
- Haemoglobin
- White cell count
- Platelets
- Bilirubin
- Random glucose

Urine

- Pregnancy test (WOCBP randomised to IMP only)
- Urine ketones
- 24 hour urine container dispensed

7.6.5 Visit 13 (@13 weeks after Second Randomisation)

For those randomised to open-label semaglutide, doses administered will be recorded (self-report). Participants will be asked to attend in the fasting state. Clinical observations will be recorded. Daily blood glucose and blood/ urine ketone levels will be reviewed and glucose levels collected. Blood glucose levels and ketone bodies will be measured via a fingerstick blood sample. A urine sample will be collected to evaluate ketone bodies and if appropriate pregnancy status. The 24 hour urine container will be collected. Insulin dose will be adjusted if needed as in as per Visit 11. Sufficient supply of IMPs will be given until the end of the study. A fasting blood sample will be taken (45 mL) to evaluate study endpoints.

Visit Procedures

Fasting visit* - as per previous fasting visits

Monitoring and treatment optimisation

- Check adequacy of contraception (WOCBP)
- Check for adverse events
- Review blood glucose diary
- Adjust insulin doses (if necessary)
- Blood ketones
- Daily urine ketones review

Clinical assessments

- Blood pressure
- Heart rate
- Weight

Quality of life assessments

- Diabetes Specific Quality of Life Scale (DSQOLS)
- Problem Areas In Diabetes (PAID) questionnaire

Blood assessments

- Na, K, urea, creatinine, eGFR (MDRD)
- AST/ ALT/ Alkaline Phosphatase
- Calcium/ Phosphate
- HbA1c
- Fructosamine
- Haemoglobin
- White cell count
- Platelets
- Bilirubin
- Random glucose

Urine

- Pregnancy test (WOCBP randomised to IMP only)
- Urine ketones
- 24 hour urine bottle collected

Medication

- Record doses of semaglutide administered since Visit 10 (self-report)
- Dispense open label semaglutide for participants randomised to active treatment at Period 1 (if required).
- Count returned dapagliflozin or placebo capsules
- Dispense dapagliflozin or placebo as required

7.6.6 Visit 14 (@23 weeks after Second Randomisation)

For those randomised to open-label semaglutide, doses administered will be recorded (self-report). Clinical observations will be recorded. Daily blood glucose and blood/ urine ketone levels will be reviewed and glucose levels collected. Blood glucose levels and ketone bodies will be measured via a fingerstick blood sample. A urine sample will be collected to evaluate ketone bodies and if appropriate pregnancy status. Insulin dose will be adjusted if as per Visit 11. All subjects will be reminded to monitor their capillary blood glucose by fingerstick at least four times a day (before meals and at bedtime). CGM (Dexcom G6) sensor will be fitted and patients asked to wear their CGM constantly for the next 20 days.

Visit Procedures

Monitoring and treatment optimisation

- Check adequacy of contraception (WOCBP)
- Check for adverse events
- Review blood glucose diary
- Adjust insulin doses (if necessary)
- Blood ketones
- Daily urine ketones review

Clinical assessments

- Blood pressure
- Heart rate
- Weight

Urine sample:

- Pregnancy test (WOCBP randomised to IMP only)
- Urine ketones

Clinical and functional assessment

- *Start* Continuous Glucose Monitoring sensor (mean fasting blood glucose, mean blood glucose, percentage time in range, glycaemic variability)

7.6.7 Visit 15 (@25 weeks after Second Randomisation)

Participants will be asked to attend in the fasting state. For those randomised to open-label semaglutide, doses administered will be recorded (self-report). Observations will be taken. Daily blood glucose and blood/ urine ketone levels will be reviewed and glucose levels collected. Blood glucose levels (fingerstick or FGM) and ketone bodies will be measured via a fingerstick blood sample. A urine sample will be collected to evaluate urine ketone levels. Insulin dose will be adjusted if needed

as per visit 11. A 24 hr urine container will be dispensed to collect a 24 hour urine sample to bring in at the Week 52 visit. A HFHC Meal Challenge Test will be performed as in Visit 2.

Visit Procedures

Fasting visit* - Procedure as per previous fasting visits

Monitoring and treatment optimisation

- Check adequacy of contraception (WOCBP)
- Check for adverse events
- Review blood glucose diary
- Adjust insulin doses (if necessary)
- Daily urine ketones review
- Blood ketones

Assessments

- Blood pressure
- Heart rate
- Weight

Urine sample:

- Pregnancy test (WOCBP randomised to IMP only)
- 24 hour urine container dispensed

Other assessments

- Meal Challenge Test

7.6.8 Visit 16 (End of study; @26 weeks after Second Randomisation)

Participants will be asked to attend in the fasting state. Clinical observations will be recorded. Daily blood glucose and blood/ urine ketone levels will be reviewed and glucose levels collected. Blood glucose levels and ketone bodies will be measured via a fingerstick blood sample. Insulin doses will be reviewed and likely requirement for an increase following discontinuation of IMP discussed with those taking open-label semaglutide. A urine sample will be collected to evaluate ketone bodies and, if appropriate pregnancy status. The CGM will be collected. The 24 hour urine container will be collected. A fasting blood sample will be taken (45 mL) to evaluate study endpoints. We will ask the participants to complete quality of life questionnaires. A physical examination will be performed as per the screening visit. This will be the final face-to-face study visit. A safety phone call will be conducted two weeks to review blood glucose data, provide further insulin dosing advice, to document any adverse events, and to ensure that follow-up in usual clinical care has been arranged.

Visit Procedures

Fasting visit*

Monitoring and treatment optimisation

- Check for adverse events
- Review blood glucose diary
- Adjust insulin doses (if necessary)
- Blood ketones
- Daily urine ketones review

Clinical assessments

- Blood pressure
- Weight
- Heart rate
- Physical examination

Quality of life assessments

- Diabetes Specific Quality of Life Scale (DSQOLS)
- Problem Areas In Diabetes (PAID) questionnaire

Blood assessments

- Na, K, urea, creatinine, eGFR (MDRD)
- AST/ ALT/ Alkaline Phosphatase
- Calcium/ Phosphate
- HbA1c
- Fructosamine
- Haemoglobin
- White cell count
- Platelets
- Bilirubin
- Random glucose

Urine

- Pregnancy test (WOCBP randomised to IMP only)
- Urine ketones
- 24 hour collection

Other

- Record doses of semaglutide administered (self-report)
- Return used study pens
- Count returned dapagliflozin or placebo capsules
- Collect Patient Alert Card

7.7 Retention and strategies for maximizing follow-up

Participants will be encouraged to attend all study visits. Prior to commencing the trial, all potential participants will have the opportunity to read the detailed Patient Information Sheet which will outline the

level of participation requested. Potential participants will be made aware of the need for frequent study visits and asked to commit to these.

Study visits can be rearranged flexibly within a period around the planned visit as specified in the timeline of visits above (Section 7) and actual dates recorded in the eCRF. This is to maximise participant ability to attend all visits. A longer or shorter interval does not constitute a protocol deviation. Where possible, visits will be arranged to coincide with other appointments to further maximise attendance and participant convenience. Telephone contact will be frequent and will help to maintain contact with participants and ensure timely reminders of upcoming study visits can be given.

Even in the scenario of treatment discontinuation (of either IMP), participants will be encouraged to continue to attend further study visits as scheduled.

7.8 Treatment Interruptions and Withdrawal criteria

Withdrawal from study drug

Treatment interruption from study IMPs may be necessary in some participants. In the case of dapagliflozin, this will occur in any acute illness or hospitalisation which increases the risk of ketosis and ketoacidosis. Circumstances in which temporary discontinuation of dapagliflozin or placebo should occur will be discussed with all study participants. Written information will be provided in the study diaries. An emergency phone line to the study team will be available to all participants for the duration of their participation in the trial. This will be operational during office hours (Monday to Friday 9am – 5pm). Patient Alert Card Version 1 will also contain unblinding information in the event of acute intercurrent illness and a requirement for unblinding in the opinion of the acute medical team treating the participant. If any participant develops diabetic ketoacidosis (DKA) as defined as per the International Consensus guidelines on two occasions with no identifiable precipitant (section 2.1) they will be withdrawn from study medication and the UK Chief and US Principal Investigators informed.

Any other intolerable side effects or acute illness may require temporary discontinuation of the study drugs (dapagliflozin or semaglutide). In these circumstances, if the study drugs cannot be safely restarted in the judgement of the trial physician or if participants are unable to tolerate side-effects, they may be withdrawn from study treatment.

Treatment withdrawal may be necessary during the trial prior to the planned end at 52 weeks following first randomisation for any of the following reasons:

1. The site PI can stop investigational study treatment or remove participants from the trial without participants approval if for any reason he/she feels is appropriate, including: severe side effects, injury or medical condition which may place the patient at risk of further complications if he/she continue to participate, failure to take the medication as instructed, failure to keep scheduled appointments, insufficient level of self-monitoring, cancellation of the study or other administrative reasons.

2. Any woman who becomes pregnant during the period of study will be withdrawn from study treatment.

3. Participants who experience persistent severe nausea or vomiting after starting semaglutide which is not managed by dose reduction will discontinue the investigational study treatment. Those with mild or moderate nausea will continue for at least one more week. For those who continue to experience significant nausea (defined as interfering with normal activities), dose will be reduced: if these side

effects persist, they will discontinue study medication(s). Participants for whom this occurs in Period 1 will remain eligible for randomisation in Period 2.

4. Excessive weight loss during either period of the study despite treatment dose reduction (> 15% body weight or BMI < 22 kg/m2 on repeated measurements more than four weeks apart).

5. New diagnosis of other conditions included in the Exclusion Criteria

6. Change in method of insulin delivery between insulin pumps to MDI or vice versa during the study unless pump treatment interruption is temporary for a period of at most 2 weeks. Participants who upgrade to “artificial pancreas” (e.g. Medtronic’s MiniMed 670G hybrid) during the study will be withdrawn.

7. Hypoglycaemia: Two episodes of severe hypoglycaemia (requiring external intervention).

8. Ketosis (with no other identifiable precipitating factors): Persistent (1 week) asymptomatic or symptomatic and unmanaged (using STICH protocol – see section 2.1 b) blood ketone levels >1.5 mmol (or ≥+2 in urine). (37)

9. Persistent (>1 week) total insulin dose reduction of >20% from baseline AND total insulin dose <0.4U/Kg.

10. DKA: Two episode of DKA (with no identifiable precipitating factors) as defined by the expert consensus statement (34)

11. Two episodes of UTI.

12. Two episodes of mycotic genital infection.

13. Significant persistent reduction in eGFR > 15 ml/min

14. Pancreatitis: any episode judged to be due to pancreatitis by the site PI.

15. Other persistent side effects specific to study drugs

Participation in this study is voluntary. Participants have the right to refuse to participate or to withdraw from participation at any time for any reason. The site PI can stop investigational study treatment or remove participants from the trial without participant’s approval. If a patient withdraws consent and decide not to participate in the study, she/he will be asked to undergo a final study visit for his/her safety. If a subject withdraws from the research, the data collected to that point will be used toward the research finding. If applicable the subject will be asked to return any unused research drug and or devices.

Withdrawal from the study

If participants are unable or unwilling to attend all study visits they will be given an option of attending less frequently or only at the end of the study. We will request participants’ consent to be contacted by telephone and for contact with their general practitioner or other health care provider to check on their current health status. No participant will be labelled as lost to follow-up. However, participants will have

the right to withdraw consent for further participation and for further data collection: all other participants will be followed up for clinical events until study completion.

7.9 Storage and analysis of samples

Blood samples will be undertaken at the beginning and at various points during the study as outlined in detail in the schedule of visits. If blood testing has recently been undertaken in the 3 weeks prior to the study commencing, some of these blood tests may be used as baseline. Blood tests will include analysis of the full blood count, renal function (urea and electrolytes), liver function tests, bone profile, lipid profile and several specialised blood tests for biomarker assays (at central laboratory) following a high fat high caloric meal challenges to examine study endpoints.

Covid-19 Mitigation

All blood sampling or trial procedures will be undertaken following local and national guidance around personal protective equipment. Mask, gloves and aprons will be used as per current guidelines.

7.9.1 Sample collection and processing

- Samples will be appropriately labelled in accordance with the trial procedures to comply with the General Data Protection Regulation (GDPR). Biological samples collected from participants as part of this trial will be transported, stored, accessed and processed in accordance with national legislation relating to the use and storage of human tissue for research purposes and such activities shall at least meet the requirements as set out in the 2004 Human Tissue Act and the 2006 Human Tissue (Scotland) Act.
- Blood will be taken according to the Schedule of Assessments (page 17). Plasma will be siphoned, aliquoted and stored locally at -80° ± 10°C until transport in batches to the central laboratory on dry ice. according to the Sample Handling Procedure as agreed by the Joint Study Management Group
- Individual patient samples will be identified with a unique, anonymised study number.

7.9.2 Sample transport to central laboratory and analysis

- Samples will be transferred to State University of New York in a single batch at the end of recruitment to the study. More frequent transfer can be organised if there are local storage limitations.
- Samples will be transported on dry ice and stored at the central laboratory at -80° ± 10° until analysis. Transport by courier will coordinated by the Trial Manager.
- At the time of analysis individual aliquots will be defrosted at room temperature and analysed in a single batch for each biomarker of interest.
- Samples will be stored at -80° ± 10° in the central laboratory for possible future analysis for novel biomarkers.

7.10 End of trial

The end of the trial will be following the last study visit of the last subject.
At the end of the specified trial period (52 weeks following first randomisation) all participants will discontinue study medications and revert to standard of care (insulin therapy only). This process will be supervised by the trial physicians, research nurses, and research pharmacists under the supervision of the site Principal Investigator. The trial team will liaise with the usual care team to ensure a safe transition back to standard therapy. Following the final scheduled trial visit there will be a safety phone call two weeks after the last visit. This will act as a further opportunity to advise on insulin dosing and ensure there are no ongoing problems or concerns related to the study.

As discussed in section 7.8, there may be circumstances in which trial drugs need to be stopped before the specified end of the trial. If this is the case, the reason(s) for discontinuation will be recorded and the trial clinical team will facilitate safe transition back to usual standard of care. Participants will however if eligible (i.e non-exclusion criteria reason for IMP discontinuation) be asked to continue with trial visits to enable ongoing close clinical observation. If consent is withdrawn and participants voluntarily chose to leave the trial before the specified end date, all efforts by the clinical trial team will be made to safely facilitate transition back to ordinary standard of care. A follow up safety visit will be offered to all such participants and ongoing contact details will be made available should there be any further unforeseen issues relating to the clinical trial.

The trial may be stopped at an earlier stage than anticipated on the advice of the independent data monitoring committee (IDMC) to the trial steering committee (TSC) for the safety reasons detailed on page 12 and in the study safety endpoints. If this occurs, all participants will be informed and safe discontinuation of study medications will be undertaken under the supervision of the site Principal Investigator.

8 TRIAL MEDICATION

8.1 Name and description of investigational medicinal product(s)

a) Semaglutide (Ozempic®): a once weekly GLP-1 agonist injection indicated for type 2 diabetes (approved by the Scottish Medicines Consortium, January 2019).

   Doses to be used: 0.25mg increased to 0.5mg at two weeks and then to 1mg weekly s.c. injection after a further two weeks. Open label semaglutide will be added to a background of insulin treatment at week 0 for 12 months in 76 participants (38 in the UK) according to

the first randomisation; at week 26 they will be further randomised (double-blind) as below into two groups: dual (placebo, semaglutide and insulin) and triple (dapagliflozin, semaglutide and insulin) therapy.

b) Dapagliflozin (as dapagliflozin propanediol monohydrate Forxiga®): a once daily SGLT-2 inhibitor that will be administered orally in a blinded fashion.

Doses to be used: 5 mg daily increased after one week to 10 mg daily (orally). Dapagliflozin will be started according to the randomisation (Interactive Web System, hosted by University of Glasgow) in 38 participants (19 in the UK) at 26 weeks as an additional treatment on a background of insulin and semaglutide therapy and end at 52 weeks (triple therapy group). Doses to be used: 5 mg increased to 10 mg after one week.

c) Placebo to dapagliflozin: Dapagliflozin matched placebo will be started in 38 participants (19 in the UK) participants at 26 weeks as an additional treatment on a background of insulin and semaglutide therapy and end at 52 weeks (dual therapy group). Doses to be used: as for active dapagliflozin.

8.2 Legal status of Semaglutide and Dapagliflozin

Semaglutide is currently licensed in the UK for those with adult type 2 diabetes which is insufficiently controlled.

Dapagliflozin is currently licensed in the UK for insufficiently controlled type 1 and type 2 diabetes.

8.3 Drug storage and supply

Semaglutide and dapagliflozin/placebo supplies must be stored in the original containers in a locked, secure area with access limited to authorised site staff. Study supplies should be used as directed in the study protocol and not be supplied to any persons other than study participants. All IMPs will be dispensed to participants from the study site pharmacy.

Covid-19 Mitigation

As detailed above in section 7, the supply of IMP may be altered to reduce risk of Covid-19 transmission in the event of future waves of infection. This may involve providing increased quantities of IMP to participants to last longer and therefore minimise physical contact with trial personnel. Use of a courier delivery service to facilitate this may also be considered.

Semaglutide: Supplies of semaglutide pre-filled pens for subcutaneous administration will be sourced from routine hospital stocks. The following semaglutide presentations will be required:

- Semaglutide 0.25 mg: Each pre-filled pen contains 2mg semaglutide in 1.5ml. Each dose contains 0.25mg of semaglutide in 0.19ml solution.
- Semaglutide 0.5 mg: Each pre-filled pen contains 2mg semaglutide in 1.5ml solution. Each dose contains 0.5 mg of semaglutide in 0.37ml solution.

- Semaglutide 1 mg: Each pre-filled pen contains 4 mg semaglutide in 3.0ml solution. Each dose contains 1mg semaglutide in 0.74ml solution.

For all presentations each pre-filled pen is capable of delivering 4 doses which is sufficient for 4 weeks treatment. Pens are for individual participant use only. In the event of a pen-failure, a new pen should be supplied to the participant.

Prior to first use, semaglutide pens must be stored at 2-8 °C in the original container and must not be frozen. Supplies which are frozen either prior to first use or during use must be discard.

Semaglutide pre-filled pens have an in-use shelf life of 6 weeks. After first use the pre-filled pens may be stored below 30oC or in a refrigerator at 2-8 °C. Participants should be advised to store packs away from the cooling element. The pen cap should be on when the pen is not in use in order to protect the contents from light.

Wherever possible, supplies of semaglutide pre-filled pens should be utilised in a pragmatic way so as to minimise unnecessary wastage. Participants should be provided with an appropriate number of pen(s) to last until the next planned visit. A small overage may be given as necessary to accommodate visit windows etc. The correct strength of pen must be supplied for each dose titration i.e. 0.25mg, 0.5mg and 1.0 mg as tolerated.

A web-based system will be used to randomise participants to semaglutide treatment. At randomisation and all subsequent visits (planned or unplanned), the system will notify pharmacy staff at study visits of the requirement for semaglutide supplies.

Dapagliflozin 5mg or placebo capsules: Supplies of dapagliflozin 5mg and matched placebo capsules will be manufactured by NHS Scotland Pharmaceuticals Specials Service/NHS Tayside Pharmaceuticals in accordance with Good Manufacturing Practice requirements. Capsules will be packed into HDPE bottles with a tamper evident seal. Each bottle will be labelled in a double-blind fashion to meet local regulatory requirements and will be assigned a unique number. Bottles containing dapagliflozin 5 mg or placebo must be stored at room temperature at 15-25°C.

Dapagliflozin 5mg and placebo capsule supplies will be provided to study site and will only be released by the sponsor once all the appropriate regulatory and governance approvals are in place. Further information on storage requirements and supply arrangements is provided in the study specific IMP Management and Accountability Manual.

A web-based system will be used for the assignment of dapagliflozin 5mg or placebo bottles at randomisation and at study visits where there is planned dispensing of the IMP or in the event that a participant requires replacement medicines.

Other medicines: Supplies of all other medicines and associated consumables will via usual NHS supply mechanisms.

8.4 Drug accountability requirements

The Investigator or designee must maintain accurate records of all study IMP movements for accountability purposes. They should include dates, quantities, batch numbers and expiry. Records must document adequately that:

- the patients were provided the doses specified by the protocol/amendment(s)
- all study drug provided was fully reconciled.

Unused study drug must not be discarded or used for any purpose other than the present study. Further information is provided in the study specific IMP Management and Accountability Manual.

8.5 Preparation and administration of semaglutide and dapagliflozin

Semaglutide (Ozempic®) is a solution for subcutaneous injection. It is administered once weekly, on the same day each week, into the subcutaneous tissues of the abdomen, thigh or upper arm. A participant may change the day of their weekly injection as long as it has been at least three days since their last injection. Thereafter, all doses should be administered once weekly on the new dosing day. The injection can be administered at any time of the day regardless of meals.

Participants must be advised to discard the injection needle after each injection and store the pen without an injection needle attached. This may help prevent blocked needles, contamination, infection, leakage of solution and inaccurate dosing. Semaglutide should not be used if the solution does not appear clear and colourless or almost colourless. The pen is designed to be used with NovoFine or NovoTwist disposable needles and pens are packed with an appropriate quantity of NovoFine Plus needles.

Detailed information on administration of semaglutide is provided in the current Summary of Product Characteristics and product Patient Information Leaflet.

Dapagliflozin (Forxiga®)) is presented as a capsule for once daily oral administration.

8.6 Dosage schedules

Semaglutide will be administered and dosed as described. Participants will be commenced on 0.25 mg s.c once weekly before up-titration as tolerated to 1.0 mg s.c once weekly. Up-titration is quicker than is ordinarily the case in clinical practice in type 2 diabetes as discussed in Section 2.1 (22) There will be increased observation and clinical access for participants in the event of side-effects. Participants randomised to receive semaglutide will continue on the 1.0 mg s.c once weekly dose for the remainder of the study following uptitration provided they tolerate the dose as detailed in Section 7. If necessary, the dose may be adjusted.

If a participant misses a dose of semaglutide it should be administered as soon as possible and within 5 days after the missed dose. If more than 5 days have passed, the missed dose should be skipped, and the next dose should be administered on the regularly scheduled day. In both cases participants, should then resume their regular once weekly dosing schedule.

Dapagliflozin will be commenced at 5mg orally once daily and uptitrated one week after commencement to a dose of 10mg which will thereafter be continued for the remainder of the trial provided they tolerate the dose. If necessary, the dose may be adjusted.

8.7 Dosage modifications

Dapagliflozin/placebo will be commenced at one capsule (5mg or placebo) orally once daily and uptitrated one week after commencement to a dose of two capsules (10mg or placebo) which will thereafter be continued for the remainder of the trial provided they tolerate the dose. If necessary, the dose may be adjusted. (See also Section 7.8 regarding treatment discontinuation) Dapagliflozin or placebo capsules can be taken at any time of day with or without food. Capsules should be swallowed whole. If participants forget to take a dose they should be advised as follows:

- If it is 12 hours or more until the next dose, the dose should be taken as soon as remembered and the next dose taken at the usual time.
- If it is less than 12 hours until the next dose, the missed dose should be skipped. The next dose should be taken at the usual time.
- Participants should not take a double dose of dapagliflozin/placebo to make up for a forgotten dose.

8.8 Known drug reactions and interaction with other therapies

Participants with hypersensitivity to either active study medication are excluded from the trial. There are no significant interactions between semaglutide and other prescribed medications other than with agents that can lower blood glucose levels. Participants on any other oral agents which can lower blood glucose levels meet Exclusion Criteria and will not be included in this trial. In combination with insulin and dapagliflozin, semaglutide may be associated with an increased risk of hypoglycaemia. Participants will be advised of the increased risk of hypoglycaemia and will be required to closely monitor their blood glucose levels for the duration of the trial. No interactions are currently listed between warfarin and semaglutide in the British National Formulary but in view of a theoretical interaction (based on protein binding), existing arrangements for INR testing will be reviewed for any participants prescribed warfarin and additional tests conducted if indicated according to the judgement of the investigators.

Dapagliflozin is known to increase the risk of hypoglycaemia when combined with other medications which also act to lower blood glucose levels. Dapagliflozin has a mild diuretic effect and therefore there is an increased risk of hypotension when dapagliflozin is combined with either medications which deplete circulating volume (e.g diuretics) or antihypertensive drugs. Dapagliflozin is however commonly prescribed with such medications in general medical practice. All participants will have their medications reviewed at the screening visit and educated regarding symptoms that may indicate dehydration/ hypovolaemia. If there is any concern of interaction with any of the IMPs, this will be discussed with the PI before a decision regarding their ability to participate is made.

8.9 Concomitant medication

Participants should not receive any adjunct glucose-lowering therapy other than as per the randomisation schedule (e.g. with either of the active study medications).

8.10 Trial restrictions

Contraindications will follow SmPC information for Ozempic® and Forxiga. Acceptable forms of contraception for WOCBP participating in the trial are listed in Appendix 2. There is no requirement for contraception for male participants.

8.11 Assessment of compliance

Treatment adherence will be assessed by the trial medical and nursing team when participants attend for scheduled study visits.

8.12 Name and description of each Non-Investigational Medicinal Product (NIMP)

Insulin therapy is a non-investigational medicinal product in this trial. The trial is in a population who have had a diagnosis of type 1 diabetes for at least one year: therefore all participants will already be established on insulin therapy. The regimen and dosage required will by differ between participants. Participants will be using a combination of long acting (basal) insulin and shorter acting (bolus) insulin or CSII (insulin pump). As in standard of care, dose titration will depend on diet, blood glucose levels and exercise. It is anticipated that dosage adjustments will be required to achieve optimal glycaemic control and maintain participant safety.

There are pre-defined guidelines for insulin titration in this study. Insulin dose reductions should not be greater than 10% (5% if HbA1c is<7%) from current total insulin dose or more than 20% from the baseline level at randomisation and then from the new baseline levels at 26 weeks following second randomisation. Participants requiring an insulin dose reduction > 20% to a total insulin <0.4 U/kg will be monitored closely for insulin dose, carbohydrate intake, ketones and blood glucose. Study

medication can be temporarily discontinued, if necessary, for one-to-two weeks. If low insulin requirements remain, the participant will be withdrawn from study (see Section 7.8).

9 PHARMACOVIGILANCE

9.1 Definitions

Term	Definition
**Adverse Event (AE)**	Any untoward medical occurrence in a participant to whom a medicinal product has been administered, including occurrences which are not necessarily caused by or related to that product.
**Adverse Reaction (AR)**	An untoward and unintended response in a participant to an investigational medicinal product which is related to any dose administered to that participant.<br>The phrase "response to an investigational medicinal product" means that a causal relationship between a trial medication and an AE is at least a reasonable possibility, i.e. the relationship cannot be ruled out.<br>All cases judged by either the reporting medically qualified professional or the Sponsor as having a reasonable suspected causal relationship to the trial medication qualify as adverse reactions.
**Serious Adverse Event (SAE)**	A serious adverse event is any untoward medical occurrence that:<br>1. results in death<br>2. is life-threatening<br>3. requires inpatient hospitalisation or prolongation of existing hospitalisation<br>4. results in persistent or significant disability/incapacity<br>5. consists of a congenital anomaly or birth defect<br>Other 'important medical events' may also be considered serious if they jeopardise the participant or require an intervention to prevent one of the above consequences.<br>NOTE: The term "life-threatening" in the definition of "serious" refers to an event in which the participant was at risk of death at the time of the event; it does not refer to an event which hypothetically might have caused death if it were more severe*.
**Serious Adverse Reaction (SAR)**	An adverse event that is both serious and, in the opinion of the reporting Investigator, believed with reasonable probability to be due to one of the trial treatments, based on the information provided.
**Suspected Unexpected Serious Adverse Reaction (SUSAR)**	A serious adverse reaction, the nature and severity of which is not consistent with the information about the medicinal product in question set out:<br>• in the case of a product with a marketing authorisation, in the summary of product characteristics (SmPC) for that product<br>• in the case of any other investigational medicinal product, in the investigator's brochure (IB) relating to the trial in question
**Reference Safety Information**	The information used for assessing whether an adverse reaction is expected. This is contained in either the investigator's brochure or the summary of product characteristics

*Note: “Severe” is often used to describe intensity of a specific event, which may be of relatively minor medical significance. “Seriousness” is the regulatory definition supplied above.

9.2 Operational definitions for (S)AEs

Adverse events (AEs) will be recorded, notified, assessed, reported, analysed and managed in accordance with the Medicines for Human Use (Clinical Trials) Regulations 2004 (as amended) and this protocol.

All Serious Adverse Events (SAEs) occurring during the trial will be recorded within the eCRF. Non-serious AEs occurring during the trial will be recorded within the eCRF if they are considered to be related to trial medication, i.e. adverse reactions (ARs).

Exclusions from SAE reporting

Hospitalisation for the following reasons will not be considered to be SAEs:

- Treatment which was elective or pre-planned, for a pre-existing condition not associated with any deterioration in condition, e.g. pre-planned hip replacement operation which does not lead to further complications.
- Any admission to hospital or other institution for general care where there was no deterioration in condition.
- Treatment on an emergency, outpatient basis for an event **not** fulfilling any of the definitions of serious as given above and not resulting in hospital admission.
- COVID-19 infection unless results in severe illness with hospital admission.

9.3 Recording and reporting of AEs, Adverse Events of Special Interest, SAEs and SUSARs

All AEs occurring during the trial that are observed by the Investigator or reported by the participant will be recorded in the participant’s medical records whether or not attributed to trial IMP. AEs will be recorded from randomisation until the later of 30 days post cessation of trial treatment or the end of the study.

With the exception of those noted in section 9.3.1 below, adverse reactions will be recorded in a log provided in the eCRF.

Adverse Events of Special Interest, SAEs and SUSARs will be recorded in the participant’s medical records and in a specific section of the eCRF from the point of randomisation until the later of 30 days post cessation of trial treatment or the end of the study.

9.3.1 Adverse Events of Special Interest

Ketosis is a known adverse effect of dapagliflozin (see Section 2.1b; also (i) below). Hypoglycaemia is a recognised adverse effect with both semaglutide and dapagliflozin when used in combination with insulin [see (ii) below] and is defined as moderate in intensity (Level 2) when blood glucose is < 3.0 mmol/L. Thus ketosis and Level 2 hypoglycaemia events will be recorded as Adverse Events of Special Interest (AESIs).

AESIswill be recorded within the relevant section of the eCRF but do not require reporting in an expedited manner (unless the event also meets the regulatory definition of an SAE). The following information will be recorded:

- Nature of the event
- Event duration (start and end dates, if applicable
- Relationship to study drug
- Outcome

Adverse Events of Special Interest will be monitored and followed up (if applicable) until the event has resolved or a final outcome has been reached.

All Adverse Events of Special Interest must be assessed for seriousness prior to recording within the eCRF. Those meeting the definition of an SAE should also be reported accordingly as per Section 9.3.2.

(i) Ketosis and diabetic ketoacidosis

This trial will be conducted in participants who have type 1 diabetes. All participants will be taking insulin. In this condition, due to the near absence of endogenous insulin, at a cellular level there is an inability to incorporate glucose into normal intra-cellular energy yielding pathways. In this context, alternative energy yielding pathways are sought including ketone body production from fatty acids in the liver. Ketones are clinically important as they easily dissociate hydrogen ions upsetting physiologically regulated acid base balance. As ketone levels rise, there is initially a state of high blood ketone levels without significant acidosis (ketosis); if this develops further, diabetic ketoacidosis (DKA) may develop. DKA requires urgent medical input. Ketones can be easily measured via a simple urine test or fingerstick capillary blood measurement. Participants eligible for this trial will already be trained in how to do this. Although dapagliflozin, one of the IMPs in this trial, has a license for use in type 1 diabetes, it is known to increase the risk of ketosis and ketoacidosis and this can occur at lower blood glucose levels than in those who are on insulin treatment only [euglycaemic DKA] (13,14). Given this known risk, ketosis and DKA are included in the safety endpoints and as such will be recorded in the eCRF as Adverse Events of Special Interest.

DKA is defined clinically and biochemically as a combination of a state of elevated ketone levels (urine ++ or higher, and/or blood > 1.5 mmol/L) in combination with biochemical evidence of a state of metabolic acidosis (pH ≤7.30 or serum bicarbonate < 15 mmol/L). This is as defined by International Consensus Guidelines (34). DKA also varies in severity. In clinical practice the diagnosis is made when a person with type 1 diabetes has elevated ketone levels and usually – although not exclusively – elevated blood glucose levels. The diagnosis is made biochemically by assessing acid base balance through blood testing. This corresponds to the following severity classifications:

- mild DKA: pH 7.25-7.30 (H^+ 50 – 56) or bicarbonate (15 -17.9 mmol/L)
- moderate DKA: pH 7.00-7.24 (H^+ 57 – 100) or bicarbonate 10 – 14.9 mmol/L)
- severe DKA: pH <7.00 (H^+ > 100) or bicarbonate < 10 mmol/L (39)

In addition to the above, the highest level of either blood or urine ketones measured, the serum bicarbonate (if measured) and blood pH (if measured) will be recorded for each episode. Given the known risk of DKA, all participants will be made aware of this condition. Patient diaries and Patient Alert Cards will summarise advice for management of high ketone levels including an emergency ketosis management protocol (STICH Protocol – see Appendix 3) (37). All participants will be

educated as to the scenarios when this should be activated and this will also be included on the Patient Alert Card which will have the contact details of the study team and participants will be asked to carry at all times.

(ii) Hypoglycaemia

Hypoglycaemia is a low blood glucose level. Hypoglycaemia occurs in diabetes owing to difficulty in precisely matching insulin dosing with the complex factors associated with carbohydrate intake and physical activity. When too much insulin relative to carbohydrate intake is administered, blood glucose levels can drop to a level which is too low. Most people with type 1 diabetes can recognize this phenomenon due to a combination of symptoms which alert them and prompt self-checking of their capillary blood glucose level. Symptoms include weakness, malaise, headache, tremulousness, diaphoresis, palpitations and mild confusion. These are known as autonomic symptoms as they are mediated by activation of the autonomic nervous system as a response to the biological stress of low serum blood glucose. As hypoglycaemia progresses, however, symptoms progress to include confusion and agitation. If untreated this can lead to coma. These symptoms are known as neuroglycopenic symptoms as they are mediated by a lack of glucose in neural cells leading to a confused state.

Hypoglycaemia is also well defined by International Consensus (34). Like DKA, it can be classified in terms of severity. This can be based either on the absolute capillary glucose value and/or clinical features and is classed into stages:

- Level 1 (mild) hypoglycaemia: Blood glucose is 3.0 – 3.8 mmol/L inclusive. At each visit the number of episodes since the last visit during which blood glucose was within this range (but not lower) will be recorded on the "Fasting Blood Glucose" tab of the eCRF following review of the participant's study diary and (if applicable) FGM/ CGM download.
- Level 2 (moderate) hypoglycaemia: Blood glucose is < 3.0 mmol/L At each visit the number of episodes since the last visit during which blood glucose was within this range at any time will be recorded on the AESI tab of the eCRF following review of the participant's study diary and (if applicable) FGM/ CGM download.
- Level 3 (severe) hypoglycaemia: A person is unable to function because of mental or physical changes due to hypoglycaemia and requires assistance from another person to recover. These events will be recorded as SAEs (see below) independent of recording of blood glucose according to the opinion of the site Principal Investigator as by definition they jeopardise the participant or require an intervention to prevent life-threatening consequences.

The IMPs in this trial are licensed clinically in type 2 diabetes to lower blood glucose levels. It therefore follows that when used in combination with insulin there may be an increased risk of low blood glucose levels. This was the case with the once daily GLP-1 analogue liraglutide in phase 3 trials (8,9), but not in the trials with dapagliflozin (13,14). All participants will have this risk discussed with them, will be performing increased blood glucose monitoring than is the case in usual care, and will be frequently reviewed to adjust insulin dosing in order to mitigate the risk of low blood glucose levels.

9.3.2 Site Reported Serious Adverse Events (SAE)

SAEs will be recorded within the eCRF from the date of randomisation until the later of 30 days post cessation of trial treatment or the end of the study.
Full details of SAEs will be recorded in the electronic Case Report Form. The following information will be collected:

- full details in medical terms and a case description
- seriousness criteria

- event duration (start and end dates, if applicable)
- action taken
- outcome
- causality (i.e. relatedness to trial drug / investigation), in the opinion of the investigator
- if related, whether the reaction would be considered expected or unexpected.

Any change of condition or other follow-up information should be added to the eCRF and forwarded to the Sponsor (if reportable SAE) as soon as it is available or at least within 24 hours of the information becoming available. Events will be followed up until the event has resolved or a final outcome has been reached.

Reporting of suspected/confirmed COVID 19 infection

If a patient presents with clinical signs and symptoms consistent with COVID-19 and a test has been performed or requested:

- If test is positive, record “COVID-19 confirmed” in the diagnosis field
- If test is negative, record the SAE signs and symptoms and/or other diagnosis in the diagnosis field.

If a test has not been performed or result is not available and signs and symptoms, as judged by the investigator, are highly suspicious of COVID-19 infection, record “COVID-19 suspected” in the diagnosis field

If other concurrent diagnoses, e.g. pneumonia, please record within the narrative of the SAE.

COVID-19 vaccination and reporting

Where a deployed COVID-19 vaccine is suspected to be involved in the onset of a reported event it should be recorded as a concomitant medication. A causal relationship between the vaccine and the event, including potential drug interactions should be assigned by the reporting investigator.
If a reported event is suspected to be due to a deployed COVID-19 vaccine alone reporting investigators should ensure that standard Yellow Card reporting procedures are followed.

Assessment of Adverse Events

All adverse events must be assessed by the reporting site for seriousness**.** All SAEs must also be assessed for causality and expectedness with reference to this protocol and the Reference Safety Information (RSI).

Assessment of seriousness

An adverse event will be considered serious if it:

1. Results in death
2. Is life threatening
3. Requires hospitalisation or prolongation of existing hospitalisation
4. Results in persistent or significant disability or incapacity
5. Consists of a congenital anomaly or birth defect

6. Required intervention to prevent one of the above.
7. Is a serious Adverse Event of Special Interest.

Assessment of causality i.e. does the event have a "reasonable causal relationship" with trial IMPs. The following categories are used:

- **Yes** - The causal and temporal relationship between the event and the absence of a more likely explanation suggest that the trial IMP is the most likely cause.
- **No** - The event is not considered related to the trial IMPs.

Causality will be assigned for each IMP the participant is randomised to receive.

Should an SAE be considered unrelated to the administration of either trial drug then the causality of the event will be collected taking into consideration other factors, for example concomitant medications or co-morbidities, etc.

Assessment of causality is the responsibility of the Principal Investigator or medically-qualified designee(s) at each site. The CI, their delegate(s), or the Sponsor may provide an opinion regarding causality but cannot downgrade the PI's assessment.

Assessment of expectedness

Should the event be considered to be related to the trial IMP(s), an assessment should be made regarding the expectedness of the reaction, i.e. whether the reaction is a recognised adverse effect of the IMP(s).

.
Expected: consistent with the relevant product information documented in the RSI(s).
Unexpected: not consistent with the relevant product information documented in the RSI(s).

The CI, their delegate(s), or the Sponsor have overall responsibility for assigning expectedness as per the RSI(s) and provide the final assessment of the event. The reporting investigator may provide an opinion on the expectedness of an event and this may be taken into account.

SAEs occurring within the USA and the UK

The US Sponsor is responsible for assigning causality and expectedness for any AEs, SAEs and SUSARs that occur within the USA. Where a SUSAR is identified by the US Sponsor this will be forwarded to the UK Sponsor for review within one working day. Following review by the UK Sponsor, appropriate US SUSARs will be subject to expedited reporting to the MHRA as per UK regulatory requirements. Similarly, where a SUSAR is identified by the UK sponsor, this will forwarded to the US Sponsor for review within two working days.

Recording and reporting of SAEs
All SAEs arising during the clinical trial will be recorded in the eCRF soon as reasonably practicable and in any event within 24hours of first becoming aware of the event. Any follow-up information should also be reported.

If recording in the eCRF is not possible a paper SAE form should be completed:
1. The SAE form is downloaded from www.glasgowctu.org, printed off, completed and signed. The form is then faxed to the Glasgow Clinical Trials Unit Pharmacovigilance (PV) Office on

+44(0)141 357 5588. As an alternative, a scanned copy of the SAE form may be emailed to: pharmacovig@glasgowctu.org. If this website is unavailable a paper copy of the SAE form should be filed in the Investigator Site File at each site pending resolution.

2. If necessary a verbal report can be given by contacting the PV Office on +44(0)141 330 4744. This must be followed up as soon as possible with an electronic or written report.

Reporting to sponsor

All SAEs will be considered reportable to the Sponsor PV office from the time of randomisation until 30 days following permanent discontinuation of all trial IMP(s). SAEs occurring following 30 days post cessation of treatment are not reportable to the Sponsor unless an SAE is considered related to IMP(s)

9.3.3 Suspected Unexpected Serious Adverse Reactions (SUSARs)

Any SAE assessed as related to trial IMP(s) by the PI or delegate, and unexpected (not documented as an expected reaction to the IMP(s) within the RSI(s)) by the CI, their delegate, or the Sponsor, will be classified as a SUSAR and therefore subject to expedited reporting to the Medicines and Healthcare products Regulatory Agency (MHRA) and the Research Ethics Committee (REC). Should the CI disagree with the PI's assessment of causality, both opinions will be provided on the report. Where the CI or UK Sponsor disagrees with the US Sponsor's assessment of expectedness both opinions will also be provided on the report.

The Sponsor will inform the MHRA of all SUSARs that occur within the trial and the REC of all SUSARs that occur within the UK within the required expedited reporting timescales:

- **Fatal or life threatening SUSARs** - no later than 7 days of Sponsor awareness that the case fulfils the criteria for a fatal or life threatening SUSAR, and any follow up information within a further 8 days.
- **All other SUSARs** - no later than 15 days of Sponsor awareness that the case fulfils the criteria for a SUSAR.

The sponsor will report SUSARs to the MHRA via the MHRA eSUSAR reporting system and to REC by email with accompanying CTIMP Safety Report Form.

9.3.4 Unblinding for SUSAR reporting purposes

In the event of a potential SUSAR, the sponsor (but not the investigators) will be unblinded to facilitate appropriate reporting to the MHRA and REC.

9.4 Responsibilities for Safety Reporting and Review

This section details the responsibilities for reporting and reviewing safety information arising from the trial.

<u>Principal Investigator (PI)</u>:

- Attendance at the initiation meeting/teleconference
- Training of new members of the trial team in the protocol and its procedures
- Ensuring that the ISF is accurately maintained

- Dissemination of important safety or trial-related information to all stakeholders at their site
- Checking for AEs and ARs when participants attend for treatment/follow-up
- Ensuring that AEs are recorded and reported in line with the requirements of the protocol
- Ensuring that all SAEs are recorded and reported to the Sponsor within 24 hours of becoming aware of the event, and providing further follow-up information as soon as available
- Using medical judgement in assigning causality of SAEs
- Providing an opinion on the expectedness of an SAE with reference to the trial protocol and Reference Safety Information
- Using definitions in this protocol, flagging events of special interest.

Chief Investigator (CI)

- Providing clinical oversight of the safety of patients participating in the trial, including an ongoing review of the risk/benefit
- Providing clinical review of section 4.8 of the representative SmPCs on an annual basis to define the Reference Safety Information for the trial
- Using medical judgement to confirm seriousness and causality of all SARs
- Reviewing expectedness of related SAEs with reference to the trial protocol and Reference Safety Information
- Preparing the clinical sections and final sign-off of the Development Safety Update Report (DSUR)
- Using definitions in this protocol, confirming events of special interest

Sponsor:

- Central data collection and verification of AEs, SAEs, SARs and SUSARs according to the trial protocol
- Reporting safety information to the CI or delegate for the ongoing assessment of the risk / benefit
- Reporting safety information to the independent oversight committees identified for the trial (Independent Data Monitoring Committee (IDMC) and / or Trial Steering Committee (TSC)) according to the Trial Monitoring Plan.
- Expedited reporting of SUSARs to the Competent Authority (MHRA in UK) and REC within required timelines.
- Notifying Investigators of SUSARs that occur within the trial.
- Checking for (annually) and notifying PIs of updates to the Reference Safety Information for the trial.
- Preparing standard tables and other relevant information for the DSUR in collaboration with the CI and ensuring timely submission to the MHRA and REC.

Trial Steering Committee:

In accordance with the Charter for the TSC, periodically reviewing recruitment and the overall progress of the trial and liaising with the IDMC and sponsor regarding safety issues.

Independent Data Monitoring Committee:

In accordance with the Charter for the IDMC, periodically reviewing unblinded safety data in individual cases and to determine patterns and trends of events, or to identify safety issues, which would not be apparent on an individual case basis, reporting concerns to the TSC and sponsor.

9.5 Pregnancy reporting

Pregnancy is not considered an AE unless a negative or consequential outcome is recorded for the mother or child/foetus. If the outcome meets the serious criteria, this would be considered an SAE and must be reported as per SAE reporting procedure above.
Any **pregnancy** occurring in a female trial participant or female partner of a male trial participant who becomes pregnant while participating in the Trial will be reported by the PI (or designee) to the Chief Investigator and the sponsor using the sponsor Pregnancy Reporting Form (available at http://www.glasgowctu.org/) within two weeks of the PI first becoming aware of the pregnancy.
The trial participant will also be followed up to determine the outcome of the pregnancy and follow-up information forwarded to the PV office. Any resulting SAEs should be reported as per SAE reporting procedure above.

9.6 Overdose

Any IMP dose which is not administered in accordance with the Protocol should be reported to the sponsor.
If an SAE is associated with an overdose ensure that the overdose if fully described in the SAE report form.

9.7 Reporting urgent safety measures

If any urgent safety measures are taken the CI/Sponsor will phone the MHRA's Clinical Trial Unit on 020 3080 6456, ideally within 24 hours. This will be followed up no later than 3 days from the date the measures are taken, giving written notice to the MHRA (who will advise the format required) and the relevant REC of the measures taken and the circumstances giving rise to those measures. A substantial amendment must also be submitted to the MHRA.

9.8 The type and duration of the follow-up of participants after adverse events.

Adverse events and reactions will be recorded, reported and followed up in line with this protocol until study completion or for a minimum of 30 days after participant's last dose of the IMP, whichever is later.
Any SUSAR identified will be reported to the Sponsor and to the Regulatory Authorities irrespective of how long after IMP administration the reaction has occurred.

9.9 Development safety update reports

A Development Safety Update Report (DSUR) will be submitted once a year, or on request, to MHRA and REC until the trial is declared ended. The report will be submitted within 60 days of the anniversary of the issue of the Clinical Trials Authorisation for the trial. The DSUR will be prepared by the sponsor (PV Office) in liaison with the CI and submitted by the sponsor (PV Office). The DSUR will include only those SAEs reported to the co-sponsors; that is: SAEs and SUSARs occurring within the UK and SUSARs originating in the USA.

10 STATISTICS AND DATA ANALYSIS

10.1 Sample size calculation

This is based on our current data showing that in type 1 diabetes, treatment with a GLP-1 agonist, liraglutide (1.2 and 1.8mg doses) for three months leads to HbA1c suppression by an average of 0.6% (SD= 0.5%). Dapagliflozin was recently shown to reduce HbA1c by 0.45% in type 1 diabetes patients (13). Additionally, empagliflozin treatment for 8 weeks was shown to reduce HbA1c by 0.4% in type 1 diabetics (20, 21) and our current data showing additional improvement in HbA1c by an additional

0.66% at 12 weeks when dapagliflozin was added to liraglutide and insulin therapy. Conservatively estimating that the combination therapy will suppress HbA1c by 0.5% (SD≤0.7%) compared to semaglutide alone, a sample size of 38 (32 evaluable patients) in each group will be sufficient to provide 80% power at a 5% significance level, taking in consideration a 15% dropout rate. 50% of participants will be recruited in Glasgow, UK and 50% at State University of New York at Buffalo, USA.

10.2 Anticipated recruitment rate

One year (one participant per week).

10.3 Statistical analysis

The primary objective of the proposed research is to evaluate the effect of addition of dapagliflozin to semaglutide on a background of insulin treatment in type 1 diabetes patients on HbA1c at six months following start of dapagliflozin. Transformations of the data in order to meet statistical assumptions will be considered. Statistical analyses will use linear regression models to compare treatment groups, with adjustment for baseline values of study outcomes, as well as factors used to stratify the randomisation scheme. Tests will be two-sided and tested at the 5% significance level. Analyses will be carried out under the intention to treat principle, that is, according to randomised treatment allocation, regardless of treatment adherence.

A detailed Statistical Analysis Plan will be completed and signed off before database lock. Study statisticians will develop and validate the analysis programs without access to randomised treatment allocations. After database lock, the treatment codes will be released and the final analysis will be carried out.

10.4 Subgroup analyses

These will be specified in the Statistical Analysis Plan. Subgroup analyses will be implemented by extending regression models to include interactions between treatment and potential treatment effect moderators.

10.5 Interim analysis and criteria for the premature termination of the trial

No interim analysis is planned. Unblinded trial data will be reviewed on an ongoing basis by the IDMC. The primary role of the IDMC will be to protect the interests of the patients. The IDMC may recommend to the TSC and Co-Sponsors that the study should stop prematurely because of concerns about patient safety. The IDMC will meet approximately every six months. The IDMC will take into account all results and the consistency and biological plausibility of the findings in making any recommendation. The final decision on continuing or stopping the trial will lie with the TSC/Co-Sponsors.

10.6 Subject population

All analyses will be carried out on an intention to treat basis, using the randomised treatment allocation.

10.7 Procedure(s) to account for missing or spurious data

Data will be analysed without imputation in the first instance. Multiple imputation procedures will be used to assess the sensitivity of selected results to missing data, according to the Statistical Analysis Plan.

10.8 Other statistical considerations.

The Statistical Analysis Plan (SAP) will be maintained as a version controlled document and will be signed off before database lock. The SAP and other statistical documentation (according to Glasgow

Clinical Trials Unit Standard Operating Procedures) will contain full details of all analyses along with assumptions and procedures for handling problematic or incomplete data (e.g. incomplete dates).

11 DATA HANDLING

11.1 Source Documentation

ICH GCP defines source data as: 'All information in original records and certified copies of original records of clinical findings, observations or other activities in a clinical trial necessary for the reconstruction and evaluation of the trial'. In this study, the location of the majority of the source data will be the hospital's medical records including subject case notes, laboratory records and ECGs. The source data transcribed into the eCRF from the medical records must be accurate and verifiable. For questionnaires completed by trial subjects, the completed questionnaires will be regarded as the source data location. In cases where data is transcribed directly into the eCRF and no other paper or electronic source exists, then the eCRF will be considered the source record. In these cases, these data should be prospectively documented in the medical records to ensure a full record of the trial is available at site.

11.2 Data collection

An eCRF, developed by the Robertson Centre for Biostatistics, will capture all data required to meet this protocol's requirements. Access to the eCRF will be restricted, via a study-specific web portal, and only authorised site-specific personnel will be able to make entries to their patients' data via the web portal. The Investigator, or his/her designee will be responsible for all entries into the eCRF and will confirm that the data are accurate, complete and verifiable. Data will be stored in a MS SQL Server database.

Direct access to the web portal will be granted, on request, to authorised representatives from the Sponsor, host institution and the regulatory authorities to permit trial-related monitoring, audits and inspections.

11.3 Data Validation

Where it is practical, data will be validated at the point of entry into the eCRF. Any additional data discrepancies will be flagged to the investigator and any data changes will be recorded to maintain a complete audit trail (reason for change, date change made, who made change).

11.4 Data Security

The Robertson Centre for Biostatistics systems are fully validated in accordance with industry and regulatory standards, and incorporate controlled access security. High volume servers are firewall protected and preventative system maintenance policies are in place to ensure no loss of service or data. Web servers are secured by digital certificates. Data integrity is assured by strictly controlled procedures, including secure data transfer procedures. Data are backed up on-site nightly and off-site to a commercial data vault weekly. The Robertson Centre for Biostatistics has an ISO 9001:2008 quality management system and ISO 27001:2013 for Information Security, and is regularly inspected against the standards by the British Standards Institution.

11.5 Archiving

The Trial Master File will be archived by the Co-Sponsors at the end of the trial for a minimum period of five years.

Archiving of Site Files will also be for a minimum of five years form completion of the trial, and this action will be delegated to the sites in the Clinical Trial Site Agreement that will be put in place

between Co-Sponsors and Sites. Sites will be notified by the Co-Sponsors when Site files can be archived.

Destruction of site files can only take place with the approval of the Co-Sponsors.

12 MONITORING, AUDIT & INSPECTION

Monitoring will be conducted by NHS Greater Glasgow and Clyde (GG&C) Monitor (s) in accordance with local Standard Operating Procedures. The level, frequency and priorities of monitoring will be based on the outcome of the completed Risk Assessment, and clearly documented in the Monitoring Plan which will be approved by the NHS GG&C Lead Monitor and/or Research Governance Manager.

13 ETHICAL AND REGULATORY CONSIDERATIONS

13.1 Research Ethics Committee (REC) review & reports

Before the start of the trial, approval will be sought from a REC for the trial protocol, informed consent forms and other relevant documents e.g. advertisements and GP information letters.
Substantial amendments that require review by REC will not be implemented until the REC grants a favourable opinion for the study (it is noted that amendments may also need to be reviewed and accepted by the MHRA and/or NHS R&D departments before they can be implemented in practice at sites).
All correspondence with the REC will be retained in the Trial Master File/Investigator Site File.
An annual progress report (APR) will be submitted to the REC within 30 days of the anniversary date on which the favourable opinion was given, and annually until the trial is declared ended (this is the Chief Investigator's responsibility).
The Chief Investigator will notify the REC of the end of the study.
If the study is ended prematurely, the Chief Investigator will notify the REC, including the reasons for the premature termination
Within one year after the end of the study, the Chief Investigator will submit a final report with the results, including any publications/abstracts, to the REC.

13.2 Peer review

The study protocol has been developed with expert and independent feedback from the JDRF (New York) and during application for funding underwent peer review by independent experts.

13.3 Public and Patient Involvement

The study was designed with input from people with type 1 diabetes via the funder, JDRF.

13.4 Regulatory Compliance

The trial will not commence until a Clinical Trial Authorisation (CTA) is obtained from the MHRA.
The protocol and trial conduct will comply with the Medicines for Human Use (Clinical Trials) Regulations 2004 and any relevant amendments.
Before any site can enrol patients into the trial, the Chief Investigator/Principal Investigator or designee will apply for NHS permission from the site's Research & Development (R&D) department.

For any amendment that will potentially affect a site's NHS permission, the Chief Investigator/ Principal Investigator or designee will confirm with that site's R&D department that NHS permission is ongoing (note that both substantial amendments, and amendments considered to be non-substantial for the purposes of REC and/or MHRA may still need to be notified to NHS R&D).

13.5 Protocol compliance

Prospective, planned deviations or waivers to the protocol are not allowed under the UK regulations on Clinical Trials and must not be used e.g. it is not acceptable to enrol a participant if they do not meet the eligibility criteria or restrictions specified in the trial protocol. Accidental protocol deviations can happen at any time. They must be adequately documented on the relevant forms and reported to the Chief Investigator, Sponsor and GCTU immediately. Deviations from the protocol which are found to frequently recur are not acceptable, will require immediate action and could potentially be classified as a serious breach.

13.6 Notification of Serious Breaches to GCP and/or the protocol

A “serious breach” is a breach which is likely to affect to a significant degree –

1. the safety or physical or mental integrity of the subjects of the trial; or
2. the scientific value of the trial

If any of the above occurs then the CI and Sponsor will be notified. The sponsor will notify the appropriate authorities in writing of any serious breach in accordance with their standard operating procedures.

13.7 Data protection and patient confidentiality

All investigators and trial site staff must comply with the requirements of the GDPR with regards to the collection, storage, processing and disclosure of personal information and will uphold the Act’s core principles.

- Personal information will be collected via the eCRF to enable record linkage to be carried out and to provide electronic access to study monitors to a copy of the signed informed consent document. These data items will be encrypted and only those individuals who require to see these data i.e. the person performing the record linkage and site research team staff or the study monitor, as appropriate, will be able to view them. All electronic data will be held securely in accordance with ISO 27001:2013 at the Robertson Centre for Biostatistics, part of the Glasgow Clinical Trials Unit. All Centre staff are required to sign confidentiality agreements and to follow Standard Operating Procedures in accordance with Good Clinical Practice and ISO certification.
- The trial data managers, statisticians, health economists or any other staff who will perform data related tasks will only be able to access depersonalised data where the participant’s identifying information is replaced by a unique study identifier.
- Only those that have been trained and approved will be able to enter or view any data via the web portal. Each site can only see their own patients’ data. Patient consent forms will be stored at the study site in a secure location accessible only to study teams.

13.8 Indemnity

The Co-Sponsors (University of Glasgow and Greater Glasgow Health Board) will ensure that provision has been made for insurance or indemnity to cover the liability of the investigator and sponsor which may arise in relation to the clinical trial in accordance with Part 2 (14) of Schedule 1 to SI 2004/1031.

13.9 Amendments

Any change in the study protocol will require an amendment. Any proposed protocol amendments will be initiated by the CI following discussion with the Sponsor and TSC and any required amendment forms will be submitted to the regulatory authority, ethics committee and Sponsor. The Sponsor will determine whether an amendment is non-substantial or substantial. All amended versions of the protocol will be signed by the CI and Sponsor representative. Following a substantial amendment, favourable opinion/approval must be sought from the original reviewing REC, MHRA (where appropriate) and Research and Development (R&D) office prior to implementation. The Chief Investigator will be responsible for informing the Trial Management Group of all protocol amendments.

13.10 Post trial care

At the end of the trial, participants will be returned to usual care as defined by local and national guidelines at that time. The results of the trial may of course have an impact on these guidelines and the future care of patients with type 1 diabetes.

13.11 Access to the final trial dataset

During the trial and in the period prior to publication of the main study results as described in the protocol, only the Glasgow CTU and Professor Paresh Dandona, US Principal Investigator, University of Buffalo will have access to the full dataset. After that period, the trial Steering Committee will conduct further data analyses for a period of three years. After that time the Trial Steering Committee will consider requests from external parties for further analyses of the study data. Proposals that are scientifically well founded and have an academic basis and where relevant data extractions and analyses are appropriately funded will not be refused. These will be considered as collaborative exercises where the contributions related to study design, conduct, database creation and maintenance and data analysis will be recognised in authorship of any scientific publication. The approach we will take will be to minimise any possibility of breach of participant confidentiality. Normally this will be achieved by minimising data travel. However, for the purposes of individual patient meta-analysis and other reasons, data may be transferred to other sites. Such transfer will require assurances on information security systems at the sites that data are to be transferred to and will involve a legal data transfer agreement. A log of all data requests and subsequent data transfers will be held at the Glasgow CTU.

14 DISSEMINATION POLICY

14.1 Dissemination policy

The study database will be owned by the University of Glasgow and maintained on behalf of the Study investigators, represented by the Trial Steering Committee as it is constituted during and after the trial.

The study protocol and a description of the recruitment experience and participant baseline characteristics will be published before study completion. On completion of the trial, the database will be locked and analysed by staff of the Robertson Centre for Biostatistics, University of Glasgow. A final study report will be prepared and the results will be published in a major medical journal.

After the main study publications, study investigators will be invited to submit requests for further analyses of the study database. These will be reviewed and prioritised by a Publications Committee made up of the study grant holders and convened by the study co-CIs.

As funder of the trial, JDRF will have the right to see and comment on any results being submitted for publication. A maximum of 28 days will be given for review of major papers and 14 days for abstracts. Such comments will be considered by the Trial Steering Committee.

A lay summary of the main results of the trial will be prepared and provided to all participants via their study site investigators. Investigators may request a copy of the study data for their participants. Providing some or all of a patient's data to that individual is at their discretion.

14.2 Authorship eligibility guidelines and any intended use of professional writers

The main results of the study will be compiled, written up and published by the study grant holders and others taking responsibility for the study results (e.g. the statistician conducting the final analysis) on behalf of the TTT1 investigators. The TTT1 investigators will be listed in an Appendix and will include all site PIs, all committee members and key members of relevant study coordinating groups (including the Sponsor and Glasgow CTU).

15 REFERENCES

1. Konrad K, Datz N, Engelsberger I, Grulich-Henn J, Hoertenhuber T, Knauth B, et al. Current use of metformin in addition to insulin in pediatric patients with type 1 diabetes mellitus: an analysis based on a large diabetes registry in Germany and Austria. *Pediatr Diabetes.* 2014.
2. Vella S, Buetow L, Royle P, Livingstone S, Colhoun HM, and Petrie JR. The use of metformin in type 1 diabetes: a systematic review of efficacy. *Diabetologia.* 2010;53(5):809-20.
3. Vella S, Buetow L, Royle P, Livingstone S, and Petrie JR. Metformin in type 1 diabetes reduces insulin requirements without significantly improving glycaemic control. Reply to Schatz H [letter]. *Diabetologia.* 2011;54(1):203-4.
4. Varanasi A, Bellini N, Rawal D, Vora M, Makdissi A, Dhindsa S, et al. Liraglutide as additional treatment for type 1 diabetes. *Eur J Endocrinol.* 2011;165(1):77-84.
5. Kuhadiya ND, Malik R, Bellini NJ, Patterson JL, Traina A, Makdissi A, et al. Liraglutide as additional treatment to insulin in obese patients with type 1 diabetes mellitus. *Endocrine practice : official journal of the American College of Endocrinology and the American Association of Clinical Endocrinologists.* 2013;19(6):963-7.
6. Kuhadiya ND, Dhindsa S, Ghanim H, Mehta A, Makdissi A, Batra M, et al. Addition of Liraglutide to Insulin in Patients With Type 1 Diabetes: A Randomized Placebo-Controlled Clinical Trial of 12 Weeks. *Diabetes Care.* 2016;39(6):1027-35.
7. Chaudhuri A, Ghanim H, Makdissi A, Green K, Abuaysheh S, Batra M, et al. Exenatide induces an increase in vasodilatory and a decrease in vasoconstrictive mediators. *Diabetes Obes Metab.* 2017;19(5):729-33.
8. Mathieu C, Zinman B, Hemmingsson JU, Woo V, Colman P, Christiansen E, et al. Efficacy and Safety of Liraglutide Added to Insulin Treatment in Type 1 Diabetes: The ADJUNCT ONE Treat-To-Target Randomized Trial. *Diabetes Care.* 2016;39(10):1702-10.
9. Ahren B, Hirsch IB, Pieber TR, Mathieu C, Gomez-Peralta F, Hansen TK, et al. Efficacy and Safety of Liraglutide Added to Capped Insulin Treatment in Subjects With Type 1 Diabetes: The ADJUNCT TWO Randomized Trial. *Diabetes Care.* 2016;39(10):1693-701.
10. Zinman B, Wanner C, Lachin JM, Fitchett D, Bluhmki E, Hantel S, et al. Empagliflozin, Cardiovascular Outcomes, and Mortality in Type 2 Diabetes. *N Engl J Med.* 2015;373(22):2117-28.
11. Petrie JR. SGLT2 inhibitors in type 1 diabetes: knocked down, but up again? *Lancet Diabetes Endocrinol.* 2017;5(11):841-3.
12. Mudaliar S, Armstrong DA, Mavian AA, O'Connor-Semmes R, Mydlow PK, Ye J, et al. Remogliflozin etabonate, a selective inhibitor of the sodium-glucose transporter 2, improves serum glucose profiles in type 1 diabetes. *Diabetes Care.* 2012;35(11):2198-200.
13. Kohan DE, Fioretto P, Tang W, List JF. Long-term study of patients with type 2 diabetes and moderate renal impairment shows that dapagliflozin reduces weight and blood pressure but does not improve glycemic control. Kidney Int. 2014 Apr;85(4):962-71. doi: 10.1038/ki.2013.356. Epub 2013 Sep 25. PMID: 24067431; PMCID: PMC3973038.
14. Dandona P, Mathieu C, Phillip M, Hansen L, Griffen SC, Tschope D, et al. Efficacy and safety of dapagliflozin in patients with inadequately controlled type 1 diabetes (DEPICT-

1): 24 week results from a multicentre, double-blind, phase 3, randomised controlled trial. *Lancet Diabetes Endocrinol.* 2017;5(11):864-76.

15. Mathieu C, Dandona P, Gillard P, Senior P, Hasslacher C, Araki E, Lind M, Bain SC, Jabbour S, Arya N, Hansen L, Thorén F, Langkilde AM; DEPICT-2 Investigators. Efficacy and Safety of Dapagliflozin in Patients With Inadequately Controlled Type 1 Diabetes (the DEPICT-2 Study): 24-Week Results From a Randomized Controlled Trial. Diabetes Care. 2018 Sep;41(9):1938-1946.

16. European Medicines Agency. Assessment Report Dapagliflozin. https://www.ema.europa.eu/en/documents/variation-report/forxiga-h-c-ws-1941-epar-assessment-report-variation_en.pdf *(accessed 11th November 2021)*

17. Groop PH, Dandona P, Phillip M, Gillard P, Edelman S, Jendle J, Xu J, Scheerer MF, Thoren F, Iqbal N, Repetto E, Mathieu C. Effect of dapagliflozin as an adjunct to insulin over 52 weeks in individuals with type 1 diabetes: post-hoc renal analysis of the DEPICT randomised controlled trials. Lancet Diabetes Endocrinol. 2020 Oct;8(10):845-854. doi: 10.1016/S2213-8587(20)30280-1. PMID: 32946821.

18. Rosenstock J, Marquard J, Laffel LM, et al. Empagliflozin as adjunctive to insulin therapy in type 1 diabetes: the EASE trials. *Diabetes Care*. 2018;41(12):2560–2569.

19. Garg SK, Henry RR, Banks P, Buse JB, Davies MJ, Fulcher GR, et al. Effects of Sotagliflozin Added to Insulin in Patients with Type 1 Diabetes. *N Engl J Med.* 2017;377(24):2337-48.

20. European Medicines Agency. First oral add-on treatment to insulin for treatment of certain patients with type 1 diabetes. https://www.ema.europa.eu/en/news/first-oral-add-treatment-insulin-treatment-certain-patients-type-1-diabetes *(accessed 11th November 2021)*

21. An overview of Zynquista and why it is authorised in the EU. 16th May 2019. https://www.ema.europa.eu/en/documents/overview/zynquista-epar-medicine-overview_en.pdf *(accessed 11th November 2021)*

22. National Institute for Health and Care Excellence. Dapagliflozin with insulin for treating type 1 diabetes. Technology appraisal guidance [TA597] https://www.nice.org.uk/guidance/ta597

23. AstraZeneca Press Release. Update on US regulatory decision for Farxiga in type-1 diabetes. https://www.astrazeneca.com/media-centre/press-releases/2019/update-on-us-regulatory-decision-for-farxiga-in-type-1-diabetes-15072019.html# *(accessed 11th November 2021)*

24. European Medicines Agency. Forxiga (dapagliflozin) 5mg should no longer be used for the treatment of Type 1 Diabetes Mellitus. https://www.ema.europa.eu/en/documents/dhpc/direct-healthcare-professional-communication-dhpc-forxiga-dapagliflozin-5mg-should-no-longer-be-used_en.pdf *(accessed 11th November 2021)*

25. AstraZeneca. Letter to UK Health Professionals. Forxiga (dapagliflozin) 5mg should no longer be used for the treatment of type 1 diabetes mellitus. (2nd November 2021)

26. Marso SP, Bain SC, Consoli A, Eliaschewitz FG, Jódar E, Leiter LA, Lingvay I, Rosenstock J, Seufert J, Warren ML, et al. Semaglutide and cardiovascular

outcomes in patients with type 2 diabetes. *N Engl J Med.* 2016;**375**(19):1834–44. doi: 10.1056/NEJMoa1607141.

27. Ozempic Summary of product characteristics. https://www.medicines.org.uk/emc/product/9728/smpc
28. Nauck MA, Petrie JR, Sesti G, Mannucci E, Courrèges JP, Lindegaard ML, Jensen CB, Atkin SL; Study 1821 Investigators. A Phase 2, Randomized, Dose-Finding Study of the Novel Once-Weekly Human GLP-1 Analog, Semaglutide, Compared With Placebo and Open-Label Liraglutide in Patients With Type 2 Diabetes. Diabetes Care. 2016 Feb;39(2):231-41.
29. Forxiga 5 mg Summary of Product Characteristics: https://www.medicines.org.uk/emc/product/2865/smpc *(accessed 11th November 2021)*
30. Wiviott SD, Raz I, Bonaca MP, Mosenzon O, Kato ET, Cahn A, Silverman MG, Zelniker TA, Kuder JF, Murphy SA, Bhatt DL, Leiter LA, McGuire DK, Wilding JPH, Ruff CT, Gause-Nilsson IAM, Fredriksson M, Johansson PA, Langkilde AM, Sabatine MS; DECLARE–TIMI 58 Investigators. Dapagliflozin and Cardiovascular Outcomes in Type 2 Diabetes. N Engl J Med. 2019 Jan 24;380(4):347-357.
31. McMurray JJV, Solomon SD, Inzucchi SE, Køber L, Kosiborod MN, Martinez FA et al. DAPA-HF Trial Committees and Investigators Dapagliflozin in patients with heart failure and reduced ejection fraction. N Engl J Med. 2019. Nov 21;381(21):1995-2008.
32. Heerspink HJL, Stefánsson BV, Correa-Rotter R, Chertow GM, Greene T, Hou FF, Mann JFE, McMurray JJV, Lindberg M, Rossing P, Sjöström CD, Toto RD, Langkilde AM, Wheeler DC; DAPA-CKD Trial Committees and Investigators. Dapagliflozin in Patients with Chronic Kidney Disease. N Engl J Med. 2020 Oct 8;383(15):1436-1446. doi: 10.1056/NEJMoa2024816. Epub 2020 Sep 24. PMID: 32970396.
33. Vilsbøll T, Bain SC, Leiter LA, et al. Semaglutide, reduction in glycated haemoglobin and the risk of diabetic retinopathy. *Diabetes Obes Metab*. 2018;20(4):889–897.
34. Lingvay I, Desouza CV, Lalic KS et al. A 25 week randomized controlled trial of semaglutide once daily versus liraglutide and placebo in patients with type 2 diabetes suboptimally controlled on diet and exercise with or without metformin. Diabetes Care. 2018;41(9): 1926-1937.
35. European Medicines Agency. Assessment Report Sotagliflozin. https://www.ema.europa.eu/en/documents/assessment-report/zynquista-epar-public-assessment-report_en.pdf *(accessed 11th November 2021)*
36. Silverii GA, Decembrini L, Monami M, Mannucci E. Fournier's gangrene and sodium glucose co-transporter 2 inhibitors: A metanalysis of randomized controlled trials. Diabetes Obes Metab. 2019; 1-4.
37. Garg SK, Peters AL, Buse JB, Danne T. Strategy for mitigating DKA risk in patients with Type 1 diabetes on adjunctive treatment with SGLT-2 inhibitors: A STICH Protocol. Diabetes Technol Ther. 2018;20(9):571-575.
38. Peters AL, Buschur EO, Buse JB, Cohan P, Diner JC, and Hirsch IB. Euglycemic Diabetic Ketoacidosis: A Potential Complication of Treatment With Sodium-Glucose Cotransporter 2 Inhibition. Diabetes Care. 2015;38(9):1687-93.
39. Agiostratidou G, Anhalt H, Ball D, Blonde L, Gourgari E, Harriman KN, Kowalski AJ, Madden P, McAuliffe-Fogarty AH, McElwee-Malloy M, Peters A, Raman S, Reifschneider

K, Rubin K, Weinzimer SA. Standardizing Clinically Meaningful Outcome Measures Beyond HbA(1c) for Type 1 Diabetes: A Consensus Report of the American Association of Clinical Endocrinologists, the American Association of Diabetes Educators, the American Diabetes Association, the Endocrine Society, JDRF International, The Leona M. and Harry B. Helmsley Charitable Trust, the Pediatric Endocrine Society, and the T1D Exchange. Diabetes Care. 2017 Dec;40(12):1622-1630.

16. APPENDICES

16.1 Appendix 1 – Risk

Risks associated with trial interventions<br>☐ LOW ≡ Comparable to the risk of standard medical care<br>☒ MODERATE ≡ Somewhat higher than the risk of standard medical care<br>☐ HIGH ≡ Markedly higher than the risk of standard medical care				
Justification: Briefly justify the risk category selected and your conclusions below (where the table is completed in detail the detail need not be repeated, however a summary should be given):<br>An IDMC will be convened to monitor all SAEs.<br>Risks and mitigations associated with the intervention are outlined in more detail in the Protocol section 2.1.<br>Co-Sponsors will also carry out a detailed risk assessment of all aspects of the study as part of the approval process (SOP 04.013)				
What are the key risks related to therapeutic interventions you plan to monitor in this trial?		How will these risks be minimised?		
IMP/Intervention	Body system/Hazard	Activity	Frequency	Comments
S/C administration of semaglutide<br>Oral admistration of dapagliflozin	Immune: Hypersensitivity/ana-phylactic reactions	Patients with known hypersensitivity to any GLP-1 agonist or SGLT-2 inhibitor have a contra-indication to the IMP according to the SmPC will not be recruited to the study	Uncommon/ Rare	
PO administration of dapagliflozin	Ketosis and/or Diabetic Ketoacidosis (DKA)	All participants will have access to blood or urine ketone monitoring equipment. An emergency ketosis management plan (STICH Protocol – see above) will be discussed with all participants. Patient alert cards will be provided to all participants. Potential	Common	“and” was an error – intention was for “either” blood or urine ketones between visits but “both” to be measured for visits. This is compatible with the eCRF, the diaries and the rest of the protocol

		participants with recent ketoacidosis will be excluded from the trial (section 6.2)		
S/C administration of semaglutide (+/-) PO administration of dapagliflozin in addition to insulin therapy	Increased risk of hypoglycaemia	Participant awareness of risk and frequent testing of capillary blood glucose. Patients with pre-existing frequent episodes of hypoglycaemia or problems with hypoglycaemia awareness will be excluded from trial participation (section 6.2)	Common	
Outline any other processes that have been put in place to mitigate risks to participant safety (e.g. IDMC, independent data review, etc.)<br>See above				

16.2 Appendix 2: Contraception

In line with recommendations related to contraception and pregnancy testing in clinical trials from the Clinical Trial Facilitation Group (CTFG), for women of childbearing potential* (WOCBP) participating in TTT1, methods of contraception considered as highly effective are defined as those that can achieve a failure rate of less than 1% per year when used consistently and correctly.

Such methods include:

- combined (oestrogen and progestogen containing) hormonal contraception associated with inhibition of ovulation[1]
    - oral
    - intravaginal
    - transdermal
- progestogen-only hormonal contraception associated with inhibition of ovulation[1]
    - oral
    - injectable
    - implantable[2]
- intrauterine device (IUD) [2]
- intrauterine hormone-releasing system (IUS)[2]
- bilateral tubal occlusion[2]
- vasectomised partner[2,3]
- true sexual abstinence[4]

Footnotes

1. An interaction between dapagliflozin and semaglutide with contraception is not anticipated.
2. Contraception methods that in the context of this guidance are considered to have low user dependency.
3. Vasectomised partner is a highly effective birth control method provided that partner is the sole sexual partner of the WOCBP trial participant and that the vasectomised partner has received medical assessment of the surgical success.
4. In the context of this guidance true sexual abstinence refers to: When this is in line with the preferred and usual lifestyle of the patient. Periodic abstinence (e.g. calendar, ovulation, symptothermal, post-ovulation methods), declaration of abstinence for the duration of a trial, and withdrawal are not acceptable methods of contraception.
The reliability of sexual abstinence needs to be evaluated in relation to the duration of the clinical trial and the preferred and usual lifestyle of the subject.

*Child bearing potential = experienced menarche; AND not undergone successful surgical sterilisation (hysterectomy, bilateral tubal ligation, or bilateral oophorectomy); AND not post-menopausal i.e. amenorrhea for ≥12 consecutive months (without another medical cause)

Source: Recommendations related to contraception and pregnancy testing in clinical trials. Clinical Trial Facilitation Group (CTFG), 15th September 2014.
See: https://www.hma.eu/fileadmin/dateien/Human_Medicines/01-About_HMA/Working_Groups/CTFG/2014_09_HMA_CTFG_Contraception.pdf (Accessed 21st January 2020)

16.3 Appendix 3

STICH Protocol *(also provided in Period 2 Participant Diary)*

As explained above, participants in this trial randomised to dapagliflozin are at an increased risk of ketosis and ketoacidosis. All participants will have access to a ketone meter as standard of care. It will be ascertained that each participant has access to a ketone meter; if there are any issues with their current ketone meter it will be replaced as per standard of care. There are mechanisms designed to mitigate the risk of early ketosis developing further into diabetic ketoacidosis. In this trial, we will be using a protocol called STICH (37). This is a mnemonic designed to act as a reminder for participants as to what to do in the event of elevated ketones following exposure to an SGLT-2 drug such as dapagliflozin:

1. <u>S</u>Top dapagliflozin or placebo temporarily
2. <u>I</u>nsulin – take a bolus of rapid acting insulin
3. <u>C</u>arbohydrate – take approximately 30g of carbohydrate
4. <u>H</u>ydrate – make sure you are taking sips of water aiming to maintain hydration.

Further guidance will be provided in both the Patient Diaries supplied to all participants and in the Patient Alert Cards that participants will be asked to carry at all times. All participants will also be advised that in the event of acute intercurrent illness or hospitalisation, dapagliflozin or placebo should be temporarily stopped. This is because intercurrent illness may additionally increase the risk of ketosis. Participants will likely already be familiar with this concept of "sick-day rules" as this is a key component of basic education for people with type 1 diabetes in clinical practice. These rules will however be re-iterated to participants.

More detailed guidance on actions participants should take for any given level of ketosis will be provided during face-to-face and telephone contact during the trial. In addition, however, the following written protocol will be provided to participants to advise them explicitly of the safest and most appropriate course of action to be taken in any given circumstance. This will take the form of a traffic light system to aid understanding and compliance.

1. Ketones 0.6 mmol/L or less: Continue - no need for additional bolus insulin.
2. Ketones 0.6 – 1.4 mmol/L: consider taking additional fast acting insulin. Follow sick day rules and monitor ketones 2 hourly until they are 0.6 mmol/L or less.
3. Ketones 1.5 – 2.9 mmol/L – Take 10% of your total daily dose of insulin (this is found by adding up all the long acting and short acting insulin for the previous day - only take a maximum of 10 units) as a bolus of fast acting insulin. Keep hydrated - aim for 100ml water per hour. Re-test your glucose and ketones in 2 hours. If not falling contact the study team during office hours. If you need urgent advice at other times please contact NHS 24 (telephone number 111) or attend your nearest Accident & Emergency Department.
4. Ketones above 3 mmol/L – Take 20% of your total daily insulin as fast acting insulin (Maximum 10 units). Hydrate with water, aim for 500 ml per hour. Attend A&E – High risk of DKA

If you develop DKA or are unwell it is important that you let the TTT1 nurse or doctor know about this as soon as you can. By following this protocol, the risk of ketosis and developing ketoacidosis will be

mitigated.

16.4 Appendix 4: Amendment History

Amendment No.	Protocol version no.	Date issued	Author(s) of changes	Details of changes made
AM02	2.0			Protocol Changes: Weight loss mitigation, 19 Risk Mitigation and visit activity corrections
AM03	2.1			Correction of Summary of changes for previous AM02.<br>Corrected pregnancy testing schedule.
AM06	3.0			Definition of AESIs narrowed to include only ketosis and level 2 hypoglycaemia; lowering eGFR inclusion criterion; correction of DSQOLS to licensed version.

List details of all protocol amendments here whenever a new version of the protocol is produced. Protocol amendments must be submitted to the Sponsor for approval prior to submission to the REC committee or MHRA.

16.5 Appendix 5: PAID Questionnaire

Problem Areas In Diabetes (PAID) scale

Instructions: Which of the following diabetes issues are **currently** a problem for you? Tick the box that gives the best answer for you. Please provide an answer for each question.

		Not a problem	Minor problem	Moderate problem	Somewhat serious problem	Serious problem
1	Not having clear and concrete goals for your diabetes care?	☐0	☐1	☐2	☐3	☐4
2	Feeling discouraged with your diabetes treatment plan?	☐0	☐1	☐2	☐3	☐4
3	Feeling scared when you think about living with diabetes?	☐0	☐1	☐2	☐3	☐4
4	Uncomfortable social situations related to your diabetes care (e.g. people telling you what to eat)?	☐0	☐1	☐2	☐3	☐4
5	Feelings of deprivation regarding food and meals?	☐0	☐1	☐2	☐3	☐4
6	Feeling depressed when you think about living with diabetes?	☐0	☐1	☐2	☐3	☐4
7	Not knowing if your mood or feelings are related to your diabetes?	☐0	☐1	☐2	☐3	☐4
8	Feeling overwhelmed by your diabetes?	☐0	☐1	☐2	☐3	☐4
9	Worrying about low blood glucose reactions?	☐0	☐1	☐2	☐3	☐4
10	Feeling angry when you think about living with diabetes?	☐0	☐1	☐2	☐3	☐4
11	Feeling constantly concerned about food and eating?	☐0	☐1	☐2	☐3	☐4
12	Worrying about the future and the possibility of serious complications?	☐0	☐1	☐2	☐3	☐4
13	Feelings of guilt or anxiety when you get off track with your diabetes management?	☐0	☐1	☐2	☐3	☐4
14	Not 'accepting' your diabetes?	☐0	☐1	☐2	☐3	☐4
15	Feeling unsatisfied with your diabetes physician?	☐0	☐1	☐2	☐3	☐4
16	Feeling that diabetes is taking up too much of your mental and physical energy every day?	☐0	☐1	☐2	☐3	☐4
17	Feeling alone with your diabetes?	☐0	☐1	☐2	☐3	☐4
18	Feeling that your friends and family are not supportive of your diabetes management efforts?	☐0	☐1	☐2	☐3	☐4
19	Coping with complications of diabetes?	☐0	☐1	☐2	☐3	☐4
20	Feeling 'burned out' by the constant effort needed to manage diabetes?	☐0	☐1	☐2	☐3	☐4

© Joslin Diabetes Center, 1999 (www.joslin.org). All rights reserved. The copyright holder/developer has given permission for the questionnaire to be reproduced in this toolkit. Readers of the toolkit are permitted to reproduce the questionnaire for clinical use and non-commercial research purposes. Readers of the toolkit are not permitted to use the questionnaire for commercial research purposes and must seek permission from the copyright holder/developer to do so.

16.6 Appendix 6: DSQOLS Questionnaire

Diabetes specific quality of life scale (DSQOLS)

Date____________ Name____ DOB______________

Please tick the appropriate box for each question –

Which burdens and restrictions from diabetes and its treatment have you experienced during the last four weeks?...	This statement meets my point of view...					
	5. Perfectly	4. Quite good	3. A little	2. Rather not	1. Hardly	0. Not at all
1. It bothers me that I have to measure my blood glucose so often	☐	☐	☐	☐	☐	☐
2. It burdens me that I always have to think about my nutrition.	☐	☐	☐	☐	☐	☐
3. I suffer from pain because of diabetes.	☐	☐	☐	☐	☐	☐
4. Because of diabetes the relationship to my partner has become worse.	☐	☐	☐	☐	☐	☐
5. I am worried about the fact that my life could be shorter because of diabetes.	☐	☐	☐	☐	☐	☐
6. I have the impression that I am less attractive for others because of diabetes.	☐	☐	☐	☐	☐	☐
7. Because of diabetes I feel sad or depressed	☐	☐	☐	☐	☐	☐
8. I am worried about my future health	☐	☐	☐	☐	☐	☐
9. It is a burden for me how other people react to my diabetes	☐	☐	☐	☐	☐	☐
10. I feel nervous and restless when I think about episodes of low blood sugar						
11. I feel tired and exhausted	☐	☐	☐	☐	☐	☐
12. It bothers me that I have to spend so much time on my diabetes treatment	☐	☐	☐	☐	☐	☐
13. Because of diabetes traveling is complicated and troublesome	☐	☐	☐	☐	☐	☐
14. I get anxious and nervous when I think about the dangers of episodes of low blood glucose	☐	☐	☐	☐	☐	☐

	5. Perfectly	4. Quite good	3. A little	2. Rather not	1. Hardly	0. Not at all
15. Diabetes prevents me from spontaneous physical activities	☐	☐	☐	☐	☐	☐
16. I suffer from frequent urination	☐	☐	☐	☐	☐	☐
17. Because of high blood glucose values after a meal I often feel unwell or less efficient	☐	☐	☐	☐	☐	☐
18. Because of diabetes I feel anxious or threatened	☐	☐	☐	☐	☐	☐
19. I suffer from thirst or having a dry mouth	☐	☐	☐	☐	☐	☐
20. Other people find it hard to understand my problems with diabetes treatment	☐	☐	☐	☐	☐	☐
21. I feel physically unwell	☐	☐	☐	☐	☐	☐
22. I unnecessarily worry too much about episodes of low blood glucose	☐	☐	☐	☐	☐	☐
23. I have to give up good-tasting foods	☐	☐	☐	☐	☐	☐
24. I cannot eat as much as I want of certain foods	☐	☐	☐	☐	☐	☐
25. Because of diabetes I often have physical complaints	☐	☐	☐	☐	☐	☐
26. Diabetes interferes with my future plans	☐	☐	☐	☐	☐	☐
27. I get an uncomfortable feeling when I think about the dangers of a severe episode of low blood glucose	☐	☐	☐	☐	☐	☐
28. Because of diabetes I have less contact with friends or acquaintances	☐	☐	☐	☐	☐	☐
29. I wish I could eat more the way I want to, without having to plan everything beforehand	☐	☐	☐	☐	☐	☐
30. I suffer from frequent infections, itching or skin problems	☐	☐	☐	☐	☐	☐
31. I am concerned about getting into embarrassing situations because of episodes of	☐	☐	☐	☐	☐	☐

		5. Perfectly	4. Quite good	3. A little	2. Rather not	1. Hardly	0. Not at all
	low blood glucose						
32.	I feel weak or lazy	☐	☐	☐	☐	☐	☐
33.	I am dissatisfied with the amount of time I have to spend for medical consultations	☐	☐	☐	☐	☐	☐
34.	Because of my diabetes I cannot spend my free time the way I would like	☐	☐	☐	☐	☐	☐
35.	I feel like a disabled person	☐	☐	☐	☐	☐	☐
36.	I am worried that I could sustain physical injury in the event of a severe episode of low blood glucose	☐	☐	☐	☐	☐	☐
37.	It bothers me that I cannot eat like other people	☐	☐	☐	☐	☐	☐
38.	I am often worried about the long-term	☐	☐	☐	☐	☐	☐
39.	Because of diabetes other people treat me like a "sick person"	☐	☐	☐	☐	☐	☐
40.	Because of diabetes I cannot pursue my hobbies as I wish	☐	☐	☐	☐	☐	☐
41.	I have to frequently think about diabetes and its consequences	☐	☐	☐	☐	☐	☐
42.	It bothers me that I cannot eat as spontaneously as people who do not have diabetes	☐	☐	☐	☐	☐	☐
43.	It upsets my stomach when I think about the dangers of severe episodes of low blood glucose	☐	☐	☐	☐	☐	☐
44.	Diabetes constantly causes problems while dealing with other people	☐	☐	☐	☐	☐	☐

With respect to diabetes treatment it is very important to me that, resp. it would be my goal that ...	5. Very Important	4. Quite Important	3. Rather Important	2. Rather Unimportant	1. Quite Unimportant	0. Totally Unimportant
45. my blood glucose values are always below 140 mg%	☐	☐	☐	☐	☐	☐
46. my blood glucose values are as constant as possible	☐	☐	☐	☐	☐	☐
47. I can plan my leisure flexibly	☐	☐	☐	☐	☐	☐
48. I can avoid even mild hypoglycaemia	☐	☐	☐	☐	☐	☐
49. I can avoid late complications of diabetes in any case, resp. avoid their progress.	☐	☐	☐	☐	☐	☐
50. I can eat flexibly in the way that I can choose the kind, amount and time of meals	☐	☐	☐	☐	☐	☐
51. I am as physically fit as possible.	☐	☐	☐	☐	☐	☐
52. I can in any case avoid severe hypoglycaemia with loss of consciousness	☐	☐	☐	☐	☐	☐
53. I have to measure my blood glucose as rarely as possible.	☐	☐	☐	☐	☐	☐
54. Other people don't find out that I have diabetes,	☐	☐	☐	☐	☐	☐

How satisfied have you been during the last weeks with ...	0. Very Satisfied	1. Quite Satisfied	2. Rather Satisfied	3. Rather dissatisfied	4. Quite dissatisfied	5. Totally dissatisfied
55. the level of your blood glucose values?	☐	☐	☐	☐	☐	☐
56. The stability of your blood glucose values?	☐	☐	☐	☐	☐	☐
57. The flexibility of planning your leisure?	☐	☐	☐	☐	☐	☐
58. The frequency of mild hypoglycaemia?	☐	☐	☐	☐	☐	☐
59. your assurance of being protected against late complications, resp. of being able to avoid their progress?	☐	☐	☐	☐	☐	☐
60. The flexibility of your diet?	☐	☐	☐	☐	☐	☐
61. your physical fitness?	☐	☐	☐	☐	☐	☐
62. your assurance of being protected against severe hypoglycaemia with loss of consciousness?	☐	☐	☐	☐	☐	☐
63. the frequency of your blood glucose self-monitoring?	☐	☐	☐	☐	☐	☐
64. The extent to what other people are informed about your diabetes?	☐	☐	☐	☐	☐	☐

FULL/LONG TITLE OF THE TRIAL
Triple Therapy for Type 1 Diabetes with Insulin, Semaglutide and Dapagliflozin (TTT1) - UK

SHORT STUDY TITLE / ACRONYM
TTT1 (UK)

PROTOCOL VERSION NUMBER AND DATE
Version 4.0 12th June 2023

RESEARCH REFERENCE NUMBERS

**IRAS Number:**	254431
**EudraCT Number:**	2018-004120-11
**ISRCTN Number / Clinical trials.gov Number:**	NCT03899402
**SPONSOR Number:**	GN17DI706
**FUNDER Number:**	3-SRA-2019-669-M-B

SIGNATURE PAGE

The undersigned confirm that the following protocol has been agreed and accepted and that the Chief Investigator agrees to conduct the trial in compliance with the approved protocol and will adhere to the principles outlined in the Medicines for Human Use (Clinical Trials) Regulations 2004 (SI 2004/1031), amended regulations (SI 2006/1928) and any subsequent amendments of the clinical trial regulations, Good Clinical Practice (GCP) guidelines, the Sponsor's (Standard Operating Procedures (SOPs), and other regulatory requirements as amended.
I agree to ensure that the confidential information contained in this document will not be used for any other purpose other than the evaluation or conduct of the clinical investigation without the prior written consent of the Sponsor
I also confirm that I will make the findings of the study publicly available through publication or other dissemination tools without any unnecessary delay and that an honest accurate and transparent account of the study will be given; and that any discrepancies from the study as planned in this protocol will be explained.

For and on behalf of the Study Co-Sponsor (University of Glasgow):
Signature: .... Date: ....../....../......
Name (please print): ....
Position: ....

For and on behalf of the Study Co-Sponsor (NHS Greater Glasgow and Clyde):
Signature: .... Date: ....../....../......
Name (please print): ....
Position: ....

UK Chief Investigator:
Signature: .... Date: ....../....../......
Name: (please print): John Petrie ....
Position: Professor of Diabetes...

Statistician:
Signature: .... Date: ....../....../......
Name: (please print): ....

Position: ....

KEY TRIAL CONTACTS

UK Chief Investigator	Professor John R Petrie<br>Professor of Diabetes<br>Director, Robertson Centre for Biostatistics<br>Director, Glasgow Clinical Trials Unit<br>School of Health and Wellbeing<br>College of Medical, Veterinary and Life Sciences<br>University of Glasgow<br>Clarice Pears Buildiing<br>90 Byres Road<br>Glasgow<br>G12 8TB<br>Secretary +44(0)141 330 4744<br>john.petrie@glasgow.ac.uk
UK Co- Investigator	Dr James Boyle<br>Consultant Diabetologist<br>Clinical Lead NHS GG&C Diabetes MCN<br>Diabetes UK Clinical Champion<br>Glasgow Royal Infirmary<br>84 Castle Street, Glasgow, G4 0SF<br>Tel: : +44(0)141 211 5996<br>james.boyle@glasgow.ac.uk
Trial Co-ordination	Miss Lisa Jolly<br>Research and Innovation<br>NHS Greater Glasgow & Clyde<br>Ward 11, Dykebar Hospital<br>Grahamston Road<br>Paisley, PA2 7DE<br>Lisa.Jolly@ggc.scot.nhs.uk
Co-Sponsor	NHS Greater Glasgow & Clyde<br><br>Contact: Dr Maureen Travers<br>Research and Development Management Office<br>Research and Innovation<br>NHS Greater Glasgow & Clyde<br>Ward 11, Dykebar Hospital<br>Grahamston Road<br>Paisley, PA2 7DE<br>Tel: +44 (0) 0141 314 4012<br>maureen.travers@ggc.scot.nhs.uk
Co-sponsor	University of Glasgow<br><br>Contact: Dr Debra Stuart<br>College of Medical, Veterinary & Life Sciences<br>Room 327 Wolfson Medical School Building

	University Avenue<br>Glasgow G12 8QQ<br>Tel: +44 (0)141 330 4539<br>Debra.Stuart@glasgow.ac.uk
Funder	JDRF<br>26 Broadway, 15th floor, New York, NY 10004, USA
Clinical Trials Unit	Glasgow Clinical Trials Unit<br>Robertson Centre for Biostatistics, 11th Floor,<br>Boyd Orr Building, University of Glasgow<br>G12 8QQ<br>Tel: 0141 330 4744<br>liz.anderson@glasgow.ac.uk
Statistician	Professor Alex McConnachie<br>Robertson Centre for Biostatistics, 11th Floor, Boyd Orr Building, University of Glasgow, Glasgow, G12 8QQ<br>Telephone: 0141 330 4744<br>Fax: 0141-330-5094
Trials pharmacist	Dr Elizabeth Douglas<br>Clinical Trials Pharmacist<br>Clinical Research and Development<br>Ward 11, Dykebar Hospital,<br>Grahamston Road | Paisley PA2 7DE<br>Tel: 0141 314 4073<br>E-mail: elizabeth.douglas@ggc.scot.nhs.uk

US Principal Investigator	Dr Paresh Dandona<br>Director, Diabetes-Endocrinology Center of Western NY<br>Chief of Endocrinology, Diabetes, Endocrinology and Metabolism, SUNY at Buffalo<br>1000 Youngs Road, Suite 105<br>Williamsville NY 14221<br>Telephone: +1(716) 535-1850<br>Fax: +1(716)-639-1200<br>pdandona@kaleidahealth.org
US Co- Investigator	Dr Husam Ghanim, PhD.<br>Director, Diabetes and Endocrinology Research Lab<br>Research Associate Professor,<br>School of Medicine, State University of NY at Buffalo<br>Clinical and Translational Research Center

	875 Ellicott St<br>Buffalo, NY 14203<br>Tel: (+1)716-881-8924<br>ghanim@buffalo.edu
Key Protocol Contributors	Professor John Petrie<br>john.petrie@glasgow.acuk<br><br>Dr Paresh Dandona<br>pdandona@kaleidahealth.org<br><br>Husam Ghanim, PhD.<br>ghanim@buffalo.edu<br><br>Dr James Boyle<br>james.boyle@glasgow.ac.uk<br><br>Dr Robert Lindsay<br>robert.lindsay@glasgow.ac.uk<br><br>Dr Joseph Timmons<br>joseph.timmons@glasgow.ac.uk<br><br>Professor Ian Ford<br>ian.ford@glasgow.ac.uk<br><br>Professor Alex McConnachie<br>Alex.McConnachie@glasgow.ac.uk

TRIAL SUMMARY

Trial Title	Triple Therapy for Type 1 Diabetes with Insulin, Semaglutide, and Dapagliflozin (UK)	
Internal ref. no. (or short title)	TTT1	
Clinical Phase	Phase 3	
Trial Design	Two six-month periods: (i) open-label randomised (2:1) parallel group semaglutide vs standard of care; (ii) additional double-blind, randomised (1:1) parallel group, dapagliflozin vs placebo for those participants initially randomised to semaglutide.<br>Regardless of each randomisation, ongoing standard of care (insulin therapy) will be provided for all participants throughout the study.	
Trial Participants	Adults with type 1 diabetes for at least one year with HbA1c 7.5-11.0% on stable multiple daily injections (MDI) (four or more) of insulin or continuous subcutaneous insulin infusion (CSII) for the previous three months.	
Planned Sample Size	114 (50 in UK)	
Treatment duration	Approximately 1 year	
Follow up duration	Approximately 1 year	
Planned Trial Period	Approximately 5 years	
	Objectives	Outcome Measures
Primary	To assess in people with type 1 diabetes whether triple therapy (dapagliflozin, semaglutide and insulin) reduces HbA1c in comparison with dual therapy (placebo, semaglutide, and insulin – period 2).	HbA1c
Secondary	To assess in people with type 1 diabetes whether triple therapy (dapagliflozin, semaglutide and insulin) reduces HbA1c in comparison with standard therapy (insulin alone)	HbA1c
	To assess in people with type 1 diabetes whether dual therapy (semaglutide and insulin - period 1) reduces HbA1c in comparison with standard therapy (insulin alone)	HbA1c
	To assess in people with type 1 diabetes whether triple therapy (dapagliflozin, semaglutide and insulin) improves glucose control	- Mean fasting blood glucose<br><br>- Mean blood glucose*<br>- Percentage time in range

	as assessed by CGM, insulin requirement, body weight, blood pressure and quality of life in comparison with (a) dual therapy (placebo, semaglutide, and insulin – period 2) and (b) standard therapy (insulin alone)	- Glycaemic variability*<br>- Percentage time in hyperglycaemia Level 1 (10.1-13.9 mmol/)*<br>- Percentage time in hyperglycaemia Level 2 (>13.9 mmol/L)*<br>-Percentage time in hypoglycaemia Level 1 (3.0 – 3.8 mmol/L)*<br>** CGM (by masked Dexcom G6)*<br><br>Insulin requirement<br>Body weight<br>Systolic/ diastolic blood pressure<br>Number of antihypertensive agents<br>Diabetes Specific Quality of Life Scale (DSQOLS)<br>Problem Areas In Diabetes (PAID) questionnaire
Safety Endpoint 1	a) Triple therapy (dapagliflozin, semaglutide and insulin) in comparison with dual therapy (placebo, semaglutide, insulin – period 2)<br><br>b) Dual therapy (semaglutide and insulin - period 1) in comparison with standard therapy (insulin only)	Difference between treatment arms in:<br>(i) Rate of Level 2 (<3.0 mmol/L) hypoglycaemic events (by Dexcom G6 CGM)<br>(ii) Rate of Level 3 hypoglycaemic events (severe event with altered mental and/or physical status requiring assistance)
Safety Endpoint 2	a) Triple therapy (dapagliflozin, semaglutide and insulin) in comparison with dual therapy (placebo, semaglutide, and insulin – period 2)<br><br>b) Dual therapy (semaglutide and insulin –period 1) in comparison with standard therapy (insulin only)	Difference between treatment arms in:<br>Rate of diabetic ketoacidosis (serum ketones or urinary ketones above normal limits AND serum bicarbonate < 15 mmol/L OR blood pH < 7.3)
Investigational Medicinal Product(s)	Semaglutide<br>Dapagliflozin<br>Placebo matching dapagliflozin	
Non-investigational Medicinal Product(s)	Insulin	
Formulation, Dose, Route of Administration	Semaglutide (subcutaneous injection, once weekly) – open label by pen device (0.25 mg/ 0.5 mg/ 1.0 mg)<br>Dapagliflozin 5 mg (oral) – one then two capsules daily (up-titration)<br>Placebo matching dapagliflozin	

FUNDING AND SUPPORT IN KIND

FUNDER(S)	FINANCIAL AND NON FINANCIALSUPPORT GIVEN
JDRF New York<br>26 Broadway, 15th floor, New York, NY 10004, United States	$1,440,000 (to University at Buffalo, the State University of New York, NY (SUNY Buffalo), USA; and University of Glasgow, UK)
Novo Nordisk, 800 Scudders Mill Road, Princeton Landing, NJ, Plainsboro, 08536.	$160,000 (see paragraph headed "Novo Nordisk" below)
AstraZeneca<br>1800 Concord Pike, Wilmington, DE 19803, USA	Provision of dapagliflozin and matched placebo investigational medicinal products to US site
Dexcom; Suite 6, 1 Tanfield, Edinburgh, EH3 5DA	Staff training and discounted supply of CGM sensors

ROLE OF STUDY SPONSOR AND FUNDER

NHS Greater Glasgow & Clyde and The University of Glasgow will be Co-sponsors of the UK arm of the trial. Prior to study initiation, a non-commercially funded clinical trial co-sponsorship agreement will be put in place between NHS Greater Glasgow & Clyde and The University of Glasgow. The roles and liabilities each organisation will take under The Medicines for Human Use (Clinical Trials) Regulations, 2004 SI 2001:1031 are laid out in this agreement signed by both organisations. The University of Glasgow shall be responsible for carrying out the obligations and responsibilities set out in the aforementioned agreement, and shall be deemed "sponsor" for the purposes of, Part 3 of the regulations in relation to the study. NHS Greater Glasgow & Clyde shall be responsible for carrying out the responsibilities set out in the agreement, and shall be deemed "sponsor" for the purposes of, Parts 4, 5, 6 and 7 of the Regulations in relation to the study.

The Co-Sponsors will delegate specific roles to the Chief Investigator, Glasgow CTU and other third parties. These arrangements will be clearly documented in agreements and/or the Sponsor Delegated Roles and Responsibilities Matrix.

The US arm of the trial has the same inclusion and exclusion criteria and the same pre-specified outcomes but an individualised Protocol. It will collect the same data via a common eCRF. Adverse event data from each site will be available to the trial Data and Safety Monitoring Board. The UK Co-Sponsors will have no responsibility for (and carry no liability for) the conduct of the US arm of the trial.

<u>JDRF</u>
The study has been funded by a grant from JDRF. JDRF does not have a designated role or responsibility in trial design, conduct, data analysis and interpretation, manuscript writing, and dissemination of results. Periodic reports (at least annual) in relation to progress of the trial will be submitted to the JDRF. Support from JDRF will be acknowledged in any publications related to the study.

<u>Astra Zeneca</u>
This is an investigator-initiated study. Astra Zeneca provided support by supplying investigational medicinal products (IMPs) dapagliflozin and matched placebo to the US site only. Representatives of

Astra Zeneca reviewed and provided comments on drafts of the Protocol but the company does not have a designated role or responsibility in trial design, conduct, data analysis and interpretation, manuscript writing, and dissemination of results.

Novo Nordisk

In November 2020, Novo Nordisk made up a shortfall of funding that resulted from a cut back by 10% of the main grant by the funder JDRF due to the COVID-19 pandemic. By this time, the Protocol had been finalised. The company does not have a designated role or responsibility in trial design, conduct, data analysis and interpretation, manuscript writing, or dissemination of results.

Supply shortages of semaglutide (various strengths) in 2022 resulted in actual and potential disruption to participant recruitment and ongoing treatment. In response to a direct request from the UK Chief Investigator, Novo Nordisk donated supplies of semaglutide to the Glasgow site based on anticipated use during the period of disruption.

ROLES AND RESPONSIBILITIES OF TRIAL MANAGEMENT COMMITTEES/GROUPS & INDIVIDUALS

Trial Steering Committee (TSC)

The role of the TSC is to provide overall supervision of the trial and ensure that it is being conducted in accordance with the principles of GCP and the relevant regulations. The TSC will:

1. agree the trial protocol and any protocol amendments
2. ensure that conduct of the trial is fully harmonised between the two sites
3. provide advice to the investigators on all aspects of the trial

It will have an independent Chairperson and include the US and UK investigators and key protocol contributors listed above.

Decisions about continuation or termination of the trial or substantial amendments to the protocol will be the responsibility of the TSC who will advise the co-sponsors. The TSC will meet at the start of the study, and four to six monthly thereafter. The TSC will have its own Charter outlining the role and responsibilities of its members. The TSC may invite other attendees from the trial team to present or participate in discussions on particular topics. These attendees will be non-voting members.

Independent data monitoring committee (IDMC)

An IDMC will be established to include two independent medical experts (covering the domains of type 1 diabetes and metabolic disease; one of the academic clinicians will act as chair) and an independent biostatistician. The Glasgow CTU will liaise with the committee and ensure that the Committee is provided with adequate information about study progress and results.

The IDMC will have a formal Charter outlining the responsibilities of the IDMC members, Glasgow CTU and the co-sponsors. Responsibilities include:

- To protect the safety of participants recruited to the trial.
- Advising the TSC and co-sponsors if it is safe and appropriate to continue with the study
- Examining information provided by the Glasgow CTU on study recruitment, adverse events and outcomes
- Providing reports for the Project Office to forward to the TSC, ethics committees, regulatory bodies, study co-sponsors, and the BHF.

The IDMC may recommend to the TSC to terminate the study or specific aspects of the study if clear harm is observed with one treatment option compared to standard of care or placebo. Specific safety endpoints that will be reviewed include rates of hypoglycaemia and ketosis/ ketoacidosis. The IDMC will not formally review efficacy or futility.

The IDMC will meet approximately every six months.

Study Management Group (SMG)

Trial activity at the UK/ Glasgow site will be coordinated from NHS Greater Glasgow & Clyde (NHS GG&C) by the TTT1 Study Management Group (SMG). The SMG will consist of the UK chief investigator, co-principal investigator other co-applicants, project manager and representatives from the Glasgow CTU, NHS GG&C and The University of Glasgow. The role of the group is to monitor all aspects

of the conduct and progress of the trial in the UK, ensuring adherence to the protocol, and take appropriate action to safeguard participants and the quality of the trial itself.

Joint Study Management Group

Coordination of trial activity between the UK/ Glasgow and the US/ Buffalo site will be facilitated by the Joint TTT1 Study Management Group. This will consist of the US and UK chief investigators, co-principal investigators, other co-applicants, study coordinators, project managers and governance/ administrative representatives as appropriate from State University of New York (SUNY), the Glasgow CTU, NHS GG&C and The University of Glasgow. It will meet approximately every 4-6 weeks. The role of the group is to coordinate all aspects of the conduct and progress of the trial between the US and UK sites, ensuring protocol adherence, and taking appropriate action to safeguard participants and the quality of the trial itself.

Protocol contributors

The protocol has been developed by a group with extensive clinical and research experience in type 1 diabetes and clinical trials including Professor John Petrie and Professor Paresh Dandona. Professor Ian Ford and Professor Alex McConnachie have research expertise in design, conduct, analysis and interpretation of clinical trials and biostatistical methods.

KEY WORDS:

Adjunct therapy
Dapagliflozin
Glycated haemoglobin (HbA1c)
Semaglutide
Type 1 diabetes

LIST of CONTENTS

RESEARCH REFERENCE NUMBERS 2
SIGNATURE PAGE 3
KEY TRIAL CONTACTS 4
LIST of CONTENTS..14
LIST OF ABBREVIATIONS...15
TRIAL FLOW CHART...17
1 BACKGROUND 20
2 RATIONALE...21
3 OBJECTIVES AND OUTCOME MEASURES/ ENDPOINTS 25
4 TRIAL DESIGN 28
5 STUDY SETTING 28
6 ELIGIBILITY CRITERIA 28
7 TRIAL PROCEDURES..30
8 TRIAL MEDICATION…………………………………………………………………..58
9 PHARMACOVIGILANCE………………………………………………………………63
10 STATISTICS AND DATA ANALYSIS...70
11 DATA HANDLING 71
12 MONITORING, AUDIT & INSPECTION 73
13 ETHICAL AND REGULATORY CONSIDERATIONS..73
14 DISSEMINATION POLICY……………………………………………………………77
15 REFERENCES…………………………………………………………………………..78
16. APPENDICES...81

LIST OF ABBREVIATIONS

ACS	Acute Coronary Syndrome
AUC	Area Under the Curve
AE	Adverse Event
AR	Adverse Reaction
CA	Competent Authority
CABG	Coronary Artery Bypass Graft
CGM	Continuous Glucose Monitoring
CHI	Community Health Index
CI	Chief Investigator
CRO	Contract Research Organisation
CRP	C-Reactive Protein
CSII	Continuous Subcutaneous Insulin Infusion
CTA	Clinical Trial Authorisation
CTU	Clinical Trials Unit
CTIMP	Clinical Trial of Investigational Medicinal Product
CV	Cardiovascular
CVA	Cerebrovascular Accident
DKA	Diabetic Ketoacidosis
DSUR	Development Safety Update Report
EC	European Commission
ECG	Electrocardiogram
eCRF	Electronic Case Report Form
EDTA	Ethylenediaminetetraacetic acid
eGFR	Estimated Glomerular Filtration Rate
EMEA	European Medicines Agency
EU	European Union
EUCTD	European Clinical Trials Directive
EudraCT	European Clinical Trials Database
EudraVIGILANCE	European database for Pharmacovigilance
FBC	Full Blood Count
FGM	Flash Glucose Monitoring
GCP	Good Clinical Practice
GDG	Guideline Development Group
GDPR	General Data Protection Regulation
GLP-1	Glucagon Like Peptide-1
GLP-1RA	Glucagon Like Peptide-1 Receptor Agonist
GMP	Good Manufacturing Practice
HbA1c	Glycated haemoglobin
IB	Investigator Brochure
ICF	Informed Consent Form
ICH	International Conference on Harmonisation of technical requirements for registration of pharmaceuticals for human use.
IDMC	Independent Data Monitoring Committee
IMP	Investigational Medicinal Product
IMPD	Investigational Medicinal Product Dossier
ISF	Investigator Site File
ISRCTN	International Standard Randomised Controlled Trials Number

IWRS	Interactive Web-based Randomisation System
LPLV	Last Patient Last Visit
MA	Marketing Authorisation
MDRD	Modification of Diet in Renal Disease
MODY	Maturity Onset Diabetes of the Young
MHRA	Medicines and Healthcare products Regulatory Agency
MI	Myocardial Infarction
MDI	Multiple Daily Injections
NHS GG&C	National Health Service Greater Glasgow & Clyde
NHS R&D	National Health Service Research & Development
NICE	The National Institute for Health and Care Excellence
NIMP	Non-Investigational Medicinal Product
PI	Principal Investigator
PIC	Participant Identification Centre
PIS	Participant Information Sheet
PV	Pharmacovigilance
QA	Quality Assurance
QC	Quality Control
QoL	Quality of Life
QP	Qualified Person
RCT	Randomised Control Trial
REC	Research Ethics Committee
SAE	Serious Adverse Event
SAP	Statistical Analysis Plan
SAR	Serious Adverse Reaction
SDV	Source Data Verification
SGLT2	Sodium Glucose Transporter 2
SOP	Standard Operating Procedure
SUNY	State University of New York
SmPC	Summary of Product Characteristics
SSI	Site Specific Information
SUSAR	Suspected Unexpected Serious Adverse Reaction
TIA	Transient Ischaemic Attack
TMG	Trial Management Group
TSC	Trial Steering Committee
TMF	Trial Master File
UB	University of Buffalo
ULN	Upper Limit of Normal reference range
WOCBP	Women Of Childbearing Potential

TRIAL FLOW CHART

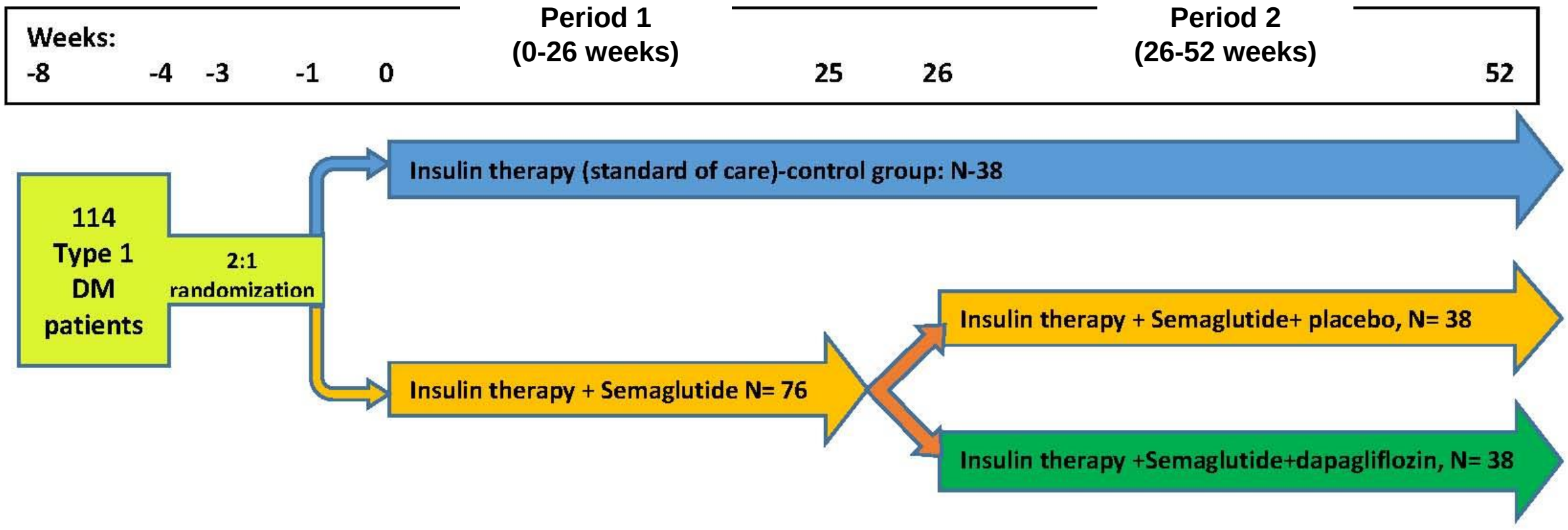

SCHEDULE OF ASSESSMENTS

Visit #	-	1	2	3	4[3]	5[3]	6	7	8	9	10	11[3]	12	13	14	15	16
**Week# (±7 days; see below)**	**-8 to -4**	**-3**	**-1**	**0**	**1**	**2**	**4**	**13**	**23**	**25**	**26**	**27**	**31**	**39**	**49**	**51**	**52**
Study Procedures	Screening	Run-in period		Randomise							Randomise						End
Informed Consent	X																
Fasting visit			X	X				X		X	X			X		X	X
History and demographics	X																
Physical	X																X
Education on hypoglycaemia and ketosis avoidance)				X							X						
BP, heart rate, weight	X	X	X	X	X	X	X	X	X	X	X	X	X	X	X	X	X
Bloods (FBC, kidney and liver profiles)	X			X				X			X		X	X			X
C-peptide	X										X						X
Pregnancy test	X			X			X[1]	X[1]	X[1]		X[1]		X[1]	X[1]	X[1]	X[1]	X[1]
Dispense open-label semaglutide[2]				X		X[2]	X[2]	X[2]			X[2]			X[2]			
Titrate open-label semaglutide[2]						X[2]	X[2]	X[2]									
Dose Semaglutide (on-site if dose is due on visit day)				X	X	X											
Dispense dapagliflozin/placebo											X	X		X			
Dapagliflozin/placebo return capsule counts												X		X			X
HbA1c	X			X				X		X				X			X
Blood endpoints (including fructosamine)				X				X			X			X			X
Masked CGM fit (enter data this visit)		X							X						X		
Masked CGM read				X							X						X
Daily fingerstick glucose review		X	X	X	X	X	X	X	X	X	X	X	X	X	X	X	X
Daily urinary ketones review												X	X	X	X	X	X
Insulin dose review		X	X	X	X	X	X	X	X	X	X	X	X	X	X	X	X
Ketone bodies (blood/urine)	X	X	X	X	X	X	X	X	X	X	X	X	X	X	X	X	X
Adverse Event reporting		X	X	X	X	X	X	X	X	X	X	X	X	X	X	X	X
Meal Challenge Test			X							X						X	
24 hr Urine dispensed			X				X			X			X			X	
24 hr Urine collected				X				X			X			X			X
Quality of life questionnaires				X							X						X

N.B. Safety phone calls will be conducted at weeks 3, 5, 8, 16, 20, 29, 35, 44 and at two weeks after the end of the study to document any side effects or other changes. **Abbreviations:** see pages 14-15
Additional visits or phone calls may be scheduled with the verbal consent of the participant if study team believe necessary e.g. closer monitoring is appropriate (see Patient Information Sheet)
Visit dates should be within seven days of the visit time; however, a longer or shorter interval does not constitute a protocol deviation
[1]Not required in women randomised to "Insulin therapy (standard of care)
[2]According to randomisation schedule: resupply of open-label semaglutide after Visit 3 will be according to individual requirements as tolerated (see Section 8.3)
[3] Visits 4, 5 and 11 have the option of being carried out by telephone for those participants allocated to the standard care arm only.

STUDY PROTOCOL
Triple Therapy for Type 1 Diabetes with Insulin, Semaglutide, and Dapagliflozin

1 BACKGROUND

The control of glucose homeostasis in type 1 diabetes is fragile since β-cell reserve is negligible. Traditionally, insulin has been the only treatment approach available. This often leads to erratic blood glucose control given the challenge of matching exogenous insulin to mealtime glucose excursions in the face of a lack of significant endogenous insulin to compensate for variation in requirements in response to meals and activity levels. Injected insulin boluses rarely match precise insulin requirements either in terms of dose administered or bio-availability. The result often is wide fluctuations in blood glucose including episodes when it is too low (hypoglycaemia). Based on our work to date and other published reports, previous attempts to add other glucose-lowering medications to insulin therapy yielded minimal benefits on glycaemia (although metformin may have other benefits) (1-3).

Dandona et al. have shown in two preliminary retrospective studies that the addition of liraglutide, a Glucagon Like Peptide -1 receptor agonist (GLP-1RA), to insulin results in consistent improvements in glycemic control in type 1 diabetes as demonstrated by reductions in HbA1c, glycemic excursions, and insulin requirements accompanied by improvements in body weight and systolic blood pressure (4, 5). As collaborators in the present trial, they also completed the first prospective randomised controlled trial of this drug in 72 people with type 1 diabetes. The data not only confirmed improvements in glycemic control (HbA1c), body weight and systolic BP, but also demonstrated a reduction in appetite, carbohydrate intake and low-grade inflammation (6). Importantly, quality of life was also improved. Their recent studies in type 2 diabetes have revealed that systolic blood pressure reduction with GLP-1RA (exenatide) treatment in type 2 diabetes is associated with increased plasma concentrations of ANP, cGMP and cAMP along with reduced concentrations of angiotensinogen, renin and angiotensin II (7). It is possible that these mechanisms play a role in the prevention of cardiovascular complications in type 1 diabetes. There have been no trials to date of semaglutide (a weekly and potent GLP-1RA) in type 1 diabetes.

GLP-1RAs, therefore, have great potential as adjunct therapy for type 1 diabetes. However, mean HbA1c was still ≥7.0% after liraglutide treatment (6) and larger pharma-sponsored trials (ADJUNCT-1 and ADJUNCT-2) reported increased rates of hypoglycaemia (8, 9). In seeking further improvement, the advent of Sodium-GLucose coTransporter 2 (SGLT2) inhibitors is of importance since their mode of action is independent of insulin secretion. Introduced for use in type 2 diabetes, SGLT2 inhibitors are selective antagonists of the sodium-glucose transporter subtype expressed in the proximal convoluted tubules of the kidneys. They act by inhibiting renal glucose re-absorption and therefore stimulating excretion of 60-100 grams per day of glucose in urine. SGLT2 inhibitors have been studied extensively in type 2 diabetes, including the landmark cardiovascular outcome trial EMPA-REG (10), but data have only more recently begun to accumulate in type 1 (11). Short term effects on urine glucose excretion of remogliflozin and empagliflozin are similar to those seen in type 2 diabetes and these translate into improved glycaemic and other metabolic indices including HbA1c, fasting blood glucose, rates of hypoglycaemia, daily insulin dose and weight (12). Glucose-lowering efficacy is attenuated in individuals with eGFR < 45/ml/min/1.73 m^2.(13) Weight loss accompanying increased urinary glucose excretion could translate into reduced cardiovascular complications as in type 2 diabetes, but there are no data so far available to support this hypothesis. More recently, in the Phase 3 trials DEPICT-1 and DEPICT-2 Dandona et al (PI of the US arm of this trial) showed that dapagliflozin improves glycaemic control and HbA1c over six months by about 0.45% without increasing hypoglycaemia and as in other conditions reduces urinary albumin excretion (14,15,16,17). In comparison to other SGLT2 inhibitors studied for use in type 1 diabetes [sotagliflozin and empagliflozin (18, 19)] the risk of diabetic ketoacidosis was relatively modest (see below). Both dapagliflozin and sotagliflozin were approved early in 2019 by the EU Committee for Medicinal Products for Human Use (CHMP) as adjunct therapy in type 1 diabetes for individuals with BMI≥27 kg/m (20,21)2. In August 2019, dapagliflozin (5 mg/ day) was recommended by the National Institute for Health and Care Excellence as cost-effective for this indication in NHS England (22 However, dapagliflozin did not gain approval for this indication in the

USA from the FDA (23). In November 2021, the manufacturer AstraZeneca voluntarily removed this Europe- and UK-specific indication (24) explicitly “not due to any safety concern for dapagliflozin in any condition, including type 1 diabetes” (25).

2 RATIONALE

General hypothesis: adding an SGLT2 inhibitor (dapagliflozin) to a combination of insulin and semaglutide will improve glycaemic control, weight and blood pressure in adults with type 1 diabetes.
Our protocol is designed to allow an objective assessment of whether this triple therapy combination will work effectively and safely so that a majority of people with type 1 diabetes can reach a glycaemic target HbA1c of ≤7.0%. In addition, since both semaglutide and dapagliflozin inhibitors reduce blood pressure and weight, it will allow an assessment of the combined effect of GLP-1RAs and SGLT2 inhibitors on these outcomes. Since there are no previous data on the effects of once weekly semaglutide in type 1 diabetes, or the combination of semaglutide and dapagliflozin compared to standard insulin treatment, the design requires allocation of some participants to insulin therapy only (the current standard of care).

As part of the process by which this trial was awarded funding by JDRF, this research question was considered by lay members of the funding panel and deemed important, relevant and timely. We considered that use of placebo matching dapagliflozin in Period 2 of the trial was justified when dapagliflozin had full approval as adjunct therapy for type 1 diabetes in Europe (although not the USA) as it remained uncertain which adults with type 1 diabetes were suitable for this treatment. In their review, NICE considered it likely that fewer than 10% of adults with type 1 diabetes would be initiated on SGLT2 inhibitor therapy over the first five years after their favourable decision. As individuals with a slightly lower BMI threshold will be included than recommended by the SmPC, trial participants will be more closely monitored for ketosis than was the case when dapagliflozin was approved for routine clinical care. Dapagliflozin (or placebo) will be administered orally once daily as per current licensed doses in type 2 diabetes (5 mg/day and 10 mg/day: the same doses as used in DEPICT-1 and DEPICT-2) (14,15,16,). We have not incorporated blinding for semaglutide in the design as the effects of the closely-related daily GLP-1RA liraglutide have been well-studied in double-blind trials in type 1 diabetes by ourselves and others, and individuals in both arms compared for the primary outcome comparison will be treated with this agent. (6, 8, 9). Semaglutide will be administered subcutaneously once weekly. The need to study combination adjunct therapy with SGLT 2 inhibitors and GLP-1 RAs is greater than ever following removal of the type 1 diabetes indication for SGLT2 inhibitor monotherapy in the UK and Europe.

2.1 Assessment and management of risk

a) Benefits

Semaglutide (Ozempic): is a once weekly injectable (subcutaneous) GLP1-RA licensed for reducing HbA1c in type 2 diabetes. The licensed doses are 0.25 mg weekly, 0.5 mg weekly and 1.0 mg weekly. In the trial, the starting dose is 0.25 mg weekly uptitrated if tolerated to 0.5 mg weekly after two weeks and then (again if tolerated) after a further two weeks to the maximum dose of 1.0 mg weekly. Semaglutide differs from other weekly GLP-1 receptor agonists in that its half-life is extended by binding to serum albumin i.e. it is not macromolecular. In type 2 diabetes, semaglutide therapy is associated with weight reduction and a reduction in rates of cardiovascular events (26). The licensed dose escalation schedule for routine treatment of type 2 diabetes is at four weekly intervals (27) but in a Phase 2 trial in type 2 diabetes authored by one of this trial's investigators (JP) doses were uptitrated at one-to-two weekly intervals and to higher doses (up to 1.6 mg weekly) (28). Phase 3 trials are currently in progress (for the indication of obesity) using a 2.4 mg semaglutide weekly. It should be noted that semaglutide 0.25 mg is considered too low a dose for maintenance therapy in type 2 diabetes but it is unclear whether this may have a beneficial effect in type 1 diabetes at this dose.

While semaglutide once weekly has not been investigated previously in type 1 diabetes, liraglutide (a GLP-1 RA for daily administration with a similar molecular structure that binds less avidly to serum albumin) has previously been investigated (see above) in the ADJUNCT 1 and ADJUNCT 2 trials (8,9) in which, compared with placebo it resulted in reductions in HbA1c, weight and insulin requirement, albeit with a small increase in rates of hypoglycaemia (see below).

Dapagliflozin (Forxiga): is a once daily oral SGLT2 inhibitor previously licensed in Europe for reducing HbA1c in people with type 1 diabetes and BMI ≥ 27 kg/m^2 when insulin alone does not provide adequate glycemic control despite optimal insulin therapy (23). Although only the 5 mg dose was licensed for type 1 diabetes,both 5 mg and 10 mg daily are licensed for type 2 diabetes (29). As mentioned above, the removal of the type 1 diabetes indication by AstraZeneca was not due to any safety concern for any indication, including type 1 diabetes. In the Phase 3 trials of dapagliflozin in type 1 diabetes (DEPICT-1 and DEPICT-2) both doses were used. In both DEPICT-1 and DEPICT-2 there were modest additional benefits with 10 mg compared to 5 mg dapagliflozin. For example, in DEPICT-1, insulin dose requirement was reduced by 13.2% vs placebo on 10mg daily compared with 8.8% on 5mg daily (14). Weight reduction vs placebo in DEPICT-1 at 52 weeks was by 4.5% on dapagliflozin 10mg daily compared with 2.95% in those receiving 5 mg daily. Given the propensity of insulin to encourage weight gain over time and the life-long insulin requirement in T1D, sustained weight reduction and insulin dose reduction - even when modest - may be clinically important. There was no increase in the rate of hypoglycaemia in either the 5 mg or 10 mg groups compared with placebo. (14,15)

Dapagliflozin therapy in type 2 diabetes has been demonstrated to be associated with cardiovascular and renal risk reduction: in DECLARE-TIMI, DAPA-HF and DAPA-CKD the 10 mg dose was used (30, 31, 32). Such cardiovascular and renal benefits may translate to type 1 diabetes with appropriate monitoring and risk mitigation, although support for such measures by the manufacturer were withdrawn in the UK at the time of the removal of the type 1 indication . Given these potential benefits with relatively low increased risk, the 10 mg rather than 5 mg dose was selected for steady state treatment in this protocol.

b) Risks/ side effects Please also refer to the respective UK SmPCs (27, 29)

Semaglutide:
Very common: nausea, diarrhoea; hypoglycaemia (when used with insulin).
Common: vomiting, abdominal pain, constipation, gastro-oesophageal reflux, cholelithiasis, weight loss.
Uncommon: injection site reactions, hypersensitivity
Rare: Anaphylactic reaction.

Hypoglycaemia/ ketosis: Semaglutide once weekly has not been studied previously in type 1 diabetes; however, the ADJUNCT-1 and ADJUNCT-2 trials examined the role of liraglutide once daily (a similar molecule in the GLP-RA class that binds less avidly to serum albumin). Although HbA1c and weight were reduced by both 1.2 mg and 1.8 mg liraglutide, rates of symptomatic hypoglycaemia increased by 20-30%. In ADJUNCT-1, insulin doses were re-uptitrated quite rapidly towards baseline levels after an initial dose reduction, but in ADJUNCT-2 were “capped” (and therefore remained lower). Perhaps for that reason, an increase in hypoglycaemia was less consistent in ADJUNCT -2 but there was an excess of ketosis the higher (1.8 mg) dose, presumably due to under-insulinisation (no lower limit for insulin dose reduction was set). It should be noted that monitoring of participants by site investigators in the ADJUNCT trials was less intensive than will be the case in the present trial with less frequent opportunities for insulin dose titration (see Section 7). Importantly, participants at high risk of hypoglycaemia, (including those with a history of severe hypoglycaemia and those with hypoglycaemia unawareness) were not excluded (8,9).

Retinopathy: In the SUSTAIN-6 cardiovascular outcome trial of semaglutide in type 2 diabetes, rapid reduction of HbA1c was associated with progression of existing proliferative retinopathy in those with a high baseline HbA1c (“early worsening”/ “normoglycaemic re-entry”). However, there was no evidence of worsening of retinopathy on treatment in the trials SUSTAIN 1-5 in which those with proliferative retinopathy were excluded (33, 3). In clinical trials of duration up to one year involving 4,807 patients with type 2 diabetes, adverse events related to diabetic retinopathy were reported in similar proportions of subjects treated with semaglutide (1.7%) and comparators (2.0%) (33).

Other adverse effects: In type 2 diabetes, gastrointestinal adverse effects of semaglutide can be prevented in the majority of cases by careful dose uptitration (28, 34) although up to 5% are ultimately unable to tolerate it. In our previous trial with liraglutide in T1D, adverse effects occurred with a similar frequency to that observed in T2D (in which there is extensive clinical experience); on this basis it is expected that this will also be the case with semaglutide (8,9).

Dapagliflozin:
Very common: hypoglycaemia (when used with insulin).

Common: ketoacidosis, genital infections (vulvovaginitis, balanitis), urinary tract infection, diabetic ketoacidosis, increased creatinine.

In the DEPICT-1 study, ketoacidosis occurred in 4 of 277 participants on 5mg dapagliflozin, 5 of 296 participants on 10 mg and 3 of 260 participants in the placebo group (summarised as 1%, 2% and 1% respectively) with one severe episode in each group (14). In pooled analyses of DEPICT-1 and DEPICT-2 (14,15) there was, however, an approximate threefold elevated rate of DKA adjudicated as “definite” over placebo on active therapy: 2.0% on 5mg dapagliflozin, 1.9% on 10mg and 0.6% on placebo – i.e. rates of DKA were similar with the two doses (14,15,16). Rates of DKA with dapagliflozin were described as “occurring in at least 1 per 100 patients” by the manufacturer at the time the indication was removed (24). Rates of DKA with sotagliflozin, the other SGLT2 inhibitor licensed for type 1 diabetes, were somewhat higher (2.9% on 200 mg, 3.8% on 400 mg, 0.2% on placebo) in pooled analyses of Phase 3 trials (19,21).

Increased creatinine: in 13 studies summarized in the Summary of Product Characteristics increases in serum creatinine (generally transient) occurred in 3.2% vs 1.8% of individuals with eGFR ≥ 60 ml/min/1.73 m^2 who received dapagliflozin 10 mg vs placebo rising to 18.5% vs 9.3% in those with eGFR <30 ml/min/1.73 m^2 (16,29). These changes are usually observed 4 weeks after SGLT2i initiation, recovering by week 12. Participants with eGFR < 45 ml ml/min/1.73 m^2 will not be included in the TTT1 trial and creatinine/ eGFR will be monitored at 5 weeks (Visit 12) and 13 weeks (Visit 13) after randomisation to dapagliflozin or placebo (Visit 10). Any participant who has persistent reduction in eGFR of 15 ml/min or more will meet withdrawal criteria from the trial (see Section 7.8).

Uncommon: fungal infection, volume depletion, constipation.
Not known (several case reports): Fournier's gangrene.

Fournier’s gangrene is a very rare clinical entity characterized by necrotizing fasciitis of the perineal area. This has been infrequently reported in association with SGLT-2 inhibitor use in both men and women. However, there was no evidence of increased risk in a meta-analysis of 84 clinical trials involving 42415 participants. Nevertheless, participants will be advised to seek urgent medical assistance if they experience erythema, pain, swelling of the perineal area associated with fever and malaise (this will be reinforced in the Patient Information Sheet). (36)

Amputation: An increased incidence of lower limb amputation has been reported in type 2 diabetes with the SGLT-2 inhibitor canagliflozin. However, this has not been demonstrated with dapagliflozin (not included in the UK SmPC). People with diabetes are at increased risk of amputation and good foot care is a key component of good diabetes care regardless of use of SGLT-2 inhibitors.

Placebo:
Placebo capsules contain microcrystalline cellulose.
This trial is therefore categorised by the Co-Sponsors as:

- Type B = Somewhat higher than the risk of standard medical care

See Appendix 1 (Risk Assessment)

3 OBJECTIVES AND OUTCOME MEASURES/ ENDPOINTS

3.1 Primary objective

- To assess in people with type 1 diabetes whether triple therapy (dapagliflozin, semaglutide and insulin) for six months improves glycaemic control (as measured by HbA1c) compared with dual therapy (placebo, semaglutide and insulin –Period 2).

3.2 Secondary objectives

To assess in people with type 1 diabetes whether:

- Triple therapy (dapagliflozin, semaglutide and insulin) improves glycaemic control (as measured by HbA1c) compared with standard therapy (insulin)
- Dual therapy (semaglutide and insulin – period 1) improves glycaemic control (as measured by HbA1c) in comparison with standard therapy (insulin only)
- Triple therapy (dapagliflozin, semaglutide and insulin) improves glycaemic control as assessed by a range of continuous glucose monitoring (CGM) parameters, lowers body weight, reduces insulin requirement, improves blood pressure and improves quality of life (as assessed by validated patient questionnaires) compared with dual therapy and standard therapy.

3.3 Outcome measures/endpoints

3.4 Primary endpoint/outcome

Change in HbA1c (week 26 to week 52)

3.5 Secondary endpoints/outcomes

Efficacy:
Change in HbA1c

Mean fasting blood glucose

Mean blood glucose*
Percentage time in range (3.9 - 10.0 mmol/)*
Standard deviation of blood glucose*
Percentage time in hyperglycaemia Level 1 (10.1 - 13.9 mmol/L/)*
Percentage time in hyperglycaemia Level 2 (>13.9 mmol/L) *
Percent time in hypoglycaemia Level 1 (3.0 - 3.8 mmol/)*
(by masked DEXCOM G6 CGM)*

Insulin requirement
Bodyweight
Systolic and diastolic BP
Number of antihypertensive medications
Diabetes Specific Quality of Life Scale (DSQOLS)
Problem Areas In Diabetes (PAID) questionnaire

Safety:
Rate of hypoglycemic events Level 2 (<3.0 mmol/L/ <54mg/dl)
Rate of hypoglycemic events Level 3 (severe event with altered mental and/or physical status requiring assistance)
Rate of diabetic ketoacidosis defined as elevated serum or urine ketones (greater than the upper limit of the normal range), and serum bicarbonate < 15 mmol/L or blood pH < 7.3

3.6 Tertiary endpoints/outcomes

Postprandial glucose (AUC) – meal tolerance test
Postprandial glucagon (AUC) – meal tolerance test
Postprandial FFA (AUC) – meal tolerance test
Postprandial GLP-1 (AUC) – meal tolerance test
Fructosamine

3.7 Exploratory endpoints/outcomes

Plasma and urine samples will be collected and frozen for later analysis for indices of inflammatory stress and other relevant measures.

3.8 Table of endpoints/outcomes

Endpoints	Outcome Measures	Timepoint(s) of evaluation of this outcome measure
**Primary endpoint**<br>Triple therapy (dapagliflozin, semaglutide and insulin) in comparison with dual therapy (placebo, semaglutide and insulin – period 2)	Change in HbA1c	Week 26 to Week 52
**Secondary endpoint 1**<br><br>Triple therapy (dapagliflozin, semaglutide and insulin) in comparison with standard therapy (insulin alone)	Change in HbA1c	Week 0 to Week 52
**Secondary endpoint 2**<br><br>Dual therapy (semaglutide and insulin – period 1) in comparison with standard therapy (insulin only)	Change in HbA1c	Week 0 to Week 26
**Other secondary endpoints**<br><br>a) Triple therapy (dapagliflozin, semaglutide and insulin) in comparison with dual therapy (semaglutide, insulin and placebo – period 2)<br><br>b) Dual therapy (semaglutide and insulin – period 1) in comparison with standard therapy (insulin only)	Change in:<br>- Mean fasting blood glucose (4 days)<br><br>- Mean blood glucose*<br>- Percentage time in range (3.9–10 mmol/L)*<br>- Glycaemic variability (SD)*<br>- Percent time in hyperglycaemia Level 1 (10.1-13.9 mmol/L)*<br>- Percent time in hyperglycaemia Level 2 (>13.9	Week 26 to Week 52<br><br>Week 0 to Week 26

	mmol/L)*<br>- Percent time in hypoglycaemia Level 1* (3.0-3.8 mmol/L)<br>*(*by masked Dexcom G6 CGM)*<br><br>Insulin requirement<br>Body Weight<br>Systolic and diastolic blood pressure<br>Number of antihypertensive medications<br>Diabetes Specific Quality of Life Scale (DSQOLS)<br>Problem Areas In Diabetes (PAID) questionnaire	
**Tertiary endpoints**<br>a) Triple therapy (dapagliflozin, semaglutide and insulin) in comparison with dual therapy (semaglutide, insulin and placebo –period 2)<br><br>b) Dual therapy (semaglutide and insulin – period 1) in comparison with standard therapy (insulin only)	Change in:<br>Postprandial glucose (AUC)<br>Postprandial FFA (AUC),<br>Postprandial GLP-1 (AUC)<br>Postprandial glucagon (AUC)	Week 26 to Week 52<br><br>Week 0 to Week 26
**Safety endpoint 1**<br>a) Triple therapy (dapagliflozin, semaglutide and insulin) in comparison with dual therapy (semaglutide, insulin and placebo – period 2)<br><br>b) Dual therapy (semaglutide and insulin – period 1) in comparison with standard therapy (insulin only)	Difference between treatment arms in:<br>(i) Rate of Level 2 (<3.0 mmol/L/ <54mg/dL) hypoglycemic events<br>(ii) Rate of Level 3 hypoglycemic events (severe event with altered mental and/or physical status requiring assistance)	Week 26 to Week 52<br><br>Week 0 to Week 26
**Safety endpoint 2**<br>a) Triple therapy (dapagliflozin, semaglutide and insulin) in comparison with dual therapy (semaglutide, insulin and placebo – period 2)	Difference between treatment arms in:<br><br>Rate of diabetic ketoacidosis (serum or urine ketones above normal limits) AND serum bicarbonate < 15 mmol/L OR blood pH < 7.3	Week 26 to Week 52

b) Dual therapy (semaglutide and insulin – period 1) in comparison with standard therapy (insulin only)		Week 0 to Week 26

4 TRIAL DESIGN

The trial has two six-month periods:

Period 1 open-label randomised (2:1) parallel group semaglutide once weekly + insulin (standard treatment) vs insulin alone (standard treatment).
Period 2 double-blind, randomised (1:1) parallel group, dapagliflozin vs placebo for those participants initially randomised to semaglutide (with ongoing standard of care for all participants)

Thus, during the first six months, approximately 114 participants with type 1 diabetes (50 in the UK) will be randomised (open label, see below) into three groups (approximately 38 each; i.e. 19 each in the UK) one of which will be maintained on their regular insulin therapy for 12 months (standard therapy group) and two of which will be started on semaglutide in addition to regular insulin treatment for six months (dual therapy). Following six months of stable treatment, those initially randomised to insulin and semaglutide will be additionally randomised (double-blind) to dapagliflozin (triple therapy) or matching placebo (dual therapy) for a further six months. The dose of dapagliflozin will not be increased to 10 mg in those who have confirmed ketonuria (+1) or ketonaemia (≥0.6 mM) after the first week of treatment.

In keeping with the intention-to-treat principle, participants in whom therapy is discontinued or downtitrated due to adverse effects will be encouraged to continue to undertake all study assessments and attend study visits.

5 STUDY SETTING

The study will be conducted at two sites: State University of New York (SUNY) at Buffalo, USA and Glasgow, UK. In Glasgow, n=50 participants (45% of the total) will be identified from secondary care sites and via the Scottish Diabetes Research Network research register. Both randomisations will be stratified by site.

6 ELIGIBILITY CRITERIA

6.1 Inclusion criteria

1) Type 1 Diabetes for at least one year on stable multiple daily (four or more) injections (MDI) of insulin or continuous subcutaneous insulin infusion (CSII) for the previous three months.
2) C-peptide < 0.23 nM
3) Minimum dose of insulin: 0.5 U/kg for MDI and 0.4 U/kg for CSII
4) Regularly measuring blood glucose: four or more times daily either by fingerstick, testing or by "flash"/ continuous glucose monitoring)
5) HbA1c 7.5 – 11.0 % inclusive
6) Well versed in carbohydrate1 counting*
7) Age 18-70 years.

8) BMI ≥25 kg/m^2.

** In the opinion of the site Principal Investigator*

6.2 Exclusion criteria

1) Type 1 diabetes for < 12 months, type 2 diabetes, chronic pancreatitis, MODY
2) Previous use of any agent other than insulin for glycaemic control in the last three months.
3) History of diabetic ketoacidosis (DKA) requiring medical intervention (e.g. emergency room visit and/or hospitalisation) within three months prior to the screening visit.
4) Following ketogenic diet, low (<50g/day) carbohydrate diet or intermittent fasting.
5) Hypoglycaemia unawareness* or frequent episodes of severe hypoglycaemia as defined by more than one episode requiring medical assistance, emergency care (paramedics or emergency room care), or glucagon therapy administered by a third-party individual within three months prior to the screening visit
6) Symptoms of poorly controlled diabetes
7) Participation in a weight loss program with ongoing weight loss, or in an intensive exercise program
8) History of bariatric or other weight-loss surgery within 12 months prior to the screening visit
9) History of Addison's disease or chronic adrenal insufficiency
10) History of diabetes insipidus
11) Hepatic disease or cirrhosis
12) Aspartate Aminotransferase (AST) > 3 X Upper limit of normal (ULN) and/or Alanine aminotransferase (ALT) > 3 X ULN
13) Serum Total Bilirubin > 2 X ULN unless exclusively caused by Gilbert's Syndrome
14) Haemoglobin < 110 g/L (11.0 g/dL) for men; haemoglobin < 100 g/L (10.0 g/dL) for women
15) Coronary event or procedure (myocardial infarction, unstable angina, coronary artery bypass, surgery or coronary angioplasty) in the previous three months or history of congestive heart failure
16) End stage renal disease on haemodialysis and/or eGFR < 45 ml/min/1.73m^2
17) HIV or Hepatitis B/C positive status
18) Any other life-threatening, non-cardiac disease
19) History of pancreatitis or cystic fibrosis
20) Women who are pregnant, planning pregnancy or breast-feeding and women of childbearing potential (WOCBP) who are not established on a method of contraception considered highly effective (see Appendix 2) and willing to continue it for the duration of the study and for at least two months after last dose of IMP.
21) Inability to give informed consent
22) History of gastroparesis
23) History of medullary thyroid carcinoma or MEN 2 syndrome
24) History of hypersensitivity reaction to GLP-1 agonists or SGLT2 inhibitors or known hypersensitivity to any of the excipients
25) Known gallstones (history of cholecystectomy is not an exclusion criterion)
26) History of alcohol problem or drug abuse
27) Hypertriglyceridemia (>500 mg/dl)
28) Recurrent genital mycotic infection (more than one episode in last six months)
29) Hypovolaemia

30) Any malignancy except treated *in situ* malignancy and basal cell carcinoma of the skin
31) Unexplained haematuria (dipstick positive on two occasions one week apart) and/or active bladder cancer
32) Proliferative retinopathy (on basis of photographic retinal screening which must have been performed within the last 12 months)
33) Use of an investigational agent or therapeutic regimen within 90 days of study
34) Participation in any other concurrent interventional clinical trial
35) Unlikely to be able to comply with study requirements*
36) Unable to undergo meal challenge for dietary reasons (e.g. vegetarian/ vegan diet)

** In the opinion of the site Principal Investigator*

7 TRIAL PROCEDURES

Also see *Schedule of Assessments*

Each participant will be enrolled in the trial for approximately 55 weeks, comprising three weeks of monitoring to stabilise treatment and collect baseline data before randomisation into the intervention or standard of care/ placebo arms for 52 weeks. Following a completed screening visit and meeting the Inclusion and Exclusion criteria, each participant will have a total of 16 scheduled study visits. In addition, at weeks 3, 5, 8, 16, 20, 29, 35, 44 and at two weeks after the end of the study, each participant will receive a phone call to document any adverse effects or other changes. No long-term follow-up is planned following completion of participation in the study.

Participants will be treated with either insulin only (standard treatment) for the whole duration of the study or with insulin and semaglutide weekly s.c. injection (titrated where possible up to 1.0 mg, see below) for the first six month period and then randomised to either oral dapagliflozin (triple therapy) or placebo (dual therapy) daily added for the second six month period. Semaglutide will be started (Visit 4) at a dose of 0.25 mg once weekly for two weeks then increased if tolerated to 0.5 mg (Visit 5). If well tolerated at week 4, it will be increased to 1.0 mg once weekly (Visit 6) or at a subsequent visit at the discretion of the PI. Dapagliflozin (or placebo) will be started at a dose of 5 mg daily at week 26 and increased at week 27 to 10 mg (2 x 5mg capsules) daily to ensure drug tolerability and minimise the risk of insulin-induced hypoglycaemia or hypotension.

Excessive weight loss: Both semaglutide and dapagliflozin are known to cause weight loss in the range of 5-10% in type 2 diabetes. However, the magnitude of weight loss when these are combined in type 1 diabetes is uncertain. Excessive weight loss may lead to increased risk of hypoglycemia or decreased insulin requirement increasing the risk of ketosis or DKA (see Section 2.1b) and other unforeseen risks. Therefore, weight loss during any phase of the study will be limited to ≤ 15% of body weight at randomisation or reaching a BMI <22 kg/m^2. If participants are approaching this threshold, IMP will be sequentially reduced at the discretion of the PI. Semaglutide may be reduced to 0.25mg or 0.5 mg and dapagliflozin to 5 mg. See also Section 7.8 regarding treatment discontinuation.

Covid-19 Mitigation

In view of the Covid-19 pandemic, certain trial procedures may be changed if necessary to reduce in person contact with participants. This will be continuously reviewed in view of the prevailing

circumstances in line with local and national guidance and with the principle of protecting participants and study personnel. In the event of future waves of infection this would be particularly important. In this context, consideration would be given to rearranging visits, changing to virtual visits using video technology or using additional phone calls to ensure patient safety. Consideration will also be given to how minimising face-to-face interaction with participants when dispensing study medication. If required, supplies of study medication sufficient to last for longer periods may be dispensed to minimise contact and visits. Courier services may additionally be used to deliver study medications to participants.

All in person visits will be subject to local and national guidance around the use of personal protective equipment including face masks and where appropriate when conducting procedures, the use of gloves and aprons.

Gastrointestinal adverse effects:

During the initial weeks of participation, those participants who report nausea and/or vomiting during uptitration of semaglutide dose that is not sufficiently severe to require treatment discontinuation will remain on their current dose until they are symptom-free prior to discussing a further dose escalation (i.e. uptitration will be to the highest dose tolerated rather than a “forced titration”). Dose will be downtitrated to the maximum tolerated in those who experience gastrointestinal adverse effects.

Retinopathy:

As there was no excess of retinopathy with semaglutide treatment in Phase 3 trials with semaglutide, in which proliferative retinopathy was an exclusion criterion (as in the current protocol), additional retinal imaging beyond annual retinal photography (the standard of care) is not proposed (30).

Insulin dose adjustment:

Minimising hypoglycaemia: All participants will be made aware of the potential for increased hypoglycaemia with addition of adjunct therapy and supported and encouraged to monitor blood glucose at least four times daily in order to minimize the risk of hypoglycaemia. Participants will have ready access to advice from specialist medical and nursing staff at face-to-face and telephone visits. Those who have ongoing access to wearable continuous glucose monitoring [or "flash" glucose monitoring, (FGM)] will continue to use these devices. Information on management of hypoglycaemia will be included within participant diaries, reinforcing the pre-existing knowledge of participants, the majority of whom will have lived with type 1 diabetes for many years.

Reductions in insulin dose will not usually be greater than 10% (5% if HbA1c is < 7.0 %) from current levels. At randomisation, daily insulin dose will not be reduced by more than 20% from the baseline level and then from the new baseline levels at 26 weeks. Participants requiring an insulin dose reduction > 20% and their total insulin dose < 0.4 U/kg (current weight) will be monitored closely for insulin dose, carbohydrate intake, ketones and blood glucose. Following the second randomisation at 26 weeks, dapagliflozin/ placebo will be temporarily discontinued if insulin dose falls below this threshold and permanently discontinued if it remains so at review in one week.

Minimising ketosis: Participants will be instructed to stay hydrated and not to reduce their caloric intake (especially carbohydrates) or start new dietary approaches (especially ketogenic diets) during the study. They will be educated to recognize the signs and symptoms of diabetic ketoacidosis (DKA) and to undertake additional self-testing of urine and/or blood ketones if they develop nausea, vomiting, or anorexia. In addition, they will be given education and a written ketosis management plan (STICH Protocol – i.e. STop SGLT-2 inhibitor or placebo (temporarily), give Insulin bolus, take additional Carbohydrate, and additional Hydration - see Appendix 3) (37).

From Visit 10 (26 weeks), those participants undergoing the second randomisation will be supported and encouraged to monitor urine or blood ketones at home at least once daily: three days before visit 10 (week 26), they will be asked to start daily measurements of urine ketones [CombiScreen® 10 SYS Plus urine testing strips, Sterilab, UK (or equivalent)] or blood ketones [CareSens™ Dual, i-SENS, Koreal; GlucoMen® Areo 2K, Menarini, UK (or equivalent]]. They will be asked to record these in the diary/ log provided. In addition, ketones (blood and urine) will be measured at every visit. If ketone bodies in urine are +1 or greater (≥0.6 mM in blood), participants will be reminded to keep well hydrated and notify the study team as soon as possible (office hours) as diabetic ketoacidosis (DKA) can occur even at glucose levels < 14 mmol/L (38).

If ketones are +2 or more in urine (> 1.5 mmol/L) participants will be advised to initiate the STICH Protocol immediately and contact the study team in office hours (or telephone NHS 24 or attend their Accident & Emergency if out of hours) (37). This information will be reinforced verbally at study visits and also in the form of a Patient Alert Card that participants will be asked to carry. This will also contain details of the investigational study drug, the study number, the investigator's name, and a contact number (for in hours contact). The study team will assess for symptoms or test results indicating a risk of ketoacidosis immediately on reporting and if blood ketone levels are persistently (one week) at these levels dapagliflozin/placebo will be permanently discontinued.

If blood ketone readings are confirmed as > 3.0 mmol/L (or urine ketones reading +++) participants will be advised to stop dapagliflozin/ placebo and attend the Accident and Emergency department without delay.

Participants will be advised that dapagliflozin/placebo may be temporarily stopped in other clinical situations known to predispose to ketoacidosis (e.g. acute serious medical illnesses or hospitalisation for major surgical procedures). Any requirement for intravenous insulin infusion (whether in Accident and Emergency or as an in-patient admission) will be recorded as a Serious Adverse Event. For further detailed procedures see section 9.31.

Volume depletion:
There is a theoretical risk of volume depletion and low blood pressure when dapagliflozin is used in combination with other anti-hypertensive drugs. All pre-existing concomitant medication will be reviewed at the screening visit as part of the medical history and examination (see Section 7.1.3 for further details). Participants undergoing the second randomisation will be educated on symptoms suggestive of low blood pressure (e.g. light-headedness, dizziness, feeling faint) and asked to contact the study team if they experience these symptoms. In clinical practice however SGLT-2 inhibitors are commonly used with anti-hypertensive drugs with no significant problems (31).

Genital mycotic infections:
Individuals who report symptoms suggestive of genital mycotic infection will be advised to self-treat with topical antifungal preparations. If a second episode occurs, a decision on treatment discontinuation will be considered in discussion between the participant and the site Principal Investigator, taking account of the severity of the symptoms.

Foot care:
Annual foot checks will be conducted for all participants as per standard of care.

7.1 Recruitment

7.1.1 Patient identification

All personal details will be managed according to ISO 27001:2013 compliant Standard Operating Procedures.

Potentially eligible participants will be identified from the Scottish Diabetes Research Network Research Register (a managed and searchable database of people with diabetes who have agreed to be contacted about clinical trial participation) and at secondary care diabetes clinics in Greater Glasgow and Clyde. The principal site for recruitment will be Stobhill Ambulatory Care Hospital but other sites may be opened if necessary as Patient Identification Centre sites. The majority of study visits (and all visits involving drug dispensing) will be conducted at the Clinical Research Facility, Glasgow Royal Infirmary. Information posters with the contact details of the research team will be displayed in recruiting clinics. .

At the principal site, clinic lists (whether remote or face-to-face) will be pre-reviewed by members of the research team. An email will be sent to the clinic Consultant physician to flag potentially eligible individuals on their list; potential eligibility will also be flagged on the clinic electronic list (on Trakcare®) to prompt reviewing physicians (as members of the usual care team) to check basic eligibility criteria, provide brief verbal information about the trial and outline the implications of participation (e.g. number of visits).

In the above email (to which a response is not required), physicians will be asked to add a note indicating the outcome of their discussion with the potential participant to the Clinical Portal system using standard text (e.g. willing to be contacted/ not willing to be contacted/ unsuitable for the trial) and the permitted and preferred method(s) of contact (e.g. mobile telephone/ home telephone/ letter/ email). Physician consultations with potentially eligible individuals that are concluded with no invitation to research participation (e.g. due to pressure of time) can be followed up with a further phone call from one of the study physicians who is also a member of the research team.

Following clinical consultations, members of the research team will identify potential participants who appear to be meet eligibility criteria and have given permission to be contacted via their access to the Clinical Portal system. These individuals will be given or sent/ emailed (as appropriate according to documented permissions) a Summary Patient Information Sheet and an Expression of Interest Form (with a prepaid envelope). Where permission has been given for phone contact, this can be followed up by up to two further phone contacts (provided permission to contact has not been withdrawn by the potential participant in an earlier call). A visit to the Clinical Research Facility will then be scheduled for further discussion with a full Patient Information Sheet sent out to arrive at least 24 hours prior to this visit.

At the screening visit, eligibility criteria will be checked and the study procedures explained. All potential participants will have had at least 24 hours to review the full Patient Information Sheet. They will then be invited to provide written, informed consent.

At other sites within NHS Greater Glasgow and Clyde the same approach will be taken for the clinic lists of those physicians who indicate willingness to support trial recruitment. The initial approach will always be from a member of the usual care team.

Covid-19 Mitigation: The delivery of outpatient clinics has been substantially changed due to the pandemic. The use of telephone clinics is now standard for many visits and video technologies are also being used. These mediums will therefore be used for inviting participants to take part when in person appointments are not taking place. This may be subject to change depending on the situation with Covid-19. Other principles of recruitment will be as described above.

7.1.2 Screening

A separate non-fasting visit will be arranged at which potential participants will have further time to discuss with the study nurse and doctor.

Prospective participants will be asked to read and understand the Participant Information Sheet. Any questions regarding the Protocol will be answered. Risks and side-effects of the active trial medication will be explained. Eligibility for trial enrolment will be confirmed by a medical practitioner.

Individual wishing to participate in the study will then be invited to sign to document their informed consent (see Section 7.2 below).

7.1.3 Screening assessments

Items from the medical history relevant to the Inclusion and Exclusion criteria will be recorded. Demographic data, medical history and current medications (including current insulin doses) will be documented. All participants will have access to a dual blood glucose and ketone meter as per the contemporary standard of care in type 1 diabetes. A physical examination will be conducted. Clinical observations will be recorded and blood and urine samples obtained (see below):

<u>Demographics</u>

- Date of birth
- Sex
- Ethnicity
- Date of diagnosis
- Ethnic group: white/black/Asian/other
- Height

<u>Clinical assessments</u>

- Systolic and diastolic blood pressure (after 5 minutes rest)
- Heart rate
- Body weight (clothed without coat and shoes)
- Capillary blood glucose

<u>Medical History</u>

- Smoking status: current/ex/never
- Alcohol consumption: units/week
- Hypertension: Y/N
- Microalbuminuria: Y/N
- Proteinuria: Y/N
- Neuropathy: Y/N
- Previous major cardiovascular event(s): Y/N (date)
- Existing CV disease of any type*: Y/N
- Amputation above forefoot: Y/N
- Hospitalisation for DKA in last year
- Level of educational attainment: University (college) educated Y/N

* Existing CV Disease is defined as any of: MI, stroke, ACS/unstable angina, coronarystent/angioplasty, CABG, TIA, heart failure, peripheral vascular disease

Insulin treatment
- Regimen: MDI, pump,
- Total dose (all); Basal (longer acting) dose/ Bolus (shorter acting) dose (for MDI only)

Other treatment (drug classes and names)
Current use Y/N. If no, has there been use in last three months?: Y/N

- ACE inhibitors
- Angiotensin receptor blocker
- Calcium channel blocker
- Alpha-blocker
- Beta-blocker
- Loop diuretic
- Thiazide like diuretic
- Statin
- Aspirin
- Clopidogrel
- Novel anticoagulant
- Warfarin

Blood assessments:
- Blood ketones
- HbA1c
- FBC
- Urea and electrolytes
- Creatinine
- Liver function tests (AST/ ALT/ Alkaline Phosphatase)
- Bone profile (Calcium/ Phosphate)
- C-peptide (capillary blood glucose must be > 4.0 mmol/L at time of sampling)
  N.B. where re-screening is undertaken (see 7.2.1 below), C-peptide does not require to be repeated

If blood tests have been assessed for clinical purposes within the last three weeks, these values will be used.

Urine sample:
- Urine ketones
- For all WOCBP, a urine pregnancy test will be checked at screening

Potential participants meeting all the inclusion and exclusion criteria based on history, examination and screening tests will be enrolled in the study.

Pregnancy: WOCBP will be asked about pregnancy status and contraceptive usage (see Appendix 2) and a pregnancy test will be conducted (following informed consent) prior to randomisation. We will not recruit those who are pregnant, wanting to become pregnant, or using birth control methods not considered highly effective. Every four weeks following randomisation, WOCBP randomised to IMP (as opposed to standard of care) will be asked about their contraception status, and a urine pregnancy test carried out. Study medication will be permanently discontinued in women who become pregnant or considered to be on contraception that is not highly effective. As the study assessments *per se* do not carry an additional risk over standard of care to either mother or unborn child, further pregnancy tests will not be routinely offered

following randomisation to those women allocated to “Insulin therapy (standard of care)” (i.e. who are not exposed to IMP). All pregnancies will be notified to the Sponsor Pharmacovigilance Officer using the standard pregnancy notification form and the pregnancy followed to outcome).

7.1.4 Payment

Participants will be offered reasonable travel expenses for visits additional to usual care.

7.2 Consent

Potential participants will be identified and screened by the Inclusion and Exclusion criteria listed above. Medical staff or appropriately trained support staff will seek consent for screening and participation in the trial from individuals that fulfil the criteria. All potential participants will have at least 24 hours to review the Patient Information Sheet before being approached (see Section 7.1.2 above). Following written consent, each signature will be dated by the signatory, the original retained in the site file, a copy provided to the participant and a copy scanned into the participant's Clinical Portal record.

Data collected for routine clinical care will be used for clinical trial documentation (e.g. blood results within the previous three weeks). In the absence of routine blood results, sampling of blood for study specific laboratory measurements can only be performed following documentation of informed consent.

7.2.1 Re-screening post-consent

Where inclusion (e.g. HbA1c ≤7.5%) or exclusion (e.g. DKA within the last three months) criteria are not met (e.g. screening visit blood results falling outside the study parameters), potential participants can be re-screened with additional urine and blood tests on one further occasion.

Where the screening period (i.e. between the Screening visit and Visit 1) exceeds 10 weeks, screening visit urine and blood tests should be repeated at an additional visit that can be arranged with verbal consent of the participant prior to Visit 1. Data will be amended on the web portal as appropriate.

If the initial consent was signed within three months of the re-testing, new consent is not required unless the consent form template has been updated in this period. If the initial consent was not signed within three months of re-testing then consent should be sought again.

Rescreened participants will keep the same Patient ID and data will be amended on the web portal as appropriate.

Where screening bloods are repeated in cases as above, C-peptide testing does not require to be repeated as values consistent with the inclusion criteria remain valid long term.

7.3 Run-in period

7.3.1 Visit 1 (@3 weeks prior to randomisation)

Participants who meet entry criteria will be scheduled for their next visit at approximately three weeks prior to randomisation. Daily fingerstick blood glucose levels (study diaries), CGM/ FGM data or insulin pump download data (as applicable) will be collected and reviewed and appropriate insulin dose adjustments made by the medical team to target fasting blood glucose concentrations between 5 -10.0 mmol/L if possible at all times without having additional hypoglycaemia (< 3.9 mmol/L). Clinical observations will be

recorded including resting BP measurement. Blood glucose and ketone bodies will be measured via a fingerstick blood sample (see Section 7). Urine for pregnancy status and ketone bodies will be assessed.

Participants will be instructed on the use of the Continuous Glucose Monitoring System (Dexcom G6 with blinded receiver). All subjects will be advised to monitor their capillary blood glucose by CGM or fingerstick at least 4 times a day (before meals and at bedtime) throughout the study and to wear their CGM constantly for next 20 days (changing the sensor at 10 days). CHO counting technique will be reviewed and appropriate support given with calculating bolus insulin doses.

Visit procedures:

Monitoring and treatment optimisation

- Check for adverse events
- Review blood glucose diary
- Blood ketones
- Adjust insulin doses (if necessary)

Clinical assessments

- Blood pressure
- Heart rate
- Weight

Urine sample:

- Check urine ketones

Other

- Fit and start masked Continuous Glucose Monitoring with Dexcom G6

7.3.2 Visit 2 (1 @ weeks prior to randomisation)

Fasting visit* - Participants will be asked to fast (no food or calorie intake) for 10 hours prior to the visit overnight. Those who plan to drive to fasting visits will be reminded of the increased risk of hypoglycaemia and DVLA guidelines. This will be repeated prior to each fasting visit and reiterated within the patient diaries. This visit will be scheduled for a morning appointment and participants will be advised not to take their breakfast bolus insulin dose prior to arrival. If participants arrive at the study visit in a non-fasted state, the visit will be terminated and a further fasting visit rearranged as soon as possible.

Daily fingerstick blood glucose levels (diaries), CGM/ FGM data or insulin pump download data (as applicable) will be collected and reviewed and appropriate insulin dose adjustments made. Clinical observations will be recorded as detailed below. Blood glucose and ketone bodies will be measured via a fingerstick blood sample (see Section 7). If blood glucose is less than 3.9 mmol/L, or more than 13.9 mmol/L, the visit will be rescheduled for an alternative date. Otherwise, an intravenous cannula will be placed in order to obtain blood samples for the duration of the visit. A Meal Challenge Test will be performed (described below). A 24 hr urine container will be dispensed to collect a 24 hour urine sample to bring in at the Week 0 visit. For this collection and all subsequent 24 hour urine collections, urine will be tested via dipstick testing and an aliquot retained for assay of albumin, creatinine, ketones, and 15-isoprostane.

Visit Procedures

Monitoring and treatment optimisation

- Check for adverse events
- Review blood glucose diary
- Blood ketones
- Adjust insulin doses (if necessary)

Clinical assessments

- Blood pressure
- Heart rate
- Weight

Urine sample:

- Check urine ketones
- 24 hour urine container dispensed

Other assessments

- Download Dexcom G6 CGM
- High fat high calorie Meal Challenge: in order to assess postprandial metabolism a Meal Challenge Test will be performed using an approximately 900 kilocalorie High Fat High Carbohydrate (HFHC) meal including an egg muffin sandwich, a sausage muffin sandwich and two hash browns [containing approximately 88g carbohydrate, 51 g fat (33% saturated) and 34 g protein]. Participants will be asked to attend fasting prior to breakfast insulin bolus (with continuing basal insulin); participants will be asked not to take bolus insulin until the 3 hour time point unless blood glucose values reach above 22.2 mmol/L. Blood samples will be collected from an indwelling intravenous canula in a superficial forearm vein for glucose, glucagon, FFA, GLP-1 (AUC). Sequential blood samples will be obtained at 0, 15, 30, 45, 60, 90, 120, 150 and 180 min after consuming the meal. Volume of samples at 15, 30, 45, 90 and 150 min will be 5ml while those at 0, 60, 120 and 180min will be 30 ml (total volume = 145 ml). A spot urine sample will be collected at baseline, 1 and 3hr for standard urinalysis and an aliquot retained for assay of ketones and 15-isoprostane.

7.4 Randomisation

Participants who are being randomised will be required to have undergone all screening assessments.

At the randomisation visit, study participants will be provided with a Patient Alert Card. There are two Versions: (1) for the intervention arm i.e. those allocated at first randomisation to open-label semaglutide who are also eligible for the second double-blind randomisation (dapagliflozin or placebo); and (2) for those allocated to standard of care. These cards supplement the study diaries and include contact information for the study team. Version 1 also contains unblinding information. Participants will be asked to carry their card at all times. These cards will be collected at the end of participation in the study.

7.4.3 First Randomisation Scheme

For the first randomisation (Period 1, open label), eligible and consenting participants will be randomised (2:1, stratified by site) to the two groups (open-label semaglutide or standard of care) using permuted blocks of variable size to minimize predictability.

7.4.4 Method of implementing the allocation sequence

Randomisation will be achieved by accessing an interactive web-based randomisation system (IWRS). The investigator will provide the participant identifier and the system will check the participant's eligibility from information already entered in the electronic Case Report Form (eCRF): if appropriate the randomisation group will be allocated.

7.5 Visit 3 (First Randomisation Visit)

Participants will be asked to attend in the fasting state (no food or calorie intake for 10 hours; see 7.3.2 above). Blood glucose levels and ketone bodies will be measured via a fingerstick blood sample using a glucose and ketone meter (see Section 7) and daily fingerstick blood glucose levels (diaries) collected and reviewed. The participant's own CGM/ FGM blood glucose data or insulin pump download data will also be collected and reviewed. Daily insulin dose will be adjusted following clinical review of study diaries: if reduced, this should not be by more than 20% from the baseline level Participants will discontinue use of the study masked CGM (Dexcom G6). Clinical observations will be recorded. Blood glucose and ketone bodies will be measured via a fingerstick blood sample (see Section 7). The 24 hour urine container will be collected. A fasting blood sample will be taken (45 mL) to evaluate study endpoints, FBC, U&E, LFT, bone profile, lipids, HbA1c, and fructosamine (detailed below). A urine sample will be collected to evaluate ketone bodies and, in females, pregnancy status.

Participants will be asked to complete Quality of Life questionnaires (see Appendices).

IMP will be dispensed for those randomised to open-label semaglutide (once weekly). Semaglutide will be started on site at the 0.25 mg dose. Participants will be instructed to call site staff directly in case of any problem or untoward side effects. An office hours phone number will be provided for advice. Outside these hours, provision of emergency medical care if required will be via NHS 24 and/or Accident & Emergency. Participants will be specifically asked to call if they have hypoglycaemia (blood glucose < 3.9 mmol/L) or Level 2 hyperglycaemia (blood glucose >13.9 mmol/L), particularly if occurring on more than one occasion. This will apply for the remainder of the trial.

Visit Procedures

Fasting visit* as per visit 2.

Monitoring and treatment optimisation

- Check for adverse events
- Check adequacy of contraception (WOCBP)
- Review blood glucose diary
- Adjust insulin doses (if necessary)
- Blood ketones

Clinical assessments

Blood pressure

- Heart rate
- Body weight

Quality of life assessments

- Diabetes Specific Quality of Life Scale (DSQOLS)
- Problem Areas In Diabetes (PAID) questionnaire

Blood assessments

- Na, K, urea, creatinine, eGFR (MDRD)
- AST/ ALT/ Alkaline Phosphatase
- Calcium/ Phosphate
- FBC
- HbA1c
- Fructosamine
- Haemoglobin
- Platelets
- Bilirubin
- Random glucose

Urine
- For WOCBP, a urine pregnancy test will be checked prior to first administration of IMP.
- Urine ketones
- 24 hour urine sample collected

Medication
- Administer open label semaglutide 0.25 mg if randomised to active treatment
- Issue Patient Alert Card (appropriate version according to randomisation)

Other:
- Read masked Continuous Glucose Monitoring with Dexcom G6

Bloods for storage
Approximately 45 mls of venous blood will be withdrawn and collected in tubes containing EDTA. Blood will be centrifuged at 1500g for 20 mins at 4ºC. Plasma will be siphoned, aliquoted and stored locally at -80° ± 10°C until transport in batches to the central laboratory on dry ice.

7.5.1 Visit 4 (@1 week after first randomisation)

Clinical observations will be recorded. Blood glucose levels and ketone bodies will be measured via a fingerstick blood sample using the participant's own glucose and ketone meter (see Section 7)) and daily blood glucose levels collected and reviewed. Insulin doses will be adjusted as in visit 3. If HbA1c is <7.0 %, insulin dose reduction should not be >5% from baseline total dose. Semaglutide 0.25mg will again be injected on site for those randomised to active therapy; however, if the participant is unable to attend within 48 hours of the scheduled appointment this dose can be self-administered at home (confirmed by a documented phone call) provided there were no adverse effects following the previous dose.

Visit Procedures

Monitoring and treatment optimisation
- Check for adverse events
- Review blood glucose diary
- Adjust insulin doses (if necessary)
- Blood ketones

Assessments
- Blood pressure
- Heart rate
- Weight

Urine sample:

- Urine ketones

Medication

- Support participant's first self-injection of open label semaglutide 0.25 mg if randomised to active treatment (dose on-site if dose is scheduled for visit day (see above)

7.5.2 Visit 5 (@2 weeks after first randomisation)

Clinical observations will be recorded. Blood glucose levels and ketone bodies will be measured via a fingerstick blood sample using glucose and ketones meter (see Section 7) and daily blood glucose levels collected and reviewed. Daily blood glucose data collected and reviewed and insulin doses adjusted as per Visit 3. If participants randomised to semaglutide are experiencing any nausea, they will continue on the 0.25 mg dose for another week (or more as required) before increasing the dose. Otherwise semaglutide 0.5mg dose will be injected on site. If dose escalation is not planned, dose can be self-administered at home (confirmed by a documented phone call) provided there were no adverse effects following the previous dose. Supply of IMP will be dispensed as appropriate to last until the next visit.

Visit Procedures

Monitoring and treatment optimisation

- Check for adverse events
- Review blood glucose diary
- Adjust insulin doses (if necessary)
- Blood ketones

Assessments

- Blood pressure
- Heart rate
- Weight

Urine sample:

- Urine ketones

Medication

- Dose titration (see above) if randomised to open-label semaglutide
- Return used study pen
- Dispense medication if randomised to open-label semaglutide (dose on-site if dose is scheduled for visit day– see above)

7.5.3 Visit 6 (@4 weeks after first randomisation)

For those randomised to open-label semaglutide, doses administered will be recorded (self-report). Clinical observations will be recorded. Daily blood glucose data will be collected and reviewed and insulin doses adjusted as per visit 3. Supply of IMP will be given to last until the next visit. A urine sample will be collected to evaluate ketone bodies and, in females randomised to IMP, pregnancy status. Unless experiencing nausea or other adverse events, the recommended dose will be increased to 1.0 mg weekly (or 0.5 mg if not previously uptitrated from 0.25 mg). A 24 hr urine container will be dispensed to collect a 24 hour urine sample to bring in at the Week 13 visit.

Visit Procedures

Monitoring and treatment optimisation

- Check for adverse events
- Check adequacy of contraception (WOCBP)
- Review blood glucose diary
- Adjust insulin doses (if necessary)
- Blood ketones

Assessments

- Blood pressure
- Heart rate
- Weight

Urine sample:

- Urine ketones
- Urine pregnancy test (WOCBP randomised to IMP only)
- 24 hour urine container dispensed

Medication

- Record doses of semaglutide self-injected since last visit (self-report)
- Dose titration if randomised to open-label semaglutide (see above)
- Return used study pens
- Dispense medication if randomised to open-label semaglutide

7.5.4 Visit 7 (@13 weeks after first randomisation)

Participants will be asked to attend in the fasting state (no food or calorie intake for 10 hours). If participant is not fasting, samples should be taken and non-fasting status noted. For those randomised to open-label semaglutide, doses administered will be recorded (self-report). For those who have remained on doses lower than 1.0 mg weekly, this visit will be the last opportunity to uptitrate if not experiencing nausea or other adverse events (from 0.25 mg to 0.5 mg weekly - or from 0.5 mg to 1.0 mg weekly). Daily fingerstick blood glucose levels (diaries), CGM/ FGM data or insulin pump download data will be collected and reviewed and appropriate insulin dose adjustments made. Supply of IMP will be given to last until visit 10. Observations will be recorded. A urine sample will be collected to evaluate ketone bodies and, in females randomised to IMP, pregnancy status. The 24 hour urine container will be collected. A fasting blood sample will be taken (45 mL) to evaluate study endpoints.

Visit Procedures

Fasting visit*

Monitoring and treatment optimisation

- Check adequacy of contraception (WOCBP)
- Check for adverse events
- Review blood glucose diary
- Adjust insulin doses (if necessary)
- Blood ketones

Clinical assessments
- Blood pressure
- Heart rate
- Weight

Blood assessments
- Na, K, urea, creatinine, eGFR (MDRD)
- AST/ ALT/ Alkaline Phosphatase
- Calcium/ Phosphate
- FBC
- HbA1c
- Fructosamine
- Haemoglobin
- White cell count
- Platelets
- Bilirubin
- Random glucose

Urine
- Pregnancy test (WOCBP randomised to IMP only)
- Urine ketones
- 24 hour urine bottle collected

Medication

- Record doses of semaglutide self-injected since last visit (self-report)
- Dose titration if randomised to open-label semaglutide (see above)
- **N.B. no further uptitration after this visit**
- Return used study pens
- Dispense medication if randomised to open-label semaglutide

7.5.5 Visit 8 (@23 weeks after first randomisation)

For those randomised to open-label semaglutide, doses administered will be recorded (self-report). Clinical observations will be recorded. Daily blood glucose data (fingerstick or FGM) will be collected and reviewed as per visit 3. Blood glucose levels and ketone bodies will be measured via a fingerstick blood sample. A urine sample will be collected to evaluate ketone bodies and, in females randomised to IMP, pregnancy status. Insulin doses will be adjusted if needed. All subjects will be reminded to monitor their capillary blood glucose by fingerstick at least 4 times a day (before meals and at bedtime) throughout the study. CGM sensor will be fitted and participants asked to wear their CGM constantly for next 20 days.

Visit Procedures

Monitoring and treatment optimisation
- Check adequacy of contraception (WOCBP)
- Check for adverse events
- Review blood glucose diary
- Blood ketones
- Adjust insulin doses (if necessary)

Clinical assessments
- Blood pressure
- Heart rate
- Weight

Urine sample:
- Pregnancy test (WOCBP randomised to IMP only)
- Urine ketones

Other assessments
- Fit and start masked Continuous Glucose Monitoring with Dexcom G6

7.5.6 Visit 9 (@25 weeks after first randomisation)

Participants will be asked to attend in the fasting state (no food or calorie intake for 10 hours). If participants arrive at the study visit in a non-fasted state, the visit will be terminated and a further fasting visit rearranged as soon as possible. Daily fingerstick blood glucose levels (diaries), CGM/ FGM data or insulin pump download data will be collected and reviewed and appropriate insulin dose adjustments made. Participants will discontinue use of the study CGM (Dexcom G6). Clinical observations will be recorded. Blood glucose levels and ketone bodies will be measured via a fingerstick blood sample (see Section 7). Supply of IMP will be given to last until the next visit. A 24 hr urine container will be dispensed to collect a 24 hour urine sample to bring in at visit 10 (Week 26) visit. A Meal Challenge Test will be performed as per visit 2. Participants will be provided with strips for urine and blood ketone measurement at home and instructed or reminded as appropriate on how to use these. They will be asked to measure urinary and blood ketones every morning for three consecutive days prior to the next visit (second randomisation: dapagliflozin or placebo) and to record the results in their diary.

Visit Procedures

Fasting visit*

Monitoring and treatment optimisation
- Check adequacy of contraception (WOCBP)
- Check for adverse events
- Review blood glucose diary
- Adjust insulin doses (if necessary)
- Blood ketones

Clinical assessments
- Blood pressure
- Heart rate
- Weight

Urine sample:
- Urine ketones
- 24 hour urine container dispensed

Other assessments
- Download Dexcom G6
- Meal Challenge Test

Blood assessments

- HbA1c

7.6 Second Randomisation Scheme

For the second randomisation (Period 2, double-blind), eligible and consenting participants previously randomized to open-label semaglutide will be randomised (1:1) to two groups (dapagliflozin or matching placebo) using permuted blocks of variable size to minimize predictability with stratification for HbA1c (≤ 7.5% vs >7.5% HbA1c) Those participants previously randomised to standard of care will continue as before.

7.6.1 Blinding (period 2 only)

For the double-blind treatment period, dapagliflozin and placebo will be packed in matching packs to maintain the blind. Each pack will contain sufficient supplies for 30 days' treatment with a small overage (excess). Packs will be labelled with a unique number that will be used to assign treatment to the participant via the IWRS system whilst maintaining the blind. Packs will be labelled in accordance with Good Manufacturing Practice and local regulatory requirements.

Unblinding. Treatment discontinuation (of dapagliflozin or placebo), rather than unblinding, will be carried out as far as possible. Participants will be advised in any case of hospitalisation with acute illness to discontinue study medication and inform the relevant clinician. These instructions will be reinforced in the Study Diary. However, where knowledge of treatment may assist emergency treatment, the investigator (or designee) may unblind using the IWRS system. Information on emergency unblinding (the investigational study drug, the study number, the investigator's name and a contact number) will be provided on the Patient Alert Card. For each unblinding an email alert is generated to the Study Coordinator and Chief Investigator. The most likely scenarios for unblinding will be: confirmed pregnancy, overdose/ accidental ingestion, or development of acute renal failure

7.6.2 Visit10 (Second Randomisation Visit; @26 weeks after First Randomisation)

will be asked to attend in the fasting state (no food or calorie intake for 10 hours). If participants arrive at the study visit in a non-fasted state, the visit will be terminated and a further fasting visit rearranged as soon as possible. Participants with normal fasting urine (<+) or blood ketone (<0.6 mmol) in the three days prior to randomisation will proceed to the next treatment phase as per this visit. The principles of the STICH Protocol (see Appendix 3) will be discussed (37). Participants with higher ketone values in at least one of the previous three days will have their glucose levels, insulin dose, and carbohydrate intake reviewed and advised on hydration, adequate CHO intake, and adequate insulin dosing. They will be rescheduled for one week and asked to repeat three days testing before the next visit.

Clinical observations will be recorded. A urine sample will be collected for ketones. Blood glucose and ketones will be measured via a fingerstick blood sample (see Section 7.2.1). Daily fingerstick blood glucose levels (diaries), CGM/ FGM data or insulin pump download data will be collected and reviewed and appropriate insulin dose adjustments made (following the guidance in Section 7, page 35). The previous three days blood or urine ketones levels will be reviewed. The adjusted total insulin dose at this visit will serve as the baseline total insulin dose for the remaining six months of the study and future limits of dose adjustments will be based on this new baseline. The blinded study CGM will be collected.

The 24 hour urine container will be collected. A fasting blood sample will be taken (45 mL) to evaluate study endpoints. Participants will be asked to complete quality of life questionnaires.

At this point, those participants previously randomised to receive open-label semaglutide will be randomised to receive dapagliflozin 5 mg daily or matching placebo. They will be educated on symptoms suggestive of low blood pressure (e.g. light-headedness, dizziness, feeling faint).

Participants will be asked to perform a daily (preferably early morning) urine (using CombiScreen® 10 SYS Plus urine testing strips or equivalent) or blood tests for ketone bodies (using the particpant's own meters as per Section 7, "Minimising ketosis": page 28) and record all outcomes in dedicated study diaries. If results of these scheduled (i.e. not done due to symptoms) ketone measurements, are +1 or higher (urine) or > 0.6 mmol/L (blood), participants will be instructed to follow the STICH Protocol as per their study diary and Appendix 3, . For any abnormal ketone levels taken due to symptoms, the DKA consensus guidelines are to be followed instead (see Section 9.3).

Participants will be instructed to call site staff directly in case of any problem or untoward side effects. They will be specifically asked to call if they have confirmed hypoglycaemia (blood glucose levels < 3.9 mmol/L) or hyperglycaemia (blood glucose > 13.9 mmol/L) occurring on more than one occasion. Participants will be advised to remain well hydrated and report any genital mycotic or urinary tract infections

Visit Procedures

Fasting visit* - Requirements and arrangements as per visits 2 and 3.

Monitoring and treatment optimisation

- Check adequacy of contraception (WOCBP)
- Check for adverse events
- Review blood glucose diary
- Adjust insulin doses (if necessary) - this insulin dose is baseline for Period 2
- Blood ketones

Clinical assessments
- Blood pressure
- Heart rate
- Body weight

Quality of life assessments
- Diabetes Specific Quality of Life Scale (DSQOLS)
- Problem Areas In Diabetes (PAID) questionnaire

Blood assessments
- Na, K, urea, creatinine, eGFR (MDRD)
- AST/ ALT/ Alkaline Phosphatase
- Calcium/ Phosphate
- FBC
- Fructosamine
- Haemoglobin
- White cell count

- Platelets
- Bilirubin
- Random glucose
- C-peptide (capillary blood glucose must be > 4.0 mmol/L at time of sampling)
-

Urine
- Pregnancy test (WOCBP randomised to IMP only)
- Urine ketones
- 24 hour urine bottle collected

Medication
- Education on symptoms suggestive of low blood pressure (e.g. light-headedness, dizziness, feeling faint) for those undergoing second randomisation
- Record doses of semaglutide self-injected since Visit 7 (self-report)
- Return used study pens
- Dispense open label semaglutide for participants randomised to active treatment at Period 1 (if required). Dispense dapagliflozin or placebo if randomised to active treatment

Other assessments
- Read masked Continuous Glucose Monitoring sensor (mean fasting blood glucose, mean blood glucose, percentage time in range, glycaemic variability)
-

7.6.3 Visit 11 (@1 week after Second Randomisation)

For those randomised to open-label semaglutide, doses administered will be recorded (self-report). Clinical observations will be recorded. Daily blood glucose and blood/ urine ketone levels will be reviewed and glucose levels collected. A urine sample will be collected to evaluate for ketones bodies. Blood glucose levels and ketones bodies will be reviewed measured via a fingerstick blood sample (see Section 7.2.1). . Daily fingerstick blood glucose levels (diaries), CGM/ FGM data or insulin pump download data will be reviewed and appropriate insulin dose adjustments made using the new baseline from Visit 10 (following the guidance in Section 7, page 35). If at any time total insulin dose reduction is >20% and total insulin dose is <0.40 U/Kg, additional monitoring (see Appendix 2) will be implemented. Unless blood (>0.6 mmol/L) or urine ketones (+ or more) have been elevated within the previous three days, dapagliflozin or placebo dose will be uptitrated to 10 mg daily (two capsules of either active or placebo medication) for the remainder of the study. Supply of IMPs will be given to last until week 39.

Visit Procedures

Monitoring and treatment optimisation
- Check adequacy of contraception.
- Check for adverse events
- Review blood glucose diary
- Adjust insulin doses (if necessary)
- Blood ketones
- Daily blood or urine ketones review

Clinical assessments
- Blood pressure
- Heart rate

- Body weight

Urine sample:

- Urine ketones

Medication

- Count returned dapagliflozin or placebo capsules
- Increase dose of dapagliflozin or placebo to 10mg (see above – review ketones)
- Dispense dapagliflozin or placebo (record dose)

7.6.4 Visit 12 (@5 weeks after Second Randomisation)

For those randomised to open-label semaglutide, doses administered will be recorded (self-report). Clinical observations will be recorded. Daily blood glucose and blood/ urine ketone levels will be reviewed and glucose levels collected. Blood glucose levels and ketone bodies will be measured via a fingerstick blood sample. A urine sample will be collected to evaluate ketone bodies and if appropriate pregnancy status. Insulin dose will be adjusted if as per Visit 11. A blood sample will be taken (15 mL) to evaluate study endpoints. A 24 hr urine container will be dispensed to collect a 24 hour urine sample to bring in at the Week 39 visit

Visit Procedures

Monitoring and treatment optimisation

- Check adequacy of contraception (WOCBP)
- Check for adverse events
- Review blood glucose diary
- Adjust insulin doses (if necessary)
- Blood ketones

Clinical assessments

- Blood pressure
- Heart rate
- Weight

Blood assessments

- Na, K, urea, creatinine, eGFR (MDRD)
- AST/ ALT/ Alkaline Phosphatase
- Calcium/ Phosphate
- FBC
- Haemoglobin
- White cell count
- Platelets
- Bilirubin
- Random glucose

Urine

- Pregnancy test (WOCBP randomised to IMP only)
- Urine ketones
- 24 hour urine container dispensed

7.6.5 Visit 13 (@13 weeks after Second Randomisation)

For those randomised to open-label semaglutide, doses administered will be recorded (self-report). Participants will be asked to attend in the fasting state. If participant is not fasting, samples should be taken and non-fasting status noted. Clinical observations will be recorded. Daily blood glucose and blood/ urine ketone levels will be reviewed and glucose levels collected. Blood glucose levels and ketone bodies will be measured via a fingerstick blood sample. A urine sample will be collected to evaluate ketone bodies and if appropriate pregnancy status. The 24 hour urine container will be collected. Insulin dose will be adjusted if needed as in as per Visit 11. Sufficient supply of IMPs will be given until the end of the study. A fasting blood sample will be taken (45 mL) to evaluate study endpoints.

Visit Procedures

Fasting visit* - as per previous fasting visits

Monitoring and treatment optimisation
- Check adequacy of contraception (WOCBP)
- Check for adverse events
- Review blood glucose diary
- Adjust insulin doses (if necessary)
- Blood ketones
- Daily urine ketones review

Clinical assessments
- Blood pressure
- Heart rate
- Weight

Quality of life assessments
- Diabetes Specific Quality of Life Scale (DSQOLS)
- Problem Areas In Diabetes (PAID) questionnaire

Blood assessments
- Na, K, urea, creatinine, eGFR (MDRD)
- AST/ ALT/ Alkaline Phosphatase
- Calcium/ Phosphate
- FBC
- HbA1c
- Fructosamine
- Haemoglobin
- Platelets
- Bilirubin
- Random glucose

Urine

- Pregnancy test (WOCBP randomised to IMP only)
- Urine ketones
- 24 hour urine bottle collected

Medication

- Record doses of semaglutide administered since Visit 10 (self-report)
- Dispense open label semaglutide for participants randomised to active treatment at Period 1 (if required).
- Count returned dapagliflozin or placebo capsules
- Dispense dapagliflozin or placebo (record dose)

7.6.6 Visit 14 (@23 weeks after Second Randomisation)

For those randomised to open-label semaglutide, doses administered will be recorded (self-report). Clinical observations will be recorded. Daily blood glucose and blood/ urine ketone levels will be reviewed and glucose levels collected. Blood glucose levels and ketone bodies will be measured via a fingerstick blood sample. A urine sample will be collected to evaluate ketone bodies and if appropriate pregnancy status. Insulin dose will be adjusted if as per Visit 11. All subjects will be reminded to monitor their capillary blood glucose by fingerstick at least four times a day (before meals and at bedtime). CGM (Dexcom G6) sensor will be fitted and patients asked to wear their CGM constantly for the next 20 days.

Visit Procedures

Monitoring and treatment optimisation

- Check adequacy of contraception (WOCBP)
- Check for adverse events
- Review blood glucose diary
- Adjust insulin doses (if necessary)
- Blood ketones
- Daily urine ketones review

Clinical assessments

- Blood pressure
- Heart rate
- Weight

Urine sample:

- Pregnancy test (WOCBP randomised to IMP only)
- Urine ketones

Clinical and functional assessment

- *Fit and start* masked Continuous Glucose Monitoring sensor (mean fasting blood glucose, mean blood glucose, percentage time in range, glycaemic variability)

7.6.7 Visit 15 (@25 weeks after Second Randomisation)

Participants will be asked to attend in the fasting state. If participants arrive at the study visit in a non-fasted state, the visit will be terminated and a further fasting visit rearranged as soon as possible. For those randomised to open-label semaglutide, doses administered will be recorded (self-report). Observations will be taken. Daily blood glucose and blood/ urine ketone levels will be reviewed and glucose levels collected. Blood glucose levels (fingerstick or FGM) and ketone bodies will be measured via a fingerstick blood sample. A urine sample will be collected to evaluate urine ketone levels. Insulin dose will be adjusted if needed as per visit 11. A 24 hr urine container will be dispensed to collect a 24 hour urine sample to bring in at the Week 52 visit. A HFHC Meal Challenge Test will be performed as in Visit 2.

Visit Procedures

Fasting visit* - Procedure as per previous fasting visits

Monitoring and treatment optimisation
- Check adequacy of contraception (WOCBP)
- Check for adverse events
- Review blood glucose diary
- Adjust insulin doses (if necessary)
- Daily urine ketones review
- Blood ketones

Assessments
- Blood pressure
- Heart rate
- Weight

Urine sample:
- Pregnancy test (WOCBP randomised to IMP only)
- 24 hour urine container dispensed

Other assessments
- Meal Challenge Test

7.6.8 Visit 16 (End of study; @26 weeks after Second Randomisation)

Participants will be asked to attend in the fasting state. If participants arrive at the study visit in a non-fasted state, the visit will be terminated and a further fasting visit rearranged as soon as possible. Clinical observations will be recorded. Daily blood glucose and blood/ urine ketone levels will be reviewed and glucose levels collected. Blood glucose levels and ketone bodies will be measured via a fingerstick blood sample. Insulin doses will be reviewed and likely requirement for an increase following discontinuation of IMP discussed with those taking open-label semaglutide. A urine sample will be collected to evaluate ketone bodies and, if appropriate pregnancy status. The CGM will be collected. The 24 hour urine container will be collected. A fasting blood sample will be taken (45 mL) to evaluate study endpoints. We will ask the participants to complete quality of life questionnaires. A physical examination will be performed as per the screening visit. This will be the final face-to-face study visit. A safety phone call will be conducted two weeks to review blood glucose data, provide further insulin

dosing advice, to document any adverse events, and to ensure that follow-up in usual clinical care has been arranged.

Visit Procedures

Fasting visit*

Monitoring and treatment optimisation

- Check for adverse events
- Review blood glucose diary
- Adjust insulin doses (if necessary)
- Blood ketones
- Daily urine ketones review

Clinical assessments

- Blood pressure
- Weight
- Heart rate
- Physical examination

Quality of life assessments

- Diabetes Specific Quality of Life Scale (DSQOLS)
- Problem Areas In Diabetes (PAID) questionnaire

Blood assessments

- Na, K, urea, creatinine, eGFR (MDRD)
- AST/ ALT/ Alkaline Phosphatase
- Calcium/ Phosphate
- FBC
- HbA1c
- Fructosamine
- Haemoglobin
- Platelets
- Bilirubin
- Random glucose
- C-peptide

Urine

- Pregnancy test (WOCBP randomised to IMP only)
- Urine ketones
- 24 hour collection

Other

- Record doses of semaglutide administered (self-report)
- Return used study pens
- Count returned dapagliflozin or placebo capsules

- Collect Patient Alert Card
- Read masked Continuous Glucose Monitoring sensor (mean fasting blood glucose, mean blood glucose, percentage time in range, glycaemic variability)

7.7 Retention and strategies for maximizing follow-up

Participants will be encouraged to attend all study visits. Prior to commencing the trial, all potential participants will have the opportunity to read the detailed Patient Information Sheet which will outline the level of participation requested. Potential participants will be made aware of the need for frequent study visits and asked to commit to these.

Study visits can be rearranged flexibly within a period around the planned visit as specified in the timeline of visits above (Section 7) and actual dates recorded in the eCRF. This is to maximise participant ability to attend all visits. A longer or shorter interval does not constitute a protocol deviation. Where possible, visits will be arranged to coincide with other appointments to further maximise attendance and participant convenience. Telephone contact will be frequent and will help to maintain contact with participants and ensure timely reminders of upcoming study visits can be given.

Even in the scenario of treatment discontinuation (of either IMP), participants will be encouraged to continue to attend further study visits as scheduled.

7.8 Treatment Interruptions and Withdrawal criteria

Withdrawal from study drug

Treatment interruption from study IMPs may be necessary in some participants. In the case of dapagliflozin, this will occur in any acute illness or hospitalisation which increases the risk of ketosis and ketoacidosis. Circumstances in which temporary discontinuation of dapagliflozin or placebo should occur will be discussed with all study participants. Written information will be provided in the study diaries. An emergency phone line to the study team will be available to all participants for the duration of their participation in the trial. This will be operational during office hours (Monday to Friday 9am – 5pm). The Patient Alert Card also contains contact details for unblinding in the event of acute intercurrent illness and a requirement for unblinding in the opinion of the acute medical team treating the participant. If any participant develops diabetic ketoacidosis (DKA) as defined as per the International Consensus guidelines on two occasions with no identifiable precipitant (section 2.1) they will be withdrawn from study medication and the UK Chief and US Principal Investigators informed.

Any other intolerable side effects or acute illness may require temporary discontinuation of the study drugs (dapagliflozin or semaglutide). In these circumstances, if the study drugs cannot be safely restarted in the judgement of the trial physician or if participants are unable to tolerate side-effects, they may be withdrawn from study treatment.

Treatment withdrawal may be necessary during the trial prior to the planned end at 52 weeks following first randomisation for any of the following reasons:

1. The site PI can stop investigational study treatment or remove participants from the trial without participants approval if for any reason he/she feels is appropriate, including: severe side effects, injury or medical condition which may place the patient at risk of further complications if he/she continue to participate, failure to take the medication as instructed, failure to keep scheduled appointments, insufficient level of self-monitoring, cancellation of the study or other administrative reasons.

2. Any woman who becomes pregnant during the period of study will be withdrawn from study treatment.

3. Participants who experience persistent severe nausea or vomiting after starting semaglutide which is not managed by dose reduction will discontinue the investigational study treatment. Those with mild or moderate nausea will continue for at least one more week. For those who continue to experience significant nausea (defined as interfering with normal activities), dose will be reduced: if these side effects persist, they will discontinue study medication(s). Participants for whom this occurs in Period 1 will remain eligible for randomisation in Period 2.

4. Excessive weight loss during either period of the study despite treatment dose reduction (> 15% body weight or BMI < 22 kg/m2 on repeated measurements more than four weeks apart).

5. New diagnosis of other conditions included in the Exclusion Criteria

6. Change in method of insulin delivery between insulin pumps to MDI or vice versa during the study unless pump treatment interruption is temporary for a period of at most 2 weeks. Participants who upgrade to “artificial pancreas” (e.g. Medtronic’s MiniMed 670G hybrid) during the study will be withdrawn.

7. Hypoglycaemia: Two episodes of severe hypoglycaemia (requiring external intervention).

8. Ketosis (with no other identifiable precipitating factors): Persistent (1 week) asymptomatic or symptomatic and unmanaged (using STICH protocol – see section 2.1 b) blood ketone levels >1.5 mmol (or ≥+2 in urine). (37)

9. Persistent (>1 week) total insulin dose reduction of >20% from baseline AND total insulin dose <0.4U/Kg.

10. DKA: Two episode of DKA (with no identifiable precipitating factors) as defined by the expert consensus statement (34)

11. Two episodes of UTI.

12. Two episodes of mycotic genital infection.

13. Significant persistent reduction in eGFR > 15 ml/min

14. Pancreatitis: any episode judged to be due to pancreatitis by the site PI.

15. Other persistent side effects specific to study drugs

Participation in this study is voluntary. Participants have the right to refuse to participate or to withdraw from participation at any time for any reason. The site PI can stop investigational study treatment or remove participants from the trial without participant's approval. If a patient withdraws consent and decides not to participate in the study, she/he will be asked to undergo a final study visit for his/her safety. If a subject withdraws from the research, the data collected to that point will be used toward the research finding. If applicable the subject will be asked to return any unused research drug and or devices.

Withdrawal from the study

If participants are unable or unwilling to attend all study visits they will be given an option of attending less frequently or only at the end of the study. We will request participants' consent to be contacted by telephone and for contact with their general practitioner or other health care provider to check on their current health status. No participant will be labelled as lost to follow-up. However, participants will have the right to withdraw consent for further participation and for further data collection: all other participants will be followed up for clinical events until study completion.

7.9 Storage and analysis of samples

Blood samples will be undertaken at the beginning and at various points during the study as outlined in detail in the schedule of visits. If blood testing has recently been undertaken in the 3 weeks prior to the study commencing, some of these blood tests may be used as baseline. Blood tests will include analysis of the full blood count, renal function (urea and electrolytes), liver function tests, bone profile, lipid profile and several specialised blood tests for biomarker assays (at central laboratory) following a high fat high caloric meal challenges to examine study endpoints.

Covid-19 Mitigation

All blood sampling or trial procedures will be undertaken following local and national guidance around personal protective equipment. Mask, gloves and aprons will be used as per current guidelines.

7.9.1 Sample collection and processing

- Samples will be appropriately labelled in accordance with the trial procedures to comply with the General Data Protection Regulation (GDPR). Biological samples collected from participants as part of this trial will be transported, stored, accessed and processed in accordance with national legislation relating to the use and storage of human tissue for research purposes and such activities shall at least meet the requirements as set out in the 2004 Human Tissue Act and the 2006 Human Tissue (Scotland) Act.
- Blood will be taken according to the Schedule of Assessments (page 17). Plasma will be siphoned, aliquoted and stored locally at -80° ± 10°C until transport in batches to the central laboratory on dry ice. according to the Sample Handling Procedure as agreed by the Joint Study Management Group
- Individual patient samples will be identified with a unique, anonymised study number.

7.9.2 Sample transport to central laboratory and analysis

- Samples will be transferred to State University of New York in a single batch at the end of recruitment to the study. More frequent transfer can be organised if there are local storage limitations.
- Samples will be transported on dry ice and stored at the central laboratory at -80° ± 10° until analysis. Transport by courier will coordinated by the Trial Manager.
- At the time of analysis individual aliquots will be defrosted at room temperature and analysed in a single batch for each biomarker of interest.
- Samples will be stored at -80° ± 10° in the central laboratory for possible future analysis for novel biomarkers.

7.10 End of trial

The end of the trial will be following the last study visit of the last subject.
At the end of the specified trial period (52 weeks following first randomisation) all participants will discontinue study medications and revert to standard of care (insulin therapy only). This process will be supervised by the trial physicians, research nurses, and research pharmacists under the supervision of the site Principal Investigator. The trial team will liaise with the usual care team to ensure a safe transition back to standard therapy. Following the final scheduled trial visit there will be a safety phone call two weeks after the last visit. This will act as a further opportunity to advise on insulin dosing and ensure there are no ongoing problems or concerns related to the study.

As discussed in section 7.8, there may be circumstances in which trial drugs need to be stopped before the specified end of the trial. If this is the case, the reason(s) for discontinuation will be recorded and the trial clinical team will facilitate safe transition back to usual standard of care. Participants will however if eligible (i.e non-exclusion criteria reason for IMP discontinuation) be asked to continue with trial visits to enable ongoing close clinical observation. If consent is withdrawn and participants voluntarily chose to leave the trial before the specified end date, all efforts by the clinical trial team will be made to safely facilitate transition back to ordinary standard of care. A follow up safety visit will be offered to all such participants and ongoing contact details will be made available should there be any further unforeseen issues relating to the clinical trial.

The trial may be stopped at an earlier stage than anticipated on the advice of the independent data monitoring committee (IDMC) to the trial steering committee (TSC) for the safety reasons detailed on page 12 and in the study safety endpoints. If this occurs, all participants will be informed and safe discontinuation of study medications will be undertaken under the supervision of the site Principal Investigator.

8 TRIAL MEDICATION

8.1 Name and description of investigational medicinal product(s)

a) Semaglutide (Ozempic®): a once weekly GLP-1 agonist injection indicated for type 2 diabetes (approved by the Scottish Medicines Consortium, January 2019).

   Doses to be used: 0.25mg increased to 0.5mg at two weeks and then to 1mg weekly s.c. injection after a further two weeks. Open label semaglutide will be added to a background of insulin treatment at week 0 for 12 months in 76 participants (38 in the UK) according to the

first randomisation; at week 26 they will be further randomised (double-blind) as below into two groups: dual (placebo, semaglutide and insulin) and triple (dapagliflozin, semaglutide and insulin) therapy.

b) Dapagliflozin (as dapagliflozin propanediol monohydrate Forxiga®): a once daily SGLT-2 inhibitor that will be administered orally in a blinded fashion.

   Doses to be used: 5 mg daily increased after one week to 10 mg daily (orally). Dapagliflozin will be started according to the randomisation (Interactive Web System, hosted by University of Glasgow) in 38 participants (19 in the UK) at 26 weeks as an additional treatment on a background of insulin and semaglutide therapy and end at 52 weeks (triple therapy group). Doses to be used: 5 mg increased to 10 mg after one week.

c) Placebo to dapagliflozin: Dapagliflozin matched placebo will be started in 38 participants (19 in the UK) participants at 26 weeks as an additional treatment on a background of insulin and semaglutide therapy and end at 52 weeks (dual therapy group). Doses to be used: as for active dapagliflozin.

8.2 Legal status of Semaglutide and Dapagliflozin

Semaglutide is currently licensed in the UK for those with adult type 2 diabetes which is insufficiently controlled.

8.3 Drug storage and supply

Semaglutide and dapagliflozin/placebo supplies must be stored in the original containers in a locked, secure area with access limited to authorised site staff. Study supplies should be used as directed in the study protocol and not be supplied to any persons other than study participants. All IMPs will be dispensed to participants from the study site pharmacy.

Covid-19 Mitigation

As detailed above in section 7, the supply of IMP may be altered to reduce risk of Covid-19 transmission in the event of future waves of infection. This may involve providing increased quantities of IMP to participants to last longer and therefore minimise physical contact with trial personnel. Use of a courier delivery service to facilitate this may also be considered.

Semaglutide: Supplies of semaglutide pre-filled pens for subcutaneous administration will be sourced from routine hospital stocks. The following semaglutide presentations will be required:

- Semaglutide 0.25 mg: Each pre-filled pen contains 2mg semaglutide in 1.5ml. Each dose contains 0.25mg of semaglutide in 0.19ml solution.
- Semaglutide 0.5 mg: Each pre-filled pen contains 2mg semaglutide in 1.5ml solution. Each dose contains 0.5 mg of semaglutide in 0.37ml solution.
- Semaglutide 1 mg: Each pre-filled pen contains 4 mg semaglutide in 3.0ml solution. Each dose contains 1mg semaglutide in 0.74ml solution.

For all presentations each pre-filled pen is capable of delivering 4 doses which is sufficient for 4 weeks treatment. Pens are for individual participant use only. In the event of a pen-failure, a new pen should be supplied to the participant.

Prior to first use, semaglutide pens must be stored at 2-8 °C in the original container and must not be frozen. Supplies which are frozen either prior to first use or during use must be discard.

Semaglutide pre-filled pens have an in-use shelf life of 6 weeks. After first use the pre-filled pens may be stored below 30oC or in a refrigerator at 2-8 °C. Participants should be advised to store packs away from the cooling element. The pen cap should be on when the pen is not in use in order to protect the contents from light.

Wherever possible, supplies of semaglutide pre-filled pens should be utilised in a pragmatic way so as to minimise unnecessary wastage. Participants should be provided with an appropriate number of pen(s) to last until the next planned visit. A small overage may be given as necessary to accommodate visit windows etc. The correct strength of pen must be supplied for each dose titration i.e. 0.25mg, 0.5mg and 1.0 mg as tolerated.

A web-based system will be used to randomise participants to semaglutide treatment. At randomisation and all subsequent visits (planned or unplanned), the system will notify pharmacy staff at study visits of the requirement for semaglutide supplies.

Dapagliflozin 5mg or placebo capsules: Supplies of dapagliflozin 5mg and matched placebo capsules will be manufactured by NHS Scotland Pharmaceuticals Specials Service/NHS Tayside Pharmaceuticals in accordance with Good Manufacturing Practice requirements. Capsules will be packed into HDPE bottles with a tamper evident seal. Each bottle will be labelled in a double-blind fashion to meet local regulatory requirements and will be assigned a unique number. Bottles containing dapagliflozin 5 mg or placebo must be stored at room temperature at 15-25°C.

Dapagliflozin 5mg and placebo capsule supplies will be provided to study site and will only be released by the sponsor once all the appropriate regulatory and governance approvals are in place. Further information on storage requirements and supply arrangements is provided in the study specific IMP Management and Accountability Manual.

A web-based system will be used for the assignment of dapagliflozin 5mg or placebo bottles at randomisation and at study visits where there is planned dispensing of the IMP or in the event that a participant requires replacement medicines.

Other medicines: Supplies of all other medicines and associated consumables will via usual NHS supply mechanisms.

8.4 Drug accountability requirements

The Investigator or designee must maintain accurate records of all study IMP movements for accountability purposes. They should include dates, quantities, batch numbers and expiry. Records must document adequately that:

- the patients were provided the doses specified by the protocol/amendment(s)
- all study drug provided was fully reconciled.

Unused study drug must not be discarded or used for any purpose other than the present study. Further information is provided in the study specific IMP Management and Accountability Manual.

8.5 Preparation and administration of semaglutide and dapagliflozin

Semaglutide (Ozempic®) is a solution for subcutaneous injection. It is administered once weekly, on the same day each week, into the subcutaneous tissues of the abdomen, thigh or upper arm. A participant may change the day of their weekly injection as long as it has been at least three days since their last injection. Thereafter, all doses should be administered once weekly on the new dosing day. The injection can be administered at any time of the day regardless of meals.

Participants must be advised to discard the injection needle after each injection and store the pen without an injection needle attached. This may help prevent blocked needles, contamination, infection, leakage of solution and inaccurate dosing. Semaglutide should not be used if the solution does not appear clear and colourless or almost colourless. The pen is designed to be used with NovoFine or NovoTwist disposable needles and pens are packed with an appropriate quantity of NovoFine Plus needles.

Detailed information on administration of semaglutide is provided in the current Summary of Product Characteristics and product Patient Information Leaflet.

Dapagliflozin (Forxiga®))and matched placebo are presented as a capsule for once daily oral administration.

8.6 Dosage schedules

Semaglutide will be administered and dosed as described. Participants will be commenced on 0.25 mg s.c once weekly before up-titration as tolerated to 1.0 mg s.c once weekly. Up-titration is quicker than is ordinarily the case in clinical practice in type 2 diabetes as discussed in Section 2.1 (22) There will be increased observation and clinical access for participants in the event of side-effects. Participants randomised to receive semaglutide will continue on the 1.0 mg s.c once weekly dose for the remainder of the study following uptitration provided they tolerate the dose as detailed in Section 7. If necessary, the dose may be adjusted.
If a participant misses a dose of semaglutide it should be administered as soon as possible and within 5 days after the missed dose. If more than 5 days have passed, the missed dose should be skipped, and the next dose should be administered on the regularly scheduled day. In both cases participants, should then resume their regular once weekly dosing schedule.

Dapagliflozin and matched placebo will be commenced at 5mg orally once daily and uptitrated one week after commencement to a dose of 10mg which will thereafter be continued for the remainder of the trial provided they tolerate the dose. If necessary, the dose may be adjusted.

8.7 Dosage modifications

Dapagliflozin/placebo will be commenced at one capsule (5mg or placebo) orally once daily and uptitrated one week after commencement to a dose of two capsules (10mg or placebo) which will thereafter be continued for the remainder of the trial provided they tolerate the dose. If necessary, the dose may be adjusted. (See also Section 7.8 regarding treatment discontinuation) Dapagliflozin or placebo capsules can be taken at any time of day with or without food. Capsules should be swallowed whole. If participants forget to take a dose they should be advised as follows:

- If it is 12 hours or more until the next dose, the dose should be taken as soon as remembered and the next dose taken at the usual time.
- If it is less than 12 hours until the next dose, the missed dose should be skipped. The next dose should be taken at the usual time.
- Participants should not take a double dose of dapagliflozin/placebo to make up for a forgotten dose.

8.8 Known drug reactions and interaction with other therapies

Participants with hypersensitivity to either active study medication are excluded from the trial. There are no significant interactions between semaglutide and other prescribed medications other than with agents that can lower blood glucose levels. Participants on any other oral agents which can lower blood glucose levels meet Exclusion Criteria and will not be included in this trial. In combination with insulin and dapagliflozin, semaglutide may be associated with an increased risk of hypoglycaemia. Participants will be advised of the increased risk of hypoglycaemia and will be required to closely monitor their blood glucose levels for the duration of the trial. No interactions are currently listed between warfarin and semaglutide in the British National Formulary but in view of a theoretical interaction (based on protein binding), existing arrangements for INR testing will be reviewed for any participants prescribed warfarin and additional tests conducted if indicated according to the judgement of the investigators.

Dapagliflozin is known to increase the risk of hypoglycaemia when combined with other medications which also act to lower blood glucose levels. Dapagliflozin has a mild diuretic effect and therefore there is an increased risk of hypotension when dapagliflozin is combined with either medications which deplete circulating volume (e.g diuretics) or antihypertensive drugs. Dapagliflozin is however commonly prescribed with such medications in general medical practice. All participants will have their medications reviewed at the screening visit and educated regarding symptoms that may indicate dehydration/ hypovolaemia.

Dapagliflozin may increase renal lithium excretion leading to decreased blood lithium concentrations. Serum lithium concentrations should be monitored for participants eligible for randomisation to dapagliflozin/placebo prior to visit 10 (week 26, second randomisation), as clinically indicated thereafter, and in the event of dose changes, taking into account the planned dose increase at visit 11 (week 27).

If there is any concern of interaction with any of the IMPs, this will be discussed with the PI before a decision regarding their ability to participate is made.

COVID-19: COVID-19 infection and the clinical management in this therapeutic area is also rapidly evolving as new medicines are approved for use by local/national treatment guidelines. It is therefore not possible to comprehensively rule out the potential for clinically significant interactions between the study IMP(s) with current or future medicines for the acute treatment of COVID-19 and a pragmatic approach is required. Medicines for acute COVID-19 infection should not be unnecessarily withheld and participants should be managed wherever possible in line with local/national clinical guidelines for this patient group. The decision to follow a particular management plan for acute COVID-19 infection (which may include the temporary withdrawal of the IMP(s) should this be in the participant's best interest), is at the treating physician's discretion. Investigators should refer to the protocol and current IMP Summary of Product Characteristics for detailed information on known mechanisms for pharmacokinetic/pharmacodynamics interactions as well as the relevant COVID-19 treatment Summary of Product Characteristics (or equivalent) to inform management decisions. Any mitigations, such as additional patient monitoring, must be documented in the participant's medical notes.

Further ad-hoc advice can also be obtained from the Sponsor/Chief Investigator and https://www.covid19-druginteractions.org/ Investigators must be vigilant at all times to the possibility of interactions and report in line with protocol pharmacovigilance requirements. Concomitant use of other medicines for COVID-19 must be recorded in the participant's CRF. Investigational treatments for acute COVID-19 treatment are not permitted without prior approval from the sponsor.

8.9 Concomitant medication

Participants should not receive any adjunct glucose-lowering therapy other than as per the randomisation schedule (e.g. with either of the active study medications).

8.10 Trial restrictions

Contraindications will follow SmPC information for Ozempic® and Forxiga. Acceptable forms of contraception for WOCBP participating in the trial are listed in Appendix 2. There is no requirement for contraception for male participants.

8.11 Assessment of compliance

Treatment adherence will be assessed by the trial medical and nursing team when participants attend for scheduled study visits.

8.12 Name and description of each Non-Investigational Medicinal Product (NIMP)

Insulin therapy is a non-investigational medicinal product in this trial. The trial is in a population who have had a diagnosis of type 1 diabetes for at least one year: therefore all participants will already be established on insulin therapy. The regimen and dosage required will by differ between participants. Participants will be using a combination of long acting (basal) insulin and shorter acting (bolus) insulin or CSII (insulin pump). As in standard of care, dose titration will depend on diet, blood glucose levels and exercise. It is anticipated that dosage adjustments will be required to achieve optimal glycaemic control and maintain participant safety.

There are pre-defined guidelines for insulin titration in this study. Insulin dose reductions should not be greater than 10% (5% if HbA1c is<7%) from current total insulin dose or more than 20% from the baseline level at randomisation and then from the new baseline levels at 26 weeks following second randomisation. Participants requiring an insulin dose reduction > 20% to a total insulin <0.4 U/kg will be monitored closely for insulin dose, carbohydrate intake, ketones and blood glucose. Study medication can be temporarily discontinued, if necessary, for one-to-two weeks. If low insulin requirements remain, the participant will be withdrawn from study (see Section 7.8).

9 PHARMACOVIGILANCE

9.1 Definitions

Term	Definition
**Adverse Event (AE)**	Any untoward medical occurrence in a participant to whom a medicinal product has been administered, including occurrences which are not necessarily caused by or related to that product.
**Adverse Reaction (AR)**	An untoward and unintended response in a participant to an investigational medicinal product which is related to any dose administered to that participant.<br>The phrase "response to an investigational medicinal product" means that a causal relationship between a trial medication and an AE is at least a reasonable possibility, i.e. the relationship cannot be ruled out.<br>All cases judged by either the reporting medically qualified professional or the Sponsor as having a reasonable suspected causal relationship to the trial medication qualify as adverse reactions.
**Serious Adverse Event (SAE)**	A serious adverse event is any untoward medical occurrence that:<br>1. results in death<br>2. is life-threatening<br>3. requires inpatient hospitalisation or prolongation of existing hospitalisation<br>4. results in persistent or significant disability/incapacity<br>5. consists of a congenital anomaly or birth defect<br><br>Other 'important medical events' may also be considered serious if they jeopardise the participant or require an intervention to prevent one of the above consequences.<br>NOTE: The term "life-threatening" in the definition of "serious" refers to an event in which the participant was at risk of death at the time of the event; it does not refer to an event which hypothetically might have caused death if it were more severe*.
**Serious Adverse Reaction (SAR)**	An adverse event that is both serious and, in the opinion of the reporting Investigator, believed with reasonable probability to be due to one of the trial treatments, based on the information provided.
**Suspected Unexpected Serious Adverse Reaction (SUSAR)**	A serious adverse reaction, the nature and severity of which is not consistent with the information about the medicinal product in question set out:<br>• in the case of a product with a marketing authorisation, in the summary of product characteristics (SmPC) for that product<br>• in the case of any other investigational medicinal product, in the investigator's brochure (IB) relating to the trial in question
**Reference Safety Information**	The information used for assessing whether an adverse reaction is expected. This is contained in either the investigator's brochure or the summary of product characteristics

*Note: "Severe" is often used to describe intensity of a specific event, which <u>may</u> be of relatively minor medical significance. "Seriousness" is the regulatory definition supplied above.

9.2 Operational definitions for (S)AEs

Adverse events (AEs) will be recorded, notified, assessed, reported, analysed and managed in accordance with the Medicines for Human Use (Clinical Trials) Regulations 2004 (as amended) and this protocol.

All Serious Adverse Events (SAEs) occurring during the trial will be recorded within the eCRF. Non-serious AEs occurring during the trial will be recorded within the eCRF if they are considered to be related to trial medication, i.e. adverse reactions (ARs).

Exclusions from SAE reporting

Hospitalisation for the following reasons will not be considered to be SAEs:

- Treatment which was elective or pre-planned, for a pre-existing condition not associated with any deterioration in condition, e.g. pre-planned hip replacement operation which does not lead to further complications.
- Any admission to hospital or other institution for general care where there was no deterioration in condition.
- Treatment on an emergency, outpatient basis for an event **not** fulfilling any of the definitions of serious as given above and not resulting in hospital admission.
- COVID-19 infection unless results in severe illness with hospital admission.

9.3 Recording and reporting of AEs, Adverse Events of Special Interest, SAEs and SUSARs

All AEs occurring during the trial that are observed by the Investigator or reported by the participant will be recorded in the participant's medical records whether or not attributed to trial IMP. AEs will be recorded from randomisation until the later of 30 days post cessation of trial treatment or the end of the study.

With the exception of those noted in section 9.3.1 below, adverse reactions will be recorded in a log provided in the eCRF.

Adverse Events of Special Interest, SAEs and SUSARs will be recorded in the participant's medical records and in a specific section of the eCRF from the point of randomisation until the later of 30 days post cessation of trial treatment or the end of the study.

9.3.1 Adverse Events of Special Interest

Ketosis is a known adverse effect of dapagliflozin (see Section 2.1b; also (i) below). Hypoglycaemia is a recognised adverse effect with both semaglutide and dapagliflozin when used in combination with insulin [see (ii) below] and is defined as moderate in intensity (Level 2) when blood glucose is < 3.0 mmol/L. Thus ketosis and Level 2 hypoglycaemia events will be recorded as Adverse Events of Special Interest (AESIs).

AESIs will be recorded within the relevant section of the eCRF but do not require reporting in an expedited manner (unless the event also meets the regulatory definition of an SAE). The following information will be recorded:

- Nature of the event
- Event duration (start and end dates, if applicable
- Relationship to study drug
- Outcome

Adverse Events of Special Interest will be monitored and followed up (if applicable) until the event has resolved or a final outcome has been reached.

All Adverse Events of Special Interest must be assessed for seriousness prior to recording within the eCRF. Those meeting the definition of an SAE should also be reported accordingly as per Section 9.3.2.

(i) Ketosis and diabetic ketoacidosis

This trial will be conducted in participants who have type 1 diabetes. All participants will be taking insulin. In this condition, due to the near absence of endogenous insulin, at a cellular level there is an inability to incorporate glucose into normal intra-cellular energy yielding pathways. In this context, alternative energy yielding pathways are sought including ketone body production from fatty acids in the liver. Ketones are clinically important as they easily dissociate hydrogen ions upsetting physiologically regulated acid base balance. As ketone levels rise, there is initially a state of high blood ketone levels without significant acidosis (ketosis); if this develops further, diabetic ketoacidosis (DKA) may develop. DKA requires urgent medical input. Ketones can be easily measured via a simple urine test or fingerstick capillary blood measurement. Participants eligible for this trial will already be trained in how to do this. As discussed in Background ((1) above), dapagliflozin, one of the IMPs in this trial, is known to increase the risk of ketosis and ketoacidosis and this can occur at lower blood glucose levels than in those who are on insulin treatment only [euglycaemic DKA] (13,14). Given this known risk, ketosis and DKA are included in the safety endpoints and as such will be recorded in the eCRF as Adverse Events of Special Interest.

DKA is defined clinically and biochemically as a combination of a state of elevated ketone levels (urine ++ or higher, and/or blood > 1.5 mmol/L) in combination with biochemical evidence of a state of metabolic acidosis (pH ≤7.30 or serum bicarbonate < 15 mmol/L). This is as defined by International Consensus Guidelines (34). DKA also varies in severity. In clinical practice the diagnosis is made when a person with type 1 diabetes has elevated ketone levels and usually – although not exclusively – elevated blood glucose levels. The diagnosis is made biochemically by assessing acid base balance through blood testing. This corresponds to the following severity classifications:

- mild DKA: pH 7.25-7.30 (H^+ 50 – 56) or bicarbonate (15 -17.9 mmol/L)
- moderate DKA: pH 7.00-7.24 (H^+ 57 – 100) or bicarbonate 10 – 14.9 mmol/L)
- severe DKA: pH <7.00 (H^+ > 100) or bicarbonate < 10 mmol/L (39)

In addition to the above, the highest level of either blood or urine ketones measured, the serum bicarbonate (if measured) and blood pH (if measured) will be recorded for each episode. Given the known risk of DKA, all participants will be made aware of this condition. Patient diaries and Patient Alert Cards will summarise advice for management of high ketone levels including an emergency ketosis management protocol (STICH Protocol – see Appendix 3) (37). All participants will be educated as to the scenarios when this should be activated and this will also be included on the Patient Alert Card which will have the contact details of the study team and also contact details for out of hours health services. Participants will be asked to carry this card at all times.

(ii) Hypoglycaemia

Hypoglycaemia is a low blood glucose level. Hypoglycaemia occurs in diabetes owing to difficulty in precisely matching insulin dosing with the complex factors associated with carbohydrate intake and physical activity. When too much insulin relative to carbohydrate intake is administered, blood glucose levels can drop to a level which is too low. Most people with type 1 diabetes can recognize this phenomenon due to a combination of symptoms which alert them and prompt self-checking of their capillary blood glucose level. Symptoms include weakness, malaise, headache, tremulousness, diaphoresis, palpitations and mild confusion. These are known as autonomic symptoms as they are mediated by activation of the autonomic nervous system as a response to the biological stress of low serum blood glucose. As hypoglycaemia progresses, however, symptoms progress to include confusion and agitation. If untreated this can lead to coma. These symptoms are known as neuroglycopenic symptoms as they are mediated by a lack of glucose in neural cells leading to a confused state.

Hypoglycaemia is also well defined by International Consensus (34). Like DKA, it can be classified in terms of severity. This can be based either on the absolute capillary glucose value and/or clinical features and is classed into stages:

- Level 1 (mild) hypoglycaemia: Blood glucose is 3.0 – 3.8 mmol/L inclusive. At each visit the number of episodes since the last visit during which blood glucose was within this range (but not lower) will be recorded on the “Fasting Blood Glucose” tab of the eCRF following review of the participant’s study diary and (if applicable) FGM/ CGM download.
- Level 2 (moderate) hypoglycaemia: Blood glucose is < 3.0 mmol/L At each visit the number of episodes since the last visit during which blood glucose was within this range at any time will be recorded on the AESI tab of the eCRF following review of the participant’s study diary and (if applicable) FGM/ CGM download.
- Level 3 (severe) hypoglycaemia: A person is unable to function because of mental or physical changes due to hypoglycaemia and requires assistance from another person to recover. These events will be recorded as SAEs (see below) independent of recording of blood glucose according to the opinion of the site Principal Investigator as by definition they jeopardise the participant or require an intervention to prevent life-threatening consequences.

The IMPs in this trial are licensed clinically in type 2 diabetes to lower blood glucose levels. It therefore follows that when used in combination with insulin there may be an increased risk of low blood glucose levels. This was the case with the once daily GLP-1 analogue liraglutide in phase 3 trials (8,9), but not in the trials with dapagliflozin (13,14). All participants will have this risk discussed with them, will be performing increased blood glucose monitoring than is the case in usual care, and will be frequently reviewed to adjust insulin dosing in order to mitigate the risk of low blood glucose levels.

9.3.2 Site Reported Serious Adverse Events (SAE)

SAEs will be recorded within the eCRF from the date of randomisation until the later of 30 days post cessation of trial treatment or the end of the study.

Full details of SAEs will be recorded in the electronic Case Report Form. The following information will be collected:

- full details in medical terms and a case description
- seriousness criteria
- event duration (start and end dates, if applicable)
- action taken
- outcome
- causality (i.e. relatedness to trial drug / investigation), in the opinion of the investigator

- if related, whether the reaction would be considered expected or unexpected.

Any change of condition or other follow-up information should be added to the eCRF and forwarded to the Sponsor (if reportable SAE) as soon as it is available or at least within 24 hours of the information becoming available. Events will be followed up until the event has resolved or a final outcome has been reached.

Reporting of suspected/confirmed COVID 19 infection

If a patient presents with clinical signs and symptoms consistent with COVID-19 and a test has been performed or requested:

- If test is positive, record “COVID-19 confirmed” in the diagnosis field
- If test is negative, record the SAE signs and symptoms and/or other diagnosis in the diagnosis field.

If a test has not been performed or result is not available and signs and symptoms, as judged by the investigator, are highly suspicious of COVID-19 infection, record “COVID-19 suspected” in the diagnosis field

If other concurrent diagnoses, e.g. pneumonia, please record within the narrative of the SAE.

COVID-19 vaccination and reporting

Where a deployed COVID-19 vaccine is suspected to be involved in the onset of a reported event it should be recorded as a concomitant medication. A causal relationship between the vaccine and the event, including potential drug interactions should be assigned by the reporting investigator.
If a reported event is suspected to be due to a deployed COVID-19 vaccine alone reporting investigators should ensure that standard Yellow Card reporting procedures are followed.

Assessment of Adverse Events

All adverse events must be assessed by the reporting site for seriousness**.** All SAEs must also be assessed for causality and expectedness with reference to this protocol and the Reference Safety Information (RSI).

Assessment of seriousness

An adverse event will be considered serious if it:

1. Results in death
2. Is life threatening
3. Requires hospitalisation or prolongation of existing hospitalisation
4. Results in persistent or significant disability or incapacity
5. Consists of a congenital anomaly or birth defect
6. Required intervention to prevent one of the above.
7. Is a serious Adverse Event of Special Interest.

Assessment of causality i.e. does the event have a “reasonable causal relationship” with trial IMPs. The following categories are used:

- **Yes** - The causal and temporal relationship between the event and the absence of a more likely explanation suggest that the trial IMP is the most likely cause.
- **No** - The event is not considered related to the trial IMPs.

Causality will be assigned for each IMP the participant is randomised to receive.

Should an SAE be considered unrelated to the administration of either trial drug then the causality of the event will be collected taking into consideration other factors, for example concomitant medications or co-morbidities, etc.

Assessment of causality is the responsibility of the Principal Investigator or medically-qualified designee(s) at each site. The CI, their delegate(s), or the Sponsor may provide an opinion regarding causality but cannot downgrade the PI's assessment.

Assessment of expectedness

Should the event be considered to be related to the trial IMP(s), an assessment should be made regarding the expectedness of the reaction, i.e. whether the reaction is a recognised adverse effect of the IMP(s).

.
Expected: consistent with the relevant product information documented in the RSI(s).
Unexpected: not consistent with the relevant product information documented in the RSI(s).

The CI, their delegate(s), or the Sponsor have overall responsibility for assigning expectedness as per the RSI(s) and provide the final assessment of the event. The reporting investigator may provide an opinion on the expectedness of an event and this may be taken into account.

SAEs occurring within the USA and the UK

The US Sponsor is responsible for assigning causality and expectedness for any AEs, SAEs and SUSARs that occur within the USA. Where a SUSAR is identified by the US Sponsor this will be forwarded to the UK Sponsor for review within one working day. Following review by the UK Sponsor, appropriate US SUSARs will be subject to expedited reporting to the MHRA as per UK regulatory requirements. Similarly, where a SUSAR is identified by the UK sponsor, this will forwarded to the US Sponsor for review within two working days.

Recording and reporting of SAEs

All SAEs arising during the clinical trial will be recorded in the eCRF soon as reasonably practicable and in any event within 24hours of first becoming aware of the event. Any follow-up information should also be reported.

If recording in the eCRF is not possible a paper SAE form should be completed:

1. The SAE form is downloaded from www.glasgowctu.org, printed off, completed and signed. The form is then faxed to the Glasgow Clinical Trials Unit Pharmacovigilance (PV) Office on +44(0)141 357 5588. As an alternative, a scanned copy of the SAE form may be emailed to: pharmacovig@glasgowctu.org. If this website is unavailable a paper copy of the SAE form should be filed in the Investigator Site File at each site pending resolution.
2. If necessary a verbal report can be given by contacting the PV Office on +44(0)141 330 4744. This must be followed up as soon as possible with an electronic or written report.

Reporting to sponsor

All SAEs will be considered reportable to the Sponsor PV office from the time of randomisation until 30 days following permanent discontinuation of all trial IMP(s). SAEs occurring following 30 days post cessation of treatment are not reportable to the Sponsor unless an SAE is considered related to IMP(s)

9.3.3 Suspected Unexpected Serious Adverse Reactions (SUSARs)

Any SAE assessed as related to trial IMP(s) by the PI or delegate, and unexpected (not documented as an expected reaction to the IMP(s) within the RSI(s)) by the CI, their delegate, or the Sponsor, will be classified as a SUSAR and therefore subject to expedited reporting to the Medicines and Healthcare products Regulatory Agency (MHRA) and the Research Ethics Committee (REC). Should the CI disagree with the PI's assessment of causality, both opinions will be provided on the report. Where the CI or UK Sponsor disagrees with the US Sponsor's assessment of expectedness both opinions will also be provided on the report.

The Sponsor will inform the MHRA of all SUSARs that occur within the trial and the REC of all SUSARs that occur within the UK within the required expedited reporting timescales:

- **Fatal or life threatening SUSARs** - no later than 7 days of Sponsor awareness that the case fulfils the criteria for a fatal or life threatening SUSAR, and any follow up information within a further 8 days.
- **All other SUSARs** - no later than 15 days of Sponsor awareness that the case fulfils the criteria for a SUSAR.

The sponsor will report SUSARs to the MHRA via the MHRA eSUSAR reporting system and to REC by email with accompanying CTIMP Safety Report Form.

9.3.4 Unblinding for SUSAR reporting purposes

In the event of a potential SUSAR, the sponsor (but not the investigators) will be unblinded to facilitate appropriate reporting to the MHRA and REC.

9.4 Responsibilities for Safety Reporting and Review

This section details the responsibilities for reporting and reviewing safety information arising from the trial.

Principal Investigator (PI):
- Attendance at the initiation meeting/teleconference
- Training of new members of the trial team in the protocol and its procedures
- Ensuring that the ISF is accurately maintained
- Dissemination of important safety or trial-related information to all stakeholders at their site
- Checking for AEs and ARs when participants attend for treatment/follow-up
- Ensuring that AEs are recorded and reported in line with the requirements of the protocol
- Ensuring that all SAEs are recorded and reported to the Sponsor within 24 hours of becoming aware of the event, and providing further follow-up information as soon as available
- Using medical judgement in assigning causality of SAEs

- Providing an opinion on the expectedness of an SAE with reference to the trial protocol and Reference Safety Information
- Using definitions in this protocol, flagging events of special interest.

Chief Investigator (CI)

- Providing clinical oversight of the safety of patients participating in the trial, including an ongoing review of the risk/benefit
- Providing clinical review of section 4.8 of the representative SmPCs on an annual basis to define the Reference Safety Information for the trial
- Using medical judgement to confirm seriousness and causality of all SARs
- Reviewing expectedness of related SAEs with reference to the trial protocol and Reference Safety Information
- Preparing the clinical sections and final sign-off of the Development Safety Update Report (DSUR)
- Using definitions in this protocol, confirming events of special interest

Sponsor:

- Central data collection and verification of AEs, SAEs, SARs and SUSARs according to the trial protocol
- Reporting safety information to the CI or delegate for the ongoing assessment of the risk / benefit
- Reporting safety information to the independent oversight committees identified for the trial (Independent Data Monitoring Committee (IDMC) and / or Trial Steering Committee (TSC)) according to the Trial Monitoring Plan.
- Expedited reporting of SUSARs to the Competent Authority (MHRA in UK) and REC within required timelines.
- Notifying Investigators of SUSARs that occur within the trial.
- Checking for (annually) and notifying PIs of updates to the Reference Safety Information for the trial.
- Preparing standard tables and other relevant information for the DSUR in collaboration with the CI and ensuring timely submission to the MHRA and REC.

Trial Steering Committee:

In accordance with the Charter for the TSC, periodically reviewing recruitment and the overall progress of the trial and liaising with the IDMC and sponsor regarding safety issues.

Independent Data Monitoring Committee:

In accordance with the Charter for the IDMC, periodically reviewing unblinded safety data in individual cases and to determine patterns and trends of events, or to identify safety issues, which would not be apparent on an individual case basis, reporting concerns to the TSC and sponsor.

9.5 Pregnancy reporting

Pregnancy is not considered an AE unless a negative or consequential outcome is recorded for the mother or child/foetus. If the outcome meets the serious criteria, this would be considered an SAE and must be reported as per SAE reporting procedure above.

Any **pregnancy** occurring in a female trial participant or female partner of a male trial participant who becomes pregnant while participating in the Trial will be reported by the PI (or designee) to the Chief Investigator and the sponsor using the sponsor Pregnancy Reporting Form (available at http://www.glasgowctu.org/) within two weeks of the PI first becoming aware of the pregnancy.

The trial participant will also be followed up to determine the outcome of the pregnancy and follow-up information forwarded to the PV office. Any resulting SAEs should be reported as per SAE reporting procedure above.

9.6 Overdose

Any IMP dose which is not administered in accordance with the Protocol should be reported to the sponsor.
If an SAE is associated with an overdose ensure that the overdose if fully described in the SAE report form.

9.7 Reporting urgent safety measures

If any urgent safety measures are taken the CI/Sponsor will phone the MHRA's Clinical Trial Unit on 020 3080 6456, ideally within 24 hours. This will be followed up no later than 3 days from the date the measures are taken, giving written notice to the MHRA (who will advise the format required) and the relevant REC of the measures taken and the circumstances giving rise to those measures. A substantial amendment must also be submitted to the MHRA.

9.8 The type and duration of the follow-up of participants after adverse events.

Adverse events and reactions will be recorded, reported and followed up in line with this protocol until study completion or for a minimum of 30 days after participant's last dose of the IMP, whichever is later. Any SUSAR identified will be reported to the Sponsor and to the Regulatory Authorities irrespective of how long after IMP administration the reaction has occurred.

9.9 Development safety update reports

A Development Safety Update Report (DSUR) will be submitted once a year, or on request, to MHRA and REC until the trial is declared ended. The report will be submitted within 60 days of the anniversary of the issue of the Clinical Trials Authorisation for the trial. The DSUR will be prepared by the sponsor (PV Office) in liaison with the CI and submitted by the sponsor (PV Office). The DSUR will include only those SAEs reported to the co-sponsors; that is: SAEs and SUSARs occurring within the UK and SUSARs originating in the USA.

10 STATISTICS AND DATA ANALYSIS

10.1 Sample size calculation

This is based on our current data showing that in type 1 diabetes, treatment with a GLP-1 agonist, liraglutide (1.2 and 1.8mg doses) for three months leads to HbA1c suppression by an average of 0.6% (SD= 0.5%). Dapagliflozin was recently shown to reduce HbA1c by 0.45% in type 1 diabetes patients (13). Additionally, empagliflozin treatment for 8 weeks was shown to reduce HbA1c by 0.4% in type 1 diabetics (20, 21) and our current data showing additional improvement in HbA1c by an additional 0.66% at 12 weeks when dapagliflozin was added to liraglutide and insulin therapy. Conservatively estimating that the combination therapy will suppress HbA1c by 0.5% (SD≤0.7%) compared to semaglutide alone, a sample size of 38 (32 evaluable patients) in each group will be sufficient to provide 80% power at a 5% significance level, taking in consideration a 15% dropout rate. 50% of participants will be recruited in Glasgow, UK and 50% at State University of New York at Buffalo, USA.

10.2 Anticipated recruitment rate

One year (one participant per week).

10.3 Statistical analysis

The primary objective of the proposed research is to evaluate the effect of addition of dapagliflozin to semaglutide on a background of insulin treatment in type 1 diabetes patients on HbA1c at six months following start of dapagliflozin. Transformations of the data in order to meet statistical assumptions will be considered. Statistical analyses will use linear regression models to compare treatment groups, with adjustment for baseline values of study outcomes, as well as factors used to stratify the randomisation scheme. Tests will be two-sided and tested at the 5% significance level. Analyses will be carried out under the intention to treat principle, that is, according to randomised treatment allocation, regardless of treatment adherence.

A detailed Statistical Analysis Plan will be completed and signed off before database lock. Study statisticians will develop and validate the analysis programs without access to randomised treatment allocations. After database lock, the treatment codes will be released and the final analysis will be carried out.

10.4 Subgroup analyses

These will be specified in the Statistical Analysis Plan. Subgroup analyses will be implemented by extending regression models to include interactions between treatment and potential treatment effect moderators.

10.5 Interim analysis and criteria for the premature termination of the trial

No interim analysis is planned. Unblinded trial data will be reviewed on an ongoing basis by the IDMC. The primary role of the IDMC will be to protect the interests of the patients. The IDMC may recommend to the TSC and Co-Sponsors that the study should stop prematurely because of concerns about patient safety. The IDMC will meet approximately every six months. The IDMC will take into account all results and the consistency and biological plausibility of the findings in making any recommendation. The final decision on continuing or stopping the trial will lie with the TSC/Co-Sponsors.

10.6 Subject population

All analyses will be carried out on an intention to treat basis, using the randomised treatment allocation.

10.7 Procedure(s) to account for missing or spurious data

Data will be analysed without imputation in the first instance. Multiple imputation procedures will be used to assess the sensitivity of selected results to missing data, according to the Statistical Analysis Plan.

10.8 Other statistical considerations.

The Statistical Analysis Plan (SAP) will be maintained as a version controlled document and will be signed off before database lock. The SAP and other statistical documentation (according to Glasgow Clinical Trials Unit Standard Operating Procedures) will contain full details of all analyses along with assumptions and procedures for handling problematic or incomplete data (e.g. incomplete dates).

11 DATA HANDLING

11.1 Source Documentation

ICH GCP defines source data as: 'All information in original records and certified copies of original records of clinical findings, observations or other activities in a clinical trial necessary for the reconstruction and evaluation of the trial'. In this study, the location of the majority of the source data will be the hospital's medical records including subject case notes, laboratory records and ECGs. The source data transcribed into the eCRF from the medical records must be accurate and verifiable. For questionnaires completed by trial subjects, the completed questionnaires will be regarded as the source data location. In cases where data is transcribed directly into the eCRF and no other paper or electronic source exists, then the eCRF will be considered the source record. In these cases, these data should be prospectively documented in the medical records to ensure a full record of the trial is available at site.

11.2 Data collection

An eCRF, developed by the Robertson Centre for Biostatistics, will capture all data required to meet this protocol's requirements. Access to the eCRF will be restricted, via a study-specific web portal, and only authorised site-specific personnel will be able to make entries to their patients' data via the web portal. The Investigator, or his/her designee will be responsible for all entries into the eCRF and will confirm that the data are accurate, complete and verifiable. Data will be stored in a MS SQL Server database. Direct access to the web portal will be granted, on request, to authorised representatives from the Sponsor, host institution and the regulatory authorities to permit trial-related monitoring, audits and inspections.

11.3 Data Validation

Where it is practical, data will be validated at the point of entry into the eCRF. Any additional data discrepancies will be flagged to the investigator and any data changes will be recorded to maintain a complete audit trail (reason for change, date change made, who made change).

11.4 Data Security

The Robertson Centre for Biostatistics systems are fully validated in accordance with industry and regulatory standards, and incorporate controlled access security. High volume servers are firewall protected and preventative system maintenance policies are in place to ensure no loss of service or data. Web servers are secured by digital certificates. Data integrity is assured by strictly controlled procedures, including secure data transfer procedures. Data are backed up on-site nightly and off-site to a commercial data vault weekly. The Robertson Centre for Biostatistics has an ISO 9001:2008 quality management system and ISO 27001:2013 for Information Security, and is regularly inspected against the standards by the British Standards Institution.

11.5 Archiving

The Trial Master File will be archived by the Co-Sponsors at the end of the trial for a minimum period of five years.

Archiving of Site Files will also be for a minimum of seven years fromm completion of the trial, and this action will be delegated to the sites in the Clinical Trial Site Agreement that will be put in place between Co-Sponsors and Sites. Sites will be notified by the Co-Sponsors when Site files can be archived.

Destruction of site files can only take place with the approval of the Co-Sponsors.

12 MONITORING, AUDIT & INSPECTION

Monitoring will be conducted by NHS Greater Glasgow and Clyde (GG&C) Monitor (s) in accordance with local Standard Operating Procedures. The level, frequency and priorities of monitoring will be based on the outcome of the completed Risk Assessment, and clearly documented in the Monitoring Plan which will be approved by the NHS GG&C Lead Monitor and/or Research Governance Manager.

13 ETHICAL AND REGULATORY CONSIDERATIONS

13.1 Research Ethics Committee (REC) review & reports

Before the start of the trial, approval will be sought from a REC for the trial protocol, informed consent forms and other relevant documents e.g. advertisements and GP information letters.
Substantial amendments that require review by REC will not be implemented until the REC grants a favourable opinion for the study (it is noted that amendments may also need to be reviewed and accepted by the MHRA and/or NHS R&D departments before they can be implemented in practice at sites).
All correspondence with the REC will be retained in the Trial Master File/Investigator Site File.
An annual progress report (APR) will be submitted to the REC within 30 days of the anniversary date on which the favourable opinion was given, and annually until the trial is declared ended (this is the Chief Investigator's responsibility).
The Chief Investigator will notify the REC of the end of the study.
If the study is ended prematurely, the Chief Investigator will notify the REC, including the reasons for the premature termination
Within one year after the end of the study, the Chief Investigator will submit a final report with the results, including any publications/abstracts, to the REC.

13.2 Peer review

The study protocol has been developed with expert and independent feedback from the JDRF (New York) and during application for funding underwent peer review by independent experts.

13.3 Public and Patient Involvement

The study was designed with input from people with type 1 diabetes via the funder, JDRF.

13.4 Regulatory Compliance

The trial will not commence until a Clinical Trial Authorisation (CTA) is obtained from the MHRA.
The protocol and trial conduct will comply with the Medicines for Human Use (Clinical Trials) Regulations 2004 and any relevant amendments.
Before any site can enrol patients into the trial, the Chief Investigator/Principal Investigator or designee will apply for NHS permission from the site's Research & Development (R&D) department.

For any amendment that will potentially affect a site's NHS permission, the Chief Investigator/ Principal Investigator or designee will confirm with that site's R&D department that NHS permission is ongoing (note that both substantial amendments, and amendments considered to be non-substantial for the purposes of REC and/or MHRA may still need to be notified to NHS R&D).

13.5 Protocol compliance

Prospective, planned deviations or waivers to the protocol are not allowed under the UK regulations on Clinical Trials and must not be used e.g. it is not acceptable to enrol a participant if they do not meet the

eligibility criteria or restrictions specified in the trial protocol. Accidental protocol deviations can happen at any time. They must be adequately documented on the relevant forms and reported to the Chief Investigator, Sponsor and GCTU immediately. Deviations from the protocol which are found to frequently recur are not acceptable, will require immediate action and could potentially be classified as a serious breach.

13.6 Notification of Serious Breaches to GCP and/or the protocol

A “serious breach” is a breach which is likely to affect to a significant degree –

1. the safety or physical or mental integrity of the subjects of the trial; or
2. the scientific value of the trial

If any of the above occurs then the CI and Sponsor will be notified. The sponsor will notify the appropriate authorities in writing of any serious breach in accordance with their standard operating procedures.

13.7 Data protection and patient confidentiality

All investigators and trial site staff must comply with the requirements of the GDPR with regards to the collection, storage, processing and disclosure of personal information and will uphold the Act’s core principles.

- Personal information will be collected via the eCRF to enable record linkage to be carried out and to provide electronic access to study monitors to a copy of the signed informed consent document. These data items will be encrypted and only those individuals who require to see these data i.e. the person performing the record linkage and site research team staff or the study monitor, as appropriate, will be able to view them. All electronic data will be held securely in accordance with ISO 27001:2013 at the Robertson Centre for Biostatistics, part of the Glasgow Clinical Trials Unit. All Centre staff are required to sign confidentiality agreements and to follow Standard Operating Procedures in accordance with Good Clinical Practice and ISO certification.
- The trial data managers, statisticians, health economists or any other staff who will perform data related tasks will only be able to access depersonalised data where the participant’s identifying information is replaced by a unique study identifier.

- Only those that have been trained and approved will be able to enter or view any data via the web portal. Each site can only see their own patients’ data. Patient consent forms will be stored at the study site in a secure location accessible only to study teams.

13.8 Indemnity

The Co-Sponsors (University of Glasgow and Greater Glasgow Health Board) will ensure that provision has been made for insurance or indemnity to cover the liability of the investigator and sponsor which may arise in relation to the clinical trial in accordance with Part 2 (14) of Schedule 1 to SI 2004/1031.

13.9 Amendments

Any change in the study protocol will require an amendment. Any proposed protocol amendments will be initiated by the CI following discussion with the Sponsor and TSC and any required amendment forms will be submitted to the regulatory authority, ethics committee and Sponsor. The Sponsor will determine whether an amendment is non-substantial or substantial. All amended versions of the protocol will be signed by the CI and Sponsor representative. Following a substantial amendment, favourable opinion/approval must be sought from the original reviewing REC, MHRA (where appropriate) and Research and Development (R&D) office prior to implementation. The Chief Investigator will be responsible for informing the Trial Management Group of all protocol amendments.

13.10 Post trial care

At the end of the trial, participants will be returned to usual care as defined by local and national guidelines at that time. The results of the trial may of course have an impact on these guidelines and the future care of patients with type 1 diabetes.

13.11 Access to the final trial dataset

During the trial and in the period prior to publication of the main study results as described in the protocol, only the Glasgow CTU and Professor Paresh Dandona, US Principal Investigator, University of Buffalo will have access to the full dataset. After that period, the trial Steering Committee will conduct further data analyses for a period of three years. After that time the Trial Steering Committee will consider requests from external parties for further analyses of the study data. Proposals that are scientifically well founded and have an academic basis and where relevant data extractions and analyses are appropriately funded will not be refused. These will be considered as collaborative exercises where the contributions related to study design, conduct, database creation and maintenance and data analysis will be recognised in authorship of any scientific publication. The approach we will take will be to minimise any possibility of breach of participant confidentiality. Normally this will be achieved by minimising data travel. However, for the purposes of individual patient meta-analysis and other reasons, data may be transferred to other sites. Such transfer will require assurances on information security systems at the sites that data are to be transferred to and will involve a legal data transfer agreement. A log of all data requests and subsequent data transfers will be held at the Glasgow CTU.

14 DISSEMINATION POLICY

14.1 Dissemination policy

The study database will be owned by the University of Glasgow and maintained on behalf of the Study investigators, represented by the Trial Steering Committee as it is constituted during and after the trial.

The study protocol and a description of the recruitment experience and participant baseline characteristics will be published before study completion. On completion of the trial, the database will be locked and analysed by staff of the Robertson Centre for Biostatistics, University of Glasgow. A final study report will be prepared and the results will be published in a major medical journal.

After the main study publications, study investigators will be invited to submit requests for further analyses of the study database. These will be reviewed and prioritised by a Publications Committee made up of the study grant holders and convened by the study co-CIs.

As funder of the trial, JDRF will have the right to see and comment on any results being submitted for publication. A maximum of 28 days will be given for review of major papers and 14 days for abstracts. Such comments will be considered by the Trial Steering Committee.

A lay summary of the main results of the trial will be prepared and provided to all participants via their study site investigators. Investigators may request a copy of the study data for their participants. Providing some or all of a patient's data to that individual is at their discretion.

14.2 Authorship eligibility guidelines and any intended use of professional writers

The main results of the study will be compiled, written up and published by the study grant holders and others taking responsibility for the study results (e.g. the statistician conducting the final analysis) on behalf of the TTT1 investigators. The TTT1 investigators will be listed in an Appendix and will include all site PIs, all committee members and key members of relevant study coordinating groups (including the Sponsor and Glasgow CTU).

15 REFERENCES

1. Konrad K, Datz N, Engelsberger I, Grulich-Henn J, Hoertenhuber T, Knauth B, et al. Current use of metformin in addition to insulin in pediatric patients with type 1 diabetes mellitus: an analysis based on a large diabetes registry in Germany and Austria. *Pediatr Diabetes.* 2014.
2. Vella S, Buetow L, Royle P, Livingstone S, Colhoun HM, and Petrie JR. The use of metformin in type 1 diabetes: a systematic review of efficacy. *Diabetologia.* 2010;53(5):809-20.
3. Vella S, Buetow L, Royle P, Livingstone S, and Petrie JR. Metformin in type 1 diabetes reduces insulin requirements without significantly improving glycaemic control. Reply to Schatz H [letter]. *Diabetologia.* 2011;54(1):203-4.
4. Varanasi A, Bellini N, Rawal D, Vora M, Makdissi A, Dhindsa S, et al. Liraglutide as additional treatment for type 1 diabetes. *Eur J Endocrinol.* 2011;165(1):77-84.
5. Kuhadiya ND, Malik R, Bellini NJ, Patterson JL, Traina A, Makdissi A, et al. Liraglutide as additional treatment to insulin in obese patients with type 1 diabetes mellitus. *Endocrine practice : official journal of the American College of Endocrinology and the American Association of Clinical Endocrinologists.* 2013;19(6):963-7.
6. Kuhadiya ND, Dhindsa S, Ghanim H, Mehta A, Makdissi A, Batra M, et al. Addition of Liraglutide to Insulin in Patients With Type 1 Diabetes: A Randomized Placebo-Controlled Clinical Trial of 12 Weeks. *Diabetes Care.* 2016;39(6):1027-35.
7. Chaudhuri A, Ghanim H, Makdissi A, Green K, Abuaysheh S, Batra M, et al. Exenatide induces an increase in vasodilatory and a decrease in vasoconstrictive mediators. *Diabetes Obes Metab.* 2017;19(5):729-33.
8. Mathieu C, Zinman B, Hemmingsson JU, Woo V, Colman P, Christiansen E, et al. Efficacy and Safety of Liraglutide Added to Insulin Treatment in Type 1 Diabetes: The ADJUNCT ONE Treat-To-Target Randomized Trial. *Diabetes Care.* 2016;39(10):1702-10.
9. Ahren B, Hirsch IB, Pieber TR, Mathieu C, Gomez-Peralta F, Hansen TK, et al. Efficacy and Safety of Liraglutide Added to Capped Insulin Treatment in Subjects With Type 1 Diabetes: The ADJUNCT TWO Randomized Trial. *Diabetes Care.* 2016;39(10):1693-701.
10. Zinman B, Wanner C, Lachin JM, Fitchett D, Bluhmki E, Hantel S, et al. Empagliflozin, Cardiovascular Outcomes, and Mortality in Type 2 Diabetes. *N Engl J Med.* 2015;373(22):2117-28.
11. Petrie JR. SGLT2 inhibitors in type 1 diabetes: knocked down, but up again? *Lancet Diabetes Endocrinol.* 2017;5(11):841-3.
12. Mudaliar S, Armstrong DA, Mavian AA, O'Connor-Semmes R, Mydlow PK, Ye J, et al. Remogliflozin etabonate, a selective inhibitor of the sodium-glucose transporter 2, improves serum glucose profiles in type 1 diabetes. *Diabetes Care.* 2012;35(11):2198-200.
13. Kohan DE, Fioretto P, Tang W, List JF. Long-term study of patients with type 2 diabetes and moderate renal impairment shows that dapagliflozin reduces weight and blood pressure but does not improve glycemic control. Kidney Int. 2014 Apr;85(4):962-71. doi: 10.1038/ki.2013.356. Epub 2013 Sep 25. PMID: 24067431; PMCID: PMC3973038.
14. Dandona P, Mathieu C, Phillip M, Hansen L, Griffen SC, Tschope D, et al. Efficacy and safety of dapagliflozin in patients with inadequately controlled type 1 diabetes (DEPICT-1): 24 week results from a multicentre, double-blind, phase 3, randomised controlled trial. *Lancet Diabetes Endocrinol.* 2017;5(11):864-76.

15. Mathieu C, Dandona P, Gillard P, Senior P, Hasslacher C, Araki E, Lind M, Bain SC, Jabbour S, Arya N, Hansen L, Thorén F, Langkilde AM; DEPICT-2 Investigators. Efficacy and Safety of Dapagliflozin in Patients With Inadequately Controlled Type 1 Diabetes (the DEPICT-2 Study): 24-Week Results From a Randomized Controlled Trial. Diabetes Care. 2018 Sep;41(9):1938-1946.
16. European Medicines Agency. Assessment Report Dapagliflozin. https://www.ema.europa.eu/en/documents/variation-report/forxiga-h-c-ws-1941-epar-assessment-report-variation_en.pdf *(accessed 11th November 2021)*
17. Groop PH, Dandona P, Phillip M, Gillard P, Edelman S, Jendle J, Xu J, Scheerer MF, Thoren F, Iqbal N, Repetto E, Mathieu C. Effect of dapagliflozin as an adjunct to insulin over 52 weeks in individuals with type 1 diabetes: post-hoc renal analysis of the DEPICT randomised controlled trials. Lancet Diabetes Endocrinol. 2020 Oct;8(10):845-854. doi: 10.1016/S2213-8587(20)30280-1. PMID: 32946821.
18. Rosenstock J, Marquard J, Laffel LM, et al. Empagliflozin as adjunctive to insulin therapy in type 1 diabetes: the EASE trials. *Diabetes Care*. 2018;41(12):2560–2569.
19. Garg SK, Henry RR, Banks P, Buse JB, Davies MJ, Fulcher GR, et al. Effects of Sotagliflozin Added to Insulin in Patients with Type 1 Diabetes. *N Engl J Med.* 2017;377(24):2337-48.
20. European Medicines Agency. First oral add-on treatment to insulin for treatment of certain patients with type 1 diabetes. https://www.ema.europa.eu/en/news/first-oral-add-treatment-insulin-treatment-certain-patients-type-1-diabetes *(accessed 11th November 2021)*
21. An overview of Zynquista and why it is authorised in the EU. 16th May 2019. https://www.ema.europa.eu/en/documents/overview/zynquista-epar-medicine-overview_en.pdf *(accessed 11th November 2021)*
22. National Institute for Health and Care Excellence. Dapagliflozin with insulin for treating type 1 diabetes. Technology appraisal guidance [TA597] https://www.nice.org.uk/guidance/ta597
23. AstraZeneca Press Release. Update on US regulatory decision for Farxiga in type-1 diabetes. https://www.astrazeneca.com/media-centre/press-releases/2019/update-on-us-regulatory-decision-for-farxiga-in-type-1-diabetes-15072019.html# *(accessed 11th November 2021)*
24. European Medicines Agency. Forxiga (dapagliflozin) 5mg should no longer be used for the treatment of Type 1 Diabetes Mellitus. https://www.ema.europa.eu/en/documents/dhpc/direct-healthcare-professional-communication-dhpc-forxiga-dapagliflozin-5mg-should-no-longer-be-used_en.pdf *(accessed 11th November 2021)*
25. AstraZeneca. Letter to UK Health Professionals. Forxiga (dapagliflozin) 5mg should no longer be used for the treatment of type 1 diabetes mellitus. (2nd November 2021)
26. Marso SP, Bain SC, Consoli A, Eliaschewitz FG, Jódar E, Leiter LA, Lingvay I, Rosenstock J, Seufert J, Warren ML, et al. Semaglutide and cardiovascular outcomes in patients with type 2 diabetes. *N Engl J Med.* 2016;**375**(19):1834–44. doi: 10.1056/NEJMoa1607141.

27. Ozempic Summary of product characteristics. https://www.medicines.org.uk/emc/product/9728/smpc
28. Nauck MA, Petrie JR, Sesti G, Mannucci E, Courrèges JP, Lindegaard ML, Jensen CB, Atkin SL; Study 1821 Investigators. A Phase 2, Randomized, Dose-Finding Study of the Novel Once-Weekly Human GLP-1 Analog, Semaglutide, Compared With Placebo and Open-Label Liraglutide in Patients With Type 2 Diabetes. Diabetes Care. 2016 Feb;39(2):231-41.
29. Forxiga 5 mg Summary of Product Characteristics: https://www.medicines.org.uk/emc/product/2865/smpc *(accessed 11th November 2021)*
30. Wiviott SD, Raz I, Bonaca MP, Mosenzon O, Kato ET, Cahn A, Silverman MG, Zelniker TA, Kuder JF, Murphy SA, Bhatt DL, Leiter LA, McGuire DK, Wilding JPH, Ruff CT, Gause-Nilsson IAM, Fredriksson M, Johansson PA, Langkilde AM, Sabatine MS; DECLARE–TIMI 58 Investigators. Dapagliflozin and Cardiovascular Outcomes in Type 2 Diabetes. N Engl J Med. 2019 Jan 24;380(4):347-357.
31. McMurray JJV, Solomon SD, Inzucchi SE, Køber L, Kosiborod MN, Martinez FA et al. DAPA-HF Trial Committees and Investigators Dapagliflozin in patients with heart failure and reduced ejection fraction. N Engl J Med. 2019. Nov 21;381(21):1995-2008.
32. Heerspink HJL, Stefánsson BV, Correa-Rotter R, Chertow GM, Greene T, Hou FF, Mann JFE, McMurray JJV, Lindberg M, Rossing P, Sjöström CD, Toto RD, Langkilde AM, Wheeler DC; DAPA-CKD Trial Committees and Investigators. Dapagliflozin in Patients with Chronic Kidney Disease. N Engl J Med. 2020 Oct 8;383(15):1436-1446. doi: 10.1056/NEJMoa2024816. Epub 2020 Sep 24. PMID: 32970396.
33. Vilsbøll T, Bain SC, Leiter LA, et al. Semaglutide, reduction in glycated haemoglobin and the risk of diabetic retinopathy. *Diabetes Obes Metab*. 2018;20(4):889–897.
34. Lingvay I, Desouza CV, Lalic KS et al. A 25 week randomized controlled trial of semaglutide once daily versus liraglutide and placebo in patients with type 2 diabetes suboptimally controlled on diet and exercise with or without metformin. Diabetes Care. 2018;41(9): 1926-1937.
35. European Medicines Agency. Assessment Report Sotagliflozin. https://www.ema.europa.eu/en/documents/assessment-report/zynquista-epar-public-assessment-report_en.pdf *(accessed 11th November 2021)*
36. Silverii GA, Decembrini L, Monami M, Mannucci E. Fournier's gangrene and sodium glucose co-transporter 2 inhibitors: A metanalysis of randomized controlled trials. Diabetes Obes Metab. 2019; 1-4.
37. Garg SK, Peters AL, Buse JB, Danne T. Strategy for mitigating DKA risk in patients with Type 1 diabetes on adjunctive treatment with SGLT-2 inhibitors: A STICH Protocol. Diabetes Technol Ther. 2018;20(9):571-575.
38. Peters AL, Buschur EO, Buse JB, Cohan P, Diner JC, and Hirsch IB. Euglycemic Diabetic Ketoacidosis: A Potential Complication of Treatment With Sodium-Glucose Cotransporter 2 Inhibition. Diabetes Care. 2015;38(9):1687-93.
39. Agiostratidou G, Anhalt H, Ball D, Blonde L, Gourgari E, Harriman KN, Kowalski AJ, Madden P, McAuliffe-Fogarty AH, McElwee-Malloy M, Peters A, Raman S, Reifschneider K, Rubin K, Weinzimer SA. Standardizing Clinically Meaningful Outcome Measures Beyond HbA(1c) for Type 1 Diabetes: A Consensus Report of the American Association

of Clinical Endocrinologists, the American Association of Diabetes Educators, the American Diabetes Association, the Endocrine Society, JDRF International, The Leona M. and Harry B. Helmsley Charitable Trust, the Pediatric Endocrine Society, and the T1D Exchange. Diabetes Care. 2017 Dec;40(12):1622-1630.

16. APPENDICES

16.1 Appendix 1 – Risk

Risks associated with trial interventions<br>☐ LOW ≡ Comparable to the risk of standard medical care<br>☒ MODERATE ≡ Somewhat higher than the risk of standard medical care<br>☐ HIGH ≡ Markedly higher than the risk of standard medical care				
Justification: Briefly justify the risk category selected and your conclusions below (where the table is completed in detail the detail need not be repeated, however a summary should be given):<br>An IDMC will be convened to monitor all SAEs.<br>Risks and mitigations associated with the intervention are outlined in more detail in the Protocol section 2.1.<br>Co-Sponsors will also carry out a detailed risk assessment of all aspects of the study as part of the approval process (SOP 04.013)				
What are the key risks related to therapeutic interventions you plan to monitor in this trial?		How will these risks be minimised?		
IMP/Intervention	Body system/Hazard	Activity	Frequency	Comments
S/C administration of semaglutide<br>Oral admistration of dapagliflozin	Immune: Hypersensitivity/ana-phylactic reactions	Patients with known hypersensitivity to any GLP-1 agonist or SGLT-2 inhibitor have a contra-indication to the IMP according to the SmPC will not be recruited to the study	Uncommon/ Rare	
PO administration of dapagliflozin	Ketosis and/or Diabetic Ketoacidosis (DKA)	All participants will have access to blood or urine ketone monitoring equipment. An emergency ketosis management plan (STICH Protocol – see above) will be discussed with all participants. Patient alert cards will be provided to all participants. Potential participants with recent ketoacidosis will be	Common	"and" was an error – intention was for "either" blood or urine ketones between visits but "both" to be measured for visits. This is compatible with the eCRF, the diaries and the rest of the protocol

		excluded from the trial (section 6.2)		
S/C administration of semaglutide (+/-) PO administration of dapagliflozin in addition to insulin therapy	Increased risk of hypoglycaemia	Participant awareness of risk and frequent testing of capillary blood glucose. Patients with pre-existing frequent episodes of hypoglycaemia or problems with hypoglycaemia awareness will be excluded from trial participation (section 6.2)	Common	
Outline any other processes that have been put in place to mitigate risks to participant safety (e.g. IDMC, independent data review, etc.)<br>See above				

16.2 Appendix 2: Contraception

In line with recommendations related to contraception and pregnancy testing in clinical trials from the Clinical Trial Facilitation Group (CTFG), for women of childbearing potential* (WOCBP) participating in TTT1, methods of contraception considered as highly effective are defined as those that can achieve a failure rate of less than 1% per year when used consistently and correctly.

Such methods include:

- combined (oestrogen and progestogen containing) hormonal contraception associated with inhibition of ovulation[1]
    - oral
    - intravaginal
    - transdermal
- progestogen-only hormonal contraception associated with inhibition of ovulation[1]
    - oral
    - injectable
    - implantable[2]
- intrauterine device (IUD) [2]
- intrauterine hormone-releasing system (IUS)[2]
- bilateral tubal occlusion[2]
- vasectomised partner[2,3]
- true sexual abstinence[4]

Footnotes

1. An interaction between dapagliflozin and semaglutide with contraception is not anticipated.
2. Contraception methods that in the context of this guidance are considered to have low user dependency.
3. Vasectomised partner is a highly effective birth control method provided that partner is the sole sexual partner of the WOCBP trial participant and that the vasectomised partner has received medical assessment of the surgical success.
4. In the context of this guidance true sexual abstinence refers to: When this is in line with the preferred and usual lifestyle of the patient. Periodic abstinence (e.g. calendar, ovulation, symptothermal, post-ovulation methods), declaration of abstinence for the duration of a trial, and withdrawal are not acceptable methods of contraception.
The reliability of sexual abstinence needs to be evaluated in relation to the duration of the clinical trial and the preferred and usual lifestyle of the subject.

*Child bearing potential = experienced menarche; AND not undergone successful surgical sterilisation (hysterectomy, bilateral tubal ligation, or bilateral oophorectomy); AND not post-menopausal i.e. amenorrhea for ≥12 consecutive months (without another medical cause)

Source: Recommendations related to contraception and pregnancy testing in clinical trials. Clinical Trial Facilitation Group (CTFG), 15th September 2014.
See: https://www.hma.eu/fileadmin/dateien/Human_Medicines/01-About_HMA/Working_Groups/CTFG/2014_09_HMA_CTFG_Contraception.pdf (Accessed 21st January 2020)

16.3 Appendix 3

STICH Protocol *(also provided in Period 2 Participant Diary)*

As explained above, participants in this trial randomised to dapagliflozin are at an increased risk of ketosis and ketoacidosis. All participants will have access to a ketone meter as standard of care. It will be ascertained that each participant has access to a ketone meter; if there are any issues with their current ketone meter it will be replaced as per standard of care. There are mechanisms designed to mitigate the risk of early ketosis developing further into diabetic ketoacidosis. In this trial, we will be using a protocol called STICH (37). This is a mnemonic designed to act as a reminder for participants as to what to do in the event of elevated ketones following exposure to an SGLT-2 drug such as dapagliflozin:

1. STop dapagliflozin or placebo temporarily
2. Insulin – take a bolus of rapid acting insulin
3. Carbohydrate – take approximately 30g of carbohydrate
4. Hydrate – make sure you are taking sips of water aiming to maintain hydration.

Further guidance will be provided in both the Patient Diaries supplied to all participants and in the Patient Alert Cards that participants will be asked to carry at all times. All participants will also be advised that in the event of acute intercurrent illness or hospitalisation, dapagliflozin or placebo should be temporarily stopped. This is because intercurrent illness may additionally increase the risk of ketosis. Participants will likely already be familiar with this concept of "sick-day rules" as this is a key component of basic education for people with type 1 diabetes in clinical practice. These rules will however be re-iterated to participants.

More detailed guidance on actions participants should take for any given level of ketosis will be provided during face-to-face and telephone contact during the trial. In addition, however, the following written protocol will be provided to participants to advise them explicitly of the safest and most appropriate course of action to be taken in any given circumstance. This will take the form of a traffic light system to aid understanding and compliance.

1. Ketones 0.6 mmol/L or less: Continue - no need for additional bolus insulin.
2. Ketones 0.6 – 1.4 mmol/L: consider taking additional fast acting insulin. Follow sick day rules and monitor ketones 2 hourly until they are 0.6 mmol/L or less.
3. Ketones 1.5 – 2.9 mmol/L – Take 10% of your total daily dose of insulin (this is found by adding up all the long acting and short acting insulin for the previous day - only take a maximum of 10 units) as a bolus of fast acting insulin. Keep hydrated - aim for 100ml water per hour. Re-test your glucose and ketones in 2 hours. If not falling contact the study team during office hours. If you need urgent advice at other times please contact NHS 24 (telephone number 111) or attend your nearest Accident & Emergency Department.
4. Ketones above 3 mmol/L – Take 20% of your total daily insulin as fast acting insulin (Maximum 10 units). Hydrate with water, aim for 500 ml per hour. Attend A&E – High risk of DKA

If you develop DKA or are unwell it is important that you let the TTT1 nurse or doctor know about this as soon as you can. By following this protocol, the risk of ketosis and developing ketoacidosis will be mitigated.

16.4 Appendix 4: Amendment History

Amendment No.	Protocol version no.	Date issued	Author(s) of changes	Details of changes made
AM02	2.0	Ethics App – 23 Nov 2020		Protocol Changes: Weight loss mitigation, 19 Risk Mitigation and visit activity corrections
AM03	2.1	Ethics App – 10th Feb 2021		Correction of Summary of changes for previous AM02.<br>Corrected pregnancy testing schedule.
AM06	3.0	Ethics App – 16th Feb 2022		Definition of AESIs narrowed to include only ketosis and level 2 hypoglycaemia; lowering eGFR inclusion criterion; correction of DSQOLS to licensed version.
AM07	4.0			Procedure for recruitment at other sites within NHS GGC described; procedures for rescreening of participants experiencing delay between the screening visit and Visit 1; updates; correction of errors

List details of all protocol amendments here whenever a new version of the protocol is produced. Protocol amendments must be submitted to the Sponsor for approval prior to submission to the REC committee or MHRA.

16.5 Appendix 5: PAID Questionnaire

Problem Areas In Diabetes (PAID) scale

Instructions: Which of the following diabetes issues are **currently** a problem for you? Tick the box that gives the best answer for you. Please provide an answer for each question.

		Not a problem	Minor problem	Moderate problem	Somewhat serious problem	Serious problem
1	Not having clear and concrete goals for your diabetes care?	☐ 0	☐ 1	☐ 2	☐ 3	☐ 4
2	Feeling discouraged with your diabetes treatment plan?	☐ 0	☐ 1	☐ 2	☐ 3	☐ 4
3	Feeling scared when you think about living with diabetes?	☐ 0	☐ 1	☐ 2	☐ 3	☐ 4
4	Uncomfortable social situations related to your diabetes care (e.g. people telling you what to eat)?	☐ 0	☐ 1	☐ 2	☐ 3	☐ 4
5	Feelings of deprivation regarding food and meals?	☐ 0	☐ 1	☐ 2	☐ 3	☐ 4
6	Feeling depressed when you think about living with diabetes?	☐ 0	☐ 1	☐ 2	☐ 3	☐ 4
7	Not knowing if your mood or feelings are related to your diabetes?	☐ 0	☐ 1	☐ 2	☐ 3	☐ 4
8	Feeling overwhelmed by your diabetes?	☐ 0	☐ 1	☐ 2	☐ 3	☐ 4
9	Worrying about low blood glucose reactions?	☐ 0	☐ 1	☐ 2	☐ 3	☐ 4
10	Feeling angry when you think about living with diabetes?	☐ 0	☐ 1	☐ 2	☐ 3	☐ 4
11	Feeling constantly concerned about food and eating?	☐ 0	☐ 1	☐ 2	☐ 3	☐ 4
12	Worrying about the future and the possibility of serious complications?	☐ 0	☐ 1	☐ 2	☐ 3	☐ 4
13	Feelings of guilt or anxiety when you get off track with your diabetes management?	☐ 0	☐ 1	☐ 2	☐ 3	☐ 4
14	Not 'accepting' your diabetes?	☐ 0	☐ 1	☐ 2	☐ 3	☐ 4
15	Feeling unsatisfied with your diabetes physician?	☐ 0	☐ 1	☐ 2	☐ 3	☐ 4
16	Feeling that diabetes is taking up too much of your mental and physical energy every day?	☐ 0	☐ 1	☐ 2	☐ 3	☐ 4
17	Feeling alone with your diabetes?	☐ 0	☐ 1	☐ 2	☐ 3	☐ 4
18	Feeling that your friends and family are not supportive of your diabetes management efforts?	☐ 0	☐ 1	☐ 2	☐ 3	☐ 4
19	Coping with complications of diabetes?	☐ 0	☐ 1	☐ 2	☐ 3	☐ 4
20	Feeling 'burned out' by the constant effort needed to manage diabetes?	☐ 0	☐ 1	☐ 2	☐ 3	☐ 4

© Joslin Diabetes Center, 1999 (www.joslin.org). All rights reserved. The copyright holder/developer has given permission for the questionnaire to be reproduced in this toolkit. Readers of the toolkit are permitted to reproduce the questionnaire for clinical use and non-commercial research purposes. Readers of the toolkit are not permitted to use the questionnaire for commercial research purposes and must seek permission from the copyright holder/developer to do so.

16.6 Appendix 6: DSQOLS Questionnaire

Diabetes specific quality of life scale (DSQOLS)

Date____________ Name____ DOB______________

Please tick the appropriate box for each question –

Which burdens and restrictions from diabetes and its treatment have you experienced during the last four weeks?...	This statement meets my point of view...					
	5. Perfectly	4. Quite good	3. A little	2. Rather not	1. Hardly	0. Not at all
1. It bothers me that I have to measure my blood glucose so often	☐	☐	☐	☐	☐	☐
2. It burdens me that I always have to think about my nutrition.	☐	☐	☐	☐	☐	☐
3. I suffer from pain because of diabetes.	☐	☐	☐	☐	☐	☐
4. Because of diabetes the relationship to my partner has become worse.	☐	☐	☐	☐	☐	☐
5. I am worried about the fact that my life could be shorter because of diabetes.	☐	☐	☐	☐	☐	☐
6. I have the impression that I am less attractive for others because of diabetes.	☐	☐	☐	☐	☐	☐
7. Because of diabetes I feel sad or depressed	☐	☐	☐	☐	☐	☐
8. I am worried about my future health	☐	☐	☐	☐	☐	☐
9. It is a burden for me how other people react to my diabetes	☐	☐	☐	☐	☐	☐
10. I feel nervous and restless when I think about episodes of low blood sugar						
11. I feel tired and exhausted	☐	☐	☐	☐	☐	☐
12. It bothers me that I have to spend so much time on my diabetes treatment	☐	☐	☐	☐	☐	☐
13. Because of diabetes traveling is complicated and troublesome	☐	☐	☐	☐	☐	☐
14. I get anxious and nervous when I think about the dangers of episodes of low blood glucose	☐	☐	☐	☐	☐	☐

	5. Perfectly	4. Quite good	3. A little	2. Rather not	1. Hardly	0. Not at all
15. Diabetes prevents me from spontaneous physical activities	☐	☐	☐	☐	☐	☐
16. I suffer from frequent urination	☐	☐	☐	☐	☐	☐
17. Because of high blood glucose values after a meal I often feel unwell or less efficient	☐	☐	☐	☐	☐	☐
18. Because of diabetes I feel anxious or threatened	☐	☐	☐	☐	☐	☐
19. I suffer from thirst or having a dry mouth	☐	☐	☐	☐	☐	☐
20. Other people find it hard to understand my problems with diabetes treatment	☐	☐	☐	☐	☐	☐
21. I feel physically unwell	☐	☐	☐	☐	☐	☐
22. I unnecessarily worry too much about episodes of low blood glucose	☐	☐	☐	☐	☐	☐
23. I have to give up good-tasting foods	☐	☐	☐	☐	☐	☐
24. I cannot eat as much as I want of certain foods	☐	☐	☐	☐	☐	☐
25. Because of diabetes I often have physical complaints	☐	☐	☐	☐	☐	☐
26. Diabetes interferes with my future plans	☐	☐	☐	☐	☐	☐
27. I get an uncomfortable feeling when I think about the dangers of a severe episode of low blood glucose	☐	☐	☐	☐	☐	☐
28. Because of diabetes I have less contact with friends or acquaintances	☐	☐	☐	☐	☐	☐
29. I wish I could eat more the way I want to, without having to plan everything beforehand	☐	☐	☐	☐	☐	☐
30. I suffer from frequent infections, itching or skin problems	☐	☐	☐	☐	☐	☐
31. I am concerned about getting into embarrassing situations because of episodes of	☐	☐	☐	☐	☐	☐

	5. Perfectly	4. Quite good	3. A little	2. Rather not	1. Hardly	0. Not at all
low blood glucose						
32. I feel weak or lazy	☐	☐	☐	☐	☐	☐
33. I am dissatisfied with the amount of time I have to spend for medical consultations	☐	☐	☐	☐	☐	☐
34. Because of my diabetes I cannot spend my free time the way I would like	☐	☐	☐	☐	☐	☐
35. I feel like a disabled person	☐	☐	☐	☐	☐	☐
36. I am worried that I could sustain physical injury in the event of a severe episode of low blood glucose	☐	☐	☐	☐	☐	☐
37. It bothers me that I cannot eat like other people	☐	☐	☐	☐	☐	☐
38. I am often worried about the long-term	☐	☐	☐	☐	☐	☐
39. Because of diabetes other people treat me like a "sick person"	☐	☐	☐	☐	☐	☐
40. Because of diabetes I cannot pursue my hobbies as I wish	☐	☐	☐	☐	☐	☐
41. I have to frequently think about diabetes and its consequences	☐	☐	☐	☐	☐	☐
42. It bothers me that I cannot eat as spontaneously as people who do not have diabetes	☐	☐	☐	☐	☐	☐
43. It upsets my stomach when I think about the dangers of severe episodes of low blood glucose	☐	☐	☐	☐	☐	☐
44. Diabetes constantly causes problems while dealing with other people	☐	☐	☐	☐	☐	☐

With respect to diabetes treatment it is very important to me that, resp. it would be my goal that ...	5. Very Important	4. Quite Important	3. Rather Important	2. Rather Unimportant	1. Quite Unimportant	0. Totally Unimportant
45. my blood glucose values are always below 140 mg%	☐	☐	☐	☐	☐	☐
46. my blood glucose values are as constant as possible	☐	☐	☐	☐	☐	☐
47. I can plan my leisure flexibly	☐	☐	☐	☐	☐	☐
48. I can avoid even mild hypoglycaemia	☐	☐	☐	☐	☐	☐
49. I can avoid late complications of diabetes in any case, resp. avoid their progress.	☐	☐	☐	☐	☐	☐
50. I can eat flexibly in the way that I can choose the kind, amount and time of meals	☐	☐	☐	☐	☐	☐
51. I am as physically fit as possible.	☐	☐	☐	☐	☐	☐
52. I can in any case avoid severe hypoglycaemia with loss of consciousness	☐	☐	☐	☐	☐	☐
53. I have to measure my blood glucose as rarely as possible.	☐	☐	☐	☐	☐	☐
54. Other people don't find out that I have diabetes,	☐	☐	☐	☐	☐	☐

How satisfied have you been during the last weeks with ...	0. Very Satisfied	1. Quite Satisfied	2. Rather Satisfied	3. Rather dissatisfied	4. Quite dissatisfied	5. Totally dissatisfied
55. the level of your blood glucose values?	☐	☐	☐	☐	☐	☐
56. The stability of your blood glucose values?	☐	☐	☐	☐	☐	☐
57. The flexibility of planning your leisure?	☐	☐	☐	☐	☐	☐
58. The frequency of mild hypoglycaemia?	☐	☐	☐	☐	☐	☐
59. your assurance of being protected against late complications, resp. of being able to avoid their progress?	☐	☐	☐	☐	☐	☐
60. The flexibility of your diet?	☐	☐	☐	☐	☐	☐
61. your physical fitness?	☐	☐	☐	☐	☐	☐
62. your assurance of being protected against severe hypoglycaemia with loss of consciousness?	☐	☐	☐	☐	☐	☐
63. the frequency of your blood glucose self-monitoring?	☐	☐	☐	☐	☐	☐
64. The extent to what other people are informed about your diabetes?	☐	☐	☐	☐	☐	☐

FULL/LONG TITLE OF THE TRIAL
Triple Therapy for Type 1 Diabetes with Insulin, Semaglutide and Dapagliflozin (TTT1) - UK

SHORT STUDY TITLE / ACRONYM
TTT1 (UK)

PROTOCOL VERSION NUMBER AND DATE
Version 5.0 28th September 2023

RESEARCH REFERENCE NUMBERS

**IRAS Number:**	254431
**EudraCT Number:**	2018-004120-11
**ISRCTN Number / Clinical trials.gov Number:**	NCT03899402
**SPONSOR Number:**	GN17DI706
**FUNDER Number:**	3-SRA-2019-669-M-B

SIGNATURE PAGE

The undersigned confirm that the following protocol has been agreed and accepted and that the Chief Investigator agrees to conduct the trial in compliance with the approved protocol and will adhere to the principles outlined in the Medicines for Human Use (Clinical Trials) Regulations 2004 (SI 2004/1031), amended regulations (SI 2006/1928) and any subsequent amendments of the clinical trial regulations, Good Clinical Practice (GCP) guidelines, the Sponsor's (Standard Operating Procedures (SOPs), and other regulatory requirements as amended.
I agree to ensure that the confidential information contained in this document will not be used for any other purpose other than the evaluation or conduct of the clinical investigation without the prior written consent of the Sponsor
I also confirm that I will make the findings of the study publicly available through publication or other dissemination tools without any unnecessary delay and that an honest accurate and transparent account of the study will be given; and that any discrepancies from the study as planned in this protocol will be explained.

For and on behalf of the Study Co-Sponsor (University of Glasgow):
Signature: .... Date: ....../....../......
Name (please print): ....
Position: ....

For and on behalf of the Study Co-Sponsor (NHS Greater Glasgow and Clyde):
Signature: .... Date: ....../....../......
Name (please print): ....
Position: ....

UK Chief Investigator:
Signature: .... Date: ....../....../......
Name: (please print): John Petrie ....
Position: Professor of Diabetes...

Statistician:
Signature: .... Date: ....../....../......
Name: (please print): ....

Position: ....

KEY TRIAL CONTACTS

UK Chief Investigator	Professor John R Petrie<br>Professor of Diabetes<br>Director, Robertson Centre for Biostatistics<br>Director, Glasgow Clinical Trials Unit<br>School of Health and Wellbeing<br>College of Medical, Veterinary and Life Sciences<br>University of Glasgow<br>Clarice Pears Buildiing<br>90 Byres Road<br>Glasgow<br>G12 8TB<br>Secretary +44(0)141 330 4744<br>john.petrie@glasgow.ac.uk
UK Co- Investigator	Dr James Boyle<br>Consultant Diabetologist<br>Clinical Lead NHS GG&C Diabetes MCN<br>Diabetes UK Clinical Champion<br>Glasgow Royal Infirmary<br>84 Castle Street, Glasgow, G4 0SF<br>Tel: : +44(0)141 211 5996<br>james.boyle@glasgow.ac.uk
Trial Co-ordination	Miss Lisa Jolly<br>Research and Innovation<br>NHS Greater Glasgow & Clyde<br>Ward 11, Dykebar Hospital<br>Grahamston Road<br>Paisley, PA2 7DE<br>Lisa.Jolly@ggc.scot.nhs.uk
Co-Sponsor	NHS Greater Glasgow & Clyde<br><br>Contact: Dr Maureen Travers<br>Research and Development Management Office<br>Research and Innovation<br>NHS Greater Glasgow & Clyde<br>Ward 11, Dykebar Hospital<br>Grahamston Road<br>Paisley, PA2 7DE<br>Tel: +44 (0) 0141 314 4012<br>maureen.travers@ggc.scot.nhs.uk
Co-sponsor	University of Glasgow<br><br>Contact: Dr Debra Stuart<br>College of Medical, Veterinary & Life Sciences<br>Room 327 Wolfson Medical School Building

	University Avenue<br>Glasgow G12 8QQ<br>Tel: +44 (0)141 330 4539<br>Debra.Stuart@glasgow.ac.uk
Funder	JDRF<br>26 Broadway, 15th floor, New York, NY 10004, USA
Clinical Trials Unit	Glasgow Clinical Trials Unit<br>Robertson Centre for Biostatistics, 11th Floor,<br>Boyd Orr Building, University of Glasgow<br>G12 8QQ<br>Tel: 0141 330 4744<br>liz.anderson@glasgow.ac.uk
Statistician	Professor Alex McConnachie<br>Robertson Centre for Biostatistics, 11th Floor, Boyd Orr Building, University of Glasgow, Glasgow, G12 8QQ<br>Telephone: 0141 330 4744<br>Fax: 0141-330-5094
Trials pharmacist	Dr Elizabeth Douglas<br>Clinical Trials Pharmacist<br>Clinical Research and Development<br>Ward 11, Dykebar Hospital,<br>Grahamston Road | Paisley PA2 7DE<br>Tel: 0141 314 4073<br>E-mail: elizabeth.douglas@ggc.scot.nhs.uk

US Principal Investigator	Dr Paresh Dandona<br>Director, Diabetes-Endocrinology Center of Western NY<br>Chief of Endocrinology, Diabetes, Endocrinology and Metabolism, SUNY at Buffalo<br>1000 Youngs Road, Suite 105<br>Williamsville NY 14221<br>Telephone: +1(716) 535-1850<br>Fax: +1(716)-639-1200<br>pdandona@kaleidahealth.org
US Co- Investigator	Dr Husam Ghanim, PhD.<br>Director, Diabetes and Endocrinology Research Lab<br>Research Associate Professor,<br>School of Medicine, State University of NY at Buffalo<br>Clinical and Translational Research Center

	875 Ellicott St<br>Buffalo, NY 14203<br>Tel: (+1)716-881-8924<br>ghanim@buffalo.edu
Key Protocol Contributors	Professor John Petrie<br>john.petrie@glasgow.acuk<br><br>Dr Paresh Dandona<br>pdandona@kaleidahealth.org<br><br>Husam Ghanim, PhD.<br>ghanim@buffalo.edu<br><br>Dr James Boyle<br>james.boyle@glasgow.ac.uk<br><br>Dr Robert Lindsay<br>robert.lindsay@glasgow.ac.uk<br><br>Dr Joseph Timmons<br>joseph.timmons@glasgow.ac.uk<br><br>Professor Ian Ford<br>ian.ford@glasgow.ac.uk<br><br>Professor Alex McConnachie<br>Alex.McConnachie@glasgow.ac.uk

TRIAL SUMMARY

Trial Title	Triple Therapy for Type 1 Diabetes with Insulin, Semaglutide, and Dapagliflozin (UK)	
Internal ref. no. (or short title)	TTT1	
Clinical Phase	Phase 3	
Trial Design	Two six-month periods: (i) open-label randomised (2:1) parallel group semaglutide vs standard of care; (ii) additional double-blind, randomised (1:1) parallel group, dapagliflozin vs placebo for those participants initially randomised to semaglutide.<br>Regardless of each randomisation, ongoing standard of care (insulin therapy) will be provided for all participants throughout the study.	
Trial Participants	Adults with type 1 diabetes for at least one year with HbA1c 7.5-11.0% on stable multiple daily injections (MDI) (four or more) of insulin or continuous subcutaneous insulin infusion (CSII) for the previous three months.	
Planned Sample Size	114 (50 in UK)	
Treatment duration	Approximately 1 year	
Follow up duration	Approximately 1 year	
Planned Trial Period	Approximately 5 years	
	Objectives	Outcome Measures
Primary	To assess in people with type 1 diabetes whether triple therapy (dapagliflozin, semaglutide and insulin) reduces HbA1c in comparison with dual therapy (placebo, semaglutide, and insulin – period 2).	HbA1c
Secondary	To assess in people with type 1 diabetes whether triple therapy (dapagliflozin, semaglutide and insulin) reduces HbA1c in comparison with standard therapy (insulin alone)	HbA1c
	To assess in people with type 1 diabetes whether dual therapy (semaglutide and insulin - period 1) reduces HbA1c in comparison with standard therapy (insulin alone)	HbA1c
	To assess in people with type 1 diabetes whether triple therapy (dapagliflozin, semaglutide and insulin) improves glucose control	- Mean fasting blood glucose<br><br>- Mean blood glucose*<br>- Percentage time in range

	as assessed by CGM, insulin requirement, body weight, blood pressure and quality of life in comparison with (a) dual therapy (placebo, semaglutide, and insulin – period 2) and (b) standard therapy (insulin alone)	- Glycaemic variability*<br>- Percentage time in hyperglycaemia Level 1 (10.1-13.9 mmol/)*<br>- Percentage time in hyperglycaemia Level 2 (>13.9 mmol/L)*<br>-Percentage time in hypoglycaemia Level 1 (3.0 – 3.8 mmol/L)*<br>** CGM (by masked Dexcom G6)*<br><br>Insulin requirement<br>Body weight<br>Systolic/ diastolic blood pressure<br>Number of antihypertensive agents<br>Diabetes Specific Quality of Life Scale (DSQOLS)<br>Problem Areas In Diabetes (PAID) questionnaire
Safety Endpoint 1	a) Triple therapy (dapagliflozin, semaglutide and insulin) in comparison with dual therapy (placebo, semaglutide, insulin – period 2)<br><br>b) Dual therapy (semaglutide and insulin - period 1) in comparison with standard therapy (insulin only)	Difference between treatment arms in:<br>(i) Rate of Level 2 (<3.0 mmol/L) hypoglycaemic events (by Dexcom G6 CGM)<br>(ii) Rate of Level 3 hypoglycaemic events (severe event with altered mental and/or physical status requiring assistance)
Safety Endpoint 2	a) Triple therapy (dapagliflozin, semaglutide and insulin) in comparison with dual therapy (placebo, semaglutide, and insulin – period 2)<br><br>b) Dual therapy (semaglutide and insulin –period 1) in comparison with standard therapy (insulin only)	Difference between treatment arms in:<br>Rate of diabetic ketoacidosis (serum ketones or urinary ketones above normal limits AND serum bicarbonate < 15 mmol/L OR blood pH < 7.3)
Investigational Medicinal Product(s)	Semaglutide<br>Dapagliflozin<br>Placebo matching dapagliflozin	
Non-investigational Medicinal Product(s)	Insulin	
Formulation, Dose, Route of Administration	Semaglutide (subcutaneous injection, once weekly) – open label by pen device (0.25 mg/ 0.5 mg/ 1.0 mg)<br>Dapagliflozin 5 mg (oral) – one then two capsules daily (up-titration)<br>Placebo matching dapagliflozin	

FUNDING AND SUPPORT IN KIND

FUNDER(S)	FINANCIAL AND NON FINANCIALSUPPORT GIVEN
JDRF New York<br>26 Broadway, 15th floor, New York, NY 10004, United States	$1,440,000 (to University at Buffalo, the State University of New York, NY (SUNY Buffalo), USA; and University of Glasgow, UK)
Novo Nordisk, 800 Scudders Mill Road, Princeton Landing, NJ, Plainsboro, 08536.	$160,000 (see paragraph headed "Novo Nordisk" below)
AstraZeneca<br>1800 Concord Pike, Wilmington, DE 19803, USA	Provision of dapagliflozin and matched placebo investigational medicinal products to US site
Dexcom; Suite 6, 1 Tanfield, Edinburgh, EH3 5DA	Staff training and discounted supply of CGM sensors

ROLE OF STUDY SPONSOR AND FUNDER

NHS Greater Glasgow & Clyde and The University of Glasgow will be Co-sponsors of the UK arm of the trial. Prior to study initiation, a non-commercially funded clinical trial co-sponsorship agreement will be put in place between NHS Greater Glasgow & Clyde and The University of Glasgow. The roles and liabilities each organisation will take under The Medicines for Human Use (Clinical Trials) Regulations, 2004 SI 2001:1031 are laid out in this agreement signed by both organisations. The University of Glasgow shall be responsible for carrying out the obligations and responsibilities set out in the aforementioned agreement, and shall be deemed "sponsor" for the purposes of, Part 3 of the regulations in relation to the study. NHS Greater Glasgow & Clyde shall be responsible for carrying out the responsibilities set out in the agreement, and shall be deemed "sponsor" for the purposes of, Parts 4, 5, 6 and 7 of the Regulations in relation to the study.

The Co-Sponsors will delegate specific roles to the Chief Investigator, Glasgow CTU and other third parties. These arrangements will be clearly documented in agreements and/or the Sponsor Delegated Roles and Responsibilities Matrix.

The US arm of the trial has the same inclusion and exclusion criteria and the same pre-specified outcomes but an individualised Protocol. It will collect the same data via a common eCRF. Adverse event data from each site will be available to the trial Data and Safety Monitoring Board. The UK Co-Sponsors will have no responsibility for (and carry no liability for) the conduct of the US arm of the trial.

<u>JDRF</u>
The study has been funded by a grant from JDRF. JDRF does not have a designated role or responsibility in trial design, conduct, data analysis and interpretation, manuscript writing, and dissemination of results. Periodic reports (at least annual) in relation to progress of the trial will be submitted to the JDRF. Support from JDRF will be acknowledged in any publications related to the study.

<u>Astra Zeneca</u>
This is an investigator-initiated study. Astra Zeneca provided support by supplying investigational medicinal products (IMPs) dapagliflozin and matched placebo to the US site only. Representatives of

Astra Zeneca reviewed and provided comments on drafts of the Protocol but the company does not have a designated role or responsibility in trial design, conduct, data analysis and interpretation, manuscript writing, and dissemination of results.

Novo Nordisk

In November 2020, Novo Nordisk made up a shortfall of funding that resulted from a cut back by 10% of the main grant by the funder JDRF due to the COVID-19 pandemic. By this time, the Protocol had been finalised. The company does not have a designated role or responsibility in trial design, conduct, data analysis and interpretation, manuscript writing, or dissemination of results.

Supply shortages of semaglutide (various strengths) in 2022 resulted in actual and potential disruption to participant recruitment and ongoing treatment. In response to a direct request from the UK Chief Investigator, Novo Nordisk donated supplies of semaglutide to the Glasgow site based on anticipated use during the period of disruption.

ROLES AND RESPONSIBILITIES OF TRIAL MANAGEMENT COMMITTEES/GROUPS & INDIVIDUALS

Trial Steering Committee (TSC)

The role of the TSC is to provide overall supervision of the trial and ensure that it is being conducted in accordance with the principles of GCP and the relevant regulations. The TSC will:

1. agree the trial protocol and any protocol amendments
2. ensure that conduct of the trial is fully harmonised between the two sites
3. provide advice to the investigators on all aspects of the trial

It will have an independent Chairperson and include the US and UK investigators and key protocol contributors listed above.

Decisions about continuation or termination of the trial or substantial amendments to the protocol will be the responsibility of the TSC who will advise the co-sponsors. The TSC will meet at the start of the study, and four to six monthly thereafter. The TSC will have its own Charter outlining the role and responsibilities of its members. The TSC may invite other attendees from the trial team to present or participate in discussions on particular topics. These attendees will be non-voting members.

Independent data monitoring committee (IDMC)

An IDMC will be established to include two independent medical experts (covering the domains of type 1 diabetes and metabolic disease; one of the academic clinicians will act as chair) and an independent biostatistician. The Glasgow CTU will liaise with the committee and ensure that the Committee is provided with adequate information about study progress and results.

The IDMC will have a formal Charter outlining the responsibilities of the IDMC members, Glasgow CTU and the co-sponsors. Responsibilities include:

- To protect the safety of participants recruited to the trial.
- Advising the TSC and co-sponsors if it is safe and appropriate to continue with the study
- Examining information provided by the Glasgow CTU on study recruitment, adverse events and outcomes
- Providing reports for the Project Office to forward to the TSC, ethics committees, regulatory bodies, study co-sponsors, and the BHF.

The IDMC may recommend to the TSC to terminate the study or specific aspects of the study if clear harm is observed with one treatment option compared to standard of care or placebo. Specific safety endpoints that will be reviewed include rates of hypoglycaemia and ketosis/ ketoacidosis. The IDMC will not formally review efficacy or futility.

The IDMC will meet approximately every six months.

Study Management Group (SMG)

Trial activity at the UK/ Glasgow site will be coordinated from NHS Greater Glasgow & Clyde (NHS GG&C) by the TTT1 Study Management Group (SMG). The SMG will consist of the UK chief investigator, co-principal investigator other co-applicants, project manager and representatives from the Glasgow CTU, NHS GG&C and The University of Glasgow. The role of the group is to monitor all aspects

of the conduct and progress of the trial in the UK, ensuring adherence to the protocol, and take appropriate action to safeguard participants and the quality of the trial itself.

<u>Joint Study Management Group</u>

Coordination of trial activity between the UK/ Glasgow and the US/ Buffalo site will be facilitated by the Joint TTT1 Study Management Group. This will consist of the US and UK chief investigators, co-principal investigators, other co-applicants, study coordinators, project managers and governance/ administrative representatives as appropriate from State University of New York (SUNY), the Glasgow CTU, NHS GG&C and The University of Glasgow. It will meet approximately every 4-6 weeks. The role of the group is to coordinate all aspects of the conduct and progress of the trial between the US and UK sites, ensuring protocol adherence, and taking appropriate action to safeguard participants and the quality of the trial itself.

Protocol contributors

The protocol has been developed by a group with extensive clinical and research experience in type 1 diabetes and clinical trials including Professor John Petrie and Professor Paresh Dandona. Professor Ian Ford and Professor Alex McConnachie have research expertise in design, conduct, analysis and interpretation of clinical trials and biostatistical methods.

KEY WORDS:

Adjunct therapy
Dapagliflozin
Glycated haemoglobin (HbA1c)
Semaglutide
Type 1 diabetes

LIST of CONTENTS

RESEARCH REFERENCE NUMBERS 2
SIGNATURE PAGE 3
KEY TRIAL CONTACTS 4
LIST of CONTENTS 14
LIST OF ABBREVIATIONS 15
TRIAL FLOW CHART 17
1 BACKGROUND 20
2 RATIONALE 21
3 OBJECTIVES AND OUTCOME MEASURES/ ENDPOINTS 25
4 TRIAL DESIGN 28
5 STUDY SETTING 28
6 ELIGIBILITY CRITERIA 28
7 TRIAL PROCEDURES 30
8 TRIAL MEDICATION 58
9 PHARMACOVIGILANCE 63
10 STATISTICS AND DATA ANALYSIS 70
11 DATA HANDLING 71
12 MONITORING, AUDIT & INSPECTION 73
13 ETHICAL AND REGULATORY CONSIDERATIONS 73
14 DISSEMINATION POLICY 77
15 REFERENCES 78
16. APPENDICES 81

LIST OF ABBREVIATIONS

ACS	Acute Coronary Syndrome
AUC	Area Under the Curve
AE	Adverse Event
AR	Adverse Reaction
CA	Competent Authority
CABG	Coronary Artery Bypass Graft
CGM	Continuous Glucose Monitoring
CHI	Community Health Index
CI	Chief Investigator
CRO	Contract Research Organisation
CRP	C-Reactive Protein
CSII	Continuous Subcutaneous Insulin Infusion
CTA	Clinical Trial Authorisation
CTU	Clinical Trials Unit
CTIMP	Clinical Trial of Investigational Medicinal Product
CV	Cardiovascular
CVA	Cerebrovascular Accident
DKA	Diabetic Ketoacidosis
DSUR	Development Safety Update Report
EC	European Commission
ECG	Electrocardiogram
eCRF	Electronic Case Report Form
EDTA	Ethylenediaminetetraacetic acid
eGFR	Estimated Glomerular Filtration Rate
EMEA	European Medicines Agency
EU	European Union
EUCTD	European Clinical Trials Directive
EudraCT	European Clinical Trials Database
EudraVIGILANCE	European database for Pharmacovigilance
FBC	Full Blood Count
FGM	Flash Glucose Monitoring
GCP	Good Clinical Practice
GDG	Guideline Development Group
GDPR	General Data Protection Regulation
GLP-1	Glucagon Like Peptide-1
GLP-1RA	Glucagon Like Peptide-1 Receptor Agonist
GMP	Good Manufacturing Practice
HbA1c	Glycated haemoglobin
IB	Investigator Brochure
ICF	Informed Consent Form
ICH	International Conference on Harmonisation of technical requirements for registration of pharmaceuticals for human use.
IDMC	Independent Data Monitoring Committee
IMP	Investigational Medicinal Product
IMPD	Investigational Medicinal Product Dossier
ISF	Investigator Site File
ISRCTN	International Standard Randomised Controlled Trials Number

IWRS	Interactive Web-based Randomisation System
LPLV	Last Patient Last Visit
MA	Marketing Authorisation
MDRD	Modification of Diet in Renal Disease
MODY	Maturity Onset Diabetes of the Young
MHRA	Medicines and Healthcare products Regulatory Agency
MI	Myocardial Infarction
MDI	Multiple Daily Injections
NHS GG&C	National Health Service Greater Glasgow & Clyde
NHS R&D	National Health Service Research & Development
NICE	The National Institute for Health and Care Excellence
NIMP	Non-Investigational Medicinal Product
PI	Principal Investigator
PIC	Participant Identification Centre
PIS	Participant Information Sheet
PV	Pharmacovigilance
QA	Quality Assurance
QC	Quality Control
QoL	Quality of Life
QP	Qualified Person
RCT	Randomised Control Trial
REC	Research Ethics Committee
SAE	Serious Adverse Event
SAP	Statistical Analysis Plan
SAR	Serious Adverse Reaction
SDV	Source Data Verification
SGLT2	Sodium Glucose Transporter 2
SOP	Standard Operating Procedure
SUNY	State University of New York
SmPC	Summary of Product Characteristics
SSI	Site Specific Information
SUSAR	Suspected Unexpected Serious Adverse Reaction
TIA	Transient Ischaemic Attack
TMG	Trial Management Group
TSC	Trial Steering Committee
TMF	Trial Master File
UB	University of Buffalo
ULN	Upper Limit of Normal reference range
WOCBP	Women Of Childbearing Potential

TRIAL FLOW CHART

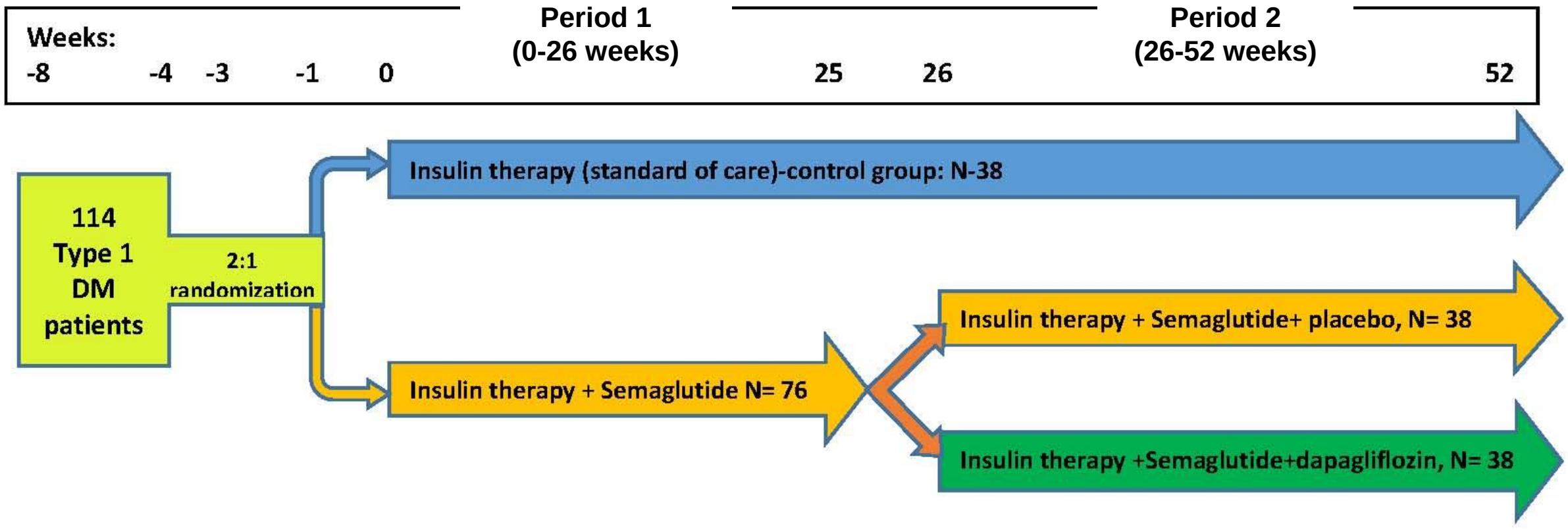

SCHEDULE OF ASSESSMENTS

**Visit #**	**-**	**1**	**2**	**3**	**4[3]**	**5[3]**	**6**	**7**	**8**	**9**	**10**	**11[3]**	**12**	**13**	**14**	**15**	**16**
**Week# (±7 days; see below)**	**-8 to -4**	**-3**	**-1**	**0**	**1**	**2**	**4**	**13**	**23**	**25**	**26**	**27**	**31**	**39**	**49**	**51**	**52**
Study Procedures	Screening	Run-in period		Randomise							Randomise						End
Informed Consent	X																
Fasting visit			X	X				X		X	X			X		X	X
History and demographics	X																
Physical	X																X
Education on hypoglycaemia and ketosis avoidance)				X							X						
BP, heart rate, weight	X	X	X	X	X	X	X	X	X	X	X	X	X	X	X	X	X
Bloods (FBC, kidney and liver profiles)	X			X				X			X		X	X			X
C-peptide	X										X						X
Pregnancy test	X			X			X[1]	X[1]	X[1]		X[1]		X[1]	X[1]	X[1]	X[1]	X[1]
Dispense open-label semaglutide[2]				X		X[2]	X[2]	X[2]			X[2]			X[2]			
Titrate open-label semaglutide[2]						X[2]	X[2]	X[2]									
Dose Semaglutide (on-site if dose is due on visit day)				X	X	X											
Dispense dapagliflozin/placebo											X	X		X			
Dapagliflozin/placebo return capsule counts												X		X			X
HbA1c	X			X				X		X				X			X
Blood endpoints (including fructosamine)				X				X			X			X			X
Masked CGM fit (enter data this visit)		X							X						X		
Masked CGM read				X							X						X
Daily fingerstick glucose review		X	X	X	X	X	X	X	X	X	X	X	X	X	X	X	X
Daily urinary ketones review												X	X	X	X	X	X
Insulin dose review		X	X	X	X	X	X	X	X	X	X	X	X	X	X	X	X
Ketone bodies (blood/urine)	X	X	X	X	X	X	X	X	X	X	X	X	X	X	X	X	X
Adverse Event reporting		X	X	X	X	X	X	X	X	X	X	X	X	X	X	X	X
Meal Challenge Test			X							X						X	
24 hr Urine dispensed			X				X			X			X			X	
24 hr Urine collected				X				X			X			X			X
Quality of life questionnaires				X							X						X

N.B. Safety phone calls will be conducted at weeks 3, 5, 8, 16, 20, 29, 35, 44 and at two weeks after the end of the study to document any side effects or other changes. **Abbreviations:** see pages 14-15

Additional visits or phone calls may be scheduled with the verbal consent of the participant if study team believe necessary e.g. closer monitoring is appropriate (see Patient Information Sheet)

Visit dates should be within seven days of the visit time; however, a longer or shorter interval does not constitute a protocol deviation

[1]Not required in women randomised to "Insulin therapy (standard of care)

[2]According to randomisation schedule: resupply of open-label semaglutide after Visit 3 will be according to individual requirements as tolerated (see Section 8.3)

[3] Visits 4, 5 and 11 have the option of being carried out by telephone for those participants allocated to the standard care arm only.

STUDY PROTOCOL
Triple Therapy for Type 1 Diabetes with Insulin, Semaglutide, and Dapagliflozin

1 BACKGROUND

The control of glucose homeostasis in type 1 diabetes is fragile since β-cell reserve is negligible. Traditionally, insulin has been the only treatment approach available. This often leads to erratic blood glucose control given the challenge of matching exogenous insulin to mealtime glucose excursions in the face of a lack of significant endogenous insulin to compensate for variation in requirements in response to meals and activity levels. Injected insulin boluses rarely match precise insulin requirements either in terms of dose administered or bio-availability. The result often is wide fluctuations in blood glucose including episodes when it is too low (hypoglycaemia). Based on our work to date and other published reports, previous attempts to add other glucose-lowering medications to insulin therapy yielded minimal benefits on glycaemia (although metformin may have other benefits) (1-3).

Dandona et al. have shown in two preliminary retrospective studies that the addition of liraglutide, a Glucagon Like Peptide -1 receptor agonist (GLP-1RA), to insulin results in consistent improvements in glycemic control in type 1 diabetes as demonstrated by reductions in HbA1c, glycemic excursions, and insulin requirements accompanied by improvements in body weight and systolic blood pressure (4, 5). As collaborators in the present trial, they also completed the first prospective randomised controlled trial of this drug in 72 people with type 1 diabetes. The data not only confirmed improvements in glycemic control (HbA1c), body weight and systolic BP, but also demonstrated a reduction in appetite, carbohydrate intake and low-grade inflammation (6). Importantly, quality of life was also improved. Their recent studies in type 2 diabetes have revealed that systolic blood pressure reduction with GLP-1RA (exenatide) treatment in type 2 diabetes is associated with increased plasma concentrations of ANP, cGMP and cAMP along with reduced concentrations of angiotensinogen, renin and angiotensin II (7). It is possible that these mechanisms play a role in the prevention of cardiovascular complications in type 1 diabetes. There have been no trials to date of semaglutide (a weekly and potent GLP-1RA) in type 1 diabetes.

GLP-1RAs, therefore, have great potential as adjunct therapy for type 1 diabetes. However, mean HbA1c was still ≥7.0% after liraglutide treatment (6) and larger pharma-sponsored trials (ADJUNCT-1 and ADJUNCT-2) reported increased rates of hypoglycaemia (8, 9). In seeking further improvement, the advent of Sodium-GLucose coTransporter 2 (SGLT2) inhibitors is of importance since their mode of action is independent of insulin secretion. Introduced for use in type 2 diabetes, SGLT2 inhibitors are selective antagonists of the sodium-glucose transporter subtype expressed in the proximal convoluted tubules of the kidneys. They act by inhibiting renal glucose re-absorption and therefore stimulating excretion of 60-100 grams per day of glucose in urine. SGLT2 inhibitors have been studied extensively in type 2 diabetes, including the landmark cardiovascular outcome trial EMPA-REG (10), but data have only more recently begun to accumulate in type 1 (11). Short term effects on urine glucose excretion of remogliflozin and empagliflozin are similar to those seen in type 2 diabetes and these translate into improved glycaemic and other metabolic indices including HbA1c, fasting blood glucose, rates of hypoglycaemia, daily insulin dose and weight (12). Glucose-lowering efficacy is attenuated in individuals with eGFR < 45/ml/min/1.73 m^2.(13) Weight loss accompanying increased urinary glucose excretion could translate into reduced cardiovascular complications as in type 2 diabetes, but there are no data so far available to support this hypothesis. More recently, in the Phase 3 trials DEPICT-1 and DEPICT-2 Dandona et al (PI of the US arm of this trial) showed that dapagliflozin improves glycaemic control and HbA1c over six months by about 0.45% without increasing hypoglycaemia and as in other conditions reduces urinary albumin excretion (14,15,16,17). In comparison to other SGLT2 inhibitors studied for use in type 1 diabetes [sotagliflozin and empagliflozin (18, 19)] the risk of diabetic ketoacidosis was relatively modest (see below). Both dapagliflozin and sotagliflozin were approved early in 2019 by the EU Committee for Medicinal Products for Human Use (CHMP) as adjunct therapy in type 1 diabetes for individuals with BMI≥27 kg/m (20,21)2. In August 2019, dapagliflozin (5 mg/ day) was recommended by the National Institute for Health and Care Excellence as cost-effective for this indication in NHS England (22 However, dapagliflozin did not gain approval for this indication in the

USA from the FDA (23). In November 2021, the manufacturer AstraZeneca voluntarily removed this Europe- and UK-specific indication (24) explicitly "not due to any safety concern for dapagliflozin in any condition, including type 1 diabetes" (25).

2 RATIONALE

General hypothesis: adding an SGLT2 inhibitor (dapagliflozin) to a combination of insulin and semaglutide will improve glycaemic control, weight and blood pressure in adults with type 1 diabetes.
Our protocol is designed to allow an objective assessment of whether this triple therapy combination will work effectively and safely so that a majority of people with type 1 diabetes can reach a glycaemic target HbA1c of ≤7.0%. In addition, since both semaglutide and dapagliflozin inhibitors reduce blood pressure and weight, it will allow an assessment of the combined effect of GLP-1RAs and SGLT2 inhibitors on these outcomes. Since there are no previous data on the effects of once weekly semaglutide in type 1 diabetes, or the combination of semaglutide and dapagliflozin compared to standard insulin treatment, the design requires allocation of some participants to insulin therapy only (the current standard of care).

As part of the process by which this trial was awarded funding by JDRF, this research question was considered by lay members of the funding panel and deemed important, relevant and timely. We considered that use of placebo matching dapagliflozin in Period 2 of the trial was justified when dapagliflozin had full approval as adjunct therapy for type 1 diabetes in Europe (although not the USA) as it remained uncertain which adults with type 1 diabetes were suitable for this treatment. In their review, NICE considered it likely that fewer than 10% of adults with type 1 diabetes would be initiated on SGLT2 inhibitor therapy over the first five years after their favourable decision. As individuals with a slightly lower BMI threshold will be included than recommended by the SmPC, trial participants will be more closely monitored for ketosis than was the case when dapagliflozin was approved for routine clinical care. Dapagliflozin (or placebo) will be administered orally once daily as per current licensed doses in type 2 diabetes (5 mg/day and 10 mg/day: the same doses as used in DEPICT-1 and DEPICT-2) (14,15,16,). We have not incorporated blinding for semaglutide in the design as the effects of the closely-related daily GLP-1RA liraglutide have been well-studied in double-blind trials in type 1 diabetes by ourselves and others, and individuals in both arms compared for the primary outcome comparison will be treated with this agent. (6, 8, 9). Semaglutide will be administered subcutaneously once weekly. The need to study combination adjunct therapy with SGLT 2 inhibitors and GLP-1 RAs is greater than ever following removal of the type 1 diabetes indication for SGLT2 inhibitor monotherapy in the UK and Europe.

2.1 Assessment and management of risk

a) Benefits

Semaglutide (Ozempic): is a once weekly injectable (subcutaneous) GLP1-RA licensed for reducing HbA1c in type 2 diabetes. The licensed doses are 0.25 mg weekly, 0.5 mg weekly and 1.0 mg weekly. In the trial, the starting dose is 0.25 mg weekly uptitrated if tolerated to 0.5 mg weekly after two weeks and then (again if tolerated) after a further two weeks to the maximum dose of 1.0 mg weekly. Semaglutide differs from other weekly GLP-1 receptor agonists in that its half-life is extended by binding to serum albumin i.e. it is not macromolecular. In type 2 diabetes, semaglutide therapy is associated with weight reduction and a reduction in rates of cardiovascular events (26). The licensed dose escalation schedule for routine treatment of type 2 diabetes is at four weekly intervals (27) but in a Phase 2 trial in type 2 diabetes authored by one of this trial's investigators (JP) doses were uptitrated at one-to-two weekly intervals and to higher doses (up to 1.6 mg weekly) (28). Phase 3 trials are currently in progress (for the indication of obesity) using a 2.4 mg semaglutide weekly. It should be noted that semaglutide 0.25 mg is considered too low a dose for maintenance therapy in type 2 diabetes but it is unclear whether this may have a beneficial effect in type 1 diabetes at this dose.

While semaglutide once weekly has not been investigated previously in type 1 diabetes, liraglutide (a GLP-1 RA for daily administration with a similar molecular structure that binds less avidly to serum albumin) has previously been investigated (see above) in the ADJUNCT 1 and ADJUNCT 2 trials (8,9) in which, compared with placebo it resulted in reductions in HbA1c, weight and insulin requirement, albeit with a small increase in rates of hypoglycaemia (see below).
Dapagliflozin (Forxiga): is a once daily oral SGLT2 inhibitor previously licensed in Europe for reducing HbA1c in people with type 1 diabetes and BMI ≥ 27 kg/m^2 when insulin alone does not provide adequate glycemic control despite optimal insulin therapy (23). Although only the 5 mg dose was licensed for type 1 diabetes,both 5 mg and 10 mg daily are licensed for type 2 diabetes (29). As mentioned above, the removal of the type 1 diabetes indication by AstraZeneca was not due to any safety concern for any indication, including type 1 diabetes. In the Phase 3 trials of dapagliflozin in type 1 diabetes (DEPICT-1 and DEPICT-2) both doses were used. In both DEPICT-1 and DEPICT-2 there were modest additional benefits with 10 mg compared to 5 mg dapagliflozin. For example, in DEPICT-1, insulin dose requirement was reduced by 13.2% vs placebo on 10mg daily compared with 8.8% on 5mg daily (14). Weight reduction vs placebo in DEPICT-1 at 52 weeks was by 4.5% on dapagliflozin 10mg daily compared with 2.95% in those receiving 5 mg daily. Given the propensity of insulin to encourage weight gain over time and the life-long insulin requirement in T1D, sustained weight reduction and insulin dose reduction - even when modest - may be clinically important. There was no increase in the rate of hypoglycaemia in either the 5 mg or 10 mg groups compared with placebo. (14,15)
Dapagliflozin therapy in type 2 diabetes has been demonstrated to be associated with cardiovascular and renal risk reduction: in DECLARE-TIMI, DAPA-HF and DAPA-CKD the 10 mg dose was used (30, 31, 32). Such cardiovascular and renal benefits may translate to type 1 diabetes with appropriate monitoring and risk mitigation, although support for such measures by the manufacturer were withdrawn in the UK at the time of the removal of the type 1 indication . Given these potential benefits with relatively low increased risk, the 10 mg rather than 5 mg dose was selected for steady state treatment in this protocol.

b) Risks/ side effects Please also refer to the respective UK SmPCs (27, 29)

<u>Semaglutide</u>:
Very common: nausea, diarrhoea; hypoglycaemia (when used with insulin).
Common: vomiting, abdominal pain, constipation, gastro-oesophageal reflux, cholelithiasis, weight loss.
Uncommon: injection site reactions, hypersensitivity
Rare: Anaphylactic reaction.

Hypoglycaemia/ ketosis: Semaglutide once weekly has not been studied previously in type 1 diabetes; however, the ADJUNCT-1 and ADJUNCT-2 trials examined the role of liraglutide once daily (a similar molecule in the GLP-RA class that binds less avidly to serum albumin). Although HbA1c and weight were reduced by both 1.2 mg and 1.8 mg liraglutide, rates of symptomatic hypoglycaemia increased by 20-30%. In ADJUNCT-1, insulin doses were re-uptitrated quite rapidly towards baseline levels after an initial dose reduction, but in ADJUNCT-2 were "capped" (and therefore remained lower). Perhaps for that reason, an increase in hypoglycaemia was less consistent in ADJUNCT -2 but there was an excess of ketosis the higher (1.8 mg) dose, presumably due to under-insulinisation (no lower limit for insulin dose reduction was set). It should be noted that monitoring of participants by site investigators in the ADJUNCT trials was less intensive than will be the case in the present trial with less frequent opportunities for insulin dose titration (see Section 7). Importantly, participants at high risk of hypoglycaemia, (including those with a history of severe hypoglycaemia and those with hypoglycaemia unawareness) were not excluded (8,9).

Retinopathy: In the SUSTAIN-6 cardiovascular outcome trial of semaglutide in type 2 diabetes, rapid reduction of HbA1c was associated with progression of existing proliferative retinopathy in those with a high baseline HbA1c ("early worsening"/ "normoglycaemic re-entry"). However, there was no evidence of worsening of retinopathy on treatment in the trials SUSTAIN 1-5 in which those with proliferative retinopathy were excluded (33, 3). In clinical trials of duration up to one year involving 4,807 patients with type 2 diabetes, adverse events related to diabetic retinopathy were reported in similar proportions of subjects treated with semaglutide (1.7%) and comparators (2.0%) (33).

Other adverse effects: In type 2 diabetes, gastrointestinal adverse effects of semaglutide can be prevented in the majority of cases by careful dose uptitration (28, 34) although up to 5% are ultimately unable to tolerate it. In our previous trial with liraglutide in T1D, adverse effects occurred with a similar frequency to that observed in T2D (in which there is extensive clinical experience); on this basis it is expected that this will also be the case with semaglutide (8,9).

Dapagliflozin:
Very common: hypoglycaemia (when used with insulin).

Common: ketoacidosis, genital infections (vulvovaginitis, balanitis), urinary tract infection, diabetic ketoacidosis, increased creatinine.

In the DEPICT-1 study, ketoacidosis occurred in 4 of 277 participants on 5mg dapagliflozin, 5 of 296 participants on 10 mg and 3 of 260 participants in the placebo group (summarised as 1%, 2% and 1% respectively) with one severe episode in each group (14). In pooled analyses of DEPICT-1 and DEPICT-2 (14,15) there was, however, an approximate threefold elevated rate of DKA adjudicated as "definite" over placebo on active therapy: 2.0% on 5mg dapagliflozin, 1.9% on 10mg and 0.6% on placebo – i.e. rates of DKA were similar with the two doses (14,15,16). Rates of DKA with dapagliflozin were described as "occurring in at least 1 per 100 patients" by the manufacturer at the time the indication was removed (24). Rates of DKA with sotagliflozin, the other SGLT2 inhibitor licensed for type 1 diabetes, were somewhat higher (2.9% on 200 mg, 3.8% on 400 mg, 0.2% on placebo) in pooled analyses of Phase 3 trials (19,21).

Increased creatinine: in 13 studies summarized in the Summary of Product Characteristics increases in serum creatinine (generally transient) occurred in 3.2% vs 1.8% of individuals with eGFR ≥ 60 ml/min/1.73 m^2 who received dapagliflozin 10 mg vs placebo rising to 18.5% vs 9.3% in those with eGFR <30 ml/min/1.73 m^2 (16,29). These changes are usually observed 4 weeks after SGLT2i initiation, recovering by week 12. Participants with eGFR < 45 ml ml/min/1.73 m^2 will not be included in the TTT1 trial and creatinine/ eGFR will be monitored at 5 weeks (Visit 12) and 13 weeks (Visit 13) after randomisation to dapagliflozin or placebo (Visit 10). Any participant who has persistent reduction in eGFR of 15 ml/min or more will meet withdrawal criteria from the trial (see Section 7.8).

Uncommon: fungal infection, volume depletion, constipation.
Not known (several case reports): Fournier's gangrene.

Fournier's gangrene is a very rare clinical entity characterized by necrotizing fasciitis of the perineal area. This has been infrequently reported in association with SGLT-2 inhibitor use in both men and women. However, there was no evidence of increased risk in a meta-analysis of 84 clinical trials involving 42415 participants. Nevertheless, participants will be advised to seek urgent medical assistance if they experience erythema, pain, swelling of the perineal area associated with fever and malaise (this will be reinforced in the Patient Information Sheet). (36)

Amputation: An increased incidence of lower limb amputation has been reported in type 2 diabetes with the SGLT-2 inhibitor canagliflozin. However, this has not been demonstrated with dapagliflozin (not included in the UK SmPC). People with diabetes are at increased risk of amputation and good foot care is a key component of good diabetes care regardless of use of SGLT-2 inhibitors.

<u>Placebo</u>:
Placebo capsules contain microcrystalline cellulose.
This trial is therefore categorised by the Co-Sponsors as:

- Type B = Somewhat higher than the risk of standard medical care

See Appendix 1 (Risk Assessment)

3 OBJECTIVES AND OUTCOME MEASURES/ ENDPOINTS

3.1 Primary objective

- To assess in people with type 1 diabetes whether triple therapy (dapagliflozin, semaglutide and insulin) for six months improves glycaemic control (as measured by HbA1c) compared with dual therapy (placebo, semaglutide and insulin –Period 2).

3.2 Secondary objectives

To assess in people with type 1 diabetes whether:

- Triple therapy (dapagliflozin, semaglutide and insulin) improves glycaemic control (as measured by HbA1c) compared with standard therapy (insulin)
- Dual therapy (semaglutide and insulin – period 1) improves glycaemic control (as measured by HbA1c) in comparison with standard therapy (insulin only)
- Triple therapy (dapagliflozin, semaglutide and insulin) improves glycaemic control as assessed by a range of continuous glucose monitoring (CGM) parameters, lowers body weight, reduces insulin requirement, improves blood pressure and improves quality of life (as assessed by validated patient questionnaires) compared with dual therapy and standard therapy.

3.3 Outcome measures/endpoints

3.4 Primary endpoint/outcome

Change in HbA1c (week 26 to week 52)

3.5 Secondary endpoints/outcomes

Efficacy:

Change in HbA1c

Mean fasting blood glucose

Mean blood glucose*
Percentage time in range (3.9 - 10.0 mmol/)*
Standard deviation of blood glucose*
Percentage time in hyperglycaemia Level 1 (10.1 - 13.9 mmol/L/)*
Percentage time in hyperglycaemia Level 2 (>13.9 mmol/L) *
Percent time in hypoglycaemia Level 1 (3.0 - 3.8 mmol/)*
(by masked DEXCOM G6 CGM)*

Insulin requirement
Bodyweight
Systolic and diastolic BP
Number of antihypertensive medications
Diabetes Specific Quality of Life Scale (DSQOLS)
Problem Areas In Diabetes (PAID) questionnaire

Safety:

Rate of hypoglycemic events Level 2 (<3.0 mmol/L/ <54mg/dl)
Rate of hypoglycemic events Level 3 (severe event with altered mental and/or physical status requiring assistance)
Rate of diabetic ketoacidosis defined as elevated serum or urine ketones (greater than the upper limit of the normal range), and serum bicarbonate < 15 mmol/L or blood pH < 7.3

3.6 Tertiary endpoints/outcomes

Postprandial glucose (AUC) – meal tolerance test
Postprandial glucagon (AUC) – meal tolerance test
Postprandial FFA (AUC) – meal tolerance test
Postprandial GLP-1 (AUC) – meal tolerance test
Fructosamine

3.7 Exploratory endpoints/outcomes

Plasma and urine samples will be collected and frozen for later analysis for indices of inflammatory stress and other relevant measures.

3.8 Table of endpoints/outcomes

Endpoints	Outcome Measures	Timepoint(s) of evaluation of this outcome measure
**Primary endpoint**<br>Triple therapy (dapagliflozin, semaglutide and insulin) in comparison with dual therapy (placebo, semaglutide and insulin – period 2)	Change in HbA1c	Week 26 to Week 52
**Secondary endpoint 1**<br><br>Triple therapy (dapagliflozin, semaglutide and insulin) in comparison with standard therapy (insulin alone)	Change in HbA1c	Week 0 to Week 52
**Secondary endpoint 2**<br><br>Dual therapy (semaglutide and insulin – period 1) in comparison with standard therapy (insulin only)	Change in HbA1c	Week 0 to Week 26
**Other secondary endpoints**<br><br>a) Triple therapy (dapagliflozin, semaglutide and insulin) in comparison with dual therapy (semaglutide, insulin and placebo – period 2)<br><br>b) Dual therapy (semaglutide and insulin – period 1) in comparison with standard therapy (insulin only)	Change in:<br>- Mean fasting blood glucose (4 days)<br><br>- Mean blood glucose*<br>- Percentage time in range (3.9–10 mmol/L)*<br>- Glycaemic variability (SD)*<br>- Percent time in hyperglycaemia Level 1 (10.1-13.9 mmol/L)*<br>- Percent time in hyperglycaemia Level 2 (>13.9	Week 26 to Week 52<br><br>Week 0 to Week 26

	mmol/L)*<br>- Percent time in hypoglycaemia Level 1* (3.0-3.8 mmol/L)<br>*(*by masked Dexcom G6 CGM)*<br><br>Insulin requirement<br>Body Weight<br>Systolic and diastolic blood pressure<br>Number of antihypertensive medications<br>Diabetes Specific Quality of Life Scale (DSQOLS)<br>Problem Areas In Diabetes (PAID) questionnaire	
**Tertiary endpoints**<br>a) Triple therapy (dapagliflozin, semaglutide and insulin) in comparison with dual therapy (semaglutide, insulin and placebo –period 2)<br><br>b) Dual therapy (semaglutide and insulin – period 1) in comparison with standard therapy (insulin only)	Change in:<br>Postprandial glucose (AUC)<br>Postprandial FFA (AUC),<br>Postprandial GLP-1 (AUC)<br>Postprandial glucagon (AUC)	Week 26 to Week 52<br><br>Week 0 to Week 26
**Safety endpoint 1**<br>a) Triple therapy (dapagliflozin, semaglutide and insulin) in comparison with dual therapy (semaglutide, insulin and placebo – period 2)<br><br>b) Dual therapy (semaglutide and insulin – period 1) in comparison with standard therapy (insulin only)	Difference between treatment arms in:<br>(i) Rate of Level 2 (<3.0 mmol/L/ <54mg/dL) hypoglycemic events<br>(ii) Rate of Level 3 hypoglycemic events (severe event with altered mental and/or physical status requiring assistance)	Week 26 to Week 52<br><br>Week 0 to Week 26
**Safety endpoint 2**<br>a) Triple therapy (dapagliflozin, semaglutide and insulin) in comparison with dual therapy (semaglutide, insulin and placebo – period 2)	Difference between treatment arms in:<br><br>Rate of diabetic ketoacidosis (serum or urine ketones above normal limits) AND serum bicarbonate < 15 mmol/L OR blood pH < 7.3	Week 26 to Week 52

b) Dual therapy (semaglutide and insulin – period 1) in comparison with standard therapy (insulin only)		Week 0 to Week 26

4 TRIAL DESIGN

The trial has two six-month periods:

Period 1 open-label randomised (2:1) parallel group semaglutide once weekly + insulin (standard treatment) vs insulin alone (standard treatment).
Period 2 double-blind, randomised (1:1) parallel group, dapagliflozin vs placebo for those participants initially randomised to semaglutide (with ongoing standard of care for all participants)

Thus, during the first six months, approximately 114 participants with type 1 diabetes (50 in the UK) will be randomised (open label, see below) into three groups (approximately 38 each; i.e. 19 each in the UK) one of which will be maintained on their regular insulin therapy for 12 months (standard therapy group) and two of which will be started on semaglutide in addition to regular insulin treatment for six months (dual therapy). Following six months of stable treatment, those initially randomised to insulin and semaglutide will be additionally randomised (double-blind) to dapagliflozin (triple therapy) or matching placebo (dual therapy) for a further six months. The dose of dapagliflozin will not be increased to 10 mg in those who have confirmed ketonuria (+1) or ketonaemia (≥0.6 mM) after the first week of treatment.

In keeping with the intention-to-treat principle, participants in whom therapy is discontinued or downtitrated due to adverse effects will be encouraged to continue to undertake all study assessments and attend study visits.

5 STUDY SETTING

The study will be conducted at two sites: State University of New York (SUNY) at Buffalo, USA and Glasgow, UK. In Glasgow, n=50 participants (45% of the total) will be identified from secondary care sites and via the Scottish Diabetes Research Network research register. Both randomisations will be stratified by site.

6 ELIGIBILITY CRITERIA

6.1 Inclusion criteria

1) Type 1 Diabetes for at least one year on stable multiple daily (four or more) injections (MDI) of insulin or continuous subcutaneous insulin infusion (CSII) for the previous three months.
2) C-peptide < 0.23 nM
3) Minimum dose of insulin: 0.5 U/kg for MDI and 0.4 U/kg for CSII
4) Regularly measuring blood glucose: four or more times daily either by fingerstick, testing or by "flash"/ continuous glucose monitoring)
5) HbA1c 7.5 – 11.0 % inclusive
6) Well versed in carbohydrate1 counting*
7) Age 18-70 years.

8) BMI ≥25 kg/m^2.

** In the opinion of the site Principal Investigator*

6.2 Exclusion criteria

1) Type 1 diabetes for < 12 months, type 2 diabetes, chronic pancreatitis, MODY
2) Previous use of any agent other than insulin for glycaemic control in the last three months.
3) History of diabetic ketoacidosis (DKA) requiring medical intervention (e.g. emergency room visit and/or hospitalisation) within three months prior to the screening visit.
4) Following ketogenic diet, low (<50g/day) carbohydrate diet or intermittent fasting.
5) Hypoglycaemia unawareness* or frequent episodes of severe hypoglycaemia as defined by more than one episode requiring medical assistance, emergency care (paramedics or emergency room care), or glucagon therapy administered by a third-party individual within three months prior to the screening visit
6) Symptoms of poorly controlled diabetes
7) Participation in a weight loss program with ongoing weight loss, or in an intensive exercise program
8) History of bariatric or other weight-loss surgery within 12 months prior to the screening visit
9) History of Addison's disease or chronic adrenal insufficiency
10) History of diabetes insipidus
11) Hepatic disease or cirrhosis
12) Aspartate Aminotransferase (AST) > 3 X Upper limit of normal (ULN) and/or Alanine aminotransferase (ALT) > 3 X ULN
13) Serum Total Bilirubin > 2 X ULN unless exclusively caused by Gilbert's Syndrome
14) Haemoglobin < 110 g/L (11.0 g/dL) for men; haemoglobin < 100 g/L (10.0 g/dL) for women
15) Coronary event or procedure (myocardial infarction, unstable angina, coronary artery bypass, surgery or coronary angioplasty) in the previous three months or history of congestive heart failure
16) End stage renal disease on haemodialysis and/or eGFR < 45 ml/min/1.73m^2
17) HIV or Hepatitis B/C positive status
18) Any other life-threatening, non-cardiac disease
19) History of pancreatitis or cystic fibrosis
20) Women who are pregnant, planning pregnancy or breast-feeding and women of childbearing potential (WOCBP) who are not established on a method of contraception considered highly effective (see Appendix 2) and willing to continue it for the duration of the study and for at least two months after last dose of IMP.
21) Inability to give informed consent
22) History of gastroparesis
23) History of medullary thyroid carcinoma or MEN 2 syndrome
24) History of hypersensitivity reaction to GLP-1 agonists or SGLT2 inhibitors or known hypersensitivity to any of the excipients
25) Known gallstones (history of cholecystectomy is not an exclusion criterion)
26) History of alcohol problem or drug abuse
27) Hypertriglyceridemia (>500 mg/dl)
28) Recurrent genital mycotic infection (more than one episode in last six months)
29) Hypovolaemia

30) Any malignancy except treated *in situ* malignancy and basal cell carcinoma of the skin
31) Unexplained haematuria (dipstick positive on two occasions one week apart) and/or active bladder cancer
32) Proliferative retinopathy (on basis of photographic retinal screening which must have been performed within the last 12 months)
33) Use of an investigational agent or therapeutic regimen within 90 days of study
34) Participation in any other concurrent interventional clinical trial
35) Unlikely to be able to comply with study requirements*
36) Unable to undergo meal challenge for dietary reasons (e.g. vegetarian/ vegan diet)

** In the opinion of the site Principal Investigator*

7 TRIAL PROCEDURES

Also see *Schedule of Assessments*

Each participant will be enrolled in the trial for approximately 55 weeks, comprising three weeks of monitoring to stabilise treatment and collect baseline data before randomisation into the intervention or standard of care/ placebo arms for 52 weeks. Following a completed screening visit and meeting the Inclusion and Exclusion criteria, each participant will have a total of 16 scheduled study visits. In addition, at weeks 3, 5, 8, 16, 20, 29, 35, 44 and at two weeks after the end of the study, each participant will receive a phone call to document any adverse effects or other changes. No long-term follow-up is planned following completion of participation in the study.

Participants will be treated with either insulin only (standard treatment) for the whole duration of the study or with insulin and semaglutide weekly s.c. injection (titrated where possible up to 1.0 mg, see below) for the first six month period and then randomised to either oral dapagliflozin (triple therapy) or placebo (dual therapy) daily added for the second six month period. Semaglutide will be started (Visit 4) at a dose of 0.25 mg once weekly for two weeks then increased if tolerated to 0.5 mg (Visit 5). If well tolerated at week 4, it will be increased to 1.0 mg once weekly (Visit 6) or at a subsequent visit at the discretion of the PI. Dapagliflozin (or placebo) will be started at a dose of 5 mg daily at week 26 and increased at week 27 to 10 mg (2 x 5mg capsules) daily to ensure drug tolerability and minimise the risk of insulin-induced hypoglycaemia or hypotension.

Excessive weight loss: Both semaglutide and dapagliflozin are known to cause weight loss in the range of 5-10% in type 2 diabetes. However, the magnitude of weight loss when these are combined in type 1 diabetes is uncertain. Excessive weight loss may lead to increased risk of hypoglycemia or decreased insulin requirement increasing the risk of ketosis or DKA (see Section 2.1b) and other unforeseen risks. Therefore, weight loss during any phase of the study will be limited to ≤ 15% of body weight at randomisation or reaching a BMI <22 kg/m^2. If participants are approaching this threshold, IMP will be sequentially reduced at the discretion of the PI. Semaglutide may be reduced to 0.25mg or 0.5 mg and dapagliflozin to 5 mg. See also Section 7.8 regarding treatment discontinuation.

Covid-19 Mitigation

In view of the Covid-19 pandemic, certain trial procedures may be changed if necessary to reduce in person contact with participants. This will be continuously reviewed in view of the prevailing

circumstances in line with local and national guidance and with the principle of protecting participants and study personnel. In the event of future waves of infection this would be particularly important. In this context, consideration would be given to rearranging visits, changing to virtual visits using video technology or using additional phone calls to ensure patient safety. Consideration will also be given to how minimising face-to-face interaction with participants when dispensing study medication. If required, supplies of study medication sufficient to last for longer periods may be dispensed to minimise contact and visits. Courier services may additionally be used to deliver study medications to participants.

All in person visits will be subject to local and national guidance around the use of personal protective equipment including face masks and where appropriate when conducting procedures, the use of gloves and aprons.

Gastrointestinal adverse effects:

During the initial weeks of participation, those participants who report nausea and/or vomiting during uptitration of semaglutide dose that is not sufficiently severe to require treatment discontinuation will remain on their current dose until they are symptom-free prior to discussing a further dose escalation (i.e. uptitration will be to the highest dose tolerated rather than a "forced titration"). Dose will be downtitrated to the maximum tolerated in those who experience gastrointestinal adverse effects.

Retinopathy:

As there was no excess of retinopathy with semaglutide treatment in Phase 3 trials with semaglutide, in which proliferative retinopathy was an exclusion criterion (as in the current protocol), additional retinal imaging beyond annual retinal photography (the standard of care) is not proposed (30).

Insulin dose adjustment:

Minimising hypoglycaemia: All participants will be made aware of the potential for increased hypoglycaemia with addition of adjunct therapy and supported and encouraged to monitor blood glucose at least four times daily in order to minimize the risk of hypoglycaemia. Participants will have ready access to advice from specialist medical and nursing staff at face-to-face and telephone visits. Those who have ongoing access to wearable continuous glucose monitoring [or "flash" glucose monitoring, (FGM)] will continue to use these devices. Information on management of hypoglycaemia will be included within participant diaries, reinforcing the pre-existing knowledge of participants, the majority of whom will have lived with type 1 diabetes for many years.

Reductions in insulin dose will not usually be greater than 10% (5% if HbA1c is < 7.0 %) from current levels. At randomisation, daily insulin dose will not be reduced by more than 20% from the baseline level and then from the new baseline levels at 26 weeks. Participants requiring an insulin dose reduction > 20% and their total insulin dose < 0.4 U/kg (current weight) will be monitored closely for insulin dose, carbohydrate intake, ketones and blood glucose. Following the second randomisation at 26 weeks, dapagliflozin/ placebo will be temporarily discontinued if insulin dose falls below this threshold and permanently discontinued if it remains so at review in one week.

Minimising ketosis: Participants will be instructed to stay hydrated and not to reduce their caloric intake (especially carbohydrates) or start new dietary approaches (especially ketogenic diets) during the study. They will be educated to recognize the signs and symptoms of diabetic ketoacidosis (DKA) and to undertake additional self-testing of urine and/or blood ketones if they develop nausea, vomiting, or anorexia. In addition, they will be given education and a written ketosis management plan (STICH Protocol – i.e. STop SGLT-2 inhibitor or placebo (temporarily), give Insulin bolus, take additional Carbohydrate, and additional Hydration - see Appendix 3) (37).

From Visit 10 (26 weeks), those participants undergoing the second randomisation will be supported and encouraged to monitor urine or blood ketones at home at least once daily: three days before visit 10 (week 26), they will be asked to start daily measurements of urine ketones [CombiScreen® 10 SYS Plus urine testing strips, Sterilab, UK (or equivalent)] or blood ketones [CareSens™ Dual, i-SENS, Koreal; GlucoMen® Areo 2K, Menarini, UK (or equivalent]]. They will be asked to record these in the diary/ log provided. In addition, ketones (blood and urine) will be measured at every visit. If ketone bodies in urine are +1 or greater (≥0.6 mM in blood), participants will be reminded to keep well hydrated and notify the study team as soon as possible (office hours) as diabetic ketoacidosis (DKA) can occur even at glucose levels < 14 mmol/L (38).

If ketones are +2 or more in urine (> 1.5 mmol/L) participants will be advised to initiate the STICH Protocol immediately and contact the study team in office hours (or telephone NHS 24 or attend their Accident & Emergency if out of hours) (37). This information will be reinforced verbally at study visits and also in the form of a Patient Alert Card that participants will be asked to carry. This will also contain details of the investigational study drug, the study number, the investigator's name, and a contact number (for in hours contact). The study team will assess for symptoms or test results indicating a risk of ketoacidosis immediately on reporting and if blood ketone levels are persistently (one week) at these levels dapagliflozin/placebo will be permanently discontinued.

If blood ketone readings are confirmed as > 3.0 mmol/L (or urine ketones reading +++) participants will be advised to stop dapagliflozin/ placebo and attend the Accident and Emergency department without delay.

Participants will be advised that dapagliflozin/placebo may be temporarily stopped in other clinical situations known to predispose to ketoacidosis (e.g. acute serious medical illnesses or hospitalisation for major surgical procedures). Any requirement for intravenous insulin infusion (whether in Accident and Emergency or as an in-patient admission) will be recorded as a Serious Adverse Event. For further detailed procedures see section 9.31.

Volume depletion:
There is a theoretical risk of volume depletion and low blood pressure when dapagliflozin is used in combination with other anti-hypertensive drugs. All pre-existing concomitant medication will be reviewed at the screening visit as part of the medical history and examination (see Section 7.1.3 for further details). Participants undergoing the second randomisation will be educated on symptoms suggestive of low blood pressure (e.g. light-headedness, dizziness, feeling faint) and asked to contact the study team if they experience these symptoms. In clinical practice however SGLT-2 inhibitors are commonly used with anti-hypertensive drugs with no significant problems (31).

Genital mycotic infections:
Individuals who report symptoms suggestive of genital mycotic infection will be advised to self-treat with topical antifungal preparations. If a second episode occurs, a decision on treatment discontinuation will be considered in discussion between the participant and the site Principal Investigator, taking account of the severity of the symptoms.

Foot care:
Annual foot checks will be conducted for all participants as per standard of care.

7.1 Recruitment

7.1.1 Patient identification

All personal details will be managed according to ISO 27001:2013 compliant Standard Operating Procedures.

Potentially eligible participants will be identified from the Scottish Diabetes Research Network Research Register (a managed and searchable database of people with diabetes who have agreed to be contacted about clinical trial participation) and at secondary care diabetes clinics in Greater Glasgow and Clyde. The principal site for recruitment will be Stobhill Ambulatory Care Hospital but other sites may be opened if necessary as Patient Identification Centre sites. The majority of study visits (and all visits involving drug dispensing) will be conducted at the Clinical Research Facility, Glasgow Royal Infirmary. Information posters with the contact details of the research team will be displayed in recruiting clinics. .

At the principal site, clinic lists (whether remote or face-to-face) will be pre-reviewed by members of the research team. An email will be sent to the clinic Consultant physician to flag potentially eligible individuals on their list; potential eligibility will also be flagged on the clinic electronic list (on Trakcare®) to prompt reviewing physicians (as members of the usual care team) to check basic eligibility criteria, provide brief verbal information about the trial and outline the implications of participation (e.g. number of visits).

In the above email (to which a response is not required), physicians will be asked to add a note indicating the outcome of their discussion with the potential participant to the Clinical Portal system using standard text (e.g. willing to be contacted/ not willing to be contacted/ unsuitable for the trial) and the permitted and preferred method(s) of contact (e.g. mobile telephone/ home telephone/ letter/ email). Physician consultations with potentially eligible individuals that are concluded with no invitation to research participation (e.g. due to pressure of time) can be followed up with a further phone call from one of the study physicians who is also a member of the research team.

Following clinical consultations, members of the research team will identify potential participants who appear to be meet eligibility criteria and have given permission to be contacted via their access to the Clinical Portal system. These individuals will be given or sent/ emailed (as appropriate according to documented permissions) a Full Patient Information Sheet and an Expression of Interest Form (with a prepaid envelope). Where permission has been given for phone contact, this can be followed up by up to two further phone contacts (provided permission to contact has not been withdrawn by the potential participant in an earlier call). A visit to the Clinical Research Facility will then be scheduled for further discussion. The person scheduling the CRF visit will confirm with the patient if they still retain a copy of the full Patient Information Sheet of if they need a further copy to be sent out in advance of the visit. This will be sent out to arrive at least 48 hours prior to the planned CRF

At the screening visit, eligibility criteria will be checked and the study procedures explained. All potential participants will have had at least 24 hours to review the full Patient Information Sheet. They will then be invited to provide written, informed consent.

At other sites within NHS Greater Glasgow and Clyde the same approach will be taken for the clinic lists of those physicians who indicate willingness to support trial recruitment. The initial approach will always be from a member of the usual care team.

Covid-19 Mitigation: The delivery of outpatient clinics has been substantially changed due to the pandemic. The use of telephone clinics is now standard for many visits and video technologies are also

being used. These mediums will therefore be used for inviting participants to take part when in person appointments are not taking place. This may be subject to change depending on the situation with Covid-19. Other principles of recruitment will be as described above.

7.1.2 Screening

A separate non-fasting visit will be arranged at which potential participants will have further time to discuss with the study nurse and doctor.

Prospective participants will be asked to read and understand the Participant Information Sheet. Any questions regarding the Protocol will be answered. Risks and side-effects of the active trial medication will be explained. Eligibility for trial enrolment will be confirmed by a medical practitioner.

Individual wishing to participate in the study will then be invited to sign to document their informed consent (see Section 7.2 below).

7.1.3 Screening assessments

Items from the medical history relevant to the Inclusion and Exclusion criteria will be recorded. Demographic data, medical history and current medications (including current insulin doses) will be documented. All participants will have access to a dual blood glucose and ketone meter as per the contemporary standard of care in type 1 diabetes. A physical examination will be conducted. Clinical observations will be recorded and blood and urine samples obtained (see below):

<u>Demographics</u>

- Date of birth
- Sex
- Ethnicity
- Date of diagnosis
- Ethnic group: white/black/Asian/other
- Height

<u>Clinical assessments</u>

- Systolic and diastolic blood pressure (after 5 minutes rest)
- Heart rate
- Body weight (clothed without coat and shoes)
- Capillary blood glucose

<u>Medical History</u>

- Smoking status: current/ex/never
- Alcohol consumption: units/week
- Hypertension: Y/N
- Microalbuminuria: Y/N
- Proteinuria: Y/N
- Neuropathy: Y/N
- Previous major cardiovascular event(s): Y/N (date)
- Existing CV disease of any type*: Y/N
- Amputation above forefoot: Y/N
- Hospitalisation for DKA in last year
- Level of educational attainment: University (college) educated Y/N

* Existing CV Disease is defined as any of: MI, stroke, ACS/unstable angina, coronarystent/angioplasty, CABG, TIA, heart failure, peripheral vascular disease

Insulin treatment
- Regimen: MDI, pump,
- Total dose (all); Basal (longer acting) dose/ Bolus (shorter acting) dose (for MDI only)

Other treatment (drug classes and names)
Current use Y/N. If no, has there been use in last three months?: Y/N

- ACE inhibitors
- Angiotensin receptor blocker
- Calcium channel blocker
- Alpha-blocker
- Beta-blocker
- Loop diuretic
- Thiazide like diuretic
- Statin
- Aspirin
- Clopidogrel
- Novel anticoagulant
- Warfarin

Blood assessments:
- Blood ketones
- HbA1c
- FBC
- Urea and electrolytes
- Creatinine
- Liver function tests (AST/ ALT/ Alkaline Phosphatase)
- Bone profile (Calcium/ Phosphate)
- C-peptide (capillary blood glucose must be > 4.0 mmol/L at time of sampling)
  N.B. where re-screening is undertaken (see 7.2.1 below), C-peptide does not require to be repeated

If blood tests have been assessed for clinical purposes within the last three weeks, these values will be used.

Urine sample:
- Urine ketones
- For all WOCBP, a urine pregnancy test will be checked at screening

Potential participants meeting all the inclusion and exclusion criteria based on history, examination and screening tests will be enrolled in the study.

Pregnancy: WOCBP will be asked about pregnancy status and contraceptive usage (see Appendix 2) and a pregnancy test will be conducted (following informed consent) prior to randomisation. We will not recruit those who are pregnant, wanting to become pregnant, or using birth control methods not considered highly effective. Every four weeks following randomisation, WOCBP randomised to IMP (as opposed to standard of care) will be asked about their contraception status, and a urine pregnancy test carried out. Study

medication will be permanently discontinued in women who become pregnant or considered to be on contraception that is not highly effective. As the study assessments *per se* do not carry an additional risk over standard of care to either mother or unborn child, further pregnancy tests will not be routinely offered following randomisation to those women allocated to “Insulin therapy (standard of care)” (i.e. who are not exposed to IMP). All pregnancies will be notified to the Sponsor Pharmacovigilance Officer using the standard pregnancy notification form and the pregnancy followed to outcome).

7.1.4 Payment

Participants will be offered reasonable travel expenses for visits additional to usual care.

7.2 Consent

Potential participants will be identified and screened by the Inclusion and Exclusion criteria listed above. Medical staff or appropriately trained support staff will seek consent for screening and participation in the trial from individuals that fulfil the criteria. All potential participants will have at least 24 hours to review the Patient Information Sheet before being approached (see Section 7.1.2 above). Following written consent, each signature will be dated by the signatory, the original retained in the site file, a copy provided to the participant and a copy scanned into the participant's Clinical Portal record.

Data collected for routine clinical care will be used for clinical trial documentation (e.g. blood results within the previous three weeks). In the absence of routine blood results, sampling of blood for study specific laboratory measurements can only be performed following documentation of informed consent.

7.2.1 Re-screening post-consent

Where inclusion (e.g. HbA1c ≤7.5%) or exclusion (e.g. DKA within the last three months) criteria are not met (e.g. screening visit blood results falling outside the study parameters), potential participants <u>can</u> be re-screened with additional urine and blood tests on one further occasion.

Where the screening period (i.e. between the Screening visit and Visit 1) exceeds 10 weeks, screening visit urine and blood tests <u>should</u> be repeated at an additional visit that can be arranged with verbal consent of the participant prior to Visit 1. Data will be amended on the web portal as appropriate.

If the initial consent was signed within three months of the re-testing, new consent is not required unless the consent form template has been updated in this period. If the initial consent was not signed within three months of re-testing then consent should be sought again.

Rescreened participants will keep the same Patient ID and data will be amended on the web portal as appropriate.

Where screening bloods are repeated in cases as above, C-peptide testing does not require to be repeated as values consistent with the inclusion criteria remain valid long term.

7.3 Run-in period

7.3.1 Visit 1 (@3 weeks prior to randomisation)

Participants who meet entry criteria will be scheduled for their next visit at approximately three weeks prior to randomisation. Daily fingerstick blood glucose levels (study diaries), CGM/ FGM data or insulin pump download data (as applicable) will be collected and reviewed and appropriate insulin dose adjustments

made by the medical team to target fasting blood glucose concentrations between 5 -10.0 mmol/L if possible at all times without having additional hypoglycaemia (< 3.9 mmol/L). Clinical observations will be recorded including resting BP measurement. Blood glucose and ketone bodies will be measured via a fingerstick blood sample (see Section 7). Urine for pregnancy status and ketone bodies will be assessed.

Participants will be instructed on the use of the Continuous Glucose Monitoring System (Dexcom G6 with blinded receiver). All subjects will be advised to monitor their capillary blood glucose by CGM or fingerstick at least 4 times a day (before meals and at bedtime) throughout the study and to wear their CGM constantly for next 20 days (changing the sensor at 10 days). CHO counting technique will be reviewed and appropriate support given with calculating bolus insulin doses.

<u>Visit procedures:</u>

<u>Monitoring and treatment optimisation</u>

- Check for adverse events
- Review blood glucose diary
- Blood ketones
- Adjust insulin doses (if necessary)

<u>Clinical assessments</u>

- Blood pressure
- Heart rate
- Weight

<u>Urine sample</u>:

- Check urine ketones

<u>Other</u>

- Fit and start masked Continuous Glucose Monitoring with Dexcom G6

7.3.2 Visit 2 (1 @ weeks prior to randomisation)

Fasting visit* - Participants will be asked to fast (no food or calorie intake) for 10 hours prior to the visit overnight. Those who plan to drive to fasting visits will be reminded of the increased risk of hypoglycaemia and DVLA guidelines. This will be repeated prior to each fasting visit and reiterated within the patient diaries. This visit will be scheduled for a morning appointment and participants will be advised not to take their breakfast bolus insulin dose prior to arrival. If participants arrive at the study visit in a non-fasted state, the visit will be terminated and a further fasting visit rearranged as soon as possible.

Daily fingerstick blood glucose levels (diaries), CGM/ FGM data or insulin pump download data (as applicable) will be collected and reviewed and appropriate insulin dose adjustments made. Clinical observations will be recorded as detailed below. Blood glucose and ketone bodies will be measured via a fingerstick blood sample (see Section 7). If blood glucose is less than 3.9 mmol/L, or more than 13.9 mmol/L, the visit will be rescheduled for an alternative date. Otherwise, an intravenous cannula will be placed in order to obtain blood samples for the duration of the visit. A Meal Challenge Test will be performed (described below). A 24 hr urine container will be dispensed to collect a 24 hour urine sample to bring in at the Week 0 visit. For this collection and all subsequent 24 hour urine collections, urine will be tested via dipstick testing and an aliquot retained for assay of albumin, creatinine, ketones, and 15-isoprostane.

Visit Procedures

Monitoring and treatment optimisation

- Check for adverse events
- Review blood glucose diary
- Blood ketones
- Adjust insulin doses (if necessary)

Clinical assessments

- Blood pressure
- Heart rate
- Weight

Urine sample:

- Check urine ketones
- 24 hour urine container dispensed

Other assessments

- Download Dexcom G6 CGM
- High fat high calorie Meal Challenge: in order to assess postprandial metabolism a Meal Challenge Test will be performed using an approximately 900 kilocalorie High Fat High Carbohydrate (HFHC) meal including an egg muffin sandwich, a sausage muffin sandwich and two hash browns [containing approximately 88g carbohydrate, 51 g fat (33% saturated) and 34 g protein]. Participants will be asked to attend fasting prior to breakfast insulin bolus (with continuing basal insulin); participants will be asked not to take bolus insulin until the 3 hour time point unless blood glucose values reach above 22.2 mmol/L. Blood samples will be collected from an indwelling intravenous canula in a superficial forearm vein for glucose, glucagon, FFA, GLP-1 (AUC). Sequential blood samples will be obtained at 0, 15, 30, 45, 60, 90, 120, 150 and 180 min after consuming the meal. Volume of samples at 15, 30, 45, 90 and 150 min will be 5ml while those at 0, 60, 120 and 180min will be 30 ml (total volume = 145 ml). A spot urine sample will be collected at baseline, 1 and 3hr for standard urinalysis and an aliquot retained for assay of ketones and 15-isoprostane.

7.4 Randomisation

Participants who are being randomised will be required to have undergone all screening assessments.

At the randomisation visit, study participants will be provided with a Patient Alert Card. There are two Versions: (1) for the intervention arm i.e. those allocated at first randomisation to open-label semaglutide who are also eligible for the second double-blind randomisation (dapagliflozin or placebo); and (2) for those allocated to standard of care. These cards supplement the study diaries and include contact information for the study team. Version 1 also contains unblinding information. Participants will be asked to carry their card at all times. These cards will be collected at the end of participation in the study.

7.4.3 First Randomisation Scheme

For the first randomisation (Period 1, open label), eligible and consenting participants will be randomised (2:1, stratified by site) to the two groups (open-label semaglutide or standard of care) using permuted blocks of variable size to minimize predictability.

7.4.4 Method of implementing the allocation sequence

Randomisation will be achieved by accessing an interactive web-based randomisation system (IWRS). The investigator will provide the participant identifier and the system will check the participant's eligibility from information already entered in the electronic Case Report Form (eCRF): if appropriate the randomisation group will be allocated.

7.5 Visit 3 (First Randomisation Visit)

Participants will be asked to attend in the fasting state (no food or calorie intake for 10 hours; see 7.3.2 above). Blood glucose levels and ketone bodies will be measured via a fingerstick blood sample using a glucose and ketone meter (see Section 7) and daily fingerstick blood glucose levels (diaries) collected and reviewed. The participant's own CGM/ FGM blood glucose data or insulin pump download data will also be collected and reviewed. Daily insulin dose will be adjusted following clinical review of study diaries: if reduced, this should not be by more than 20% from the baseline level Participants will discontinue use of the study masked CGM (Dexcom G6). Clinical observations will be recorded. Blood glucose and ketone bodies will be measured via a fingerstick blood sample (see Section 7). The 24 hour urine container will be collected. A fasting blood sample will be taken (45 mL) to evaluate study endpoints, FBC, U&E, LFT, bone profile, lipids, HbA1c, and fructosamine (detailed below). A urine sample will be collected to evaluate ketone bodies and, in females, pregnancy status.

Participants will be asked to complete Quality of Life questionnaires (see Appendices).

IMP will be dispensed for those randomised to open-label semaglutide (once weekly). Semaglutide will be started on site at the 0.25 mg dose. Participants will be instructed to call site staff directly in case of any problem or untoward side effects. An office hours phone number will be provided for advice. Outside these hours, provision of emergency medical care if required will be via NHS 24 and/or Accident & Emergency. Participants will be specifically asked to call if they have hypoglycaemia (blood glucose < 3.9 mmol/L) or Level 2 hyperglycaemia (blood glucose >13.9 mmol/L), particularly if occurring on more than one occasion. This will apply for the remainder of the trial.

Visit Procedures

Fasting visit* as per visit 2.

<u>Monitoring and treatment optimisation</u>

- Check for adverse events
- Check adequacy of contraception (WOCBP)
- Review blood glucose diary
- Adjust insulin doses (if necessary)
- Blood ketones

<u>Clinical assessments</u>

Blood pressure

- Heart rate
- Body weight

<u>Quality of life assessments</u>

- Diabetes Specific Quality of Life Scale (DSQOLS)
- Problem Areas In Diabetes (PAID) questionnaire

Blood assessments

- Na, K, urea, creatinine, eGFR (MDRD)
- AST/ ALT/ Alkaline Phosphatase
- Calcium/ Phosphate
- FBC
- HbA1c
- Fructosamine
- Haemoglobin
- Platelets
- Bilirubin
- Random glucose

Urine

- For WOCBP, a urine pregnancy test will be checked prior to first administration of IMP.
- Urine ketones
- 24 hour urine sample collected

Medication

- Administer open label semaglutide 0.25 mg if randomised to active treatment
- Issue Patient Alert Card (appropriate version according to randomisation)

Other:

- Read masked Continuous Glucose Monitoring with Dexcom G6

Bloods for storage

Approximately 45 mls of venous blood will be withdrawn and collected in tubes containing EDTA. Blood will be centrifuged at 1500g for 20 mins at 4ºC. Plasma will be siphoned, aliquoted and stored locally at -80° ± 10°C until transport in batches to the central laboratory on dry ice.

7.5.1 Visit 4 (@1 week after first randomisation)

Clinical observations will be recorded. Blood glucose levels and ketone bodies will be measured via a fingerstick blood sample using the participant's own glucose and ketone meter (see Section 7)) and daily blood glucose levels collected and reviewed. Insulin doses will be adjusted as in visit 3. If HbA1c is <7.0 %, insulin dose reduction should not be >5% from baseline total dose. Semaglutide 0.25mg will again be injected on site for those randomised to active therapy; however, if the participant is unable to attend within 48 hours of the scheduled appointment this dose can be self-administered at home (confirmed by a documented phone call) provided there were no adverse effects following the previous dose.

Visit Procedures

Monitoring and treatment optimisation

- Check for adverse events
- Review blood glucose diary
- Adjust insulin doses (if necessary)
- Blood ketones

Assessments

- Blood pressure

- Heart rate
- Weight

Urine sample:
- Urine ketones

Medication
- Support participant's first self-injection of open label semaglutide 0.25 mg if randomised to active treatment (dose on-site if dose is scheduled for visit day (see above)

7.5.2 Visit 5 (@2 weeks after first randomisation)

Clinical observations will be recorded. Blood glucose levels and ketone bodies will be measured via a fingerstick blood sample using glucose and ketones meter (see Section 7) and daily blood glucose levels collected and reviewed. Daily blood glucose data collected and reviewed and insulin doses adjusted as per Visit 3. If participants randomised to semaglutide are experiencing any nausea, they will continue on the 0.25 mg dose for another week (or more as required) before increasing the dose. Otherwise semaglutide 0.5mg dose will be injected on site. If dose escalation is not planned, dose can be self-administered at home (confirmed by a documented phone call) provided there were no adverse effects following the previous dose. Supply of IMP will be dispensed as appropriate to last until the next visit.

Visit Procedures

Monitoring and treatment optimisation
- Check for adverse events
- Review blood glucose diary
- Adjust insulin doses (if necessary)
- Blood ketones

Assessments
- Blood pressure
- Heart rate
- Weight

Urine sample:
- Urine ketones

Medication
- Dose titration (see above) if randomised to open-label semaglutide
- Return used study pen
- Dispense medication if randomised to open-label semaglutide (dose on-site if dose is scheduled for visit day– see above)

7.5.3 Visit 6 (@4 weeks after first randomisation)

For those randomised to open-label semaglutide, doses administered will be recorded (self-report). Clinical observations will be recorded. Daily blood glucose data will be collected and reviewed and insulin doses adjusted as per visit 3. Supply of IMP will be given to last until the next visit. A urine sample will be collected to evaluate ketone bodies and, in females randomised to IMP, pregnancy status. Unless experiencing nausea or other adverse events, the recommended dose will be increased to 1.0 mg

weekly (or 0.5 mg if not previously uptitrated from 0.25 mg). A 24 hr urine container will be dispensed to collect a 24 hour urine sample to bring in at the Week 13 visit.

Visit Procedures

Monitoring and treatment optimisation
- Check for adverse events
- Check adequacy of contraception (WOCBP)
- Review blood glucose diary
- Adjust insulin doses (if necessary)
- Blood ketones

Assessments
- Blood pressure
- Heart rate
- Weight

Urine sample:
- Urine ketones
- Urine pregnancy test (WOCBP randomised to IMP only)
- 24 hour urine container dispensed

Medication
- Record doses of semaglutide self-injected since last visit (self-report)
- Dose titration if randomised to open-label semaglutide (see above)
- Return used study pens
- Dispense medication if randomised to open-label semaglutide

7.5.4 Visit 7 (@13 weeks after first randomisation)

Participants will be asked to attend in the fasting state (no food or calorie intake for 10 hours). If participant is not fasting, samples should be taken and non-fasting status noted. For those randomised to open-label semaglutide, doses administered will be recorded (self-report). For those who have remained on doses lower than 1.0 mg weekly, this visit will be the last opportunity to uptitrate if not experiencing nausea or other adverse events (from 0.25 mg to 0.5 mg weekly - or from 0.5 mg to 1.0 mg weekly). Daily fingerstick blood glucose levels (diaries), CGM/ FGM data or insulin pump download data will be collected and reviewed and appropriate insulin dose adjustments made. Supply of IMP will be given to last until visit 10. Observations will be recorded. A urine sample will be collected to evaluate ketone bodies and, in females randomised to IMP, pregnancy status. The 24 hour urine container will be collected. A fasting blood sample will be taken (45 mL) to evaluate study endpoints.

Visit Procedures

Fasting visit*

Monitoring and treatment optimisation
- Check adequacy of contraception (WOCBP)
- Check for adverse events
- Review blood glucose diary

- Adjust insulin doses (if necessary)
- Blood ketones

Clinical assessments
- Blood pressure
- Heart rate
- Weight

Blood assessments
- Na, K, urea, creatinine, eGFR (MDRD)
- AST/ ALT/ Alkaline Phosphatase
- Calcium/ Phosphate
- FBC
- HbA1c
- Fructosamine
- Haemoglobin
- White cell count
- Platelets
- Bilirubin
- Random glucose

Urine
- Pregnancy test (WOCBP randomised to IMP only)
- Urine ketones
- 24 hour urine bottle collected

Medication

- Record doses of semaglutide self-injected since last visit (self-report)
- Dose titration if randomised to open-label semaglutide (see above)
- **N.B. no further uptitration after this visit**
- Return used study pens
- Dispense medication if randomised to open-label semaglutide

7.5.5 Visit 8 (@23 weeks after first randomisation)

For those randomised to open-label semaglutide, doses administered will be recorded (self-report). Clinical observations will be recorded. Daily blood glucose data (fingerstick or FGM) will be collected and reviewed as per visit 3. Blood glucose levels and ketone bodies will be measured via a fingerstick blood sample. A urine sample will be collected to evaluate ketone bodies and, in females randomised to IMP, pregnancy status. Insulin doses will be adjusted if needed. All subjects will be reminded to monitor their capillary blood glucose by fingerstick at least 4 times a day (before meals and at bedtime) throughout the study. CGM sensor will be fitted and participants asked to wear their CGM constantly for next 20 days.

Visit Procedures

Monitoring and treatment optimisation
- Check adequacy of contraception (WOCBP)
- Check for adverse events
- Review blood glucose diary

- Blood ketones
- Adjust insulin doses (if necessary)

Clinical assessments
- Blood pressure
- Heart rate
- Weight

Urine sample:
- Pregnancy test (WOCBP randomised to IMP only)
- Urine ketones

Other assessments
- Fit and start masked Continuous Glucose Monitoring with Dexcom G6

7.5.6 Visit 9 (@25 weeks after first randomisation)

Participants will be asked to attend in the fasting state (no food or calorie intake for 10 hours). If participants arrive at the study visit in a non-fasted state, the visit will be terminated and a further fasting visit rearranged as soon as possible. Daily fingerstick blood glucose levels (diaries), CGM/ FGM data or insulin pump download data will be collected and reviewed and appropriate insulin dose adjustments made. Participants will discontinue use of the study CGM (Dexcom G6). Clinical observations will be recorded. Blood glucose levels and ketone bodies will be measured via a fingerstick blood sample (see Section 7). Supply of IMP will be given to last until the next visit. A 24 hr urine container will be dispensed to collect a 24 hour urine sample to bring in at visit 10 (Week 26) visit. A Meal Challenge Test will be performed as per visit 2. Participants will be provided with strips for urine and blood ketone measurement at home and instructed or reminded as appropriate on how to use these. They will be asked to measure urinary and blood ketones every morning for three consecutive days prior to the next visit (second randomisation: dapagliflozin or placebo) and to record the results in their diary.

Visit Procedures

Fasting visit*

Monitoring and treatment optimisation
- Check adequacy of contraception (WOCBP)
- Check for adverse events
- Review blood glucose diary
- Adjust insulin doses (if necessary)
- Blood ketones

Clinical assessments
- Blood pressure
- Heart rate
- Weight

Urine sample:
- Urine ketones
- 24 hour urine container dispensed

Other assessments

- Download Dexcom G6
- Meal Challenge Test

Blood assessments

- HbA1c

7.6 Second Randomisation Scheme

For the second randomisation (Period 2, double-blind), eligible and consenting participants previously randomized to open-label semaglutide will be randomised (1:1) to two groups (dapagliflozin or matching placebo) using permuted blocks of variable size to minimize predictability with stratification for HbA1c (≤ 7.5% vs >7.5% HbA1c) Those participants previously randomised to standard of care will continue as before.

7.6.1 Blinding (period 2 only)

For the double-blind treatment period, dapagliflozin and placebo will be packed in matching packs to maintain the blind. Each pack will contain sufficient supplies for 30 days' treatment with a small overage (excess). Packs will be labelled with a unique number that will be used to assign treatment to the participant via the IWRS system whilst maintaining the blind. Packs will be labelled in accordance with Good Manufacturing Practice and local regulatory requirements.

Unblinding. Treatment discontinuation (of dapagliflozin or placebo), rather than unblinding, will be carried out as far as possible. Participants will be advised in any case of hospitalisation with acute illness to discontinue study medication and inform the relevant clinician. These instructions will be reinforced in the Study Diary. However, where knowledge of treatment may assist emergency treatment, the investigator (or designee) may unblind using the IWRS system. Information on emergency unblinding (the investigational study drug, the study number, the investigator's name and a contact number) will be provided on the Patient Alert Card. For each unblinding an email alert is generated to the Study Coordinator and Chief Investigator. The most likely scenarios for unblinding will be: confirmed pregnancy, overdose/ accidental ingestion, or development of acute renal failure

7.6.2 Visit10 (Second Randomisation Visit; @26 weeks after First Randomisation)

will be asked to attend in the fasting state (no food or calorie intake for 10 hours). If participants arrive at the study visit in a non-fasted state, the visit will be terminated and a further fasting visit rearranged as soon as possible. Participants with normal fasting urine (<+) or blood ketone (<0.6 mmol) in the three days prior to randomisation will proceed to the next treatment phase as per this visit. The principles of the STICH Protocol (see Appendix 3) will be discussed (37). Participants with higher ketone values in at least one of the previous three days will have their glucose levels, insulin dose, and carbohydrate intake reviewed and advised on hydration, adequate CHO intake, and adequate insulin dosing. They will be rescheduled for one week and asked to repeat three days testing before the next visit.

Clinical observations will be recorded. A urine sample will be collected for ketones. Blood glucose and ketones will be measured via a fingerstick blood sample (see Section 7.2.1). Daily fingerstick blood glucose levels (diaries), CGM/ FGM data or insulin pump download data will be collected and reviewed and appropriate insulin dose adjustments made (following the guidance in Section 7, page 35). The previous three days blood or urine ketones levels will be reviewed. The adjusted total insulin dose at

this visit will serve as the baseline total insulin dose for the remaining six months of the study and future limits of dose adjustments will be based on this new baseline. The blinded study CGM will be collected. The 24 hour urine container will be collected. A fasting blood sample will be taken (45 mL) to evaluate study endpoints. Participants will be asked to complete quality of life questionnaires.

At this point, those participants previously randomised to receive open-label semaglutide will be randomised to receive dapagliflozin 5 mg daily or matching placebo. They will be educated on symptoms suggestive of low blood pressure (e.g. light-headedness, dizziness, feeling faint).

Participants will be asked to perform a daily (preferably early morning) urine (using CombiScreen® 10 SYS Plus urine testing strips or equivalent) or blood tests for ketone bodies (using the particpant's own meters as per Section 7, "Minimising ketosis": page 28) and record all outcomes in dedicated study diaries. If results of these scheduled (i.e. not done due to symptoms) ketone measurements, are +1 or higher (urine) or > 0.6 mmol/L (blood), participants will be instructed to follow the STICH Protocol as per their study diary and Appendix 3, . For any abnormal ketone levels taken due to symptoms, the DKA consensus guidelines are to be followed instead (see Section 9.3).

Participants will be instructed to call site staff directly in case of any problem or untoward side effects. They will be specifically asked to call if they have confirmed hypoglycaemia (blood glucose levels < 3.9 mmol/L) or hyperglycaemia (blood glucose > 13.9 mmol/L) occurring on more than one occasion. Participants will be advised to remain well hydrated and report any genital mycotic or urinary tract infections

Visit Procedures

Fasting visit* - Requirements and arrangements as per visits 2 and 3.

Monitoring and treatment optimisation

- Check adequacy of contraception (WOCBP)
- Check for adverse events
- Review blood glucose diary
- Adjust insulin doses (if necessary) - this insulin dose is baseline for Period 2
- Blood ketones

Clinical assessments
- Blood pressure
- Heart rate
- Body weight

Quality of life assessments
- Diabetes Specific Quality of Life Scale (DSQOLS)
- Problem Areas In Diabetes (PAID) questionnaire

Blood assessments
- Na, K, urea, creatinine, eGFR (MDRD)
- AST/ ALT/ Alkaline Phosphatase
- Calcium/ Phosphate
- FBC
- Fructosamine

- Haemoglobin
- White cell count
- Platelets
- Bilirubin
- Random glucose
- C-peptide (capillary blood glucose must be > 4.0 mmol/L at time of sampling)
-

Urine

- Pregnancy test (WOCBP randomised to IMP only)
- Urine ketones
- 24 hour urine bottle collected

Medication

- Education on symptoms suggestive of low blood pressure (e.g. light-headedness, dizziness, feeling faint) for those undergoing second randomisation
- Record doses of semaglutide self-injected since Visit 7 (self-report)
- Return used study pens
- Dispense open label semaglutide for participants randomised to active treatment at Period 1 (if required). Dispense dapagliflozin or placebo if randomised to active treatment

Other assessments

- Read masked Continuous Glucose Monitoring sensor (mean fasting blood glucose, mean blood glucose, percentage time in range, glycaemic variability)
-

7.6.3 Visit 11 (@1 week after Second Randomisation)

For those randomised to open-label semaglutide, doses administered will be recorded (self-report). Clinical observations will be recorded. Daily blood glucose and blood/ urine ketone levels will be reviewed and glucose levels collected. A urine sample will be collected to evaluate for ketones bodies. Blood glucose levels and ketones bodies will be reviewed measured via a fingerstick blood sample (see Section 7.2.1). . Daily fingerstick blood glucose levels (diaries), CGM/ FGM data or insulin pump download data will be reviewed and appropriate insulin dose adjustments made using the new baseline from Visit 10 (following the guidance in Section 7, page 35). If at any time total insulin dose reduction is >20% and total insulin dose is <0.40 U/Kg, additional monitoring (see Appendix 2) will be implemented. Unless blood (>0.6 mmol/L) or urine ketones (+ or more) have been elevated within the previous three days, dapagliflozin or placebo dose will be uptitrated to 10 mg daily (two capsules of either active or placebo medication) for the remainder of the study. Supply of IMPs will be given to last until week 39.

Visit Procedures

Monitoring and treatment optimisation

- Check adequacy of contraception.
- Check for adverse events
- Review blood glucose diary
- Adjust insulin doses (if necessary)
- Blood ketones
- Daily blood or urine ketones review

Clinical assessments

- Blood pressure
- Heart rate
- Body weight

Urine sample:
- Urine ketones

Medication
- Count returned dapagliflozin or placebo capsules
- Increase dose of dapagliflozin or placebo to 10mg (see above – review ketones)
- Dispense dapagliflozin or placebo (record dose)

7.6.4 Visit 12 (@5 weeks after Second Randomisation)

For those randomised to open-label semaglutide, doses administered will be recorded (self-report). Clinical observations will be recorded. Daily blood glucose and blood/ urine ketone levels will be reviewed and glucose levels collected. Blood glucose levels and ketone bodies will be measured via a fingerstick blood sample. A urine sample will be collected to evaluate ketone bodies and if appropriate pregnancy status. Insulin dose will be adjusted if as per Visit 11. A blood sample will be taken (15 mL) to evaluate study endpoints. A 24 hr urine container will be dispensed to collect a 24 hour urine sample to bring in at the Week 39 visit

Visit Procedures

Monitoring and treatment optimisation
- Check adequacy of contraception (WOCBP)
- Check for adverse events
- Review blood glucose diary
- Adjust insulin doses (if necessary)
- Blood ketones

Clinical assessments
- Blood pressure
- Heart rate
- Weight

Blood assessments
- Na, K, urea, creatinine, eGFR (MDRD)
- AST/ ALT/ Alkaline Phosphatase
- Calcium/ Phosphate
- FBC
- Haemoglobin
- White cell count
- Platelets
- Bilirubin
- Random glucose

Urine

- Pregnancy test (WOCBP randomised to IMP only)
- Urine ketones
- 24 hour urine container dispensed

7.6.5 Visit 13 (@13 weeks after Second Randomisation)

For those randomised to open-label semaglutide, doses administered will be recorded (self-report). Participants will be asked to attend in the fasting state. If participant is not fasting, samples should be taken and non-fasting status noted. Clinical observations will be recorded. Daily blood glucose and blood/ urine ketone levels will be reviewed and glucose levels collected. Blood glucose levels and ketone bodies will be measured via a fingerstick blood sample. A urine sample will be collected to evaluate ketone bodies and if appropriate pregnancy status. The 24 hour urine container will be collected. Insulin dose will be adjusted if needed as in as per Visit 11. Sufficient supply of IMPs will be given until the end of the study. A fasting blood sample will be taken (45 mL) to evaluate study endpoints.

Visit Procedures

Fasting visit* - as per previous fasting visits

Monitoring and treatment optimisation

- Check adequacy of contraception (WOCBP)
- Check for adverse events
- Review blood glucose diary
- Adjust insulin doses (if necessary)
- Blood ketones
- Daily urine ketones review

Clinical assessments

- Blood pressure
- Heart rate
- Weight

Quality of life assessments

- Diabetes Specific Quality of Life Scale (DSQOLS)
- Problem Areas In Diabetes (PAID) questionnaire

Blood assessments

- Na, K, urea, creatinine, eGFR (MDRD)
- AST/ ALT/ Alkaline Phosphatase
- Calcium/ Phosphate
- FBC
- HbA1c
- Fructosamine
- Haemoglobin
- Platelets

- Bilirubin
- Random glucose

Urine
- Pregnancy test (WOCBP randomised to IMP only)
- Urine ketones
- 24 hour urine bottle collected

Medication
- Record doses of semaglutide administered since Visit 10 (self-report)
- Dispense open label semaglutide for participants randomised to active treatment at Period 1 (if required).
- Count returned dapagliflozin or placebo capsules
- Dispense dapagliflozin or placebo (record dose)

7.6.6 Visit 14 (@23 weeks after Second Randomisation)

For those randomised to open-label semaglutide, doses administered will be recorded (self-report). Clinical observations will be recorded. Daily blood glucose and blood/ urine ketone levels will be reviewed and glucose levels collected. Blood glucose levels and ketone bodies will be measured via a fingerstick blood sample. A urine sample will be collected to evaluate ketone bodies and if appropriate pregnancy status. Insulin dose will be adjusted if as per Visit 11. All subjects will be reminded to monitor their capillary blood glucose by fingerstick at least four times a day (before meals and at bedtime). CGM (Dexcom G6) sensor will be fitted and patients asked to wear their CGM constantly for the next 20 days.

Visit Procedures

Monitoring and treatment optimisation
- Check adequacy of contraception (WOCBP)
- Check for adverse events
- Review blood glucose diary
- Adjust insulin doses (if necessary)
- Blood ketones
- Daily urine ketones review

Clinical assessments
- Blood pressure
- Heart rate
- Weight

Urine sample:
- Pregnancy test (WOCBP randomised to IMP only)
- Urine ketones

Clinical and functional assessment
- *Fit and start* masked Continuous Glucose Monitoring sensor (mean fasting blood glucose, mean blood glucose, percentage time in range, glycaemic variability)

7.6.7 Visit 15 (@25 weeks after Second Randomisation)

Participants will be asked to attend in the fasting state. If participants arrive at the study visit in a non-fasted state, the visit will be terminated and a further fasting visit rearranged as soon as possible. For those randomised to open-label semaglutide, doses administered will be recorded (self-report). Observations will be taken. Daily blood glucose and blood/ urine ketone levels will be reviewed and glucose levels collected. Blood glucose levels (fingerstick or FGM) and ketone bodies will be measured via a fingerstick blood sample. A urine sample will be collected to evaluate urine ketone levels. Insulin dose will be adjusted if needed as per visit 11. A 24 hr urine container will be dispensed to collect a 24 hour urine sample to bring in at the Week 52 visit. A HFHC Meal Challenge Test will be performed as in Visit 2.

Visit Procedures

Fasting visit* - Procedure as per previous fasting visits

Monitoring and treatment optimisation

- Check adequacy of contraception (WOCBP)
- Check for adverse events
- Review blood glucose diary
- Adjust insulin doses (if necessary)
- Daily urine ketones review
- Blood ketones

Assessments

- Blood pressure
- Heart rate
- Weight

Urine sample:

- Pregnancy test (WOCBP randomised to IMP only)
- 24 hour urine container dispensed

Other assessments

- Meal Challenge Test

7.6.8 Visit 16 (End of study; @26 weeks after Second Randomisation)

Participants will be asked to attend in the fasting state. If participants arrive at the study visit in a non-fasted state, the visit will be terminated and a further fasting visit rearranged as soon as possible. Clinical observations will be recorded. Daily blood glucose and blood/ urine ketone levels will be reviewed and glucose levels collected. Blood glucose levels and ketone bodies will be measured via a fingerstick blood sample. Insulin doses will be reviewed and likely requirement for an increase following discontinuation of IMP discussed with those taking open-label semaglutide. A urine sample will be collected to evaluate ketone bodies and, if appropriate pregnancy status. The CGM will be collected. The 24 hour urine container will be collected. A fasting blood sample will be taken (45 mL) to evaluate

study endpoints. We will ask the participants to complete quality of life questionnaires. A physical examination will be performed as per the screening visit. This will be the final face-to-face study visit. A safety phone call will be conducted two weeks to review blood glucose data, provide further insulin dosing advice, to document any adverse events, and to ensure that follow-up in usual clinical care has been arranged.

Visit Procedures

Fasting visit*

Monitoring and treatment optimisation

- Check for adverse events
- Review blood glucose diary
- Adjust insulin doses (if necessary)
- Blood ketones
- Daily urine ketones review

Clinical assessments

- Blood pressure
- Weight
- Heart rate
- Physical examination

Quality of life assessments

- Diabetes Specific Quality of Life Scale (DSQOLS)
- Problem Areas In Diabetes (PAID) questionnaire

Blood assessments

- Na, K, urea, creatinine, eGFR (MDRD)
- AST/ ALT/ Alkaline Phosphatase
- Calcium/ Phosphate
- FBC
- HbA1c
- Fructosamine
- Haemoglobin
- Platelets
- Bilirubin
- Random glucose
- C-peptide

Urine

- Pregnancy test (WOCBP randomised to IMP only)
- Urine ketones
- 24 hour collection

Other

- Record doses of semaglutide administered (self-report)

- Return used study pens
- Count returned dapagliflozin or placebo capsules
- Collect Patient Alert Card
- Read masked Continuous Glucose Monitoring sensor (mean fasting blood glucose, mean blood glucose, percentage time in range, glycaemic variability)

7.7 Retention and strategies for maximizing follow-up

Participants will be encouraged to attend all study visits. Prior to commencing the trial, all potential participants will have the opportunity to read the detailed Patient Information Sheet which will outline the level of participation requested. Potential participants will be made aware of the need for frequent study visits and asked to commit to these.

Study visits can be rearranged flexibly within a period around the planned visit as specified in the timeline of visits above (Section 7) and actual dates recorded in the eCRF. This is to maximise participant ability to attend all visits. A longer or shorter interval does not constitute a protocol deviation. Where possible, visits will be arranged to coincide with other appointments to further maximise attendance and participant convenience. Telephone contact will be frequent and will help to maintain contact with participants and ensure timely reminders of upcoming study visits can be given.

Even in the scenario of treatment discontinuation (of either IMP), participants will be encouraged to continue to attend further study visits as scheduled.

7.8 Treatment Interruptions and Withdrawal criteria

Withdrawal from study drug

Treatment interruption from study IMPs may be necessary in some participants. In the case of dapagliflozin, this will occur in any acute illness or hospitalisation which increases the risk of ketosis and ketoacidosis. Circumstances in which temporary discontinuation of dapagliflozin or placebo should occur will be discussed with all study participants. Written information will be provided in the study diaries. An emergency phone line to the study team will be available to all participants for the duration of their participation in the trial. This will be operational during office hours (Monday to Friday 9am – 5pm). The Patient Alert Card also contains contact details for unblinding in the event of acute intercurrent illness and a requirement for unblinding in the opinion of the acute medical team treating the participant. If any participant develops diabetic ketoacidosis (DKA) as defined as per the International Consensus guidelines on two occasions with no identifiable precipitant (section 2.1) they will be withdrawn from study medication and the UK Chief and US Principal Investigators informed.

Any other intolerable side effects or acute illness may require temporary discontinuation of the study drugs (dapagliflozin or semaglutide). In these circumstances, if the study drugs cannot be safely restarted in the judgement of the trial physician or if participants are unable to tolerate side-effects, they may be withdrawn from study treatment.

Treatment withdrawal may be necessary during the trial prior to the planned end at 52 weeks following first randomisation for any of the following reasons:

1. The site PI can stop investigational study treatment or remove participants from the trial without participants approval if for any reason he/she feels is appropriate, including: severe side effects, injury or medical condition which may place the patient at risk of further complications if he/she continue to participate, failure to take the medication as instructed, failure to keep scheduled appointments, insufficient level of self-monitoring, cancellation of the study or other administrative reasons.

2. Any woman who becomes pregnant during the period of study will be withdrawn from study treatment.

3. Participants who experience persistent severe nausea or vomiting after starting semaglutide which is not managed by dose reduction will discontinue the investigational study treatment. Those with mild or moderate nausea will continue for at least one more week. For those who continue to experience significant nausea (defined as interfering with normal activities), dose will be reduced: if these side effects persist, they will discontinue study medication(s). Participants for whom this occurs in Period 1 will remain eligible for randomisation in Period 2.

4. Excessive weight loss during either period of the study despite treatment dose reduction (> 15% body weight or BMI < 22 kg/m2 on repeated measurements more than four weeks apart).

5. New diagnosis of other conditions included in the Exclusion Criteria

6. Change in method of insulin delivery between insulin pumps to MDI or vice versa during the study unless pump treatment interruption is temporary for a period of at most 2 weeks. Participants who upgrade to "artificial pancreas" (e.g. Medtronic's MiniMed 670G hybrid) during the study will be withdrawn.

7. Hypoglycaemia: Two episodes of severe hypoglycaemia (requiring external intervention).

8. Ketosis (with no other identifiable precipitating factors): Persistent (1 week) asymptomatic or symptomatic and unmanaged (using STICH protocol – see section 2.1 b) blood ketone levels >1.5 mmol (or ≥+2 in urine). (37)

9. Persistent (>1 week) total insulin dose reduction of >20% from baseline AND total insulin dose <0.4U/Kg.

10. DKA: Two episode of DKA (with no identifiable precipitating factors) as defined by the expert consensus statement (34)

11. Two episodes of UTI.

12. Two episodes of mycotic genital infection.

13. Significant persistent reduction in eGFR > 15 ml/min

14. Pancreatitis: any episode judged to be due to pancreatitis by the site PI.

15. Other persistent side effects specific to study drugs

Participation in this study is voluntary. Participants have the right to refuse to participate or to withdraw from participation at any time for any reason. The site PI can stop investigational study treatment or remove participants from the trial without participant's approval. If a patient withdraws consent and decides not to participate in the study, she/he will be asked to undergo a final study visit for his/her safety. If a subject withdraws from the research, the data collected to that point will be used toward the research finding. If applicable the subject will be asked to return any unused research drug and or devices.

Withdrawal from the study

If participants are unable or unwilling to attend all study visits they will be given an option of attending less frequently or only at the end of the study. We will request participants' consent to be contacted by telephone and for contact with their general practitioner or other health care provider to check on their current health status. No participant will be labelled as lost to follow-up. However, participants will have the right to withdraw consent for further participation and for further data collection: all other participants will be followed up for clinical events until study completion.

7.9 Storage and analysis of samples

Blood samples will be undertaken at the beginning and at various points during the study as outlined in detail in the schedule of visits. If blood testing has recently been undertaken in the 3 weeks prior to the study commencing, some of these blood tests may be used as baseline. Blood tests will include analysis of the full blood count, renal function (urea and electrolytes), liver function tests, bone profile, lipid profile and several specialised blood tests for biomarker assays (at central laboratory) following a high fat high caloric meal challenges to examine study endpoints.

Covid-19 Mitigation

All blood sampling or trial procedures will be undertaken following local and national guidance around personal protective equipment. Mask, gloves and aprons will be used as per current guidelines.

7.9.1 Sample collection and processing

- Samples will be appropriately labelled in accordance with the trial procedures to comply with the General Data Protection Regulation (GDPR). Biological samples collected from participants as part of this trial will be transported, stored, accessed and processed in accordance with national legislation relating to the use and storage of human tissue for research purposes and such activities shall at least meet the requirements as set out in the 2004 Human Tissue Act and the 2006 Human Tissue (Scotland) Act.
- Blood will be taken according to the Schedule of Assessments (page 17). Plasma will be siphoned, aliquoted and stored locally at -80° ± 10°C until transport in batches to the central laboratory on dry ice. according to the Sample Handling Procedure as agreed by the Joint Study Management Group
- Individual patient samples will be identified with a unique, anonymised study number.

7.9.2 Sample transport to central laboratory and analysis

- Samples will be transferred to State University of New York in a single batch at the end of recruitment to the study. More frequent transfer can be organised if there are local storage limitations.
- Samples will be transported on dry ice and stored at the central laboratory at -80° ± 10° until analysis. Transport by courier will coordinated by the Trial Manager.
- At the time of analysis individual aliquots will be defrosted at room temperature and analysed in a single batch for each biomarker of interest.
- Samples will be stored at -80° ± 10° in the central laboratory for possible future analysis for novel biomarkers.

7.10 End of trial

The end of the trial will be following the last study visit of the last subject.
At the end of the specified trial period (52 weeks following first randomisation) all participants will discontinue study medications and revert to standard of care (insulin therapy only). This process will be supervised by the trial physicians, research nurses, and research pharmacists under the supervision of the site Principal Investigator. The trial team will liaise with the usual care team to ensure a safe transition back to standard therapy. Following the final scheduled trial visit there will be a safety phone call two weeks after the last visit. This will act as a further opportunity to advise on insulin dosing and ensure there are no ongoing problems or concerns related to the study.

As discussed in section 7.8, there may be circumstances in which trial drugs need to be stopped before the specified end of the trial. If this is the case, the reason(s) for discontinuation will be recorded and the trial clinical team will facilitate safe transition back to usual standard of care. Participants will however if eligible (i.e non-exclusion criteria reason for IMP discontinuation) be asked to continue with trial visits to enable ongoing close clinical observation. If consent is withdrawn and participants voluntarily chose to leave the trial before the specified end date, all efforts by the clinical trial team will be made to safely facilitate transition back to ordinary standard of care. A follow up safety visit will be offered to all such participants and ongoing contact details will be made available should there be any further unforeseen issues relating to the clinical trial.

The trial may be stopped at an earlier stage than anticipated on the advice of the independent data monitoring committee (IDMC) to the trial steering committee (TSC) for the safety reasons detailed on page 12 and in the study safety endpoints. If this occurs, all participants will be informed and safe discontinuation of study medications will be undertaken under the supervision of the site Principal Investigator.

8 TRIAL MEDICATION

8.1 Name and description of investigational medicinal product(s)

a) <u>Semaglutide (Ozempic®)</u>: a once weekly GLP-1 agonist injection indicated for type 2 diabetes (approved by the Scottish Medicines Consortium, January 2019).

   Doses to be used: 0.25mg increased to 0.5mg at two weeks and then to 1mg weekly s.c. injection after a further two weeks. Open label semaglutide will be added to a background of insulin treatment at week 0 for 12 months in 76 participants (38 in the UK) according to the

first randomisation; at week 26 they will be further randomised (double-blind) as below into two groups: dual (placebo, semaglutide and insulin) and triple (dapagliflozin, semaglutide and insulin) therapy.

b) Dapagliflozin (as dapagliflozin propanediol monohydrate Forxiga®): a once daily SGLT-2 inhibitor that will be administered orally in a blinded fashion.

Doses to be used: 5 mg daily increased after one week to 10 mg daily (orally). Dapagliflozin will be started according to the randomisation (Interactive Web System, hosted by University of Glasgow) in 38 participants (19 in the UK) at 26 weeks as an additional treatment on a background of insulin and semaglutide therapy and end at 52 weeks (triple therapy group). Doses to be used: 5 mg increased to 10 mg after one week.

c) Placebo to dapagliflozin: Dapagliflozin matched placebo will be started in 38 participants (19 in the UK) participants at 26 weeks as an additional treatment on a background of insulin and semaglutide therapy and end at 52 weeks (dual therapy group). Doses to be used: as for active dapagliflozin.

8.2 Legal status of Semaglutide and Dapagliflozin

Semaglutide is currently licensed in the UK for those with adult type 2 diabetes which is insufficiently controlled.

8.3 Drug storage and supply

Semaglutide and dapagliflozin/placebo supplies must be stored in the original containers in a locked, secure area with access limited to authorised site staff. Study supplies should be used as directed in the study protocol and not be supplied to any persons other than study participants. All IMPs will be dispensed to participants from the study site pharmacy.

Covid-19 Mitigation

As detailed above in section 7, the supply of IMP may be altered to reduce risk of Covid-19 transmission in the event of future waves of infection. This may involve providing increased quantities of IMP to participants to last longer and therefore minimise physical contact with trial personnel. Use of a courier delivery service to facilitate this may also be considered.

Semaglutide: Supplies of semaglutide pre-filled pens for subcutaneous administration will be sourced from routine hospital stocks. The following semaglutide presentations will be required:

- Semaglutide 0.25 mg: Each pre-filled pen contains 2mg semaglutide in 1.5ml. Each dose contains 0.25mg of semaglutide in 0.19ml solution.
- Semaglutide 0.5 mg: Each pre-filled pen contains 2mg semaglutide in 1.5ml solution. Each dose contains 0.5 mg of semaglutide in 0.37ml solution.
- Semaglutide 1 mg: Each pre-filled pen contains 4 mg semaglutide in 3.0ml solution. Each dose contains 1mg semaglutide in 0.74ml solution.

For all presentations each pre-filled pen is capable of delivering 4 doses which is sufficient for 4 weeks treatment. Pens are for individual participant use only. In the event of a pen-failure, a new pen should be supplied to the participant.

Prior to first use, semaglutide pens must be stored at 2-8 °C in the original container and must not be frozen. Supplies which are frozen either prior to first use or during use must be discard.

Semaglutide pre-filled pens have an in-use shelf life of 6 weeks. After first use the pre-filled pens may be stored below 30oC or in a refrigerator at 2-8 °C. Participants should be advised to store packs away from the cooling element. The pen cap should be on when the pen is not in use in order to protect the contents from light.

Wherever possible, supplies of semaglutide pre-filled pens should be utilised in a pragmatic way so as to minimise unnecessary wastage. Participants should be provided with an appropriate number of pen(s) to last until the next planned visit. A small overage may be given as necessary to accommodate visit windows etc. The correct strength of pen must be supplied for each dose titration i.e. 0.25mg, 0.5mg and 1.0 mg as tolerated.

A web-based system will be used to randomise participants to semaglutide treatment. At randomisation and all subsequent visits (planned or unplanned), the system will notify pharmacy staff at study visits of the requirement for semaglutide supplies.

Dapagliflozin 5mg or placebo capsules: Supplies of dapagliflozin 5mg and matched placebo capsules will be manufactured by NHS Scotland Pharmaceuticals Specials Service/NHS Tayside Pharmaceuticals in accordance with Good Manufacturing Practice requirements. Capsules will be packed into HDPE bottles with a tamper evident seal. Each bottle will be labelled in a double-blind fashion to meet local regulatory requirements and will be assigned a unique number. Bottles containing dapagliflozin 5 mg or placebo must be stored at room temperature at 15-25°C.

Dapagliflozin 5mg and placebo capsule supplies will be provided to study site and will only be released by the sponsor once all the appropriate regulatory and governance approvals are in place. Further information on storage requirements and supply arrangements is provided in the study specific IMP Management and Accountability Manual.

A web-based system will be used for the assignment of dapagliflozin 5mg or placebo bottles at randomisation and at study visits where there is planned dispensing of the IMP or in the event that a participant requires replacement medicines.

Other medicines: Supplies of all other medicines and associated consumables will via usual NHS supply mechanisms.

8.4 Drug accountability requirements

The Investigator or designee must maintain accurate records of all study IMP movements for accountability purposes. They should include dates, quantities, batch numbers and expiry. Records must document adequately that:

- the patients were provided the doses specified by the protocol/amendment(s)
- all study drug provided was fully reconciled.

Unused study drug must not be discarded or used for any purpose other than the present study. Further information is provided in the study specific IMP Management and Accountability Manual.

8.5 Preparation and administration of semaglutide and dapagliflozin

Semaglutide (Ozempic®) is a solution for subcutaneous injection. It is administered once weekly, on the same day each week, into the subcutaneous tissues of the abdomen, thigh or upper arm. A participant may change the day of their weekly injection as long as it has been at least three days since their last injection. Thereafter, all doses should be administered once weekly on the new dosing day. The injection can be administered at any time of the day regardless of meals.

Participants must be advised to discard the injection needle after each injection and store the pen without an injection needle attached. This may help prevent blocked needles, contamination, infection, leakage of solution and inaccurate dosing. Semaglutide should not be used if the solution does not appear clear and colourless or almost colourless. The pen is designed to be used with NovoFine or NovoTwist disposable needles and pens are packed with an appropriate quantity of NovoFine Plus needles.

Detailed information on administration of semaglutide is provided in the current Summary of Product Characteristics and product Patient Information Leaflet.

Dapagliflozin (Forxiga®))and matched placebo are presented as a capsule for once daily oral administration.

8.6 Dosage schedules

Semaglutide will be administered and dosed as described. Participants will be commenced on 0.25 mg s.c once weekly before up-titration as tolerated to 1.0 mg s.c once weekly. Up-titration is quicker than is ordinarily the case in clinical practice in type 2 diabetes as discussed in Section 2.1 (22) There will be increased observation and clinical access for participants in the event of side-effects. Participants randomised to receive semaglutide will continue on the 1.0 mg s.c once weekly dose for the remainder of the study following uptitration provided they tolerate the dose as detailed in Section 7. If necessary, the dose may be adjusted.
If a participant misses a dose of semaglutide it should be administered as soon as possible and within 5 days after the missed dose. If more than 5 days have passed, the missed dose should be skipped, and the next dose should be administered on the regularly scheduled day. In both cases participants, should then resume their regular once weekly dosing schedule.

Dapagliflozin and matched placebo will be commenced at 5mg orally once daily and uptitrated one week after commencement to a dose of 10mg which will thereafter be continued for the remainder of the trial provided they tolerate the dose. If necessary, the dose may be adjusted.

8.7 Dosage modifications

Dapagliflozin/placebo will be commenced at one capsule (5mg or placebo) orally once daily and uptitrated one week after commencement to a dose of two capsules (10mg or placebo) which will thereafter be continued for the remainder of the trial provided they tolerate the dose. If necessary, the dose may be adjusted. (See also Section 7.8 regarding treatment discontinuation) Dapagliflozin or placebo capsules can be taken at any time of day with or without food. Capsules should be swallowed whole. If participants forget to take a dose they should be advised as follows:

- If it is 12 hours or more until the next dose, the dose should be taken as soon as remembered and the next dose taken at the usual time.
- If it is less than 12 hours until the next dose, the missed dose should be skipped. The next dose should be taken at the usual time.
- Participants should not take a double dose of dapagliflozin/placebo to make up for a forgotten dose.

8.8 Known drug reactions and interaction with other therapies

Participants with hypersensitivity to either active study medication are excluded from the trial. There are no significant interactions between semaglutide and other prescribed medications other than with agents that can lower blood glucose levels. Participants on any other oral agents which can lower blood glucose levels meet Exclusion Criteria and will not be included in this trial. In combination with insulin and dapagliflozin, semaglutide may be associated with an increased risk of hypoglycaemia. Participants will be advised of the increased risk of hypoglycaemia and will be required to closely monitor their blood glucose levels for the duration of the trial. No interactions are currently listed between warfarin and semaglutide in the British National Formulary but in view of a theoretical interaction (based on protein binding), existing arrangements for INR testing will be reviewed for any participants prescribed warfarin and additional tests conducted if indicated according to the judgement of the investigators.

Dapagliflozin is known to increase the risk of hypoglycaemia when combined with other medications which also act to lower blood glucose levels. Dapagliflozin has a mild diuretic effect and therefore there is an increased risk of hypotension when dapagliflozin is combined with either medications which deplete circulating volume (e.g diuretics) or antihypertensive drugs. Dapagliflozin is however commonly prescribed with such medications in general medical practice. All participants will have their medications reviewed at the screening visit and educated regarding symptoms that may indicate dehydration/ hypovolaemia.

Dapagliflozin may increase renal lithium excretion leading to decreased blood lithium concentrations. Serum lithium concentrations should be monitored for participants eligible for randomisation to dapagliflozin/placebo prior to visit 10 (week 26, second randomisation), as clinically indicated thereafter, and in the event of dose changes, taking into account the planned dose increase at visit 11 (week 27).

If there is any concern of interaction with any of the IMPs, this will be discussed with the PI before a decision regarding their ability to participate is made.

COVID-19: COVID-19 infection and the clinical management in this therapeutic area is also rapidly evolving as new medicines are approved for use by local/national treatment guidelines. It is therefore not possible to comprehensively rule out the potential for clinically significant interactions between the study IMP(s) with current or future medicines for the acute treatment of COVID-19 and a pragmatic approach is required. Medicines for acute COVID-19 infection should not be unnecessarily withheld and participants should be managed wherever possible in line with local/national clinical guidelines for this patient group. The decision to follow a particular management plan for acute COVID-19 infection (which may include the temporary withdrawal of the IMP(s) should this be in the participant’s best interest), is at the treating physician’s discretion. Investigators should refer to the protocol and current IMP Summary of Product Characteristics for detailed information on known mechanisms for pharmacokinetic/pharmacodynamics interactions as well as the relevant COVID-19 treatment Summary of Product Characteristics (or equivalent) to inform management decisions. Any mitigations, such as additional patient monitoring, must be documented in the participant's medical notes.

Further ad-hoc advice can also be obtained from the Sponsor/Chief Investigator and https://www.covid19-druginteractions.org/ Investigators must be vigilant at all times to the possibility of interactions and report in line with protocol pharmacovigilance requirements. Concomitant use of other medicines for COVID-19 must be recorded in the participant’s CRF. Investigational treatments for acute COVID-19 treatment are not permitted without prior approval from the sponsor.

8.9 Concomitant medication

Participants should not receive any adjunct glucose-lowering therapy other than as per the randomisation schedule (e.g. with either of the active study medications).

8.10 Trial restrictions

Contraindications will follow SmPC information for Ozempic® and Forxiga. Acceptable forms of contraception for WOCBP participating in the trial are listed in Appendix 2. There is no requirement for contraception for male participants.

8.11 Assessment of compliance

Treatment adherence will be assessed by the trial medical and nursing team when participants attend for scheduled study visits.

8.12 Name and description of each Non-Investigational Medicinal Product (NIMP)

Insulin therapy is a non-investigational medicinal product in this trial. The trial is in a population who have had a diagnosis of type 1 diabetes for at least one year: therefore all participants will already be established on insulin therapy. The regimen and dosage required will by differ between participants. Participants will be using a combination of long acting (basal) insulin and shorter acting (bolus) insulin or CSII (insulin pump). As in standard of care, dose titration will depend on diet, blood glucose levels and exercise. It is anticipated that dosage adjustments will be required to achieve optimal glycaemic control and maintain participant safety.

There are pre-defined guidelines for insulin titration in this study. Insulin dose reductions should not be greater than 10% (5% if HbA1c is<7%) from current total insulin dose or more than 20% from the baseline level at randomisation and then from the new baseline levels at 26 weeks following second randomisation. Participants requiring an insulin dose reduction > 20% to a total insulin <0.4 U/kg will be monitored closely for insulin dose, carbohydrate intake, ketones and blood glucose. Study medication can be temporarily discontinued, if necessary, for one-to-two weeks. If low insulin requirements remain, the participant will be withdrawn from study (see Section 7.8).

9 PHARMACOVIGILANCE

9.1 Definitions

Term	Definition
**Adverse Event (AE)**	Any untoward medical occurrence in a participant to whom a medicinal product has been administered, including occurrences which are not necessarily caused by or related to that product.
**Adverse Reaction (AR)**	An untoward and unintended response in a participant to an investigational medicinal product which is related to any dose administered to that participant.<br>The phrase "response to an investigational medicinal product" means that a causal relationship between a trial medication and an AE is at least a reasonable possibility, i.e. the relationship cannot be ruled out.<br>All cases judged by either the reporting medically qualified professional or the Sponsor as having a reasonable suspected causal relationship to the trial medication qualify as adverse reactions.
**Serious Adverse Event (SAE)**	A serious adverse event is any untoward medical occurrence that:<br>1. results in death<br>2. is life-threatening<br>3. requires inpatient hospitalisation or prolongation of existing hospitalisation<br>4. results in persistent or significant disability/incapacity<br>5. consists of a congenital anomaly or birth defect<br><br>Other ‘important medical events’ may also be considered serious if they jeopardise the participant or require an intervention to prevent one of the above consequences.<br>NOTE: The term "life-threatening" in the definition of "serious" refers to an event in which the participant was at risk of death at the time of the event; it does not refer to an event which hypothetically might have caused death if it were more severe*.
**Serious Adverse Reaction (SAR)**	An adverse event that is both serious and, in the opinion of the reporting Investigator, believed with reasonable probability to be due to one of the trial treatments, based on the information provided.
**Suspected Unexpected Serious Adverse Reaction (SUSAR)**	A serious adverse reaction, the nature and severity of which is not consistent with the information about the medicinal product in question set out:<br>• in the case of a product with a marketing authorisation, in the summary of product characteristics (SmPC) for that product<br>• in the case of any other investigational medicinal product, in the investigator’s brochure (IB) relating to the trial in question
**Reference Safety Information**	The information used for assessing whether an adverse reaction is expected. This is contained in either the investigator’s brochure or the summary of product characteristics

*Note: “Severe” is often used to describe intensity of a specific event, which may be of relatively minor medical significance. “Seriousness” is the regulatory definition supplied above.

9.2 Operational definitions for (S)AEs

Adverse events (AEs) will be recorded, notified, assessed, reported, analysed and managed in accordance with the Medicines for Human Use (Clinical Trials) Regulations 2004 (as amended) and this protocol.

All Serious Adverse Events (SAEs) occurring during the trial will be recorded within the eCRF. Non-serious AEs occurring during the trial will be recorded within the eCRF if they are considered to be related to trial medication, i.e. adverse reactions (ARs).

Exclusions from SAE reporting

Hospitalisation for the following reasons will not be considered to be SAEs:

- Treatment which was elective or pre-planned, for a pre-existing condition not associated with any deterioration in condition, e.g. pre-planned hip replacement operation which does not lead to further complications.
- Any admission to hospital or other institution for general care where there was no deterioration in condition.
- Treatment on an emergency, outpatient basis for an event **not** fulfilling any of the definitions of serious as given above and not resulting in hospital admission.
- COVID-19 infection unless results in severe illness with hospital admission.

9.3 Recording and reporting of AEs, Adverse Events of Special Interest, SAEs and SUSARs

All AEs occurring during the trial that are observed by the Investigator or reported by the participant will be recorded in the participant's medical records whether or not attributed to trial IMP. AEs will be recorded from randomisation until the later of 30 days post cessation of trial treatment or the end of the study.

With the exception of those noted in section 9.3.1 below, adverse reactions will be recorded in a log provided in the eCRF.

Adverse Events of Special Interest, SAEs and SUSARs will be recorded in the participant's medical records and in a specific section of the eCRF from the point of randomisation until the later of 30 days post cessation of trial treatment or the end of the study.

9.3.1 Adverse Events of Special Interest

Ketosis is a known adverse effect of dapagliflozin (see Section 2.1b; also (i) below). Hypoglycaemia is a recognised adverse effect with both semaglutide and dapagliflozin when used in combination with insulin [see (ii) below] and is defined as moderate in intensity (Level 2) when blood glucose is < 3.0 mmol/L. Thus ketosis and Level 2 hypoglycaemia events will be recorded as Adverse Events of Special Interest (AESIs).

AESIs will be recorded within the relevant section of the eCRF but do not require reporting in an expedited manner (unless the event also meets the regulatory definition of an SAE). The following information will be recorded:

- Nature of the event
- Event duration (start and end dates, if applicable
- Relationship to study drug
- Outcome

Adverse Events of Special Interest will be monitored and followed up (if applicable) until the event has resolved or a final outcome has been reached.

All Adverse Events of Special Interest must be assessed for seriousness prior to recording within the eCRF. Those meeting the definition of an SAE should also be reported accordingly as per Section 9.3.2.

(i) Ketosis and diabetic ketoacidosis

This trial will be conducted in participants who have type 1 diabetes. All participants will be taking insulin. In this condition, due to the near absence of endogenous insulin, at a cellular level there is an inability to incorporate glucose into normal intra-cellular energy yielding pathways. In this context, alternative energy yielding pathways are sought including ketone body production from fatty acids in the liver. Ketones are clinically important as they easily dissociate hydrogen ions upsetting physiologically regulated acid base balance. As ketone levels rise, there is initially a state of high blood ketone levels without significant acidosis (ketosis); if this develops further, diabetic ketoacidosis (DKA) may develop. DKA requires urgent medical input. Ketones can be easily measured via a simple urine test or fingerstick capillary blood measurement. Participants eligible for this trial will already be trained in how to do this. As discussed in Background ((1) above), dapagliflozin, one of the IMPs in this trial, is known to increase the risk of ketosis and ketoacidosis and this can occur at lower blood glucose levels than in those who are on insulin treatment only [euglycaemic DKA] (13,14). Given this known risk, ketosis and DKA are included in the safety endpoints and as such will be recorded in the eCRF as Adverse Events of Special Interest.

DKA is defined clinically and biochemically as a combination of a state of elevated ketone levels (urine ++ or higher, and/or blood > 1.5 mmol/L) in combination with biochemical evidence of a state of metabolic acidosis (pH ≤7.30 or serum bicarbonate < 15 mmol/L). This is as defined by International Consensus Guidelines (34). DKA also varies in severity. In clinical practice the diagnosis is made when a person with type 1 diabetes has elevated ketone levels and usually – although not exclusively – elevated blood glucose levels. The diagnosis is made biochemically by assessing acid base balance through blood testing. This corresponds to the following severity classifications:

- mild DKA: pH 7.25-7.30 (H^+ 50 – 56) or bicarbonate (15 -17.9 mmol/L)
- moderate DKA: pH 7.00-7.24 (H^+ 57 – 100) or bicarbonate 10 – 14.9 mmol/L)
- severe DKA: pH <7.00 (H^+ > 100) or bicarbonate < 10 mmol/L (39)

In addition to the above, the highest level of either blood or urine ketones measured, the serum bicarbonate (if measured) and blood pH (if measured) will be recorded for each episode. Given the known risk of DKA, all participants will be made aware of this condition. Patient diaries and Patient Alert Cards will summarise advice for management of high ketone levels including an emergency ketosis management protocol (STICH Protocol – see Appendix 3) (37). All participants will be educated as to the scenarios when this should be activated and this will also be included on the Patient Alert Card which will have the contact details of the study team and also contact details for out of hours health services. Participants will be asked to carry this card at all times.

(ii) Hypoglycaemia

Hypoglycaemia is a low blood glucose level. Hypoglycaemia occurs in diabetes owing to difficulty in precisely matching insulin dosing with the complex factors associated with carbohydrate intake and physical activity. When too much insulin relative to carbohydrate intake is administered, blood glucose levels can drop to a level which is too low. Most people with type 1 diabetes can recognize this phenomenon due to a combination of symptoms which alert them and prompt self-checking of their capillary blood glucose level. Symptoms include weakness, malaise, headache, tremulousness, diaphoresis, palpitations and mild confusion. These are known as autonomic symptoms as they are mediated by activation of the autonomic nervous system as a response to the biological stress of low serum blood glucose. As hypoglycaemia progresses, however, symptoms progress to include confusion and agitation. If untreated this can lead to coma. These symptoms are known as neuroglycopenic symptoms as they are mediated by a lack of glucose in neural cells leading to a confused state.

Hypoglycaemia is also well defined by International Consensus (34). Like DKA, it can be classified in terms of severity. This can be based either on the absolute capillary glucose value and/or clinical features and is classed into stages:

- Level 1 (mild) hypoglycaemia: Blood glucose is 3.0 – 3.8 mmol/L inclusive. At each visit the number of episodes since the last visit during which blood glucose was within this range (but not lower) will be recorded on the “Fasting Blood Glucose” tab of the eCRF following review of the participant’s study diary and (if applicable) FGM/ CGM download.
- Level 2 (moderate) hypoglycaemia: Blood glucose is < 3.0 mmol/L At each visit the number of episodes since the last visit during which blood glucose was within this range at any time will be recorded on the AESI tab of the eCRF following review of the participant’s study diary and (if applicable) FGM/ CGM download.
- Level 3 (severe) hypoglycaemia: A person is unable to function because of mental or physical changes due to hypoglycaemia and requires assistance from another person to recover. These events will be recorded as SAEs (see below) independent of recording of blood glucose according to the opinion of the site Principal Investigator as by definition they jeopardise the participant or require an intervention to prevent life-threatening consequences.

The IMPs in this trial are licensed clinically in type 2 diabetes to lower blood glucose levels. It therefore follows that when used in combination with insulin there may be an increased risk of low blood glucose levels. This was the case with the once daily GLP-1 analogue liraglutide in phase 3 trials (8,9), but not in the trials with dapagliflozin (13,14). All participants will have this risk discussed with them, will be performing increased blood glucose monitoring than is the case in usual care, and will be frequently reviewed to adjust insulin dosing in order to mitigate the risk of low blood glucose levels.

9.3.2 Site Reported Serious Adverse Events (SAE)

SAEs will be recorded within the eCRF from the date of randomisation until the later of 30 days post cessation of trial treatment or the end of the study.

Full details of SAEs will be recorded in the electronic Case Report Form. The following information will be collected:

- full details in medical terms and a case description
- seriousness criteria
- event duration (start and end dates, if applicable)
- action taken
- outcome
- causality (i.e. relatedness to trial drug / investigation), in the opinion of the investigator

- if related, whether the reaction would be considered expected or unexpected.

Any change of condition or other follow-up information should be added to the eCRF and forwarded to the Sponsor (if reportable SAE) as soon as it is available or at least within 24 hours of the information becoming available. Events will be followed up until the event has resolved or a final outcome has been reached.

Reporting of suspected/confirmed COVID 19 infection

If a patient presents with clinical signs and symptoms consistent with COVID-19 and a test has been performed or requested:

- If test is positive, record “COVID-19 confirmed” in the diagnosis field
- If test is negative, record the SAE signs and symptoms and/or other diagnosis in the diagnosis field.

If a test has not been performed or result is not available and signs and symptoms, as judged by the investigator, are highly suspicious of COVID-19 infection, record “COVID-19 suspected” in the diagnosis field

If other concurrent diagnoses, e.g. pneumonia, please record within the narrative of the SAE.

COVID-19 vaccination and reporting

Where a deployed COVID-19 vaccine is suspected to be involved in the onset of a reported event it should be recorded as a concomitant medication. A causal relationship between the vaccine and the event, including potential drug interactions should be assigned by the reporting investigator.
If a reported event is suspected to be due to a deployed COVID-19 vaccine alone reporting investigators should ensure that standard Yellow Card reporting procedures are followed.

Assessment of Adverse Events

All adverse events must be assessed by the reporting site for seriousness**.** All SAEs must also be assessed for causality and expectedness with reference to this protocol and the Reference Safety Information (RSI).

Assessment of seriousness

An adverse event will be considered serious if it:

1. Results in death
2. Is life threatening
3. Requires hospitalisation or prolongation of existing hospitalisation
4. Results in persistent or significant disability or incapacity
5. Consists of a congenital anomaly or birth defect
6. Required intervention to prevent one of the above.
7. Is a serious Adverse Event of Special Interest.

Assessment of causality i.e. does the event have a “reasonable causal relationship” with trial IMPs. The following categories are used:

- **Yes** - The causal and temporal relationship between the event and the absence of a more likely explanation suggest that the trial IMP is the most likely cause.
- **No** - The event is not considered related to the trial IMPs.

Causality will be assigned for each IMP the participant is randomised to receive.

Should an SAE be considered unrelated to the administration of either trial drug then the causality of the event will be collected taking into consideration other factors, for example concomitant medications or co-morbidities, etc.

Assessment of causality is the responsibility of the Principal Investigator or medically-qualified designee(s) at each site. The CI, their delegate(s), or the Sponsor may provide an opinion regarding causality but cannot downgrade the PI's assessment.

Assessment of expectedness

Should the event be considered to be related to the trial IMP(s), an assessment should be made regarding the expectedness of the reaction, i.e. whether the reaction is a recognised adverse effect of the IMP(s).

.
Expected: consistent with the relevant product information documented in the RSI(s).
Unexpected: not consistent with the relevant product information documented in the RSI(s).

The CI, their delegate(s), or the Sponsor have overall responsibility for assigning expectedness as per the RSI(s) and provide the final assessment of the event. The reporting investigator may provide an opinion on the expectedness of an event and this may be taken into account.

SAEs occurring within the USA and the UK

The US Sponsor is responsible for assigning causality and expectedness for any AEs, SAEs and SUSARs that occur within the USA. Where a SUSAR is identified by the US Sponsor this will be forwarded to the UK Sponsor for review within one working day. Following review by the UK Sponsor, appropriate US SUSARs will be subject to expedited reporting to the MHRA as per UK regulatory requirements. Similarly, where a SUSAR is identified by the UK sponsor, this will forwarded to the US Sponsor for review within two working days.

Recording and reporting of SAEs
All SAEs arising during the clinical trial will be recorded in the eCRF soon as reasonably practicable and in any event within 24hours of first becoming aware of the event. Any follow-up information should also be reported.

If recording in the eCRF is not possible a paper SAE form should be completed:

1. The SAE form is downloaded from www.glasgowctu.org, printed off, completed and signed. The form is then faxed to the Glasgow Clinical Trials Unit Pharmacovigilance (PV) Office on +44(0)141 357 5588. As an alternative, a scanned copy of the SAE form may be emailed to: pharmacovig@glasgowctu.org. If this website is unavailable a paper copy of the SAE form should be filed in the Investigator Site File at each site pending resolution.
2. If necessary a verbal report can be given by contacting the PV Office on +44(0)141 330 4744. This must be followed up as soon as possible with an electronic or written report.

Reporting to sponsor

All SAEs will be considered reportable to the Sponsor PV office from the time of randomisation until 30 days following permanent discontinuation of all trial IMP(s). SAEs occurring following 30 days post cessation of treatment are not reportable to the Sponsor unless an SAE is considered related to IMP(s)

9.3.3 Suspected Unexpected Serious Adverse Reactions (SUSARs)

Any SAE assessed as related to trial IMP(s) by the PI or delegate, and unexpected (not documented as an expected reaction to the IMP(s) within the RSI(s)) by the CI, their delegate, or the Sponsor, will be classified as a SUSAR and therefore subject to expedited reporting to the Medicines and Healthcare products Regulatory Agency (MHRA) and the Research Ethics Committee (REC). Should the CI disagree with the PI's assessment of causality, both opinions will be provided on the report. Where the CI or UK Sponsor disagrees with the US Sponsor's assessment of expectedness both opinions will also be provided on the report.

The Sponsor will inform the MHRA of all SUSARs that occur within the trial and the REC of all SUSARs that occur within the UK within the required expedited reporting timescales:

- **Fatal or life threatening SUSARs** - no later than 7 days of Sponsor awareness that the case fulfils the criteria for a fatal or life threatening SUSAR, and any follow up information within a further 8 days.
- **All other SUSARs** - no later than 15 days of Sponsor awareness that the case fulfils the criteria for a SUSAR.

The sponsor will report SUSARs to the MHRA via the MHRA eSUSAR reporting system and to REC by email with accompanying CTIMP Safety Report Form.

9.3.4 Unblinding for SUSAR reporting purposes

In the event of a potential SUSAR, the sponsor (but not the investigators) will be unblinded to facilitate appropriate reporting to the MHRA and REC.

9.4 Responsibilities for Safety Reporting and Review

This section details the responsibilities for reporting and reviewing safety information arising from the trial.

Principal Investigator (PI):

- Attendance at the initiation meeting/teleconference
- Training of new members of the trial team in the protocol and its procedures
- Ensuring that the ISF is accurately maintained
- Dissemination of important safety or trial-related information to all stakeholders at their site
- Checking for AEs and ARs when participants attend for treatment/follow-up
- Ensuring that AEs are recorded and reported in line with the requirements of the protocol
- Ensuring that all SAEs are recorded and reported to the Sponsor within 24 hours of becoming aware of the event, and providing further follow-up information as soon as available
- Using medical judgement in assigning causality of SAEs

- Providing an opinion on the expectedness of an SAE with reference to the trial protocol and Reference Safety Information
- Using definitions in this protocol, flagging events of special interest.

Chief Investigator (CI)

- Providing clinical oversight of the safety of patients participating in the trial, including an ongoing review of the risk/benefit
- Providing clinical review of section 4.8 of the representative SmPCs on an annual basis to define the Reference Safety Information for the trial
- Using medical judgement to confirm seriousness and causality of all SARs
- Reviewing expectedness of related SAEs with reference to the trial protocol and Reference Safety Information
- Preparing the clinical sections and final sign-off of the Development Safety Update Report (DSUR)
- Using definitions in this protocol, confirming events of special interest

Sponsor:

- Central data collection and verification of AEs, SAEs, SARs and SUSARs according to the trial protocol
- Reporting safety information to the CI or delegate for the ongoing assessment of the risk / benefit
- Reporting safety information to the independent oversight committees identified for the trial (Independent Data Monitoring Committee (IDMC) and / or Trial Steering Committee (TSC)) according to the Trial Monitoring Plan.
- Expedited reporting of SUSARs to the Competent Authority (MHRA in UK) and REC within required timelines.
- Notifying Investigators of SUSARs that occur within the trial.
- Checking for (annually) and notifying PIs of updates to the Reference Safety Information for the trial.
- Preparing standard tables and other relevant information for the DSUR in collaboration with the CI and ensuring timely submission to the MHRA and REC.

Trial Steering Committee:

In accordance with the Charter for the TSC, periodically reviewing recruitment and the overall progress of the trial and liaising with the IDMC and sponsor regarding safety issues.

Independent Data Monitoring Committee:

In accordance with the Charter for the IDMC, periodically reviewing unblinded safety data in individual cases and to determine patterns and trends of events, or to identify safety issues, which would not be apparent on an individual case basis, reporting concerns to the TSC and sponsor.

9.5 Pregnancy reporting

Pregnancy is not considered an AE unless a negative or consequential outcome is recorded for the mother or child/foetus. If the outcome meets the serious criteria, this would be considered an SAE and must be reported as per SAE reporting procedure above.

Any **pregnancy** occurring in a female trial participant or female partner of a male trial participant who becomes pregnant while participating in the Trial will be reported by the PI (or designee) to the Chief Investigator and the sponsor using the sponsor Pregnancy Reporting Form (available at http://www.glasgowctu.org/) within two weeks of the PI first becoming aware of the pregnancy.

The trial participant will also be followed up to determine the outcome of the pregnancy and follow-up information forwarded to the PV office. Any resulting SAEs should be reported as per SAE reporting procedure above.

9.6 Overdose

Any IMP dose which is not administered in accordance with the Protocol should be reported to the sponsor.
If an SAE is associated with an overdose ensure that the overdose if fully described in the SAE report form.

9.7 Reporting urgent safety measures

If any urgent safety measures are taken the CI/Sponsor will phone the MHRA's Clinical Trial Unit on 020 3080 6456, ideally within 24 hours. This will be followed up no later than 3 days from the date the measures are taken, giving written notice to the MHRA (who will advise the format required) and the relevant REC of the measures taken and the circumstances giving rise to those measures. A substantial amendment must also be submitted to the MHRA.

9.8 The type and duration of the follow-up of participants after adverse events.

Adverse events and reactions will be recorded, reported and followed up in line with this protocol until study completion or for a minimum of 30 days after participant's last dose of the IMP, whichever is later. Any SUSAR identified will be reported to the Sponsor and to the Regulatory Authorities irrespective of how long after IMP administration the reaction has occurred.

9.9 Development safety update reports

A Development Safety Update Report (DSUR) will be submitted once a year, or on request, to MHRA and REC until the trial is declared ended. The report will be submitted within 60 days of the anniversary of the issue of the Clinical Trials Authorisation for the trial. The DSUR will be prepared by the sponsor (PV Office) in liaison with the CI and submitted by the sponsor (PV Office). The DSUR will include only those SAEs reported to the co-sponsors; that is: SAEs and SUSARs occurring within the UK and SUSARs originating in the USA.

10 STATISTICS AND DATA ANALYSIS

10.1 Sample size calculation

This is based on our current data showing that in type 1 diabetes, treatment with a GLP-1 agonist, liraglutide (1.2 and 1.8mg doses) for three months leads to HbA1c suppression by an average of 0.6% (SD= 0.5%). Dapagliflozin was recently shown to reduce HbA1c by 0.45% in type 1 diabetes patients (13). Additionally, empagliflozin treatment for 8 weeks was shown to reduce HbA1c by 0.4% in type 1 diabetics (20, 21) and our current data showing additional improvement in HbA1c by an additional 0.66% at 12 weeks when dapagliflozin was added to liraglutide and insulin therapy. Conservatively estimating that the combination therapy will suppress HbA1c by 0.5% (SD≤0.7%) compared to semaglutide alone, a sample size of 38 (32 evaluable patients) in each group will be sufficient to provide 80% power at a 5% significance level, taking in consideration a 15% dropout rate. 50% of participants will be recruited in Glasgow, UK and 50% at State University of New York at Buffalo, USA.

10.2 Anticipated recruitment rate

One year (one participant per week).

10.3 Statistical analysis

The primary objective of the proposed research is to evaluate the effect of addition of dapagliflozin to semaglutide on a background of insulin treatment in type 1 diabetes patients on HbA1c at six months following start of dapagliflozin. Transformations of the data in order to meet statistical assumptions will be considered. Statistical analyses will use linear regression models to compare treatment groups, with adjustment for baseline values of study outcomes, as well as factors used to stratify the randomisation scheme. Tests will be two-sided and tested at the 5% significance level. Analyses will be carried out under the intention to treat principle, that is, according to randomised treatment allocation, regardless of treatment adherence.

A detailed Statistical Analysis Plan will be completed and signed off before database lock. Study statisticians will develop and validate the analysis programs without access to randomised treatment allocations. After database lock, the treatment codes will be released and the final analysis will be carried out.

10.4 Subgroup analyses

These will be specified in the Statistical Analysis Plan. Subgroup analyses will be implemented by extending regression models to include interactions between treatment and potential treatment effect moderators.

10.5 Interim analysis and criteria for the premature termination of the trial

No interim analysis is planned. Unblinded trial data will be reviewed on an ongoing basis by the IDMC. The primary role of the IDMC will be to protect the interests of the patients. The IDMC may recommend to the TSC and Co-Sponsors that the study should stop prematurely because of concerns about patient safety. The IDMC will meet approximately every six months. The IDMC will take into account all results and the consistency and biological plausibility of the findings in making any recommendation. The final decision on continuing or stopping the trial will lie with the TSC/Co-Sponsors.

10.6 Subject population

All analyses will be carried out on an intention to treat basis, using the randomised treatment allocation.

10.7 Procedure(s) to account for missing or spurious data

Data will be analysed without imputation in the first instance. Multiple imputation procedures will be used to assess the sensitivity of selected results to missing data, according to the Statistical Analysis Plan.

10.8 Other statistical considerations.

The Statistical Analysis Plan (SAP) will be maintained as a version controlled document and will be signed off before database lock. The SAP and other statistical documentation (according to Glasgow Clinical Trials Unit Standard Operating Procedures) will contain full details of all analyses along with assumptions and procedures for handling problematic or incomplete data (e.g. incomplete dates).

11 DATA HANDLING

11.1 Source Documentation

ICH GCP defines source data as: 'All information in original records and certified copies of original records of clinical findings, observations or other activities in a clinical trial necessary for the reconstruction and evaluation of the trial'. In this study, the location of the majority of the source data will be the hospital's medical records including subject case notes, laboratory records and ECGs. The source data transcribed into the eCRF from the medical records must be accurate and verifiable. For questionnaires completed by trial subjects, the completed questionnaires will be regarded as the source data location. In cases where data is transcribed directly into the eCRF and no other paper or electronic source exists, then the eCRF will be considered the source record. In these cases, these data should be prospectively documented in the medical records to ensure a full record of the trial is available at site.

11.2 Data collection

An eCRF, developed by the Robertson Centre for Biostatistics, will capture all data required to meet this protocol's requirements. Access to the eCRF will be restricted, via a study-specific web portal, and only authorised site-specific personnel will be able to make entries to their patients' data via the web portal. The Investigator, or his/her designee will be responsible for all entries into the eCRF and will confirm that the data are accurate, complete and verifiable. Data will be stored in a MS SQL Server database. Direct access to the web portal will be granted, on request, to authorised representatives from the Sponsor, host institution and the regulatory authorities to permit trial-related monitoring, audits and inspections.

11.3 Data Validation

Where it is practical, data will be validated at the point of entry into the eCRF. Any additional data discrepancies will be flagged to the investigator and any data changes will be recorded to maintain a complete audit trail (reason for change, date change made, who made change).

11.4 Data Security

The Robertson Centre for Biostatistics systems are fully validated in accordance with industry and regulatory standards, and incorporate controlled access security. High volume servers are firewall protected and preventative system maintenance policies are in place to ensure no loss of service or data. Web servers are secured by digital certificates. Data integrity is assured by strictly controlled procedures, including secure data transfer procedures. Data are backed up on-site nightly and off-site to a commercial data vault weekly. The Robertson Centre for Biostatistics has an ISO 9001:2008 quality management system and ISO 27001:2013 for Information Security, and is regularly inspected against the standards by the British Standards Institution.

11.5 Archiving

The Trial Master File will be archived by the Co-Sponsors at the end of the trial for a minimum period of five years.

Archiving of Site Files will also be for a minimum of seven years fromm completion of the trial, and this action will be delegated to the sites in the Clinical Trial Site Agreement that will be put in place between Co-Sponsors and Sites. Sites will be notified by the Co-Sponsors when Site files can be archived.

Destruction of site files can only take place with the approval of the Co-Sponsors.

12 MONITORING, AUDIT & INSPECTION

Monitoring will be conducted by NHS Greater Glasgow and Clyde (GG&C) Monitor (s) in accordance with local Standard Operating Procedures. The level, frequency and priorities of monitoring will be based on the outcome of the completed Risk Assessment, and clearly documented in the Monitoring Plan which will be approved by the NHS GG&C Lead Monitor and/or Research Governance Manager.

13 ETHICAL AND REGULATORY CONSIDERATIONS

13.1 Research Ethics Committee (REC) review & reports

Before the start of the trial, approval will be sought from a REC for the trial protocol, informed consent forms and other relevant documents e.g. advertisements and GP information letters.
Substantial amendments that require review by REC will not be implemented until the REC grants a favourable opinion for the study (it is noted that amendments may also need to be reviewed and accepted by the MHRA and/or NHS R&D departments before they can be implemented in practice at sites).
All correspondence with the REC will be retained in the Trial Master File/Investigator Site File.
An annual progress report (APR) will be submitted to the REC within 30 days of the anniversary date on which the favourable opinion was given, and annually until the trial is declared ended (this is the Chief Investigator's responsibility).
The Chief Investigator will notify the REC of the end of the study.
If the study is ended prematurely, the Chief Investigator will notify the REC, including the reasons for the premature termination
Within one year after the end of the study, the Chief Investigator will submit a final report with the results, including any publications/abstracts, to the REC.

13.2 Peer review

The study protocol has been developed with expert and independent feedback from the JDRF (New York) and during application for funding underwent peer review by independent experts.

13.3 Public and Patient Involvement

The study was designed with input from people with type 1 diabetes via the funder, JDRF.

13.4 Regulatory Compliance

The trial will not commence until a Clinical Trial Authorisation (CTA) is obtained from the MHRA.
The protocol and trial conduct will comply with the Medicines for Human Use (Clinical Trials) Regulations 2004 and any relevant amendments.
Before any site can enrol patients into the trial, the Chief Investigator/Principal Investigator or designee will apply for NHS permission from the site's Research & Development (R&D) department.

For any amendment that will potentially affect a site's NHS permission, the Chief Investigator/ Principal Investigator or designee will confirm with that site's R&D department that NHS permission is ongoing (note that both substantial amendments, and amendments considered to be non-substantial for the purposes of REC and/or MHRA may still need to be notified to NHS R&D).

13.5 Protocol compliance

Prospective, planned deviations or waivers to the protocol are not allowed under the UK regulations on Clinical Trials and must not be used e.g. it is not acceptable to enrol a participant if they do not meet the

eligibility criteria or restrictions specified in the trial protocol. Accidental protocol deviations can happen at any time. They must be adequately documented on the relevant forms and reported to the Chief Investigator, Sponsor and GCTU immediately. Deviations from the protocol which are found to frequently recur are not acceptable, will require immediate action and could potentially be classified as a serious breach.

13.6 Notification of Serious Breaches to GCP and/or the protocol

A "serious breach" is a breach which is likely to affect to a significant degree –

1. the safety or physical or mental integrity of the subjects of the trial; or
2. the scientific value of the trial

If any of the above occurs then the CI and Sponsor will be notified. The sponsor will notify the appropriate authorities in writing of any serious breach in accordance with their standard operating procedures.

13.7 Data protection and patient confidentiality

All investigators and trial site staff must comply with the requirements of the GDPR with regards to the collection, storage, processing and disclosure of personal information and will uphold the Act's core principles.

- Personal information will be collected via the eCRF to enable record linkage to be carried out and to provide electronic access to study monitors to a copy of the signed informed consent document. These data items will be encrypted and only those individuals who require to see these data i.e. the person performing the record linkage and site research team staff or the study monitor, as appropriate, will be able to view them. All electronic data will be held securely in accordance with ISO 27001:2013 at the Robertson Centre for Biostatistics, part of the Glasgow Clinical Trials Unit. All Centre staff are required to sign confidentiality agreements and to follow Standard Operating Procedures in accordance with Good Clinical Practice and ISO certification.
- The trial data managers, statisticians, health economists or any other staff who will perform data related tasks will only be able to access depersonalised data where the participant's identifying information is replaced by a unique study identifier.

- Only those that have been trained and approved will be able to enter or view any data via the web portal. Each site can only see their own patients' data. Patient consent forms will be stored at the study site in a secure location accessible only to study teams.

13.8 Indemnity

The Co-Sponsors (University of Glasgow and Greater Glasgow Health Board) will ensure that provision has been made for insurance or indemnity to cover the liability of the investigator and sponsor which may arise in relation to the clinical trial in accordance with Part 2 (14) of Schedule 1 to SI 2004/1031.

13.9 Amendments

Any change in the study protocol will require an amendment. Any proposed protocol amendments will be initiated by the CI following discussion with the Sponsor and TSC and any required amendment forms will be submitted to the regulatory authority, ethics committee and Sponsor. The Sponsor will determine whether an amendment is non-substantial or substantial. All amended versions of the protocol will be signed by the CI and Sponsor representative. Following a substantial amendment, favourable opinion/approval must be sought from the original reviewing REC, MHRA (where appropriate) and Research and Development (R&D) office prior to implementation. The Chief Investigator will be responsible for informing the Trial Management Group of all protocol amendments.

13.10 Post trial care

At the end of the trial, participants will be returned to usual care as defined by local and national guidelines at that time. The results of the trial may of course have an impact on these guidelines and the future care of patients with type 1 diabetes.

13.11 Access to the final trial dataset

During the trial and in the period prior to publication of the main study results as described in the protocol, only the Glasgow CTU and Professor Paresh Dandona, US Principal Investigator, University of Buffalo will have access to the full dataset. After that period, the trial Steering Committee will conduct further data analyses for a period of three years. After that time the Trial Steering Committee will consider requests from external parties for further analyses of the study data. Proposals that are scientifically well founded and have an academic basis and where relevant data extractions and analyses are appropriately funded will not be refused. These will be considered as collaborative exercises where the contributions related to study design, conduct, database creation and maintenance and data analysis will be recognised in authorship of any scientific publication. The approach we will take will be to minimise any possibility of breach of participant confidentiality. Normally this will be achieved by minimising data travel. However, for the purposes of individual patient meta-analysis and other reasons, data may be transferred to other sites. Such transfer will require assurances on information security systems at the sites that data are to be transferred to and will involve a legal data transfer agreement. A log of all data requests and subsequent data transfers will be held at the Glasgow CTU.

14 DISSEMINATION POLICY

14.1 Dissemination policy

The study database will be owned by the University of Glasgow and maintained on behalf of the Study investigators, represented by the Trial Steering Committee as it is constituted during and after the trial.

The study protocol and a description of the recruitment experience and participant baseline characteristics will be published before study completion. On completion of the trial, the database will be locked and analysed by staff of the Robertson Centre for Biostatistics, University of Glasgow. A final study report will be prepared and the results will be published in a major medical journal.

After the main study publications, study investigators will be invited to submit requests for further analyses of the study database. These will be reviewed and prioritised by a Publications Committee made up of the study grant holders and convened by the study co-CIs.

As funder of the trial, JDRF will have the right to see and comment on any results being submitted for publication. A maximum of 28 days will be given for review of major papers and 14 days for abstracts. Such comments will be considered by the Trial Steering Committee.

A lay summary of the main results of the trial will be prepared and provided to all participants via their study site investigators. Investigators may request a copy of the study data for their participants. Providing some or all of a patient's data to that individual is at their discretion.

14.2 Authorship eligibility guidelines and any intended use of professional writers

The main results of the study will be compiled, written up and published by the study grant holders and others taking responsibility for the study results (e.g. the statistician conducting the final analysis) on behalf of the TTT1 investigators. The TTT1 investigators will be listed in an Appendix and will include all site PIs, all committee members and key members of relevant study coordinating groups (including the Sponsor and Glasgow CTU).

15 REFERENCES

1. Konrad K, Datz N, Engelsberger I, Grulich-Henn J, Hoertenhuber T, Knauth B, et al. Current use of metformin in addition to insulin in pediatric patients with type 1 diabetes mellitus: an analysis based on a large diabetes registry in Germany and Austria. *Pediatr Diabetes.* 2014.
2. Vella S, Buetow L, Royle P, Livingstone S, Colhoun HM, and Petrie JR. The use of metformin in type 1 diabetes: a systematic review of efficacy. *Diabetologia.* 2010;53(5):809-20.
3. Vella S, Buetow L, Royle P, Livingstone S, and Petrie JR. Metformin in type 1 diabetes reduces insulin requirements without significantly improving glycaemic control. Reply to Schatz H [letter]. *Diabetologia.* 2011;54(1):203-4.
4. Varanasi A, Bellini N, Rawal D, Vora M, Makdissi A, Dhindsa S, et al. Liraglutide as additional treatment for type 1 diabetes. *Eur J Endocrinol.* 2011;165(1):77-84.
5. Kuhadiya ND, Malik R, Bellini NJ, Patterson JL, Traina A, Makdissi A, et al. Liraglutide as additional treatment to insulin in obese patients with type 1 diabetes mellitus. *Endocrine practice : official journal of the American College of Endocrinology and the American Association of Clinical Endocrinologists.* 2013;19(6):963-7.
6. Kuhadiya ND, Dhindsa S, Ghanim H, Mehta A, Makdissi A, Batra M, et al. Addition of Liraglutide to Insulin in Patients With Type 1 Diabetes: A Randomized Placebo-Controlled Clinical Trial of 12 Weeks. *Diabetes Care.* 2016;39(6):1027-35.
7. Chaudhuri A, Ghanim H, Makdissi A, Green K, Abuaysheh S, Batra M, et al. Exenatide induces an increase in vasodilatory and a decrease in vasoconstrictive mediators. *Diabetes Obes Metab.* 2017;19(5):729-33.
8. Mathieu C, Zinman B, Hemmingsson JU, Woo V, Colman P, Christiansen E, et al. Efficacy and Safety of Liraglutide Added to Insulin Treatment in Type 1 Diabetes: The ADJUNCT ONE Treat-To-Target Randomized Trial. *Diabetes Care.* 2016;39(10):1702-10.
9. Ahren B, Hirsch IB, Pieber TR, Mathieu C, Gomez-Peralta F, Hansen TK, et al. Efficacy and Safety of Liraglutide Added to Capped Insulin Treatment in Subjects With Type 1 Diabetes: The ADJUNCT TWO Randomized Trial. *Diabetes Care.* 2016;39(10):1693-701.
10. Zinman B, Wanner C, Lachin JM, Fitchett D, Bluhmki E, Hantel S, et al. Empagliflozin, Cardiovascular Outcomes, and Mortality in Type 2 Diabetes. *N Engl J Med.* 2015;373(22):2117-28.
11. Petrie JR. SGLT2 inhibitors in type 1 diabetes: knocked down, but up again? *Lancet Diabetes Endocrinol.* 2017;5(11):841-3.
12. Mudaliar S, Armstrong DA, Mavian AA, O'Connor-Semmes R, Mydlow PK, Ye J, et al. Remogliflozin etabonate, a selective inhibitor of the sodium-glucose transporter 2, improves serum glucose profiles in type 1 diabetes. *Diabetes Care.* 2012;35(11):2198-200.
13. Kohan DE, Fioretto P, Tang W, List JF. Long-term study of patients with type 2 diabetes and moderate renal impairment shows that dapagliflozin reduces weight and blood pressure but does not improve glycemic control. Kidney Int. 2014 Apr;85(4):962-71. doi: 10.1038/ki.2013.356. Epub 2013 Sep 25. PMID: 24067431; PMCID: PMC3973038.
14. Dandona P, Mathieu C, Phillip M, Hansen L, Griffen SC, Tschope D, et al. Efficacy and safety of dapagliflozin in patients with inadequately controlled type 1 diabetes (DEPICT-1): 24 week results from a multicentre, double-blind, phase 3, randomised controlled trial. *Lancet Diabetes Endocrinol.* 2017;5(11):864-76.

15. Mathieu C, Dandona P, Gillard P, Senior P, Hasslacher C, Araki E, Lind M, Bain SC, Jabbour S, Arya N, Hansen L, Thorén F, Langkilde AM; DEPICT-2 Investigators. Efficacy and Safety of Dapagliflozin in Patients With Inadequately Controlled Type 1 Diabetes (the DEPICT-2 Study): 24-Week Results From a Randomized Controlled Trial. Diabetes Care. 2018 Sep;41(9):1938-1946.
16. European Medicines Agency. Assessment Report Dapagliflozin. https://www.ema.europa.eu/en/documents/variation-report/forxiga-h-c-ws-1941-epar-assessment-report-variation_en.pdf *(accessed 11th November 2021)*
17. Groop PH, Dandona P, Phillip M, Gillard P, Edelman S, Jendle J, Xu J, Scheerer MF, Thoren F, Iqbal N, Repetto E, Mathieu C. Effect of dapagliflozin as an adjunct to insulin over 52 weeks in individuals with type 1 diabetes: post-hoc renal analysis of the DEPICT randomised controlled trials. Lancet Diabetes Endocrinol. 2020 Oct;8(10):845-854. doi: 10.1016/S2213-8587(20)30280-1. PMID: 32946821.
18. Rosenstock J, Marquard J, Laffel LM, et al. Empagliflozin as adjunctive to insulin therapy in type 1 diabetes: the EASE trials. *Diabetes Care*. 2018;41(12):2560–2569.
19. Garg SK, Henry RR, Banks P, Buse JB, Davies MJ, Fulcher GR, et al. Effects of Sotagliflozin Added to Insulin in Patients with Type 1 Diabetes. *N Engl J Med.* 2017;377(24):2337-48.
20. European Medicines Agency. First oral add-on treatment to insulin for treatment of certain patients with type 1 diabetes. https://www.ema.europa.eu/en/news/first-oral-add-treatment-insulin-treatment-certain-patients-type-1-diabetes *(accessed 11th November 2021)*
21. An overview of Zynquista and why it is authorised in the EU. 16th May 2019. https://www.ema.europa.eu/en/documents/overview/zynquista-epar-medicine-overview_en.pdf *(accessed 11th November 2021)*
22. National Institute for Health and Care Excellence. Dapagliflozin with insulin for treating type 1 diabetes. Technology appraisal guidance [TA597] https://www.nice.org.uk/guidance/ta597
23. AstraZeneca Press Release. Update on US regulatory decision for Farxiga in type-1 diabetes. https://www.astrazeneca.com/media-centre/press-releases/2019/update-on-us-regulatory-decision-for-farxiga-in-type-1-diabetes-15072019.html# *(accessed 11th November 2021)*
24. European Medicines Agency. Forxiga (dapagliflozin) 5mg should no longer be used for the treatment of Type 1 Diabetes Mellitus. https://www.ema.europa.eu/en/documents/dhpc/direct-healthcare-professional-communication-dhpc-forxiga-dapagliflozin-5mg-should-no-longer-be-used_en.pdf *(accessed 11th November 2021)*
25. AstraZeneca. Letter to UK Health Professionals. Forxiga (dapagliflozin) 5mg should no longer be used for the treatment of type 1 diabetes mellitus. (2nd November 2021)
26. Marso SP, Bain SC, Consoli A, Eliaschewitz FG, Jódar E, Leiter LA, Lingvay I, Rosenstock J, Seufert J, Warren ML, et al. Semaglutide and cardiovascular outcomes in patients with type 2 diabetes. *N Engl J Med.* 2016;**375**(19):1834–44. doi: 10.1056/NEJMoa1607141.

27. Ozempic Summary of product characteristics. https://www.medicines.org.uk/emc/product/9728/smpc
28. Nauck MA, Petrie JR, Sesti G, Mannucci E, Courrèges JP, Lindegaard ML, Jensen CB, Atkin SL; Study 1821 Investigators. A Phase 2, Randomized, Dose-Finding Study of the Novel Once-Weekly Human GLP-1 Analog, Semaglutide, Compared With Placebo and Open-Label Liraglutide in Patients With Type 2 Diabetes. Diabetes Care. 2016 Feb;39(2):231-41.
29. Forxiga 5 mg Summary of Product Characteristics: https://www.medicines.org.uk/emc/product/2865/smpc *(accessed 11th November 2021)*
30. Wiviott SD, Raz I, Bonaca MP, Mosenzon O, Kato ET, Cahn A, Silverman MG, Zelniker TA, Kuder JF, Murphy SA, Bhatt DL, Leiter LA, McGuire DK, Wilding JPH, Ruff CT, Gause-Nilsson IAM, Fredriksson M, Johansson PA, Langkilde AM, Sabatine MS; DECLARE–TIMI 58 Investigators. Dapagliflozin and Cardiovascular Outcomes in Type 2 Diabetes. N Engl J Med. 2019 Jan 24;380(4):347-357.
31. McMurray JJV, Solomon SD, Inzucchi SE, Køber L, Kosiborod MN, Martinez FA et al. DAPA-HF Trial Committees and Investigators Dapagliflozin in patients with heart failure and reduced ejection fraction. N Engl J Med. 2019. Nov 21;381(21):1995-2008.
32. Heerspink HJL, Stefánsson BV, Correa-Rotter R, Chertow GM, Greene T, Hou FF, Mann JFE, McMurray JJV, Lindberg M, Rossing P, Sjöström CD, Toto RD, Langkilde AM, Wheeler DC; DAPA-CKD Trial Committees and Investigators. Dapagliflozin in Patients with Chronic Kidney Disease. N Engl J Med. 2020 Oct 8;383(15):1436-1446. doi: 10.1056/NEJMoa2024816. Epub 2020 Sep 24. PMID: 32970396.
33. Vilsbøll T, Bain SC, Leiter LA, et al. Semaglutide, reduction in glycated haemoglobin and the risk of diabetic retinopathy. *Diabetes Obes Metab*. 2018;20(4):889–897.
34. Lingvay I, Desouza CV, Lalic KS et al. A 25 week randomized controlled trial of semaglutide once daily versus liraglutide and placebo in patients with type 2 diabetes suboptimally controlled on diet and exercise with or without metformin. Diabetes Care. 2018;41(9): 1926-1937.
35. European Medicines Agency. Assessment Report Sotagliflozin. https://www.ema.europa.eu/en/documents/assessment-report/zynquista-epar-public-assessment-report_en.pdf *(accessed 11th November 2021)*
36. Silverii GA, Decembrini L, Monami M, Mannucci E. Fournier's gangrene and sodium glucose co-transporter 2 inhibitors: A metanalysis of randomized controlled trials. Diabetes Obes Metab. 2019; 1-4.
37. Garg SK, Peters AL, Buse JB, Danne T. Strategy for mitigating DKA risk in patients with Type 1 diabetes on adjunctive treatment with SGLT-2 inhibitors: A STICH Protocol. Diabetes Technol Ther. 2018;20(9):571-575.
38. Peters AL, Buschur EO, Buse JB, Cohan P, Diner JC, and Hirsch IB. Euglycemic Diabetic Ketoacidosis: A Potential Complication of Treatment With Sodium-Glucose Cotransporter 2 Inhibition. Diabetes Care. 2015;38(9):1687-93.
39. Agiostratidou G, Anhalt H, Ball D, Blonde L, Gourgari E, Harriman KN, Kowalski AJ, Madden P, McAuliffe-Fogarty AH, McElwee-Malloy M, Peters A, Raman S, Reifschneider K, Rubin K, Weinzimer SA. Standardizing Clinically Meaningful Outcome Measures Beyond HbA(1c) for Type 1 Diabetes: A Consensus Report of the American Association

of Clinical Endocrinologists, the American Association of Diabetes Educators, the American Diabetes Association, the Endocrine Society, JDRF International, The Leona M. and Harry B. Helmsley Charitable Trust, the Pediatric Endocrine Society, and the T1D Exchange. Diabetes Care. 2017 Dec;40(12):1622-1630.

16. APPENDICES

16.1 Appendix 1 – Risk

<table>
<tr><td colspan="5">Risks associated with trial interventions
☐ LOW ≡ Comparable to the risk of standard medical care
☒ MODERATE ≡ Somewhat higher than the risk of standard medical care
☐ HIGH ≡ Markedly higher than the risk of standard medical care</td></tr>
<tr><td colspan="5">Justification: Briefly justify the risk category selected and your conclusions below (where the table is completed in detail the detail need not be repeated, however a summary should be given):
An IDMC will be convened to monitor all SAEs.
Risks and mitigations associated with the intervention are outlined in more detail in the Protocol section 2.1.
Co-Sponsors will also carry out a detailed risk assessment of all aspects of the study as part of the approval process (SOP 04.013)</td></tr>
<tr><td colspan="2">What are the key risks related to therapeutic interventions you plan to monitor in this trial?</td><td colspan="3">How will these risks be minimised?</td></tr>
<tr><td>IMP/Intervention</td><td>Body system/Hazard</td><td>Activity</td><td>Frequency</td><td>Comments</td></tr>
<tr><td>S/C administration of semaglutide
Oral admistration of dapagliflozin</td><td>Immune: Hypersensitivity/ana-phylactic reactions</td><td>Patients with known hypersensitivity to any GLP-1 agonist or SGLT-2 inhibitor have a contra-indication to the IMP according to the SmPC will not be recruited to the study</td><td>Uncommon/ Rare</td><td></td></tr>
<tr><td>PO administration of dapagliflozin</td><td>Ketosis and/or Diabetic Ketoacidosis (DKA)</td><td>All participants will have access to blood or urine ketone monitoring equipment. An emergency ketosis management plan (STICH Protocol – see above) will be discussed with all participants. Patient alert cards will be provided to all participants. Potential participants with recent ketoacidosis will be</td><td>Common</td><td>“and” was an error – intention was for “either” blood or urine ketones between visits but “both” to be measured for visits. This is compatible with the eCRF, the diaries and the rest of the protocol</td></tr>
</table>

		excluded from the trial (section 6.2)		
S/C administration of semaglutide (+/-) PO administration of dapagliflozin in addition to insulin therapy	Increased risk of hypoglycaemia	Participant awareness of risk and frequent testing of capillary blood glucose. Patients with pre-existing frequent episodes of hypoglycaemia or problems with hypoglycaemia awareness will be excluded from trial participation (section 6.2)	Common	
Outline any other processes that have been put in place to mitigate risks to participant safety (e.g. IDMC, independent data review, etc.)<br>See above				

16.2 Appendix 2: Contraception

In line with recommendations related to contraception and pregnancy testing in clinical trials from the Clinical Trial Facilitation Group (CTFG), for women of childbearing potential* (WOCBP) participating in TTT1, methods of contraception considered as highly effective are defined as those that can achieve a failure rate of less than 1% per year when used consistently and correctly.

Such methods include:

- combined (oestrogen and progestogen containing) hormonal contraception associated with inhibition of ovulation[1]
    - oral
    - intravaginal
    - transdermal
- progestogen-only hormonal contraception associated with inhibition of ovulation[1]
    - oral
    - injectable
    - implantable[2]
- intrauterine device (IUD) [2]
- intrauterine hormone-releasing system (IUS)[2]
- bilateral tubal occlusion[2]
- vasectomised partner[2,3]
- true sexual abstinence[4]

Footnotes

1. An interaction between dapagliflozin and semaglutide with contraception is not anticipated.
2. Contraception methods that in the context of this guidance are considered to have low user dependency.
3. Vasectomised partner is a highly effective birth control method provided that partner is the sole sexual partner of the WOCBP trial participant and that the vasectomised partner has received medical assessment of the surgical success.
4. In the context of this guidance true sexual abstinence refers to: When this is in line with the preferred and usual lifestyle of the patient. Periodic abstinence (e.g. calendar, ovulation, symptothermal, post-ovulation methods), declaration of abstinence for the duration of a trial, and withdrawal are not acceptable methods of contraception.
The reliability of sexual abstinence needs to be evaluated in relation to the duration of the clinical trial and the preferred and usual lifestyle of the subject.

*Child bearing potential = experienced menarche; AND not undergone successful surgical sterilisation (hysterectomy, bilateral tubal ligation, or bilateral oophorectomy); AND not post-menopausal i.e. amenorrhea for ≥12 consecutive months (without another medical cause)

Source: Recommendations related to contraception and pregnancy testing in clinical trials. Clinical Trial Facilitation Group (CTFG), 15th September 2014.
See: https://www.hma.eu/fileadmin/dateien/Human_Medicines/01-About_HMA/Working_Groups/CTFG/2014_09_HMA_CTFG_Contraception.pdf (Accessed 21st January 2020)

16.3 Appendix 3

STICH Protocol *(also provided in Period 2 Participant Diary)*

As explained above, participants in this trial randomised to dapagliflozin are at an increased risk of ketosis and ketoacidosis. All participants will have access to a ketone meter as standard of care. It will be ascertained that each participant has access to a ketone meter; if there are any issues with their current ketone meter it will be replaced as per standard of care. There are mechanisms designed to mitigate the risk of early ketosis developing further into diabetic ketoacidosis. In this trial, we will be using a protocol called STICH (37). This is a mnemonic designed to act as a reminder for participants as to what to do in the event of elevated ketones following exposure to an SGLT-2 drug such as dapagliflozin:

1. STop dapagliflozin or placebo temporarily
2. Insulin – take a bolus of rapid acting insulin
3. Carbohydrate – take approximately 30g of carbohydrate
4. Hydrate – make sure you are taking sips of water aiming to maintain hydration.

Further guidance will be provided in both the Patient Diaries supplied to all participants and in the Patient Alert Cards that participants will be asked to carry at all times. All participants will also be advised that in the event of acute intercurrent illness or hospitalisation, dapagliflozin or placebo should be temporarily stopped. This is because intercurrent illness may additionally increase the risk of ketosis. Participants will likely already be familiar with this concept of “sick-day rules” as this is a key component of basic education for people with type 1 diabetes in clinical practice. These rules will however be re-iterated to participants.

More detailed guidance on actions participants should take for any given level of ketosis will be provided during face-to-face and telephone contact during the trial. In addition, however, the following written protocol will be provided to participants to advise them explicitly of the safest and most appropriate course of action to be taken in any given circumstance. This will take the form of a traffic light system to aid understanding and compliance.

1. Ketones 0.6 mmol/L or less: Continue - no need for additional bolus insulin.
2. Ketones 0.6 – 1.4 mmol/L: consider taking additional fast acting insulin. Follow sick day rules and monitor ketones 2 hourly until they are 0.6 mmol/L or less.
3. Ketones 1.5 – 2.9 mmol/L – Take 10% of your total daily dose of insulin (this is found by adding up all the long acting and short acting insulin for the previous day - only take a maximum of 10 units) as a bolus of fast acting insulin. Keep hydrated - aim for 100ml water per hour. Re-test your glucose and ketones in 2 hours. If not falling contact the study team during office hours. If you need urgent advice at other times please contact NHS 24 (telephone number 111) or attend your nearest Accident & Emergency Department.
4. Ketones above 3 mmol/L – Take 20% of your total daily insulin as fast acting insulin (Maximum 10 units). Hydrate with water, aim for 500 ml per hour. Attend A&E – High risk of DKA

If you develop DKA or are unwell it is important that you let the TTT1 nurse or doctor know about this as soon as you can. By following this protocol, the risk of ketosis and developing ketoacidosis will be mitigated.

16.4 Appendix 4: Amendment History

**Amendment No.**	**Protocol version no.**	**Date issued**	**Author(s) of changes**	**Details of changes made**
AM02	2.0	Ethics App – 23 Nov 2020		Protocol Changes: Weight loss mitigation, 19 Risk Mitigation and visit activity corrections
AM03	2.1	Ethics App – 10th Feb 2021		Correction of Summary of changes for previous AM02.<br>Corrected pregnancy testing schedule.
AM06	3.0	Ethics App – 16th Feb 2022		Definition of AESIs narrowed to include only ketosis and level 2 hypoglycaemia; lowering eGFR inclusion criterion; correction of DSQOLS to licensed version.
AM07	4.0			Procedure for recruitment at other sites within NHS GGC described; procedures for rescreening of participants experiencing delay between the screening visit and Visit 1; updates; correction of errors

List details of all protocol amendments here whenever a new version of the protocol is produced. Protocol amendments must be submitted to the Sponsor for approval prior to submission to the REC committee or MHRA.

16.5 Appendix 5: PAID Questionnaire

Problem Areas In Diabetes (PAID) scale

Instructions: Which of the following diabetes issues are **currently** a problem for you? Tick the box that gives the best answer for you. Please provide an answer for each question.

		Not a problem	Minor problem	Moderate problem	Somewhat serious problem	Serious problem
1	Not having clear and concrete goals for your diabetes care?	☐0	☐1	☐2	☐3	☐4
2	Feeling discouraged with your diabetes treatment plan?	☐0	☐1	☐2	☐3	☐4
3	Feeling scared when you think about living with diabetes?	☐0	☐1	☐2	☐3	☐4
4	Uncomfortable social situations related to your diabetes care (e.g. people telling you what to eat)?	☐0	☐1	☐2	☐3	☐4
5	Feelings of deprivation regarding food and meals?	☐0	☐1	☐2	☐3	☐4
6	Feeling depressed when you think about living with diabetes?	☐0	☐1	☐2	☐3	☐4
7	Not knowing if your mood or feelings are related to your diabetes?	☐0	☐1	☐2	☐3	☐4
8	Feeling overwhelmed by your diabetes?	☐0	☐1	☐2	☐3	☐4
9	Worrying about low blood glucose reactions?	☐0	☐1	☐2	☐3	☐4
10	Feeling angry when you think about living with diabetes?	☐0	☐1	☐2	☐3	☐4
11	Feeling constantly concerned about food and eating?	☐0	☐1	☐2	☐3	☐4
12	Worrying about the future and the possibility of serious complications?	☐0	☐1	☐2	☐3	☐4
13	Feelings of guilt or anxiety when you get off track with your diabetes management?	☐0	☐1	☐2	☐3	☐4
14	Not 'accepting' your diabetes?	☐0	☐1	☐2	☐3	☐4
15	Feeling unsatisfied with your diabetes physician?	☐0	☐1	☐2	☐3	☐4
16	Feeling that diabetes is taking up too much of your mental and physical energy every day?	☐0	☐1	☐2	☐3	☐4
17	Feeling alone with your diabetes?	☐0	☐1	☐2	☐3	☐4
18	Feeling that your friends and family are not supportive of your diabetes management efforts?	☐0	☐1	☐2	☐3	☐4
19	Coping with complications of diabetes?	☐0	☐1	☐2	☐3	☐4
20	Feeling 'burned out' by the constant effort needed to manage diabetes?	☐0	☐1	☐2	☐3	☐4

© Joslin Diabetes Center, 1999 (www.joslin.org). All rights reserved. The copyright holder/developer has given permission for the questionnaire to be reproduced in this toolkit. Readers of the toolkit are permitted to reproduce the questionnaire for clinical use and non-commercial research purposes. Readers of the toolkit are not permitted to use the questionnaire for commercial research purposes and must seek permission from the copyright holder/developer to do so.

16.6 Appendix 6: DSQOLS Questionnaire

Diabetes specific quality of life scale (DSQOLS)

Date____________ Name____ DOB______________

Please tick the appropriate box for each question –

Which burdens and restrictions from diabetes and its treatment have you experienced during the last four weeks?…	This statement meets my point of view…					
	5. Perfectly	4. Quite good	3. A little	2. Rather not	1. Hardly	0. Not at all
1. It bothers me that I have to measure my blood glucose so often	☐	☐	☐	☐	☐	☐
2. It burdens me that I always have to think about my nutrition.	☐	☐	☐	☐	☐	☐
3. I suffer from pain because of diabetes.	☐	☐	☐	☐	☐	☐
4. Because of diabetes the relationship to my partner has become worse.	☐	☐	☐	☐	☐	☐
5. I am worried about the fact that my life could be shorter because of diabetes.	☐	☐	☐	☐	☐	☐
6. I have the impression that I am less attractive for others because of diabetes.	☐	☐	☐	☐	☐	☐
7. Because of diabetes I feel sad or depressed	☐	☐	☐	☐	☐	☐
8. I am worried about my future health	☐	☐	☐	☐	☐	☐
9. It is a burden for me how other people react to my diabetes	☐	☐	☐	☐	☐	☐
10. I feel nervous and restless when I think about episodes of low blood sugar						
11. I feel tired and exhausted	☐	☐	☐	☐	☐	☐
12. It bothers me that I have to spend so much time on my diabetes treatment	☐	☐	☐	☐	☐	☐
13. Because of diabetes traveling is complicated and troublesome	☐	☐	☐	☐	☐	☐
14. I get anxious and nervous when I think about the dangers of episodes of low blood glucose	☐	☐	☐	☐	☐	☐

	5. Perfectly	4. Quite good	3. A little	2. Rather not	1. Hardly	0. Not at all
15. Diabetes prevents me from spontaneous physical activities	☐	☐	☐	☐	☐	☐
16. I suffer from frequent urination	☐	☐	☐	☐	☐	☐
17. Because of high blood glucose values after a meal I often feel unwell or less efficient	☐	☐	☐	☐	☐	☐
18. Because of diabetes I feel anxious or threatened	☐	☐	☐	☐	☐	☐
19. I suffer from thirst or having a dry mouth	☐	☐	☐	☐	☐	☐
20. Other people find it hard to understand my problems with diabetes treatment	☐	☐	☐	☐	☐	☐
21. I feel physically unwell	☐	☐	☐	☐	☐	☐
22. I unnecessarily worry too much about episodes of low blood glucose	☐	☐	☐	☐	☐	☐
23. I have to give up good-tasting foods	☐	☐	☐	☐	☐	☐
24. I cannot eat as much as I want of certain foods	☐	☐	☐	☐	☐	☐
25. Because of diabetes I often have physical complaints	☐	☐	☐	☐	☐	☐
26. Diabetes interferes with my future plans	☐	☐	☐	☐	☐	☐
27. I get an uncomfortable feeling when I think about the dangers of a severe episode of low blood glucose	☐	☐	☐	☐	☐	☐
28. Because of diabetes I have less contact with friends or acquaintances	☐	☐	☐	☐	☐	☐
29. I wish I could eat more the way I want to, without having to plan everything beforehand	☐	☐	☐	☐	☐	☐
30. I suffer from frequent infections, itching or skin problems	☐	☐	☐	☐	☐	☐
31. I am concerned about getting into embarrassing situations because of episodes of	☐	☐	☐	☐	☐	☐

	5. Perfectly	4. Quite good	3. A little	2. Rather not	1. Hardly	0. Not at all
low blood glucose						
32. I feel weak or lazy	☐	☐	☐	☐	☐	☐
33. I am dissatisfied with the amount of time I have to spend for medical consultations	☐	☐	☐	☐	☐	☐
34. Because of my diabetes I cannot spend my free time the way I would like	☐	☐	☐	☐	☐	☐
35. I feel like a disabled person	☐	☐	☐	☐	☐	☐
36. I am worried that I could sustain physical injury in the event of a severe episode of low blood glucose	☐	☐	☐	☐	☐	☐
37. It bothers me that I cannot eat like other people	☐	☐	☐	☐	☐	☐
38. I am often worried about the long-term	☐	☐	☐	☐	☐	☐
39. Because of diabetes other people treat me like a "sick person"	☐	☐	☐	☐	☐	☐
40. Because of diabetes I cannot pursue my hobbies as I wish	☐	☐	☐	☐	☐	☐
41. I have to frequently think about diabetes and its consequences	☐	☐	☐	☐	☐	☐
42. It bothers me that I cannot eat as spontaneously as people who do not have diabetes	☐	☐	☐	☐	☐	☐
43. It upsets my stomach when I think about the dangers of severe episodes of low blood glucose	☐	☐	☐	☐	☐	☐
44. Diabetes constantly causes problems while dealing with other people	☐	☐	☐	☐	☐	☐

With respect to diabetes treatment it is very important to me that, resp. it would be my goal that …	5. Very Important	4. Quite Important	3. Rather Important	2. Rather Unimportant	1. Quite Unimportant	0. Totally Unimportant
45. my blood glucose values are always below 140 mg%	☐	☐	☐	☐	☐	☐
46. my blood glucose values are as constant as possible	☐	☐	☐	☐	☐	☐
47. I can plan my leisure flexibly	☐	☐	☐	☐	☐	☐
48. I can avoid even mild hypoglycaemia	☐	☐	☐	☐	☐	☐
49. I can avoid late complications of diabetes in any case, resp. avoid their progress.	☐	☐	☐	☐	☐	☐
50. I can eat flexibly in the way that I can choose the kind, amount and time of meals	☐	☐	☐	☐	☐	☐
51. I am as physically fit as possible.	☐	☐	☐	☐	☐	☐
52. I can in any case avoid severe hypoglycaemia with loss of consciousness	☐	☐	☐	☐	☐	☐
53. I have to measure my blood glucose as rarely as possible.	☐	☐	☐	☐	☐	☐
54. Other people don't find out that I have diabetes.	☐	☐	☐	☐	☐	☐

How satisfied have you been during the last weeks with …	0. Very Satisfied	1. Quite Satisfied	2. Rather Satisfied	3. Rather dissatisfied	4. Quite dissatisfied	5. Totally dissatisfied
55. the level of your blood glucose values?	☐	☐	☐	☐	☐	☐
56. The stability of your blood glucose values?	☐	☐	☐	☐	☐	☐
57. The flexibility of planning your leisure?	☐	☐	☐	☐	☐	☐
58. The frequency of mild hypoglycaemia?	☐	☐	☐	☐	☐	☐
59. your assurance of being protected against late complications, resp. of being able to avoid their progress?	☐	☐	☐	☐	☐	☐
60. The flexibility of your diet?	☐	☐	☐	☐	☐	☐
61. your physical fitness?	☐	☐	☐	☐	☐	☐
62. your assurance of being protected against severe hypoglycaemia with loss of consciousness?	☐	☐	☐	☐	☐	☐
63. the frequency of your blood glucose self-monitoring?	☐	☐	☐	☐	☐	☐
64. The extent to what other people are informed about your diabetes?	☐	☐	☐	☐	☐	☐

FULL/LONG TITLE OF THE TRIAL
Triple Therapy for Type 1 Diabetes with Insulin, Semaglutide and Dapagliflozin (TTT1) - UK

SHORT STUDY TITLE / ACRONYM
TTT1 (UK)

PROTOCOL VERSION NUMBER AND DATE
Version 6.0 15th Feb 2024

RESEARCH REFERENCE NUMBERS

**IRAS Number:**	254431
**EudraCT Number:**	2018-004120-11
**ISRCTN Number / Clinical trials.gov Number:**	NCT03899402
**SPONSOR Number:**	GN17DI706
**FUNDER Number:**	3-SRA-2019-669-M-B

SIGNATURE PAGE

The undersigned confirm that the following protocol has been agreed and accepted and that the Chief Investigator agrees to conduct the trial in compliance with the approved protocol and will adhere to the principles outlined in the Medicines for Human Use (Clinical Trials) Regulations 2004 (SI 2004/1031), amended regulations (SI 2006/1928) and any subsequent amendments of the clinical trial regulations, Good Clinical Practice (GCP) guidelines, the Sponsor's (Standard Operating Procedures (SOPs), and other regulatory requirements as amended.
I agree to ensure that the confidential information contained in this document will not be used for any other purpose other than the evaluation or conduct of the clinical investigation without the prior written consent of the Sponsor
I also confirm that I will make the findings of the study publicly available through publication or other dissemination tools without any unnecessary delay and that an honest accurate and transparent account of the study will be given; and that any discrepancies from the study as planned in this protocol will be explained.

For and on behalf of the Study Co-Sponsor (University of Glasgow):
Signature: .... Date: ....../....../......
Name (please print): ....
Position: ....

For and on behalf of the Study Co-Sponsor (NHS Greater Glasgow and Clyde):
Signature: .... Date: ....../....../......
Name (please print): ....
Position: ....

UK Chief Investigator:
Signature: .... Date: ....../....../......
Name: (please print): John Petrie ....
Position: Professor of Diabetes...

Statistician:
Signature: .... Date: ....../....../......
Name: (please print): ....

Position: ....

KEY TRIAL CONTACTS

UK Chief Investigator	Professor John R Petrie<br>Professor of Diabetes<br>Director, Robertson Centre for Biostatistics<br>Director, Glasgow Clinical Trials Unit<br>School of Health and Wellbeing<br>College of Medical, Veterinary and Life Sciences<br>University of Glasgow<br>Clarice Pears Building, Level 5<br>90 Byres Road<br>Glasgow<br>G12 8TB<br>Secretary +44(0)141 330 4744<br>john.petrie@glasgow.ac.uk
UK Co- Investigator	Dr James Boyle<br>Consultant Diabetologist<br>Glasgow Royal Infirmary<br>84 Castle Street, Glasgow, G4 0SF<br>Tel: : +44(0)141 211 5996<br>james.boyle@glasgow.ac.uk
Trial Co-ordination	Miss Lisa Jolly<br>Research and Innovation<br>NHS Greater Glasgow & Clyde<br>Ward 11, Dykebar Hospital<br>Grahamston Road<br>Paisley, PA2 7DE<br>Lisa.Jolly@ggc.scot.nhs.uk
Co-Sponsor	NHS Greater Glasgow & Clyde<br><br>Contact: Dr Maureen Travers<br>Research and Development Management Office<br>Research and Innovation<br>NHS Greater Glasgow & Clyde<br>Ward 11, Dykebar Hospital<br>Grahamston Road<br>Paisley, PA2 7DE<br>Tel: +44 (0) 0141 314 4012<br>maureen.travers@ggc.scot.nhs.uk
Co-sponsor	University of Glasgow<br><br>Contact: Dr Debra Stuart<br>College of Medical, Veterinary & Life Sciences<br>Room 327 Wolfson Medical School Building<br>University Avenue<br>Glasgow G12 8QQ

	Tel: +44 (0)141 330 4539<br>Debra.Stuart@glasgow.ac.uk
Funder	JDRF<br>26 Broadway, 15th floor, New York, NY 10004, USA
Clinical Trials Unit	Glasgow Clinical Trials Unit<br>Robertson Centre for Biostatistics<br>Clarice Pears Building, Level 5<br>90 Byres Road<br>Glasgow<br>G12 8TB<br>Tel: 0141 330 4744<br>liz.anderson@glasgow.ac.uk
Statisticians	Professor Alex McConnachie<br>Robertson Centre for Biostatistics<br>Clarice Pears Building, Level 5<br>90 Byres Road<br>Glasgow<br>G12 8TB<br>Telephone: 0141 330 4744<br><br>Ms Nicola Greenlaw<br>Robertson Centre for Biostatistics<br>Clarice Pears Building, Level 5<br>90 Byres Road<br>Glasgow<br>G12 8TB
Trials pharmacist	Dr Elizabeth Douglas<br>Clinical Trials Pharmacist<br>Clinical Research and Development<br>Ward 11, Dykebar Hospital,<br>Grahamston Road | Paisley PA2 7DE<br>Tel: 0141 314 4073<br>E-mail: elizabeth.douglas@ggc.scot.nhs.uk

US Principal Investigator	Dr Paresh Dandona<br>Director, Diabetes-Endocrinology Center of Western NY<br>Chief of Endocrinology, Diabetes, Endocrinology and Metabolism, SUNY at Buffalo<br>1000 Youngs Road, Suite 105<br>Williamsville NY 14221<br>Telephone: +1(716) 535-1850<br>Fax: +1(716)-639-1200

	pdandona@kaleidahealth.org
US Co- Investigator	Dr Husam Ghanim, PhD.<br>Director, Diabetes and Endocrinology Research Lab<br>Research Associate Professor,<br>School of Medicine, State University of NY at Buffalo<br>Clinical and Translational Research Center<br>875 Ellicott St<br>Buffalo, NY 14203<br>Tel: (+1)716-881-8924<br>ghanim@buffalo.edu
Key Protocol Contributors	Professor John Petrie<br>john.petrie@glasgow.acuk<br><br>Dr Paresh Dandona<br>pdandona@kaleidahealth.org<br><br>Husam Ghanim, PhD.<br>ghanim@buffalo.edu<br><br>Dr James Boyle<br>james.boyle@glasgow.ac.uk<br><br>Dr Robert Lindsay<br>robert.lindsay@glasgow.ac.uk<br><br>Dr Joseph Timmons<br>joseph.timmons@glasgow.ac.uk<br><br>Professor Alex McConnachie<br>Alex.McConnachie@glasgow.ac.uk

TRIAL SUMMARY

Trial Title	Triple Therapy for Type 1 Diabetes with Insulin, Semaglutide, and Dapagliflozin (UK)	
Internal ref. no. (or short title)	TTT1	
Clinical Phase	Phase 3	
Trial Design	Two six-month periods: (i) open-label randomised (2:1) parallel group semaglutide vs standard of care; (ii) additional double-blind, randomised (1:1) parallel group, dapagliflozin vs placebo for those participants initially randomised to semaglutide.<br>Regardless of each randomisation, ongoing standard of care (insulin therapy) will be provided for all participants throughout the study.	
Trial Participants	Adults with type 1 diabetes for at least one year with HbA1c 7.5-11.0% on stable multiple daily injections (MDI) (four or more) of insulin or continuous subcutaneous insulin infusion (CSII) for the previous three months.	
Planned Sample Size	82 (41 in UK)	
Treatment duration	Approximately 1 year	
Follow up duration	Approximately 1 year	
Planned Trial Period	Approximately 5 years	
	Objectives	Outcome Measures
Primary	To assess in people with type 1 diabetes whether triple therapy (dapagliflozin, semaglutide and insulin) reduces HbA1c in comparison with dual therapy (placebo, semaglutide, and insulin – period 2).	HbA1c
Secondary	To assess in people with type 1 diabetes whether triple therapy (dapagliflozin, semaglutide and insulin) reduces HbA1c in comparison with standard therapy (insulin alone)	HbA1c
	To assess in people with type 1 diabetes whether dual therapy (semaglutide and insulin - period 1) reduces HbA1c in comparison with standard therapy (insulin alone)	HbA1c
	To assess in people with type 1 diabetes whether triple therapy (dapagliflozin, semaglutide and insulin) improves glucose control	- Mean fasting blood glucose<br><br>- Mean blood glucose*<br>- Percentage time in range

	as assessed by CGM, insulin requirement, body weight, blood pressure and quality of life in comparison with (a) dual therapy (placebo, semaglutide, and insulin – period 2) and (b) standard therapy (insulin alone)	- Glycaemic variability*<br>- Percentage time in hyperglycaemia Level 1 (10.1-13.9 mmol/)*<br>- Percentage time in hyperglycaemia Level 2 (>13.9 mmol/L)*<br>-Percentage time in hypoglycaemia Level 1 (3.0 – 3.8 mmol/L)*<br>** CGM (by masked Dexcom G6)*<br><br>Insulin requirement<br>Body weight<br>Systolic/ diastolic blood pressure<br>Number of antihypertensive agents<br>Diabetes Specific Quality of Life Scale (DSQOLS)<br>Problem Areas In Diabetes (PAID) questionnaire
Safety Endpoint 1	a) Triple therapy (dapagliflozin, semaglutide and insulin) in comparison with dual therapy (placebo, semaglutide, insulin – period 2)<br><br>b) Dual therapy (semaglutide and insulin - period 1) in comparison with standard therapy (insulin only)	Difference between treatment arms in:<br>(i) Rate of Level 2 (<3.0 mmol/L) hypoglycaemic events (by Dexcom G6 CGM)<br>(ii) Rate of Level 3 hypoglycaemic events (severe event with altered mental and/or physical status requiring assistance)
Safety Endpoint 2	a) Triple therapy (dapagliflozin, semaglutide and insulin) in comparison with dual therapy (placebo, semaglutide, and insulin – period 2)<br><br>b) Dual therapy (semaglutide and insulin –period 1) in comparison with standard therapy (insulin only)	Difference between treatment arms in:<br>Rate of diabetic ketoacidosis (serum ketones or urinary ketones above normal limits AND serum bicarbonate < 15 mmol/L OR blood pH < 7.3)
Investigational Medicinal Product(s)	Semaglutide<br>Dapagliflozin<br>Placebo matching dapagliflozin	
Non-investigational Medicinal Product(s)	Insulin	
Formulation, Dose, Route of Administration	Semaglutide (subcutaneous injection, once weekly) – open label by pen device (0.25 mg/ 0.5 mg/ 1.0 mg)<br>Dapagliflozin 5 mg (oral) – one then two capsules daily (up-titration)<br>Placebo matching dapagliflozin	

FUNDING AND SUPPORT IN KIND

**FUNDER(S)**	**FINANCIAL AND NON FINANCIALSUPPORT GIVEN**
JDRF New York<br>26 Broadway, 15th floor, New York, NY 10004, United States	$1,440,000 (to University at Buffalo, the State University of New York, NY (SUNY Buffalo), USA; and University of Glasgow, UK)
Novo Nordisk, 800 Scudders Mill Road, Princeton Landing, NJ, Plainsboro, 08536.	$160,000 and donation of semaglutide to Glasgow site during period of supply disruption (see paragraph headed "Novo Nordisk" below)
AstraZeneca<br>1800 Concord Pike, Wilmington, DE 19803, USA	Provision of dapagliflozin and matched placebo investigational medicinal products to US site
Dexcom; Suite 6, 1 Tanfield, Edinburgh, EH3 5DA	Staff training and discounted supply of CGM sensors

ROLE OF STUDY SPONSOR AND FUNDER

NHS Greater Glasgow & Clyde and The University of Glasgow will be Co-sponsors of the UK arm of the trial. Prior to study initiation, a non-commercially funded clinical trial co-sponsorship agreement will be put in place between NHS Greater Glasgow & Clyde and The University of Glasgow. The roles and liabilities each organisation will take under The Medicines for Human Use (Clinical Trials) Regulations, 2004 SI 2001:1031 are laid out in this agreement signed by both organisations. The University of Glasgow shall be responsible for carrying out the obligations and responsibilities set out in the aforementioned agreement, and shall be deemed "sponsor" for the purposes of, Part 3 of the regulations in relation to the study. NHS Greater Glasgow & Clyde shall be responsible for carrying out the responsibilities set out in the agreement, and shall be deemed "sponsor" for the purposes of, Parts 4, 5, 6 and 7 of the Regulations in relation to the study.

The Co-Sponsors will delegate specific roles to the Chief Investigator, Glasgow CTU and other third parties. These arrangements will be clearly documented in agreements and/or the Sponsor Delegated Roles and Responsibilities Matrix.

The US arm of the trial has the same inclusion and exclusion criteria and the same pre-specified outcomes but an individualised Protocol. It will collect the same data via a common eCRF. Adverse event data from each site will be available to the trial Data and Safety Monitoring Board. The UK Co-Sponsors will have no responsibility for (and carry no liability for) the conduct of the US arm of the trial.

JDRF

The study has been funded by a grant from JDRF. JDRF does not have a designated role or responsibility in trial design, conduct, data analysis and interpretation, manuscript writing, and dissemination of results. Periodic reports (at least annual) in relation to progress of the trial will be submitted to the JDRF. Support from JDRF will be acknowledged in any publications related to the study.

Astra Zeneca

This is an investigator-initiated study. Astra Zeneca provided support by supplying investigational medicinal products (IMPs) dapagliflozin and matched placebo to the US site only. Representatives of Astra Zeneca reviewed and provided comments on drafts of the Protocol but the company does not

have a designated role or responsibility in trial design, conduct, data analysis and interpretation, manuscript writing, and dissemination of results.

Novo Nordisk
In November 2020, Novo Nordisk made up a shortfall of funding that resulted from a cut back by 10% of the main grant by the funder JDRF due to the COVID-19 pandemic. By this time, the Protocol had been finalised. The company does not have a designated role or responsibility in trial design, conduct, data analysis and interpretation, manuscript writing, or dissemination of results.

Supply shortages of semaglutide (various strengths) from 2022 onwards resulted in actual and potential disruption to participant recruitment and ongoing treatment. In response to a direct request from the UK Chief Investigator, Novo Nordisk donated supplies of semaglutide to the Glasgow site based on anticipated use during the period of disruption.

ROLES AND RESPONSIBILITIES OF TRIAL MANAGEMENT COMMITTEES/GROUPS & INDIVIDUALS

Trial Steering Committee (TSC)

The role of the TSC is to provide overall supervision of the trial and ensure that it is being conducted in accordance with the principles of GCP and the relevant regulations. The TSC will:
1. agree the trial protocol and any protocol amendments
2. ensure that conduct of the trial is fully harmonised between the two sites
3. provide advice to the investigators on all aspects of the trial

It will have an independent Chairperson and include the US and UK investigators and key protocol contributors listed above.

Decisions about continuation or termination of the trial or substantial amendments to the protocol will be the responsibility of the TSC who will advise the co-sponsors. The TSC will meet at the start of the study, and four to six monthly thereafter. The TSC will have its own Charter outlining the role and responsibilities of its members. The TSC may invite other attendees from the trial team to present or participate in discussions on particular topics. These attendees will be non-voting members.

Independent data monitoring committee (IDMC)

An IDMC will be established to include two independent medical experts (covering the domains of type 1 diabetes and metabolic disease; one of the academic clinicians will act as chair) and an independent biostatistician. The Glasgow CTU will liaise with the committee and ensure that the Committee is provided with adequate information about study progress and results.

The IDMC will have a formal Charter outlining the responsibilities of the IDMC members, Glasgow CTU and the co-sponsors. Responsibilities include:

• To protect the safety of participants recruited to the trial.
• Advising the TSC and co-sponsors if it is safe and appropriate to continue with the study
• Examining information provided by the Glasgow CTU on study recruitment, adverse eventsand outcomes
  - • Consider the need for additional unscheduled reviews of study data.
• Providing reports for the Project Office to forward to the TSC, ethics committees, regulatory bodies, study co-sponsors, and the BHF.

The IDMC may recommend to the TSC to terminate the study or specific aspects of the study if clear harm is observed with one treatment option compared to standard of care or placebo. Specific safety endpoints that will be reviewed include rates of hypoglycaemia and ketosis/ ketoacidosis.

The IDMC will meet approximately every six months.

Study Management Group (SMG)

Trial activity at the UK/ Glasgow site will be coordinated from NHS Greater Glasgow & Clyde (NHS GG&C) by the TTT1 Study Management Group (SMG). The SMG will consist of the UK chief investigator, co-principal investigator other co-applicants, project manager and representatives from the Glasgow CTU, NHS GG&C and The University of Glasgow. The role of the group is to monitor all

aspects of the conduct and progress of the trial in the UK, ensuring adherence to the protocol, and take appropriate action to safeguard participants and the quality of the trial itself.

Joint Study Management Group

Coordination of trial activity between the UK/ Glasgow and the US/ Buffalo site will be facilitated by the Joint TTT1 Study Management Group. This will consist of the US and UK chief investigators, co-principal investigators, other co-applicants, study coordinators, project managers and governance/ administrative representatives as appropriate from State University of New York (SUNY), the Glasgow CTU, NHS GG&C and The University of Glasgow. It will meet approximately every 4-6 weeks. The role of the group is to coordinate all aspects of the conduct and progress of the trial between the US and UK sites, ensuring protocol adherence, and taking appropriate action to safeguard participants and the quality of the trial itself.

Protocol contributors

The protocol has been developed by a group with extensive clinical and research experience in type 1 diabetes and clinical trials including Professor John Petrie and Professor Paresh Dandona. Professor Alex McConnachie has research expertise in design, conduct, analysis and interpretation of clinical trials and biostatistical methods.

KEY WORDS:

Adjunct therapy
Dapagliflozin
Glycated haemoglobin (HbA1c)
Semaglutide
Type 1 diabetes

LIST of CONTENTS

RESEARCH REFERENCE NUMBERS 2
SIGNATURE PAGE 3
KEY TRIAL CONTACTS 4
LIST of CONTENTS..14
LIST OF ABBREVIATIONS...15
TRIAL FLOW CHART..17
1 BACKGROUND 20
2 RATIONALE..21
3 OBJECTIVES AND OUTCOME MEASURES/ ENDPOINTS.......................................25
4 TRIAL DESIGN 28
5 STUDY SETTING 28
6 ELIGIBILITY CRITERIA 28
7 TRIAL PROCEDURES...30
8 TRIAL MEDICATION………………………………………………………………..58
9 PHARMACOVIGILANCE……………………………………………………………63
10 STATISTICS AND DATA ANALYSIS...71
11 DATA HANDLING 73
12 MONITORING, AUDIT & INSPECTION 74
13 ETHICAL AND REGULATORY CONSIDERATIONS..75
14 DISSEMINATION POLICY……………………………………………………………77
15 REFERENCES…………………………………………………………………….78
16. APPENDICES...83

LIST OF ABBREVIATIONS

ACS	Acute Coronary Syndrome
AUC	Area Under the Curve
AE	Adverse Event
AR	Adverse Reaction
CA	Competent Authority
CABG	Coronary Artery Bypass Graft
CGM	Continuous Glucose Monitoring
CHI	Community Health Index
CI	Chief Investigator
CRO	Contract Research Organisation
CRP	C-Reactive Protein
CSII	Continuous Subcutaneous Insulin Infusion
CTA	Clinical Trial Authorisation
CTU	Clinical Trials Unit
CTIMP	Clinical Trial of Investigational Medicinal Product
CV	Cardiovascular
CVA	Cerebrovascular Accident
DKA	Diabetic Ketoacidosis
DSUR	Development Safety Update Report
EC	European Commission
ECG	Electrocardiogram
eCRF	Electronic Case Report Form
EDTA	Ethylenediaminetetraacetic acid
eGFR	Estimated Glomerular Filtration Rate
EMEA	European Medicines Agency
EU	European Union
EUCTD	European Clinical Trials Directive
EudraCT	European Clinical Trials Database
EudraVIGILANCE	European database for Pharmacovigilance
FBC	Full Blood Count
FGM	Flash Glucose Monitoring
GCP	Good Clinical Practice
GDG	Guideline Development Group
GDPR	General Data Protection Regulation
GLP-1	Glucagon Like Peptide-1
GLP-1RA	Glucagon Like Peptide-1 Receptor Agonist
GMP	Good Manufacturing Practice
HbA1c	Glycated haemoglobin
IB	Investigator Brochure
ICF	Informed Consent Form
ICH	International Conference on Harmonisation of technical requirements for registration of pharmaceuticals for human use.
IDMC	Independent Data Monitoring Committee
IMP	Investigational Medicinal Product
IMPD	Investigational Medicinal Product Dossier

ISF	Investigator Site File
ISRCTN	International Standard Randomised Controlled Trials Number
IWRS	Interactive Web-based Randomisation System
LPLV	Last Patient Last Visit
MA	Marketing Authorisation
MDRD	Modification of Diet in Renal Disease
MODY	Maturity Onset Diabetes of the Young
MHRA	Medicines and Healthcare products Regulatory Agency
MI	Myocardial Infarction
MDI	Multiple Daily Injections
NHS GG&C	National Health Service Greater Glasgow & Clyde
NHS R&D	National Health Service Research & Development
NICE	The National Institute for Health and Care Excellence
NIMP	Non-Investigational Medicinal Product
PI	Principal Investigator
PIC	Participant Identification Centre
PIS	Participant Information Sheet
PV	Pharmacovigilance
QA	Quality Assurance
QC	Quality Control
QoL	Quality of Life
QP	Qualified Person
RCT	Randomised Control Trial
REC	Research Ethics Committee
SAE	Serious Adverse Event
SAP	Statistical Analysis Plan
SAR	Serious Adverse Reaction
SDV	Source Data Verification
SGLT2	Sodium Glucose Transporter 2
SOP	Standard Operating Procedure
SUNY	State University of New York
SmPC	Summary of Product Characteristics
SSI	Site Specific Information
SUSAR	Suspected Unexpected Serious Adverse Reaction
TIA	Transient Ischaemic Attack
TMG	Trial Management Group
TSC	Trial Steering Committee
TMF	Trial Master File
UB	University of Buffalo
ULN	Upper Limit of Normal reference range
WOCBP	Women Of Childbearing Potential

TRIAL FLOW CHART

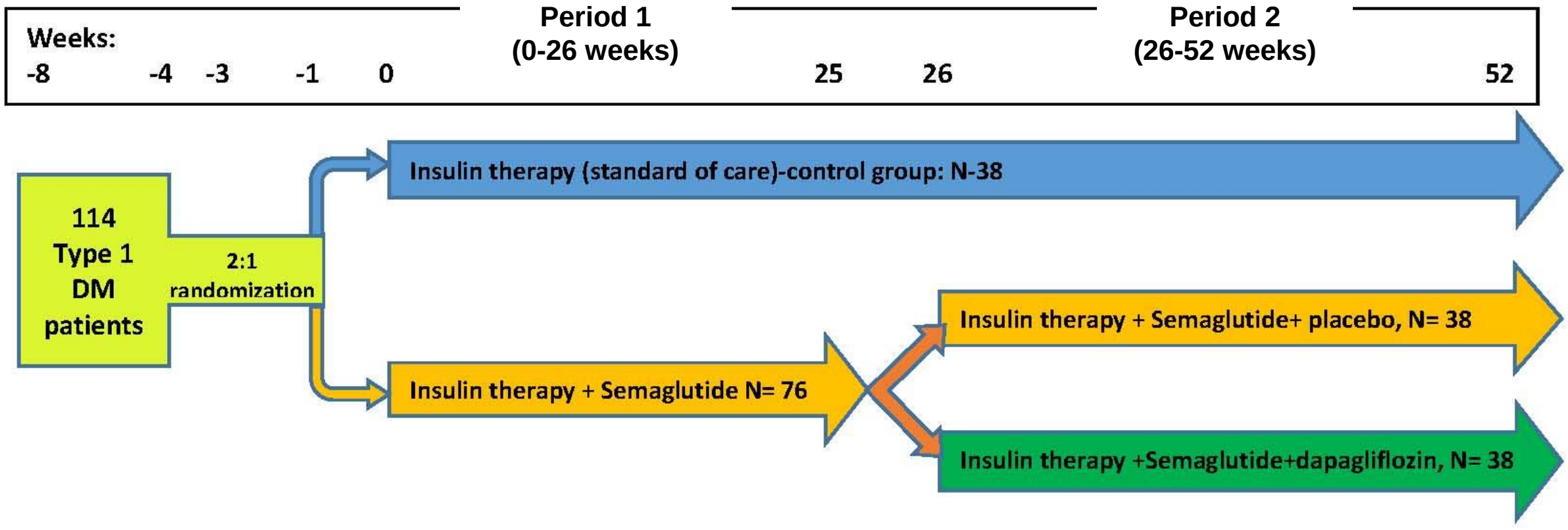

Revised Flow Chart
At this time, a revised sample size in Period 2 of around 23 evaluable participants per group for dapagliflozin vs placebo. This reduces the recruitment target for first randomisation from n=114 to n=82

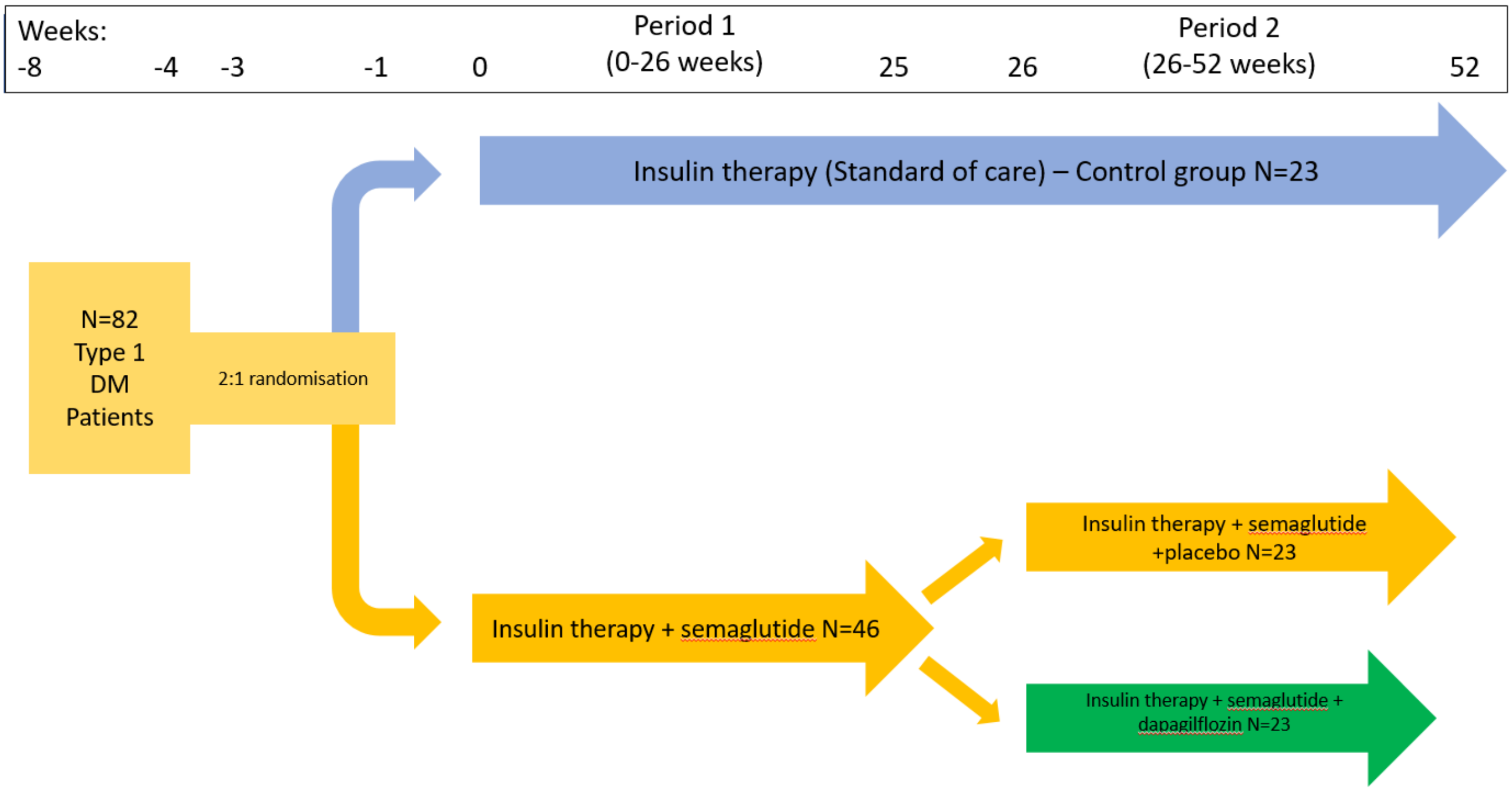
Weeks:
-8
-4
-3
-1
0
Period 1
(0-26 weeks)
25
26
Period 2
(26-52 weeks)
52
Insulin therapy (Standard of care) – Control group N=23
N=82
Type 1
DM
Patients
2:1 randomisation
Insulin therapy + semaglutide N=46
Insulin therapy + semaglutide
+placebo N=23
Insulin therapy + semaglutide +
dapagilflozin N=23

SCHEDULE OF ASSESSMENTS

**Visit #**	**-**	**1**	**2**	**3**	**4[3]**	**5[3]**	**6**	**7**	**8**	**9**	**10**	**11[3]**	**12**	**13**	**14**	**15**	**16**
**Week# (±7 days; see below)**	**-8 to -4**	**-3**	**-1**	**0**	**1**	**2**	**4**	**13**	**23**	**25**	**26**	**27**	**31**	**39**	**49**	**51**	**52**
Study Procedures	Screening	Run-in period		Randomise							Randomise						End
Informed Consent	X																
Fasting visit			X	X				X		X	X			X		X	X
History and demographics	X																
Physical	X																X
Education on hypoglycaemia and ketosis avoidance				X							X						
BP, heart rate, weight	X	X	X	X	X	X	X	X	X	X	X	X	X	X	X	X	X
Bloods (FBC, kidney and liver profiles)	X			X				X			X		X	X			X
C-peptide	X										X						X
Pregnancy test	X			X			X[1]	X[1]	X[1]		X[1]		X[1]	X[1]	X[1]	X[1]	X[1]
Dispense open-label semaglutide[2]				X		X[2]	X[2]	X[2]			X[2]			X[2]			
Titrate open-label semaglutide[2]						X[2]	X[2]	X[2]									
Dose Semaglutide (on-site if dose is due on visit day)				X	X	X											
Dispense dapagliflozin/placebo											X	X		X			
Dapagliflozin/placebo return capsule counts												X		X			X
HbA1c	X			X				X		X				X			X
Blood endpoints (including fructosamine)				X				X			X			X			X
Masked CGM fit (enter data this visit)		X							X						X		
Masked CGM read				X							X						X
Daily fingerstick glucose review		X	X	X	X	X	X	X	X	X	X	X	X	X	X	X	X
Daily urinary ketones review												X	X	X	X	X	X
Insulin dose review		X	X	X	X	X	X	X	X	X	X	X	X	X	X	X	X
Ketone bodies (blood/urine)	X	X	X	X	X	X	X	X	X	X	X	X	X	X	X	X	X
Adverse Event reporting		X	X	X	X	X	X	X	X	X	X	X	X	X	X	X	X
Meal Challenge Test			X							X						X	
24 hr Urine dispensed			X				X			X			X			X	
24 hr Urine collected				X				X			X			X			X
Quality of life questionnaires				X							X						X

N.B. Safety phone calls will be conducted at weeks 3, 5, 8, 16, 20, 29, 35, 44 and <u>at two weeks after the end of the study</u> to document any side effects or other changes. **Abbreviations:** see pages 14-15

Additional visits or phone calls may be scheduled with the verbal consent of the participant if study team believe necessary e.g. closer monitoring is appropriate (see Patient Information Sheet)

Visit dates should be within seven days of the visit time; however, a longer or shorter interval does not constitute a protocol deviation

[1]Not required in women randomised to "Insulin therapy (standard of care)

[2]According to randomisation schedule: resupply of open-label semaglutide after Visit 3 will be according to individual requirements as tolerated (see Section 8.3)

[3] Visits 4, 5 and 11 have the option of being carried out by telephone for those participants allocated to the standard care arm only.

STUDY PROTOCOL
Triple Therapy for Type 1 Diabetes with Insulin, Semaglutide, and Dapagliflozin

1 BACKGROUND

The control of glucose homeostasis in type 1 diabetes is fragile since β-cell reserve is negligible. Traditionally, insulin has been the only treatment approach available. This often leads to erratic blood glucose control given the challenge of matching exogenous insulin to mealtime glucose excursions in the face of a lack of significant endogenous insulin to compensate for variation in requirements in response to meals and activity levels. Injected insulin boluses rarely match precise insulin requirements either in terms of dose administered or bio-availability. The result often is wide fluctuations in blood glucose including episodes when it is too low (hypoglycaemia). Based on our work to date and other published reports, previous attempts to add other glucose-lowering medications to insulin therapy yielded minimal benefits on glycaemia (although metformin may have other benefits) (1-3).

Dandona et al. have shown in two preliminary retrospective studies that the addition of liraglutide, a Glucagon Like Peptide -1 receptor agonist (GLP-1RA), to insulin results in consistent improvements in glycemic control in type 1 diabetes as demonstrated by reductions in HbA1c, glycemic excursions, and insulin requirements accompanied by improvements in body weight and systolic blood pressure (4, 5). As collaborators in the present trial, they also completed the first prospective randomised controlled trial of this drug in 72 people with type 1 diabetes. The data not only confirmed improvements in glycemic control (HbA1c), body weight and systolic BP, but also demonstrated a reduction in appetite, carbohydrate intake and low-grade inflammation (6). Importantly, quality of life was also improved. Their recent studies in type 2 diabetes have revealed that systolic blood pressure reduction with GLP-1RA (exenatide) treatment in type 2 diabetes is associated with increased plasma concentrations of ANP, cGMP and cAMP along with reduced concentrations of angiotensinogen, renin and angiotensin II (7). It is possible that these mechanisms play a role in the prevention of cardiovascular complications in type 1 diabetes. There have been no trials to date of semaglutide (a weekly and potent GLP-1RA) in type 1 diabetes.

GLP-1RAs, therefore, have great potential as adjunct therapy for type 1 diabetes. However, mean HbA1c was still ≥7.0% after liraglutide treatment (6) and larger pharma-sponsored trials (ADJUNCT-1 and ADJUNCT-2) reported increased rates of hypoglycaemia (8, 9). In seeking further improvement, the advent of Sodium-GLucose coTransporter 2 (SGLT2) inhibitors is of importance since their mode of action is independent of insulin secretion. Introduced for use in type 2 diabetes, SGLT2 inhibitors are selective antagonists of the sodium-glucose transporter subtype expressed in the proximal convoluted tubules of the kidneys. They act by inhibiting renal glucose re-absorption and therefore stimulating excretion of 60-100 grams per day of glucose in urine. SGLT2 inhibitors have been studied extensively in type 2 diabetes, including the landmark cardiovascular outcome trial EMPA-REG (10), but data have only more recently begun to accumulate in type 1 (11). Short term effects on urine glucose excretion of remogliflozin and empagliflozin are similar to those seen in type 2 diabetes and these translate into improved glycaemic and other metabolic indices including HbA1c, fasting blood glucose, rates of hypoglycaemia, daily insulin dose and weight (12). Glucose-lowering efficacy is attenuated in individuals with eGFR < 45/ml/min/1.73 m^2.(13) Weight loss accompanying increased urinary glucose excretion could translate into reduced cardiovascular complications as in type 2 diabetes, but there are no data so far available to support this hypothesis. More recently, in the Phase 3 trials DEPICT-1 and DEPICT-2 Dandona et al (PI of the US arm of this trial) showed that dapagliflozin improves glycaemic control and HbA1c over six months by about 0.45% without increasing hypoglycaemia and as in other conditions reduces urinary albumin excretion (14,15,16,17). In comparison to other SGLT2 inhibitors studied for use in type 1 diabetes [sotagliflozin and empagliflozin (18, 19)] the risk of diabetic ketoacidosis was relatively modest (see below). Both dapagliflozin and sotagliflozin were approved early in 2019 by the EU Committee for Medicinal Products for Human Use (CHMP) as adjunct therapy in type 1 diabetes for individuals with BMI≥27 kg/m (20,21)2. In August 2019, dapagliflozin (5 mg/ day) was recommended by the National Institute

for Health and Care Excellence as cost-effective for this indication in NHS England (22 However, dapagliflozin did not gain approval for this indication in the USA from the FDA (23). In November 2021, the manufacturer AstraZeneca voluntarily removed this Europe- and UK-specific indication (24) explicitly “not due to any safety concern for dapagliflozin in any condition, including type 1 diabetes” (25).

2 RATIONALE

General hypothesis: adding an SGLT2 inhibitor (dapagliflozin) to a combination of insulin and semaglutide will improve glycaemic control, weight and blood pressure in adults with type 1 diabetes.
Our protocol is designed to allow an objective assessment of whether this triple therapy combination will work effectively and safely so that a majority of people with type 1 diabetes can reach a glycaemic target HbA1c of ≤7.0%. In addition, since both semaglutide and dapagliflozin inhibitors reduce blood pressure and weight, it will allow an assessment of the combined effect of GLP-1RAs and SGLT2 inhibitors on these outcomes. Since there are no previous data on the effects of once weekly semaglutide in type 1 diabetes, or the combination of semaglutide and dapagliflozin compared to standard insulin treatment, the design requires allocation of some participants to insulin therapy only (the current standard of care).

As part of the process by which this trial was awarded funding by JDRF, this research question was considered by lay members of the funding panel and deemed important, relevant and timely. We considered that use of placebo matching dapagliflozin in Period 2 of the trial was justified when dapagliflozin had full approval as adjunct therapy for type 1 diabetes in Europe (although not the USA) as it remained uncertain which adults with type 1 diabetes were suitable for this treatment. In their review, The UK National Institute for Health and Care Excellence (NICE) considered it likely that fewer than 10% of adults with type 1 diabetes would be initiated on SGLT2 inhibitor therapy over the first five years after their favourable decision. As individuals with a slightly lower BMI threshold will be included than recommended by the SmPC, trial participants will be more closely monitored for ketosis than was the case when dapagliflozin was approved for routine clinical care. Dapagliflozin (or placebo) will be administered orally once daily as per current licensed doses in type 2 diabetes (5 mg/day and 10 mg/day: the same doses as used in DEPICT-1 and DEPICT-2) (14,15,16,). We have not incorporated blinding for semaglutide in the design as the effects of the closely-related daily GLP-1RA liraglutide have been well-studied in double-blind trials in type 1 diabetes by ourselves and others, and individuals in both arms compared for the primary outcome comparison will be treated with this agent. (6, 8, 9). Semaglutide will be administered subcutaneously once weekly. The need to study combination adjunct therapy with SGLT 2 inhibitors and GLP-1 RAs is greater than ever following removal of the type 1 diabetes indication for SGLT2 inhibitor monotherapy in the UK and Europe.

2.1 Assessment and management of risk

a) Benefits

Semaglutide (Ozempic): is a once weekly injectable (subcutaneous) GLP1-RA licensed for reducing HbA1c in type 2 diabetes. The licensed doses are 0.25 mg weekly, 0.5 mg weekly and 1.0 mg weekly. In the trial, the starting dose is 0.25 mg weekly uptitrated if tolerated to 0.5 mg weekly after two weeks and then (again if tolerated) after a further two weeks to the maximum dose of 1.0 mg weekly. Semaglutide differs from other weekly GLP-1 receptor agonists in that its half-life is extended by binding to serum albumin i.e. it is not macromolecular. In type 2 diabetes, semaglutide therapy is associated with weight reduction and a reduction in rates of cardiovascular events (26). The licensed dose escalation schedule for routine treatment of type 2 diabetes is at four weekly intervals (27) but in a Phase 2 trial in type 2 diabetes authored by one of this trial's investigators (JP) doses were uptitrated at one-to-two weekly intervals and to higher doses (up to 1.6 mg weekly) (28). Phase 3 trials are currently in progress (for the indication of obesity) using a 2.4 mg semaglutide weekly. It

should be noted that semaglutide 0.25 mg is considered too low a dose for maintenance therapy in type 2 diabetes but it is unclear whether this may have a beneficial effect in type 1 diabetes at this dose.

While semaglutide once weekly has not been investigated previously in type 1 diabetes, liraglutide (a GLP-1 RA for daily administration with a similar molecular structure that binds less avidly to serum albumin) has previously been investigated (see above) in the ADJUNCT 1 and ADJUNCT 2 trials (8,9) in which, compared with placebo it resulted in reductions in HbA1c, weight and insulin requirement, albeit with a small increase in rates of hypoglycaemia (see below).

Dapagliflozin (Forxiga): is a once daily oral SGLT2 inhibitor previously licensed in Europe for reducing HbA1c in people with type 1 diabetes and BMI ≥ 27 kg/m^2 when insulin alone does not provide adequate glycemic control despite optimal insulin therapy (23). Although only the 5 mg dose was licensed for type 1 diabetes,both 5 mg and 10 mg daily are licensed for type 2 diabetes (29). As mentioned above, the removal of the type 1 diabetes indication by AstraZeneca was not due to any safety concern for any indication, including type 1 diabetes. In the Phase 3 trials of dapagliflozin in type 1 diabetes (DEPICT-1 and DEPICT-2) both doses were used. In both DEPICT-1 and DEPICT-2 there were modest additional benefits with 10 mg compared to 5 mg dapagliflozin. For example, in DEPICT-1, insulin dose requirement was reduced by 13.2% vs placebo on 10mg daily compared with 8.8% on 5mg daily (14). Weight reduction vs placebo in DEPICT-1 at 52 weeks was by 4.5% on dapagliflozin 10mg daily compared with 2.95% in those receiving 5 mg daily. Given the propensity of insulin to encourage weight gain over time and the life-long insulin requirement in T1D, sustained weight reduction and insulin dose reduction - even when modest - may be clinically important. There was no increase in the rate of hypoglycaemia in either the 5 mg or 10 mg groups compared with placebo. (14,15)

Dapagliflozin therapy in type 2 diabetes has been demonstrated to be associated with cardiovascular and renal risk reduction: in DECLARE-TIMI, DAPA-HF and DAPA-CKD the 10 mg dose was used (30, 31, 32). Such cardiovascular and renal benefits may translate to type 1 diabetes with appropriate monitoring and risk mitigation, although support for such measures by the manufacturer were withdrawn in the UK at the time of the removal of the type 1 diabetes indication . Given these potential benefits with relatively low increased risk, the 10 mg rather than 5 mg dose was selected for steady state treatment in this protocol.

b) Risks/ side effects Please also refer to the respective UK SmPCs (27, 29)

Semaglutide:
Very common: nausea, diarrhoea; hypoglycaemia (when used with insulin).
Common: vomiting, abdominal pain, constipation, gastro-oesophageal reflux, cholelithiasis, weight loss.
Uncommon: injection site reactions, hypersensitivity
Rare: Anaphylactic reaction.

Hypoglycaemia/ ketosis: Semaglutide once weekly has not been studied previously in type 1 diabetes; however, the ADJUNCT-1 and ADJUNCT-2 trials examined the role of liraglutide once daily (a similar molecule in the GLP-RA class that binds less avidly to serum albumin). Although HbA1c and weight were reduced by both 1.2 mg and 1.8 mg liraglutide, rates of symptomatic hypoglycaemia increased by 20-30%. In ADJUNCT-1, insulin doses were re-uptitrated quite rapidly towards baseline levels after an initial dose reduction, but in ADJUNCT-2 were “capped” (and therefore remained lower). Perhaps for that reason, an increase in hypoglycaemia was less consistent in ADJUNCT -2 but there was an excess of ketosis at the higher (1.8 mg) dose, presumably due to under-insulinisation (no lower limit for insulin dose reduction was set). It should be noted that monitoring of participants by site investigators in the ADJUNCT trials was less intensive than will be the case in the present trial with

less frequent opportunities for insulin dose titration (see Section 7). Importantly, participants at high risk of hypoglycaemia, (including those with a history of severe hypoglycaemia and those with hypoglycaemia unawareness) were not excluded from the ADJUNCT trials (8,9).

Retinopathy: In the SUSTAIN-6 cardiovascular outcome trial of semaglutide in type 2 diabetes, rapid reduction of HbA1c was associated with progression of existing proliferative retinopathy in those with a high baseline HbA1c ("early worsening"/ "normoglycaemic re-entry"). However, there was no evidence of worsening of retinopathy on treatment in the trials SUSTAIN 1-5 in which those with proliferative retinopathy were excluded (33, 3). In clinical trials of duration up to one year involving 4,807 patients with type 2 diabetes, adverse events related to diabetic retinopathy were reported in similar proportions of subjects treated with semaglutide (1.7%) and comparators (2.0%) (33).

Other adverse effects: In type 2 diabetes, gastrointestinal adverse effects of semaglutide can be prevented in the majority of cases by careful dose uptitration (28, 34) although up to 5% are ultimately unable to tolerate it. In our previous trial with liraglutide in T1D, adverse effects occurred with a similar frequency to that observed in T2D (in which there is extensive clinical experience); on this basis it is expected that this will also be the case with semaglutide (8,9).

Dapagliflozin:
Very common: hypoglycaemia (when used with insulin).

Common: ketoacidosis, genital infections (vulvovaginitis, balanitis), urinary tract infection, diabetic ketoacidosis, increased creatinine.

In the DEPICT-1 study, ketoacidosis occurred in 4 of 277 participants on 5mg dapagliflozin, 5 of 296 participants on 10 mg and 3 of 260 participants in the placebo group (summarised as 1%, 2% and 1% respectively) with one severe episode in each group (14). In pooled analyses of DEPICT-1 and DEPICT-2 (14,15) there was, however, an approximate threefold elevated rate of DKA adjudicated as "definite" over placebo on active therapy: 2.0% on 5 mg dapagliflozin, 1.9% on 10 mg and 0.6% on placebo – i.e. rates of DKA were similar with the two doses (14,15,16). Rates of DKA with dapagliflozin were described as "occurring in at least 1 per 100 patients" by the manufacturer at the time the indication was removed (24). Rates of DKA with sotagliflozin, the other SGLT2 inhibitor licensed for type 1 diabetes, were somewhat higher (2.9% on 200 mg, 3.8% on 400 mg, 0.2% on placebo) in pooled analyses of Phase 3 trials (19,21).

Increased creatinine: in 13 studies summarized in the Summary of Product Characteristics increases in serum creatinine (generally transient) occurred in 3.2% vs 1.8% of individuals with eGFR ≥ 60 ml/min/1.73 m^2 who received dapagliflozin 10 mg vs placebo rising to 18.5% vs 9.3% in those with eGFR <30 ml/min/1.73 m^2 (16,29). These changes are usually observed 4 weeks after SGLT2i initiation, recovering by week 12. Participants with eGFR < 45 ml ml/min/1.73 m^2 will not be included in the TTT1 trial and creatinine/ eGFR will be monitored at 5 weeks (Visit 12) and 13 weeks (Visit 13) after randomisation to dapagliflozin or placebo (Visit 10). Any participant who has persistent reduction in eGFR of 15 ml/min or more will meet withdrawal criteria from the trial (see Section 7.8).

Uncommon: fungal infection, volume depletion, constipation.
Not known (several case reports): Fournier's gangrene.

Fournier's gangrene is a very rare clinical entity characterized by necrotizing fasciitis of the perineal area. This has been infrequently reported in association with SGLT-2 inhibitor use in both men and women. However, there was no evidence of increased risk in a meta-analysis of 84 clinical trials

involving 42415 participants. Nevertheless, participants will be advised to seek urgent medical assistance if they experience erythema, pain, swelling of the perineal area associated with fever and malaise (this will be reinforced in the Patient Information Sheet). (36)

Amputation: An increased incidence of lower limb amputation has been reported in type 2 diabetes with the SGLT-2 inhibitor canagliflozin. However, this has not been demonstrated with dapagliflozin (not included in the UK SmPC). People with diabetes are at increased risk of amputation and good foot care is a key component of good diabetes care regardless of use of SGLT-2 inhibitors.

<u>Placebo</u>:
Placebo capsules contain microcrystalline cellulose.
This trial is therefore categorised by the Co-Sponsors as:

- Type B = Somewhat higher than the risk of standard medical care

  See Appendix 1 (Risk Assessment)

3 OBJECTIVES AND OUTCOME MEASURES/ ENDPOINTS

3.1 Primary objective

- To assess in people with type 1 diabetes whether triple therapy (dapagliflozin, semaglutide and insulin) for six months improves glycaemic control (as measured by HbA1c) compared with dual therapy (placebo, semaglutide and insulin – Period 2).

3.2 Secondary objectives

To assess in people with type 1 diabetes whether:

- Triple therapy (dapagliflozin, semaglutide and insulin) improves glycaemic control (as measured by HbA1c) compared with standard therapy (insulin)
- Dual therapy (semaglutide and insulin – period 1) improves glycaemic control (as measured by HbA1c) in comparison with standard therapy (insulin only)
- Triple therapy (dapagliflozin, semaglutide and insulin) improves glycaemic control as assessed by a range of continuous glucose monitoring (CGM) parameters, lowers body weight, reduces insulin requirement, improves blood pressure and improves quality of life (as assessed by validated patient questionnaires) compared with dual therapy and standard therapy.

3.3 Outcome measures/endpoints

3.4 Primary endpoint/outcome

Change in HbA1c (week 26 to week 52)

3.5 Secondary endpoints/outcomes

Efficacy:

Change in HbA1c

Mean fasting blood glucose

Mean blood glucose*
Percentage time in range (3.9 - 10.0 mmol/)*
Standard deviation of blood glucose*
Percentage time in hyperglycaemia Level 1 (10.1 - 13.9 mmol/L/)*
Percentage time in hyperglycaemia Level 2 (>13.9 mmol/L) *
Percent time in hypoglycaemia Level 1 (3.0 - 3.8 mmol/)*
(by masked DEXCOM G6 CGM)*

Insulin requirement
Bodyweight
Systolic and diastolic BP
Number of antihypertensive medications
Diabetes Specific Quality of Life Scale (DSQOLS)
Problem Areas In Diabetes (PAID) questionnaire

Safety:

Rate of hypoglycemic events Level 2 (<3.0 mmol/L/ <54mg/dl)
Rate of hypoglycemic events Level 3 (severe event with altered mental and/or physical status requiring assistance)
Rate of diabetic ketoacidosis defined as elevated serum or urine ketones (greater than the upper limit of the normal range), and serum bicarbonate < 15 mmol/L or blood pH < 7.3

3.6 Tertiary endpoints/outcomes

Postprandial glucose (AUC) – meal tolerance test
Postprandial glucagon (AUC) – meal tolerance test
Postprandial FFA (AUC) – meal tolerance test
Postprandial GLP-1 (AUC) – meal tolerance test
Fructosamine

3.7 Exploratory endpoints/outcomes

Plasma and urine samples will be collected and frozen for later analysis for indices of inflammatory stress and other relevant measures.

3.8 Table of endpoints/outcomes

Endpoints	Outcome Measures	Timepoint(s) of evaluation of this outcome measure
**Primary endpoint**<br>Triple therapy (dapagliflozin, semaglutide and insulin) in comparison with dual therapy (placebo, semaglutide and insulin – period 2)	Change in HbA1c	Week 26 to Week 52
**Secondary endpoint 1**<br><br>Triple therapy (dapagliflozin, semaglutide and insulin) in comparison with standard therapy (insulin alone)	Change in HbA1c	Week 0 to Week 52
**Secondary endpoint 2**<br><br>Dual therapy (semaglutide and insulin – period 1) in comparison with standard therapy (insulin only)	Change in HbA1c	Week 0 to Week 26
**Other secondary endpoints**<br><br>a) Triple therapy (dapagliflozin, semaglutide and insulin) in comparison with dual therapy (semaglutide, insulin and placebo – period 2)<br><br>b) Dual therapy (semaglutide and insulin – period 1) in comparison with standard therapy (insulin only)	Change in:<br>- Mean fasting blood glucose (4 days)<br><br>- Mean blood glucose*<br>- Percentage time in range (3.9–10 mmol/L)*<br>- Glycaemic variability (SD)*<br>- Percent time in hyperglycaemia Level 1 (10.1-13.9 mmol/L)*<br>- Percent time in	Week 26 to Week 52<br><br>Week 0 to Week 26

	hyperglycaemia Level 2 (>13.9 mmol/L)*<br>- Percent time in hypoglycaemia Level 1* (3.0-3.8 mmol/L)<br>*(*by masked Dexcom G6 CGM)*<br><br>Insulin requirement<br>Body Weight<br>Systolic and diastolic blood pressure<br>Number of antihypertensive medications<br>Diabetes Specific Quality of Life Scale (DSQOLS)<br>Problem Areas In Diabetes (PAID) questionnaire	
**Tertiary endpoints**<br>a) Triple therapy (dapagliflozin, semaglutide and insulin) in comparison with dual therapy (semaglutide, insulin and placebo –period 2)<br><br>b) Dual therapy (semaglutide and insulin – period 1) in comparison with standard therapy (insulin only)	Change in:<br>Postprandial glucose (AUC)<br>Postprandial FFA (AUC),<br>Postprandial GLP-1 (AUC)<br>Postprandial glucagon (AUC)	Week 26 to Week 52<br><br>Week 0 to Week 26
**Safety endpoint 1**<br>a) Triple therapy (dapagliflozin, semaglutide and insulin) in comparison with dual therapy (semaglutide, insulin and placebo – period 2)<br><br>b) Dual therapy (semaglutide and insulin – period 1) in comparison with standard therapy (insulin only)	Difference between treatment arms in:<br>(i) Rate of Level 2 (<3.0 mmol/L/ <54mg/dL) hypoglycemic events<br>(ii) Rate of Level 3 hypoglycemic events (severe event with altered mental and/or physical status requiring assistance)	Week 26 to Week 52<br><br>Week 0 to Week 26
**Safety endpoint 2**<br>a) Triple therapy (dapagliflozin, semaglutide and insulin) in comparison with dual therapy (semaglutide, insulin and placebo – period 2)	Difference between treatment arms in:<br><br>Rate of diabetic ketoacidosis (serum or urine ketones above normal limits) AND serum bicarbonate < 15 mmol/L OR	Week 26 to Week 52

b) Dual therapy (semaglutide and insulin – period 1) in comparison with standard therapy (insulin only)	blood pH < 7.3	Week 0 to Week 26

4 TRIAL DESIGN

The trial has two six-month periods:

Period 1 open-label randomised (2:1) parallel group semaglutide once weekly + insulin (standard treatment) vs insulin alone (standard treatment).
Period 2 double-blind, randomised (1:1) parallel group, dapagliflozin vs placebo for those participants initially randomised to semaglutide (with ongoing standard of care for all participants)

Thus, according to the original sample size calculation (from Protocol versions 1.0-5.0) but revised in this Protocol version 6.0 (see Section 10), during the first six months, approximately 114 participants with type 1 diabetes (50 in the UK) will be randomised (open label, see below) into three groups (approximately 38 each; i.e. 19 each in the UK) one of which will be maintained on their regular insulin therapy for 12 months (standard therapy group) and two of which will be started on semaglutide in addition to regular insulin treatment for six months (dual therapy). Following six months of stable treatment, those initially randomised to insulin and semaglutide will be additionally randomised (double-blind) to dapagliflozin (triple therapy) or matching placebo (dual therapy) for a further six months. The dose of dapagliflozin will not be increased to 10 mg in those who have confirmed ketonuria (+1) or ketonaemia (≥0.6 mM) after the first week of treatment.

In keeping with the intention-to-treat principle, participants in whom therapy is discontinued or downtitrated due to adverse effects will be encouraged to continue to undertake all study assessments and attend study visits.

5 STUDY SETTING

The study will be conducted at two sites: State University of New York (SUNY) at Buffalo, USA and Glasgow, UK. In Glasgow, n=41 participants (50% of the new total) will be identified from secondary care sites and via the Scottish Diabetes Research Network research register. Both randomisations will be stratified by site.

6 ELIGIBILITY CRITERIA

6.1 Inclusion criteria

1) Type 1 Diabetes for at least one year on stable multiple daily (four or more) injections (MDI) of insulin or continuous subcutaneous insulin infusion (CSII) for the previous three months.
2) C-peptide < 0.23 nM
3) Minimum dose of insulin: 0.5 U/kg for MDI and 0.4 U/kg for CSII
4) Regularly measuring blood glucose: four or more times daily either by fingerstick, testing or by "flash"/ continuous glucose monitoring)
5) HbA1c 7.5 – 11.0 % inclusive
6) Well versed in carbohydrate counting*

7) Age 18-70 years.
8) BMI ≥25 kg/m^2.

** In the opinion of the site Principal Investigator*

6.2 Exclusion criteria

1) Type 1 diabetes for < 12 months, type 2 diabetes, chronic pancreatitis, MODY
2) Previous use of any agent other than insulin for glycaemic control in the last three months.
3) History of diabetic ketoacidosis (DKA) requiring medical intervention (e.g. emergency room visit and/or hospitalisation) within three months prior to the screening visit.
4) Following ketogenic diet, low (<50g/day) carbohydrate diet or intermittent fasting.
5) Hypoglycaemia unawareness* or frequent episodes of severe hypoglycaemia as defined by more than one episode requiring medical assistance, emergency care (paramedics or emergency room care), or glucagon therapy administered by a third-party individual within three months prior to the screening visit
6) Symptoms of poorly controlled diabetes
7) Participation in a weight loss program with ongoing weight loss, or in an intensive exercise program
8) History of bariatric or other weight-loss surgery within 12 months prior to the screening visit
9) History of Addison's disease or chronic adrenal insufficiency
10) History of diabetes insipidus
11) Hepatic disease or cirrhosis
12) Aspartate Aminotransferase (AST) > 3 X Upper limit of normal (ULN) and/or Alanine aminotransferase (ALT) > 3 X ULN
13) Serum Total Bilirubin > 2 X ULN unless exclusively caused by Gilbert's Syndrome
14) Haemoglobin < 110 g/L (11.0 g/dL) for men; haemoglobin < 100 g/L (10.0 g/dL) for women
15) Coronary event or procedure (myocardial infarction, unstable angina, coronary artery bypass, surgery or coronary angioplasty) in the previous three months or history of congestive heart failure
16) End stage renal disease on haemodialysis and/or eGFR < 45 ml/min/1.73m^2
17) HIV or Hepatitis B/C positive status
18) Any other life-threatening, non-cardiac disease
19) History of pancreatitis or cystic fibrosis
20) Women who are pregnant, planning pregnancy or breast-feeding and women of childbearing potential (WOCBP) who are not established on a method of contraception considered highly effective (see Appendix 2) and willing to continue it for the duration of the study and for at least two months after last dose of IMP.
21) Inability to give informed consent
22) History of gastroparesis
23) History of medullary thyroid carcinoma or MEN 2 syndrome
24) History of hypersensitivity reaction to GLP-1 agonists or SGLT2 inhibitors or known hypersensitivity to any of the excipients
25) Known gallstones (history of cholecystectomy is not an exclusion criterion)
26) History of alcohol problem or drug abuse
27) Hypertriglyceridemia (>500 mg/dl)
28) Recurrent genital mycotic infection (more than one episode in last six months)

29) Hypovolaemia
30) Any malignancy except treated *in situ* malignancy and basal cell carcinoma of the skin
31) Unexplained haematuria (dipstick positive on two occasions one week apart) and/or active bladder cancer
32) Proliferative retinopathy (on basis of photographic retinal screening which must have been performed within the last 24 months)
33) Use of an investigational agent or therapeutic regimen within 90 days of study
34) Participation in any other concurrent interventional clinical trial
35) Unlikely to be able to comply with study requirements*
36) Unable to undergo meal challenge for dietary reasons (e.g. vegetarian/ vegan diet)

** In the opinion of the site Principal Investigator*

7 TRIAL PROCEDURES

Also see *Schedule of Assessments*

Each participant will be enrolled in the trial for approximately 55 weeks, comprising three weeks of monitoring to stabilise treatment and collect baseline data before randomisation into the intervention or standard of care/ placebo arms for 52 weeks. Following a completed screening visit and meeting the Inclusion and Exclusion criteria, each participant will have a total of 16 scheduled study visits. In addition, at weeks 3, 5, 8, 16, 20, 29, 35, 44 and at two weeks after the end of the study, each participant will receive a phone call to document any adverse effects or other changes. No long-term follow-up is planned following completion of participation in the study.

Participants will be treated with either insulin only (standard treatment) for the whole duration of the study or with insulin and semaglutide weekly s.c. injection (titrated where possible up to 1.0 mg, see below) for the first six month period and then randomised to either oral dapagliflozin (triple therapy) or placebo (dual therapy) daily added for the second six month period. Semaglutide will be started (Visit 4) at a dose of 0.25 mg once weekly for two weeks then increased if tolerated to 0.5 mg (Visit 5). If well tolerated at week 4, it will be increased to 1.0 mg once weekly (Visit 6) or at a subsequent visit at the discretion of the PI. Dapagliflozin (or placebo) will be started at a dose of 5 mg daily at week 26 and increased at week 27 to 10 mg (2 x 5mg capsules) daily to ensure drug tolerability and minimise the risk of insulin-induced hypoglycaemia or hypotension.

Excessive weight loss: Both semaglutide and dapagliflozin are known to cause weight loss in the range of 5-10% in type 2 diabetes. However, the magnitude of weight loss when these are combined in type 1 diabetes is uncertain. Excessive weight loss may lead to increased risk of hypoglycemia or decreased insulin requirement increasing the risk of ketosis or DKA (see Section 2.1b) and other unforeseen risks. Therefore, weight loss during any phase of the study will be limited to ≤ 15% of body weight at randomisation or reaching a BMI <22 kg/m^2. If participants are approaching this threshold, IMP will be sequentially reduced at the discretion of the PI. Semaglutide may be reduced to 0.25mg or 0.5 mg and dapagliflozin to 5 mg. See also Section 7.8 regarding treatment interruption and withdrawal.

Covid-19 Mitigation

In view of the Covid-19 pandemic, certain trial procedures may be changed if necessary to reduce in person contact with participants. This will be continuously reviewed in view of the prevailing circumstances in line with local and national guidance and with the principle of protecting participants and study personnel. In the event of future waves of infection this would be particularly important. In this context, consideration would be given to rearranging visits, changing to virtual visits using video technology or using additional phone calls to ensure patient safety. Consideration will also be given to how minimising face-to-face interaction with participants when dispensing study medication. If required, supplies of study medication sufficient to last for longer periods may be dispensed to minimise contact and visits. Courier services may additionally be used to deliver study medications to participants.

All in person visits will be subject to local and national guidance around the use of personal protective equipment including face masks and where appropriate when conducting procedures, the use of gloves and aprons.

Gastrointestinal adverse effects:
During the initial weeks of participation, those participants who report nausea and/or vomiting during uptitration of semaglutide dose that is not sufficiently severe to require treatment discontinuation will remain on their current dose until they are symptom-free prior to discussing a further dose escalation (i.e. uptitration will be to the highest dose tolerated rather than a “forced titration”). Dose will be downtitrated to the maximum tolerated in those who experience gastrointestinal adverse effects.

Retinopathy:
As there was no excess of retinopathy with semaglutide treatment in Phase 3 trials with semaglutide, in which proliferative retinopathy was an exclusion criterion (as in the current protocol), additional retinal imaging beyond annual retinal photography (the standard of care) is not proposed (30).

Insulin dose adjustment:
Minimising hypoglycaemia: All participants will be made aware of the potential for increased hypoglycaemia with addition of adjunct therapy and supported and encouraged to monitor blood glucose at least four times daily in order to minimize the risk of hypoglycaemia. Participants will have ready access to advice from specialist medical and nursing staff at face-to-face and telephone visits. Those who have ongoing access to wearable continuous glucose monitoring [or "flash" glucose monitoring, (FGM)] will continue to use these devices. Information on management of hypoglycaemia will be included within participant diaries, reinforcing the pre-existing knowledge of participants, the majority of whom will have lived with type 1 diabetes for many years.

Reductions in insulin dose will not usually be greater than 10% (5% if HbA1c is < 7.0 %) from current levels. At randomisation, daily insulin dose will not be reduced by more than 20% from the baseline level and then not more than 20% from the new baseline levels at 26 weeks. Participants requiring an insulin dose reduction > 20% and their total insulin dose < 0.4 U/kg (current weight) will be monitored closely for insulin dose, carbohydrate intake, ketones and blood glucose. Following the second randomisation at 26 weeks, dapagliflozin/ placebo will be temporarily discontinued if insulin dose falls below this threshold. The participant will be withdrawn from the study if this remains so at review in one week [see also Withdrawal Criterion 9 and Section 8.12

Minimising ketosis: Participants will be instructed to stay hydrated and not to reduce their caloric intake (especially carbohydrates) or start new dietary approaches (especially ketogenic diets) during the study. They will be educated to recognize the signs and symptoms of diabetic ketoacidosis (DKA)

and to undertake additional self-testing of urine and/or blood ketones if they develop nausea, vomiting, or anorexia. In addition, they will be given education and a written ketosis management plan (STICH Protocol – i.e. STop SGLT-2 inhibitor or placebo (temporarily), give Insulin bolus, take additional Carbohydrate, and additional Hydration - see Appendix 3) (37).

From Visit 10 (26 weeks), those participants undergoing the second randomisation will be supported and encouraged to monitor urine or blood ketones at home at least once daily: three days before visit 10 (week 26), they will be asked to start daily measurements of urine ketones [CombiScreen® 10 SYS Plus urine testing strips, Sterilab, UK (or equivalent)] or blood ketones [CareSens™ Dual, i-SENS, Koreal; GlucoMen® Areo 2K, Menarini, UK (or equivalent]]. They will be asked to record these in the diary/ log provided. In addition, ketones (blood and urine) will be measured at every visit. If ketone bodies in urine are +1 or greater (≥0.6 mM in blood), participants will be reminded to keep well hydrated and notify the study team as soon as possible (office hours) as diabetic ketoacidosis (DKA) can occur even at glucose levels < 14 mmol/L (38).

If ketones are +2 or more in urine (> 1.5 mmol/L) participants will be advised to initiate the STICH Protocol immediately and contact the study team in office hours (or telephone NHS 24 or attend their Accident & Emergency if out of hours) (37). This information will be reinforced verbally at study visits and also in the form of a Patient Alert Card that participants will be asked to carry. This will also contain details of the investigational study drug, the study number, the investigator’s name, and a contact number (for in hours contact). The study team will assess for symptoms or test results indicating a risk of ketoacidosis immediately on reporting and if blood ketone levels are persistently (one week) at these levels dapagliflozin/placebo will be permanently discontinued.

If blood ketone readings are confirmed as > 3.0 mmol/L (or urine ketones reading +++) participants will be advised to stop dapagliflozin/ placebo and attend the Accident and Emergency department without delay.

Participants will be advised that dapagliflozin/placebo may be temporarily stopped in other clinical situations known to predispose to ketoacidosis (e.g. acute serious medical illnesses or hospitalisation for major surgical procedures). Any requirement for intravenous insulin infusion (whether in Accident and Emergency or as an in-patient admission) will be recorded as a Serious Adverse Event. For further detailed procedures see section 9.31.

Volume depletion:
There is a theoretical risk of volume depletion and low blood pressure when dapagliflozin is used in combination with other antihypertensive drugs. All pre-existing concomitant medication will be reviewed at the screening visit as part of the medical history and examination (see Section 7.1.3 for further details). Participants undergoing the second randomisation will be educated on symptoms suggestive of low blood pressure (e.g. light-headedness, dizziness, feeling faint) and asked to contact the study team if they experience these symptoms. In clinical practice however SGLT-2 inhibitors are commonly used with anti-hypertensive drugs with no significant problems (31).

Genital mycotic infections:
Individuals who report symptoms suggestive of genital mycotic infection will be advised to self-treat with topical antifungal preparations. If a second episode occurs, a decision on treatment discontinuation will be considered in discussion between the participant and the site Principal Investigator, taking account of the severity of the symptoms.

Foot care:
Annual foot checks will be conducted for all participants as per standard of care.

7.1 Recruitment

7.1.1 Patient identification

All personal details will be managed according to ISO 27001:2013 compliant Standard Operating Procedures.

Potentially eligible participants will be identified from the Scottish Diabetes Research Network Research Register (a managed and searchable database of people with diabetes who have agreed to be contacted about clinical trial participation) and at secondary care diabetes clinics in Greater Glasgow and Clyde. The principal site for recruitment will be Stobhill Ambulatory Care Hospital but other sites may be opened if necessary as Patient Identification Centre sites. The majority of study visits (and all visits involving drug dispensing) will be conducted at the Clinical Research Facility, Glasgow Royal Infirmary. Information posters with the contact details of the research team will be displayed in recruiting clinics.

At the principal site, clinic lists (whether remote or face-to-face) will be pre-reviewed by members of the research team. An email will be sent to the clinic Consultant physician to flag potentially eligible individuals on their list; potential eligibility will also be flagged on the clinic electronic list (on Trakcare®) to prompt reviewing physicians (as members of the usual care team) to ask if the potential participant would be interested in receiving more information from the research team.

In the above email (to which a response is not required), physicians will be asked to record the outcome of their discussion with the potential participant; either: (i) by adding a note to the Clinical Portal system; (ii) adding an appropriate statement to their clinic letter; or (iii) by return email using standard text (e.g. willing to be contacted/ not willing to be contacted/ unsuitable for the trial). The permitted and preferred method(s) of contact (e.g. mobile telephone/ home telephone/ letter/ email should also be recorded. Telephone contact will be the preferred contact method with other methods offered where this is declined. If no preferred method is indicated by consultant it will be presumed that telephone contact is acceptable Physician consultations with potentially eligible individuals that are concluded with no invitation to research participation (e.g. due to pressure of time) can be followed up with a phone call from one of the study physicians who is also a member of the research team.

Following clinical consultations, members of the research team will identify potential participants who appear to be meet eligibility criteria and have given permission to be contacted.. These individuals will be called and/or sent/emailed (as appropriate according to documented permissions) a Patient Information Sheet. An invite letter and Patient Information Sheet will be sent to potential participants who have declined telephone contact but agreed to mail or email contact. Where permission has been given for phone contact, this can be followed up by up to two further phone contacts (provided permission to contact is not withdrawn by the potential participant in an earlier call). A visit to the Clinical Research Facility will then be scheduled for informed consent and screening. The person scheduling the CRF visit will confirm with the patient if they have a copy of the full Patient Information Sheet or if they need a further copy to be sent out in advance of the visit. This will be sent out to arrive at least 48 hours prior to the planned CRF visit or emailed to potential participant.

At the screening visit, eligibility criteria will be checked and the study procedures explained. All potential participants will have had at least 24 hours to review the full Patient Information Sheet. They will then be invited to provide written, informed consent.

At other sites within NHS Greater Glasgow and Clyde the same approach will be taken for the clinic lists of those physicians who indicate willingness to support trial recruitment. The initial approach will always be from a member of the usual care team.

Covid-19 Mitigation: The delivery of outpatient clinics has been substantially changed due to the pandemic. The use of telephone clinics is now standard for many visits and video technologies are also being used. These mediums will therefore be used for inviting participants to take part when in person appointments are not taking place. This may be subject to change depending on the situation with Covid-19. Other principles of recruitment will be as described above.

7.1.2 Screening

A separate non-fasting visit will be arranged at which potential participants will have further time to discuss with the study nurse and doctor.

Prospective participants will be asked to read and understand the Participant Information Sheet. Any questions regarding the Protocol will be answered. Risks and side-effects of the active trial medication will be explained. Eligibility for trial enrolment will be confirmed by a medical practitioner.

Individual wishing to participate in the study will then be invited to sign to document their informed consent (see Section 7.2 below).

7.1.3 Screening assessments

Items from the medical history relevant to the Inclusion and Exclusion criteria will be recorded. Demographic data, medical history and current medications (including current insulin doses) will be documented. All participants will have access to a dual blood glucose and ketone meter as per the contemporary standard of care in type 1 diabetes. A physical examination will be conducted. Clinical observations will be recorded and blood and urine samples obtained (see below):

Demographics

- Date of birth
- Sex
- Ethnicity
- Date of diagnosis
- Ethnic group: white/black/Asian/other
- Height

Clinical assessments

- Systolic and diastolic blood pressure (after 5 minutes rest)
- Heart rate
- Body weight (clothed without coat and shoes)
- Capillary blood glucose

Medical History

- Smoking status: current/ex/never
- Alcohol consumption: units/week
- Hypertension: Y/N
- Microalbuminuria: Y/N
- Proteinuria: Y/N
- Neuropathy: Y/N
- Previous major cardiovascular event(s): Y/N (date)

- Existing CV disease of any type*: Y/N
- Amputation above forefoot: Y/N
- Hospitalisation for DKA in last year
- Level of educational attainment: University (college) educated Y/N

* Existing CV Disease is defined as any of: MI, stroke, ACS/unstable angina, coronary stent/angioplasty, CABG, TIA, heart failure, peripheral vascular disease

Insulin treatment

- Regimen: MDI, pump,
- Total dose (all); Basal (longer acting) dose/ Bolus (shorter acting) dose (for MDI only)

Other treatment (drug classes and names)

Current use Y/N. If no, has there been use in last three months?: Y/N

- ACE inhibitors
- Angiotensin receptor blocker
- Calcium channel blocker
- Alpha-blocker
- Beta-blocker
- Loop diuretic
- Thiazide like diuretic
- Statin
- Aspirin
- Clopidogrel
- Novel anticoagulant
- Warfarin

Blood assessments:

- Blood ketones
- HbA1c
- FBC
- Urea and electrolytes
- Creatinine
- Liver function tests (AST/ ALT/ Alkaline Phosphatase)
- Bone profile (Calcium/ Phosphate)
- C-peptide (capillary blood glucose must be > 4.0 mmol/L at time of sampling)
  N.B. where re-screening is undertaken (see 7.2.1 below), C-peptide does not require to be repeated

If blood tests have been assessed for clinical purposes within the last three weeks, these values will be used.

Urine sample:

- Urine ketones
- For all WOCBP, a urine pregnancy test will be checked at screening

Potential participants meeting all the inclusion and exclusion criteria based on history, examination and screening tests will be enrolled in the study.

Pregnancy: WOCBP will be asked about pregnancy status and contraceptive usage (see Appendix 2) and a pregnancy test will be conducted (following informed consent) prior to randomisation. We will not recruit those who are pregnant, wanting to become pregnant, or using birth control methods not considered highly effective. Every four weeks following randomisation, WOCBP randomised to IMP (as opposed to standard of care) will be asked about their contraception status, and a urine pregnancy test carried out. Study medication will be permanently discontinued in women who become pregnant or considered to be on contraception that is not highly effective. As the study assessments *per se* do not carry an additional risk over standard of care to either mother or unborn child, further pregnancy tests will not be routinely offered following randomisation to those women allocated to “Insulin therapy (standard of care)” (i.e. who are not exposed to IMP). All pregnancies will be notified to the Sponsor Pharmacovigilance Officer using the standard pregnancy notification form and the pregnancy followed to outcome).

7.1.4 Payment

Participants will be offered reasonable travel expenses for visits additional to usual care.

7.2 Consent

Potential participants will be identified and screened by the Inclusion and Exclusion criteria listed above. Medical staff or appropriately trained support staff will seek consent for screening and participation in the trial from individuals that fulfil the criteria. All potential participants will have at least 24 hours to review the Patient Information Sheet before being approached (see Section 7.1.2 above). Following written consent, each signature will be dated by the signatory, the original retained in the site file, a copy provided to the participant and a copy scanned into the participant's Clinical Portal record.

Data collected for routine clinical care will be used for clinical trial documentation (e.g. blood results within the previous three weeks). In the absence of routine blood results, sampling of blood for study specific laboratory measurements can only be performed following documentation of informed consent.

7.2.1 Re-screening post-consent

Where inclusion (e.g. HbA1c ≤7.5%) or exclusion (e.g. DKA within the last three months) criteria are not met (e.g. screening visit blood results falling outside the study parameters), potential participants can be re-screened with additional urine and blood tests on one further occasion.

Where the screening period (i.e. between the Screening visit and Visit 1) exceeds 10 weeks, screening visit urine and blood tests should be repeated at an additional visit that can be arranged with verbal consent of the participant prior to Visit 1. Data will be amended on the web portal as appropriate.

If the initial consent was signed within three months of the re-testing, new consent is not required unless the consent form template has been updated in this period. If the initial consent was not signed within three months of re-testing then consent should be sought again.

Rescreened participants will keep the same Patient ID and data will be amended on the web portal as appropriate.

Where screening bloods are repeated in cases as above, C-peptide testing does not require to be repeated as values consistent with the inclusion criteria remain valid long term.

7.3 Run-in period

7.3.1 Visit 1 (@3 weeks prior to randomisation)

Participants who meet entry criteria will be scheduled for their next visit at approximately three weeks prior to randomisation. Daily fingerstick blood glucose levels (study diaries), CGM/ FGM data or insulin pump download data (as applicable) will be collected and reviewed and appropriate insulin dose adjustments made by the medical team to target fasting blood glucose concentrations between 5 -10.0 mmol/L if possible at all times without having additional hypoglycaemia (< 3.9 mmol/L). Clinical observations will be recorded including resting BP measurement. Blood glucose and ketone bodies will be measured via a fingerstick blood sample (see Section 7). Urine for pregnancy status and ketone bodies will be assessed.

Participants will be instructed on the use of the Continuous Glucose Monitoring System (Dexcom G6 with blinded receiver). All subjects will be advised to monitor their capillary blood glucose by CGM or fingerstick at least 4 times a day (before meals and at bedtime) throughout the study and to wear their CGM constantly for next 20 days (changing the sensor at 10 days). CHO counting technique will be reviewed and appropriate support given with calculating bolus insulin doses.

Visit procedures:

Monitoring and treatment optimisation

- Check for adverse events
- Review blood glucose diary
- Blood ketones
- Adjust insulin doses (if necessary)

Clinical assessments

- Blood pressure
- Heart rate
- Weight

Urine sample:

- Check urine ketones

Other

- Fit and start masked Continuous Glucose Monitoring with Dexcom G6

7.3.2 Visit 2 (1 @ weeks prior to randomisation)

Fasting visit* - Participants will be asked to fast (no food or calorie intake) for 10 hours prior to the visit overnight. Those who plan to drive to fasting visits will be reminded of the increased risk of hypoglycaemia and DVLA guidelines. This will be repeated prior to each fasting visit and reiterated within the patient diaries. This visit will be scheduled for a morning appointment and participants will be advised not to take their breakfast bolus insulin dose prior to arrival. If participants arrive at the study visit in a non-fasted state, the visit will be terminated and a further fasting visit rearranged as soon as possible.

Daily fingerstick blood glucose levels (diaries), CGM/ FGM data or insulin pump download data (as applicable) will be collected and reviewed and appropriate insulin dose adjustments made. Clinical observations will be recorded as detailed below. Blood glucose and ketone bodies will be measured via a fingerstick blood sample (see Section 7). If blood glucose is less than 3.9 mmol/L, or more than 13.9 mmol/L, the visit will be rescheduled for an alternative date. Otherwise, an intravenous cannula will be placed in order to obtain blood samples for the duration of the visit. A Meal Challenge Test will be performed (described below). A 24 hr urine container will be dispensed to collect a 24 hour urine sample to bring in at the Week 0 visit. For this collection and all subsequent 24 hour urine collections, urine will be tested via dipstick testing and an aliquot retained for assay of albumin, creatinine, ketones, and 15-isoprostane.

Visit Procedures

Monitoring and treatment optimisation

- Check for adverse events
- Review blood glucose diary
- Blood ketones
- Adjust insulin doses (if necessary)

Clinical assessments

- Blood pressure
- Heart rate
- Weight

Urine sample:

- Check urine ketones
- 24 hour urine container dispensed

Other assessments

- Download Dexcom G6 CGM
- High fat high calorie Meal Challenge: in order to assess postprandial metabolism a Meal Challenge Test will be performed using an approximately 900 kilocalorie High Fat High Carbohydrate (HFHC) meal including an egg muffin sandwich, a sausage muffin sandwich and two hash browns [containing approximately 88g carbohydrate, 51 g fat (33% saturated) and 34 g protein]. Participants will be asked to attend fasting prior to breakfast insulin bolus (with continuing basal insulin); participants will be asked not to take bolus insulin until the 3 hour time point unless blood glucose values reach above 22.2 mmol/L. Blood samples will be collected from an indwelling intravenous canula in a superficial forearm vein for glucose, glucagon, FFA, GLP-1 (AUC). Sequential blood samples will be obtained at 0, 15, 30, 45, 60, 90, 120, 150 and 180 min after consuming the meal. Volume of samples at 15, 30, 45, 90 and 150 min will be 5ml while those at 0, 60, 120 and 180min will be 30 ml (total volume = 145 ml). A spot urine sample will be collected at baseline, 1 and 3hr for standard urinalysis and an aliquot retained for assay of ketones and 15-isoprostane.

7.4 Randomisation

Participants who are being randomised will be required to have undergone all screening assessments.

At the randomisation visit, study participants will be provided with a Patient Alert Card. There are two Versions: (1) for the intervention arm i.e. those allocated at first randomisation to open-label semaglutide who are also eligible for the second double-blind randomisation (dapagliflozin or placebo); and (2) for those allocated to standard of care. These cards supplement the study diaries and include contact information for the study team. Version 1 also contains unblinding information. Participants will be asked to carry their card at all times. These cards will be collected at the end of participation in the study.

7.4.3 First Randomisation Scheme

For the first randomisation (Period 1, open label), eligible and consenting participants will be randomised (2:1, stratified by site) to the two groups (open-label semaglutide or standard of care) using permuted blocks of variable size to minimize predictability.

7.4.4 Method of implementing the allocation sequence

Randomisation will be achieved by accessing an interactive web-based randomisation system (IWRS). The investigator will provide the participant identifier and the system will check the participant's eligibility from information already entered in the electronic Case Report Form (eCRF): if appropriate the randomisation group will be allocated.

7.5 Visit 3 (First Randomisation Visit)

Participants will be asked to attend in the fasting state (no food or calorie intake for 10 hours; see 7.3.2 above). Blood glucose levels and ketone bodies will be measured via a fingerstick blood sample using a glucose and ketone meter (see Section 7) and daily fingerstick blood glucose levels (diaries) collected and reviewed. The participant's own CGM/ FGM blood glucose data or insulin pump download data will also be collected and reviewed. Daily insulin dose will be adjusted following clinical review of study diaries: if reduced, this should not be by more than 20% from the baseline level Participants will discontinue use of the study masked CGM (Dexcom G6). Clinical observations will be recorded. Blood glucose and ketone bodies will be measured via a fingerstick blood sample (see Section 7). The 24 hour urine container will be collected. A fasting blood sample will be taken (45 mL) to evaluate study endpoints, FBC, U&E, LFT, bone profile, lipids, HbA1c, and fructosamine (detailed below). A urine sample will be collected to evaluate ketone bodies and, in females, pregnancy status.

Participants will be asked to complete Quality of Life questionnaires (see Appendices).

IMP will be dispensed for those randomised to open-label semaglutide (once weekly). Semaglutide will be started on site at the 0.25 mg dose. Where both research staff and participant are in agreement, the first dose can be self-administered with staff observing; otherwise research staff will administer the first dose. Participants will be instructed to call site staff directly in case of any problem or untoward side effects. An office hours phone number will be provided for advice. Outside of these hours, provision of emergency medical care if required will be via NHS 24 and/or Accident & Emergency. Participants will be specifically asked to call to inform research staff if they have hypoglycaemia (blood glucose < 3.9 mmol/L) or Level 2 hyperglycaemia (blood glucose >13.9 mmol/L), particularly if occurring on more than one occasion. This will apply for the remainder of the trial.

Visit Procedures

Fasting visit* as per visit 2.

Monitoring and treatment optimisation

- Check for adverse events
- Check adequacy of contraception (WOCBP)
- Review blood glucose diary
- Adjust insulin doses (if necessary)
- Blood ketones

Clinical assessments
Blood pressure

- Heart rate
- Body weight

Quality of life assessments

- Diabetes Specific Quality of Life Scale (DSQOLS)
- Problem Areas In Diabetes (PAID) questionnaire

Blood assessments

- Na, K, urea, creatinine, eGFR (MDRD)
- AST/ ALT/ Alkaline Phosphatase
- Calcium/ Phosphate
- FBC
- HbA1c
- Fructosamine
- Bilirubin
- Random glucose

Urine

- For WOCBP, a urine pregnancy test will be checked prior to first administration of IMP.
- Urine ketones
- 24 hour urine sample collected

Medication

- Administer open label semaglutide 0.25 mg if randomised to active treatment
- Issue Patient Alert Card (appropriate version according to randomisation)

Other:

- Read masked Continuous Glucose Monitoring with Dexcom G6

Bloods for storage – see Sample Handling Manual
Approximately 45 mls of venous blood will be withdrawn and collected in tubes containing EDTA. Blood will be centrifuged at 1500g for 20 mins at 4ºC. Plasma will be siphoned, aliquoted and stored locally at -80° ± 10°C until transport in batches to the central laboratory on dry ice.

7.5.1 Visit 4 (@1 week after first randomisation)

Clinical observations will be recorded. Blood glucose levels and ketone bodies will be measured via a fingerstick blood sample using the participant's own glucose and ketone meter (see Section 7)) and daily blood glucose levels collected and reviewed. Insulin doses will be adjusted as in visit 3. If HbA1c is <7.0

%, insulin dose reduction should not be >5% from baseline total dose. As for Visit 3, semaglutide 0.25mg will be injected on site for those randomised to active therapy; however, if the participant is unable to attend within 48 hours of the scheduled appointment, this dose can be self-administered at home (confirmed by a documented phone call) provided there were no adverse effects following the previous dose.

Visit Procedures

Monitoring and treatment optimisation
- Check for adverse events
- Review blood glucose diary
- Adjust insulin doses (if necessary)
- Blood ketones

Assessments
- Blood pressure
- Heart rate
- Weight

Urine sample:
- Urine ketones

Medication
- Support participant's self-injection of open label semaglutide 0.25 mg if randomised to active treatment (dose on-site if dose is scheduled for visit day (see above)

7.5.2 Visit 5 (@2 weeks after first randomisation)

Clinical observations will be recorded. Blood glucose levels and ketone bodies will be measured via a fingerstick blood sample using glucose and ketones meter (see Section 7) and daily blood glucose levels collected and reviewed. Daily blood glucose data collected and reviewed and insulin doses adjusted as per Visit 3. Provided uptitration is considered appropriate by the site PI (or designee) (see also "Gastrointestinal adverse effects," page 31), the semaglutide dose will be increased to 0.5 mg weekly. This will be administered on-site if dose is scheduled for a visit day but can be self-administered at home (confirmed by a documented phone call) provided both research staff and participant are in agreement and there were no adverse effects following the previous dose. All subsequent trial doses of semaglutide can be self-administered on-site or at home as convenient and appropriate. Supply of IMP will be dispensed as appropriate to last until the next visit.

Visit Procedures

Monitoring and treatment optimisation
- Check for adverse events
- Review blood glucose diary
- Adjust insulin doses (if necessary)
- Blood ketones

Assessments
- Blood pressure
- Heart rate
- Weight

Urine sample:

- Urine ketones

Medication

- Dose titration (see above) if randomised to open-label semaglutide
- Return used study pen
- Dispense medication if randomised to open-label semaglutide (dose on-site if dose is scheduled for visit day– see above)

7.5.3 Visit 6 (@4 weeks after first randomisation)

For those randomised to open-label semaglutide, doses administered will be recorded (self-report). Clinical observations will be recorded. Daily blood glucose data will be collected and reviewed and insulin doses adjusted as per visit 3. Supply of IMP will be given to last until the next visit. A urine sample will be collected to evaluate ketone bodies and, in females randomised to IMP, pregnancy status.

Provided uptitration is considered appropriate by the site PI (or designee) (see also "Gastrointestinal adverse effects," page 31), the recommended semaglutide dose will be increased to 1.0 mg weekly (or 0.5 mg if not previously uptitrated from 0.25 mg). A 24 hr urine container will be dispensed to collect a 24 hour urine sample to bring in at the Week 13 visit.

Visit Procedures

Monitoring and treatment optimisation

- Check for adverse events
- Check adequacy of contraception (WOCBP)
- Review blood glucose diary
- Adjust insulin doses (if necessary)
- Blood ketones

Assessments

- Blood pressure
- Heart rate
- Weight

Urine sample:

- Urine ketones
- Urine pregnancy test (WOCBP randomised to IMP only)
- 24 hour urine container dispensed

Medication

- Record doses of semaglutide self-injected since last visit (self-report)
- Dose titration if randomised to open-label semaglutide (see above)
- Return used study pens
- Dispense medication if randomised to open-label semaglutide

7.5.4 Visit 7 (@13 weeks after first randomisation)

Participants will be asked to attend in the fasting state (no food or calorie intake for 10 hours). If participant is not fasting, samples should be taken and non-fasting status noted. For those randomised to open-label semaglutide, doses administered will be recorded (self-report). For those who have remained on doses lower than 1.0 mg weekly, this visit will be the last opportunity to uptitrate. Provided uptitration is considered appropriate by the site PI (or designee) (see also "Gastrointestinal adverse effects," page 31) the semaglutide dose will be increased from 0.25 mg to 0.5 mg weekly or from 0.5 mg to 1.0 mg weekly. Daily fingerstick blood glucose levels (diaries), CGM/ FGM data or insulin pump download data will be collected and reviewed and appropriate insulin dose adjustments made. Supply of IMP will be given to last until visit 10. Observations will be recorded. A urine sample will be collected to evaluate ketone bodies and, in females randomised to IMP, pregnancy status. The 24 hour urine container will be collected. A fasting blood sample will be taken (45 mL) to evaluate study endpoints.

Visit Procedures

Fasting visit*

Monitoring and treatment optimisation

- Check adequacy of contraception (WOCBP)
- Check for adverse events
- Review blood glucose diary
- Adjust insulin doses (if necessary)
- Blood ketones

Clinical assessments

- Blood pressure
- Heart rate
- Weight

Blood assessments

- Na, K, urea, creatinine, eGFR (MDRD)
- AST/ ALT/ Alkaline Phosphatase
- Calcium/ Phosphate
- FBC
- HbA1c
- Fructosamine
- Bilirubin
- Random glucose

Urine

- Pregnancy test (WOCBP randomised to IMP only)
- Urine ketones
- 24 hour urine bottle collected

Medication

- Record doses of semaglutide self-injected since last visit (self-report)
- Dose titration if randomised to open-label semaglutide (see above)
- **N.B. no further uptitration after this visit**
- Return used study pens
- Dispense medication if randomised to open-label semaglutide

7.5.5 Visit 8 (@23 weeks after first randomisation)

For those randomised to open-label semaglutide, doses administered will be recorded (self-report). Clinical observations will be recorded. Daily blood glucose data (fingerstick or FGM) will be collected and reviewed as per visit 3. Blood glucose levels and ketone bodies will be measured via a fingerstick blood sample. A urine sample will be collected to evaluate ketone bodies and, in females randomised to IMP, pregnancy status. Insulin doses will be adjusted if needed. All subjects will be reminded to monitor their capillary blood glucose by fingerstick at least 4 times a day (before meals and at bedtime) throughout the study. CGM sensor will be fitted and participants asked to wear their CGM constantly for next 20 days.

Visit Procedures

Monitoring and treatment optimisation

- Check adequacy of contraception (WOCBP)
- Check for adverse events
- Review blood glucose diary
- Blood ketones
- Adjust insulin doses (if necessary)

Clinical assessments

- Blood pressure
- Heart rate
- Weight

Urine sample:

- Pregnancy test (WOCBP randomised to IMP only)
- Urine ketones

Other assessments

- Fit and start masked Continuous Glucose Monitoring with Dexcom G6

7.5.6 Visit 9 (@25 weeks after first randomisation)

Participants will be asked to attend in the fasting state (no food or calorie intake for 10 hours). If participants arrive at the study visit in a non-fasted state, the visit will be terminated and a further fasting visit rearranged as soon as possible. Daily fingerstick blood glucose levels (diaries), CGM/ FGM data or insulin pump download data will be collected and reviewed and appropriate insulin dose adjustments made. Participants will discontinue use of the study CGM (Dexcom G6). Clinical observations will be recorded. Blood glucose levels and ketone bodies will be measured via a fingerstick blood sample (see Section 7). Supply of IMP will be given to last until the next visit. A 24 hr urine container will be dispensed to collect a 24 hour urine sample to bring in at visit 10 (Week 26) visit. A Meal Challenge Test will be performed as per visit 2. Participants will be provided with strips for urine or blood ketone measurement at home and instructed or reminded as appropriate on how to use these. They will be asked to measure urinary and blood ketones every morning for three consecutive days prior to the next visit (second randomisation: dapagliflozin or placebo) and to record the results in their diary.

Visit Procedures

Fasting visit*

Monitoring and treatment optimisation

- Check adequacy of contraception (WOCBP)
- Check for adverse events
- Review blood glucose diary
- Adjust insulin doses (if necessary)
- Blood ketones

Clinical assessments

- Blood pressure
- Heart rate
- Weight

Urine sample:

- Urine ketones
- 24 hour urine container dispensed

Other assessments

- Download Dexcom G6
- Meal Challenge Test

Blood assessments

- HbA1c

7.6 Second Randomisation Scheme

For the second randomisation (Period 2, double-blind), eligible and consenting participants previously randomized to open-label semaglutide will be randomised (1:1) to two groups (dapagliflozin or matching placebo) using permuted blocks of variable size to minimize predictability with stratification for HbA1c (≤ 7.5% vs >7.5% HbA1c) Those participants previously randomised to standard of care will continue as before.

7.6.1 Blinding (period 2 only)

For the double-blind treatment period, dapagliflozin and placebo will be packed in matching packs to maintain the blind. Each pack will contain sufficient supplies for 30 days' treatment with a small overage (excess). Packs will be labelled with a unique number that will be used to assign treatment to the participant via the IWRS system whilst maintaining the blind. Packs will be labelled in accordance with Good Manufacturing Practice and local regulatory requirements.

Unblinding. Treatment discontinuation (of dapagliflozin or placebo), rather than unblinding, will be carried out as far as possible. Participants will be advised in any case of hospitalisation with acute illness to discontinue study medication and inform the relevant clinician. These instructions will be reinforced in the Study Diary. However, where knowledge of treatment may assist emergency treatment, the investigator (or designee) may unblind using the IWRS system. Information on emergency unblinding (the investigational study drug, the study number, the investigator's name and a contact number) will be provided on the Patient Alert Card. For each unblinding an email alert is generated to the Study

Coordinator and Chief Investigator. The most likely scenarios for unblinding will be: confirmed pregnancy, overdose/ accidental ingestion, or development of acute renal failure

7.6.2 Visit 10 (Second Randomisation Visit; @26 weeks after First Randomisation)

Participants will be asked to attend in the fasting state (no food or calorie intake for 10 hours). For those who arrive in a non-fasted state, the visit will be terminated and a further fasting visit rearranged as soon as possible. Participants with normal fasting urine (<+) or blood ketone (<0.6 mmol) in the three days prior to randomisation will proceed to the next treatment phase as per this visit. The principles of the STICH Protocol (see Appendix 3) will be discussed (37). Participants with higher ketone values in at least one of the previous three days will have their glucose levels, insulin dose, and carbohydrate intake reviewed and advised on hydration, adequate CHO intake, and adequate insulin dosing. They will be rescheduled for one week and asked to repeat three days testing before the next visit.

Clinical observations will be recorded. A urine sample will be collected for ketones. Blood glucose or ketones will be measured via a fingerstick blood sample (see Section 7.2.1). Daily fingerstick blood glucose levels (diaries), CGM/ FGM data or insulin pump download data will be collected and reviewed and appropriate insulin dose adjustments made (following the guidance in Section 7, page 35). The previous three days blood or urine ketones levels will be reviewed. The adjusted total insulin dose at this visit will serve as the baseline total insulin dose for the remaining six months of the study and future limits of dose adjustments will be based on this new baseline. The blinded study CGM will be collected. The 24 hour urine container will be collected. A fasting blood sample will be taken (45 mL) to evaluate study endpoints. Participants will be asked to complete quality of life questionnaires.

At this point, those participants previously randomised to receive open-label semaglutide will be randomised to receive dapagliflozin 5 mg daily or matching placebo. They will be educated on symptoms suggestive of low blood pressure (e.g. light-headedness, dizziness, feeling faint).

Participants will be asked to perform a daily (preferably early morning) urine (using CombiScreen® 10 SYS Plus urine testing strips or equivalent) or blood tests for ketone bodies (using the particpant's own meters as per Section 7, "Minimising ketosis": page 28) and record all outcomes in dedicated study diaries. If results of these scheduled (i.e. not done due to symptoms) ketone measurements, are +1 or higher (urine) or > 0.6 mmol/L (blood), participants will be instructed to follow the STICH Protocol as per their study diary and Appendix 3, . For any abnormal ketone levels taken due to symptoms, the DKA consensus guidelines are to be followed instead (see Section 9.3).

Participants will be instructed to call site staff directly in case of any problem or untoward side effects. They will be specifically asked to call if they have confirmed hypoglycaemia (blood glucose levels < 3.9 mmol/L) or hyperglycaemia (blood glucose > 13.9 mmol/L) occurring on more than one occasion. Participants will be advised to remain well hydrated and report any genital mycotic or urinary tract infections

Visit Procedures

Fasting visit* - Requirements and arrangements as per visits 2 and 3.

Monitoring and treatment optimisation

- Check adequacy of contraception (WOCBP)
- Check for adverse events

- Review blood glucose diary
- Adjust insulin doses (if necessary) - this insulin dose is baseline for Period 2
- Blood ketones

Clinical assessments
- Blood pressure
- Heart rate
- Body weight

Quality of life assessments
- Diabetes Specific Quality of Life Scale (DSQOLS)
- Problem Areas In Diabetes (PAID) questionnaire

Blood assessments
- Na, K, urea, creatinine, eGFR (MDRD)
- AST/ ALT/ Alkaline Phosphatase
- Calcium/ Phosphate
- FBC
- Fructosamine
- Bilirubin
- Random glucose
- C-peptide (capillary blood glucose must be > 4.0 mmol/L at time of sampling)
-

Urine
- Pregnancy test (WOCBP randomised to IMP only)
- Urine ketones
- 24 hour urine bottle collected

Medication
- Education on symptoms suggestive of low blood pressure (e.g. light-headedness, dizziness, feeling faint) for those undergoing second randomisation
- Record doses of semaglutide self-injected since Visit 7 (self-report)
- Return used study pens
- Dispense open label semaglutide for participants randomised to active treatment at Period 1 (if required). Dispense dapagliflozin or placebo if randomised to active treatment

Other assessments
- Read masked Continuous Glucose Monitoring sensor (mean fasting blood glucose, mean blood glucose, percentage time in range, glycaemic variability)
-

7.6.3 Visit 11 (@1 week after Second Randomisation)

For those randomised to open-label semaglutide, doses administered will be recorded (self-report). Clinical observations will be recorded. Daily blood glucose and blood/ urine ketone levels will be reviewed and glucose levels collected. A urine sample will be collected to evaluate for ketones bodies. Blood glucose levels and ketones bodies will be reviewed measured via a fingerstick blood sample (see Section 7.2.1). . Daily fingerstick blood glucose levels (diaries), CGM/ FGM data or insulin pump download data will be reviewed and appropriate insulin dose adjustments made using the new baseline from Visit 10

(following the guidance in Section 7, page 35). If at any time total insulin dose reduction is >20% and total insulin dose is <0.40 U/Kg, additional monitoring (see Appendix 2) will be implemented. Unless blood (>0.6 mmol/L) or urine ketones (+ or more) have been elevated within the previous three days, dapagliflozin or placebo dose will be uptitrated to 10 mg daily (two capsules of either active or placebo medication) for the remainder of the study. Supply of IMPs will be given to last until week 39.

Visit Procedures

Monitoring and treatment optimisation

- Check adequacy of contraception.
- Check for adverse events
- Review blood glucose diary
- Adjust insulin doses (if necessary)
- Blood ketones
- Daily blood or urine ketones review

Clinical assessments

- Blood pressure
- Heart rate
- Body weight

Urine sample:

- Urine ketones

Medication

- Count returned dapagliflozin or placebo capsules
- Increase dose of dapagliflozin or placebo to 10mg (see above – review ketones)
- Dispense dapagliflozin or placebo (record dose)

7.6.4 Visit 12 (@5 weeks after Second Randomisation)

For those randomised to open-label semaglutide, doses administered will be recorded (self-report). Clinical observations will be recorded. Daily blood glucose and blood/ urine ketone levels will be reviewed and glucose levels collected. Blood glucose levels and ketone bodies will be measured via a fingerstick blood sample. A urine sample will be collected to evaluate ketone bodies and if appropriate pregnancy status. Insulin dose will be adjusted if as per Visit 11. A blood sample will be taken (15 mL) to evaluate study endpoints. A 24 hr urine container will be dispensed to collect a 24 hour urine sample to bring in at the Week 39 visit

Visit Procedures

Monitoring and treatment optimisation

- Check adequacy of contraception (WOCBP)
- Check for adverse events
- Review blood glucose diary
- Adjust insulin doses (if necessary)
- Blood ketones

Clinical assessments

- Blood pressure
- Heart rate
- Weight

Blood assessments

- Na, K, urea, creatinine, eGFR (MDRD)
- AST/ ALT/ Alkaline Phosphatase
- Calcium/ Phosphate
- FBC
- Bilirubin
- Random glucose

Urine

- Pregnancy test (WOCBP randomised to IMP only)
- Urine ketones
- 24 hour urine container dispensed

7.6.5 Visit 13 (@13 weeks after Second Randomisation)

For those randomised to open-label semaglutide, doses administered will be recorded (self-report). Participants will be asked to attend in the fasting state. If participant is not fasting, samples should be taken and non-fasting status noted. Clinical observations will be recorded. Daily blood glucose and blood/ urine ketone levels will be reviewed and glucose levels collected. Blood glucose levels and ketone bodies will be measured via a fingerstick blood sample. A urine sample will be collected to evaluate ketone bodies and if appropriate pregnancy status. The 24 hour urine container will be collected. Insulin dose will be adjusted if needed as in as per Visit 11. Sufficient supply of IMPs will be given until the end of the study. A fasting blood sample will be taken (45 mL) to evaluate study endpoints.

Visit Procedures

Fasting visit* - as per previous fasting visits

Monitoring and treatment optimisation

- Check adequacy of contraception (WOCBP)
- Check for adverse events
- Review blood glucose diary
- Adjust insulin doses (if necessary)
- Blood ketones
- Daily urine ketones review

Clinical assessments

- Blood pressure
- Heart rate
- Weight

Quality of life assessments

- Diabetes Specific Quality of Life Scale (DSQOLS)
- Problem Areas In Diabetes (PAID) questionnaire

Blood assessments

- Na, K, urea, creatinine, eGFR (MDRD)
- AST/ ALT/ Alkaline Phosphatase
- Calcium/ Phosphate
- FBC
- HbA1c
- Fructosamine
- Bilirubin
- Random glucose

Urine

- Pregnancy test (WOCBP randomised to IMP only)
- Urine ketones
- 24 hour urine bottle collected

Medication

- Record doses of semaglutide administered since Visit 10 (self-report)
- Dispense open label semaglutide for participants randomised to active treatment at Period 1 (if required).
- Count returned dapagliflozin or placebo capsules
- Dispense dapagliflozin or placebo (record dose)

7.6.6 Visit 14 (@23 weeks after Second Randomisation)

For those randomised to open-label semaglutide, doses administered will be recorded (self-report). Clinical observations will be recorded. Daily blood glucose and blood/ urine ketone levels will be reviewed and glucose levels collected. Blood glucose levels and ketone bodies will be measured via a fingerstick blood sample. A urine sample will be collected to evaluate ketone bodies and if appropriate pregnancy status. Insulin dose will be adjusted if as per Visit 11. All subjects will be reminded to monitor their capillary blood glucose by fingerstick at least four times a day (before meals and at bedtime). CGM (Dexcom G6) sensor will be fitted and patients asked to wear their CGM constantly for the next 20 days.

Visit Procedures

Monitoring and treatment optimisation

- Check adequacy of contraception (WOCBP)
- Check for adverse events
- Review blood glucose diary
- Adjust insulin doses (if necessary)
- Blood ketones
- Daily urine ketones review

Clinical assessments

- Blood pressure
- Heart rate
- Weight

<u>Urine sample</u>:
- Pregnancy test (WOCBP randomised to IMP only)
- Urine ketones

<u>Clinical and functional assessment</u>
- *Fit and start* masked Continuous Glucose Monitoring sensor (mean fasting blood glucose, mean blood glucose, percentage time in range, glycaemic variability)

7.6.7 Visit 15 (@25 weeks after Second Randomisation)

Participants will be asked to attend in the fasting state. If participants arrive at the study visit in a non-fasted state, the visit will be terminated and a further fasting visit rearranged as soon as possible. For those randomised to open-label semaglutide, doses administered will be recorded (self-report). Observations will be taken. Daily blood glucose and blood/ urine ketone levels will be reviewed and glucose levels collected. Blood glucose levels (fingerstick or FGM) and ketone bodies will be measured via a fingerstick blood sample. A urine sample will be collected to evaluate urine ketone levels. Insulin dose will be adjusted if needed as per visit 11. A 24 hr urine container will be dispensed to collect a 24 hour urine sample to bring in at the Week 52 visit. A HFHC Meal Challenge Test will be performed as in Visit 2.

<u>Visit Procedures</u>

Fasting visit* - Procedure as per previous fasting visits

<u>Monitoring and treatment optimisation</u>
- Check adequacy of contraception (WOCBP)
- Check for adverse events
- Review blood glucose diary
- Adjust insulin doses (if necessary)
- Daily urine ketones review
- Blood ketones

<u>Assessments</u>
- Blood pressure
- Heart rate
- Weight

<u>Urine sample</u>:
- Pregnancy test (WOCBP randomised to IMP only)
- 24 hour urine container dispensed

<u>Other assessments</u>
- Meal Challenge Test

7.6.8 Visit 16 (End of study; @26 weeks after Second Randomisation)

Participants will be asked to attend in the fasting state. If participants arrive at the study visit in a non-fasted state, the visit will be terminated and a further fasting visit rearranged as soon as possible. Clinical observations will be recorded. Daily blood glucose and blood/ urine ketone levels will be reviewed and glucose levels collected. Blood glucose levels and ketone bodies will be measured via a fingerstick blood sample. Insulin doses will be reviewed and likely requirement for an increase following discontinuation of IMP discussed with those taking open-label semaglutide. A urine sample will be collected to evaluate ketone bodies and, if appropriate pregnancy status. The CGM will be collected. The 24 hour urine container will be collected. A fasting blood sample will be taken (45 mL) to evaluate study endpoints. We will ask the participants to complete quality of life questionnaires. A physical examination will be performed as per the screening visit. This will be the final face-to-face study visit. A safety phone call will be conducted two weeks to review blood glucose data, provide further insulin dosing advice, to document any adverse events, and to ensure that follow-up in usual clinical care has been arranged.

Visit Procedures

Fasting visit*

Monitoring and treatment optimisation

- Check for adverse events
- Review blood glucose diary
- Adjust insulin doses (if necessary)
- Blood ketones
- Daily urine ketones review

Clinical assessments

- Blood pressure
- Weight
- Heart rate
- Physical examination

Quality of life assessments

- Diabetes Specific Quality of Life Scale (DSQOLS)
- Problem Areas In Diabetes (PAID) questionnaire

Blood assessments

- Na, K, urea, creatinine, eGFR (MDRD)
- AST/ ALT/ Alkaline Phosphatase
- Calcium/ Phosphate
- FBC
- HbA1c
- Fructosamine
- Bilirubin
- Random glucose
- C-peptide

Urine

- Pregnancy test (WOCBP randomised to IMP only)
- Urine ketones
- 24 hour collection

Other

- Record doses of semaglutide administered (self-report)
- Return used study pens
- Count returned dapagliflozin or placebo capsules
- Collect Patient Alert Card
- Read masked Continuous Glucose Monitoring sensor (mean fasting blood glucose, mean blood glucose, percentage time in range, glycaemic variability)

7.7 Retention and strategies for maximizing follow-up

Participants will be encouraged to attend all study visits. Prior to commencing the trial, all potential participants will have the opportunity to read the detailed Patient Information Sheet which will outline the level of participation requested. Potential participants will be made aware of the need for frequent study visits and asked to commit to these.

Study visits can be rearranged flexibly within a period around the planned visit as specified in the timeline of visits above (Section 7) and actual dates recorded in the eCRF. This is to maximise participant ability to attend all visits. A longer or shorter interval does not constitute a protocol deviation. Where possible, visits will be arranged to coincide with other appointments to further maximise attendance and participant convenience. Telephone contact will be frequent and will help to maintain contact with participants and ensure timely reminders of upcoming study visits can be given.

Even in the scenario of treatment discontinuation (of either IMP), participants will be encouraged to continue to attend further study visits as scheduled.

As stated in point 3 of the Schedule of Assessments, telephone visits are allowed for V4, V5 and V11 for those randomised to the standard of care arm. Participants who have discontinued both study IMPs (see below Section 7.8 for criteria) can also be offered telephone visits for V4, V5 and V11 if this is preferable and encourages retention in the study.

7.8 Treatment Interruptions and Withdrawal criteria

Treatment Interruptions

Treatment interruption from study IMPs may be necessary in some participants. In the case of dapagliflozin, this will occur in any acute illness or hospitalisation which increases the risk of ketosis and ketoacidosis. Circumstances in which temporary discontinuation of dapagliflozin or placebo should occur will be discussed with all study participants. Written information will be provided in the study diaries. An emergency phone line to the study team will be available to all participants for the duration of their participation in the trial. This will be operational during office hours (Monday to Friday 9am – 5pm). The Patient Alert Card also contains contact details for unblinding in the event of acute intercurrent illness and a requirement for unblinding in the opinion of the acute medical team treating the participant. If any participant develops diabetic ketoacidosis (DKA) as defined as per the International Consensus

guidelines on two occasions with no identifiable precipitant (section 2.1) they will be withdrawn from the study and the UK Chief and US Principal Investigators informed.

Any other intolerable side effects, acute illness, or other clinical change which may impact participant safety (e.g. excessive weight loss) may require temporary discontinuation of the study drug(s) (dapagliflozin/placebo or semaglutide). In these circumstances, any decision to recommence the study drug(s), the dose and the appropriateness of re-uptitration, will be taken in consultation with the participant, including consideration of the total participant burden, particularly given the need for additional safety checks. If in the judgement of the site PI the study drug(s) cannot be safely restarted, if side-effects remain intolerable or if any of the criteria for study drug withdrawal are met, the treatment with the study medicine(s) must be permanently stopped. Participants who have withdrawn from treatment will be encouraged to continue to attend study visits.

Criteria for withdrawal

Treatment withdrawal or withdrawal from the trial as appropriate (see below) may be necessary prior to the planned end at 52 weeks following first randomisation for any of the following reasons:

1. The site PI can stop investigational study treatment or remove participants from the trial without participants approval if for any reason they feels is appropriate, including: severe side effects, injury or medical condition which may place the patient at risk of further complications if he/she continue to participate, failure to take the medication as instructed, failure to keep scheduled appointments, insufficient level of self-monitoring, cancellation of the study or other administrative reasons.

2. Any woman who becomes pregnant during the period of study will be withdrawn from the trial.

3. Participants who experience persistent severe nausea or vomiting after starting semaglutide which is not managed by dose reduction will discontinue the investigational study treatment. Those with mild or moderate nausea will continue for at least one more week. For those who continue to experience significant nausea (defined as interfering with normal activities), dose will be reduced: if these side effects persist, they will discontinue study medication(s). Participants for whom this occurs in Period 1 will remain eligible for randomisation in Period 2.

4. Excessive weight loss during either period of the study despite treatment dose reduction (> 15% body weight or BMI < 22 kg/m^2 on repeated measurements more than four weeks apart) will lead to treatment withdrawal. If the participant is willing, they may continue to attend study visits (at the discretion of the site PI).

5. New diagnosis of other conditions included in the Exclusion Criteria will result in withdrawal of the participant from the trial.

6. Change in method of insulin delivery: any participant in whom insulin delivery is changed from CSII to MDI or vice versa during the study will be withdrawn from the trial unless pump treatment interruption is temporary for a period of at most 2 weeks. Participants who upgrade to “artificial pancreas” (e.g. Medtronic’s MiniMed 670G hybrid) or any form of hybrid closed-loop therapy during the study will be withdrawn from the trial.

7. Hypoglycaemia: any participant who experiences two episodes of severe hypoglycaemia (requiring external intervention) will be withdrawn from the trial.

8. Ketosis (with no other identifiable precipitating factors): any participant who experiences persistent (1 week) asymptomatic or symptomatic and unmanaged (using STICH protocol – see section 2.1 b) blood ketone levels >1.5 mmol (or ≥+2 in urine) will be withdrawn from the trial. (37)

9. Insulin dose: any participant who experiences a persistent (>1 week) total insulin dose reduction of >20% from baseline AND total insulin dose <0.4U/Kg in Period 2 will be withdrawn from the trial.

10. DKA: any participant who experiences two episode of DKA (with no identifiable precipitating factors) as defined by the expert consensus statement will be withdrawn from the trial (39)

11. UTI: any participant who experiences two episodes of UTI will be withdrawn from the trial.

12. Genital mycotic infection: any participant who experiences two episodes of mycotic genital infection with severe symptoms will be usually be withdrawn from the trial (see also heading "Genital mycotic infections," page 32).

13. Any participant who experiences significant persistent reduction in eGFR > 15 ml/min will be withdrawn from the trial.

14. Pancreatitis: any participant who experiences an episode judged to be due to pancreatitis by the site PI will be withdrawn from the trial.

15. Participants who experience other intolerable and persistent side effects specific to study drugs will be offered treatment downtitration or treatment withdrawal with continued follow-up within the trial if they are willing - except when this is considered inappropriate by the site PI, in which case they will be withdrawn.

Participation in this study is voluntary. Participants have the right to refuse to participate or to withdraw from participation at any time for any reason. The site PI can stop investigational study treatment or remove participants from the trial without participant's approval. If a patient withdraws consent and decides not to participate in the study or is withdrawn from the trial according to the above criteria, she/he will be asked to undergo a final study visit for his/her safety. This visit will follow the same procedures for Visit 16 provided there is ongoing consent. The research team will arrange appropriate handover to the usual clinical team – additional telephone (of if necessary face to face) contact can be arranged if necessary in the interim. If a subject withdraws from the research, the data collected to that point will be used toward the research finding, including study data collected at final study visit, provided there is ongoing consent. If applicable the subject will be asked to return any unused research drug and or devices.

7.9 Storage and analysis of samples

Blood samples will be undertaken at the beginning and at various points during the study as outlined in detail in the schedule of visits. If blood testing has recently been undertaken in the 3 weeks prior to the study commencing, some of these blood tests may be used as baseline. Blood tests will include analysis of the full blood count, renal function (urea and electrolytes), liver function tests, bone profile, lipid profile and several specialised blood tests for biomarker assays (at central laboratory) following a high fat high caloric meal challenges to examine study endpoints.

Covid-19 Mitigation

All blood sampling or trial procedures will be undertaken following local and national guidance around personal protective equipment. Mask, gloves and aprons will be used as per current guidelines.

7.9.1 Sample collection and processing

- Samples will be appropriately labelled in accordance with the trial procedures to comply with the General Data Protection Regulation (GDPR). Biological samples collected from participants as part of this trial will be transported, stored, accessed and processed in accordance with national legislation relating to the use and storage of human tissue for research purposes and such activities shall at least meet the requirements as set out in the 2004 Human Tissue Act and the 2006 Human Tissue (Scotland) Act.
- Blood will be taken according to the Schedule of Assessments (page 17). Plasma will be siphoned, aliquoted and stored locally at -80° ± 10°C until transport in batches to the central laboratory on dry ice. according to the Sample Handling Procedure as agreed by the Joint Study Management Group
- Individual patient samples will be identified with a unique, anonymised study number.

7.9.2 Sample transport to central laboratory and analysis

- Samples will be transferred to State University of New York in a single batch at the end of recruitment to the study. More frequent transfer can be organised if there are local storage limitations.
- Samples will be transported on dry ice and stored at the central laboratory at -80° ± 10° until analysis. Transport by courier will coordinated by the Trial Manager.
- At the time of analysis individual aliquots will be defrosted at room temperature and analysed in a single batch for each biomarker of interest.
- Samples will be stored at -80° ± 10° in the central laboratory for possible future analysis for novel biomarkers.

7.10 End of trial

The end of the trial will be following the last study visit of the last subject.
At the end of the specified trial period (approximately 52 weeks following first randomisation) all participants will discontinue study medications and revert to standard of care (insulin therapy only). This process will be supervised by the trial physicians, research nurses, and research pharmacists under the supervision of the site Principal Investigator. The trial team will liaise with the usual care team to ensure a safe transition back to standard therapy. Following the final scheduled trial visit there will be a safety phone call two weeks after the last visit. This will act as a further opportunity to advise on insulin dosing and ensure there are no ongoing problems or concerns related to the study.

As discussed in section 7.8, there may be circumstances in which trial drugs need to be stopped before the specified end of the trial. If this is the case, the reason(s) for discontinuation will be recorded and the trial clinical team will facilitate safe transition back to usual standard of care when appropriate. Participants will however if eligible (i.e non-exclusion criteria reason for IMP

discontinuation – as listed in Section 7.8) be asked to continue with trial visits. If consent is withdrawn and participants voluntarily choose to leave the trial before the specified end date, all efforts by the clinical trial team will be made to safely facilitate transition back to ordinary standard of care. A follow up safety visit will be offered to all such participants and ongoing contact details will be made available should there be any further unforeseen issues relating to the clinical trial.

The trial may be stopped at an earlier stage than anticipated on the advice of the independent data monitoring committee (IDMC) to the trial steering committee (TSC) for the safety reasons detailed on page 12 and in the study safety endpoints. If this occurs, all participants will be informed and safe discontinuation of study medications will be undertaken under the supervision of the site Principal Investigator.

8 TRIAL MEDICATION

8.1 Name and description of investigational medicinal product(s)

a) Semaglutide (Ozempic®): a once weekly GLP-1 agonist injection indicated for type 2 diabetes (approved by the Scottish Medicines Consortium, January 2019).

   Doses to be used: 0.25mg increased to 0.5mg at two weeks and then to 1mg weekly s.c. injection after a further two weeks. Open label semaglutide will be added to a background of insulin treatment at week 0 for 12 months in 76 participants (38 in the UK) according to the first randomisation; at week 26 they will be further randomised (double-blind) as below into two groups: dual (placebo, semaglutide and insulin) and triple (dapagliflozin, semaglutide and insulin) therapy.

b) Dapagliflozin (as dapagliflozin propanediol monohydrate Forxiga®): a once daily SGLT-2 inhibitor that will be administered orally in a blinded fashion.

   Doses to be used: 5 mg daily increased after one week to 10 mg daily (orally). Dapagliflozin will be started according to the randomisation (Interactive Web System, hosted by University of Glasgow) in 38 participants (19 in the UK) at 26 weeks as an additional treatment on a background of insulin and semaglutide therapy and end at 52 weeks (triple therapy group). Doses to be used: 5 mg increased to 10 mg after one week.

c) Placebo to dapagliflozin: Dapagliflozin matched placebo will be started in 38 participants (19 in the UK) participants at 26 weeks as an additional treatment on a background of insulin and semaglutide therapy and end at 52 weeks (dual therapy group). Doses to be used: as for active dapagliflozin.

8.2 Legal status of Semaglutide and Dapagliflozin

Semaglutide is currently licensed in the UK for those with adult type 2 diabetes which is insufficiently controlled.

8.3 Drug storage and supply

Semaglutide and dapagliflozin/placebo supplies must be stored in the original containers in a locked, secure area with access limited to authorised site staff. Study supplies should be used as directed in the study protocol and not be supplied to any persons other than study participants. All IMPs will be dispensed to participants from the study site pharmacy.

Covid-19 Mitigation

As detailed above in section 7, the supply of IMP may be altered to reduce risk of Covid-19 transmission in the event of future waves of infection. This may involve providing increased quantities of IMP to participants to last longer and therefore minimise physical contact with trial personnel. Use of a courier delivery service to facilitate this may also be considered.

Semaglutide: Supplies of semaglutide pre-filled pens for subcutaneous administration will be sourced from routine hospital stocks. The following semaglutide presentations will be required:

- Semaglutide 0.25 mg: Each pre-filled pen contains 2mg semaglutide in 1.5ml. Each dose contains 0.25mg of semaglutide in 0.19ml solution.
- Semaglutide 0.5 mg: Each pre-filled pen contains 2mg semaglutide in 1.5ml solution. Each dose contains 0.5 mg of semaglutide in 0.37ml solution.
- Semaglutide 1 mg: Each pre-filled pen contains 4 mg semaglutide in 3.0ml solution. Each dose contains 1mg semaglutide in 0.74ml solution.

For all presentations each pre-filled pen is capable of delivering 4 doses which is sufficient for 4 weeks treatment. Pens are for individual participant use only. In the event of a pen-failure, a new pen should be supplied to the participant.

Prior to first use, semaglutide pens must be stored at 2-8 °C in the original container and must not be frozen. Supplies which are frozen either prior to first use or during use must be discard.

Semaglutide pre-filled pens have an in-use shelf life of 6 weeks. After first use the pre-filled pens may be stored below 30oC or in a refrigerator at 2-8 °C. Participants should be advised to store packs away from the cooling element. The pen cap should be on when the pen is not in use in order to protect the contents from light.

Wherever possible, supplies of semaglutide pre-filled pens should be utilised in a pragmatic way so as to minimise unnecessary wastage. Participants should be provided with an appropriate number of pen(s) to last until the next planned dispensing visit. A small overage may be given as necessary to accommodate visit windows etc. The correct strength of pen must be supplied for each dose titration i.e. 0.25mg, 0.5mg and 1.0 mg as tolerated.

A web-based system will be used to randomise participants to semaglutide treatment.

Dapagliflozin 5 mg or placebo capsules: Supplies of dapagliflozin 5mg and matched placebo capsules will be manufactured by NHS Scotland Pharmaceuticals Specials Service/NHS Tayside Pharmaceuticals in accordance with Good Manufacturing Practice requirements. Capsules will be packed into HDPE bottles with a tamper evident seal. Each bottle will be labelled in a double-blind fashion to meet local regulatory requirements and will be assigned a unique number. Bottles containing dapagliflozin 5 mg or placebo must be stored at room temperature at 15-25°C.

Dapagliflozin 5 mg and placebo capsule supplies will be provided to study site and will only be released by the sponsor once all the appropriate regulatory and governance approvals are in place.

Further information on storage requirements and supply arrangements is provided in the study specific IMP Management and Accountability Manual.

A web-based system will be used for the assignment of dapagliflozin 5mg or placebo bottles at randomisation and at study visits where there is planned dispensing of the IMP or in the event that a participant requires replacement medicines.

Other medicines: Supplies of all other medicines and associated consumables will via usual NHS supply mechanisms.

8.4 Drug accountability requirements

The Investigator or designee must maintain accurate records of all study IMP movements for accountability purposes. They should include dates, quantities, batch numbers and expiry. Records must document adequately that:

- the patients were provided the doses specified by the protocol/amendment(s)
- all study drug provided was fully reconciled.

Unused study drug must not be discarded or used for any purpose other than the present study. Further information is provided in the study specific IMP Management and Accountability Manual.

8.5 Preparation and administration of semaglutide and dapagliflozin

Semaglutide (Ozempic®) is a solution for subcutaneous injection. It is administered once weekly, on the same day each week, into the subcutaneous tissues of the abdomen, thigh or upper arm. A participant may change the day of their weekly injection as long as it has been at least three days since their last injection. Thereafter, all doses should be administered once weekly on the new dosing day. The injection can be administered at any time of the day regardless of meals.

Participants must be advised to discard the injection needle after each injection and store the pen without an injection needle attached. This may help prevent blocked needles, contamination, infection, leakage of solution and inaccurate dosing. Semaglutide should not be used if the solution does not appear clear and colourless or almost colourless. The pen is designed to be used with NovoFine or NovoTwist disposable needles and pens are packed with an appropriate quantity of NovoFine Plus needles.

Detailed information on administration of semaglutide is provided in the current Summary of Product Characteristics and product Patient Information Leaflet.

Dapagliflozin (Forxiga®))and matched placebo are presented as a capsule for once daily oral administration.

8.6 Dosage schedules

Semaglutide will be administered and dosed as described. Participants will be commenced on 0.25 mg s.c once weekly before up-titration as tolerated to 1.0 mg s.c once weekly. Up-titration is quicker than is ordinarily the case in clinical practice in type 2 diabetes as discussed in Section 2.1 (22) There will be increased observation and clinical access for participants in the event of side-effects. Participants randomised to receive semaglutide will continue on the 1.0 mg s.c once weekly dose for the remainder of the study following uptitration provided they tolerate the dose as detailed in Section 7. If necessary, the dose may be adjusted.
If a participant misses a dose of semaglutide it should be administered as soon as possible and within 5 days after the missed dose. If more than 5 days have passed, the missed dose should be skipped, and

the next dose should be administered on the regularly scheduled day. In both cases participants, should then resume their regular once weekly dosing schedule.

Dapagliflozin and matched placebo will be commenced at 5mg orally once daily and uptitrated one week after commencement to a dose of 10mg which will thereafter be continued for the remainder of the trial provided they tolerate the dose. If necessary, the dose may be adjusted.

8.7 Dosage modifications

Dapagliflozin/placebo will be commenced at one capsule (5mg or placebo) orally once daily and uptitrated one week after commencement to a dose of two capsules (10mg or placebo) which will thereafter be continued for the remainder of the trial provided they tolerate the dose. If necessary, the dose may be adjusted. (See also Section 7.8 regarding treatment discontinuation) Dapagliflozin or placebo capsules can be taken at any time of day with or without food. Capsules should be swallowed whole. If participants forget to take a dose they should be advised as follows:

- If it is 12 hours or more until the next dose, the dose should be taken as soon as remembered and the next dose taken at the usual time.
- If it is less than 12 hours until the next dose, the missed dose should be skipped. The next dose should be taken at the usual time.
- Participants should not take a double dose of dapagliflozin/placebo to make up for a forgotten dose.

8.8 Known drug reactions and interaction with other therapies

Participants with hypersensitivity to either active study medication are excluded from the trial. There are no significant interactions between semaglutide and other prescribed medications other than with agents that can lower blood glucose levels. Participants on any other oral agents which can lower blood glucose levels meet Exclusion Criteria and will not be included in this trial. In combination with insulin and dapagliflozin, semaglutide may be associated with an increased risk of hypoglycaemia. Participants will be advised of the increased risk of hypoglycaemia and will be required to closely monitor their blood glucose levels for the duration of the trial. No interactions are currently listed between warfarin and semaglutide in the British National Formulary but in view of a theoretical interaction (based on protein binding), existing arrangements for INR testing will be reviewed for any participants prescribed warfarin and additional tests conducted if indicated according to the judgement of the investigators.

Dapagliflozin is known to increase the risk of hypoglycaemia when combined with other medications which also act to lower blood glucose levels. Dapagliflozin has a mild diuretic effect and therefore there is an increased risk of hypotension when dapagliflozin is combined with either medications which deplete circulating volume (e.g diuretics) or antihypertensive drugs. Dapagliflozin is however commonly prescribed with such medications in general medical practice. All participants will have their medications reviewed at the screening visit and educated regarding symptoms that may indicate dehydration/ hypovolaemia.

Dapagliflozin may increase renal lithium excretion leading to decreased blood lithium concentrations. Serum lithium concentrations should be monitored for participants eligible for randomisation to dapagliflozin/placebo prior to visit 10 (week 26, second randomisation), as clinically indicated thereafter, and in the event of dose changes, taking into account the planned dose increase at visit 11 (week 27).

If there is any concern of interaction with any of the IMPs, this will be discussed with the PI before a decision regarding their ability to participate is made.

COVID-19: COVID-19 infection and the clinical management in this therapeutic area is also rapidly evolving as new medicines are approved for use by local/national treatment guidelines. It is therefore not possible to comprehensively rule out the potential for clinically significant interactions between the study IMP(s) with current or future medicines for the acute treatment of COVID-19 and a pragmatic approach is required. Medicines for acute COVID-19 infection should not be unnecessarily withheld and participants should be managed wherever possible in line with local/national clinical guidelines for this patient group. The decision to follow a particular management plan for acute COVID-19 infection (which may include the temporary withdrawal of the IMP(s) should this be in the participant's best interest), is at the treating physician's discretion. Investigators should refer to the protocol and current IMP Summary of Product Characteristics for detailed information on known mechanisms for pharmacokinetic/pharmacodynamics interactions as well as the relevant COVID-19 treatment Summary of Product Characteristics (or equivalent) to inform management decisions. Any mitigations, such as additional patient monitoring, must be documented in the participant's medical notes.

Further ad-hoc advice can also be obtained from the Sponsor/Chief Investigator and https://www.covid19-druginteractions.org/ Investigators must be vigilant at all times to the possibility of interactions and report in line with protocol pharmacovigilance requirements. Concomitant use of other medicines for COVID-19 must be recorded in the participant's CRF. Investigational treatments for acute COVID-19 treatment are not permitted without prior approval from the sponsor.

8.9 Concomitant medication

Participants should not receive any adjunct glucose-lowering therapy other than as per the randomisation schedule (e.g. with either of the active study medications).

8.10 Trial restrictions

Contraindications will follow SmPC information for Ozempic® and Forxiga. Acceptable forms of contraception for WOCBP participating in the trial are listed in Appendix 2. There is no requirement for contraception for male participants.

8.11 Assessment of compliance

Treatment adherence will be assessed by the trial medical and nursing team when participants attend for scheduled study visits.

8.12 Name and description of each Non-Investigational Medicinal Product (NIMP)

Insulin therapy is a non-investigational medicinal product in this trial. The trial is in a population who have had a diagnosis of type 1 diabetes for at least one year: therefore all participants will already be established on insulin therapy. The regimen and dosage required will by differ between participants. Participants will be using a combination of long acting (basal) insulin and shorter acting (bolus) insulin or CSII (insulin pump). As in standard of care, dose titration will depend on diet, blood glucose levels and exercise. It is anticipated that dosage adjustments will be required to achieve optimal glycaemic control and maintain participant safety.

There are pre-defined guidelines for insulin titration in this study. Insulin dose reductions should not be greater than 10% (5% if HbA1c is<7%) from current total insulin dose or more than 20% from the baseline level at randomisation and then from the new baseline levels at 26 weeks following second randomisation. Participants requiring an insulin dose reduction > 20% to a total insulin <0.4 U/kg will

be monitored closely for insulin dose, carbohydrate intake, ketones and blood glucose. Study medication can be temporarily discontinued, if necessary, for one-to-two weeks. If low insulin requirements remain, the participant will be withdrawn from study (see Section 7.8).

9 PHARMACOVIGILANCE

9.1 Definitions

Term	Definition
**Adverse Event (AE)**	Any untoward medical occurrence in a participant to whom a medicinal product has been administered, including occurrences which are not necessarily caused by or related to that product.
**Adverse Reaction (AR)**	An untoward and unintended response in a participant to an investigational medicinal product which is related to any dose administered to that participant.<br>The phrase "response to an investigational medicinal product" means that a causal relationship between a trial medication and an AE is at least a reasonable possibility, i.e. the relationship cannot be ruled out.<br>All cases judged by either the reporting medically qualified professional or the Sponsor as having a reasonable suspected causal relationship to the trial medication qualify as adverse reactions.
**Serious Adverse Event (SAE)**	A serious adverse event is any untoward medical occurrence that:<br>1. results in death<br>2. is life-threatening<br>3. requires inpatient hospitalisation or prolongation of existing hospitalisation<br>4. results in persistent or significant disability/incapacity<br>5. consists of a congenital anomaly or birth defect<br>Other 'important medical events' may also be considered serious if they jeopardise the participant or require an intervention to prevent one of the above consequences.<br>NOTE: The term "life-threatening" in the definition of "serious" refers to an event in which the participant was at risk of death at the time of the event; it does not refer to an event which hypothetically might have caused death if it were more severe*.
**Serious Adverse Reaction (SAR)**	An adverse event that is both serious and, in the opinion of the reporting Investigator, believed with reasonable probability to be due to one of the trial treatments, based on the information provided.
**Suspected Unexpected Serious Adverse Reaction (SUSAR)**	A serious adverse reaction, the nature and severity of which is not consistent with the information about the medicinal product in question set out:<br>• in the case of a product with a marketing authorisation, in the summary of product characteristics (SmPC) for that product<br>• in the case of any other investigational medicinal product, in the investigator's brochure (IB) relating to the trial in question
**Reference Safety Information**	The information used for assessing whether an adverse reaction is expected. This is contained in either the investigator's brochure or the summary of product characteristics

*Note: “Severe” is often used to describe intensity of a specific event, which may be of relatively minor medical significance. “Seriousness” is the regulatory definition supplied above.

9.2 Operational definitions for (S)AEs

Adverse events (AEs) will be recorded, notified, assessed, reported, analysed and managed in accordance with the Medicines for Human Use (Clinical Trials) Regulations 2004 (as amended) and this protocol.

All Serious Adverse Events (SAEs) occurring during the trial will be recorded within the eCRF. Non-serious AEs occurring during the trial will be recorded within the eCRF if they are considered to be related to trial medication, i.e. adverse reactions (ARs).

Exclusions from SAE reporting

Hospitalisation for the following reasons will not be considered to be SAEs:

- Treatment which was elective or pre-planned, for a pre-existing condition not associated with any deterioration in condition, e.g. pre-planned hip replacement operation which does not lead to further complications.
- Any admission to hospital or other institution for general care for less than 24 hours where there was no deterioration in condition.
- Treatment on an emergency, outpatient basis for an event **not** fulfilling any of the definitions of serious as given above and not resulting in hospital admission.
- COVID-19 infection unless results in severe illness with hospital admission.

9.3 Recording and reporting of AEs, Adverse Events of Special Interest, SAEs and SUSARs

All AEs occurring during the trial that are observed by the Investigator or reported by the participant will be recorded in the participant’s medical records whether or not attributed to trial IMP. AEs will be recorded from randomisation until the later of 30 days post cessation of trial treatment or the end of the study.

With the exception of those noted in section 9.3.1 below, adverse reactions will be recorded in a log provided in the eCRF.

Adverse Events of Special Interest, SAEs and SUSARs will be recorded in the participant’s medical records and in a specific section of the eCRF from the point of randomisation until the later of 30 days post cessation of trial treatment or the end of the study.

9.3.1 Adverse Events of Special Interest

Ketosis is a known adverse effect of dapagliflozin (see Section 2.1b; also (i) below). Hypoglycaemia is a recognised adverse effect with both semaglutide and dapagliflozin when used in combination with insulin [see (ii) below] and is defined as moderate in intensity (Level 2) when blood glucose is < 3.0 mmol/L. Thus ketosis and Level 2 hypoglycaemia events will be recorded as Adverse Events of Special Interest (AESIs).

AESIs will be recorded within the relevant section of the eCRF but do not require reporting in an expedited manner (unless the event also meets the regulatory definition of an SAE). The following information will be recorded:

- Nature of the event
- Event duration (start and end dates, if applicable
- Relationship to study drug
- Outcome

Adverse Events of Special Interest will be monitored and followed up (if applicable) until the event has resolved or a final outcome has been reached.

All Adverse Events of Special Interest must be assessed for seriousness prior to recording within the eCRF. Those meeting the definition of an SAE should also be reported accordingly as per Section 9.3.2.

(i) Ketosis and diabetic ketoacidosis

This trial will be conducted in participants who have type 1 diabetes. All participants will be taking insulin. In this condition, due to the near absence of endogenous insulin, at a cellular level there is an inability to incorporate glucose into normal intra-cellular energy yielding pathways. In this context, alternative energy yielding pathways are sought including ketone body production from fatty acids in the liver. Ketones are clinically important as they easily dissociate hydrogen ions upsetting physiologically regulated acid base balance. As ketone levels rise, there is initially a state of high blood ketone levels without significant acidosis (ketosis); if this develops further, diabetic ketoacidosis (DKA) may develop. DKA requires urgent medical input. Ketones can be easily measured via a simple urine test or fingerstick capillary blood measurement. Participants eligible for this trial will already be trained in how to do this. As discussed in Background ((1) above), dapagliflozin, one of the IMPs in this trial, is known to increase the risk of ketosis and ketoacidosis and this can occur at lower blood glucose levels than in those who are on insulin treatment only [euglycaemic DKA] (13,14). Given this known risk, ketosis and DKA are included in the safety endpoints and as such will be recorded in the eCRF as Adverse Events of Special Interest.

DKA is defined clinically and biochemically as a combination of a state of elevated ketone levels (urine ++ or higher, and/or blood > 1.5 mmol/L) in combination with biochemical evidence of a state of metabolic acidosis (pH ≤7.30 or serum bicarbonate < 15 mmol/L). This is as defined by International Consensus Guidelines (39). DKA also varies in severity. In clinical practice the diagnosis is made when a person with type 1 diabetes has elevated ketone levels and usually – although not exclusively – elevated blood glucose levels. The diagnosis is made biochemically by assessing acid base balance through blood testing. This corresponds to the following severity classifications:

- mild DKA: pH 7.25-7.30 (H^+ 50 – 56) or bicarbonate (15 -17.9 mmol/L)
- moderate DKA: pH 7.00-7.24 (H^+ 57 – 100) or bicarbonate 10 – 14.9 mmol/L)
- severe DKA: pH <7.00 (H^+ > 100) or bicarbonate < 10 mmol/L (39)

In addition to the above, the highest level of either blood or urine ketones measured, the serum bicarbonate (if measured) and blood pH (if measured) will be recorded for each episode. Given the known risk of DKA, all participants will be made aware of this condition. Patient diaries and Patient Alert Cards will summarise advice for management of high ketone levels including an emergency ketosis management protocol (STICH Protocol – see Appendix 3) (37). All participants will be

educated as to the scenarios when this should be activated and this will also be included on the Patient Alert Card which will have the contact details of the study team and also contact details for out of hours health services. Participants will be asked to carry this card at all times.

(ii) Hypoglycaemia

Hypoglycaemia is a low blood glucose level. Hypoglycaemia occurs in diabetes owing to difficulty in precisely matching insulin dosing with the complex factors associated with carbohydrate intake and physical activity. When too much insulin relative to carbohydrate intake is administered, blood glucose levels can drop to a level which is too low. Most people with type 1 diabetes can recognize this phenomenon due to a combination of symptoms which alert them and prompt self-checking of their capillary blood glucose level. Symptoms include weakness, malaise, headache, tremulousness, diaphoresis, palpitations and mild confusion. These are known as autonomic symptoms as they are mediated by activation of the autonomic nervous system as a response to the biological stress of low serum blood glucose. As hypoglycaemia progresses, however, symptoms progress to include confusion and agitation. If untreated this can lead to coma. These symptoms are known as neuroglycopenic symptoms as they are mediated by a lack of glucose in neural cells leading to a confused state.

Hypoglycaemia is also well defined by International Consensus (39). Like DKA, it can be classified in terms of severity. This can be based either on the absolute capillary glucose value and/or clinical features and is classed into stages:

- Level 1 (mild) hypoglycaemia: Blood glucose is 3.0 – 3.8 mmol/L inclusive. At each visit the number of episodes since the last visit during which blood glucose was within this range (but not lower) will be recorded on the "Fasting Blood Glucose" tab of the eCRF following review of the participant's study diary and (if applicable) FGM/ CGM download.
- Level 2 (moderate) hypoglycaemia: Blood glucose is < 3.0 mmol/L At each visit the number of episodes since the last visit during which blood glucose was within this range at any time will be recorded on the AESI tab of the eCRF following review of the participant's study diary and (if applicable) FGM/ CGM download.
- Level 3 (severe) hypoglycaemia: A person is unable to function because of mental or physical changes due to hypoglycaemia and requires assistance from another person to recover. These events will be recorded as SAEs (see below) independent of recording of blood glucose according to the opinion of the site Principal Investigator as by definition they jeopardise the participant or require an intervention to prevent life-threatening consequences.

The IMPs in this trial are licensed clinically in type 2 diabetes to lower blood glucose levels. It therefore follows that when used in combination with insulin there may be an increased risk of low blood glucose levels. This was the case with the once daily GLP-1 analogue liraglutide in phase 3 trials (8,9), but not in the trials with dapagliflozin (13,14). All participants will have this risk discussed with them, will be performing increased blood glucose monitoring than is the case in usual care, and will be frequently reviewed to adjust insulin dosing in order to mitigate the risk of low blood glucose levels.

9.3.2 Site Reported Serious Adverse Events (SAE)

SAEs will be recorded within the eCRF from the date of randomisation until the later of 30 days post cessation of trial treatment or the end of the study.

Full details of SAEs will be recorded in the electronic Case Report Form. The following information will be collected:

- full details in medical terms and a case description
- seriousness criteria

- event duration (start and end dates, if applicable)
- action taken
- outcome
- causality (i.e. relatedness to trial drug / investigation), in the opinion of the investigator
- if related, whether the reaction would be considered expected or unexpected.

Any change of condition or other follow-up information should be added to the eCRF and forwarded to the Sponsor (if reportable SAE) as soon as it is available or at least within 24 hours of the information becoming available. Events will be followed up until the event has resolved or a final outcome has been reached.

Reporting of suspected/ confirmed COVID 19 infection

If a patient presents with clinical signs and symptoms consistent with COVID-19 and a test has been performed or requested:

- If test is positive, record "COVID-19 confirmed" in the diagnosis field
- If test is negative, record the SAE signs and symptoms and/or other diagnosis in the diagnosis field.

If a test has not been performed or result is not available and signs and symptoms, as judged by the investigator, are highly suspicious of COVID-19 infection, record "COVID-19 suspected" in the diagnosis field

If other concurrent diagnoses, e.g. pneumonia, please record within the narrative of the SAE.

COVID-19 vaccination and reporting

Where a deployed COVID-19 vaccine is suspected to be involved in the onset of a reported event it should be recorded as a concomitant medication. A causal relationship between the vaccine and the event, including potential drug interactions should be assigned by the reporting investigator.
If a reported event is suspected to be due to a deployed COVID-19 vaccine alone reporting investigators should ensure that standard Yellow Card reporting procedures are followed.

Assessment of Adverse Events

All adverse events must be assessed by the reporting site for seriousness**.** All SAEs must also be assessed for causality and expectedness with reference to this protocol and the Reference Safety Information (RSI).

Assessment of seriousness

An adverse event will be considered serious if it:

1. Results in death
2. Is life threatening
3. Requires hospitalisation or prolongation of existing hospitalisation
4. Results in persistent or significant disability or incapacity
5. Consists of a congenital anomaly or birth defect

6. Required intervention to prevent one of the above.
7. Is a serious Adverse Event of Special Interest.

Assessment of causality i.e. does the event have a “reasonable causal relationship” with trial IMPs. The following categories are used:

- **Yes** - The causal and temporal relationship between the event and the absence of a more likely explanation suggest that the trial IMP is the most likely cause.
- **No** - The event is not considered related to the trial IMPs.

Causality will be assigned for each IMP the participant is randomised to receive.

Should an SAE be considered unrelated to the administration of either trial drug then the causality of the event will be collected taking into consideration other factors, for example concomitant medications or co-morbidities, etc.

Assessment of causality is the responsibility of the Principal Investigator or medically-qualified designee(s) at each site. The CI, their delegate(s), or the Sponsor may provide an opinion regarding causality but cannot downgrade the PI’s assessment.

Assessment of expectedness

Should the event be considered to be related to the trial IMP(s), an assessment should be made regarding the expectedness of the reaction, i.e. whether the reaction is a recognised adverse effect of the IMP(s).

.
Expected: consistent with the relevant product information documented in the RSI(s).
Unexpected: not consistent with the relevant product information documented in the RSI(s).

The CI, their delegate(s), or the Sponsor have overall responsibility for assigning expectedness as per the RSI(s) and provide the final assessment of the event. The reporting investigator may provide an opinion on the expectedness of an event and this may be taken into account.

SAEs occurring within the USA and the UK

The US Sponsor is responsible for assigning causality and expectedness for any AEs, SAEs and SUSARs that occur within the USA. Where a SUSAR is identified by the US Sponsor this will be forwarded to the UK Sponsor for review within one working day. Following review by the UK Sponsor, appropriate US SUSARs will be subject to expedited reporting to the MHRA as per UK regulatory requirements. Similarly, where a SUSAR is identified by the UK sponsor, this will forwarded to the US Sponsor for review within two working days.

Recording and reporting of SAEs
All SAEs arising during the clinical trial will be recorded in the eCRF soon as reasonably practicable and in any event within 24hours of first becoming aware of the event. Any follow-up information should also be reported.

If recording in the eCRF is not possible a paper SAE form should be completed:

1. The SAE form is downloaded from www.glasgowctu.org, printed off, completed and signed. The form is then faxed to the Glasgow Clinical Trials Unit Pharmacovigilance (PV) Office on

+44(0)141 357 5588. As an alternative, a scanned copy of the SAE form may be emailed to: pharmacovig@glasgowctu.org. If this website is unavailable a paper copy of the SAE form should be filed in the Investigator Site File at each site pending resolution.
2. If necessary a verbal report can be given by contacting the PV Office on +44(0)141 330 4744. This must be followed up as soon as possible with an electronic or written report.

Reporting to sponsor

All SAEs will be considered reportable to the Sponsor PV office from the time of randomisation until 30 days following permanent discontinuation of all trial IMP(s). SAEs occurring following 30 days post cessation of treatment are not reportable to the Sponsor unless an SAE is considered related to IMP(s)

9.3.3 Suspected Unexpected Serious Adverse Reactions (SUSARs)

Any SAE assessed as related to trial IMP(s) by the PI or delegate, and unexpected (not documented as an expected reaction to the IMP(s) within the RSI(s)) by the CI, their delegate, or the Sponsor, will be classified as a SUSAR and therefore subject to expedited reporting to the Medicines and Healthcare products Regulatory Agency (MHRA) and the Research Ethics Committee (REC). Should the CI disagree with the PI's assessment of causality, both opinions will be provided on the report. Where the CI or UK Sponsor disagrees with the US Sponsor's assessment of expectedness both opinions will also be provided on the report.

The Sponsor will inform the MHRA of all SUSARs that occur within the trial and the REC of all SUSARs that occur within the UK within the required expedited reporting timescales:

- **Fatal or life threatening SUSARs** - no later than 7 days of Sponsor awareness that the case fulfils the criteria for a fatal or life threatening SUSAR, and any follow up information within a further 8 days.
- **All other SUSARs** - no later than 15 days of Sponsor awareness that the case fulfils the criteria for a SUSAR.

The sponsor will report SUSARs to the MHRA via the MHRA eSUSAR reporting system and to REC by email with accompanying CTIMP Safety Report Form.

9.3.4 Unblinding for SUSAR reporting purposes

In the event of a potential SUSAR, the sponsor (but not the investigators) will be unblinded to facilitate appropriate reporting to the MHRA and REC.

9.4 Responsibilities for Safety Reporting and Review

This section details the responsibilities for reporting and reviewing safety information arising from the trial.

Principal Investigator (PI):

- Attendance at the initiation meeting/teleconference
- Training of new members of the trial team in the protocol and its procedures
- Ensuring that the ISF is accurately maintained

- Dissemination of important safety or trial-related information to all stakeholders at their site
- Checking for AEs and ARs when participants attend for treatment/follow-up
- Ensuring that AEs are recorded and reported in line with the requirements of the protocol
- Ensuring that all SAEs are recorded and reported to the Sponsor within 24 hours of becoming aware of the event, and providing further follow-up information as soon as available
- Using medical judgement in assigning causality of SAEs
- Providing an opinion on the expectedness of an SAE with reference to the trial protocol and Reference Safety Information
- Using definitions in this protocol, flagging events of special interest.

Chief Investigator (CI)

- Providing clinical oversight of the safety of patients participating in the trial, including an ongoing review of the risk/benefit
- Providing clinical review of section 4.8 of the representative SmPCs on an annual basis to define the Reference Safety Information for the trial
- Using medical judgement to confirm seriousness and causality of all SARs
- Reviewing expectedness of related SAEs with reference to the trial protocol and Reference Safety Information
- Preparing the clinical sections and final sign-off of the Development Safety Update Report (DSUR)
- Using definitions in this protocol, confirming events of special interest

Sponsor:

- Central data collection and verification of AEs, SAEs, SARs and SUSARs according to the trial protocol
- Reporting safety information to the CI or delegate for the ongoing assessment of the risk / benefit
- Reporting safety information to the independent oversight committees identified for the trial (Independent Data Monitoring Committee (IDMC) and / or Trial Steering Committee (TSC)) according to the Trial Monitoring Plan.
- Expedited reporting of SUSARs to the Competent Authority (MHRA in UK) and REC within required timelines.
- Notifying Investigators of SUSARs that occur within the trial.
- Checking for (annually) and notifying PIs of updates to the Reference Safety Information for the trial.
- Preparing standard tables and other relevant information for the DSUR in collaboration with the CI and ensuring timely submission to the MHRA and REC.

Trial Steering Committee:

In accordance with the Charter for the TSC, periodically reviewing recruitment and the overall progress of the trial and liaising with the IDMC and sponsor regarding safety issues.

Independent Data Monitoring Committee:

In accordance with the Charter for the IDMC, periodically reviewing unblinded safety data in individual cases and to determine patterns and trends of events, or to identify safety issues, which would not be apparent on an individual case basis, reporting concerns to the TSC and sponsor.

9.5 Pregnancy reporting

Pregnancy is not considered an AE unless a negative or consequential outcome is recorded for the mother or child/foetus. If the outcome meets the serious criteria, this would be considered an SAE and must be reported as per SAE reporting procedure above.
Any **pregnancy** occurring in a female trial participant or female partner of a male trial participant who becomes pregnant while participating in the Trial will be reported by the PI (or designee) to the Chief Investigator and the sponsor using the sponsor Pregnancy Reporting Form (available at http://www.glasgowctu.org/) within two weeks of the PI first becoming aware of the pregnancy.
The trial participant will also be followed up to determine the outcome of the pregnancy and follow-up information forwarded to the PV office. Any resulting SAEs should be reported as per SAE reporting procedure above.

9.6 Overdose

Any IMP dose which is not administered in accordance with the Protocol should be reported to the sponsor.
If an SAE is associated with an overdose ensure that the overdose if fully described in the SAE report form.

9.7 Reporting urgent safety measures

If any urgent safety measures are taken the CI/Sponsor will phone the MHRA's Clinical Trial Unit on 020 3080 6456, ideally within 24 hours. This will be followed up no later than 3 days from the date the measures are taken, giving written notice to the MHRA (who will advise the format required) and the relevant REC of the measures taken and the circumstances giving rise to those measures. A substantial amendment must also be submitted to the MHRA.

9.8 The type and duration of the follow-up of participants after adverse events.

Adverse events and reactions will be recorded, reported and followed up in line with this protocol until study completion or for a minimum of 30 days after participant's last dose of the IMP, whichever is later.
Any SUSAR identified will be reported to the Sponsor and to the Regulatory Authorities irrespective of how long after IMP administration the reaction has occurred.

9.9 Development safety update reports

A Development Safety Update Report (DSUR) will be submitted once a year, or on request, to MHRA and REC until the trial is declared ended. The report will be submitted within 60 days of the anniversary of the issue of the Clinical Trials Authorisation for the trial. The DSUR will be prepared by the sponsor (PV Office) in liaison with the CI and submitted by the sponsor (PV Office). The DSUR will include only those SAEs reported to the co-sponsors; that is: SAEs and SUSARs occurring within the UK and SUSARs originating in the USA.

10 STATISTICS AND DATA ANALYSIS

10.1 (a)Sample size calculation

The sample size in Protocol versions 1.0 - 5.0 was as follows: "This is based on our current data showing that in type 1 diabetes, treatment with a GLP-1 agonist, liraglutide (1.2 and 1.8mg doses) for three months leads to HbA1c suppression by an average of 0.6% (SD=0.5%). Dapagliflozin was recently shown to reduce HbA1c by 0.45% in type 1 diabetes patients (13). Additionally, empagliflozin treatment for 8 weeks was shown to reduce HbA1c by 0.4% in type 1 diabetics (20, 21) and our

current data showing additional improvement in HbA1c by an additional 0.66% at 12 weeks when dapagliflozin was added to liraglutide and insulin therapy. Conservatively estimating that the combination therapy will suppress HbA1c by 0.5% (SD≤0.7%) compared to semaglutide alone, a sample size of 38 (32 evaluable patients) in each group will be sufficient to provide 80% power at a 5% significance level, taking in consideration a 15% dropout rate. 50% of participants will be recruited in Glasgow, UK and 50% at State University of New York at Buffalo, USA."

10.1 (b) Revised sample size calculation (from Protocol version 6.0)

At their meeting in May 2023, the TSC asked the trial statisticians to review the sample size required on the basis of blinded review of primary outcome data (SD of change of HbA1c, correlation of HbA1c at baseline and end of Period 2). In August 2023 the trial statisticians recommended on the basis of this review a reduction in the sample size by a factor of 0.59 i.e. from 32 to 19 evaluable patients per group. This recommendation was reviewed by the IDMC in August 2023, by the TSC in September 2023 and at an additional IDMC meeting in October 2023 (at which additional information was provided on changes in insulin delivery devices during the trial). At this time, the IDMC unanimously agreed that the study should continue, aiming conservatively for a revised sample size in Period 2 of around 23/ evaluable participants per group for dapagliflozin vs placebo. This reduces the recruitment target for first randomisation from n=114 to n=82 (i.e. 23/32*114) maintaining the same assumptions on power, significance level and drop-out (amended accordingly on page 7 above).

The IDMC further required a formal interim analysis on all those with mature data on the primary outcome at around the end of April 2024 with recruitment to continue unless formally stopped by the funder following a subsequent recommendation by the TSC. The study should continue after interim analysis: (a) unless there is overwhelming evidence of benefit, or evidence of futility; and (b) if the study has recruited sufficient numbers between Nov 23 – Apr 24 to make reaching the new target in a reasonable time feasible within budget (minimum 8-10 new participants recruited)). The IDMC agreed to specify the exact detail of the interim analysis in time for this analysis to be conducted, to review the results and make a recommendation to the TSC.

This recommendation was accepted unanimously by the independent members of the TSC in November 2023.

10.2 Anticipated recruitment rate

Originally one year (one participant per week). The rate has been considerably slower due mainly to the COVID-19 pandemic and global semaglutide supply issues. 60 participants had been randomised by November 2023.

10.3 Statistical analysis

The primary objective of the proposed research is to evaluate the effect of addition of dapagliflozin to semaglutide on a background of insulin treatment in type 1 diabetes patients on HbA1c at six months following start of dapagliflozin. Transformations of the data in order to meet statistical assumptions will be considered. Statistical analyses will use linear regression models to compare treatment groups, with adjustment for baseline values of study outcomes, as well as factors used to stratify the randomisation scheme. Tests will be two-sided and tested at the 5% significance level. Analyses will be carried out under the intention to treat principle, that is, according to randomised treatment allocation, regardless of treatment adherence.

A detailed Statistical Analysis Plan will be completed and signed off before database lock. Study statisticians will develop and validate the analysis programs without access to randomised treatment

allocations. After database lock, the treatment codes will be released and the final analysis will be carried out.

10.4 Subgroup analyses

These will be specified in the Statistical Analysis Plan. Subgroup analyses will be implemented by extending regression models to include interactions between treatment and potential treatment effect moderators.

10.5 Interim analysis and criteria for the premature termination of the trial

No interim analysis was initially planned but an interim analysis was recommended by the IDMC in November 2023 – to be conducted in April 2024 (see section 10.1(b) above). Unblinded trial data will be reviewed on an ongoing basis by the IDMC. The primary role of the IDMC will be to protect the interests of the patients. The IDMC may recommend to the TSC and Co-Sponsors that the study should stop prematurely because of concerns about patient safety. The IDMC will meet approximately every six months. The IDMC will take into account all results and the consistency and biological plausibility of the findings in making any recommendation. The final decision on continuing or stopping the trial will lie with the TSC/Co-Sponsors.

10.6 Subject population

All analyses will be carried out on an intention to treat basis, using the randomised treatment allocation.

10.7 Procedure(s) to account for missing or spurious data

Data will be analysed without imputation in the first instance. Multiple imputation procedures will be used to assess the sensitivity of selected results to missing data, according to the Statistical Analysis Plan.

10.8 Other statistical considerations.

The Statistical Analysis Plan (SAP) will be maintained as a version controlled document and will be signed off before database lock. The SAP and other statistical documentation (according to Glasgow Clinical Trials Unit Standard Operating Procedures) will contain full details of all analyses along with assumptions and procedures for handling problematic or incomplete data (e.g. incomplete dates).

11 DATA HANDLING

11.1 Source Documentation

ICH GCP defines source data as: 'All information in original records and certified copies of original records of clinical findings, observations or other activities in a clinical trial necessary for the reconstruction and evaluation of the trial'. In this study, the location of the majority of the source data will be the hospital's medical records including subject case notes, laboratory records and ECGs. The source data transcribed into the eCRF from the medical records must be accurate and verifiable. For questionnaires completed by trial subjects, the completed questionnaires will be regarded as the source data location. In cases where data are transcribed directly into the eCRF and no other paper or electronic source exists, then the eCRF will be considered the source record. In these cases, these data should be prospectively documented in the medical records to ensure a full record of the trial is available at site. Identification of unverifiable data entries will be reported as non-compliances.

11.2 Data collection

An eCRF, developed by the Robertson Centre for Biostatistics, will capture all data required to meet this protocol's requirements. Access to the eCRF will be restricted, via a study-specific web portal, and only authorised site-specific personnel will be able to make entries to their patients' data via the web portal. The Investigator, or his/her designee will be responsible for all entries into the eCRF and will confirm that the data are accurate, complete and verifiable. Data will be stored in a MS SQL Server database.

Direct access to the web portal will be granted, on request, to authorised representatives from the Sponsor, host institution and the regulatory authorities to permit trial-related monitoring, audits and inspections.

11.3 Data Validation

Where it is practical, data will be validated at the point of entry into the eCRF. Any additional data discrepancies will be flagged to the investigator and any data changes will be recorded to maintain a complete audit trail (reason for change, date change made, who made change).

11.4 Data Security

The Robertson Centre for Biostatistics systems are fully validated in accordance with industry and regulatory standards, and incorporate controlled access security. High volume servers are firewall protected and preventative system maintenance policies are in place to ensure no loss of service or data. Web servers are secured by digital certificates. Data integrity is assured by strictly controlled procedures, including secure data transfer procedures. Data are backed up on-site nightly and off-site to a commercial data vault weekly. The Robertson Centre for Biostatistics has an ISO 9001:2008 quality management system and ISO 27001:2013 for Information Security, and is regularly inspected against the standards by the British Standards Institution.

11.5 Archiving

The Trial Master File will be archived by the Co-Sponsors at the end of the trial for a minimum period of five years.

Archiving of Site Files will also be for a minimum of seven years from completion of the trial, and this action will be delegated to the sites in the Clinical Trial Site Agreement that will be put in place between Co-Sponsors and Sites. Sites will be notified by the Co-Sponsors when Site files can be archived.

Destruction of site files can only take place with the approval of the Co-Sponsors.

12 MONITORING, AUDIT & INSPECTION

Monitoring will be conducted by NHS Greater Glasgow and Clyde (GG&C) Monitor(s) in accordance with local Standard Operating Procedures. The level, frequency, objectives and priorities of monitoring will be based on the outcome of the completed Risk Assessment, and clearly documented in the Monitoring Plan which will be approved by the members of the TMG and the NHS GG&C Lead Monitor and/or Research Governance Manager. The Monitoring Plan is subject to updates during the life of the study in line with Protocol Amendments.

13 ETHICAL AND REGULATORY CONSIDERATIONS

13.1 Research Ethics Committee (REC) review & reports

Before the start of the trial, approval will be sought from a REC for the trial protocol, informed consent forms and other relevant documents e.g. advertisements and GP information letters.
Substantial amendments that require review by REC will not be implemented until the REC grants a favourable opinion for the study (it is noted that amendments may also need to be reviewed and accepted by the MHRA and/or NHS R&D departments before they can be implemented in practice at sites).
All correspondence with the REC will be retained in the Trial Master File/Investigator Site File.
An annual progress report (APR) will be submitted to the REC within 30 days of the anniversary date on which the favourable opinion was given, and annually until the trial is declared ended (this is the Chief Investigator's responsibility).
The Chief Investigator will notify the REC of the end of the study.
If the study is ended prematurely, the Chief Investigator will notify the REC, including the reasons for the premature termination
Within one year after the end of the study, the Chief Investigator will submit a final report with the results, including any publications/abstracts, to the REC.

13.2 Peer review

The study protocol has been developed with expert and independent feedback from the JDRF (New York) and during application for funding underwent peer review by independent experts.

13.3 Public and Patient Involvement

The study was designed with input from people with type 1 diabetes via the funder, JDRF.

13.4 Regulatory Compliance

The trial will not commence until a Clinical Trial Authorisation (CTA) is obtained from the MHRA.
The protocol and trial conduct will comply with the Medicines for Human Use (Clinical Trials) Regulations 2004 and any relevant amendments.
Before any site can enrol patients into the trial, the Chief Investigator/Principal Investigator or designee will apply for NHS permission from the site's Research & Development (R&D) department.

For any amendment that will potentially affect a site's NHS permission, the Chief Investigator/ Principal Investigator or designee will confirm with that site's R&D department that NHS permission is ongoing (note that both substantial amendments, and amendments considered to be non-substantial for the purposes of REC and/or MHRA may still need to be notified to NHS R&D).

13.5 Protocol compliance

Prospective, planned deviations or waivers to the protocol are not allowed under the UK regulations on Clinical Trials and must not be used e.g. it is not acceptable to enrol a participant if they do not meet the eligibility criteria or restrictions specified in the trial protocol. Accidental protocol deviations can happen at any time. They must be adequately documented on the relevant forms and reported to the Chief Investigator, Sponsor and GCTU immediately. Deviations from the protocol which are found to frequently recur are not acceptable, will require immediate action and could potentially be classified as a serious breach.

13.6 Notification of Serious Breaches to GCP and/or the protocol

A "serious breach" is a breach which is likely to affect to a significant degree –

1. the safety or physical or mental integrity of the subjects of the trial; or
2. the scientific value of the trial

If any of the above occurs then the CI and Sponsor will be notified. The sponsor will notify the appropriate authorities in writing of any serious breach in accordance with their standard operating procedures.

13.7 Data protection and patient confidentiality

All investigators and trial site staff must comply with the requirements of the GDPR with regards to the collection, storage, processing and disclosure of personal information and will uphold the Act's core principles.

- Only those individuals who require to see personal information i.e. site research team staff or the study monitor, as appropriate, will be able to view them. All electronic data will be held securely in accordance with ISO 27001:2013 at the Robertson Centre for Biostatistics, part of the Glasgow Clinical Trials Unit. All Centre staff are required to sign confidentiality agreements and to follow Standard Operating Procedures in accordance with Good Clinical Practice and ISO certification.
- The trial data managers, statisticians, health economists or any other staff who will perform data related tasks will only be able to access depersonalised data where the participant's identifying information is replaced by a unique study identifier.

- Only those that have been trained and approved will be able to enter or view any data via the web portal. Each site can only see their own patients' data. Patient consent forms will be stored at the study site in a secure location accessible only to study teams.

13.8 Indemnity

The Co-Sponsors (University of Glasgow and Greater Glasgow Health Board) will ensure that provision has been made for insurance or indemnity to cover the liability of the investigator and sponsor which may arise in relation to the clinical trial in accordance with Part 2 (14) of Schedule 1 to SI 2004/1031.

13.9 Amendments

Any change in the study protocol will require an amendment. Any proposed protocol amendments will be initiated by the CI following discussion with the Sponsor and TSC and any required amendment forms will be submitted to the regulatory authority, ethics committee and Sponsor. The Sponsor will determine whether an amendment is non-substantial or substantial. All amended versions of the protocol will be signed by the CI and Sponsor representative. Following a substantial amendment, favourable opinion/approval must be sought from the original reviewing REC, MHRA (where appropriate) and Research and Development (R&D) office prior to implementation. The Chief Investigator will be responsible for informing the Trial Management Group of all protocol amendments.

13.10 Post trial care

At the end of the trial, participants will be returned to usual care as defined by local and national guidelines at that time. The results of the trial may of course have an impact on these guidelines and the future care of patients with type 1 diabetes.

13.11 Access to the final trial dataset

During the trial and in the period prior to publication of the main study results as described in the protocol, only the Glasgow CTU, Professor Paresh Dandona and Dr Husam Ghanim US Principal Investigators, University of Buffalo will have access to the full dataset. After that period, the trial Steering Committee will conduct further data analyses for a period of three years. After that time the Trial Steering Committee will consider requests from external parties for further analyses of the study data. Proposals that are scientifically well founded and have an academic basis and where relevant data extractions and analyses are appropriately funded will not be refused. These will be considered as collaborative exercises where the contributions related to study design, conduct, database creation and maintenance and data analysis will be recognised in authorship of any scientific publication. The approach we will take will be to minimise any possibility of breach of participant confidentiality. Normally this will be achieved by minimising data travel. However, for the purposes of individual patient meta-analysis and other reasons, data may be transferred to other sites. Such transfer will require assurances on information security systems at the sites that data are to be transferred to and will involve a legal data transfer agreement. A log of all data requests and subsequent data transfers will be held at the Glasgow CTU.

14 DISSEMINATION POLICY

14.1 Dissemination policy

The study database will be owned by the University of Glasgow and maintained on behalf of the Study investigators, represented by the Trial Steering Committee as it is constituted during and after the trial.

The study protocol and a description of the recruitment experience and participant baseline characteristics will be published before study completion. On completion of the trial, the database will be locked and analysed by staff of the Robertson Centre for Biostatistics, University of Glasgow. A final study report will be prepared and the results will be published in a major medical journal.

After the main study publications, study investigators will be invited to submit requests for further analyses of the study database. These will be reviewed and prioritised by a Publications Committee made up of the study grant holders and convened by the study co-CIs.

As funder of the trial, JDRF will have the right to see and comment on any results being submitted for publication. A maximum of 28 days will be given for review of major papers and 14 days for abstracts. Such comments will be considered by the Trial Steering Committee.

A lay summary of the main results of the trial will be prepared and provided to all participants via their study site investigators. Investigators may request a copy of the study data for their participants. Providing some or all of a patient's data to that individual is at their discretion.

14.2 Authorship eligibility guidelines and any intended use of professional writers

The main results of the study will be compiled, written up and published by the study grant holders and others taking responsibility for the study results (e.g. the statistician conducting the final analysis) on behalf of the TTT1 investigators. The TTT1 investigators will be listed in an Appendix and will include all site PIs, all committee members and key members of relevant study coordinating groups (including the Sponsor and Glasgow CTU).

15 REFERENCES

1. Konrad K, Datz N, Engelsberger I, Grulich-Henn J, Hoertenhuber T, Knauth B, et al. Current use of metformin in addition to insulin in pediatric patients with type 1 diabetes mellitus: an analysis based on a large diabetes registry in Germany and Austria. *Pediatr Diabetes.* 2014.
2. Vella S, Buetow L, Royle P, Livingstone S, Colhoun HM, and Petrie JR. The use of metformin in type 1 diabetes: a systematic review of efficacy. *Diabetologia.* 2010;53(5):809-20.
3. Vella S, Buetow L, Royle P, Livingstone S, and Petrie JR. Metformin in type 1 diabetes reduces insulin requirements without significantly improving glycaemic control. Reply to Schatz H [letter]. *Diabetologia.* 2011;54(1):203-4.
4. Varanasi A, Bellini N, Rawal D, Vora M, Makdissi A, Dhindsa S, et al. Liraglutide as additional treatment for type 1 diabetes. *Eur J Endocrinol.* 2011;165(1):77-84.
5. Kuhadiya ND, Malik R, Bellini NJ, Patterson JL, Traina A, Makdissi A, et al. Liraglutide as additional treatment to insulin in obese patients with type 1 diabetes mellitus. *Endocrine practice : official journal of the American College of Endocrinology and the American Association of Clinical Endocrinologists.* 2013;19(6):963-7.
6. Kuhadiya ND, Dhindsa S, Ghanim H, Mehta A, Makdissi A, Batra M, et al. Addition of Liraglutide to Insulin in Patients With Type 1 Diabetes: A Randomized Placebo-Controlled Clinical Trial of 12 Weeks. *Diabetes Care.* 2016;39(6):1027-35.
7. Chaudhuri A, Ghanim H, Makdissi A, Green K, Abuaysheh S, Batra M, et al. Exenatide induces an increase in vasodilatory and a decrease in vasoconstrictive mediators. *Diabetes Obes Metab.* 2017;19(5):729-33.
8. Mathieu C, Zinman B, Hemmingsson JU, Woo V, Colman P, Christiansen E, et al. Efficacy and Safety of Liraglutide Added to Insulin Treatment in Type 1 Diabetes: The ADJUNCT ONE Treat-To-Target Randomized Trial. *Diabetes Care.* 2016;39(10):1702-10.
9. Ahren B, Hirsch IB, Pieber TR, Mathieu C, Gomez-Peralta F, Hansen TK, et al. Efficacy and Safety of Liraglutide Added to Capped Insulin Treatment in Subjects With Type 1 Diabetes: The ADJUNCT TWO Randomized Trial. *Diabetes Care.* 2016;39(10):1693-701.
10. Zinman B, Wanner C, Lachin JM, Fitchett D, Bluhmki E, Hantel S, et al. Empagliflozin, Cardiovascular Outcomes, and Mortality in Type 2 Diabetes. *N Engl J Med.* 2015;373(22):2117-28.
11. Petrie JR. SGLT2 inhibitors in type 1 diabetes: knocked down, but up again? *Lancet Diabetes Endocrinol.* 2017;5(11):841-3.
12. Mudaliar S, Armstrong DA, Mavian AA, O'Connor-Semmes R, Mydlow PK, Ye J, et al. Remogliflozin etabonate, a selective inhibitor of the sodium-glucose transporter 2, improves serum glucose profiles in type 1 diabetes. *Diabetes Care.* 2012;35(11):2198-200.
13. Kohan DE, Fioretto P, Tang W, List JF. Long-term study of patients with type 2 diabetes and moderate renal impairment shows that dapagliflozin reduces weight and blood pressure but does not improve glycemic control. Kidney Int. 2014 Apr;85(4):962-71. doi: 10.1038/ki.2013.356. Epub 2013 Sep 25. PMID: 24067431; PMCID: PMC3973038.
14. Dandona P, Mathieu C, Phillip M, Hansen L, Griffen SC, Tschope D, et al. Efficacy and safety of dapagliflozin in patients with inadequately controlled type 1 diabetes (DEPICT-

1): 24 week results from a multicentre, double-blind, phase 3, randomised controlled trial. *Lancet Diabetes Endocrinol.* 2017;5(11):864-76.

15. Mathieu C, Dandona P, Gillard P, Senior P, Hasslacher C, Araki E, Lind M, Bain SC, Jabbour S, Arya N, Hansen L, Thorén F, Langkilde AM; DEPICT-2 Investigators. Efficacy and Safety of Dapagliflozin in Patients With Inadequately Controlled Type 1 Diabetes (the DEPICT-2 Study): 24-Week Results From a Randomized Controlled Trial. Diabetes Care. 2018 Sep;41(9):1938-1946.
16. European Medicines Agency. Assessment Report Dapagliflozin. https://www.ema.europa.eu/en/documents/variation-report/forxiga-h-c-ws-1941-epar-assessment-report-variation_en.pdf *(accessed 11th November 2021)*
17. Groop PH, Dandona P, Phillip M, Gillard P, Edelman S, Jendle J, Xu J, Scheerer MF, Thoren F, Iqbal N, Repetto E, Mathieu C. Effect of dapagliflozin as an adjunct to insulin over 52 weeks in individuals with type 1 diabetes: post-hoc renal analysis of the DEPICT randomised controlled trials. Lancet Diabetes Endocrinol. 2020 Oct;8(10):845-854. doi: 10.1016/S2213-8587(20)30280-1. PMID: 32946821.
18. Rosenstock J, Marquard J, Laffel LM, et al. Empagliflozin as adjunctive to insulin therapy in type 1 diabetes: the EASE trials. *Diabetes Care*. 2018;41(12):2560–2569.
19. Garg SK, Henry RR, Banks P, Buse JB, Davies MJ, Fulcher GR, et al. Effects of Sotagliflozin Added to Insulin in Patients with Type 1 Diabetes. *N Engl J Med.* 2017;377(24):2337-48.
20. European Medicines Agency. First oral add-on treatment to insulin for treatment of certain patients with type 1 diabetes. https://www.ema.europa.eu/en/news/first-oral-add-treatment-insulin-treatment-certain-patients-type-1-diabetes *(accessed 11th November 2021)*
21. An overview of Zynquista and why it is authorised in the EU. 16th May 2019. https://www.ema.europa.eu/en/documents/overview/zynquista-epar-medicine-overview_en.pdf *(accessed 11th November 2021)*
22. National Institute for Health and Care Excellence. Dapagliflozin with insulin for treating type 1 diabetes. Technology appraisal guidance [TA597] https://www.nice.org.uk/guidance/ta597
23. AstraZeneca Press Release. Update on US regulatory decision for Farxiga in type-1 diabetes. https://www.astrazeneca.com/media-centre/press-releases/2019/update-on-us-regulatory-decision-for-farxiga-in-type-1-diabetes-15072019.html# *(accessed 11th November 2021)*
24. European Medicines Agency. Forxiga (dapagliflozin) 5mg should no longer be used for the treatment of Type 1 Diabetes Mellitus. https://www.ema.europa.eu/en/documents/dhpc/direct-healthcare-professional-communication-dhpc-forxiga-dapagliflozin-5mg-should-no-longer-be-used_en.pdf *(accessed 11th November 2021)*
25. AstraZeneca. Letter to UK Health Professionals. Forxiga (dapagliflozin) 5mg should no longer be used for the treatment of type 1 diabetes mellitus. (2nd November 2021)
26. Marso SP, Bain SC, Consoli A, Eliaschewitz FG, Jódar E, Leiter LA, Lingvay I, Rosenstock J, Seufert J, Warren ML, et al. Semaglutide and cardiovascular

outcomes in patients with type 2 diabetes. *N Engl J Med.* 2016;**375**(19):1834–44. doi: 10.1056/NEJMoa1607141.
27. Ozempic Summary of product characteristics. https://www.medicines.org.uk/emc/product/9728/smpc
28. Nauck MA, Petrie JR, Sesti G, Mannucci E, Courrèges JP, Lindegaard ML, Jensen CB, Atkin SL; Study 1821 Investigators. A Phase 2, Randomized, Dose-Finding Study of the Novel Once-Weekly Human GLP-1 Analog, Semaglutide, Compared With Placebo and Open-Label Liraglutide in Patients With Type 2 Diabetes. Diabetes Care. 2016 Feb;39(2):231-41.
29. Forxiga 5 mg Summary of Product Characteristics: https://www.medicines.org.uk/emc/product/2865/smpc *(accessed 11th November 2021)*
30. Wiviott SD, Raz I, Bonaca MP, Mosenzon O, Kato ET, Cahn A, Silverman MG, Zelniker TA, Kuder JF, Murphy SA, Bhatt DL, Leiter LA, McGuire DK, Wilding JPH, Ruff CT, Gause-Nilsson IAM, Fredriksson M, Johansson PA, Langkilde AM, Sabatine MS; DECLARE–TIMI 58 Investigators. Dapagliflozin and Cardiovascular Outcomes in Type 2 Diabetes. N Engl J Med. 2019 Jan 24;380(4):347-357.
31. McMurray JJV, Solomon SD, Inzucchi SE, Køber L, Kosiborod MN, Martinez FA et al. DAPA-HF Trial Committees and Investigators Dapagliflozin in patients with heart failure and reduced ejection fraction. N Engl J Med. 2019. Nov 21;381(21):1995-2008.
32. Heerspink HJL, Stefánsson BV, Correa-Rotter R, Chertow GM, Greene T, Hou FF, Mann JFE, McMurray JJV, Lindberg M, Rossing P, Sjöström CD, Toto RD, Langkilde AM, Wheeler DC; DAPA-CKD Trial Committees and Investigators. Dapagliflozin in Patients with Chronic Kidney Disease. N Engl J Med. 2020 Oct 8;383(15):1436-1446. doi: 10.1056/NEJMoa2024816. Epub 2020 Sep 24. PMID: 32970396.
33. Vilsbøll T, Bain SC, Leiter LA, et al. Semaglutide, reduction in glycated haemoglobin and the risk of diabetic retinopathy. *Diabetes Obes Metab*. 2018;20(4):889–897.
34. Lingvay I, Desouza CV, Lalic KS et al. A 25 week randomized controlled trial of semaglutide once daily versus liraglutide and placebo in patients with type 2 diabetes suboptimally controlled on diet and exercise with or without metformin. Diabetes Care. 2018;41(9): 1926-1937.
35. European Medicines Agency. Assessment Report Sotagliflozin. https://www.ema.europa.eu/en/documents/assessment-report/zynquista-epar-public-assessment-report_en.pdf *(accessed 11th November 2021)*
36. Silverii GA, Decembrini L, Monami M, Mannucci E. Fournier's gangrene and sodium glucose co-transporter 2 inhibitors: A metanalysis of randomized controlled trials. Diabetes Obes Metab. 2019; 1-4.
37. Garg SK, Peters AL, Buse JB, Danne T. Strategy for mitigating DKA risk in patients with Type 1 diabetes on adjunctive treatment with SGLT-2 inhibitors: A STICH Protocol. Diabetes Technol Ther. 2018;20(9):571-575.
38. Peters AL, Buschur EO, Buse JB, Cohan P, Diner JC, and Hirsch IB. Euglycemic Diabetic Ketoacidosis: A Potential Complication of Treatment With Sodium-Glucose Cotransporter 2 Inhibition. Diabetes Care. 2015;38(9):1687-93.
39. Agiostratidou G, Anhalt H, Ball D, Blonde L, Gourgari E, Harriman KN, Kowalski AJ, Madden P, McAuliffe-Fogarty AH, McElwee-Malloy M, Peters A, Raman S, Reifschneider

K, Rubin K, Weinzimer SA. Standardizing Clinically Meaningful Outcome Measures Beyond HbA(1c) for Type 1 Diabetes: A Consensus Report of the American Association of Clinical Endocrinologists, the American Association of Diabetes Educators, the American Diabetes Association, the Endocrine Society, JDRF International, The Leona M. and Harry B. Helmsley Charitable Trust, the Pediatric Endocrine Society, and the T1D Exchange. Diabetes Care. 2017 Dec;40(12):1622-1630

16. APPENDICES

16.1 Appendix 1 – Risk

Risks associated with trial interventions<br>☐ LOW ≡ Comparable to the risk of standard medical care<br>☒ MODERATE ≡ Somewhat higher than the risk of standard medical care<br>☐ HIGH ≡ Markedly higher than the risk of standard medical care				
Justification: Briefly justify the risk category selected and your conclusions below (where the table is completed in detail the detail need not be repeated, however a summary should be given):<br>An IDMC will be convened to monitor all SAEs.<br>Risks and mitigations associated with the intervention are outlined in more detail in the Protocol section 2.1.<br>Co-Sponsors will also carry out a detailed risk assessment of all aspects of the study as part of the approval process (SOP 04.013)				
What are the key risks related to therapeutic interventions you plan to monitor in this trial?		How will these risks be minimised?		
IMP/Intervention	Body system/Hazard	Activity	Frequency	Comments
S/C administration of semaglutide<br>Oral admistration of dapagliflozin	Immune: Hypersensitivity/ana-phylactic reactions	Patients with known hypersensitivity to any GLP-1 agonist or SGLT-2 inhibitor have a contra-indication to the IMP according to the SmPC will not be recruited to the study	Uncommon/ Rare	
PO administration of dapagliflozin	Ketosis and/or Diabetic Ketoacidosis (DKA)	All participants will have access to blood or urine ketone monitoring equipment. An emergency ketosis management plan (STICH Protocol – see above) will be discussed with all participants. Patient alert cards will be provided to all participants. Potential	Common	“and” was an error – intention was for “either” blood or urine ketones between visits but “both” to be measured for visits. This is compatible with the eCRF, the diaries and the rest of the protocol

		participants with recent ketoacidosis will be excluded from the trial (section 6.2)		
S/C administration of semaglutide (+/-) PO administration of dapagliflozin in addition to insulin therapy	Increased risk of hypoglycaemia	Participant awareness of risk and frequent testing of capillary blood glucose. Patients with pre-existing frequent episodes of hypoglycaemia or problems with hypoglycaemia awareness will be excluded from trial participation (section 6.2)	Common	
Outline any other processes that have been put in place to mitigate risks to participant safety (e.g. IDMC, independent data review, etc.)<br>See above				

16.2 Appendix 2: Contraception

In line with recommendations related to contraception and pregnancy testing in clinical trials from the Clinical Trial Facilitation Group (CTFG), for women of childbearing potential* (WOCBP) participating in TTT1, methods of contraception considered as highly effective are defined as those that can achieve a failure rate of less than 1% per year when used consistently and correctly.

Such methods include:

- combined (oestrogen and progestogen containing) hormonal contraception associated with inhibition of ovulation[1]
  - oral
  - intravaginal
  - transdermal
- progestogen-only hormonal contraception associated with inhibition of ovulation[1]
  - oral
  - injectable
  - implantable[2]
- intrauterine device (IUD) [2]
- intrauterine hormone-releasing system (IUS)[2]
- bilateral tubal occlusion[2]
- vasectomised partner[2,3]
- true sexual abstinence[4]

Footnotes

1. An interaction between dapagliflozin and semaglutide with contraception is not anticipated.
2. Contraception methods that in the context of this guidance are considered to have low user dependency.
3. Vasectomised partner is a highly effective birth control method provided that partner is the sole sexual partner of the WOCBP trial participant and that the vasectomised partner has received medical assessment of the surgical success.
4. In the context of this guidance true sexual abstinence refers to: When this is in line with the preferred and usual lifestyle of the patient. Periodic abstinence (e.g. calendar, ovulation, symptothermal, post-ovulation methods), declaration of abstinence for the duration of a trial, and withdrawal are not acceptable methods of contraception.

The reliability of sexual abstinence needs to be evaluated in relation to the duration of the clinical trial and the preferred and usual lifestyle of the subject.

*Child bearing potential = experienced menarche; AND not undergone successful surgical sterilisation (hysterectomy, bilateral tubal ligation, or bilateral oophorectomy); AND not post-menopausal i.e. amenorrhea for ≥12 consecutive months (without another medical cause)

Source: Recommendations related to contraception and pregnancy testing in clinical trials. Clinical Trial Facilitation Group (CTFG), 15th September 2014.
See: https://www.hma.eu/fileadmin/dateien/Human_Medicines/01-About_HMA/Working_Groups/CTFG/2014_09_HMA_CTFG_Contraception.pdf (Accessed 21st January 2020)

16.3 Appendix 3

STICH Protocol *(also provided in Period 2 Participant Diary)*

As explained above, participants in this trial randomised to dapagliflozin are at an increased risk of ketosis and ketoacidosis. All participants will have access to a ketone meter as standard of care. It will be ascertained that each participant has access to a ketone meter; if there are any issues with their current ketone meter it will be replaced as per standard of care. There are mechanisms designed to mitigate the risk of early ketosis developing further into diabetic ketoacidosis. In this trial, we will be using a protocol called STICH (37). This is a mnemonic designed to act as a reminder for participants as to what to do in the event of elevated ketones following exposure to an SGLT-2 drug such as dapagliflozin:

1. STop dapagliflozin or placebo temporarily
2. Insulin – take a bolus of rapid acting insulin
3. Carbohydrate – take approximately 30g of carbohydrate
4. Hydrate – make sure you are taking sips of water aiming to maintain hydration.

Further guidance will be provided in both the Patient Diaries supplied to all participants and in the Patient Alert Cards that participants will be asked to carry at all times. All participants will also be advised that in the event of acute intercurrent illness or hospitalisation, dapagliflozin or placebo should be temporarily stopped. This is because intercurrent illness may additionally increase the risk of ketosis. Participants will likely already be familiar with this concept of "sick-day rules" as this is a key component of basic education for people with type 1 diabetes in clinical practice. These rules will however be re-iterated to participants.

More detailed guidance on actions participants should take for any given level of ketosis will be provided during face-to-face and telephone contact during the trial. In addition, however, the following written protocol will be provided to participants to advise them explicitly of the safest and most appropriate course of action to be taken in any given circumstance. This will take the form of a traffic light system to aid understanding and compliance.

1. Ketones 0.6 mmol/L or less: Continue - no need for additional bolus insulin.
2. Ketones 0.6 – 1.4 mmol/L: consider taking additional fast acting insulin. Follow sick day rules and monitor ketones 2 hourly until they are 0.6 mmol/L or less.
3. Ketones 1.5 – 2.9 mmol/L – Take 10% of your total daily dose of insulin (this is found by adding up all the long acting and short acting insulin for the previous day - only take a maximum of 10 units) as a bolus of fast acting insulin. Keep hydrated - aim for 100ml water per hour. Re-test your glucose and ketones in 2 hours. If not falling contact the study team during office hours. If you need urgent advice at other times please contact NHS 24 (telephone number 111) or attend your nearest Accident & Emergency Department.
4. Ketones above 3 mmol/L – Take 20% of your total daily insulin as fast acting insulin (Maximum 10 units). Hydrate with water, aim for 500 ml per hour. Attend A&E – High risk of DKA

If you develop DKA or are unwell it is important that you let the TTT1 nurse or doctor know about this as soon as you can. By following this protocol, the risk of ketosis and developing ketoacidosis will be

mitigated.

16.4 Appendix 4: Amendment History

Sponsor Amendment No.	Protocol version no.	Date issued	Author(s) of changes	Details of changes made
AM02	2.0	Ethics App – 23 Nov 2020		Protocol Changes: Weight loss mitigation, 19 Risk Mitigation and visit activity corrections
AM03	2.1	Ethics App – 10th Feb 2021		Correction of Summary of changes for previous AM02.<br>Corrected pregnancy testing schedule.
AM06	3.0	Ethics App – 16th Feb 2022		Definition of AESIs narrowed to include only ketosis and level 2 hypoglycaemia; lowering eGFR inclusion criterion; correction of DSQOLS to licensed version.
AM08	4.0	Ethics App – 26th June 2023		Procedure for recruitment at other sites within NHS GGC described; procedures for rescreening of participants experiencing delay between the screening visit and Visit 1; updates; correction of errors
AM09	5.0	Not required		Following discussion at TMG (21st Sept) and Sponsor reps (26th Sept), it was agreed it was appropriate to remove the word "Summary" and addition of text to avoid duplication of sending PIS twice, from page 33 of the protocol. (Recruitment section). While the Summary PIS was produced and approved during the study set up, in practice it has not been required, with telephone calls and full PIS being sent (following initial discussion in clinics) being the preferred method. For this reason the Summary PIS has not been updated in line with the protocol amendments and is now out of date, therefore is not appropriate to be sent.
AM11	6.0	Awaited		Clarification to the recruitment process, Updated information of interim analysis, now planned. Changes to further align with US protocol with regard withdrawals.

List details of all protocol amendments here whenever a new version of the protocol is produced. Protocol amendments must be submitted to the Sponsor for approval prior to submission to the REC committee or MHRA.

16.5 Appendix 5: PAID Questionnaire

Problem Areas In Diabetes (PAID) scale

Instructions: Which of the following diabetes issues are **currently** a problem for you? Tick the box that gives the best answer for you. Please provide an answer for each question.

		Not a problem	Minor problem	Moderate problem	Somewhat serious problem	Serious problem
1	Not having clear and concrete goals for your diabetes care?	☐ 0	☐ 1	☐ 2	☐ 3	☐ 4
2	Feeling discouraged with your diabetes treatment plan?	☐ 0	☐ 1	☐ 2	☐ 3	☐ 4
3	Feeling scared when you think about living with diabetes?	☐ 0	☐ 1	☐ 2	☐ 3	☐ 4
4	Uncomfortable social situations related to your diabetes care (e.g. people telling you what to eat)?	☐ 0	☐ 1	☐ 2	☐ 3	☐ 4
5	Feelings of deprivation regarding food and meals?	☐ 0	☐ 1	☐ 2	☐ 3	☐ 4
6	Feeling depressed when you think about living with diabetes?	☐ 0	☐ 1	☐ 2	☐ 3	☐ 4
7	Not knowing if your mood or feelings are related to your diabetes?	☐ 0	☐ 1	☐ 2	☐ 3	☐ 4
8	Feeling overwhelmed by your diabetes?	☐ 0	☐ 1	☐ 2	☐ 3	☐ 4
9	Worrying about low blood glucose reactions?	☐ 0	☐ 1	☐ 2	☐ 3	☐ 4
10	Feeling angry when you think about living with diabetes?	☐ 0	☐ 1	☐ 2	☐ 3	☐ 4
11	Feeling constantly concerned about food and eating?	☐ 0	☐ 1	☐ 2	☐ 3	☐ 4
12	Worrying about the future and the possibility of serious complications?	☐ 0	☐ 1	☐ 2	☐ 3	☐ 4
13	Feelings of guilt or anxiety when you get off track with your diabetes management?	☐ 0	☐ 1	☐ 2	☐ 3	☐ 4
14	Not 'accepting' your diabetes?	☐ 0	☐ 1	☐ 2	☐ 3	☐ 4
15	Feeling unsatisfied with your diabetes physician?	☐ 0	☐ 1	☐ 2	☐ 3	☐ 4
16	Feeling that diabetes is taking up too much of your mental and physical energy every day?	☐ 0	☐ 1	☐ 2	☐ 3	☐ 4
17	Feeling alone with your diabetes?	☐ 0	☐ 1	☐ 2	☐ 3	☐ 4
18	Feeling that your friends and family are not supportive of your diabetes management efforts?	☐ 0	☐ 1	☐ 2	☐ 3	☐ 4
19	Coping with complications of diabetes?	☐ 0	☐ 1	☐ 2	☐ 3	☐ 4
20	Feeling 'burned out' by the constant effort needed to manage diabetes?	☐ 0	☐ 1	☐ 2	☐ 3	☐ 4

© Joslin Diabetes Center, 1999 (www.joslin.org). All rights reserved. The copyright holder/developer has given permission for the questionnaire to be reproduced in this toolkit. Readers of the toolkit are permitted to reproduce the questionnaire for clinical use and non-commercial research purposes. Readers of the toolkit are not permitted to use the questionnaire for commercial research purposes and must seek permission from the copyright holder/developer to do so.

16.6 Appendix 6: DSQOLS Questionnaire

Diabetes specific quality of life scale (DSQOLS)

Date____________ Name____ DOB______________

Please tick the appropriate box for each question –

Which burdens and restrictions from diabetes and its treatment have you experienced during the last four weeks?…	This statement meets my point of view…					
	5. Perfectly	4. Quite good	3. A little	2. Rather not	1. Hardly	0. Not at all
1. It bothers me that I have to measure my blood glucose so often	☐	☐	☐	☐	☐	☐
2. It burdens me that I always have to think about my nutrition.	☐	☐	☐	☐	☐	☐
3. I suffer from pain because of diabetes.	☐	☐	☐	☐	☐	☐
4. Because of diabetes the relationship to my partner has become worse.	☐	☐	☐	☐	☐	☐
5. I am worried about the fact that my life could be shorter because of diabetes.	☐	☐	☐	☐	☐	☐
6. I have the impression that I am less attractive for others because of diabetes.	☐	☐	☐	☐	☐	☐
7. Because of diabetes I feel sad or depressed	☐	☐	☐	☐	☐	☐
8. I am worried about my future health	☐	☐	☐	☐	☐	☐
9. It is a burden for me how other people react to my diabetes	☐	☐	☐	☐	☐	☐
10. I feel nervous and restless when I think about episodes of low blood sugar						
11. I feel tired and exhausted	☐	☐	☐	☐	☐	☐
12. It bothers me that I have to spend so much time on my diabetes treatment	☐	☐	☐	☐	☐	☐
13. Because of diabetes traveling is complicated and troublesome	☐	☐	☐	☐	☐	☐
14. I get anxious and nervous when I think about the dangers of episodes of low blood glucose	☐	☐	☐	☐	☐	☐

	5. Perfectly	4. Quite good	3. A little	2. Rather not	1. Hardly	0. Not at all
15. Diabetes prevents me from spontaneous physical activities	☐	☐	☐	☐	☐	☐
16. I suffer from frequent urination	☐	☐	☐	☐	☐	☐
17. Because of high blood glucose values after a meal I often feel unwell or less efficient	☐	☐	☐	☐	☐	☐
18. Because of diabetes I feel anxious or threatened	☐	☐	☐	☐	☐	☐
19. I suffer from thirst or having a dry mouth	☐	☐	☐	☐	☐	☐
20. Other people find it hard to understand my problems with diabetes treatment	☐	☐	☐	☐	☐	☐
21. I feel physically unwell	☐	☐	☐	☐	☐	☐
22. I unnecessarily worry too much about episodes of low blood glucose	☐	☐	☐	☐	☐	☐
23. I have to give up good-tasting foods	☐	☐	☐	☐	☐	☐
24. I cannot eat as much as I want of certain foods	☐	☐	☐	☐	☐	☐
25. Because of diabetes I often have physical complaints	☐	☐	☐	☐	☐	☐
26. Diabetes interferes with my future plans	☐	☐	☐	☐	☐	☐
27. I get an uncomfortable feeling when I think about the dangers of a severe episode of low blood glucose	☐	☐	☐	☐	☐	☐
28. Because of diabetes I have less contact with friends or acquaintances	☐	☐	☐	☐	☐	☐
29. I wish I could eat more the way I want to, without having to plan everything beforehand	☐	☐	☐	☐	☐	☐
30. I suffer from frequent infections, itching or skin problems	☐	☐	☐	☐	☐	☐
31. I am concerned about getting into embarrassing situations because of episodes of	☐	☐	☐	☐	☐	☐

	5. Perfectly	4. Quite good	3. A little	2. Rather not	1. Hardly	0. Not at all
low blood glucose						
32. I feel weak or lazy	☐	☐	☐	☐	☐	☐
33. I am dissatisfied with the amount of time I have to spend for medical consultations	☐	☐	☐	☐	☐	☐
34. Because of my diabetes I cannot spend my free time the way I would like	☐	☐	☐	☐	☐	☐
35. I feel like a disabled person	☐	☐	☐	☐	☐	☐
36. I am worried that I could sustain physical injury in the event of a severe episode of low blood glucose	☐	☐	☐	☐	☐	☐
37. It bothers me that I cannot eat like other people	☐	☐	☐	☐	☐	☐
38. I am often worried about the long-term	☐	☐	☐	☐	☐	☐
39. Because of diabetes other people treat me like a "sick person"	☐	☐	☐	☐	☐	☐
40. Because of diabetes I cannot pursue my hobbies as I wish	☐	☐	☐	☐	☐	☐
41. I have to frequently think about diabetes and its consequences	☐	☐	☐	☐	☐	☐
42. It bothers me that I cannot eat as spontaneously as people who do not have diabetes	☐	☐	☐	☐	☐	☐
43. It upsets my stomach when I think about the dangers of severe episodes of low blood glucose	☐	☐	☐	☐	☐	☐
44. Diabetes constantly causes problems while dealing with other people	☐	☐	☐	☐	☐	☐

With respect to diabetes treatment it is very important to me that, resp. it would be my goal that …	5. Very Important	4. Quite Important	3. Rather Important	2. Rather Unimportant	1. Quite Unimportant	0. Totally Unimportant
45. my blood glucose values are always below 140 mg%	☐	☐	☐	☐	☐	☐
46. my blood glucose values are as constant as possible	☐	☐	☐	☐	☐	☐
47. I can plan my leisure flexibly	☐	☐	☐	☐	☐	☐
48. I can avoid even mild hypoglycaemia	☐	☐	☐	☐	☐	☐
49. I can avoid late complications of diabetes in any case, resp. avoid their progress.	☐	☐	☐	☐	☐	☐
50. I can eat flexibly in the way that I can choose the kind, amount and time of meals	☐	☐	☐	☐	☐	☐
51. I am as physically fit as possible.	☐	☐	☐	☐	☐	☐
52. I can in any case avoid severe hypoglycaemia with loss of consciousness	☐	☐	☐	☐	☐	☐
53. I have to measure my blood glucose as rarely as possible.	☐	☐	☐	☐	☐	☐
54. Other people don't find out that I have diabetes,	☐	☐	☐	☐	☐	☐

How satisfied have you been during the last weeks with …	0. Very Satisfied	1. Quite Satisfied	2. Rather Satisfied	3. Rather dissatisfied	4. Quite dissatisfied	5. Totally dissatisfied
55. the level of your blood glucose values?	☐	☐	☐	☐	☐	☐
56. The stability of your blood glucose values?	☐	☐	☐	☐	☐	☐
57. The flexibility of planning your leisure?	☐	☐	☐	☐	☐	☐
58. The frequency of mild hypoglycaemia?	☐	☐	☐	☐	☐	☐
59. your assurance of being protected against late complications, resp. of being able to avoid their progress?	☐	☐	☐	☐	☐	☐
60. The flexibility of your diet?	☐	☐	☐	☐	☐	☐
61. your physical fitness?	☐	☐	☐	☐	☐	☐
62. your assurance of being protected against severe hypoglycaemia with loss of consciousness?	☐	☐	☐	☐	☐	☐
63. the frequency of your blood glucose self-monitoring?	☐	☐	☐	☐	☐	☐
64. The extent to what other people are informed about your diabetes?	☐	☐	☐	☐	☐	☐

FULL/LONG TITLE OF THE TRIAL
Triple Therapy for Type 1 Diabetes with Insulin, Semaglutide and Dapagliflozin (TTT1) - UK

SHORT STUDY TITLE / ACRONYM
TTT1 (UK)

PROTOCOL VERSION NUMBER AND DATE
Version 7.0 12th Dec 2024

RESEARCH REFERENCE NUMBERS

**IRAS Number:**	254431
**EudraCT Number:**	2018-004120-11
**ISRCTN Number / Clinical trials.gov Number:**	NCT03899402
**SPONSOR Number:**	GN17DI706
**FUNDER Number:**	3-SRA-2019-669-M-B

SIGNATURE PAGE

The undersigned confirm that the following protocol has been agreed and accepted and that the Chief Investigator agrees to conduct the trial in compliance with the approved protocol and will adhere to the principles outlined in the Medicines for Human Use (Clinical Trials) Regulations 2004 (SI 2004/1031), amended regulations (SI 2006/1928) and any subsequent amendments of the clinical trial regulations, Good Clinical Practice (GCP) guidelines, the Sponsor's (Standard Operating Procedures (SOPs), and other regulatory requirements as amended.
I agree to ensure that the confidential information contained in this document will not be used for any other purpose other than the evaluation or conduct of the clinical investigation without the prior written consent of the Sponsor
I also confirm that I will make the findings of the study publicly available through publication or other dissemination tools without any unnecessary delay and that an honest accurate and transparent account of the study will be given; and that any discrepancies from the study as planned in this protocol will be explained.

For and on behalf of the Study Co-Sponsor (University of Glasgow):
Signature: .... Date: ....../....../......
Name (please print): ....
Position: ....

For and on behalf of the Study Co-Sponsor (NHS Greater Glasgow and Clyde):
Signature: .... Date: ....../....../......
Name (please print): ....
Position: ....

UK Chief Investigator:
Signature: .... Date: ....../....../......
Name: (please print): John Petrie ....
Position: Professor of Diabetes..

Statistician:
Signature: .... Date: ....../....../......
Name: (please print): ....

Position: ....

KEY TRIAL CONTACTS

UK Chief Investigator	Professor John R Petrie<br>Professor of Diabetes<br>Director, Robertson Centre for Biostatistics<br>Director, Glasgow Clinical Trials Unit<br>School of Health and Wellbeing<br>College of Medical, Veterinary and Life Sciences<br>University of Glasgow<br>Clarice Pears Building, Level 5<br>90 Byres Road<br>Glasgow<br>G12 8TB<br>Secretary +44(0)141 330 4744<br>john.petrie@glasgow.ac.uk
UK Co- Investigator	Dr James Boyle<br>Consultant Diabetologist<br>Glasgow Royal Infirmary<br>84 Castle Street, Glasgow, G4 0SF<br>Tel: : +44(0)141 211 5996<br>james.boyle@glasgow.ac.uk
Trial Co-ordination	Miss Lisa Jolly<br>Research and Innovation<br>NHS Greater Glasgow & Clyde<br>Ward 11, Dykebar Hospital<br>Grahamston Road<br>Paisley, PA2 7DE<br>Lisa.Jolly@ggc.scot.nhs.uk
Co-Sponsor	NHS Greater Glasgow & Clyde<br><br>Contact: Dr Maureen Travers<br>Research and Development Management Office<br>Research and Innovation<br>NHS Greater Glasgow & Clyde<br>Ward 11, Dykebar Hospital<br>Grahamston Road<br>Paisley, PA2 7DE<br>Tel: +44 (0) 0141 314 4012<br>maureen.travers@ggc.scot.nhs.uk
Co-sponsor	University of Glasgow<br><br>Contact: Dr Debra Stuart<br>College of Medical, Veterinary & Life Sciences<br>Room 327 Wolfson Medical School Building<br>University Avenue<br>Glasgow G12 8QQ

	Tel: +44 (0)141 330 4539<br>Debra.Stuart@glasgow.ac.uk
Funder	JDRF<br>26 Broadway, 15th floor, New York, NY 10004, USA
Clinical Trials Unit	Glasgow Clinical Trials Unit<br>Robertson Centre for Biostatistics<br>Clarice Pears Building, Level 5<br>90 Byres Road<br>Glasgow<br>G12 8TB<br>Tel: 0141 330 4744<br>liz.anderson@glasgow.ac.uk
Statisticians	Professor Alex McConnachie<br>Robertson Centre for Biostatistics<br>Clarice Pears Building, Level 5<br>90 Byres Road<br>Glasgow<br>G12 8TB<br>Telephone: 0141 330 4744<br><br>Ms Nicola Greenlaw<br>Robertson Centre for Biostatistics<br>Clarice Pears Building, Level 5<br>90 Byres Road<br>Glasgow<br>G12 8TB
Trials pharmacist	Dr Elizabeth Douglas<br>Clinical Trials Pharmacist<br>Clinical Research and Development<br>Ward 11, Dykebar Hospital,<br>Grahamston Road | Paisley PA2 7DE<br>Tel: 0141 314 4073<br>E-mail: elizabeth.douglas@ggc.scot.nhs.uk

US Principal Investigator	Dr Paresh Dandona<br>Director, Diabetes-Endocrinology Center of Western NY<br>Chief of Endocrinology, Diabetes, Endocrinology and Metabolism, SUNY at Buffalo<br>1000 Youngs Road, Suite 105<br>Williamsville NY 14221<br>Telephone: +1(716) 535-1850<br>Fax: +1(716)-639-1200

	pdandona@kaleidahealth.org
US Co- Investigator	Dr Husam Ghanim, PhD.<br>Director, Diabetes and Endocrinology Research Lab<br>Research Associate Professor,<br>School of Medicine, State University of NY at Buffalo<br>Clinical and Translational Research Center<br>875 Ellicott St<br>Buffalo, NY 14203<br>Tel: (+1)716-881-8924<br>ghanim@buffalo.edu
Key Protocol Contributors	Professor John Petrie<br>john.petrie@glasgow.acuk<br><br>Dr Paresh Dandona<br>pdandona@kaleidahealth.org<br><br>Husam Ghanim, PhD.<br>ghanim@buffalo.edu<br><br>Dr James Boyle<br>james.boyle@glasgow.ac.uk<br><br>Dr Robert Lindsay<br>robert.lindsay@glasgow.ac.uk<br><br>Dr Joseph Timmons<br>joseph.timmons@glasgow.ac.uk<br><br>Professor Alex McConnachie<br>Alex.McConnachie@glasgow.ac.uk

TRIAL SUMMARY

Trial Title	Triple Therapy for Type 1 Diabetes with Insulin, Semaglutide, and Dapagliflozin (UK)	
Internal ref. no. (or short title)	TTT1	
Clinical Phase	Phase 3	
Trial Design	Two six-month periods: (i) open-label randomised (2:1) parallel group semaglutide vs standard of care; (ii) additional double-blind, randomised (1:1) parallel group, dapagliflozin vs placebo for those participants initially randomised to semaglutide.<br>Regardless of each randomisation, ongoing standard of care (insulin therapy) will be provided for all participants throughout the study.	
Trial Participants	Adults with type 1 diabetes for at least one year with HbA1c 7.5-11.0% on stable multiple daily injections (MDI) (four or more) of insulin or continuous subcutaneous insulin infusion (CSII) for the previous three months.	
Planned Sample Size	82 (41 in UK)	
Treatment duration	Approximately 1 year	
Follow up duration	Approximately 1 year	
Planned Trial Period	Approximately 5 years	
	Objectives	Outcome Measures
Primary	To assess in people with type 1 diabetes whether triple therapy (dapagliflozin, semaglutide and insulin) reduces HbA1c in comparison with dual therapy (placebo, semaglutide, and insulin – period 2).	HbA1c
Secondary	To assess in people with type 1 diabetes whether triple therapy (dapagliflozin, semaglutide and insulin) reduces HbA1c in comparison with standard therapy (insulin alone)	HbA1c
	To assess in people with type 1 diabetes whether dual therapy (semaglutide and insulin - period 1) reduces HbA1c in comparison with standard therapy (insulin alone)	HbA1c
	To assess in people with type 1 diabetes whether triple therapy (dapagliflozin, semaglutide and insulin) improves glucose control	- Mean fasting blood glucose<br><br>- Mean blood glucose*<br>- Percentage time in range

	as assessed by CGM, insulin requirement, body weight, blood pressure and quality of life in comparison with (a) dual therapy (placebo, semaglutide, and insulin – period 2) and (b) standard therapy (insulin alone)	- Glycaemic variability*<br>- Percentage time in hyperglycaemia Level 1 (10.1-13.9 mmol/)*<br>- Percentage time in hyperglycaemia Level 2 (>13.9 mmol/L)*<br>-Percentage time in hypoglycaemia Level 1 (3.0 – 3.8 mmol/L)*<br>** CGM (by masked Dexcom G6)*<br><br>Insulin requirement<br>Body weight<br>Systolic/ diastolic blood pressure<br>Number of antihypertensive agents<br>Diabetes Specific Quality of Life Scale (DSQOLS)<br>Problem Areas In Diabetes (PAID) questionnaire
Safety Endpoint 1	a) Triple therapy (dapagliflozin, semaglutide and insulin) in comparison with dual therapy (placebo, semaglutide, insulin – period 2)<br><br>b) Dual therapy (semaglutide and insulin - period 1) in comparison with standard therapy (insulin only)	Difference between treatment arms in:<br>(i) Rate of Level 2 (<3.0 mmol/L) hypoglycaemic events (by Dexcom G6 CGM)<br>(ii) Rate of Level 3 hypoglycaemic events (severe event with altered mental and/or physical status requiring assistance)
Safety Endpoint 2	a) Triple therapy (dapagliflozin, semaglutide and insulin) in comparison with dual therapy (placebo, semaglutide, and insulin – period 2)<br><br>b) Dual therapy (semaglutide and insulin –period 1) in comparison with standard therapy (insulin only)	Difference between treatment arms in:<br>Rate of diabetic ketoacidosis (serum ketones or urinary ketones above normal limits AND serum bicarbonate < 15 mmol/L OR blood pH < 7.3)
Investigational Medicinal Product(s)	Semaglutide<br>Dapagliflozin<br>Placebo matching dapagliflozin	
Non-investigational Medicinal Product(s)	Insulin	
Formulation, Dose, Route of Administration	Semaglutide (subcutaneous injection, once weekly) – open label by pen device (0.25 mg/ 0.5 mg/ 1.0 mg)<br>Dapagliflozin 5 mg (oral) – one then two capsules daily (up-titration)<br>Placebo matching dapagliflozin	

FUNDING AND SUPPORT IN KIND

FUNDER(S)	FINANCIAL AND NON FINANCIALSUPPORT GIVEN
JDRF New York<br>26 Broadway, 15th floor, New York, NY 10004, United States	$1,440,000 (to University at Buffalo, the State University of New York, NY (SUNY Buffalo), USA; and University of Glasgow, UK)
Novo Nordisk, 800 Scudders Mill Road, Princeton Landing, NJ, Plainsboro, 08536.	$160,000 and donation of semaglutide to Glasgow site during period of supply disruption (see paragraph headed "Novo Nordisk" below)
AstraZeneca<br>1800 Concord Pike, Wilmington, DE 19803, USA	Provision of dapagliflozin and matched placebo investigational medicinal products to US site
Dexcom; Suite 6, 1 Tanfield, Edinburgh, EH3 5DA	Staff training and discounted supply of CGM sensors

ROLE OF STUDY SPONSOR AND FUNDER

NHS Greater Glasgow & Clyde and The University of Glasgow will be Co-sponsors of the UK arm of the trial. Prior to study initiation, a non-commercially funded clinical trial co-sponsorship agreement will be put in place between NHS Greater Glasgow & Clyde and The University of Glasgow. The roles and liabilities each organisation will take under The Medicines for Human Use (Clinical Trials) Regulations, 2004 SI 2001:1031 are laid out in this agreement signed by both organisations. The University of Glasgow shall be responsible for carrying out the obligations and responsibilities set out in the aforementioned agreement, and shall be deemed "sponsor" for the purposes of, Part 3 of the regulations in relation to the study. NHS Greater Glasgow & Clyde shall be responsible for carrying out the responsibilities set out in the agreement, and shall be deemed "sponsor" for the purposes of, Parts 4, 5, 6 and 7 of the Regulations in relation to the study.

The Co-Sponsors will delegate specific roles to the Chief Investigator, Glasgow CTU and other third parties. These arrangements will be clearly documented in agreements and/or the Sponsor Delegated Roles and Responsibilities Matrix.

The US arm of the trial has the same inclusion and exclusion criteria and the same pre-specified outcomes but an individualised Protocol. It will collect the same data via a common eCRF. Adverse event data from each site will be available to the trial Data and Safety Monitoring Board. The UK Co-Sponsors will have no responsibility for (and carry no liability for) the conduct of the US arm of the trial.

<u>JDRF</u>
The study has been funded by a grant from JDRF. JDRF does not have a designated role or responsibility in trial design, conduct, data analysis and interpretation, manuscript writing, and dissemination of results. Periodic reports (at least annual) in relation to progress of the trial will be submitted to the JDRF. Support from JDRF will be acknowledged in any publications related to the study.

<u>Astra Zeneca</u>
This is an investigator-initiated study. Astra Zeneca provided support by supplying investigational medicinal products (IMPs) dapagliflozin and matched placebo to the US site only. Representatives of Astra Zeneca reviewed and provided comments on drafts of the Protocol but the company does not

have a designated role or responsibility in trial design, conduct, data analysis and interpretation, manuscript writing, and dissemination of results.

Novo Nordisk
In November 2020, Novo Nordisk made up a shortfall of funding that resulted from a cut back by 10% of the main grant by the funder JDRF due to the COVID-19 pandemic. By this time, the Protocol had been finalised. The company does not have a designated role or responsibility in trial design, conduct, data analysis and interpretation, manuscript writing, or dissemination of results.

Supply shortages of semaglutide (various strengths) from 2022 onwards resulted in actual and potential disruption to participant recruitment and ongoing treatment. In response to a direct request from the UK Chief Investigator, Novo Nordisk donated supplies of semaglutide to the Glasgow site based on anticipated use during the period of disruption.

ROLES AND RESPONSIBILITIES OF TRIAL MANAGEMENT COMMITTEES/GROUPS & INDIVIDUALS

Trial Steering Committee (TSC)

The role of the TSC is to provide overall supervision of the trial and ensure that it is being conducted in accordance with the principles of GCP and the relevant regulations. The TSC will:
1. agree the trial protocol and any protocol amendments
2. ensure that conduct of the trial is fully harmonised between the two sites
3. provide advice to the investigators on all aspects of the trial

It will have an independent Chairperson and include the US and UK investigators and key protocol contributors listed above.

Decisions about continuation or termination of the trial or substantial amendments to the protocol will be the responsibility of the TSC who will advise the co-sponsors. The TSC will meet at the start of the study, and four to six monthly thereafter. The TSC will have its own Charter outlining the role and responsibilities of its members. The TSC may invite other attendees from the trial team to present or participate in discussions on particular topics. These attendees will be non-voting members.

Independent data monitoring committee (IDMC)

An IDMC will be established to include two independent medical experts (covering the domains of type 1 diabetes and metabolic disease; one of the academic clinicians will act as chair) and an independent biostatistician. The Glasgow CTU will liaise with the committee and ensure that the Committee is provided with adequate information about study progress and results.

The IDMC will have a formal Charter outlining the responsibilities of the IDMC members, Glasgow CTU and the co-sponsors. Responsibilities include:

- To protect the safety of participants recruited to the trial.
- Advising the TSC and co-sponsors if it is safe and appropriate to continue with the study
- Examining information provided by the Glasgow CTU on study recruitment, adverse eventsand outcomes
- Consider the need for additional unscheduled reviews of study data.
- Providing reports for the Project Office to forward to the TSC, ethics committees, regulatory bodies, study co-sponsors, and the BHF.

The IDMC may recommend to the TSC to terminate the study or specific aspects of the study if clear harm is observed with one treatment option compared to standard of care or placebo. Specific safety endpoints that will be reviewed include rates of hypoglycaemia and ketosis/ ketoacidosis.

The IDMC will meet approximately every six months.

Study Management Group (SMG)

Trial activity at the UK/ Glasgow site will be coordinated from NHS Greater Glasgow & Clyde (NHS GG&C) by the TTT1 Study Management Group (SMG). The SMG will consist of the UK chief investigator, co-principal investigator other co-applicants, project manager and representatives from the Glasgow CTU, NHS GG&C and The University of Glasgow. The role of the group is to monitor all

aspects of the conduct and progress of the trial in the UK, ensuring adherence to the protocol, and take appropriate action to safeguard participants and the quality of the trial itself.

Joint Study Management Group

Coordination of trial activity between the UK/ Glasgow and the US/ Buffalo site will be facilitated by the Joint TTT1 Study Management Group. This will consist of the US and UK chief investigators, co-principal investigators, other co-applicants, study coordinators, project managers and governance/ administrative representatives as appropriate from State University of New York (SUNY), the Glasgow CTU, NHS GG&C and The University of Glasgow. It will meet approximately every 4-6 weeks. The role of the group is to coordinate all aspects of the conduct and progress of the trial between the US and UK sites, ensuring protocol adherence, and taking appropriate action to safeguard participants and the quality of the trial itself.

Protocol contributors

The protocol has been developed by a group with extensive clinical and research experience in type 1 diabetes and clinical trials including Professor John Petrie and Professor Paresh Dandona. Professor Alex McConnachie has research expertise in design, conduct, analysis and interpretation of clinical trials and biostatistical methods.

KEY WORDS:

Adjunct therapy
Dapagliflozin
Glycated haemoglobin (HbA1c)
Semaglutide
Type 1 diabetes

LIST of CONTENTS

RESEARCH REFERENCE NUMBERS 2
SIGNATURE PAGE 3
KEY TRIAL CONTACTS 4
LIST of CONTENTS..14
LIST OF ABBREVIATIONS..15
TRIAL FLOW CHART..17
1 BACKGROUND 20
2 RATIONALE..21
3 OBJECTIVES AND OUTCOME MEASURES/ ENDPOINTS.......................................25
4 TRIAL DESIGN 28
5 STUDY SETTING 28
6 ELIGIBILITY CRITERIA 28
7 TRIAL PROCEDURES..30
8 TRIAL MEDICATION…………………………………………………………...58
9 PHARMACOVIGILANCE……………………………………………………63
10 STATISTICS AND DATA ANALYSIS...71
11 DATA HANDLING 73
12 MONITORING, AUDIT & INSPECTION 74
13 ETHICAL AND REGULATORY CONSIDERATIONS...75
14 DISSEMINATION POLICY…………………………………………………77
15 REFERENCES……………………………………………………………..78
16. APPENDICES..83

LIST OF ABBREVIATIONS

ACS	Acute Coronary Syndrome
AUC	Area Under the Curve
AE	Adverse Event
AR	Adverse Reaction
CA	Competent Authority
CABG	Coronary Artery Bypass Graft
CGM	Continuous Glucose Monitoring
CHI	Community Health Index
CI	Chief Investigator
CRO	Contract Research Organisation
CRP	C-Reactive Protein
CSII	Continuous Subcutaneous Insulin Infusion
CTA	Clinical Trial Authorisation
CTU	Clinical Trials Unit
CTIMP	Clinical Trial of Investigational Medicinal Product
CV	Cardiovascular
CVA	Cerebrovascular Accident
DKA	Diabetic Ketoacidosis
DSUR	Development Safety Update Report
EC	European Commission
ECG	Electrocardiogram
eCRF	Electronic Case Report Form
EDTA	Ethylenediaminetetraacetic acid
eGFR	Estimated Glomerular Filtration Rate
EMEA	European Medicines Agency
EU	European Union
EUCTD	European Clinical Trials Directive
EudraCT	European Clinical Trials Database
EudraVIGILANCE	European database for Pharmacovigilance
FBC	Full Blood Count
FGM	Flash Glucose Monitoring
GCP	Good Clinical Practice
GDG	Guideline Development Group
GDPR	General Data Protection Regulation
GLP-1	Glucagon Like Peptide-1
GLP-1RA	Glucagon Like Peptide-1 Receptor Agonist
GMP	Good Manufacturing Practice
HbA1c	Glycated haemoglobin
IB	Investigator Brochure
ICF	Informed Consent Form
ICH	International Conference on Harmonisation of technical requirements for registration of pharmaceuticals for human use.
IDMC	Independent Data Monitoring Committee
IMP	Investigational Medicinal Product
IMPD	Investigational Medicinal Product Dossier

ISF	Investigator Site File
ISRCTN	International Standard Randomised Controlled Trials Number
IWRS	Interactive Web-based Randomisation System
LPLV	Last Patient Last Visit
MA	Marketing Authorisation
MDRD	Modification of Diet in Renal Disease
MODY	Maturity Onset Diabetes of the Young
MHRA	Medicines and Healthcare products Regulatory Agency
MI	Myocardial Infarction
MDI	Multiple Daily Injections
NHS GG&C	National Health Service Greater Glasgow & Clyde
NHS R&D	National Health Service Research & Development
NICE	The National Institute for Health and Care Excellence
NIMP	Non-Investigational Medicinal Product
PI	Principal Investigator
PIC	Participant Identification Centre
PIS	Participant Information Sheet
PV	Pharmacovigilance
QA	Quality Assurance
QC	Quality Control
QoL	Quality of Life
QP	Qualified Person
RCT	Randomised Control Trial
REC	Research Ethics Committee
SAE	Serious Adverse Event
SAP	Statistical Analysis Plan
SAR	Serious Adverse Reaction
SDV	Source Data Verification
SGLT2	Sodium Glucose Transporter 2
SOP	Standard Operating Procedure
SUNY	State University of New York
SmPC	Summary of Product Characteristics
SSI	Site Specific Information
SUSAR	Suspected Unexpected Serious Adverse Reaction
TIA	Transient Ischaemic Attack
TMG	Trial Management Group
TSC	Trial Steering Committee
TMF	Trial Master File
UB	University of Buffalo
ULN	Upper Limit of Normal reference range
WOCBP	Women Of Childbearing Potential

TRIAL FLOW CHART

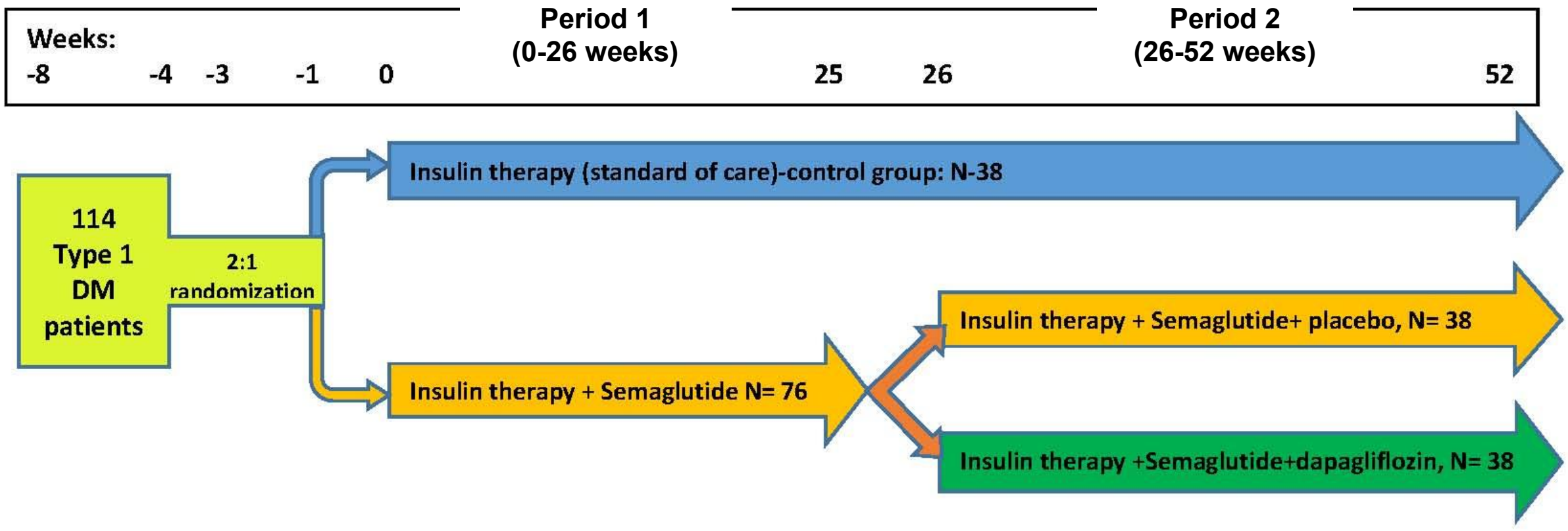

Revised Flow Chart
At this time, a revised sample size in Period 2 of around 23 evaluable participants per group for dapagliflozin vs placebo. This reduces the recruitment target for first randomisation from n=114 to n=82

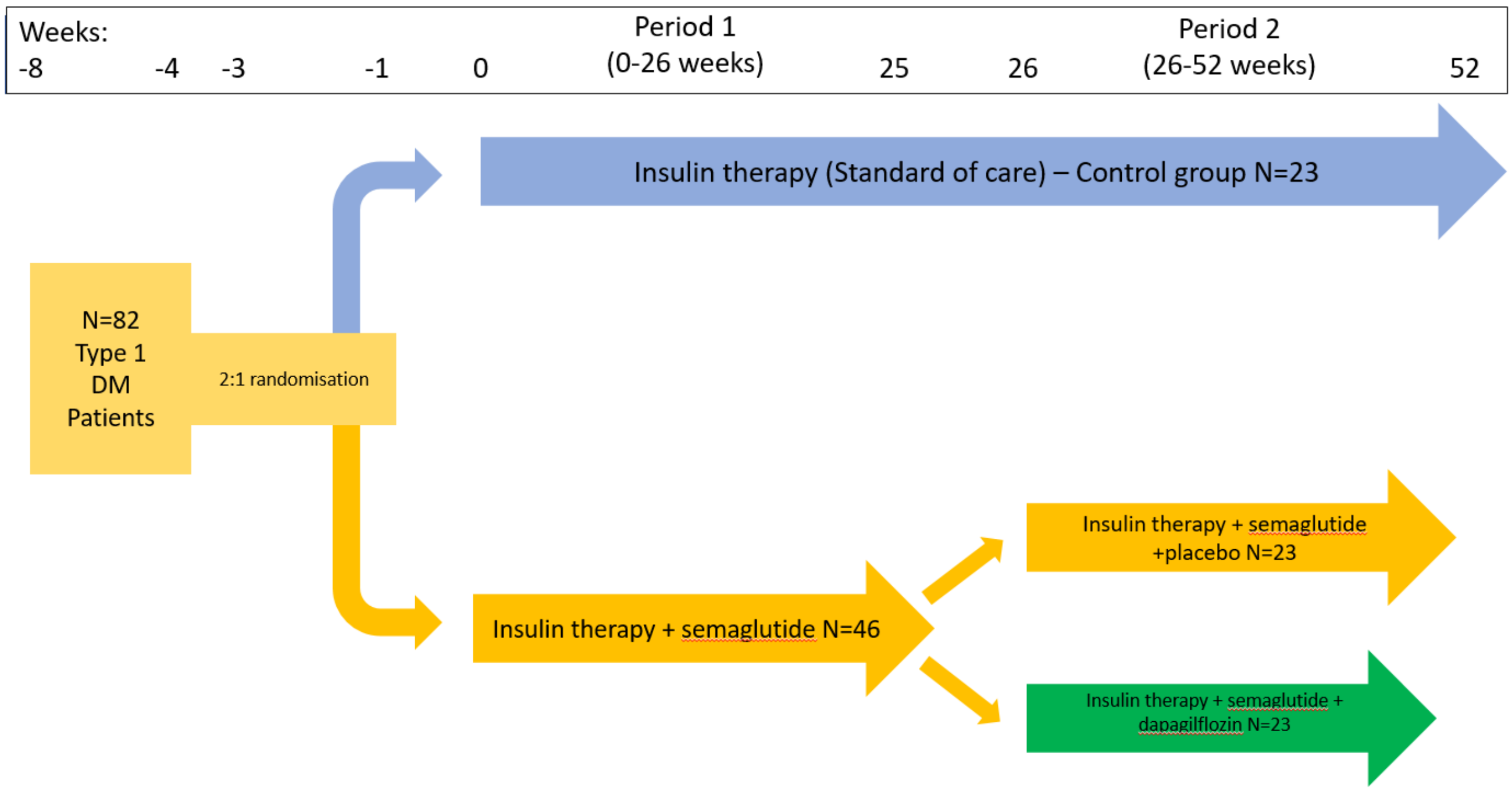
Weeks:
-8
-4
-3
-1
0
Period 1
(0-26 weeks)
25
26
Period 2
(26-52 weeks)
52
Insulin therapy (Standard of care) – Control group N=23
N=82
Type 1
DM
Patients
2:1 randomisation
Insulin therapy + semaglutide N=46
Insulin therapy + semaglutide
+placebo N=23
Insulin therapy + semaglutide +
dapagilflozin N=23

SCHEDULE OF ASSESSMENTS

Visit #	-	1	2	3	4[3]	5[3]	6	7	8	9	10	11[3]	12	13	14	15	16
**Week# (±7 days; see below)**	**-8 to -4**	**-3**	**-1**	**0**	**1**	**2**	**4**	**13**	**23**	**25**	**26**	**27**	**31**	**39**	**49**	**51**	**52**
Study Procedures	Screening	Run-in period		Randomise							Randomise						End
Informed Consent	X																
Fasting visit			X	X				X		X	X			X		X	X
History and demographics	X																
Physical	X																X
Education on hypoglycaemia and ketosis avoidance				X							X						
BP, heart rate, weight	X	X	X	X	X	X	X	X	X	X	X	X	X	X	X	X	X
Bloods (FBC, kidney and liver profiles)	X			X				X			X		X	X			X
C-peptide	X										X						X
Pregnancy test	X			X			X[1]	X[1]	X[1]		X[1]		X[1]	X[1]	X[1]	X[1]	X[1]
Dispense open-label semaglutide[2]				X		X[2]	X[2]	X[2]			X[2]			X[2]			
Titrate open-label semaglutide[2]						X[2]	X[2]	X[2]									
Dose Semaglutide (on-site if dose is due on visit day)				X	X	X											
Dispense dapagliflozin/placebo											X	X		X			
Dapagliflozin/placebo return capsule counts												X		X			X
HbA1c	X			X				X		X				X			X
Blood endpoints (including fructosamine)				X				X			X			X			X
Masked CGM fit (enter data this visit)		X							X						X		
Masked CGM read				X							X						X
Daily glucose monitoring review		X	X	X	X	X	X	X	X	X	X	X	X	X	X	X	X
Daily urinary ketones review												X	X	X	X	X	X
Insulin dose review		X	X	X	X	X	X	X	X	X	X	X	X	X	X	X	X
Ketone bodies (blood/urine)	X	X	X	X	X	X	X	X	X	X	X	X	X	X	X	X	X
Adverse Event reporting		X	X	X	X	X	X	X	X	X	X	X	X	X	X	X	X
Meal Challenge Test			X							X						X	
24 hr Urine dispensed			X				X			X			X			X	
24 hr Urine collected				X				X			X			X			X
Quality of life questionnaires				X							X						X

N.B. Safety phone calls will be conducted at weeks 3, 5, 8, 16, 20, 29, 35, 44 and at two weeks after the end of the study to document any side effects or other changes. **Abbreviations:** see pages 14-15
Additional visits or phone calls may be scheduled with the verbal consent of the participant if study team believe necessary e.g. closer monitoring is appropriate (see Patient Information Sheet)
Visit dates should be within seven days of the visit time; however, a longer or shorter interval does not constitute a protocol deviation
[1]Not required in women randomised to "Insulin therapy (standard of care)
[2]According to randomisation schedule: resupply of open-label semaglutide after Visit 3 will be according to individual requirements as tolerated (see Section 8.3)
[3] Visits 4, 5 and 11 have the option of being carried out by telephone for those participants allocated to the standard care arm only.

STUDY PROTOCOL
Triple Therapy for Type 1 Diabetes with Insulin, Semaglutide, and Dapagliflozin

1 BACKGROUND

The control of glucose homeostasis in type 1 diabetes is fragile since β-cell reserve is negligible. Traditionally, insulin has been the only treatment approach available. This often leads to erratic blood glucose control given the challenge of matching exogenous insulin to mealtime glucose excursions in the face of a lack of significant endogenous insulin to compensate for variation in requirements in response to meals and activity levels. Injected insulin boluses rarely match precise insulin requirements either in terms of dose administered or bio-availability. The result often is wide fluctuations in blood glucose including episodes when it is too low (hypoglycaemia). Based on our work to date and other published reports, previous attempts to add other glucose-lowering medications to insulin therapy yielded minimal benefits on glycaemia (although metformin may have other benefits) (1-3).

Dandona et al. have shown in two preliminary retrospective studies that the addition of liraglutide, a Glucagon Like Peptide -1 receptor agonist (GLP-1RA), to insulin results in consistent improvements in glycemic control in type 1 diabetes as demonstrated by reductions in HbA1c, glycemic excursions, and insulin requirements accompanied by improvements in body weight and systolic blood pressure (4, 5). As collaborators in the present trial, they also completed the first prospective randomised controlled trial of this drug in 72 people with type 1 diabetes. The data not only confirmed improvements in glycemic control (HbA1c), body weight and systolic BP, but also demonstrated a reduction in appetite, carbohydrate intake and low-grade inflammation (6). Importantly, quality of life was also improved. Their recent studies in type 2 diabetes have revealed that systolic blood pressure reduction with GLP-1RA (exenatide) treatment in type 2 diabetes is associated with increased plasma concentrations of ANP, cGMP and cAMP along with reduced concentrations of angiotensinogen, renin and angiotensin II (7). It is possible that these mechanisms play a role in the prevention of cardiovascular complications in type 1 diabetes. There have been no trials to date of semaglutide (a weekly and potent GLP-1RA) in type 1 diabetes.

GLP-1RAs, therefore, have great potential as adjunct therapy for type 1 diabetes. However, mean HbA1c was still ≥7.0% after liraglutide treatment (6) and larger pharma-sponsored trials (ADJUNCT-1 and ADJUNCT-2) reported increased rates of hypoglycaemia (8, 9). In seeking further improvement, the advent of Sodium-GLucose coTransporter 2 (SGLT2) inhibitors is of importance since their mode of action is independent of insulin secretion. Introduced for use in type 2 diabetes, SGLT2 inhibitors are selective antagonists of the sodium-glucose transporter subtype expressed in the proximal convoluted tubules of the kidneys. They act by inhibiting renal glucose re-absorption and therefore stimulating excretion of 60-100 grams per day of glucose in urine. SGLT2 inhibitors have been studied extensively in type 2 diabetes, including the landmark cardiovascular outcome trial EMPA-REG (10), but data have only more recently begun to accumulate in type 1 (11). Short term effects on urine glucose excretion of remogliflozin and empagliflozin are similar to those seen in type 2 diabetes and these translate into improved glycaemic and other metabolic indices including HbA1c, fasting blood glucose, rates of hypoglycaemia, daily insulin dose and weight (12). Glucose-lowering efficacy is attenuated in individuals with eGFR < 45/ml/min/1.73 m^2.(13) Weight loss accompanying increased urinary glucose excretion could translate into reduced cardiovascular complications as in type 2 diabetes, but there are no data so far available to support this hypothesis. More recently, in the Phase 3 trials DEPICT-1 and DEPICT-2 Dandona et al (PI of the US arm of this trial) showed that dapagliflozin improves glycaemic control and HbA1c over six months by about 0.45% without increasing hypoglycaemia and as in other conditions reduces urinary albumin excretion (14,15,16,17). In comparison to other SGLT2 inhibitors studied for use in type 1 diabetes [sotagliflozin and empagliflozin (18, 19)] the risk of diabetic ketoacidosis was relatively modest (see below). Both dapagliflozin and sotagliflozin were approved early in 2019 by the EU Committee for Medicinal Products for Human Use (CHMP) as adjunct therapy in type 1 diabetes for individuals with BMI≥27 kg/m (20,21)2. In August 2019, dapagliflozin (5 mg/ day) was recommended by the National Institute

for Health and Care Excellence as cost-effective for this indication in NHS England (22 However, dapagliflozin did not gain approval for this indication in the USA from the FDA (23). In November 2021, the manufacturer AstraZeneca voluntarily removed this Europe- and UK-specific indication (24) explicitly “not due to any safety concern for dapagliflozin in any condition, including type 1 diabetes” (25).

2 RATIONALE

General hypothesis: adding an SGLT2 inhibitor (dapagliflozin) to a combination of insulin and semaglutide will improve glycaemic control, weight and blood pressure in adults with type 1 diabetes.
Our protocol is designed to allow an objective assessment of whether this triple therapy combination will work effectively and safely so that a majority of people with type 1 diabetes can reach a glycaemic target HbA1c of ≤7.0%. In addition, since both semaglutide and dapagliflozin inhibitors reduce blood pressure and weight, it will allow an assessment of the combined effect of GLP-1RAs and SGLT2 inhibitors on these outcomes. Since there are no previous data on the effects of once weekly semaglutide in type 1 diabetes, or the combination of semaglutide and dapagliflozin compared to standard insulin treatment, the design requires allocation of some participants to insulin therapy only (the current standard of care).

As part of the process by which this trial was awarded funding by JDRF, this research question was considered by lay members of the funding panel and deemed important, relevant and timely. We considered that use of placebo matching dapagliflozin in Period 2 of the trial was justified when dapagliflozin had full approval as adjunct therapy for type 1 diabetes in Europe (although not the USA) as it remained uncertain which adults with type 1 diabetes were suitable for this treatment. In their review, The UK National Institute for Health and Care Excellence (NICE) considered it likely that fewer than 10% of adults with type 1 diabetes would be initiated on SGLT2 inhibitor therapy over the first five years after their favourable decision. As individuals with a slightly lower BMI threshold will be included than recommended by the SmPC, trial participants will be more closely monitored for ketosis than was the case when dapagliflozin was approved for routine clinical care. Dapagliflozin (or placebo) will be administered orally once daily as per current licensed doses in type 2 diabetes (5 mg/day and 10 mg/day: the same doses as used in DEPICT-1 and DEPICT-2) (14,15,16,). We have not incorporated blinding for semaglutide in the design as the effects of the closely-related daily GLP-1RA liraglutide have been well-studied in double-blind trials in type 1 diabetes by ourselves and others, and individuals in both arms compared for the primary outcome comparison will be treated with this agent. (6, 8, 9). Semaglutide will be administered subcutaneously once weekly. The need to study combination adjunct therapy with SGLT 2 inhibitors and GLP-1 RAs is greater than ever following removal of the type 1 diabetes indication for SGLT2 inhibitor monotherapy in the UK and Europe.

2.1 Assessment and management of risk

a) Benefits

Semaglutide (Ozempic): is a once weekly injectable (subcutaneous) GLP1-RA licensed for reducing HbA1c in type 2 diabetes. The licensed doses are 0.25 mg weekly, 0.5 mg weekly and 1.0 mg weekly. In the trial, the starting dose is 0.25 mg weekly uptitrated if tolerated to 0.5 mg weekly after two weeks and then (again if tolerated) after a further two weeks to the maximum dose of 1.0 mg weekly. Semaglutide differs from other weekly GLP-1 receptor agonists in that its half-life is extended by binding to serum albumin i.e. it is not macromolecular. In type 2 diabetes, semaglutide therapy is associated with weight reduction and a reduction in rates of cardiovascular events (26). The licensed dose escalation schedule for routine treatment of type 2 diabetes is at four weekly intervals (27) but in a Phase 2 trial in type 2 diabetes authored by one of this trial's investigators (JP) doses were uptitrated at one-to-two weekly intervals and to higher doses (up to 1.6 mg weekly) (28). Phase 3 trials are currently in progress (for the indication of obesity) using a 2.4 mg semaglutide weekly. It

should be noted that semaglutide 0.25 mg is considered too low a dose for maintenance therapy in type 2 diabetes but it is unclear whether this may have a beneficial effect in type 1 diabetes at this dose.

While semaglutide once weekly has not been investigated previously in type 1 diabetes, liraglutide (a GLP-1 RA for daily administration with a similar molecular structure that binds less avidly to serum albumin) has previously been investigated (see above) in the ADJUNCT 1 and ADJUNCT 2 trials (8,9) in which, compared with placebo it resulted in reductions in HbA1c, weight and insulin requirement, albeit with a small increase in rates of hypoglycaemia (see below).
Dapagliflozin (Forxiga): is a once daily oral SGLT2 inhibitor previously licensed in Europe for reducing HbA1c in people with type 1 diabetes and BMI ≥ 27 kg/m^2 when insulin alone does not provide adequate glycemic control despite optimal insulin therapy (23). Although only the 5 mg dose was licensed for type 1 diabetes,both 5 mg and 10 mg daily are licensed for type 2 diabetes (29). As mentioned above, the removal of the type 1 diabetes indication by AstraZeneca was not due to any safety concern for any indication, including type 1 diabetes. In the Phase 3 trials of dapagliflozin in type 1 diabetes (DEPICT-1 and DEPICT-2) both doses were used. In both DEPICT-1 and DEPICT-2 there were modest additional benefits with 10 mg compared to 5 mg dapagliflozin. For example, in DEPICT-1, insulin dose requirement was reduced by 13.2% vs placebo on 10mg daily compared with 8.8% on 5mg daily (14). Weight reduction vs placebo in DEPICT-1 at 52 weeks was by 4.5% on dapagliflozin 10mg daily compared with 2.95% in those receiving 5 mg daily. Given the propensity of insulin to encourage weight gain over time and the life-long insulin requirement in T1D, sustained weight reduction and insulin dose reduction - even when modest - may be clinically important. There was no increase in the rate of hypoglycaemia in either the 5 mg or 10 mg groups compared with placebo. (14,15)
Dapagliflozin therapy in type 2 diabetes has been demonstrated to be associated with cardiovascular and renal risk reduction: in DECLARE-TIMI, DAPA-HF and DAPA-CKD the 10 mg dose was used (30, 31, 32). Such cardiovascular and renal benefits may translate to type 1 diabetes with appropriate monitoring and risk mitigation, although support for such measures by the manufacturer were withdrawn in the UK at the time of the removal of the type 1 diabetes indication . Given these potential benefits with relatively low increased risk, the 10 mg rather than 5 mg dose was selected for steady state treatment in this protocol.

b) Risks/ side effects Please also refer to the respective UK SmPCs (27, 29)

Semaglutide:
Very common: nausea, diarrhoea; hypoglycaemia (when used with insulin).
Common: vomiting, abdominal pain, constipation, gastro-oesophageal reflux, cholelithiasis, weight loss.
Uncommon: injection site reactions, hypersensitivity, delayed gastric emptying.
Rare: Anaphylactic reaction.

Hypoglycaemia/ ketosis: Semaglutide once weekly has not been studied previously in type 1 diabetes; however, the ADJUNCT-1 and ADJUNCT-2 trials examined the role of liraglutide once daily (a similar molecule in the GLP-RA class that binds less avidly to serum albumin). Although HbA1c and weight were reduced by both 1.2 mg and 1.8 mg liraglutide, rates of symptomatic hypoglycaemia increased by 20-30%. In ADJUNCT-1, insulin doses were re-uptitrated quite rapidly towards baseline levels after an initial dose reduction, but in ADJUNCT-2 were “capped” (and therefore remained lower). Perhaps for that reason, an increase in hypoglycaemia was less consistent in ADJUNCT -2 but there was an excess of ketosis at the higher (1.8 mg) dose, presumably due to under-insulinisation (no lower limit for insulin dose reduction was set). It should be noted that monitoring of participants by site investigators in the ADJUNCT trials was less intensive than will be the case in the present trial with

less frequent opportunities for insulin dose titration (see Section 7). Importantly, participants at high risk of hypoglycaemia, (including those with a history of severe hypoglycaemia and those with hypoglycaemia unawareness) were not excluded from the ADJUNCT trials (8,9).

Retinopathy: In the SUSTAIN-6 cardiovascular outcome trial of semaglutide in type 2 diabetes, rapid reduction of HbA1c was associated with progression of existing proliferative retinopathy in those with a high baseline HbA1c ("early worsening"/ "normoglycaemic re-entry"). However, there was no evidence of worsening of retinopathy on treatment in the trials SUSTAIN 1-5 in which those with proliferative retinopathy were excluded (33, 3). In clinical trials of duration up to one year involving 4,807 patients with type 2 diabetes, adverse events related to diabetic retinopathy were reported in similar proportions of subjects treated with semaglutide (1.7%) and comparators (2.0%) (33).

Other adverse effects: In type 2 diabetes, gastrointestinal adverse effects of semaglutide can be prevented in the majority of cases by careful dose uptitration (28, 34) although up to 5% are ultimately unable to tolerate it and in post-marketing reports (updated SMPC 2024) intestinal obstruction occurs with "unknown" frequency. In our previous trial with liraglutide in T1D, adverse effects occurred with a similar frequency to that observed in T2D (in which there is extensive clinical experience); on this basis it is expected that this will also be the case with semaglutide (8,9).

Dapagliflozin:
Very common: hypoglycaemia (when used with insulin).

Common: ketoacidosis, genital infections (vulvovaginitis, balanitis), urinary tract infection, diabetic ketoacidosis, increased creatinine.

In the DEPICT-1 study, ketoacidosis occurred in 4 of 277 participants on 5mg dapagliflozin, 5 of 296 participants on 10 mg and 3 of 260 participants in the placebo group (summarised as 1%, 2% and 1% respectively) with one severe episode in each group (14). In pooled analyses of DEPICT-1 and DEPICT-2 (14,15) there was, however, an approximate threefold elevated rate of DKA adjudicated as "definite" over placebo on active therapy: 2.0% on 5 mg dapagliflozin, 1.9% on 10 mg and 0.6% on placebo – i.e. rates of DKA were similar with the two doses (14,15,16). Rates of DKA with dapagliflozin were described as "occurring in at least 1 per 100 patients" by the manufacturer at the time the indication was removed (24). Rates of DKA with sotagliflozin, the other SGLT2 inhibitor licensed for type 1 diabetes, were somewhat higher (2.9% on 200 mg, 3.8% on 400 mg, 0.2% on placebo) in pooled analyses of Phase 3 trials (19,21).

Increased creatinine: in 13 studies summarized in the Summary of Product Characteristics increases in serum creatinine (generally transient) occurred in 3.2% vs 1.8% of individuals with eGFR ≥ 60 ml/min/1.73 m^2 who received dapagliflozin 10 mg vs placebo rising to 18.5% vs 9.3% in those with eGFR <30 ml/min/1.73 m^2 (16,29). These changes are usually observed 4 weeks after SGLT2i initiation, recovering by week 12. Participants with eGFR < 45 ml ml/min/1.73 m^2 will not be included in the TTT1 trial and creatinine/ eGFR will be monitored at 5 weeks (Visit 12) and 13 weeks (Visit 13) after randomisation to dapagliflozin or placebo (Visit 10). Any participant who has persistent reduction in eGFR of 15 ml/min or more will meet withdrawal criteria from the trial (see Section 7.8).

Uncommon: fungal infection, volume depletion, constipation.
Not known (several case reports): Fournier's gangrene.

Fournier's gangrene is a very rare clinical entity characterized by necrotizing fasciitis of the perineal area. This has been infrequently reported in association with SGLT-2 inhibitor use in both men and

women. However, there was no evidence of increased risk in a meta-analysis of 84 clinical trials involving 42415 participants. Nevertheless, participants will be advised to seek urgent medical assistance if they experience erythema, pain, swelling of the perineal area associated with fever and malaise (this will be reinforced in the Patient Information Sheet). (36)

Amputation: An increased incidence of lower limb amputation has been reported in type 2 diabetes with the SGLT-2 inhibitor canagliflozin. However, this has not been demonstrated with dapagliflozin (not included in the UK SmPC). People with diabetes are at increased risk of amputation and good foot care is a key component of good diabetes care regardless of use of SGLT-2 inhibitors.

Placebo:
Placebo capsules contain microcrystalline cellulose.
This trial is therefore categorised by the Co-Sponsors as:

- Type B = Somewhat higher than the risk of standard medical care

See Appendix 1 (Risk Assessment)

3 OBJECTIVES AND OUTCOME MEASURES/ ENDPOINTS

3.1 Primary objective

- To assess in people with type 1 diabetes whether triple therapy (dapagliflozin, semaglutide and insulin) for six months improves glycaemic control (as measured by HbA1c) compared with dual therapy (placebo, semaglutide and insulin – Period 2).

3.2 Secondary objectives

To assess in people with type 1 diabetes whether:

- Triple therapy (dapagliflozin, semaglutide and insulin) improves glycaemic control (as measured by HbA1c) compared with standard therapy (insulin)
- Dual therapy (semaglutide and insulin – period 1) improves glycaemic control (as measured by HbA1c) in comparison with standard therapy (insulin only)
- Triple therapy (dapagliflozin, semaglutide and insulin) improves glycaemic control as assessed by a range of continuous glucose monitoring (CGM) parameters, lowers body weight, reduces insulin requirement, improves blood pressure and improves quality of life (as assessed by validated patient questionnaires) compared with dual therapy and standard therapy.

3.3 Outcome measures/endpoints

3.4 Primary endpoint/outcome

Change in HbA1c (week 26 to week 52)

3.5 Secondary endpoints/outcomes

Efficacy:

Change in HbA1c

Mean fasting blood glucose

Mean blood glucose*
Percentage time in range (3.9 - 10.0 mmol/)*
Standard deviation of blood glucose*
Percentage time in hyperglycaemia Level 1 (10.1 - 13.9 mmol/L/)*
Percentage time in hyperglycaemia Level 2 (>13.9 mmol/L) *
Percent time in hypoglycaemia Level 1 (3.0 - 3.8 mmol/)*
(by masked DEXCOM G6 CGM)*

Insulin requirement
Bodyweight
Systolic and diastolic BP
Number of antihypertensive medications
Diabetes Specific Quality of Life Scale (DSQOLS)
Problem Areas In Diabetes (PAID) questionnaire

Safety:

Rate of hypoglycemic events Level 2 (<3.0 mmol/L/ <54mg/dl)
Rate of hypoglycemic events Level 3 (severe event with altered mental and/or physical status requiring assistance)
Rate of diabetic ketoacidosis defined as elevated serum or urine ketones (greater than the upper limit of the normal range), and serum bicarbonate < 15 mmol/L or blood pH < 7.3

3.6 Tertiary endpoints/outcomes

Postprandial glucose (AUC) – meal tolerance test
Postprandial glucagon (AUC) – meal tolerance test
Postprandial FFA (AUC) – meal tolerance test
Postprandial GLP-1 (AUC) – meal tolerance test
Fructosamine

3.7 Exploratory endpoints/outcomes

Plasma and urine samples will be collected and frozen for later analysis for indices of inflammatory stress and other relevant measures.

3.8 Table of endpoints/outcomes

Endpoints	Outcome Measures	Timepoint(s) of evaluation of this outcome measure
**Primary endpoint**<br>Triple therapy (dapagliflozin, semaglutide and insulin) in comparison with dual therapy (placebo, semaglutide and insulin – period 2)	Change in HbA1c	Week 26 to Week 52
**Secondary endpoint 1**<br><br>Triple therapy (dapagliflozin, semaglutide and insulin) in comparison with standard therapy (insulin alone)	Change in HbA1c	Week 0 to Week 52
**Secondary endpoint 2**<br><br>Dual therapy (semaglutide and insulin – period 1) in comparison with standard therapy (insulin only)	Change in HbA1c	Week 0 to Week 26
**Other secondary endpoints**<br><br>a) Triple therapy (dapagliflozin, semaglutide and insulin) in comparison with dual therapy (semaglutide, insulin and placebo – period 2)<br><br>b) Dual therapy (semaglutide and insulin – period 1) in comparison with standard therapy (insulin only)	Change in:<br>- Mean fasting blood glucose (4 days)[†]<br><br>- Mean blood glucose*<br>- Percentage time in range (3.9–10 mmol/L)*<br>- Glycaemic variability (SD)*<br>- Percent time in hyperglycaemia Level 1 (10.1-13.9 mmol/L)*<br>- Percent time in	Week 26 to Week 52<br><br>Week 0 to Week 26

	hyperglycaemia Level 2 (>13.9 mmol/L)*<br>- Percent time in hypoglycaemia Level 1* (3.0-3.8 mmol/L)<br><br>*(‡usually 4 days data but may be fewer if missing data)*<br>*(*by masked Dexcom G6 CGM)*<br><br>Insulin requirement<br>Body Weight<br>Systolic and diastolic blood pressure<br>Number of antihypertensive medications<br>Diabetes Specific Quality of Life Scale (DSQOLS)<br>Problem Areas In Diabetes (PAID) questionnaire	
**Tertiary endpoints**<br>a) Triple therapy (dapagliflozin, semaglutide and insulin) in comparison with dual therapy (semaglutide, insulin and placebo –period 2)<br><br>b) Dual therapy (semaglutide and insulin – period 1) in comparison with standard therapy (insulin only)	Change in:<br>Postprandial glucose (AUC)<br>Postprandial FFA (AUC),<br>Postprandial GLP-1 (AUC)<br>Postprandial glucagon (AUC)	Week 26 to Week 52<br><br>Week 0 to Week 26
**Safety endpoint 1**<br>a) Triple therapy (dapagliflozin, semaglutide and insulin) in comparison with dual therapy (semaglutide, insulin and placebo – period 2)<br><br>b) Dual therapy (semaglutide and insulin – period 1) in comparison with standard therapy (insulin only)	Difference between treatment arms in:<br>(i) Rate of Level 2 (<3.0 mmol/L/ <54mg/dL) hypoglycemic events<br>(ii) Rate of Level 3 hypoglycemic events (severe event with altered mental and/or physical status requiring assistance)	Week 26 to Week 52<br><br>Week 0 to Week 26
**Safety endpoint 2**<br>a) Triple therapy (dapagliflozin, semaglutide and insulin) in comparison with dual therapy (	Difference between treatment arms in:<br><br>Rate of diabetic ketoacidosis	Week 26 to Week 52

semaglutide, insulin and placebo – period 2)<br><br>b) Dual therapy (semaglutide and insulin – period 1) in comparison with standard therapy (insulin only)	(serum or urine ketones above normal limits) AND serum bicarbonate < 15 mmol/L OR blood pH < 7.3	Week 0 to Week 26

4 TRIAL DESIGN

The trial has two six-month periods:

Period 1 open-label randomised (2:1) parallel group semaglutide once weekly + insulin (standard treatment) vs insulin alone (standard treatment).
Period 2 double-blind, randomised (1:1) parallel group, dapagliflozin vs placebo for those participants initially randomised to semaglutide (with ongoing standard of care for all participants)

Thus, according to the original sample size calculation (from Protocol versions 1.0-5.0) but revised in this Protocol version 6.0 (see Section 10), during the first six months, approximately 114 participants with type 1 diabetes (50 in the UK) will be randomised (open label, see below) into three groups (approximately 38 each; i.e. 19 each in the UK) one of which will be maintained on their regular insulin therapy for 12 months (standard therapy group) and two of which will be started on semaglutide in addition to regular insulin treatment for six months (dual therapy). Following six months of stable treatment, those initially randomised to insulin and semaglutide will be additionally randomised (double-blind) to dapagliflozin (triple therapy) or matching placebo (dual therapy) for a further six months. The dose of dapagliflozin will not be increased to 10 mg in those who have confirmed ketonuria (+1) or ketonaemia (≥0.6 mM) after the first week of treatment.

In keeping with the intention-to-treat principle, participants in whom therapy is discontinued or downtitrated due to adverse effects will be encouraged to continue to undertake all study assessments and attend study visits.

5 STUDY SETTING

The study will be conducted at two sites: State University of New York (SUNY) at Buffalo, USA and Glasgow, UK. In Glasgow, n=41 participants (50% of the new total) will be identified from secondary care sites and via the Scottish Diabetes Research Network research register. Both randomisations will be stratified by site.

6 ELIGIBILITY CRITERIA

6.1 Inclusion criteria

1) Type 1 Diabetes for at least one year on stable multiple daily (four or more) injections (MDI) of insulin or continuous subcutaneous insulin infusion (CSII) for the previous three months.
2) C-peptide < 0.23 nM
3) Minimum dose of insulin: 0.5 U/kg for MDI and 0.4 U/kg for CSII

4) Regularly measuring blood glucose: four or more times daily either by fingerstick, testing or by "flash"/ continuous glucose monitoring)
5) HbA1c 7.5 – 11.0 % inclusive
6) Well versed in carbohydrate counting*
7) Age 18-70 years.
8) BMI ≥25 kg/m^2.

** In the opinion of the site Principal Investigator*

6.2 Exclusion criteria

1) Type 1 diabetes for < 12 months, type 2 diabetes, chronic pancreatitis, MODY
2) Previous use of any agent other than insulin for glycaemic control in the last three months.
3) History of diabetic ketoacidosis (DKA) requiring medical intervention (e.g. emergency room visit and/or hospitalisation) within three months prior to the screening visit.
4) Following ketogenic diet, low (<50g/day) carbohydrate diet or intermittent fasting.
5) Hypoglycaemia unawareness* or frequent episodes of severe hypoglycaemia as defined by more than one episode requiring medical assistance, emergency care (paramedics or emergency room care), or glucagon therapy administered by a third-party individual within three months prior to the screening visit
6) Symptoms of poorly controlled diabetes
7) Participation in a weight loss program with ongoing weight loss, or in an intensive exercise program
8) History of bariatric or other weight-loss surgery within 12 months prior to the screening visit
9) History of Addison's disease or chronic adrenal insufficiency
10) History of diabetes insipidus
11) Hepatic disease or cirrhosis
12) Aspartate Aminotransferase (AST) > 3 X Upper limit of normal (ULN) and/or Alanine aminotransferase (ALT) > 3 X ULN
13) Serum Total Bilirubin > 2 X ULN unless exclusively caused by Gilbert's Syndrome
14) Haemoglobin < 110 g/L (11.0 g/dL) for men; haemoglobin < 100 g/L (10.0 g/dL) for women
15) Coronary event or procedure (myocardial infarction, unstable angina, coronary artery bypass, surgery or coronary angioplasty) in the previous three months or history of congestive heart failure
16) End stage renal disease on haemodialysis and/or eGFR < 45 ml/min/1.73m^2
17) HIV or Hepatitis B/C positive status
18) Any other life-threatening, non-cardiac disease
19) History of pancreatitis or cystic fibrosis
20) Women who are pregnant, planning pregnancy or breast-feeding and women of childbearing potential (WOCBP) who are not established on a method of contraception considered highly effective (see Appendix 2) and willing to continue it for the duration of the study and for at least two months after last dose of IMP.
21) Inability to give informed consent
22) History of gastroparesis
23) History of medullary thyroid carcinoma or MEN 2 syndrome
24) History of hypersensitivity reaction to GLP-1 agonists or SGLT2 inhibitors or known hypersensitivity to any of the excipients

25) Known gallstones (history of cholecystectomy is not an exclusion criterion)
26) History of alcohol problem or drug abuse
27) Hypertriglyceridemia (>500 mg/dl)
28) Recurrent genital mycotic infection (more than one episode in last six months)
29) Hypovolaemia
30) Any malignancy except treated *in situ* malignancy and basal cell carcinoma of the skin
31) Unexplained haematuria (dipstick positive on two occasions one week apart) and/or active bladder cancer
32) Proliferative retinopathy (on basis of photographic retinal screening which must have been performed within the last 24 months)
33) Use of an investigational agent or therapeutic regimen within 90 days of study
34) Participation in any other concurrent interventional clinical trial
35) Unlikely to be able to comply with study requirements*
36) Unable to undergo meal challenge for dietary reasons (e.g. vegetarian/ vegan diet)

** In the opinion of the site Principal Investigator*

7 TRIAL PROCEDURES

Also see *Schedule of Assessments*

Each participant will be enrolled in the trial for approximately 55 weeks, comprising three weeks of monitoring to stabilise treatment and collect baseline data before randomisation into the intervention or standard of care/ placebo arms for 52 weeks. Following a completed screening visit and meeting the Inclusion and Exclusion criteria, each participant will have a total of 16 scheduled study visits. In addition, at weeks 3, 5, 8, 16, 20, 29, 35, 44 and at two weeks after the end of the study, each participant will receive a phone call to document any adverse effects or other changes. No long-term follow-up is planned following completion of participation in the study.

Participants will be treated with either insulin only (standard treatment) for the whole duration of the study or with insulin and semaglutide weekly s.c. injection (titrated where possible up to 1.0 mg, see below) for the first six month period and then randomised to either oral dapagliflozin (triple therapy) or placebo (dual therapy) daily added for the second six month period. Semaglutide will be started (Visit 3) at a dose of 0.25 mg once weekly for two weeks then increased if tolerated to 0.5 mg (Visit 5). If well tolerated at week 4, it will be increased to 1.0 mg once weekly (Visit 6) or at a subsequent visit at the discretion of the PI. Dapagliflozin (or placebo) will be started at a dose of 5 mg daily at week 26 and increased at week 27 to 10 mg (2 x 5mg capsules) daily to ensure drug tolerability and minimise the risk of insulin-induced hypoglycaemia or hypotension.

Excessive weight loss: Both semaglutide and dapagliflozin are known to cause weight loss in the range of 5-10% in type 2 diabetes. However, the magnitude of weight loss when these are combined in type 1 diabetes is uncertain. Excessive weight loss may lead to increased risk of hypoglycemia or decreased insulin requirement increasing the risk of ketosis or DKA (see Section 2.1b) and other unforeseen risks. Therefore, weight loss during any phase of the study will be limited to ≤ 15% of body weight at randomisation or reaching a BMI <22 kg/m^2. If participants are approaching this threshold, IMP will be sequentially reduced at the discretion of the PI. Semaglutide may be reduced

to 0.25mg or 0.5 mg and dapagliflozin to 5 mg. See also Section 7.8 regarding treatment interruption and withdrawal.

Covid-19 Mitigation

In view of the Covid-19 pandemic, certain trial procedures may be changed if necessary to reduce in person contact with participants. This will be continuously reviewed in view of the prevailing circumstances in line with local and national guidance and with the principle of protecting participants and study personnel. In the event of future waves of infection this would be particularly important. In this context, consideration would be given to rearranging visits, changing to virtual visits using video technology or using additional phone calls to ensure patient safety. Consideration will also be given to how minimising face-to-face interaction with participants when dispensing study medication. If required, supplies of study medication sufficient to last for longer periods may be dispensed to minimise contact and visits. Courier services may additionally be used to deliver study medications to participants.

All in person visits will be subject to local and national guidance around the use of personal protective equipment including face masks and where appropriate when conducting procedures, the use of gloves and aprons.

Gastrointestinal adverse effects:

During the initial weeks of participation, those participants who report nausea and/or vomiting during uptitration of semaglutide dose that is not sufficiently severe to require treatment discontinuation will remain on their current dose until they are symptom-free prior to discussing a further dose escalation (i.e. uptitration will be to the highest dose tolerated rather than a “forced titration”). Dose will be downtitrated to the maximum tolerated in those who experience gastrointestinal adverse effects.

Retinopathy:

As there was no excess of retinopathy with semaglutide treatment in Phase 3 trials with semaglutide, in which proliferative retinopathy was an exclusion criterion (as in the current protocol), additional retinal imaging beyond annual retinal photography (the standard of care) is not proposed (30).

Insulin dose adjustment:

Minimising hypoglycaemia: All participants will be made aware of the potential for increased hypoglycaemia with addition of adjunct therapy and supported and encouraged to monitor blood glucose at least four times daily in order to minimize the risk of hypoglycaemia. Participants will have ready access to advice from specialist medical and nursing staff at face-to-face and telephone visits. Those who have ongoing access to wearable continuous glucose monitoring [or "flash" glucose monitoring, (FGM)] will continue to use these devices. Information on management of hypoglycaemia will be included within participant diaries, reinforcing the pre-existing knowledge of participants, the majority of whom will have lived with type 1 diabetes for many years.

Reductions in insulin dose will not usually be greater than 10% (5% if HbA1c is < 7.0 %) from current levels. Daily insulin dose will not be reduced by more than 20% from the baseline level (i.e. following adjustment at the randomisation visit) and then not more than 20% from the new baseline levels following adjustment at 26 weeks. Participants requiring an insulin dose reduction > 20% and their total insulin dose < 0.4 U/kg (current weight) will be monitored closely for insulin dose, carbohydrate intake, ketones and blood glucose. Following the second randomisation at 26 weeks, dapagliflozin/ placebo will be temporarily discontinued if insulin dose falls below this threshold. The participant will

be withdrawn from the study if this remains so at review in one week [see also Withdrawal Criterion 9 and Section 8.12

Minimising ketosis: Participants will be instructed to stay hydrated and not to reduce their caloric intake (especially carbohydrates) or start new dietary approaches (especially ketogenic diets) during the study. They will be educated to recognize the signs and symptoms of diabetic ketoacidosis (DKA) and to undertake additional self-testing of urine and/or blood ketones if they develop nausea, vomiting, or anorexia. In addition, they will be given education and a written ketosis management plan (STICH Protocol – i.e. STop SGLT-2 inhibitor or placebo (temporarily), give Insulin bolus, take additional Carbohydrate, and additional Hydration - see Appendix 3) (37).

From Visit 10 (26 weeks), those participants undergoing the second randomisation will be supported and encouraged to monitor urine or blood ketones at home at least once daily: three days before visit 10 (week 26), they will be asked to start daily measurements of urine ketones [CombiScreen® 10 SYS Plus urine testing strips, Sterilab, UK (or equivalent)] or blood ketones [CareSens™ Dual, i-SENS, Koreal; GlucoMen® Areo 2K, Menarini, UK (or equivalent]]. They will be asked to record these in the diary/ log provided. In addition, ketones (blood and urine) will be measured at every visit. If ketone bodies in urine are +1 or greater (≥0.6 mM in blood), participants will be reminded to keep well hydrated and notify the study team as soon as possible (office hours) as diabetic ketoacidosis (DKA) can occur even at glucose levels < 14 mmol/L (38).

If ketones are +2 or more in urine (> 1.5 mmol/L) participants will be advised to initiate the STICH Protocol immediately and contact the study team in office hours (or telephone NHS 24 or attend their Accident & Emergency if out of hours) (37). This information will be reinforced verbally at study visits and also in the form of a Patient Alert Card that participants will be asked to carry. This will also contain details of the investigational study drug, the study number, the investigator's name, and a contact number (for in hours contact). The study team will assess for symptoms or test results indicating a risk of ketoacidosis immediately on reporting and if blood ketone levels are persistently (one week) at these levels dapagliflozin/placebo will be permanently discontinued.

If blood ketone readings are confirmed as > 3.0 mmol/L (or urine ketones reading +++) participants will be advised to stop dapagliflozin/ placebo and attend the Accident and Emergency department without delay.

Participants will be advised that dapagliflozin/placebo may be temporarily stopped in other clinical situations known to predispose to ketoacidosis (e.g. acute serious medical illnesses or hospitalisation for major surgical procedures). Any requirement for intravenous insulin infusion (whether in Accident and Emergency or as an in-patient admission) will be recorded as a Serious Adverse Event. For further detailed procedures see section 9.31.

Volume depletion:
There is a theoretical risk of volume depletion and low blood pressure when dapagliflozin is used in combination with other antihypertensive drugs. All pre-existing concomitant medication will be reviewed at the screening visit as part of the medical history and examination (see Section 7.1.3 for further details). Participants undergoing the second randomisation will be educated on symptoms suggestive of low blood pressure (e.g. light-headedness, dizziness, feeling faint) and asked to contact the study team if they experience these symptoms. In clinical practice however SGLT-2 inhibitors are commonly used with anti-hypertensive drugs with no significant problems (31).

Genital mycotic infections:

Individuals who report symptoms suggestive of genital mycotic infection will be advised to self-treat with topical antifungal preparations. If a second episode occurs, a decision on treatment discontinuation will be considered in discussion between the participant and the site Principal Investigator, taking account of the severity of the symptoms.

Foot care:
Annual foot checks will be conducted for all participants as per standard of care.

7.1 Recruitment

7.1.1 Patient identification

All personal details will be managed according to ISO 27001:2013 compliant Standard Operating Procedures.

Potentially eligible participants will be identified from the Scottish Diabetes Research Network Research Register (a managed and searchable database of people with diabetes who have agreed to be contacted about clinical trial participation) and at secondary care diabetes clinics in Greater Glasgow and Clyde. The principal site for recruitment will be Stobhill Ambulatory Care Hospital but other sites may be opened if necessary as Patient Identification Centre sites. The majority of study visits (and all visits involving drug dispensing) will be conducted at the Clinical Research Facility, Glasgow Royal Infirmary. Information posters with the contact details of the research team will be displayed in recruiting clinics.

At the principal site, clinic lists (whether remote or face-to-face) will be pre-reviewed by members of the research team. An email will be sent to the clinic Consultant physician to flag potentially eligible individuals on their list; potential eligibility will also be flagged on the clinic electronic list (on Trakcare®) to prompt reviewing physicians (as members of the usual care team) to ask if the potential participant would be interested in receiving more information from the research team.

In the above email (to which a response is not required), physicians will be asked to record the outcome of their discussion with the potential participant; either: (i) by adding a note to the Clinical Portal system; (ii) adding an appropriate statement to their clinic letter; or (iii) by return email using standard text (e.g. willing to be contacted/ not willing to be contacted/ unsuitable for the trial). The permitted and preferred method(s) of contact (e.g. mobile telephone/ home telephone/ letter/ email should also be recorded. Telephone contact will be the preferred contact method with other methods offered where this is declined. If no preferred method is indicated by consultant it will be presumed that telephone contact is acceptable Physician consultations with potentially eligible individuals that are concluded with no invitation to research participation (e.g. due to pressure of time) can be followed up with a phone call from one of the study physicians who is also a member of the research team.

Following clinical consultations, members of the research team will identify potential participants who appear to be meet eligibility criteria and have given permission to be contacted.. These individuals will be called and/or sent/emailed (as appropriate according to documented permissions) a Patient Information Sheet. An invite letter and Patient Information Sheet will be sent to potential participants who have declined telephone contact but agreed to mail or email contact. Where permission has been given for phone contact, this can be followed up by up to two further phone contacts (provided permission to contact is not withdrawn by the potential participant in an earlier call). A visit to the Clinical Research Facility will then be scheduled for informed consent and screening. The person scheduling the CRF visit will confirm with the patient if they have a copy of the full Patient Information

Sheet or if they need a further copy to be sent out in advance of the visit. This will be sent out to arrive at least 48 hours prior to the planned CRF visit or emailed to potential participant.

At the screening visit, eligibility criteria will be checked and the study procedures explained. All potential participants will have had at least 24 hours to review the full Patient Information Sheet. They will then be invited to provide written, informed consent.

At other sites within NHS Greater Glasgow and Clyde the same approach will be taken for the clinic lists of those physicians who indicate willingness to support trial recruitment. The initial approach will always be from a member of the usual care team.

Covid-19 Mitigation: The delivery of outpatient clinics has been substantially changed due to the pandemic. The use of telephone clinics is now standard for many visits and video technologies are also being used. These mediums will therefore be used for inviting participants to take part when in person appointments are not taking place. This may be subject to change depending on the situation with Covid-19. Other principles of recruitment will be as described above.

7.1.2 Screening

A separate non-fasting visit will be arranged at which potential participants will have further time to discuss with the study nurse and doctor.

Prospective participants will be asked to read and understand the Participant Information Sheet. Any questions regarding the Protocol will be answered. Risks and side-effects of the active trial medication will be explained. Eligibility for trial enrolment will be confirmed by a medical practitioner.

Individual wishing to participate in the study will then be invited to sign to document their informed consent (see Section 7.2 below).

7.1.3 Screening assessments

Items from the medical history relevant to the Inclusion and Exclusion criteria will be recorded. Demographic data, medical history and current medications (including current insulin doses) will be documented. All participants will have access to a dual blood glucose and ketone meter as per the contemporary standard of care in type 1 diabetes. A physical examination will be conducted. Clinical observations will be recorded and blood and urine samples obtained (see below):

<u>Demographics</u>

- Date of birth
- Sex
- Ethnicity
- Date of diagnosis
- Ethnic group: white/black/Asian/other
- Height

<u>Clinical assessments</u>

- Systolic and diastolic blood pressure (after 5 minutes rest)
- Heart rate
- Body weight (clothed without coat and shoes)
- Capillary blood glucose

<u>Medical History</u>

- Smoking status: current/ex/never
- Alcohol consumption: units/week
- Hypertension: Y/N
- Microalbuminuria: Y/N
- Proteinuria: Y/N
- Neuropathy: Y/N
- Previous major cardiovascular event(s): Y/N (date)
- Existing CV disease of any type*: Y/N
- Amputation above forefoot: Y/N
- Hospitalisation for DKA in last year
- Level of educational attainment: University (college) educated Y/N

* Existing CV Disease is defined as any of: MI, stroke, ACS/unstable angina, coronary stent/angioplasty, CABG, TIA, heart failure, peripheral vascular disease

Insulin treatment
- Regimen: MDI, pump,
- Total dose (all); Basal (longer acting) dose/ Bolus (shorter acting) dose (for MDI only)

Other treatment (drug classes and names)

Current use Y/N. If no, has there been use in last three months?: Y/N

- ACE inhibitors
- Angiotensin receptor blocker
- Calcium channel blocker
- Alpha-blocker
- Beta-blocker
- Loop diuretic
- Thiazide like diuretic
- Statin
- Aspirin
- Clopidogrel
- Novel anticoagulant
- Warfarin

Blood assessments:
- Blood ketones
- HbA1c
- FBC
- Urea and electrolytes
- Creatinine
- Liver function tests (AST/ ALT/ Alkaline Phosphatase)
- Bone profile (Calcium/ Phosphate)
- C-peptide (capillary blood glucose must be > 4.0 mmol/L at time of sampling)

  N.B. where re-screening is undertaken (see 7.2.1 below), C-peptide does not require to be repeated

If blood tests have been assessed for clinical purposes within the last three weeks, these values will be used.

Urine sample:

- Urine ketones
- For all WOCBP, a urine pregnancy test will be checked at screening

Potential participants meeting all the inclusion and exclusion criteria based on history, examination and screening tests will be enrolled in the study.

Pregnancy: WOCBP will be asked about pregnancy status and contraceptive usage (see Appendix 2) and a pregnancy test will be conducted (following informed consent) prior to randomisation. We will not recruit those who are pregnant, wanting to become pregnant, or using birth control methods not considered highly effective. At specified study visits following randomisation, WOCBP randomised to IMP (as opposed to standard of care) will be asked about their contraception status, and a urine pregnancy test carried out. Study medication will be permanently discontinued in women who become pregnant or considered to be on contraception that is not highly effective. As the study assessments *per se* do not carry an additional risk over standard of care to either mother or unborn child, further pregnancy tests will not be routinely offered following randomisation to those women allocated to “Insulin therapy (standard of care)” (i.e. who are not exposed to IMP). All pregnancies will be notified to the Sponsor Pharmacovigilance Officer using the standard pregnancy notification form and the pregnancy followed to outcome).

7.1.4 Payment

Participants will be offered reasonable travel expenses for visits additional to usual care.

7.2 Consent

Potential participants will be identified and screened by the Inclusion and Exclusion criteria listed above. Medical staff or appropriately trained support staff will seek consent for screening and participation in the trial from individuals that fulfil the criteria. All potential participants will have at least 24 hours to review the Patient Information Sheet before being approached (see Section 7.1.2 above). Following written consent, each signature will be dated by the signatory, the original retained in the site file, a copy provided to the participant and a copy scanned into the participant's Clinical Portal record.

Data collected for routine clinical care will be used for clinical trial documentation (e.g. blood results within the previous three weeks). In the absence of routine blood results, sampling of blood for study specific laboratory measurements can only be performed following documentation of informed consent.

7.2.1 Re-screening post-consent

Where inclusion (e.g. HbA1c ≤7.5%) or exclusion (e.g. DKA within the last three months) criteria are not met (e.g. screening visit blood results falling outside the study parameters), potential participants can be re-screened with additional urine and blood tests on one further occasion.

Where the screening period (i.e. between the Screening visit and Visit 1) exceeds 10 weeks, screening visit urine and blood tests should be repeated at an additional visit that can be arranged with verbal consent of the participant prior to Visit 1. Data will be amended on the web portal as appropriate.

If the initial consent was signed within three months of the re-testing, new consent is not required unless the consent form template has been updated in this period. If the initial consent was not signed within three months of re-testing then consent should be sought again.

Rescreened participants will keep the same Patient ID and data will be amended on the web portal as appropriate.

Where screening bloods are repeated in cases as above, C-peptide testing does not require to be repeated as values consistent with the inclusion criteria remain valid long term.

7.3 Run-in period

7.3.1 Visit 1 (@3 weeks prior to randomisation)

Participants who meet entry criteria will be scheduled for their next visit at approximately three weeks prior to randomisation. Daily fingerstick blood glucose levels (study diaries), CGM/ FGM data or insulin pump download data (as applicable) will be collected and reviewed and appropriate insulin dose adjustments made by the medical team to target fasting blood glucose concentrations between 5 -10.0 mmol/L if possible at all times without having additional hypoglycaemia (< 3.9 mmol/L). Clinical observations will be recorded including resting BP measurement. Blood glucose and ketone bodies will be measured via a fingerstick blood sample (see Section 7). Urine for pregnancy status and ketone bodies will be assessed.

Participants will be instructed on the use of the Continuous Glucose Monitoring System (Dexcom G6 with blinded receiver). All subjects will be advised to monitor their capillary blood glucose by CGM or fingerstick at least 4 times a day (before meals and at bedtime) throughout the study and to wear their CGM constantly for next 20 days (changing the sensor at 10 days). CHO counting technique will be reviewed and appropriate support given with calculating bolus insulin doses.

Visit procedures:

Monitoring and treatment optimisation

- Check for adverse events
- Review blood glucose diary
- Blood ketones
- Adjust insulin doses (if necessary)

Clinical assessments

- Blood pressure
- Heart rate
- Weight

Urine sample:

- Check urine ketones

Other

- Fit and start masked Continuous Glucose Monitoring with Dexcom G6

7.3.2 Visit 2 (1 @ weeks prior to randomisation)

Fasting visit* - Participants will be asked to fast (no food or calorie intake) for 10 hours prior to the visit overnight. Those who plan to drive to fasting visits will be reminded of the increased risk of hypoglycaemia and DVLA guidelines. This will be repeated prior to each fasting visit and reiterated within the patient diaries. This visit will be scheduled for a morning appointment and participants will be advised not to take their breakfast bolus insulin dose prior to arrival. If participants arrive at the study visit in a non-fasted state, the visit will be terminated and a further fasting visit rearranged as soon as possible.

Daily fingerstick blood glucose levels (diaries), CGM/ FGM data or insulin pump download data (as applicable) will be collected and reviewed and appropriate insulin dose adjustments made. Clinical observations will be recorded as detailed below. Blood glucose and ketone bodies will be measured via a fingerstick blood sample (see Section 7). If blood glucose is less than 3.9 mmol/L, or more than 13.9 mmol/L, or if blood (≥ 0.6 mM), or urine ketones (+1 or greater) are positive, the visit will be rescheduled for an alternative date. Otherwise, an intravenous cannula will be placed in order to obtain blood samples for the duration of the visit. A Meal Challenge Test will be performed (described below). A 24 hr urine container will be dispensed to collect a 24 hour urine sample to bring in at the Week 0 visit. For this collection and all subsequent 24 hour urine collections, urine will be tested via dipstick testing and an aliquot retained for assay of albumin, creatinine, ketones, and 15-isoprostane.

Visit Procedures

Monitoring and treatment optimisation

- Check for adverse events
- Review blood glucose diary
- Blood ketones
- Adjust insulin doses (if necessary)

Clinical assessments

- Blood pressure
- Heart rate
- Weight

Urine sample:

- Check urine ketones
- 24 hour urine container dispensed

Other assessments

- Download Dexcom G6 CGM
- High fat high calorie Meal Challenge: in order to assess postprandial metabolism a Meal Challenge Test will be performed using an approximately 900 kilocalorie High Fat High Carbohydrate (HFHC) meal including an egg muffin sandwich, a sausage muffin sandwich and two hash browns [containing approximately 88g carbohydrate, 51 g fat (33% saturated) and 34 g protein]. Participants will be asked to attend fasting prior to breakfast insulin bolus (with continuing basal insulin); participants will be asked not to take bolus insulin until the 3 hour time point unless blood glucose values reach above 22.2 mmol/L. Blood samples will be collected from an indwelling intravenous canula in a superficial forearm vein for glucose, glucagon, FFA, GLP-1 (AUC). Sequential blood samples will be obtained at 0, 15, 30, 45, 60, 90, 120, 150 and 180 min after consuming the meal. Volume of samples at 15, 30, 45, 90 and 150 min will be 5ml while those at 0, 60, 120 and 180min will be 30 ml (total volume = 145 ml).

A spot urine sample will be collected at baseline, 1 and 3hr for standard urinalysis and an aliquot retained for assay of ketones and 15-isoprostane.

7.4 Randomisation

Participants who are being randomised will be required to have undergone all screening assessments.

At the randomisation visit, study participants will be provided with a Patient Alert Card. There are two Versions: (1) for the intervention arm i.e. those allocated at first randomisation to open-label semaglutide who are also eligible for the second double-blind randomisation (dapagliflozin or placebo); and (2) for those allocated to standard of care. These cards supplement the study diaries and include contact information for the study team. Version 1 also contains unblinding information. Participants will be asked to carry their card at all times. These cards will be collected at the end of participation in the study.

7.4.3 First Randomisation Scheme

For the first randomisation (Period 1, open label), eligible and consenting participants will be randomised (2:1, stratified by site) to the two groups (open-label semaglutide or standard of care) using permuted blocks of variable size to minimize predictability.

7.4.4 Method of implementing the allocation sequence

Randomisation will be achieved by accessing an interactive web-based randomisation system (IWRS). The investigator will provide the participant identifier and the system will check the participant's eligibility from information already entered in the electronic Case Report Form (eCRF): if appropriate the randomisation group will be allocated.

7.5 Visit 3 (First Randomisation Visit)

Participants will be asked to attend in the fasting state (no food or calorie intake for 10 hours; see 7.3.2 above). Blood glucose levels and ketone bodies will be measured via a fingerstick blood sample using a glucose and ketone meter (see Section 7) and daily fingerstick blood glucose levels (diaries) collected and reviewed. The participant's own CGM/ FGM blood glucose data or insulin pump download data will also be collected and reviewed. Daily insulin dose will be adjusted following clinical review of study diaries: if reduced, this should not be by more than 20% from the baseline level Participants will discontinue use of the study masked CGM (Dexcom G6). Clinical observations will be recorded. Blood glucose and ketone bodies will be measured via a fingerstick blood sample (see Section 7). The 24 hour urine container will be collected. A fasting blood sample will be taken (45 mL) to evaluate study endpoints, FBC, U&E, LFT, bone profile, HbA1c, and fructosamine (detailed below). A urine sample will be collected to evaluate ketone bodies and, in females, pregnancy status.

Participants will be asked to complete Quality of Life questionnaires (see Appendices).

IMP will be dispensed for those randomised to open-label semaglutide (once weekly). Semaglutide will be started on site at the 0.25 mg dose. Where both research staff and participant are in agreement, the first dose can be self-administered with staff observing; otherwise research staff will administer the first dose. Participants will be instructed to call site staff directly in case of any problem or untoward side effects. An office hours phone number will be provided for advice. Outside of these hours, provision of emergency medical care if required will be via NHS 24 and/or Accident & Emergency. Participants will be specifically asked to call to inform research staff if they have hypoglycaemia (blood glucose < 3.9 mmol/L) or Level 2 hyperglycaemia (blood glucose >13.9 mmol/L), particularly if occurring on more than one occasion. This will apply for the remainder of the trial.

Visit Procedures

Fasting visit* as per visit 2.

Monitoring and treatment optimisation

- Check for adverse events
- Check adequacy of contraception (WOCBP)
- Review blood glucose diary
- Adjust insulin doses (if necessary)
- Blood ketones

Clinical assessments
Blood pressure

- Heart rate
- Body weight

Quality of life assessments

- Diabetes Specific Quality of Life Scale (DSQOLS)
- Problem Areas In Diabetes (PAID) questionnaire

Blood assessments

- Na, K, urea, creatinine, eGFR (MDRD)
- AST/ ALT/ Alkaline Phosphatase
- Calcium/ Phosphate
- FBC
- HbA1c
- Fructosamine
- Bilirubin
- Random glucose

Urine

- For WOCBP, a urine pregnancy test will be checked prior to first administration of IMP.
- Urine ketones
- 24 hour urine sample collected

Medication

- Administer open label semaglutide 0.25 mg if randomised to active treatment
- Issue Patient Alert Card (appropriate version according to randomisation)

Other:

- Read masked Continuous Glucose Monitoring with Dexcom G6

Bloods for storage – see Sample Handling Manual
Approximately 45 mls of venous blood will be withdrawn and collected in tubes containing EDTA. Blood will be centrifuged at 1500g for 20 mins at 4°C. Plasma will be siphoned, aliquoted and stored locally at -80° ± 10°C until transport in batches to the central laboratory on dry ice.

7.5.1 Visit 4 (@1 week after first randomisation)

Clinical observations will be recorded. Blood glucose levels and ketone bodies will be measured via a fingerstick blood sample using the participant's own glucose and ketone meter (see Section 7)) and daily blood glucose levels collected and reviewed. Insulin doses will be adjusted as in visit 3. If HbA1c is <7.0 %, insulin dose reduction should not be >5% from baseline total dose. As for Visit 3, semaglutide 0.25mg will be injected on site for those randomised to active therapy; however, if the participant is unable to attend within 48 hours of the scheduled appointment, this dose can be self-administered at home (confirmed by a documented phone call) provided there were no adverse effects following the previous dose.

Visit Procedures

Monitoring and treatment optimisation

- Check for adverse events
- Review blood glucose diary
- Adjust insulin doses (if necessary)
- Blood ketones

Assessments

- Blood pressure
- Heart rate
- Weight

Urine sample:

- Urine ketones

Medication

- Support participant's self-injection of open label semaglutide 0.25 mg if randomised to active treatment (dose on-site if dose is scheduled for visit day (see above)

7.5.2 Visit 5 (@2 weeks after first randomisation)

Clinical observations will be recorded. Blood glucose levels and ketone bodies will be measured via a fingerstick blood sample using glucose and ketones meter (see Section 7) and daily blood glucose levels collected and reviewed. Daily blood glucose data collected and reviewed and insulin doses adjusted as per Visit 3. Provided uptitration is considered appropriate by the site PI (or designee) (see also "Gastrointestinal adverse effects," page 31), the semaglutide dose will be increased to 0.5 mg weekly. This will be administered on-site if dose is scheduled for a visit day but can be self-administered at home (confirmed by a documented phone call) provided both research staff and participant are in agreement and there were no adverse effects following the previous dose. All subsequent trial doses of semaglutide can be self-administered on-site or at home as convenient and appropriate. Supply of IMP will be dispensed as appropriate to last until the next visit.

Visit Procedures

Monitoring and treatment optimisation

- Check for adverse events
- Review blood glucose diary
- Adjust insulin doses (if necessary)
- Blood ketones

Assessments

- Blood pressure
- Heart rate
- Weight

Urine sample:

- Urine ketones

Medication

- Dose titration (see above) if randomised to open-label semaglutide
- Return used study pen
- Dispense medication if randomised to open-label semaglutide (dose on-site if dose is scheduled for visit day– see above)

7.5.3 Visit 6 (@4 weeks after first randomisation)

For those randomised to open-label semaglutide, doses administered will be recorded (self-report). Clinical observations will be recorded. Daily blood glucose data will be collected and reviewed and insulin doses adjusted as per visit 3. Supply of IMP will be given to last until the next visit. A urine sample will be collected to evaluate ketone bodies and, in females randomised to IMP, pregnancy status.

Provided uptitration is considered appropriate by the site PI (or designee) (see also "Gastrointestinal adverse effects," page 31), the recommended semaglutide dose will be increased to 1.0 mg weekly (or 0.5 mg if not previously uptitrated from 0.25 mg). A 24 hr urine container will be dispensed to collect a 24 hour urine sample to bring in at the Week 13 visit.

Visit Procedures

Monitoring and treatment optimisation

- Check for adverse events
- Check adequacy of contraception (WOCBP)
- Review blood glucose diary
- Adjust insulin doses (if necessary)
- Blood ketones

Assessments

- Blood pressure
- Heart rate
- Weight

Urine sample:

- Urine ketones
- Urine pregnancy test (WOCBP randomised to IMP only)
- 24 hour urine container dispensed

Medication

- Record doses of semaglutide self-injected since last visit (self-report)
- Dose titration if randomised to open-label semaglutide (see above)

- Return used study pens
- Dispense medication if randomised to open-label semaglutide

7.5.4 Visit 7 (@13 weeks after first randomisation)

Participants will be asked to attend in the fasting state (no food or calorie intake for 10 hours). If participant is not fasting, samples should be taken and non-fasting status noted. For those randomised to open-label semaglutide, doses administered will be recorded (self-report). For those who have remained on doses lower than 1.0 mg weekly, this visit will be the last opportunity to uptitrate. Provided uptitration is considered appropriate by the site PI (or designee) (see also "Gastrointestinal adverse effects," page 31) the semaglutide dose will be increased from 0.25 mg to 0.5 mg weekly or from 0.5 mg to 1.0 mg weekly. Daily fingerstick blood glucose levels (diaries), CGM/ FGM data or insulin pump download data will be collected and reviewed and appropriate insulin dose adjustments made. Supply of IMP will be given to last until visit 10. Observations will be recorded. A urine sample will be collected to evaluate ketone bodies and, in females randomised to IMP, pregnancy status. The 24 hour urine container will be collected. A fasting blood sample will be taken (45 mL) to evaluate study endpoints.

Visit Procedures

Fasting visit*

Monitoring and treatment optimisation
- Check adequacy of contraception (WOCBP)
- Check for adverse events
- Review blood glucose diary
- Adjust insulin doses (if necessary)
- Blood ketones

Clinical assessments
- Blood pressure
- Heart rate
- Weight

Blood assessments
- Na, K, urea, creatinine, eGFR (MDRD)
- AST/ ALT/ Alkaline Phosphatase
- Calcium/ Phosphate
- FBC
- HbA1c
- Fructosamine
- Bilirubin
- Random glucose

Urine
- Pregnancy test (WOCBP randomised to IMP only)
- Urine ketones
- 24 hour urine bottle collected

Medication

- Record doses of semaglutide self-injected since last visit (self-report)
- Dose titration if randomised to open-label semaglutide (see above)
- **N.B. no further uptitration after this visit**
- Return used study pens
- Dispense medication if randomised to open-label semaglutide

7.5.5 Visit 8 (@23 weeks after first randomisation)

For those randomised to open-label semaglutide, doses administered will be recorded (self-report). Clinical observations will be recorded. Daily blood glucose data (fingerstick or FGM) will be collected and reviewed as per visit 3. Blood glucose levels and ketone bodies will be measured via a fingerstick blood sample. A urine sample will be collected to evaluate ketone bodies and, in females randomised to IMP, pregnancy status. Insulin doses will be adjusted if needed. All subjects will be reminded to monitor their capillary blood glucose by fingerstick at least 4 times a day (before meals and at bedtime) throughout the study. CGM sensor will be fitted and participants asked to wear their CGM constantly for next 20 days.

Visit Procedures

Monitoring and treatment optimisation

- Check adequacy of contraception (WOCBP)
- Check for adverse events
- Review blood glucose diary
- Blood ketones
- Adjust insulin doses (if necessary)

Clinical assessments

- Blood pressure
- Heart rate
- Weight

Urine sample:

- Pregnancy test (WOCBP randomised to IMP only)
- Urine ketones

Other assessments

- Fit and start masked Continuous Glucose Monitoring with Dexcom G6

7.5.6 Visit 9 (@25 weeks after first randomisation)

Participants will be asked to attend in the fasting state (no food or calorie intake for 10 hours). If participants arrive at the study visit in a non-fasted state, the visit will be terminated and a further fasting visit rearranged as soon as possible. Daily fingerstick blood glucose levels (diaries), CGM/ FGM data or insulin pump download data will be collected and reviewed and appropriate insulin dose adjustments made. Participants will discontinue use of the study CGM (Dexcom G6). Clinical observations will be recorded. Blood glucose levels and ketone bodies will be measured via a fingerstick blood sample (see Section 7). Supply of IMP will be given to last until the next visit. A 24 hr urine container will be dispensed to collect a 24 hour urine sample to bring in at visit 10 (Week 26) visit. A Meal Challenge Test will be performed as per visit 2. Participants will be provided with strips for urine or blood ketone measurement at home and instructed or reminded as appropriate on how to use these. They will be asked to measure urinary and blood ketones every morning for three consecutive

days prior to the next visit (second randomisation: dapagliflozin or placebo) and to record the results in their diary.

<u>Visit Procedures</u>

Fasting visit*

<u>Monitoring and treatment optimisation</u>
- Check adequacy of contraception (WOCBP)
- Check for adverse events
- Review blood glucose diary
- Adjust insulin doses (if necessary)
- Blood ketones

<u>Clinical assessments</u>
- Blood pressure
- Heart rate
- Weight

<u>Urine sample</u>:
- Urine ketones
- 24 hour urine container dispensed

<u>Other assessments</u>
- Download Dexcom G6
- Meal Challenge Test

<u>Blood assessments</u>
- HbA1c

7.6 Second Randomisation Scheme

For the second randomisation (Period 2, double-blind), eligible and consenting participants previously randomized to open-label semaglutide will be randomised (1:1) to two groups (dapagliflozin or matching placebo) using permuted blocks of variable size to minimize predictability with stratification for HbA1c <u>(≤ 7.5% vs >7.5% HbA1c)</u> Those participants previously randomised to standard of care will continue as before.

7.6.1 Blinding (period 2 only)

For the double-blind treatment period, dapagliflozin and placebo will be packed in matching packs to maintain the blind. Each pack will contain sufficient supplies for 30 days' treatment with a small overage (excess). Packs will be labelled with a unique number that will be used to assign treatment to the participant via the IWRS system whilst maintaining the blind. Packs will be labelled in accordance with Good Manufacturing Practice and local regulatory requirements.

Unblinding. Treatment discontinuation (of dapagliflozin or placebo), rather than unblinding, will be carried out as far as possible. Participants will be advised in any case of hospitalisation with acute illness to

discontinue study medication and inform the relevant clinician. These instructions will be reinforced in the Study Diary. However, where knowledge of treatment may assist emergency treatment, the investigator (or designee) may unblind using the IWRS system. Information on emergency unblinding (the investigational study drug, the study number, the investigator's name and a contact number) will be provided on the Patient Alert Card. For each unblinding an email alert is generated to the Study Coordinator and Chief Investigator. The most likely scenarios for unblinding will be: confirmed pregnancy, overdose/ accidental ingestion, or development of acute renal failure

7.6.2 Visit 10 (Second Randomisation Visit; @26 weeks after First Randomisation)

Participants will be asked to attend in the fasting state (no food or calorie intake for 10 hours). For those who arrive in a non-fasted state, the visit will be terminated and a further fasting visit rearranged as soon as possible. Participants with normal fasting urine (<+) or blood ketone (<0.6 mmol) in the three days prior to randomisation will proceed to the next treatment phase as per this visit. The principles of the STICH Protocol (see Appendix 3) will be discussed (37). Participants with higher ketone values in at least one of the previous three days will have their glucose levels, insulin dose, and carbohydrate intake reviewed and advised on hydration, adequate CHO intake, and adequate insulin dosing. They will be rescheduled for one week and asked to repeat three days testing before the next visit.

Clinical observations will be recorded. A urine sample will be collected for ketones. Blood glucose or ketones will be measured via a fingerstick blood sample (see Section 7.2.1). Daily fingerstick blood glucose levels (diaries), CGM/ FGM data or insulin pump download data will be collected and reviewed and appropriate insulin dose adjustments made (following the guidance in Section 7, page 35). The previous three days blood or urine ketones levels will be reviewed. The adjusted total insulin dose at this visit will serve as the baseline total insulin dose for the remaining six months of the study and future limits of dose adjustments will be based on this new baseline. The blinded study CGM will be collected. The 24 hour urine container will be collected. A fasting blood sample will be taken (45 mL) to evaluate study endpoints. Participants will be asked to complete quality of life questionnaires.

At this point, those participants previously randomised to receive open-label semaglutide will be randomised to receive dapagliflozin 5 mg daily or matching placebo. They will be educated on symptoms suggestive of low blood pressure (e.g. light-headedness, dizziness, feeling faint).

Participants will be asked to perform a daily (preferably early morning) urine (using CombiScreen® 10 SYS Plus urine testing strips or equivalent) or blood tests for ketone bodies (using the particpant's own meters as per Section 7, "Minimising ketosis": page 28) and record all outcomes in dedicated study diaries. If results of these scheduled (i.e. not done due to symptoms) ketone measurements, are +1 or higher (urine) or > 0.6 mmol/L (blood), participants will be instructed to follow the STICH Protocol as per their study diary and Appendix 3, . For any abnormal ketone levels taken due to symptoms, the DKA consensus guidelines are to be followed instead (see Section 9.3).

Participants will be instructed to call site staff directly in case of any problem or untoward side effects. They will be specifically asked to call if they have confirmed hypoglycaemia (blood glucose levels < 3.9 mmol/L) or hyperglycaemia (blood glucose > 13.9 mmol/L) occurring on more than one occasion. Participants will be advised to remain well hydrated and report any genital mycotic or urinary tract infections

Visit Procedures

Fasting visit* - Requirements and arrangements as per visits 2 and 3.

Monitoring and treatment optimisation

- Check adequacy of contraception (WOCBP)
- Check for adverse events
- Review blood glucose diary
- Adjust insulin doses (if necessary) - this insulin dose is baseline for Period 2
- Blood ketones

Clinical assessments
- Blood pressure
- Heart rate
- Body weight

Quality of life assessments
- Diabetes Specific Quality of Life Scale (DSQOLS)
- Problem Areas In Diabetes (PAID) questionnaire

Blood assessments
- Na, K, urea, creatinine, eGFR (MDRD)
- AST/ ALT/ Alkaline Phosphatase
- Calcium/ Phosphate
- FBC
- Fructosamine
- Bilirubin
- Random glucose
- C-peptide (capillary blood glucose must be > 4.0 mmol/L at time of sampling)
-

Urine
- Pregnancy test (WOCBP randomised to IMP only)
- Urine ketones
- 24 hour urine bottle collected

Medication
- Education on symptoms suggestive of low blood pressure (e.g. light-headedness, dizziness, feeling faint) for those undergoing second randomisation
- Record doses of semaglutide self-injected since Visit 7 (self-report)
- Return used study pens
- Dispense open label semaglutide for participants randomised to active treatment at Period 1 (if required). Dispense dapagliflozin or placebo if randomised to active treatment

Other assessments
- Read masked Continuous Glucose Monitoring sensor (mean fasting blood glucose, mean blood glucose, percentage time in range, glycaemic variability)
-

7.6.3 Visit 11 (@1 week after Second Randomisation)

For those randomised to open-label semaglutide, doses administered will be recorded (self-report). Clinical observations will be recorded. Daily blood glucose and blood/ urine ketone levels will be reviewed and glucose levels collected. A urine sample will be collected to evaluate for ketones bodies. Blood glucose levels and ketones bodies will be reviewed measured via a fingerstick blood sample (see Section 7.2.1). . Daily fingerstick blood glucose levels (diaries), CGM/ FGM data or insulin pump download data will be reviewed and appropriate insulin dose adjustments made using the new baseline from Visit 10 (following the guidance in Section 7, page 35). If at any time total insulin dose reduction is >20% and total insulin dose is <0.40 U/Kg, additional monitoring (see Appendix 2) will be implemented. Unless blood (>0.6 mmol/L) or urine ketones (+ or more) have been elevated within the previous three days, dapagliflozin or placebo dose will be uptitrated to 10 mg daily (two capsules of either active or placebo medication) for the remainder of the study. Supply of IMPs will be given to last until week 39.

Visit Procedures

Monitoring and treatment optimisation

- Check adequacy of contraception.
- Check for adverse events
- Review blood glucose diary
- Adjust insulin doses (if necessary)
- Blood ketones
- Daily blood or urine ketones review

Clinical assessments

- Blood pressure
- Heart rate
- Body weight

Urine sample:

- Urine ketones

Medication

- Count returned dapagliflozin or placebo capsules
- Increase dose of dapagliflozin or placebo to 10mg (see above – review ketones)
- Dispense dapagliflozin or placebo (record dose)

7.6.4 Visit 12 (@5 weeks after Second Randomisation)

For those randomised to open-label semaglutide, doses administered will be recorded (self-report). Clinical observations will be recorded. Daily blood glucose and blood/ urine ketone levels will be reviewed and glucose levels collected. Blood glucose levels and ketone bodies will be measured via a fingerstick blood sample. A urine sample will be collected to evaluate ketone bodies and if appropriate pregnancy status. Insulin dose will be adjusted if as per Visit 11. A blood sample will be taken (15 mL) to evaluate study endpoints. A 24 hr urine container will be dispensed to collect a 24 hour urine sample to bring in at the Week 39 visit

Visit Procedures

Monitoring and treatment optimisation

- Check adequacy of contraception (WOCBP)

- Check for adverse events
- Review blood glucose diary
- Adjust insulin doses (if necessary)
- Blood ketones

Clinical assessments
- Blood pressure
- Heart rate
- Weight

Blood assessments
- Na, K, urea, creatinine, eGFR (MDRD)
- AST/ ALT/ Alkaline Phosphatase
- Calcium/ Phosphate
- FBC
- Bilirubin
- Random glucose

Urine
- Pregnancy test (WOCBP randomised to IMP only)
- Urine ketones
- 24 hour urine container dispensed

7.6.5 Visit 13 (@13 weeks after Second Randomisation)

For those randomised to open-label semaglutide, doses administered will be recorded (self-report). Participants will be asked to attend in the fasting state. If participant is not fasting, samples should be taken and non-fasting status noted. Clinical observations will be recorded. Daily blood glucose and blood/ urine ketone levels will be reviewed and glucose levels collected. Blood glucose levels and ketone bodies will be measured via a fingerstick blood sample. A urine sample will be collected to evaluate ketone bodies and if appropriate pregnancy status. The 24 hour urine container will be collected. Insulin dose will be adjusted if needed as in as per Visit 11. Sufficient supply of IMPs will be given until the end of the study. A fasting blood sample will be taken (45 mL) to evaluate study endpoints.

Visit Procedures

Fasting visit* - as per previous fasting visits

Monitoring and treatment optimisation
- Check adequacy of contraception (WOCBP)
- Check for adverse events
- Review blood glucose diary
- Adjust insulin doses (if necessary)
- Blood ketones
- Daily urine ketones review

Clinical assessments

- Blood pressure
- Heart rate
- Weight

Quality of life assessments
- Diabetes Specific Quality of Life Scale (DSQOLS)
- Problem Areas In Diabetes (PAID) questionnaire

Blood assessments
- Na, K, urea, creatinine, eGFR (MDRD)
- AST/ ALT/ Alkaline Phosphatase
- Calcium/ Phosphate
- FBC
- HbA1c
- Fructosamine
- Bilirubin
- Random glucose

Urine
- Pregnancy test (WOCBP randomised to IMP only)
- Urine ketones
- 24 hour urine bottle collected

Medication
- Record doses of semaglutide administered since Visit 10 (self-report)
- Dispense open label semaglutide for participants randomised to active treatment at Period 1 (if required).
- Count returned dapagliflozin or placebo capsules
- Dispense dapagliflozin or placebo (record dose)

7.6.6 Visit 14 (@23 weeks after Second Randomisation)

For those randomised to open-label semaglutide, doses administered will be recorded (self-report). Clinical observations will be recorded. Daily blood glucose and blood/ urine ketone levels will be reviewed and glucose levels collected. Blood glucose levels and ketone bodies will be measured via a fingerstick blood sample. A urine sample will be collected to evaluate ketone bodies and if appropriate pregnancy status. Insulin dose will be adjusted if as per Visit 11. All subjects will be reminded to monitor their capillary blood glucose by fingerstick at least four times a day (before meals and at bedtime). CGM (Dexcom G6) sensor will be fitted and patients asked to wear their CGM constantly for the next 20 days.

Visit Procedures

Monitoring and treatment optimisation
- Check adequacy of contraception (WOCBP)
- Check for adverse events
- Review blood glucose diary

- Adjust insulin doses (if necessary)
- Blood ketones
- Daily urine ketones review

Clinical assessments
- Blood pressure
- Heart rate
- Weight

Urine sample:
- Pregnancy test (WOCBP randomised to IMP only)
- Urine ketones

Clinical and functional assessment
- *Fit and start* masked Continuous Glucose Monitoring sensor (mean fasting blood glucose, mean blood glucose, percentage time in range, glycaemic variability)

7.6.7 Visit 15 (@25 weeks after Second Randomisation)

Participants will be asked to attend in the fasting state. If participants arrive at the study visit in a non-fasted state, the visit will be terminated and a further fasting visit rearranged as soon as possible. For those randomised to open-label semaglutide, doses administered will be recorded (self-report). Observations will be taken. Daily blood glucose and blood/ urine ketone levels will be reviewed and glucose levels collected. Blood glucose levels (fingerstick or FGM) and ketone bodies will be measured via a fingerstick blood sample. A urine sample will be collected to evaluate urine ketone levels. Insulin dose will be adjusted if needed as per visit 11. A 24 hr urine container will be dispensed to collect a 24 hour urine sample to bring in at the Week 52 visit. A HFHC Meal Challenge Test will be performed as in Visit 2.

Visit Procedures

Fasting visit* - Procedure as per previous fasting visits

Monitoring and treatment optimisation
- Check adequacy of contraception (WOCBP)
- Check for adverse events
- Review blood glucose diary
- Adjust insulin doses (if necessary)
- Daily urine ketones review
- Blood ketones

Assessments
- Blood pressure
- Heart rate
- Weight

Urine sample:
- Pregnancy test (WOCBP randomised to IMP only)
- 24 hour urine container dispensed

Other assessments

- Meal Challenge Test

7.6.8 Visit 16 (End of study; @26 weeks after Second Randomisation)

Participants will be asked to attend in the fasting state. If participants arrive at the study visit in a non-fasted state, the visit will be terminated and a further fasting visit rearranged as soon as possible. Clinical observations will be recorded. Daily blood glucose and blood/ urine ketone levels will be reviewed and glucose levels collected. Blood glucose levels and ketone bodies will be measured via a fingerstick blood sample. Insulin doses will be reviewed and likely requirement for an increase following discontinuation of IMP discussed with those taking open-label semaglutide. A urine sample will be collected to evaluate ketone bodies and, if appropriate pregnancy status. The CGM will be collected. The 24 hour urine container will be collected. A fasting blood sample will be taken (45 mL) to evaluate study endpoints. We will ask the participants to complete quality of life questionnaires. A physical examination will be performed as per the screening visit. This will be the final face-to-face study visit. A safety phone call will be conducted two weeks to review blood glucose data, provide further insulin dosing advice, to document any adverse events, and to ensure that follow-up in usual clinical care has been arranged.

Visit Procedures

Fasting visit*

Monitoring and treatment optimisation

- Check for adverse events
- Review blood glucose diary
- Adjust insulin doses (if necessary)
- Blood ketones
- Daily urine ketones review

Clinical assessments

- Blood pressure
- Weight
- Heart rate
- Physical examination

Quality of life assessments

- Diabetes Specific Quality of Life Scale (DSQOLS)
- Problem Areas In Diabetes (PAID) questionnaire

Blood assessments

- Na, K, urea, creatinine, eGFR (MDRD)
- AST/ ALT/ Alkaline Phosphatase
- Calcium/ Phosphate
- FBC
- HbA1c

- Fructosamine
- Bilirubin
- Random glucose
- C-peptide

Urine
- Pregnancy test (WOCBP randomised to IMP only)
- Urine ketones
- 24 hour collection

Other

- Record doses of semaglutide administered (self-report)
- Return used study pens
- Count returned dapagliflozin or placebo capsules
- Collect Patient Alert Card
- Read masked Continuous Glucose Monitoring sensor (mean fasting blood glucose, mean blood glucose, percentage time in range, glycaemic variability)

7.7 Retention and strategies for maximizing follow-up

Participants will be encouraged to attend all study visits. Prior to commencing the trial, all potential participants will have the opportunity to read the detailed Patient Information Sheet which will outline the level of participation requested. Potential participants will be made aware of the need for frequent study visits and asked to commit to these.

Study visits can be rearranged flexibly within a period around the planned visit as specified in the timeline of visits above (Section 7) and actual dates recorded in the eCRF. This is to maximise participant ability to attend all visits. A longer or shorter interval does not constitute a protocol deviation. Where possible, visits will be arranged to coincide with other appointments to further maximise attendance and participant convenience. Telephone contact will be frequent and will help to maintain contact with participants and ensure timely reminders of upcoming study visits can be given.

Even in the scenario of treatment discontinuation (of either IMP), participants will be encouraged to continue to attend further study visits as scheduled.

As stated in point 3 of the Schedule of Assessments, telephone visits are allowed for V4, V5 and V11 for those randomised to the standard of care arm. Participants who have discontinued both study IMPs (see below Section 7.8 for criteria) can also be offered telephone visits for V4, V5 and V11 if this is preferable and encourages retention in the study.

7.8 Treatment Interruptions and Withdrawal criteria

Treatment Interruptions

Treatment interruption from study IMPs may be necessary in some participants. In the case of dapagliflozin, this will occur in any acute illness or hospitalisation which increases the risk of ketosis and ketoacidosis. Circumstances in which temporary discontinuation of dapagliflozin or placebo should occur will be discussed with all study participants. Written information will be provided in the study diaries. An emergency phone line to the study team will be available to all participants for the duration of their

participation in the trial. This will be operational during office hours (Monday to Friday 9am – 5pm). The Patient Alert Card also contains contact details for unblinding in the event of acute intercurrent illness and a requirement for unblinding in the opinion of the acute medical team treating the participant. If any participant develops diabetic ketoacidosis (DKA) as defined as per the International Consensus guidelines on two occasions with no identifiable precipitant (section 2.1) they will be withdrawn from the study and the UK Chief and US Principal Investigators informed.

Any other intolerable side effects, acute illness, or other clinical change which may impact participant safety (e.g. excessive weight loss) may require temporary discontinuation of the study drug(s) (dapagliflozin/placebo or semaglutide). In these circumstances, any decision to recommence the study drug(s), the dose and the appropriateness of re-uptitration, will be taken in consultation with the participant, including consideration of the total participant burden, particularly given the need for additional safety checks. If in the judgement of the site PI the study drug(s) cannot be safely restarted, if side-effects remain intolerable or if any of the criteria for study drug withdrawal are met, the treatment with the study medicine(s) must be permanently stopped. Participants who have withdrawn from treatment will be encouraged to continue to attend study visits.

Criteria for withdrawal

Treatment withdrawal or withdrawal from the trial as appropriate (see below) may be necessary prior to the planned end at 52 weeks following first randomisation for any of the following reasons:

1. The site PI can stop investigational study treatment or remove participants from the trial without participants approval if for any reason they feels is appropriate, including: severe side effects, injury or medical condition which may place the patient at risk of further complications if he/she continue to participate, failure to take the medication as instructed, failure to keep scheduled appointments, insufficient level of self-monitoring, cancellation of the study or other administrative reasons.

2. Any woman who becomes pregnant during the period of study will be withdrawn from the trial.

3. Participants who experience persistent severe nausea or vomiting after starting semaglutide which is not managed by dose reduction will discontinue the investigational study treatment. Those with mild or moderate nausea will continue for at least one more week. For those who continue to experience significant nausea (defined as interfering with normal activities), dose will be reduced: if these side effects persist, they will discontinue study medication(s). Participants for whom this occurs in Period 1 will remain eligible for randomisation in Period 2.

4. Excessive weight loss during either period of the study despite treatment dose reduction (> 15% body weight or BMI < 22 kg/m^2 on repeated measurements more than four weeks apart) will lead to treatment withdrawal. If the participant is willing, they may continue to attend study visits (at the discretion of the site PI).

5. New diagnosis of other conditions included in the Exclusion Criteria will result in withdrawal of the participant from the trial.

6. Change in method of insulin delivery: any participant in whom insulin delivery is changed from CSII to MDI or vice versa during the study will be withdrawn from the trial unless pump treatment interruption is temporary for a period of at most 2 weeks. Participants who upgrade to "artificial

pancreas" (e.g. Medtronic's MiniMed 670G hybrid) or any form of hybrid closed-loop therapy during the study will be withdrawn from the trial.

7. Hypoglycaemia: any participant who experiences two episodes of severe hypoglycaemia (requiring external intervention) will be withdrawn from the trial.

8. Ketosis (with no other identifiable precipitating factors): any participant who experiences persistent (1 week) asymptomatic or symptomatic and unmanaged (using STICH protocol – see section 2.1 b) blood ketone levels >1.5 mmol (or ≥+2 in urine) will be withdrawn from the trial. (37)

9. Insulin dose: any participant who experiences a persistent (>1 week) total insulin dose reduction of >20% from baseline AND total insulin dose <0.4U/Kg in Period 2 will be withdrawn from the trial.

10. DKA: any participant who experiences two episode of DKA (with no identifiable precipitating factors) as defined by the expert consensus statement will be withdrawn from the trial (39)

11. UTI: any participant who experiences two episodes of UTI will be withdrawn from the trial.

12. Genital mycotic infection: any participant who experiences two episodes of mycotic genital infection with severe symptoms will be usually be withdrawn from the trial (see also heading "Genital mycotic infections," page 32).

13. Any participant who experiences significant persistent reduction in eGFR > 15 ml/min will be withdrawn from the trial.

14. Pancreatitis: any participant who experiences an episode judged to be due to pancreatitis by the site PI will be withdrawn from the trial.

15. Participants who experience other intolerable and persistent side effects specific to study drugs will be offered treatment downtitration or treatment withdrawal with continued follow-up within the trial if they are willing - except when this is considered inappropriate by the site PI, in which case they will be withdrawn.

Participation in this study is voluntary. Participants have the right to refuse to participate or to withdraw from participation at any time for any reason. The site PI can stop investigational study treatment or remove participants from the trial without participant's approval. If a patient withdraws consent and decides not to participate in the study or is withdrawn from the trial according to the above criteria, she/he will be asked to undergo a final study visit for his/her safety. This visit will follow the same procedures for Visit 16 provided there is ongoing consent. The research team will arrange appropriate handover to the usual clinical team – additional telephone (of if necessary face to face) contact can be arranged if necessary in the interim. If a subject withdraws from the research, the data collected to that point will be used toward the research finding, including study data collected at final study visit, provided there is ongoing consent. If applicable the subject will be asked to return any unused research drug and or devices.

7.9 Storage and analysis of samples

Blood samples will be undertaken at the beginning and at various points during the study as outlined in detail in the schedule of visits. If blood testing has recently been undertaken in the 3 weeks prior to the

study commencing, some of these blood tests may be used as baseline. Blood tests will include analysis of the full blood count, renal function (urea and electrolytes), liver function tests, bone profile, and several specialised blood tests for biomarker assays (at central laboratory) following a high fat high caloric meal challenges to examine study endpoints.

Covid-19 Mitigation

All blood sampling or trial procedures will be undertaken following local and national guidance around personal protective equipment. Mask, gloves and aprons will be used as per current guidelines.

7.9.1 Sample collection and processing

- Samples will be appropriately labelled in accordance with the trial procedures to comply with the General Data Protection Regulation (GDPR). Biological samples collected from participants as part of this trial will be transported, stored, accessed and processed in accordance with national legislation relating to the use and storage of human tissue for research purposes and such activities shall at least meet the requirements as set out in the 2004 Human Tissue Act and the 2006 Human Tissue (Scotland) Act.
- Blood will be taken according to the Schedule of Assessments (page 17). Plasma will be siphoned, aliquoted and stored locally at -80° ± 10°C until transport in batches to the central laboratory on dry ice. according to the Sample Handling Procedure as agreed by the Joint Study Management Group
- Individual patient samples will be identified with a unique, anonymised study number.

7.9.2 Sample transport to central laboratory and analysis

- Samples will be transferred to State University of New York in a single batch at the end of recruitment to the study. More frequent transfer can be organised if there are local storage limitations.
- Samples will be transported on dry ice and stored at the central laboratory at -80° ± 10° until analysis. Transport by courier will coordinated by the Trial Manager.
- At the time of analysis individual aliquots will be defrosted at room temperature and analysed in a single batch for each biomarker of interest.
- Samples will be stored at -80° ± 10° in the central laboratory for possible future analysis for novel biomarkers.

7.10 End of trial

The end of the trial will be following the last study visit of the last subject.
At the end of the specified trial period (approximately 52 weeks following first randomisation) all participants will discontinue study medications and revert to standard of care (insulin therapy only). This process will be supervised by the trial physicians, research nurses, and research pharmacists under the supervision of the site Principal Investigator. The trial team will liaise with the usual care team to ensure a safe transition back to standard therapy. Following the final scheduled trial visit there

will be a safety phone call two weeks after the last visit. This will act as a further opportunity to advise on insulin dosing and ensure there are no ongoing problems or concerns related to the study.

As discussed in section 7.8, there may be circumstances in which trial drugs need to be stopped before the specified end of the trial. If this is the case, the reason(s) for discontinuation will be recorded and the trial clinical team will facilitate safe transition back to usual standard of care when appropriate. Participants will however if eligible (i.e non-exclusion criteria reason for IMP discontinuation – as listed in Section 7.8) be asked to continue with trial visits. If consent is withdrawn and participants voluntarily choose to leave the trial before the specified end date, all efforts by the clinical trial team will be made to safely facilitate transition back to ordinary standard of care. A follow up safety visit will be offered to all such participants and ongoing contact details will be made available should there be any further unforeseen issues relating to the clinical trial.

The trial may be stopped at an earlier stage than anticipated on the advice of the independent data monitoring committee (IDMC) to the trial steering committee (TSC) for the safety reasons detailed on page 12 and in the study safety endpoints. If this occurs, all participants will be informed and safe discontinuation of study medications will be undertaken under the supervision of the site Principal Investigator.

8 TRIAL MEDICATION

8.1 Name and description of investigational medicinal product(s)

a) Semaglutide (Ozempic®): a once weekly GLP-1 agonist injection indicated for type 2 diabetes (approved by the Scottish Medicines Consortium, January 2019).

   Doses to be used: 0.25mg increased to 0.5mg at two weeks and then to 1mg weekly s.c. injection after a further two weeks. Open label semaglutide will be added to a background of insulin treatment at week 0 for 12 months in 76 participants (38 in the UK) according to the first randomisation; at week 26 they will be further randomised (double-blind) as below into two groups: dual (placebo, semaglutide and insulin) and triple (dapagliflozin, semaglutide and insulin) therapy.

b) Dapagliflozin (as dapagliflozin propanediol monohydrate Forxiga®): a once daily SGLT-2 inhibitor that will be administered orally in a blinded fashion.

   Doses to be used: 5 mg daily increased after one week to 10 mg daily (orally). Dapagliflozin will be started according to the randomisation (Interactive Web System, hosted by University of Glasgow) in 38 participants (19 in the UK) at 26 weeks as an additional treatment on a background of insulin and semaglutide therapy and end at 52 weeks (triple therapy group). Doses to be used: 5 mg increased to 10 mg after one week.

c) Placebo to dapagliflozin: Dapagliflozin matched placebo will be started in 38 participants (19 in the UK) participants at 26 weeks as an additional treatment on a background of insulin and semaglutide therapy and end at 52 weeks (dual therapy group). Doses to be used: as for active dapagliflozin.

8.2 Legal status of Semaglutide and Dapagliflozin

Semaglutide is currently licensed in the UK for those with adult type 2 diabetes which is insufficiently controlled.

8.3 Drug storage and supply

Semaglutide and dapagliflozin/placebo supplies must be stored in the original containers in a locked, secure area with access limited to authorised site staff. Study supplies should be used as directed in the study protocol and not be supplied to any persons other than study participants. All IMPs will be dispensed to participants from the study site pharmacy.

Covid-19 Mitigation

As detailed above in section 7, the supply of IMP may be altered to reduce risk of Covid-19 transmission in the event of future waves of infection. This may involve providing increased quantities of IMP to participants to last longer and therefore minimise physical contact with trial personnel. Use of a courier delivery service to facilitate this may also be considered.

Semaglutide: Supplies of semaglutide pre-filled pens for subcutaneous administration will be sourced from routine hospital stocks. The following semaglutide presentations will be required:

- Semaglutide 0.25 mg: Each pre-filled pen contains 2mg semaglutide in 1.5ml. Each dose contains 0.25mg of semaglutide in 0.19ml solution.
- Semaglutide 0.5 mg: Each pre-filled pen contains 2mg semaglutide in 1.5ml solution. Each dose contains 0.5 mg of semaglutide in 0.37ml solution.
- Semaglutide 1 mg: Each pre-filled pen contains 4 mg semaglutide in 3.0ml solution. Each dose contains 1mg semaglutide in 0.74ml solution.

For all presentations each pre-filled pen is capable of delivering 4 doses which is sufficient for 4 weeks treatment. Pens are for individual participant use only. In the event of a pen-failure, a new pen should be supplied to the participant.

Prior to first use, semaglutide pens must be stored at 2-8 °C in the original container and must not be frozen. Supplies which are frozen either prior to first use or during use must be discard.

Semaglutide pre-filled pens have an in-use shelf life of 6 weeks. After first use the pre-filled pens may be stored below 30oC or in a refrigerator at 2-8 °C. Participants should be advised to store packs away from the cooling element. The pen cap should be on when the pen is not in use in order to protect the contents from light.

Wherever possible, supplies of semaglutide pre-filled pens should be utilised in a pragmatic way so as to minimise unnecessary wastage. Participants should be provided with an appropriate number of pen(s) to last until the next planned dispensing visit. A small overage may be given as necessary to accommodate visit windows etc. The correct strength of pen must be supplied for each dose titration i.e. 0.25mg, 0.5mg and 1.0 mg as tolerated.

A web-based system will be used to randomise participants to semaglutide treatment.

Dapagliflozin 5 mg or placebo capsules: Supplies of dapagliflozin 5mg and matched placebo capsules will be manufactured by NHS Scotland Pharmaceuticals Specials Service/NHS Tayside

Pharmaceuticals in accordance with Good Manufacturing Practice requirements. Capsules will be packed into HDPE bottles with a tamper evident seal. Each bottle will be labelled in a double-blind fashion to meet local regulatory requirements and will be assigned a unique number. Bottles containing dapagliflozin 5 mg or placebo must be stored at room temperature at 15-25°C.

Dapagliflozin 5 mg and placebo capsule supplies will be provided to study site and will only be released by the sponsor once all the appropriate regulatory and governance approvals are in place. Further information on storage requirements and supply arrangements is provided in the study specific IMP Management and Accountability Manual.

A web-based system will be used for the assignment of dapagliflozin 5mg or placebo bottles at randomisation and at study visits where there is planned dispensing of the IMP or in the event that a participant requires replacement medicines.

Other medicines: Supplies of all other medicines and associated consumables will via usual NHS supply mechanisms.

8.4 Drug accountability requirements

The Investigator or designee must maintain accurate records of all study IMP movements for accountability purposes. They should include dates, quantities, batch numbers and expiry. Records must document adequately that:

- the patients were provided the doses specified by the protocol/amendment(s)
- all study drug provided was fully reconciled.

Unused study drug must not be discarded or used for any purpose other than the present study. Further information is provided in the study specific IMP Management and Accountability Manual.

8.5 Preparation and administration of semaglutide and dapagliflozin

Semaglutide (Ozempic®) is a solution for subcutaneous injection. It is administered once weekly, on the same day each week, into the subcutaneous tissues of the abdomen, thigh or upper arm. A participant may change the day of their weekly injection as long as it has been at least three days since their last injection. Thereafter, all doses should be administered once weekly on the new dosing day. The injection can be administered at any time of the day regardless of meals.

Participants must be advised to discard the injection needle after each injection and store the pen without an injection needle attached. This may help prevent blocked needles, contamination, infection, leakage of solution and inaccurate dosing. Semaglutide should not be used if the solution does not appear clear and colourless or almost colourless. The pen is designed to be used with NovoFine or NovoTwist disposable needles and pens are packed with an appropriate quantity of NovoFine Plus needles.

Detailed information on administration of semaglutide is provided in the current Summary of Product Characteristics and product Patient Information Leaflet.

Dapagliflozin (Forxiga®))and matched placebo are presented as a capsule for once daily oral administration.

8.6 Dosage schedules

Semaglutide will be administered and dosed as described. Participants will be commenced on 0.25 mg s.c once weekly before up-titration as tolerated to 1.0 mg s.c once weekly. Up-titration is quicker than is ordinarily the case in clinical practice in type 2 diabetes as discussed in Section 2.1 (22) There will be increased observation and clinical access for participants in the event of side-effects. Participants

randomised to receive semaglutide will continue on the 1.0 mg s.c once weekly dose for the remainder of the study following uptitration provided they tolerate the dose as detailed in Section 7. If necessary, the dose may be adjusted.
If a participant misses a dose of semaglutide it should be administered as soon as possible and within 5 days after the missed dose. If more than 5 days have passed, the missed dose should be skipped, and the next dose should be administered on the regularly scheduled day. In both cases participants, should then resume their regular once weekly dosing schedule.

Dapagliflozin and matched placebo will be commenced at 5mg orally once daily and uptitrated one week after commencement to a dose of 10mg which will thereafter be continued for the remainder of the trial provided they tolerate the dose. If necessary, the dose may be adjusted.

8.7 Dosage modifications

Dapagliflozin/placebo will be commenced at one capsule (5mg or placebo) orally once daily and uptitrated one week after commencement to a dose of two capsules (10mg or placebo) which will thereafter be continued for the remainder of the trial provided they tolerate the dose. If necessary, the dose may be adjusted. (See also Section 7.8 regarding treatment discontinuation) Dapagliflozin or placebo capsules can be taken at any time of day with or without food. Capsules should be swallowed whole. If participants forget to take a dose they should be advised as follows:

- If it is 12 hours or more until the next dose, the dose should be taken as soon as remembered and the next dose taken at the usual time.
- If it is less than 12 hours until the next dose, the missed dose should be skipped. The next dose should be taken at the usual time.
- Participants should not take a double dose of dapagliflozin/placebo to make up for a forgotten dose.

8.8 Known drug reactions and interaction with other therapies

Participants with hypersensitivity to either active study medication are excluded from the trial. There are no significant interactions between semaglutide and other prescribed medications other than with agents that can lower blood glucose levels. Participants on any other oral agents which can lower blood glucose levels meet Exclusion Criteria and will not be included in this trial. In combination with insulin and dapagliflozin, semaglutide may be associated with an increased risk of hypoglycaemia. Participants will be advised of the increased risk of hypoglycaemia and will be required to closely monitor their blood glucose levels for the duration of the trial. No interactions are currently listed between warfarin and semaglutide in the British National Formulary but in view of a theoretical interaction (based on protein binding), existing arrangements for INR testing will be reviewed for any participants prescribed warfarin and additional tests conducted if indicated according to the judgement of the investigators.

Dapagliflozin is known to increase the risk of hypoglycaemia when combined with other medications which also act to lower blood glucose levels. Dapagliflozin has a mild diuretic effect and therefore there is an increased risk of hypotension when dapagliflozin is combined with either medications which deplete circulating volume (e.g diuretics) or antihypertensive drugs. Dapagliflozin is however commonly prescribed with such medications in general medical practice. All participants will have their medications reviewed at the screening visit and educated regarding symptoms that may indicate dehydration/ hypovolaemia.

Dapagliflozin may increase renal lithium excretion leading to decreased blood lithium concentrations. Serum lithium concentrations should be monitored for participants eligible for randomisation to dapagliflozin/placebo prior to visit 10 (week 26, second randomisation), as clinically indicated thereafter, and in the event of dose changes, taking into account the planned dose increase at visit 11 (week 27).

If there is any concern of interaction with any of the IMPs, this will be discussed with the PI before a decision regarding their ability to participate is made.

COVID-19: COVID-19 infection and the clinical management in this therapeutic area is also rapidly evolving as new medicines are approved for use by local/national treatment guidelines. It is therefore not possible to comprehensively rule out the potential for clinically significant interactions between the study IMP(s) with current or future medicines for the acute treatment of COVID-19 and a pragmatic approach is required. Medicines for acute COVID-19 infection should not be unnecessarily withheld and participants should be managed wherever possible in line with local/national clinical guidelines for this patient group. The decision to follow a particular management plan for acute COVID-19 infection (which may include the temporary withdrawal of the IMP(s) should this be in the participant's best interest), is at the treating physician's discretion. Investigators should refer to the protocol and current IMP Summary of Product Characteristics for detailed information on known mechanisms for pharmacokinetic/pharmacodynamics interactions as well as the relevant COVID-19 treatment Summary of Product Characteristics (or equivalent) to inform management decisions. Any mitigations, such as additional patient monitoring, must be documented in the participant's medical notes.

Further ad-hoc advice can also be obtained from the Sponsor/Chief Investigator and https://www.covid19-druginteractions.org/ Investigators must be vigilant at all times to the possibility of interactions and report in line with protocol pharmacovigilance requirements. Concomitant use of other medicines for COVID-19 must be recorded in the participant's CRF. Investigational treatments for acute COVID-19 treatment are not permitted without prior approval from the sponsor.

8.9 Concomitant medication

Participants should not receive any adjunct glucose-lowering therapy other than as per the randomisation schedule (e.g. with either of the active study medications).

8.10 Trial restrictions

Contraindications will follow SmPC information for Ozempic® and Forxiga. Acceptable forms of contraception for WOCBP participating in the trial are listed in Appendix 2. There is no requirement for contraception for male participants.

8.11 Assessment of compliance

Treatment adherence will be assessed by the trial medical and nursing team when participants attend for scheduled study visits.

8.12 Name and description of each Non-Investigational Medicinal Product (NIMP)

Insulin therapy is a non-investigational medicinal product in this trial. The trial is in a population who have had a diagnosis of type 1 diabetes for at least one year: therefore all participants will already be established on insulin therapy. The regimen and dosage required will by differ between participants. Participants will be using a combination of long acting (basal) insulin and shorter acting (bolus) insulin or CSII (insulin pump). As in standard of care, dose titration will depend on diet, blood glucose levels and exercise. It is anticipated that dosage adjustments will be required to achieve optimal glycaemic control and maintain participant safety.

There are pre-defined guidelines for insulin titration in this study. Insulin dose reductions should not be greater than 10% (5% if HbA1c is<7%) from current total insulin dose or more than 20% from the baseline level recommended following review at the randomisation visit and then from the new baseline level on review at 26 weeks (second randomisation). Participants requiring an insulin dose reduction > 20% to a total insulin <0.4 U/kg will be monitored closely for insulin dose, carbohydrate intake, ketones and blood glucose. Study medication can be temporarily discontinued, if necessary, for one-to-two weeks. If low insulin requirements remain, the participant will be withdrawn from study (see Section 7.8).

9 PHARMACOVIGILANCE

9.1 Definitions

Term	Definition
**Adverse Event (AE)**	Any untoward medical occurrence in a participant to whom a medicinal product has been administered, including occurrences which are not necessarily caused by or related to that product.
**Adverse Reaction (AR)**	An untoward and unintended response in a participant to an investigational medicinal product which is related to any dose administered to that participant.<br>The phrase "response to an investigational medicinal product" means that a causal relationship between a trial medication and an AE is at least a reasonable possibility, i.e. the relationship cannot be ruled out.<br>All cases judged by either the reporting medically qualified professional or the Sponsor as having a reasonable suspected causal relationship to the trial medication qualify as adverse reactions.
**Serious Adverse Event (SAE)**	A serious adverse event is any untoward medical occurrence that:<br>1. results in death<br>2. is life-threatening<br>3. requires inpatient hospitalisation or prolongation of existing hospitalisation<br>4. results in persistent or significant disability/incapacity<br>5. consists of a congenital anomaly or birth defect<br>Other ‘important medical events’ may also be considered serious if they jeopardise the participant or require an intervention to prevent one of the above consequences.<br>NOTE: The term "life-threatening" in the definition of "serious" refers to an event in which the participant was at risk of death at the time of the event; it does not refer to an event which hypothetically might have caused death if it were more severe*.
**Serious Adverse Reaction (SAR)**	An adverse event that is both serious and, in the opinion of the reporting Investigator, believed with reasonable probability to be due to one of the trial treatments, based on the information provided.
**Suspected Unexpected Serious Adverse Reaction (SUSAR)**	A serious adverse reaction, the nature and severity of which is not consistent with the information about the medicinal product in question set out:<br>• in the case of a product with a marketing authorisation, in the summary of product characteristics (SmPC) for that product<br>• in the case of any other investigational medicinal product, in the investigator’s brochure (IB) relating to the trial in question
**Reference Safety Information**	The information used for assessing whether an adverse reaction is expected. This is contained in either the investigator’s brochure or the summary of product characteristics

*Note: “Severe” is often used to describe intensity of a specific event, which may be of relatively minor medical significance. “Seriousness” is the regulatory definition supplied above.

9.2 Operational definitions for (S)AEs

Adverse events (AEs) will be recorded, notified, assessed, reported, analysed and managed in accordance with the Medicines for Human Use (Clinical Trials) Regulations 2004 (as amended) and this protocol.

All Serious Adverse Events (SAEs) occurring during the trial will be recorded within the eCRF. Non-serious AEs occurring during the trial will be recorded within the eCRF if they are considered to be related to trial medication, i.e. adverse reactions (ARs).

Exclusions from SAE reporting

Hospitalisation for the following reasons will not be considered to be SAEs:

- Treatment which was elective or pre-planned, for a pre-existing condition not associated with any deterioration in condition, e.g. pre-planned hip replacement operation which does not lead to further complications.
- Any admission to hospital or other institution for general care for less than 24 hours where there was no deterioration in condition.
- Treatment on an emergency, outpatient basis for an event **not** fulfilling any of the definitions of serious as given above and not resulting in hospital admission.
- COVID-19 infection unless results in severe illness with hospital admission.

9.3 Recording and reporting of AEs, Adverse Events of Special Interest, SAEs and SUSARs

All AEs occurring during the trial that are observed by the Investigator or reported by the participant will be recorded in the participant’s medical records whether or not attributed to trial IMP. AEs will be recorded from randomisation until the later of 30 days post cessation of trial treatment or the end of the study.

With the exception of those noted in section 9.3.1 below, adverse reactions will be recorded in a log provided in the eCRF.

Adverse Events of Special Interest, SAEs and SUSARs will be recorded in the participant’s medical records and in a specific section of the eCRF from the point of randomisation until the later of 30 days post cessation of trial treatment or the end of the study.

9.3.1 Adverse Events of Special Interest

Ketosis is a known adverse effect of dapagliflozin (see Section 2.1b; also (i) below). Hypoglycaemia is a recognised adverse effect with both semaglutide and dapagliflozin when used in combination with insulin [see (ii) below] and is defined as moderate in intensity (Level 2) when blood glucose is < 3.0 mmol/L. Thus ketosis and Level 2 hypoglycaemia events will be recorded as Adverse Events of Special Interest (AESIs).

AESIs will be recorded within the relevant section of the eCRF but do not require reporting in an expedited manner (unless the event also meets the regulatory definition of an SAE). The following information will be recorded:

- Nature of the event
- Event duration (start and end dates, if applicable
- Relationship to study drug
- Outcome

Adverse Events of Special Interest will be monitored and followed up (if applicable) until the event has resolved or a final outcome has been reached.

All Adverse Events of Special Interest must be assessed for seriousness prior to recording within the eCRF. Those meeting the definition of an SAE should also be reported accordingly as per Section 9.3.2.

(i) Ketosis and diabetic ketoacidosis

This trial will be conducted in participants who have type 1 diabetes. All participants will be taking insulin. In this condition, due to the near absence of endogenous insulin, at a cellular level there is an inability to incorporate glucose into normal intra-cellular energy yielding pathways. In this context, alternative energy yielding pathways are sought including ketone body production from fatty acids in the liver. Ketones are clinically important as they easily dissociate hydrogen ions upsetting physiologically regulated acid base balance. As ketone levels rise, there is initially a state of high blood ketone levels without significant acidosis (ketosis); if this develops further, diabetic ketoacidosis (DKA) may develop. DKA requires urgent medical input. Ketones can be easily measured via a simple urine test or fingerstick capillary blood measurement. Participants eligible for this trial will already be trained in how to do this. As discussed in Background ((1) above), dapagliflozin, one of the IMPs in this trial, is known to increase the risk of ketosis and ketoacidosis and this can occur at lower blood glucose levels than in those who are on insulin treatment only [euglycaemic DKA] (13,14). Given this known risk, ketosis and DKA are included in the safety endpoints and as such will be recorded in the eCRF as Adverse Events of Special Interest.

DKA is defined clinically and biochemically as a combination of a state of elevated ketone levels (urine ++ or higher, and/or blood > 1.5 mmol/L) in combination with biochemical evidence of a state of metabolic acidosis (pH ≤7.30 or serum bicarbonate < 15 mmol/L). This is as defined by International Consensus Guidelines (39). DKA also varies in severity. In clinical practice the diagnosis is made when a person with type 1 diabetes has elevated ketone levels and usually – although not exclusively – elevated blood glucose levels. The diagnosis is made biochemically by assessing acid base balance through blood testing. This corresponds to the following severity classifications:

- mild DKA: pH 7.25-7.30 (H^+ 50 – 56) or bicarbonate (15 -17.9 mmol/L)
- moderate DKA: pH 7.00-7.24 (H^+ 57 – 100) or bicarbonate 10 – 14.9 mmol/L)
- severe DKA: pH <7.00 (H^+ > 100) or bicarbonate < 10 mmol/L (39)

In addition to the above, the highest level of either blood or urine ketones measured, the serum bicarbonate (if measured) and blood pH (if measured) will be recorded for each episode. Given the known risk of DKA, all participants will be made aware of this condition. Patient diaries and Patient Alert Cards will summarise advice for management of high ketone levels including an emergency ketosis management protocol (STICH Protocol – see Appendix 3) (37). All participants will be

educated as to the scenarios when this should be activated and this will also be included on the Patient Alert Card which will have the contact details of the study team and also contact details for out of hours health services. Participants will be asked to carry this card at all times.

(ii) Hypoglycaemia

Hypoglycaemia is a low blood glucose level. Hypoglycaemia occurs in diabetes owing to difficulty in precisely matching insulin dosing with the complex factors associated with carbohydrate intake and physical activity. When too much insulin relative to carbohydrate intake is administered, blood glucose levels can drop to a level which is too low. Most people with type 1 diabetes can recognize this phenomenon due to a combination of symptoms which alert them and prompt self-checking of their capillary blood glucose level. Symptoms include weakness, malaise, headache, tremulousness, diaphoresis, palpitations and mild confusion. These are known as autonomic symptoms as they are mediated by activation of the autonomic nervous system as a response to the biological stress of low serum blood glucose. As hypoglycaemia progresses, however, symptoms progress to include confusion and agitation. If untreated this can lead to coma. These symptoms are known as neuroglycopenic symptoms as they are mediated by a lack of glucose in neural cells leading to a confused state.

Hypoglycaemia is also well defined by International Consensus (39). Like DKA, it can be classified in terms of severity. This can be based either on the absolute capillary glucose value and/or clinical features and is classed into stages:

- Level 1 (mild) hypoglycaemia: Blood glucose is 3.0 – 3.8 mmol/L inclusive. At each visit the number of episodes since the last visit during which blood glucose was within this range (but not lower) will be recorded on the “Fasting Blood Glucose” tab of the eCRF following review of the participant’s study diary and (if applicable) FGM/ CGM download.
- Level 2 (moderate) hypoglycaemia: Blood glucose is < 3.0 mmol/L At each visit the number of episodes since the last visit during which blood glucose was within this range at any time will be recorded on the AESI tab of the eCRF following review of the participant’s study diary and (if applicable) FGM/ CGM download.
- Level 3 (severe) hypoglycaemia: A person is unable to function because of mental or physical changes due to hypoglycaemia and requires assistance from another person to recover. These events will be recorded as SAEs (see below) independent of recording of blood glucose according to the opinion of the site Principal Investigator as by definition they jeopardise the participant or require an intervention to prevent life-threatening consequences.

The IMPs in this trial are licensed clinically in type 2 diabetes to lower blood glucose levels. It therefore follows that when used in combination with insulin there may be an increased risk of low blood glucose levels. This was the case with the once daily GLP-1 analogue liraglutide in phase 3 trials (8,9), but not in the trials with dapagliflozin (13,14). All participants will have this risk discussed with them, will be performing increased blood glucose monitoring than is the case in usual care, and will be frequently reviewed to adjust insulin dosing in order to mitigate the risk of low blood glucose levels.

9.3.2 Site Reported Serious Adverse Events (SAE)

SAEs will be recorded within the eCRF from the date of randomisation until the later of 30 days post cessation of trial treatment or the end of the study.

Full details of SAEs will be recorded in the electronic Case Report Form. The following information will be collected:

- full details in medical terms and a case description
- seriousness criteria

- event duration (start and end dates, if applicable)
- action taken
- outcome
- causality (i.e. relatedness to trial drug / investigation), in the opinion of the investigator
- if related, whether the reaction would be considered expected or unexpected.

Any change of condition or other follow-up information should be added to the eCRF and forwarded to the Sponsor (if reportable SAE) as soon as it is available or at least within 24 hours of the information becoming available. Events will be followed up until the event has resolved or a final outcome has been reached.

Reporting of suspected/ confirmed COVID 19 infection

If a patient presents with clinical signs and symptoms consistent with COVID-19 and a test has been performed or requested:

- If test is positive, record "COVID-19 confirmed" in the diagnosis field
- If test is negative, record the SAE signs and symptoms and/or other diagnosis in the diagnosis field.

If a test has not been performed or result is not available and signs and symptoms, as judged by the investigator, are highly suspicious of COVID-19 infection, record "COVID-19 suspected" in the diagnosis field

If other concurrent diagnoses, e.g. pneumonia, please record within the narrative of the SAE.

COVID-19 vaccination and reporting

Where a deployed COVID-19 vaccine is suspected to be involved in the onset of a reported event it should be recorded as a concomitant medication. A causal relationship between the vaccine and the event, including potential drug interactions should be assigned by the reporting investigator.
If a reported event is suspected to be due to a deployed COVID-19 vaccine alone reporting investigators should ensure that standard Yellow Card reporting procedures are followed.

Assessment of Adverse Events

All adverse events must be assessed by the reporting site for seriousness**.** All SAEs must also be assessed for causality and expectedness with reference to this protocol and the Reference Safety Information (RSI).

Assessment of seriousness

An adverse event will be considered serious if it:

1. Results in death
2. Is life threatening
3. Requires hospitalisation or prolongation of existing hospitalisation
4. Results in persistent or significant disability or incapacity
5. Consists of a congenital anomaly or birth defect

1. Required intervention to prevent one of the above.
2. Is a serious Adverse Event of Special Interest.

Assessment of causality i.e. does the event have a "reasonable causal relationship" with trial IMPs. The following categories are used:

- **Yes** - The causal and temporal relationship between the event and the absence of a more likely explanation suggest that the trial IMP is the most likely cause.
- **No** - The event is not considered related to the trial IMPs.

Causality will be assigned for each IMP the participant is randomised to receive.

Should an SAE be considered unrelated to the administration of either trial drug then the causality of the event will be collected taking into consideration other factors, for example concomitant medications or co-morbidities, etc.

Assessment of causality is the responsibility of the Principal Investigator or medically-qualified designee(s) at each site. The CI, their delegate(s), or the Sponsor may provide an opinion regarding causality but cannot downgrade the PI's assessment.

Assessment of expectedness

Should the event be considered to be related to the trial IMP(s), an assessment should be made regarding the expectedness of the reaction, i.e. whether the reaction is a recognised adverse effect of the IMP(s).
.
Expected: consistent with the relevant product information documented in the RSI(s).
Unexpected: not consistent with the relevant product information documented in the RSI(s).

The CI, their delegate(s), or the Sponsor have overall responsibility for assigning expectedness as per the RSI(s) and provide the final assessment of the event. The reporting investigator may provide an opinion on the expectedness of an event and this may be taken into account.

SAEs occurring within the USA and the UK

The US Sponsor is responsible for assigning causality and expectedness for any AEs, SAEs and SUSARs that occur within the USA. Where a SUSAR is identified by the US Sponsor this will be forwarded to the UK Sponsor for review within one working day. Following review by the UK Sponsor, appropriate US SUSARs will be subject to expedited reporting to the MHRA as per UK regulatory requirements. Similarly, where a SUSAR is identified by the UK sponsor, this will forwarded to the US Sponsor for review within two working days.

Recording and reporting of SAEs
All SAEs arising during the clinical trial will be recorded in the eCRF soon as reasonably practicable and in any event within 24hours of first becoming aware of the event. Any follow-up information should also be reported.

If recording in the eCRF is not possible a paper SAE form should be completed:
1. The SAE form is downloaded from www.glasgowctu.org, printed off, completed and signed. The form is then faxed to the Glasgow Clinical Trials Unit Pharmacovigilance (PV) Office on

+44(0)141 357 5588. As an alternative, a scanned copy of the SAE form may be emailed to: pharmacovig@glasgowctu.org. If this website is unavailable a paper copy of the SAE form should be filed in the Investigator Site File at each site pending resolution.
2. If necessary a verbal report can be given by contacting the PV Office on +44(0)141 330 4744. This must be followed up as soon as possible with an electronic or written report.

Reporting to sponsor

All SAEs will be considered reportable to the Sponsor PV office from the time of randomisation until 30 days following permanent discontinuation of all trial IMP(s). SAEs occurring following 30 days post cessation of treatment are not reportable to the Sponsor unless an SAE is considered related to IMP(s)

9.3.3 Suspected Unexpected Serious Adverse Reactions (SUSARs)

Any SAE assessed as related to trial IMP(s) by the PI or delegate, and unexpected (not documented as an expected reaction to the IMP(s) within the RSI(s)) by the CI, their delegate, or the Sponsor, will be classified as a SUSAR and therefore subject to expedited reporting to the Medicines and Healthcare products Regulatory Agency (MHRA) and the Research Ethics Committee (REC). Should the CI disagree with the PI's assessment of causality, both opinions will be provided on the report. Where the CI or UK Sponsor disagrees with the US Sponsor's assessment of expectedness both opinions will also be provided on the report.

The Sponsor will inform the MHRA of all SUSARs that occur within the trial and the REC of all SUSARs that occur within the UK within the required expedited reporting timescales:

- **Fatal or life threatening SUSARs** - no later than 7 days of Sponsor awareness that the case fulfils the criteria for a fatal or life threatening SUSAR, and any follow up information within a further 8 days.
- **All other SUSARs** - no later than 15 days of Sponsor awareness that the case fulfils the criteria for a SUSAR.

The sponsor will report SUSARs to the MHRA via the MHRA eSUSAR reporting system and to REC by email with accompanying CTIMP Safety Report Form.

9.3.4 Unblinding for SUSAR reporting purposes

In the event of a potential SUSAR, the sponsor (but not the investigators) will be unblinded to facilitate appropriate reporting to the MHRA and REC.

9.4 Responsibilities for Safety Reporting and Review

This section details the responsibilities for reporting and reviewing safety information arising from the trial.

Principal Investigator (PI):
- Attendance at the initiation meeting/teleconference
- Training of new members of the trial team in the protocol and its procedures
- Ensuring that the ISF is accurately maintained

- Dissemination of important safety or trial-related information to all stakeholders at their site
- Checking for AEs and ARs when participants attend for treatment/follow-up
- Ensuring that AEs are recorded and reported in line with the requirements of the protocol
- Ensuring that all SAEs are recorded and reported to the Sponsor within 24 hours of becoming aware of the event, and providing further follow-up information as soon as available
- Using medical judgement in assigning causality of SAEs
- Providing an opinion on the expectedness of an SAE with reference to the trial protocol and Reference Safety Information
- Using definitions in this protocol, flagging events of special interest.

Chief Investigator (CI)

- Providing clinical oversight of the safety of patients participating in the trial, including an ongoing review of the risk/benefit
- Providing clinical review of section 4.8 of the representative SmPCs on an annual basis to define the Reference Safety Information for the trial
- Using medical judgement to confirm seriousness and causality of all SARs
- Reviewing expectedness of related SAEs with reference to the trial protocol and Reference Safety Information
- Preparing the clinical sections and final sign-off of the Development Safety Update Report (DSUR)
- Using definitions in this protocol, confirming events of special interest

Sponsor:

- Central data collection and verification of AEs, SAEs, SARs and SUSARs according to the trial protocol
- Reporting safety information to the CI or delegate for the ongoing assessment of the risk / benefit
- Reporting safety information to the independent oversight committees identified for the trial (Independent Data Monitoring Committee (IDMC) and / or Trial Steering Committee (TSC)) according to the Trial Monitoring Plan.
- Expedited reporting of SUSARs to the Competent Authority (MHRA in UK) and REC within required timelines.
- Notifying Investigators of SUSARs that occur within the trial.
- Checking for (annually) and notifying PIs of updates to the Reference Safety Information for the trial.
- Preparing standard tables and other relevant information for the DSUR in collaboration with the CI and ensuring timely submission to the MHRA and REC.

Trial Steering Committee:

In accordance with the Charter for the TSC, periodically reviewing recruitment and the overall progress of the trial and liaising with the IDMC and sponsor regarding safety issues.

Independent Data Monitoring Committee:

In accordance with the Charter for the IDMC, periodically reviewing unblinded safety data in individual cases and to determine patterns and trends of events, or to identify safety issues, which would not be apparent on an individual case basis, reporting concerns to the TSC and sponsor.

9.5 Pregnancy reporting

Pregnancy is not considered an AE unless a negative or consequential outcome is recorded for the mother or child/foetus. If the outcome meets the serious criteria, this would be considered an SAE and must be reported as per SAE reporting procedure above.
Any **pregnancy** occurring in a female trial participant or female partner of a male trial participant who becomes pregnant while participating in the Trial will be reported by the PI (or designee) to the Chief Investigator and the sponsor using the sponsor Pregnancy Reporting Form (available at http://www.glasgowctu.org/) within two weeks of the PI first becoming aware of the pregnancy.
The trial participant will also be followed up to determine the outcome of the pregnancy and follow-up information forwarded to the PV office. Any resulting SAEs should be reported as per SAE reporting procedure above.

9.6 Overdose

Any IMP dose which is not administered in accordance with the Protocol should be reported to the sponsor.
If an SAE is associated with an overdose ensure that the overdose if fully described in the SAE report form.

9.7 Reporting urgent safety measures

If any urgent safety measures are taken the CI/Sponsor will phone the MHRA's Clinical Trial Unit on 020 3080 6456, ideally within 24 hours. This will be followed up no later than 3 days from the date the measures are taken, giving written notice to the MHRA (who will advise the format required) and the relevant REC of the measures taken and the circumstances giving rise to those measures. A substantial amendment must also be submitted to the MHRA.

9.8 The type and duration of the follow-up of participants after adverse events.

Adverse events and reactions will be recorded, reported and followed up in line with this protocol until study completion or for a minimum of 30 days after participant's last dose of the IMP, whichever is later.
Any SUSAR identified will be reported to the Sponsor and to the Regulatory Authorities irrespective of how long after IMP administration the reaction has occurred.

9.9 Development safety update reports

A Development Safety Update Report (DSUR) will be submitted once a year, or on request, to MHRA and REC until the trial is declared ended. The report will be submitted within 60 days of the anniversary of the issue of the Clinical Trials Authorisation for the trial. The DSUR will be prepared by the sponsor (PV Office) in liaison with the CI and submitted by the sponsor (PV Office). The DSUR will include only those SAEs reported to the co-sponsors; that is: SAEs and SUSARs occurring within the UK and SUSARs originating in the USA.

10 STATISTICS AND DATA ANALYSIS

10.1 (a)Sample size calculation

The sample size in Protocol versions 1.0 - 5.0 was as follows: "This is based on our current data showing that in type 1 diabetes, treatment with a GLP-1 agonist, liraglutide (1.2 and 1.8mg doses) for three months leads to HbA1c suppression by an average of 0.6% (SD=0.5%). Dapagliflozin was recently shown to reduce HbA1c by 0.45% in type 1 diabetes patients (13). Additionally, empagliflozin treatment for 8 weeks was shown to reduce HbA1c by 0.4% in type 1 diabetics (20, 21) and our

current data showing additional improvement in HbA1c by an additional 0.66% at 12 weeks when dapagliflozin was added to liraglutide and insulin therapy. Conservatively estimating that the combination therapy will suppress HbA1c by 0.5% (SD≤0.7%) compared to semaglutide alone, a sample size of 38 (32 evaluable patients) in each group will be sufficient to provide 80% power at a 5% significance level, taking in consideration a 15% dropout rate. 50% of participants will be recruited in Glasgow, UK and 50% at State University of New York at Buffalo, USA."

10.1 (b) Revised sample size calculation (from Protocol version 6.0)

At their meeting in May 2023, the TSC asked the trial statisticians to review the sample size required on the basis of blinded review of primary outcome data (SD of change of HbA1c, correlation of HbA1c at baseline and end of Period 2). In August 2023 the trial statisticians recommended on the basis of this review a reduction in the sample size by a factor of 0.59 i.e. from 32 to 19 evaluable patients per group. This recommendation was reviewed by the IDMC in August 2023, by the TSC in September 2023 and at an additional IDMC meeting in October 2023 (at which additional information was provided on changes in insulin delivery devices during the trial). At this time, the IDMC unanimously agreed that the study should continue, aiming conservatively for a revised sample size in Period 2 of around 23/ evaluable participants per group for dapagliflozin vs placebo. This reduces the recruitment target for first randomisation from n=114 to n=82 (i.e. 23/32*114) maintaining the same assumptions on power, significance level and drop-out (amended accordingly on page 7 above).

The IDMC further required a formal interim analysis on all those with mature data on the primary outcome at around the end of April 2024 with recruitment to continue unless formally stopped by the funder following a subsequent recommendation by the TSC. The study should continue after interim analysis: (a) unless there is overwhelming evidence of benefit, or evidence of futility; and (b) if the study has recruited sufficient numbers between Nov 23 – Apr 24 to make reaching the new target in a reasonable time feasible within budget (minimum 8-10 new participants recruited)). The IDMC agreed to specify the exact detail of the interim analysis in time for this analysis to be conducted, to review the results and make a recommendation to the TSC.

This recommendation was accepted unanimously by the independent members of the TSC in November 2023.

10.2 Anticipated recruitment rate

Originally one year (one participant per week). The rate has been considerably slower due mainly to the COVID-19 pandemic and global semaglutide supply issues. 60 participants had been randomised by November 2023.

10.3 Statistical analysis

The primary objective of the proposed research is to evaluate the effect of addition of dapagliflozin to semaglutide on a background of insulin treatment in type 1 diabetes patients on HbA1c at six months following start of dapagliflozin. Transformations of the data in order to meet statistical assumptions will be considered. Statistical analyses will use linear regression models to compare treatment groups, with adjustment for baseline values of study outcomes, as well as factors used to stratify the randomisation scheme. Tests will be two-sided and tested at the 5% significance level. Analyses will be carried out under the intention to treat principle, that is, according to randomised treatment allocation, regardless of treatment adherence.

A detailed Statistical Analysis Plan will be completed and signed off before database lock. Study statisticians will develop and validate the analysis programs without access to randomised treatment

allocations. After database lock, the treatment codes will be released and the final analysis will be carried out.

10.4 Subgroup analyses

These will be specified in the Statistical Analysis Plan. Subgroup analyses will be implemented by extending regression models to include interactions between treatment and potential treatment effect moderators.

10.5 Interim analysis and criteria for the premature termination of the trial

No interim analysis was initially planned but an interim analysis was recommended by the IDMC in November 2023 – to be conducted in April 2024 (see section 10.1(b) above). Unblinded trial data will be reviewed on an ongoing basis by the IDMC. The primary role of the IDMC will be to protect the interests of the patients. The IDMC may recommend to the TSC and Co-Sponsors that the study should stop prematurely because of concerns about patient safety. The IDMC will meet approximately every six months. The IDMC will take into account all results and the consistency and biological plausibility of the findings in making any recommendation. The final decision on continuing or stopping the trial will lie with the TSC/Co-Sponsors.

10.6 Subject population

All analyses will be carried out on an intention to treat basis, using the randomised treatment allocation.

10.7 Procedure(s) to account for missing or spurious data

Data will be analysed without imputation in the first instance. Multiple imputation procedures will be used to assess the sensitivity of selected results to missing data, according to the Statistical Analysis Plan.

10.8 Other statistical considerations.

The Statistical Analysis Plan (SAP) will be maintained as a version controlled document and will be signed off before database lock. The SAP and other statistical documentation (according to Glasgow Clinical Trials Unit Standard Operating Procedures) will contain full details of all analyses along with assumptions and procedures for handling problematic or incomplete data (e.g. incomplete dates).

11 DATA HANDLING

11.1 Source Documentation

ICH GCP defines source data as: 'All information in original records and certified copies of original records of clinical findings, observations or other activities in a clinical trial necessary for the reconstruction and evaluation of the trial'. In this study, the location of the majority of the source data will be the hospital's medical records including subject case notes, laboratory records and ECGs. The source data transcribed into the eCRF from the medical records must be accurate and verifiable. For questionnaires completed by trial subjects, the completed questionnaires will be regarded as the source data location. In cases where data are transcribed directly into the eCRF and no other paper or electronic source exists, then the eCRF will be considered the source record. In these cases, these data should be prospectively documented in the medical records to ensure a full record of the trial is available at site. Identification of unverifiable data entries will be reported as non-compliances.

11.2 Data collection

An eCRF, developed by the Robertson Centre for Biostatistics, will capture all data required to meet this protocol's requirements. Access to the eCRF will be restricted, via a study-specific web portal, and only authorised site-specific personnel will be able to make entries to their patients' data via the web portal. The Investigator, or his/her designee will be responsible for all entries into the eCRF and will confirm that the data are accurate, complete and verifiable. Data will be stored in a MS SQL Server database.

Direct access to the web portal will be granted, on request, to authorised representatives from the Sponsor, host institution and the regulatory authorities to permit trial-related monitoring, audits and inspections.

11.3 Data Validation

Where it is practical, data will be validated at the point of entry into the eCRF. Any additional data discrepancies will be flagged to the investigator and any data changes will be recorded to maintain a complete audit trail (reason for change, date change made, who made change).

11.4 Data Security

The Robertson Centre for Biostatistics systems are fully validated in accordance with industry and regulatory standards, and incorporate controlled access security. High volume servers are firewall protected and preventative system maintenance policies are in place to ensure no loss of service or data. Web servers are secured by digital certificates. Data integrity is assured by strictly controlled procedures, including secure data transfer procedures. Data are backed up on-site nightly and off-site to a commercial data vault weekly. The Robertson Centre for Biostatistics has an ISO 9001:2008 quality management system and ISO 27001:2013 for Information Security, and is regularly inspected against the standards by the British Standards Institution.

11.5 Archiving

The Trial Master File will be archived by the Co-Sponsors at the end of the trial for a minimum period of five years.

Archiving of Site Files will also be for a minimum of seven years from completion of the trial, and this action will be delegated to the sites in the Clinical Trial Site Agreement that will be put in place between Co-Sponsors and Sites. Sites will be notified by the Co-Sponsors when Site files can be archived.

Destruction of site files can only take place with the approval of the Co-Sponsors.

12 MONITORING, AUDIT & INSPECTION

Monitoring will be conducted by NHS Greater Glasgow and Clyde (GG&C) Monitor(s) in accordance with local Standard Operating Procedures. The level, frequency, objectives and priorities of monitoring will be based on the outcome of the completed Risk Assessment, and clearly documented in the Monitoring Plan which will be approved by the members of the TMG and the NHS GG&C Lead Monitor and/or Research Governance Manager. The Monitoring Plan is subject to updates during the life of the study in line with Protocol Amendments.

13 ETHICAL AND REGULATORY CONSIDERATIONS

13.1 Research Ethics Committee (REC) review & reports

Before the start of the trial, approval will be sought from a REC for the trial protocol, informed consent forms and other relevant documents e.g. advertisements and GP information letters.
Substantial amendments that require review by REC will not be implemented until the REC grants a favourable opinion for the study (it is noted that amendments may also need to be reviewed and accepted by the MHRA and/or NHS R&D departments before they can be implemented in practice at sites).
All correspondence with the REC will be retained in the Trial Master File/Investigator Site File.
An annual progress report (APR) will be submitted to the REC within 30 days of the anniversary date on which the favourable opinion was given, and annually until the trial is declared ended (this is the Chief Investigator's responsibility).
The Chief Investigator will notify the REC of the end of the study.
If the study is ended prematurely, the Chief Investigator will notify the REC, including the reasons for the premature termination
Within one year after the end of the study, the Chief Investigator will submit a final report with the results, including any publications/abstracts, to the REC.

13.2 Peer review

The study protocol has been developed with expert and independent feedback from the JDRF (New York) and during application for funding underwent peer review by independent experts.

13.3 Public and Patient Involvement

The study was designed with input from people with type 1 diabetes via the funder, JDRF.

13.4 Regulatory Compliance

The trial will not commence until a Clinical Trial Authorisation (CTA) is obtained from the MHRA.
The protocol and trial conduct will comply with the Medicines for Human Use (Clinical Trials) Regulations 2004 and any relevant amendments.
Before any site can enrol patients into the trial, the Chief Investigator/Principal Investigator or designee will apply for NHS permission from the site's Research & Development (R&D) department.

For any amendment that will potentially affect a site's NHS permission, the Chief Investigator/ Principal Investigator or designee will confirm with that site's R&D department that NHS permission is ongoing (note that both substantial amendments, and amendments considered to be non-substantial for the purposes of REC and/or MHRA may still need to be notified to NHS R&D).

13.5 Protocol compliance

Prospective, planned deviations or waivers to the protocol are not allowed under the UK regulations on Clinical Trials and must not be used e.g. it is not acceptable to enrol a participant if they do not meet the eligibility criteria or restrictions specified in the trial protocol. Accidental protocol deviations can happen at any time. They must be adequately documented on the relevant forms and reported to the Chief Investigator, Sponsor and GCTU immediately. Deviations from the protocol which are found to frequently recur are not acceptable, will require immediate action and could potentially be classified as a serious breach.

13.6 Notification of Serious Breaches to GCP and/or the protocol

A "serious breach" is a breach which is likely to affect to a significant degree –

1. the safety or physical or mental integrity of the subjects of the trial; or
2. the scientific value of the trial

If any of the above occurs then the CI and Sponsor will be notified. The sponsor will notify the appropriate authorities in writing of any serious breach in accordance with their standard operating procedures.

13.7 Data protection and patient confidentiality

All investigators and trial site staff must comply with the requirements of the GDPR with regards to the collection, storage, processing and disclosure of personal information and will uphold the Act's core principles.

- Only those individuals who require to see personal information i.e. site research team staff or the study monitor, as appropriate, will be able to view them. All electronic data will be held securely in accordance with ISO 27001:2013 at the Robertson Centre for Biostatistics, part of the Glasgow Clinical Trials Unit. All Centre staff are required to sign confidentiality agreements and to follow Standard Operating Procedures in accordance with Good Clinical Practice and ISO certification.
- The trial data managers, statisticians, health economists or any other staff who will perform data related tasks will only be able to access depersonalised data where the participant's identifying information is replaced by a unique study identifier.
- Only those that have been trained and approved will be able to enter or view any data via the web portal. Each site can only see their own patients' data. Patient consent forms will be stored at the study site in a secure location accessible only to study teams.

13.8 Indemnity

The Co-Sponsors (University of Glasgow and Greater Glasgow Health Board) will ensure that provision has been made for insurance or indemnity to cover the liability of the investigator and sponsor which may arise in relation to the clinical trial in accordance with Part 2 (14) of Schedule 1 to SI 2004/1031.

13.9 Amendments

Any change in the study protocol will require an amendment. Any proposed protocol amendments will be initiated by the CI following discussion with the Sponsor and TSC and any required amendment forms will be submitted to the regulatory authority, ethics committee and Sponsor. The Sponsor will determine whether an amendment is non-substantial or substantial. All amended versions of the protocol will be signed by the CI and Sponsor representative. Following a substantial amendment, favourable opinion/approval must be sought from the original reviewing REC, MHRA (where appropriate) and Research and Development (R&D) office prior to implementation. The Chief Investigator will be responsible for informing the Trial Management Group of all protocol amendments.

13.10 Post trial care

At the end of the trial, participants will be returned to usual care as defined by local and national guidelines at that time. The results of the trial may of course have an impact on these guidelines and the future care of patients with type 1 diabetes.

13.11 Access to the final trial dataset

During the trial and in the period prior to publication of the main study results as described in the protocol, only the Glasgow CTU, Professor Paresh Dandona and Dr Husam Ghanim US Principal Investigators, University of Buffalo will have access to the full dataset. After that period, the trial Steering Committee will conduct further data analyses for a period of three years. After that time the Trial Steering Committee will consider requests from external parties for further analyses of the study data. Proposals that are scientifically well founded and have an academic basis and where relevant data extractions and analyses are appropriately funded will not be refused. These will be considered as collaborative exercises where the contributions related to study design, conduct, database creation and maintenance and data analysis will be recognised in authorship of any scientific publication. The approach we will take will be to minimise any possibility of breach of participant confidentiality. Normally this will be achieved by minimising data travel. However, for the purposes of individual patient meta-analysis and other reasons, data may be transferred to other sites. Such transfer will require assurances on information security systems at the sites that data are to be transferred to and will involve a legal data transfer agreement. A log of all data requests and subsequent data transfers will be held at the Glasgow CTU.

14 DISSEMINATION POLICY

14.1 Dissemination policy

The study database will be owned by the University of Glasgow and maintained on behalf of the Study investigators, represented by the Trial Steering Committee as it is constituted during and after the trial.

The study protocol and a description of the recruitment experience and participant baseline characteristics will be published before study completion. On completion of the trial, the database will be locked and analysed by staff of the Robertson Centre for Biostatistics, University of Glasgow. A final study report will be prepared and the results will be published in a major medical journal.

After the main study publications, study investigators will be invited to submit requests for further analyses of the study database. These will be reviewed and prioritised by a Publications Committee made up of the study grant holders and convened by the study co-CIs.

As funder of the trial, JDRF will have the right to see and comment on any results being submitted for publication. A maximum of 28 days will be given for review of major papers and 14 days for abstracts. Such comments will be considered by the Trial Steering Committee.

A lay summary of the main results of the trial will be prepared and provided to all participants via their study site investigators. Investigators may request a copy of the study data for their participants. Providing some or all of a patient's data to that individual is at their discretion.

14.2 Authorship eligibility guidelines and any intended use of professional writers

The main results of the study will be compiled, written up and published by the study grant holders and others taking responsibility for the study results (e.g. the statistician conducting the final analysis) on behalf of the TTT1 investigators. The TTT1 investigators will be listed in an Appendix and will include all site PIs, all committee members and key members of relevant study coordinating groups (including the Sponsor and Glasgow CTU).

15 REFERENCES

1. Konrad K, Datz N, Engelsberger I, Grulich-Henn J, Hoertenhuber T, Knauth B, et al. Current use of metformin in addition to insulin in pediatric patients with type 1 diabetes mellitus: an analysis based on a large diabetes registry in Germany and Austria. *Pediatr Diabetes.* 2014.
2. Vella S, Buetow L, Royle P, Livingstone S, Colhoun HM, and Petrie JR. The use of metformin in type 1 diabetes: a systematic review of efficacy. *Diabetologia.* 2010;53(5):809-20.
3. Vella S, Buetow L, Royle P, Livingstone S, and Petrie JR. Metformin in type 1 diabetes reduces insulin requirements without significantly improving glycaemic control. Reply to Schatz H [letter]. *Diabetologia.* 2011;54(1):203-4.
4. Varanasi A, Bellini N, Rawal D, Vora M, Makdissi A, Dhindsa S, et al. Liraglutide as additional treatment for type 1 diabetes. *Eur J Endocrinol.* 2011;165(1):77-84.
5. Kuhadiya ND, Malik R, Bellini NJ, Patterson JL, Traina A, Makdissi A, et al. Liraglutide as additional treatment to insulin in obese patients with type 1 diabetes mellitus. *Endocrine practice : official journal of the American College of Endocrinology and the American Association of Clinical Endocrinologists.* 2013;19(6):963-7.
6. Kuhadiya ND, Dhindsa S, Ghanim H, Mehta A, Makdissi A, Batra M, et al. Addition of Liraglutide to Insulin in Patients With Type 1 Diabetes: A Randomized Placebo-Controlled Clinical Trial of 12 Weeks. *Diabetes Care.* 2016;39(6):1027-35.
7. Chaudhuri A, Ghanim H, Makdissi A, Green K, Abuaysheh S, Batra M, et al. Exenatide induces an increase in vasodilatory and a decrease in vasoconstrictive mediators. *Diabetes Obes Metab.* 2017;19(5):729-33.
8. Mathieu C, Zinman B, Hemmingsson JU, Woo V, Colman P, Christiansen E, et al. Efficacy and Safety of Liraglutide Added to Insulin Treatment in Type 1 Diabetes: The ADJUNCT ONE Treat-To-Target Randomized Trial. *Diabetes Care.* 2016;39(10):1702-10.
9. Ahren B, Hirsch IB, Pieber TR, Mathieu C, Gomez-Peralta F, Hansen TK, et al. Efficacy and Safety of Liraglutide Added to Capped Insulin Treatment in Subjects With Type 1 Diabetes: The ADJUNCT TWO Randomized Trial. *Diabetes Care.* 2016;39(10):1693-701.
10. Zinman B, Wanner C, Lachin JM, Fitchett D, Bluhmki E, Hantel S, et al. Empagliflozin, Cardiovascular Outcomes, and Mortality in Type 2 Diabetes. *N Engl J Med.* 2015;373(22):2117-28.
11. Petrie JR. SGLT2 inhibitors in type 1 diabetes: knocked down, but up again? *Lancet Diabetes Endocrinol.* 2017;5(11):841-3.
12. Mudaliar S, Armstrong DA, Mavian AA, O'Connor-Semmes R, Mydlow PK, Ye J, et al. Remogliflozin etabonate, a selective inhibitor of the sodium-glucose transporter 2, improves serum glucose profiles in type 1 diabetes. *Diabetes Care.* 2012;35(11):2198-200.
13. Kohan DE, Fioretto P, Tang W, List JF. Long-term study of patients with type 2 diabetes and moderate renal impairment shows that dapagliflozin reduces weight and blood pressure but does not improve glycemic control. Kidney Int. 2014 Apr;85(4):962-71. doi: 10.1038/ki.2013.356. Epub 2013 Sep 25. PMID: 24067431; PMCID: PMC3973038.
14. Dandona P, Mathieu C, Phillip M, Hansen L, Griffen SC, Tschope D, et al. Efficacy and safety of dapagliflozin in patients with inadequately controlled type 1 diabetes (DEPICT-

1): 24 week results from a multicentre, double-blind, phase 3, randomised controlled trial. *Lancet Diabetes Endocrinol.* 2017;5(11):864-76.

15. Mathieu C, Dandona P, Gillard P, Senior P, Hasslacher C, Araki E, Lind M, Bain SC, Jabbour S, Arya N, Hansen L, Thorén F, Langkilde AM; DEPICT-2 Investigators. Efficacy and Safety of Dapagliflozin in Patients With Inadequately Controlled Type 1 Diabetes (the DEPICT-2 Study): 24-Week Results From a Randomized Controlled Trial. Diabetes Care. 2018 Sep;41(9):1938-1946.

16. European Medicines Agency. Assessment Report Dapagliflozin. https://www.ema.europa.eu/en/documents/variation-report/forxiga-h-c-ws-1941-epar-assessment-report-variation_en.pdf *(accessed 11th November 2021)*

17. Groop PH, Dandona P, Phillip M, Gillard P, Edelman S, Jendle J, Xu J, Scheerer MF, Thoren F, Iqbal N, Repetto E, Mathieu C. Effect of dapagliflozin as an adjunct to insulin over 52 weeks in individuals with type 1 diabetes: post-hoc renal analysis of the DEPICT randomised controlled trials. Lancet Diabetes Endocrinol. 2020 Oct;8(10):845-854. doi: 10.1016/S2213-8587(20)30280-1. PMID: 32946821.

18. Rosenstock J, Marquard J, Laffel LM, et al. Empagliflozin as adjunctive to insulin therapy in type 1 diabetes: the EASE trials. *Diabetes Care*. 2018;41(12):2560–2569.

19. Garg SK, Henry RR, Banks P, Buse JB, Davies MJ, Fulcher GR, et al. Effects of Sotagliflozin Added to Insulin in Patients with Type 1 Diabetes. *N Engl J Med.* 2017;377(24):2337-48.

20. European Medicines Agency. First oral add-on treatment to insulin for treatment of certain patients with type 1 diabetes. https://www.ema.europa.eu/en/news/first-oral-add-treatment-insulin-treatment-certain-patients-type-1-diabetes *(accessed 11th November 2021)*

21. An overview of Zynquista and why it is authorised in the EU. 16th May 2019. https://www.ema.europa.eu/en/documents/overview/zynquista-epar-medicine-overview_en.pdf *(accessed 11th November 2021)*

22. National Institute for Health and Care Excellence. Dapagliflozin with insulin for treating type 1 diabetes. Technology appraisal guidance [TA597] https://www.nice.org.uk/guidance/ta597

23. AstraZeneca Press Release. Update on US regulatory decision for Farxiga in type-1 diabetes. https://www.astrazeneca.com/media-centre/press-releases/2019/update-on-us-regulatory-decision-for-farxiga-in-type-1-diabetes-15072019.html# *(accessed 11th November 2021)*

24. European Medicines Agency. Forxiga (dapagliflozin) 5mg should no longer be used for the treatment of Type 1 Diabetes Mellitus. https://www.ema.europa.eu/en/documents/dhpc/direct-healthcare-professional-communication-dhpc-forxiga-dapagliflozin-5mg-should-no-longer-be-used_en.pdf *(accessed 11th November 2021)*

25. AstraZeneca. Letter to UK Health Professionals. Forxiga (dapagliflozin) 5mg should no longer be used for the treatment of type 1 diabetes mellitus. (2nd November 2021)

26. Marso SP, Bain SC, Consoli A, Eliaschewitz FG, Jódar E, Leiter LA, Lingvay I, Rosenstock J, Seufert J, Warren ML, et al. Semaglutide and cardiovascular

outcomes in patients with type 2 diabetes. *N Engl J Med.* 2016;**375**(19):1834–44. doi: 10.1056/NEJMoa1607141.

27. Ozempic Summary of product characteristics. [Ozempic 0.5 mg solution for injection in pre-filled pen - Summary of Product Characteristics (SmPC) - (emc) (medicines.org.uk)]

28. Nauck MA, Petrie JR, Sesti G, Mannucci E, Courrèges JP, Lindegaard ML, Jensen CB, Atkin SL; Study 1821 Investigators. A Phase 2, Randomized, Dose-Finding Study of the Novel Once-Weekly Human GLP-1 Analog, Semaglutide, Compared With Placebo and Open-Label Liraglutide in Patients With Type 2 Diabetes. Diabetes Care. 2016 Feb;39(2):231-41.

29. Forxiga 5 mg Summary of Product Characteristics: https://www.medicines.org.uk/emc/product/2865/smpc *(accessed 11th November 2021)*

30. Wiviott SD, Raz I, Bonaca MP, Mosenzon O, Kato ET, Cahn A, Silverman MG, Zelniker TA, Kuder JF, Murphy SA, Bhatt DL, Leiter LA, McGuire DK, Wilding JPH, Ruff CT, Gause-Nilsson IAM, Fredriksson M, Johansson PA, Langkilde AM, Sabatine MS; DECLARE–TIMI 58 Investigators. Dapagliflozin and Cardiovascular Outcomes in Type 2 Diabetes. N Engl J Med. 2019 Jan 24;380(4):347-357.

31. McMurray JJV, Solomon SD, Inzucchi SE, Køber L, Kosiborod MN, Martinez FA et al. DAPA-HF Trial Committees and Investigators Dapagliflozin in patients with heart failure and reduced ejection fraction. N Engl J Med. 2019. Nov 21;381(21):1995-2008.

32. Heerspink HJL, Stefánsson BV, Correa-Rotter R, Chertow GM, Greene T, Hou FF, Mann JFE, McMurray JJV, Lindberg M, Rossing P, Sjöström CD, Toto RD, Langkilde AM, Wheeler DC; DAPA-CKD Trial Committees and Investigators. Dapagliflozin in Patients with Chronic Kidney Disease. N Engl J Med. 2020 Oct 8;383(15):1436-1446. doi: 10.1056/NEJMoa2024816. Epub 2020 Sep 24. PMID: 32970396.

33. Vilsbøll T, Bain SC, Leiter LA, et al. Semaglutide, reduction in glycated haemoglobin and the risk of diabetic retinopathy. *Diabetes Obes Metab*. 2018;20(4):889–897.

34. Lingvay I, Desouza CV, Lalic KS et al. A 25 week randomized controlled trial of semaglutide once daily versus liraglutide and placebo in patients with type 2 diabetes suboptimally controlled on diet and exercise with or without metformin. Diabetes Care. 2018;41(9): 1926-1937.

35. European Medicines Agency. Assessment Report Sotagliflozin. https://www.ema.europa.eu/en/documents/assessment-report/zynquista-epar-public-assessment-report_en.pdf *(accessed 11th November 2021)*

36. Silverii GA, Decembrini L, Monami M, Mannucci E. Fournier's gangrene and sodium glucose co-transporter 2 inhibitors: A metanalysis of randomized controlled trials. Diabetes Obes Metab. 2019; 1-4.

37. Garg SK, Peters AL, Buse JB, Danne T. Strategy for mitigating DKA risk in patients with Type 1 diabetes on adjunctive treatment with SGLT-2 inhibitors: A STICH Protocol. Diabetes Technol Ther. 2018;20(9):571-575.

38. Peters AL, Buschur EO, Buse JB, Cohan P, Diner JC, and Hirsch IB. Euglycemic Diabetic Ketoacidosis: A Potential Complication of Treatment With Sodium-Glucose Cotransporter 2 Inhibition. Diabetes Care. 2015;38(9):1687-93.

39. Agiostratidou G, Anhalt H, Ball D, Blonde L, Gourgari E, Harriman KN, Kowalski AJ, Madden P, McAuliffe-Fogarty AH, McElwee-Malloy M, Peters A, Raman S, Reifschneider

K, Rubin K, Weinzimer SA. Standardizing Clinically Meaningful Outcome Measures Beyond HbA(1c) for Type 1 Diabetes: A Consensus Report of the American Association of Clinical Endocrinologists, the American Association of Diabetes Educators, the American Diabetes Association, the Endocrine Society, JDRF International, The Leona M. and Harry B. Helmsley Charitable Trust, the Pediatric Endocrine Society, and the T1D Exchange. Diabetes Care. 2017 Dec;40(12):1622-1630

16. APPENDICES

16.1 Appendix 1 – Risk

Risks associated with trial interventions<br>☐ LOW ≡ Comparable to the risk of standard medical care<br>☒ MODERATE ≡ Somewhat higher than the risk of standard medical care<br>☐ HIGH ≡ Markedly higher than the risk of standard medical care				
Justification: Briefly justify the risk category selected and your conclusions below (where the table is completed in detail the detail need not be repeated, however a summary should be given):<br>An IDMC will be convened to monitor all SAEs.<br>Risks and mitigations associated with the intervention are outlined in more detail in the Protocol section 2.1.<br>Co-Sponsors will also carry out a detailed risk assessment of all aspects of the study as part of the approval process (SOP 04.013)				
What are the key risks related to therapeutic interventions you plan to monitor in this trial?		How will these risks be minimised?		
IMP/Intervention	Body system/Hazard	Activity	Frequency	Comments
S/C administration of semaglutide<br>Oral admistration of dapagliflozin	Immune: Hypersensitivity/ana-phylactic reactions	Patients with known hypersensitivity to any GLP-1 agonist or SGLT-2 inhibitor have a contra-indication to the IMP according to the SmPC will not be recruited to the study	Uncommon/ Rare	
PO administration of dapagliflozin	Ketosis and/or Diabetic Ketoacidosis (DKA)	All participants will have access to blood or urine ketone monitoring equipment. An emergency ketosis management plan (STICH Protocol – see above) will be discussed with all participants. Patient alert cards will be provided to all participants. Potential	Common	“and” was an error – intention was for “either” blood or urine ketones between visits but “both” to be measured for visits. This is compatible with the eCRF, the diaries and the rest of the protocol

		participants with recent ketoacidosis will be excluded from the trial (section 6.2)		
S/C administration of semaglutide (+/-) PO administration of dapagliflozin in addition to insulin therapy	Increased risk of hypoglycaemia	Participant awareness of risk and frequent testing of capillary blood glucose. Patients with pre-existing frequent episodes of hypoglycaemia or problems with hypoglycaemia awareness will be excluded from trial participation (section 6.2)	Common	

Outline any other processes that have been put in place to mitigate risks to participant safety (e.g. IDMC, independent data review, etc.)
See above

16.2 Appendix 2: Contraception

In line with recommendations related to contraception and pregnancy testing in clinical trials from the Clinical Trial Facilitation Group (CTFG), for women of childbearing potential* (WOCBP) participating in TTT1, methods of contraception considered as highly effective are defined as those that can achieve a failure rate of less than 1% per year when used consistently and correctly.

Such methods include:

- combined (oestrogen and progestogen containing) hormonal contraception associated with inhibition of ovulation[1]
  - oral
  - intravaginal
  - transdermal
- progestogen-only hormonal contraception associated with inhibition of ovulation[1]
  - oral
  - injectable
  - implantable[2]
- intrauterine device (IUD) [2]
- intrauterine hormone-releasing system (IUS)[2]
- bilateral tubal occlusion[2]
- vasectomised partner[2,3]
- true sexual abstinence[4]

Footnotes

1. An interaction between dapagliflozin and semaglutide with contraception is not anticipated.
2. Contraception methods that in the context of this guidance are considered to have low user dependency.
3. Vasectomised partner is a highly effective birth control method provided that partner is the sole sexual partner of the WOCBP trial participant and that the vasectomised partner has received medical assessment of the surgical success.
4. In the context of this guidance true sexual abstinence refers to: When this is in line with the preferred and usual lifestyle of the patient. Periodic abstinence (e.g. calendar, ovulation, symptothermal, post-ovulation methods), declaration of abstinence for the duration of a trial, and withdrawal are not acceptable methods of contraception.
The reliability of sexual abstinence needs to be evaluated in relation to the duration of the clinical trial and the preferred and usual lifestyle of the subject.

*Child bearing potential = experienced menarche; AND not undergone successful surgical sterilisation (hysterectomy, bilateral tubal ligation, or bilateral oophorectomy); AND not post-menopausal i.e. amenorrhea for ≥12 consecutive months (without another medical cause)

Source: Recommendations related to contraception and pregnancy testing in clinical trials. Clinical Trial Facilitation Group (CTFG), 15th September 2014.
See: https://www.hma.eu/fileadmin/dateien/Human_Medicines/01-About_HMA/Working_Groups/CTFG/2014_09_HMA_CTFG_Contraception.pdf (Accessed 21st January 2020)

16.3 Appendix 3

STICH Protocol *(also provided in Period 2 Participant Diary)*

As explained above, participants in this trial randomised to dapagliflozin are at an increased risk of ketosis and ketoacidosis. All participants will have access to a ketone meter as standard of care. It will be ascertained that each participant has access to a ketone meter; if there are any issues with their current ketone meter it will be replaced as per standard of care. There are mechanisms designed to mitigate the risk of early ketosis developing further into diabetic ketoacidosis. In this trial, we will be using a protocol called STICH (37). This is a mnemonic designed to act as a reminder for participants as to what to do in the event of elevated ketones following exposure to an SGLT-2 drug such as dapagliflozin:

1. STop dapagliflozin or placebo temporarily
2. Insulin – take a bolus of rapid acting insulin
3. Carbohydrate – take approximately 30g of carbohydrate
4. Hydrate – make sure you are taking sips of water aiming to maintain hydration.

Further guidance will be provided in both the Patient Diaries supplied to all participants and in the Patient Alert Cards that participants will be asked to carry at all times. All participants will also be advised that in the event of acute intercurrent illness or hospitalisation, dapagliflozin or placebo should be temporarily stopped. This is because intercurrent illness may additionally increase the risk of ketosis. Participants will likely already be familiar with this concept of "sick-day rules" as this is a key component of basic education for people with type 1 diabetes in clinical practice. These rules will however be re-iterated to participants.

More detailed guidance on actions participants should take for any given level of ketosis will be provided during face-to-face and telephone contact during the trial. In addition, however, the following written protocol will be provided to participants to advise them explicitly of the safest and most appropriate course of action to be taken in any given circumstance. This will take the form of a traffic light system to aid understanding and compliance.

1. Ketones 0.6 mmol/L or less: Continue - no need for additional bolus insulin.
2. Ketones 0.6 – 1.4 mmol/L: consider taking additional fast acting insulin. Follow sick day rules and monitor ketones 2 hourly until they are 0.6 mmol/L or less.
3. Ketones 1.5 – 2.9 mmol/L – Take 10% of your total daily dose of insulin (this is found by adding up all the long acting and short acting insulin for the previous day - only take a maximum of 10 units) as a bolus of fast acting insulin. Keep hydrated - aim for 100ml water per hour. Re-test your glucose and ketones in 2 hours. If not falling contact the study team during office hours. If you need urgent advice at other times please contact NHS 24 (telephone number 111) or attend your nearest Accident & Emergency Department.
4. Ketones above 3 mmol/L – Take 20% of your total daily insulin as fast acting insulin (Maximum 10 units). Hydrate with water, aim for 500 ml per hour. Attend A&E – High risk of DKA

If you develop DKA or are unwell it is important that you let the TTT1 nurse or doctor know about this as soon as you can. By following this protocol, the risk of ketosis and developing ketoacidosis will be

mitigated.

16.4 Appendix 4: Amendment History

Sponsor Amendment No.	Protocol version no.	Date issued	Author(s) of changes	Details of changes made
AM02	2.0	Ethics App – 23 Nov 2020		Protocol Changes: Weight loss mitigation, 19 Risk Mitigation and visit activity corrections
AM03	2.1	Ethics App – 10th Feb 2021		Correction of Summary of changes for previous AM02.<br>Corrected pregnancy testing schedule.
AM06	3.0	Ethics App – 16th Feb 2022		Definition of AESIs narrowed to include only ketosis and level 2 hypoglycaemia; lowering eGFR inclusion criterion; correction of DSQOLS to licensed version.
AM08	4.0	Ethics App – 26th June 2023		Procedure for recruitment at other sites within NHS GGC described; procedures for rescreening of participants experiencing delay between the screening visit and Visit 1; updates; correction of errors
AM09	5.0	Not required		Following discussion at TMG (21st Sept) and Sponsor reps (26th Sept), it was agreed it was appropriate to remove the word "Summary" and addition of text to avoid duplication of sending PIS twice, from page 33 of the protocol. (Recruitment section). While the Summary PIS was produced and approved during the study set up, in practice it has not been required, with telephone calls and full PIS being sent (following initial discussion in clinics) being the preferred method. For this reason the Summary PIS has not been updated in line with the protocol amendments and is now out of date, therefore is not appropriate to be sent.
AM11	6.0	Ethics App – 21st Mar 2024		Clarification to the recruitment process, Updated information of interim analysis, now planned. Changes to further align with US protocol with regard withdrawals.
AM12	7.0	Awaited		Protocol and PIS updated in line with recent Semaglutide SmPC.<br>Re the protocol, a number of points of clarification and correction of errors in text have been included. These are listed in the

				Summary of Protocol Changes document submitted with this application.<br>The PIS has also been updated re the SmPC update and again a number of clarifications/ corrections have also been included.

List details of all protocol amendments here whenever a new version of the protocol is produced. Protocol amendments must be submitted to the Sponsor for approval prior to submission to the REC committee or MHRA.

16.5 Appendix 5: PAID Questionnaire

Problem Areas In Diabetes (PAID) scale

Instructions: Which of the following diabetes issues are **currently** a problem for you? Tick the box that gives the best answer for you. Please provide an answer for each question.

		Not a problem	Minor problem	Moderate problem	Somewhat serious problem	Serious problem
1	Not having clear and concrete goals for your diabetes care?	☐ 0	☐ 1	☐ 2	☐ 3	☐ 4
2	Feeling discouraged with your diabetes treatment plan?	☐ 0	☐ 1	☐ 2	☐ 3	☐ 4
3	Feeling scared when you think about living with diabetes?	☐ 0	☐ 1	☐ 2	☐ 3	☐ 4
4	Uncomfortable social situations related to your diabetes care (e.g. people telling you what to eat)?	☐ 0	☐ 1	☐ 2	☐ 3	☐ 4
5	Feelings of deprivation regarding food and meals?	☐ 0	☐ 1	☐ 2	☐ 3	☐ 4
6	Feeling depressed when you think about living with diabetes?	☐ 0	☐ 1	☐ 2	☐ 3	☐ 4
7	Not knowing if your mood or feelings are related to your diabetes?	☐ 0	☐ 1	☐ 2	☐ 3	☐ 4
8	Feeling overwhelmed by your diabetes?	☐ 0	☐ 1	☐ 2	☐ 3	☐ 4
9	Worrying about low blood glucose reactions?	☐ 0	☐ 1	☐ 2	☐ 3	☐ 4
10	Feeling angry when you think about living with diabetes?	☐ 0	☐ 1	☐ 2	☐ 3	☐ 4
11	Feeling constantly concerned about food and eating?	☐ 0	☐ 1	☐ 2	☐ 3	☐ 4
12	Worrying about the future and the possibility of serious complications?	☐ 0	☐ 1	☐ 2	☐ 3	☐ 4
13	Feelings of guilt or anxiety when you get off track with your diabetes management?	☐ 0	☐ 1	☐ 2	☐ 3	☐ 4
14	Not 'accepting' your diabetes?	☐ 0	☐ 1	☐ 2	☐ 3	☐ 4
15	Feeling unsatisfied with your diabetes physician?	☐ 0	☐ 1	☐ 2	☐ 3	☐ 4
16	Feeling that diabetes is taking up too much of your mental and physical energy every day?	☐ 0	☐ 1	☐ 2	☐ 3	☐ 4
17	Feeling alone with your diabetes?	☐ 0	☐ 1	☐ 2	☐ 3	☐ 4
18	Feeling that your friends and family are not supportive of your diabetes management efforts?	☐ 0	☐ 1	☐ 2	☐ 3	☐ 4
19	Coping with complications of diabetes?	☐ 0	☐ 1	☐ 2	☐ 3	☐ 4
20	Feeling 'burned out' by the constant effort needed to manage diabetes?	☐ 0	☐ 1	☐ 2	☐ 3	☐ 4

© Joslin Diabetes Center, 1999 (www.joslin.org). All rights reserved. The copyright holder/developer has given permission for the questionnaire to be reproduced in this toolkit. Readers of the toolkit are permitted to reproduce the questionnaire for clinical use and non-commercial research purposes. Readers of the toolkit are not permitted to use the questionnaire for commercial research purposes and must seek permission from the copyright holder/developer to do so.

16.6 Appendix 6: DSQOLS Questionnaire

Diabetes specific quality of life scale (DSQOLS)

Date____________ Name____ DOB______________

Please tick the appropriate box for each question –

Which burdens and restrictions from diabetes and its treatment have you experienced during the last four weeks?...	This statement meets my point of view...					
	5. Perfectly	4. Quite good	3. A little	2. Rather not	1. Hardly	0. Not at all
1. It bothers me that I have to measure my blood glucose so often	☐	☐	☐	☐	☐	☐
2. It burdens me that I always have to think about my nutrition.	☐	☐	☐	☐	☐	☐
3. I suffer from pain because of diabetes.	☐	☐	☐	☐	☐	☐
4. Because of diabetes the relationship to my partner has become worse.	☐	☐	☐	☐	☐	☐
5. I am worried about the fact that my life could be shorter because of diabetes.	☐	☐	☐	☐	☐	☐
6. I have the impression that I am less attractive for others because of diabetes.	☐	☐	☐	☐	☐	☐
7. Because of diabetes I feel sad or depressed	☐	☐	☐	☐	☐	☐
8. I am worried about my future health	☐	☐	☐	☐	☐	☐
9. It is a burden for me how other people react to my diabetes	☐	☐	☐	☐	☐	☐
10. I feel nervous and restless when I think about episodes of low blood sugar						
11. I feel tired and exhausted	☐	☐	☐	☐	☐	☐
12. It bothers me that I have to spend so much time on my diabetes treatment	☐	☐	☐	☐	☐	☐
13. Because of diabetes traveling is complicated and troublesome	☐	☐	☐	☐	☐	☐
14. I get anxious and nervous when I think about the dangers of episodes of low blood glucose	☐	☐	☐	☐	☐	☐

	5. Perfectly	4. Quite good	3. A little	2. Rather not	1. Hardly	0. Not at all
15. Diabetes prevents me from spontaneous physical activities	☐	☐	☐	☐	☐	☐
16. I suffer from frequent urination	☐	☐	☐	☐	☐	☐
17. Because of high blood glucose values after a meal I often feel unwell or less efficient	☐	☐	☐	☐	☐	☐
18. Because of diabetes I feel anxious or threatened	☐	☐	☐	☐	☐	☐
19. I suffer from thirst or having a dry mouth	☐	☐	☐	☐	☐	☐
20. Other people find it hard to understand my problems with diabetes treatment	☐	☐	☐	☐	☐	☐
21. I feel physically unwell	☐	☐	☐	☐	☐	☐
22. I unnecessarily worry too much about episodes of low blood glucose	☐	☐	☐	☐	☐	☐
23. I have to give up good-tasting foods	☐	☐	☐	☐	☐	☐
24. I cannot eat as much as I want of certain foods	☐	☐	☐	☐	☐	☐
25. Because of diabetes I often have physical complaints	☐	☐	☐	☐	☐	☐
26. Diabetes interferes with my future plans	☐	☐	☐	☐	☐	☐
27. I get an uncomfortable feeling when I think about the dangers of a severe episode of low blood glucose	☐	☐	☐	☐	☐	☐
28. Because of diabetes I have less contact with friends or acquaintances	☐	☐	☐	☐	☐	☐
29. I wish I could eat more the way I want to, without having to plan everything beforehand	☐	☐	☐	☐	☐	☐
30. I suffer from frequent infections, itching or skin problems	☐	☐	☐	☐	☐	☐
31. I am concerned about getting into embarrassing situations because of episodes of	☐	☐	☐	☐	☐	☐

	5. Perfectly	4. Quite good	3. A little	2. Rather not	1. Hardly	0. Not at all
low blood glucose						
32. I feel weak or lazy	☐	☐	☐	☐	☐	☐
33. I am dissatisfied with the amount of time I have to spend for medical consultations	☐	☐	☐	☐	☐	☐
34. Because of my diabetes I cannot spend my free time the way I would like	☐	☐	☐	☐	☐	☐
35. I feel like a disabled person	☐	☐	☐	☐	☐	☐
36. I am worried that I could sustain physical injury in the event of a severe episode of low blood glucose	☐	☐	☐	☐	☐	☐
37. It bothers me that I cannot eat like other people	☐	☐	☐	☐	☐	☐
38. I am often worried about the long-term	☐	☐	☐	☐	☐	☐
39. Because of diabetes other people treat me like a "sick person"	☐	☐	☐	☐	☐	☐
40. Because of diabetes I cannot pursue my hobbies as I wish	☐	☐	☐	☐	☐	☐
41. I have to frequently think about diabetes and its consequences	☐	☐	☐	☐	☐	☐
42. It bothers me that I cannot eat as spontaneously as people who do not have diabetes	☐	☐	☐	☐	☐	☐
43. It upsets my stomach when I think about the dangers of severe episodes of low blood glucose	☐	☐	☐	☐	☐	☐
44. Diabetes constantly causes problems while dealing with other people	☐	☐	☐	☐	☐	☐

With respect to diabetes treatment it is very important to me that, resp. it would be my goal that …	5. Very Important	4. Quite Important	3. Rather Important	2. Rather Unimportant	1. Quite Unimportant	0. Totally Unimportant
45. my blood glucose values are always below 140 mg%	☐	☐	☐	☐	☐	☐
46. my blood glucose values are as constant as possible	☐	☐	☐	☐	☐	☐
47. I can plan my leisure flexibly	☐	☐	☐	☐	☐	☐
48. I can avoid even mild hypoglycaemia	☐	☐	☐	☐	☐	☐
49. I can avoid late complications of diabetes in any case, resp. avoid their progress.	☐	☐	☐	☐	☐	☐
50. I can eat flexibly in the way that I can choose the kind, amount and time of meals	☐	☐	☐	☐	☐	☐
51. I am as physically fit as possible.	☐	☐	☐	☐	☐	☐
52. I can in any case avoid severe hypoglycaemia with loss of consciousness	☐	☐	☐	☐	☐	☐
53. I have to measure my blood glucose as rarely as possible.	☐	☐	☐	☐	☐	☐
54. Other people don't find out that I have diabetes,	☐	☐	☐	☐	☐	☐

How satisfied have you been during the last weeks with …	0. Very Satisfied	1. Quite Satisfied	2. Rather Satisfied	3. Rather dissatisfied	4. Quite dissatisfied	5. Totally dissatisfied
55. the level of your blood glucose values?	☐	☐	☐	☐	☐	☐
56. The stability of your blood glucose values?	☐	☐	☐	☐	☐	☐
57. The flexibility of planning your leisure?	☐	☐	☐	☐	☐	☐
58. The frequency of mild hypoglycaemia?	☐	☐	☐	☐	☐	☐
59. your assurance of being protected against late complications, resp. of being able to avoid their progress?	☐	☐	☐	☐	☐	☐
60. The flexibility of your diet?	☐	☐	☐	☐	☐	☐
61. your physical fitness?	☐	☐	☐	☐	☐	☐
62. your assurance of being protected against severe hypoglycaemia with loss of consciousness?	☐	☐	☐	☐	☐	☐
63. the frequency of your blood glucose self-monitoring?	☐	☐	☐	☐	☐	☐
64. The extent to what other people are informed about your diabetes?	☐	☐	☐	☐	☐	☐

FULL/LONG TITLE OF THE TRIAL
Triple Therapy for Type 1 Diabetes with Insulin, Semaglutide and Dapagliflozin (TTT1) - UK

SHORT STUDY TITLE / ACRONYM
TTT1 (UK)

PROTOCOL VERSION NUMBER AND DATE
Version 8.0 5th Feb 2026

RESEARCH REFERENCE NUMBERS

**IRAS Number:**	254431
**EudraCT Number:**	2018-004120-11
**ISRCTN Number / Clinical trials.gov Number:**	NCT03899402
**SPONSOR Number:**	GN17DI706
**FUNDER Number:**	3-SRA-2019-669-M-B

SIGNATURE PAGE

The undersigned confirm that the following protocol has been agreed and accepted and that the Chief Investigator agrees to conduct the trial in compliance with the approved protocol and will adhere to the principles outlined in the Medicines for Human Use (Clinical Trials) Regulations 2004 (SI 2004/1031), amended regulations (SI 2006/1928) and any subsequent amendments of the clinical trial regulations, Good Clinical Practice (GCP) guidelines, the Sponsor's (Standard Operating Procedures (SOPs), and other regulatory requirements as amended.
I agree to ensure that the confidential information contained in this document will not be used for any other purpose other than the evaluation or conduct of the clinical investigation without the prior written consent of the Sponsor
I also confirm that I will make the findings of the study publicly available through publication or other dissemination tools without any unnecessary delay and that an honest accurate and transparent account of the study will be given; and that any discrepancies from the study as planned in this protocol will be explained.

For and on behalf of the Study Co-Sponsor (University of Glasgow):
Signature: .... Date: ....../....../......
Name (please print): ....
Position: ....

For and on behalf of the Study Co-Sponsor (NHS Greater Glasgow and Clyde):
Signature: .... Date: ....../....../......
Name (please print): ....
Position: ....

UK Chief Investigator:
Signature: .... Date: ....../....../......
Name: (please print): John Petrie ....
Position: Professor of Diabetes..

Statistician:
Signature: .... Date: ....../....../......
Name: (please print): ....

Position: ....

KEY TRIAL CONTACTS

UK Chief Investigator	Professor John R Petrie<br>Professor of Diabetes<br>Director, Robertson Centre for Biostatistics<br>Director, Glasgow Clinical Trials Unit<br>School of Health and Wellbeing<br>College of Medical, Veterinary and Life Sciences<br>University of Glasgow<br>Clarice Pears Building, Level 5<br>90 Byres Road<br>Glasgow<br>G12 8TB<br>Secretary +44(0)141 330 4744<br>john.petrie@glasgow.ac.uk
UK Co- Investigator	Dr James Boyle<br>Consultant Diabetologist<br>Glasgow Royal Infirmary<br>84 Castle Street, Glasgow, G4 0SF<br>Tel: : +44(0)141 211 5996<br>james.boyle@glasgow.ac.uk
Trial Co-ordination	Miss Lisa Jolly<br>Research and Innovation<br>NHS Greater Glasgow & Clyde<br>Ward 11, Dykebar Hospital<br>Grahamston Road<br>Paisley, PA2 7DE<br>Lisa.Jolly@ggc.scot.nhs.uk
Co-Sponsor	NHS Greater Glasgow & Clyde<br><br>Contact: Dr Maureen Travers<br>Research and Development Management Office<br>Research and Innovation<br>NHS Greater Glasgow & Clyde<br>Ward 11, Dykebar Hospital<br>Grahamston Road<br>Paisley, PA2 7DE<br>Tel: +44 (0) 0141 314 4012<br>maureen.travers@ggc.scot.nhs.uk
Co-sponsor	University of Glasgow<br><br>Contact: Dr Debra Stuart<br>College of Medical, Veterinary & Life Sciences<br>Room 327 Wolfson Medical School Building<br>University Avenue<br>Glasgow G12 8QQ

	Tel: +44 (0)141 330 4539<br>Debra.Stuart@glasgow.ac.uk
Funder	JDRF<br>26 Broadway, 15th floor, New York, NY 10004, USA
Clinical Trials Unit	Glasgow Clinical Trials Unit<br>Robertson Centre for Biostatistics<br>Clarice Pears Building, Level 5<br>90 Byres Road<br>Glasgow<br>G12 8TB<br>Tel: 0141 330 4744<br>liz.anderson@glasgow.ac.uk
Statisticians	Professor Alex McConnachie<br>Robertson Centre for Biostatistics<br>Clarice Pears Building, Level 5<br>90 Byres Road<br>Glasgow<br>G12 8TB<br>Telephone: 0141 330 4744<br><br>Ms Nicola Greenlaw<br>Robertson Centre for Biostatistics<br>Clarice Pears Building, Level 5<br>90 Byres Road<br>Glasgow<br>G12 8TB
Trials pharmacist	Dr Elizabeth Douglas<br>Clinical Trials Pharmacist<br>Clinical Research and Development<br>Ward 11, Dykebar Hospital,<br>Grahamston Road	Paisley PA2 7DE<br>Tel: 0141 314 4073<br>E-mail: elizabeth.douglas@ggc.scot.nhs.uk

US Principal Investigator	Dr Paresh Dandona<br>Director, Diabetes-Endocrinology Center of Western NY<br>Chief of Endocrinology, Diabetes, Endocrinology and Metabolism, SUNY at Buffalo<br>1000 Youngs Road, Suite 105<br>Williamsville NY 14221<br>Telephone: +1(716) 535-1850<br>Fax: +1(716)-639-1200

	pdandona@kaleidahealth.org
US Co- Investigator	Dr Husam Ghanim, PhD.<br>Director, Diabetes and Endocrinology Research Lab<br>Research Associate Professor,<br>School of Medicine, State University of NY at Buffalo<br>Clinical and Translational Research Center<br>875 Ellicott St<br>Buffalo, NY 14203<br>Tel: (+1)716-881-8924<br>ghanim@buffalo.edu
Key Protocol Contributors	Professor John Petrie<br>john.petrie@glasgow.acuk<br><br>Dr Paresh Dandona<br>pdandona@kaleidahealth.org<br><br>Husam Ghanim, PhD.<br>ghanim@buffalo.edu<br><br>Dr James Boyle<br>james.boyle@glasgow.ac.uk<br><br>Dr Robert Lindsay<br>robert.lindsay@glasgow.ac.uk<br><br>Dr Joseph Timmons<br>joseph.timmons@glasgow.ac.uk<br><br>Professor Alex McConnachie<br>Alex.McConnachie@glasgow.ac.uk

TRIAL SUMMARY

Trial Title	Triple Therapy for Type 1 Diabetes with Insulin, Semaglutide, and Dapagliflozin (UK)	
Internal ref. no. (or short title)	TTT1	
Clinical Phase	Phase 3	
Trial Design	Two six-month periods: (i) open-label randomised (2:1) parallel group semaglutide vs standard of care; (ii) additional double-blind, randomised (1:1) parallel group, dapagliflozin vs placebo for those participants initially randomised to semaglutide.<br>Regardless of each randomisation, ongoing standard of care (insulin therapy) will be provided for all participants throughout the study.	
Trial Participants	Adults with type 1 diabetes for at least one year with HbA1c 7.5-11.0% on stable multiple daily injections (MDI) (four or more) of insulin or continuous subcutaneous insulin infusion (CSII) for the previous three months.	
Planned Sample Size	82 (41 in UK)	
Treatment duration	Approximately 1 year	
Follow up duration	Approximately 1 year	
Planned Trial Period	Approximately 5 years	
	Objectives	Outcome Measures
Primary	To assess in people with type 1 diabetes whether triple therapy (dapagliflozin, semaglutide and insulin) reduces HbA1c in comparison with dual therapy (placebo, semaglutide, and insulin – period 2).	HbA1c
Secondary	To assess in people with type 1 diabetes whether triple therapy (dapagliflozin, semaglutide and insulin) reduces HbA1c in comparison with standard therapy (insulin alone)	HbA1c
	To assess in people with type 1 diabetes whether dual therapy (semaglutide and insulin - period 1) reduces HbA1c in comparison with standard therapy (insulin alone)	HbA1c
	To assess in people with type 1 diabetes whether triple therapy (dapagliflozin, semaglutide and insulin) improves glucose control	- Mean fasting blood glucose<br><br>- Mean blood glucose*<br>- Percentage time in range

	as assessed by CGM, insulin requirement, body weight, blood pressure and quality of life in comparison with (a) dual therapy (placebo, semaglutide, and insulin – period 2) and (b) standard therapy (insulin alone)	- Glycaemic variability*<br>- Percentage time in hyperglycaemia Level 1 (10.1-13.9 mmol/)*<br>- Percentage time in hyperglycaemia Level 2 (>13.9 mmol/L)*<br>-Percentage time in hypoglycaemia Level 1 (3.0 – 3.8 mmol/L)*<br>** CGM (by masked Dexcom G6)*<br><br>Insulin requirement<br>Body weight<br>Systolic/ diastolic blood pressure<br>Number of antihypertensive agents<br>Diabetes Specific Quality of Life Scale (DSQOLS)<br>Problem Areas In Diabetes (PAID) questionnaire
Safety Endpoint 1	a) Triple therapy (dapagliflozin, semaglutide and insulin) in comparison with dual therapy (placebo, semaglutide, insulin – period 2)<br><br>b) Dual therapy (semaglutide and insulin - period 1) in comparison with standard therapy (insulin only)	Difference between treatment arms in:<br>(i) Rate of Level 2 (<3.0 mmol/L) hypoglycaemic events (by Dexcom G6 CGM)<br>(ii) Rate of Level 3 hypoglycaemic events (severe event with altered mental and/or physical status requiring assistance)
Safety Endpoint 2	a) Triple therapy (dapagliflozin, semaglutide and insulin) in comparison with dual therapy (placebo, semaglutide, and insulin – period 2)<br><br>b) Dual therapy (semaglutide and insulin –period 1) in comparison with standard therapy (insulin only)	Difference between treatment arms in:<br>Rate of diabetic ketoacidosis (serum ketones or urinary ketones above normal limits AND serum bicarbonate < 15 mmol/L OR blood pH < 7.3)
Investigational Medicinal Product(s)	Semaglutide<br>Dapagliflozin<br>Placebo matching dapagliflozin	
Non-investigational Medicinal Product(s)	Insulin	
Formulation, Dose, Route of Administration	Semaglutide (subcutaneous injection, once weekly) – open label by pen device (0.25 mg/ 0.5 mg/ 1.0 mg)<br>Dapagliflozin 5 mg (oral) – one then two capsules daily (up-titration)<br>Placebo matching dapagliflozin	

FUNDING AND SUPPORT IN KIND

FUNDER(S)	FINANCIAL AND NON FINANCIALSUPPORT GIVEN
JDRF New York<br>26 Broadway, 15th floor, New York, NY 10004, United States	$1,440,000 (to University at Buffalo, the State University of New York, NY (SUNY Buffalo), USA; and University of Glasgow, UK)
Novo Nordisk, 800 Scudders Mill Road, Princeton Landing, NJ, Plainsboro, 08536.	$160,000 and donation of semaglutide to Glasgow site during period of supply disruption (see paragraph headed "Novo Nordisk" below)
AstraZeneca<br>1800 Concord Pike, Wilmington, DE 19803, USA	Provision of dapagliflozin and matched placebo investigational medicinal products to US site
Dexcom; Suite 6, 1 Tanfield, Edinburgh, EH3 5DA	Staff training and discounted supply of CGM sensors

ROLE OF STUDY SPONSOR AND FUNDER

NHS Greater Glasgow & Clyde and The University of Glasgow will be Co-sponsors of the UK arm of the trial. Prior to study initiation, a non-commercially funded clinical trial co-sponsorship agreement will be put in place between NHS Greater Glasgow & Clyde and The University of Glasgow. The roles and liabilities each organisation will take under The Medicines for Human Use (Clinical Trials) Regulations, 2004 SI 2001:1031 are laid out in this agreement signed by both organisations. The University of Glasgow shall be responsible for carrying out the obligations and responsibilities set out in the aforementioned agreement, and shall be deemed "sponsor" for the purposes of, Part 3 of the regulations in relation to the study. NHS Greater Glasgow & Clyde shall be responsible for carrying out the responsibilities set out in the agreement, and shall be deemed "sponsor" for the purposes of, Parts 4, 5, 6 and 7 of the Regulations in relation to the study.

The Co-Sponsors will delegate specific roles to the Chief Investigator, Glasgow CTU and other third parties. These arrangements will be clearly documented in agreements and/or the Sponsor Delegated Roles and Responsibilities Matrix.

The US arm of the trial has the same inclusion and exclusion criteria and the same pre-specified outcomes but an individualised Protocol. It will collect the same data via a common eCRF. Adverse event data from each site will be available to the trial Data and Safety Monitoring Board. The UK Co-Sponsors will have no responsibility for (and carry no liability for) the conduct of the US arm of the trial.

JDRF

The study has been funded by a grant from JDRF. JDRF does not have a designated role or responsibility in trial design, conduct, data analysis and interpretation, manuscript writing, and dissemination of results. Periodic reports (at least annual) in relation to progress of the trial will be submitted to the JDRF. Support from JDRF will be acknowledged in any publications related to the study.

Astra Zeneca

This is an investigator-initiated study. Astra Zeneca provided support by supplying investigational medicinal products (IMPs) dapagliflozin and matched placebo to the US site only. Representatives of Astra Zeneca reviewed and provided comments on drafts of the Protocol but the company does not

have a designated role or responsibility in trial design, conduct, data analysis and interpretation, manuscript writing, and dissemination of results.

Novo Nordisk
In November 2020, Novo Nordisk made up a shortfall of funding that resulted from a cut back by 10% of the main grant by the funder JDRF due to the COVID-19 pandemic. By this time, the Protocol had been finalised. The company does not have a designated role or responsibility in trial design, conduct, data analysis and interpretation, manuscript writing, or dissemination of results.

Supply shortages of semaglutide (various strengths) from 2022 onwards resulted in actual and potential disruption to participant recruitment and ongoing treatment. In response to a direct request from the UK Chief Investigator, Novo Nordisk donated supplies of semaglutide to the Glasgow site based on anticipated use during the period of disruption.

ROLES AND RESPONSIBILITIES OF TRIAL MANAGEMENT COMMITTEES/GROUPS & INDIVIDUALS

Trial Steering Committee (TSC)

The role of the TSC is to provide overall supervision of the trial and ensure that it is being conducted in accordance with the principles of GCP and the relevant regulations. The TSC will:

1. agree the trial protocol and any protocol amendments
2. ensure that conduct of the trial is fully harmonised between the two sites
3. provide advice to the investigators on all aspects of the trial

It will have an independent Chairperson and include the US and UK investigators and key protocol contributors listed above.

Decisions about continuation or termination of the trial or substantial amendments to the protocol will be the responsibility of the TSC who will advise the co-sponsors. The TSC will meet at the start of the study, and four to six monthly thereafter. The TSC will have its own Charter outlining the role and responsibilities of its members. The TSC may invite other attendees from the trial team to present or participate in discussions on particular topics. These attendees will be non-voting members.

Independent data monitoring committee (IDMC)

An IDMC will be established to include two independent medical experts (covering the domains of type 1 diabetes and metabolic disease; one of the academic clinicians will act as chair) and an independent biostatistician. The Glasgow CTU will liaise with the committee and ensure that the Committee is provided with adequate information about study progress and results.

The IDMC will have a formal Charter outlining the responsibilities of the IDMC members, Glasgow CTU and the co-sponsors. Responsibilities include:

• To protect the safety of participants recruited to the trial.
• Advising the TSC and co-sponsors if it is safe and appropriate to continue with the study
• Examining information provided by the Glasgow CTU on study recruitment, adverse eventsand outcomes
- • Consider the need for additional unscheduled reviews of study data.
• Providing reports for the Project Office to forward to the TSC, ethics committees, regulatory bodies, study co-sponsors, and the BHF.

The IDMC may recommend to the TSC to terminate the study or specific aspects of the study if clear harm is observed with one treatment option compared to standard of care or placebo. Specific safety endpoints that will be reviewed include rates of hypoglycaemia and ketosis/ ketoacidosis.

The IDMC will meet approximately every six months.

Study Management Group (SMG)

Trial activity at the UK/ Glasgow site will be coordinated from NHS Greater Glasgow & Clyde (NHS GG&C) by the TTT1 Study Management Group (SMG). The SMG will consist of the UK chief investigator, co-principal investigator other co-applicants, project manager and representatives from the Glasgow CTU, NHS GG&C and The University of Glasgow. The role of the group is to monitor all

aspects of the conduct and progress of the trial in the UK, ensuring adherence to the protocol, and take appropriate action to safeguard participants and the quality of the trial itself.

Joint Study Management Group

Coordination of trial activity between the UK/ Glasgow and the US/ Buffalo site will be facilitated by the Joint TTT1 Study Management Group. This will consist of the US and UK chief investigators, co-principal investigators, other co-applicants, study coordinators, project managers and governance/ administrative representatives as appropriate from State University of New York (SUNY), the Glasgow CTU, NHS GG&C and The University of Glasgow. It will meet approximately every 4-6 weeks. The role of the group is to coordinate all aspects of the conduct and progress of the trial between the US and UK sites, ensuring protocol adherence, and taking appropriate action to safeguard participants and the quality of the trial itself.

Protocol contributors

The protocol has been developed by a group with extensive clinical and research experience in type 1 diabetes and clinical trials including Professor John Petrie and Professor Paresh Dandona. Professor Alex McConnachie has research expertise in design, conduct, analysis and interpretation of clinical trials and biostatistical methods.

KEY WORDS:

Adjunct therapy
Dapagliflozin
Glycated haemoglobin (HbA1c)
Semaglutide
Type 1 diabetes

LIST of CONTENTS

RESEARCH REFERENCE NUMBERS 2
SIGNATURE PAGE 3
KEY TRIAL CONTACTS 4
LIST of CONTENTS....................14
LIST OF ABBREVIATIONS....................15
TRIAL FLOW CHART....................17
1 BACKGROUND 20
2 RATIONALE....................21
3 OBJECTIVES AND OUTCOME MEASURES/ ENDPOINTS....................25
4 TRIAL DESIGN 28
5 STUDY SETTING 28
6 ELIGIBILITY CRITERIA 28
7 TRIAL PROCEDURES....................30
8 TRIAL MEDICATION…………………..58
9 PHARMACOVIGILANCE…………………63
10 STATISTICS AND DATA ANALYSIS....................71
11 DATA HANDLING 73
12 MONITORING, AUDIT & INSPECTION 74
13 ETHICAL AND REGULATORY CONSIDERATIONS....................75
14 DISSEMINATION POLICY…………………77
15 REFERENCES…………………..78
16. APPENDICES....................83

LIST OF ABBREVIATIONS

ACS	Acute Coronary Syndrome
AUC	Area Under the Curve
AE	Adverse Event
AR	Adverse Reaction
CA	Competent Authority
CABG	Coronary Artery Bypass Graft
CGM	Continuous Glucose Monitoring
CHI	Community Health Index
CI	Chief Investigator
CRO	Contract Research Organisation
CRP	C-Reactive Protein
CSII	Continuous Subcutaneous Insulin Infusion
CTA	Clinical Trial Authorisation
CTU	Clinical Trials Unit
CTIMP	Clinical Trial of Investigational Medicinal Product
CV	Cardiovascular
CVA	Cerebrovascular Accident
DKA	Diabetic Ketoacidosis
DSUR	Development Safety Update Report
EC	European Commission
ECG	Electrocardiogram
eCRF	Electronic Case Report Form
EDTA	Ethylenediaminetetraacetic acid
eGFR	Estimated Glomerular Filtration Rate
EMEA	European Medicines Agency
EU	European Union
EUCTD	European Clinical Trials Directive
EudraCT	European Clinical Trials Database
EudraVIGILANCE	European database for Pharmacovigilance
FBC	Full Blood Count
FGM	Flash Glucose Monitoring
GCP	Good Clinical Practice
GDG	Guideline Development Group
GDPR	General Data Protection Regulation
GLP-1	Glucagon Like Peptide-1
GLP-1RA	Glucagon Like Peptide-1 Receptor Agonist
GMP	Good Manufacturing Practice
HbA1c	Glycated haemoglobin
IB	Investigator Brochure
ICF	Informed Consent Form
ICH	International Conference on Harmonisation of technical requirements for registration of pharmaceuticals for human use.
IDMC	Independent Data Monitoring Committee
IMP	Investigational Medicinal Product
IMPD	Investigational Medicinal Product Dossier

ISF	Investigator Site File
ISRCTN	International Standard Randomised Controlled Trials Number
IWRS	Interactive Web-based Randomisation System
LPLV	Last Patient Last Visit
MA	Marketing Authorisation
MDRD	Modification of Diet in Renal Disease
MODY	Maturity Onset Diabetes of the Young
MHRA	Medicines and Healthcare products Regulatory Agency
MI	Myocardial Infarction
MDI	Multiple Daily Injections
NHS GG&C	National Health Service Greater Glasgow & Clyde
NHS R&D	National Health Service Research & Development
NICE	The National Institute for Health and Care Excellence
NIMP	Non-Investigational Medicinal Product
PI	Principal Investigator
PIC	Participant Identification Centre
PIS	Participant Information Sheet
PV	Pharmacovigilance
QA	Quality Assurance
QC	Quality Control
QoL	Quality of Life
QP	Qualified Person
RCT	Randomised Control Trial
REC	Research Ethics Committee
SAE	Serious Adverse Event
SAP	Statistical Analysis Plan
SAR	Serious Adverse Reaction
SDV	Source Data Verification
SGLT2	Sodium Glucose Transporter 2
SOP	Standard Operating Procedure
SUNY	State University of New York
SmPC	Summary of Product Characteristics
SSI	Site Specific Information
SUSAR	Suspected Unexpected Serious Adverse Reaction
TIA	Transient Ischaemic Attack
TMG	Trial Management Group
TSC	Trial Steering Committee
TMF	Trial Master File
UB	University of Buffalo
ULN	Upper Limit of Normal reference range
WOCBP	Women Of Childbearing Potential

TRIAL FLOW CHART

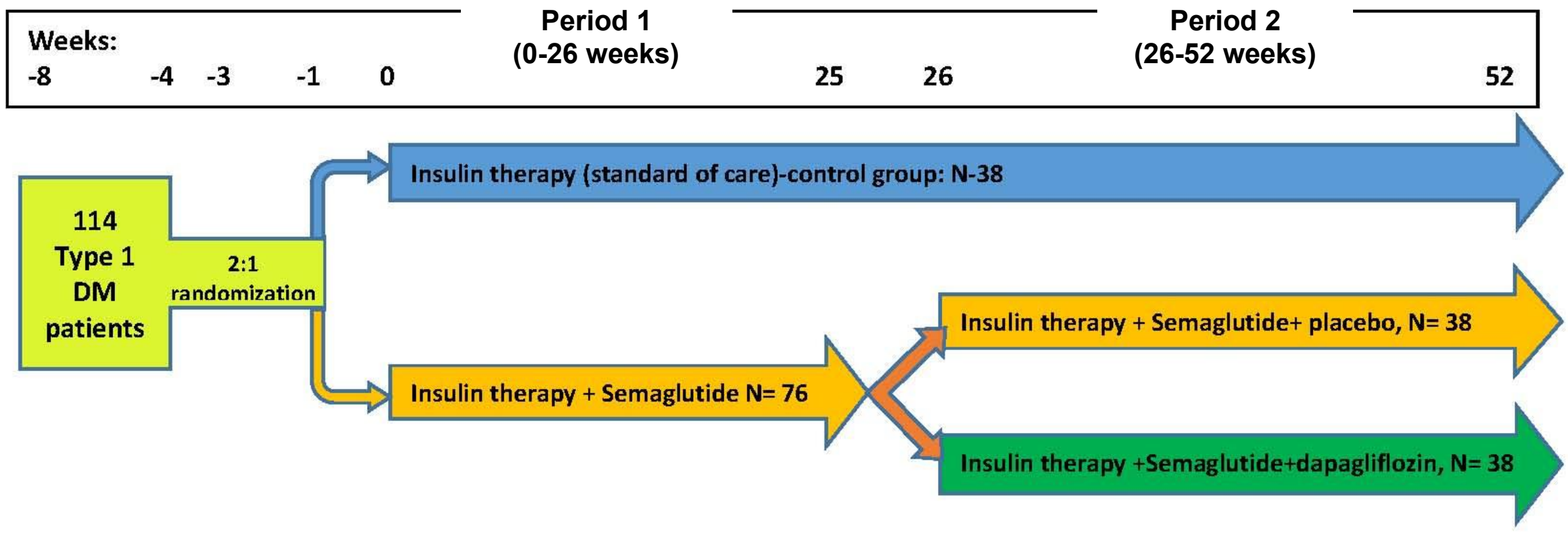

Revised Flow Chart
At this time, a revised sample size in Period 2 of around 23 evaluable participants per group for dapagliflozin vs placebo. This reduces the recruitment target for first randomisation from n=114 to n=82

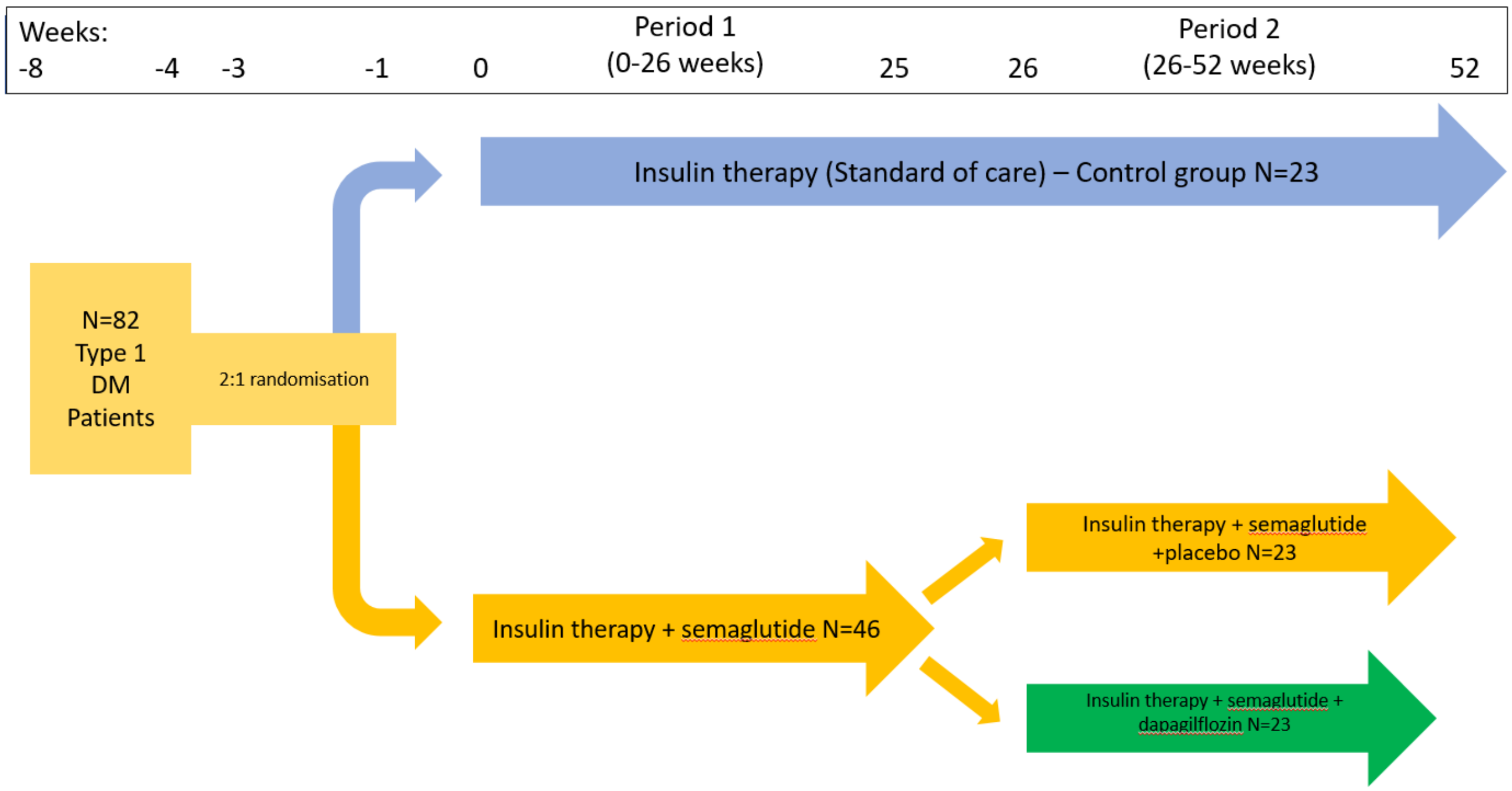
Weeks:
-8
-4
-3
-1
0
Period 1
(0-26 weeks)
25
26
Period 2
(26-52 weeks)
52
Insulin therapy (Standard of care) – Control group N=23
N=82
Type 1
DM
Patients
2:1 randomisation
Insulin therapy + semaglutide N=46
Insulin therapy + semaglutide
+placebo N=23
Insulin therapy + semaglutide +
dapagilflozin N=23

SCHEDULE OF ASSESSMENTS

**Visit #**	**-**	**1**	**2**	**3**	**4[3]**	**5[3]**	**6**	**7**	**8**	**9**	**10**	**11[3]**	**12**	**13**	**14**	**15**	**16**
**Week# (±7 days; see below)**	**-8 to -4**	**-3**	**-1**	**0**	**1**	**2**	**4**	**13**	**23**	**25**	**26**	**27**	**31**	**39**	**49**	**51**	**52**
Study Procedures	Screening	Run-in period		Randomise							Randomise						End
Informed Consent	X																
Fasting visit			X	X				X		X	X			X		X	X
History and demographics	X																
Physical	X																X
Education on hypoglycaemia and ketosis avoidance				X							X						
BP, heart rate, weight	X	X	X	X	X	X	X	X	X	X	X	X	X	X	X	X	X
Bloods (FBC, kidney and liver profiles)	X			X				X			X		X	X			X
C-peptide	X										X						X
Pregnancy test	X			X			X[1]	X[1]	X[1]		X[1]		X[1]	X[1]	X[1]	X[1]	X[1]
Dispense open-label semaglutide[2]				X		X[2]	X[2]	X[2]			X[2]			X[2]			
Titrate open-label semaglutide[2]						X[2]	X[2]	X[2]									
Dose Semaglutide (on-site if dose is due on visit day)				X	X	X											
Dispense dapagliflozin/placebo											X	X		X			
Dapagliflozin/placebo return capsule counts												X		X			X
HbA1c	X			X				X		X				X			X
Blood endpoints				X				X			X			X			X
Masked CGM fit (enter data this visit)		X							X						X		
Masked CGM read				X							X						X
Daily glucose monitoring review		X	X	X	X	X	X	X	X	X	X	X	X	X	X	X	X
Daily urinary ketones review												X	X	X	X	X	X
Insulin dose review		X	X	X	X	X	X	X	X	X	X	X	X	X	X	X	X
Ketone bodies (blood/urine)	X	X	X	X	X	X	X	X	X	X	X	X	X	X	X	X	X
Adverse Event reporting		X	X	X	X	X	X	X	X	X	X	X	X	X	X	X	X
Meal Challenge Test			X							X						X	
24 hr Urine dispensed			X				X			X			X			X	
24 hr Urine collected				X				X			X			X			X
Quality of life questionnaires				X							X						X

N.B. Safety phone calls will be conducted at weeks 3, 5, 8, 16, 20, 29, 35, 44 and at two weeks after the end of the study to document any side effects or other changes. **Abbreviations:** see pages 14-15

Additional visits or phone calls may be scheduled with the verbal consent of the participant if study team believe necessary e.g. closer monitoring is appropriate (see Patient Information Sheet)

Visit dates should be within seven days of the visit time; however, a longer or shorter interval does not constitute a protocol deviation

[1]Not required in women randomised to "Insulin therapy (standard of care)

[2]According to randomisation schedule: resupply of open-label semaglutide after Visit 3 will be according to individual requirements as tolerated (see Section 8.3)

[3] Visits 4, 5 and 11 have the option of being carried out by telephone for those participants allocated to the standard care arm only.

STUDY PROTOCOL

Triple Therapy for Type 1 Diabetes with Insulin, Semaglutide, and Dapagliflozin

1 BACKGROUND

The control of glucose homeostasis in type 1 diabetes is fragile since β-cell reserve is negligible. Traditionally, insulin has been the only treatment approach available. This often leads to erratic blood glucose control given the challenge of matching exogenous insulin to mealtime glucose excursions in the face of a lack of significant endogenous insulin to compensate for variation in requirements in response to meals and activity levels. Injected insulin boluses rarely match precise insulin requirements either in terms of dose administered or bio-availability. The result often is wide fluctuations in blood glucose including episodes when it is too low (hypoglycaemia). Based on our work to date and other published reports, previous attempts to add other glucose-lowering medications to insulin therapy yielded minimal benefits on glycaemia (although metformin may have other benefits) (1-3).

Dandona et al. have shown in two preliminary retrospective studies that the addition of liraglutide, a Glucagon Like Peptide -1 receptor agonist (GLP-1RA), to insulin results in consistent improvements in glycemic control in type 1 diabetes as demonstrated by reductions in HbA1c, glycemic excursions, and insulin requirements accompanied by improvements in body weight and systolic blood pressure (4, 5). As collaborators in the present trial, they also completed the first prospective randomised controlled trial of this drug in 72 people with type 1 diabetes. The data not only confirmed improvements in glycemic control (HbA1c), body weight and systolic BP, but also demonstrated a reduction in appetite, carbohydrate intake and low-grade inflammation (6). Importantly, quality of life was also improved. Their recent studies in type 2 diabetes have revealed that systolic blood pressure reduction with GLP-1RA (exenatide) treatment in type 2 diabetes is associated with increased plasma concentrations of ANP, cGMP and cAMP along with reduced concentrations of angiotensinogen, renin and angiotensin II (7). It is possible that these mechanisms play a role in the prevention of cardiovascular complications in type 1 diabetes. There have been no trials to date of semaglutide (a weekly and potent GLP-1RA) in type 1 diabetes.

GLP-1RAs, therefore, have great potential as adjunct therapy for type 1 diabetes. However, mean HbA1c was still ≥7.0% after liraglutide treatment (6) and larger pharma-sponsored trials (ADJUNCT-1 and ADJUNCT-2) reported increased rates of hypoglycaemia (8, 9). In seeking further improvement, the advent of Sodium-GLucose coTransporter 2 (SGLT2) inhibitors is of importance since their mode of action is independent of insulin secretion. Introduced for use in type 2 diabetes, SGLT2 inhibitors are selective antagonists of the sodium-glucose transporter subtype expressed in the proximal convoluted tubules of the kidneys. They act by inhibiting renal glucose re-absorption and therefore stimulating excretion of 60-100 grams per day of glucose in urine. SGLT2 inhibitors have been studied extensively in type 2 diabetes, including the landmark cardiovascular outcome trial EMPA-REG (10), but data have only more recently begun to accumulate in type 1 (11). Short term effects on urine glucose excretion of remogliflozin and empagliflozin are similar to those seen in type 2 diabetes and these translate into improved glycaemic and other metabolic indices including HbA1c, fasting blood glucose, rates of hypoglycaemia, daily insulin dose and weight (12). Glucose-lowering efficacy is attenuated in individuals with eGFR < 45/ml/min/1.73 m^2.(13) Weight loss accompanying increased urinary glucose excretion could translate into reduced cardiovascular complications as in type 2 diabetes, but there are no data so far available to support this hypothesis. More recently, in the Phase 3 trials DEPICT-1 and DEPICT-2 Dandona et al (PI of the US arm of this trial) showed that dapagliflozin improves glycaemic control and HbA1c over six months by about 0.45% without increasing hypoglycaemia and as in other conditions reduces urinary albumin excretion (14,15,16,17). In comparison to other SGLT2 inhibitors studied for use in type 1 diabetes [sotagliflozin and empagliflozin (18, 19)] the risk of diabetic ketoacidosis was relatively modest (see below). Both dapagliflozin and sotagliflozin were approved early in 2019 by the EU Committee for Medicinal Products for Human Use (CHMP) as adjunct therapy in type 1 diabetes for individuals with BMI≥27 kg/m (20,21)2. In August 2019, dapagliflozin (5 mg/ day) was recommended by the National Institute

for Health and Care Excellence as cost-effective for this indication in NHS England (22 However, dapagliflozin did not gain approval for this indication in the USA from the FDA (23). In November 2021, the manufacturer AstraZeneca voluntarily removed this Europe- and UK-specific indication (24) explicitly “not due to any safety concern for dapagliflozin in any condition, including type 1 diabetes” (25).

2 RATIONALE

General hypothesis: adding an SGLT2 inhibitor (dapagliflozin) to a combination of insulin and semaglutide will improve glycaemic control, weight and blood pressure in adults with type 1 diabetes.
Our protocol is designed to allow an objective assessment of whether this triple therapy combination will work effectively and safely so that a majority of people with type 1 diabetes can reach a glycaemic target HbA1c of ≤7.0%. In addition, since both semaglutide and dapagliflozin inhibitors reduce blood pressure and weight, it will allow an assessment of the combined effect of GLP-1RAs and SGLT2 inhibitors on these outcomes. Since there are no previous data on the effects of once weekly semaglutide in type 1 diabetes, or the combination of semaglutide and dapagliflozin compared to standard insulin treatment, the design requires allocation of some participants to insulin therapy only (the current standard of care).

As part of the process by which this trial was awarded funding by JDRF, this research question was considered by lay members of the funding panel and deemed important, relevant and timely. We considered that use of placebo matching dapagliflozin in Period 2 of the trial was justified when dapagliflozin had full approval as adjunct therapy for type 1 diabetes in Europe (although not the USA) as it remained uncertain which adults with type 1 diabetes were suitable for this treatment. In their review, The UK National Institute for Health and Care Excellence (NICE) considered it likely that fewer than 10% of adults with type 1 diabetes would be initiated on SGLT2 inhibitor therapy over the first five years after their favourable decision. As individuals with a slightly lower BMI threshold will be included than recommended by the SmPC, trial participants will be more closely monitored for ketosis than was the case when dapagliflozin was approved for routine clinical care. Dapagliflozin (or placebo) will be administered orally once daily as per current licensed doses in type 2 diabetes (5 mg/day and 10 mg/day: the same doses as used in DEPICT-1 and DEPICT-2) (14,15,16,). We have not incorporated blinding for semaglutide in the design as the effects of the closely-related daily GLP-1RA liraglutide have been well-studied in double-blind trials in type 1 diabetes by ourselves and others, and individuals in both arms compared for the primary outcome comparison will be treated with this agent. (6, 8, 9). Semaglutide will be administered subcutaneously once weekly. The need to study combination adjunct therapy with SGLT 2 inhibitors and GLP-1 RAs is greater than ever following removal of the type 1 diabetes indication for SGLT2 inhibitor monotherapy in the UK and Europe.

2.1 Assessment and management of risk

a) Benefits

Semaglutide (Ozempic): is a once weekly injectable (subcutaneous) GLP1-RA licensed for reducing HbA1c in type 2 diabetes. The licensed doses are 0.25 mg weekly, 0.5 mg weekly and 1.0 mg weekly. In the trial, the starting dose is 0.25 mg weekly uptitrated if tolerated to 0.5 mg weekly after two weeks and then (again if tolerated) after a further two weeks to the maximum dose of 1.0 mg weekly. Semaglutide differs from other weekly GLP-1 receptor agonists in that its half-life is extended by binding to serum albumin i.e. it is not macromolecular. In type 2 diabetes, semaglutide therapy is associated with weight reduction and a reduction in rates of cardiovascular events (26). The licensed dose escalation schedule for routine treatment of type 2 diabetes is at four weekly intervals (27) but in a Phase 2 trial in type 2 diabetes authored by one of this trial's investigators (JP) doses were uptitrated at one-to-two weekly intervals and to higher doses (up to 1.6 mg weekly) (28). Phase 3 trials are currently in progress (for the indication of obesity) using a 2.4 mg semaglutide weekly. It

should be noted that semaglutide 0.25 mg is considered too low a dose for maintenance therapy in type 2 diabetes but it is unclear whether this may have a beneficial effect in type 1 diabetes at this dose.

While semaglutide once weekly has not been investigated previously in type 1 diabetes, liraglutide (a GLP-1 RA for daily administration with a similar molecular structure that binds less avidly to serum albumin) has previously been investigated (see above) in the ADJUNCT 1 and ADJUNCT 2 trials (8,9) in which, compared with placebo it resulted in reductions in HbA1c, weight and insulin requirement, albeit with a small increase in rates of hypoglycaemia (see below).

Dapagliflozin (Forxiga): is a once daily oral SGLT2 inhibitor previously licensed in Europe for reducing HbA1c in people with type 1 diabetes and BMI ≥ 27 kg/m^2 when insulin alone does not provide adequate glycemic control despite optimal insulin therapy (23). Although only the 5 mg dose was licensed for type 1 diabetes,both 5 mg and 10 mg daily are licensed for type 2 diabetes (29). As mentioned above, the removal of the type 1 diabetes indication by AstraZeneca was not due to any safety concern for any indication, including type 1 diabetes. In the Phase 3 trials of dapagliflozin in type 1 diabetes (DEPICT-1 and DEPICT-2) both doses were used. In both DEPICT-1 and DEPICT-2 there were modest additional benefits with 10 mg compared to 5 mg dapagliflozin. For example, in DEPICT-1, insulin dose requirement was reduced by 13.2% vs placebo on 10mg daily compared with 8.8% on 5mg daily (14). Weight reduction vs placebo in DEPICT-1 at 52 weeks was by 4.5% on dapagliflozin 10mg daily compared with 2.95% in those receiving 5 mg daily. Given the propensity of insulin to encourage weight gain over time and the life-long insulin requirement in T1D, sustained weight reduction and insulin dose reduction - even when modest - may be clinically important. There was no increase in the rate of hypoglycaemia in either the 5 mg or 10 mg groups compared with placebo. (14,15)

Dapagliflozin therapy in type 2 diabetes has been demonstrated to be associated with cardiovascular and renal risk reduction: in DECLARE-TIMI, DAPA-HF and DAPA-CKD the 10 mg dose was used (30, 31, 32). Such cardiovascular and renal benefits may translate to type 1 diabetes with appropriate monitoring and risk mitigation, although support for such measures by the manufacturer were withdrawn in the UK at the time of the removal of the type 1 diabetes indication . Given these potential benefits with relatively low increased risk, the 10 mg rather than 5 mg dose was selected for steady state treatment in this protocol.

b) Risks/ side effects Please also refer to the respective UK SmPCs (27, 29)

<u>Semaglutide</u>:

Very common: nausea, diarrhoea; hypoglycaemia (when used with insulin).

Common: vomiting, abdominal pain, constipation, gastro-oesophageal reflux, cholelithiasis, weight loss.

Uncommon: injection site reactions, hypersensitivity, delayed gastric emptying.

Rare: Anaphylactic reaction.

Hypoglycaemia/ ketosis: Semaglutide once weekly has not been studied previously in type 1 diabetes; however, the ADJUNCT-1 and ADJUNCT-2 trials examined the role of liraglutide once daily (a similar molecule in the GLP-RA class that binds less avidly to serum albumin). Although HbA1c and weight were reduced by both 1.2 mg and 1.8 mg liraglutide, rates of symptomatic hypoglycaemia increased by 20-30%. In ADJUNCT-1, insulin doses were re-uptitrated quite rapidly towards baseline levels after an initial dose reduction, but in ADJUNCT-2 were “capped” (and therefore remained lower). Perhaps for that reason, an increase in hypoglycaemia was less consistent in ADJUNCT -2 but there was an excess of ketosis at the higher (1.8 mg) dose, presumably due to under-insulinisation (no lower limit for insulin dose reduction was set). It should be noted that monitoring of participants by site investigators in the ADJUNCT trials was less intensive than will be the case in the present trial with

less frequent opportunities for insulin dose titration (see Section 7). Importantly, participants at high risk of hypoglycaemia, (including those with a history of severe hypoglycaemia and those with hypoglycaemia unawareness) were not excluded from the ADJUNCT trials (8,9).

Retinopathy: In the SUSTAIN-6 cardiovascular outcome trial of semaglutide in type 2 diabetes, rapid reduction of HbA1c was associated with progression of existing proliferative retinopathy in those with a high baseline HbA1c ("early worsening"/ "normoglycaemic re-entry"). However, there was no evidence of worsening of retinopathy on treatment in the trials SUSTAIN 1-5 in which those with proliferative retinopathy were excluded (33, 3). In clinical trials of duration up to one year involving 4,807 patients with type 2 diabetes, adverse events related to diabetic retinopathy were reported in similar proportions of subjects treated with semaglutide (1.7%) and comparators (2.0%) (33).

Other adverse effects: In type 2 diabetes, gastrointestinal adverse effects of semaglutide can be prevented in the majority of cases by careful dose uptitration (28, 34) although up to 5% are ultimately unable to tolerate it and in post-marketing reports (updated SMPC 2024) intestinal obstruction occurs with "unknown" frequency. In our previous trial with liraglutide in T1D, adverse effects occurred with a similar frequency to that observed in T2D (in which there is extensive clinical experience); on this basis it is expected that this will also be the case with semaglutide (8,9).

Dapagliflozin:
Very common: hypoglycaemia (when used with insulin).

Common: ketoacidosis, genital infections (vulvovaginitis, balanitis), urinary tract infection, diabetic ketoacidosis, increased creatinine.

In the DEPICT-1 study, ketoacidosis occurred in 4 of 277 participants on 5mg dapagliflozin, 5 of 296 participants on 10 mg and 3 of 260 participants in the placebo group (summarised as 1%, 2% and 1% respectively) with one severe episode in each group (14). In pooled analyses of DEPICT-1 and DEPICT-2 (14,15) there was, however, an approximate threefold elevated rate of DKA adjudicated as "definite" over placebo on active therapy: 2.0% on 5 mg dapagliflozin, 1.9% on 10 mg and 0.6% on placebo – i.e. rates of DKA were similar with the two doses (14,15,16). Rates of DKA with dapagliflozin were described as "occurring in at least 1 per 100 patients" by the manufacturer at the time the indication was removed (24). Rates of DKA with sotagliflozin, the other SGLT2 inhibitor licensed for type 1 diabetes, were somewhat higher (2.9% on 200 mg, 3.8% on 400 mg, 0.2% on placebo) in pooled analyses of Phase 3 trials (19,21).

Increased creatinine: in 13 studies summarized in the Summary of Product Characteristics increases in serum creatinine (generally transient) occurred in 3.2% vs 1.8% of individuals with eGFR ≥ 60 ml/min/1.73 m^2 who received dapagliflozin 10 mg vs placebo rising to 18.5% vs 9.3% in those with eGFR <30 ml/min/1.73 m^2 (16,29). These changes are usually observed 4 weeks after SGLT2i initiation, recovering by week 12. Participants with eGFR < 45 ml ml/min/1.73 m^2 will not be included in the TTT1 trial and creatinine/ eGFR will be monitored at 5 weeks (Visit 12) and 13 weeks (Visit 13) after randomisation to dapagliflozin or placebo (Visit 10). Any participant who has persistent reduction in eGFR of 15 ml/min or more will meet withdrawal criteria from the trial (see Section 7.8).

Uncommon: fungal infection, volume depletion, constipation.
Not known (several case reports): Fournier's gangrene.

Fournier's gangrene is a very rare clinical entity characterized by necrotizing fasciitis of the perineal area. This has been infrequently reported in association with SGLT-2 inhibitor use in both men and

women. However, there was no evidence of increased risk in a meta-analysis of 84 clinical trials involving 42415 participants. Nevertheless, participants will be advised to seek urgent medical assistance if they experience erythema, pain, swelling of the perineal area associated with fever and malaise (this will be reinforced in the Patient Information Sheet). (36)

Amputation: An increased incidence of lower limb amputation has been reported in type 2 diabetes with the SGLT-2 inhibitor canagliflozin. However, this has not been demonstrated with dapagliflozin (not included in the UK SmPC). People with diabetes are at increased risk of amputation and good foot care is a key component of good diabetes care regardless of use of SGLT-2 inhibitors.

Placebo:
Placebo capsules contain microcrystalline cellulose.
This trial is therefore categorised by the Co-Sponsors as:

- Type B = Somewhat higher than the risk of standard medical care

See Appendix 1 (Risk Assessment)

3 OBJECTIVES AND OUTCOME MEASURES/ ENDPOINTS

3.1 Primary objective

- To assess in people with type 1 diabetes whether triple therapy (dapagliflozin, semaglutide and insulin) for six months improves glycaemic control (as measured by HbA1c) compared with dual therapy (placebo, semaglutide and insulin – Period 2).

3.2 Secondary objectives

To assess in people with type 1 diabetes whether:

- Triple therapy (dapagliflozin, semaglutide and insulin) improves glycaemic control (as measured by HbA1c) compared with standard therapy (insulin)
- Dual therapy (semaglutide and insulin – period 1) improves glycaemic control (as measured by HbA1c) in comparison with standard therapy (insulin only)
- Triple therapy (dapagliflozin, semaglutide and insulin) improves glycaemic control as assessed by a range of continuous glucose monitoring (CGM) parameters, lowers body weight, reduces insulin requirement, improves blood pressure and improves quality of life (as assessed by validated patient questionnaires) compared with dual therapy and standard therapy.

3.3 Outcome measures/endpoints

3.4 Primary endpoint/outcome

Change in HbA1c (week 26 to week 52)

3.5 Secondary endpoints/outcomes

Efficacy:

Change in HbA1c

Mean fasting blood glucose

Mean blood glucose*
Percentage time in range (3.9 - 10.0 mmol/)*
Standard deviation of blood glucose*
Percentage time in hyperglycaemia Level 1 (10.1 - 13.9 mmol/L/)*
Percentage time in hyperglycaemia Level 2 (>13.9 mmol/L) *
Percent time in hypoglycaemia Level 1 (3.0 - 3.8 mmol/)*
(by masked DEXCOM G6 CGM)*

Insulin requirement
Bodyweight
Systolic and diastolic BP
Number of antihypertensive medications
Diabetes Specific Quality of Life Scale (DSQOLS)
Problem Areas In Diabetes (PAID) questionnaire

Safety:

Rate of hypoglycemic events Level 2 (<3.0 mmol/L/ <54mg/dl)
Rate of hypoglycemic events Level 3 (severe event with altered mental and/or physical status requiring assistance)
Rate of diabetic ketoacidosis defined as elevated serum or urine ketones (greater than the upper limit of the normal range), and serum bicarbonate < 15 mmol/L or blood pH < 7.3

3.6 Tertiary endpoints/outcomes

Postprandial glucose (AUC) – meal tolerance test
Postprandial glucagon (AUC) – meal tolerance test
Postprandial FFA (AUC) – meal tolerance test
Postprandial GLP-1 (AUC) – meal tolerance test

3.7 Exploratory endpoints/outcomes

Plasma and urine samples will be collected and frozen for later analysis for indices of inflammatory stress and other relevant measures.

3.8 Table of endpoints/outcomes

Endpoints	Outcome Measures	Timepoint(s) of evaluation of this outcome measure
**Primary endpoint**<br>Triple therapy (dapagliflozin, semaglutide and insulin) in comparison with dual therapy (placebo, semaglutide and insulin – period 2)	Change in HbA1c	Week 26 to Week 52
**Secondary endpoint 1**<br><br>Triple therapy (dapagliflozin, semaglutide and insulin) in comparison with standard therapy (insulin alone)	Change in HbA1c	Week 0 to Week 52
**Secondary endpoint 2**<br><br>Dual therapy (semaglutide and insulin – period 1) in comparison with standard therapy (insulin only)	Change in HbA1c	Week 0 to Week 26
**Other secondary endpoints**<br><br>a) Triple therapy (dapagliflozin, semaglutide and insulin) in comparison with dual therapy (semaglutide, insulin and placebo – period 2)<br><br>b) Dual therapy (semaglutide and insulin – period 1) in comparison with standard therapy (insulin only)	Change in:<br>- Mean fasting blood glucose (4 days)†<br><br>- Mean blood glucose*<br>- Percentage time in range (3.9–10 mmol/L)*<br>- Glycaemic variability (SD)*<br>- Percent time in hyperglycaemia Level 1 (10.1-13.9 mmol/L)*<br>- Percent time in hyperglycaemia Level 2 (>13.9	Week 26 to Week 52<br><br>Week 0 to Week 26

	mmol/L)*<br>- Percent time in hypoglycaemia Level 1* (3.0-3.8 mmol/L)<br><br>*(‡usually 4 days data but may be fewer if missing data)*<br>*(*by masked Dexcom G6 CGM)*<br><br>Insulin requirement<br>Body Weight<br>Systolic and diastolic blood pressure<br>Number of antihypertensive medications<br>Diabetes Specific Quality of Life Scale (DSQOLS)<br>Problem Areas In Diabetes (PAID) questionnaire	
**Tertiary endpoints**<br>a) Triple therapy (dapagliflozin, semaglutide and insulin) in comparison with dual therapy (semaglutide, insulin and placebo –period 2)<br><br>b) Dual therapy (semaglutide and insulin – period 1) in comparison with standard therapy (insulin only)	Change in:<br>Postprandial glucose (AUC)<br>Postprandial FFA (AUC),<br>Postprandial GLP-1 (AUC)<br>Postprandial glucagon (AUC)	Week 26 to Week 52<br><br>Week 0 to Week 26
**Safety endpoint 1**<br>a) Triple therapy (dapagliflozin, semaglutide and insulin) in comparison with dual therapy (semaglutide, insulin and placebo – period 2)<br><br>b) Dual therapy (semaglutide and insulin – period 1) in comparison with standard therapy (insulin only)	Difference between treatment arms in:<br>(i) Rate of Level 2 (<3.0 mmol/L/ <54mg/dL) hypoglycemic events<br>(ii) Rate of Level 3 hypoglycemic events (severe event with altered mental and/or physical status requiring assistance)	Week 26 to Week 52<br><br>Week 0 to Week 26
**Safety endpoint 2**<br>a) Triple therapy (dapagliflozin, semaglutide and insulin) in comparison with dual therapy (semaglutide, insulin and	Difference between treatment arms in:<br><br>Rate of diabetic ketoacidosis (serum or urine ketones above	Week 26 to Week 52

placebo – period 2)<br><br><br>b) Dual therapy (semaglutide and insulin – period 1) in comparison with standard therapy (insulin only)	normal limits) AND serum bicarbonate < 15 mmol/L OR blood pH < 7.3	Week 0 to Week 26

4 TRIAL DESIGN

The trial has two six-month periods:

Period 1 open-label randomised (2:1) parallel group semaglutide once weekly + insulin (standard treatment) vs insulin alone (standard treatment).
Period 2 double-blind, randomised (1:1) parallel group, dapagliflozin vs placebo for those participants initially randomised to semaglutide (with ongoing standard of care for all participants)

Thus, according to the original sample size calculation (from Protocol versions 1.0-5.0) but revised in this Protocol version 6.0 (see Section 10), during the first six months, approximately 114 participants with type 1 diabetes (50 in the UK) will be randomised (open label, see below) into three groups (approximately 38 each; i.e. 19 each in the UK) one of which will be maintained on their regular insulin therapy for 12 months (standard therapy group) and two of which will be started on semaglutide in addition to regular insulin treatment for six months (dual therapy). Following six months of stable treatment, those initially randomised to insulin and semaglutide will be additionally randomised (double-blind) to dapagliflozin (triple therapy) or matching placebo (dual therapy) for a further six months. The dose of dapagliflozin will not be increased to 10 mg in those who have confirmed ketonuria (+1) or ketonaemia (≥0.6 mM) after the first week of treatment.

In keeping with the intention-to-treat principle, participants in whom therapy is discontinued or downtitrated due to adverse effects will be encouraged to continue to undertake all study assessments and attend study visits.

5 STUDY SETTING

The study will be conducted at two sites: State University of New York (SUNY) at Buffalo, USA and Glasgow, UK. In Glasgow, n=41 participants (50% of the new total) will be identified from secondary care sites and via the Scottish Diabetes Research Network research register. Both randomisations will be stratified by site.

6 ELIGIBILITY CRITERIA

6.1 Inclusion criteria

1) Type 1 Diabetes for at least one year on stable multiple daily (four or more) injections (MDI) of insulin or continuous subcutaneous insulin infusion (CSII) for the previous three months.
2) C-peptide < 0.23 nM
3) Minimum dose of insulin: 0.5 U/kg for MDI and 0.4 U/kg for CSII
4) Regularly measuring blood glucose: four or more times daily either by fingerstick, testing or by "flash"/ continuous glucose monitoring)

5) HbA1c 7.5 – 11.0 % inclusive
6) Well versed in carbohydrate counting*
7) Age 18-70 years.
8) BMI ≥25 kg/m^2.

** In the opinion of the site Principal Investigator*

6.2 Exclusion criteria

1) Type 1 diabetes for < 12 months, type 2 diabetes, chronic pancreatitis, MODY
2) Previous use of any agent other than insulin for glycaemic control in the last three months.
3) History of diabetic ketoacidosis (DKA) requiring medical intervention (e.g. emergency room visit and/or hospitalisation) within three months prior to the screening visit.
4) Following ketogenic diet, low (<50g/day) carbohydrate diet or intermittent fasting.
5) Hypoglycaemia unawareness* or frequent episodes of severe hypoglycaemia as defined by more than one episode requiring medical assistance, emergency care (paramedics or emergency room care), or glucagon therapy administered by a third-party individual within three months prior to the screening visit
6) Symptoms of poorly controlled diabetes
7) Participation in a weight loss program with ongoing weight loss, or in an intensive exercise program
8) History of bariatric or other weight-loss surgery within 12 months prior to the screening visit
9) History of Addison's disease or chronic adrenal insufficiency
10) History of diabetes insipidus
11) Hepatic disease or cirrhosis
12) Aspartate Aminotransferase (AST) > 3 X Upper limit of normal (ULN) and/or Alanine aminotransferase (ALT) > 3 X ULN
13) Serum Total Bilirubin > 2 X ULN unless exclusively caused by Gilbert's Syndrome
14) Haemoglobin < 110 g/L (11.0 g/dL) for men; haemoglobin < 100 g/L (10.0 g/dL) for women
15) Coronary event or procedure (myocardial infarction, unstable angina, coronary artery bypass, surgery or coronary angioplasty) in the previous three months or history of congestive heart failure
16) End stage renal disease on haemodialysis and/or eGFR < 45 ml/min/1.73m^2
17) HIV or Hepatitis B/C positive status
18) Any other life-threatening, non-cardiac disease
19) History of pancreatitis or cystic fibrosis
20) Women who are pregnant, planning pregnancy or breast-feeding and women of childbearing potential (WOCBP) who are not established on a method of contraception considered highly effective (see Appendix 2) and willing to continue it for the duration of the study and for at least two months after last dose of IMP.
21) Inability to give informed consent
22) History of gastroparesis
23) History of medullary thyroid carcinoma or MEN 2 syndrome
24) History of hypersensitivity reaction to GLP-1 agonists or SGLT2 inhibitors or known hypersensitivity to any of the excipients
25) Known gallstones (history of cholecystectomy is not an exclusion criterion)
26) History of alcohol problem or drug abuse

27) Hypertriglyceridemia (>500 mg/dl)
28) Recurrent genital mycotic infection (more than one episode in last six months)
29) Hypovolaemia
30) Any malignancy except treated *in situ* malignancy and basal cell carcinoma of the skin
31) Unexplained haematuria (dipstick positive on two occasions one week apart) and/or active bladder cancer
32) Proliferative retinopathy (on basis of photographic retinal screening which must have been performed within the last 24 months)
33) Use of an investigational agent or therapeutic regimen within 90 days of study
34) Participation in any other concurrent interventional clinical trial
35) Unlikely to be able to comply with study requirements*
36) Unable to undergo meal challenge for dietary reasons (e.g. vegetarian/ vegan diet)

** In the opinion of the site Principal Investigator*

7 TRIAL PROCEDURES

Also see *Schedule of Assessments*

Each participant will be enrolled in the trial for approximately 55 weeks, comprising three weeks of monitoring to stabilise treatment and collect baseline data before randomisation into the intervention or standard of care/ placebo arms for 52 weeks. Following a completed screening visit and meeting the Inclusion and Exclusion criteria, each participant will have a total of 16 scheduled study visits. In addition, at weeks 3, 5, 8, 16, 20, 29, 35, 44 and at two weeks after the end of the study, each participant will receive a phone call to document any adverse effects or other changes. No long-term follow-up is planned following completion of participation in the study.

Participants will be treated with either insulin only (standard treatment) for the whole duration of the study or with insulin and semaglutide weekly s.c. injection (titrated where possible up to 1.0 mg, see below) for the first six month period and then randomised to either oral dapagliflozin (triple therapy) or placebo (dual therapy) daily added for the second six month period. Semaglutide will be started (Visit 3) at a dose of 0.25 mg once weekly for two weeks then increased if tolerated to 0.5 mg (Visit 5). If well tolerated at week 4, it will be increased to 1.0 mg once weekly (Visit 6) or at a subsequent visit at the discretion of the PI. Dapagliflozin (or placebo) will be started at a dose of 5 mg daily at week 26 and increased at week 27 to 10 mg (2 x 5mg capsules) daily to ensure drug tolerability and minimise the risk of insulin-induced hypoglycaemia or hypotension.

Excessive weight loss: Both semaglutide and dapagliflozin are known to cause weight loss in the range of 5-10% in type 2 diabetes. However, the magnitude of weight loss when these are combined in type 1 diabetes is uncertain. Excessive weight loss may lead to increased risk of hypoglycemia or decreased insulin requirement increasing the risk of ketosis or DKA (see Section 2.1b) and other unforeseen risks. Therefore, weight loss during any phase of the study will be limited to ≤ 15% of body weight at randomisation or reaching a BMI <22 kg/m^2. If participants are approaching this threshold, IMP will be sequentially reduced at the discretion of the PI. Semaglutide may be reduced to 0.25mg or 0.5 mg and dapagliflozin to 5 mg. See also Section 7.8 regarding treatment interruption and withdrawal.

Covid-19 Mitigation

In view of the Covid-19 pandemic, certain trial procedures may be changed if necessary to reduce in person contact with participants. This will be continuously reviewed in view of the prevailing circumstances in line with local and national guidance and with the principle of protecting participants and study personnel. In the event of future waves of infection this would be particularly important. In this context, consideration would be given to rearranging visits, changing to virtual visits using video technology or using additional phone calls to ensure patient safety. Consideration will also be given to how minimising face-to-face interaction with participants when dispensing study medication. If required, supplies of study medication sufficient to last for longer periods may be dispensed to minimise contact and visits. Courier services may additionally be used to deliver study medications to participants.

All in person visits will be subject to local and national guidance around the use of personal protective equipment including face masks and where appropriate when conducting procedures, the use of gloves and aprons.

Gastrointestinal adverse effects:

During the initial weeks of participation, those participants who report nausea and/or vomiting during uptitration of semaglutide dose that is not sufficiently severe to require treatment discontinuation will remain on their current dose until they are symptom-free prior to discussing a further dose escalation (i.e. uptitration will be to the highest dose tolerated rather than a “forced titration”). Dose will be downtitrated to the maximum tolerated in those who experience gastrointestinal adverse effects.

Retinopathy:

As there was no excess of retinopathy with semaglutide treatment in Phase 3 trials with semaglutide, in which proliferative retinopathy was an exclusion criterion (as in the current protocol), additional retinal imaging beyond annual retinal photography (the standard of care) is not proposed (30).

Insulin dose adjustment:

Minimising hypoglycaemia: All participants will be made aware of the potential for increased hypoglycaemia with addition of adjunct therapy and supported and encouraged to monitor blood glucose at least four times daily in order to minimize the risk of hypoglycaemia. Participants will have ready access to advice from specialist medical and nursing staff at face-to-face and telephone visits. Those who have ongoing access to wearable continuous glucose monitoring [or "flash" glucose monitoring, (FGM)] will continue to use these devices. Information on management of hypoglycaemia will be included within participant diaries, reinforcing the pre-existing knowledge of participants, the majority of whom will have lived with type 1 diabetes for many years.

Reductions in insulin dose will not usually be greater than 10% (5% if HbA1c is < 7.0 %) from current levels. Daily insulin dose will not be reduced by more than 20% from the baseline level (i.e. following adjustment at the randomisation visit) and then not more than 20% from the new baseline levels following adjustment at 26 weeks. Participants requiring an insulin dose reduction > 20% and their total insulin dose < 0.4 U/kg (current weight) will be monitored closely for insulin dose, carbohydrate intake, ketones and blood glucose. Following the second randomisation at 26 weeks, dapagliflozin/ placebo will be temporarily discontinued if insulin dose falls below this threshold. The participant will be withdrawn from the study if this remains so at review in one week [see also Withdrawal Criterion 9 and Section 8.12

Minimising ketosis: Participants will be instructed to stay hydrated and not to reduce their caloric intake (especially carbohydrates) or start new dietary approaches (especially ketogenic diets) during the study. They will be educated to recognize the signs and symptoms of diabetic ketoacidosis (DKA) and to undertake additional self-testing of urine and/or blood ketones if they develop nausea, vomiting, or anorexia. In addition, they will be given education and a written ketosis management plan (STICH Protocol – i.e. STop SGLT-2 inhibitor or placebo (temporarily), give Insulin bolus, take additional Carbohydrate, and additional Hydration - see Appendix 3) (37).

From Visit 10 (26 weeks), those participants undergoing the second randomisation will be supported and encouraged to monitor urine or blood ketones at home at least once daily: three days before visit 10 (week 26), they will be asked to start daily measurements of urine ketones [CombiScreen® 10 SYS Plus urine testing strips, Sterilab, UK (or equivalent)] or blood ketones [CareSens™ Dual, i-SENS, Koreal; GlucoMen® Areo 2K, Menarini, UK (or equivalent]]. They will be asked to record these in the diary/ log provided. In addition, ketones (blood and urine) will be measured at every visit. If ketone bodies in urine are +1 or greater (≥0.6 mM in blood), participants will be reminded to keep well hydrated and notify the study team as soon as possible (office hours) as diabetic ketoacidosis (DKA) can occur even at glucose levels < 14 mmol/L (38).

If ketones are +2 or more in urine (> 1.5 mmol/L) participants will be advised to initiate the STICH Protocol immediately and contact the study team in office hours (or telephone NHS 24 or attend their Accident & Emergency if out of hours) (37). This information will be reinforced verbally at study visits and also in the form of a Patient Alert Card that participants will be asked to carry. This will also contain details of the investigational study drug, the study number, the investigator's name, and a contact number (for in hours contact). The study team will assess for symptoms or test results indicating a risk of ketoacidosis immediately on reporting and if blood ketone levels are persistently (one week) at these levels dapagliflozin/placebo will be permanently discontinued.

If blood ketone readings are confirmed as > 3.0 mmol/L (or urine ketones reading +++) participants will be advised to stop dapagliflozin/ placebo and attend the Accident and Emergency department without delay.

Participants will be advised that dapagliflozin/placebo may be temporarily stopped in other clinical situations known to predispose to ketoacidosis (e.g. acute serious medical illnesses or hospitalisation for major surgical procedures). Any requirement for intravenous insulin infusion (whether in Accident and Emergency or as an in-patient admission) will be recorded as a Serious Adverse Event. For further detailed procedures see section 9.31.

Volume depletion:
There is a theoretical risk of volume depletion and low blood pressure when dapagliflozin is used in combination with other antihypertensive drugs. All pre-existing concomitant medication will be reviewed at the screening visit as part of the medical history and examination (see Section 7.1.3 for further details). Participants undergoing the second randomisation will be educated on symptoms suggestive of low blood pressure (e.g. light-headedness, dizziness, feeling faint) and asked to contact the study team if they experience these symptoms. In clinical practice however SGLT-2 inhibitors are commonly used with anti-hypertensive drugs with no significant problems (31).

Genital mycotic infections:
Individuals who report symptoms suggestive of genital mycotic infection will be advised to self-treat with topical antifungal preparations. If a second episode occurs, a decision on treatment discontinuation will be considered in discussion between the participant and the site Principal Investigator, taking account of the severity of the symptoms.

Foot care:
Annual foot checks will be conducted for all participants as per standard of care.

7.1 Recruitment

7.1.1 Patient identification

All personal details will be managed according to ISO 27001:2013 compliant Standard Operating Procedures.

Potentially eligible participants will be identified from the Scottish Diabetes Research Network Research Register (a managed and searchable database of people with diabetes who have agreed to be contacted about clinical trial participation) and at secondary care diabetes clinics in Greater Glasgow and Clyde. The principal site for recruitment will be Stobhill Ambulatory Care Hospital but other sites may be opened if necessary as Patient Identification Centre sites. The majority of study visits (and all visits involving drug dispensing) will be conducted at the Clinical Research Facility, Glasgow Royal Infirmary. Information posters with the contact details of the research team will be displayed in recruiting clinics.

At the principal site, clinic lists (whether remote or face-to-face) will be pre-reviewed by members of the research team. An email will be sent to the clinic Consultant physician to flag potentially eligible individuals on their list; potential eligibility will also be flagged on the clinic electronic list (on Trakcare®) to prompt reviewing physicians (as members of the usual care team) to ask if the potential participant would be interested in receiving more information from the research team.

In the above email (to which a response is not required), physicians will be asked to record the outcome of their discussion with the potential participant; either: (i) by adding a note to the Clinical Portal system; (ii) adding an appropriate statement to their clinic letter; or (iii) by return email using standard text (e.g. willing to be contacted/ not willing to be contacted/ unsuitable for the trial). The permitted and preferred method(s) of contact (e.g. mobile telephone/ home telephone/ letter/ email should also be recorded. Telephone contact will be the preferred contact method with other methods offered where this is declined. If no preferred method is indicated by consultant it will be presumed that telephone contact is acceptable Physician consultations with potentially eligible individuals that are concluded with no invitation to research participation (e.g. due to pressure of time) can be followed up with a phone call from one of the study physicians who is also a member of the research team.

Following clinical consultations, members of the research team will identify potential participants who appear to be meet eligibility criteria and have given permission to be contacted.. These individuals will be called and/or sent/emailed (as appropriate according to documented permissions) a Patient Information Sheet. An invite letter and Patient Information Sheet will be sent to potential participants who have declined telephone contact but agreed to mail or email contact. Where permission has been given for phone contact, this can be followed up by up to two further phone contacts (provided permission to contact is not withdrawn by the potential participant in an earlier call). A visit to the Clinical Research Facility will then be scheduled for informed consent and screening. The person scheduling the CRF visit will confirm with the patient if they have a copy of the full Patient Information Sheet or if they need a further copy to be sent out in advance of the visit. This will be sent out to arrive at least 48 hours prior to the planned CRF visit or emailed to potential participant.

At the screening visit, eligibility criteria will be checked and the study procedures explained. All potential participants will have had at least 24 hours to review the full Patient Information Sheet. They will then be invited to provide written, informed consent.

At other sites within NHS Greater Glasgow and Clyde the same approach will be taken for the clinic lists of those physicians who indicate willingness to support trial recruitment. The initial approach will always be from a member of the usual care team.

Covid-19 Mitigation: The delivery of outpatient clinics has been substantially changed due to the pandemic. The use of telephone clinics is now standard for many visits and video technologies are also being used. These mediums will therefore be used for inviting participants to take part when in person appointments are not taking place. This may be subject to change depending on the situation with Covid-19. Other principles of recruitment will be as described above.

7.1.2 Screening

A separate non-fasting visit will be arranged at which potential participants will have further time to discuss with the study nurse and doctor.

Prospective participants will be asked to read and understand the Participant Information Sheet. Any questions regarding the Protocol will be answered. Risks and side-effects of the active trial medication will be explained. Eligibility for trial enrolment will be confirmed by a medical practitioner.

Individual wishing to participate in the study will then be invited to sign to document their informed consent (see Section 7.2 below).

7.1.3 Screening assessments

Items from the medical history relevant to the Inclusion and Exclusion criteria will be recorded. Demographic data, medical history and current medications (including current insulin doses) will be documented. All participants will have access to a dual blood glucose and ketone meter as per the contemporary standard of care in type 1 diabetes. A physical examination will be conducted. Clinical observations will be recorded and blood and urine samples obtained (see below):

<u>Demographics</u>

- Date of birth
- Sex
- Ethnicity
- Date of diagnosis
- Ethnic group: white/black/Asian/other
- Height

<u>Clinical assessments</u>

- Systolic and diastolic blood pressure (after 5 minutes rest)
- Heart rate
- Body weight (clothed without coat and shoes)
- Capillary blood glucose

<u>Medical History</u>

- Smoking status: current/ex/never
- Alcohol consumption: units/week
- Hypertension: Y/N

- Microalbuminuria: Y/N
- Proteinuria: Y/N
- Neuropathy: Y/N
- Previous major cardiovascular event(s): Y/N (date)
- Existing CV disease of any type*: Y/N
- Amputation above forefoot: Y/N
- Hospitalisation for DKA in last year
- Level of educational attainment: University (college) educated Y/N

* Existing CV Disease is defined as any of: MI, stroke, ACS/unstable angina, coronary stent/angioplasty, CABG, TIA, heart failure, peripheral vascular disease

Insulin treatment

- Regimen: MDI, pump,
- Total dose (all); Basal (longer acting) dose/ Bolus (shorter acting) dose (for MDI only)

Other treatment (drug classes and names)

Current use Y/N. If no, has there been use in last three months?: Y/N

- ACE inhibitors
- Angiotensin receptor blocker
- Calcium channel blocker
- Alpha-blocker
- Beta-blocker
- Loop diuretic
- Thiazide like diuretic
- Statin
- Aspirin
- Clopidogrel
- Novel anticoagulant
- Warfarin

Blood assessments:

- Blood ketones
- HbA1c
- FBC
- Urea and electrolytes
- Creatinine
- Liver function tests (AST/ ALT/ Alkaline Phosphatase)
- Bone profile (Calcium/ Phosphate)
- C-peptide (capillary blood glucose must be > 4.0 mmol/L at time of sampling)

  N.B. where re-screening is undertaken (see 7.2.1 below), C-peptide does not require to be repeated

If blood tests have been assessed for clinical purposes within the last three weeks, these values will be used.

Urine sample:

- Urine ketones

- For all WOCBP, a urine pregnancy test will be checked at screening

Potential participants meeting all the inclusion and exclusion criteria based on history, examination and screening tests will be enrolled in the study.

Pregnancy: WOCBP will be asked about pregnancy status and contraceptive usage (see Appendix 2) and a pregnancy test will be conducted (following informed consent) prior to randomisation. We will not recruit those who are pregnant, wanting to become pregnant, or using birth control methods not considered highly effective. At specified study visits following randomisation, WOCBP randomised to IMP (as opposed to standard of care) will be asked about their contraception status, and a urine pregnancy test carried out. Study medication will be permanently discontinued in women who become pregnant or considered to be on contraception that is not highly effective. As the study assessments *per se* do not carry an additional risk over standard of care to either mother or unborn child, further pregnancy tests will not be routinely offered following randomisation to those women allocated to “Insulin therapy (standard of care)” (i.e. who are not exposed to IMP). All pregnancies will be notified to the Sponsor Pharmacovigilance Officer using the standard pregnancy notification form and the pregnancy followed to outcome).

7.1.4 Payment

Participants will be offered reasonable travel expenses for visits additional to usual care.

7.2 Consent

Potential participants will be identified and screened by the Inclusion and Exclusion criteria listed above. Medical staff or appropriately trained support staff will seek consent for screening and participation in the trial from individuals that fulfil the criteria. All potential participants will have at least 24 hours to review the Patient Information Sheet before being approached (see Section 7.1.2 above). Following written consent, each signature will be dated by the signatory, the original retained in the site file, a copy provided to the participant and a copy scanned into the participant's Clinical Portal record.

Data collected for routine clinical care will be used for clinical trial documentation (e.g. blood results within the previous three weeks). In the absence of routine blood results, sampling of blood for study specific laboratory measurements can only be performed following documentation of informed consent.

7.2.1 Re-screening post-consent

Where inclusion (e.g. HbA1c ≤7.5%) or exclusion (e.g. DKA within the last three months) criteria are not met (e.g. screening visit blood results falling outside the study parameters), potential participants can be re-screened with additional urine and blood tests on one further occasion.

Where the screening period (i.e. between the Screening visit and Visit 1) exceeds 10 weeks, screening visit urine and blood tests should be repeated at an additional visit that can be arranged with verbal consent of the participant prior to Visit 1. Data will be amended on the web portal as appropriate.

If the initial consent was signed within three months of the re-testing, new consent is not required unless the consent form template has been updated in this period. If the initial consent was not signed within three months of re-testing then consent should be sought again.

Rescreened participants will keep the same Patient ID and data will be amended on the web portal as appropriate.

Where screening bloods are repeated in cases as above, C-peptide testing does not require to be repeated as values consistent with the inclusion criteria remain valid long term.

7.3 Run-in period

7.3.1 Visit 1 (@3 weeks prior to randomisation)

Participants who meet entry criteria will be scheduled for their next visit at approximately three weeks prior to randomisation. Daily fingerstick blood glucose levels (study diaries), CGM/ FGM data or insulin pump download data (as applicable) will be collected and reviewed and appropriate insulin dose adjustments made by the medical team to target fasting blood glucose concentrations between 5 -10.0 mmol/L if possible at all times without having additional hypoglycaemia (< 3.9 mmol/L). Clinical observations will be recorded including resting BP measurement. Blood glucose and ketone bodies will be measured via a fingerstick blood sample (see Section 7). Urine for pregnancy status and ketone bodies will be assessed.

Participants will be instructed on the use of the Continuous Glucose Monitoring System (Dexcom G6 with blinded receiver). All subjects will be advised to monitor their capillary blood glucose by CGM or fingerstick at least 4 times a day (before meals and at bedtime) throughout the study and to wear their CGM constantly for next 20 days (changing the sensor at 10 days). CHO counting technique will be reviewed and appropriate support given with calculating bolus insulin doses.

Visit procedures:

Monitoring and treatment optimisation
- Check for adverse events
- Review blood glucose diary
- Blood ketones
- Adjust insulin doses (if necessary)

Clinical assessments
- Blood pressure
- Heart rate
- Weight

Urine sample:
- Check urine ketones

Other
- Fit and start masked Continuous Glucose Monitoring with Dexcom G6

7.3.2 Visit 2 (1 @ weeks prior to randomisation)

Fasting visit* - Participants will be asked to fast (no food or calorie intake) for 10 hours prior to the visit overnight. Those who plan to drive to fasting visits will be reminded of the increased risk of hypoglycaemia and DVLA guidelines. This will be repeated prior to each fasting visit and reiterated

within the patient diaries. This visit will be scheduled for a morning appointment and participants will be advised not to take their breakfast bolus insulin dose prior to arrival. If participants arrive at the study visit in a non-fasted state, the visit will be terminated and a further fasting visit rearranged as soon as possible.

Daily fingerstick blood glucose levels (diaries), CGM/ FGM data or insulin pump download data (as applicable) will be collected and reviewed and appropriate insulin dose adjustments made. Clinical observations will be recorded as detailed below. Blood glucose and ketone bodies will be measured via a fingerstick blood sample (see Section 7). If blood glucose is less than 3.9 mmol/L, or more than 13.9 mmol/L, or if blood (≥ 0.6 mM), or urine ketones (+1 or greater) are positive, the visit will be rescheduled for an alternative date. Otherwise, an intravenous cannula will be placed in order to obtain blood samples for the duration of the visit. A Meal Challenge Test will be performed (described below). A 24 hr urine container will be dispensed to collect a 24 hour urine sample to bring in at the Week 0 visit. For this collection and all subsequent 24 hour urine collections, urine will be tested via dipstick testing and an aliquot retained for assay of albumin, creatinine, ketones, and 15-isoprostane.

Visit Procedures

Monitoring and treatment optimisation

- Check for adverse events
- Review blood glucose diary
- Blood ketones
- Adjust insulin doses (if necessary)

Clinical assessments

- Blood pressure
- Heart rate
- Weight

Urine sample:

- Check urine ketones
- 24 hour urine container dispensed

Other assessments

- Download Dexcom G6 CGM
- High fat high calorie Meal Challenge: in order to assess postprandial metabolism a Meal Challenge Test will be performed using an approximately 900 kilocalorie High Fat High Carbohydrate (HFHC) meal including an egg muffin sandwich, a sausage muffin sandwich and two hash browns [containing approximately 88g carbohydrate, 51 g fat (33% saturated) and 34 g protein]. Participants will be asked to attend fasting prior to breakfast insulin bolus (with continuing basal insulin); participants will be asked not to take bolus insulin until the 3 hour time point unless blood glucose values reach above 22.2 mmol/L. Blood samples will be collected from an indwelling intravenous canula in a superficial forearm vein for glucose, glucagon, FFA, GLP-1 (AUC). Sequential blood samples will be obtained at 0, 15, 30, 45, 60, 90, 120, 150 and 180 min after consuming the meal. Volume of samples at 15, 30, 45, 90 and 150 min will be 5ml while those at 0, 60, 120 and 180min will be 30 ml (total volume = 145 ml). A spot urine sample will be collected at baseline, 1 and 3hr for standard urinalysis and an aliquot retained for assay of ketones and 15-isoprostane.

7.4 Randomisation

Participants who are being randomised will be required to have undergone all screening assessments.

At the randomisation visit, study participants will be provided with a Patient Alert Card. There are two Versions: (1) for the intervention arm i.e. those allocated at first randomisation to open-label semaglutide who are also eligible for the second double-blind randomisation (dapagliflozin or placebo); and (2) for those allocated to standard of care. These cards supplement the study diaries and include contact information for the study team. Version 1 also contains unblinding information. Participants will be asked to carry their card at all times. These cards will be collected at the end of participation in the study.

7.4.3 First Randomisation Scheme

For the first randomisation (Period 1, open label), eligible and consenting participants will be randomised (2:1, stratified by site) to the two groups (open-label semaglutide or standard of care) using permuted blocks of variable size to minimize predictability.

7.4.4 Method of implementing the allocation sequence

Randomisation will be achieved by accessing an interactive web-based randomisation system (IWRS). The investigator will provide the participant identifier and the system will check the participant's eligibility from information already entered in the electronic Case Report Form (eCRF): if appropriate the randomisation group will be allocated.

7.5 Visit 3 (First Randomisation Visit)

Participants will be asked to attend in the fasting state (no food or calorie intake for 10 hours; see 7.3.2 above). Blood glucose levels and ketone bodies will be measured via a fingerstick blood sample using a glucose and ketone meter (see Section 7) and daily fingerstick blood glucose levels (diaries) collected and reviewed. The participant's own CGM/ FGM blood glucose data or insulin pump download data will also be collected and reviewed. Daily insulin dose will be adjusted following clinical review of study diaries: if reduced, this should not be by more than 20% from the baseline level Participants will discontinue use of the study masked CGM (Dexcom G6). Clinical observations will be recorded. Blood glucose and ketone bodies will be measured via a fingerstick blood sample (see Section 7). The 24 hour urine container will be collected. A fasting blood sample will be taken (45 mL) to evaluate study endpoints, FBC, U&E, LFT, bone profile and HbA1c, (detailed below). A urine sample will be collected to evaluate ketone bodies and, in females, pregnancy status.

Participants will be asked to complete Quality of Life questionnaires (see Appendices).

IMP will be dispensed for those randomised to open-label semaglutide (once weekly). Semaglutide will be started on site at the 0.25 mg dose. Where both research staff and participant are in agreement, the first dose can be self-administered with staff observing; otherwise research staff will administer the first dose. Participants will be instructed to call site staff directly in case of any problem or untoward side effects. An office hours phone number will be provided for advice. Outside of these hours, provision of emergency medical care if required will be via NHS 24 and/or Accident & Emergency. Participants will be specifically asked to call to inform research staff if they have hypoglycaemia (blood glucose < 3.9 mmol/L) or Level 2 hyperglycaemia (blood glucose >13.9 mmol/L), particularly if occurring on more than one occasion. This will apply for the remainder of the trial.

Visit Procedures

Fasting visit* as per visit 2.

Monitoring and treatment optimisation
- Check for adverse events
- Check adequacy of contraception (WOCBP)
- Review blood glucose diary
- Adjust insulin doses (if necessary)
- Blood ketones

Clinical assessments
Blood pressure
- Heart rate
- Body weight

Quality of life assessments
- Diabetes Specific Quality of Life Scale (DSQOLS)
- Problem Areas In Diabetes (PAID) questionnaire

Blood assessments
- Na, K, urea, creatinine, eGFR (MDRD)
- AST/ ALT/ Alkaline Phosphatase
- Calcium/ Phosphate
- FBC
- HbA1c
- Bilirubin
- Random glucose

Urine
- For WOCBP, a urine pregnancy test will be checked prior to first administration of IMP.
- Urine ketones
- 24 hour urine sample collected

Medication
- Administer open label semaglutide 0.25 mg if randomised to active treatment
- Issue Patient Alert Card (appropriate version according to randomisation)

Other:
- Read masked Continuous Glucose Monitoring with Dexcom G6

Bloods for storage – see Sample Handling Manual
Approximately 45 mls of venous blood will be withdrawn and collected in tubes containing EDTA. Blood will be centrifuged at 1500g for 20 mins at 4°C. Plasma will be siphoned, aliquoted and stored locally at -80° ± 10°C until transport in batches to the central laboratory on dry ice.

7.5.1 Visit 4 (@1 week after first randomisation)

Clinical observations will be recorded. Blood glucose levels and ketone bodies will be measured via a fingerstick blood sample using the participant's own glucose and ketone meter (see Section 7)) and daily

blood glucose levels collected and reviewed. Insulin doses will be adjusted as in visit 3. If HbA1c is <7.0 %, insulin dose reduction should not be >5% from baseline total dose. As for Visit 3, semaglutide 0.25mg will be injected on site for those randomised to active therapy; however, if the participant is unable to attend within 48 hours of the scheduled appointment, this dose can be self-administered at home (confirmed by a documented phone call) provided there were no adverse effects following the previous dose.

Visit Procedures

Monitoring and treatment optimisation

- Check for adverse events
- Review blood glucose diary
- Adjust insulin doses (if necessary)
- Blood ketones

Assessments

- Blood pressure
- Heart rate
- Weight

Urine sample:

- Urine ketones

Medication

- Support participant's self-injection of open label semaglutide 0.25 mg if randomised to active treatment (dose on-site if dose is scheduled for visit day (see above)

7.5.2 Visit 5 (@2 weeks after first randomisation)

Clinical observations will be recorded. Blood glucose levels and ketone bodies will be measured via a fingerstick blood sample using glucose and ketones meter (see Section 7) and daily blood glucose levels collected and reviewed. Daily blood glucose data collected and reviewed and insulin doses adjusted as per Visit 3. Provided uptitration is considered appropriate by the site PI (or designee) (see also "Gastrointestinal adverse effects," page 31), the semaglutide dose will be increased to 0.5 mg weekly. This will be administered on-site if dose is scheduled for a visit day but can be self-administered at home (confirmed by a documented phone call) provided both research staff and participant are in agreement and there were no adverse effects following the previous dose. All subsequent trial doses of semaglutide can be self-administered on-site or at home as convenient and appropriate. Supply of IMP will be dispensed as appropriate to last until the next visit.

Visit Procedures

Monitoring and treatment optimisation

- Check for adverse events
- Review blood glucose diary
- Adjust insulin doses (if necessary)
- Blood ketones

Assessments

- Blood pressure
- Heart rate

- Weight

Urine sample:
- Urine ketones

Medication
- Dose titration (see above) if randomised to open-label semaglutide
- Return used study pen
- Dispense medication if randomised to open-label semaglutide (dose on-site if dose is scheduled for visit day– see above)

7.5.3 Visit 6 (@4 weeks after first randomisation)

For those randomised to open-label semaglutide, doses administered will be recorded (self-report). Clinical observations will be recorded. Daily blood glucose data will be collected and reviewed and insulin doses adjusted as per visit 3. Supply of IMP will be given to last until the next visit. A urine sample will be collected to evaluate ketone bodies and, in females randomised to IMP, pregnancy status.

Provided uptitration is considered appropriate by the site PI (or designee) (see also "Gastrointestinal adverse effects," page 31), the recommended semaglutide dose will be increased to 1.0 mg weekly (or 0.5 mg if not previously uptitrated from 0.25 mg). A 24 hr urine container will be dispensed to collect a 24 hour urine sample to bring in at the Week 13 visit.

Visit Procedures

Monitoring and treatment optimisation
- Check for adverse events
- Check adequacy of contraception (WOCBP)
- Review blood glucose diary
- Adjust insulin doses (if necessary)
- Blood ketones

Assessments
- Blood pressure
- Heart rate
- Weight

Urine sample:
- Urine ketones
- Urine pregnancy test (WOCBP randomised to IMP only)
- 24 hour urine container dispensed

Medication
- Record doses of semaglutide self-injected since last visit (self-report)
- Dose titration if randomised to open-label semaglutide (see above)
- Return used study pens
- Dispense medication if randomised to open-label semaglutide

7.5.4 Visit 7 (@13 weeks after first randomisation)

Participants will be asked to attend in the fasting state (no food or calorie intake for 10 hours). If participant is not fasting, samples should be taken and non-fasting status noted. For those randomised to open-label semaglutide, doses administered will be recorded (self-report). For those who have remained on doses lower than 1.0 mg weekly, this visit will be the last opportunity to uptitrate. Provided uptitration is considered appropriate by the site PI (or designee) (see also "Gastrointestinal adverse effects," page 31) the semaglutide dose will be increased from 0.25 mg to 0.5 mg weekly or from 0.5 mg to 1.0 mg weekly. Daily fingerstick blood glucose levels (diaries), CGM/ FGM data or insulin pump download data will be collected and reviewed and appropriate insulin dose adjustments made. Supply of IMP will be given to last until visit 10. Observations will be recorded. A urine sample will be collected to evaluate ketone bodies and, in females randomised to IMP, pregnancy status. The 24 hour urine container will be collected. A fasting blood sample will be taken (45 mL) to evaluate study endpoints.

Visit Procedures

Fasting visit*

Monitoring and treatment optimisation
- Check adequacy of contraception (WOCBP)
- Check for adverse events
- Review blood glucose diary
- Adjust insulin doses (if necessary)
- Blood ketones

Clinical assessments
- Blood pressure
- Heart rate
- Weight

Blood assessments
- Na, K, urea, creatinine, eGFR (MDRD)
- AST/ ALT/ Alkaline Phosphatase
- Calcium/ Phosphate
- FBC
- HbA1c
- Bilirubin
- Random glucose

Urine
- Pregnancy test (WOCBP randomised to IMP only)
- Urine ketones
- 24 hour urine bottle collected

Medication
- Record doses of semaglutide self-injected since last visit (self-report)
- Dose titration if randomised to open-label semaglutide (see above)
- **N.B. no further uptitration after this visit**
- Return used study pens
- Dispense medication if randomised to open-label semaglutide

7.5.5 Visit 8 (@23 weeks after first randomisation)

For those randomised to open-label semaglutide, doses administered will be recorded (self-report). Clinical observations will be recorded. Daily blood glucose data (fingerstick or FGM) will be collected and reviewed as per visit 3. Blood glucose levels and ketone bodies will be measured via a fingerstick blood sample. A urine sample will be collected to evaluate ketone bodies and, in females randomised to IMP, pregnancy status. Insulin doses will be adjusted if needed. All subjects will be reminded to monitor their capillary blood glucose by fingerstick at least 4 times a day (before meals and at bedtime) throughout the study. CGM sensor will be fitted and participants asked to wear their CGM constantly for next 20 days.

Visit Procedures

Monitoring and treatment optimisation

- Check adequacy of contraception (WOCBP)
- Check for adverse events
- Review blood glucose diary
- Blood ketones
- Adjust insulin doses (if necessary)

Clinical assessments

- Blood pressure
- Heart rate
- Weight

Urine sample:

- Pregnancy test (WOCBP randomised to IMP only)
- Urine ketones

Other assessments

- Fit and start masked Continuous Glucose Monitoring with Dexcom G6

7.5.6 Visit 9 (@25 weeks after first randomisation)

Participants will be asked to attend in the fasting state (no food or calorie intake for 10 hours). If participants arrive at the study visit in a non-fasted state, the visit will be terminated and a further fasting visit rearranged as soon as possible. Daily fingerstick blood glucose levels (diaries), CGM/ FGM data or insulin pump download data will be collected and reviewed and appropriate insulin dose adjustments made. Participants will discontinue use of the study CGM (Dexcom G6). Clinical observations will be recorded. Blood glucose levels and ketone bodies will be measured via a fingerstick blood sample (see Section 7). Supply of IMP will be given to last until the next visit. A 24 hr urine container will be dispensed to collect a 24 hour urine sample to bring in at visit 10 (Week 26) visit. A Meal Challenge Test will be performed as per visit 2. Participants will be provided with strips for urine or blood ketone measurement at home and instructed or reminded as appropriate on how to use these. They will be asked to measure urinary and blood ketones every morning for three consecutive days prior to the next visit (second randomisation: dapagliflozin or placebo) and to record the results in their diary.

Visit Procedures

Fasting visit*

Monitoring and treatment optimisation

- Check adequacy of contraception (WOCBP)
- Check for adverse events
- Review blood glucose diary
- Adjust insulin doses (if necessary)
- Blood ketones

Clinical assessments

- Blood pressure
- Heart rate
- Weight

Urine sample:

- Urine ketones
- 24 hour urine container dispensed

Other assessments

- Download Dexcom G6
- Meal Challenge Test

Blood assessments

- HbA1c

7.6 Second Randomisation Scheme

For the second randomisation (Period 2, double-blind), eligible and consenting participants previously randomized to open-label semaglutide will be randomised (1:1) to two groups (dapagliflozin or matching placebo) using permuted blocks of variable size to minimize predictability with stratification for HbA1c (≤ 7.5% vs >7.5% HbA1c) Those participants previously randomised to standard of care will continue as before.

7.6.1 Blinding (period 2 only)

For the double-blind treatment period, dapagliflozin and placebo will be packed in matching packs to maintain the blind. Each pack will contain sufficient supplies for 30 days' treatment with a small overage (excess). Packs will be labelled with a unique number that will be used to assign treatment to the participant via the IWRS system whilst maintaining the blind. Packs will be labelled in accordance with Good Manufacturing Practice and local regulatory requirements.

Unblinding. Treatment discontinuation (of dapagliflozin or placebo), rather than unblinding, will be carried out as far as possible. Participants will be advised in any case of hospitalisation with acute illness to discontinue study medication and inform the relevant clinician. These instructions will be reinforced in the Study Diary. However, where knowledge of treatment may assist emergency treatment, the investigator (or designee) may unblind using the IWRS system. Information on emergency unblinding (the investigational study drug, the study number, the investigator's name and a contact number) will be provided on the Patient Alert Card. For each unblinding an email alert is generated to the Study

Coordinator and Chief Investigator. The most likely scenarios for unblinding will be: confirmed pregnancy, overdose/ accidental ingestion, or development of acute renal failure

7.6.2 Visit 10 (Second Randomisation Visit; @26 weeks after First Randomisation)

Participants will be asked to attend in the fasting state (no food or calorie intake for 10 hours). For those who arrive in a non-fasted state, the visit will be terminated and a further fasting visit rearranged as soon as possible. Participants with normal fasting urine (<+) or blood ketone (<0.6 mmol) in the three days prior to randomisation will proceed to the next treatment phase as per this visit. The principles of the STICH Protocol (see Appendix 3) will be discussed (37). Participants with higher ketone values in at least one of the previous three days will have their glucose levels, insulin dose, and carbohydrate intake reviewed and advised on hydration, adequate CHO intake, and adequate insulin dosing. They will be rescheduled for one week and asked to repeat three days testing before the next visit.

Clinical observations will be recorded. A urine sample will be collected for ketones. Blood glucose or ketones will be measured via a fingerstick blood sample (see Section 7.2.1). Daily fingerstick blood glucose levels (diaries), CGM/ FGM data or insulin pump download data will be collected and reviewed and appropriate insulin dose adjustments made (following the guidance in Section 7, page 35). The previous three days blood or urine ketones levels will be reviewed. The adjusted total insulin dose at this visit will serve as the baseline total insulin dose for the remaining six months of the study and future limits of dose adjustments will be based on this new baseline. The blinded study CGM will be collected. The 24 hour urine container will be collected. A fasting blood sample will be taken (45 mL) to evaluate study endpoints. Participants will be asked to complete quality of life questionnaires.

At this point, those participants previously randomised to receive open-label semaglutide will be randomised to receive dapagliflozin 5 mg daily or matching placebo. They will be educated on symptoms suggestive of low blood pressure (e.g. light-headedness, dizziness, feeling faint).

Participants will be asked to perform a daily (preferably early morning) urine (using CombiScreen® 10 SYS Plus urine testing strips or equivalent) or blood tests for ketone bodies (using the particpant's own meters as per Section 7, "Minimising ketosis": page 28) and record all outcomes in dedicated study diaries. If results of these scheduled (i.e. not done due to symptoms) ketone measurements, are +1 or higher (urine) or > 0.6 mmol/L (blood), participants will be instructed to follow the STICH Protocol as per their study diary and Appendix 3, . For any abnormal ketone levels taken due to symptoms, the DKA consensus guidelines are to be followed instead (see Section 9.3).

Participants will be instructed to call site staff directly in case of any problem or untoward side effects. They will be specifically asked to call if they have confirmed hypoglycaemia (blood glucose levels < 3.9 mmol/L) or hyperglycaemia (blood glucose > 13.9 mmol/L) occurring on more than one occasion. Participants will be advised to remain well hydrated and report any genital mycotic or urinary tract infections

Visit Procedures

Fasting visit* - Requirements and arrangements as per visits 2 and 3.

Monitoring and treatment optimisation

- Check adequacy of contraception (WOCBP)
- Check for adverse events

- Review blood glucose diary
- Adjust insulin doses (if necessary) - this insulin dose is baseline for Period 2
- Blood ketones

Clinical assessments
- Blood pressure
- Heart rate
- Body weight

Quality of life assessments
- Diabetes Specific Quality of Life Scale (DSQOLS)
- Problem Areas In Diabetes (PAID) questionnaire

Blood assessments
- Na, K, urea, creatinine, eGFR (MDRD)
- AST/ ALT/ Alkaline Phosphatase
- Calcium/ Phosphate
- FBC
- Bilirubin
- Random glucose
- C-peptide (capillary blood glucose must be > 4.0 mmol/L at time of sampling)
-

Urine
- Pregnancy test (WOCBP randomised to IMP only)
- Urine ketones
- 24 hour urine bottle collected

Medication
- Education on symptoms suggestive of low blood pressure (e.g. light-headedness, dizziness, feeling faint) for those undergoing second randomisation
- Record doses of semaglutide self-injected since Visit 7 (self-report)
- Return used study pens
- Dispense open label semaglutide for participants randomised to active treatment at Period 1 (if required). Dispense dapagliflozin or placebo if randomised to active treatment

Other assessments
- Read masked Continuous Glucose Monitoring sensor (mean fasting blood glucose, mean blood glucose, percentage time in range, glycaemic variability)
-

7.6.3 Visit 11 (@1 week after Second Randomisation)

For those randomised to open-label semaglutide, doses administered will be recorded (self-report). Clinical observations will be recorded. Daily blood glucose and blood/ urine ketone levels will be reviewed and glucose levels collected. A urine sample will be collected to evaluate for ketones bodies. Blood glucose levels and ketones bodies will be reviewed measured via a fingerstick blood sample (see Section 7.2.1). . Daily fingerstick blood glucose levels (diaries), CGM/ FGM data or insulin pump download data will be reviewed and appropriate insulin dose adjustments made using the new baseline from Visit 10 (following the guidance in Section 7, page 35). If at any time total insulin dose reduction is >20% and total

insulin dose is <0.40 U/Kg, additional monitoring (see Appendix 2) will be implemented. Unless blood (>0.6 mmol/L) or urine ketones (+ or more) have been elevated within the previous three days, dapagliflozin or placebo dose will be uptitrated to 10 mg daily (two capsules of either active or placebo medication) for the remainder of the study. Supply of IMPs will be given to last until week 39.

Visit Procedures

Monitoring and treatment optimisation

- Check adequacy of contraception.
- Check for adverse events
- Review blood glucose diary
- Adjust insulin doses (if necessary)
- Blood ketones
- Daily blood or urine ketones review

Clinical assessments

- Blood pressure
- Heart rate
- Body weight

Urine sample:

- Urine ketones

Medication

- Count returned dapagliflozin or placebo capsules
- Increase dose of dapagliflozin or placebo to 10mg (see above – review ketones)
- Dispense dapagliflozin or placebo (record dose)

7.6.4 Visit 12 (@5 weeks after Second Randomisation)

For those randomised to open-label semaglutide, doses administered will be recorded (self-report). Clinical observations will be recorded. Daily blood glucose and blood/ urine ketone levels will be reviewed and glucose levels collected. Blood glucose levels and ketone bodies will be measured via a fingerstick blood sample. A urine sample will be collected to evaluate ketone bodies and if appropriate pregnancy status. Insulin dose will be adjusted if as per Visit 11. A blood sample will be taken (15 mL) to evaluate study endpoints. A 24 hr urine container will be dispensed to collect a 24 hour urine sample to bring in at the Week 39 visit

Visit Procedures

Monitoring and treatment optimisation

- Check adequacy of contraception (WOCBP)
- Check for adverse events
- Review blood glucose diary
- Adjust insulin doses (if necessary)
- Blood ketones

Clinical assessments

- Blood pressure
- Heart rate
- Weight

Blood assessments
- Na, K, urea, creatinine, eGFR (MDRD)
- AST/ ALT/ Alkaline Phosphatase
- Calcium/ Phosphate
- FBC
- Bilirubin
- Random glucose

Urine
- Pregnancy test (WOCBP randomised to IMP only)
- Urine ketones
- 24 hour urine container dispensed

7.6.5 Visit 13 (@13 weeks after Second Randomisation)

For those randomised to open-label semaglutide, doses administered will be recorded (self-report). Participants will be asked to attend in the fasting state. If participant is not fasting, samples should be taken and non-fasting status noted. Clinical observations will be recorded. Daily blood glucose and blood/ urine ketone levels will be reviewed and glucose levels collected. Blood glucose levels and ketone bodies will be measured via a fingerstick blood sample. A urine sample will be collected to evaluate ketone bodies and if appropriate pregnancy status. The 24 hour urine container will be collected. Insulin dose will be adjusted if needed as in as per Visit 11. Sufficient supply of IMPs will be given until the end of the study. A fasting blood sample will be taken (45 mL) to evaluate study endpoints.

Visit Procedures

Fasting visit* - as per previous fasting visits

Monitoring and treatment optimisation
- Check adequacy of contraception (WOCBP)
- Check for adverse events
- Review blood glucose diary
- Adjust insulin doses (if necessary)
- Blood ketones
- Daily urine ketones review

Clinical assessments
- Blood pressure
- Heart rate
- Weight

Quality of life assessments

- Diabetes Specific Quality of Life Scale (DSQOLS)
- Problem Areas In Diabetes (PAID) questionnaire

Blood assessments
- Na, K, urea, creatinine, eGFR (MDRD)
- AST/ ALT/ Alkaline Phosphatase
- Calcium/ Phosphate
- FBC
- HbA1c
- Bilirubin
- Random glucose

Urine
- Pregnancy test (WOCBP randomised to IMP only)
- Urine ketones
- 24 hour urine bottle collected

Medication
- Record doses of semaglutide administered since Visit 10 (self-report)
- Dispense open label semaglutide for participants randomised to active treatment at Period 1 (if required).
- Count returned dapagliflozin or placebo capsules
- Dispense dapagliflozin or placebo (record dose)

7.6.6 Visit 14 (@23 weeks after Second Randomisation)

For those randomised to open-label semaglutide, doses administered will be recorded (self-report). Clinical observations will be recorded. Daily blood glucose and blood/ urine ketone levels will be reviewed and glucose levels collected. Blood glucose levels and ketone bodies will be measured via a fingerstick blood sample. A urine sample will be collected to evaluate ketone bodies and if appropriate pregnancy status. Insulin dose will be adjusted if as per Visit 11. All subjects will be reminded to monitor their capillary blood glucose by fingerstick at least four times a day (before meals and at bedtime). CGM (Dexcom G6) sensor will be fitted and patients asked to wear their CGM constantly for the next 20 days.

Visit Procedures

Monitoring and treatment optimisation
- Check adequacy of contraception (WOCBP)
- Check for adverse events
- Review blood glucose diary
- Adjust insulin doses (if necessary)
- Blood ketones
- Daily urine ketones review

Clinical assessments
- Blood pressure
- Heart rate

- Weight

Urine sample:
- Pregnancy test (WOCBP randomised to IMP only)
- Urine ketones

Clinical and functional assessment
- *Fit and start* masked Continuous Glucose Monitoring sensor (mean fasting blood glucose, mean blood glucose, percentage time in range, glycaemic variability)

7.6.7 Visit 15 (@25 weeks after Second Randomisation)

Participants will be asked to attend in the fasting state. If participants arrive at the study visit in a non-fasted state, the visit will be terminated and a further fasting visit rearranged as soon as possible. For those randomised to open-label semaglutide, doses administered will be recorded (self-report). Observations will be taken. Daily blood glucose and blood/ urine ketone levels will be reviewed and glucose levels collected. Blood glucose levels (fingerstick or FGM) and ketone bodies will be measured via a fingerstick blood sample. A urine sample will be collected to evaluate urine ketone levels. Insulin dose will be adjusted if needed as per visit 11. A 24 hr urine container will be dispensed to collect a 24 hour urine sample to bring in at the Week 52 visit. A HFHC Meal Challenge Test will be performed as in Visit 2.

Visit Procedures

Fasting visit* - Procedure as per previous fasting visits

Monitoring and treatment optimisation
- Check adequacy of contraception (WOCBP)
- Check for adverse events
- Review blood glucose diary
- Adjust insulin doses (if necessary)
- Daily urine ketones review
- Blood ketones

Assessments
- Blood pressure
- Heart rate
- Weight

Urine sample:
- Pregnancy test (WOCBP randomised to IMP only)
- 24 hour urine container dispensed

Other assessments
- Meal Challenge Test

7.6.8 Visit 16 (End of study; @26 weeks after Second Randomisation)

Participants will be asked to attend in the fasting state. If participants arrive at the study visit in a non-fasted state, the visit will be terminated and a further fasting visit rearranged as soon as possible. Clinical observations will be recorded. Daily blood glucose and blood/ urine ketone levels will be reviewed and glucose levels collected. Blood glucose levels and ketone bodies will be measured via a fingerstick blood sample. Insulin doses will be reviewed and likely requirement for an increase following discontinuation of IMP discussed with those taking open-label semaglutide. A urine sample will be collected to evaluate ketone bodies and, if appropriate pregnancy status. The CGM will be collected. The 24 hour urine container will be collected. A fasting blood sample will be taken (45 mL) to evaluate study endpoints. We will ask the participants to complete quality of life questionnaires. A physical examination will be performed as per the screening visit. This will be the final face-to-face study visit. A safety phone call will be conducted two weeks to review blood glucose data, provide further insulin dosing advice, to document any adverse events, and to ensure that follow-up in usual clinical care has been arranged.

Visit Procedures

Fasting visit*

Monitoring and treatment optimisation

- Check for adverse events
- Review blood glucose diary
- Adjust insulin doses (if necessary)
- Blood ketones
- Daily urine ketones review

Clinical assessments

- Blood pressure
- Weight
- Heart rate
- Physical examination

Quality of life assessments

- Diabetes Specific Quality of Life Scale (DSQOLS)
- Problem Areas In Diabetes (PAID) questionnaire

Blood assessments

- Na, K, urea, creatinine, eGFR (MDRD)
- AST/ ALT/ Alkaline Phosphatase
- Calcium/ Phosphate
- FBC
- HbA1c
- Bilirubin
- Random glucose
- C-peptide

Urine

- Pregnancy test (WOCBP randomised to IMP only)

- Urine ketones
- 24 hour collection

Other

- Record doses of semaglutide administered (self-report)
- Return used study pens
- Count returned dapagliflozin or placebo capsules
- Collect Patient Alert Card
- Read masked Continuous Glucose Monitoring sensor (mean fasting blood glucose, mean blood glucose, percentage time in range, glycaemic variability)

7.7 Retention and strategies for maximizing follow-up

Participants will be encouraged to attend all study visits. Prior to commencing the trial, all potential participants will have the opportunity to read the detailed Patient Information Sheet which will outline the level of participation requested. Potential participants will be made aware of the need for frequent study visits and asked to commit to these.

Study visits can be rearranged flexibly within a period around the planned visit as specified in the timeline of visits above (Section 7) and actual dates recorded in the eCRF. This is to maximise participant ability to attend all visits. A longer or shorter interval does not constitute a protocol deviation. Where possible, visits will be arranged to coincide with other appointments to further maximise attendance and participant convenience. Telephone contact will be frequent and will help to maintain contact with participants and ensure timely reminders of upcoming study visits can be given.

Even in the scenario of treatment discontinuation (of either IMP), participants will be encouraged to continue to attend further study visits as scheduled.

As stated in point 3 of the Schedule of Assessments, telephone visits are allowed for V4, V5 and V11 for those randomised to the standard of care arm. Participants who have discontinued both study IMPs (see below Section 7.8 for criteria) can also be offered telephone visits for V4, V5 and V11 if this is preferable and encourages retention in the study.

7.8 Treatment Interruptions and Withdrawal criteria

Treatment Interruptions

Treatment interruption from study IMPs may be necessary in some participants. In the case of dapagliflozin, this will occur in any acute illness or hospitalisation which increases the risk of ketosis and ketoacidosis. Circumstances in which temporary discontinuation of dapagliflozin or placebo should occur will be discussed with all study participants. Written information will be provided in the study diaries. An emergency phone line to the study team will be available to all participants for the duration of their participation in the trial. This will be operational during office hours (Monday to Friday 9am – 5pm). The Patient Alert Card also contains contact details for unblinding in the event of acute intercurrent illness and a requirement for unblinding in the opinion of the acute medical team treating the participant. If any participant develops diabetic ketoacidosis (DKA) as defined as per the International Consensus guidelines on two occasions with no identifiable precipitant (section 2.1) they will be withdrawn from the study and the UK Chief and US Principal Investigators informed.

Any other intolerable side effects, acute illness, or other clinical change which may impact participant safety (e.g. excessive weight loss) may require temporary discontinuation of the study drug(s) (dapagliflozin/placebo or semaglutide). In these circumstances, any decision to recommence the study drug(s), the dose and the appropriateness of re-uptitration, will be taken in consultation with the participant, including consideration of the total participant burden, particularly given the need for additional safety checks. If in the judgement of the site PI the study drug(s) cannot be safely restarted, if side-effects remain intolerable or if any of the criteria for study drug withdrawal are met, the treatment with the study medicine(s) must be permanently stopped. Participants who have withdrawn from treatment will be encouraged to continue to attend study visits.

Criteria for withdrawal

Treatment withdrawal or withdrawal from the trial as appropriate (see below) may be necessary prior to the planned end at 52 weeks following first randomisation for any of the following reasons:

1. The site PI can stop investigational study treatment or remove participants from the trial without participants approval if for any reason they feels is appropriate, including: severe side effects, injury or medical condition which may place the patient at risk of further complications if he/she continue to participate, failure to take the medication as instructed, failure to keep scheduled appointments, insufficient level of self-monitoring, cancellation of the study or other administrative reasons.

2. Any woman who becomes pregnant during the period of study will be withdrawn from the trial.

3. Participants who experience persistent severe nausea or vomiting after starting semaglutide which is not managed by dose reduction will discontinue the investigational study treatment. Those with mild or moderate nausea will continue for at least one more week. For those who continue to experience significant nausea (defined as interfering with normal activities), dose will be reduced: if these side effects persist, they will discontinue study medication(s). Participants for whom this occurs in Period 1 will remain eligible for randomisation in Period 2.

4. Excessive weight loss during either period of the study despite treatment dose reduction (> 15% body weight or BMI < 22 kg/m^2 on repeated measurements more than four weeks apart) will lead to treatment withdrawal. If the participant is willing, they may continue to attend study visits (at the discretion of the site PI).

5. New diagnosis of other conditions included in the Exclusion Criteria will result in withdrawal of the participant from the trial.

6. Change in method of insulin delivery: any participant in whom insulin delivery is changed from CSII to MDI or vice versa during the study will be withdrawn from the trial unless pump treatment interruption is temporary for a period of at most 2 weeks. Participants who upgrade to “artificial pancreas” (e.g. Medtronic’s MiniMed 670G hybrid) or any form of hybrid closed-loop therapy during the study will be withdrawn from the trial.

7. Hypoglycaemia: any participant who experiences two episodes of severe hypoglycaemia (requiring external intervention) will be withdrawn from the trial.

8. Ketosis (with no other identifiable precipitating factors): any participant who experiences persistent (1 week) asymptomatic or symptomatic and unmanaged (using STICH protocol – see section 2.1 b) blood ketone levels >1.5 mmol (or ≥+2 in urine) will be withdrawn from the trial. (37)

9. Insulin dose: any participant who experiences a persistent (>1 week) total insulin dose reduction of >20% from baseline AND total insulin dose <0.4U/Kg in Period 2 will be withdrawn from the trial.

10. DKA: any participant who experiences two episode of DKA (with no identifiable precipitating factors) as defined by the expert consensus statement will be withdrawn from the trial (39)

11. UTI: any participant who experiences two episodes of UTI will be withdrawn from the trial.

12. Genital mycotic infection: any participant who experiences two episodes of mycotic genital infection with severe symptoms will be usually be withdrawn from the trial (see also heading "Genital mycotic infections," page 32).

13. Any participant who experiences significant persistent reduction in eGFR > 15 ml/min will be withdrawn from the trial.

14. Pancreatitis: any participant who experiences an episode judged to be due to pancreatitis by the site PI will be withdrawn from the trial.

15. Participants who experience other intolerable and persistent side effects specific to study drugs will be offered treatment downtitration or treatment withdrawal with continued follow-up within the trial if they are willing - except when this is considered inappropriate by the site PI, in which case they will be withdrawn.

Participation in this study is voluntary. Participants have the right to refuse to participate or to withdraw from participation at any time for any reason. The site PI can stop investigational study treatment or remove participants from the trial without participant's approval. If a patient withdraws consent and decides not to participate in the study or is withdrawn from the trial according to the above criteria, she/he will be asked to undergo a final study visit for his/her safety. This visit will follow the same procedures for Visit 16 provided there is ongoing consent. The research team will arrange appropriate handover to the usual clinical team – additional telephone (of if necessary face to face) contact can be arranged if necessary in the interim. If a subject withdraws from the research, the data collected to that point will be used toward the research finding, including study data collected at final study visit, provided there is ongoing consent. If applicable the subject will be asked to return any unused research drug and or devices.

7.9 Storage and analysis of samples

Blood samples will be undertaken at the beginning and at various points during the study as outlined in detail in the schedule of visits. If blood testing has recently been undertaken in the 3 weeks prior to the study commencing, some of these blood tests may be used as baseline. Blood tests will include analysis of the full blood count, renal function (urea and electrolytes), liver function tests, bone profile, and several specialised blood tests for biomarker assays (at central laboratory) following a high fat high caloric meal challenges to examine study endpoints.

Covid-19 Mitigation

All blood sampling or trial procedures will be undertaken following local and national guidance around personal protective equipment. Mask, gloves and aprons will be used as per current guidelines.

7.9.1 Sample collection and processing

- Samples will be appropriately labelled in accordance with the trial procedures to comply with the General Data Protection Regulation (GDPR). Biological samples collected from participants as part of this trial will be transported, stored, accessed and processed in accordance with national legislation relating to the use and storage of human tissue for research purposes and such activities shall at least meet the requirements as set out in the 2004 Human Tissue Act and the 2006 Human Tissue (Scotland) Act.
- Blood will be taken according to the Schedule of Assessments (page 17). Plasma will be siphoned, aliquoted and stored locally at -80° ± 10°C until transport in batches to the central laboratory on dry ice. according to the Sample Handling Procedure as agreed by the Joint Study Management Group
- Individual patient samples will be identified with a unique, anonymised study number.

7.9.2 Sample transport to central laboratory and analysis

- Samples will be transferred to State University of New York in a single batch at the end of recruitment to the study. More frequent transfer can be organised if there are local storage limitations.
- Samples will be transported on dry ice and stored at the central laboratory at -80° ± 10° until analysis. Transport by courier will coordinated by the Trial Manager.
- At the time of analysis individual aliquots will be defrosted at room temperature and analysed in a single batch for each biomarker of interest.
- Samples will be stored at -80° ± 10° in the central laboratory for possible future analysis for novel biomarkers.

7.10 End of trial

The end of the trial will be following the last study visit of the last subject.
At the end of the specified trial period (approximately 52 weeks following first randomisation) all participants will discontinue study medications and revert to standard of care (insulin therapy only). This process will be supervised by the trial physicians, research nurses, and research pharmacists under the supervision of the site Principal Investigator. The trial team will liaise with the usual care team to ensure a safe transition back to standard therapy. Following the final scheduled trial visit there will be a safety phone call two weeks after the last visit. This will act as a further opportunity to advise on insulin dosing and ensure there are no ongoing problems or concerns related to the study.

As discussed in section 7.8, there may be circumstances in which trial drugs need to be stopped before the specified end of the trial. If this is the case, the reason(s) for discontinuation will be recorded and the trial clinical team will facilitate safe transition back to usual standard of care when appropriate. Participants will however if eligible (i.e non-exclusion criteria reason for IMP

discontinuation – as listed in Section 7.8) be asked to continue with trial visits. If consent is withdrawn and participants voluntarily choose to leave the trial before the specified end date, all efforts by the clinical trial team will be made to safely facilitate transition back to ordinary standard of care. A follow up safety visit will be offered to all such participants and ongoing contact details will be made available should there be any further unforeseen issues relating to the clinical trial.

The trial may be stopped at an earlier stage than anticipated on the advice of the independent data monitoring committee (IDMC) to the trial steering committee (TSC) for the safety reasons detailed on page 12 and in the study safety endpoints. If this occurs, all participants will be informed and safe discontinuation of study medications will be undertaken under the supervision of the site Principal Investigator.

8 TRIAL MEDICATION

8.1 Name and description of investigational medicinal product(s)

a) Semaglutide (Ozempic®): a once weekly GLP-1 agonist injection indicated for type 2 diabetes (approved by the Scottish Medicines Consortium, January 2019).

   Doses to be used: 0.25mg increased to 0.5mg at two weeks and then to 1mg weekly s.c. injection after a further two weeks. Open label semaglutide will be added to a background of insulin treatment at week 0 for 12 months in 76 participants (38 in the UK) according to the first randomisation; at week 26 they will be further randomised (double-blind) as below into two groups: dual (placebo, semaglutide and insulin) and triple (dapagliflozin, semaglutide and insulin) therapy.

b) Dapagliflozin (as dapagliflozin propanediol monohydrate Forxiga®): a once daily SGLT-2 inhibitor that will be administered orally in a blinded fashion.

   Doses to be used: 5 mg daily increased after one week to 10 mg daily (orally). Dapagliflozin will be started according to the randomisation (Interactive Web System, hosted by University of Glasgow) in 38 participants (19 in the UK) at 26 weeks as an additional treatment on a background of insulin and semaglutide therapy and end at 52 weeks (triple therapy group). Doses to be used: 5 mg increased to 10 mg after one week.

c) Placebo to dapagliflozin: Dapagliflozin matched placebo will be started in 38 participants (19 in the UK) participants at 26 weeks as an additional treatment on a background of insulin and semaglutide therapy and end at 52 weeks (dual therapy group). Doses to be used: as for active dapagliflozin.

8.2 Legal status of Semaglutide and Dapagliflozin

Semaglutide is currently licensed in the UK for those with adult type 2 diabetes which is insufficiently controlled.

8.3 Drug storage and supply

Semaglutide and dapagliflozin/placebo supplies must be stored in the original containers in a locked, secure area with access limited to authorised site staff. Study supplies should be used as directed in the study protocol and not be supplied to any persons other than study participants. All IMPs will be dispensed to participants from the study site pharmacy.

Covid-19 Mitigation

As detailed above in section 7, the supply of IMP may be altered to reduce risk of Covid-19 transmission in the event of future waves of infection. This may involve providing increased quantities of IMP to participants to last longer and therefore minimise physical contact with trial personnel. Use of a courier delivery service to facilitate this may also be considered.

Semaglutide: Supplies of semaglutide pre-filled pens for subcutaneous administration will be sourced from routine hospital stocks. The following semaglutide presentations will be required:

- Semaglutide 0.25 mg: Each pre-filled pen contains 2mg semaglutide in 1.5ml. Each dose contains 0.25mg of semaglutide in 0.19ml solution.
- Semaglutide 0.5 mg: Each pre-filled pen contains 2mg semaglutide in 1.5ml solution. Each dose contains 0.5 mg of semaglutide in 0.37ml solution.
- Semaglutide 1 mg: Each pre-filled pen contains 4 mg semaglutide in 3.0ml solution. Each dose contains 1mg semaglutide in 0.74ml solution.

For all presentations each pre-filled pen is capable of delivering 4 doses which is sufficient for 4 weeks treatment. Pens are for individual participant use only. In the event of a pen-failure, a new pen should be supplied to the participant.

Prior to first use, semaglutide pens must be stored at 2-8 °C in the original container and must not be frozen. Supplies which are frozen either prior to first use or during use must be discard.

Semaglutide pre-filled pens have an in-use shelf life of 6 weeks. After first use the pre-filled pens may be stored below 30oC or in a refrigerator at 2-8 °C. Participants should be advised to store packs away from the cooling element. The pen cap should be on when the pen is not in use in order to protect the contents from light.

Wherever possible, supplies of semaglutide pre-filled pens should be utilised in a pragmatic way so as to minimise unnecessary wastage. Participants should be provided with an appropriate number of pen(s) to last until the next planned dispensing visit. A small overage may be given as necessary to accommodate visit windows etc. <u>The correct strength of pen must be supplied for each dose titration i.e. 0.25mg, 0.5mg and 1.0 mg as tolerated.</u>

A web-based system will be used to randomise participants to semaglutide treatment.

Dapagliflozin 5 mg or placebo capsules: Supplies of dapagliflozin 5mg and matched placebo capsules will be manufactured by NHS Scotland Pharmaceuticals Specials Service/NHS Tayside Pharmaceuticals in accordance with Good Manufacturing Practice requirements. Capsules will be packed into HDPE bottles with a tamper evident seal. Each bottle will be labelled in a double-blind fashion to meet local regulatory requirements and will be assigned a unique number. Bottles containing dapagliflozin 5 mg or placebo must be stored at room temperature at 15-25°C.

Dapagliflozin 5 mg and placebo capsule supplies will be provided to study site and will only be released by the sponsor once all the appropriate regulatory and governance approvals are in place.

Further information on storage requirements and supply arrangements is provided in the study specific IMP Management and Accountability Manual.

A web-based system will be used for the assignment of dapagliflozin 5mg or placebo bottles at randomisation and at study visits where there is planned dispensing of the IMP or in the event that a participant requires replacement medicines.

Other medicines: Supplies of all other medicines and associated consumables will via usual NHS supply mechanisms.

8.4 Drug accountability requirements

The Investigator or designee must maintain accurate records of all study IMP movements for accountability purposes. They should include dates, quantities, batch numbers and expiry. Records must document adequately that:

- the patients were provided the doses specified by the protocol/amendment(s)
- all study drug provided was fully reconciled.

Unused study drug must not be discarded or used for any purpose other than the present study. Further information is provided in the study specific IMP Management and Accountability Manual.

8.5 Preparation and administration of semaglutide and dapagliflozin

Semaglutide (Ozempic®) is a solution for subcutaneous injection. It is administered once weekly, on the same day each week, into the subcutaneous tissues of the abdomen, thigh or upper arm. A participant may change the day of their weekly injection as long as it has been at least three days since their last injection. Thereafter, all doses should be administered once weekly on the new dosing day. The injection can be administered at any time of the day regardless of meals.

Participants must be advised to discard the injection needle after each injection and store the pen without an injection needle attached. This may help prevent blocked needles, contamination, infection, leakage of solution and inaccurate dosing. Semaglutide should not be used if the solution does not appear clear and colourless or almost colourless. The pen is designed to be used with NovoFine or NovoTwist disposable needles and pens are packed with an appropriate quantity of NovoFine Plus needles.

Detailed information on administration of semaglutide is provided in the current Summary of Product Characteristics and product Patient Information Leaflet.

Dapagliflozin (Forxiga®))and matched placebo are presented as a capsule for once daily oral administration.

8.6 Dosage schedules

Semaglutide will be administered and dosed as described. Participants will be commenced on 0.25 mg s.c once weekly before up-titration as tolerated to 1.0 mg s.c once weekly. Up-titration is quicker than is ordinarily the case in clinical practice in type 2 diabetes as discussed in Section 2.1 (22) There will be increased observation and clinical access for participants in the event of side-effects. Participants randomised to receive semaglutide will continue on the 1.0 mg s.c once weekly dose for the remainder of the study following uptitration provided they tolerate the dose as detailed in Section 7. If necessary, the dose may be adjusted.

If a participant misses a dose of semaglutide it should be administered as soon as possible and within 5 days after the missed dose. If more than 5 days have passed, the missed dose should be skipped, and

the next dose should be administered on the regularly scheduled day. In both cases participants, should then resume their regular once weekly dosing schedule.

Dapagliflozin and matched placebo will be commenced at 5mg orally once daily and uptitrated one week after commencement to a dose of 10mg which will thereafter be continued for the remainder of the trial provided they tolerate the dose. If necessary, the dose may be adjusted.

8.7 Dosage modifications

Dapagliflozin/placebo will be commenced at one capsule (5mg or placebo) orally once daily and uptitrated one week after commencement to a dose of two capsules (10mg or placebo) which will thereafter be continued for the remainder of the trial provided they tolerate the dose. If necessary, the dose may be adjusted. (See also Section 7.8 regarding treatment discontinuation) Dapagliflozin or placebo capsules can be taken at any time of day with or without food. Capsules should be swallowed whole. If participants forget to take a dose they should be advised as follows:

- If it is 12 hours or more until the next dose, the dose should be taken as soon as remembered and the next dose taken at the usual time.
- If it is less than 12 hours until the next dose, the missed dose should be skipped. The next dose should be taken at the usual time.
- Participants should not take a double dose of dapagliflozin/placebo to make up for a forgotten dose.

8.8 Known drug reactions and interaction with other therapies

Participants with hypersensitivity to either active study medication are excluded from the trial. There are no significant interactions between semaglutide and other prescribed medications other than with agents that can lower blood glucose levels. Participants on any other oral agents which can lower blood glucose levels meet Exclusion Criteria and will not be included in this trial. In combination with insulin and dapagliflozin, semaglutide may be associated with an increased risk of hypoglycaemia. Participants will be advised of the increased risk of hypoglycaemia and will be required to closely monitor their blood glucose levels for the duration of the trial. No interactions are currently listed between warfarin and semaglutide in the British National Formulary but in view of a theoretical interaction (based on protein binding), existing arrangements for INR testing will be reviewed for any participants prescribed warfarin and additional tests conducted if indicated according to the judgement of the investigators.

Dapagliflozin is known to increase the risk of hypoglycaemia when combined with other medications which also act to lower blood glucose levels. Dapagliflozin has a mild diuretic effect and therefore there is an increased risk of hypotension when dapagliflozin is combined with either medications which deplete circulating volume (e.g diuretics) or antihypertensive drugs. Dapagliflozin is however commonly prescribed with such medications in general medical practice. All participants will have their medications reviewed at the screening visit and educated regarding symptoms that may indicate dehydration/ hypovolaemia.

Dapagliflozin may increase renal lithium excretion leading to decreased blood lithium concentrations. Serum lithium concentrations should be monitored for participants eligible for randomisation to dapagliflozin/placebo prior to visit 10 (week 26, second randomisation), as clinically indicated thereafter, and in the event of dose changes, taking into account the planned dose increase at visit 11 (week 27).

If there is any concern of interaction with any of the IMPs, this will be discussed with the PI before a decision regarding their ability to participate is made.

COVID-19: COVID-19 infection and the clinical management in this therapeutic area is also rapidly evolving as new medicines are approved for use by local/national treatment guidelines. It is therefore not possible to comprehensively rule out the potential for clinically significant interactions between the study IMP(s) with current or future medicines for the acute treatment of COVID-19 and a pragmatic approach is required. Medicines for acute COVID-19 infection should not be unnecessarily withheld and participants should be managed wherever possible in line with local/national clinical guidelines for this patient group. The decision to follow a particular management plan for acute COVID-19 infection (which may include the temporary withdrawal of the IMP(s) should this be in the participant's best interest), is at the treating physician's discretion. Investigators should refer to the protocol and current IMP Summary of Product Characteristics for detailed information on known mechanisms for pharmacokinetic/pharmacodynamics interactions as well as the relevant COVID-19 treatment Summary of Product Characteristics (or equivalent) to inform management decisions. Any mitigations, such as additional patient monitoring, must be documented in the participant's medical notes.

Further ad-hoc advice can also be obtained from the Sponsor/Chief Investigator and https://www.covid19-druginteractions.org/ Investigators must be vigilant at all times to the possibility of interactions and report in line with protocol pharmacovigilance requirements. Concomitant use of other medicines for COVID-19 must be recorded in the participant's CRF. Investigational treatments for acute COVID-19 treatment are not permitted without prior approval from the sponsor.

8.9 Concomitant medication

Participants should not receive any adjunct glucose-lowering therapy other than as per the randomisation schedule (e.g. with either of the active study medications).

8.10 Trial restrictions

Contraindications will follow SmPC information for Ozempic® and Forxiga. Acceptable forms of contraception for WOCBP participating in the trial are listed in Appendix 2. There is no requirement for contraception for male participants.

8.11 Assessment of compliance

Treatment adherence will be assessed by the trial medical and nursing team when participants attend for scheduled study visits.

8.12 Name and description of each Non-Investigational Medicinal Product (NIMP)

Insulin therapy is a non-investigational medicinal product in this trial. The trial is in a population who have had a diagnosis of type 1 diabetes for at least one year: therefore all participants will already be established on insulin therapy. The regimen and dosage required will by differ between participants. Participants will be using a combination of long acting (basal) insulin and shorter acting (bolus) insulin or CSII (insulin pump). As in standard of care, dose titration will depend on diet, blood glucose levels and exercise. It is anticipated that dosage adjustments will be required to achieve optimal glycaemic control and maintain participant safety.

There are pre-defined guidelines for insulin titration in this study. Insulin dose reductions should not be greater than 10% (5% if HbA1c is<7%) from current total insulin dose or more than 20% from the baseline level recommended following review at the randomisation visit and then from the new baseline level on review at 26 weeks (second randomisation). Participants requiring an insulin dose

reduction > 20% to a total insulin <0.4 U/kg will be monitored closely for insulin dose, carbohydrate intake, ketones and blood glucose. Study medication can be temporarily discontinued, if necessary, for one-to-two weeks. If low insulin requirements remain, the participant will be withdrawn from study (see Section 7.8).

9 PHARMACOVIGILANCE

9.1 Definitions

Term	Definition
**Adverse Event (AE)**	Any untoward medical occurrence in a participant to whom a medicinal product has been administered, including occurrences which are not necessarily caused by or related to that product.
**Adverse Reaction (AR)**	An untoward and unintended response in a participant to an investigational medicinal product which is related to any dose administered to that participant.<br>The phrase "response to an investigational medicinal product" means that a causal relationship between a trial medication and an AE is at least a reasonable possibility, i.e. the relationship cannot be ruled out.<br>All cases judged by either the reporting medically qualified professional or the Sponsor as having a reasonable suspected causal relationship to the trial medication qualify as adverse reactions.
**Serious Adverse Event (SAE)**	A serious adverse event is any untoward medical occurrence that:<br>1. results in death<br>2. is life-threatening<br>3. requires inpatient hospitalisation or prolongation of existing hospitalisation<br>4. results in persistent or significant disability/incapacity<br>5. consists of a congenital anomaly or birth defect<br>Other 'important medical events' may also be considered serious if they jeopardise the participant or require an intervention to prevent one of the above consequences.<br>NOTE: The term "life-threatening" in the definition of "serious" refers to an event in which the participant was at risk of death at the time of the event; it does not refer to an event which hypothetically might have caused death if it were more severe*.
**Serious Adverse Reaction (SAR)**	An adverse event that is both serious and, in the opinion of the reporting Investigator, believed with reasonable probability to be due to one of the trial treatments, based on the information provided.
**Suspected Unexpected Serious Adverse Reaction (SUSAR)**	A serious adverse reaction, the nature and severity of which is not consistent with the information about the medicinal product in question set out:<br>• in the case of a product with a marketing authorisation, in the summary of product characteristics (SmPC) for that product<br>• in the case of any other investigational medicinal product, in the investigator's brochure (IB) relating to the trial in question
**Reference Safety Information**	The information used for assessing whether an adverse reaction is expected. This is contained in either the investigator's brochure or the summary of product characteristics

*Note: “Severe” is often used to describe intensity of a specific event, which may be of relatively minor medical significance. “Seriousness” is the regulatory definition supplied above.

9.2 Operational definitions for (S)AEs

Adverse events (AEs) will be recorded, notified, assessed, reported, analysed and managed in accordance with the Medicines for Human Use (Clinical Trials) Regulations 2004 (as amended) and this protocol.

All Serious Adverse Events (SAEs) occurring during the trial will be recorded within the eCRF. Non-serious AEs occurring during the trial will be recorded within the eCRF if they are considered to be related to trial medication, i.e. adverse reactions (ARs).

Exclusions from SAE reporting

Hospitalisation for the following reasons will not be considered to be SAEs:

- Treatment which was elective or pre-planned, for a pre-existing condition not associated with any deterioration in condition, e.g. pre-planned hip replacement operation which does not lead to further complications.
- Any admission to hospital or other institution for general care for less than 24 hours where there was no deterioration in condition.
- Treatment on an emergency, outpatient basis for an event **not** fulfilling any of the definitions of serious as given above and not resulting in hospital admission.
- COVID-19 infection unless results in severe illness with hospital admission.

9.3 Recording and reporting of AEs, Adverse Events of Special Interest, SAEs and SUSARs

All AEs occurring during the trial that are observed by the Investigator or reported by the participant will be recorded in the participant’s medical records whether or not attributed to trial IMP. AEs will be recorded from randomisation until the later of 30 days post cessation of trial treatment or the end of the study.

With the exception of those noted in section 9.3.1 below, adverse reactions will be recorded in a log provided in the eCRF.

Adverse Events of Special Interest, SAEs and SUSARs will be recorded in the participant’s medical records and in a specific section of the eCRF from the point of randomisation until the later of 30 days post cessation of trial treatment or the end of the study.

9.3.1 Adverse Events of Special Interest

Ketosis is a known adverse effect of dapagliflozin (see Section 2.1b; also (i) below). Hypoglycaemia is a recognised adverse effect with both semaglutide and dapagliflozin when used in combination with insulin [see (ii) below] and is defined as moderate in intensity (Level 2) when blood glucose is < 3.0 mmol/L. Thus ketosis and Level 2 hypoglycaemia events will be recorded as Adverse Events of Special Interest (AESIs).

AESIs will be recorded within the relevant section of the eCRF but do not require reporting in an expedited manner (unless the event also meets the regulatory definition of an SAE). The following information will be recorded:

- Nature of the event
- Event duration (start and end dates, if applicable
- Relationship to study drug
- Outcome

Adverse Events of Special Interest will be monitored and followed up (if applicable) until the event has resolved or a final outcome has been reached.

All Adverse Events of Special Interest must be assessed for seriousness prior to recording within the eCRF. Those meeting the definition of an SAE should also be reported accordingly as per Section 9.3.2.

(i) Ketosis and diabetic ketoacidosis

This trial will be conducted in participants who have type 1 diabetes. All participants will be taking insulin. In this condition, due to the near absence of endogenous insulin, at a cellular level there is an inability to incorporate glucose into normal intra-cellular energy yielding pathways. In this context, alternative energy yielding pathways are sought including ketone body production from fatty acids in the liver. Ketones are clinically important as they easily dissociate hydrogen ions upsetting physiologically regulated acid base balance. As ketone levels rise, there is initially a state of high blood ketone levels without significant acidosis (ketosis); if this develops further, diabetic ketoacidosis (DKA) may develop. DKA requires urgent medical input. Ketones can be easily measured via a simple urine test or fingerstick capillary blood measurement. Participants eligible for this trial will already be trained in how to do this. As discussed in Background ((1) above), dapagliflozin, one of the IMPs in this trial, is known to increase the risk of ketosis and ketoacidosis and this can occur at lower blood glucose levels than in those who are on insulin treatment only [euglycaemic DKA] (13,14). Given this known risk, ketosis and DKA are included in the safety endpoints and as such will be recorded in the eCRF as Adverse Events of Special Interest.

DKA is defined clinically and biochemically as a combination of a state of elevated ketone levels (urine ++ or higher, and/or blood > 1.5 mmol/L) in combination with biochemical evidence of a state of metabolic acidosis (pH ≤7.30 or serum bicarbonate < 15 mmol/L). This is as defined by International Consensus Guidelines (39). DKA also varies in severity. In clinical practice the diagnosis is made when a person with type 1 diabetes has elevated ketone levels and usually – although not exclusively – elevated blood glucose levels. The diagnosis is made biochemically by assessing acid base balance through blood testing. This corresponds to the following severity classifications:

- mild DKA: pH 7.25-7.30 (H^+ 50 – 56) or bicarbonate (15 -17.9 mmol/L)
- moderate DKA: pH 7.00-7.24 (H^+ 57 – 100) or bicarbonate 10 – 14.9 mmol/L)
- severe DKA: pH <7.00 (H^+ > 100) or bicarbonate < 10 mmol/L (39)

In addition to the above, the highest level of either blood or urine ketones measured, the serum bicarbonate (if measured) and blood pH (if measured) will be recorded for each episode. Given the known risk of DKA, all participants will be made aware of this condition. Patient diaries and Patient Alert Cards will summarise advice for management of high ketone levels including an emergency ketosis management protocol (STICH Protocol – see Appendix 3) (37). All participants will be

educated as to the scenarios when this should be activated and this will also be included on the Patient Alert Card which will have the contact details of the study team and also contact details for out of hours health services. Participants will be asked to carry this card at all times.

(ii) Hypoglycaemia

Hypoglycaemia is a low blood glucose level. Hypoglycaemia occurs in diabetes owing to difficulty in precisely matching insulin dosing with the complex factors associated with carbohydrate intake and physical activity. When too much insulin relative to carbohydrate intake is administered, blood glucose levels can drop to a level which is too low. Most people with type 1 diabetes can recognize this phenomenon due to a combination of symptoms which alert them and prompt self-checking of their capillary blood glucose level. Symptoms include weakness, malaise, headache, tremulousness, diaphoresis, palpitations and mild confusion. These are known as autonomic symptoms as they are mediated by activation of the autonomic nervous system as a response to the biological stress of low serum blood glucose. As hypoglycaemia progresses, however, symptoms progress to include confusion and agitation. If untreated this can lead to coma. These symptoms are known as neuroglycopenic symptoms as they are mediated by a lack of glucose in neural cells leading to a confused state.

Hypoglycaemia is also well defined by International Consensus (39). Like DKA, it can be classified in terms of severity. This can be based either on the absolute capillary glucose value and/or clinical features and is classed into stages:

- Level 1 (mild) hypoglycaemia: Blood glucose is 3.0 – 3.8 mmol/L inclusive. At each visit the number of episodes since the last visit during which blood glucose was within this range (but not lower) will be recorded on the "Fasting Blood Glucose" tab of the eCRF following review of the participant's study diary and (if applicable) FGM/ CGM download.
- Level 2 (moderate) hypoglycaemia: Blood glucose is < 3.0 mmol/L At each visit the number of episodes since the last visit during which blood glucose was within this range at any time will be recorded on the AESI tab of the eCRF following review of the participant's study diary and (if applicable) FGM/ CGM download.
- Level 3 (severe) hypoglycaemia: A person is unable to function because of mental or physical changes due to hypoglycaemia and requires assistance from another person to recover. These events will be recorded as SAEs (see below) independent of recording of blood glucose according to the opinion of the site Principal Investigator as by definition they jeopardise the participant or require an intervention to prevent life-threatening consequences.

The IMPs in this trial are licensed clinically in type 2 diabetes to lower blood glucose levels. It therefore follows that when used in combination with insulin there may be an increased risk of low blood glucose levels. This was the case with the once daily GLP-1 analogue liraglutide in phase 3 trials (8,9), but not in the trials with dapagliflozin (13,14). All participants will have this risk discussed with them, will be performing increased blood glucose monitoring than is the case in usual care, and will be frequently reviewed to adjust insulin dosing in order to mitigate the risk of low blood glucose levels.

9.3.2 Site Reported Serious Adverse Events (SAE)

SAEs will be recorded within the eCRF from the date of randomisation until the later of 30 days post cessation of trial treatment or the end of the study.

Full details of SAEs will be recorded in the electronic Case Report Form. The following information will be collected:

- full details in medical terms and a case description
- seriousness criteria

- event duration (start and end dates, if applicable)
- action taken
- outcome
- causality (i.e. relatedness to trial drug / investigation), in the opinion of the investigator
- if related, whether the reaction would be considered expected or unexpected.

Any change of condition or other follow-up information should be added to the eCRF and forwarded to the Sponsor (if reportable SAE) as soon as it is available or at least within 24 hours of the information becoming available. Events will be followed up until the event has resolved or a final outcome has been reached.

Reporting of suspected/ confirmed COVID 19 infection

If a patient presents with clinical signs and symptoms consistent with COVID-19 and a test has been performed or requested:

- If test is positive, record "COVID-19 confirmed" in the diagnosis field
- If test is negative, record the SAE signs and symptoms and/or other diagnosis in the diagnosis field.

If a test has not been performed or result is not available and signs and symptoms, as judged by the investigator, are highly suspicious of COVID-19 infection, record "COVID-19 suspected" in the diagnosis field

If other concurrent diagnoses, e.g. pneumonia, please record within the narrative of the SAE.

COVID-19 vaccination and reporting

Where a deployed COVID-19 vaccine is suspected to be involved in the onset of a reported event it should be recorded as a concomitant medication. A causal relationship between the vaccine and the event, including potential drug interactions should be assigned by the reporting investigator.
If a reported event is suspected to be due to a deployed COVID-19 vaccine alone reporting investigators should ensure that standard Yellow Card reporting procedures are followed.

Assessment of Adverse Events

All adverse events must be assessed by the reporting site for seriousness**.** All SAEs must also be assessed for causality and expectedness with reference to this protocol and the Reference Safety Information (RSI).

Assessment of seriousness

An adverse event will be considered serious if it:

1. Results in death
2. Is life threatening
3. Requires hospitalisation or prolongation of existing hospitalisation
4. Results in persistent or significant disability or incapacity
5. Consists of a congenital anomaly or birth defect

1. Required intervention to prevent one of the above.
2. Is a serious Adverse Event of Special Interest.

Assessment of causality i.e. does the event have a “reasonable causal relationship” with trial IMPs. The following categories are used:

- **Yes** - The causal and temporal relationship between the event and the absence of a more likely explanation suggest that the trial IMP is the most likely cause.
- **No** - The event is not considered related to the trial IMPs.

Causality will be assigned for each IMP the participant is randomised to receive.

Should an SAE be considered unrelated to the administration of either trial drug then the causality of the event will be collected taking into consideration other factors, for example concomitant medications or co-morbidities, etc.

Assessment of causality is the responsibility of the Principal Investigator or medically-qualified designee(s) at each site. The CI, their delegate(s), or the Sponsor may provide an opinion regarding causality but cannot downgrade the PI’s assessment.

Assessment of expectedness

Should the event be considered to be related to the trial IMP(s), an assessment should be made regarding the expectedness of the reaction, i.e. whether the reaction is a recognised adverse effect of the IMP(s).

.

Expected: consistent with the relevant product information documented in the RSI(s).
Unexpected: not consistent with the relevant product information documented in the RSI(s).

The CI, their delegate(s), or the Sponsor have overall responsibility for assigning expectedness as per the RSI(s) and provide the final assessment of the event. The reporting investigator may provide an opinion on the expectedness of an event and this may be taken into account.

SAEs occurring within the USA and the UK

The US Sponsor is responsible for assigning causality and expectedness for any AEs, SAEs and SUSARs that occur within the USA. Where a SUSAR is identified by the US Sponsor this will be forwarded to the UK Sponsor for review within one working day. Following review by the UK Sponsor, appropriate US SUSARs will be subject to expedited reporting to the MHRA as per UK regulatory requirements. Similarly, where a SUSAR is identified by the UK sponsor, this will forwarded to the US Sponsor for review within two working days.

Recording and reporting of SAEs
All SAEs arising during the clinical trial will be recorded in the eCRF soon as reasonably practicable and in any event within 24hours of first becoming aware of the event. Any follow-up information should also be reported.

If recording in the eCRF is not possible a paper SAE form should be completed:

1. The SAE form is downloaded from www.glasgowctu.org, printed off, completed and signed. The form is then faxed to the Glasgow Clinical Trials Unit Pharmacovigilance (PV) Office on

+44(0)141 357 5588. As an alternative, a scanned copy of the SAE form may be emailed to: pharmacovig@glasgowctu.org. If this website is unavailable a paper copy of the SAE form should be filed in the Investigator Site File at each site pending resolution.

2. If necessary a verbal report can be given by contacting the PV Office on +44(0)141 330 4744. This must be followed up as soon as possible with an electronic or written report.

Reporting to sponsor

All SAEs will be considered reportable to the Sponsor PV office from the time of randomisation until 30 days following permanent discontinuation of all trial IMP(s). SAEs occurring following 30 days post cessation of treatment are not reportable to the Sponsor unless an SAE is considered related to IMP(s)

9.3.3 Suspected Unexpected Serious Adverse Reactions (SUSARs)

Any SAE assessed as related to trial IMP(s) by the PI or delegate, and unexpected (not documented as an expected reaction to the IMP(s) within the RSI(s)) by the CI, their delegate, or the Sponsor, will be classified as a SUSAR and therefore subject to expedited reporting to the Medicines and Healthcare products Regulatory Agency (MHRA) and the Research Ethics Committee (REC). Should the CI disagree with the PI's assessment of causality, both opinions will be provided on the report. Where the CI or UK Sponsor disagrees with the US Sponsor's assessment of expectedness both opinions will also be provided on the report.

The Sponsor will inform the MHRA of all SUSARs that occur within the trial and the REC of all SUSARs that occur within the UK within the required expedited reporting timescales:

- **Fatal or life threatening SUSARs** - no later than 7 days of Sponsor awareness that the case fulfils the criteria for a fatal or life threatening SUSAR, and any follow up information within a further 8 days.
- **All other SUSARs** - no later than 15 days of Sponsor awareness that the case fulfils the criteria for a SUSAR.

The sponsor will report SUSARs to the MHRA via the MHRA eSUSAR reporting system and to REC by email with accompanying CTIMP Safety Report Form.

9.3.4 Unblinding for SUSAR reporting purposes

In the event of a potential SUSAR, the sponsor (but not the investigators) will be unblinded to facilitate appropriate reporting to the MHRA and REC.

9.4 Responsibilities for Safety Reporting and Review

This section details the responsibilities for reporting and reviewing safety information arising from the trial.

Principal Investigator (PI):

- Attendance at the initiation meeting/teleconference
- Training of new members of the trial team in the protocol and its procedures
- Ensuring that the ISF is accurately maintained

- Dissemination of important safety or trial-related information to all stakeholders at their site
- Checking for AEs and ARs when participants attend for treatment/follow-up
- Ensuring that AEs are recorded and reported in line with the requirements of the protocol
- Ensuring that all SAEs are recorded and reported to the Sponsor within 24 hours of becoming aware of the event, and providing further follow-up information as soon as available
- Using medical judgement in assigning causality of SAEs
- Providing an opinion on the expectedness of an SAE with reference to the trial protocol and Reference Safety Information
- Using definitions in this protocol, flagging events of special interest.

Chief Investigator (CI)

- Providing clinical oversight of the safety of patients participating in the trial, including an ongoing review of the risk/benefit
- Providing clinical review of section 4.8 of the representative SmPCs on an annual basis to define the Reference Safety Information for the trial
- Using medical judgement to confirm seriousness and causality of all SARs
- Reviewing expectedness of related SAEs with reference to the trial protocol and Reference Safety Information
- Preparing the clinical sections and final sign-off of the Development Safety Update Report (DSUR)
- Using definitions in this protocol, confirming events of special interest

Sponsor:

- Central data collection and verification of AEs, SAEs, SARs and SUSARs according to the trial protocol
- Reporting safety information to the CI or delegate for the ongoing assessment of the risk / benefit
- Reporting safety information to the independent oversight committees identified for the trial (Independent Data Monitoring Committee (IDMC) and / or Trial Steering Committee (TSC)) according to the Trial Monitoring Plan.
- Expedited reporting of SUSARs to the Competent Authority (MHRA in UK) and REC within required timelines.
- Notifying Investigators of SUSARs that occur within the trial.
- Checking for (annually) and notifying PIs of updates to the Reference Safety Information for the trial.
- Preparing standard tables and other relevant information for the DSUR in collaboration with the CI and ensuring timely submission to the MHRA and REC.

Trial Steering Committee:

In accordance with the Charter for the TSC, periodically reviewing recruitment and the overall progress of the trial and liaising with the IDMC and sponsor regarding safety issues.

Independent Data Monitoring Committee:

In accordance with the Charter for the IDMC, periodically reviewing unblinded safety data in individual cases and to determine patterns and trends of events, or to identify safety issues, which would not be apparent on an individual case basis, reporting concerns to the TSC and sponsor.

9.5 Pregnancy reporting

Pregnancy is not considered an AE unless a negative or consequential outcome is recorded for the mother or child/foetus. If the outcome meets the serious criteria, this would be considered an SAE and must be reported as per SAE reporting procedure above.
Any **pregnancy** occurring in a female trial participant or female partner of a male trial participant who becomes pregnant while participating in the Trial will be reported by the PI (or designee) to the Chief Investigator and the sponsor using the sponsor Pregnancy Reporting Form (available at http://www.glasgowctu.org/) within two weeks of the PI first becoming aware of the pregnancy.
The trial participant will also be followed up to determine the outcome of the pregnancy and follow-up information forwarded to the PV office. Any resulting SAEs should be reported as per SAE reporting procedure above.

9.6 Overdose

Any IMP dose which is not administered in accordance with the Protocol should be reported to the sponsor.
If an SAE is associated with an overdose ensure that the overdose if fully described in the SAE report form.

9.7 Reporting urgent safety measures

If any urgent safety measures are taken the CI/Sponsor will phone the MHRA's Clinical Trial Unit on 020 3080 6456, ideally within 24 hours. This will be followed up no later than 3 days from the date the measures are taken, giving written notice to the MHRA (who will advise the format required) and the relevant REC of the measures taken and the circumstances giving rise to those measures. A substantial amendment must also be submitted to the MHRA.

9.8 The type and duration of the follow-up of participants after adverse events.

Adverse events and reactions will be recorded, reported and followed up in line with this protocol until study completion or for a minimum of 30 days after participant's last dose of the IMP, whichever is later.
Any SUSAR identified will be reported to the Sponsor and to the Regulatory Authorities irrespective of how long after IMP administration the reaction has occurred.

9.9 Development safety update reports

A Development Safety Update Report (DSUR) will be submitted once a year, or on request, to MHRA and REC until the trial is declared ended. The report will be submitted within 60 days of the anniversary of the issue of the Clinical Trials Authorisation for the trial. The DSUR will be prepared by the sponsor (PV Office) in liaison with the CI and submitted by the sponsor (PV Office). The DSUR will include only those SAEs reported to the co-sponsors; that is: SAEs and SUSARs occurring within the UK and SUSARs originating in the USA.

10 STATISTICS AND DATA ANALYSIS

10.1 (a)Sample size calculation

The sample size in Protocol versions 1.0 - 5.0 was as follows: "This is based on our current data showing that in type 1 diabetes, treatment with a GLP-1 agonist, liraglutide (1.2 and 1.8mg doses) for three months leads to HbA1c suppression by an average of 0.6% (SD=0.5%). Dapagliflozin was recently shown to reduce HbA1c by 0.45% in type 1 diabetes patients (13). Additionally, empagliflozin treatment for 8 weeks was shown to reduce HbA1c by 0.4% in type 1 diabetics (20, 21) and our

current data showing additional improvement in HbA1c by an additional 0.66% at 12 weeks when dapagliflozin was added to liraglutide and insulin therapy. Conservatively estimating that the combination therapy will suppress HbA1c by 0.5% (SD≤0.7%) compared to semaglutide alone, a sample size of 38 (32 evaluable patients) in each group will be sufficient to provide 80% power at a 5% significance level, taking in consideration a 15% dropout rate. 50% of participants will be recruited in Glasgow, UK and 50% at State University of New York at Buffalo, USA."

10.1 (b) Revised sample size calculation (from Protocol version 6.0)

At their meeting in May 2023, the TSC asked the trial statisticians to review the sample size required on the basis of blinded review of primary outcome data (SD of change of HbA1c, correlation of HbA1c at baseline and end of Period 2). In August 2023 the trial statisticians recommended on the basis of this review a reduction in the sample size by a factor of 0.59 i.e. from 32 to 19 evaluable patients per group. This recommendation was reviewed by the IDMC in August 2023, by the TSC in September 2023 and at an additional IDMC meeting in October 2023 (at which additional information was provided on changes in insulin delivery devices during the trial). At this time, the IDMC unanimously agreed that the study should continue, aiming conservatively for a revised sample size in Period 2 of around 23/ evaluable participants per group for dapagliflozin vs placebo. This reduces the recruitment target for first randomisation from n=114 to n=82 (i.e. 23/32*114) maintaining the same assumptions on power, significance level and drop-out (amended accordingly on page 7 above).

The IDMC further required a formal interim analysis on all those with mature data on the primary outcome at around the end of April 2024 with recruitment to continue unless formally stopped by the funder following a subsequent recommendation by the TSC. The study should continue after interim analysis: (a) unless there is overwhelming evidence of benefit, or evidence of futility; and (b) if the study has recruited sufficient numbers between Nov 23 – Apr 24 to make reaching the new target in a reasonable time feasible within budget (minimum 8-10 new participants recruited)). The IDMC agreed to specify the exact detail of the interim analysis in time for this analysis to be conducted, to review the results and make a recommendation to the TSC.

This recommendation was accepted unanimously by the independent members of the TSC in November 2023.

10.2 Anticipated recruitment rate

Originally one year (one participant per week). The rate has been considerably slower due mainly to the COVID-19 pandemic and global semaglutide supply issues. 60 participants had been randomised by November 2023.

10.3 Statistical analysis

The primary objective of the proposed research is to evaluate the effect of addition of dapagliflozin to semaglutide on a background of insulin treatment in type 1 diabetes patients on HbA1c at six months following start of dapagliflozin. Transformations of the data in order to meet statistical assumptions will be considered. Statistical analyses will use linear regression models to compare treatment groups, with adjustment for baseline values of study outcomes, as well as factors used to stratify the randomisation scheme. Tests will be two-sided and tested at the 5% significance level. Analyses will be carried out under the intention to treat principle, that is, according to randomised treatment allocation, regardless of treatment adherence.

A detailed Statistical Analysis Plan will be completed and signed off before database lock. Study statisticians will develop and validate the analysis programs without access to randomised treatment

allocations. After database lock, the treatment codes will be released and the final analysis will be carried out.

10.4 Subgroup analyses

These will be specified in the Statistical Analysis Plan. Subgroup analyses will be implemented by extending regression models to include interactions between treatment and potential treatment effect moderators.

10.5 Interim analysis and criteria for the premature termination of the trial

No interim analysis was initially planned but an interim analysis was recommended by the IDMC in November 2023 – to be conducted in April 2024 (see section 10.1(b) above). Unblinded trial data will be reviewed on an ongoing basis by the IDMC. The primary role of the IDMC will be to protect the interests of the patients. The IDMC may recommend to the TSC and Co-Sponsors that the study should stop prematurely because of concerns about patient safety. The IDMC will meet approximately every six months. The IDMC will take into account all results and the consistency and biological plausibility of the findings in making any recommendation. The final decision on continuing or stopping the trial will lie with the TSC/Co-Sponsors.

10.6 Subject population

All analyses will be carried out on an intention to treat basis, using the randomised treatment allocation.

10.7 Procedure(s) to account for missing or spurious data

Data will be analysed without imputation in the first instance. Multiple imputation procedures will be used to assess the sensitivity of selected results to missing data, according to the Statistical Analysis Plan.

10.8 Other statistical considerations.

The Statistical Analysis Plan (SAP) will be maintained as a version controlled document and will be signed off before database lock. The SAP and other statistical documentation (according to Glasgow Clinical Trials Unit Standard Operating Procedures) will contain full details of all analyses along with assumptions and procedures for handling problematic or incomplete data (e.g. incomplete dates).

11 DATA HANDLING

11.1 Source Documentation

ICH GCP defines source data as: ‘All information in original records and certified copies of original records of clinical findings, observations or other activities in a clinical trial necessary for the reconstruction and evaluation of the trial’. In this study, the location of the majority of the source data will be the hospital's medical records including subject case notes, laboratory records and ECGs. The source data transcribed into the eCRF from the medical records must be accurate and verifiable. For questionnaires completed by trial subjects, the completed questionnaires will be regarded as the source data location. In cases where data are transcribed directly into the eCRF and no other paper or electronic source exists, then the eCRF will be considered the source record. In these cases, these data should be prospectively documented in the medical records to ensure a full record of the trial is available at site. Identification of unverifiable data entries will be reported as non-compliances.

11.2 Data collection

An eCRF, developed by the Robertson Centre for Biostatistics, will capture all data required to meet this protocol's requirements. Access to the eCRF will be restricted, via a study-specific web portal, and only authorised site-specific personnel will be able to make entries to their patients' data via the web portal. The Investigator, or his/her designee will be responsible for all entries into the eCRF and will confirm that the data are accurate, complete and verifiable. Data will be stored in a MS SQL Server database.
Direct access to the web portal will be granted, on request, to authorised representatives from the Sponsor, host institution and the regulatory authorities to permit trial-related monitoring, audits and inspections.

11.3 Data Validation

Where it is practical, data will be validated at the point of entry into the eCRF. Any additional data discrepancies will be flagged to the investigator and any data changes will be recorded to maintain a complete audit trail (reason for change, date change made, who made change).

11.4 Data Security

The Robertson Centre for Biostatistics systems are fully validated in accordance with industry and regulatory standards, and incorporate controlled access security. High volume servers are firewall protected and preventative system maintenance policies are in place to ensure no loss of service or data. Web servers are secured by digital certificates. Data integrity is assured by strictly controlled procedures, including secure data transfer procedures. Data are backed up on-site nightly and off-site to a commercial data vault weekly. The Robertson Centre for Biostatistics has an ISO 9001:2008 quality management system and ISO 27001:2013 for Information Security, and is regularly inspected against the standards by the British Standards Institution.

11.5 Archiving

The Trial Master File will be archived by the Co-Sponsors at the end of the trial for a minimum period of five years.

Archiving of Site Files will also be for a minimum of seven years from completion of the trial, and this action will be delegated to the sites in the Clinical Trial Site Agreement that will be put in place between Co-Sponsors and Sites. Sites will be notified by the Co-Sponsors when Site files can be archived.

Destruction of site files can only take place with the approval of the Co-Sponsors.

12 MONITORING, AUDIT & INSPECTION

Monitoring will be conducted by NHS Greater Glasgow and Clyde (GG&C) Monitor(s) in accordance with local Standard Operating Procedures. The level, frequency, objectives and priorities of monitoring will be based on the outcome of the completed Risk Assessment, and clearly documented in the Monitoring Plan which will be approved by the members of the TMG and the NHS GG&C Lead Monitor and/or Research Governance Manager. The Monitoring Plan is subject to updates during the life of the study in line with Protocol Amendments.

13 ETHICAL AND REGULATORY CONSIDERATIONS

13.1 Research Ethics Committee (REC) review & reports

Before the start of the trial, approval will be sought from a REC for the trial protocol, informed consent forms and other relevant documents e.g. advertisements and GP information letters.
Substantial amendments that require review by REC will not be implemented until the REC grants a favourable opinion for the study (it is noted that amendments may also need to be reviewed and accepted by the MHRA and/or NHS R&D departments before they can be implemented in practice at sites).
All correspondence with the REC will be retained in the Trial Master File/Investigator Site File.
An annual progress report (APR) will be submitted to the REC within 30 days of the anniversary date on which the favourable opinion was given, and annually until the trial is declared ended (this is the Chief Investigator's responsibility).
The Chief Investigator will notify the REC of the end of the study.
If the study is ended prematurely, the Chief Investigator will notify the REC, including the reasons for the premature termination
Within one year after the end of the study, the Chief Investigator will submit a final report with the results, including any publications/abstracts, to the REC.

13.2 Peer review

The study protocol has been developed with expert and independent feedback from the JDRF (New York) and during application for funding underwent peer review by independent experts.

13.3 Public and Patient Involvement

The study was designed with input from people with type 1 diabetes via the funder, JDRF.

13.4 Regulatory Compliance

The trial will not commence until a Clinical Trial Authorisation (CTA) is obtained from the MHRA.
The protocol and trial conduct will comply with the Medicines for Human Use (Clinical Trials) Regulations 2004 and any relevant amendments.
Before any site can enrol patients into the trial, the Chief Investigator/Principal Investigator or designee will apply for NHS permission from the site's Research & Development (R&D) department.

For any amendment that will potentially affect a site's NHS permission, the Chief Investigator/ Principal Investigator or designee will confirm with that site's R&D department that NHS permission is ongoing (note that both substantial amendments, and amendments considered to be non-substantial for the purposes of REC and/or MHRA may still need to be notified to NHS R&D).

13.5 Protocol compliance

Prospective, planned deviations or waivers to the protocol are not allowed under the UK regulations on Clinical Trials and must not be used e.g. it is not acceptable to enrol a participant if they do not meet the eligibility criteria or restrictions specified in the trial protocol. Accidental protocol deviations can happen at any time. They must be adequately documented on the relevant forms and reported to the Chief Investigator, Sponsor and GCTU immediately. Deviations from the protocol which are found to frequently recur are not acceptable, will require immediate action and could potentially be classified as a serious breach.

13.6 Notification of Serious Breaches to GCP and/or the protocol

A "serious breach" is a breach which is likely to affect to a significant degree –

1. the safety or physical or mental integrity of the subjects of the trial; or
2. the scientific value of the trial

If any of the above occurs then the CI and Sponsor will be notified. The sponsor will notify the appropriate authorities in writing of any serious breach in accordance with their standard operating procedures.

13.7 Data protection and patient confidentiality

All investigators and trial site staff must comply with the requirements of the GDPR with regards to the collection, storage, processing and disclosure of personal information and will uphold the Act's core principles.

- Only those individuals who require to see personal information i.e. site research team staff or the study monitor, as appropriate, will be able to view them. All electronic data will be held securely in accordance with ISO 27001:2013 at the Robertson Centre for Biostatistics, part of the Glasgow Clinical Trials Unit. All Centre staff are required to sign confidentiality agreements and to follow Standard Operating Procedures in accordance with Good Clinical Practice and ISO certification.
- The trial data managers, statisticians, health economists or any other staff who will perform data related tasks will only be able to access depersonalised data where the participant's identifying information is replaced by a unique study identifier.
- Only those that have been trained and approved will be able to enter or view any data via the web portal. Each site can only see their own patients' data. Patient consent forms will be stored at the study site in a secure location accessible only to study teams.

13.8 Indemnity

The Co-Sponsors (University of Glasgow and Greater Glasgow Health Board) will ensure that provision has been made for insurance or indemnity to cover the liability of the investigator and sponsor which may arise in relation to the clinical trial in accordance with Part 2 (14) of Schedule 1 to SI 2004/1031.

13.9 Amendments

Any change in the study protocol will require an amendment. Any proposed protocol amendments will be initiated by the CI following discussion with the Sponsor and TSC and any required amendment forms will be submitted to the regulatory authority, ethics committee and Sponsor. The Sponsor will determine whether an amendment is non-substantial or substantial. All amended versions of the protocol will be signed by the CI and Sponsor representative. Following a substantial amendment, favourable opinion/approval must be sought from the original reviewing REC, MHRA (where appropriate) and Research and Development (R&D) office prior to implementation. The Chief Investigator will be responsible for informing the Trial Management Group of all protocol amendments.

13.10 Post trial care

At the end of the trial, participants will be returned to usual care as defined by local and national guidelines at that time. The results of the trial may of course have an impact on these guidelines and the future care of patients with type 1 diabetes.

13.11 Access to the final trial dataset

During the trial and in the period prior to publication of the main study results as described in the protocol, only the Glasgow CTU, Professor Paresh Dandona and Dr Husam Ghanim US Principal Investigators, University of Buffalo will have access to the full dataset. After that period, the trial Steering Committee will conduct further data analyses for a period of three years. After that time the Trial Steering Committee will consider requests from external parties for further analyses of the study data. Proposals that are scientifically well founded and have an academic basis and where relevant data extractions and analyses are appropriately funded will not be refused. These will be considered as collaborative exercises where the contributions related to study design, conduct, database creation and maintenance and data analysis will be recognised in authorship of any scientific publication. The approach we will take will be to minimise any possibility of breach of participant confidentiality. Normally this will be achieved by minimising data travel. However, for the purposes of individual patient meta-analysis and other reasons, data may be transferred to other sites. Such transfer will require assurances on information security systems at the sites that data are to be transferred to and will involve a legal data transfer agreement. A log of all data requests and subsequent data transfers will be held at the Glasgow CTU.

14 DISSEMINATION POLICY

14.1 Dissemination policy

The study database will be owned by the University of Glasgow and maintained on behalf of the Study investigators, represented by the Trial Steering Committee as it is constituted during and after the trial.

The study protocol and a description of the recruitment experience and participant baseline characteristics will be published before study completion. On completion of the trial, the database will be locked and analysed by staff of the Robertson Centre for Biostatistics, University of Glasgow. A final study report will be prepared and the results will be published in a major medical journal.

After the main study publications, study investigators will be invited to submit requests for further analyses of the study database. These will be reviewed and prioritised by a Publications Committee made up of the study grant holders and convened by the study co-CIs.

As funder of the trial, JDRF will have the right to see and comment on any results being submitted for publication. A maximum of 28 days will be given for review of major papers and 14 days for abstracts. Such comments will be considered by the Trial Steering Committee.

A lay summary of the main results of the trial will be prepared and provided to all participants via their study site investigators. Investigators may request a copy of the study data for their participants. Providing some or all of a patient's data to that individual is at their discretion.

14.2 Authorship eligibility guidelines and any intended use of professional writers

The main results of the study will be compiled, written up and published by the study grant holders and others taking responsibility for the study results (e.g. the statistician conducting the final analysis) on behalf of the TTT1 investigators. The TTT1 investigators will be listed in an Appendix and will include all site PIs, all committee members and key members of relevant study coordinating groups (including the Sponsor and Glasgow CTU).

15 REFERENCES

1. Konrad K, Datz N, Engelsberger I, Grulich-Henn J, Hoertenhuber T, Knauth B, et al. Current use of metformin in addition to insulin in pediatric patients with type 1 diabetes mellitus: an analysis based on a large diabetes registry in Germany and Austria. *Pediatr Diabetes.* 2014.
2. Vella S, Buetow L, Royle P, Livingstone S, Colhoun HM, and Petrie JR. The use of metformin in type 1 diabetes: a systematic review of efficacy. *Diabetologia.* 2010;53(5):809-20.
3. Vella S, Buetow L, Royle P, Livingstone S, and Petrie JR. Metformin in type 1 diabetes reduces insulin requirements without significantly improving glycaemic control. Reply to Schatz H [letter]. *Diabetologia.* 2011;54(1):203-4.
4. Varanasi A, Bellini N, Rawal D, Vora M, Makdissi A, Dhindsa S, et al. Liraglutide as additional treatment for type 1 diabetes. *Eur J Endocrinol.* 2011;165(1):77-84.
5. Kuhadiya ND, Malik R, Bellini NJ, Patterson JL, Traina A, Makdissi A, et al. Liraglutide as additional treatment to insulin in obese patients with type 1 diabetes mellitus. *Endocrine practice : official journal of the American College of Endocrinology and the American Association of Clinical Endocrinologists.* 2013;19(6):963-7.
6. Kuhadiya ND, Dhindsa S, Ghanim H, Mehta A, Makdissi A, Batra M, et al. Addition of Liraglutide to Insulin in Patients With Type 1 Diabetes: A Randomized Placebo-Controlled Clinical Trial of 12 Weeks. *Diabetes Care.* 2016;39(6):1027-35.
7. Chaudhuri A, Ghanim H, Makdissi A, Green K, Abuaysheh S, Batra M, et al. Exenatide induces an increase in vasodilatory and a decrease in vasoconstrictive mediators. *Diabetes Obes Metab.* 2017;19(5):729-33.
8. Mathieu C, Zinman B, Hemmingsson JU, Woo V, Colman P, Christiansen E, et al. Efficacy and Safety of Liraglutide Added to Insulin Treatment in Type 1 Diabetes: The ADJUNCT ONE Treat-To-Target Randomized Trial. *Diabetes Care.* 2016;39(10):1702-10.
9. Ahren B, Hirsch IB, Pieber TR, Mathieu C, Gomez-Peralta F, Hansen TK, et al. Efficacy and Safety of Liraglutide Added to Capped Insulin Treatment in Subjects With Type 1 Diabetes: The ADJUNCT TWO Randomized Trial. *Diabetes Care.* 2016;39(10):1693-701.
10. Zinman B, Wanner C, Lachin JM, Fitchett D, Bluhmki E, Hantel S, et al. Empagliflozin, Cardiovascular Outcomes, and Mortality in Type 2 Diabetes. *N Engl J Med.* 2015;373(22):2117-28.
11. Petrie JR. SGLT2 inhibitors in type 1 diabetes: knocked down, but up again? *Lancet Diabetes Endocrinol.* 2017;5(11):841-3.
12. Mudaliar S, Armstrong DA, Mavian AA, O'Connor-Semmes R, Mydlow PK, Ye J, et al. Remogliflozin etabonate, a selective inhibitor of the sodium-glucose transporter 2, improves serum glucose profiles in type 1 diabetes. *Diabetes Care.* 2012;35(11):2198-200.
13. Kohan DE, Fioretto P, Tang W, List JF. Long-term study of patients with type 2 diabetes and moderate renal impairment shows that dapagliflozin reduces weight and blood pressure but does not improve glycemic control. Kidney Int. 2014 Apr;85(4):962-71. doi: 10.1038/ki.2013.356. Epub 2013 Sep 25. PMID: 24067431; PMCID: PMC3973038.
14. Dandona P, Mathieu C, Phillip M, Hansen L, Griffen SC, Tschope D, et al. Efficacy and safety of dapagliflozin in patients with inadequately controlled type 1 diabetes (DEPICT-

1): 24 week results from a multicentre, double-blind, phase 3, randomised controlled trial. *Lancet Diabetes Endocrinol.* 2017;5(11):864-76.

15. Mathieu C, Dandona P, Gillard P, Senior P, Hasslacher C, Araki E, Lind M, Bain SC, Jabbour S, Arya N, Hansen L, Thorén F, Langkilde AM; DEPICT-2 Investigators. Efficacy and Safety of Dapagliflozin in Patients With Inadequately Controlled Type 1 Diabetes (the DEPICT-2 Study): 24-Week Results From a Randomized Controlled Trial. Diabetes Care. 2018 Sep;41(9):1938-1946.

16. European Medicines Agency. Assessment Report Dapagliflozin. https://www.ema.europa.eu/en/documents/variation-report/forxiga-h-c-ws-1941-epar-assessment-report-variation_en.pdf *(accessed 11th November 2021)*

17. Groop PH, Dandona P, Phillip M, Gillard P, Edelman S, Jendle J, Xu J, Scheerer MF, Thoren F, Iqbal N, Repetto E, Mathieu C. Effect of dapagliflozin as an adjunct to insulin over 52 weeks in individuals with type 1 diabetes: post-hoc renal analysis of the DEPICT randomised controlled trials. Lancet Diabetes Endocrinol. 2020 Oct;8(10):845-854. doi: 10.1016/S2213-8587(20)30280-1. PMID: 32946821.

18. Rosenstock J, Marquard J, Laffel LM, et al. Empagliflozin as adjunctive to insulin therapy in type 1 diabetes: the EASE trials. *Diabetes Care*. 2018;41(12):2560–2569.

19. Garg SK, Henry RR, Banks P, Buse JB, Davies MJ, Fulcher GR, et al. Effects of Sotagliflozin Added to Insulin in Patients with Type 1 Diabetes. *N Engl J Med.* 2017;377(24):2337-48.

20. European Medicines Agency. First oral add-on treatment to insulin for treatment of certain patients with type 1 diabetes. https://www.ema.europa.eu/en/news/first-oral-add-treatment-insulin-treatment-certain-patients-type-1-diabetes *(accessed 11th November 2021)*

21. An overview of Zynquista and why it is authorised in the EU. 16th May 2019. https://www.ema.europa.eu/en/documents/overview/zynquista-epar-medicine-overview_en.pdf *(accessed 11th November 2021)*

22. National Institute for Health and Care Excellence. Dapagliflozin with insulin for treating type 1 diabetes. Technology appraisal guidance [TA597] https://www.nice.org.uk/guidance/ta597

23. AstraZeneca Press Release. Update on US regulatory decision for Farxiga in type-1 diabetes. https://www.astrazeneca.com/media-centre/press-releases/2019/update-on-us-regulatory-decision-for-farxiga-in-type-1-diabetes-15072019.html# *(accessed 11th November 2021)*

24. European Medicines Agency. Forxiga (dapagliflozin) 5mg should no longer be used for the treatment of Type 1 Diabetes Mellitus. https://www.ema.europa.eu/en/documents/dhpc/direct-healthcare-professional-communication-dhpc-forxiga-dapagliflozin-5mg-should-no-longer-be-used_en.pdf *(accessed 11th November 2021)*

25. AstraZeneca. Letter to UK Health Professionals. Forxiga (dapagliflozin) 5mg should no longer be used for the treatment of type 1 diabetes mellitus. (2nd November 2021)

26. Marso SP, Bain SC, Consoli A, Eliaschewitz FG, Jódar E, Leiter LA, Lingvay I, Rosenstock J, Seufert J, Warren ML, et al. Semaglutide and cardiovascular

outcomes in patients with type 2 diabetes. *N Engl J Med.* 2016;**375**(19):1834–44. doi: 10.1056/NEJMoa1607141.
27. Ozempic Summary of product characteristics. [Ozempic 0.5 mg solution for injection in pre-filled pen - Summary of Product Characteristics (SmPC) - (emc) (medicines.org.uk)]
28. Nauck MA, Petrie JR, Sesti G, Mannucci E, Courrèges JP, Lindegaard ML, Jensen CB, Atkin SL; Study 1821 Investigators. A Phase 2, Randomized, Dose-Finding Study of the Novel Once-Weekly Human GLP-1 Analog, Semaglutide, Compared With Placebo and Open-Label Liraglutide in Patients With Type 2 Diabetes. Diabetes Care. 2016 Feb;39(2):231-41.
29. Forxiga 5 mg Summary of Product Characteristics: https://www.medicines.org.uk/emc/product/2865/smpc *(accessed 11th November 2021)*
30. Wiviott SD, Raz I, Bonaca MP, Mosenzon O, Kato ET, Cahn A, Silverman MG, Zelniker TA, Kuder JF, Murphy SA, Bhatt DL, Leiter LA, McGuire DK, Wilding JPH, Ruff CT, Gause-Nilsson IAM, Fredriksson M, Johansson PA, Langkilde AM, Sabatine MS; DECLARE–TIMI 58 Investigators. Dapagliflozin and Cardiovascular Outcomes in Type 2 Diabetes. N Engl J Med. 2019 Jan 24;380(4):347-357.
31. McMurray JJV, Solomon SD, Inzucchi SE, Køber L, Kosiborod MN, Martinez FA et al. DAPA-HF Trial Committees and Investigators Dapagliflozin in patients with heart failure and reduced ejection fraction. N Engl J Med. 2019. Nov 21;381(21):1995-2008.
32. Heerspink HJL, Stefánsson BV, Correa-Rotter R, Chertow GM, Greene T, Hou FF, Mann JFE, McMurray JJV, Lindberg M, Rossing P, Sjöström CD, Toto RD, Langkilde AM, Wheeler DC; DAPA-CKD Trial Committees and Investigators. Dapagliflozin in Patients with Chronic Kidney Disease. N Engl J Med. 2020 Oct 8;383(15):1436-1446. doi: 10.1056/NEJMoa2024816. Epub 2020 Sep 24. PMID: 32970396.
33. Vilsbøll T, Bain SC, Leiter LA, et al. Semaglutide, reduction in glycated haemoglobin and the risk of diabetic retinopathy. *Diabetes Obes Metab*. 2018;20(4):889–897.
34. Lingvay I, Desouza CV, Lalic KS et al. A 25 week randomized controlled trial of semaglutide once daily versus liraglutide and placebo in patients with type 2 diabetes suboptimally controlled on diet and exercise with or without metformin. Diabetes Care. 2018;41(9): 1926-1937.
35. European Medicines Agency. Assessment Report Sotagliflozin. https://www.ema.europa.eu/en/documents/assessment-report/zynquista-epar-public-assessment-report_en.pdf *(accessed 11th November 2021)*
36. Silverii GA, Decembrini L, Monami M, Mannucci E. Fournier's gangrene and sodium glucose co-transporter 2 inhibitors: A metanalysis of randomized controlled trials. Diabetes Obes Metab. 2019; 1-4.
37. Garg SK, Peters AL, Buse JB, Danne T. Strategy for mitigating DKA risk in patients with Type 1 diabetes on adjunctive treatment with SGLT-2 inhibitors: A STICH Protocol. Diabetes Technol Ther. 2018;20(9):571-575.
38. Peters AL, Buschur EO, Buse JB, Cohan P, Diner JC, and Hirsch IB. Euglycemic Diabetic Ketoacidosis: A Potential Complication of Treatment With Sodium-Glucose Cotransporter 2 Inhibition. Diabetes Care. 2015;38(9):1687-93.
39. Agiostratidou G, Anhalt H, Ball D, Blonde L, Gourgari E, Harriman KN, Kowalski AJ, Madden P, McAuliffe-Fogarty AH, McElwee-Malloy M, Peters A, Raman S, Reifschneider

K, Rubin K, Weinzimer SA. Standardizing Clinically Meaningful Outcome Measures Beyond HbA(1c) for Type 1 Diabetes: A Consensus Report of the American Association of Clinical Endocrinologists, the American Association of Diabetes Educators, the American Diabetes Association, the Endocrine Society, JDRF International, The Leona M. and Harry B. Helmsley Charitable Trust, the Pediatric Endocrine Society, and the T1D Exchange. Diabetes Care. 2017 Dec;40(12):1622-1630

16. APPENDICES

16.1 Appendix 1 – Risk

Risks associated with trial interventions<br>☐ LOW ≡ Comparable to the risk of standard medical care<br>☒ MODERATE ≡ Somewhat higher than the risk of standard medical care<br>☐ HIGH ≡ Markedly higher than the risk of standard medical care				
Justification: Briefly justify the risk category selected and your conclusions below (where the table is completed in detail the detail need not be repeated, however a summary should be given):<br>An IDMC will be convened to monitor all SAEs.<br>Risks and mitigations associated with the intervention are outlined in more detail in the Protocol section 2.1.<br>Co-Sponsors will also carry out a detailed risk assessment of all aspects of the study as part of the approval process (SOP 04.013)				
What are the key risks related to therapeutic interventions you plan to monitor in this trial?		How will these risks be minimised?		
IMP/Intervention	Body system/Hazard	Activity	Frequency	Comments
S/C administration of semaglutide<br>Oral admistration of dapagliflozin	Immune: Hypersensitivity/ana-phylactic reactions	Patients with known hypersensitivity to any GLP-1 agonist or SGLT-2 inhibitor have a contra-indication to the IMP according to the SmPC will not be recruited to the study	Uncommon/ Rare	
PO administration of dapagliflozin	Ketosis and/or Diabetic Ketoacidosis (DKA)	All participants will have access to blood or urine ketone monitoring equipment. An emergency ketosis management plan (STICH Protocol – see above) will be discussed with all participants. Patient alert cards will be provided to all participants. Potential	Common	“and” was an error – intention was for “either” blood or urine ketones between visits but “both” to be measured for visits. This is compatible with the eCRF, the diaries and the rest of the protocol

		participants with recent ketoacidosis will be excluded from the trial (section 6.2)		
S/C administration of semaglutide (+/-) PO administration of dapagliflozin in addition to insulin therapy	Increased risk of hypoglycaemia	Participant awareness of risk and frequent testing of capillary blood glucose. Patients with pre-existing frequent episodes of hypoglycaemia or problems with hypoglycaemia awareness will be excluded from trial participation (section 6.2)	Common	
Outline any other processes that have been put in place to mitigate risks to participant safety (e.g. IDMC, independent data review, etc.)<br>See above				

16.2 Appendix 2: Contraception

In line with recommendations related to contraception and pregnancy testing in clinical trials from the Clinical Trial Facilitation Group (CTFG), for women of childbearing potential* (WOCBP) participating in TTT1, methods of contraception considered as highly effective are defined as those that can achieve a failure rate of less than 1% per year when used consistently and correctly.

Such methods include:

- combined (oestrogen and progestogen containing) hormonal contraception associated with inhibition of ovulation[1]
  - oral
  - intravaginal
  - transdermal
- progestogen-only hormonal contraception associated with inhibition of ovulation[1]
  - oral
  - injectable
  - implantable[2]
- intrauterine device (IUD) [2]
- intrauterine hormone-releasing system (IUS)[2]
- bilateral tubal occlusion[2]
- vasectomised partner[2,3]
- true sexual abstinence[4]

Footnotes

1. An interaction between dapagliflozin and semaglutide with contraception is not anticipated.
2. Contraception methods that in the context of this guidance are considered to have low user dependency.
3. Vasectomised partner is a highly effective birth control method provided that partner is the sole sexual partner of the WOCBP trial participant and that the vasectomised partner has received medical assessment of the surgical success.
4. In the context of this guidance true sexual abstinence refers to: When this is in line with the preferred and usual lifestyle of the patient. Periodic abstinence (e.g. calendar, ovulation, symptothermal, post-ovulation methods), declaration of abstinence for the duration of a trial, and withdrawal are not acceptable methods of contraception.
The reliability of sexual abstinence needs to be evaluated in relation to the duration of the clinical trial and the preferred and usual lifestyle of the subject.

*Child bearing potential = experienced menarche; AND not undergone successful surgical sterilisation (hysterectomy, bilateral tubal ligation, or bilateral oophorectomy); AND not post-menopausal i.e. amenorrhea for ≥12 consecutive months (without another medical cause)

Source: Recommendations related to contraception and pregnancy testing in clinical trials. Clinical Trial Facilitation Group (CTFG), 15th September 2014.
See: https://www.hma.eu/fileadmin/dateien/Human_Medicines/01-About_HMA/Working_Groups/CTFG/2014_09_HMA_CTFG_Contraception.pdf (Accessed 21st January 2020)

16.3 Appendix 3

STICH Protocol *(also provided in Period 2 Participant Diary)*

As explained above, participants in this trial randomised to dapagliflozin are at an increased risk of ketosis and ketoacidosis. All participants will have access to a ketone meter as standard of care. It will be ascertained that each participant has access to a ketone meter; if there are any issues with their current ketone meter it will be replaced as per standard of care. There are mechanisms designed to mitigate the risk of early ketosis developing further into diabetic ketoacidosis. In this trial, we will be using a protocol called STICH (37). This is a mnemonic designed to act as a reminder for participants as to what to do in the event of elevated ketones following exposure to an SGLT-2 drug such as dapagliflozin:

1. STop dapagliflozin or placebo temporarily
2. Insulin – take a bolus of rapid acting insulin
3. Carbohydrate – take approximately 30g of carbohydrate
4. Hydrate – make sure you are taking sips of water aiming to maintain hydration.

Further guidance will be provided in both the Patient Diaries supplied to all participants and in the Patient Alert Cards that participants will be asked to carry at all times. All participants will also be advised that in the event of acute intercurrent illness or hospitalisation, dapagliflozin or placebo should be temporarily stopped. This is because intercurrent illness may additionally increase the risk of ketosis. Participants will likely already be familiar with this concept of "sick-day rules" as this is a key component of basic education for people with type 1 diabetes in clinical practice. These rules will however be re-iterated to participants.

More detailed guidance on actions participants should take for any given level of ketosis will be provided during face-to-face and telephone contact during the trial. In addition, however, the following written protocol will be provided to participants to advise them explicitly of the safest and most appropriate course of action to be taken in any given circumstance. This will take the form of a traffic light system to aid understanding and compliance.

1. Ketones 0.6 mmol/L or less: Continue - no need for additional bolus insulin.
2. Ketones 0.6 – 1.4 mmol/L: consider taking additional fast acting insulin. Follow sick day rules and monitor ketones 2 hourly until they are 0.6 mmol/L or less.
3. Ketones 1.5 – 2.9 mmol/L – Take 10% of your total daily dose of insulin (this is found by adding up all the long acting and short acting insulin for the previous day - only take a maximum of 10 units) as a bolus of fast acting insulin. Keep hydrated - aim for 100ml water per hour. Re-test your glucose and ketones in 2 hours. If not falling contact the study team during office hours. If you need urgent advice at other times please contact NHS 24 (telephone number 111) or attend your nearest Accident & Emergency Department.
4. Ketones above 3 mmol/L – Take 20% of your total daily insulin as fast acting insulin (Maximum 10 units). Hydrate with water, aim for 500 ml per hour. Attend A&E – High risk of DKA

If you develop DKA or are unwell it is important that you let the TTT1 nurse or doctor know about this as soon as you can. By following this protocol, the risk of ketosis and developing ketoacidosis will be

mitigated.

16.4 Appendix 4: Amendment History

Sponsor Amendment No.	Protocol version no.	Date issued	Author(s) of changes	Details of changes made
AM02	2.0	Ethics App – 23 Nov 2020		Protocol Changes: Weight loss mitigation, 19 Risk Mitigation and visit activity corrections
AM03	2.1	Ethics App – 10th Feb 2021		Correction of Summary of changes for previous AM02.<br>Corrected pregnancy testing schedule.
AM06	3.0	Ethics App – 16th Feb 2022		Definition of AESIs narrowed to include only ketosis and level 2 hypoglycaemia; lowering eGFR inclusion criterion; correction of DSQOLS to licensed version.
AM08	4.0	Ethics App – 26th June 2023		Procedure for recruitment at other sites within NHS GGC described; procedures for rescreening of participants experiencing delay between the screening visit and Visit 1; updates; correction of errors
AM09	5.0	Not required		Following discussion at TMG (21st Sept) and Sponsor reps (26th Sept), it was agreed it was appropriate to remove the word "Summary" and addition of text to avoid duplication of sending PIS twice, from page 33 of the protocol. (Recruitment section). While the Summary PIS was produced and approved during the study set up, in practice it has not been required, with telephone calls and full PIS being sent (following initial discussion in clinics) being the preferred method. For this reason the Summary PIS has not been updated in line with the protocol amendments and is now out of date, therefore is not appropriate to be sent.
AM11	6.0	Ethics App – 21st Mar 2024		Clarification to the recruitment process, Updated information of interim analysis, now planned. Changes to further align with US protocol with regard withdrawals.
AM12	7.0	Awaited		Protocol and PIS updated in line with recent Semaglutide SmPC.<br>Re the protocol, a number of points of clarification and correction of errors in text have been included. These are listed in the

				Summary of Protocol Changes document submitted with this application.<br>The PIS has also been updated re the SmPC update and again a number of clarifications/ corrections have also been included.

List details of all protocol amendments here whenever a new version of the protocol is produced. Protocol amendments must be submitted to the Sponsor for approval prior to submission to the REC committee or MHRA.

16.5 Appendix 5: PAID Questionnaire

Problem Areas In Diabetes (PAID) scale

Instructions: Which of the following diabetes issues are **currently** a problem for you? Tick the box that gives the best answer for you. Please provide an answer for each question.

		Not a problem	Minor problem	Moderate problem	Somewhat serious problem	Serious problem
1	Not having clear and concrete goals for your diabetes care?	☐0	☐1	☐2	☐3	☐4
2	Feeling discouraged with your diabetes treatment plan?	☐0	☐1	☐2	☐3	☐4
3	Feeling scared when you think about living with diabetes?	☐0	☐1	☐2	☐3	☐4
4	Uncomfortable social situations related to your diabetes care (e.g. people telling you what to eat)?	☐0	☐1	☐2	☐3	☐4
5	Feelings of deprivation regarding food and meals?	☐0	☐1	☐2	☐3	☐4
6	Feeling depressed when you think about living with diabetes?	☐0	☐1	☐2	☐3	☐4
7	Not knowing if your mood or feelings are related to your diabetes?	☐0	☐1	☐2	☐3	☐4
8	Feeling overwhelmed by your diabetes?	☐0	☐1	☐2	☐3	☐4
9	Worrying about low blood glucose reactions?	☐0	☐1	☐2	☐3	☐4
10	Feeling angry when you think about living with diabetes?	☐0	☐1	☐2	☐3	☐4
11	Feeling constantly concerned about food and eating?	☐0	☐1	☐2	☐3	☐4
12	Worrying about the future and the possibility of serious complications?	☐0	☐1	☐2	☐3	☐4
13	Feelings of guilt or anxiety when you get off track with your diabetes management?	☐0	☐1	☐2	☐3	☐4
14	Not 'accepting' your diabetes?	☐0	☐1	☐2	☐3	☐4
15	Feeling unsatisfied with your diabetes physician?	☐0	☐1	☐2	☐3	☐4
16	Feeling that diabetes is taking up too much of your mental and physical energy every day?	☐0	☐1	☐2	☐3	☐4
17	Feeling alone with your diabetes?	☐0	☐1	☐2	☐3	☐4
18	Feeling that your friends and family are not supportive of your diabetes management efforts?	☐0	☐1	☐2	☐3	☐4
19	Coping with complications of diabetes?	☐0	☐1	☐2	☐3	☐4
20	Feeling 'burned out' by the constant effort needed to manage diabetes?	☐0	☐1	☐2	☐3	☐4

© Joslin Diabetes Center, 1999 (www.joslin.org). All rights reserved. The copyright holder/developer has given permission for the questionnaire to be reproduced in this toolkit. Readers of the toolkit are permitted to reproduce the questionnaire for clinical use and non-commercial research purposes. Readers of the toolkit are not permitted to use the questionnaire for commercial research purposes and must seek permission from the copyright holder/developer to do so.

16.6 Appendix 6: DSQOLS Questionnaire

Diabetes specific quality of life scale (DSQOLS)

Date____________ Name____ DOB______________

Please tick the appropriate box for each question –

Which burdens and restrictions from diabetes and its treatment have you experienced during the last four weeks?...	This statement meets my point of view...					
	5. Perfectly	4. Quite good	3. A little	2. Rather not	1. Hardly	0. Not at all
1. It bothers me that I have to measure my blood glucose so often	☐	☐	☐	☐	☐	☐
2. It burdens me that I always have to think about my nutrition.	☐	☐	☐	☐	☐	☐
3. I suffer from pain because of diabetes.	☐	☐	☐	☐	☐	☐
4. Because of diabetes the relationship to my partner has become worse.	☐	☐	☐	☐	☐	☐
5. I am worried about the fact that my life could be shorter because of diabetes.	☐	☐	☐	☐	☐	☐
6. I have the impression that I am less attractive for others because of diabetes.	☐	☐	☐	☐	☐	☐
7. Because of diabetes I feel sad or depressed	☐	☐	☐	☐	☐	☐
8. I am worried about my future health	☐	☐	☐	☐	☐	☐
9. It is a burden for me how other people react to my diabetes	☐	☐	☐	☐	☐	☐
10. I feel nervous and restless when I think about episodes of low blood sugar						
11. I feel tired and exhausted	☐	☐	☐	☐	☐	☐
12. It bothers me that I have to spend so much time on my diabetes treatment	☐	☐	☐	☐	☐	☐
13. Because of diabetes traveling is complicated and troublesome	☐	☐	☐	☐	☐	☐
14. I get anxious and nervous when I think about the dangers of episodes of low blood glucose	☐	☐	☐	☐	☐	☐

	5. Perfectly	4. Quite good	3. A little	2. Rather not	1. Hardly	0. Not at all
15. Diabetes prevents me from spontaneous physical activities	☐	☐	☐	☐	☐	☐
16. I suffer from frequent urination	☐	☐	☐	☐	☐	☐
17. Because of high blood glucose values after a meal I often feel unwell or less efficient	☐	☐	☐	☐	☐	☐
18. Because of diabetes I feel anxious or threatened	☐	☐	☐	☐	☐	☐
19. I suffer from thirst or having a dry mouth	☐	☐	☐	☐	☐	☐
20. Other people find it hard to understand my problems with diabetes treatment	☐	☐	☐	☐	☐	☐
21. I feel physically unwell	☐	☐	☐	☐	☐	☐
22. I unnecessarily worry too much about episodes of low blood glucose	☐	☐	☐	☐	☐	☐
23. I have to give up good-tasting foods	☐	☐	☐	☐	☐	☐
24. I cannot eat as much as I want of certain foods	☐	☐	☐	☐	☐	☐
25. Because of diabetes I often have physical complaints	☐	☐	☐	☐	☐	☐
26. Diabetes interferes with my future plans	☐	☐	☐	☐	☐	☐
27. I get an uncomfortable feeling when I think about the dangers of a severe episode of low blood glucose	☐	☐	☐	☐	☐	☐
28. Because of diabetes I have less contact with friends or acquaintances	☐	☐	☐	☐	☐	☐
29. I wish I could eat more the way I want to, without having to plan everything beforehand	☐	☐	☐	☐	☐	☐
30. I suffer from frequent infections, itching or skin problems	☐	☐	☐	☐	☐	☐
31. I am concerned about getting into embarrassing situations because of episodes of	☐	☐	☐	☐	☐	☐

low blood glucose						
	5. Perfectly	4. Quite good	3. A little	2. Rather not	1. Hardly	0. Not at all
32. I feel weak or lazy	☐	☐	☐	☐	☐	☐
33. I am dissatisfied with the amount of time I have to spend for medical consultations	☐	☐	☐	☐	☐	☐
34. Because of my diabetes I cannot spend my free time the way I would like	☐	☐	☐	☐	☐	☐
35. I feel like a disabled person	☐	☐	☐	☐	☐	☐
36. I am worried that I could sustain physical injury in the event of a severe episode of low blood glucose	☐	☐	☐	☐	☐	☐
37. It bothers me that I cannot eat like other people	☐	☐	☐	☐	☐	☐
38. I am often worried about the long-term	☐	☐	☐	☐	☐	☐
39. Because of diabetes other people treat me like a "sick person"	☐	☐	☐	☐	☐	☐
40. Because of diabetes I cannot pursue my hobbies as I wish	☐	☐	☐	☐	☐	☐
41. I have to frequently think about diabetes and its consequences	☐	☐	☐	☐	☐	☐
42. It bothers me that I cannot eat as spontaneously as people who do not have diabetes	☐	☐	☐	☐	☐	☐
43. It upsets my stomach when I think about the dangers of severe episodes of low blood glucose	☐	☐	☐	☐	☐	☐
44. Diabetes constantly causes problems while dealing with other people	☐	☐	☐	☐	☐	☐

With respect to diabetes treatment it is very important to me that, resp. it would be my goal that …	5. Very Important	4. Quite Important	3. Rather Important	2. Rather Unimportant	1. Quite Unimportant	0. Totally Unimportant
45. my blood glucose values are always below 140 mg%	☐	☐	☐	☐	☐	☐
46. my blood glucose values are as constant as possible	☐	☐	☐	☐	☐	☐
47. I can plan my leisure flexibly	☐	☐	☐	☐	☐	☐
48. I can avoid even mild hypoglycaemia	☐	☐	☐	☐	☐	☐
49. I can avoid late complications of diabetes in any case, resp. avoid their progress.	☐	☐	☐	☐	☐	☐
50. I can eat flexibly in the way that I can choose the kind, amount and time of meals	☐	☐	☐	☐	☐	☐
51. I am as physically fit as possible.	☐	☐	☐	☐	☐	☐
52. I can in any case avoid severe hypoglycaemia with loss of consciousness	☐	☐	☐	☐	☐	☐
53. I have to measure my blood glucose as rarely as possible.	☐	☐	☐	☐	☐	☐
54. Other people don't find out that I have diabetes,	☐	☐	☐	☐	☐	☐

How satisfied have you been during the last weeks with …	0. Very Satisfied	1. Quite Satisfied	2. Rather Satisfied	3. Rather dissatisfied	4. Quite dissatisfied	5. Totally dissatisfied
55. the level of your blood glucose values?	☐	☐	☐	☐	☐	☐
56. The stability of your blood glucose values?	☐	☐	☐	☐	☐	☐
57. The flexibility of planning your leisure?	☐	☐	☐	☐	☐	☐
58. The frequency of mild hypoglycaemia?	☐	☐	☐	☐	☐	☐
59. your assurance of being protected against late complications, resp. of being able to avoid their progress?	☐	☐	☐	☐	☐	☐
60. The flexibility of your diet?	☐	☐	☐	☐	☐	☐
61. your physical fitness?	☐	☐	☐	☐	☐	☐
62. your assurance of being protected against severe hypoglycaemia with loss of consciousness?	☐	☐	☐	☐	☐	☐
63. the frequency of your blood glucose self-monitoring?	☐	☐	☐	☐	☐	☐
64. The extent to what other people are informed about your diabetes?	☐	☐	☐	☐	☐	☐

Amendments to UK Protocol (substantial modifications)

**Modification**	**Protocol version**	**Date**	**Details**
AM02 (REC ref AM01)	2.0	4th Nov 2020	End of Trial definition, Covid-19 mitigation, study arm clarification, text corrections,<br><br>Patient Information Sheet – updates text re women of childbearing age and contraception.<br><br>Clarification of name of investigational medicinal product (IMP) supplier<br><br>Interventional Alert card – text changes
AM03	2.1	25th Nov 2020	Correction to the Summary of changes document from AM02<br><br>Correction to the protocol to ensure pregnancy testing indicated in the schedule of assessments matches the text within the body of the Protocol.
AM06	3.0	11th Jan 2022	Patient Information Sheet updated to include COVID 19 vaccination Guide. Definition of adverse events of special interest (AESIs) narrowed to include only ketosis and level 2 hypoglycaemia. Change to inclusion criteria to permit individuals with moderate impairment. Correction of questionnaire version to reflect licensing. Clarification in text that blood C-peptide is measured at Visit 10. Option for some visits to be by telephone for those allocated to the standard care arm. Updates to text following manufacturer's voluntary removal of type 1 diabetes indication of IMP dapagliflozin.
AM08	4.0	23rd Feb 2023	Expanding the pool of potential recruits by including other clinic locations within NHS Greater Glasgow and Clyde to address slow recruitment.<br><br>Extend the study to 31.12.2023<br><br>Add information on addressing supply shortages of semaglutide<br><br>Clarifying the visits at which titration of IMP can occur-<br><br>Clarification of when participants can administer semaglutide at home<br><br>Clarifying that additional contact may be made with participants, which had been occurring, and was an oversight in protocol.

			Recruitment target in Glasgow reduced to 50, with a concurrent increase of 7 to the other site for the study in the USA<br><br>Additional information added re COVID-19 and possible drug interactions with COVID vaccines, and drug treatments<br><br>Clarification re re-screening of participants post-consent-<br><br>Additional text re possible interaction with Lithium (from updated SmPC)<br><br>PIS updated in relation to the above changes where required.<br><br>Period 2 Diary amended re an update to the section on out of hours contact.
AM11	5.0	13th Mar 2024	Protocol - Clarification to the recruitment process to more closely align with the most efficient process.<br><br>Updated information of the planned interim analysis on masked data following discussions with IDMC (TSC approval).<br><br>Other changes to treatment and study withdrawal criteria to further align with US protocol.<br><br>PIS - Change of text to align recruitment section with the protocol changes.<br><br>Additional section to explain possible implications following interim analysis.
AM12	6.0	12th Dec 2024	Protocol and PIS updated in line with recent Semaglutide SmPC.<br><br>A number of points of clarification and correction of errors in text<br><br>PIS updated re the SmPC update<br><br>A number of further clarifications/ corrections also included.
