# Supplementary material for: Insulin, Semaglutide and Dapagliflozin in Adults With Type 1 Diabetes: Design and Methods of Triple Therapy for Type 1 Diabetes (TTT1)—An International Phase 3 Clinical Trial
Source: Diabetes Obes Metab. 2026 Jul 19;28(10):8812–24. doi: 10.1111/dom.71076 (PMC13538731; doi:10.1111/dom.71076)
Supplement: Supplementary file 3 [file DOM-28-8812-s002.docx]

**Electronic supplementary material**

**Table S3: Summary of Amendments to US Protocol**

| **Protocol 1.0**  **Section** | **Previous Text -V1.0** | **Protocol**  **V2.0**  **Section** | **Revised or new text – V2.0** | **Justification** |
| --- | --- | --- | --- | --- |
| 1.2 | Primary: combined dapagliflozin and semaglutide treatment in addition to insulin will improve HbA1c levels compared with semaglutide in addition to insulin (by ≥0.5%) or insulin therapy alone (by ≥1%). | 1.2 | Primary: combined dapagliflozin and semaglutide treatment in addition to insulin will improve HbA1c levels compared with semaglutide in addition to insulin. | The thresholds for HbA1c improvement are removed due to risk thresholds set in the primary objective are unmet in the study, and the study could be considered to not have fully met the primary objective. |
| 1.2 | Secondary 1: semaglutide in addition to insulin treatment over 26 and 52 weeks will improve HbA1c levels compared with insulin therapy alone. | 1.2 | Secondary 1: semaglutide in addition to insulin treatment over 26 weeks will improve HbA1c levels compared with insulin therapy alone. |  |
| 1.2 | Secondary 2: combined dapagliflozin and semaglutide treatment in addition to insulin will improve CGM based weekly mean and fasting blood glucose, glycemic oscillations and percent of time in range (80-160mg/dl) with lower insulin requirements compared with semaglutide and insulin (dual therapy) or insulin only (standard) treatment. | 1.2 | Secondary 2: combined dapagliflozin and semaglutide treatment in addition to insulin will improve CGM based weekly mean and fasting blood glucose, glycemic oscillations and percent of time in range (70-180mg/dl) with lower insulin requirements compared with semaglutide and insulin (dual therapy) or insulin only (standard) treatment. |  |
| 1.2 | Secondary 2: combined dapagliflozin and semaglutide treatment in addition to insulin will improve CGM based weekly mean and fasting blood glucose, glycemic oscillations and percent of time in range (80-160mg/dl) with lower insulin requirements compared with semaglutide and insulin (dual therapy) or insulin only (standard) treatment. | 1.2 | Tertiary 1: combined dapagliflozin and semaglutide treatment in addition to insulin therapy will improve fasting and postprandial glucose excursions and suppress postprandial glucagon, FFA, GLP-1 compared with semaglutide and insulin or insulin therapy alone. | Changed from secondary 2 into Tertiary 1 and 2 |
| 1.2 | no statement | 1.2 | Tertiary 2: semaglutide in addition to insulin treatment over 52 weeks will improve HbA1c levels compared with insulin therapy alone. | additional data analysis |
| 2.1 | Change in HbA1c from baseline at six and 12 months in dual therapy group compared to insulin only group. | 2.1 | Change in HbA1c from baseline at 6 months in dual therapy group compared to insulin only group. |  |
| 2.1 | Other indices of glycemic control: fructosamine, weekly mean and fasting glucose, standard deviation, insulin requirements, postprandial glucose excursions and postprandial (Area Under curve) FFA, GLP-1 and glucagon. | 2.1 | Other indices of glycemic control: fructosamine, weekly mean and fasting glucose, standard deviation, insulin requirements. | Items in red- changed into #1 in tertiary |
| 3.1 | None | 3.1 | Due to the potential ketogenic effect of SGLT-2 inhibitors, data from SGLT-2 inhibitors studies in type 1 diabetes were analyzed for it ketogenic effects and a consensus statement and guidelines on management of patients was recently published (Danne T et al., Diabetes Care. 2019 Feb 6). | added this statement to new protocol before preliminary data |
| 6.1 | HbA1c > 7.5% | 6.1 | HbA1c between 7.5-11% inclusive | per FDA letter from 3/26/2019 |
| 6.1 | C-peptide >0.23 nM | 6.1 | C-peptide ≤ 0.23 nM | correct typo |
| 6.1 | Regularly measuring blood sugars four or more times daily | 6.1 | Regularly measuring blood sugars four or more times daily or use of CGM | Add CGM as method of monitoring glucose for patients who have their own CGM instead of finger stick |
| 6.2 | Combined exclusion 11 and 15 with 10 | 6.2 |  |  |
| 6.2 | No statement | 6.2 | Patients with any malignancy except treated in situ malignancy and basal cell carcinoma of the skin | added this exclusion as #28 |
| 6.2 | No statement | 6.2 | Patients on ketogenic or low (<50g/day) carbohydrate diet or who practice intermittent fasting. | added based on FDA letter from 3/26/2019 |
| 10.1 | Study Visit 7: deleted "the next" | 10.1 | Added "10" | Changed number of times we are distributing study drug |
| 10.1 | Study Visit 8: deleted "supply of study drug will be given to last until the next visit" | 10.1 |  | Changed number of times we are distributing study drug |
| 10.1 | Study visit 10: Refer to protocol, many changes | 10.1 |  |  |
| 10.1 | Study visit 11: refer to protocol, many changes | 10.1 |  |  |
| 10.1 | Study Visit 12: deleted "supply of study drug will be given to last until the next visit" | 10.1 |  | Changed number of times we are distributing study drug |
| 10.1 | Study visit 12: changed week 39 to visit 13 | 10.1 |  |  |
| 10.1 | Study visit 13: changed "next visit" to "end of study | 10.1 |  |  |
| 10.1 | Study Visit 14: deleted "supply of study drug will be given to last until the next visit" | 10.1 |  |  |
| 10.1 | Study Visit 15: deleted "supply of study drug will be given to last until the next visit" | 10.1 |  |  |
| 10.1 | Chart- many changes made | 10.1 |  |  |
| 10.1 | no statement | 10.1 | Screening visit: added C-peptide to list of blood drawn. Also added to chart | additional testing |
| 10.1 | no statement | 10.1 | Study visit 9: Add HbA1c testing and measurement of ketones | Phase 2 randomization will be balanced in terms of HbA1c values. Ketones will be measured to insure patients are not ketogenic at start of dapagliflozin treatment |
| 10.1 | Dapagliflozin (or placebo) will be started at a dose of 5 mg daily at week 26 and increased after 1 week to 10 mg (2x5mg tablets) daily to ensure drug tolerability and to avoid any hypoglycemia or hypotension. | 10.1 | . Dapagliflozin (or placebo) will be started at a dose of 5 mg daily at week 26 and if well tolerated increased after 1 week to 10 mg (2x5mg tablets) daily to ensure drug tolerability and to avoid any hypoglycemia or hypotension. | additional safety |
| 10.1 | no statement | 10.1 | Patients who do not tolerate the increase in dosing of either medications will be maintained at the highest tolerated dose. | as per FDA letter 3/26/2019 |
| 10.1 | Astra Zeneca Inc. will supply dapagliflozin and matching placebo tablets in identical packaging; semaglutide pens will be obtained commercially and will be used in an open label fashion. | 10.1 | Astra Zeneca Inc. will supply dapagliflozin and matching placebo tablets in unlabelled and identical packaging; semaglutide pens will be obtained commercially and will be used in an open label fashion. | clarity |
| 10.1 | Meal challenge: no statement | 10.1 | Spot urine will be collected at baseline, 1 and 3hr. | additional sample collection |
| 10.1 drug blinding | Each randomization will be stratified by study site | 10.1 | Each randomization will be stratified by study site and the second randomization will also include HbA1c stratification. | to avoid HbA1c imbalance between groups in 2nd phase |
| 17.1 | no statement | 17.1 | Since the secondary end-points are pre-specified and may as well be correlated, multiplicity control for multiple tests across secondary endpoints may not be necessary. However, Holm-Bonferroni correction for multiplicity testing will be implemented if additional endpoints are tested or if the pre-specified secondary end-points are found to be not correlated. | statistical justification |
| 19.2 | no statement | 19.2 | Included definition of adverse event, serious adverse event, who to report to, etc. Look at section for changes made | As per FDA letter 3/26/2019 and Astra Zeneca |
| 20.1 | Many changes made, look at this section | 20.1 |  |  |
| 21.1 | As mentioned above, there is a risk of ketoacidosis, as shown by some studies and by our data, when SGLT2 inhibitors are used in type 1 diabetes. In our own preliminary studies on triple therapy, we observed euglycemic ketoacidosis in participants whose insulin doses had been reduced by > 20% of baseline levels. Thus, this adverse effect is largely attributable to insulinopenia as well as anti-insulin effects of SGLT2 inhibitors at the hepatic level (e.g. increased gluconeogenesis). | 21.1 | As mentioned above, there is a risk of ketoacidosis, as shown by some studies and by our data, when SGLT2 inhibitors are used in type 1 diabetes. In our own preliminary studies on triple therapy, we observed euglycemic ketoacidosis in participants whose insulin doses had been reduced by > 20% of baseline levels or in patients who are not adequately insulinzed. Thus, this adverse effect is largely attributable to insulinopenia as well as anti-insulin effects of SGLT2 inhibitors at the hepatic level (e.g. increased gluconeogenesis). |  |
| 21.2 | Daily fingerstick glucose, CGM and insulin pump data will be reviewed by the investigators for optimal titration of insulin dose to target glucose levels between 90-160mg/dl. | 21.2 | Daily fingerstick glucose, CGM and insulin pump data will be reviewed by the investigators for optimal titration of insulin dose to target glucose levels between 90-180mg/dl. |  |
