# Supplementary material for: Insulin, Semaglutide and Dapagliflozin in Adults With Type 1 Diabetes: Design and Methods of Triple Therapy for Type 1 Diabetes (TTT1)—An International Phase 3 Clinical Trial
Source: Diabetes Obes Metab. 2026 Jul 19;28(10):8812–24. doi: 10.1111/dom.71076 (PMC13538731; doi:10.1111/dom.71076)
Supplement: Supplementary file 4 [file DOM-28-8812-s004.docx]

**Electronic supplementary material**

## **Table S1: Entry criteria**

| **Inclusion** | **Exclusion** |
| --- | --- |
| **1.** Type 1 diabetes for ≥1 year on stable multiple daily insulin injections (≥4 doses/day) or continuous subcutaneous insulin infusion (CSII) for the past 3 months. | **1.** Type 1 diabetes for <12 months, type 2 diabetes, MODY, or chronic pancreatitis. |
| **2.** C-peptide < 0.23 nM. | **2.** Use of any glucose-lowering agent other than insulin within the last 3 months. |
| **3.** Minimum insulin dose: 0.5 U/kg (MDI) or 0.4 U/kg (CSII). | **3.** Diabetic ketoacidosis (DKA) requiring medical intervention within 3 months prior to screening. |
| **4.** Regular blood glucose monitoring (≥4 times/day by fingerstick, flash, or continuous glucose monitoring). | **4.** Following a ketogenic, low (<50 g/day carbohydrate), or intermittent fasting diet. |
| **5.** HbA1c 7.5–11.0% (inclusive). | **5.** Hypoglycemia unawareness or frequent severe hypoglycemia (more than one episode requiring medical intervention within 3 months). |
| **6.** Proficient in carbohydrate counting (in the opinion of the site Principal Investigator). | **6.** Symptoms of poorly controlled diabetes. |
| **7.** Aged 18–70 years. | **7.** Participation in weight-loss or intensive exercise programmes. |
| **8.** BMI ≥ 25 kg/m². | **8.** Bariatric or other weight-loss surgery within the last 12 months. |
|  | **9.** History of Addison’s disease or chronic adrenal insufficiency. |
|  | **10.** History of diabetes insipidus. |
|  | **11.** Hepatic disease or cirrhosis. |
|  | **12.** AST or ALT >3× upper limit of normal (ULN). |
|  | **13.** Total bilirubin >2× ULN (unless due to Gilbert’s syndrome). |
|  | **14.** Haemoglobin <110 g/L (men) or <100 g/L (women). |
|  | **15.** Coronary event/procedure (MI, unstable angina, CABG, angioplasty) within 3 months or history of heart failure. |
|  | **16.** End-stage renal disease on dialysis or eGFR <45 mL/min/1.73m². |
|  | **17.** Positive HIV, Hepatitis B, or Hepatitis C status. |
|  | **18.** Any other life-threatening, non-cardiac disease. |
|  | **19.** History of pancreatitis or cystic fibrosis. |
|  | **20.** Women who are pregnant, planning pregnancy, breastfeeding, or not on highly effective contraception. |
|  | **21.** Inability to give informed consent. |
|  | **22.** History of gastroparesis. |
|  | **23.** History of medullary thyroid carcinoma or MEN 2 syndrome. |
|  | **24.** Hypersensitivity to GLP-1 agonists, SGLT-2 inhibitors, or any excipients. |
|  | **25.** Known gallstones (cholecystectomy not an exclusion). |
|  | **26.** History of alcohol or drug misuse. |
|  | **27.** Hypertriglyceridaemia >500 mg/dL. |
|  | **28.** Recurrent genital mycotic infections (≥2 episodes in last 6 months). |
|  | **29.** Hypovolaemia. |
|  | **30.** Any malignancy (except treated in situ or basal cell carcinoma). |
|  | **31.** Unexplained haematuria (on 2 occasions) or active bladder cancer. |
|  | **32.** Proliferative retinopathy (based on retinal screening within last 24 months). |
|  | **33.** Use of investigational drug or therapy within the past 90 days. |
|  | **34.** Participation in another interventional clinical trial. |
|  | **35.** Unlikely to comply with study requirements (in the opinion of the Principal Investigator). |
|  | **36.** Unable to undergo meal challenge test for dietary reasons (e.g., vegetarian/vegan diet) |
