# Supplementary material for: Insulin, Semaglutide and Dapagliflozin in Adults With Type 1 Diabetes: Design and Methods of Triple Therapy for Type 1 Diabetes (TTT1)—An International Phase 3 Clinical Trial
Source: Diabetes Obes Metab. 2026 Jul 19;28(10):8812–24. doi: 10.1111/dom.71076 (PMC13538731; doi:10.1111/dom.71076)
Supplement: Supplementary file 5 [file DOM-28-8812-s005.docx]

**Electronic supplementary material**

**Table S2: Summary of Amendments to UK Protocol**

| **Modification** | **Protocol version** | **Date** | **Details** |
| --- | --- | --- | --- |
| AM02 (REC ref AM01) | 2.0 | 4^th^ Nov 2020 | End of Trial definition, Covid-19 mitigation, study arm clarification, text corrections,  Patient Information Sheet – updates text re women of childbearing age and contraception.  Clarification of name of investigational medicinal product (IMP) supplier  Interventional Alert card – text changes |
| AM03 | 2.1 | 25^th^ Nov 2020 | Correction to the Summary of changes document from AM02  Correction to the protocol to ensure pregnancy testing indicated in the schedule of assessments matches the text within the body of the Protocol. |
| AM06 | 3.0 | 11^th^ Jan 2022 | Patient Information Sheet updated to include COVID 19 vaccination Guide. Definition of adverse events of special interest (AESIs) narrowed to include only ketosis and level 2 hypoglycaemia. Change to inclusion criteria to permit individuals with moderate impairment. Correction of questionnaire version to reflect licensing. Clarification in text that blood C-peptide is measured at Visit 10. Option for some visits to be by telephone for those allocated to the standard care arm. Updates to text following manufacturer's voluntary removal of type 1 diabetes indication of IMP dapagliflozin. |
| AM08 | 4.0 | 23^rd^ Feb 2023 | Expanding the pool of potential recruits by including other clinic locations within NHS Greater Glasgow and Clyde to address slow recruitment.  Extend the study to 31.12.2023  Add information on addressing supply shortages of semaglutide  Clarifying the visits at which titration of IMP can occur-  Clarification of when participants can administer semaglutide at home  Clarifying that additional contact may be made with participants, which had been occurring, and was an oversight in protocol.  Recruitment target in Glasgow reduced to 50, with a concurrent increase of 7 to the other site for the study in the USA  Additional information added re COVID-19 and possible drug interactions with COVID vaccines, and drug treatments  Clarification re re-screening of participants post-consent-  Additional text re possible interaction with Lithium ( from updated SmPC)  PIS updated in relation to the above changes where required.  Period 2 Diary amended re an update to the section on out of hours contact. |
| AM11 | 6.0 | 13^th^ Mar 2024 | Protocol - Clarification to the recruitment process to more closely align with the most efficient process.  Updated information of the planned interim analysis on masked data following discussions with IDMC (TSC approval).  Other changes to treatment and study withdrawal criteria to further align with US protocol.  PIS - Change of text to align recruitment section with the protocol changes.  Additional section to explain possible implications following interim analysis. |
| AM12 | 7.0 | 12^th^ Dec 2024 | Protocol and PIS updated in line with recent Semaglutide SmPC.  A number of points of clarification and correction of errors in text  PIS updated re the SmPC update  A number of further clarifications/ corrections also included. |
| Non-Substantial Amendments | | | |
| AM01 | V1.1 | 5^th^ Mar 2020 | Change to IMP supplier name from Tayside Pharmaceuticals to NHS Scotland Pharmaceuticals Specials Service |
| AM09 | 5.0 | 28^th^ Sep 2023 | The Summary PIS for the TTT1 study had, in error, not been updated in line with changes to the Full PIS, and was therefore no longer appropriate to be provided to patients. Following discussion at TMG (21st Sept), which included Sponsor reps (26th Sept), it was agreed that it would be appropriate to send the Full PIS to patients as this had all of the information available in the Summary PIS, and this would allow recruitment to continue. The submission for approval of these changes included copies of the clean and tracked protocol, a file note explaining the reason for the changes and justification of the these, and an amendment tool |
| AM14 | 8.0 | 5^th^ Feb 2026 | Protocol - during resolution of the data queries it was realised that one particular exploratory outcome proposed to be measured in blood samples was no longer possible. During set up, it was noted that Buffalo site routinely performed the Fructosamine assay at local labs. The intention was that Glasgow samples shipped at the end of the study would be assayed as a batch. During the time of the study from 2019, Fructosamine is no longer routinely measured at Buffalo. While this was acknowledged at some point and removed from the eCRF, it was not removed from the Protocol. Since Fructosamine was one of a number of measurements performed as exploratory endpoints there is little impact to the study data. PIS was also reviewed and confirmed no mention of fructosamine. |
